# Association of Sleep Duration, Metabolic Markers, and Risk of All-Cause and Cause-Specific Mortality in China and the UK

**DOI:** 10.5334/gh.1582

**Published:** 2026-09-01

**Authors:** Guangyao Hua, Lijun Zhang, Cong Li, Yan Wang, Xue He, Chingyu Cheng, Lei Liu, Xiaohong Yang, Jinghua Jiao

**Affiliations:** 1 Guangdong Cardiovascular Institute, Guangdong Provincial People’s Hospital (Guangdong Academy of Medical Sciences), Southern Medical University, Guangzhou, China; 2 Third People’s Hospital of Dalian, Dalian Eye Hospital, Dalian, China; 3 Department of Ophthalmology, Guangdong Eye Institute, Guangdong Provincial People’s Hospital (Guangdong Academy of Medical Sciences), Southern Medical University, Guangzhou, China; 4 Singapore Eye Research Institute, Singapore National Eye Centre, 20 College Road, The Academia, Singapore, Singapore; 5 Eye-ACP, Duke-NUS Medical School, Singapore; 6 Department of Ophthalmology, Yong Loo Lin School of Medicine, National University of Singapore, Singapore; 7 Department of Ophthalmology, The Third People’s Hospital Affiliated to Dalian University of Technology, China; 8 Department of Ophthalmology, Jincheng People’s Hospital, China; 9 Department of Anesthesiology, The Affiliated Panyu Central Hospital of Guangzhou Medical University, Guangzhou, China

**Keywords:** sleep duration, metabolic markers, all-cause mortality, cause-specific mortality, population-based study

## Abstract

**Background:**

Sleep duration is associated with various health outcomes like metabolic disturbances and mortality, but the underlying mechanisms remain unclear.

**Objectives:**

To investigate the link between sleep duration, metabolic markers, and mortality using data from the China Kadoorie Biobank (CKB) and the UK Biobank (UKB).

**Methods:**

Sleep duration was self-reported, and metabolic markers were quantified using nuclear magnetic resonance spectroscopy. The outcomes of interest were all-cause and cause-specific mortality. Linear regression was conducted to assess the association between sleep duration and metabolic markers, which were further compared with the association between metabolic markers and all-cause and cause-specific mortality using Cox models.

**Results:**

In both cohorts, sleep duration showed positive associations with all-cause and cause-specific mortality after adjustment. A total of 17 metabolic markers—encompassing lipoprotein subclasses in high-density lipoprotein cholesterol, relative lipoprotein lipid concentrations in very-low-density lipoproteins, cholesterol, and ketone bodies—were significantly associated with sleep duration and mortality. In general, the associations of sleep duration with metabolic markers and of these markers with mortality showed similar patterns for lipoprotein subclasses and relative lipoprotein lipid concentrations, whereas contrary patterns were observed for cholesterol and ketone bodies in the CKB. Furthermore, significant associations were also observed in the UKB cohort, with metabolic markers presenting generally consistent association patterns with sleep duration and mortality. Gender-specific analyses revealed significant links in females in the CKB and in both genders in the UKB. Sensitivity analyses confirmed the stability of these associations.

**Conclusions:**

Our findings suggest that lipid metabolism may partially explain the effect of sleep duration on mortality. These insights highlight the potential of targeted metabolic interventions to mitigate the risks associated with abnormal sleep patterns, especially when modifying sleep duration itself is challenging, and the projected health burden associated with sleep disorders could be reduced through metabolic-focused strategies.

**Lay Summary:**

- **What question did this study address, and why does it matter?**
  This study examined whether sleep duration is associated with metabolic markers and whether these markers, in turn, are associated with mortality risk, using data from two large population-based cohorts in China and the UK. Sleep problems are becoming increasingly common worldwide, and identifying metabolic pathways that may link sleep to health outcomes could inform future prevention strategies.
- **Where was the study done and what were the main results?**
  The study used data from the China Kadoorie Biobank (CKB) and the UK Biobank (UKB). In both cohorts, sleep duration was associated with changes in specific metabolic markers, including lipoprotein subclasses, cholesterol, and ketone bodies. These metabolic changes were further associated with all-cause and cause-specific mortality. While lipoprotein subclasses showed broadly consistent patterns across the two cohorts, cholesterol and ketone bodies exhibited contrary associations with mortality in the CKB compared to the UKB. Sex-specific differences were also observed, particularly in the CKB cohort, where significant associations were found only in women.
- **What do these findings mean in practice?**
  These findings suggest that lipid metabolism may play a role in the relationship between sleep duration and mortality. This raises the possibility that metabolic monitoring or targeted interventions—such as lifestyle or dietary modifications—could potentially help reduce the health risks associated with abnormal sleep duration, particularly in settings where sleep modification itself is challenging. However, further research is needed to determine whether these associations are causal and to explore their underlying mechanisms.

## Graphical Abstract

**Figure:**
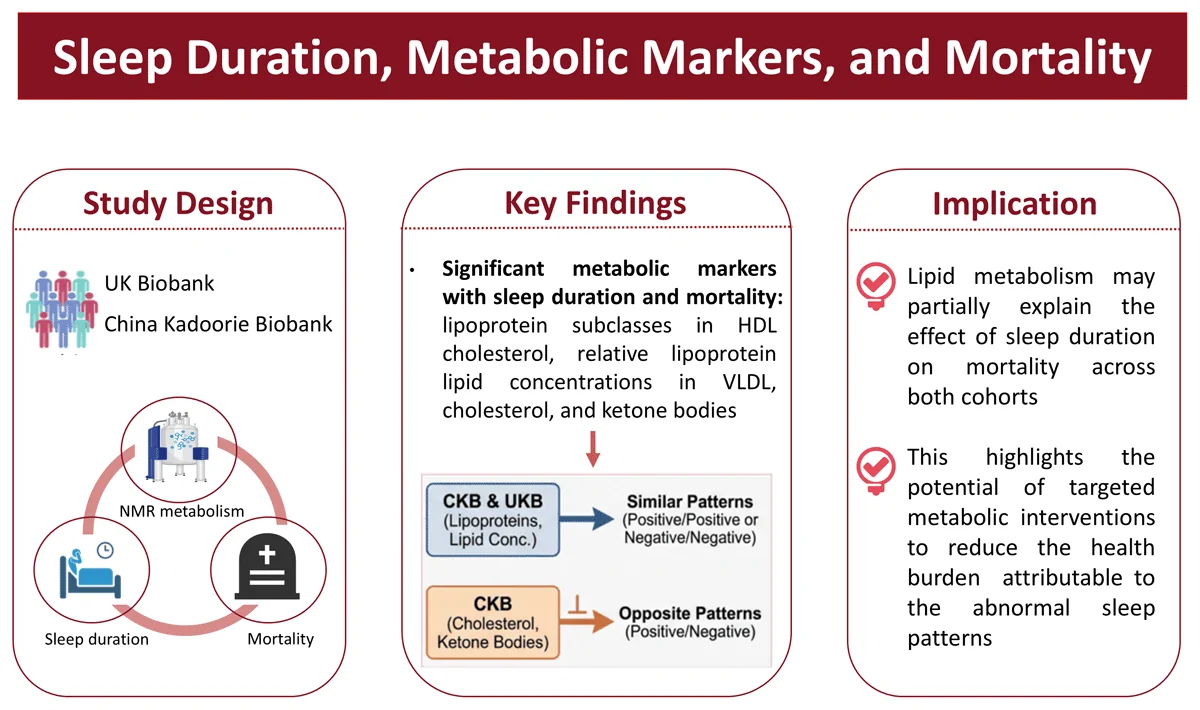


## Introduction

In recent years, sleep disorders have been increasingly recognized as a significant public health issue (1,2). In China, studies indicate that a substantial proportion of adults reports insufficient sleep, with urban populations particularly affected due to lifestyle changes and work demands (3,4). Similarly, in the UK, the National Health Service (NHS) reports that sleep problems are prevalent, with nearly one in five adults experiencing difficulties in sleeping (5). Numerous studies have documented a U-shaped relationship between sleep duration and all-cause mortality, as well as mortality from specific causes, including cardiovascular disease and accidents. However, the findings have been inconsistent, with some research indicating no significant association (6,7,8,9,10).

Moreover, a wealth of research highlights the significant impact of sleep duration on various aspects of metabolic health. Specifically, it has been shown that inadequate sleep duration influences lipid and lipoprotein levels, contributing to dyslipidemia, which is associated with the development of cardiovascular diseases (11,12,13). Additionally, insufficient sleep is linked to an increased risk of overweight and obesity, as it disrupts hormonal regulation of appetite and metabolism, leading to altered energy balance and weight gain (14). Collectively, these findings underscore the importance of adequate sleep in maintaining metabolic homeostasis and preventing obesity-related complications. Despite these findings, there remains a notable gap in research exploring the links between sleep disorders, metabolic markers, and mortality.

Cultural background, economic development, and genetic factors can also influence sleep patterns and their health implications. Different lifestyles and dietary habits across cultures affect sleep quality and duration, while socioeconomic status can dictate access to healthcare and health education, further impacting sleep health (15,16). Genetic predispositions may also play a role, as certain populations may have inherent differences in sleep architecture and metabolic responses to sleep deprivation (17,18). Additionally, the considerable differences in lifestyle between developing and developed countries suggest that the relationship between sleep, metabolism, and mortality may vary significantly. This variation underscores the importance of investigating databases that encompass a wide range of countries globally.

Our study aims to address these gaps by utilizing data from the China Kadoorie Biobank (CKB) and the UK Biobank (UKB). Both datasets provide extensive health information from large, well-characterized populations, enhancing the robustness of our analysis. The CKB offers valuable insights into diverse dietary habits and health behaviors in the Chinese population, while the UKB provides rich data on a wide range of health indicators in a UK cohort. By investigating the relationship between metabolites and sleep in these well-phenotyped participants, we aim to simultaneously explore the associations of sleep duration with metabolic markers, and of these markers with mortality risk across different cultural and economic contexts.

## Methods

### Study population and data collection

Detailed descriptions of the CKB and UKB studies have been previously published (19,20,21). Briefly, the CKB study was a prospective cohort that enrolled 512,724 participants aged 30–79 from five urban and five rural regions in China between 2004 and 2008. Since then, morbidity and mortality data have been continuously followed up. The study gathered comprehensive data on demographic factors, lifestyle habits, and medical and family history through a laptop-based questionnaire. Physical measurements, including blood pressure and random blood glucose, were also recorded, with blood samples (10 mL non-fasting) stored for long-term analysis. Every five years, around 5% of participants are randomly re-surveyed.

The UKB is a large-scale, population-based prospective cohort study that includes 502,409 adults of middle and old age residing in the UK. Between 2006 and 2010, approximately 9.2 million adults registered with the National Health Service were invited to take part in the study. Around 5% of these individuals, aged 40 to 69, agreed to participate, forming the baseline cohort. Participants attended an assessment center where they completed a touchscreen questionnaire addressing sociodemographic details, medical history, and lifestyle factors. Additionally, physical measurements and biological samples were collected at baseline.

### Assessment of sleep and nuclear magnetic resonance metabolisms

In the CKB, participants self-reported their sleep duration at baseline by answering the question, ‘On average, how many hours of sleep do you get in a 24-hour period (including naps)?’ Responses were recorded in one-hour increments (22). In the UKB, sleep duration was also self-reported, with participants responding to the standardized question, ‘About how many hours of sleep do you get in every 24 hours? (Please include naps)’, with answers provided in hours (23).

In the CKB, baseline plasma samples from participants were processed and sent to Oxford for long-term storage in liquid nitrogen tanks after centrifugation. These samples were then analyzed using high-throughput targeted nuclear magnetic resonance (NMR) spectroscopy at the Brainshake Laboratory, Finland, to quantify 225 metabolic markers, with quality control procedures involving two reference samples for consistency and performance checks. The analyses were randomized and blinded, but some metabolic markers were excluded from the final analysis due to quality control rejections, resulting in a smaller sample size for certain markers. In the UKB, Nightingale Health’s laboratories measured concentrations of 168 blood metabolites in absolute level (including 107 non-derived metabolites and 61 composite metabolites) and 81 in ratio measurement for a randomly selected subset of UK Biobank participants. These metabolites included lipoprotein lipids, fatty acids, and their compositions, along with various other low-molecular-weight compounds, using NMR spectroscopy (24). The correspondence of metabolic markers between the CKB and UKB studies is shown in Supplementary Table 1.

### Outcomes

Follow-up for the study began upon completion of the baseline survey. Mortality data were obtained through linkage with the national death surveillance system and population registration system, supplemented by data from routine disease surveillance systems, the national health insurance database, and active targeted surveillance in the CKB cohort. Disease coding was based on the International Classification of Diseases, 10th Revision (ICD-10). The underlying causes of death included ischemic heart disease (I20–I25), cerebrovascular (I61 and I63), cancers (C00–C97), and other causes.

Similarly, in the UKB cohort, the primary outcomes were all-cause, ischemic heart disease-related, cerebrovascular disease-related, and cancer-related mortalities. Data on deaths were obtained through death certificates held within the NHS Information Centre (England and Wales) and the NHS Central Register (Scotland). Outcomes were also classified using ICD-10 codes.

### Covariates

In the CKB, the covariates included sex, age, level of education (high, medium, and low level of education refers to college and above, secondary school, and primary school and below), region, smoking (never, former, or current), drinking (never, former, or current), and self-rated health status (25). We considered the following covariates for adjustment in the UKB: sex, age, educational (high, medium, and low level of education refers to college or university degree, A/AS levels or equivalent and O levels/General Certificate of Secondary Education (GCSE) or equivalent, and none of the aforementioned), ethnicity, smoking (never, former, or current), drinking (never, former, or current), and self-rated health status (26).

### Statistical analysis

Quantitative variables were reported as means with standard deviations (SD), while categorical variables were expressed as numbers and percentages. Baseline characteristics across groups were compared using the chi-squared test or independent t-test, as applicable. To address skewed distributions and enhance interpretability, all metabolites were standardized using *Z*-score normalization.

We performed linear regression to assess the association of sleep duration with the metabolic markers. Levels of metabolic markers were used as an exposure variable in the regression model, and the analyses were adjusted for age, sex, region (CKB only), ethnicity (UKB only), levels of education, smoking status, alcohol consumption, and self-rated health status. The associations were adjusted for multiple testing for each metabolic biomarker using the false discovery rate (FDR) method by Benjamini and Hochberg, with findings deemed significant at FDR P value < 0.05 in the overall analysis.

The association of sleep duration with all-cause and cause-specific mortality was assessed using Cox proportional hazards models to estimate hazard ratios (HRs), with their 95% confidence intervals (CIs) adjusted for age, sex, region, levels of education, smoking status, alcohol consumption, and self-rated health status. Separate Cox regression analyses, adjusting for the same variables, examined the associations of metabolic markers and all-cause and cause-specific mortality.

We then examined whether our results were only significant in a certain subgroup by age (< 60 vs ≥ 60 years), sex, region (CKB only), ethnicity (UKB only), smoking, and alcohol intake. In order to examine the robustness of associations between metabolic markers and all-cause and cause-specific mortality, we performed several sensitivity analyses: excluding participants with mortality that occurred within two years of follow-up; excluding participants with prevalent cardiovascular diseases or cancer at baseline; and additionally adjusting for other covariates, including body mass index (BMI) and medical history such as diabetes and hypertension.

All analyses were conducted using R version 4.2.3 (R Foundation for Statistical Computing, Vienna, Austria).

### Ethical approval

All participants provided written informed consent for the two studies. In the CKB, the study protocol was approved by the Oxford Tropical Research Ethics Committee (reference: 025–04) and the Ethics Review Committee of the Chinese Center for Disease Control and Prevention (approval notice: 005/2004). Ethics approval for the UKB was obtained from the North West Multi-centre Research Ethics Committee (reference: 11/NW/0382, application no. 82906). This research was conducted using the China Kadoorie Biobank (CKB) resource (www.ckbiobank.org, Request No. DAR-2022-00236 and DAR-2024-00089). Publication of results does not require or imply approval by the membership of the CKB Collaborative Group.

### Role of the funding source

The funders of the study had no role in the design of the study; collection, analysis or interpretation of data; or the writing of the report.

## Results

### Characteristics of participants in the CKB and UKB

In the CKB study, metabolic data were available for 7,041 participants. After a mean follow-up of 9.03 years, 2,005 deaths occurred (including 600 from Ischaemic heart disease (IHD), 526 from cerebrovascular causes, 675 from cancer, and 204 from other causes). In the UKB, metabolic data were present for 274,257 participants, with 20,701 deaths after a mean follow-up of 12.20 years, including 2,258 from IHD, 893 from cerebrovascular causes, 10,146 from cancer, and 7,404 from other causes. Table 1 summarizes participant characteristics in both cohorts by all-cause mortality status. In the CKB, deceased individuals were older, more likely to be male and rural residents, while in the UKB, they were older, predominantly female, with higher rates of former smoking and current alcohol use. Both studies showed differences in education, self-rated health, medical history, BMI, and sleep duration across mortality groups.

**Table 1:** Baseline characteristics of participants from CKB and UKB by incidence of all-cause mortality.


	CKB			UKB		
	
	INCIDENT ALL-CAUSE MORTALITY			INCIDENT ALL-CAUSE MORTALITY		

CHARACTERISTIC	NO	YES	P VALUE	NO	YES	P VALUE

**N**	5,036	2,005		253,556	20,701	

**Age, mean (SD), years**	47.62 (9.08)	55.04 (9.65)	<0.001	56.15 (8.06)	61.67 (6.45)	<0.001

**Sex**			<0.001			<0.001

Male	2,207 (43.8%)	1,080 (53.9%)		139,665 (55.1%)	8,335 (40.3%)	

Female	2,829 (56.2%)	925 (46.1%)		113,891 (44.9%)	12,366 (59.7%)	

**Region**			0.010			–

Rural	3,231 (64.2%)	1,351 (67.4%)		–	–	

Urban	1,805 (35.8%)	654 (32.6%)		–	–	

**Ethnicity**			–			<0.001

White	–	–		239,660 (94.9%)	19,931 (96.8%)	

Non-white	–	–		12,788 (5.1%)	650 (3.2%)	

**Smoking status**			<0.001			<0.001

Never	3,300 (65.5%)	1,097 (54.7%)		141,160 (55.9%)	7,944 (38.7%)	

Former	252 (5.0%)	159 (7.9%)		86,291 (34.2%)	8,667 (42.2%)	

Current	1,484 (29.5%)	749 (37.4%)		24,942 (9.9%)	3,917 (19.1%)	

**Alcohol consumption status**			<0.001			<0.001

Never	4,018 (79.8%)	1,486 (74.1%)		10,894 (4.3%)	1,017 (4.9%)	

Former	194 (3.9%)	134 (6.7%)		8431 (3.3%)	1,358 (6.6%)	

Current	824 (16.4%)	385 (19.2%)		233,655 (92.4%)	18,258 (88.5%)	

**Self-rated health**			<0.001			<0.001

Excellent	926 (18.4%)	273 (13.6%)		42,546 (16.9%)	1,852 (9.0%)	

Good	1,426 (28.3%)	487 (24.3%)		148,554 (58.9%)	9,624 (47.0%)	

Fair	2,198 (43.6%)	872 (43.5%)		51,364 (20.4%)	6,273 (30.6%)	

Poor	486 (9.7%)	373 (18.6%)		9,688 (3.8%)	2,737 (13.4%)	

**Education** ^a^			<0.001			<0.001

Low	2,289 (45.5%)	1,186 (59.2%)		110,489 (44.1%)	11,100 (54.6%)	

Medium	2,470 (49.0%)	759 (37.9%)		58,116 (23.2%)	4,632 (22.8%)	

High	277 (5.5%)	60 (3.0%)		82,112 (32.8%)	4,616 (22.7%)	

**BMI, mean (SD), kg/m** ^2^	24.33 (3.51)	23.97 (3.79)	<0.001	27.38 (4.72)	28.37 (5.44)	<0.001

**Sleep duration, mean (SD), h/day**	7.55 (1.33)	7.39 (1.50)	<0.001	7.15 (1.09)	7.25 (1.34)	<0.001

**Hypertension**			<0.001			<0.001

No	2,950 (58.6%)	787 (39.3%)		118,196 (46.6%)	5,798 (28.0%)	

Yes	2,086 (41.4%)	1,218 (60.7%)		135,360 (53.4%)	14,903 (72.0%)	

**Diabetes**			<0.001			<0.001

No	4,816 (95.6%)	1,761 (87.8%)		239,924 (94.6%)	17,692 (85.5%)	

Yes	220 (4.4%)	244 (12.2%)		13,632 (5.4%)	3,009 (14.5%)	


SD, standard deviation; BMI, body mass index. ^a^ In CKB, high, medium, and low level of education refers to college and above, secondary school, and primary school and below. In UKB, high, medium, and low level of education refers to college or university degree, A/AS levels or equivalent and O levels/GCSEs or equivalent, and none of the aforementioned.

### The association between sleep duration and mortality

Supplementary Table 2 details the associations between sleep duration and mortality in the CKB and UKB cohorts. In the CKB, unadjusted models showed a significant inverse link between sleep duration and all-cause mortality (HR: 0.91), but adjusted models indicated a minimal increase in risk (HR: 1.01). Similar trends were noted for IHD and cerebrovascular mortality, while cancer showed no significant change post-adjustment. The UKB findings were consistent with the CKB, showing significant associations for all-cause mortality and increased risks for IHD and cerebrovascular diseases. In metabolic analysis, 17 of 225 markers in the CKB and 214 of 249 in the UKB were linked to sleep duration (FDR < 5%) (Supplementary Table 3).

### The association between sleep duration, metabolic markers, and mortality

Among the 225 metabolic markers or derived traits, 105, 94, 83, 48, and 16 were significantly associated with all-cause mortality, and mortality due to IHD, cerebrovascular disease, cancer, and other causes, respectively, at FDR < 5% in the CKB. Similarly, in the UKB, 202, 189, 148, 179, and 219 markers were associated with these mortality outcomes (Supplementary Tables 4 and 5). A total of 17 metabolic markers were linked to sleep duration and mortality, including seven lipoprotein subclasses in high-density lipoprotein (HDL) cholesterol, seven relative lipoprotein lipid concentrations in very-low-density lipoproteins (VLDLs), two in cholesterol (HDL cholesterol and HDL2 cholesterol), and one in ketone bodies (3-Hydroxybutyrate).

**Figure 1:**
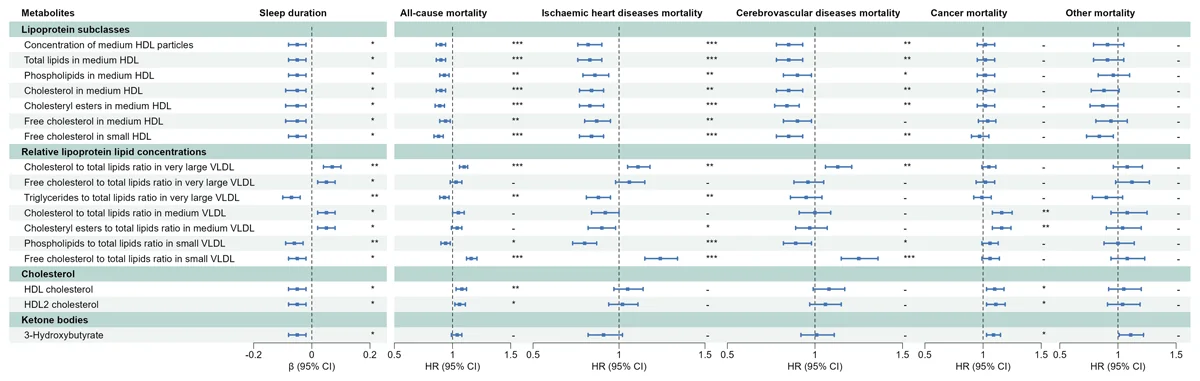
Association of metabolic markers with sleep duration and all-cause and cause-specific mortality in the CKB. Models were adjusted for age, sex, regions, levels of education, smoking status, alcohol consumption, and self-rated health status. P values are corrected for multiple testing using FDR adjustment. Significance: ***p < 0.0001, **P < 0.01, *P < 0.05, - P > 0.05. HR, hazard ratio; CI, confidence interval; HDL, High-Density Lipoprotein; VLDL, Very-Low-Density Lipoprotein.

**Figure 2:**
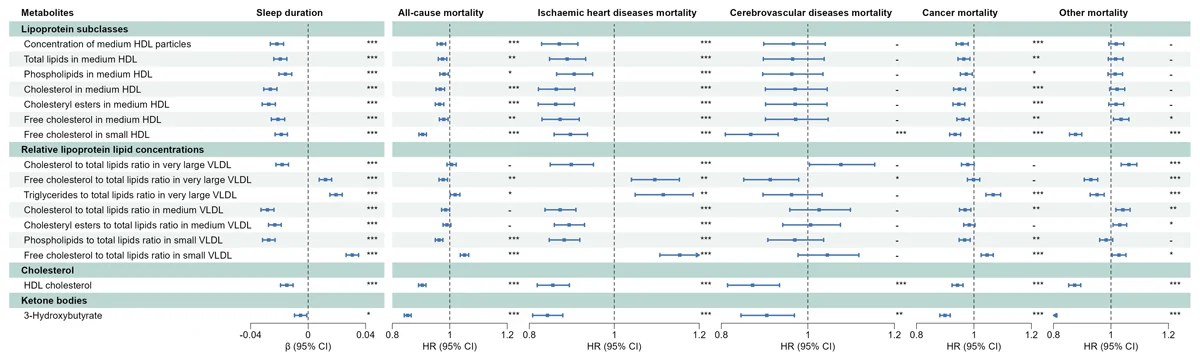
Association of metabolic markers with sleep duration and all-cause and cause-specific mortality in the UKB. Models were adjusted for age, sex, ethnicity, levels of education, smoking status, alcohol consumption, and self-rated health status. P values are corrected for multiple testing using FDR adjustment. Significance: ***p < 0.0001, **P < 0.01, *P < 0.05, - P > 0.05. HR, hazard ratio; CI, confidence interval; HDL, High-Density Lipoprotein; VLDL, Very-Low-Density Lipoprotein.

Within the lipoprotein subclass category in the CKB cohort, five medium HDL particle traits—namely particle concentration, total lipids, phospholipids, cholesterol, and cholesteryl esters—were significantly associated with sleep duration (Figure 1). For mortality outcomes, these five medium HDL markers were consistently and inversely associated with all-cause mortality (HR range: 0.89–0.93) and with both IHD mortality and cerebrovascular disease mortality. For cholesterol metabolites, HDL cholesterol and HDL2 cholesterol were inversely associated with sleep duration but positively associated with all-cause and cancer mortality. Additionally, 3-Hydroxybutyrate was also inversely associated with sleep duration and positively associated with cancer mortality. In the UKB cohort, lipoprotein subclasses were inversely associated with sleep duration, all-cause mortality, IHD mortality, and cancer mortality. Notably, HDL cholesterol and 3-Hydroxybutyrate were also inversely associated with sleep duration, all-cause mortality, and cause-specific mortality (Figure 2).

### Subgroup analysis

**Figure 3:**
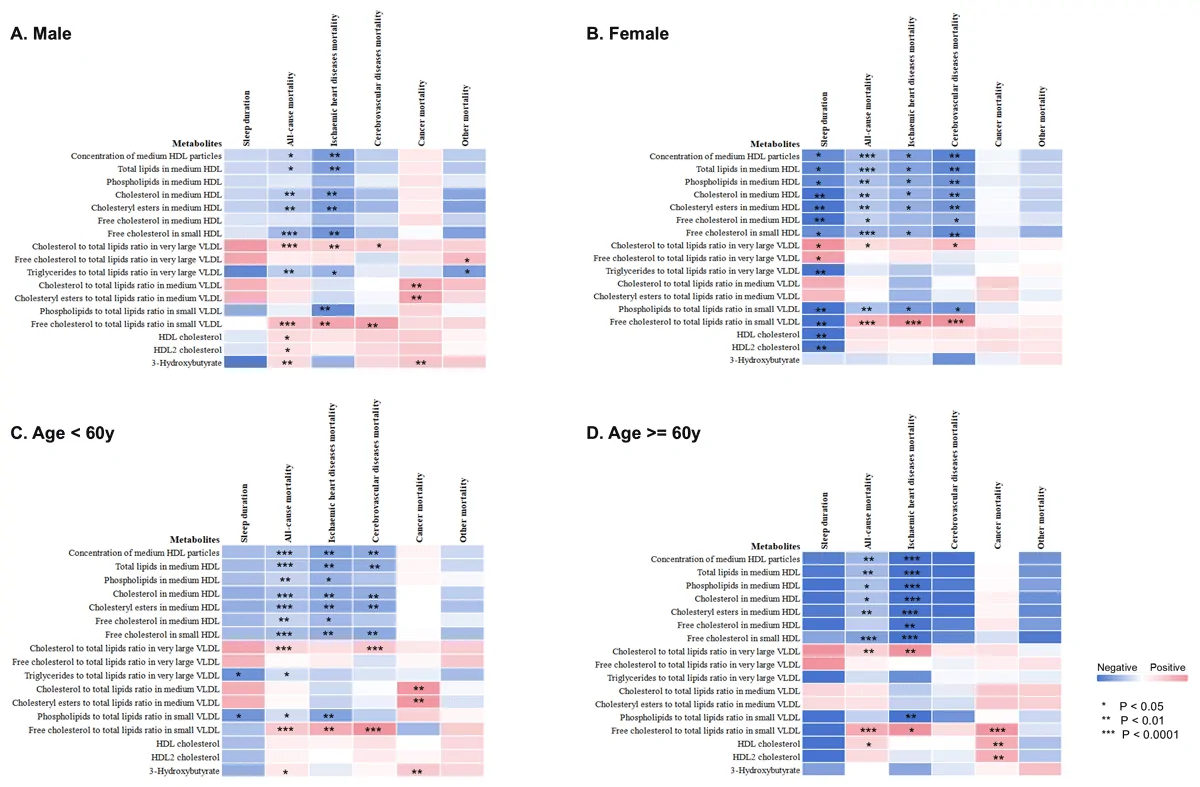
Association of metabolic markers with sleep duration and all-cause and cause-specific mortality in the CKB by sex **(A, B)** and age group **(C, D)**. This heatmap shows the metabolic markers and their β coefficient from multivariable-adjusted linear regression (sleep duration), and HRs from multivariable-adjusted Cox regressions (mortality). Results are adjusted for age, sex, regions, levels of education, smoking status, alcohol consumption, and self-rated health status. P values are corrected for multiple testing using FDR adjustment. Dark pink indicates a strong positive association and dark blue a strong negative association. HDL, High-Density Lipoprotein; VLDL, Very-Low-Density Lipoprotein.

**Figure 4:**
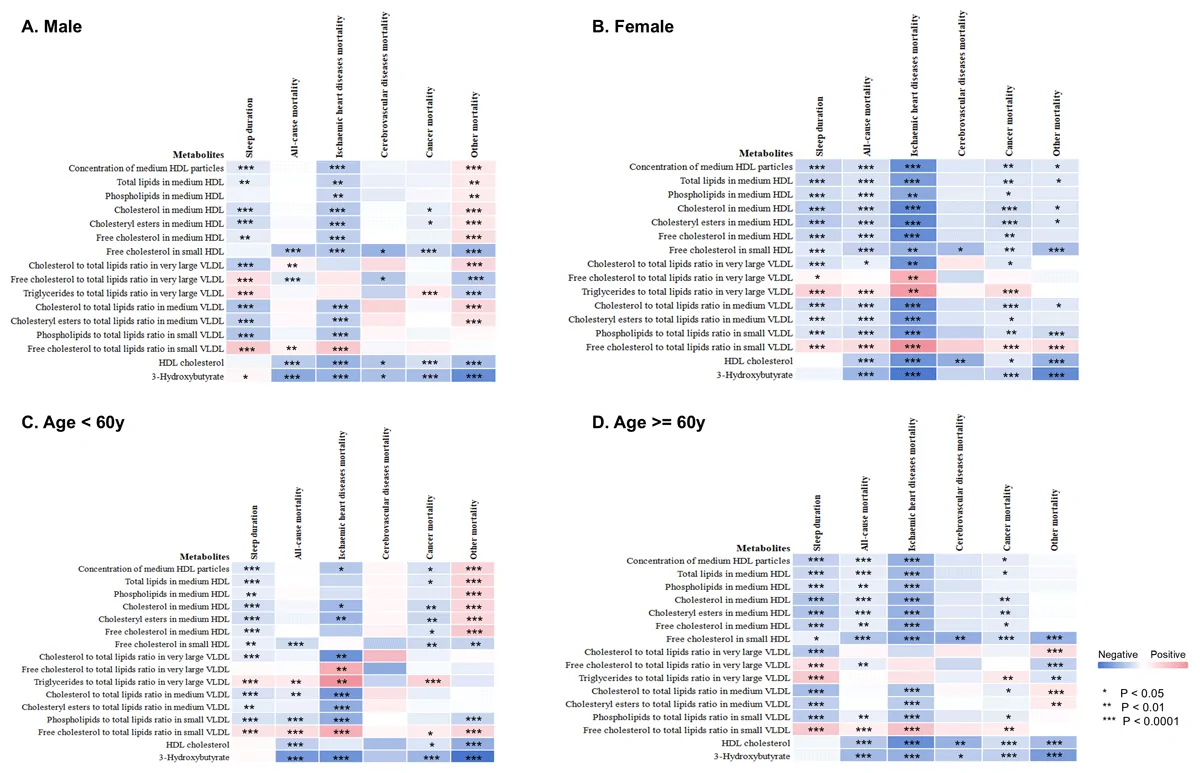
Association of metabolic markers with sleep duration and all-cause and cause-specific mortality in the UKB by sex **(A, B)** and age group **(C, D)**. This heatmap shows the metabolic markers and their β coefficient from multivariable-adjusted linear regression (sleep duration), and HRs from multivariable-adjusted Cox regressions (mortality). Results are adjusted for age, sex, regions, levels of education, smoking status, alcohol consumption, and self-rated health status. P values are corrected for multiple testing using FDR adjustment. Dark pink indicates a strong positive association and dark blue a strong negative association. HDL, High-Density Lipoprotein; VLDL, Very-Low-Density Lipoprotein.

In the gender subgroup analysis, sleep duration in the CKB cohort was significantly associated with metabolic markers only in females, while no associations were found in males (Figure 3). In contrast, the UKB cohort showed significant associations in both genders (Figure 4). For the age analysis, no associations between sleep duration and metabolic markers were observed in CKB participants aged ≥ 60 years, whereas in the UKB, associations were consistent across both age groups (< 60 and ≥ 60 years) (Figures 3 and 4). The metabolic markers’ links to all-cause and cause-specific mortality were generally consistent with the main findings in both cohorts (Figures 3 and 4). Detailed subgroup analysis for associations between all metabolic markers, sleep duration, and mortality can be found in Supplementary Tables 6–12.

### Sensitivity analysis

In sensitivity analyses, after excluding deaths within the first two years (Supplementary Table 13), participants with baseline cardiovascular disease or cancer (Supplementary Table 14), and adjusting for additional confounders (Supplementary Table 15), the associations between sleep duration, metabolic markers, and all-cause mortality remained largely consistent with the main findings.

## Discussion

This study highlights the intricate relationships between sleep duration, metabolic markers, and mortality outcomes in the CKB and the UKB. In both cohorts, sleep duration was positively associated with all-cause and cause-specific mortality after adjustment. In the CKB cohort, 17 metabolic markers—encompassing lipoprotein subclasses in HDL cholesterol, relative lipoprotein lipid concentrations in VLDL, cholesterol, and ketone bodies—were significantly associated with sleep duration, with distinct patterns observed across mortality outcomes: lipoprotein subclasses were inversely associated with mortality, whereas cholesterol and ketone bodies showed positive associations. In contrast, the UKB cohort exhibited generally consistent inverse associations for all metabolic marker categories with sleep duration and mortality, highlighting substantial cross-population heterogeneity in the metabolic pathways linking sleep to mortality. Gender-specific and sensitivity analyses confirmed the stability of these associations, showing significant results in females in the CKB and in both genders in the UKB, with consistent patterns of association for metabolic markers with sleep duration and mortality across both cohorts. Overall, these findings reveal that sleep duration significantly influences metabolic factors, subsequently affecting overall and cause-specific mortality outcomes.

Our findings align with existing research indicating a significant relationship between sleep duration and mortality outcomes. Previous studies in the US have consistently shown that both short and long sleep durations are associated with increased all-cause mortality and specific causes of death, particularly cardiovascular diseases and cancer (27,28). A meta-analysis found that individuals sleeping more than nine hours per day had a significantly positive association with all-cause and cardiovascular disease mortality (29), and the All of Us Research Program observed J-shaped associations between daily sleep duration and chronic disease development (30). Our results corroborate these observations, demonstrating a clear connection between sleep duration and mortality in both the CKB and UKB cohorts. Notably, in the CKB cohort, the initial negative association between sleep duration and all-cause mortality shifted to a positive correlation after confounder adjustment, which may reflect population-specific characteristics—particularly the predominantly rural composition of the CKB sample—and underscores the need for tailored health interventions based on regional contexts.

Our investigation into the associations between sleep duration and metabolic markers sheds light on how sleep influences mortality through metabolic pathways. Previous findings on sleep–lipid associations have been inconsistent: shorter sleep was associated with higher HDL in young children (31), and both short and long sleep correlated with lower HDL in women (11,32), while longer sleep was linked to higher HDL in African adults (33). These discrepancies may stem from the fact that previous studies primarily relied on traditional lipid measurements, whereas our NMR-based approach enabled more refined metabolic profiling. In our study, HDL cholesterol was negatively associated with sleep duration in both cohorts. However, high HDL cholesterol was linked to increased risks of all-cause and cancer mortality in the CKB cohort, but decreased mortality risks in the UKB cohort. Although HDL-C is often referred to as ‘good cholesterol’—aligning with our UKB findings—recent evidence from population studies suggests that very high HDL-C levels might paradoxically be linked to increased mortality (34,35,36), consistent with our CKB observations. Taken together, these contrasting patterns suggest that the relationship between HDL-C and mortality is more complex than previously appreciated, and may differ across populations. Given the observational nature of our study, we cannot determine whether the association between HDL-C and mortality reflects a causal relationship or is driven by residual confounding or reverse causation. Therefore, our findings should be interpreted as hypothesis-generating, and whether targeted interventions to address the risks associated with high HDL-C levels will be effective remains a crucial question that requires further investigation.

Subgroup analyses revealed notable heterogeneity. Significant associations between sleep duration and metabolic markers were observed only in females in the CKB cohort, but in both genders in the UKB cohort. Several factors may explain these cross-cohort and sex-specific differences. First, the CKB cohort predominantly comprises rural residents with distinct lifestyle profiles—including different dietary patterns, higher levels of occupational physical activity, and lower obesity prevalence compared to the UKB population—which may modify the metabolic effects of sleep disruption and its downstream health consequences. Second, hormonal factors, particularly menopausal status in women, may influence lipid metabolism and its responsiveness to sleep duration, potentially contributing to the sex-specific associations observed in the CKB (37,38). Third, differences in health behaviors, socioeconomic conditions, and environmental exposures between the two cohorts may further modulate these associations (39). However, given the observational nature of our study and the post-hoc nature of the subgroup analyses, these interpretations remain speculative. Future studies with more detailed characterization of sex hormones, menopausal status, and lifestyle factors are warranted to elucidate the underlying mechanisms driving this heterogeneity. Furthermore, the lack of significant associations observed in specific subgroups, particularly older adults in the CKB cohort, may reflect varying biological resilience or metabolic adaptations in aging populations.

Several potential mechanisms may explain the relationship between sleep duration, lipid metabolism, and mortality. Sleep restriction can increase total cholesterol and LDL-C levels (40), alter plasma lipid species (41), and elevate inflammatory cytokines (42,43), while long sleep duration may involve altered VLDL and HDL metabolism, with sleep fragmentation further contributing to inflammation and lipid disruption (44). These mechanisms underline the significant role of sleep duration in lipid metabolism and its impact on mortality risk. However, as these mechanistic pathways are largely derived from experimental or preclinical studies, their applicability to our observational findings remains speculative and requires further validation.

One of the strengths of our study is the use of large, well-characterized cohorts from diverse populations, allowing for a comprehensive analysis of the associations between sleep duration, metabolic markers, and mortality risk. The availability of detailed metabolic data enabled us to explore specific biochemical pathways linked to mortality, providing insights into potential mechanisms. However, several limitations warrant consideration. First, sleep duration was assessed by self-report without objective assessment of sleep quality, sleep disorders, or circadian variability. While self-reported sleep duration has been widely used and validated in large-scale epidemiological studies (6,28,45), and our sensitivity analyses support the robustness of our findings, we cannot exclude the possibility of misclassification or residual confounding. Future studies incorporating objective sleep assessments, such as actigraphy or polysomnography, are warranted to further elucidate the relationships between sleep phenotypes, metabolic alterations, and mortality. Second, despite multivariable adjustment, residual confounding remains a concern, particularly regarding physical activity, dietary factors, socioeconomic variables, and underlying chronic illnesses. Moreover, our use of NMR-based metabolomics to analyze metabolite profiles may not capture the full spectrum of metabolic changes due to undetected low-concentration metabolites. The reliance on baseline metabolomic data also restricted our ability to assess temporal variations in metabolite levels, which are crucial for understanding dynamic metabolic changes and their relationship with health outcomes. Furthermore, genetic and environmental differences between the cohorts may impact the consistency of our findings.

## Conclusion

In conclusion, our study reinforces the established links between sleep duration and mortality, as well as the critical role of metabolic markers in this relationship. By demonstrating how sleep influences metabolic health and, in turn, affects mortality outcomes, we highlight the intricate pathways through which sleep may impact health. Ultimately, our findings support the hypothesis that sleep duration may influence metabolic processes that affect mortality and cause-specific deaths, emphasizing the need for further research to unravel these complex interactions.

## Additional File

The additional file for this article can be found as follows:

10.5334/gh.1582.s1 Supplementary Files. Supplementary Tables 1 to 15.

## Data Availability

The datasets used and/or analyzed during the current study are available from the corresponding author on reasonable request.
