# Supplementary material for: Association of Sleep Duration, Metabolic Markers, and Risk of All-Cause and Cause-Specific Mortality in China and the UK
Source: Glob Heart. 2026 Sep 1;21(1):65. doi: 10.5334/gh.1582 (PMC13544049; doi:10.5334/gh.1582)
Supplement: Supplementary file 1 [file gh-21-1-1582-s1.pdf]

Supplementary Files

S1	Supplementary Table 1. The correspondence of metabolic markers between the CKB and U
S2	Supplementary Table 1. Association of sleep duration with all-cause and cause-specific mo
S3	Supplementary Table 2. Association of all metabolites with sleep duration in the CKB and
S4	Supplementary Table 3. Association of all metabolites with all-cause mortality in the CKB
S5	Supplementary Table 4. Association of all metabolites with cause-specific mortality in the (
S6	Supplementary Table 5. Association of all metabolites with sleep duration in the CKB in su
S7	Supplementary Table 6. Association of all metabolites with sleep duration in the UKB in su
S8	Supplementary Table 7. Association of all metabolites with all-cause and cause-specific mo
S9	Supplementary Table 8. Association of all metabolites with all-cause and cause-specific mo
S10	Supplementary Table 9. Association of all metabolites with all-cause and cause-specific mo
S11	Supplementary Table 10. Association of all metabolites with all-cause and cause-specific m
S12	Supplementary Table 11. Association of all metabolites with all-cause and cause-specific m
S13	Supplementary Table 12. Association of all metabolites with sleep duration and all-cause a
S14	Supplementary Table 13. Association of all metabolites with sleep duration and all-cause a
S15	Supplementary Table 14. Association of all metabolites with sleep duration and all-cause a

JKB studies
rtality in the CKB and UKB
UKB
and UKB
CKB and UKB
ıbgroups analysis
ıbgroups analysis
ortality in the CKB and UKB by sex
ortality in the CKB and UKB by age
ortality in the CKB and UKB by region or ethnicity
ıortality in the CKB and UKB by smoking status
ıortality in the CKB and UKB by alcohol consumption status
nd cause-specific mortality in the CKB and UKB after excluding participants with mortality occured w
nd cause-specific mortality in the CKB and UKB after excluding participants with prevalent cardiovasc
nd cause-specific mortality in the CKB and UKB after after adjusted additional covariates

ithin two years of follow-up
:ular diseaseas or cancer

Supplementary Table 1. The correspondence of metabolic markers betwe

CKB	UKB
nmr_xxl_vldl_p	Concentration of chylomicrons and extremely large VLDL particles
nmr_xxl_vldl_l	Total lipids in chylomicrons and extremely large VLDL
nmr_xxl_vldl_pl	Phospholipids in chylomicrons and extremely large VLDL
nmr_xxl_vldl_c	Cholesterol in chylomicrons and extremely large VLDL
nmr_xxl_vldl_ce	Cholesteryl esters in chylomicrons and extremely large VLDL
nmr_xxl_vldl_fc	Free cholesterol in chylomicrons and extremely large VLDL
nmr_xxl_vldl_tg	Triglycerides in chylomicrons and extremely large VLDL
nmr_xl_vldl_p	Concentration of very large VLDL particles
nmr_xl_vldl_l	Total lipids in very large VLDL
nmr_xl_vldl_pl	Phospholipids in very large VLDL
nmr_xl_vldl_c	Cholesterol in very large VLDL
nmr_xl_vldl_ce	Cholesteryl esters in very large VLDL
nmr_xl_vldl_fc	Free cholesterol in very large VLDL
nmr_xl_vldl_tg	Triglycerides in very large VLDL
nmr_l_vldl_p	Concentration of large VLDL particles
nmr_l_vldl_l	Total lipids in large VLDL
nmr_l_vldl_pl	Phospholipids in large VLDL
nmr_l_vldl_c	Cholesterol in large VLDL
nmr_l_vldl_ce	Cholesteryl esters in large VLDL
nmr_l_vldl_fc	Free cholesterol in large VLDL
nmr_l_vldl_tg	Triglycerides in large VLDL
nmr_m_vldl_p	Concentration of medium VLDL particles
nmr_m_vldl_l	Total lipids in medium VLDL
nmr_m_vldl_pl	Phospholipids in medium VLDL
nmr_m_vldl_c	Cholesterol in medium VLDL
nmr_m_vldl_ce	Cholesteryl esters in medium VLDL
nmr_m_vldl_fc	Free cholesterol in medium VLDL
nmr_m_vldl_tg	Triglycerides in medium VLDL
nmr_s_vldl_p	Concentration of small VLDL particles
nmr_s_vldl_l	Total lipids in small VLDL
nmr_s_vldl_pl	Phospholipids in small VLDL
nmr_s_vldl_c	Cholesterol in small VLDL
nmr_s_vldl_ce	Cholesteryl esters in small VLDL
nmr_s_vldl_fc	Free cholesterol in small VLDL
nmr_s_vldl_tg	Triglycerides in small VLDL
nmr_xs_vldl_p	Concentration of very small VLDL particles
nmr_xs_vldl_l	Total lipids in very small VLDL
nmr_xs_vldl_pl	Phospholipids in very small VLDL
nmr_xs_vldl_c	Cholesterol in very small VLDL
nmr_xs_vldl_ce	Cholesteryl esters in very small VLDL
nmr_xs_vldl_fc	Free cholesterol in very small VLDL

nmr_xs_vldl_tg	Triglycerides in very small VLDL
nmr_idl_p	Concentration of IDL particles
nmr_idl_l	Total lipids in IDL
nmr_idl_pl	Phospholipids in IDL
nmr_idl_c	Cholesterol in IDL
nmr_idl_ce	Cholesteryl esters in IDL
nmr_idl_fc	Free cholesterol in IDL
nmr_idl_tg	Triglycerides in IDL
nmr_l_ldl_p	Concentration of large LDL particles
nmr_l_ldl_l	Total lipids in large LDL
nmr_l_ldl_pl	Phospholipids in large LDL
nmr_l_ldl_c	Cholesterol in large LDL
nmr_l_ldl_ce	Cholesteryl esters in large LDL
nmr_l_ldl_fc	Free cholesterol in large LDL
nmr_l_ldl_tg	Triglycerides in large LDL
nmr_m_ldl_p	Concentration of medium LDL particles
nmr_m_ldl_l	Total lipids in medium LDL
nmr_m_ldl_pl	Phospholipids in medium LDL
nmr_m_ldl_c	Cholesterol in medium LDL
nmr_m_ldl_ce	Cholesteryl esters in medium LDL
nmr_m_ldl_fc	Free cholesterol in medium LDL
nmr_m_ldl_tg	Triglycerides in medium LDL
nmr_s_ldl_p	Concentration of small LDL particles
nmr_s_ldl_l	Total lipids in small LDL
nmr_s_ldl_pl	Phospholipids in small LDL
nmr_s_ldl_c	Cholesterol in small LDL
nmr_s_ldl_ce	Cholesteryl esters in small LDL
nmr_s_ldl_fc	Free cholesterol in small LDL
nmr_s_ldl_tg	Triglycerides in small LDL
nmr_xl_hdl_p	Concentration of very large HDL particles
nmr_xl_hdl_l	Total lipids in very large HDL
nmr_xl_hdl_pl	Phospholipids in very large HDL
nmr_xl_hdl_c	Cholesterol in very large HDL
nmr_xl_hdl_ce	Cholesteryl esters in very large HDL
nmr_xl_hdl_fc	Free cholesterol in very large HDL
nmr_xl_hdl_tg	Triglycerides in very large HDL
nmr_l_hdl_p	Concentration of large HDL particles
nmr_l_hdl_l	Total lipids in large HDL
nmr_l_hdl_pl	Phospholipids in large HDL
nmr_l_hdl_c	Cholesterol in large HDL
nmr_l_hdl_ce	Cholesteryl esters in large HDL
nmr_l_hdl_fc	Free cholesterol in large HDL
nmr_l_hdl_tg	Triglycerides in large HDL
nmr_m_hdl_p	Concentration of medium HDL particles

nmr_m_hdl_l	Total lipids in medium HDL
nmr_m_hdl_pl	Phospholipids in medium HDL
nmr_m_hdl_c	Cholesterol in medium HDL
nmr_m_hdl_ce	Cholesteryl esters in medium HDL
nmr_m_hdl_fc	Free cholesterol in medium HDL
nmr_m_hdl_tg	Triglycerides in medium HDL
nmr_s_hdl_p	Concentration of small HDL particles
nmr_s_hdl_l	Total lipids in small HDL
nmr_s_hdl_pl	Phospholipids in small HDL
nmr_s_hdl_c	Cholesterol in small HDL
nmr_s_hdl_ce	Cholesteryl esters in small HDL
nmr_s_hdl_fc	Free cholesterol in small HDL
nmr_s_hdl_tg	Triglycerides in small HDL
nmr_xxl_vldl_pl_per	Phospholipids to total lipids ratio in chylomicrons and extremely large
nmr_xxl_vldl_c_per	Cholesterol to total lipids ratio in chylomicrons and extremely large V
nmr_xxl_vldl_ce_per	Cholesteryl esters to total lipids ratio in chylomicrons and extremely l
nmr_xxl_vldl_fc_per	Free cholesterol to total lipids ratio in chylomicrons and extremely lar
nmr_xxl_vldl_tg_per	Triglycerides to total lipids ratio in chylomicrons and extremely large
nmr_xl_vldl_pl_per	Phospholipids to total lipids ratio in very large VLDL /(lipds in ckb)
nmr_xl_vldl_c_per	Cholesterol to total lipids ratio in very large VLDL
nmr_xl_vldl_ce_per	Cholesteryl esters to total lipids ratio in very large VLDL
nmr_xl_vldl_fc_per	Free cholesterol to total lipids ratio in very large VLDL
nmr_xl_vldl_tg_per	Triglycerides to total lipids ratio in very large VLDL
nmr_l_vldl_pl_per	Phospholipids to total lipids ratio in large VLDL
nmr_l_vldl_c_per	Cholesterol to total lipids ratio in large VLDL
nmr_l_vldl_ce_per	Cholesteryl esters to total lipids ratio in large VLDL
nmr_l_vldl_fc_per	Free cholesterol to total lipids ratio in large VLDL
nmr_l_vldl_tg_per	Triglycerides to total lipids ratio in large VLDL
nmr_m_vldl_pl_per	Phospholipids to total lipids ratio in medium VLDL
nmr_m_vldl_c_per	Cholesterol to total lipids ratio in medium VLDL
nmr_m_vldl_ce_per	Cholesteryl esters to total lipids ratio in medium VLDL
nmr_m_vldl_fc_per	Free cholesterol to total lipids ratio in medium VLDL
nmr_m_vldl_tg_per	Triglycerides to total lipids ratio in medium VLDL
nmr_s_vldl_pl_per	Phospholipids to total lipids ratio in small VLDL
nmr_s_vldl_c_per	Cholesterol to total lipids ratio in small VLDL
nmr_s_vldl_ce_per	Cholesteryl esters to total lipids ratio in small VLDL
nmr_s_vldl_fc_per	Free cholesterol to total lipids ratio in small VLDL
nmr_s_vldl_tg_per	Triglycerides to total lipids ratio in small VLDL
nmr_xs_vldl_pl_per	Phospholipids to total lipids ratio in very small VLDL
nmr_xs_vldl_c_per	Cholesterol to total lipids ratio in very small VLDL
nmr_xs_vldl_ce_per	Cholesteryl esters to total lipids ratio in very small VLDL
nmr_xs_vldl_fc_per	Free cholesterol to total lipids ratio in very small VLDL
nmr_xs_vldl_tg_per	Triglycerides to total lipids ratio in very small VLDL
nmr_idl_pl_per	Phospholipids to total lipids ratio in IDL

nmr_idl_c_per	Cholesterol to total lipids ratio in IDL
nmr_idl_ce_per	Cholesteryl esters to total lipids ratio in IDL
nmr_idl_fc_per	Free cholesterol to total lipids ratio in IDL
nmr_idl_tg_per	Triglycerides to total lipids ratio in IDL
nmr_l_ldl_pl_per	Phospholipids to total lipids ratio in large LDL
nmr_l_ldl_c_per	Cholesterol to total lipids ratio in large LDL
nmr_l_ldl_ce_per	Cholesteryl esters to total lipids ratio in large LDL
nmr_l_ldl_fc_per	Free cholesterol to total lipids ratio in large LDL
nmr_l_ldl_tg_per	Triglycerides to total lipids ratio in large LDL
nmr_m_ldl_pl_per	Phospholipids to total lipids ratio in medium LDL
nmr_m_ldl_c_per	Cholesterol to total lipids ratio in medium LDL
nmr_m_ldl_ce_per	Cholesteryl esters to total lipids ratio in medium LDL
nmr_m_ldl_fc_per	Free cholesterol to total lipids ratio in medium LDL
nmr_m_ldl_tg_per	Triglycerides to total lipids ratio in medium LDL
nmr_s_ldl_pl_per	Phospholipids to total lipids ratio in small LDL
nmr_s_ldl_c_per	Cholesterol to total lipids ratio in small LDL
nmr_s_ldl_ce_per	Cholesteryl esters to total lipids ratio in small LDL
nmr_s_ldl_fc_per	Free cholesterol to total lipids ratio in small LDL
nmr_s_ldl_tg_per	Triglycerides to total lipids ratio in small LDL
nmr_xl_hdl_pl_per	Phospholipids to total lipids ratio in very large HDL
nmr_xl_hdl_c_per	Cholesterol to total lipids ratio in very large HDL
nmr_xl_hdl_ce_per	Cholesteryl esters to total lipids ratio in very large HDL
nmr_xl_hdl_fc_per	Free cholesterol to total lipids ratio in very large HDL
nmr_xl_hdl_tg_per	Triglycerides to total lipids ratio in very large HDL
nmr_l_hdl_pl_per	Phospholipids to total lipids ratio in large HDL
nmr_l_hdl_c_per	Cholesterol to total lipids ratio in large HDL
nmr_l_hdl_ce_per	Cholesteryl esters to total lipids ratio in large HDL
nmr_l_hdl_fc_per	Free cholesterol to total lipids ratio in large HDL
nmr_l_hdl_tg_per	Triglycerides to total lipids ratio in large HDL
nmr_m_hdl_pl_per	Phospholipids to total lipids ratio in medium HDL
nmr_m_hdl_c_per	Cholesterol to total lipids ratio in medium HDL
nmr_m_hdl_ce_per	Cholesteryl esters to total lipids ratio in medium HDL
nmr_m_hdl_fc_per	Free cholesterol to total lipids ratio in medium HDL
nmr_m_hdl_tg_per	Triglycerides to total lipids ratio in medium HDL
nmr_s_hdl_pl_per	Phospholipids to total lipids ratio in small HDL
nmr_s_hdl_c_per	Cholesterol to total lipids ratio in small HDL
nmr_s_hdl_ce_per	Cholesteryl esters to total lipids ratio in small HDL
nmr_s_hdl_fc_per	Free cholesterol to total lipids ratio in small HDL
nmr_s_hdl_tg_per	Triglycerides to total lipids ratio in small HDL
nmr_vldl_d	Average diameter for VLDL particles
nmr_ldl_d	Average diameter for LDL particles
nmr_hdl_d	Average diameter for HDL particles
nmr_serum_c	Total cholesterol
nmr_vldl_c	VLDL cholesterol

nmr_remnant_c	Remnant cholesterol (non-HDL, non-LDL -cholesterol)
nmr_ldl_c	LDL cholesterol
nmr_hdl_c	HDL cholesterol/(Total cholesterol in HDL ckb)
nmr_hdl2_c	
nmr_hdl3_c	
nmr_estc	Total esterified cholesterol
nmr_freec	Total free cholesterol
nmr_serum_tg	Total triglycerides
nmr_vldl_tg	Triglycerides in VLDL
nmr_ldl_tg	Triglycerides in LDL
nmr_hdl_tg	Triglycerides in HDL
nmr_totpg	Phosphoglycerides
nmr_tg_pg	Ratio of triglycerides to phosphoglycerides
nmr_pc	Phosphatidylcholines
nmr_sm	Sphingomyelins
nmr_totcho	Total cholines
nmr_apoa1	Apolipoprotein A1
nmr_apob	Apolipoprotein B
nmr_apob_apoa1	Ratio of apolipoprotein B to apolipoprotein A1
nmr_totfa	Total fatty acids
nmr_unsat	Degree of unsaturation
nmr_dha	Docosahexaenoic acid
nmr_la	Linoleic acid
nmr_faw3	Omega-3 fatty acids
nmr_faw6	Omega-6 fatty acids
nmr_pufa	Polyunsaturated fatty acids
nmr_mufa	Monounsaturated fatty acids
nmr_sfa	Saturated fatty acids
nmr_dha_fa	Ratio of docosahexaenoic acid to total fatty acids
nmr_la_fa	Ratio of linoleic acid to total fatty acids
nmr_faw3_fa	Ratio of omega-3 fatty acids to total fatty acids
nmr_faw6_fa	Ratio of omega-6 fatty acids to total fatty acids
nmr_pufa_fa	Ratio of polyunsaturated fatty acids to total fatty acids
nmr_mufa_fa	Ratio of monounsaturated fatty acids to total fatty acids
nmr_sfa_fa	Ratio of saturated fatty acids to total fatty acids
nmr_glc	Glucose
nmr_lac	Lactate
nmr_cit	Citrate
nmr_ala	Alanine
nmr_gln	Glutamine
nmr_his	Histidine
nmr_ile	Isoleucine
nmr_leu	Leucine
nmr_val	Valine

nmr_phe	Phenylalanine
nmr_tyr	Tyrosine
nmr_ace	Acetate
nmr_acace	Acetoacetate
nmr_bohbut	3-Hydroxybutyrate
nmr_crea	Creatinine
nmr_alb	Albumin
nmr_gp	Glycoprotein acetyls
	Acetone
	Cholesteryl esters in HDL
	Cholesteryl esters in LDL
	Cholesteryl esters in VLDL/ (Cholesterol in ckb)
	Clinical LDL cholesterol
	Concentration of HDL particles
	Concentration of LDL particles
	Concentration of VLDL particles
	Free cholesterol in HDL
	Free cholesterol in LDL
	Free cholesterol in VLDL
	Glycine
	Phospholipids in HDL
	Phospholipids in LDL
	Phospholipids in VLDL
	Pyruvate
	Ratio of omega-6 fatty acids to omega-3 fatty acids
	Ratio of polyunsaturated fatty acids to monounsaturated fatty acids
	Total cholesterol minus HDL-C
	Total concentration of branched-chain amino acids (leucine + isoleuci
	Total concentration of lipoprotein particles
	Total lipids in HDL
	Total lipids in LDL
	Total lipids in lipoprotein particles
	Total lipids in VLDL
	Total phospholipids in lipoprotein particles

en the CKB and UKB studies

Field_id in UKB	Group
23481	Lipoprotein subclasses
23482	Lipoprotein subclasses
23483	Lipoprotein subclasses
23484	Lipoprotein subclasses
23485	Lipoprotein subclasses
23486	Lipoprotein subclasses
23487	Lipoprotein subclasses
23488	Lipoprotein subclasses
23489	Lipoprotein subclasses
23490	Lipoprotein subclasses
23491	Lipoprotein subclasses
23492	Lipoprotein subclasses
23493	Lipoprotein subclasses
23494	Lipoprotein subclasses
23495	Lipoprotein subclasses
23496	Lipoprotein subclasses
23497	Lipoprotein subclasses
23498	Lipoprotein subclasses
23499	Lipoprotein subclasses
23500	Lipoprotein subclasses
23501	Lipoprotein subclasses
23502	Lipoprotein subclasses
23503	Lipoprotein subclasses
23504	Lipoprotein subclasses
23505	Lipoprotein subclasses
23506	Lipoprotein subclasses
23507	Lipoprotein subclasses
23508	Lipoprotein subclasses
23509	Lipoprotein subclasses
23510	Lipoprotein subclasses
23511	Lipoprotein subclasses
23512	Lipoprotein subclasses
23513	Lipoprotein subclasses
23514	Lipoprotein subclasses
23515	Lipoprotein subclasses
23516	Lipoprotein subclasses
23517	Lipoprotein subclasses
23518	Lipoprotein subclasses
23519	Lipoprotein subclasses
23520	Lipoprotein subclasses
23521	Lipoprotein subclasses

23522	Lipoprotein subclasses
23523	Lipoprotein subclasses
23524	Lipoprotein subclasses
23525	Lipoprotein subclasses
23526	Lipoprotein subclasses
23527	Lipoprotein subclasses
23528	Lipoprotein subclasses
23529	Lipoprotein subclasses
23530	Lipoprotein subclasses
23531	Lipoprotein subclasses
23532	Lipoprotein subclasses
23533	Lipoprotein subclasses
23534	Lipoprotein subclasses
23535	Lipoprotein subclasses
23536	Lipoprotein subclasses
23537	Lipoprotein subclasses
23538	Lipoprotein subclasses
23539	Lipoprotein subclasses
23540	Lipoprotein subclasses
23541	Lipoprotein subclasses
23542	Lipoprotein subclasses
23543	Lipoprotein subclasses
23544	Lipoprotein subclasses
23545	Lipoprotein subclasses
23546	Lipoprotein subclasses
23547	Lipoprotein subclasses
23548	Lipoprotein subclasses
23549	Lipoprotein subclasses
23550	Lipoprotein subclasses
23551	Lipoprotein subclasses
23552	Lipoprotein subclasses
23553	Lipoprotein subclasses
23554	Lipoprotein subclasses
23555	Lipoprotein subclasses
23556	Lipoprotein subclasses
23557	Lipoprotein subclasses
23558	Lipoprotein subclasses
23559	Lipoprotein subclasses
23560	Lipoprotein subclasses
23561	Lipoprotein subclasses
23562	Lipoprotein subclasses
23563	Lipoprotein subclasses
23564	Lipoprotein subclasses
23565	Lipoprotein subclasses

23566	Lipoprotein subclasses
23567	Lipoprotein subclasses
23568	Lipoprotein subclasses
23569	Lipoprotein subclasses
23570	Lipoprotein subclasses
23571	Lipoprotein subclasses
23572	Lipoprotein subclasses
23573	Lipoprotein subclasses
23574	Lipoprotein subclasses
23575	Lipoprotein subclasses
23576	Lipoprotein subclasses
23577	Lipoprotein subclasses
23578	Lipoprotein subclasses
23579	Relative lipoprotein lipid concentrations
23580	Relative lipoprotein lipid concentrations
23581	Relative lipoprotein lipid concentrations
23582	Relative lipoprotein lipid concentrations
23583	Relative lipoprotein lipid concentrations
23585	Relative lipoprotein lipid concentrations
23586	Relative lipoprotein lipid concentrations
23587	Relative lipoprotein lipid concentrations
23588	Relative lipoprotein lipid concentrations
23589	Relative lipoprotein lipid concentrations
23590	Relative lipoprotein lipid concentrations
23591	Relative lipoprotein lipid concentrations
23592	Relative lipoprotein lipid concentrations
23593	Relative lipoprotein lipid concentrations
23594	Relative lipoprotein lipid concentrations
23595	Relative lipoprotein lipid concentrations
23596	Relative lipoprotein lipid concentrations
23597	Relative lipoprotein lipid concentrations
23598	Relative lipoprotein lipid concentrations
23599	Relative lipoprotein lipid concentrations
23600	Relative lipoprotein lipid concentrations
23601	Relative lipoprotein lipid concentrations
23602	Relative lipoprotein lipid concentrations
23603	Relative lipoprotein lipid concentrations
23604	Relative lipoprotein lipid concentrations
23605	Relative lipoprotein lipid concentrations
23606	Relative lipoprotein lipid concentrations
23607	Relative lipoprotein lipid concentrations
23608	Relative lipoprotein lipid concentrations
23609	Relative lipoprotein lipid concentrations
23610	Relative lipoprotein lipid concentrations

23611	Relative lipoprotein lipid concentrations
23612	Relative lipoprotein lipid concentrations
23613	Relative lipoprotein lipid concentrations
23614	Relative lipoprotein lipid concentrations
23615	Relative lipoprotein lipid concentrations
23616	Relative lipoprotein lipid concentrations
23617	Relative lipoprotein lipid concentrations
23618	Relative lipoprotein lipid concentrations
23619	Relative lipoprotein lipid concentrations
23620	Relative lipoprotein lipid concentrations
23621	Relative lipoprotein lipid concentrations
23622	Relative lipoprotein lipid concentrations
23623	Relative lipoprotein lipid concentrations
23624	Relative lipoprotein lipid concentrations
23625	Relative lipoprotein lipid concentrations
23626	Relative lipoprotein lipid concentrations
23627	Relative lipoprotein lipid concentrations
23628	Relative lipoprotein lipid concentrations
23629	Relative lipoprotein lipid concentrations
23630	Relative lipoprotein lipid concentrations
23631	Relative lipoprotein lipid concentrations
23632	Relative lipoprotein lipid concentrations
23633	Relative lipoprotein lipid concentrations
23634	Relative lipoprotein lipid concentrations
23635	Relative lipoprotein lipid concentrations
23636	Relative lipoprotein lipid concentrations
23637	Relative lipoprotein lipid concentrations
23638	Relative lipoprotein lipid concentrations
23639	Relative lipoprotein lipid concentrations
23640	Relative lipoprotein lipid concentrations
23641	Relative lipoprotein lipid concentrations
23642	Relative lipoprotein lipid concentrations
23643	Relative lipoprotein lipid concentrations
23644	Relative lipoprotein lipid concentrations
23645	Relative lipoprotein lipid concentrations
23646	Relative lipoprotein lipid concentrations
23647	Relative lipoprotein lipid concentrations
23648	Relative lipoprotein lipid concentrations
23431	Relative lipoprotein lipid concentrations
23432	Lipoprotein particle sizes
23433	Lipoprotein particle sizes
23400	Lipoprotein particle sizes
23403	Cholesterol
23405	Cholesterol

23402	Cholesterol
23415	Cholesterol
23419	Cholesterol

23407	Cholesteryl esters
23408	Free cholesterol
23409	Triglycerides
23410	Triglycerides
23434	Triglycerides
23435	Triglycerides
23437	Other lipids
23438	Other lipids
23436	Other lipids
23440	Other lipids
23439	Other lipids
23441	Apolipoproteins
23442	Apolipoproteins
23443	Apolipoproteins
23450	Fatty acids
23449	Fatty acids
23444	Fatty acids
23445	Fatty acids
23446	Fatty acids
23447	Fatty acids
23448	Fatty acids
23457	Fatty acids
23456	Fatty acids
23451	Fatty acids
23452	Fatty acids
23453	Fatty acids
23454	Fatty acids
23455	Fatty acids
23470	Fatty acids
23471	Fatty acids
23473	Glycolysis related metabolites
23460	Glycolysis related metabolites
23461	Glycolysis related metabolites
23463	Amino acids
23465	Amino acids
23466	Amino acids
23467	Amino acids
23468	Amino acids
23469	Amino acids

23475	Amino acids
23476	Amino acids
23474	Ketone bodies
23478	Ketone bodies
23479	Ketone bodies
23480	Fluid balance
23462	Fluid balance
23464	Inflammation
23401	Ketone bodies
23404	Cholesteryl esters
23417	Cholesteryl esters
23418	Cholesteryl esters
23458	Cholesterol
23459	Lipoprotein particle concentrations
23420	Lipoprotein particle concentrations
23421	Lipoprotein particle concentrations
23422	Free cholesterol
23472	Free cholesterol
23477	Free cholesterol
23427	Amino acids
23428	Phospholipids
23429	Phospholipids
23430	Phospholipids
23411	Glycolysis related metabolites
23412	Fatty acids
23413	Fatty acids
23414	Cholesterol
23423	Amino acids
23424	Lipoprotein particle concentrations
23425	Total lipids
23426	Total lipids
23416	Total lipids
23406	Total lipids
23584	Phospholipids

Supplementary Table 2. Association of sleep duration with all-

	CKB		
**Cause of death**	**Unadjusted HR (95% CI)**	**P value**	**Adjusted HR (95% CI)**
All causes	0.91 (0.91, 0.92)	**1.12E-156**	1.01 (1.00, 1.02)
Ischaemic heart diseases	0.90 (0.90, 0.91)	**1.42E-34**	1.02 (1.00, 1.04)
Cerebrovascular diseases	0.96 (0.96, 0.97)	**1.42E-08**	1.06 (1.04, 1.07)
Cancer	0.93 (0.93, 0.94)	**2.27E-37**	1.01 (0.99, 1.02)
Other	0.88 (0.87, 0.89)	**1.03E-103**	0.98 (0.97, 0.99)

Models were adjusted for age, sex, regions (CKB only), ethnicity (UKB only), levels of education, smoking status, al

HR, hazard ratio; CI, confidence interval.

cause and cause-specific mortality in the CKB and UKB

	UKB			
P value	Unadjusted HR (95% CI)	P value	Adjusted HR (95% CI)	P value
**8.69E-04**	1.08 (1.07, 1.09)	**1.68E-56**	1.03 (1.03, 1.04)	**1.84E-15**
**1.19E-02**	1.07 (1.04, 1.10)	**4.72E-06**	1.02 (1.00, 1.05)	6.42E-02
**1.71E-17**	1.10 (1.06, 1.15)	**1.28E-05**	1.04 (1.00, 1.09)	**4.00E-02**
3.59E-01	1.07 (1.05, 1.08)	**1.07E-21**	1.03 (1.02, 1.04)	**7.44E-06**
**2.36E-04**	1.09 (1.08, 1.11)	**1.94E-29**	1.04 (1.03, 1.06)	**8.39E-10**

cohol consumption, and self-rated health status.

Suppler

Metabolites	Group
Concentration of chylomicrons and extremely large VLDL particles	Lipoprotein subclasses
Total lipids in chylomicrons and extremely large VLDL	Lipoprotein subclasses
Phospholipids in chylomicrons and extremely large VLDL	Lipoprotein subclasses
Cholesterol in chylomicrons and extremely large VLDL	Lipoprotein subclasses
Cholesteryl esters in chylomicrons and extremely large VLDL	Lipoprotein subclasses
Free cholesterol in chylomicrons and extremely large VLDL	Lipoprotein subclasses
Triglycerides in chylomicrons and extremely large VLDL	Lipoprotein subclasses
Concentration of very large VLDL particles	Lipoprotein subclasses
Total lipids in very large VLDL	Lipoprotein subclasses
Phospholipids in very large VLDL	Lipoprotein subclasses
Cholesterol in very large VLDL	Lipoprotein subclasses
Cholesteryl esters in very large VLDL	Lipoprotein subclasses
Free cholesterol in very large VLDL	Lipoprotein subclasses
Triglycerides in very large VLDL	Lipoprotein subclasses
Concentration of large VLDL particles	Lipoprotein subclasses
Total lipids in large VLDL	Lipoprotein subclasses
Phospholipids in large VLDL	Lipoprotein subclasses
Cholesterol in large VLDL	Lipoprotein subclasses
Cholesteryl esters in large VLDL	Lipoprotein subclasses
Free cholesterol in large VLDL	Lipoprotein subclasses
Triglycerides in large VLDL	Lipoprotein subclasses
Concentration of medium VLDL particles	Lipoprotein subclasses
Total lipids in medium VLDL	Lipoprotein subclasses
Phospholipids in medium VLDL	Lipoprotein subclasses
Cholesterol in medium VLDL	Lipoprotein subclasses
Cholesteryl esters in medium VLDL	Lipoprotein subclasses
Free cholesterol in medium VLDL	Lipoprotein subclasses
Triglycerides in medium VLDL	Lipoprotein subclasses
Concentration of small VLDL particles	Lipoprotein subclasses
Total lipids in small VLDL	Lipoprotein subclasses
Phospholipids in small VLDL	Lipoprotein subclasses
Cholesterol in small VLDL	Lipoprotein subclasses
Cholesteryl esters in small VLDL	Lipoprotein subclasses
Free cholesterol in small VLDL	Lipoprotein subclasses
Triglycerides in small VLDL	Lipoprotein subclasses
Concentration of very small VLDL particles	Lipoprotein subclasses
Total lipids in very small VLDL	Lipoprotein subclasses
Phospholipids in very small VLDL	Lipoprotein subclasses
Cholesterol in very small VLDL	Lipoprotein subclasses
Cholesteryl esters in very small VLDL	Lipoprotein subclasses
Free cholesterol in very small VLDL	Lipoprotein subclasses
Triglycerides in very small VLDL	Lipoprotein subclasses
Concentration of IDL particles	Lipoprotein subclasses

Total lipids in IDL	Lipoprotein subclasses
Phospholipids in IDL	Lipoprotein subclasses
Cholesterol in IDL	Lipoprotein subclasses
Cholesteryl esters in IDL	Lipoprotein subclasses
Free cholesterol in IDL	Lipoprotein subclasses
Triglycerides in IDL	Lipoprotein subclasses
Concentration of large LDL particles	Lipoprotein subclasses
Total lipids in large LDL	Lipoprotein subclasses
Phospholipids in large LDL	Lipoprotein subclasses
Cholesterol in large LDL	Lipoprotein subclasses
Cholesteryl esters in large LDL	Lipoprotein subclasses
Free cholesterol in large LDL	Lipoprotein subclasses
Triglycerides in large LDL	Lipoprotein subclasses
Concentration of medium LDL particles	Lipoprotein subclasses
Total lipids in medium LDL	Lipoprotein subclasses
Phospholipids in medium LDL	Lipoprotein subclasses
Cholesterol in medium LDL	Lipoprotein subclasses
Cholesteryl esters in medium LDL	Lipoprotein subclasses
Free cholesterol in medium LDL	Lipoprotein subclasses
Triglycerides in medium LDL	Lipoprotein subclasses
Concentration of small LDL particles	Lipoprotein subclasses
Total lipids in small LDL	Lipoprotein subclasses
Phospholipids in small LDL	Lipoprotein subclasses
Cholesterol in small LDL	Lipoprotein subclasses
Cholesteryl esters in small LDL	Lipoprotein subclasses
Free cholesterol in small LDL	Lipoprotein subclasses
Triglycerides in small LDL	Lipoprotein subclasses
Concentration of very large HDL particles	Lipoprotein subclasses
Total lipids in very large HDL	Lipoprotein subclasses
Phospholipids in very large HDL	Lipoprotein subclasses
Cholesterol in very large HDL	Lipoprotein subclasses
Cholesteryl esters in very large HDL	Lipoprotein subclasses
Free cholesterol in very large HDL	Lipoprotein subclasses
Triglycerides in very large HDL	Lipoprotein subclasses
Concentration of large HDL particles	Lipoprotein subclasses
Total lipids in large HDL	Lipoprotein subclasses
Phospholipids in large HDL	Lipoprotein subclasses
Cholesterol in large HDL	Lipoprotein subclasses
Cholesteryl esters in large HDL	Lipoprotein subclasses
Free cholesterol in large HDL	Lipoprotein subclasses
Triglycerides in large HDL	Lipoprotein subclasses
**Concentration of medium HDL particles**	Lipoprotein subclasses
**Total lipids in medium HDL**	Lipoprotein subclasses
**Phospholipids in medium HDL**	Lipoprotein subclasses
**Cholesterol in medium HDL**	Lipoprotein subclasses
**Cholesteryl esters in medium HDL**	Lipoprotein subclasses
**Free cholesterol in medium HDL**	Lipoprotein subclasses
Triglycerides in medium HDL	Lipoprotein subclasses

Concentration of small HDL particles	Lipoprotein subclasses
Total lipids in small HDL	Lipoprotein subclasses
Phospholipids in small HDL	Lipoprotein subclasses
Cholesterol in small HDL	Lipoprotein subclasses
Cholesteryl esters in small HDL	Lipoprotein subclasses
**Free cholesterol in small HDL**	Lipoprotein subclasses
Triglycerides in small HDL	Lipoprotein subclasses
Phospholipids to total lipids ratio in chylomicrons and extremely large	Relative lipoprotein lipid concen
Cholesterol to total lipids ratio in chylomicrons and extremely large V	Relative lipoprotein lipid concen
Cholesteryl esters to total lipids ratio in chylomicrons and extremely la	Relative lipoprotein lipid concen
Free cholesterol to total lipids ratio in chylomicrons and extremely lar	Relative lipoprotein lipid concen
Triglycerides to total lipids ratio in chylomicrons and extremely large	Relative lipoprotein lipid concen
Phospholipids to total lipids ratio in very large VLDL /(lipds in ckb)	Relative lipoprotein lipid concen
**Cholesterol to total lipids ratio in very large VLDL**	Relative lipoprotein lipid concen
Cholesteryl esters to total lipids ratio in very large VLDL	Relative lipoprotein lipid concen
**Free cholesterol to total lipids ratio in very large VLDL**	Relative lipoprotein lipid concen
**Triglycerides to total lipids ratio in very large VLDL**	Relative lipoprotein lipid concen
Phospholipids to total lipids ratio in large VLDL	Relative lipoprotein lipid concen
Cholesterol to total lipids ratio in large VLDL	Relative lipoprotein lipid concen
Cholesteryl esters to total lipids ratio in large VLDL	Relative lipoprotein lipid concen
Free cholesterol to total lipids ratio in large VLDL	Relative lipoprotein lipid concen
Triglycerides to total lipids ratio in large VLDL	Relative lipoprotein lipid concen
Phospholipids to total lipids ratio in medium VLDL	Relative lipoprotein lipid concen
**Cholesterol to total lipids ratio in medium VLDL**	Relative lipoprotein lipid concen
**Cholesteryl esters to total lipids ratio in medium VLDL**	Relative lipoprotein lipid concen
Free cholesterol to total lipids ratio in medium VLDL	Relative lipoprotein lipid concen
Triglycerides to total lipids ratio in medium VLDL	Relative lipoprotein lipid concen
**Phospholipids to total lipids ratio in small VLDL**	Relative lipoprotein lipid concen
Cholesterol to total lipids ratio in small VLDL	Relative lipoprotein lipid concen
Cholesteryl esters to total lipids ratio in small VLDL	Relative lipoprotein lipid concen
**Free cholesterol to total lipids ratio in small VLDL**	Relative lipoprotein lipid concen
Triglycerides to total lipids ratio in small VLDL	Relative lipoprotein lipid concen
Phospholipids to total lipids ratio in very small VLDL	Relative lipoprotein lipid concen
Cholesterol to total lipids ratio in very small VLDL	Relative lipoprotein lipid concen
Cholesteryl esters to total lipids ratio in very small VLDL	Relative lipoprotein lipid concen
Free cholesterol to total lipids ratio in very small VLDL	Relative lipoprotein lipid concen
Triglycerides to total lipids ratio in very small VLDL	Relative lipoprotein lipid concen
Phospholipids to total lipids ratio in IDL	Relative lipoprotein lipid concen
Cholesterol to total lipids ratio in IDL	Relative lipoprotein lipid concen
Cholesteryl esters to total lipids ratio in IDL	Relative lipoprotein lipid concen
Free cholesterol to total lipids ratio in IDL	Relative lipoprotein lipid concen
Triglycerides to total lipids ratio in IDL	Relative lipoprotein lipid concen
Phospholipids to total lipids ratio in large LDL	Relative lipoprotein lipid concen
Cholesterol to total lipids ratio in large LDL	Relative lipoprotein lipid concen
Cholesteryl esters to total lipids ratio in large LDL	Relative lipoprotein lipid concen
Free cholesterol to total lipids ratio in large LDL	Relative lipoprotein lipid concen
Triglycerides to total lipids ratio in large LDL	Relative lipoprotein lipid concen
Phospholipids to total lipids ratio in medium LDL	Relative lipoprotein lipid concen

Cholesterol to total lipids ratio in medium LDL	Relative lipoprotein lipid concer
Cholesteryl esters to total lipids ratio in medium LDL	Relative lipoprotein lipid concer
Free cholesterol to total lipids ratio in medium LDL	Relative lipoprotein lipid concer
Triglycerides to total lipids ratio in medium LDL	Relative lipoprotein lipid concer
Phospholipids to total lipids ratio in small LDL	Relative lipoprotein lipid concer
Cholesterol to total lipids ratio in small LDL	Relative lipoprotein lipid concer
Cholesteryl esters to total lipids ratio in small LDL	Relative lipoprotein lipid concer
Free cholesterol to total lipids ratio in small LDL	Relative lipoprotein lipid concer
Triglycerides to total lipids ratio in small LDL	Relative lipoprotein lipid concer
Phospholipids to total lipids ratio in very large HDL	Relative lipoprotein lipid concer
Cholesterol to total lipids ratio in very large HDL	Relative lipoprotein lipid concer
Cholesteryl esters to total lipids ratio in very large HDL	Relative lipoprotein lipid concer
Free cholesterol to total lipids ratio in very large HDL	Relative lipoprotein lipid concer
Triglycerides to total lipids ratio in very large HDL	Relative lipoprotein lipid concer
Phospholipids to total lipids ratio in large HDL	Relative lipoprotein lipid concer
Cholesterol to total lipids ratio in large HDL	Relative lipoprotein lipid concer
Cholesteryl esters to total lipids ratio in large HDL	Relative lipoprotein lipid concer
Free cholesterol to total lipids ratio in large HDL	Relative lipoprotein lipid concer
Triglycerides to total lipids ratio in large HDL	Relative lipoprotein lipid concer
Phospholipids to total lipids ratio in medium HDL	Relative lipoprotein lipid concer
Cholesterol to total lipids ratio in medium HDL	Relative lipoprotein lipid concer
Cholesteryl esters to total lipids ratio in medium HDL	Relative lipoprotein lipid concer
Free cholesterol to total lipids ratio in medium HDL	Relative lipoprotein lipid concer
Triglycerides to total lipids ratio in medium HDL	Relative lipoprotein lipid concer
Phospholipids to total lipids ratio in small HDL	Relative lipoprotein lipid concer
Cholesterol to total lipids ratio in small HDL	Relative lipoprotein lipid concer
Cholesteryl esters to total lipids ratio in small HDL	Relative lipoprotein lipid concer
Free cholesterol to total lipids ratio in small HDL	Relative lipoprotein lipid concer
Triglycerides to total lipids ratio in small HDL	Relative lipoprotein lipid concer
Average diameter for VLDL particles	Lipoprotein particle sizes
Average diameter for LDL particles	Lipoprotein particle sizes
Average diameter for HDL particles	Lipoprotein particle sizes
Total cholesterol	Cholesterol
VLDL cholesterol	Cholesterol
Remnant cholesterol (non-HDL, non-LDL -cholesterol)	Cholesterol
LDL cholesterol	Cholesterol
**HDL cholesterol**	Cholesterol
**HDL2 cholesterol**	Cholesterol
HDL3 cholesterol	Cholesterol
Total esterified cholesterol	Cholesteryl esters
Total free cholesterol	Free cholesterol
Total triglycerides	Triglycerides
Triglycerides in VLDL	Triglycerides
Triglycerides in LDL	Triglycerides
Triglycerides in HDL	Triglycerides
Phosphoglycerides	Other lipids
Ratio of triglycerides to phosphoglycerides	Other lipids
Phosphatidylcholines	Other lipids

Sphingomyelins	Other lipids
Total cholines	Other lipids
Apolipoprotein A1	Apolipoproteins
Apolipoprotein B	Apolipoproteins
Ratio of apolipoprotein B to apolipoprotein A1	Apolipoproteins
Total fatty acids	Fatty acids
Degree of unsaturation	Fatty acids
Docosahexaenoic acid	Fatty acids
Linoleic acid	Fatty acids
Omega-3 fatty acids	Fatty acids
Omega-6 fatty acids	Fatty acids
Polyunsaturated fatty acids	Fatty acids
Monounsaturated fatty acids	Fatty acids
Saturated fatty acids	Fatty acids
Ratio of docosahexaenoic acid to total fatty acids	Fatty acids
Ratio of linoleic acid to total fatty acids	Fatty acids
Ratio of omega-3 fatty acids to total fatty acids	Fatty acids
Ratio of omega-6 fatty acids to total fatty acids	Fatty acids
Ratio of polyunsaturated fatty acids to total fatty acids	Fatty acids
Ratio of monounsaturated fatty acids to total fatty acids	Fatty acids
Ratio of saturated fatty acids to total fatty acids	Fatty acids
Glucose	Glycolysis related metabolites
Lactate	Glycolysis related metabolites
Citrate	Glycolysis related metabolites
Alanine	Amino acids
Glutamine	Amino acids
Histidine	Amino acids
Isoleucine	Amino acids
Leucine	Amino acids
Valine	Amino acids
Phenylalanine	Amino acids
Tyrosine	Amino acids
Acetate	Ketone bodies
Acetoacetate	Ketone bodies
**3-Hydroxybutyrate**	Ketone bodies
Creatinine	Fluid balance
Albumin	Fluid balance
Glycoprotein acetyls	Inflammation
Acetone	Ketone bodies
Cholesteryl esters in HDL	Cholesteryl esters
Cholesteryl esters in LDL	Cholesteryl esters
Cholesteryl esters in VLDL/ (Cholesterol in ckb)	Cholesteryl esters
Clinical LDL cholesterol	Cholesterol
Concentration of HDL particles	Lipoprotein particle concentratio
Concentration of LDL particles	Lipoprotein particle concentratio
Concentration of VLDL particles	Lipoprotein particle concentratio
Free cholesterol in HDL	Free cholesterol
Free cholesterol in LDL	Free cholesterol

Free cholesterol in VLDL	Free cholesterol
Glycine	Amino acids
Phospholipids in HDL	Phospholipids
Phospholipids in LDL	Phospholipids
Phospholipids in VLDL	Phospholipids
Pyruvate	Glycolysis related metabolites
Ratio of omega-6 fatty acids to omega-3 fatty acids	Fatty acids
Ratio of polyunsaturated fatty acids to monounsaturated fatty acids	Fatty acids
Total cholesterol minus HDL-C	Cholesterol
Total concentration of branched-chain amino acids (leucine + isoleuci	Amino acids
Total concentration of lipoprotein particles	Lipoprotein particle concentratic
Total lipids in HDL	Total lipids
Total lipids in LDL	Total lipids
Total lipids in lipoprotein particles	Total lipids
Total lipids in VLDL	Total lipids
Total phospholipids in lipoprotein particles	Phospholipids

Models were adjusted for age, sex, regions (CKB only), ethnicity (UKB only), levels of education, smoking statu

CI, confidence interval; LDL, Low Density Lipoprotein; HDL, High Density Lipoprotein; VLDL, Very Low De

mentary Table 3. Association of all metabolites with sleep duration in the CKB and UKB

CKB			
Unadjusted β (95% CI)	P value	Adjusted β (95% CI)	P value
-0.02 (-0.05, 0.01)	3.67E-01	0.01 (-0.03, 0.04)	8.65E-01
-0.02 (-0.05, 0.01)	3.69E-01	0.01 (-0.03, 0.04)	8.61E-01
-0.02 (-0.05, 0.01)	3.27E-01	0.00 (-0.03, 0.03)	9.69E-01
-0.01 (-0.05, 0.02)	4.91E-01	0.02 (-0.02, 0.05)	5.44E-01
-0.01 (-0.04, 0.02)	5.53E-01	0.03 (-0.01, 0.06)	2.31E-01
-0.02 (-0.05, 0.02)	3.97E-01	0.00 (-0.03, 0.03)	9.80E-01
-0.02 (-0.05, 0.01)	3.59E-01	0.00 (-0.03, 0.04)	9.01E-01
-0.02 (-0.05, 0.02)	4.14E-01	0.00 (-0.03, 0.03)	9.69E-01
-0.02 (-0.05, 0.02)	4.19E-01	0.00 (-0.03, 0.04)	9.44E-01
-0.02 (-0.05, 0.01)	3.57E-01	0.01 (-0.03, 0.04)	8.76E-01
-0.01 (-0.04, 0.02)	5.30E-01	0.01 (-0.02, 0.04)	6.62E-01
-0.01 (-0.04, 0.02)	6.80E-01	0.02 (-0.02, 0.05)	5.48E-01
-0.02 (-0.05, 0.01)	3.78E-01	0.01 (-0.02, 0.04)	8.21E-01
-0.02 (-0.05, 0.02)	4.01E-01	0.00 (-0.03, 0.03)	9.97E-01
-0.02 (-0.05, 0.02)	3.97E-01	0.00 (-0.03, 0.03)	9.78E-01
-0.02 (-0.05, 0.02)	3.90E-01	0.00 (-0.03, 0.03)	9.74E-01
-0.02 (-0.05, 0.01)	3.50E-01	0.00 (-0.03, 0.03)	9.69E-01
-0.02 (-0.05, 0.01)	3.59E-01	0.01 (-0.02, 0.04)	7.83E-01
-0.02 (-0.05, 0.01)	3.73E-01	0.02 (-0.02, 0.05)	5.11E-01
-0.02 (-0.05, 0.01)	3.57E-01	0.00 (-0.03, 0.03)	9.80E-01
-0.02 (-0.05, 0.02)	4.14E-01	0.00 (-0.03, 0.03)	9.97E-01
-0.02 (-0.05, 0.01)	3.45E-01	0.01 (-0.02, 0.04)	8.16E-01
-0.02 (-0.05, 0.01)	3.30E-01	0.01 (-0.02, 0.04)	7.83E-01
-0.02 (-0.05, 0.01)	2.77E-01	0.01 (-0.02, 0.04)	7.68E-01
-0.02 (-0.06, 0.01)	2.11E-01	0.02 (-0.01, 0.05)	3.92E-01
-0.03 (-0.06, 0.01)	1.82E-01	0.03 (0.00, 0.06)	1.54E-01
-0.02 (-0.05, 0.01)	2.74E-01	0.01 (-0.03, 0.04)	8.61E-01
-0.02 (-0.05, 0.02)	3.99E-01	0.00 (-0.03, 0.03)	9.44E-01
-0.04 (-0.07, -0.01)	5.26E-02	0.01 (-0.02, 0.05)	5.95E-01
-0.04 (-0.07, -0.01)	**4.71E-02**	0.02 (-0.02, 0.05)	5.48E-01
-0.05 (-0.08, -0.02)	**1.04E-02**	0.01 (-0.02, 0.04)	7.90E-01
-0.03 (-0.07, 0.00)	7.44E-02	0.03 (0.00, 0.07)	1.54E-01
-0.03 (-0.06, 0.01)	2.04E-01	0.04 (0.01, 0.08)	6.56E-02
-0.04 (-0.08, -0.01)	**1.70E-02**	0.01 (-0.02, 0.04)	7.65E-01
-0.03 (-0.06, 0.00)	1.03E-01	0.01 (-0.03, 0.04)	8.61E-01
-0.06 (-0.09, -0.03)	**1.54E-03**	0.02 (-0.01, 0.05)	4.21E-01
-0.06 (-0.09, -0.03)	**2.31E-03**	0.02 (-0.01, 0.05)	3.68E-01
-0.07 (-0.10, -0.04)	**4.51E-04**	0.02 (-0.01, 0.05)	4.55E-01
-0.03 (-0.07, 0.00)	7.37E-02	0.03 (0.00, 0.06)	2.03E-01
-0.03 (-0.06, 0.00)	1.68E-01	0.03 (0.00, 0.07)	1.42E-01
-0.05 (-0.08, -0.02)	**1.00E-02**	0.02 (-0.02, 0.05)	5.44E-01
-0.06 (-0.09, -0.02)	**2.37E-03**	0.00 (-0.03, 0.03)	9.78E-01
-0.06 (-0.09, -0.03)	**1.89E-03**	0.03 (-0.01, 0.06)	2.57E-01

-0.06 (-0.09, -0.03)	**2.31E-03**	0.03 (-0.01, 0.06)	2.31E-01
-0.06 (-0.09, -0.03)	**2.31E-03**	0.03 (0.00, 0.06)	2.13E-01
-0.05 (-0.08, -0.02)	**6.81E-03**	0.03 (0.00, 0.06)	2.14E-01
-0.05 (-0.08, -0.02)	**7.49E-03**	0.03 (0.00, 0.06)	1.95E-01
-0.05 (-0.08, -0.02)	**7.58E-03**	0.02 (-0.01, 0.06)	3.25E-01
-0.07 (-0.10, -0.04)	**1.45E-04**	-0.01 (-0.04, 0.02)	7.74E-01
-0.06 (-0.09, -0.03)	**2.17E-03**	0.03 (0.00, 0.06)	2.17E-01
-0.06 (-0.09, -0.02)	**2.35E-03**	0.03 (0.00, 0.06)	2.11E-01
-0.06 (-0.09, -0.03)	**2.16E-03**	0.03 (0.00, 0.06)	2.10E-01
-0.05 (-0.08, -0.02)	**5.81E-03**	0.03 (0.00, 0.06)	1.78E-01
-0.05 (-0.08, -0.02)	**5.81E-03**	0.03 (0.00, 0.07)	1.60E-01
-0.05 (-0.08, -0.02)	**6.95E-03**	0.03 (-0.01, 0.06)	2.33E-01
-0.07 (-0.10, -0.04)	**2.17E-04**	-0.01 (-0.04, 0.02)	8.20E-01
-0.06 (-0.09, -0.03)	**2.31E-03**	0.03 (0.00, 0.06)	2.13E-01
-0.06 (-0.09, -0.02)	**2.82E-03**	0.03 (0.00, 0.06)	2.11E-01
-0.06 (-0.09, -0.03)	**2.17E-03**	0.03 (0.00, 0.06)	2.11E-01
-0.05 (-0.08, -0.02)	**7.10E-03**	0.03 (0.00, 0.06)	1.95E-01
-0.05 (-0.08, -0.02)	**8.42E-03**	0.03 (0.00, 0.06)	1.82E-01
-0.05 (-0.08, -0.02)	**5.74E-03**	0.02 (-0.01, 0.06)	2.84E-01
-0.07 (-0.10, -0.04)	**1.45E-04**	0.00 (-0.04, 0.03)	9.17E-01
-0.06 (-0.09, -0.03)	**2.28E-03**	0.02 (-0.01, 0.06)	3.22E-01
-0.06 (-0.09, -0.03)	**2.31E-03**	0.02 (-0.01, 0.06)	3.22E-01
-0.06 (-0.09, -0.03)	**1.08E-03**	0.02 (-0.02, 0.05)	5.60E-01
-0.05 (-0.08, -0.02)	**6.97E-03**	0.03 (-0.01, 0.06)	2.75E-01
-0.05 (-0.08, -0.02)	**6.10E-03**	0.02 (-0.01, 0.06)	2.93E-01
-0.05 (-0.09, -0.02)	**4.14E-03**	0.02 (-0.02, 0.05)	5.50E-01
-0.05 (-0.09, -0.02)	**3.78E-03**	0.01 (-0.03, 0.04)	8.39E-01
-0.03 (-0.06, 0.00)	1.44E-01	-0.03 (-0.06, 0.00)	1.54E-01
-0.03 (-0.06, 0.00)	1.48E-01	-0.03 (-0.06, 0.00)	1.54E-01
-0.03 (-0.06, 0.00)	1.02E-01	-0.03 (-0.06, 0.00)	1.54E-01
-0.02 (-0.05, 0.01)	2.70E-01	-0.03 (-0.06, 0.00)	1.48E-01
-0.02 (-0.05, 0.01)	3.36E-01	-0.03 (-0.06, 0.00)	1.58E-01
-0.03 (-0.06, 0.00)	1.35E-01	-0.04 (-0.07, 0.00)	1.08E-01
-0.01 (-0.04, 0.02)	5.32E-01	0.00 (-0.03, 0.04)	8.87E-01
-0.03 (-0.06, 0.00)	1.17E-01	-0.04 (-0.07, -0.01)	6.63E-02
-0.03 (-0.06, 0.00)	1.28E-01	-0.04 (-0.08, -0.01)	6.63E-02
-0.03 (-0.06, 0.00)	9.65E-02	-0.04 (-0.07, -0.01)	6.63E-02
-0.03 (-0.06, 0.01)	1.95E-01	-0.04 (-0.08, -0.01)	6.56E-02
-0.03 (-0.06, 0.00)	1.70E-01	-0.05 (-0.08, -0.01)	6.56E-02
-0.02 (-0.05, 0.01)	2.81E-01	-0.04 (-0.07, -0.01)	6.72E-02
-0.03 (-0.06, 0.00)	9.60E-02	0.00 (-0.04, 0.03)	9.80E-01
-0.06 (-0.09, -0.03)	**2.31E-03**	-0.05 (-0.08, -0.02)	**4.50E-02**
-0.06 (-0.09, -0.02)	**2.71E-03**	-0.05 (-0.08, -0.02)	**4.50E-02**
-0.06 (-0.09, -0.03)	**2.31E-03**	-0.05 (-0.08, -0.02)	**4.50E-02**
-0.05 (-0.08, -0.01)	**1.21E-02**	-0.05 (-0.09, -0.02)	**4.10E-02**
-0.04 (-0.08, -0.01)	**1.91E-02**	-0.05 (-0.09, -0.02)	**4.10E-02**
-0.05 (-0.09, -0.02)	**3.46E-03**	-0.05 (-0.09, -0.02)	**4.10E-02**
-0.05 (-0.08, -0.02)	**6.66E-03**	0.01 (-0.02, 0.05)	5.77E-01

-0.07 (-0.10, -0.03)	**6.59E-04**	-0.03 (-0.06, 0.00)	1.78E-01
-0.07 (-0.10, -0.03)	**6.31E-04**	-0.03 (-0.06, 0.00)	1.60E-01
-0.03 (-0.07, 0.00)	7.36E-02	-0.04 (-0.07, 0.00)	1.09E-01
-0.06 (-0.09, -0.03)	**2.29E-03**	-0.01 (-0.04, 0.02)	7.79E-01
-0.05 (-0.08, -0.02)	**6.98E-03**	0.00 (-0.03, 0.03)	9.95E-01
-0.04 (-0.07, -0.01)	**2.92E-02**	-0.05 (-0.08, -0.02)	**4.50E-02**
-0.03 (-0.07, 0.00)	7.37E-02	0.01 (-0.02, 0.04)	8.39E-01
-0.02 (-0.05, 0.01)	3.33E-01	-0.01 (-0.04, 0.02)	7.59E-01
-0.02 (-0.01, 0.05)	3.58E-01	0.03 (0.00, 0.06)	1.60E-01
-0.01 (-0.02, 0.05)	5.17E-01	0.03 (0.00, 0.07)	1.37E-01
-0.02 (-0.01, 0.05)	3.19E-01	0.01 (-0.03, 0.04)	8.84E-01
-0.01 (-0.04, 0.02)	6.71E-01	-0.02 (-0.06, 0.01)	2.93E-01
-0.02 (-0.05, 0.01)	3.56E-01	0.03 (0.00, 0.06)	1.95E-01
0.03 (0.00, 0.07)	1.02E-01	0.07 (0.04, 0.10)	**4.94E-03**
0.04 (0.00, 0.07)	7.77E-02	0.04 (0.01, 0.08)	6.63E-02
0.00 (-0.03, 0.04)	8.22E-01	0.05 (0.02, 0.08)	**4.50E-02**
-0.01 (-0.05, 0.02)	5.17E-01	-0.07 (-0.10, -0.04)	**4.94E-03**
-0.04 (-0.07, -0.01)	**2.70E-02**	0.01 (-0.02, 0.05)	5.99E-01
-0.02 (-0.05, 0.02)	4.01E-01	0.04 (0.01, 0.07)	6.63E-02
-0.02 (-0.05, 0.02)	3.97E-01	0.04 (0.01, 0.08)	6.63E-02
0.00 (-0.04, 0.03)	8.38E-01	0.01 (-0.02, 0.04)	6.95E-01
0.02 (-0.01, 0.06)	2.36E-01	-0.04 (-0.07, -0.01)	1.08E-01
-0.06 (-0.10, -0.03)	**7.37E-04**	0.00 (-0.03, 0.03)	9.97E-01
-0.04 (-0.07, 0.00)	5.55E-02	0.05 (0.02, 0.08)	**4.50E-02**
-0.03 (-0.06, 0.00)	1.02E-01	0.05 (0.02, 0.08)	**4.50E-02**
-0.01 (-0.05, 0.02)	4.73E-01	0.00 (-0.03, 0.03)	9.78E-01
-0.04 (-0.01, 0.08)	**2.10E-02**	-0.05 (-0.08, -0.01)	6.56E-02
-0.05 (-0.08, -0.02)	**5.74E-03**	-0.06 (-0.09, -0.03)	**7.61E-03**
-0.00 (-0.03, 0.04)	8.41E-01	0.03 (0.00, 0.06)	1.60E-01
-0.01 (-0.02, 0.04)	5.84E-01	0.04 (0.01, 0.07)	6.94E-02
-0.05 (-0.08, -0.01)	**1.40E-02**	-0.05 (-0.08, -0.02)	**4.50E-02**
-0.01 (-0.02, 0.04)	6.59E-01	-0.02 (-0.05, 0.02)	5.31E-01
-0.04 (-0.07, -0.01)	**2.70E-02**	0.01 (-0.03, 0.04)	8.76E-01
0.05 (0.02, 0.09)	**3.41E-03**	0.03 (0.00, 0.07)	1.39E-01
0.06 (0.03, 0.09)	**1.54E-03**	0.04 (0.01, 0.07)	7.74E-02
0.02 (-0.02, 0.05)	4.22E-01	0.01 (-0.03, 0.04)	8.76E-01
-0.02 (-0.05, 0.01)	2.77E-01	-0.03 (-0.06, 0.00)	1.95E-01
0.03 (0.00, 0.06)	1.02E-01	0.01 (-0.02, 0.04)	6.12E-01
0.03 (-0.01, 0.06)	2.11E-01	0.04 (0.01, 0.07)	8.83E-02
0.02 (-0.01, 0.05)	3.44E-01	0.03 (0.00, 0.06)	1.54E-01
0.01 (-0.02, 0.05)	4.50E-01	0.02 (-0.01, 0.05)	4.42E-01
-0.03 (-0.07, 0.00)	7.70E-02	-0.04 (-0.07, -0.01)	6.63E-02
0.03 (0.00, 0.06)	1.67E-01	-0.03 (-0.06, 0.00)	1.95E-01
0.01 (-0.03, 0.04)	7.81E-01	0.04 (0.01, 0.07)	6.63E-02
-0.01 (-0.04, 0.02)	6.44E-01	0.04 (0.01, 0.08)	6.56E-02
0.04 (0.01, 0.07)	**4.12E-02**	0.00 (-0.03, 0.03)	9.80E-01
-0.03 (-0.06, 0.00)	1.23E-01	-0.04 (-0.08, -0.01)	6.63E-02
0.02 (-0.02, 0.05)	4.01E-01	-0.03 (-0.06, 0.01)	2.58E-01

0.00 (-0.03, 0.03)	9.63E-01	0.03 (0.00, 0.07)	1.54E-01
-0.01 (-0.04, 0.02)	5.30E-01	0.04 (0.00, 0.07)	1.22E-01
0.04 (0.01, 0.07)	**3.17E-02**	-0.04 (-0.07, 0.00)	1.16E-01
-0.03 (-0.06, 0.00)	1.37E-01	-0.04 (-0.07, -0.01)	6.63E-02
0.01 (-0.02, 0.05)	4.47E-01	-0.03 (-0.06, 0.00)	1.63E-01
0.00 (-0.04, 0.03)	8.03E-01	0.03 (0.00, 0.06)	1.95E-01
-0.01 (-0.04, 0.02)	5.41E-01	0.03 (0.00, 0.07)	1.54E-01
0.04 (0.01, 0.07)	5.25E-02	-0.04 (-0.07, -0.01)	7.09E-02
-0.01 (-0.05, 0.02)	4.36E-01	-0.02 (-0.05, 0.01)	3.76E-01
-0.01 (-0.05, 0.02)	4.91E-01	0.00 (-0.03, 0.04)	9.69E-01
0.02 (-0.01, 0.05)	3.90E-01	-0.01 (-0.04, 0.02)	6.93E-01
0.02 (-0.01, 0.05)	3.16E-01	0.00 (-0.04, 0.03)	9.21E-01
-0.01 (-0.04, 0.03)	7.62E-01	-0.04 (-0.07, 0.00)	1.15E-01
0.00 (-0.04, 0.03)	8.22E-01	0.02 (-0.01, 0.05)	3.64E-01
-0.01 (-0.04, 0.02)	5.54E-01	0.02 (-0.01, 0.05)	3.49E-01
0.02 (-0.01, 0.05)	3.30E-01	-0.04 (-0.07, -0.01)	8.83E-02
0.01 (-0.02, 0.05)	4.34E-01	-0.05 (-0.08, -0.01)	6.22E-02
0.02 (-0.01, 0.05)	2.70E-01	-0.02 (-0.05, 0.01)	3.56E-01
-0.02 (-0.05, 0.01)	3.69E-01	0.04 (0.00, 0.07)	1.09E-01
0.00 (-0.03, 0.04)	8.33E-01	0.01 (-0.02, 0.05)	5.95E-01
0.01 (-0.02, 0.04)	5.99E-01	-0.03 (-0.07, 0.00)	1.36E-01
0.02 (-0.01, 0.05)	2.67E-01	-0.03 (-0.06, 0.01)	2.31E-01
-0.04 (-0.07, -0.01)	**4.52E-02**	-0.05 (-0.08, -0.01)	5.50E-02
-0.02 (-0.05, 0.01)	3.58E-01	0.04 (0.01, 0.07)	6.63E-02
0.03 (0.00, 0.06)	1.65E-01	-0.02 (-0.06, 0.01)	3.24E-01
-0.02 (-0.05, 0.01)	3.27E-01	0.01 (-0.02, 0.05)	6.03E-01
-0.02 (-0.05, 0.01)	3.00E-01	0.02 (-0.01, 0.05)	4.77E-01
0.03 (-0.01, 0.06)	2.04E-01	-0.05 (-0.08, -0.01)	6.56E-02
-0.01 (-0.04, 0.02)	5.31E-01	0.02 (-0.01, 0.05)	4.38E-01
0.02 (-0.01, 0.05)	2.98E-01	0.01 (-0.02, 0.04)	8.43E-01
0.03 (0.00, 0.06)	1.32E-01	0.03 (-0.01, 0.06)	2.60E-01
-0.01 (-0.04, 0.02)	6.80E-01	-0.03 (-0.06, 0.00)	2.11E-01
-0.06 (-0.10, -0.03)	**7.37E-04**	0.01 (-0.02, 0.04)	6.84E-01
-0.03 (-0.06, 0.00)	1.25E-01	0.03 (0.00, 0.06)	2.26E-01
-0.04 (-0.07, -0.01)	**2.25E-02**	0.03 (0.00, 0.07)	1.54E-01
-0.05 (-0.08, -0.02)	**5.81E-03**	0.03 (0.00, 0.06)	2.19E-01
-0.05 (-0.08, -0.02)	**1.02E-02**	-0.05 (-0.08, -0.02)	**4.10E-02**
-0.05 (-0.08, -0.02)	**9.35E-03**	-0.05 (-0.08, -0.02)	**4.10E-02**
-0.03 (-0.06, 0.00)	1.01E-01	-0.04 (-0.07, -0.01)	9.71E-02
-0.09 (-0.12, -0.06)	**9.74E-07**	0.01 (-0.02, 0.04)	7.83E-01
-0.08 (-0.11, -0.04)	**5.92E-05**	0.02 (-0.02, 0.05)	5.77E-01
-0.03 (-0.06, 0.00)	1.22E-01	0.00 (-0.03, 0.03)	9.91E-01
-0.02 (-0.05, 0.01)	3.00E-01	0.00 (-0.03, 0.03)	9.78E-01
-0.08 (-0.11, -0.05)	**4.12E-05**	-0.01 (-0.05, 0.02)	6.39E-01
-0.04 (-0.07, -0.01)	**2.36E-02**	0.01 (-0.02, 0.04)	8.16E-01
-0.08 (-0.11, -0.05)	**4.12E-05**	-0.01 (-0.05, 0.02)	6.40E-01
0.00 (-0.03, 0.04)	8.50E-01	0.01 (-0.02, 0.04)	6.12E-01
-0.09 (-0.12, -0.06)	**5.91E-07**	-0.02 (-0.05, 0.02)	5.11E-01

-0.10 (-0.13, -0.06)	**1.95E-07**	0.00 (-0.04, 0.03)	9.80E-01
-0.09 (-0.13, -0.06)	**5.04E-07**	-0.02 (-0.05, 0.02)	5.50E-01
-0.06 (-0.09, -0.03)	**2.31E-03**	-0.04 (-0.07, -0.01)	6.94E-02
-0.05 (-0.08, -0.02)	**5.81E-03**	0.03 (-0.01, 0.06)	2.70E-01
-0.02 (-0.06, 0.01)	2.28E-01	0.04 (0.01, 0.08)	6.56E-02
-0.07 (-0.10, -0.04)	**1.11E-04**	0.00 (-0.03, 0.03)	9.91E-01
-0.06 (-0.09, -0.03)	**1.01E-03**	0.02 (-0.01, 0.06)	3.46E-01
0.00 (-0.04, 0.03)	8.35E-01	0.02 (-0.01, 0.05)	3.45E-01
-0.09 (-0.12, -0.05)	**2.88E-06**	0.00 (-0.03, 0.04)	9.69E-01
-0.04 (-0.07, -0.01)	**4.08E-02**	0.02 (-0.01, 0.05)	3.80E-01
-0.10 (-0.13, -0.07)	**1.95E-07**	0.00 (-0.03, 0.04)	9.73E-01
-0.09 (-0.12, -0.06)	**7.27E-07**	0.01 (-0.03, 0.04)	8.61E-01
-0.06 (-0.09, -0.03)	**2.17E-03**	-0.01 (-0.04, 0.02)	7.84E-01
-0.06 (-0.09, -0.03)	**1.53E-03**	0.00 (-0.03, 0.03)	9.95E-01
0.06 (0.03, 0.09)	**9.65E-04**	0.03 (0.00, 0.06)	2.20E-01
-0.04 (-0.07, 0.00)	6.03E-02	0.01 (-0.02, 0.04)	7.72E-01
0.03 (-0.01, 0.06)	2.04E-01	0.04 (0.01, 0.07)	8.35E-02
-0.06 (-0.09, -0.03)	**2.29E-03**	0.01 (-0.03, 0.04)	8.39E-01
-0.05 (-0.08, -0.02)	**6.27E-03**	0.02 (-0.01, 0.05)	4.21E-01
0.02 (-0.02, 0.05)	4.22E-01	-0.02 (-0.05, 0.01)	3.22E-01
0.06 (0.03, 0.10)	**7.37E-04**	0.00 (-0.03, 0.03)	9.97E-01
-0.04 (-0.07, -0.01)	**4.71E-02**	0.00 (-0.03, 0.03)	9.80E-01
0.00 (-0.03, 0.03)	9.36E-01	-0.01 (-0.04, 0.03)	8.86E-01
-0.09 (-0.12, -0.06)	**1.20E-06**	-0.01 (-0.05, 0.02)	6.39E-01
0.00 (-0.03, 0.03)	9.25E-01	0.01 (-0.02, 0.04)	8.16E-01
0.02 (-0.01, 0.05)	3.74E-01	0.00 (-0.03, 0.03)	9.80E-01
0.09 (0.06, 0.12)	**5.91E-07**	0.03 (0.00, 0.06)	1.54E-01
-0.02 (-0.05, 0.02)	4.22E-01	0.01 (-0.02, 0.04)	6.50E-01
0.00 (-0.03, 0.03)	8.77E-01	0.01 (-0.02, 0.05)	6.39E-01
-0.04 (-0.07, -0.01)	**4.90E-02**	0.01 (-0.02, 0.04)	8.16E-01
0.04 (0.01, 0.07)	5.26E-02	0.04 (0.01, 0.07)	6.94E-02
-0.01 (-0.04, 0.02)	5.64E-01	0.02 (-0.01, 0.05)	3.26E-01
0.00 (-0.04, 0.03)	8.33E-01	0.00 (-0.03, 0.03)	9.97E-01
-0.08 (-0.11, -0.04)	**5.79E-05**	-0.03 (-0.07, 0.00)	1.54E-01
-0.11 (-0.14, -0.07)	**1.82E-08**	-0.05 (-0.08, -0.02)	**4.50E-02**
-0.03 (-0.06, 0.00)	1.01E-01	-0.01 (-0.04, 0.03)	8.43E-01
-0.02 (-0.05, 0.01)	2.70E-01	-0.02 (-0.05, 0.01)	3.48E-01
-0.01 (-0.04, 0.03)	7.21E-01	0.01 (-0.02, 0.04)	6.91E-01

ons
ons
ons

ons

s, alcohol consumption, and self-rated health status. P values are corrected for multiple testing using a false
nsity Lipoprotein; IDL, Intermediate Density Lipoprotein..

UKB			
**Unadjusted β (95% CI)**	**P value**	**Adjusted β (95% CI)**	**P value**
0.01 (0.01, 0.01)	**2.04E-06**	0.02 (0.02, 0.02)	**6.07E-18**
0.01 (0.01, 0.00)	**2.71E-05**	0.02 (0.01, 0.02)	**2.29E-16**
0.01 (0.01, 0.01)	**2.51E-06**	0.02 (0.02, 0.02)	**1.19E-18**
0.01 (0.00, 0.01)	**3.11E-05**	0.02 (0.01, 0.02)	**7.97E-16**
0.01 (0.00, 0.01)	**6.16E-04**	0.02 (0.01, 0.02)	**1.05E-13**
0.01 (0.01, 0.02)	**5.57E-07**	0.02 (0.02, 0.02)	**3.67E-18**
0.01 (0.00, 0.01)	**5.34E-05**	0.02 (0.01, 0.02)	**1.34E-15**
0.01 (0.00, 0.01)	**1.21E-04**	0.02 (0.01, 0.02)	**6.77E-13**
0.01 (0.00, 0.01)	**1.77E-04**	0.02 (0.01, 0.02)	**1.81E-12**
0.01 (0.00, 0.01)	**6.74E-05**	0.02 (0.01, 0.02)	**9.86E-13**
0.01 (0.00, 0.01)	**3.01E-03**	0.01 (0.01, 0.02)	**1.51E-07**
0.00 (0.00, 0.01)	9.73E-02	0.01 (0.00, 0.01)	**6.19E-04**
0.01 (0.01, 0.01)	**2.90E-05**	0.02 (0.01, 0.02)	**3.55E-12**
0.01 (0.00, 0.01)	**1.19E-04**	0.02 (0.01, 0.02)	**8.84E-14**
0.01 (0.00, 0.01)	**1.23E-04**	0.01 (0.01, 0.02)	**4.88E-11**
0.01 (0.00, 0.01)	**7.93E-04**	0.01 (0.01, 0.02)	**2.21E-09**
0.01 (0.00, 0.01)	**2.17E-04**	0.02 (0.01, 0.02)	**1.35E-11**
0.01 (0.00, 0.01)	**1.94E-04**	0.01 (0.01, 0.02)	**7.16E-08**
0.01 (0.00, 0.01)	**3.75E-05**	0.01 (0.01, 0.01)	**3.87E-06**
0.01 (0.00, 0.01)	**1.13E-03**	0.01 (0.01, 0.02)	**2.09E-09**
0.01 (0.00, 0.01)	**3.51E-03**	0.01 (0.01, 0.02)	**8.33E-09**
0.00 (0.00, 0.01)	1.14E-01	0.00 (0.00, 0.00)	7.58E-01
0.01 (0.00, 0.01)	**1.86E-02**	0.00 (0.00, 0.01)	8.63E-02
0.00 (0.00, 0.01)	8.43E-02	0.00 (0.00, 0.00)	9.45E-01
0.00 (0.00, 0.00)	8.19E-01	-0.01 (-0.01, 0.00)	**6.30E-05**
0.00 (-0.01, 0.00)	1.06E-01	-0.01 (-0.02, -0.01)	**9.61E-10**
0.00 (0.00, 0.01)	1.22E-01	0.00 (-0.01, 0.00)	3.03E-01
0.01 (0.00, 0.01)	**3.45E-04**	0.01 (0.01, 0.02)	**1.06E-07**
0.01 (0.01, 0.02)	**2.55E-10**	0.01 (0.01, 0.02)	**7.40E-10**
0.01 (0.01, 0.02)	**5.37E-09**	0.01 (0.01, 0.02)	**1.47E-07**
0.01 (0.00, 0.01)	**1.53E-03**	0.00 (0.00, 0.01)	1.50E-01
0.01 (0.00, 0.01)	**8.57E-03**	0.00 (0.00, 0.01)	4.74E-01
0.01 (0.00, 0.01)	**5.63E-04**	0.00 (0.00, 0.01)	**4.78E-02**
0.00 (0.00, 0.01)	2.54E-01	0.00 (-0.01, 0.00)	1.42E-01
0.02 (0.01, 0.02)	**2.42E-18**	0.02 (0.02, 0.03)	**1.70E-23**
0.02 (0.01, 0.02)	**1.48E-17**	0.01 (0.00, 0.01)	**7.01E-04**
0.02 (0.02, 0.03)	**5.95E-22**	0.01 (0.01, 0.01)	**1.95E-05**
0.02 (0.02, 0.03)	**5.05E-26**	0.01 (0.01, 0.02)	**3.28E-09**
0.01 (0.01, 0.02)	**3.40E-10**	0.00 (-0.01, 0.00)	6.26E-01
0.01 (0.01, 0.02)	**1.87E-07**	0.00 (-0.01, 0.00)	5.45E-02
0.02 (0.01, 0.02)	**1.30E-17**	0.01 (0.00, 0.01)	**4.71E-03**
0.03 (0.02, 0.03)	**1.47E-33**	0.03 (0.02, 0.03)	**7.76E-30**
0.00 (-0.01, 0.00)	7.99E-02	-0.01 (-0.02, -0.01)	**2.25E-11**

0.00 (-0.01, 0.00)	6.28E-01	-0.02 (-0.02, -0.01)	**2.40E-14**
0.00 (0.00, 0.01)	1.38E-01	-0.01 (-0.02, -0.01)	**2.73E-08**
-0.01 (-0.01, 0.00)	**1.18E-02**	-0.02 (-0.03, -0.02)	**1.29E-22**
-0.01 (-0.01, 0.00)	**3.74E-03**	-0.02 (-0.03, -0.02)	**1.37E-23**
0.00 (-0.01, 0.00)	1.66E-01	-0.02 (-0.02, -0.02)	**5.19E-19**
0.02 (0.02, 0.03)	**2.43E-29**	0.02 (0.02, 0.02)	**1.09E-20**
0.00 (-0.01, 0.00)	2.15E-01	-0.01 (-0.01, -0.01)	**1.44E-05**
-0.01 (-0.01, 0.00)	**4.93E-05**	-0.02 (-0.02, -0.02)	**8.62E-19**
-0.01 (-0.02, -0.01)	**3.85E-07**	-0.02 (-0.03, -0.02)	**1.01E-21**
-0.01 (-0.01, -0.01)	**2.77E-06**	-0.02 (-0.03, -0.02)	**4.06E-22**
-0.01 (-0.01, -0.01)	**2.48E-05**	-0.02 (-0.02, -0.02)	**4.41E-19**
-0.01 (-0.02, -0.01)	**6.16E-09**	-0.03 (-0.03, -0.02)	**1.84E-30**
0.02 (0.01, 0.02)	**7.46E-17**	0.02 (0.01, 0.02)	**8.32E-13**
0.00 (-0.01, 0.00)	1.24E-01	-0.01 (-0.01, 0.00)	**1.75E-04**
-0.01 (-0.01, -0.01)	**1.63E-06**	-0.01 (-0.02, -0.01)	**2.88E-11**
-0.01 (-0.02, -0.01)	**1.93E-09**	-0.02 (-0.02, -0.01)	**5.91E-14**
-0.01 (-0.02, -0.01)	**1.87E-07**	-0.02 (-0.02, -0.01)	**2.42E-13**
-0.01 (-0.01, 0.00)	**3.62E-05**	-0.01 (-0.02, -0.01)	**6.72E-09**
-0.02 (-0.02, -0.01)	**4.72E-15**	-0.02 (-0.03, -0.02)	**5.52E-28**
0.01 (0.01, 0.02)	**8.05E-12**	0.02 (0.01, 0.02)	**1.02E-13**
0.00 (0.00, 0.00)	8.44E-01	-0.01 (-0.01, 0.00)	**1.72E-02**
-0.01 (-0.01, 0.00)	**2.56E-04**	-0.01 (-0.02, -0.01)	**3.33E-08**
-0.01 (-0.01, 0.00)	**1.17E-03**	-0.01 (-0.02, -0.01)	**9.13E-09**
-0.01 (-0.02, -0.01)	**7.99E-07**	-0.01 (-0.02, -0.01)	**7.56E-12**
-0.01 (-0.01, 0.00)	**1.91E-04**	-0.01 (-0.02, -0.01)	**2.01E-07**
-0.02 (-0.02, -0.01)	**2.58E-14**	-0.02 (-0.03, -0.02)	**1.21E-25**
0.02 (0.01, 0.02)	**1.66E-12**	0.02 (0.02, 0.02)	**1.75E-18**
0.02 (0.01, 0.02)	**1.35E-15**	0.00 (-0.01, 0.00)	4.52E-01
0.02 (0.01, 0.02)	**6.35E-15**	0.00 (-0.01, 0.01)	5.13E-01
0.02 (0.01, 0.02)	**2.96E-17**	0.00 (0.00, 0.00)	9.14E-01
0.01 (0.01, 0.02)	**5.27E-09**	-0.01 (-0.01, 0.00)	**9.15E-03**
0.01 (0.01, 0.02)	**6.75E-08**	-0.01 (-0.01, 0.00)	**8.90E-04**
0.02 (0.01, 0.02)	**3.30E-13**	0.00 (0.00, 0.00)	9.45E-01
0.03 (0.03, 0.04)	**1.40E-48**	0.02 (0.02, 0.03)	**1.12E-27**
0.01 (0.01, 0.02)	**5.81E-07**	-0.01 (-0.02, -0.01)	**2.63E-06**
0.01 (0.01, 0.02)	**6.66E-07**	-0.01 (-0.02, -0.01)	**4.80E-06**
0.01 (0.01, 0.02)	**1.92E-07**	-0.01 (-0.02, -0.01)	**1.91E-05**
0.01 (0.00, 0.01)	**1.68E-04**	-0.01 (-0.02, -0.01)	**1.66E-08**
0.01 (0.00, 0.01)	**1.07E-03**	-0.01 (-0.02, -0.01)	**1.24E-09**
0.01 (0.01, 0.02)	**5.89E-08**	-0.01 (-0.01, -0.01)	**4.83E-05**
0.03 (0.03, 0.04)	**1.11E-51**	0.02 (0.02, 0.02)	**2.12E-19**
0.00 (-0.01, 0.00)	6.06E-01	-0.02 (-0.03, -0.02)	**3.68E-20**
0.00 (0.00, 0.00)	8.45E-01	-0.02 (-0.02, -0.01)	**1.27E-16**
0.00 (0.00, 0.01)	4.95E-01	-0.02 (-0.02, -0.01)	**9.03E-12**
-0.01 (-0.01, 0.00)	**1.27E-02**	-0.03 (-0.03, -0.02)	**9.53E-29**
-0.01 (-0.01, 0.00)	**1.19E-03**	-0.03 (-0.03, -0.02)	**3.00E-31**
0.00 (0.00, 0.01)	6.30E-01	-0.02 (-0.03, -0.02)	**1.66E-18**
0.02 (0.02, 0.03)	**2.35E-23**	0.02 (0.01, 0.02)	**7.06E-18**

-0.02 (-0.03, -0.02)	**5.72E-28**	-0.03 (-0.03, -0.02)	**6.93E-40**
-0.01 (-0.02, -0.01)	**1.58E-10**	-0.02 (-0.02, -0.02)	**9.15E-20**
-0.01 (-0.01, -0.01)	**7.98E-06**	-0.02 (-0.02, -0.01)	**7.83E-15**
-0.03 (-0.03, -0.02)	**1.88E-31**	-0.03 (-0.04, -0.03)	**4.39E-46**
-0.03 (-0.03, -0.03)	**7.70E-45**	-0.03 (-0.04, -0.03)	**2.48E-54**
-0.01 (-0.01, 0.00)	**2.26E-03**	-0.02 (-0.02, -0.01)	**4.06E-17**
0.01 (0.01, 0.02)	**1.27E-10**	0.02 (0.02, 0.02)	**1.73E-19**
0.01 (0.01, 0.02)	**4.57E-10**	0.02 (0.01, 0.02)	**4.79E-13**
-0.01 (-0.01, 0.00)	**3.03E-04**	-0.01 (-0.01, -0.01)	**1.82E-06**
-0.01 (-0.02, -0.01)	**1.40E-06**	-0.01 (-0.02, -0.01)	**3.43E-07**
0.00 (0.00, 0.00)	9.47E-01	-0.01 (-0.01, 0.00)	**5.92E-03**
0.00 (0.00, 0.01)	1.69E-01	0.00 (0.00, 0.01)	**3.40E-02**
-0.01 (-0.01, -0.01)	**6.17E-06**	-0.02 (-0.02, -0.01)	**1.27E-12**
-0.01 (-0.02, -0.01)	**5.43E-09**	-0.02 (-0.02, -0.01)	**1.84E-15**
0.00 (0.00, 0.01)	6.28E-02	0.00 (-0.01, 0.00)	1.02E-01
0.01 (0.00, 0.01)	**5.23E-03**	0.01 (0.01, 0.02)	**8.86E-08**
0.01 (0.01, 0.02)	**2.02E-08**	0.02 (0.02, 0.02)	**3.67E-18**
0.00 (0.00, 0.01)	3.43E-01	0.00 (-0.01, 0.00)	8.03E-02
0.00 (0.00, 0.01)	4.99E-01	-0.01 (-0.01, 0.00)	**3.94E-04**
0.00 (0.00, 0.01)	2.03E-01	0.01 (0.00, 0.01)	**2.94E-03**
-0.01 (-0.01, 0.00)	**8.60E-05**	-0.01 (-0.01, 0.00)	**3.94E-04**
-0.01 (-0.01, -0.01)	**1.41E-05**	-0.02 (-0.03, -0.02)	**2.64E-20**
-0.01 (-0.02, -0.01)	**9.89E-11**	-0.03 (-0.03, -0.02)	**5.54E-32**
-0.02 (-0.02, -0.01)	**6.36E-13**	-0.03 (-0.03, -0.02)	**3.93E-34**
-0.01 (-0.01, -0.01)	**2.91E-05**	-0.02 (-0.03, -0.02)	**4.82E-24**
0.01 (0.01, 0.02)	**9.84E-10**	-0.03 (-0.02, 0.03)	**3.33E-30**
-0.03 (-0.03, -0.02)	**2.06E-34**	-0.04 (-0.04, -0.03)	**3.40E-58**
-0.02 (-0.02, -0.02)	**2.72E-21**	-0.03 (-0.03, -0.02)	**1.62E-34**
-0.01 (-0.02, -0.01)	**3.74E-11**	-0.02 (-0.02, -0.01)	**7.97E-16**
-0.03 (-0.03, -0.02)	**1.07E-31**	-0.04 (-0.04, -0.03)	**5.42E-58**
0.02 (0.02, 0.03)	**8.34E-26**	0.03 (0.03, 0.04)	**1.60E-42**
0.02 (0.02, 0.03)	**1.79E-28**	0.04 (0.03, 0.04)	**3.42E-58**
-0.02 (-0.03, -0.02)	**4.62E-23**	-0.04 (-0.04, -0.03)	**8.96E-56**
-0.02 (-0.03, -0.02)	**1.34E-23**	-0.04 (-0.04, -0.03)	**8.69E-58**
-0.01 (-0.02, -0.01)	**1.78E-10**	-0.02 (-0.02, -0.02)	**8.86E-23**
0.02 (0.01, 0.02)	**4.63E-18**	0.03 (0.03, 0.04)	**2.84E-44**
0.03 (0.03, 0.03)	**8.79E-43**	0.03 (0.03, 0.04)	**3.11E-51**
-0.03 (-0.04, -0.03)	**4.38E-53**	-0.04 (-0.05, -0.04)	**2.16E-86**
-0.03 (-0.04, -0.03)	**8.78E-59**	-0.04 (-0.05, -0.05)	**6.69E-85**
-0.01 (-0.02, -0.01)	**2.96E-08**	-0.02 (-0.03, -0.02)	**1.09E-21**
0.03 (0.02, 0.03)	**1.50E-40**	0.04 (0.04, 0.05)	**1.63E-72**
-0.01 (-0.02, -0.01)	**2.79E-09**	-0.01 (-0.01, 0.00)	**1.86E-02**
-0.02 (-0.03, -0.02)	**2.09E-23**	-0.03 (-0.04, -0.03)	**5.68E-52**
-0.01 (-0.01, -0.01)	**2.77E-05**	-0.01 (-0.02, -0.01)	**5.23E-11**
-0.02 (-0.03, -0.02)	**1.16E-23**	-0.03 (-0.04, -0.03)	**5.68E-52**
0.03 (0.02, 0.03)	**1.06E-38**	0.04 (0.03, 0.04)	**3.42E-63**
-0.02 (-0.02, -0.01)	**6.56E-18**	-0.01 (-0.01, -0.01)	**2.05E-06**
-0.02 (-0.02, -0.01)	**2.42E-18**	-0.03 (-0.03, -0.02)	**8.49E-38**

0.00 (0.00, 0.01)	**3.54E-02**	0.00 (0.00, 0.01)	6.02E-02
-0.02 (-0.03, -0.02)	**6.62E-23**	-0.03 (-0.04, -0.03)	**1.98E-42**
0.03 (0.03, 0.03)	**4.20E-44**	0.04 (0.03, 0.04)	**8.52E-61**
0.01 (0.00, 0.01)	**4.32E-03**	0.00 (0.00, 0.01)	1.82E-01
-0.03 (-0.03, -0.02)	**8.56E-41**	-0.03 (-0.04, -0.03)	**9.31E-49**
-0.01 (-0.01, 0.00)	**1.94E-02**	0.00 (-0.01, 0.00)	1.66E-01
-0.02 (-0.03, -0.02)	**1.28E-27**	-0.03 (-0.03, -0.03)	**4.25E-41**
0.03 (0.02, 0.03)	**9.00E-33**	0.03 (0.03, 0.04)	**5.44E-52**
0.03 (0.02, 0.03)	**5.27E-35**	0.01 (0.01, 0.02)	**3.31E-09**
-0.04 (-0.04, -0.04)	**5.05E-74**	-0.03 (-0.03, -0.02)	**6.52E-37**
-0.04 (-0.04, -0.03)	**3.71E-60**	-0.04 (-0.04, -0.03)	**8.70E-61**
-0.02 (-0.02, -0.02)	**1.54E-20**	0.00 (-0.01, 0.00)	2.52E-01
0.00 (0.00, 0.01)	5.25E-02	0.01 (0.01, 0.02)	**2.93E-10**
0.00 (0.00, 0.01)	2.28E-01	0.01 (0.01, 0.02)	**1.21E-09**
-0.01 (-0.01, 0.00)	**2.13E-04**	-0.02 (-0.03, -0.02)	**2.05E-20**
-0.01 (-0.02, -0.01)	**4.69E-09**	-0.02 (-0.03, -0.02)	**2.03E-26**
0.01 (0.01, 0.02)	**2.88E-10**	0.00 (0.00, 0.00)	9.86E-01
0.01 (0.01, 0.02)	**3.41E-07**	0.02 (0.02, 0.03)	**2.58E-22**
0.02 (0.01, 0.02)	**1.04E-15**	0.03 (0.03, 0.04)	**2.49E-48**
-0.02 (-0.03, -0.02)	**2.73E-23**	-0.03 (-0.04, -0.03)	**9.08E-51**
-0.03 (-0.03, -0.02)	**8.14E-34**	-0.03 (-0.04, -0.03)	**7.65E-50**
0.01 (0.00, 0.01)	**8.29E-03**	-0.02 (-0.02, -0.02)	**2.74E-16**
0.02 (0.02, 0.03)	**7.29E-26**	0.03 (0.03, 0.04)	**8.60E-45**
0.02 (0.02, 0.03)	**2.03E-30**	0.02 (0.01, 0.02)	**1.48E-12**
-0.03 (-0.04, -0.03)	**7.75E-52**	-0.03 (-0.04, -0.03)	**1.81E-50**
-0.04 (-0.04, -0.03)	**5.68E-69**	-0.03 (-0.04, -0.03)	**3.15E-52**
0.02 (0.01, 0.02)	**5.01E-17**	0.00 (0.00, 0.01)	3.00E-01
0.02 (0.02, 0.03)	**6.74E-27**	0.03 (0.03, 0.04)	**1.48E-52**
0.00 (0.00, 0.01)	6.59E-01	0.01 (0.01, 0.02)	**2.52E-08**
-0.01 (-0.01, 0.00)	**7.63E-03**	-0.01 (-0.02, -0.01)	**7.58E-08**
0.02 (0.01, 0.02)	**2.95E-18**	0.00 (-0.01, 0.00)	9.03E-01
0.00 (-0.01, 0.00)	**3.26E-02**	-0.02 (-0.03, -0.02)	**1.08E-20**
0.01 (0.00, 0.01)	**4.21E-04**	0.00 (0.00, 0.01)	5.45E-02
-0.01 (-0.02, -0.01)	**8.28E-07**	-0.02 (-0.02, -0.02)	**6.43E-19**
0.00 (0.00, 0.01)	4.50E-01	-0.01 (-0.01, 0.00)	**2.25E-04**
-0.01 (-0.01, 0.00)	**3.78E-03**	-0.02 (-0.03, -0.02)	**4.86E-25**
0.00 (0.00, 0.00)	8.19E-01	-0.01 (-0.02, -0.01)	**2.66E-11**
0.01 (0.01, 0.02)	**2.16E-09**	0.02 (0.01, 0.02)	**5.56E-16**
0.01 (0.01, 0.01)	**1.66E-06**	0.02 (0.01, 0.02)	**3.27E-14**
0.02 (0.01, 0.02)	**2.64E-15**	0.02 (0.01, 0.02)	**4.67E-14**
0.02 (0.02, 0.03)	**1.62E-28**	0.02 (0.02, 0.03)	**7.59E-22**
0.01 (0.00, 0.01)	**3.77E-04**	-0.01 (-0.01, -0.01)	**1.85E-05**
0.01 (0.01, 0.02)	**3.56E-08**	0.03 (0.02, 0.03)	**3.03E-30**
0.01 (0.00, 0.01)	**3.77E-04**	-0.01 (-0.02, -0.01)	**3.75E-07**
0.00 (0.00, 0.00)	9.78E-01	-0.02 (-0.02, -0.02)	**8.48E-18**
0.00 (0.00, 0.01)	**3.34E-02**	-0.01 (-0.02, -0.01)	**1.09E-09**

0.00 (0.00, 0.00)	9.44E-01	-0.02 (-0.03, -0.02)	**3.02E-20**
0.00 (-0.01, 0.00)	5.78E-01	-0.01 (-0.01, 0.00)	**2.10E-04**
0.00 (-0.01, 0.00)	2.47E-01	0.00 (0.00, 0.01)	1.67E-01
0.01 (0.01, 0.02)	**2.56E-07**	0.01 (0.00, 0.01)	**2.25E-02**
0.01 (0.01, 0.01)	**2.20E-06**	0.00 (-0.01, 0.00)	7.29E-02
0.03 (0.03, 0.04)	**2.03E-54**	0.01 (0.01, 0.02)	**1.74E-09**
0.00 (-0.01, 0.00)	4.50E-01	-0.01 (-0.01, 0.00)	**6.70E-05**
0.04 (0.03, 0.04)	**1.10E-66**	0.02 (0.02, 0.02)	**1.11E-19**
0.00 (0.00, 0.00)	8.34E-01	-0.01 (-0.01, -0.01)	**1.24E-05**
0.01 (0.01, 0.01)	**8.70E-06**	0.00 (-0.01, 0.00)	2.10E-01
0.01 (0.01, 0.01)	**2.04E-06**	0.01 (0.01, 0.02)	**1.57E-07**
0.01 (0.01, 0.02)	**3.10E-07**	0.01 (0.00, 0.01)	**1.91E-02**
0.03 (0.02, 0.03)	**1.05E-37**	0.01 (0.01, 0.02)	**1.45E-07**
-0.02 (-0.03, -0.02)	**4.99E-28**	-0.03 (-0.03, -0.02)	**5.35E-28**
0.04 (0.03, 0.04)	**1.14E-69**	0.02 (0.02, 0.03)	**4.06E-22**
-0.03 (-0.03, -0.02)	**6.91E-33**	-0.03 (-0.03, -0.02)	**1.83E-30**
-0.01 (-0.01, -0.01)	**2.89E-05**	-0.02 (-0.02, -0.01)	**1.51E-12**
0.01 (0.00, 0.01)	**1.44E-04**	0.02 (0.02, 0.03)	**1.45E-21**
0.01 (0.00, 0.01)	**4.08E-03**	0.00 (0.00, 0.01)	4.88E-01
0.02 (0.01, 0.02)	**4.47E-13**	0.02 (0.01, 0.02)	**8.93E-14**
0.00 (-0.01, 0.00)	4.25E-01	0.00 (0.00, 0.01)	1.89E-01
0.01 (0.01, 0.02)	**1.55E-07**	0.00 (-0.01, 0.00)	3.88E-01
0.01 (0.01, 0.01)	**2.93E-06**	0.01 (0.01, 0.01)	**1.61E-06**
-0.02 (-0.02, -0.02)	**4.25E-19**	-0.03 (-0.03, -0.02)	**1.22E-31**
0.00 (-0.01, 0.00)	6.96E-02	0.00 (0.00, 0.00)	7.74E-01
0.00 (0.00, 0.00)	7.69E-01	0.01 (0.00, 0.01)	**6.15E-03**
0.00 (-0.01, 0.00)	1.27E-01	0.00 (0.00, 0.01)	5.24E-02
0.01 (0.01, 0.01)	**1.82E-06**	0.02 (0.01, 0.02)	**2.12E-12**
0.00 (0.00, 0.01)	**3.76E-02**	0.01 (0.00, 0.01)	**2.60E-02**
0.01 (0.00, 0.01)	**1.30E-03**	0.00 (0.00, 0.00)	7.58E-01
-0.01 (-0.01, 0.00)	**3.95E-04**	-0.01 (-0.01, 0.00)	**3.15E-03**
0.01 (0.00, 0.01)	**1.77E-03**	0.00 (0.00, 0.01)	1.16E-01
0.01 (0.00, 0.01)	**8.22E-05**	0.01 (0.00, 0.01)	**1.85E-02**
0.02 (0.01, 0.02)	**2.20E-15**	0.03 (0.03, 0.03)	**4.35E-34**
0.00 (-0.01, 0.00)	7.81E-02	-0.01 (-0.01, 0.00)	**1.83E-02**
0.01 (0.01, 0.02)	**1.97E-09**	0.02 (0.01, 0.02)	**2.12E-16**
-0.01 (-0.01, 0.00)	**1.75E-03**	-0.02 (-0.02, -0.01)	**2.26E-11**
0.00 (0.00, 0.01)	5.85E-02	0.01 (0.01, 0.01)	**2.36E-06**
0.00 (-0.01, 0.00)	**3.00E-02**	-0.01 (-0.02, -0.01)	**8.36E-11**
-0.01 (-0.02, -0.01)	**1.77E-08**	-0.02 (-0.03, -0.02)	**1.21E-22**
-0.01 (-0.01, -0.01)	**2.31E-05**	-0.02 (-0.02, -0.01)	**3.83E-15**
0.00 (-0.01, 0.00)	1.14E-01	-0.03 (-0.03, -0.02)	**1.18E-29**
-0.01 (-0.01, -0.01)	**8.96E-06**	-0.02 (-0.02, -0.02)	**1.12E-17**
-0.04 (-0.05, -0.04)	**3.80E-87**	-0.03 (-0.03, -0.02)	**9.21E-33**
0.01 (0.00, 0.01)	**5.69E-05**	0.01 (0.00, 0.01)	**3.09E-04**
-0.01 (-0.02, -0.01)	**4.17E-11**	-0.02 (-0.03, -0.02)	**7.76E-30**
0.01 (0.00, 0.01)	**2.99E-03**	-0.02 (-0.02, -0.01)	**5.76E-13**
0.00 (0.00, 0.01)	**2.62E-02**	0.00 (0.00, 0.01)	**4.82E-02**

0.00 (0.00, 0.00)	8.92E-01	0.00 (-0.01, 0.00)	5.06E-02
-0.01 (-0.01, 0.00)	**4.17E-05**	-0.03 (-0.03, -0.02)	**5.93E-36**
0.01 (0.01, 0.02)	**9.77E-09**	0.01 (0.01, 0.01)	**1.32E-05**
0.00 (-0.01, 0.00)	2.25E-01	-0.01 (-0.01, 0.00)	**5.52E-05**
-0.01 (-0.01, 0.00)	**2.97E-05**	-0.03 (-0.03, -0.02)	**1.77E-35**
0.00 (0.00, 0.01)	**3.11E-02**	-0.01 (-0.02, -0.01)	**9.19E-09**
0.01 (0.01, 0.01)	**2.39E-06**	0.01 (0.01, 0.02)	**3.90E-07**
-0.01 (-0.02, -0.01)	**1.92E-07**	-0.02 (-0.02, -0.01)	**7.19E-18**
0.01 (0.00, 0.01)	**1.18E-02**	-0.02 (-0.02, -0.01)	**2.18E-11**
0.00 (0.00, 0.01)	1.37E-01	-0.01 (-0.01, 0.00)	**8.30E-05**
0.01 (0.01, 0.01)	**2.57E-06**	0.01 (0.01, 0.02)	**4.61E-09**
-0.01 (-0.01, -0.01)	**1.92E-05**	-0.02 (-0.02, -0.01)	**1.34E-15**
0.00 (0.00, 0.01)	7.98E-02	-0.02 (-0.02, -0.01)	**3.83E-15**
0.01 (0.00, 0.01)	**2.30E-03**	0.00 (0.00, 0.01)	5.22E-01
0.00 (-0.01, 0.00)	5.78E-01	-0.03 (-0.03, -0.02)	**9.84E-26**
0.01 (0.01, 0.02)	**3.94E-07**	0.01 (0.01, 0.01)	**1.82E-06**

e discovery rate (FDR) adjustment.

Supplement

Metabolites	Group
Concentration of chylomicrons and extremely large VLDL particles	Lipoprotein subclasses
Total lipids in chylomicrons and extremely large VLDL	Lipoprotein subclasses
Phospholipids in chylomicrons and extremely large VLDL	Lipoprotein subclasses
Cholesterol in chylomicrons and extremely large VLDL	Lipoprotein subclasses
Cholesteryl esters in chylomicrons and extremely large VLDL	Lipoprotein subclasses
Free cholesterol in chylomicrons and extremely large VLDL	Lipoprotein subclasses
Triglycerides in chylomicrons and extremely large VLDL	Lipoprotein subclasses
Concentration of very large VLDL particles	Lipoprotein subclasses
Total lipids in very large VLDL	Lipoprotein subclasses
Phospholipids in very large VLDL	Lipoprotein subclasses
Cholesterol in very large VLDL	Lipoprotein subclasses
Cholesteryl esters in very large VLDL	Lipoprotein subclasses
Free cholesterol in very large VLDL	Lipoprotein subclasses
Triglycerides in very large VLDL	Lipoprotein subclasses
Concentration of large VLDL particles	Lipoprotein subclasses
Total lipids in large VLDL	Lipoprotein subclasses
Phospholipids in large VLDL	Lipoprotein subclasses
Cholesterol in large VLDL	Lipoprotein subclasses
Cholesteryl esters in large VLDL	Lipoprotein subclasses
Free cholesterol in large VLDL	Lipoprotein subclasses
Triglycerides in large VLDL	Lipoprotein subclasses
Concentration of medium VLDL particles	Lipoprotein subclasses
Total lipids in medium VLDL	Lipoprotein subclasses
Phospholipids in medium VLDL	Lipoprotein subclasses
Cholesterol in medium VLDL	Lipoprotein subclasses
Cholesteryl esters in medium VLDL	Lipoprotein subclasses
Free cholesterol in medium VLDL	Lipoprotein subclasses
Triglycerides in medium VLDL	Lipoprotein subclasses
Concentration of small VLDL particles	Lipoprotein subclasses
Total lipids in small VLDL	Lipoprotein subclasses
Phospholipids in small VLDL	Lipoprotein subclasses
Cholesterol in small VLDL	Lipoprotein subclasses
Cholesteryl esters in small VLDL	Lipoprotein subclasses
Free cholesterol in small VLDL	Lipoprotein subclasses
Triglycerides in small VLDL	Lipoprotein subclasses
Concentration of very small VLDL particles	Lipoprotein subclasses
Total lipids in very small VLDL	Lipoprotein subclasses
Phospholipids in very small VLDL	Lipoprotein subclasses
Cholesterol in very small VLDL	Lipoprotein subclasses
Cholesteryl esters in very small VLDL	Lipoprotein subclasses
Free cholesterol in very small VLDL	Lipoprotein subclasses
Triglycerides in very small VLDL	Lipoprotein subclasses
Concentration of IDL particles	Lipoprotein subclasses

Total lipids in IDL	Lipoprotein subclasses
Phospholipids in IDL	Lipoprotein subclasses
Cholesterol in IDL	Lipoprotein subclasses
Cholesteryl esters in IDL	Lipoprotein subclasses
Free cholesterol in IDL	Lipoprotein subclasses
Triglycerides in IDL	Lipoprotein subclasses
Concentration of large LDL particles	Lipoprotein subclasses
Total lipids in large LDL	Lipoprotein subclasses
Phospholipids in large LDL	Lipoprotein subclasses
Cholesterol in large LDL	Lipoprotein subclasses
Cholesteryl esters in large LDL	Lipoprotein subclasses
Free cholesterol in large LDL	Lipoprotein subclasses
Triglycerides in large LDL	Lipoprotein subclasses
Concentration of medium LDL particles	Lipoprotein subclasses
Total lipids in medium LDL	Lipoprotein subclasses
Phospholipids in medium LDL	Lipoprotein subclasses
Cholesterol in medium LDL	Lipoprotein subclasses
Cholesteryl esters in medium LDL	Lipoprotein subclasses
Free cholesterol in medium LDL	Lipoprotein subclasses
Triglycerides in medium LDL	Lipoprotein subclasses
Concentration of small LDL particles	Lipoprotein subclasses
Total lipids in small LDL	Lipoprotein subclasses
Phospholipids in small LDL	Lipoprotein subclasses
Cholesterol in small LDL	Lipoprotein subclasses
Cholesteryl esters in small LDL	Lipoprotein subclasses
Free cholesterol in small LDL	Lipoprotein subclasses
Triglycerides in small LDL	Lipoprotein subclasses
Concentration of very large HDL particles	Lipoprotein subclasses
Total lipids in very large HDL	Lipoprotein subclasses
Phospholipids in very large HDL	Lipoprotein subclasses
Cholesterol in very large HDL	Lipoprotein subclasses
Cholesteryl esters in very large HDL	Lipoprotein subclasses
Free cholesterol in very large HDL	Lipoprotein subclasses
Triglycerides in very large HDL	Lipoprotein subclasses
Concentration of large HDL particles	Lipoprotein subclasses
Total lipids in large HDL	Lipoprotein subclasses
Phospholipids in large HDL	Lipoprotein subclasses
Cholesterol in large HDL	Lipoprotein subclasses
Cholesteryl esters in large HDL	Lipoprotein subclasses
Free cholesterol in large HDL	Lipoprotein subclasses
Triglycerides in large HDL	Lipoprotein subclasses
**Concentration of medium HDL particles**	Lipoprotein subclasses
**Total lipids in medium HDL**	Lipoprotein subclasses
**Phospholipids in medium HDL**	Lipoprotein subclasses
**Cholesterol in medium HDL**	Lipoprotein subclasses
**Cholesteryl esters in medium HDL**	Lipoprotein subclasses
**Free cholesterol in medium HDL**	Lipoprotein subclasses
Triglycerides in medium HDL	Lipoprotein subclasses

Concentration of small HDL particles	Lipoprotein subclasses
Total lipids in small HDL	Lipoprotein subclasses
Phospholipids in small HDL	Lipoprotein subclasses
Cholesterol in small HDL	Lipoprotein subclasses
Cholesteryl esters in small HDL	Lipoprotein subclasses
**Free cholesterol in small HDL**	Lipoprotein subclasses
Triglycerides in small HDL	Lipoprotein subclasses
Phospholipids to total lipids ratio in chylomicrons and extremely large VI	Relative lipoprotein lipid cc
Cholesterol to total lipids ratio in chylomicrons and extremely large VLD	Relative lipoprotein lipid cc
Cholesteryl esters to total lipids ratio in chylomicrons and extremely larg	Relative lipoprotein lipid cc
Free cholesterol to total lipids ratio in chylomicrons and extremely large	Relative lipoprotein lipid cc
Triglycerides to total lipids ratio in chylomicrons and extremely large VL	Relative lipoprotein lipid cc
Phospholipids to total lipids ratio in very large VLDL /(lipds in ckb)	Relative lipoprotein lipid cc
**Cholesterol to total lipids ratio in very large VLDL**	Relative lipoprotein lipid cc
Cholesteryl esters to total lipids ratio in very large VLDL	Relative lipoprotein lipid cc
**Free cholesterol to total lipids ratio in very large VLDL**	Relative lipoprotein lipid cc
**Triglycerides to total lipids ratio in very large VLDL**	Relative lipoprotein lipid cc
Phospholipids to total lipids ratio in large VLDL	Relative lipoprotein lipid cc
Cholesterol to total lipids ratio in large VLDL	Relative lipoprotein lipid cc
Cholesteryl esters to total lipids ratio in large VLDL	Relative lipoprotein lipid cc
Free cholesterol to total lipids ratio in large VLDL	Relative lipoprotein lipid cc
Triglycerides to total lipids ratio in large VLDL	Relative lipoprotein lipid cc
Phospholipids to total lipids ratio in medium VLDL	Relative lipoprotein lipid cc
**Cholesterol to total lipids ratio in medium VLDL**	Relative lipoprotein lipid cc
**Cholesteryl esters to total lipids ratio in medium VLDL**	Relative lipoprotein lipid cc
Free cholesterol to total lipids ratio in medium VLDL	Relative lipoprotein lipid cc
Triglycerides to total lipids ratio in medium VLDL	Relative lipoprotein lipid cc
**Phospholipids to total lipids ratio in small VLDL**	Relative lipoprotein lipid cc
Cholesterol to total lipids ratio in small VLDL	Relative lipoprotein lipid cc
Cholesteryl esters to total lipids ratio in small VLDL	Relative lipoprotein lipid cc
**Free cholesterol to total lipids ratio in small VLDL**	Relative lipoprotein lipid cc
Triglycerides to total lipids ratio in small VLDL	Relative lipoprotein lipid cc
Phospholipids to total lipids ratio in very small VLDL	Relative lipoprotein lipid cc
Cholesterol to total lipids ratio in very small VLDL	Relative lipoprotein lipid cc
Cholesteryl esters to total lipids ratio in very small VLDL	Relative lipoprotein lipid cc
Free cholesterol to total lipids ratio in very small VLDL	Relative lipoprotein lipid cc
Triglycerides to total lipids ratio in very small VLDL	Relative lipoprotein lipid cc
Phospholipids to total lipids ratio in IDL	Relative lipoprotein lipid cc
Cholesterol to total lipids ratio in IDL	Relative lipoprotein lipid cc
Cholesteryl esters to total lipids ratio in IDL	Relative lipoprotein lipid cc
Free cholesterol to total lipids ratio in IDL	Relative lipoprotein lipid cc
Triglycerides to total lipids ratio in IDL	Relative lipoprotein lipid cc
Phospholipids to total lipids ratio in large LDL	Relative lipoprotein lipid cc
Cholesterol to total lipids ratio in large LDL	Relative lipoprotein lipid cc
Cholesteryl esters to total lipids ratio in large LDL	Relative lipoprotein lipid cc
Free cholesterol to total lipids ratio in large LDL	Relative lipoprotein lipid cc
Triglycerides to total lipids ratio in large LDL	Relative lipoprotein lipid cc
Phospholipids to total lipids ratio in medium LDL	Relative lipoprotein lipid cc

Cholesterol to total lipids ratio in medium LDL	Relative lipoprotein lipid co
Cholesteryl esters to total lipids ratio in medium LDL	Relative lipoprotein lipid co
Free cholesterol to total lipids ratio in medium LDL	Relative lipoprotein lipid co
Triglycerides to total lipids ratio in medium LDL	Relative lipoprotein lipid co
Phospholipids to total lipids ratio in small LDL	Relative lipoprotein lipid co
Cholesterol to total lipids ratio in small LDL	Relative lipoprotein lipid co
Cholesteryl esters to total lipids ratio in small LDL	Relative lipoprotein lipid co
Free cholesterol to total lipids ratio in small LDL	Relative lipoprotein lipid co
Triglycerides to total lipids ratio in small LDL	Relative lipoprotein lipid co
Phospholipids to total lipids ratio in very large HDL	Relative lipoprotein lipid co
Cholesterol to total lipids ratio in very large HDL	Relative lipoprotein lipid co
Cholesteryl esters to total lipids ratio in very large HDL	Relative lipoprotein lipid co
Free cholesterol to total lipids ratio in very large HDL	Relative lipoprotein lipid co
Triglycerides to total lipids ratio in very large HDL	Relative lipoprotein lipid co
Phospholipids to total lipids ratio in large HDL	Relative lipoprotein lipid co
Cholesterol to total lipids ratio in large HDL	Relative lipoprotein lipid co
Cholesteryl esters to total lipids ratio in large HDL	Relative lipoprotein lipid co
Free cholesterol to total lipids ratio in large HDL	Relative lipoprotein lipid co
Triglycerides to total lipids ratio in large HDL	Relative lipoprotein lipid co
Phospholipids to total lipids ratio in medium HDL	Relative lipoprotein lipid co
Cholesterol to total lipids ratio in medium HDL	Relative lipoprotein lipid co
Cholesteryl esters to total lipids ratio in medium HDL	Relative lipoprotein lipid co
Free cholesterol to total lipids ratio in medium HDL	Relative lipoprotein lipid co
Triglycerides to total lipids ratio in medium HDL	Relative lipoprotein lipid co
Phospholipids to total lipids ratio in small HDL	Relative lipoprotein lipid co
Cholesterol to total lipids ratio in small HDL	Relative lipoprotein lipid co
Cholesteryl esters to total lipids ratio in small HDL	Relative lipoprotein lipid co
Free cholesterol to total lipids ratio in small HDL	Relative lipoprotein lipid co
Triglycerides to total lipids ratio in small HDL	Relative lipoprotein lipid co
Average diameter for VLDL particles	Lipoprotein particle sizes
Average diameter for LDL particles	Lipoprotein particle sizes
Average diameter for HDL particles	Lipoprotein particle sizes
Total cholesterol	Cholesterol
VLDL cholesterol	Cholesterol
Remnant cholesterol (non-HDL, non-LDL -cholesterol)	Cholesterol
LDL cholesterol	Cholesterol
**HDL cholesterol**	Cholesterol
**HDL2 cholesterol**	Cholesterol
HDL3 cholesterol	Cholesterol
Total esterified cholesterol	Cholesteryl esters
Total free cholesterol	Free cholesterol
Total triglycerides	Triglycerides
Triglycerides in VLDL	Triglycerides
Triglycerides in LDL	Triglycerides
Triglycerides in HDL	Triglycerides
Phosphoglycerides	Other lipids
Ratio of triglycerides to phosphoglycerides	Other lipids
Phosphatidylcholines	Other lipids

Sphingomyelins	Other lipids
Total cholines	Other lipids
Apolipoprotein A1	Apolipoproteins
Apolipoprotein B	Apolipoproteins
Ratio of apolipoprotein B to apolipoprotein A1	Apolipoproteins
Total fatty acids	Fatty acids
Degree of unsaturation	Fatty acids
Docosahexaenoic acid	Fatty acids
Linoleic acid	Fatty acids
Omega-3 fatty acids	Fatty acids
Omega-6 fatty acids	Fatty acids
Polyunsaturated fatty acids	Fatty acids
Monounsaturated fatty acids	Fatty acids
Saturated fatty acids	Fatty acids
Ratio of docosahexaenoic acid to total fatty acids	Fatty acids
Ratio of linoleic acid to total fatty acids	Fatty acids
Ratio of omega-3 fatty acids to total fatty acids	Fatty acids
Ratio of omega-6 fatty acids to total fatty acids	Fatty acids
Ratio of polyunsaturated fatty acids to total fatty acids	Fatty acids
Ratio of monounsaturated fatty acids to total fatty acids	Fatty acids
Ratio of saturated fatty acids to total fatty acids	Fatty acids
Glucose	Glycolysis related metabolit
Lactate	Glycolysis related metabolit
Citrate	Glycolysis related metabolit
Alanine	Amino acids
Glutamine	Amino acids
Histidine	Amino acids
Isoleucine	Amino acids
Leucine	Amino acids
Valine	Amino acids
Phenylalanine	Amino acids
Tyrosine	Amino acids
Acetate	Ketone bodies
Acetoacetate	Ketone bodies
**3-Hydroxybutyrate**	Ketone bodies
Creatinine	Fluid balance
Albumin	Fluid balance
Glycoprotein acetyls	Inflammation
Acetone	Ketone bodies
Cholesteryl esters in HDL	Cholesteryl esters
Cholesteryl esters in LDL	Cholesteryl esters
Cholesteryl esters in VLDL/ (Cholesterol in ckb)	Cholesteryl esters
Clinical LDL cholesterol	Cholesterol
Concentration of HDL particles	Lipoprotein particle concent
Concentration of LDL particles	Lipoprotein particle concent
Concentration of VLDL particles	Lipoprotein particle concent
Free cholesterol in HDL	Free cholesterol
Free cholesterol in LDL	Free cholesterol

Free cholesterol in VLDL	Free cholesterol
Glycine	Amino acids
Phospholipids in HDL	Phospholipids
Phospholipids in LDL	Phospholipids
Phospholipids in VLDL	Phospholipids
Pyruvate	Glycolysis related metabolit
Ratio of omega-6 fatty acids to omega-3 fatty acids	Fatty acids
Ratio of polyunsaturated fatty acids to monounsaturated fatty acids	Fatty acids
Total cholesterol minus HDL-C	Cholesterol
Total concentration of branched-chain amino acids (leucine + isoleucine	Amino acids
Total concentration of lipoprotein particles	Lipoprotein particle concent
Total lipids in HDL	Total lipids
Total lipids in LDL	Total lipids
Total lipids in lipoprotein particles	Total lipids
Total lipids in VLDL	Total lipids
Total phospholipids in lipoprotein particles	Phospholipids

Models were adjusted for age, sex, regions (CKB only), ethnicity (UKB only), levels of education, smoking stat

HR, hazard ratio; CI, confidence interval; LDL, Low Density Lipoprotein; HDL, High Density Lipoprotein; VI

ary Table 4. Association of all metabolites with all-cause mortality in the CKB and UK

CKB			
**Unadjusted HR (95% CI)**	**P value**	**Adjusted HR (95% CI)**	**P value**
0.98 (0.94, 1.02)	4.49E-01	0.94 (0.90, 0.99)	**2.91E-02**
0.98 (0.94, 1.03)	5.20E-01	0.94 (0.90, 0.99)	**3.77E-02**
0.99 (0.94, 1.03)	7.10E-01	0.95 (0.91, 1.00)	8.35E-02
1.02 (0.98, 1.07)	3.72E-01	0.98 (0.94, 1.02)	4.55E-01
1.04 (1.00, 1.09)	9.63E-02	0.99 (0.95, 1.04)	7.80E-01
1.00 (0.96, 1.05)	8.89E-01	0.96 (0.92, 1.01)	1.92E-01
0.97 (0.93, 1.01)	2.76E-01	0.94 (0.89, 0.98)	**1.26E-02**
1.00 (0.96, 1.04)	9.68E-01	0.97 (0.92, 1.01)	2.09E-01
1.00 (0.96, 1.05)	9.80E-01	0.97 (0.92, 1.01)	2.28E-01
1.01 (0.96, 1.05)	8.40E-01	0.97 (0.93, 1.02)	2.81E-01
1.02 (0.98, 1.06)	5.13E-01	0.98 (0.94, 1.03)	5.25E-01
1.02 (0.98, 1.07)	4.55E-01	0.98 (0.94, 1.03)	5.83E-01
1.02 (0.97, 1.06)	5.82E-01	0.98 (0.94, 1.02)	4.44E-01
0.99 (0.95, 1.04)	8.39E-01	0.96 (0.92, 1.01)	1.68E-01
1.00 (0.95, 1.04)	8.68E-01	0.96 (0.92, 1.01)	1.81E-01
1.00 (0.95, 1.04)	9.03E-01	0.96 (0.92, 1.01)	1.84E-01
1.00 (0.96, 1.05)	9.61E-01	0.96 (0.92, 1.01)	1.87E-01
1.01 (0.96, 1.05)	8.40E-01	0.96 (0.92, 1.01)	1.90E-01
1.01 (0.96, 1.05)	8.39E-01	0.96 (0.92, 1.00)	1.48E-01
1.01 (0.96, 1.05)	8.39E-01	0.97 (0.93, 1.01)	2.48E-01
0.99 (0.95, 1.04)	7.89E-01	0.96 (0.92, 1.01)	1.80E-01
0.99 (0.95, 1.04)	8.27E-01	0.97 (0.92, 1.01)	1.97E-01
1.00 (0.95, 1.04)	8.79E-01	0.97 (0.92, 1.01)	2.09E-01
1.00 (0.96, 1.04)	9.46E-01	0.96 (0.92, 1.01)	1.81E-01
1.02 (0.98, 1.07)	4.59E-01	0.97 (0.93, 1.02)	3.38E-01
1.03 (0.99, 1.07)	2.87E-01	0.98 (0.93, 1.02)	3.44E-01
1.01 (0.97, 1.06)	7.29E-01	0.98 (0.93, 1.02)	3.44E-01
0.98 (0.94, 1.03)	5.76E-01	0.96 (0.92, 1.01)	1.84E-01
1.03 (0.98, 1.07)	3.02E-01	0.98 (0.94, 1.02)	4.55E-01
1.03 (0.99, 1.08)	2.05E-01	0.99 (0.94, 1.03)	6.23E-01
1.03 (0.99, 1.07)	2.87E-01	0.97 (0.93, 1.01)	2.75E-01
1.07 (1.03, 1.12)	**3.53E-03**	1.04 (1.00, 1.09)	9.84E-02
1.07 (1.03, 1.12)	**3.30E-03**	1.06 (1.02, 1.11)	**1.71E-02**
1.06 (1.02, 1.11)	**1.59E-02**	1.01 (0.97, 1.05)	7.23E-01
1.01 (0.97, 1.05)	8.03E-01	0.96 (0.92, 1.00)	1.23E-01
1.13 (1.08, 1.18)	**2.70E-08**	1.05 (1.00, 1.09)	8.29E-02
1.13 (1.08, 1.18)	**2.70E-08**	1.05 (1.01, 1.10)	**3.44E-02**
1.10 (1.05, 1.15)	**3.93E-05**	1.02 (0.97, 1.06)	5.72E-01
1.13 (1.09, 1.18)	**3.02E-08**	1.09 (1.05, 1.14)	**6.59E-05**
1.13 (1.09, 1.18)	**3.02E-08**	1.10 (1.06, 1.15)	**2.81E-05**
1.12 (1.08, 1.17)	**2.30E-07**	1.08 (1.04, 1.13)	**1.01E-03**
1.09 (1.05, 1.14)	**5.95E-05**	0.99 (0.95, 1.04)	7.34E-01
1.10 (1.05, 1.14)	**4.26E-05**	1.01 (0.97, 1.05)	7.33E-01

1.09 (1.05, 1.14)	**1.54E-04**	1.01 (0.97, 1.06)	7.13E-01
1.07 (1.03, 1.12)	**4.05E-03**	1.00 (0.95, 1.04)	8.64E-01
1.07 (1.02, 1.12)	**4.91E-03**	1.01 (0.97, 1.05)	7.33E-01
1.06 (1.02, 1.11)	**1.17E-02**	1.00 (0.96, 1.05)	9.84E-01
1.08 (1.04, 1.13)	**8.47E-04**	1.03 (0.99, 1.07)	2.80E-01
1.19 (1.14, 1.23)	**2.50E-18**	1.03 (0.99, 1.08)	2.10E-01
1.07 (1.03, 1.12)	**3.53E-03**	0.99 (0.95, 1.04)	7.33E-01
1.07 (1.02, 1.11)	**7.72E-03**	0.99 (0.95, 1.03)	7.16E-01
1.05 (1.00, 1.09)	7.49E-02	0.97 (0.93, 1.01)	2.75E-01
1.05 (1.00, 1.10)	5.24E-02	0.99 (0.95, 1.03)	6.70E-01
1.04 (1.00, 1.09)	8.39E-02	0.98 (0.94, 1.03)	5.36E-01
1.06 (1.02, 1.11)	**1.08E-02**	1.00 (0.96, 1.05)	9.38E-01
1.21 (1.17, 1.26)	**2.13E-23**	1.06 (1.02, 1.11)	**1.94E-02**
1.04 (0.99, 1.08)	1.45E-01	0.97 (0.92, 1.01)	2.09E-01
1.03 (0.99, 1.08)	2.21E-01	0.97 (0.92, 1.01)	1.97E-01
1.01 (0.96, 1.05)	8.08E-01	0.92 (0.88, 0.96)	**1.07E-03**
1.02 (0.98, 1.07)	4.24E-01	0.97 (0.93, 1.02)	3.14E-01
1.02 (0.98, 1.07)	4.21E-01	0.98 (0.93, 1.02)	3.95E-01
1.02 (0.97, 1.06)	5.25E-01	0.95 (0.91, 1.00)	7.73E-02
1.20 (1.15, 1.24)	**1.31E-18**	1.04 (0.99, 1.09)	1.66E-01
1.03 (0.98, 1.07)	3.65E-01	0.96 (0.92, 1.01)	1.96E-01
1.02 (0.98, 1.07)	4.36E-01	0.96 (0.92, 1.01)	1.96E-01
0.99 (0.95, 1.04)	8.08E-01	0.92 (0.88, 0.96)	**5.29E-04**
1.02 (0.97, 1.06)	5.26E-01	0.98 (0.94, 1.02)	4.26E-01
1.02 (0.98, 1.06)	4.95E-01	0.98 (0.94, 1.03)	5.36E-01
1.01 (0.96, 1.05)	8.27E-01	0.95 (0.91, 1.00)	8.46E-02
1.12 (1.08, 1.17)	**2.62E-07**	1.01 (0.96, 1.05)	8.07E-01
1.20 (1.16, 1.25)	**1.61E-17**	1.18 (1.14, 1.23)	**1.21E-15**
1.20 (1.15, 1.25)	**5.76E-17**	1.18 (1.14, 1.23)	**1.56E-15**
1.20 (1.15, 1.25)	**6.01E-17**	1.17 (1.12, 1.22)	**1.99E-13**
1.18 (1.13, 1.23)	**6.10E-13**	1.18 (1.14, 1.23)	**1.92E-15**
1.17 (1.12, 1.22)	**3.26E-12**	1.19 (1.14, 1.24)	**9.03E-16**
1.18 (1.14, 1.23)	**2.62E-14**	1.17 (1.13, 1.22)	**9.95E-14**
1.19 (1.15, 1.24)	**1.26E-17**	1.14 (1.09, 1.18)	**5.72E-09**
1.14 (1.10, 1.19)	**3.49E-09**	1.13 (1.08, 1.18)	**5.18E-08**
1.14 (1.09, 1.19)	**5.91E-09**	1.13 (1.08, 1.18)	**4.81E-08**
1.12 (1.08, 1.17)	**3.06E-07**	1.12 (1.08, 1.17)	**4.53E-07**
1.14 (1.09, 1.19)	**9.23E-09**	1.13 (1.09, 1.18)	**2.34E-08**
1.14 (1.09, 1.19)	**9.23E-09**	1.13 (1.08, 1.18)	**5.18E-08**
1.14 (1.09, 1.19)	**1.21E-08**	1.14 (1.10, 1.19)	**1.70E-09**
1.23 (1.19, 1.28)	**5.39E-24**	1.12 (1.08, 1.17)	**6.07E-07**
0.90 (0.86, 0.94)	**2.42E-05**	0.90 (0.86, 0.94)	**1.81E-05**
0.90 (0.86, 0.94)	**1.50E-05**	0.90 (0.86, 0.94)	**3.30E-05**
0.95 (0.91, 0.99)	**3.29E-02**	0.93 (0.89, 0.97)	**2.93E-03**
0.86 (0.83, 0.90)	**1.03E-09**	0.90 (0.86, 0.94)	**2.05E-05**
0.85 (0.81, 0.89)	**5.45E-12**	0.89 (0.85, 0.93)	**3.19E-06**
0.93 (0.89, 0.97)	**2.03E-03**	0.94 (0.89, 0.98)	**8.93E-03**
0.99 (0.95, 1.04)	8.27E-01	0.89 (0.85, 0.93)	**1.59E-06**

0.86 (0.83, 0.90)	**1.41E-11**	0.83 (0.79, 0.86)	**1.68E-17**
0.86 (0.83, 0.90)	**4.60E-12**	0.83 (0.80, 0.86)	**3.68E-17**
0.87 (0.83, 0.91)	**2.50E-09**	0.86 (0.82, 0.89)	**6.00E-11**
0.91 (0.87, 0.95)	**4.56E-05**	0.90 (0.86, 0.94)	**4.67E-06**
0.93 (0.89, 0.97)	**2.66E-03**	0.92 (0.88, 0.96)	**5.57E-04**
0.88 (0.84, 0.92)	**7.35E-08**	0.88 (0.84, 0.92)	**4.81E-08**
1.03 (0.99, 1.08)	2.62E-01	0.91 (0.87, 0.95)	**2.00E-04**
0.97 (0.93, 1.01)	2.74E-01	0.95 (0.91, 1.00)	6.95E-02
1.19 (1.15, 1.23)	**8.26E-28**	1.16 (1.12, 1.20)	**1.56E-15**
1.19 (1.15, 1.23)	**2.59E-25**	1.15 (1.12, 1.19)	**1.99E-15**
1.10 (1.06, 1.15)	**2.51E-06**	1.07 (1.02, 1.12)	**7.79E-03**
0.84 (0.81, 0.87)	**8.24E-19**	0.88 (0.84, 0.91)	**3.33E-09**
0.99 (0.95, 1.04)	8.39E-01	0.96 (0.92, 1.00)	1.05E-01
1.14 (1.10, 1.18)	**6.46E-14**	1.10 (1.06, 1.13)	**1.18E-07**
1.13 (1.08, 1.17)	**1.67E-09**	1.11 (1.07, 1.14)	**7.46E-08**
1.08 (1.03, 1.13)	**1.82E-03**	1.03 (0.98, 1.08)	3.14E-01
0.88 (0.84, 0.93)	**7.34E-07**	0.93 (0.89, 0.97)	**5.16E-03**
1.08 (1.03, 1.13)	**2.35E-03**	0.96 (0.92, 1.00)	1.47E-01
1.06 (1.02, 1.11)	**1.79E-02**	0.98 (0.94, 1.03)	4.70E-01
1.06 (1.01, 1.10)	**3.20E-02**	1.01 (0.96, 1.06)	7.40E-01
1.02 (0.98, 1.07)	4.36E-01	0.97 (0.93, 1.01)	2.36E-01
0.93 (0.89, 0.98)	**7.03E-03**	1.03 (0.98, 1.08)	3.20E-01
1.06 (1.02, 1.11)	**9.06E-03**	0.95 (0.91, 0.99)	**4.90E-02**
1.14 (1.09, 1.19)	**2.67E-08**	1.05 (1.00, 1.10)	8.61E-02
1.10 (1.05, 1.15)	**5.43E-05**	1.04 (0.99, 1.08)	1.94E-01
1.11 (1.06, 1.16)	**5.93E-05**	1.03 (0.98, 1.07)	3.14E-01
0.88 (0.84, 0.92)	**3.57E-08**	0.97 (0.92, 1.01)	2.15E-01
0.98 (0.94, 1.03)	5.76E-01	0.94 (0.90, 0.98)	**1.02E-02**
1.06 (1.02, 1.11)	**1.08E-02**	1.12 (1.07, 1.17)	**1.15E-06**
1.05 (1.00, 1.09)	7.76E-02	1.11 (1.06, 1.16)	**1.78E-05**
1.17 (1.12, 1.22)	**4.60E-12**	1.16 (1.12, 1.21)	**1.86E-11**
0.94 (0.90, 0.99)	**1.85E-02**	0.91 (0.87, 0.95)	**3.30E-05**
0.96 (0.92, 1.00)	1.33E-01	0.93 (0.89, 0.96)	**7.38E-04**
1.03 (0.99, 1.08)	2.55E-01	1.15 (1.10, 1.20)	**9.04E-09**
1.03 (0.98, 1.07)	3.02E-01	1.13 (1.08, 1.18)	**1.29E-07**
1.02 (0.98, 1.07)	4.40E-01	1.11 (1.06, 1.16)	**6.01E-05**
0.99 (0.95, 1.04)	8.50E-01	0.93 (0.89, 0.98)	**6.75E-03**
0.88 (0.84, 0.92)	**2.13E-08**	0.93 (0.89, 0.97)	**2.12E-03**
0.92 (0.89, 0.96)	**2.06E-04**	1.00 (0.96, 1.04)	9.71E-01
0.89 (0.85, 0.92)	**5.61E-08**	0.95 (0.92, 0.99)	**4.84E-02**
1.01 (0.97, 1.06)	7.19E-01	1.06 (1.01, 1.11)	**2.74E-02**
1.12 (1.08, 1.16)	**4.53E-08**	1.02 (0.98, 1.07)	3.67E-01
0.91 (0.87, 0.96)	**7.48E-04**	0.97 (0.93, 1.02)	3.10E-01
0.94 (0.91, 0.98)	**1.08E-02**	0.97 (0.93, 1.01)	2.84E-01
0.94 (0.90, 0.98)	**6.54E-03**	0.95 (0.91, 0.99)	**3.61E-02**
1.00 (0.96, 1.05)	9.78E-01	1.07 (1.02, 1.12)	**1.13E-02**
1.14 (1.10, 1.18)	**1.19E-12**	1.06 (1.02, 1.11)	**6.46E-03**
0.96 (0.92, 1.01)	1.45E-01	0.97 (0.92, 1.01)	2.28E-01

0.97 (0.93, 1.01)	1.66E-01	0.99 (0.95, 1.04)	8.37E-01
0.98 (0.94, 1.02)	4.59E-01	0.99 (0.95, 1.03)	6.72E-01
0.97 (0.93, 1.02)	3.47E-01	1.03 (0.98, 1.08)	2.94E-01
1.16 (1.12, 1.21)	**4.95E-14**	1.08 (1.03, 1.12)	**1.46E-03**
0.97 (0.93, 1.01)	2.44E-01	0.97 (0.93, 1.02)	2.94E-01
0.99 (0.94, 1.03)	6.13E-01	1.01 (0.96, 1.05)	8.36E-01
0.99 (0.95, 1.04)	8.08E-01	1.00 (0.96, 1.05)	9.84E-01
0.98 (0.93, 1.02)	3.83E-01	1.02 (0.98, 1.07)	4.26E-01
1.10 (1.06, 1.14)	**1.31E-05**	1.04 (1.00, 1.08)	1.42E-01
1.06 (1.01, 1.11)	**1.78E-02**	0.99 (0.95, 1.04)	7.52E-01
0.93 (0.89, 0.97)	**1.86E-03**	1.03 (0.98, 1.08)	2.83E-01
0.93 (0.89, 0.97)	**1.05E-03**	1.03 (0.98, 1.07)	3.51E-01
0.98 (0.94, 1.02)	3.72E-01	1.03 (0.99, 1.08)	2.09E-01
1.02 (0.98, 1.06)	4.36E-01	0.95 (0.91, 1.00)	6.79E-02
0.85 (0.81, 0.89)	**3.54E-11**	0.88 (0.84, 0.92)	**8.18E-08**
1.06 (1.02, 1.11)	**1.38E-02**	1.12 (1.07, 1.17)	**4.78E-06**
1.05 (1.00, 1.09)	8.17E-02	1.07 (1.03, 1.12)	**5.11E-03**
1.08 (1.03, 1.13)	**3.77E-03**	1.16 (1.11, 1.22)	**2.16E-09**
1.13 (1.09, 1.17)	**5.14E-09**	1.00 (0.96, 1.04)	9.38E-01
1.24 (1.20, 1.27)	**1.04E-48**	1.17 (1.13, 1.21)	**1.25E-17**
0.84 (0.81, 0.87)	**6.32E-23**	0.93 (0.89, 0.96)	**7.38E-04**
0.83 (0.80, 0.86)	**1.26E-27**	0.92 (0.88, 0.96)	**1.10E-04**
0.95 (0.91, 1.00)	6.50E-02	0.99 (0.95, 1.03)	6.91E-01
1.07 (1.03, 1.12)	**3.30E-03**	0.96 (0.92, 1.01)	1.71E-01
0.96 (0.91, 1.00)	7.76E-02	0.98 (0.94, 1.02)	4.52E-01
1.01 (0.97, 1.06)	7.17E-01	1.02 (0.98, 1.07)	3.94E-01
1.01 (0.97, 1.06)	7.19E-01	1.02 (0.97, 1.06)	5.66E-01
0.99 (0.95, 1.04)	8.37E-01	1.05 (1.00, 1.09)	8.95E-02
1.09 (1.05, 1.14)	**7.89E-05**	0.98 (0.94, 1.03)	4.79E-01
0.92 (0.88, 0.97)	**1.19E-03**	0.93 (0.89, 0.97)	**3.42E-03**
1.18 (1.13, 1.23)	**2.00E-13**	1.10 (1.05, 1.15)	**1.46E-04**
1.17 (1.12, 1.22)	**2.69E-12**	1.18 (1.14, 1.23)	**3.85E-14**
1.07 (1.03, 1.12)	**3.41E-03**	1.03 (0.98, 1.07)	2.94E-01
1.06 (1.01, 1.10)	**1.79E-02**	1.02 (0.97, 1.06)	5.33E-01
1.07 (1.03, 1.12)	**3.12E-03**	1.02 (0.97, 1.06)	5.36E-01
1.03 (0.99, 1.08)	2.12E-01	0.99 (0.95, 1.03)	6.74E-01
1.06 (1.01, 1.11)	**1.98E-02**	1.08 (1.03, 1.12)	**2.15E-03**
1.05 (1.00, 1.09)	7.19E-02	1.06 (1.02, 1.11)	**1.26E-02**
1.15 (1.10, 1.20)	**1.55E-09**	1.16 (1.12, 1.21)	**5.92E-12**
1.04 (0.99, 1.08)	1.54E-01	0.92 (0.87, 0.96)	**6.43E-04**
1.03 (0.99, 1.07)	2.87E-01	0.94 (0.89, 0.98)	**1.30E-02**
1.03 (0.98, 1.07)	3.08E-01	0.97 (0.93, 1.02)	3.14E-01
0.99 (0.95, 1.04)	8.50E-01	0.96 (0.92, 1.01)	1.84E-01
1.20 (1.15, 1.24)	**4.80E-20**	1.05 (1.01, 1.10)	**4.43E-02**
1.12 (1.07, 1.17)	**2.74E-07**	0.99 (0.95, 1.04)	7.66E-01
1.05 (1.01, 1.10)	**4.22E-02**	1.00 (0.96, 1.05)	9.71E-01
0.97 (0.93, 1.02)	3.65E-01	0.97 (0.93, 1.02)	3.38E-01
1.01 (0.97, 1.06)	7.17E-01	0.90 (0.86, 0.94)	**1.17E-05**

0.99 (0.95, 1.03)	7.04E-01	0.86 (0.82, 0.90)	**5.14E-09**
1.07 (1.02, 1.12)	**7.31E-03**	0.97 (0.93, 1.02)	3.38E-01
1.07 (1.02, 1.11)	**9.15E-03**	1.07 (1.03, 1.12)	**2.29E-03**
1.04 (1.00, 1.09)	8.73E-02	0.98 (0.94, 1.03)	5.36E-01
1.02 (0.98, 1.06)	4.95E-01	0.95 (0.91, 0.99)	5.32E-02
1.01 (0.96, 1.05)	8.39E-01	0.94 (0.90, 0.98)	**1.96E-02**
0.87 (0.84, 0.91)	**1.49E-09**	0.80 (0.77, 0.84)	**2.78E-23**
1.00 (0.96, 1.05)	9.27E-01	1.11 (1.07, 1.16)	**3.90E-06**
0.98 (0.93, 1.02)	4.19E-01	0.92 (0.88, 0.96)	**9.87E-04**
0.97 (0.93, 1.02)	3.52E-01	1.01 (0.97, 1.06)	6.51E-01
0.99 (0.95, 1.03)	7.10E-01	0.90 (0.86, 0.94)	**4.56E-05**
0.98 (0.94, 1.03)	6.11E-01	0.91 (0.87, 0.96)	**7.38E-04**
1.01 (0.97, 1.05)	7.89E-01	0.95 (0.91, 1.00)	8.46E-02
1.02 (0.98, 1.07)	4.20E-01	0.96 (0.91, 1.00)	9.54E-02
1.00 (0.96, 1.04)	9.80E-01	1.20 (1.15, 1.26)	**9.03E-16**
0.91 (0.87, 0.95)	**2.12E-05**	0.91 (0.87, 0.95)	**1.15E-04**
0.94 (0.90, 0.98)	**9.58E-03**	1.08 (1.04, 1.13)	**1.12E-03**
0.94 (0.90, 0.98)	**1.29E-02**	0.90 (0.86, 0.94)	**1.53E-05**
0.92 (0.88, 0.96)	**8.09E-04**	0.92 (0.88, 0.96)	**6.75E-04**
1.02 (0.98, 1.07)	4.23E-01	1.02 (0.98, 1.07)	4.12E-01
1.11 (1.06, 1.16)	**1.10E-05**	1.11 (1.07, 1.17)	**1.14E-05**
1.21 (1.17, 1.25)	**1.85E-32**	1.13 (1.09, 1.17)	**4.86E-11**
0.96 (0.92, 1.00)	1.06E-01	0.97 (0.93, 1.01)	2.63E-01
1.23 (1.18, 1.28)	**8.23E-20**	0.99 (0.95, 1.04)	8.33E-01
0.95 (0.91, 1.00)	5.36E-02	0.93 (0.89, 0.97)	**1.77E-03**
1.01 (0.97, 1.06)	6.13E-01	0.90 (0.86, 0.95)	**4.30E-05**
0.90 (0.86, 0.94)	**4.06E-06**	0.99 (0.95, 1.04)	7.52E-01
0.95 (0.91, 0.99)	**4.04E-02**	0.93 (0.89, 0.97)	**4.24E-03**
0.95 (0.91, 1.00)	7.19E-02	0.96 (0.91, 1.00)	1.15E-01
0.89 (0.85, 0.93)	**2.00E-06**	0.82 (0.79, 0.86)	**3.29E-14**
1.14 (1.11, 1.17)	**3.05E-18**	1.12 (1.08, 1.15)	**5.27E-11**
1.10 (1.05, 1.15)	**5.97E-05**	1.03 (0.99, 1.08)	2.41E-01
1.10 (1.07, 1.13)	**1.23E-08**	1.06 (1.03, 1.10)	**1.91E-03**
1.07 (1.03, 1.11)	**7.58E-04**	1.02 (0.97, 1.07)	5.36E-01
1.10 (1.06, 1.13)	**1.96E-07**	1.04 (0.99, 1.08)	1.56E-01
1.06 (1.05, 1.08)	**4.27E-11**	1.04 (1.01, 1.07)	**1.97E-02**
0.82 (0.79, 0.86)	**4.07E-21**	0.85 (0.82, 0.88)	**1.73E-14**
1.05 (1.01, 1.10)	**3.05E-02**	1.15 (1.10, 1.20)	**1.01E-09**

trations
trations
trations

tes

trations

tus, alcohol consumption, and self-rated health status. P values are corrected for multiple testing using
LDL, Very Low Density Lipoprotein; IDL, Intermediate Density Lipoprotein..

B			
UKB			
**Unadjusted HR (95% CI)**	**P value**	**Adjusted HR (95% CI)**	**P value**
1.11 (1.10, 1.13)	**5.53E-64**	0.98 (0.97, 1.00)	**1.54E-02**
1.11 (1.09, 1.12)	**1.60E-56**	0.98 (0.97, 1.00)	**9.12E-03**
1.12 (1.10, 1.13)	**1.21E-68**	0.98 (0.97, 1.00)	**3.03E-02**
1.10 (1.08, 1.11)	**4.00E-44**	0.98 (0.96, 0.99)	**9.99E-04**
1.08 (1.07, 1.10)	**1.11E-33**	0.97 (0.96, 0.99)	**1.75E-04**
1.11 (1.09, 1.12)	**1.98E-57**	0.98 (0.97, 0.99)	**7.14E-03**
1.10 (1.09, 1.12)	**1.55E-56**	0.98 (0.97, 1.00)	**1.36E-02**
1.08 (1.06, 1.09)	**5.31E-29**	0.95 (0.93, 0.96)	**8.88E-13**
1.07 (1.06, 1.09)	**2.54E-25**	0.95 (0.93, 0.96)	**1.08E-13**
1.07 (1.05, 1.08)	**3.52E-22**	0.95 (0.94, 0.96)	**1.89E-12**
1.01 (1.00, 1.03)	6.63E-02	0.93 (0.92, 0.94)	**4.42E-22**
0.97 (0.96, 0.99)	**7.35E-05**	0.92 (0.91, 0.93)	**4.80E-30**
1.05 (1.04, 1.07)	**1.18E-14**	0.95 (0.93, 0.96)	**3.64E-14**
1.09 (1.08, 1.11)	**1.82E-40**	0.95 (0.94, 0.97)	**4.10E-11**
1.05 (1.03, 1.06)	**2.88E-12**	0.93 (0.92, 0.95)	**2.78E-20**
1.03 (1.02, 1.05)	**2.22E-06**	0.92 (0.91, 0.94)	**3.73E-26**
1.06 (1.04, 1.07)	**1.09E-15**	0.94 (0.92, 0.95)	**3.08E-18**
1.00 (0.99, 1.02)	4.79E-01	0.92 (0.91, 0.94)	**2.96E-27**
0.97 (0.96, 0.98)	**1.86E-05**	0.92 (0.91, 0.93)	**1.35E-29**
1.04 (1.02, 1.05)	**8.29E-08**	0.93 (0.91, 0.94)	**7.89E-24**
1.04 (1.02, 1.05)	**1.55E-07**	0.92 (0.91, 0.94)	**1.35E-27**
0.88 (0.87, 0.90)	**3.20E-66**	0.90 (0.89, 0.91)	**1.29E-46**
0.91 (0.90, 0.92)	**2.10E-39**	0.90 (0.89, 0.91)	**6.49E-48**
0.88 (0.86, 0.89)	**5.92E-76**	0.90 (0.89, 0.92)	**2.27E-44**
0.79 (0.78, 0.81)	**2.51E-215**	0.90 (0.89, 0.92)	**1.34E-44**
0.76 (0.75, 0.78)	**4.41E-293**	0.91 (0.90, 0.92)	**7.16E-40**
0.85 (0.84, 0.86)	**2.26E-109**	0.90 (0.89, 0.92)	**7.48E-45**
1.01 (1.00, 1.02)	1.20E-01	0.91 (0.90, 0.93)	**8.74E-34**
1.01 (1.00, 1.03)	5.84E-02	0.94 (0.93, 0.96)	**3.57E-16**
0.99 (0.98, 1.01)	4.00E-01	0.94 (0.92, 0.95)	**1.43E-19**
0.91 (0.90, 0.93)	**3.16E-36**	0.92 (0.91, 0.93)	**2.42E-31**
0.91 (0.90, 0.92)	**9.78E-40**	0.93 (0.91, 0.94)	**2.73E-25**
0.94 (0.93, 0.96)	**3.92E-17**	0.94 (0.92, 0.95)	**1.71E-18**
0.86 (0.84, 0.87)	**1.50E-100**	0.91 (0.90, 0.92)	**1.39E-38**
1.11 (1.09, 1.12)	**7.64E-52**	0.96 (0.95, 0.98)	**1.06E-06**
0.96 (0.94, 0.97)	**4.64E-10**	1.00 (0.98, 1.01)	5.56E-01
0.97 (0.95, 0.98)	**1.68E-06**	1.00 (0.99, 1.02)	7.81E-01
1.01 (0.99, 1.02)	3.26E-01	1.02 (1.00, 1.03)	**1.19E-02**
0.87 (0.85, 0.88)	**7.83E-87**	0.98 (0.97, 0.99)	**4.42E-03**
0.84 (0.83, 0.85)	**4.96E-132**	0.97 (0.96, 0.99)	**1.04E-04**
0.95 (0.93, 0.96)	**6.53E-15**	1.00 (0.98, 1.01)	7.36E-01
1.16 (1.15, 1.18)	**2.50E-125**	1.03 (1.02, 1.05)	**9.46E-06**
0.80 (0.79, 0.81)	**2.31E-203**	0.93 (0.91, 0.94)	**2.05E-25**

0.78 (0.77, 0.79)	**4.73E-260**	0.93 (0.91, 0.94)	**6.74E-25**
0.80 (0.78, 0.81)	**1.35E-216**	0.94 (0.93, 0.95)	**6.51E-17**
0.75 (0.74, 0.76)	**0.00E+00**	0.91 (0.90, 0.93)	**3.60E-34**
0.74 (0.73, 0.75)	**0.00E+00**	0.91 (0.90, 0.92)	**6.65E-38**
0.77 (0.76, 0.79)	**7.82E-273**	0.93 (0.92, 0.94)	**1.85E-23**
1.15 (1.13, 1.16)	**3.03E-103**	1.05 (1.03, 1.06)	**8.69E-12**
0.80 (0.79, 0.82)	**7.54E-192**	0.91 (0.89, 0.92)	**7.43E-43**
0.77 (0.76, 0.78)	**2.15E-286**	0.89 (0.87, 0.90)	**4.54E-60**
0.76 (0.75, 0.77)	**3.60E-309**	0.89 (0.87, 0.90)	**3.02E-62**
0.75 (0.74, 0.76)	**0.00E+00**	0.88 (0.87, 0.89)	**7.40E-68**
0.76 (0.75, 0.77)	**3.45E-307**	0.88 (0.87, 0.89)	**9.29E-70**
0.73 (0.72, 0.74)	**0.00E+00**	0.89 (0.87, 0.90)	**1.70E-59**
1.14 (1.12, 1.15)	**1.64E-89**	1.04 (1.02, 1.05)	**3.44E-07**
0.84 (0.83, 0.85)	**1.78E-130**	0.89 (0.88, 0.91)	**4.82E-54**
0.81 (0.80, 0.82)	**6.55E-178**	0.87 (0.86, 0.89)	**1.90E-75**
0.80 (0.79, 0.82)	**2.63E-197**	0.87 (0.86, 0.88)	**7.55E-79**
0.80 (0.79, 0.81)	**7.17E-211**	0.87 (0.86, 0.88)	**1.42E-82**
0.82 (0.81, 0.83)	**1.06E-160**	0.87 (0.86, 0.88)	**1.46E-77**
0.75 (0.74, 0.76)	**0.00E+00**	0.87 (0.85, 0.88)	**3.97E-85**
1.13 (1.11, 1.14)	**1.77E-77**	1.01 (0.99, 1.02)	2.55E-01
0.85 (0.84, 0.86)	**6.73E-109**	0.91 (0.89, 0.92)	**6.11E-42**
0.82 (0.81, 0.83)	**2.04E-168**	0.89 (0.88, 0.90)	**1.13E-60**
0.82 (0.81, 0.83)	**9.16E-160**	0.90 (0.89, 0.92)	**1.53E-46**
0.79 (0.78, 0.81)	**1.98E-219**	0.88 (0.86, 0.89)	**1.15E-72**
0.82 (0.81, 0.83)	**5.36E-165**	0.88 (0.87, 0.89)	**1.45E-66**
0.76 (0.75, 0.77)	**0.00E+00**	0.88 (0.87, 0.89)	**1.21E-73**
1.11 (1.10, 1.12)	**3.59E-60**	0.99 (0.98, 1.00)	1.49E-01
0.89 (0.88, 0.91)	**1.77E-49**	1.09 (1.07, 1.11)	**9.00E-28**
0.91 (0.89, 0.92)	**8.86E-41**	1.10 (1.09, 1.12)	**1.85E-36**
0.92 (0.91, 0.94)	**2.24E-28**	1.11 (1.10, 1.13)	**3.21E-44**
0.88 (0.86, 0.89)	**7.44E-69**	1.08 (1.07, 1.10)	**4.71E-25**
0.86 (0.85, 0.88)	**3.47E-82**	1.08 (1.06, 1.09)	**4.30E-20**
0.93 (0.91, 0.94)	**1.67E-25**	1.11 (1.09, 1.13)	**8.72E-45**
1.10 (1.08, 1.11)	**1.28E-44**	1.05 (1.03, 1.06)	**2.62E-11**
0.87 (0.85, 0.88)	**3.80E-82**	1.06 (1.05, 1.08)	**1.93E-13**
0.88 (0.86, 0.89)	**1.46E-69**	1.07 (1.05, 1.09)	**5.31E-16**
0.89 (0.88, 0.90)	**3.41E-55**	1.07 (1.05, 1.09)	**2.78E-17**
0.85 (0.84, 0.87)	**1.20E-94**	1.06 (1.04, 1.08)	**1.05E-12**
0.85 (0.84, 0.86)	**8.34E-102**	1.06 (1.04, 1.07)	**4.29E-11**
0.88 (0.86, 0.89)	**2.86E-70**	1.07 (1.06, 1.09)	**6.47E-19**
1.08 (1.07, 1.10)	**1.44E-32**	1.07 (1.05, 1.08)	**5.66E-21**
0.86 (0.84, 0.87)	**5.48E-101**	0.97 (0.96, 0.99)	**2.01E-04**
0.88 (0.86, 0.89)	**4.45E-75**	0.97 (0.96, 0.99)	**1.26E-03**
0.90 (0.89, 0.91)	**1.13E-49**	0.98 (0.97, 0.99)	**1.04E-02**
0.84 (0.82, 0.85)	**3.96E-130**	0.97 (0.95, 0.98)	**2.73E-05**
0.83 (0.82, 0.85)	**6.12E-135**	0.96 (0.95, 0.98)	**5.17E-06**
0.85 (0.84, 0.86)	**2.13E-106**	0.98 (0.96, 0.99)	**8.93E-03**
1.12 (1.11, 1.14)	**4.36E-69**	1.01 (1.00, 1.03)	5.64E-02

0.82 (0.80, 0.83)	**4.19E-175**	0.86 (0.84, 0.87)	**4.24E-99**
0.88 (0.87, 0.89)	**4.11E-68**	0.89 (0.88, 0.90)	**8.84E-57**
0.91 (0.89, 0.92)	**1.19E-43**	0.91 (0.90, 0.92)	**8.47E-39**
0.80 (0.79, 0.81)	**8.51E-203**	0.86 (0.85, 0.87)	**2.76E-95**
0.79 (0.78, 0.80)	**2.98E-227**	0.85 (0.84, 0.86)	**1.48E-109**
0.86 (0.85, 0.87)	**8.34E-96**	0.91 (0.89, 0.92)	**1.34E-40**
1.16 (1.14, 1.17)	**5.40E-108**	0.99 (0.97, 1.00)	6.89E-02
1.19 (1.17, 1.20)	**2.80E-113**	1.04 (1.03, 1.06)	**3.73E-07**
0.94 (0.92, 0.95)	**5.79E-17**	1.02 (1.00, 1.03)	**3.48E-02**
0.93 (0.91, 0.94)	**1.66E-20**	1.01 (0.99, 1.02)	2.61E-01
0.97 (0.96, 0.98)	**2.94E-05**	1.03 (1.01, 1.04)	**3.55E-04**
1.01 (0.99, 1.02)	4.27E-01	0.97 (0.96, 0.99)	**7.64E-04**
0.81 (0.80, 0.83)	**2.12E-136**	1.02 (1.00, 1.03)	**2.79E-02**
0.80 (0.79, 0.82)	**1.24E-146**	1.01 (0.99, 1.02)	4.53E-01
0.90 (0.89, 0.91)	**6.27E-48**	1.06 (1.04, 1.07)	**2.76E-13**
1.18 (1.17, 1.20)	**1.82E-112**	0.98 (0.96, 0.99)	**3.69E-03**
1.20 (1.18, 1.22)	**3.88E-98**	1.02 (1.00, 1.04)	**3.19E-02**
0.93 (0.92, 0.94)	**8.19E-25**	1.05 (1.04, 1.07)	**1.23E-11**
0.88 (0.86, 0.89)	**2.87E-74**	1.05 (1.03, 1.06)	**1.02E-10**
1.10 (1.08, 1.11)	**1.07E-38**	1.04 (1.03, 1.06)	**2.69E-08**
0.97 (0.95, 0.98)	**3.65E-06**	0.95 (0.94, 0.97)	**4.24E-10**
0.81 (0.80, 0.82)	**1.71E-238**	0.99 (0.98, 1.00)	1.96E-01
0.78 (0.77, 0.79)	**5.71E-317**	0.99 (0.97, 1.00)	6.82E-02
0.77 (0.76, 0.78)	**0.00E+00**	0.99 (0.97, 1.00)	**4.96E-02**
0.80 (0.79, 0.81)	**2.90E-250**	0.99 (0.98, 1.00)	1.71E-01
1.28 (1.26, 1.30)	**1.76E-307**	1.01 (1.00, 1.03)	8.27E-02
0.72 (0.71, 0.73)	**0.00E+00**	0.93 (0.91, 0.94)	**2.68E-25**
0.78 (0.77, 0.79)	**4.89E-288**	0.96 (0.95, 0.98)	**9.82E-08**
0.86 (0.84, 0.87)	**5.98E-118**	0.99 (0.98, 1.01)	2.60E-01
0.72 (0.71, 0.73)	**0.00E+00**	0.93 (0.91, 0.94)	**1.11E-24**
1.31 (1.29, 1.33)	**0.00E+00**	1.05 (1.04, 1.07)	**3.62E-12**
1.42 (1.40, 1.44)	**0.00E+00**	1.15 (1.14, 1.17)	**2.80E-85**
0.73 (0.72, 0.74)	**0.00E+00**	0.94 (0.92, 0.95)	**2.12E-21**
0.72 (0.71, 0.73)	**0.00E+00**	0.93 (0.92, 0.94)	**1.41E-23**
0.85 (0.84, 0.86)	**3.51E-138**	0.97 (0.96, 0.99)	**6.21E-05**
1.32 (1.31, 1.34)	**0.00E+00**	1.05 (1.03, 1.06)	**1.52E-10**
1.26 (1.24, 1.28)	**5.78E-250**	1.12 (1.11, 1.14)	**2.83E-63**
0.72 (0.71, 0.73)	**0.00E+00**	0.89 (0.87, 0.90)	**6.36E-81**
0.70 (0.69, 0.71)	**0.00E+00**	0.87 (0.85, 0.88)	**5.47E-109**
0.87 (0.86, 0.88)	**1.35E-102**	0.98 (0.97, 0.99)	**1.81E-03**
1.35 (1.34, 1.37)	**0.00E+00**	1.11 (1.10, 1.12)	**7.02E-63**
1.01 (0.99, 1.02)	4.19E-01	1.02 (1.01, 1.03)	**3.04E-03**
0.88 (0.88, 0.89)	**0.00E+00**	0.92 (0.92, 0.93)	**8.93E-126**
0.90 (0.90, 0.91)	**0.00E+00**	0.92 (0.92, 0.93)	**1.29E-149**
0.79 (0.78, 0.80)	**0.00E+00**	0.95 (0.94, 0.96)	**9.37E-15**
1.18 (1.17, 1.18)	**0.00E+00**	1.11 (1.10, 1.12)	**1.42E-124**
0.98 (0.96, 0.99)	**4.49E-04**	1.02 (1.00, 1.03)	**1.05E-02**
0.88 (0.88, 0.89)	**0.00E+00**	0.92 (0.91, 0.92)	**1.05E-135**

0.94 (0.93, 0.95)	**9.24E-21**	0.91 (0.90, 0.92)	**2.49E-62**
0.80 (0.79, 0.81)	**1.15E-306**	0.96 (0.95, 0.97)	**1.94E-09**
1.16 (1.16, 1.17)	**0.00E+00**	1.10 (1.09, 1.11)	**6.58E-155**
1.10 (1.09, 1.12)	**8.06E-48**	1.11 (1.10, 1.12)	**1.31E-57**
0.84 (0.83, 0.84)	**0.00E+00**	0.89 (0.88, 0.90)	**6.08E-159**
0.90 (0.89, 0.91)	**4.43E-57**	0.91 (0.89, 0.92)	**8.14E-53**
0.81 (0.80, 0.82)	**6.18E-298**	0.94 (0.93, 0.95)	**2.68E-21**
1.17 (1.17, 1.18)	**0.00E+00**	1.08 (1.06, 1.09)	**3.16E-36**
0.99 (0.98, 1.00)	1.15E-01	1.11 (1.09, 1.13)	**1.06E-39**
0.90 (0.89, 0.92)	**7.25E-42**	0.89 (0.88, 0.91)	**8.55E-53**
0.81 (0.80, 0.82)	**5.71E-317**	0.92 (0.91, 0.93)	**3.99E-45**
1.10 (1.09, 1.11)	**3.02E-58**	0.95 (0.94, 0.97)	**1.23E-10**
1.10 (1.09, 1.11)	**3.25E-95**	0.98 (0.97, 0.99)	**2.94E-03**
1.24 (1.22, 1.25)	**3.19E-278**	1.01 (0.99, 1.02)	4.35E-01
0.82 (0.81, 0.83)	**6.33E-274**	0.99 (0.98, 1.01)	3.98E-01
0.81 (0.80, 0.82)	**1.27E-284**	0.98 (0.97, 1.00)	**1.12E-02**
0.89 (0.88, 0.90)	**1.39E-83**	1.04 (1.03, 1.06)	**6.09E-10**
1.12 (1.11, 1.13)	**1.33E-181**	1.00 (0.99, 1.02)	4.87E-01
1.29 (1.27, 1.30)	**0.00E+00**	1.06 (1.05, 1.08)	**9.33E-18**
0.80 (0.79, 0.81)	**1.13E-300**	0.95 (0.94, 0.96)	**8.48E-13**
0.81 (0.80, 0.82)	**9.21E-244**	0.95 (0.94, 0.96)	**9.27E-13**
0.80 (0.79, 0.82)	**1.25E-228**	0.97 (0.96, 0.99)	**2.86E-04**
1.13 (1.13, 1.14)	**0.00E+00**	1.04 (1.03, 1.06)	**1.64E-08**
1.15 (1.13, 1.16)	**5.22E-90**	1.13 (1.11, 1.15)	**2.43E-63**
0.78 (0.77, 0.79)	**0.00E+00**	0.90 (0.89, 0.91)	**6.40E-55**
0.79 (0.78, 0.80)	**3.01E-294**	0.89 (0.87, 0.90)	**2.17E-70**
0.93 (0.92, 0.94)	**1.87E-22**	1.06 (1.04, 1.07)	**4.24E-14**
1.25 (1.24, 1.27)	**0.00E+00**	1.05 (1.04, 1.07)	**3.45E-13**
1.07 (1.05, 1.08)	**1.57E-21**	0.91 (0.89, 0.92)	**2.66E-40**
0.85 (0.84, 0.86)	**4.36E-135**	0.99 (0.98, 1.00)	1.63E-01
0.92 (0.91, 0.94)	**5.41E-29**	1.12 (1.10, 1.13)	**1.46E-44**
0.75 (0.74, 0.76)	**0.00E+00**	0.90 (0.88, 0.91)	**1.57E-48**
0.92 (0.90, 0.93)	**1.33E-34**	0.93 (0.92, 0.94)	**1.56E-24**
0.76 (0.75, 0.77)	**1.18E-296**	0.88 (0.86, 0.89)	**5.64E-74**
0.82 (0.81, 0.83)	**3.47E-167**	0.92 (0.90, 0.93)	**3.72E-34**
0.75 (0.74, 0.76)	**0.00E+00**	0.90 (0.88, 0.91)	**3.33E-50**
0.78 (0.76, 0.79)	**1.21E-265**	0.90 (0.89, 0.92)	**7.27E-43**
1.09 (1.08, 1.10)	**1.20E-38**	0.96 (0.95, 0.98)	**1.37E-07**
1.07 (1.06, 1.09)	**2.14E-27**	0.95 (0.93, 0.96)	**4.73E-13**
1.13 (1.12, 1.15)	**2.22E-84**	1.02 (1.01, 1.04)	**1.28E-03**
1.13 (1.12, 1.15)	**5.57E-81**	1.02 (1.01, 1.03)	**6.01E-03**
0.86 (0.84, 0.87)	**7.94E-102**	0.94 (0.93, 0.96)	**4.51E-14**
1.18 (1.17, 1.20)	**7.08E-147**	0.98 (0.97, 1.00)	**2.07E-02**
0.83 (0.82, 0.84)	**1.08E-142**	0.94 (0.92, 0.95)	**1.53E-17**
0.82 (0.81, 0.83)	**1.05E-170**	0.96 (0.95, 0.98)	**1.95E-07**
0.82 (0.81, 0.84)	**2.53E-158**	0.94 (0.92, 0.95)	**1.87E-17**

0.85 (0.83, 0.86)	**7.43E-116**	0.97 (0.95, 0.98)	**1.25E-04**
0.82 (0.81, 0.83)	**1.62E-156**	0.91 (0.89, 0.92)	**2.23E-41**
0.94 (0.92, 0.95)	**4.47E-20**	0.94 (0.93, 0.95)	**1.02E-17**
0.97 (0.96, 0.99)	**1.50E-04**	0.95 (0.94, 0.96)	**1.56E-12**
0.76 (0.75, 0.77)	**0.00E+00**	0.87 (0.85, 0.88)	**1.56E-82**
0.84 (0.83, 0.85)	**9.81E-115**	0.88 (0.86, 0.89)	**4.75E-60**
0.78 (0.77, 0.79)	**5.81E-249**	0.90 (0.89, 0.91)	**1.02E-47**
0.91 (0.89, 0.92)	**6.21E-40**	0.87 (0.86, 0.89)	**1.33E-65**
0.80 (0.79, 0.81)	**3.36E-207**	0.90 (0.88, 0.91)	**4.16E-49**
0.80 (0.79, 0.82)	**4.28E-194**	0.88 (0.86, 0.89)	**3.89E-67**
1.09 (1.08, 1.11)	**6.36E-43**	0.99 (0.98, 1.01)	4.56E-01
1.02 (1.01, 1.04)	**8.06E-04**	0.98 (0.96, 0.99)	**1.90E-03**
0.85 (0.84, 0.87)	**9.32E-101**	0.91 (0.89, 0.92)	**6.63E-37**
0.71 (0.70, 0.72)	**0.00E+00**	0.92 (0.90, 0.93)	**5.79E-35**
0.90 (0.89, 0.92)	**4.22E-42**	0.88 (0.86, 0.89)	**2.88E-63**
0.78 (0.77, 0.79)	**0.00E+00**	0.95 (0.94, 0.96)	**2.76E-13**
0.76 (0.75, 0.77)	**0.00E+00**	0.91 (0.90, 0.92)	**1.10E-43**
1.33 (1.31, 1.34)	**0.00E+00**	1.09 (1.07, 1.10)	**3.64E-30**
1.19 (1.17, 1.20)	**1.23E-142**	1.08 (1.06, 1.09)	**1.20E-28**
1.18 (1.17, 1.19)	**0.00E+00**	1.10 (1.09, 1.11)	**1.02E-74**
1.09 (1.07, 1.10)	**6.82E-34**	1.04 (1.03, 1.06)	**2.28E-08**
1.11 (1.10, 1.13)	**2.28E-78**	1.07 (1.06, 1.08)	**1.66E-27**
1.05 (1.04, 1.07)	**8.61E-14**	0.99 (0.98, 1.00)	2.05E-01
0.98 (0.97, 1.00)	**1.06E-02**	0.95 (0.94, 0.97)	**1.21E-11**
0.87 (0.86, 0.88)	**2.60E-75**	0.92 (0.90, 0.93)	**7.54E-29**
1.04 (1.02, 1.05)	**6.19E-08**	0.97 (0.96, 0.99)	**2.18E-04**
0.99 (0.98, 1.01)	2.53E-01	0.92 (0.91, 0.94)	**8.56E-25**
1.01 (0.99, 1.02)	2.40E-01	0.93 (0.91, 0.94)	**6.87E-25**
1.04 (1.04, 1.05)	**1.02E-65**	1.04 (1.03, 1.05)	**4.56E-11**
1.11 (1.10, 1.12)	**1.11E-56**	1.03 (1.01, 1.04)	**1.88E-04**
1.08 (1.07, 1.08)	**2.63E-137**	1.07 (1.06, 1.07)	**2.17E-88**
1.09 (1.08, 1.10)	**8.71E-185**	1.06 (1.05, 1.07)	**8.86E-48**
1.08 (1.08, 1.09)	**4.23E-122**	1.07 (1.06, 1.08)	**5.70E-49**
1.10 (1.10, 1.11)	**0.00E+00**	1.05 (1.04, 1.06)	**6.65E-38**
0.76 (0.75, 0.77)	**0.00E+00**	0.85 (0.84, 0.87)	**4.14E-106**
1.30 (1.29, 1.32)	**0.00E+00**	1.13 (1.11, 1.15)	**2.65E-65**
0.85 (0.84, 0.86)	**2.10E-100**	1.00 (0.98, 1.01)	5.83E-01
1.01 (1.00, 1.02)	1.43E-01	0.93 (0.92, 0.95)	**5.01E-20**
0.79 (0.77, 0.80)	**9.71E-238**	0.89 (0.88, 0.91)	**1.45E-54**
0.75 (0.74, 0.76)	**0.00E+00**	0.88 (0.87, 0.89)	**8.82E-68**
0.78 (0.77, 0.79)	**1.90E-259**	0.88 (0.86, 0.89)	**5.55E-74**
0.81 (0.79, 0.82)	**1.28E-179**	0.98 (0.97, 1.00)	**2.17E-02**
0.74 (0.73, 0.75)	**0.00E+00**	0.91 (0.89, 0.92)	**2.50E-36**
1.05 (1.04, 1.06)	**6.06E-17**	1.09 (1.08, 1.10)	**4.77E-80**
0.96 (0.95, 0.97)	**9.60E-09**	0.93 (0.92, 0.94)	**1.05E-23**
0.74 (0.73, 0.75)	**0.00E+00**	0.88 (0.87, 0.89)	**1.53E-68**
0.85 (0.83, 0.86)	**1.13E-109**	1.01 (1.00, 1.03)	1.25E-01
1.05 (1.04, 1.05)	**8.53E-22**	1.04 (1.03, 1.05)	**6.48E-14**

1.07 (1.07, 1.08)	**1.21E-154**	1.07 (1.06, 1.08)	**5.75E-65**
0.79 (0.78, 0.80)	**2.31E-236**	0.91 (0.89, 0.92)	**4.57E-37**
0.97 (0.96, 0.99)	**4.93E-05**	0.94 (0.93, 0.96)	**4.82E-16**
0.81 (0.80, 0.83)	**1.06E-171**	0.90 (0.89, 0.91)	**1.27E-47**
0.80 (0.79, 0.81)	**1.06E-196**	0.92 (0.90, 0.93)	**4.52E-28**
0.82 (0.81, 0.84)	**1.96E-159**	0.93 (0.92, 0.94)	**6.49E-22**
1.00 (0.98, 1.01)	5.48E-01	0.94 (0.93, 0.95)	**1.44E-17**
0.78 (0.77, 0.79)	**1.61E-262**	0.88 (0.87, 0.89)	**7.18E-67**
0.88 (0.87, 0.89)	**2.94E-70**	1.00 (0.99, 1.02)	5.98E-01
0.83 (0.82, 0.85)	**3.01E-139**	0.91 (0.90, 0.92)	**5.83E-37**
1.02 (1.00, 1.03)	**1.19E-02**	0.94 (0.92, 0.95)	**1.46E-18**
0.78 (0.77, 0.79)	**1.93E-248**	0.88 (0.87, 0.90)	**9.61E-66**
0.85 (0.84, 0.87)	**9.22E-101**	1.00 (0.98, 1.01)	8.08E-01
0.89 (0.87, 0.90)	**9.78E-64**	0.93 (0.92, 0.94)	**6.71E-24**
0.81 (0.80, 0.83)	**6.01E-165**	0.99 (0.97, 1.00)	1.46E-01
1.06 (1.04, 1.07)	**1.52E-12**	1.02 (1.00, 1.04)	**2.16E-02**

a false discovery rate (FDR) adjustment.

Supplementary

Metabolites	Group
Concentration of chylomicrons and extremely large VLDL particles	Lipoprotein subclasses
Total lipids in chylomicrons and extremely large VLDL	Lipoprotein subclasses
Phospholipids in chylomicrons and extremely large VLDL	Lipoprotein subclasses
Cholesterol in chylomicrons and extremely large VLDL	Lipoprotein subclasses
Cholesteryl esters in chylomicrons and extremely large VLDL	Lipoprotein subclasses
Free cholesterol in chylomicrons and extremely large VLDL	Lipoprotein subclasses
Triglycerides in chylomicrons and extremely large VLDL	Lipoprotein subclasses
Concentration of very large VLDL particles	Lipoprotein subclasses
Total lipids in very large VLDL	Lipoprotein subclasses
Phospholipids in very large VLDL	Lipoprotein subclasses
Cholesterol in very large VLDL	Lipoprotein subclasses
Cholesteryl esters in very large VLDL	Lipoprotein subclasses
Free cholesterol in very large VLDL	Lipoprotein subclasses
Triglycerides in very large VLDL	Lipoprotein subclasses
Concentration of large VLDL particles	Lipoprotein subclasses
Total lipids in large VLDL	Lipoprotein subclasses
Phospholipids in large VLDL	Lipoprotein subclasses
Cholesterol in large VLDL	Lipoprotein subclasses
Cholesteryl esters in large VLDL	Lipoprotein subclasses
Free cholesterol in large VLDL	Lipoprotein subclasses
Triglycerides in large VLDL	Lipoprotein subclasses
Concentration of medium VLDL particles	Lipoprotein subclasses
Total lipids in medium VLDL	Lipoprotein subclasses
Phospholipids in medium VLDL	Lipoprotein subclasses
Cholesterol in medium VLDL	Lipoprotein subclasses
Cholesteryl esters in medium VLDL	Lipoprotein subclasses
Free cholesterol in medium VLDL	Lipoprotein subclasses
Triglycerides in medium VLDL	Lipoprotein subclasses
Concentration of small VLDL particles	Lipoprotein subclasses
Total lipids in small VLDL	Lipoprotein subclasses
Phospholipids in small VLDL	Lipoprotein subclasses
Cholesterol in small VLDL	Lipoprotein subclasses
Cholesteryl esters in small VLDL	Lipoprotein subclasses
Free cholesterol in small VLDL	Lipoprotein subclasses
Triglycerides in small VLDL	Lipoprotein subclasses
Concentration of very small VLDL particles	Lipoprotein subclasses
Total lipids in very small VLDL	Lipoprotein subclasses
Phospholipids in very small VLDL	Lipoprotein subclasses
Cholesterol in very small VLDL	Lipoprotein subclasses
Cholesteryl esters in very small VLDL	Lipoprotein subclasses
Free cholesterol in very small VLDL	Lipoprotein subclasses

Triglycerides in very small VLDL	Lipoprotein subclasses
Concentration of IDL particles	Lipoprotein subclasses
Total lipids in IDL	Lipoprotein subclasses
Phospholipids in IDL	Lipoprotein subclasses
Cholesterol in IDL	Lipoprotein subclasses
Cholesteryl esters in IDL	Lipoprotein subclasses
Free cholesterol in IDL	Lipoprotein subclasses
Triglycerides in IDL	Lipoprotein subclasses
Concentration of large LDL particles	Lipoprotein subclasses
Total lipids in large LDL	Lipoprotein subclasses
Phospholipids in large LDL	Lipoprotein subclasses
Cholesterol in large LDL	Lipoprotein subclasses
Cholesteryl esters in large LDL	Lipoprotein subclasses
Free cholesterol in large LDL	Lipoprotein subclasses
Triglycerides in large LDL	Lipoprotein subclasses
Concentration of medium LDL particles	Lipoprotein subclasses
Total lipids in medium LDL	Lipoprotein subclasses
Phospholipids in medium LDL	Lipoprotein subclasses
Cholesterol in medium LDL	Lipoprotein subclasses
Cholesteryl esters in medium LDL	Lipoprotein subclasses
Free cholesterol in medium LDL	Lipoprotein subclasses
Triglycerides in medium LDL	Lipoprotein subclasses
Concentration of small LDL particles	Lipoprotein subclasses
Total lipids in small LDL	Lipoprotein subclasses
Phospholipids in small LDL	Lipoprotein subclasses
Cholesterol in small LDL	Lipoprotein subclasses
Cholesteryl esters in small LDL	Lipoprotein subclasses
Free cholesterol in small LDL	Lipoprotein subclasses
Triglycerides in small LDL	Lipoprotein subclasses
Concentration of very large HDL particles	Lipoprotein subclasses
Total lipids in very large HDL	Lipoprotein subclasses
Phospholipids in very large HDL	Lipoprotein subclasses
Cholesterol in very large HDL	Lipoprotein subclasses
Cholesteryl esters in very large HDL	Lipoprotein subclasses
Free cholesterol in very large HDL	Lipoprotein subclasses
Triglycerides in very large HDL	Lipoprotein subclasses
Concentration of large HDL particles	Lipoprotein subclasses
Total lipids in large HDL	Lipoprotein subclasses
Phospholipids in large HDL	Lipoprotein subclasses
Cholesterol in large HDL	Lipoprotein subclasses
Cholesteryl esters in large HDL	Lipoprotein subclasses
Free cholesterol in large HDL	Lipoprotein subclasses
Triglycerides in large HDL	Lipoprotein subclasses
**Concentration of medium HDL particles**	Lipoprotein subclasses
**Total lipids in medium HDL**	Lipoprotein subclasses
**Phospholipids in medium HDL**	Lipoprotein subclasses
**Cholesterol in medium HDL**	Lipoprotein subclasses
**Cholesteryl esters in medium HDL**	Lipoprotein subclasses

**Free cholesterol in medium HDL**	Lipoprotein subclasses
Triglycerides in medium HDL	Lipoprotein subclasses
Concentration of small HDL particles	Lipoprotein subclasses
Total lipids in small HDL	Lipoprotein subclasses
Phospholipids in small HDL	Lipoprotein subclasses
Cholesterol in small HDL	Lipoprotein subclasses
Cholesteryl esters in small HDL	Lipoprotein subclasses
**Free cholesterol in small HDL**	Lipoprotein subclasses
Triglycerides in small HDL	Lipoprotein subclasses
Phospholipids to total lipids ratio in chylomicrons and extremely larg	Relative lipoprotein lipid cc
Cholesterol to total lipids ratio in chylomicrons and extremely large V	Relative lipoprotein lipid cc
Cholesteryl esters to total lipids ratio in chylomicrons and extremely l	Relative lipoprotein lipid cc
Free cholesterol to total lipids ratio in chylomicrons and extremely la	Relative lipoprotein lipid cc
Triglycerides to total lipids ratio in chylomicrons and extremely large	Relative lipoprotein lipid cc
Phospholipids to total lipids ratio in very large VLDL /(lipds in ckb)	Relative lipoprotein lipid cc
**Cholesterol to total lipids ratio in very large VLDL**	Relative lipoprotein lipid cc
Cholesteryl esters to total lipids ratio in very large VLDL	Relative lipoprotein lipid cc
**Free cholesterol to total lipids ratio in very large VLDL**	Relative lipoprotein lipid cc
**Triglycerides to total lipids ratio in very large VLDL**	Relative lipoprotein lipid cc
Phospholipids to total lipids ratio in large VLDL	Relative lipoprotein lipid cc
Cholesterol to total lipids ratio in large VLDL	Relative lipoprotein lipid cc
Cholesteryl esters to total lipids ratio in large VLDL	Relative lipoprotein lipid cc
Free cholesterol to total lipids ratio in large VLDL	Relative lipoprotein lipid cc
Triglycerides to total lipids ratio in large VLDL	Relative lipoprotein lipid cc
Phospholipids to total lipids ratio in medium VLDL	Relative lipoprotein lipid cc
**Cholesterol to total lipids ratio in medium VLDL**	Relative lipoprotein lipid cc
**Cholesteryl esters to total lipids ratio in medium VLDL**	Relative lipoprotein lipid cc
Free cholesterol to total lipids ratio in medium VLDL	Relative lipoprotein lipid cc
Triglycerides to total lipids ratio in medium VLDL	Relative lipoprotein lipid cc
**Phospholipids to total lipids ratio in small VLDL**	Relative lipoprotein lipid cc
Cholesterol to total lipids ratio in small VLDL	Relative lipoprotein lipid cc
Cholesteryl esters to total lipids ratio in small VLDL	Relative lipoprotein lipid cc
**Free cholesterol to total lipids ratio in small VLDL**	Relative lipoprotein lipid cc
Triglycerides to total lipids ratio in small VLDL	Relative lipoprotein lipid cc
Phospholipids to total lipids ratio in very small VLDL	Relative lipoprotein lipid cc
Cholesterol to total lipids ratio in very small VLDL	Relative lipoprotein lipid cc
Cholesteryl esters to total lipids ratio in very small VLDL	Relative lipoprotein lipid cc
Free cholesterol to total lipids ratio in very small VLDL	Relative lipoprotein lipid cc
Triglycerides to total lipids ratio in very small VLDL	Relative lipoprotein lipid cc
Phospholipids to total lipids ratio in IDL	Relative lipoprotein lipid cc
Cholesterol to total lipids ratio in IDL	Relative lipoprotein lipid cc
Cholesteryl esters to total lipids ratio in IDL	Relative lipoprotein lipid cc
Free cholesterol to total lipids ratio in IDL	Relative lipoprotein lipid cc
Triglycerides to total lipids ratio in IDL	Relative lipoprotein lipid cc
Phospholipids to total lipids ratio in large LDL	Relative lipoprotein lipid cc
Cholesterol to total lipids ratio in large LDL	Relative lipoprotein lipid cc
Cholesteryl esters to total lipids ratio in large LDL	Relative lipoprotein lipid cc
Free cholesterol to total lipids ratio in large LDL	Relative lipoprotein lipid cc

Triglycerides to total lipids ratio in large LDL	Relative lipoprotein lipid cc
Phospholipids to total lipids ratio in medium LDL	Relative lipoprotein lipid cc
Cholesterol to total lipids ratio in medium LDL	Relative lipoprotein lipid cc
Cholesteryl esters to total lipids ratio in medium LDL	Relative lipoprotein lipid cc
Free cholesterol to total lipids ratio in medium LDL	Relative lipoprotein lipid cc
Triglycerides to total lipids ratio in medium LDL	Relative lipoprotein lipid cc
Phospholipids to total lipids ratio in small LDL	Relative lipoprotein lipid cc
Cholesterol to total lipids ratio in small LDL	Relative lipoprotein lipid cc
Cholesteryl esters to total lipids ratio in small LDL	Relative lipoprotein lipid cc
Free cholesterol to total lipids ratio in small LDL	Relative lipoprotein lipid cc
Triglycerides to total lipids ratio in small LDL	Relative lipoprotein lipid cc
Phospholipids to total lipids ratio in very large HDL	Relative lipoprotein lipid cc
Cholesterol to total lipids ratio in very large HDL	Relative lipoprotein lipid cc
Cholesteryl esters to total lipids ratio in very large HDL	Relative lipoprotein lipid cc
Free cholesterol to total lipids ratio in very large HDL	Relative lipoprotein lipid cc
Triglycerides to total lipids ratio in very large HDL	Relative lipoprotein lipid cc
Phospholipids to total lipids ratio in large HDL	Relative lipoprotein lipid cc
Cholesterol to total lipids ratio in large HDL	Relative lipoprotein lipid cc
Cholesteryl esters to total lipids ratio in large HDL	Relative lipoprotein lipid cc
Free cholesterol to total lipids ratio in large HDL	Relative lipoprotein lipid cc
Triglycerides to total lipids ratio in large HDL	Relative lipoprotein lipid cc
Phospholipids to total lipids ratio in medium HDL	Relative lipoprotein lipid cc
Cholesterol to total lipids ratio in medium HDL	Relative lipoprotein lipid cc
Cholesteryl esters to total lipids ratio in medium HDL	Relative lipoprotein lipid cc
Free cholesterol to total lipids ratio in medium HDL	Relative lipoprotein lipid cc
Triglycerides to total lipids ratio in medium HDL	Relative lipoprotein lipid cc
Phospholipids to total lipids ratio in small HDL	Relative lipoprotein lipid cc
Cholesterol to total lipids ratio in small HDL	Relative lipoprotein lipid cc
Cholesteryl esters to total lipids ratio in small HDL	Relative lipoprotein lipid cc
Free cholesterol to total lipids ratio in small HDL	Relative lipoprotein lipid cc
Triglycerides to total lipids ratio in small HDL	Relative lipoprotein lipid cc
Average diameter for VLDL particles	Lipoprotein particle sizes
Average diameter for LDL particles	Lipoprotein particle sizes
Average diameter for HDL particles	Lipoprotein particle sizes
Total cholesterol	Cholesterol
VLDL cholesterol	Cholesterol
Remnant cholesterol (non-HDL, non-LDL -cholesterol)	Cholesterol
LDL cholesterol	Cholesterol
**HDL cholesterol**	Cholesterol
**HDL2 cholesterol**	Cholesterol
HDL3 cholesterol	Cholesterol
Total esterified cholesterol	Cholesteryl esters
Total free cholesterol	Free cholesterol
Total triglycerides	Triglycerides
Triglycerides in VLDL	Triglycerides
Triglycerides in LDL	Triglycerides
Triglycerides in HDL	Triglycerides
Phosphoglycerides	Other lipids

Ratio of triglycerides to phosphoglycerides	Other lipids
Phosphatidylcholines	Other lipids
Sphingomyelins	Other lipids
Total cholines	Other lipids
Apolipoprotein A1	Apolipoproteins
Apolipoprotein B	Apolipoproteins
Ratio of apolipoprotein B to apolipoprotein A1	Apolipoproteins
Total fatty acids	Fatty acids
Degree of unsaturation	Fatty acids
Docosahexaenoic acid	Fatty acids
Linoleic acid	Fatty acids
Omega-3 fatty acids	Fatty acids
Omega-6 fatty acids	Fatty acids
Polyunsaturated fatty acids	Fatty acids
Monounsaturated fatty acids	Fatty acids
Saturated fatty acids	Fatty acids
Ratio of docosahexaenoic acid to total fatty acids	Fatty acids
Ratio of linoleic acid to total fatty acids	Fatty acids
Ratio of omega-3 fatty acids to total fatty acids	Fatty acids
Ratio of omega-6 fatty acids to total fatty acids	Fatty acids
Ratio of polyunsaturated fatty acids to total fatty acids	Fatty acids
Ratio of monounsaturated fatty acids to total fatty acids	Fatty acids
Ratio of saturated fatty acids to total fatty acids	Fatty acids
Glucose	Glycolysis related metabolit
Lactate	Glycolysis related metabolit
Citrate	Glycolysis related metabolit
Alanine	Amino acids
Glutamine	Amino acids
Histidine	Amino acids
Isoleucine	Amino acids
Leucine	Amino acids
Valine	Amino acids
Phenylalanine	Amino acids
Tyrosine	Amino acids
Acetate	Ketone bodies
Acetoacetate	Ketone bodies
**3-Hydroxybutyrate**	Ketone bodies
Creatinine	Fluid balance
Albumin	Fluid balance
Glycoprotein acetyls	Inflammation
Acetone	Ketone bodies
Cholesteryl esters in HDL	Cholesteryl esters
Cholesteryl esters in LDL	Cholesteryl esters
Cholesteryl esters in VLDL/ (Cholesterol in ckb)	Cholesteryl esters
Clinical LDL cholesterol	Cholesterol
Concentration of HDL particles	Lipoprotein particle concent
Concentration of LDL particles	Lipoprotein particle concent
Concentration of VLDL particles	Lipoprotein particle concent

Free cholesterol in HDL	Free cholesterol
Free cholesterol in LDL	Free cholesterol
Free cholesterol in VLDL	Free cholesterol
Glycine	Amino acids
Phospholipids in HDL	Phospholipids
Phospholipids in LDL	Phospholipids
Phospholipids in VLDL	Phospholipids
Pyruvate	Glycolysis related metabolit
Ratio of omega-6 fatty acids to omega-3 fatty acids	Fatty acids
Ratio of polyunsaturated fatty acids to monounsaturated fatty acids	Fatty acids
Total cholesterol minus HDL-C	Cholesterol
Total concentration of branched-chain amino acids (leucine + isoleuci	Amino acids
Total concentration of lipoprotein particles	Lipoprotein particle concent
Total lipids in HDL	Total lipids
Total lipids in LDL	Total lipids
Total lipids in lipoprotein particles	Total lipids
Total lipids in VLDL	Total lipids
Total phospholipids in lipoprotein particles	Phospholipids

Models were adjusted for age, sex, regions (CKB only), ethnicity (UKB only), levels of education, smoking

HR, hazard ratio; CI, confidence interval; LDL, Low Density Lipoprotein; HDL, High Density Lipoprotein

Table 5. Association of all metabolites with cause-specific mortality in the CKB a

Ischaemic heart			
CKB			
**Unadjusted HR (95% CI)**	**P value**	**Adjusted HR (95% CI)**	**P value**
1.00 (0.93, 1.09)	9.49E-01	0.99 (0.92, 1.08)	9.32E-01
1.01 (0.93, 1.09)	9.29E-01	1.00 (0.92, 1.08)	9.59E-01
1.02 (0.94, 1.10)	7.63E-01	1.01 (0.93, 1.09)	9.01E-01
1.04 (0.97, 1.13)	4.31E-01	1.03 (0.95, 1.11)	6.59E-01
1.06 (0.98, 1.14)	2.59E-01	1.04 (0.96, 1.13)	4.54E-01
1.02 (0.94, 1.11)	7.35E-01	1.00 (0.92, 1.09)	9.70E-01
1.00 (0.92, 1.08)	9.41E-01	0.99 (0.91, 1.07)	8.57E-01
1.01 (0.93, 1.09)	8.66E-01	1.00 (0.92, 1.08)	9.70E-01
1.01 (0.94, 1.10)	8.38E-01	1.00 (0.92, 1.09)	1.00E+00
1.02 (0.94, 1.10)	7.64E-01	1.01 (0.93, 1.09)	9.25E-01
1.04 (0.96, 1.12)	5.47E-01	1.02 (0.94, 1.11)	7.28E-01
1.04 (0.96, 1.13)	4.85E-01	1.03 (0.95, 1.11)	6.77E-01
1.03 (0.95, 1.11)	6.53E-01	1.02 (0.94, 1.10)	8.36E-01
1.00 (0.93, 1.09)	9.41E-01	0.99 (0.91, 1.08)	9.00E-01
1.03 (0.95, 1.11)	6.53E-01	1.02 (0.94, 1.10)	8.37E-01
1.03 (0.95, 1.11)	6.53E-01	1.02 (0.94, 1.10)	8.37E-01
1.03 (0.95, 1.11)	6.53E-01	1.01 (0.93, 1.10)	8.57E-01
1.02 (0.95, 1.11)	7.09E-01	1.01 (0.93, 1.09)	9.04E-01
1.01 (0.94, 1.10)	8.09E-01	1.00 (0.92, 1.09)	9.87E-01
1.03 (0.95, 1.12)	6.15E-01	1.02 (0.94, 1.10)	8.37E-01
1.03 (0.95, 1.11)	6.53E-01	1.02 (0.94, 1.10)	8.20E-01
1.04 (0.96, 1.12)	5.28E-01	1.04 (0.96, 1.12)	5.55E-01
1.04 (0.96, 1.12)	5.28E-01	1.04 (0.96, 1.12)	5.56E-01
1.04 (0.96, 1.12)	5.54E-01	1.03 (0.95, 1.12)	6.15E-01
1.04 (0.96, 1.12)	5.47E-01	1.03 (0.95, 1.12)	6.13E-01
1.02 (0.95, 1.11)	7.09E-01	1.02 (0.94, 1.11)	7.28E-01
1.05 (0.97, 1.13)	3.95E-01	1.04 (0.96, 1.13)	5.16E-01
1.04 (0.96, 1.12)	5.15E-01	1.04 (0.96, 1.13)	5.18E-01
1.06 (0.98, 1.15)	2.48E-01	1.06 (0.98, 1.15)	2.74E-01
1.07 (0.99, 1.16)	1.67E-01	1.07 (0.99, 1.17)	1.67E-01
1.05 (0.97, 1.14)	3.44E-01	1.05 (0.96, 1.14)	4.36E-01
1.15 (1.06, 1.24)	**1.89E-03**	1.18 (1.10, 1.28)	**9.69E-05**
1.15 (1.07, 1.25)	**9.87E-04**	1.21 (1.12, 1.31)	**7.42E-06**
1.11 (1.03, 1.20)	**2.37E-02**	1.11 (1.03, 1.20)	**2.44E-02**
1.03 (0.95, 1.11)	6.86E-01	1.01 (0.93, 1.10)	9.01E-01
1.15 (1.07, 1.24)	**7.42E-04**	1.14 (1.06, 1.23)	**3.02E-03**
1.16 (1.08, 1.26)	**3.18E-04**	1.16 (1.07, 1.25)	**7.85E-04**
1.07 (0.99, 1.15)	2.00E-01	1.06 (0.97, 1.15)	3.19E-01
1.23 (1.14, 1.33)	**3.93E-07**	1.25 (1.16, 1.34)	**4.01E-08**
1.24 (1.15, 1.34)	**1.54E-07**	1.26 (1.17, 1.36)	**6.36E-09**
1.20 (1.11, 1.29)	**2.10E-05**	1.21 (1.12, 1.30)	**7.06E-06**

1.07 (0.99, 1.16)	1.44E-01	1.02 (0.94, 1.10)	8.37E-01
1.05 (0.97, 1.14)	3.44E-01	1.04 (0.96, 1.13)	5.56E-01
1.05 (0.97, 1.14)	3.73E-01	1.04 (0.96, 1.13)	5.11E-01
1.02 (0.94, 1.10)	7.56E-01	1.02 (0.94, 1.11)	8.36E-01
1.04 (0.96, 1.13)	4.79E-01	1.05 (0.97, 1.14)	3.92E-01
1.04 (0.96, 1.13)	5.15E-01	1.05 (0.97, 1.14)	4.36E-01
1.05 (0.97, 1.13)	4.13E-01	1.06 (0.97, 1.15)	3.19E-01
1.12 (1.04, 1.20)	**8.46E-03**	1.01 (0.93, 1.09)	9.32E-01
1.03 (0.95, 1.12)	5.77E-01	1.03 (0.95, 1.12)	6.76E-01
1.03 (0.95, 1.11)	6.50E-01	1.03 (0.95, 1.12)	6.80E-01
1.00 (0.92, 1.09)	9.86E-01	1.00 (0.92, 1.09)	9.79E-01
1.02 (0.94, 1.11)	7.35E-01	1.03 (0.95, 1.12)	6.29E-01
1.02 (0.94, 1.11)	7.35E-01	1.03 (0.95, 1.12)	6.13E-01
1.02 (0.94, 1.11)	7.35E-01	1.03 (0.95, 1.12)	6.80E-01
1.14 (1.07, 1.23)	**8.06E-04**	1.04 (0.95, 1.12)	5.70E-01
1.01 (0.93, 1.09)	8.77E-01	1.01 (0.93, 1.10)	9.00E-01
1.01 (0.93, 1.09)	9.25E-01	1.01 (0.93, 1.10)	9.00E-01
0.95 (0.88, 1.03)	3.66E-01	0.94 (0.86, 1.02)	2.35E-01
1.01 (0.93, 1.10)	8.47E-01	1.03 (0.95, 1.12)	6.35E-01
1.02 (0.94, 1.10)	7.64E-01	1.04 (0.96, 1.13)	5.20E-01
0.98 (0.91, 1.07)	8.08E-01	0.98 (0.90, 1.07)	8.37E-01
1.11 (1.03, 1.20)	**1.53E-02**	1.01 (0.92, 1.09)	9.32E-01
1.02 (0.94, 1.10)	7.58E-01	1.03 (0.95, 1.12)	6.37E-01
1.02 (0.94, 1.10)	7.64E-01	1.03 (0.95, 1.12)	6.33E-01
0.95 (0.88, 1.03)	3.76E-01	0.94 (0.87, 1.03)	3.19E-01
1.03 (0.95, 1.11)	6.53E-01	1.05 (0.97, 1.15)	3.58E-01
1.03 (0.95, 1.12)	6.14E-01	1.06 (0.98, 1.15)	2.80E-01
1.01 (0.93, 1.09)	9.32E-01	1.01 (0.93, 1.10)	8.57E-01
1.10 (1.02, 1.19)	**4.14E-02**	1.04 (0.96, 1.13)	5.39E-01
1.24 (1.15, 1.33)	**2.61E-07**	1.22 (1.13, 1.31)	**9.16E-07**
1.24 (1.15, 1.34)	**2.25E-07**	1.22 (1.14, 1.32)	**6.27E-07**
1.16 (1.07, 1.25)	**8.27E-04**	1.14 (1.05, 1.23)	**3.12E-03**
1.31 (1.22, 1.42)	**4.20E-11**	1.31 (1.21, 1.41)	**1.48E-11**
1.35 (1.25, 1.46)	**5.71E-13**	1.35 (1.25, 1.45)	**6.16E-14**
1.23 (1.14, 1.32)	**1.28E-06**	1.21 (1.12, 1.30)	**3.41E-06**
1.22 (1.14, 1.31)	**3.32E-07**	1.19 (1.10, 1.28)	**3.66E-05**
1.08 (1.00, 1.17)	1.31E-01	1.07 (0.99, 1.16)	1.75E-01
1.08 (1.00, 1.17)	1.28E-01	1.07 (0.99, 1.16)	1.61E-01
1.07 (0.99, 1.16)	2.00E-01	1.08 (0.99, 1.16)	1.57E-01
1.08 (1.00, 1.17)	1.04E-01	1.08 (0.99, 1.17)	1.47E-01
1.08 (0.99, 1.16)	1.38E-01	1.07 (0.99, 1.16)	2.08E-01
1.10 (1.02, 1.19)	**3.23E-02**	1.11 (1.02, 1.20)	**3.05E-02**
1.09 (1.01, 1.18)	6.42E-02	1.01 (0.93, 1.10)	9.28E-01
0.80 (0.73, 0.87)	**1.56E-06**	0.82 (0.76, 0.90)	**4.61E-05**
0.80 (0.74, 0.87)	**1.79E-06**	0.83 (0.76, 0.90)	**7.86E-05**
0.85 (0.78, 0.93)	**7.42E-04**	0.86 (0.79, 0.94)	**1.83E-03**
0.79 (0.73, 0.86)	**2.02E-07**	0.84 (0.77, 0.91)	**1.50E-04**
0.78 (0.72, 0.85)	**3.14E-08**	0.83 (0.77, 0.91)	**6.51E-05**

0.84 (0.77, 0.91)	**1.61E-04**	0.87 (0.80, 0.95)	**3.67E-03**
0.84 (0.77, 0.92)	**3.51E-04**	0.79 (0.73, 0.87)	**3.60E-06**
0.78 (0.73, 0.83)	**3.87E-12**	0.77 (0.72, 0.83)	**2.63E-11**
0.78 (0.73, 0.84)	**4.63E-12**	0.78 (0.72, 0.84)	**9.23E-11**
0.81 (0.75, 0.88)	**1.68E-06**	0.81 (0.75, 0.88)	**3.94E-06**
0.83 (0.77, 0.90)	**9.50E-06**	0.85 (0.79, 0.92)	**3.96E-04**
0.86 (0.80, 0.93)	**3.71E-04**	0.88 (0.82, 0.96)	**6.47E-03**
0.83 (0.77, 0.90)	**2.10E-05**	0.84 (0.77, 0.91)	**7.95E-05**
0.93 (0.86, 1.01)	2.00E-01	0.85 (0.78, 0.93)	**9.55E-04**
1.02 (0.94, 1.11)	7.63E-01	1.02 (0.93, 1.10)	8.40E-01
1.17 (1.10, 1.24)	**1.28E-06**	1.14 (1.07, 1.21)	**3.77E-04**
1.17 (1.10, 1.24)	**2.47E-06**	1.14 (1.07, 1.21)	**1.53E-04**
1.08 (1.00, 1.16)	8.34E-02	1.04 (0.95, 1.13)	5.75E-01
0.85 (0.79, 0.91)	**1.26E-05**	0.88 (0.81, 0.94)	**1.37E-03**
1.02 (0.93, 1.11)	7.69E-01	1.03 (0.94, 1.12)	6.59E-01
1.14 (1.08, 1.21)	**7.40E-05**	1.11 (1.05, 1.18)	**8.97E-04**
1.13 (1.06, 1.21)	**1.32E-03**	1.11 (1.04, 1.19)	**4.67E-03**
1.08 (0.99, 1.17)	1.38E-01	1.06 (0.98, 1.15)	2.30E-01
0.87 (0.80, 0.95)	**4.04E-03**	0.88 (0.81, 0.95)	**5.67E-03**
0.99 (0.91, 1.07)	8.47E-01	0.93 (0.86, 1.01)	1.47E-01
0.94 (0.87, 1.01)	1.99E-01	0.91 (0.84, 0.98)	**4.54E-02**
0.89 (0.82, 0.97)	**1.55E-02**	0.89 (0.81, 0.97)	**1.63E-02**
1.03 (0.95, 1.12)	6.68E-01	0.99 (0.91, 1.08)	9.01E-01
1.06 (0.98, 1.14)	2.80E-01	1.10 (1.02, 1.19)	**4.05E-02**
0.86 (0.79, 0.94)	**2.68E-03**	0.81 (0.74, 0.88)	**8.95E-06**
0.94 (0.86, 1.02)	2.12E-01	0.92 (0.84, 1.00)	9.95E-02
0.90 (0.83, 0.98)	**2.76E-02**	0.90 (0.82, 0.98)	**3.48E-02**
1.13 (1.04, 1.23)	**1.57E-02**	1.06 (0.97, 1.16)	3.13E-01
1.09 (1.00, 1.18)	1.03E-01	1.12 (1.03, 1.23)	**2.24E-02**
0.84 (0.77, 0.92)	**6.00E-04**	0.80 (0.73, 0.87)	**6.03E-06**
1.14 (1.05, 1.23)	**6.65E-03**	1.23 (1.13, 1.33)	**6.23E-06**
1.11 (1.02, 1.20)	**2.76E-02**	1.21 (1.11, 1.31)	**3.09E-05**
1.25 (1.15, 1.35)	**2.61E-07**	1.24 (1.15, 1.34)	**3.70E-07**
0.92 (0.84, 0.99)	7.41E-02	0.86 (0.79, 0.93)	**8.82E-04**
0.84 (0.78, 0.90)	**4.37E-06**	0.84 (0.78, 0.90)	**3.60E-06**
1.30 (1.19, 1.42)	**2.02E-07**	1.43 (1.31, 1.57)	**3.77E-13**
1.27 (1.16, 1.38)	**5.90E-07**	1.37 (1.26, 1.50)	**7.91E-12**
1.17 (1.07, 1.28)	**1.78E-03**	1.27 (1.15, 1.39)	**5.81E-06**
0.92 (0.84, 1.00)	1.03E-01	0.85 (0.78, 0.93)	**1.25E-03**
0.84 (0.78, 0.90)	**2.24E-05**	0.87 (0.80, 0.94)	**1.21E-03**
0.98 (0.91, 1.06)	7.62E-01	1.09 (1.00, 1.18)	1.16E-01
0.96 (0.88, 1.04)	4.20E-01	1.04 (0.96, 1.13)	4.59E-01
1.02 (0.94, 1.11)	7.15E-01	1.08 (0.99, 1.17)	1.96E-01
1.08 (1.00, 1.16)	1.04E-01	0.97 (0.90, 1.05)	6.35E-01
0.94 (0.86, 1.02)	2.66E-01	0.95 (0.87, 1.04)	4.36E-01
0.95 (0.88, 1.02)	2.59E-01	1.00 (0.92, 1.09)	9.70E-01
0.94 (0.87, 1.02)	2.18E-01	0.99 (0.92, 1.07)	9.01E-01
1.00 (0.93, 1.09)	9.49E-01	1.03 (0.95, 1.12)	6.03E-01

1.13 (1.05, 1.20)	**1.68E-03**	1.03 (0.96, 1.12)	5.75E-01
0.93 (0.86, 1.02)	2.12E-01	0.91 (0.83, 0.99)	8.72E-02
1.00 (0.92, 1.09)	9.86E-01	1.06 (0.97, 1.15)	3.26E-01
1.00 (0.92, 1.09)	9.86E-01	1.05 (0.96, 1.14)	4.59E-01
1.00 (0.92, 1.08)	9.91E-01	0.99 (0.91, 1.08)	9.28E-01
1.12 (1.04, 1.20)	**7.02E-03**	1.02 (0.94, 1.10)	7.89E-01
0.92 (0.85, 1.00)	1.31E-01	0.89 (0.82, 0.98)	**3.49E-02**
1.03 (0.94, 1.11)	6.94E-01	1.07 (0.98, 1.17)	2.08E-01
1.02 (0.94, 1.11)	7.55E-01	1.06 (0.98, 1.16)	2.80E-01
1.00 (0.93, 1.09)	9.41E-01	0.99 (0.91, 1.08)	8.91E-01
1.08 (1.00, 1.16)	9.76E-02	1.02 (0.94, 1.10)	8.35E-01
0.85 (0.79, 0.91)	**8.86E-05**	0.80 (0.74, 0.87)	**2.55E-07**
1.24 (1.14, 1.34)	**1.65E-06**	1.37 (1.26, 1.49)	**8.06E-12**
1.24 (1.15, 1.34)	**6.59E-07**	1.37 (1.26, 1.48)	**8.96E-13**
1.10 (1.01, 1.20)	8.35E-02	1.13 (1.04, 1.24)	**1.23E-02**
0.97 (0.90, 1.06)	6.58E-01	0.93 (0.85, 1.01)	1.57E-01
0.88 (0.81, 0.95)	**5.56E-03**	0.93 (0.86, 1.01)	1.92E-01
1.12 (1.03, 1.22)	**2.34E-02**	1.12 (1.03, 1.22)	**2.72E-02**
1.04 (0.95, 1.12)	5.56E-01	1.01 (0.93, 1.09)	9.24E-01
1.24 (1.13, 1.36)	**3.63E-05**	1.28 (1.17, 1.41)	**1.46E-06**
1.01 (0.94, 1.10)	8.40E-01	0.91 (0.84, 1.00)	9.96E-02
1.25 (1.19, 1.31)	**8.73E-19**	1.18 (1.12, 1.25)	**8.33E-08**
0.87 (0.81, 0.93)	**1.08E-04**	0.94 (0.87, 1.02)	2.67E-01
0.86 (0.81, 0.92)	**6.34E-05**	0.94 (0.87, 1.02)	2.62E-01
0.93 (0.86, 1.00)	1.18E-01	0.97 (0.89, 1.05)	5.85E-01
0.96 (0.88, 1.04)	4.85E-01	0.90 (0.82, 0.98)	**3.46E-02**
1.01 (0.93, 1.09)	9.41E-01	0.99 (0.92, 1.08)	9.32E-01
0.99 (0.91, 1.07)	8.57E-01	1.02 (0.94, 1.11)	7.14E-01
0.98 (0.91, 1.06)	7.56E-01	1.01 (0.93, 1.10)	8.76E-01
1.08 (1.00, 1.17)	1.11E-01	1.08 (1.00, 1.18)	1.13E-01
1.03 (0.95, 1.11)	6.53E-01	0.94 (0.86, 1.02)	2.30E-01
0.99 (0.91, 1.07)	8.40E-01	0.98 (0.91, 1.07)	8.37E-01
1.05 (0.97, 1.14)	3.84E-01	0.96 (0.88, 1.04)	4.95E-01
1.20 (1.11, 1.30)	**2.72E-05**	1.20 (1.11, 1.30)	**2.55E-05**
1.07 (0.99, 1.16)	1.80E-01	1.10 (1.02, 1.19)	**4.74E-02**
1.12 (1.04, 1.21)	**9.48E-03**	1.14 (1.05, 1.23)	**4.67E-03**
1.11 (1.02, 1.19)	**2.83E-02**	1.12 (1.03, 1.21)	**1.57E-02**
1.03 (0.95, 1.11)	6.53E-01	1.05 (0.97, 1.14)	3.95E-01
1.02 (0.94, 1.11)	7.47E-01	1.05 (0.97, 1.14)	3.58E-01
0.99 (0.91, 1.07)	8.91E-01	1.02 (0.94, 1.11)	7.50E-01
1.30 (1.20, 1.40)	**1.36E-09**	1.31 (1.21, 1.41)	**5.75E-11**
0.89 (0.82, 0.97)	**1.43E-02**	0.85 (0.78, 0.93)	**1.13E-03**
0.94 (0.87, 1.02)	2.59E-01	0.92 (0.84, 1.01)	1.48E-01
1.05 (0.97, 1.13)	3.67E-01	1.03 (0.95, 1.11)	6.88E-01
1.04 (0.96, 1.12)	5.04E-01	1.03 (0.95, 1.12)	6.08E-01
1.14 (1.06, 1.22)	**1.71E-03**	1.04 (0.96, 1.13)	5.18E-01
0.99 (0.92, 1.08)	9.10E-01	0.91 (0.83, 0.99)	7.64E-02
0.99 (0.91, 1.08)	8.96E-01	1.01 (0.93, 1.09)	9.28E-01

1.04 (0.96, 1.12)	5.28E-01	1.05 (0.97, 1.13)	4.09E-01
0.86 (0.79, 0.93)	**1.13E-03**	0.81 (0.75, 0.89)	**1.17E-05**
0.85 (0.79, 0.93)	**7.25E-04**	0.80 (0.74, 0.88)	**5.63E-06**
0.95 (0.88, 1.04)	4.20E-01	0.93 (0.86, 1.01)	2.08E-01
1.06 (0.98, 1.15)	3.00E-01	1.10 (1.02, 1.19)	**3.82E-02**
1.06 (0.98, 1.15)	2.57E-01	1.07 (0.99, 1.16)	2.08E-01
1.03 (0.95, 1.12)	6.31E-01	1.01 (0.93, 1.10)	9.00E-01
0.95 (0.87, 1.03)	3.66E-01	0.95 (0.87, 1.04)	3.96E-01
0.74 (0.69, 0.79)	**3.37E-19**	0.72 (0.67, 0.77)	**4.64E-20**
1.29 (1.20, 1.39)	**2.50E-11**	1.45 (1.36, 1.55)	**2.59E-25**
0.88 (0.81, 0.96)	**9.14E-03**	0.90 (0.82, 0.98)	**3.64E-02**
1.10 (1.02, 1.19)	**2.63E-02**	1.23 (1.14, 1.32)	**1.53E-06**
0.86 (0.79, 0.93)	**1.11E-03**	0.85 (0.77, 0.93)	**1.20E-03**
0.89 (0.82, 0.97)	**2.12E-02**	0.91 (0.83, 0.99)	8.11E-02
0.99 (0.92, 1.08)	9.33E-01	0.99 (0.91, 1.07)	8.78E-01
0.97 (0.89, 1.05)	5.92E-01	0.95 (0.88, 1.04)	4.57E-01
1.53 (1.41, 1.66)	**1.68E-23**	1.76 (1.63, 1.89)	**1.23E-46**
0.83 (0.78, 0.89)	**3.54E-07**	0.85 (0.79, 0.91)	**3.69E-05**
1.26 (1.17, 1.36)	**4.69E-08**	1.46 (1.36, 1.57)	**2.41E-22**
0.78 (0.72, 0.84)	**1.21E-09**	0.77 (0.71, 0.84)	**2.78E-09**
0.82 (0.76, 0.89)	**6.29E-06**	0.86 (0.80, 0.94)	**1.88E-03**
1.16 (1.07, 1.26)	**8.37E-04**	1.13 (1.04, 1.22)	**8.67E-03**
1.14 (1.06, 1.24)	**3.24E-03**	1.07 (0.99, 1.17)	1.96E-01
1.22 (1.15, 1.29)	**2.12E-11**	1.15 (1.09, 1.22)	**7.90E-06**
1.12 (1.04, 1.21)	**5.97E-03**	1.13 (1.05, 1.22)	**3.35E-03**
0.96 (0.89, 1.05)	5.45E-01	0.81 (0.74, 0.88)	**1.49E-05**
1.07 (0.99, 1.15)	2.01E-01	1.04 (0.96, 1.12)	5.24E-01
1.11 (1.03, 1.21)	**2.34E-02**	0.96 (0.88, 1.04)	4.95E-01
1.16 (1.08, 1.25)	**6.04E-04**	1.22 (1.13, 1.32)	**4.39E-06**
1.00 (0.92, 1.08)	9.41E-01	1.00 (0.92, 1.09)	9.90E-01
1.12 (1.04, 1.21)	**9.52E-03**	1.14 (1.05, 1.23)	**3.55E-03**
0.89 (0.82, 0.97)	**1.51E-02**	0.85 (0.78, 0.93)	**1.20E-03**
1.21 (1.17, 1.25)	**8.94E-25**	1.19 (1.15, 1.24)	**3.09E-18**
1.15 (1.06, 1.24)	**1.62E-03**	1.11 (1.02, 1.20)	**3.03E-02**
1.10 (1.04, 1.16)	**3.45E-03**	1.08 (1.01, 1.15)	**3.90E-02**
0.89 (0.81, 0.99)	6.32E-02	0.85 (0.76, 0.95)	**9.10E-03**
0.96 (0.87, 1.06)	6.14E-01	0.91 (0.82, 1.02)	2.14E-01
1.06 (1.02, 1.10)	**3.13E-03**	1.04 (0.98, 1.09)	3.15E-01
0.72 (0.68, 0.76)	**2.68E-27**	0.75 (0.70, 0.79)	**1.23E-19**
1.23 (1.15, 1.33)	**9.69E-08**	1.38 (1.29, 1.49)	**1.05E-16**

trations
trations
trations

es

trations

status, alcohol consumption, and self-rated health status. P values are corrected for multiple te
; VLDL, Very Low Density Lipoprotein; IDL, Intermediate Density Lipoprotein..

nd UKB

t diseases mortality

UKB			
Unadjusted HR (95% CI)	P value	Adjusted HR (95% CI)	P value
1.24 (1.20, 1.28)	**4.51E-35**	1.04 (1.00, 1.08)	8.43E-02
1.23 (1.19, 1.27)	**3.76E-33**	1.03 (0.99, 1.07)	1.21E-01
1.25 (1.21, 1.29)	**1.25E-36**	1.04 (1.00, 1.08)	7.32E-02
1.21 (1.17, 1.26)	**3.93E-25**	1.02 (0.98, 1.07)	3.00E-01
1.19 (1.15, 1.24)	**9.41E-21**	1.02 (0.98, 1.06)	4.70E-01
1.23 (1.19, 1.27)	**2.35E-30**	1.03 (0.99, 1.07)	1.60E-01
1.23 (1.19, 1.27)	**2.49E-34**	1.03 (1.00, 1.07)	1.05E-01
1.22 (1.17, 1.26)	**2.93E-25**	1.02 (0.98, 1.06)	4.84E-01
1.21 (1.16, 1.25)	**3.07E-23**	1.01 (0.97, 1.05)	6.53E-01
1.19 (1.15, 1.24)	**2.79E-20**	1.01 (0.97, 1.05)	6.67E-01
1.10 (1.06, 1.14)	**3.36E-06**	0.98 (0.94, 1.02)	3.37E-01
1.03 (0.99, 1.07)	2.00E-01	0.95 (0.91, 1.00)	**3.96E-02**
1.17 (1.12, 1.21)	**2.20E-15**	1.00 (0.96, 1.05)	9.29E-01
1.24 (1.19, 1.28)	**8.61E-32**	1.02 (0.98, 1.06)	3.56E-01
1.17 (1.13, 1.22)	**2.34E-16**	1.00 (0.96, 1.04)	9.35E-01
1.15 (1.11, 1.20)	**4.15E-13**	0.99 (0.94, 1.03)	5.41E-01
1.19 (1.14, 1.23)	**1.41E-18**	1.00 (0.96, 1.05)	8.69E-01
1.09 (1.05, 1.13)	**3.23E-05**	0.97 (0.93, 1.02)	2.50E-01
1.01 (0.97, 1.05)	6.39E-01	0.95 (0.91, 0.99)	**3.92E-02**
1.16 (1.12, 1.21)	**5.82E-14**	0.99 (0.95, 1.04)	7.56E-01
1.17 (1.12, 1.21)	**2.16E-15**	0.98 (0.94, 1.03)	5.15E-01
0.86 (0.82, 0.89)	**2.12E-12**	0.91 (0.87, 0.95)	**3.93E-05**
0.91 (0.88, 0.95)	**4.01E-05**	0.92 (0.88, 0.96)	**3.81E-04**
0.83 (0.80, 0.87)	**4.11E-16**	0.91 (0.87, 0.95)	**2.71E-05**
0.67 (0.64, 0.71)	**2.31E-64**	0.87 (0.83, 0.91)	**1.92E-09**
0.62 (0.59, 0.65)	**6.33E-95**	0.86 (0.82, 0.90)	**4.17E-11**
0.78 (0.75, 0.82)	**1.65E-27**	0.90 (0.86, 0.94)	**1.90E-06**
1.11 (1.07, 1.16)	**1.01E-07**	0.97 (0.93, 1.01)	2.37E-01
1.07 (1.03, 1.12)	**7.19E-04**	1.00 (0.96, 1.04)	9.18E-01
1.03 (0.99, 1.08)	1.02E-01	0.98 (0.94, 1.03)	5.10E-01
0.90 (0.86, 0.93)	**4.70E-07**	0.94 (0.90, 0.98)	**1.08E-02**
0.88 (0.84, 0.92)	**2.00E-09**	0.94 (0.90, 0.98)	**1.05E-02**
0.93 (0.89, 0.97)	**6.35E-04**	0.96 (0.92, 1.00)	1.00E-01
0.79 (0.76, 0.83)	**2.94E-25**	0.91 (0.87, 0.95)	**4.34E-05**
1.23 (1.18, 1.28)	**1.13E-27**	1.04 (1.00, 1.09)	7.32E-02
0.84 (0.81, 0.88)	**1.41E-14**	0.98 (0.94, 1.03)	5.22E-01
0.85 (0.81, 0.89)	**1.59E-13**	0.99 (0.95, 1.04)	7.28E-01
0.92 (0.88, 0.96)	**5.44E-05**	1.02 (0.98, 1.07)	3.47E-01
0.68 (0.65, 0.71)	**1.08E-60**	0.92 (0.88, 0.96)	**5.50E-04**
0.64 (0.61, 0.67)	**5.38E-82**	0.90 (0.86, 0.94)	**8.61E-06**
0.81 (0.78, 0.85)	**2.99E-20**	0.98 (0.93, 1.02)	3.34E-01

1.24 (1.20, 1.29)	**4.44E-33**	1.11 (1.06, 1.15)	**1.24E-06**
0.65 (0.62, 0.68)	**7.32E-76**	0.88 (0.84, 0.92)	**3.30E-08**
0.60 (0.57, 0.62)	**7.49E-112**	0.86 (0.82, 0.90)	**3.44E-10**
0.61 (0.58, 0.64)	**1.31E-100**	0.88 (0.84, 0.92)	**3.72E-08**
0.57 (0.54, 0.59)	**5.25E-136**	0.84 (0.80, 0.88)	**1.65E-13**
0.56 (0.54, 0.59)	**7.30E-141**	0.83 (0.80, 0.87)	**1.49E-14**
0.59 (0.57, 0.62)	**8.78E-117**	0.86 (0.83, 0.90)	**2.54E-10**
1.18 (1.14, 1.23)	**8.62E-19**	1.11 (1.06, 1.15)	**9.16E-07**
0.71 (0.68, 0.74)	**9.56E-50**	0.90 (0.86, 0.94)	**1.62E-06**
0.64 (0.61, 0.67)	**3.80E-84**	0.86 (0.82, 0.90)	**3.92E-11**
0.63 (0.61, 0.66)	**1.53E-89**	0.85 (0.81, 0.89)	**3.67E-12**
0.62 (0.59, 0.65)	**2.63E-97**	0.85 (0.81, 0.88)	**5.47E-13**
0.64 (0.61, 0.67)	**2.96E-86**	0.85 (0.81, 0.89)	**3.40E-12**
0.59 (0.56, 0.61)	**1.22E-124**	0.84 (0.80, 0.87)	**1.09E-14**
1.19 (1.15, 1.23)	**8.46E-21**	1.10 (1.06, 1.15)	**1.91E-06**
0.78 (0.75, 0.81)	**1.36E-28**	0.89 (0.85, 0.93)	**1.98E-07**
0.76 (0.73, 0.80)	**3.65E-33**	0.88 (0.84, 0.92)	**3.21E-08**
0.76 (0.73, 0.79)	**1.97E-34**	0.88 (0.84, 0.92)	**4.11E-08**
0.74 (0.71, 0.77)	**1.22E-41**	0.87 (0.83, 0.91)	**5.88E-10**
0.78 (0.74, 0.81)	**2.75E-29**	0.88 (0.84, 0.92)	**1.50E-08**
0.66 (0.63, 0.69)	**3.36E-78**	0.85 (0.81, 0.89)	**8.05E-13**
1.22 (1.18, 1.27)	**3.53E-29**	1.08 (1.04, 1.13)	**7.88E-05**
0.80 (0.76, 0.83)	**3.15E-24**	0.91 (0.87, 0.95)	**1.42E-05**
0.77 (0.74, 0.80)	**1.82E-31**	0.90 (0.86, 0.94)	**1.49E-06**
0.77 (0.74, 0.81)	**6.04E-31**	0.92 (0.88, 0.95)	**6.93E-05**
0.73 (0.70, 0.77)	**2.30E-43**	0.88 (0.84, 0.92)	**6.38E-09**
0.77 (0.74, 0.80)	**4.17E-31**	0.89 (0.85, 0.93)	**1.17E-07**
0.68 (0.65, 0.71)	**2.34E-73**	0.86 (0.83, 0.90)	**8.44E-11**
1.22 (1.18, 1.26)	**9.49E-29**	1.06 (1.02, 1.10)	**6.85E-03**
0.61 (0.58, 0.64)	**5.08E-72**	0.95 (0.90, 1.00)	7.54E-02
0.62 (0.59, 0.66)	**7.11E-67**	0.96 (0.91, 1.01)	1.80E-01
0.64 (0.61, 0.68)	**1.49E-59**	0.97 (0.92, 1.03)	3.63E-01
0.59 (0.56, 0.63)	**1.01E-79**	0.94 (0.89, 0.99)	**3.28E-02**
0.57 (0.54, 0.61)	**7.93E-86**	0.93 (0.88, 0.98)	**8.51E-03**
0.68 (0.65, 0.72)	**3.48E-52**	0.99 (0.94, 1.04)	7.31E-01
1.06 (1.02, 1.10)	**6.64E-03**	1.05 (1.01, 1.09)	**1.79E-02**
0.56 (0.53, 0.60)	**1.91E-90**	0.89 (0.84, 0.95)	**1.97E-04**
0.58 (0.55, 0.62)	**7.92E-84**	0.91 (0.86, 0.96)	**8.79E-04**
0.61 (0.58, 0.64)	**3.99E-76**	0.92 (0.87, 0.97)	**3.48E-03**
0.56 (0.53, 0.59)	**8.52E-93**	0.89 (0.84, 0.94)	**1.29E-04**
0.55 (0.52, 0.59)	**1.12E-94**	0.89 (0.84, 0.94)	**5.32E-05**
0.58 (0.55, 0.61)	**6.49E-85**	0.91 (0.86, 0.97)	**2.49E-03**
0.95 (0.91, 0.99)	**1.78E-02**	1.03 (0.98, 1.07)	2.99E-01
0.64 (0.61, 0.67)	**3.70E-79**	0.87 (0.83, 0.91)	**7.52E-08**
0.67 (0.64, 0.70)	**2.40E-64**	0.89 (0.85, 0.93)	**2.94E-06**
0.71 (0.68, 0.74)	**1.90E-50**	0.91 (0.86, 0.95)	**5.74E-05**
0.62 (0.59, 0.65)	**1.01E-87**	0.86 (0.82, 0.91)	**1.72E-08**
0.62 (0.59, 0.65)	**2.31E-87**	0.86 (0.82, 0.91)	**1.10E-08**

0.62 (0.59, 0.65)	**2.48E-85**	0.87 (0.83, 0.92)	**2.47E-07**
1.14 (1.10, 1.18)	**4.04E-11**	1.04 (0.99, 1.08)	1.17E-01
0.75 (0.72, 0.79)	**5.21E-37**	0.85 (0.82, 0.89)	**4.63E-12**
0.82 (0.79, 0.86)	**9.42E-19**	0.89 (0.85, 0.93)	**3.09E-07**
0.83 (0.79, 0.87)	**3.19E-17**	0.90 (0.86, 0.94)	**3.70E-06**
0.73 (0.70, 0.76)	**1.31E-45**	0.85 (0.81, 0.89)	**2.05E-12**
0.74 (0.70, 0.77)	**2.37E-44**	0.84 (0.81, 0.88)	**6.79E-14**
0.75 (0.72, 0.79)	**5.87E-37**	0.90 (0.86, 0.94)	**2.17E-06**
1.31 (1.26, 1.35)	**1.38E-46**	1.07 (1.03, 1.12)	**2.49E-03**
1.33 (1.28, 1.40)	**7.00E-37**	1.15 (1.09, 1.21)	**7.99E-07**
0.82 (0.78, 0.86)	**3.29E-13**	0.96 (0.91, 1.01)	1.01E-01
0.81 (0.77, 0.86)	**2.87E-13**	0.94 (0.89, 0.99)	**3.59E-02**
0.87 (0.83, 0.92)	**9.87E-08**	0.99 (0.95, 1.04)	7.66E-01
1.07 (1.02, 1.12)	**3.23E-03**	1.00 (0.96, 1.05)	8.69E-01
0.60 (0.57, 0.64)	**7.02E-68**	0.90 (0.86, 0.96)	**6.26E-04**
0.59 (0.56, 0.63)	**2.91E-66**	0.90 (0.85, 0.95)	**3.48E-04**
0.73 (0.70, 0.76)	**5.00E-45**	0.95 (0.90, 1.00)	6.13E-02
1.49 (1.42, 1.56)	**3.41E-65**	1.10 (1.04, 1.15)	**8.03E-04**
1.49 (1.40, 1.58)	**2.46E-36**	1.12 (1.05, 1.19)	**7.16E-04**
0.80 (0.77, 0.83)	**2.91E-26**	0.99 (0.95, 1.04)	8.40E-01
0.70 (0.67, 0.73)	**2.37E-58**	0.96 (0.92, 1.00)	1.00E-01
1.16 (1.11, 1.21)	**6.30E-12**	1.10 (1.05, 1.15)	**1.93E-04**
1.01 (0.97, 1.05)	6.15E-01	0.96 (0.92, 1.01)	1.33E-01
0.68 (0.66, 0.70)	**1.58E-144**	0.90 (0.86, 0.93)	**1.26E-07**
0.61 (0.59, 0.63)	**1.16E-146**	0.88 (0.84, 0.91)	**1.65E-09**
0.60 (0.58, 0.62)	**3.79E-149**	0.87 (0.84, 0.91)	**5.83E-10**
0.64 (0.62, 0.67)	**2.36E-132**	0.89 (0.86, 0.93)	**7.64E-08**
1.63 (1.57, 1.69)	**1.78E-146**	1.14 (1.09, 1.18)	**2.67E-09**
0.58 (0.56, 0.60)	**7.99E-166**	0.83 (0.80, 0.87)	**8.70E-16**
0.65 (0.62, 0.67)	**1.68E-107**	0.88 (0.85, 0.92)	**5.60E-09**
0.74 (0.72, 0.77)	**2.08E-50**	0.93 (0.89, 0.97)	**6.26E-04**
0.57 (0.55, 0.59)	**1.64E-174**	0.83 (0.79, 0.86)	**4.97E-17**
1.60 (1.54, 1.67)	**1.65E-127**	1.15 (1.11, 1.20)	**5.20E-11**
1.80 (1.74, 1.87)	**4.96E-203**	1.31 (1.26, 1.37)	**5.35E-35**
0.58 (0.56, 0.60)	**1.23E-228**	0.82 (0.79, 0.85)	**2.21E-23**
0.57 (0.55, 0.59)	**6.99E-244**	0.81 (0.78, 0.84)	**1.01E-25**
0.75 (0.72, 0.77)	**4.44E-63**	0.92 (0.89, 0.95)	**4.12E-06**
1.64 (1.59, 1.69)	**7.20E-198**	1.18 (1.14, 1.23)	**3.03E-17**
1.40 (1.35, 1.45)	**5.42E-83**	1.20 (1.15, 1.25)	**4.97E-17**
0.61 (0.59, 0.63)	**3.75E-247**	0.80 (0.78, 0.83)	**1.06E-35**
0.59 (0.57, 0.60)	**1.29E-283**	0.78 (0.75, 0.80)	**6.59E-44**
0.77 (0.74, 0.80)	**7.50E-47**	0.93 (0.90, 0.97)	**2.45E-04**
1.55 (1.52, 1.59)	**2.40E-255**	1.21 (1.17, 1.25)	**4.92E-32**
1.02 (0.98, 1.06)	3.77E-01	0.99 (0.95, 1.03)	6.82E-01
0.87 (0.86, 0.88)	**2.97E-183**	0.91 (0.90, 0.93)	**1.59E-30**
0.89 (0.88, 0.91)	**4.27E-61**	0.93 (0.91, 0.94)	**1.51E-16**
0.71 (0.70, 0.73)	**2.62E-224**	0.86 (0.83, 0.89)	**1.36E-18**
1.21 (1.19, 1.22)	**1.58E-241**	1.13 (1.11, 1.15)	**1.05E-37**

1.06 (1.02, 1.10)	**6.40E-03**	1.06 (1.02, 1.10)	**4.02E-03**
0.87 (0.86, 0.88)	**5.08E-182**	0.91 (0.89, 0.92)	**3.60E-35**
0.94 (0.91, 0.98)	**3.25E-03**	0.91 (0.88, 0.94)	**7.25E-09**
0.70 (0.68, 0.73)	**1.47E-116**	0.89 (0.86, 0.92)	**1.43E-09**
1.18 (1.17, 1.19)	**1.23E-228**	1.12 (1.10, 1.14)	**3.91E-38**
1.13 (1.09, 1.18)	**3.67E-10**	1.13 (1.09, 1.17)	**6.79E-11**
0.82 (0.81, 0.83)	**2.04E-234**	0.87 (0.86, 0.89)	**1.37E-42**
0.85 (0.82, 0.89)	**1.32E-17**	0.88 (0.85, 0.91)	**7.23E-13**
0.75 (0.72, 0.77)	**9.91E-81**	0.90 (0.87, 0.94)	**8.62E-08**
1.20 (1.19, 1.21)	**2.58E-192**	1.12 (1.09, 1.14)	**6.92E-16**
0.84 (0.82, 0.87)	**2.93E-25**	1.02 (0.98, 1.06)	4.67E-01
1.06 (1.02, 1.10)	**3.99E-03**	0.96 (0.93, 1.00)	7.32E-02
0.78 (0.76, 0.81)	**5.72E-59**	0.93 (0.90, 0.96)	**8.43E-06**
1.27 (1.24, 1.30)	**6.70E-81**	1.04 (1.00, 1.08)	**4.75E-02**
1.16 (1.14, 1.18)	**1.16E-48**	1.01 (0.98, 1.05)	3.99E-01
1.46 (1.42, 1.50)	**3.78E-142**	1.10 (1.06, 1.14)	**1.92E-07**
0.70 (0.69, 0.72)	**2.26E-137**	0.91 (0.88, 0.95)	**3.90E-07**
0.70 (0.68, 0.72)	**2.36E-127**	0.91 (0.88, 0.94)	**6.56E-08**
0.77 (0.75, 0.79)	**3.15E-81**	0.97 (0.93, 1.00)	5.67E-02
1.18 (1.16, 1.19)	**1.54E-92**	1.06 (1.03, 1.09)	**2.17E-04**
1.57 (1.51, 1.62)	**5.48E-138**	1.17 (1.12, 1.22)	**2.34E-13**
0.70 (0.68, 0.72)	**2.57E-144**	0.87 (0.84, 0.91)	**6.59E-11**
0.73 (0.70, 0.75)	**2.46E-85**	0.89 (0.86, 0.93)	**1.88E-08**
0.62 (0.61, 0.64)	**2.00E-190**	0.85 (0.81, 0.89)	**6.37E-12**
1.16 (1.14, 1.17)	**4.22E-136**	1.10 (1.07, 1.14)	**3.12E-10**
1.01 (0.97, 1.05)	5.85E-01	1.05 (1.01, 1.10)	**3.54E-02**
0.74 (0.71, 0.76)	**5.25E-58**	0.88 (0.85, 0.92)	**1.78E-09**
0.79 (0.76, 0.82)	**5.85E-37**	0.88 (0.85, 0.92)	**7.47E-10**
0.75 (0.72, 0.79)	**1.40E-33**	1.01 (0.96, 1.06)	7.12E-01
1.37 (1.34, 1.40)	**2.00E-192**	1.16 (1.12, 1.21)	**2.06E-12**
1.29 (1.24, 1.35)	**6.62E-34**	0.98 (0.93, 1.02)	3.08E-01
0.75 (0.72, 0.78)	**2.35E-52**	0.96 (0.93, 1.01)	1.06E-01
0.64 (0.61, 0.67)	**5.03E-71**	0.94 (0.89, 0.99)	**3.59E-02**
0.58 (0.56, 0.61)	**1.45E-125**	0.84 (0.80, 0.88)	**6.89E-14**
0.88 (0.84, 0.92)	**3.80E-09**	0.93 (0.89, 0.98)	**3.01E-03**
0.66 (0.63, 0.69)	**6.89E-77**	0.85 (0.82, 0.89)	**5.38E-12**
0.69 (0.66, 0.72)	**1.82E-59**	0.88 (0.84, 0.92)	**3.68E-08**
0.57 (0.55, 0.60)	**1.29E-134**	0.83 (0.80, 0.87)	**8.63E-15**
0.62 (0.59, 0.64)	**1.61E-99**	0.86 (0.82, 0.89)	**2.37E-11**
1.21 (1.16, 1.25)	**6.41E-24**	1.03 (0.99, 1.07)	2.41E-01
1.20 (1.16, 1.25)	**2.15E-23**	1.01 (0.97, 1.06)	5.45E-01
1.20 (1.16, 1.25)	**1.97E-24**	1.09 (1.05, 1.13)	**1.54E-05**
1.16 (1.11, 1.20)	**6.60E-14**	1.05 (1.01, 1.09)	**3.61E-02**
0.68 (0.65, 0.71)	**2.93E-67**	0.89 (0.85, 0.93)	**1.91E-06**
1.45 (1.40, 1.50)	**8.79E-91**	1.09 (1.05, 1.14)	**3.90E-05**
0.63 (0.60, 0.66)	**4.82E-90**	0.86 (0.82, 0.90)	**1.95E-09**

0.62 (0.60, 0.65)	**1.89E-98**	0.90 (0.86, 0.94)	**6.44E-06**
0.63 (0.60, 0.66)	**8.17E-93**	0.87 (0.83, 0.91)	**9.55E-09**
0.62 (0.59, 0.65)	**1.80E-88**	0.86 (0.82, 0.91)	**1.90E-08**
0.73 (0.70, 0.77)	**4.14E-42**	0.90 (0.86, 0.94)	**1.43E-06**
1.00 (0.96, 1.04)	9.97E-01	0.98 (0.94, 1.02)	4.20E-01
0.93 (0.90, 0.97)	**1.90E-03**	0.97 (0.93, 1.01)	2.34E-01
0.63 (0.60, 0.66)	**1.42E-108**	0.83 (0.79, 0.86)	**1.91E-16**
0.66 (0.63, 0.70)	**1.29E-57**	0.83 (0.79, 0.87)	**5.52E-13**
0.64 (0.61, 0.67)	**3.11E-82**	0.85 (0.81, 0.89)	**4.63E-12**
0.81 (0.77, 0.85)	**3.56E-19**	0.87 (0.83, 0.92)	**6.14E-08**
0.66 (0.63, 0.69)	**3.72E-72**	0.86 (0.83, 0.90)	**5.88E-10**
0.67 (0.64, 0.70)	**3.24E-70**	0.85 (0.81, 0.89)	**1.36E-11**
1.17 (1.12, 1.21)	**1.22E-15**	1.05 (1.01, 1.09)	**1.33E-02**
1.00 (0.96, 1.04)	9.89E-01	1.00 (0.96, 1.04)	9.75E-01
0.72 (0.69, 0.75)	**6.51E-43**	0.86 (0.82, 0.90)	**1.93E-09**
0.59 (0.57, 0.61)	**2.09E-162**	0.81 (0.78, 0.85)	**2.26E-21**
0.82 (0.79, 0.86)	**1.64E-16**	0.88 (0.84, 0.92)	**1.14E-07**
0.68 (0.66, 0.71)	**1.90E-94**	0.88 (0.85, 0.91)	**4.14E-10**
0.65 (0.63, 0.68)	**3.07E-119**	0.85 (0.81, 0.88)	**5.26E-16**
1.65 (1.59, 1.71)	**5.86E-152**	1.23 (1.18, 1.28)	**5.28E-21**
1.20 (1.15, 1.25)	**5.17E-19**	1.07 (1.03, 1.11)	**1.87E-03**
1.26 (1.24, 1.28)	**6.74E-170**	1.18 (1.15, 1.20)	**3.67E-52**
1.16 (1.12, 1.21)	**1.11E-13**	1.07 (1.03, 1.12)	**1.26E-03**
1.11 (1.07, 1.15)	**5.74E-09**	1.12 (1.08, 1.15)	**2.10E-11**
1.20 (1.15, 1.24)	**1.64E-19**	1.08 (1.04, 1.13)	**1.20E-04**
0.95 (0.91, 0.99)	**2.73E-02**	0.92 (0.89, 0.96)	**3.24E-04**
0.86 (0.83, 0.90)	**4.81E-10**	0.88 (0.84, 0.93)	**5.87E-07**
1.18 (1.14, 1.23)	**9.39E-20**	1.06 (1.02, 1.10)	**9.34E-03**
1.18 (1.13, 1.22)	**9.08E-18**	1.03 (0.99, 1.07)	2.40E-01
1.21 (1.16, 1.25)	**2.26E-22**	1.05 (1.00, 1.09)	**4.06E-02**
1.05 (1.04, 1.06)	**8.04E-18**	1.05 (1.03, 1.08)	**2.05E-05**
1.15 (1.11, 1.20)	**3.52E-13**	1.08 (1.03, 1.12)	**6.25E-04**
1.07 (1.05, 1.09)	**3.07E-12**	1.05 (1.03, 1.07)	**7.17E-06**
1.10 (1.08, 1.12)	**2.87E-37**	1.07 (1.05, 1.09)	**5.77E-11**
1.09 (1.07, 1.11)	**4.74E-18**	1.08 (1.05, 1.11)	**8.27E-07**
1.13 (1.12, 1.13)	**1.03E-184**	1.07 (1.05, 1.08)	**5.96E-20**
0.74 (0.71, 0.77)	**5.14E-47**	0.84 (0.81, 0.88)	**1.54E-14**
1.45 (1.40, 1.51)	**4.46E-88**	1.25 (1.20, 1.31)	**1.36E-26**
0.66 (0.63, 0.69)	**6.66E-62**	0.89 (0.84, 0.94)	**4.45E-05**
1.20 (1.16, 1.24)	**6.83E-22**	1.05 (1.00, 1.09)	**4.75E-02**
0.67 (0.64, 0.70)	**1.29E-70**	0.86 (0.83, 0.90)	**2.49E-10**
0.63 (0.61, 0.66)	**8.17E-90**	0.85 (0.81, 0.89)	**1.04E-12**
0.68 (0.65, 0.71)	**1.26E-64**	0.86 (0.82, 0.90)	**5.42E-11**
0.55 (0.52, 0.58)	**1.79E-116**	0.84 (0.80, 0.89)	**5.06E-10**
0.59 (0.56, 0.61)	**3.87E-133**	0.81 (0.77, 0.84)	**2.45E-19**
1.05 (1.01, 1.08)	**7.77E-03**	1.05 (1.01, 1.08)	**1.20E-02**
0.97 (0.93, 1.02)	2.33E-01	0.96 (0.92, 1.00)	5.68E-02
0.61 (0.58, 0.64)	**5.52E-108**	0.84 (0.80, 0.88)	**5.98E-14**

0.58 (0.55, 0.61)	**1.23E-98**	0.88 (0.83, 0.92)	**3.11E-06**
1.05 (1.03, 1.08)	**1.65E-05**	1.06 (1.03, 1.08)	**2.62E-05**
1.08 (1.06, 1.09)	**4.90E-23**	1.07 (1.05, 1.10)	**1.05E-07**
0.60 (0.57, 0.63)	**1.61E-111**	0.83 (0.79, 0.87)	**1.07E-13**
0.97 (0.93, 1.01)	1.93E-01	0.97 (0.93, 1.01)	2.22E-01
0.73 (0.70, 0.77)	**4.27E-42**	0.89 (0.85, 0.93)	**7.10E-07**
0.61 (0.58, 0.64)	**1.92E-99**	0.84 (0.80, 0.88)	**2.93E-12**
0.64 (0.61, 0.67)	**3.03E-86**	0.87 (0.84, 0.91)	**1.17E-08**
1.04 (1.00, 1.08)	6.22E-02	0.98 (0.94, 1.02)	3.72E-01
0.68 (0.65, 0.71)	**1.26E-64**	0.87 (0.83, 0.91)	**4.38E-10**
0.64 (0.61, 0.67)	**1.56E-74**	0.89 (0.85, 0.94)	**1.52E-05**
0.70 (0.67, 0.74)	**2.71E-54**	0.89 (0.85, 0.93)	**1.25E-07**
1.09 (1.05, 1.14)	**1.40E-05**	0.99 (0.94, 1.03)	5.40E-01
0.68 (0.65, 0.71)	**1.49E-63**	0.87 (0.83, 0.91)	**5.66E-10**
0.61 (0.58, 0.64)	**8.24E-90**	0.87 (0.83, 0.92)	**7.07E-07**
0.81 (0.78, 0.85)	**1.89E-20**	0.92 (0.88, 0.96)	**2.38E-04**
0.55 (0.52, 0.58)	**5.11E-114**	0.84 (0.80, 0.89)	**2.74E-09**
1.04 (1.00, 1.09)	7.03E-02	1.00 (0.94, 1.05)	8.69E-01

sting using a false discovery rate (FDR) adjustme

Cerebrovascular di			
CKB			
**Unadjusted HR (95% CI)**	**P value**	**Adjusted HR (95% CI)**	**P value**
0.92 (0.84, 1.01)	1.24E-01	0.90 (0.82, 0.99)	7.59E-02
0.92 (0.84, 1.01)	1.38E-01	0.90 (0.82, 0.99)	8.49E-02
0.94 (0.85, 1.03)	2.29E-01	0.92 (0.84, 1.00)	1.44E-01
0.95 (0.87, 1.04)	3.21E-01	0.93 (0.85, 1.02)	2.58E-01
0.94 (0.86, 1.03)	2.72E-01	0.94 (0.86, 1.03)	3.10E-01
0.95 (0.87, 1.04)	3.51E-01	0.92 (0.84, 1.01)	1.91E-01
0.91 (0.83, 1.00)	9.99E-02	0.90 (0.82, 0.98)	5.60E-02
0.95 (0.87, 1.04)	3.24E-01	0.93 (0.84, 1.01)	1.99E-01
0.95 (0.87, 1.04)	3.24E-01	0.93 (0.85, 1.02)	2.04E-01
0.94 (0.86, 1.03)	2.67E-01	0.92 (0.84, 1.01)	1.85E-01
0.95 (0.87, 1.04)	3.76E-01	0.94 (0.86, 1.03)	3.02E-01
0.96 (0.88, 1.05)	4.39E-01	0.95 (0.87, 1.04)	3.48E-01
0.95 (0.86, 1.04)	3.14E-01	0.93 (0.85, 1.02)	2.29E-01
0.95 (0.87, 1.04)	3.24E-01	0.92 (0.84, 1.01)	1.85E-01
0.96 (0.88, 1.05)	4.47E-01	0.94 (0.86, 1.03)	3.02E-01
0.96 (0.88, 1.05)	4.39E-01	0.94 (0.86, 1.03)	3.02E-01
0.96 (0.88, 1.05)	4.30E-01	0.94 (0.86, 1.03)	3.02E-01
0.95 (0.87, 1.03)	3.10E-01	0.93 (0.85, 1.02)	2.56E-01
0.93 (0.85, 1.01)	1.48E-01	0.92 (0.84, 1.01)	1.83E-01
0.97 (0.89, 1.06)	5.20E-01	0.95 (0.86, 1.03)	3.40E-01
0.97 (0.88, 1.06)	5.01E-01	0.94 (0.86, 1.03)	3.23E-01
0.95 (0.87, 1.04)	3.51E-01	0.95 (0.87, 1.04)	3.53E-01
0.95 (0.87, 1.04)	3.43E-01	0.95 (0.87, 1.04)	3.53E-01
0.95 (0.86, 1.03)	2.98E-01	0.94 (0.86, 1.03)	3.24E-01
0.93 (0.85, 1.02)	1.99E-01	0.95 (0.86, 1.03)	3.40E-01
0.91 (0.83, 0.99)	6.35E-02	0.93 (0.85, 1.02)	2.56E-01
0.97 (0.88, 1.06)	5.02E-01	0.96 (0.88, 1.05)	4.76E-01
0.96 (0.88, 1.05)	4.48E-01	0.95 (0.87, 1.04)	3.83E-01
0.94 (0.86, 1.03)	2.67E-01	0.96 (0.88, 1.05)	5.05E-01
0.95 (0.87, 1.03)	3.11E-01	0.97 (0.89, 1.06)	6.27E-01
0.93 (0.85, 1.02)	1.85E-01	0.95 (0.87, 1.04)	3.84E-01
0.99 (0.91, 1.08)	8.17E-01	1.05 (0.96, 1.15)	3.84E-01
0.99 (0.91, 1.08)	8.57E-01	1.07 (0.98, 1.17)	2.38E-01
0.98 (0.90, 1.07)	7.68E-01	1.01 (0.92, 1.10)	8.72E-01
0.93 (0.85, 1.02)	1.99E-01	0.93 (0.85, 1.02)	2.63E-01
1.00 (0.92, 1.09)	9.95E-01	1.05 (0.96, 1.15)	3.84E-01
1.01 (0.93, 1.10)	8.56E-01	1.06 (0.97, 1.16)	2.90E-01
0.93 (0.85, 1.02)	1.89E-01	0.99 (0.91, 1.09)	8.83E-01
1.07 (0.99, 1.17)	1.60E-01	1.14 (1.04, 1.24)	**1.01E-02**
1.08 (0.99, 1.18)	1.23E-01	1.14 (1.05, 1.25)	**6.35E-03**
1.05 (0.97, 1.14)	3.24E-01	1.11 (1.02, 1.21)	**4.35E-02**

0.98 (0.90, 1.07)	6.92E-01	0.98 (0.89, 1.07)	7.19E-01
0.93 (0.85, 1.01)	1.48E-01	0.98 (0.89, 1.07)	7.26E-01
0.93 (0.85, 1.01)	1.41E-01	0.98 (0.90, 1.07)	7.35E-01
0.90 (0.83, 0.98)	**4.67E-02**	0.96 (0.88, 1.05)	5.05E-01
0.92 (0.84, 1.00)	1.09E-01	0.98 (0.89, 1.07)	7.15E-01
0.91 (0.83, 0.99)	7.02E-02	0.96 (0.88, 1.05)	5.32E-01
0.95 (0.87, 1.03)	3.07E-01	1.01 (0.93, 1.11)	8.37E-01
1.05 (0.96, 1.14)	3.51E-01	1.04 (0.95, 1.13)	5.33E-01
0.91 (0.83, 0.99)	6.29E-02	0.96 (0.87, 1.05)	4.92E-01
0.90 (0.83, 0.99)	5.64E-02	0.96 (0.87, 1.05)	4.90E-01
0.87 (0.80, 0.95)	**7.93E-03**	0.92 (0.84, 1.01)	1.84E-01
0.90 (0.82, 0.98)	**4.35E-02**	0.96 (0.87, 1.05)	4.86E-01
0.89 (0.82, 0.98)	**3.32E-02**	0.95 (0.87, 1.04)	4.10E-01
0.92 (0.84, 1.00)	1.03E-01	0.98 (0.89, 1.07)	7.19E-01
1.08 (1.00, 1.17)	1.03E-01	1.07 (0.98, 1.16)	2.29E-01
0.89 (0.82, 0.97)	**2.60E-02**	0.94 (0.86, 1.04)	3.40E-01
0.89 (0.81, 0.97)	**2.30E-02**	0.94 (0.86, 1.03)	3.35E-01
0.85 (0.78, 0.92)	**4.48E-04**	0.88 (0.81, 0.96)	**1.61E-02**
0.90 (0.82, 0.98)	**3.32E-02**	0.96 (0.87, 1.05)	4.42E-01
0.90 (0.82, 0.98)	**3.85E-02**	0.96 (0.88, 1.05)	5.05E-01
0.89 (0.81, 0.97)	**1.73E-02**	0.93 (0.85, 1.02)	2.38E-01
1.06 (0.98, 1.15)	2.36E-01	1.06 (0.97, 1.15)	3.24E-01
0.90 (0.82, 0.98)	**3.84E-02**	0.95 (0.87, 1.04)	3.83E-01
0.90 (0.82, 0.98)	**3.76E-02**	0.95 (0.87, 1.04)	3.83E-01
0.86 (0.79, 0.93)	**1.16E-03**	0.89 (0.82, 0.97)	**1.78E-02**
0.91 (0.83, 0.99)	6.88E-02	0.97 (0.89, 1.06)	6.27E-01
0.91 (0.84, 1.00)	7.52E-02	0.98 (0.89, 1.07)	7.09E-01
0.91 (0.83, 0.99)	**4.90E-02**	0.94 (0.86, 1.03)	3.32E-01
1.01 (0.93, 1.10)	8.24E-01	1.01 (0.93, 1.11)	8.37E-01
1.25 (1.16, 1.36)	**3.34E-07**	1.23 (1.14, 1.34)	**3.19E-06**
1.25 (1.16, 1.36)	**3.34E-07**	1.23 (1.14, 1.34)	**3.43E-06**
1.21 (1.12, 1.31)	**2.78E-05**	1.20 (1.11, 1.30)	**9.22E-05**
1.28 (1.18, 1.39)	**6.35E-08**	1.25 (1.16, 1.36)	**1.25E-06**
1.30 (1.20, 1.42)	**8.81E-09**	1.27 (1.17, 1.38)	**1.98E-07**
1.23 (1.13, 1.33)	**5.08E-06**	1.21 (1.11, 1.31)	**5.96E-05**
1.20 (1.11, 1.29)	**2.78E-05**	1.17 (1.08, 1.27)	**4.20E-04**
1.19 (1.09, 1.29)	**2.02E-04**	1.15 (1.06, 1.25)	**2.69E-03**
1.19 (1.09, 1.29)	**1.90E-04**	1.16 (1.06, 1.26)	**2.55E-03**
1.17 (1.08, 1.27)	**6.24E-04**	1.14 (1.05, 1.24)	**6.00E-03**
1.19 (1.10, 1.29)	**1.35E-04**	1.16 (1.07, 1.26)	**2.33E-03**
1.19 (1.09, 1.29)	**1.89E-04**	1.15 (1.06, 1.25)	**3.29E-03**
1.20 (1.11, 1.31)	**4.96E-05**	1.18 (1.08, 1.28)	**7.30E-04**
1.17 (1.08, 1.27)	**3.77E-04**	1.17 (1.07, 1.27)	**1.48E-03**
0.88 (0.80, 0.96)	**1.21E-02**	0.85 (0.78, 0.93)	**1.79E-03**
0.88 (0.81, 0.96)	**1.62E-02**	0.85 (0.78, 0.93)	**2.33E-03**
0.93 (0.85, 1.02)	1.99E-01	0.90 (0.82, 0.98)	**4.60E-02**
0.87 (0.80, 0.95)	**6.86E-03**	0.85 (0.78, 0.93)	**1.48E-03**
0.86 (0.79, 0.94)	**2.03E-03**	0.84 (0.77, 0.91)	**4.32E-04**

0.93 (0.85, 1.01)	1.48E-01	0.90 (0.82, 0.98)	5.53E-02
0.82 (0.75, 0.90)	**1.09E-04**	0.81 (0.74, 0.90)	**1.63E-04**
0.81 (0.75, 0.87)	**1.61E-07**	0.79 (0.73, 0.85)	**2.03E-08**
0.81 (0.75, 0.87)	**1.61E-07**	0.79 (0.73, 0.85)	**2.30E-08**
0.86 (0.79, 0.94)	**3.72E-03**	0.82 (0.75, 0.89)	**5.97E-05**
0.83 (0.77, 0.90)	**4.83E-05**	0.86 (0.79, 0.94)	**2.55E-03**
0.85 (0.78, 0.92)	**2.51E-04**	0.89 (0.82, 0.97)	**1.95E-02**
0.90 (0.83, 0.98)	**4.19E-02**	0.85 (0.78, 0.93)	**1.46E-03**
0.91 (0.83, 0.99)	7.28E-02	0.88 (0.81, 0.97)	**2.56E-02**
1.05 (0.96, 1.15)	3.70E-01	1.02 (0.93, 1.12)	7.59E-01
1.18 (1.11, 1.25)	**5.05E-07**	1.17 (1.10, 1.24)	**3.04E-06**
1.17 (1.09, 1.25)	**1.80E-05**	1.17 (1.10, 1.24)	**3.07E-06**
1.13 (1.05, 1.21)	**2.03E-03**	1.08 (1.00, 1.17)	1.21E-01
0.83 (0.77, 0.89)	**1.36E-06**	0.84 (0.79, 0.91)	**2.34E-05**
0.90 (0.83, 0.97)	**2.13E-02**	0.91 (0.84, 0.99)	6.07E-02
1.11 (1.03, 1.19)	**1.76E-02**	1.13 (1.06, 1.21)	**1.10E-03**
1.15 (1.07, 1.23)	**3.47E-04**	1.19 (1.11, 1.27)	**1.01E-05**
0.95 (0.87, 1.04)	3.51E-01	0.96 (0.88, 1.05)	5.34E-01
0.99 (0.90, 1.08)	7.87E-01	0.95 (0.86, 1.04)	3.51E-01
0.98 (0.90, 1.07)	7.50E-01	0.98 (0.90, 1.07)	7.32E-01
0.92 (0.85, 1.00)	8.84E-02	0.96 (0.88, 1.04)	4.12E-01
0.86 (0.78, 0.94)	**2.03E-03**	0.93 (0.85, 1.02)	2.29E-01
1.04 (0.95, 1.13)	4.92E-01	1.01 (0.92, 1.10)	8.86E-01
1.07 (0.99, 1.17)	1.48E-01	1.04 (0.96, 1.14)	4.59E-01
0.86 (0.78, 0.94)	**3.53E-03**	0.88 (0.81, 0.97)	**2.26E-02**
0.93 (0.85, 1.01)	1.38E-01	1.00 (0.91, 1.09)	9.80E-01
0.89 (0.81, 0.97)	**2.21E-02**	0.97 (0.89, 1.07)	6.38E-01
1.13 (1.03, 1.24)	**2.41E-02**	1.08 (0.99, 1.19)	1.87E-01
1.10 (1.01, 1.20)	6.88E-02	1.02 (0.93, 1.12)	7.19E-01
0.93 (0.85, 1.02)	1.70E-01	0.89 (0.82, 0.98)	**4.03E-02**
1.08 (0.99, 1.18)	1.24E-01	1.17 (1.07, 1.28)	**1.88E-03**
1.05 (0.96, 1.15)	3.35E-01	1.15 (1.05, 1.25)	**8.78E-03**
1.27 (1.17, 1.38)	**1.66E-07**	1.25 (1.15, 1.36)	**1.29E-06**
0.94 (0.86, 1.03)	2.38E-01	0.88 (0.80, 0.96)	**1.31E-02**
0.83 (0.77, 0.89)	**4.09E-06**	0.87 (0.81, 0.93)	**8.44E-04**
1.22 (1.11, 1.34)	**1.75E-04**	1.28 (1.16, 1.41)	**6.88E-06**
1.20 (1.10, 1.31)	**3.77E-04**	1.24 (1.13, 1.36)	**3.97E-05**
1.14 (1.04, 1.25)	**1.88E-02**	1.17 (1.07, 1.29)	**3.29E-03**
0.97 (0.88, 1.05)	4.90E-01	0.91 (0.83, 1.00)	1.21E-01
0.89 (0.82, 0.97)	**1.74E-02**	0.91 (0.84, 0.99)	9.06E-02
0.93 (0.86, 1.01)	1.38E-01	0.99 (0.91, 1.07)	7.84E-01
0.89 (0.82, 0.97)	**1.88E-02**	0.93 (0.86, 1.01)	1.52E-01
1.02 (0.93, 1.11)	7.13E-01	1.07 (0.98, 1.17)	2.56E-01
1.11 (1.02, 1.20)	**2.46E-02**	1.04 (0.96, 1.13)	4.08E-01
0.98 (0.90, 1.07)	6.92E-01	0.93 (0.85, 1.02)	2.29E-01
0.92 (0.86, 1.00)	7.91E-02	0.98 (0.91, 1.07)	7.59E-01
0.90 (0.83, 0.97)	**1.66E-02**	0.96 (0.88, 1.04)	3.91E-01
1.08 (0.99, 1.18)	1.58E-01	1.09 (0.99, 1.19)	1.48E-01

1.14 (1.06, 1.22)	**8.91E-04**	1.08 (1.00, 1.16)	1.21E-01
0.99 (0.91, 1.08)	8.53E-01	0.94 (0.85, 1.02)	2.69E-01
0.95 (0.87, 1.03)	2.78E-01	1.01 (0.92, 1.10)	9.14E-01
0.94 (0.86, 1.02)	1.69E-01	1.00 (0.91, 1.08)	9.33E-01
1.10 (1.01, 1.19)	5.76E-02	1.03 (0.95, 1.13)	5.57E-01
1.16 (1.08, 1.25)	**2.81E-04**	1.10 (1.02, 1.19)	**3.82E-02**
1.00 (0.92, 1.09)	9.87E-01	0.94 (0.85, 1.03)	2.77E-01
0.97 (0.89, 1.05)	4.78E-01	1.03 (0.94, 1.12)	6.71E-01
0.95 (0.88, 1.03)	3.21E-01	1.01 (0.93, 1.11)	7.96E-01
1.11 (1.02, 1.20)	**3.76E-02**	1.03 (0.95, 1.13)	5.57E-01
1.10 (1.02, 1.19)	**4.24E-02**	1.05 (0.97, 1.14)	3.40E-01
0.92 (0.84, 1.00)	7.94E-02	0.92 (0.84, 1.01)	1.52E-01
1.13 (1.03, 1.23)	**1.73E-02**	1.13 (1.03, 1.24)	**2.45E-02**
1.13 (1.04, 1.23)	**1.28E-02**	1.14 (1.04, 1.25)	**1.31E-02**
1.05 (0.96, 1.14)	3.98E-01	1.02 (0.93, 1.12)	7.19E-01
0.95 (0.87, 1.04)	3.51E-01	0.94 (0.86, 1.03)	3.35E-01
0.84 (0.77, 0.92)	**4.72E-04**	0.87 (0.79, 0.95)	**8.27E-03**
1.19 (1.08, 1.31)	**1.12E-03**	1.15 (1.04, 1.26)	**1.78E-02**
1.12 (1.02, 1.23)	**3.18E-02**	1.06 (0.97, 1.16)	3.40E-01
1.26 (1.14, 1.39)	**5.14E-05**	1.25 (1.13, 1.38)	**1.52E-04**
0.99 (0.90, 1.08)	7.88E-01	1.00 (0.91, 1.09)	9.60E-01
1.27 (1.20, 1.33)	**7.81E-19**	1.23 (1.17, 1.30)	**7.96E-14**
0.87 (0.81, 0.94)	**8.24E-04**	0.87 (0.81, 0.94)	**1.81E-03**
0.86 (0.80, 0.92)	**1.33E-04**	0.87 (0.81, 0.93)	**4.20E-04**
0.99 (0.90, 1.07)	7.88E-01	0.97 (0.89, 1.06)	6.63E-01
0.92 (0.84, 1.00)	1.08E-01	0.94 (0.85, 1.03)	3.00E-01
1.05 (0.96, 1.14)	3.51E-01	0.98 (0.89, 1.06)	6.71E-01
0.96 (0.88, 1.05)	4.24E-01	1.03 (0.94, 1.12)	6.55E-01
0.95 (0.87, 1.03)	2.94E-01	1.02 (0.93, 1.11)	7.75E-01
1.16 (1.07, 1.27)	**1.23E-03**	1.08 (0.99, 1.18)	1.98E-01
1.00 (0.92, 1.09)	9.49E-01	0.98 (0.90, 1.07)	7.70E-01
0.98 (0.90, 1.07)	6.69E-01	0.94 (0.86, 1.03)	3.02E-01
1.08 (0.99, 1.17)	1.40E-01	1.05 (0.96, 1.15)	3.82E-01
1.27 (1.17, 1.38)	**2.87E-07**	1.26 (1.15, 1.37)	**2.56E-06**
0.95 (0.87, 1.04)	3.70E-01	1.01 (0.92, 1.10)	8.83E-01
0.98 (0.90, 1.07)	6.92E-01	1.01 (0.92, 1.10)	8.57E-01
0.95 (0.87, 1.04)	3.24E-01	1.00 (0.91, 1.09)	9.74E-01
0.90 (0.83, 0.99)	**4.67E-02**	0.97 (0.88, 1.06)	5.57E-01
1.10 (1.01, 1.19)	6.07E-02	1.08 (0.99, 1.17)	1.84E-01
1.08 (0.99, 1.17)	1.48E-01	1.06 (0.97, 1.15)	3.40E-01
1.28 (1.18, 1.39)	**1.66E-07**	1.26 (1.16, 1.37)	**1.96E-06**
0.82 (0.75, 0.90)	**1.12E-04**	0.85 (0.77, 0.93)	**2.92E-03**
0.80 (0.73, 0.88)	**2.47E-05**	0.86 (0.78, 0.94)	**6.22E-03**
0.97 (0.89, 1.06)	6.26E-01	0.96 (0.88, 1.05)	5.01E-01
0.96 (0.88, 1.05)	4.56E-01	0.95 (0.87, 1.04)	3.83E-01
1.08 (0.99, 1.17)	1.34E-01	1.07 (0.98, 1.16)	2.45E-01
0.99 (0.91, 1.08)	9.19E-01	0.98 (0.89, 1.07)	7.19E-01
0.90 (0.83, 0.99)	5.28E-02	0.94 (0.86, 1.02)	2.68E-01

0.98 (0.89, 1.06)	6.32E-01	0.97 (0.89, 1.06)	6.27E-01
0.79 (0.73, 0.87)	**2.54E-06**	0.81 (0.74, 0.89)	**5.36E-05**
0.75 (0.69, 0.82)	**4.17E-09**	0.79 (0.72, 0.86)	**3.04E-06**
0.87 (0.79, 0.95)	**4.64E-03**	0.90 (0.83, 0.99)	7.06E-02
1.09 (1.00, 1.18)	1.03E-01	1.07 (0.99, 1.17)	2.02E-01
0.91 (0.83, 1.00)	7.52E-02	0.95 (0.87, 1.04)	4.04E-01
0.88 (0.81, 0.97)	**1.72E-02**	0.92 (0.84, 1.01)	1.84E-01
0.83 (0.76, 0.91)	**4.72E-04**	0.87 (0.79, 0.95)	**9.31E-03**
0.70 (0.66, 0.75)	**2.92E-25**	0.75 (0.70, 0.81)	**7.96E-14**
1.13 (1.04, 1.23)	**1.09E-02**	1.21 (1.11, 1.31)	**5.97E-05**
0.78 (0.71, 0.85)	**3.60E-07**	0.85 (0.77, 0.93)	**1.82E-03**
0.96 (0.88, 1.05)	3.98E-01	1.03 (0.94, 1.13)	5.98E-01
0.76 (0.69, 0.83)	**7.19E-08**	0.81 (0.73, 0.89)	**1.19E-04**
0.78 (0.71, 0.85)	**1.32E-06**	0.84 (0.76, 0.92)	**1.72E-03**
0.90 (0.82, 0.99)	5.13E-02	0.91 (0.83, 1.00)	1.24E-01
0.84 (0.77, 0.92)	**1.01E-03**	0.86 (0.78, 0.94)	**6.00E-03**
1.39 (1.27, 1.51)	**8.62E-12**	1.45 (1.33, 1.58)	**1.37E-14**
0.82 (0.77, 0.88)	**5.41E-07**	0.90 (0.83, 0.97)	**1.93E-02**
1.14 (1.05, 1.24)	**8.17E-03**	1.23 (1.13, 1.33)	**2.28E-05**
0.79 (0.73, 0.86)	**3.34E-07**	0.86 (0.79, 0.94)	**2.33E-03**
0.82 (0.75, 0.89)	**1.56E-05**	0.91 (0.83, 0.99)	7.60E-02
1.18 (1.08, 1.28)	**5.86E-04**	1.10 (1.01, 1.20)	7.50E-02
1.13 (1.04, 1.23)	**1.43E-02**	1.03 (0.94, 1.12)	6.60E-01
1.22 (1.15, 1.29)	**2.37E-10**	1.20 (1.13, 1.28)	**2.22E-08**
1.06 (0.98, 1.16)	1.97E-01	1.05 (0.97, 1.14)	3.48E-01
0.84 (0.77, 0.92)	**6.94E-04**	0.84 (0.76, 0.92)	**1.53E-03**
0.97 (0.89, 1.05)	5.01E-01	0.98 (0.90, 1.07)	7.35E-01
1.09 (1.00, 1.19)	1.03E-01	1.02 (0.94, 1.11)	7.19E-01
1.16 (1.07, 1.26)	**1.23E-03**	1.14 (1.05, 1.24)	**8.83E-03**
0.92 (0.84, 1.01)	1.12E-01	0.92 (0.84, 1.01)	1.49E-01
0.99 (0.91, 1.08)	8.57E-01	0.97 (0.89, 1.06)	6.30E-01
0.77 (0.70, 0.84)	**3.34E-07**	0.77 (0.70, 0.85)	**2.09E-06**
1.18 (1.13, 1.23)	**7.17E-12**	1.16 (1.11, 1.21)	**1.63E-09**
1.12 (1.03, 1.22)	**1.87E-02**	1.12 (1.03, 1.22)	**2.24E-02**
1.18 (1.13, 1.23)	**1.08E-11**	1.13 (1.08, 1.19)	**1.64E-05**
1.02 (0.94, 1.12)	6.48E-01	1.00 (0.92, 1.10)	9.48E-01
0.98 (0.89, 1.09)	7.90E-01	1.01 (0.92, 1.11)	8.72E-01
1.07 (1.03, 1.10)	**2.81E-04**	1.07 (1.03, 1.11)	**1.88E-03**
0.76 (0.71, 0.82)	**1.27E-12**	0.78 (0.73, 0.84)	**2.29E-10**
1.15 (1.07, 1.25)	**1.56E-03**	1.20 (1.10, 1.30)	**1.02E-04**

seases mortality

UKB			
**Unadjusted HR (95% CI)**	**P value**	**Adjusted HR (95% CI)**	**P value**
1.05 (0.98, 1.11)	2.07E-01	0.93 (0.87, 1.00)	7.37E-02
1.04 (0.97, 1.10)	3.34E-01	0.93 (0.87, 1.00)	6.62E-02
1.05 (0.99, 1.12)	1.43E-01	0.94 (0.87, 1.00)	1.01E-01
1.03 (0.97, 1.10)	3.71E-01	0.93 (0.87, 1.00)	9.05E-02
1.02 (0.96, 1.09)	5.56E-01	0.93 (0.87, 1.00)	8.76E-02
1.05 (0.98, 1.12)	1.98E-01	0.94 (0.87, 1.00)	9.87E-02
1.03 (0.97, 1.10)	3.85E-01	0.93 (0.86, 0.99)	5.66E-02
1.01 (0.95, 1.08)	8.12E-01	0.90 (0.84, 0.97)	**8.11E-03**
1.00 (0.94, 1.07)	9.37E-01	0.90 (0.84, 0.96)	**6.72E-03**
1.00 (0.93, 1.07)	9.67E-01	0.90 (0.84, 0.97)	**8.94E-03**
0.95 (0.89, 1.02)	1.63E-01	0.89 (0.83, 0.95)	**3.26E-03**
0.91 (0.85, 0.98)	**1.35E-02**	0.88 (0.82, 0.95)	**1.71E-03**
0.99 (0.93, 1.06)	7.52E-01	0.90 (0.84, 0.97)	**8.45E-03**
1.02 (0.96, 1.09)	5.40E-01	0.90 (0.84, 0.97)	**9.74E-03**
0.99 (0.92, 1.05)	7.48E-01	0.89 (0.83, 0.96)	**4.24E-03**
0.97 (0.91, 1.04)	4.89E-01	0.88 (0.82, 0.95)	**2.36E-03**
0.99 (0.93, 1.06)	8.80E-01	0.90 (0.84, 0.96)	**6.35E-03**
0.95 (0.89, 1.02)	1.98E-01	0.89 (0.83, 0.96)	**3.35E-03**
0.93 (0.87, 0.99)	**4.57E-02**	0.89 (0.83, 0.96)	**4.67E-03**
0.98 (0.91, 1.04)	5.39E-01	0.89 (0.83, 0.95)	**3.16E-03**
0.98 (0.91, 1.04)	5.40E-01	0.88 (0.82, 0.95)	**1.80E-03**
0.84 (0.79, 0.90)	**3.22E-06**	0.87 (0.81, 0.93)	**4.51E-04**
0.87 (0.81, 0.93)	**1.34E-04**	0.87 (0.81, 0.93)	**3.71E-04**
0.84 (0.78, 0.90)	**1.06E-06**	0.87 (0.82, 0.94)	**5.43E-04**
0.77 (0.72, 0.83)	**3.55E-12**	0.89 (0.83, 0.95)	**1.82E-03**
0.75 (0.70, 0.81)	**1.20E-14**	0.90 (0.84, 0.96)	**5.62E-03**
0.82 (0.76, 0.88)	**5.05E-08**	0.88 (0.82, 0.94)	**7.36E-04**
0.97 (0.90, 1.03)	3.58E-01	0.88 (0.82, 0.95)	**1.82E-03**
0.98 (0.92, 1.05)	6.16E-01	0.92 (0.86, 0.98)	**2.63E-02**
0.96 (0.90, 1.03)	2.81E-01	0.91 (0.85, 0.97)	**1.30E-02**
0.88 (0.82, 0.94)	**5.32E-04**	0.89 (0.83, 0.96)	**3.16E-03**
0.88 (0.82, 0.94)	**3.34E-04**	0.90 (0.84, 0.97)	**7.04E-03**
0.91 (0.85, 0.97)	**7.73E-03**	0.91 (0.85, 0.98)	**1.55E-02**
0.83 (0.77, 0.89)	**2.70E-07**	0.89 (0.83, 0.95)	**1.88E-03**
1.08 (1.01, 1.15)	**2.99E-02**	0.94 (0.88, 1.01)	1.23E-01
0.93 (0.87, 1.00)	**4.97E-02**	0.95 (0.89, 1.02)	2.00E-01
0.95 (0.88, 1.01)	1.36E-01	0.96 (0.90, 1.03)	3.02E-01
0.98 (0.92, 1.05)	6.67E-01	0.98 (0.91, 1.04)	5.46E-01
0.85 (0.80, 0.91)	**1.21E-05**	0.94 (0.88, 1.01)	1.29E-01
0.83 (0.77, 0.88)	**1.23E-07**	0.94 (0.88, 1.00)	9.26E-02
0.93 (0.87, 0.99)	**4.45E-02**	0.96 (0.90, 1.03)	2.99E-01

1.14 (1.07, 1.21)	**4.84E-05**	1.00 (0.93, 1.06)	9.43E-01
0.76 (0.71, 0.81)	**2.70E-14**	0.88 (0.82, 0.94)	**5.46E-04**
0.76 (0.70, 0.81)	**9.10E-15**	0.88 (0.82, 0.95)	**1.44E-03**
0.78 (0.73, 0.83)	**3.43E-12**	0.90 (0.84, 0.96)	**6.72E-03**
0.73 (0.68, 0.78)	**2.29E-18**	0.87 (0.82, 0.94)	**5.17E-04**
0.72 (0.67, 0.77)	**1.46E-19**	0.87 (0.81, 0.93)	**2.67E-04**
0.76 (0.71, 0.81)	**2.18E-14**	0.90 (0.84, 0.96)	**4.24E-03**
1.12 (1.05, 1.19)	**6.51E-04**	1.00 (0.93, 1.07)	9.82E-01
0.78 (0.72, 0.83)	**7.03E-12**	0.88 (0.82, 0.94)	**1.01E-03**
0.74 (0.69, 0.79)	**6.38E-17**	0.85 (0.79, 0.91)	**2.84E-05**
0.73 (0.68, 0.78)	**2.06E-18**	0.85 (0.79, 0.91)	**2.41E-05**
0.72 (0.68, 0.78)	**1.01E-18**	0.85 (0.79, 0.91)	**1.85E-05**
0.73 (0.68, 0.78)	**1.27E-17**	0.84 (0.79, 0.90)	**1.42E-05**
0.71 (0.66, 0.76)	**6.95E-21**	0.86 (0.80, 0.92)	**6.68E-05**
1.09 (1.03, 1.16)	**9.35E-03**	0.98 (0.91, 1.04)	5.63E-01
0.79 (0.74, 0.85)	**1.85E-10**	0.86 (0.80, 0.92)	**6.30E-05**
0.76 (0.71, 0.82)	**1.14E-13**	0.83 (0.78, 0.89)	**3.15E-06**
0.75 (0.70, 0.81)	**8.28E-15**	0.83 (0.77, 0.89)	**2.91E-06**
0.75 (0.70, 0.80)	**3.47E-15**	0.83 (0.77, 0.89)	**2.91E-06**
0.77 (0.72, 0.82)	**6.92E-13**	0.83 (0.77, 0.89)	**2.91E-06**
0.72 (0.67, 0.77)	**5.70E-20**	0.84 (0.78, 0.90)	**5.53E-06**
1.07 (1.01, 1.14)	**3.76E-02**	0.95 (0.89, 1.02)	2.26E-01
0.80 (0.74, 0.86)	**6.03E-10**	0.86 (0.81, 0.92)	**1.28E-04**
0.77 (0.71, 0.82)	**2.60E-13**	0.84 (0.79, 0.90)	**1.42E-05**
0.77 (0.72, 0.83)	**1.30E-12**	0.86 (0.80, 0.92)	**6.30E-05**
0.75 (0.70, 0.80)	**9.35E-16**	0.84 (0.78, 0.90)	**6.64E-06**
0.76 (0.71, 0.82)	**2.93E-13**	0.84 (0.78, 0.90)	**8.35E-06**
0.73 (0.68, 0.78)	**1.24E-19**	0.85 (0.80, 0.91)	**2.49E-05**
1.06 (0.99, 1.13)	1.07E-01	0.94 (0.88, 1.01)	1.25E-01
0.96 (0.90, 1.02)	2.58E-01	1.10 (1.03, 1.18)	**1.25E-02**
0.97 (0.91, 1.04)	4.84E-01	1.12 (1.05, 1.20)	**3.16E-03**
0.99 (0.93, 1.06)	8.42E-01	1.13 (1.06, 1.22)	**1.17E-03**
0.94 (0.88, 1.01)	1.08E-01	1.10 (1.03, 1.18)	**1.03E-02**
0.94 (0.87, 1.00)	7.70E-02	1.10 (1.03, 1.18)	**1.30E-02**
0.97 (0.90, 1.03)	3.58E-01	1.11 (1.04, 1.18)	**6.72E-03**
1.09 (1.02, 1.16)	**1.06E-02**	1.02 (0.95, 1.09)	6.82E-01
0.94 (0.88, 1.01)	1.29E-01	1.09 (1.01, 1.17)	**3.16E-02**
0.95 (0.89, 1.02)	2.07E-01	1.09 (1.02, 1.18)	**2.76E-02**
0.96 (0.90, 1.03)	3.40E-01	1.09 (1.01, 1.17)	**3.69E-02**
0.94 (0.87, 1.00)	7.53E-02	1.10 (1.02, 1.18)	**2.55E-02**
0.93 (0.87, 1.00)	6.02E-02	1.10 (1.02, 1.18)	**2.55E-02**
0.95 (0.89, 1.01)	1.51E-01	1.09 (1.02, 1.18)	**2.68E-02**
1.10 (1.04, 1.17)	**2.85E-03**	1.05 (0.98, 1.12)	2.48E-01
0.90 (0.84, 0.96)	**3.95E-03**	0.97 (0.90, 1.04)	4.52E-01
0.91 (0.86, 0.98)	**1.48E-02**	0.96 (0.90, 1.04)	4.17E-01
0.93 (0.87, 1.00)	5.51E-02	0.96 (0.90, 1.04)	3.85E-01
0.89 (0.83, 0.95)	**8.02E-04**	0.97 (0.90, 1.04)	5.13E-01
0.88 (0.82, 0.95)	**6.30E-04**	0.97 (0.90, 1.04)	5.12E-01

0.90 (0.84, 0.96)	**2.85E-03**	0.97 (0.90, 1.05)	5.37E-01
1.10 (1.03, 1.17)	**6.02E-03**	0.97 (0.91, 1.04)	4.99E-01
0.79 (0.74, 0.85)	**9.86E-11**	0.83 (0.77, 0.88)	**2.79E-06**
0.86 (0.80, 0.92)	**2.37E-05**	0.85 (0.79, 0.91)	**4.19E-05**
0.88 (0.83, 0.95)	**7.65E-04**	0.87 (0.81, 0.93)	**3.07E-04**
0.79 (0.73, 0.84)	**2.69E-11**	0.83 (0.78, 0.89)	**4.51E-06**
0.78 (0.73, 0.83)	**1.48E-12**	0.83 (0.77, 0.89)	**2.91E-06**
0.85 (0.80, 0.91)	**8.77E-06**	0.87 (0.81, 0.93)	**3.66E-04**
1.10 (1.03, 1.17)	**4.77E-03**	0.94 (0.88, 1.01)	1.13E-01
1.25 (1.16, 1.34)	**3.77E-09**	1.10 (1.02, 1.19)	**2.90E-02**
1.01 (0.95, 1.08)	7.49E-01	1.09 (1.02, 1.16)	**2.03E-02**
0.98 (0.91, 1.05)	5.49E-01	1.06 (0.99, 1.13)	1.45E-01
1.08 (1.01, 1.14)	**2.27E-02**	1.12 (1.06, 1.19)	**7.36E-04**
0.93 (0.88, 0.99)	**4.42E-02**	0.91 (0.85, 0.97)	**6.58E-03**
0.89 (0.83, 0.96)	**2.85E-03**	1.10 (1.02, 1.17)	**1.72E-02**
0.87 (0.81, 0.94)	**6.57E-04**	1.08 (1.00, 1.15)	6.44E-02
0.98 (0.92, 1.05)	5.74E-01	1.15 (1.07, 1.22)	**2.61E-04**
1.11 (1.04, 1.19)	**5.53E-03**	0.91 (0.85, 0.98)	**1.93E-02**
1.10 (1.02, 1.19)	**1.42E-02**	0.96 (0.90, 1.03)	3.67E-01
0.97 (0.91, 1.04)	5.04E-01	1.09 (1.02, 1.17)	**2.10E-02**
0.94 (0.88, 1.01)	9.31E-02	1.11 (1.03, 1.18)	**7.05E-03**
1.08 (1.01, 1.15)	**4.69E-02**	1.03 (0.96, 1.10)	5.62E-01
0.97 (0.90, 1.04)	3.92E-01	0.95 (0.89, 1.02)	2.57E-01
0.81 (0.77, 0.86)	**2.11E-11**	0.99 (0.93, 1.06)	8.29E-01
0.80 (0.75, 0.86)	**4.06E-11**	1.02 (0.95, 1.09)	6.30E-01
0.80 (0.75, 0.85)	**2.53E-11**	1.03 (0.96, 1.10)	5.46E-01
0.82 (0.77, 0.87)	**8.16E-10**	1.01 (0.94, 1.08)	9.03E-01
1.25 (1.17, 1.33)	**2.07E-11**	0.99 (0.92, 1.05)	7.64E-01
0.72 (0.68, 0.77)	**5.48E-22**	0.93 (0.87, 0.99)	5.35E-02
0.78 (0.73, 0.83)	**1.23E-13**	0.97 (0.91, 1.04)	4.54E-01
0.85 (0.80, 0.90)	**9.60E-07**	1.00 (0.93, 1.07)	9.59E-01
0.73 (0.68, 0.77)	**1.43E-21**	0.94 (0.87, 1.00)	9.18E-02
1.31 (1.23, 1.40)	**1.69E-16**	1.05 (0.98, 1.12)	2.59E-01
1.41 (1.32, 1.50)	**9.88E-24**	1.15 (1.07, 1.23)	**3.51E-04**
0.74 (0.70, 0.78)	**9.88E-24**	0.94 (0.88, 1.00)	9.80E-02
0.73 (0.69, 0.77)	**1.50E-25**	0.93 (0.87, 1.00)	6.97E-02
0.86 (0.81, 0.92)	**3.92E-06**	0.99 (0.93, 1.06)	8.32E-01
1.32 (1.24, 1.40)	**8.83E-20**	1.04 (0.98, 1.11)	2.96E-01
1.31 (1.23, 1.39)	**1.56E-16**	1.16 (1.09, 1.24)	**4.47E-05**
0.71 (0.67, 0.75)	**1.81E-36**	0.87 (0.82, 0.93)	**4.71E-05**
0.68 (0.64, 0.72)	**3.57E-44**	0.84 (0.79, 0.89)	**2.00E-06**
0.89 (0.84, 0.95)	**5.81E-04**	1.00 (0.94, 1.07)	9.82E-01
1.36 (1.30, 1.43)	**1.83E-34**	1.12 (1.05, 1.19)	**6.74E-04**
1.01 (0.94, 1.08)	8.18E-01	1.05 (0.99, 1.11)	1.37E-01
0.88 (0.87, 0.90)	**4.16E-35**	0.91 (0.89, 0.94)	**5.52E-07**
0.90 (0.88, 0.92)	**5.12E-19**	0.91 (0.89, 0.94)	**2.15E-08**
0.79 (0.75, 0.83)	**2.15E-21**	0.95 (0.89, 1.01)	1.23E-01
1.18 (1.15, 1.21)	**4.91E-44**	1.11 (1.07, 1.16)	**2.79E-06**

0.99 (0.92, 1.05)	7.22E-01	1.04 (0.98, 1.11)	2.86E-01
0.88 (0.86, 0.90)	**6.03E-33**	0.92 (0.89, 0.95)	**2.98E-06**
0.90 (0.85, 0.95)	**3.97E-04**	0.88 (0.84, 0.93)	**1.38E-05**
0.83 (0.78, 0.88)	**9.63E-10**	1.00 (0.93, 1.07)	9.53E-01
1.16 (1.14, 1.19)	**3.57E-44**	1.10 (1.06, 1.14)	**3.03E-06**
1.15 (1.08, 1.22)	**3.29E-05**	1.13 (1.06, 1.19)	**3.53E-04**
0.84 (0.82, 0.86)	**4.91E-44**	0.89 (0.86, 0.93)	**2.79E-06**
0.86 (0.81, 0.92)	**2.20E-06**	0.89 (0.84, 0.94)	**3.28E-04**
0.83 (0.79, 0.88)	**9.44E-11**	0.97 (0.91, 1.03)	3.47E-01
1.17 (1.13, 1.20)	**1.36E-24**	1.06 (1.00, 1.12)	8.76E-02
1.07 (1.00, 1.15)	8.44E-02	1.15 (1.06, 1.24)	**1.82E-03**
0.85 (0.79, 0.92)	**2.61E-05**	0.89 (0.83, 0.95)	**3.15E-03**
0.83 (0.79, 0.88)	**2.55E-11**	0.95 (0.89, 1.01)	1.15E-01
1.01 (0.95, 1.08)	7.52E-01	0.90 (0.84, 0.97)	**1.62E-02**
1.06 (1.01, 1.12)	**2.63E-02**	0.94 (0.88, 1.01)	1.36E-01
1.16 (1.09, 1.23)	**1.60E-06**	0.94 (0.89, 1.00)	1.03E-01
0.86 (0.82, 0.91)	**8.19E-07**	1.04 (0.98, 1.12)	2.58E-01
0.86 (0.81, 0.91)	**1.07E-06**	1.05 (0.98, 1.12)	2.57E-01
0.90 (0.85, 0.96)	**9.98E-04**	1.03 (0.96, 1.10)	4.99E-01
1.10 (1.05, 1.15)	**5.13E-05**	0.98 (0.92, 1.05)	7.04E-01
1.21 (1.14, 1.29)	**6.04E-09**	1.01 (0.95, 1.09)	7.68E-01
0.84 (0.79, 0.89)	**2.43E-08**	0.99 (0.93, 1.06)	8.48E-01
0.85 (0.80, 0.90)	**5.39E-07**	1.00 (0.93, 1.07)	9.53E-01
0.84 (0.79, 0.90)	**5.56E-07**	0.97 (0.90, 1.04)	4.69E-01
1.12 (1.08, 1.16)	**1.62E-10**	1.01 (0.94, 1.08)	9.29E-01
1.17 (1.10, 1.25)	**4.62E-06**	1.11 (1.03, 1.18)	**9.74E-03**
0.79 (0.75, 0.84)	**8.48E-13**	0.93 (0.87, 1.00)	6.44E-02
0.80 (0.76, 0.85)	**3.97E-12**	0.92 (0.86, 0.98)	**2.25E-02**
0.97 (0.90, 1.04)	3.92E-01	1.05 (0.99, 1.13)	1.64E-01
1.20 (1.13, 1.28)	**2.19E-09**	1.01 (0.95, 1.09)	7.82E-01
0.98 (0.92, 1.04)	5.49E-01	0.85 (0.79, 0.91)	**4.62E-05**
0.90 (0.85, 0.96)	**3.31E-03**	1.05 (0.98, 1.12)	2.48E-01
1.00 (0.94, 1.07)	9.87E-01	1.14 (1.06, 1.23)	**9.33E-04**
0.74 (0.69, 0.79)	**9.61E-17**	0.87 (0.81, 0.93)	**2.63E-04**
0.87 (0.82, 0.94)	**2.11E-04**	0.89 (0.84, 0.96)	**3.99E-03**
0.73 (0.68, 0.78)	**5.33E-18**	0.84 (0.78, 0.90)	**7.73E-06**
0.78 (0.73, 0.84)	**1.77E-11**	0.88 (0.82, 0.94)	**5.46E-04**
0.74 (0.69, 0.79)	**1.63E-17**	0.86 (0.81, 0.93)	**2.34E-04**
0.76 (0.71, 0.81)	**2.61E-14**	0.87 (0.81, 0.93)	**4.36E-04**
1.03 (0.97, 1.10)	3.71E-01	0.92 (0.85, 0.98)	**2.55E-02**
1.02 (0.95, 1.08)	6.74E-01	0.90 (0.84, 0.97)	**1.12E-02**
1.08 (1.02, 1.15)	**1.78E-02**	0.96 (0.90, 1.03)	3.85E-01
1.11 (1.04, 1.18)	**2.22E-03**	0.98 (0.91, 1.05)	6.20E-01
0.86 (0.81, 0.92)	**4.00E-05**	0.91 (0.84, 0.97)	**1.40E-02**
1.12 (1.05, 1.19)	**1.19E-03**	0.94 (0.88, 1.01)	1.43E-01
0.84 (0.79, 0.90)	**2.65E-06**	0.90 (0.84, 0.97)	**1.30E-02**

0.82 (0.77, 0.88)	**5.89E-08**	0.93 (0.87, 1.00)	7.39E-02
0.83 (0.78, 0.89)	**3.81E-07**	0.91 (0.84, 0.97)	**1.40E-02**
0.89 (0.83, 0.95)	**1.13E-03**	0.96 (0.89, 1.04)	4.02E-01
0.78 (0.73, 0.84)	**2.58E-11**	0.87 (0.81, 0.93)	**3.45E-04**
0.88 (0.82, 0.94)	**4.51E-04**	0.92 (0.85, 0.98)	**2.29E-02**
0.94 (0.88, 1.00)	8.53E-02	0.90 (0.84, 0.96)	**6.37E-03**
0.85 (0.79, 0.91)	**2.45E-06**	0.93 (0.87, 1.00)	7.37E-02
0.93 (0.87, 1.00)	5.74E-02	0.92 (0.85, 0.98)	**2.93E-02**
0.78 (0.73, 0.84)	**8.70E-12**	0.89 (0.83, 0.95)	**2.49E-03**
0.97 (0.91, 1.04)	4.22E-01	0.89 (0.82, 0.95)	**3.09E-03**
0.79 (0.74, 0.85)	**2.18E-10**	0.87 (0.81, 0.94)	**5.87E-04**
0.82 (0.76, 0.87)	**1.99E-08**	0.86 (0.80, 0.92)	**1.83E-04**
1.04 (0.98, 1.11)	2.40E-01	0.94 (0.88, 1.01)	1.34E-01
0.97 (0.91, 1.03)	3.92E-01	0.91 (0.85, 0.97)	**1.40E-02**
0.98 (0.91, 1.04)	5.44E-01	0.99 (0.92, 1.06)	7.94E-01
0.75 (0.70, 0.80)	**1.96E-18**	0.98 (0.92, 1.05)	6.98E-01
1.01 (0.95, 1.08)	7.68E-01	0.93 (0.87, 1.00)	6.95E-02
0.82 (0.77, 0.87)	**6.81E-10**	1.02 (0.96, 1.10)	5.93E-01
0.83 (0.78, 0.88)	**6.28E-09**	0.99 (0.93, 1.06)	8.99E-01
1.24 (1.16, 1.32)	**6.21E-11**	1.03 (0.96, 1.10)	4.94E-01
1.08 (1.01, 1.15)	**2.77E-02**	0.98 (0.91, 1.04)	5.62E-01
1.17 (1.12, 1.22)	**2.93E-13**	1.08 (1.02, 1.13)	**9.74E-03**
1.05 (0.98, 1.12)	2.12E-01	1.00 (0.94, 1.07)	9.53E-01
1.14 (1.09, 1.19)	**6.08E-08**	1.06 (1.01, 1.13)	5.30E-02
1.07 (1.00, 1.14)	5.60E-02	1.01 (0.95, 1.08)	7.43E-01
1.14 (1.07, 1.22)	**1.51E-04**	1.11 (1.04, 1.19)	**4.31E-03**
0.90 (0.84, 0.97)	**9.15E-03**	0.99 (0.92, 1.06)	8.32E-01
1.00 (0.94, 1.07)	9.99E-01	0.96 (0.90, 1.03)	3.05E-01
0.91 (0.85, 0.98)	**1.47E-02**	0.88 (0.82, 0.95)	**2.29E-03**
0.95 (0.88, 1.01)	1.34E-01	0.88 (0.82, 0.95)	**2.29E-03**
1.04 (1.01, 1.07)	**5.99E-03**	1.02 (0.95, 1.09)	6.51E-01
1.10 (1.04, 1.17)	**3.34E-03**	1.00 (0.94, 1.07)	9.95E-01
1.08 (1.05, 1.11)	**6.87E-07**	1.07 (1.04, 1.10)	**3.04E-05**
1.09 (1.06, 1.12)	**1.08E-10**	1.07 (1.03, 1.11)	**9.39E-04**
1.09 (1.05, 1.12)	**2.70E-07**	1.08 (1.03, 1.13)	**1.73E-03**
1.11 (1.09, 1.13)	**5.00E-26**	1.07 (1.04, 1.10)	**5.25E-05**
0.78 (0.73, 0.83)	**1.89E-13**	0.91 (0.85, 0.97)	**8.94E-03**
1.26 (1.19, 1.34)	**3.65E-13**	1.07 (1.00, 1.15)	6.97E-02
0.95 (0.89, 1.02)	2.01E-01	1.09 (1.01, 1.17)	**3.49E-02**
0.94 (0.88, 1.01)	1.21E-01	0.89 (0.83, 0.96)	**5.67E-03**
0.75 (0.70, 0.80)	**3.97E-15**	0.85 (0.80, 0.91)	**4.62E-05**
0.72 (0.67, 0.77)	**1.74E-19**	0.85 (0.79, 0.91)	**1.52E-05**
0.74 (0.69, 0.79)	**1.69E-16**	0.84 (0.78, 0.90)	**6.38E-06**
0.87 (0.81, 0.93)	**1.38E-04**	1.00 (0.93, 1.08)	9.53E-01
0.81 (0.76, 0.86)	**7.44E-10**	0.99 (0.92, 1.06)	8.32E-01
0.97 (0.90, 1.04)	4.03E-01	1.07 (1.02, 1.13)	**8.93E-03**
0.91 (0.85, 0.97)	**9.95E-03**	0.89 (0.83, 0.95)	**3.16E-03**
0.71 (0.66, 0.76)	**6.24E-21**	0.85 (0.79, 0.91)	**2.65E-05**

0.90 (0.84, 0.96)	**4.37E-03**	1.01 (0.94, 1.09)	8.32E-01
1.03 (0.97, 1.08)	4.17E-01	1.00 (0.94, 1.08)	9.48E-01
1.08 (1.05, 1.10)	**3.24E-09**	1.08 (1.05, 1.12)	**1.54E-05**
0.81 (0.75, 0.86)	**2.36E-09**	0.89 (0.83, 0.96)	**4.01E-03**
0.93 (0.87, 0.99)	**4.74E-02**	0.91 (0.85, 0.97)	**9.74E-03**
0.78 (0.73, 0.84)	**9.21E-12**	0.87 (0.81, 0.93)	**3.07E-04**
0.83 (0.77, 0.89)	**2.86E-07**	0.91 (0.84, 0.98)	**1.61E-02**
0.82 (0.77, 0.88)	**2.26E-08**	0.89 (0.83, 0.96)	**3.38E-03**
0.94 (0.88, 1.01)	1.02E-01	0.90 (0.84, 0.96)	**5.81E-03**
0.74 (0.69, 0.79)	**4.20E-17**	0.84 (0.79, 0.90)	**1.08E-05**
0.93 (0.87, 0.99)	**3.78E-02**	1.00 (0.92, 1.07)	9.53E-01
0.81 (0.75, 0.86)	**2.59E-09**	0.87 (0.81, 0.93)	**2.85E-04**
0.96 (0.90, 1.03)	2.79E-01	0.89 (0.83, 0.96)	**4.66E-03**
0.74 (0.69, 0.80)	**4.61E-16**	0.84 (0.79, 0.90)	**1.25E-05**
0.91 (0.85, 0.97)	**8.33E-03**	1.00 (0.93, 1.08)	9.82E-01
0.85 (0.79, 0.91)	**7.95E-06**	0.90 (0.84, 0.96)	**5.93E-03**
0.87 (0.82, 0.94)	**2.92E-04**	1.01 (0.93, 1.08)	9.40E-01
1.02 (0.96, 1.10)	5.47E-01	1.00 (0.93, 1.08)	9.61E-01

			**Cancer**
**CKB**			
**Unadjusted HR (95% CI)**	**P value**	**Adjusted HR (95% CI)**	**P value**
0.98 (0.91, 1.06)	7.09E-01	0.93 (0.85, 1.01)	2.50E-01
0.99 (0.91, 1.06)	7.63E-01	0.93 (0.85, 1.01)	2.76E-01
0.98 (0.91, 1.06)	7.03E-01	0.93 (0.86, 1.01)	2.88E-01
1.05 (0.97, 1.13)	2.89E-01	0.97 (0.89, 1.05)	6.34E-01
1.09 (1.01, 1.17)	**3.79E-02**	0.98 (0.91, 1.06)	7.77E-01
1.01 (0.93, 1.09)	8.93E-01	0.96 (0.88, 1.04)	5.23E-01
0.97 (0.90, 1.05)	5.43E-01	0.92 (0.84, 1.00)	1.91E-01
1.01 (0.94, 1.09)	7.69E-01	0.98 (0.90, 1.06)	7.45E-01
1.02 (0.94, 1.10)	7.37E-01	0.98 (0.90, 1.06)	7.45E-01
1.03 (0.95, 1.11)	5.43E-01	0.98 (0.90, 1.06)	7.45E-01
1.04 (0.97, 1.12)	3.55E-01	0.99 (0.91, 1.07)	8.28E-01
1.04 (0.97, 1.12)	3.56E-01	0.99 (0.91, 1.07)	8.28E-01
1.04 (0.97, 1.12)	3.32E-01	0.99 (0.91, 1.07)	8.52E-01
1.01 (0.93, 1.09)	8.96E-01	0.97 (0.90, 1.06)	7.35E-01
0.98 (0.91, 1.06)	6.98E-01	0.94 (0.87, 1.02)	3.65E-01
0.98 (0.91, 1.06)	7.54E-01	0.94 (0.87, 1.02)	3.79E-01
1.00 (0.92, 1.07)	9.37E-01	0.94 (0.87, 1.02)	3.80E-01
1.02 (0.95, 1.10)	6.19E-01	0.96 (0.88, 1.03)	4.74E-01
1.05 (0.98, 1.13)	2.45E-01	0.96 (0.89, 1.04)	5.45E-01
1.00 (0.93, 1.08)	9.83E-01	0.95 (0.88, 1.03)	4.13E-01
0.97 (0.89, 1.04)	4.64E-01	0.94 (0.87, 1.02)	3.47E-01
0.98 (0.91, 1.06)	7.01E-01	0.94 (0.87, 1.01)	3.03E-01
0.99 (0.92, 1.07)	8.13E-01	0.94 (0.87, 1.01)	3.08E-01
1.00 (0.93, 1.08)	9.83E-01	0.94 (0.87, 1.01)	3.08E-01
1.07 (0.99, 1.15)	1.04E-01	0.96 (0.89, 1.03)	5.24E-01
1.12 (1.05, 1.21)	**2.12E-03**	0.98 (0.91, 1.05)	7.14E-01
1.01 (0.94, 1.08)	8.87E-01	0.95 (0.88, 1.02)	3.80E-01
0.95 (0.88, 1.03)	2.55E-01	0.93 (0.86, 1.00)	2.35E-01
1.07 (0.99, 1.15)	1.03E-01	0.95 (0.88, 1.02)	3.95E-01
1.07 (1.00, 1.15)	7.77E-02	0.95 (0.89, 1.02)	4.00E-01
1.09 (1.01, 1.17)	**3.68E-02**	0.95 (0.88, 1.02)	3.53E-01
1.10 (1.02, 1.18)	**1.55E-02**	0.97 (0.90, 1.04)	6.09E-01
1.10 (1.02, 1.18)	**1.64E-02**	0.98 (0.91, 1.05)	7.45E-01
1.09 (1.01, 1.17)	**3.72E-02**	0.96 (0.89, 1.03)	4.67E-01
1.04 (0.97, 1.12)	3.27E-01	0.95 (0.88, 1.02)	3.80E-01
1.21 (1.14, 1.30)	**6.35E-08**	0.98 (0.91, 1.05)	7.45E-01
1.20 (1.12, 1.29)	**3.46E-07**	0.98 (0.91, 1.05)	7.45E-01
1.25 (1.17, 1.34)	**2.17E-10**	1.00 (0.93, 1.07)	9.32E-01
1.12 (1.04, 1.20)	**4.35E-03**	0.97 (0.91, 1.04)	6.67E-01
1.10 (1.03, 1.19)	**1.27E-02**	0.97 (0.91, 1.04)	6.17E-01
1.14 (1.06, 1.22)	**6.44E-04**	0.98 (0.92, 1.05)	7.56E-01

1.19 (1.11, 1.27)	**1.64E-06**	0.98 (0.92, 1.05)	7.80E-01
1.26 (1.18, 1.34)	**3.01E-11**	1.00 (0.93, 1.07)	9.84E-01
1.25 (1.17, 1.33)	**2.48E-10**	1.00 (0.93, 1.07)	9.84E-01
1.25 (1.17, 1.33)	**6.36E-10**	1.00 (0.93, 1.07)	9.32E-01
1.21 (1.13, 1.30)	**7.89E-08**	1.00 (0.93, 1.07)	9.84E-01
1.21 (1.13, 1.29)	**2.38E-07**	0.99 (0.92, 1.06)	9.06E-01
1.23 (1.15, 1.32)	**3.47E-08**	1.01 (0.95, 1.09)	8.15E-01
1.31 (1.23, 1.38)	**2.38E-19**	1.02 (0.95, 1.10)	7.48E-01
1.24 (1.16, 1.32)	**3.83E-09**	0.98 (0.92, 1.06)	7.92E-01
1.23 (1.15, 1.31)	**1.36E-08**	0.98 (0.92, 1.06)	7.92E-01
1.23 (1.15, 1.32)	**1.38E-08**	0.98 (0.91, 1.06)	7.80E-01
1.20 (1.12, 1.29)	**7.12E-07**	0.98 (0.91, 1.05)	7.50E-01
1.19 (1.11, 1.28)	**2.20E-06**	0.97 (0.91, 1.05)	6.73E-01
1.22 (1.14, 1.31)	**4.45E-08**	1.00 (0.93, 1.07)	9.84E-01
1.32 (1.25, 1.39)	**1.04E-21**	1.04 (0.97, 1.12)	5.36E-01
1.19 (1.11, 1.28)	**2.59E-06**	0.96 (0.89, 1.03)	4.63E-01
1.19 (1.10, 1.27)	**6.81E-06**	0.96 (0.89, 1.03)	4.53E-01
1.21 (1.13, 1.30)	**4.57E-07**	0.95 (0.88, 1.02)	4.00E-01
1.15 (1.07, 1.24)	**2.56E-04**	0.95 (0.89, 1.03)	4.24E-01
1.14 (1.07, 1.23)	**4.70E-04**	0.95 (0.89, 1.03)	4.13E-01
1.18 (1.10, 1.27)	**2.52E-05**	0.96 (0.89, 1.03)	4.96E-01
1.33 (1.25, 1.41)	**2.13E-20**	1.02 (0.95, 1.10)	7.42E-01
1.15 (1.07, 1.24)	**2.42E-04**	0.94 (0.88, 1.02)	3.52E-01
1.15 (1.07, 1.23)	**4.43E-04**	0.94 (0.88, 1.02)	3.47E-01
1.17 (1.09, 1.26)	**1.02E-04**	0.94 (0.87, 1.02)	3.36E-01
1.12 (1.04, 1.20)	**4.33E-03**	0.95 (0.88, 1.02)	3.53E-01
1.12 (1.04, 1.20)	**5.04E-03**	0.95 (0.88, 1.02)	3.53E-01
1.12 (1.04, 1.21)	**5.14E-03**	0.94 (0.87, 1.01)	3.08E-01
1.21 (1.13, 1.30)	**8.39E-08**	0.98 (0.91, 1.05)	7.56E-01
1.15 (1.07, 1.23)	**3.07E-04**	1.12 (1.05, 1.19)	**4.75E-03**
1.14 (1.06, 1.23)	**6.66E-04**	1.12 (1.05, 1.19)	**5.91E-03**
1.23 (1.15, 1.32)	**2.07E-08**	1.16 (1.08, 1.23)	**1.55E-04**
1.02 (0.95, 1.10)	6.62E-01	1.07 (1.00, 1.14)	1.97E-01
0.98 (0.91, 1.05)	6.35E-01	1.05 (0.98, 1.12)	3.99E-01
1.13 (1.06, 1.22)	**1.23E-03**	1.12 (1.05, 1.19)	**4.50E-03**
1.14 (1.07, 1.22)	**2.98E-04**	1.05 (0.97, 1.13)	4.22E-01
1.16 (1.08, 1.24)	**1.79E-04**	1.14 (1.06, 1.22)	**2.04E-03**
1.15 (1.07, 1.23)	**3.05E-04**	1.14 (1.06, 1.22)	**2.04E-03**
1.13 (1.05, 1.22)	**1.63E-03**	1.13 (1.05, 1.21)	**4.72E-03**
1.14 (1.06, 1.22)	**8.36E-04**	1.14 (1.06, 1.22)	**1.62E-03**
1.14 (1.07, 1.23)	**4.67E-04**	1.14 (1.07, 1.22)	**1.52E-03**
1.12 (1.04, 1.20)	**5.40E-03**	1.13 (1.06, 1.21)	**2.08E-03**
1.35 (1.27, 1.44)	**3.06E-19**	1.12 (1.04, 1.20)	**9.52E-03**
1.02 (0.95, 1.10)	6.43E-01	1.02 (0.95, 1.10)	7.75E-01
1.01 (0.94, 1.09)	7.80E-01	1.02 (0.95, 1.10)	7.56E-01
1.05 (0.97, 1.13)	3.11E-01	1.02 (0.95, 1.10)	7.75E-01
0.95 (0.88, 1.02)	1.96E-01	1.02 (0.95, 1.10)	7.45E-01
0.93 (0.86, 1.00)	7.55E-02	1.02 (0.95, 1.10)	7.75E-01

1.02 (0.94, 1.10)	7.20E-01	1.04 (0.96, 1.11)	5.67E-01
1.24 (1.15, 1.32)	**4.29E-09**	1.00 (0.93, 1.07)	9.84E-01
1.03 (0.96, 1.11)	4.71E-01	0.95 (0.88, 1.02)	4.00E-01
1.03 (0.95, 1.11)	5.62E-01	0.95 (0.88, 1.03)	4.13E-01
0.93 (0.86, 1.00)	9.06E-02	0.95 (0.88, 1.02)	4.00E-01
1.09 (1.01, 1.18)	**3.10E-02**	0.99 (0.92, 1.07)	9.28E-01
1.11 (1.03, 1.20)	**1.18E-02**	1.00 (0.93, 1.08)	9.84E-01
0.93 (0.86, 1.01)	1.04E-01	0.97 (0.90, 1.05)	6.99E-01
1.17 (1.09, 1.26)	**1.54E-05**	0.96 (0.90, 1.04)	5.49E-01
0.87 (0.81, 0.93)	**1.04E-04**	0.86 (0.81, 0.92)	**2.71E-04**
1.22 (1.16, 1.28)	**1.91E-14**	1.16 (1.09, 1.23)	**1.05E-04**
1.23 (1.17, 1.29)	**3.46E-15**	1.15 (1.08, 1.23)	**1.67E-04**
1.08 (1.01, 1.16)	**4.04E-02**	1.08 (0.99, 1.16)	2.53E-01
0.85 (0.79, 0.90)	**3.07E-06**	0.92 (0.85, 1.00)	1.85E-01
1.03 (0.95, 1.11)	5.74E-01	0.93 (0.86, 1.00)	1.85E-01
1.16 (1.10, 1.22)	**5.63E-08**	1.05 (0.99, 1.11)	3.08E-01
1.12 (1.05, 1.20)	**6.36E-04**	1.05 (0.99, 1.11)	3.08E-01
1.15 (1.07, 1.23)	**2.56E-04**	1.02 (0.94, 1.10)	7.92E-01
0.84 (0.78, 0.91)	**3.23E-05**	0.99 (0.92, 1.07)	8.63E-01
1.23 (1.13, 1.34)	**1.04E-05**	0.94 (0.87, 1.02)	3.53E-01
1.36 (1.24, 1.49)	**9.96E-11**	1.04 (0.96, 1.13)	5.45E-01
1.38 (1.29, 1.47)	**9.91E-20**	1.14 (1.06, 1.22)	**1.95E-03**
0.98 (0.91, 1.06)	6.97E-01	0.91 (0.85, 0.98)	6.21E-02
0.73 (0.67, 0.80)	**2.26E-10**	0.98 (0.90, 1.07)	7.99E-01
1.39 (1.30, 1.48)	**1.92E-22**	1.10 (1.02, 1.17)	**4.42E-02**
1.51 (1.41, 1.62)	**5.10E-30**	1.16 (1.08, 1.25)	**7.95E-04**
1.45 (1.36, 1.55)	**3.93E-27**	1.16 (1.08, 1.24)	**4.02E-04**
1.05 (0.97, 1.13)	2.74E-01	0.96 (0.90, 1.03)	5.36E-01
0.65 (0.61, 0.70)	**7.51E-33**	0.86 (0.80, 0.92)	**5.21E-04**
1.13 (1.05, 1.21)	**1.43E-03**	1.06 (0.99, 1.13)	3.09E-01
1.04 (0.96, 1.12)	4.00E-01	1.04 (0.97, 1.11)	5.24E-01
1.03 (0.96, 1.11)	5.12E-01	1.03 (0.96, 1.11)	5.67E-01
1.07 (0.99, 1.15)	1.11E-01	1.06 (0.99, 1.14)	3.32E-01
0.94 (0.87, 1.01)	1.11E-01	0.95 (0.88, 1.02)	3.65E-01
1.28 (1.18, 1.40)	**1.95E-08**	1.07 (0.99, 1.16)	2.50E-01
0.83 (0.78, 0.89)	**6.31E-08**	0.96 (0.89, 1.02)	4.10E-01
0.82 (0.77, 0.88)	**8.39E-08**	0.95 (0.89, 1.02)	3.65E-01
0.90 (0.85, 0.96)	**3.72E-03**	0.99 (0.92, 1.06)	8.23E-01
1.06 (0.99, 1.13)	1.67E-01	1.01 (0.94, 1.08)	9.28E-01
0.92 (0.85, 0.99)	**3.10E-02**	0.99 (0.92, 1.07)	9.16E-01
0.90 (0.84, 0.96)	**3.60E-03**	0.98 (0.92, 1.04)	7.14E-01
0.86 (0.80, 0.92)	**2.27E-05**	0.94 (0.87, 1.00)	2.20E-01
1.01 (0.94, 1.09)	7.63E-01	1.04 (0.96, 1.12)	5.28E-01
1.13 (1.06, 1.21)	**4.91E-04**	1.03 (0.96, 1.10)	6.82E-01
0.82 (0.75, 0.90)	**3.69E-05**	1.01 (0.93, 1.09)	9.28E-01
0.99 (0.92, 1.07)	8.16E-01	0.97 (0.90, 1.04)	6.01E-01
1.01 (0.93, 1.08)	8.97E-01	0.95 (0.88, 1.01)	3.46E-01
0.96 (0.89, 1.03)	2.93E-01	1.07 (0.99, 1.16)	3.08E-01

1.13 (1.06, 1.20)	**3.05E-04**	1.04 (0.97, 1.12)	4.35E-01
0.93 (0.86, 1.01)	1.28E-01	1.02 (0.95, 1.10)	7.80E-01
0.98 (0.91, 1.06)	7.01E-01	0.96 (0.90, 1.04)	5.36E-01
1.03 (0.95, 1.11)	5.49E-01	0.96 (0.89, 1.03)	5.24E-01
0.85 (0.78, 0.92)	**1.65E-04**	1.04 (0.96, 1.12)	5.50E-01
1.16 (1.09, 1.24)	**1.21E-05**	1.07 (1.00, 1.15)	2.39E-01
0.96 (0.89, 1.03)	3.11E-01	1.04 (0.97, 1.12)	5.24E-01
1.00 (0.93, 1.07)	9.73E-01	0.96 (0.90, 1.03)	5.24E-01
1.03 (0.95, 1.11)	5.43E-01	0.96 (0.89, 1.03)	5.24E-01
0.85 (0.78, 0.92)	**1.36E-04**	1.03 (0.95, 1.11)	6.73E-01
1.09 (1.01, 1.16)	**2.99E-02**	1.03 (0.96, 1.11)	6.23E-01
1.40 (1.30, 1.51)	**4.10E-18**	1.19 (1.11, 1.28)	**5.79E-05**
0.70 (0.66, 0.75)	**7.91E-27**	0.84 (0.79, 0.90)	**2.80E-05**
0.67 (0.63, 0.72)	**4.57E-30**	0.81 (0.76, 0.87)	**3.26E-07**
0.90 (0.84, 0.96)	**2.98E-03**	1.01 (0.95, 1.08)	8.07E-01
1.06 (0.99, 1.14)	1.07E-01	0.95 (0.89, 1.02)	4.06E-01
0.87 (0.81, 0.94)	**1.19E-03**	0.89 (0.82, 0.96)	**1.03E-02**
0.96 (0.89, 1.03)	2.72E-01	1.09 (1.02, 1.17)	7.02E-02
0.99 (0.92, 1.06)	7.83E-01	1.10 (1.03, 1.19)	**4.08E-02**
0.92 (0.86, 0.99)	**3.39E-02**	1.06 (0.99, 1.14)	2.88E-01
1.25 (1.18, 1.33)	**6.31E-14**	1.01 (0.94, 1.08)	9.28E-01
1.17 (1.10, 1.23)	**5.29E-07**	1.01 (0.93, 1.09)	9.28E-01
0.82 (0.77, 0.86)	**1.95E-12**	0.99 (0.92, 1.07)	9.16E-01
0.81 (0.77, 0.85)	**1.09E-15**	0.98 (0.91, 1.06)	7.79E-01
0.97 (0.90, 1.05)	5.70E-01	1.03 (0.96, 1.11)	6.44E-01
1.24 (1.16, 1.32)	**3.45E-10**	1.01 (0.94, 1.08)	9.28E-01
0.84 (0.78, 0.91)	**2.91E-05**	0.97 (0.90, 1.05)	6.82E-01
1.10 (1.02, 1.19)	**2.95E-02**	1.03 (0.95, 1.11)	7.07E-01
1.11 (1.03, 1.20)	**1.18E-02**	1.02 (0.95, 1.10)	7.50E-01
0.82 (0.76, 0.89)	**2.46E-06**	1.02 (0.94, 1.10)	7.94E-01
1.17 (1.10, 1.26)	**7.75E-06**	0.99 (0.92, 1.06)	8.56E-01
0.82 (0.76, 0.89)	**2.07E-06**	0.88 (0.82, 0.95)	**7.16E-03**
1.31 (1.22, 1.40)	**3.73E-14**	1.19 (1.10, 1.28)	**7.30E-05**
1.09 (1.01, 1.17)	**3.39E-02**	1.12 (1.04, 1.19)	**1.01E-02**
1.18 (1.10, 1.27)	**7.84E-06**	1.00 (0.93, 1.07)	9.84E-01
1.07 (1.00, 1.15)	9.02E-02	0.95 (0.88, 1.02)	3.95E-01
1.15 (1.07, 1.23)	**3.05E-04**	0.96 (0.90, 1.04)	5.36E-01
1.15 (1.08, 1.24)	**1.65E-04**	0.96 (0.90, 1.04)	5.40E-01
1.08 (1.00, 1.16)	5.51E-02	1.10 (1.03, 1.18)	**3.05E-02**
1.09 (1.01, 1.17)	**3.09E-02**	1.11 (1.03, 1.19)	**2.03E-02**
0.99 (0.92, 1.06)	7.69E-01	1.04 (0.97, 1.11)	5.28E-01
1.32 (1.24, 1.41)	**2.89E-16**	0.99 (0.92, 1.07)	9.28E-01
1.23 (1.16, 1.30)	**2.53E-12**	0.99 (0.92, 1.07)	8.56E-01
1.03 (0.96, 1.11)	4.41E-01	0.94 (0.88, 1.02)	3.55E-01
0.97 (0.90, 1.05)	5.49E-01	0.93 (0.86, 1.00)	2.39E-01
1.30 (1.23, 1.38)	**3.30E-18**	1.02 (0.95, 1.10)	7.45E-01
1.27 (1.19, 1.36)	**1.95E-12**	1.03 (0.96, 1.11)	6.37E-01
1.23 (1.14, 1.32)	**7.90E-08**	1.05 (0.98, 1.13)	4.04E-01

0.93 (0.86, 1.00)	6.94E-02	0.94 (0.87, 1.01)	3.08E-01
1.32 (1.23, 1.41)	**1.43E-14**	1.02 (0.95, 1.10)	7.56E-01
1.30 (1.21, 1.40)	**1.85E-12**	0.95 (0.88, 1.03)	4.50E-01
1.32 (1.23, 1.42)	**1.91E-14**	1.05 (0.97, 1.13)	4.13E-01
1.08 (1.00, 1.16)	5.75E-02	1.07 (1.00, 1.15)	1.97E-01
1.14 (1.06, 1.22)	**8.48E-04**	0.95 (0.89, 1.02)	4.00E-01
1.11 (1.03, 1.19)	**1.10E-02**	0.92 (0.86, 0.99)	1.40E-01
1.18 (1.10, 1.27)	**5.00E-06**	0.99 (0.92, 1.07)	9.28E-01
1.36 (1.25, 1.47)	**2.99E-13**	0.99 (0.91, 1.07)	8.52E-01
0.76 (0.71, 0.82)	**4.92E-11**	0.89 (0.83, 0.96)	**8.13E-03**
1.23 (1.14, 1.32)	**6.28E-08**	0.99 (0.92, 1.07)	9.17E-01
0.92 (0.85, 0.99)	5.46E-02	0.89 (0.83, 0.97)	**2.12E-02**
1.29 (1.21, 1.38)	**1.67E-12**	1.01 (0.93, 1.08)	9.32E-01
1.23 (1.15, 1.32)	**1.42E-08**	0.98 (0.91, 1.06)	7.92E-01
1.10 (1.02, 1.18)	**1.71E-02**	0.97 (0.90, 1.05)	6.73E-01
1.20 (1.12, 1.28)	**4.42E-07**	1.03 (0.96, 1.11)	6.73E-01
0.63 (0.59, 0.68)	**2.59E-37**	0.84 (0.78, 0.91)	**6.33E-05**
1.12 (1.03, 1.22)	**1.38E-02**	1.01 (0.93, 1.10)	9.28E-01
0.67 (0.62, 0.72)	**1.54E-24**	0.81 (0.75, 0.87)	**3.71E-07**
1.28 (1.19, 1.39)	**8.65E-10**	1.05 (0.98, 1.14)	4.00E-01
1.14 (1.06, 1.23)	**1.23E-03**	0.98 (0.91, 1.06)	7.92E-01
0.81 (0.75, 0.87)	**2.19E-07**	0.90 (0.84, 0.97)	**3.65E-02**
1.06 (0.98, 1.14)	1.99E-01	1.19 (1.10, 1.28)	**1.49E-04**
1.14 (1.07, 1.21)	**6.47E-05**	0.99 (0.91, 1.06)	8.15E-01
0.76 (0.70, 0.82)	**3.45E-10**	0.77 (0.71, 0.84)	**1.93E-07**
1.73 (1.63, 1.82)	**4.14E-81**	1.27 (1.18, 1.37)	**6.33E-08**
0.84 (0.78, 0.91)	**1.68E-05**	0.80 (0.74, 0.86)	**3.26E-07**
0.90 (0.84, 0.97)	**9.41E-03**	0.79 (0.74, 0.86)	**1.93E-07**
0.59 (0.55, 0.64)	**2.58E-39**	0.74 (0.68, 0.80)	**5.58E-11**
0.93 (0.86, 1.00)	8.87E-02	0.89 (0.82, 0.96)	**1.54E-02**
0.81 (0.74, 0.87)	**5.18E-07**	0.83 (0.77, 0.90)	**1.49E-04**
0.96 (0.89, 1.03)	3.19E-01	0.83 (0.76, 0.89)	**6.44E-05**
0.89 (0.82, 0.97)	**1.33E-02**	0.86 (0.79, 0.93)	**1.78E-03**
0.99 (0.92, 1.07)	8.09E-01	0.87 (0.81, 0.94)	**2.94E-03**
1.01 (0.94, 1.09)	7.63E-01	0.96 (0.89, 1.04)	5.67E-01
1.16 (1.12, 1.21)	**4.47E-13**	1.13 (1.08, 1.20)	**6.61E-05**
1.17 (1.13, 1.22)	**1.51E-16**	1.09 (1.03, 1.15)	**1.03E-02**
1.04 (0.99, 1.09)	1.40E-01	0.73 (0.62, 0.87)	**2.31E-03**
1.07 (0.99, 1.15)	1.40E-01	1.09 (1.01, 1.17)	1.38E-01
0.86 (0.80, 0.94)	**7.61E-04**	1.00 (0.92, 1.07)	9.32E-01

mortality

UKB			
**Unadjusted HR (95% CI)**	**P value**	**Adjusted HR (95% CI)**	**P value**
1.11 (1.09, 1.13)	**1.11E-31**	1.02 (1.00, 1.04)	8.50E-02
1.11 (1.09, 1.13)	**2.73E-28**	1.02 (1.00, 1.04)	1.10E-01
1.12 (1.10, 1.14)	**9.75E-33**	1.02 (1.00, 1.04)	6.54E-02
1.10 (1.08, 1.12)	**2.62E-25**	1.02 (1.00, 1.04)	1.36E-01
1.09 (1.07, 1.11)	**3.06E-21**	1.01 (0.99, 1.03)	1.92E-01
1.11 (1.09, 1.13)	**4.94E-30**	1.02 (1.00, 1.04)	9.18E-02
1.10 (1.08, 1.12)	**1.55E-27**	1.02 (1.00, 1.04)	1.23E-01
1.10 (1.08, 1.12)	**5.85E-22**	1.00 (0.98, 1.02)	9.43E-01
1.09 (1.07, 1.11)	**3.09E-20**	1.00 (0.98, 1.02)	8.46E-01
1.09 (1.07, 1.11)	**3.10E-19**	1.00 (0.98, 1.02)	9.57E-01
1.05 (1.03, 1.07)	**6.04E-08**	0.99 (0.97, 1.01)	3.44E-01
1.03 (1.01, 1.05)	**1.04E-02**	0.98 (0.96, 1.00)	7.81E-02
1.08 (1.06, 1.10)	**3.69E-16**	1.00 (0.98, 1.02)	9.08E-01
1.10 (1.08, 1.12)	**3.42E-25**	1.00 (0.98, 1.02)	9.72E-01
1.08 (1.06, 1.10)	**6.01E-15**	0.99 (0.97, 1.01)	3.51E-01
1.07 (1.05, 1.09)	**2.76E-11**	0.98 (0.96, 1.00)	1.10E-01
1.08 (1.06, 1.10)	**1.44E-16**	0.99 (0.97, 1.01)	5.27E-01
1.05 (1.03, 1.07)	**3.26E-07**	0.98 (0.96, 1.00)	1.45E-01
1.03 (1.01, 1.05)	**4.09E-03**	0.98 (0.96, 1.00)	9.86E-02
1.07 (1.05, 1.09)	**1.63E-12**	0.99 (0.97, 1.01)	2.18E-01
1.07 (1.05, 1.09)	**5.21E-11**	0.98 (0.96, 1.00)	**4.59E-02**
0.96 (0.94, 0.98)	**1.26E-04**	0.96 (0.94, 0.98)	**1.24E-04**
0.98 (0.96, 1.00)	8.08E-02	0.96 (0.94, 0.98)	**1.39E-04**
0.96 (0.94, 0.97)	**8.31E-06**	0.96 (0.94, 0.98)	**1.72E-04**
0.89 (0.87, 0.90)	**5.96E-32**	0.95 (0.93, 0.97)	**2.73E-06**
0.85 (0.84, 0.87)	**2.22E-52**	0.95 (0.93, 0.97)	**6.51E-07**
0.94 (0.92, 0.96)	**1.30E-10**	0.96 (0.94, 0.98)	**6.33E-05**
1.05 (1.03, 1.07)	**2.49E-07**	0.97 (0.95, 0.99)	**1.08E-02**
1.06 (1.04, 1.08)	**6.84E-09**	0.99 (0.97, 1.01)	4.57E-01
1.05 (1.03, 1.07)	**5.45E-06**	0.99 (0.97, 1.01)	2.45E-01
0.98 (0.97, 1.00)	1.20E-01	0.97 (0.95, 0.99)	**5.75E-03**
0.98 (0.96, 1.00)	5.09E-02	0.98 (0.96, 1.00)	**2.90E-02**
1.00 (0.99, 1.02)	6.20E-01	0.99 (0.97, 1.01)	2.02E-01
0.94 (0.92, 0.96)	**4.37E-10**	0.96 (0.94, 0.98)	**1.57E-04**
1.12 (1.10, 1.14)	**2.35E-33**	1.01 (0.99, 1.03)	5.28E-01
1.02 (1.00, 1.04)	**4.26E-02**	1.02 (1.00, 1.04)	5.11E-02
1.03 (1.01, 1.05)	**4.18E-03**	1.03 (1.01, 1.05)	**1.61E-02**
1.06 (1.04, 1.08)	**1.06E-08**	1.04 (1.02, 1.06)	**2.91E-04**
0.95 (0.93, 0.97)	**2.20E-07**	1.00 (0.98, 1.02)	6.93E-01
0.92 (0.90, 0.94)	**4.08E-15**	1.00 (0.98, 1.02)	7.91E-01
1.01 (0.99, 1.03)	1.88E-01	1.02 (1.00, 1.04)	**4.13E-02**

1.16 (1.14, 1.18)	**5.26E-61**	1.05 (1.03, 1.07)	**4.81E-06**
0.89 (0.87, 0.91)	**3.26E-31**	0.96 (0.94, 0.98)	**3.67E-04**
0.87 (0.85, 0.89)	**5.26E-43**	0.96 (0.94, 0.97)	**2.26E-05**
0.88 (0.87, 0.90)	**8.48E-33**	0.97 (0.95, 0.99)	**2.85E-03**
0.84 (0.83, 0.86)	**4.28E-63**	0.94 (0.92, 0.96)	**4.12E-08**
0.84 (0.82, 0.85)	**7.01E-68**	0.94 (0.92, 0.96)	**5.21E-09**
0.86 (0.84, 0.88)	**5.80E-48**	0.95 (0.94, 0.97)	**1.43E-05**
1.15 (1.13, 1.17)	**4.74E-53**	1.06 (1.04, 1.08)	**1.18E-07**
0.89 (0.87, 0.91)	**2.16E-30**	0.95 (0.93, 0.97)	**2.36E-07**
0.86 (0.84, 0.88)	**2.37E-49**	0.93 (0.91, 0.95)	**4.23E-12**
0.85 (0.83, 0.87)	**7.42E-55**	0.93 (0.91, 0.95)	**2.27E-12**
0.84 (0.83, 0.86)	**1.11E-60**	0.92 (0.90, 0.94)	**4.04E-14**
0.85 (0.84, 0.87)	**1.29E-53**	0.92 (0.90, 0.94)	**5.97E-14**
0.82 (0.81, 0.84)	**2.05E-78**	0.92 (0.90, 0.94)	**5.94E-14**
1.14 (1.12, 1.16)	**2.51E-45**	1.04 (1.02, 1.06)	**3.72E-05**
0.92 (0.90, 0.94)	**1.41E-15**	0.95 (0.93, 0.97)	**5.40E-07**
0.90 (0.88, 0.92)	**2.93E-26**	0.93 (0.91, 0.95)	**1.66E-12**
0.89 (0.87, 0.90)	**2.08E-32**	0.92 (0.90, 0.94)	**4.04E-14**
0.89 (0.87, 0.90)	**2.39E-32**	0.92 (0.91, 0.94)	**9.94E-14**
0.91 (0.89, 0.93)	**2.11E-21**	0.93 (0.91, 0.95)	**1.07E-11**
0.84 (0.82, 0.85)	**2.81E-67**	0.91 (0.89, 0.93)	**2.22E-18**
1.13 (1.11, 1.15)	**8.79E-39**	1.03 (1.01, 1.05)	**1.53E-02**
0.93 (0.91, 0.95)	**7.07E-13**	0.95 (0.94, 0.97)	**1.21E-05**
0.90 (0.88, 0.92)	**5.45E-26**	0.94 (0.92, 0.95)	**2.52E-10**
0.90 (0.88, 0.91)	**1.43E-26**	0.94 (0.92, 0.96)	**5.65E-09**
0.88 (0.86, 0.90)	**1.98E-36**	0.93 (0.91, 0.95)	**1.24E-12**
0.90 (0.88, 0.92)	**7.56E-24**	0.94 (0.92, 0.95)	**2.47E-10**
0.83 (0.82, 0.85)	**1.15E-71**	0.92 (0.90, 0.93)	**5.67E-17**
1.11 (1.10, 1.14)	**1.74E-32**	1.02 (1.00, 1.04)	9.27E-02
0.92 (0.90, 0.94)	**8.09E-16**	1.03 (1.01, 1.05)	**1.93E-02**
0.92 (0.90, 0.94)	**7.46E-15**	1.04 (1.01, 1.06)	**3.24E-03**
0.93 (0.91, 0.95)	**3.27E-11**	1.04 (1.02, 1.07)	**3.07E-04**
0.90 (0.88, 0.92)	**3.88E-24**	1.02 (1.00, 1.04)	8.79E-02
0.89 (0.87, 0.91)	**2.17E-28**	1.01 (0.99, 1.04)	3.18E-01
0.94 (0.92, 0.96)	**5.80E-10**	1.05 (1.03, 1.07)	**2.10E-05**
1.11 (1.09, 1.13)	**1.84E-27**	1.05 (1.03, 1.07)	**2.54E-06**
0.89 (0.88, 0.91)	**3.44E-26**	1.00 (0.98, 1.02)	9.08E-01
0.90 (0.88, 0.92)	**8.85E-23**	1.01 (0.98, 1.03)	6.65E-01
0.91 (0.90, 0.93)	**6.82E-18**	1.01 (0.99, 1.03)	4.77E-01
0.88 (0.86, 0.90)	**3.09E-32**	1.00 (0.97, 1.02)	8.44E-01
0.88 (0.86, 0.89)	**4.33E-35**	0.99 (0.97, 1.02)	6.11E-01
0.90 (0.88, 0.92)	**8.26E-23**	1.01 (0.99, 1.03)	3.96E-01
1.10 (1.08, 1.12)	**1.30E-22**	1.06 (1.04, 1.08)	**3.58E-08**
0.90 (0.89, 0.92)	**6.93E-23**	0.96 (0.94, 0.98)	**2.94E-04**
0.92 (0.90, 0.94)	**6.36E-16**	0.96 (0.94, 0.99)	**2.01E-03**
0.94 (0.92, 0.96)	**3.99E-09**	0.97 (0.95, 0.99)	**1.93E-02**
0.88 (0.86, 0.90)	**5.70E-34**	0.95 (0.93, 0.97)	**9.95E-06**
0.88 (0.86, 0.90)	**2.43E-36**	0.95 (0.93, 0.97)	**3.05E-06**

0.90 (0.88, 0.92)	**3.88E-24**	0.96 (0.94, 0.98)	**1.02E-03**
1.14 (1.12, 1.16)	**1.15E-42**	1.04 (1.02, 1.06)	**2.22E-04**
0.89 (0.87, 0.91)	**1.64E-31**	0.90 (0.88, 0.92)	**2.84E-22**
0.95 (0.93, 0.96)	**3.67E-08**	0.93 (0.91, 0.95)	**2.08E-11**
0.97 (0.95, 0.98)	**4.62E-04**	0.95 (0.93, 0.96)	**1.57E-07**
0.87 (0.85, 0.89)	**1.05E-40**	0.90 (0.88, 0.92)	**1.36E-23**
0.86 (0.84, 0.88)	**1.55E-49**	0.89 (0.87, 0.91)	**3.34E-27**
0.93 (0.91, 0.95)	**4.07E-13**	0.93 (0.92, 0.95)	**3.78E-10**
1.16 (1.14, 1.18)	**6.67E-54**	1.03 (1.01, 1.05)	**2.09E-02**
1.15 (1.13, 1.17)	**4.99E-38**	1.04 (1.02, 1.06)	**1.35E-03**
0.95 (0.93, 0.97)	**2.99E-06**	1.00 (0.98, 1.03)	6.83E-01
0.94 (0.92, 0.96)	**2.12E-07**	1.00 (0.98, 1.02)	9.05E-01
0.98 (0.96, 1.00)	**2.10E-02**	1.01 (0.99, 1.03)	3.70E-01
1.00 (0.98, 1.02)	7.31E-01	0.99 (0.96, 1.01)	1.91E-01
0.84 (0.82, 0.86)	**2.39E-49**	0.99 (0.97, 1.01)	3.16E-01
0.83 (0.81, 0.85)	**2.95E-53**	0.98 (0.96, 1.00)	7.94E-02
0.92 (0.90, 0.93)	**3.52E-17**	1.02 (1.00, 1.05)	**3.56E-02**
1.14 (1.12, 1.17)	**9.15E-37**	1.00 (0.98, 1.02)	9.08E-01
1.20 (1.17, 1.23)	**1.78E-48**	1.07 (1.04, 1.09)	**3.32E-07**
0.95 (0.93, 0.97)	**2.29E-07**	1.03 (1.01, 1.05)	**5.75E-03**
0.90 (0.89, 0.92)	**1.39E-22**	1.02 (1.00, 1.04)	5.11E-02
1.09 (1.06, 1.11)	**1.02E-15**	1.05 (1.02, 1.07)	**7.57E-05**
0.95 (0.93, 0.97)	**3.11E-06**	0.95 (0.93, 0.97)	**1.23E-06**
0.86 (0.85, 0.88)	**2.24E-55**	0.99 (0.97, 1.01)	4.77E-01
0.82 (0.81, 0.84)	**7.68E-94**	0.97 (0.95, 0.99)	**1.19E-02**
0.81 (0.80, 0.83)	**1.28E-102**	0.97 (0.95, 0.99)	**4.40E-03**
0.85 (0.83, 0.87)	**1.59E-64**	0.98 (0.96, 1.00)	1.32E-01
1.21 (1.19, 1.23)	**2.31E-87**	1.02 (1.00, 1.04)	**3.15E-02**
0.78 (0.76, 0.79)	**2.38E-141**	0.93 (0.91, 0.95)	**4.48E-11**
0.83 (0.82, 0.85)	**9.61E-79**	0.97 (0.95, 0.99)	**1.92E-03**
0.89 (0.88, 0.91)	**4.30E-30**	1.00 (0.98, 1.02)	7.71E-01
0.78 (0.76, 0.79)	**6.10E-144**	0.93 (0.91, 0.95)	**8.16E-11**
1.23 (1.20, 1.25)	**3.87E-99**	1.05 (1.03, 1.07)	**2.58E-05**
1.30 (1.27, 1.32)	**6.92E-150**	1.12 (1.10, 1.15)	**3.02E-27**
0.79 (0.78, 0.80)	**1.50E-149**	0.94 (0.93, 0.96)	**4.06E-08**
0.78 (0.77, 0.79)	**2.99E-162**	0.94 (0.92, 0.96)	**4.39E-09**
0.90 (0.88, 0.91)	**1.99E-31**	0.98 (0.96, 1.00)	9.18E-02
1.24 (1.22, 1.26)	**5.09E-123**	1.04 (1.02, 1.06)	**1.60E-04**
1.19 (1.17, 1.21)	**1.51E-67**	1.10 (1.08, 1.13)	**4.98E-22**
0.78 (0.76, 0.79)	**7.34E-199**	0.91 (0.89, 0.92)	**5.40E-25**
0.76 (0.74, 0.77)	**6.81E-224**	0.89 (0.87, 0.91)	**7.78E-32**
0.90 (0.88, 0.92)	**3.28E-27**	0.98 (0.96, 1.00)	**4.92E-02**
1.27 (1.25, 1.29)	**1.04E-194**	1.09 (1.07, 1.11)	**8.81E-19**
0.98 (0.96, 1.00)	1.13E-01	1.01 (0.99, 1.03)	1.90E-01
0.90 (0.89, 0.90)	**3.77E-190**	0.93 (0.92, 0.94)	**3.33E-31**
0.92 (0.91, 0.93)	**1.59E-69**	0.94 (0.93, 0.95)	**2.80E-28**
0.82 (0.81, 0.84)	**2.20E-133**	0.95 (0.93, 0.97)	**1.71E-07**
1.16 (1.15, 1.16)	**1.34E-239**	1.09 (1.07, 1.10)	**1.46E-29**

0.93 (0.91, 0.95)	**6.71E-13**	0.98 (0.96, 1.00)	**4.41E-02**
0.90 (0.89, 0.90)	**5.15E-151**	0.94 (0.92, 0.95)	**1.44E-22**
1.01 (0.99, 1.03)	2.48E-01	0.97 (0.95, 0.99)	**3.15E-03**
0.83 (0.81, 0.84)	**5.44E-105**	0.94 (0.93, 0.96)	**2.64E-08**
1.14 (1.13, 1.15)	**1.30E-249**	1.09 (1.07, 1.10)	**1.00E-32**
1.04 (1.02, 1.06)	**2.37E-04**	1.05 (1.03, 1.07)	**5.24E-06**
0.86 (0.85, 0.87)	**3.75E-194**	0.92 (0.90, 0.93)	**6.78E-28**
0.96 (0.95, 0.98)	**2.48E-04**	0.96 (0.94, 0.98)	**1.35E-04**
0.84 (0.82, 0.85)	**1.39E-97**	0.94 (0.92, 0.96)	**4.34E-11**
1.15 (1.14, 1.17)	**1.38E-176**	1.07 (1.05, 1.09)	**3.07E-13**
0.99 (0.97, 1.01)	4.46E-01	1.06 (1.04, 1.09)	**1.30E-07**
0.92 (0.90, 0.94)	**1.49E-16**	0.92 (0.90, 0.94)	**2.32E-13**
0.84 (0.83, 0.85)	**5.23E-100**	0.92 (0.91, 0.94)	**3.37E-19**
1.08 (1.06, 1.10)	**1.96E-16**	0.99 (0.97, 1.01)	3.56E-01
1.09 (1.08, 1.11)	**2.39E-34**	1.00 (0.98, 1.02)	8.74E-01
1.19 (1.17, 1.21)	**2.29E-86**	1.03 (1.01, 1.05)	**1.16E-02**
0.84 (0.83, 0.86)	**1.34E-89**	0.97 (0.95, 0.99)	**2.45E-03**
0.84 (0.82, 0.85)	**5.02E-98**	0.96 (0.94, 0.98)	**4.43E-05**
0.92 (0.91, 0.94)	**1.01E-18**	1.02 (1.00, 1.04)	**3.11E-02**
1.11 (1.10, 1.12)	**5.19E-62**	1.03 (1.01, 1.05)	**6.73E-03**
1.23 (1.21, 1.26)	**1.30E-111**	1.09 (1.06, 1.11)	**4.97E-15**
0.82 (0.80, 0.83)	**4.19E-110**	0.93 (0.91, 0.94)	**2.08E-13**
0.83 (0.81, 0.84)	**1.25E-99**	0.93 (0.91, 0.95)	**6.39E-13**
0.86 (0.84, 0.87)	**2.24E-55**	0.95 (0.93, 0.97)	**1.21E-05**
1.13 (1.12, 1.14)	**5.78E-156**	1.07 (1.05, 1.09)	**3.13E-11**
1.13 (1.11, 1.15)	**1.42E-33**	1.10 (1.08, 1.12)	**1.78E-18**
0.80 (0.79, 0.82)	**4.90E-122**	0.90 (0.88, 0.92)	**5.40E-25**
0.82 (0.80, 0.83)	**6.61E-109**	0.90 (0.88, 0.91)	**8.58E-27**
0.95 (0.93, 0.97)	**3.39E-07**	1.01 (0.99, 1.03)	2.69E-01
1.22 (1.20, 1.24)	**2.89E-116**	1.08 (1.05, 1.10)	**4.61E-12**
1.08 (1.06, 1.11)	**3.30E-16**	0.97 (0.95, 0.99)	**8.70E-03**
0.88 (0.86, 0.89)	**8.43E-43**	0.97 (0.95, 0.99)	**1.41E-02**
0.93 (0.91, 0.95)	**7.79E-12**	1.04 (1.02, 1.06)	**9.21E-04**
0.85 (0.83, 0.86)	**1.45E-58**	0.93 (0.91, 0.95)	**5.48E-11**
0.99 (0.97, 1.01)	2.48E-01	0.98 (0.96, 1.00)	8.47E-02
0.86 (0.84, 0.87)	**1.98E-51**	0.92 (0.90, 0.94)	**3.57E-14**
0.91 (0.89, 0.93)	**1.29E-20**	0.96 (0.94, 0.98)	**1.55E-04**
0.84 (0.82, 0.86)	**4.86E-65**	0.93 (0.91, 0.95)	**4.78E-12**
0.87 (0.85, 0.89)	**7.21E-42**	0.94 (0.92, 0.96)	**3.33E-08**
1.11 (1.09, 1.13)	**6.22E-27**	1.01 (0.99, 1.03)	6.13E-01
1.09 (1.07, 1.11)	**3.74E-21**	1.00 (0.98, 1.02)	7.75E-01
1.13 (1.11, 1.15)	**6.45E-43**	1.04 (1.02, 1.06)	**6.41E-04**
1.14 (1.12, 1.16)	**1.32E-46**	1.04 (1.02, 1.06)	**7.77E-05**
0.93 (0.91, 0.95)	**2.54E-12**	0.96 (0.94, 0.98)	**1.06E-03**
1.16 (1.14, 1.19)	**8.85E-58**	1.03 (1.00, 1.05)	**2.72E-02**
0.91 (0.89, 0.93)	**2.56E-20**	0.96 (0.94, 0.98)	**6.49E-05**

0.89 (0.88, 0.91)	**1.63E-27**	0.97 (0.95, 0.99)	**8.49E-03**
0.90 (0.89, 0.92)	**3.66E-23**	0.96 (0.94, 0.98)	**1.24E-04**
0.90 (0.88, 0.92)	**1.28E-25**	0.96 (0.94, 0.98)	**2.22E-04**
0.91 (0.89, 0.93)	**9.09E-21**	0.95 (0.93, 0.97)	**6.80E-06**
0.98 (0.96, 1.00)	**4.06E-02**	0.98 (0.96, 1.00)	1.03E-01
1.03 (1.01, 1.05)	**4.65E-03**	0.99 (0.97, 1.01)	1.90E-01
0.81 (0.80, 0.83)	**4.11E-95**	0.88 (0.86, 0.90)	**1.76E-31**
0.91 (0.89, 0.93)	**4.11E-20**	0.90 (0.88, 0.92)	**9.92E-22**
0.87 (0.85, 0.88)	**2.04E-43**	0.94 (0.93, 0.96)	**1.06E-07**
0.97 (0.95, 0.99)	**2.07E-03**	0.91 (0.89, 0.93)	**2.19E-18**
0.89 (0.87, 0.90)	**8.18E-32**	0.94 (0.92, 0.96)	**2.99E-08**
0.89 (0.88, 0.91)	**2.79E-27**	0.92 (0.91, 0.94)	**7.45E-13**
1.11 (1.09, 1.13)	**2.60E-28**	1.02 (1.00, 1.04)	5.65E-02
1.07 (1.05, 1.09)	**2.98E-11**	1.01 (0.99, 1.03)	5.29E-01
0.89 (0.87, 0.90)	**3.91E-30**	0.90 (0.88, 0.92)	**2.35E-20**
0.76 (0.75, 0.77)	**8.38E-181**	0.93 (0.91, 0.95)	**1.05E-11**
0.95 (0.93, 0.97)	**1.34E-07**	0.89 (0.87, 0.91)	**3.46E-23**
0.80 (0.78, 0.81)	**1.86E-131**	0.94 (0.92, 0.96)	**2.19E-08**
0.79 (0.78, 0.80)	**2.92E-145**	0.91 (0.89, 0.92)	**1.28E-21**
1.26 (1.24, 1.28)	**3.72E-130**	1.09 (1.07, 1.12)	**1.45E-16**
1.17 (1.15, 1.19)	**5.28E-60**	1.07 (1.05, 1.10)	**2.07E-12**
1.13 (1.11, 1.14)	**8.41E-64**	1.06 (1.04, 1.08)	**4.84E-11**
1.06 (1.04, 1.08)	**1.97E-08**	1.03 (1.01, 1.05)	**1.72E-02**
1.09 (1.07, 1.11)	**3.32E-22**	1.04 (1.02, 1.06)	**6.82E-05**
1.02 (1.00, 1.04)	5.09E-02	0.98 (0.96, 1.00)	5.07E-02
0.98 (0.96, 0.99)	**1.43E-02**	0.95 (0.93, 0.96)	**1.06E-07**
0.90 (0.88, 0.92)	**3.69E-21**	0.95 (0.93, 0.97)	**8.51E-06**
1.03 (1.01, 1.05)	**1.63E-03**	0.99 (0.97, 1.01)	5.50E-01
1.00 (0.98, 1.02)	9.67E-01	0.97 (0.95, 0.99)	**1.68E-03**
1.02 (1.00, 1.04)	**3.38E-02**	0.97 (0.95, 0.99)	**4.87E-03**
1.04 (1.03, 1.05)	**6.75E-19**	1.03 (1.02, 1.05)	**3.62E-04**
1.09 (1.07, 1.11)	**6.86E-20**	1.02 (1.00, 1.04)	9.18E-02
1.05 (1.04, 1.06)	**7.61E-14**	1.04 (1.03, 1.05)	**1.98E-08**
1.08 (1.07, 1.09)	**2.01E-51**	1.05 (1.03, 1.06)	**3.70E-10**
1.07 (1.06, 1.08)	**3.35E-32**	1.05 (1.03, 1.07)	**4.20E-09**
1.09 (1.08, 1.10)	**3.04E-93**	1.03 (1.01, 1.04)	**1.86E-03**
0.82 (0.80, 0.84)	**2.66E-88**	0.90 (0.88, 0.92)	**1.12E-23**
1.28 (1.25, 1.30)	**3.20E-151**	1.14 (1.12, 1.16)	**1.27E-35**
0.89 (0.87, 0.91)	**1.77E-28**	0.98 (0.96, 1.00)	1.53E-01
1.02 (1.00, 1.04)	7.30E-02	0.97 (0.95, 0.99)	**1.28E-02**
0.88 (0.86, 0.90)	**7.08E-36**	0.94 (0.92, 0.96)	**4.90E-09**
0.85 (0.83, 0.86)	**1.23E-59**	0.93 (0.91, 0.94)	**2.08E-13**
0.87 (0.85, 0.89)	**4.18E-42**	0.93 (0.91, 0.94)	**2.32E-13**
0.85 (0.83, 0.87)	**8.49E-52**	0.95 (0.93, 0.97)	**2.08E-05**
0.77 (0.76, 0.79)	**1.76E-144**	0.90 (0.88, 0.92)	**1.14E-20**
1.01 (0.99, 1.03)	3.81E-01	1.07 (1.06, 1.09)	**7.40E-22**
1.02 (1.00, 1.04)	**3.64E-02**	0.98 (0.96, 1.00)	1.45E-01
0.83 (0.81, 0.84)	**2.34E-76**	0.92 (0.90, 0.94)	**1.22E-15**

0.89 (0.88, 0.91)	**6.99E-27**	0.98 (0.96, 1.00)	7.79E-02
1.03 (1.02, 1.05)	**5.19E-05**	1.02 (1.01, 1.04)	**1.13E-02**
1.06 (1.05, 1.07)	**1.66E-27**	1.04 (1.02, 1.06)	**4.56E-06**
0.86 (0.84, 0.88)	**5.71E-48**	0.92 (0.90, 0.94)	**5.89E-13**
1.03 (1.01, 1.05)	**1.78E-03**	0.99 (0.97, 1.01)	4.77E-01
0.90 (0.88, 0.92)	**1.07E-24**	0.95 (0.93, 0.97)	**2.59E-07**
0.87 (0.85, 0.89)	**1.63E-42**	0.93 (0.91, 0.95)	**1.42E-11**
0.91 (0.89, 0.93)	**3.49E-21**	0.96 (0.94, 0.98)	**7.39E-05**
1.05 (1.03, 1.07)	**4.88E-06**	0.99 (0.97, 1.01)	4.79E-01
0.86 (0.85, 0.88)	**2.74E-45**	0.93 (0.91, 0.95)	**8.83E-13**
0.92 (0.90, 0.94)	**2.68E-15**	0.98 (0.96, 1.00)	1.09E-01
0.92 (0.90, 0.94)	**9.87E-16**	0.95 (0.93, 0.97)	**5.86E-06**
1.06 (1.04, 1.08)	**2.52E-09**	0.99 (0.97, 1.01)	4.26E-01
0.87 (0.85, 0.89)	**7.45E-41**	0.93 (0.91, 0.95)	**2.75E-12**
0.90 (0.88, 0.92)	**4.83E-24**	0.97 (0.95, 0.99)	**1.90E-02**
0.96 (0.95, 0.98)	**3.42E-04**	0.98 (0.96, 1.00)	5.85E-02
0.86 (0.84, 0.88)	**3.59E-46**	0.96 (0.93, 0.98)	**1.76E-04**
1.07 (1.05, 1.10)	**3.87E-10**	1.04 (1.02, 1.07)	**5.35E-04**

			Othe
CKB			
Unadjusted HR (95% CI)	P value	Adjusted HR (95% CI)	P value
1.04 (0.91, 1.19)	7.60E-01	1.00 (0.87, 1.16)	9.99E-01
1.04 (0.91, 1.19)	7.52E-01	1.01 (0.88, 1.16)	9.99E-01
1.05 (0.92, 1.20)	7.27E-01	1.02 (0.88, 1.17)	9.63E-01
1.09 (0.96, 1.24)	4.18E-01	1.04 (0.91, 1.20)	8.15E-01
1.10 (0.97, 1.26)	3.48E-01	1.05 (0.91, 1.20)	7.95E-01
1.08 (0.94, 1.23)	5.31E-01	1.04 (0.9, 1.19)	8.42E-01
1.03 (0.90, 1.18)	7.92E-01	1.00 (0.87, 1.15)	9.99E-01
1.04 (0.91, 1.19)	7.60E-01	1.01 (0.87, 1.16)	9.99E-01
1.04 (0.91, 1.19)	7.60E-01	1.01 (0.88, 1.16)	9.99E-01
1.06 (0.93, 1.21)	6.62E-01	1.02 (0.89, 1.18)	9.08E-01
1.06 (0.92, 1.21)	6.93E-01	1.02 (0.88, 1.17)	9.63E-01
1.05 (0.92, 1.20)	7.27E-01	1.01 (0.88, 1.16)	9.99E-01
1.07 (0.93, 1.22)	6.31E-01	1.03 (0.89, 1.18)	8.98E-01
1.04 (0.91, 1.19)	7.83E-01	1.00 (0.87, 1.16)	9.99E-01
1.03 (0.90, 1.18)	7.92E-01	1.00 (0.87, 1.15)	9.99E-01
1.04 (0.91, 1.18)	7.86E-01	1.00 (0.87, 1.15)	9.99E-01
1.05 (0.92, 1.20)	7.45E-01	1.01 (0.88, 1.16)	9.99E-01
1.05 (0.91, 1.19)	7.51E-01	1.00 (0.87, 1.15)	9.99E-01
1.03 (0.90, 1.18)	7.92E-01	0.98 (0.85, 1.13)	9.36E-01
1.05 (0.92, 1.20)	6.98E-01	1.02 (0.89, 1.17)	9.53E-01
1.03 (0.90, 1.18)	8.17E-01	1.00 (0.87, 1.15)	9.99E-01
1.00 (0.88, 1.15)	9.67E-01	0.97 (0.84, 1.11)	8.53E-01
1.01 (0.88, 1.15)	9.52E-01	0.97 (0.84, 1.11)	8.59E-01
1.01 (0.88, 1.16)	9.23E-01	0.97 (0.84, 1.11)	8.53E-01
1.03 (0.90, 1.18)	7.95E-01	0.97 (0.85, 1.12)	8.98E-01
1.03 (0.90, 1.18)	7.92E-01	0.96 (0.84, 1.11)	8.50E-01
1.03 (0.90, 1.18)	8.17E-01	0.99 (0.86, 1.13)	9.66E-01
0.99 (0.87, 1.14)	9.55E-01	0.97 (0.84, 1.11)	8.53E-01
1.01 (0.88, 1.16)	9.23E-01	0.95 (0.83, 1.09)	7.91E-01
1.01 (0.88, 1.16)	9.23E-01	0.95 (0.83, 1.09)	7.74E-01
1.01 (0.89, 1.16)	9.23E-01	0.94 (0.82, 1.08)	7.39E-01
0.98 (0.85, 1.13)	8.90E-01	0.94 (0.82, 1.08)	6.98E-01
0.96 (0.83, 1.10)	7.60E-01	0.93 (0.81, 1.07)	6.42E-01
1.02 (0.89, 1.17)	8.76E-01	0.96 (0.84, 1.10)	8.38E-01
1.03 (0.90, 1.18)	8.17E-01	0.97 (0.84, 1.11)	8.53E-01
1.10 (0.97, 1.26)	3.48E-01	1.00 (0.87, 1.15)	9.99E-01
1.09 (0.96, 1.25)	4.14E-01	1.00 (0.87, 1.14)	9.99E-01
1.09 (0.95, 1.24)	4.55E-01	0.98 (0.86, 1.13)	9.60E-01
1.05 (0.92, 1.20)	7.38E-01	1.00 (0.88, 1.15)	9.99E-01
1.05 (0.92, 1.20)	7.35E-01	1.01 (0.89, 1.15)	9.91E-01
1.05 (0.91, 1.20)	7.51E-01	1.00 (0.87, 1.14)	9.99E-01

1.12 (0.99, 1.28)	2.01E-01	1.01 (0.88, 1.15)	9.99E-01
1.10 (0.97, 1.26)	3.50E-01	0.99 (0.87, 1.14)	9.99E-01
1.09 (0.95, 1.24)	4.55E-01	0.99 (0.86, 1.13)	9.66E-01
1.06 (0.92, 1.21)	6.91E-01	0.96 (0.84, 1.11)	8.38E-01
1.04 (0.91, 1.19)	7.83E-01	0.96 (0.84, 1.10)	8.38E-01
1.03 (0.90, 1.18)	8.14E-01	0.95 (0.83, 1.09)	7.95E-01
1.05 (0.92, 1.20)	7.27E-01	0.98 (0.86, 1.13)	9.53E-01
1.28 (1.15, 1.42)	**9.52E-05**	1.13 (1.00, 1.28)	2.19E-01
1.05 (0.92, 1.20)	7.45E-01	0.95 (0.82, 1.09)	7.52E-01
1.04 (0.91, 1.19)	7.83E-01	0.94 (0.82, 1.08)	7.02E-01
1.01 (0.88, 1.16)	9.23E-01	0.92 (0.80, 1.06)	5.20E-01
1.01 (0.88, 1.16)	9.51E-01	0.93 (0.81, 1.07)	5.81E-01
1.00 (0.87, 1.15)	9.74E-01	0.92 (0.80, 1.06)	5.43E-01
1.03 (0.90, 1.17)	8.47E-01	0.95 (0.82, 1.09)	7.58E-01
1.29 (1.17, 1.44)	**3.22E-05**	1.15 (1.02, 1.30)	1.52E-01
0.99 (0.87, 1.14)	9.52E-01	0.90 (0.78, 1.03)	3.72E-01
0.98 (0.86, 1.13)	9.20E-01	0.89 (0.77, 1.03)	3.36E-01
0.98 (0.85, 1.12)	8.77E-01	0.87 (0.75, 1.00)	2.13E-01
0.96 (0.84, 1.10)	7.60E-01	0.89 (0.77, 1.02)	3.07E-01
0.96 (0.84, 1.10)	7.60E-01	0.89 (0.77, 1.03)	3.14E-01
0.96 (0.83, 1.10)	7.55E-01	0.87 (0.76, 1.00)	2.19E-01
1.28 (1.14, 1.43)	**3.13E-04**	1.11 (0.98, 1.27)	3.13E-01
0.96 (0.83, 1.10)	7.60E-01	0.88 (0.76, 1.01)	2.54E-01
0.95 (0.83, 1.09)	7.35E-01	0.87 (0.76, 1.01)	2.30E-01
0.94 (0.82, 1.07)	6.48E-01	0.85 (0.74, 0.98)	1.30E-01
0.94 (0.82, 1.08)	6.62E-01	0.88 (0.76, 1.01)	2.54E-01
0.94 (0.82, 1.08)	6.86E-01	0.88 (0.77, 1.02)	2.75E-01
0.92 (0.81, 1.06)	5.14E-01	0.86 (0.75, 0.99)	1.52E-01
1.15 (1.02, 1.31)	9.48E-02	1.03 (0.89, 1.18)	8.98E-01
1.16 (1.02, 1.33)	7.84E-02	1.17 (1.04, 1.33)	9.46E-02
1.16 (1.01, 1.32)	9.96E-02	1.17 (1.03, 1.32)	1.09E-01
1.19 (1.05, 1.36)	**3.79E-02**	1.19 (1.05, 1.35)	6.56E-02
1.08 (0.94, 1.24)	5.14E-01	1.12 (0.99, 1.27)	2.56E-01
1.06 (0.93, 1.22)	6.62E-01	1.11 (0.98, 1.26)	3.07E-01
1.12 (0.98, 1.28)	2.32E-01	1.14 (1.01, 1.29)	1.75E-01
1.27 (1.13, 1.42)	**8.91E-04**	1.22 (1.08, 1.38)	**2.69E-02**
1.18 (1.03, 1.34)	6.30E-02	1.20 (1.06, 1.37)	6.40E-02
1.17 (1.03, 1.33)	7.42E-02	1.20 (1.05, 1.36)	6.56E-02
1.14 (1.00, 1.30)	1.71E-01	1.17 (1.03, 1.34)	1.14E-01
1.17 (1.02, 1.33)	7.87E-02	1.20 (1.05, 1.36)	6.56E-02
1.17 (1.03, 1.34)	6.80E-02	1.20 (1.06, 1.37)	6.40E-02
1.14 (1.00, 1.30)	1.66E-01	1.18 (1.04, 1.34)	9.35E-02
1.40 (1.25, 1.57)	**5.87E-07**	1.32 (1.16, 1.49)	**8.73E-04**
0.88 (0.76, 1.01)	2.14E-01	0.91 (0.79, 1.05)	4.81E-01
0.87 (0.76, 1.01)	1.71E-01	0.91 (0.79, 1.05)	4.68E-01
0.94 (0.82, 1.08)	6.72E-01	0.96 (0.83, 1.10)	8.13E-01
0.81 (0.70, 0.93)	**1.84E-02**	0.88 (0.77, 1.01)	2.56E-01
0.79 (0.69, 0.91)	**7.33E-03**	0.87 (0.76, 1.00)	1.96E-01

0.89 (0.77, 1.02)	2.67E-01	0.94 (0.81, 1.08)	6.81E-01
1.08 (0.95, 1.23)	5.09E-01	0.97 (0.84, 1.11)	8.53E-01
0.83 (0.73, 0.94)	**1.67E-02**	0.80 (0.71, 0.91)	**1.22E-02**
0.82 (0.73, 0.92)	**7.99E-03**	0.80 (0.70, 0.90)	**8.93E-03**
0.85 (0.74, 0.98)	7.70E-02	0.86 (0.75, 0.99)	1.52E-01
0.84 (0.74, 0.95)	**3.36E-02**	0.81 (0.71, 0.93)	**3.62E-02**
0.87 (0.76, 0.99)	9.48E-02	0.84 (0.74, 0.96)	9.31E-02
0.82 (0.72, 0.94)	**2.48E-02**	0.84 (0.73, 0.96)	1.00E-01
1.13 (1.00, 1.29)	1.68E-01	1.00 (0.87, 1.14)	9.99E-01
1.08 (0.92, 1.26)	6.40E-01	1.07 (0.92, 1.25)	6.95E-01
1.19 (1.07, 1.31)	**6.25E-03**	1.15 (1.02, 1.29)	1.43E-01
1.17 (1.04, 1.31)	**4.12E-02**	1.11 (0.98, 1.26)	3.08E-01
1.15 (1.03, 1.28)	**4.61E-02**	1.15 (1.01, 1.31)	1.56E-01
0.82 (0.73, 0.92)	**6.07E-03**	0.86 (0.75, 0.98)	1.30E-01
1.12 (0.96, 1.31)	3.74E-01	1.07 (0.91, 1.24)	7.39E-01
1.13 (1.02, 1.26)	7.35E-02	1.08 (0.96, 1.21)	4.52E-01
1.05 (0.92, 1.21)	7.10E-01	1.03 (0.90, 1.17)	8.97E-01
1.18 (1.05, 1.33)	**3.36E-02**	1.12 (0.98, 1.27)	2.71E-01
0.83 (0.72, 0.96)	5.18E-02	0.90 (0.78, 1.04)	3.77E-01
1.29 (1.09, 1.52)	**1.67E-02**	1.11 (0.95, 1.30)	4.70E-01
1.16 (1.00, 1.36)	1.68E-01	1.04 (0.89, 1.21)	8.50E-01
1.05 (0.92, 1.21)	7.22E-01	0.98 (0.85, 1.13)	9.22E-01
1.13 (0.98, 1.32)	2.63E-01	1.07 (0.92, 1.24)	6.95E-01
0.83 (0.71, 0.97)	7.52E-02	0.94 (0.81, 1.10)	7.71E-01
1.09 (0.96, 1.25)	4.18E-01	0.96 (0.84, 1.10)	8.15E-01
1.19 (1.04, 1.37)	**4.89E-02**	1.08 (0.94, 1.25)	5.87E-01
1.12 (0.98, 1.29)	2.32E-01	1.04 (0.90, 1.20)	8.38E-01
1.19 (1.03, 1.39)	7.70E-02	1.10 (0.95, 1.28)	4.53E-01
0.84 (0.73, 0.96)	**4.89E-02**	0.94 (0.81, 1.08)	6.98E-01
1.03 (0.90, 1.18)	7.92E-01	1.00 (0.88, 1.14)	9.99E-01
0.93 (0.81, 1.06)	5.42E-01	0.98 (0.86, 1.12)	9.22E-01
0.92 (0.8, 1.05)	4.28E-01	0.97 (0.85, 1.10)	8.52E-01
1.06 (0.92, 1.21)	6.91E-01	1.08 (0.94, 1.23)	5.46E-01
1.07 (0.93, 1.22)	6.31E-01	1.02 (0.89, 1.16)	9.22E-01
1.00 (0.88, 1.15)	9.63E-01	0.95 (0.83, 1.08)	7.69E-01
0.92 (0.81, 1.04)	4.14E-01	1.03 (0.90, 1.17)	8.98E-01
0.93 (0.82, 1.06)	5.50E-01	1.03 (0.91, 1.18)	8.50E-01
0.92 (0.81, 1.04)	4.09E-01	0.99 (0.87, 1.13)	9.99E-01
1.07 (0.94, 1.21)	5.88E-01	1.00 (0.88, 1.14)	9.99E-01
0.88 (0.77, 1.00)	1.65E-01	0.92 (0.80, 1.05)	4.87E-01
0.84 (0.75, 0.93)	**7.99E-03**	0.90 (0.81, 1.01)	2.56E-01
0.81 (0.72, 0.91)	**3.06E-03**	0.87 (0.77, 0.98)	1.30E-01
0.95 (0.84, 1.08)	7.10E-01	0.99 (0.87, 1.13)	9.99E-01
1.23 (1.10, 1.36)	**1.90E-03**	1.13 (1.01, 1.27)	1.52E-01
0.98 (0.85, 1.13)	8.77E-01	1.05 (0.92, 1.20)	7.58E-01
0.87 (0.78, 0.97)	**4.25E-02**	0.88 (0.79, 0.98)	1.30E-01
0.86 (0.77, 0.96)	**4.16E-02**	0.86 (0.77, 0.96)	6.56E-02
0.97 (0.85, 1.11)	8.42E-01	1.05 (0.91, 1.21)	7.95E-01

1.23 (1.12, 1.36)	**1.70E-04**	1.18 (1.06, 1.31)	**3.51E-02**
1.05 (0.93, 1.20)	6.93E-01	1.07 (0.95, 1.22)	5.54E-01
0.87 (0.78, 0.99)	9.48E-02	0.89 (0.79, 1.00)	1.98E-01
0.9 (0.79, 1.01)	2.18E-01	0.89 (0.78, 1.00)	2.19E-01
1.02 (0.89, 1.16)	9.20E-01	1.10 (0.96, 1.26)	4.33E-01
1.27 (1.15, 1.41)	**9.52E-05**	1.21 (1.08, 1.36)	**2.10E-02**
1.06 (0.93, 1.21)	6.62E-01	1.08 (0.95, 1.24)	4.90E-01
0.9 (0.79, 1.02)	2.32E-01	0.90 (0.79, 1.02)	3.07E-01
0.91 (0.8, 1.03)	3.48E-01	0.90 (0.79, 1.02)	3.14E-01
1.00 (0.87, 1.15)	9.68E-01	1.08 (0.94, 1.24)	5.81E-01
1.19 (1.06, 1.34)	**1.68E-02**	1.14 (1.01, 1.29)	1.52E-01
1.18 (1.03, 1.35)	7.11E-02	1.11 (0.97, 1.27)	3.60E-01
0.79 (0.70, 0.89)	**1.90E-03**	0.86 (0.76, 0.98)	1.43E-01
0.79 (0.69, 0.90)	**2.91E-03**	0.86 (0.75, 0.98)	1.43E-01
0.85 (0.76, 0.96)	**3.33E-02**	0.92 (0.82, 1.03)	4.02E-01
1.16 (1.04, 1.29)	**4.43E-02**	1.08 (0.96, 1.21)	4.86E-01
0.75 (0.65, 0.87)	**1.12E-03**	0.75 (0.65, 0.87)	**5.93E-03**
1.05 (0.91, 1.21)	7.46E-01	1.14 (0.99, 1.31)	2.56E-01
1.11 (0.96, 1.28)	3.58E-01	1.17 (1.02, 1.35)	1.52E-01
0.96 (0.85, 1.10)	7.83E-01	1.06 (0.93, 1.22)	6.95E-01
1.30 (1.17, 1.43)	**1.44E-05**	1.18 (1.05, 1.32)	6.60E-02
1.29 (1.20, 1.39)	**4.61E-09**	1.23 (1.12, 1.35)	**8.73E-04**
0.78 (0.71, 0.85)	**1.99E-06**	0.85 (0.76, 0.95)	5.96E-02
0.78 (0.71, 0.85)	**3.50E-07**	0.84 (0.76, 0.94)	**2.69E-02**
0.90 (0.79, 1.03)	3.15E-01	0.97 (0.85, 1.11)	8.50E-01
1.19 (1.05, 1.34)	**3.36E-02**	1.05 (0.92, 1.20)	7.71E-01
0.99 (0.87, 1.14)	9.52E-01	1.05 (0.91, 1.20)	7.95E-01
0.95 (0.83, 1.08)	6.86E-01	0.94 (0.82, 1.07)	6.76E-01
0.96 (0.84, 1.09)	7.46E-01	0.94 (0.82, 1.07)	6.95E-01
0.94 (0.82, 1.08)	6.91E-01	1.03 (0.90, 1.18)	8.67E-01
1.22 (1.08, 1.38)	**7.99E-03**	1.09 (0.96, 1.25)	4.53E-01
0.97 (0.85, 1.11)	8.17E-01	0.98 (0.85, 1.12)	9.08E-01
1.35 (1.20, 1.53)	**3.52E-05**	1.30 (1.14, 1.48)	**4.71E-03**
1.14 (1.00, 1.31)	1.65E-01	1.19 (1.04, 1.35)	8.75E-02
1.01 (0.88, 1.16)	9.47E-01	0.96 (0.84, 1.10)	8.25E-01
1.03 (0.90, 1.18)	7.92E-01	0.98 (0.85, 1.12)	9.22E-01
1.04 (0.91, 1.19)	7.55E-01	0.97 (0.85, 1.11)	8.72E-01
0.99 (0.86, 1.13)	9.23E-01	0.92 (0.80, 1.06)	5.43E-01
0.99 (0.86, 1.14)	9.47E-01	1.05 (0.92, 1.20)	7.95E-01
0.99 (0.86, 1.13)	9.23E-01	1.04 (0.91, 1.19)	8.20E-01
1.03 (0.90, 1.18)	8.17E-01	1.08 (0.95, 1.23)	5.43E-01
1.09 (0.95, 1.24)	4.56E-01	0.95 (0.82, 1.10)	7.74E-01
1.07 (0.94, 1.21)	6.31E-01	0.95 (0.83, 1.10)	8.13E-01
1.07 (0.94, 1.22)	5.46E-01	1.01 (0.88, 1.16)	9.66E-01
1.02 (0.89, 1.17)	8.77E-01	0.98 (0.86, 1.13)	9.60E-01
1.29 (1.15, 1.43)	**9.52E-05**	1.14 (1.01, 1.29)	1.75E-01
1.27 (1.12, 1.43)	**1.18E-03**	1.15 (1.01, 1.31)	1.75E-01
1.05 (0.92, 1.21)	7.27E-01	1.00 (0.87, 1.15)	9.99E-01

0.96 (0.84, 1.11)	7.83E-01	0.96 (0.83, 1.10)	7.95E-01
1.09 (0.95, 1.24)	4.81E-01	0.96 (0.84, 1.11)	8.40E-01
1.09 (0.96, 1.25)	4.18E-01	0.95 (0.82, 1.10)	7.71E-01
1.13 (0.98, 1.29)	2.32E-01	1.02 (0.89, 1.18)	9.22E-01
0.99 (0.86, 1.13)	9.23E-01	1.03 (0.90, 1.18)	8.50E-01
1.03 (0.9, 1.17)	8.47E-01	0.95 (0.82, 1.09)	7.52E-01
1.05 (0.92, 1.21)	7.17E-01	0.95 (0.83, 1.09)	7.58E-01
1.02 (0.89, 1.17)	8.90E-01	0.94 (0.81, 1.09)	7.18E-01
0.88 (0.77, 1.00)	1.68E-01	0.79 (0.69, 0.90)	**9.18E-03**
0.77 (0.67, 0.89)	**2.91E-03**	0.89 (0.77, 1.02)	2.68E-01
1.01 (0.88, 1.16)	9.51E-01	0.92 (0.80, 1.07)	5.70E-01
0.82 (0.70, 0.95)	**4.61E-02**	0.86 (0.74, 1.00)	1.92E-01
1.02 (0.89, 1.17)	8.67E-01	0.91 (0.78, 1.06)	4.89E-01
0.99 (0.86, 1.13)	9.23E-01	0.89 (0.77, 1.04)	3.77E-01
1.02 (0.89, 1.17)	8.67E-01	0.96 (0.84, 1.11)	8.47E-01
1.04 (0.91, 1.19)	7.60E-01	0.97 (0.84, 1.11)	8.50E-01
0.77 (0.67, 0.87)	**7.37E-04**	0.94 (0.82, 1.07)	6.77E-01
0.93 (0.81, 1.06)	5.14E-01	0.90 (0.79, 1.04)	3.77E-01
0.73 (0.64, 0.84)	**1.42E-04**	0.87 (0.76, 0.99)	1.75E-01
0.96 (0.84, 1.10)	7.83E-01	0.90 (0.78, 1.03)	3.54E-01
0.88 (0.77, 1.01)	1.87E-01	0.85 (0.74, 0.98)	1.43E-01
1.06 (0.93, 1.22)	6.62E-01	1.08 (0.94, 1.24)	5.46E-01
1.14 (1.00, 1.31)	1.71E-01	1.16 (1.01, 1.33)	1.70E-01
1.33 (1.23, 1.43)	**5.14E-11**	1.25 (1.15, 1.36)	**4.30E-05**
0.91 (0.79, 1.05)	4.14E-01	0.91 (0.78, 1.05)	4.63E-01
1.35 (1.19, 1.54)	**9.52E-05**	1.08 (0.94, 1.25)	5.54E-01
1.00 (0.88, 1.15)	9.63E-01	0.96 (0.84, 1.10)	8.20E-01
0.99 (0.86, 1.13)	9.23E-01	0.86 (0.75, 0.99)	1.75E-01
0.84 (0.73, 0.96)	5.18E-02	0.93 (0.81, 1.08)	6.68E-01
0.96 (0.84, 1.10)	7.60E-01	0.92 (0.80, 1.06)	5.46E-01
0.91 (0.79, 1.05)	4.14E-01	0.90 (0.78, 1.04)	3.77E-01
0.99 (0.87, 1.14)	9.56E-01	0.90 (0.78, 1.04)	4.01E-01
1.16 (1.08, 1.25)	**9.67E-04**	1.15 (1.05, 1.25)	**3.62E-02**
1.27 (1.12, 1.44)	**2.05E-03**	1.18 (1.04, 1.35)	9.46E-02
1.02 (0.90, 1.17)	8.60E-01	0.99 (0.85, 1.14)	9.66E-01
1.09 (0.97, 1.22)	3.48E-01	1.02 (0.89, 1.18)	9.08E-01
1.15 (1.07, 1.24)	**1.90E-03**	1.11 (1.01, 1.22)	1.74E-01
1.10 (1.06, 1.13)	**9.72E-07**	1.08 (1.03, 1.12)	**8.93E-03**
0.80 (0.71, 0.90)	**1.70E-03**	0.83 (0.74, 0.94)	**3.81E-02**
0.91 (0.79, 1.05)	4.36E-01	1.00 (0.87, 1.15)	9.99E-01

ːr mortality			
UKB			
**Unadjusted HR (95% CI)**	**P value**	**Adjusted HR (95% CI)**	**P value**
1.08 (1.06, 1.10)	**8.52E-13**	0.93 (0.90, 0.95)	**3.96E-10**
1.07 (1.05, 1.10)	**7.04E-11**	0.93 (0.90, 0.95)	**3.17E-10**
1.09 (1.07, 1.11)	**1.21E-14**	0.93 (0.91, 0.95)	**1.37E-09**
1.06 (1.03, 1.08)	**5.86E-07**	0.92 (0.89, 0.94)	**2.12E-12**
1.04 (1.02, 1.07)	**2.17E-04**	0.91 (0.89, 0.93)	**1.90E-13**
1.07 (1.05, 1.10)	**8.19E-11**	0.92 (0.90, 0.94)	**6.94E-11**
1.07 (1.05, 1.10)	**3.25E-11**	0.93 (0.91, 0.95)	**1.68E-09**
1.02 (1.00, 1.04)	1.26E-01	0.87 (0.85, 0.89)	**7.76E-28**
1.01 (0.99, 1.04)	3.00E-01	0.87 (0.85, 0.89)	**1.69E-28**
1.01 (0.98, 1.03)	5.61E-01	0.87 (0.85, 0.89)	**4.34E-27**
0.94 (0.92, 0.96)	**1.20E-07**	0.85 (0.83, 0.87)	**5.40E-38**
0.89 (0.87, 0.91)	**3.22E-21**	0.83 (0.81, 0.86)	**6.27E-45**
0.99 (0.97, 1.01)	3.66E-01	0.87 (0.84, 0.89)	**2.97E-29**
1.04 (1.02, 1.06)	**8.22E-04**	0.88 (0.86, 0.90)	**1.10E-24**
0.98 (0.96, 1.00)	6.28E-02	0.85 (0.83, 0.87)	**2.85E-37**
0.96 (0.94, 0.98)	**3.10E-04**	0.84 (0.81, 0.86)	**1.91E-43**
0.99 (0.96, 1.01)	2.80E-01	0.85 (0.83, 0.87)	**1.25E-35**
0.92 (0.90, 0.95)	**3.85E-11**	0.83 (0.81, 0.85)	**3.29E-45**
0.89 (0.87, 0.91)	**7.97E-24**	0.83 (0.81, 0.85)	**5.33E-46**
0.96 (0.94, 0.99)	**1.34E-03**	0.84 (0.82, 0.86)	**3.76E-42**
0.96 (0.94, 0.99)	**2.29E-03**	0.84 (0.81, 0.86)	**2.87E-43**
0.79 (0.77, 0.81)	**1.29E-77**	0.82 (0.80, 0.84)	**1.06E-52**
0.82 (0.80, 0.84)	**3.58E-59**	0.82 (0.80, 0.84)	**3.12E-57**
0.79 (0.77, 0.81)	**3.04E-83**	0.83 (0.81, 0.85)	**2.86E-49**
0.71 (0.70, 0.73)	**1.18E-156**	0.85 (0.83, 0.87)	**4.78E-38**
0.69 (0.68, 0.71)	**9.84E-186**	0.87 (0.85, 0.89)	**1.80E-28**
0.76 (0.74, 0.78)	**1.13E-103**	0.83 (0.81, 0.86)	**1.28E-46**
0.93 (0.91, 0.95)	**1.92E-09**	0.82 (0.80, 0.85)	**8.36E-50**
0.94 (0.92, 0.96)	**1.32E-07**	0.87 (0.84, 0.89)	**2.71E-30**
0.92 (0.90, 0.94)	**4.28E-13**	0.86 (0.84, 0.88)	**3.76E-33**
0.83 (0.81, 0.85)	**1.22E-51**	0.85 (0.83, 0.87)	**1.65E-39**
0.83 (0.81, 0.85)	**1.26E-51**	0.86 (0.84, 0.88)	**2.09E-32**
0.87 (0.85, 0.89)	**5.19E-32**	0.87 (0.85, 0.89)	**1.52E-27**
0.77 (0.75, 0.79)	**8.08E-95**	0.85 (0.83, 0.87)	**2.12E-40**
1.05 (1.03, 1.07)	**1.44E-05**	0.89 (0.87, 0.91)	**5.70E-20**
0.91 (0.89, 0.93)	**5.16E-15**	0.97 (0.95, 1.00)	**2.54E-02**
0.92 (0.90, 0.94)	**1.54E-11**	0.98 (0.96, 1.00)	1.13E-01
0.97 (0.95, 0.99)	**8.33E-03**	1.00 (0.97, 1.02)	8.55E-01
0.82 (0.80, 0.84)	**1.89E-59**	0.97 (0.95, 0.99)	**1.02E-02**
0.79 (0.77, 0.81)	**9.56E-82**	0.97 (0.94, 0.99)	**4.52E-03**
0.90 (0.88, 0.92)	**2.50E-18**	0.98 (0.95, 1.00)	6.56E-02

1.14 (1.12, 1.17)	**4.60E-35**	0.99 (0.97, 1.02)	6.26E-01
0.74 (0.72, 0.76)	**2.63E-130**	0.90 (0.88, 0.92)	**4.09E-17**
0.72 (0.71, 0.74)	**1.07E-151**	0.92 (0.89, 0.94)	**1.16E-12**
0.74 (0.72, 0.76)	**7.58E-130**	0.93 (0.91, 0.95)	**2.18E-09**
0.70 (0.68, 0.71)	**1.08E-189**	0.90 (0.88, 0.93)	**6.90E-16**
0.69 (0.67, 0.71)	**7.62E-200**	0.90 (0.88, 0.92)	**1.35E-17**
0.72 (0.71, 0.74)	**2.57E-154**	0.92 (0.90, 0.94)	**5.04E-11**
1.14 (1.11, 1.16)	**1.04E-32**	1.03 (1.01, 1.05)	**1.22E-02**
0.73 (0.71, 0.74)	**5.68E-141**	0.86 (0.84, 0.88)	**1.21E-35**
0.69 (0.68, 0.71)	**5.83E-190**	0.85 (0.83, 0.87)	**6.94E-41**
0.69 (0.67, 0.70)	**1.76E-201**	0.85 (0.83, 0.87)	**7.48E-42**
0.68 (0.66, 0.69)	**4.95E-216**	0.84 (0.82, 0.86)	**1.13E-44**
0.68 (0.67, 0.70)	**5.86E-206**	0.83 (0.81, 0.85)	**2.33E-48**
0.67 (0.65, 0.69)	**5.63E-232**	0.86 (0.84, 0.88)	**2.14E-33**
1.12 (1.10, 1.15)	**3.60E-27**	1.02 (0.99, 1.04)	1.80E-01
0.75 (0.73, 0.77)	**1.79E-115**	0.83 (0.81, 0.85)	**5.32E-51**
0.72 (0.71, 0.74)	**8.74E-147**	0.81 (0.79, 0.83)	**1.79E-64**
0.72 (0.70, 0.74)	**4.22E-156**	0.81 (0.79, 0.83)	**3.14E-65**
0.71 (0.69, 0.73)	**1.10E-167**	0.80 (0.78, 0.82)	**7.09E-69**
0.73 (0.71, 0.75)	**2.53E-140**	0.80 (0.78, 0.82)	**2.45E-69**
0.67 (0.66, 0.69)	**1.53E-224**	0.82 (0.80, 0.84)	**1.58E-58**
1.10 (1.08, 1.12)	**7.76E-19**	0.97 (0.95, 0.99)	**1.27E-02**
0.77 (0.75, 0.79)	**4.51E-96**	0.85 (0.83, 0.87)	**1.73E-39**
0.73 (0.72, 0.75)	**1.09E-135**	0.83 (0.81, 0.85)	**9.10E-52**
0.75 (0.73, 0.77)	**7.36E-123**	0.86 (0.84, 0.88)	**4.37E-38**
0.71 (0.69, 0.73)	**6.18E-167**	0.82 (0.80, 0.84)	**2.29E-59**
0.73 (0.71, 0.75)	**5.28E-137**	0.82 (0.80, 0.84)	**1.58E-58**
0.69 (0.67, 0.70)	**2.16E-216**	0.84 (0.82, 0.86)	**1.89E-49**
1.08 (1.05, 1.10)	**3.17E-11**	0.94 (0.92, 0.96)	**2.09E-07**
0.94 (0.92, 0.96)	**7.33E-07**	1.20 (1.17, 1.23)	**2.16E-51**
0.96 (0.94, 0.98)	**6.47E-04**	1.23 (1.20, 1.25)	**8.48E-63**
0.98 (0.96, 1.00)	1.10E-01	1.24 (1.21, 1.27)	**5.81E-71**
0.92 (0.90, 0.95)	**1.45E-10**	1.20 (1.17, 1.23)	**5.32E-51**
0.91 (0.89, 0.93)	**2.34E-14**	1.19 (1.17, 1.22)	**5.53E-46**
0.98 (0.96, 1.01)	1.38E-01	1.22 (1.19, 1.25)	**5.58E-66**
1.09 (1.07, 1.12)	**2.11E-16**	1.05 (1.02, 1.07)	**7.86E-05**
0.91 (0.89, 0.93)	**4.26E-15**	1.19 (1.16, 1.22)	**4.90E-39**
0.92 (0.90, 0.94)	**2.92E-11**	1.19 (1.16, 1.22)	**1.49E-42**
0.94 (0.91, 0.96)	**6.95E-08**	1.20 (1.17, 1.23)	**4.29E-43**
0.90 (0.88, 0.92)	**1.21E-17**	1.19 (1.16, 1.22)	**2.88E-39**
0.89 (0.87, 0.92)	**1.26E-19**	1.18 (1.15, 1.21)	**4.17E-37**
0.92 (0.90, 0.94)	**2.37E-11**	1.20 (1.17, 1.23)	**2.98E-46**
1.10 (1.08, 1.12)	**8.37E-18**	1.10 (1.07, 1.12)	**1.70E-15**
0.86 (0.84, 0.88)	**1.48E-36**	1.02 (0.99, 1.04)	1.82E-01
0.88 (0.86, 0.90)	**1.24E-27**	1.02 (0.99, 1.04)	2.34E-01
0.90 (0.88, 0.92)	**6.93E-19**	1.01 (0.99, 1.04)	2.69E-01
0.84 (0.82, 0.86)	**4.91E-45**	1.02 (1.00, 1.05)	1.17E-01
0.84 (0.82, 0.86)	**1.63E-46**	1.02 (0.99, 1.04)	1.88E-01

0.85 (0.83, 0.88)	**1.58E-37**	1.04 (1.01, 1.06)	**1.04E-02**
1.10 (1.08, 1.12)	**7.44E-18**	0.98 (0.96, 1.00)	1.16E-01
0.74 (0.73, 0.76)	**8.40E-129**	0.80 (0.78, 0.82)	**2.45E-69**
0.82 (0.80, 0.84)	**1.49E-59**	0.84 (0.82, 0.86)	**8.26E-43**
0.85 (0.83, 0.87)	**1.75E-39**	0.87 (0.85, 0.89)	**6.16E-29**
0.74 (0.72, 0.75)	**3.61E-137**	0.81 (0.79, 0.83)	**8.24E-62**
0.73 (0.71, 0.74)	**1.85E-151**	0.80 (0.78, 0.82)	**2.32E-71**
0.81 (0.79, 0.83)	**2.64E-69**	0.88 (0.86, 0.90)	**8.06E-26**
1.12 (1.09, 1.14)	**1.29E-22**	0.92 (0.90, 0.94)	**2.85E-11**
1.19 (1.16, 1.22)	**1.10E-42**	1.02 (0.99, 1.05)	1.64E-01
0.94 (0.92, 0.97)	**4.73E-06**	1.04 (1.01, 1.07)	**2.17E-03**
0.93 (0.91, 0.96)	**2.82E-07**	1.03 (1.01, 1.06)	**1.69E-02**
0.97 (0.95, 1.00)	**3.14E-02**	1.05 (1.02, 1.07)	**2.73E-04**
1.00 (0.98, 1.02)	9.65E-01	0.96 (0.94, 0.98)	**1.53E-03**
0.83 (0.81, 0.85)	**2.22E-42**	1.08 (1.05, 1.11)	**6.25E-09**
0.82 (0.79, 0.84)	**1.69E-47**	1.06 (1.04, 1.09)	**5.01E-06**
0.92 (0.90, 0.94)	**2.46E-11**	1.12 (1.09, 1.15)	**6.75E-20**
1.17 (1.14, 1.20)	**2.07E-36**	0.93 (0.91, 0.95)	**2.56E-08**
1.15 (1.12, 1.18)	**2.05E-24**	0.95 (0.93, 0.98)	**1.98E-04**
0.94 (0.92, 0.96)	**5.07E-07**	1.09 (1.07, 1.12)	**5.20E-13**
0.89 (0.87, 0.91)	**2.10E-21**	1.11 (1.08, 1.13)	**1.49E-16**
1.10 (1.07, 1.13)	**5.78E-15**	1.03 (1.01, 1.06)	**1.34E-02**
0.97 (0.95, 1.00)	**2.74E-02**	0.96 (0.93, 0.98)	**6.14E-04**
0.80 (0.79, 0.82)	**2.55E-98**	1.02 (1.00, 1.05)	6.20E-02
0.78 (0.76, 0.79)	**3.93E-115**	1.04 (1.01, 1.06)	**1.75E-03**
0.77 (0.76, 0.79)	**1.06E-118**	1.04 (1.02, 1.07)	**9.38E-04**
0.80 (0.78, 0.81)	**2.38E-97**	1.03 (1.01, 1.05)	**1.21E-02**
1.29 (1.26, 1.31)	**2.69E-114**	0.97 (0.94, 0.99)	**3.80E-03**
0.70 (0.69, 0.72)	**1.77E-209**	0.95 (0.93, 0.97)	**2.93E-05**
0.77 (0.75, 0.78)	**2.14E-124**	0.98 (0.96, 1.01)	1.64E-01
0.84 (0.82, 0.86)	**4.12E-52**	1.01 (0.98, 1.03)	5.45E-01
0.70 (0.69, 0.72)	**1.39E-214**	0.95 (0.93, 0.98)	**7.86E-05**
1.34 (1.31, 1.37)	**7.42E-153**	1.03 (1.00, 1.05)	**2.41E-02**
1.49 (1.46, 1.53)	**1.13E-270**	1.15 (1.12, 1.17)	**6.97E-30**
0.72 (0.70, 0.73)	**7.54E-237**	0.97 (0.95, 0.99)	**4.87E-03**
0.71 (0.69, 0.72)	**5.24E-252**	0.97 (0.94, 0.99)	**3.91E-03**
0.84 (0.82, 0.85)	**2.07E-66**	0.98 (0.96, 1.00)	8.80E-02
1.35 (1.32, 1.37)	**1.26E-189**	1.01 (0.99, 1.03)	4.47E-01
1.30 (1.27, 1.33)	**6.98E-124**	1.12 (1.10, 1.15)	**2.03E-23**
0.70 (0.68, 0.71)	**0.00E+00**	0.90 (0.88, 0.91)	**4.23E-26**
0.67 (0.66, 0.68)	**0.00E+00**	0.87 (0.85, 0.89)	**7.46E-38**
0.86 (0.84, 0.88)	**3.54E-45**	0.99 (0.97, 1.01)	5.33E-01
1.39 (1.37, 1.41)	**0.00E+00**	1.10 (1.08, 1.12)	**1.76E-19**
1.03 (1.01, 1.05)	**8.25E-03**	1.03 (1.01, 1.05)	**2.33E-03**
0.88 (0.87, 0.88)	**0.00E+00**	0.92 (0.91, 0.93)	**8.24E-62**
0.89 (0.88, 0.90)	**1.26E-252**	0.91 (0.90, 0.92)	**4.67E-108**
0.79 (0.77, 0.80)	**9.20E-189**	0.99 (0.97, 1.01)	3.04E-01
1.19 (1.18, 1.20)	**0.00E+00**	1.11 (1.10, 1.13)	**4.22E-60**

1.01 (0.99, 1.04)	2.47E-01	1.05 (1.03, 1.08)	**2.69E-06**
0.87 (0.87, 0.88)	**0.00E+00**	0.91 (0.90, 0.92)	**4.98E-86**
0.87 (0.86, 0.89)	**3.09E-52**	0.86 (0.85, 0.88)	**1.15E-90**
0.80 (0.78, 0.82)	**5.85E-111**	1.00 (0.98, 1.02)	1.00E+00
1.17 (1.17, 1.18)	**0.00E+00**	1.11 (1.10, 1.12)	**1.73E-89**
1.18 (1.15, 1.20)	**1.95E-54**	1.16 (1.14, 1.18)	**1.12E-63**
0.83 (0.82, 0.83)	**0.00E+00**	0.88 (0.87, 0.89)	**2.58E-102**
0.85 (0.83, 0.86)	**7.00E-65**	0.86 (0.85, 0.88)	**1.84E-57**
0.80 (0.78, 0.81)	**6.51E-130**	0.95 (0.93, 0.97)	**6.15E-06**
1.18 (1.17, 1.19)	654831133646e	1.07 (1.05, 1.09)	**1.63E-12**
1.04 (1.02, 1.07)	**9.17E-04**	1.22 (1.19, 1.26)	**3.28E-43**
0.84 (0.82, 0.87)	**1.36E-39**	0.84 (0.82, 0.86)	**1.57E-47**
0.79 (0.78, 0.80)	**2.82E-166**	0.91 (0.90, 0.93)	**3.42E-21**
1.07 (1.05, 1.10)	**1.06E-11**	0.88 (0.85, 0.90)	**1.69E-22**
1.09 (1.08, 1.11)	**2.98E-29**	0.94 (0.92, 0.96)	**6.25E-07**
1.23 (1.21, 1.25)	**8.54E-96**	0.96 (0.94, 0.98)	**4.24E-05**
0.83 (0.81, 0.84)	**8.72E-89**	1.06 (1.03, 1.08)	**3.84E-06**
0.82 (0.80, 0.83)	**9.01E-94**	1.04 (1.01, 1.06)	**1.42E-03**
0.90 (0.88, 0.92)	**1.57E-24**	1.11 (1.08, 1.14)	**8.87E-18**
1.12 (1.10, 1.13)	**3.28E-54**	0.95 (0.93, 0.98)	**7.85E-05**
1.28 (1.26, 1.31)	**2.66E-117**	1.01 (0.99, 1.03)	4.71E-01
0.80 (0.79, 0.82)	**3.03E-97**	1.01 (0.98, 1.03)	6.79E-01
0.82 (0.80, 0.84)	**1.33E-77**	1.00 (0.97, 1.02)	7.71E-01
0.81 (0.79, 0.82)	**2.15E-81**	1.04 (1.02, 1.07)	**1.20E-03**
1.13 (1.12, 1.14)	**1.06E-118**	0.99 (0.96, 1.01)	2.35E-01
1.22 (1.19, 1.25)	**2.08E-68**	1.20 (1.17, 1.23)	**6.63E-52**
0.75 (0.74, 0.77)	**1.50E-154**	0.89 (0.87, 0.91)	**6.73E-22**
0.77 (0.75, 0.78)	**8.74E-147**	0.87 (0.85, 0.89)	**1.43E-35**
0.96 (0.94, 0.98)	**8.92E-04**	1.13 (1.10, 1.15)	**3.82E-25**
1.25 (1.22, 1.27)	**1.08E-110**	1.00 (0.98, 1.03)	9.60E-01
1.00 (0.98, 1.02)	8.89E-01	0.82 (0.80, 0.84)	**7.15E-61**
0.84 (0.82, 0.86)	**9.24E-56**	1.01 (0.99, 1.04)	2.71E-01
0.99 (0.97, 1.01)	4.66E-01	1.27 (1.24, 1.30)	**7.52E-80**
0.69 (0.67, 0.71)	**1.75E-198**	0.87 (0.85, 0.90)	**5.18E-27**
0.84 (0.82, 0.86)	**8.15E-48**	0.86 (0.84, 0.88)	**2.84E-32**
0.68 (0.67, 0.70)	**3.93E-204**	0.83 (0.81, 0.85)	**2.65E-53**
0.74 (0.73, 0.76)	**1.42E-124**	0.87 (0.85, 0.89)	**2.14E-28**
0.69 (0.67, 0.70)	**1.01E-206**	0.88 (0.85, 0.90)	**2.49E-26**
0.71 (0.69, 0.73)	**7.54E-171**	0.87 (0.85, 0.89)	**8.86E-28**
1.04 (1.01, 1.06)	**1.49E-03**	0.89 (0.87, 0.91)	**1.99E-19**
1.02 (0.99, 1.04)	1.73E-01	0.87 (0.85, 0.89)	**3.40E-27**
1.11 (1.09, 1.14)	**5.11E-23**	0.99 (0.97, 1.02)	6.14E-01
1.11 (1.09, 1.14)	**2.07E-22**	0.99 (0.97, 1.01)	3.85E-01
0.82 (0.80, 0.84)	**1.48E-61**	0.94 (0.92, 0.96)	**1.17E-06**
1.14 (1.12, 1.17)	**5.53E-33**	0.90 (0.88, 0.92)	**2.12E-16**
0.79 (0.77, 0.81)	**1.19E-79**	0.94 (0.91, 0.96)	**7.22E-07**

0.78 (0.76, 0.80)	**4.96E-91**	0.97 (0.95, 1.00)	**3.19E-02**
0.78 (0.76, 0.80)	**8.74E-90**	0.93 (0.91, 0.96)	**1.66E-07**
0.84 (0.82, 0.86)	**4.39E-43**	1.02 (0.99, 1.05)	1.70E-01
0.74 (0.73, 0.76)	**5.97E-124**	0.86 (0.84, 0.88)	**8.40E-37**
0.87 (0.85, 0.89)	**2.73E-31**	0.88 (0.85, 0.90)	**1.66E-26**
0.92 (0.89, 0.94)	**2.75E-13**	0.91 (0.88, 0.93)	**4.30E-16**
0.72 (0.70, 0.74)	**1.17E-175**	0.85 (0.83, 0.87)	**9.31E-39**
0.79 (0.77, 0.81)	**4.02E-71**	0.86 (0.84, 0.88)	**1.33E-28**
0.71 (0.69, 0.73)	**1.62E-161**	0.85 (0.83, 0.88)	**2.66E-36**
0.84 (0.82, 0.87)	**5.48E-40**	0.83 (0.80, 0.85)	**2.41E-44**
0.73 (0.71, 0.75)	**2.54E-142**	0.85 (0.83, 0.87)	**4.37E-38**
0.73 (0.71, 0.75)	**3.10E-140**	0.83 (0.81, 0.85)	**6.12E-50**
1.06 (1.04, 1.08)	**3.26E-07**	0.95 (0.93, 0.97)	**3.43E-05**
0.98 (0.96, 1.00)	6.89E-02	0.94 (0.92, 0.97)	**1.80E-06**
0.83 (0.81, 0.85)	**3.53E-47**	0.92 (0.89, 0.94)	**7.67E-12**
0.69 (0.68, 0.71)	**2.89E-242**	0.92 (0.90, 0.94)	**4.99E-12**
0.86 (0.84, 0.88)	**1.17E-33**	0.84 (0.82, 0.87)	**8.50E-37**
0.77 (0.76, 0.79)	**6.69E-126**	0.97 (0.95, 1.00)	**2.34E-02**
0.74 (0.73, 0.76)	**9.35E-172**	0.92 (0.90, 0.94)	**4.50E-13**
1.34 (1.31, 1.37)	**2.82E-156**	1.05 (1.02, 1.07)	**2.05E-04**
1.22 (1.19, 1.24)	**4.29E-69**	1.10 (1.08, 1.13)	**2.87E-18**
1.20 (1.19, 1.22)	**8.84E-193**	1.10 (1.09, 1.12)	**2.06E-34**
1.11 (1.09, 1.13)	**8.73E-20**	1.06 (1.03, 1.08)	**3.72E-06**
1.13 (1.12, 1.15)	**9.36E-54**	1.09 (1.07, 1.11)	**9.30E-21**
1.05 (1.03, 1.08)	**8.72E-06**	0.98 (0.95, 1.00)	**3.88E-02**
0.98 (0.96, 1.00)	1.26E-01	0.95 (0.93, 0.98)	**7.24E-05**
0.82 (0.80, 0.84)	**2.42E-52**	0.87 (0.85, 0.89)	**1.85E-25**
1.00 (0.98, 1.03)	7.43E-01	0.92 (0.90, 0.94)	**1.10E-10**
0.93 (0.91, 0.95)	**6.92E-09**	0.84 (0.82, 0.87)	**2.74E-37**
0.94 (0.92, 0.96)	**1.13E-07**	0.84 (0.82, 0.86)	**1.12E-42**
1.05 (1.04, 1.06)	**9.90E-34**	1.04 (1.02, 1.06)	**2.37E-05**
1.12 (1.10, 1.15)	**9.33E-27**	1.03 (1.00, 1.05)	**2.36E-02**
1.10 (1.09, 1.11)	**3.23E-150**	1.09 (1.08, 1.10)	**2.58E-102**
1.10 (1.09, 1.11)	**4.89E-99**	1.07 (1.06, 1.08)	**7.32E-31**
1.09 (1.08, 1.10)	**6.15E-78**	1.09 (1.08, 1.11)	**2.65E-43**
1.11 (1.11, 1.12)	**1.59E-262**	1.05 (1.04, 1.06)	**1.05E-20**
0.70 (0.68, 0.71)	**1.53E-224**	0.79 (0.77, 0.81)	**4.56E-83**
1.30 (1.27, 1.32)	**4.94E-126**	1.09 (1.06, 1.11)	**1.63E-12**
0.85 (0.83, 0.87)	**2.03E-38**	1.03 (1.00, 1.06)	**4.73E-02**
0.95 (0.93, 0.97)	**1.36E-05**	0.85 (0.83, 0.87)	**1.52E-34**
0.71 (0.69, 0.72)	**4.98E-169**	0.85 (0.83, 0.87)	**1.21E-41**
0.68 (0.66, 0.69)	**6.46E-214**	0.84 (0.82, 0.86)	**3.45E-46**
0.69 (0.68, 0.71)	**6.62E-188**	0.82 (0.80, 0.84)	**1.55E-56**
0.82 (0.80, 0.84)	**9.73E-57**	1.06 (1.03, 1.09)	**8.52E-06**
0.73 (0.71, 0.74)	**4.44E-160**	0.94 (0.92, 0.96)	**7.33E-07**
1.09 (1.07, 1.10)	**2.71E-38**	1.11 (1.10, 1.12)	**4.14E-71**
0.88 (0.86, 0.90)	**1.46E-25**	0.86 (0.83, 0.88)	**2.87E-35**
0.67 (0.65, 0.69)	**1.52E-233**	0.85 (0.83, 0.87)	**8.59E-42**

0.86 (0.84, 0.88)	**1.37E-32**	1.10 (1.07, 1.13)	**1.37E-12**
1.06 (1.04, 1.07)	**9.50E-18**	1.05 (1.04, 1.07)	**9.56E-13**
1.08 (1.08, 1.09)	**6.52E-118**	1.09 (1.08, 1.10)	**1.64E-76**
0.75 (0.73, 0.77)	**3.83E-120**	0.91 (0.89, 0.93)	**5.63E-13**
0.90 (0.88, 0.92)	**2.20E-19**	0.88 (0.85, 0.90)	**2.14E-26**
0.73 (0.71, 0.75)	**1.27E-134**	0.85 (0.83, 0.87)	**1.02E-41**
0.78 (0.76, 0.80)	**4.02E-93**	0.93 (0.91, 0.96)	**8.52E-08**
0.77 (0.76, 0.79)	**3.72E-98**	0.92 (0.90, 0.94)	**1.52E-11**
0.92 (0.90, 0.94)	**1.24E-11**	0.87 (0.85, 0.89)	**2.99E-30**
0.70 (0.68, 0.71)	**2.07E-183**	0.83 (0.81, 0.85)	**2.86E-49**
0.89 (0.87, 0.91)	**3.15E-21**	1.07 (1.04, 1.10)	**2.40E-07**
0.76 (0.75, 0.78)	**3.76E-106**	0.87 (0.85, 0.89)	**1.63E-28**
0.94 (0.92, 0.97)	**2.11E-06**	0.86 (0.84, 0.88)	**1.43E-32**
0.70 (0.68, 0.72)	**6.99E-177**	0.83 (0.81, 0.85)	**3.80E-49**
0.87 (0.85, 0.89)	**2.19E-31**	1.07 (1.04, 1.10)	**3.89E-07**
0.81 (0.79, 0.83)	**2.34E-66**	0.87 (0.85, 0.89)	**1.79E-28**
0.83 (0.81, 0.85)	**1.63E-51**	1.07 (1.04, 1.10)	**3.81E-07**
1.04 (1.02, 1.07)	**1.33E-03**	1.00 (0.97, 1.02)	8.55E-01

Supple

Metabolites	Group
Concentration of chylomicrons and extremely large VLDL particles	Lipoprotein subclasses
Total lipids in chylomicrons and extremely large VLDL	Lipoprotein subclasses
Phospholipids in chylomicrons and extremely large VLDL	Lipoprotein subclasses
Cholesterol in chylomicrons and extremely large VLDL	Lipoprotein subclasses
Cholesteryl esters in chylomicrons and extremely large VLDL	Lipoprotein subclasses
Free cholesterol in chylomicrons and extremely large VLDL	Lipoprotein subclasses
Triglycerides in chylomicrons and extremely large VLDL	Lipoprotein subclasses
Concentration of very large VLDL particles	Lipoprotein subclasses
Total lipids in very large VLDL	Lipoprotein subclasses
Phospholipids in very large VLDL	Lipoprotein subclasses
Cholesterol in very large VLDL	Lipoprotein subclasses
Cholesteryl esters in very large VLDL	Lipoprotein subclasses
Free cholesterol in very large VLDL	Lipoprotein subclasses
Triglycerides in very large VLDL	Lipoprotein subclasses
Concentration of large VLDL particles	Lipoprotein subclasses
Total lipids in large VLDL	Lipoprotein subclasses
Phospholipids in large VLDL	Lipoprotein subclasses
Cholesterol in large VLDL	Lipoprotein subclasses
Cholesteryl esters in large VLDL	Lipoprotein subclasses
Free cholesterol in large VLDL	Lipoprotein subclasses
Triglycerides in large VLDL	Lipoprotein subclasses
Concentration of medium VLDL particles	Lipoprotein subclasses
Total lipids in medium VLDL	Lipoprotein subclasses
Phospholipids in medium VLDL	Lipoprotein subclasses
Cholesterol in medium VLDL	Lipoprotein subclasses
Cholesteryl esters in medium VLDL	Lipoprotein subclasses
Free cholesterol in medium VLDL	Lipoprotein subclasses
Triglycerides in medium VLDL	Lipoprotein subclasses
Concentration of small VLDL particles	Lipoprotein subclasses
Total lipids in small VLDL	Lipoprotein subclasses
Phospholipids in small VLDL	Lipoprotein subclasses
Cholesterol in small VLDL	Lipoprotein subclasses
Cholesteryl esters in small VLDL	Lipoprotein subclasses
Free cholesterol in small VLDL	Lipoprotein subclasses
Triglycerides in small VLDL	Lipoprotein subclasses
Concentration of very small VLDL particles	Lipoprotein subclasses
Total lipids in very small VLDL	Lipoprotein subclasses
Phospholipids in very small VLDL	Lipoprotein subclasses
Cholesterol in very small VLDL	Lipoprotein subclasses
Cholesteryl esters in very small VLDL	Lipoprotein subclasses
Free cholesterol in very small VLDL	Lipoprotein subclasses
Triglycerides in very small VLDL	Lipoprotein subclasses
Concentration of IDL particles	Lipoprotein subclasses

Total lipids in IDL	Lipoprotein subclasses
Phospholipids in IDL	Lipoprotein subclasses
Cholesterol in IDL	Lipoprotein subclasses
Cholesteryl esters in IDL	Lipoprotein subclasses
Free cholesterol in IDL	Lipoprotein subclasses
Triglycerides in IDL	Lipoprotein subclasses
Concentration of large LDL particles	Lipoprotein subclasses
Total lipids in large LDL	Lipoprotein subclasses
Phospholipids in large LDL	Lipoprotein subclasses
Cholesterol in large LDL	Lipoprotein subclasses
Cholesteryl esters in large LDL	Lipoprotein subclasses
Free cholesterol in large LDL	Lipoprotein subclasses
Triglycerides in large LDL	Lipoprotein subclasses
Concentration of medium LDL particles	Lipoprotein subclasses
Total lipids in medium LDL	Lipoprotein subclasses
Phospholipids in medium LDL	Lipoprotein subclasses
Cholesterol in medium LDL	Lipoprotein subclasses
Cholesteryl esters in medium LDL	Lipoprotein subclasses
Free cholesterol in medium LDL	Lipoprotein subclasses
Triglycerides in medium LDL	Lipoprotein subclasses
Concentration of small LDL particles	Lipoprotein subclasses
Total lipids in small LDL	Lipoprotein subclasses
Phospholipids in small LDL	Lipoprotein subclasses
Cholesterol in small LDL	Lipoprotein subclasses
Cholesteryl esters in small LDL	Lipoprotein subclasses
Free cholesterol in small LDL	Lipoprotein subclasses
Triglycerides in small LDL	Lipoprotein subclasses
Concentration of very large HDL particles	Lipoprotein subclasses
Total lipids in very large HDL	Lipoprotein subclasses
Phospholipids in very large HDL	Lipoprotein subclasses
Cholesterol in very large HDL	Lipoprotein subclasses
Cholesteryl esters in very large HDL	Lipoprotein subclasses
Free cholesterol in very large HDL	Lipoprotein subclasses
Triglycerides in very large HDL	Lipoprotein subclasses
Concentration of large HDL particles	Lipoprotein subclasses
Total lipids in large HDL	Lipoprotein subclasses
Phospholipids in large HDL	Lipoprotein subclasses
Cholesterol in large HDL	Lipoprotein subclasses
Cholesteryl esters in large HDL	Lipoprotein subclasses
Free cholesterol in large HDL	Lipoprotein subclasses
Triglycerides in large HDL	Lipoprotein subclasses
**Concentration of medium HDL particles**	Lipoprotein subclasses
**Total lipids in medium HDL**	Lipoprotein subclasses
**Phospholipids in medium HDL**	Lipoprotein subclasses
**Cholesterol in medium HDL**	Lipoprotein subclasses
**Cholesteryl esters in medium HDL**	Lipoprotein subclasses
**Free cholesterol in medium HDL**	Lipoprotein subclasses
Triglycerides in medium HDL	Lipoprotein subclasses

Concentration of small HDL particles	Lipoprotein subclasses
Total lipids in small HDL	Lipoprotein subclasses
Phospholipids in small HDL	Lipoprotein subclasses
Cholesterol in small HDL	Lipoprotein subclasses
Cholesteryl esters in small HDL	Lipoprotein subclasses
**Free cholesterol in small HDL**	Lipoprotein subclasses
Triglycerides in small HDL	Lipoprotein subclasses
Phospholipids to total lipids ratio in chylomicrons and extremely large	Relative lipoprotein lipid
Cholesterol to total lipids ratio in chylomicrons and extremely large V	Relative lipoprotein lipid
Cholesteryl esters to total lipids ratio in chylomicrons and extremely l	Relative lipoprotein lipid
Free cholesterol to total lipids ratio in chylomicrons and extremely lar	Relative lipoprotein lipid
Triglycerides to total lipids ratio in chylomicrons and extremely large	Relative lipoprotein lipid
Phospholipids to total lipids ratio in very large VLDL /(lipds in ckb)	Relative lipoprotein lipid
**Cholesterol to total lipids ratio in very large VLDL**	Relative lipoprotein lipid
Cholesteryl esters to total lipids ratio in very large VLDL	Relative lipoprotein lipid
**Free cholesterol to total lipids ratio in very large VLDL**	Relative lipoprotein lipid
**Triglycerides to total lipids ratio in very large VLDL**	Relative lipoprotein lipid
Phospholipids to total lipids ratio in large VLDL	Relative lipoprotein lipid
Cholesterol to total lipids ratio in large VLDL	Relative lipoprotein lipid
Cholesteryl esters to total lipids ratio in large VLDL	Relative lipoprotein lipid
Free cholesterol to total lipids ratio in large VLDL	Relative lipoprotein lipid
Triglycerides to total lipids ratio in large VLDL	Relative lipoprotein lipid
Phospholipids to total lipids ratio in medium VLDL	Relative lipoprotein lipid
**Cholesterol to total lipids ratio in medium VLDL**	Relative lipoprotein lipid
**Cholesteryl esters to total lipids ratio in medium VLDL**	Relative lipoprotein lipid
Free cholesterol to total lipids ratio in medium VLDL	Relative lipoprotein lipid
Triglycerides to total lipids ratio in medium VLDL	Relative lipoprotein lipid
**Phospholipids to total lipids ratio in small VLDL**	Relative lipoprotein lipid
Cholesterol to total lipids ratio in small VLDL	Relative lipoprotein lipid
Cholesteryl esters to total lipids ratio in small VLDL	Relative lipoprotein lipid
**Free cholesterol to total lipids ratio in small VLDL**	Relative lipoprotein lipid
Triglycerides to total lipids ratio in small VLDL	Relative lipoprotein lipid
Phospholipids to total lipids ratio in very small VLDL	Relative lipoprotein lipid
Cholesterol to total lipids ratio in very small VLDL	Relative lipoprotein lipid
Cholesteryl esters to total lipids ratio in very small VLDL	Relative lipoprotein lipid
Free cholesterol to total lipids ratio in very small VLDL	Relative lipoprotein lipid
Triglycerides to total lipids ratio in very small VLDL	Relative lipoprotein lipid
Phospholipids to total lipids ratio in IDL	Relative lipoprotein lipid
Cholesterol to total lipids ratio in IDL	Relative lipoprotein lipid
Cholesteryl esters to total lipids ratio in IDL	Relative lipoprotein lipid
Free cholesterol to total lipids ratio in IDL	Relative lipoprotein lipid
Triglycerides to total lipids ratio in IDL	Relative lipoprotein lipid
Phospholipids to total lipids ratio in large LDL	Relative lipoprotein lipid
Cholesterol to total lipids ratio in large LDL	Relative lipoprotein lipid
Cholesteryl esters to total lipids ratio in large LDL	Relative lipoprotein lipid
Free cholesterol to total lipids ratio in large LDL	Relative lipoprotein lipid
Triglycerides to total lipids ratio in large LDL	Relative lipoprotein lipid
Phospholipids to total lipids ratio in medium LDL	Relative lipoprotein lipid

Cholesterol to total lipids ratio in medium LDL	Relative lipoprotein lipid
Cholesteryl esters to total lipids ratio in medium LDL	Relative lipoprotein lipid
Free cholesterol to total lipids ratio in medium LDL	Relative lipoprotein lipid
Triglycerides to total lipids ratio in medium LDL	Relative lipoprotein lipid
Phospholipids to total lipids ratio in small LDL	Relative lipoprotein lipid
Cholesterol to total lipids ratio in small LDL	Relative lipoprotein lipid
Cholesteryl esters to total lipids ratio in small LDL	Relative lipoprotein lipid
Free cholesterol to total lipids ratio in small LDL	Relative lipoprotein lipid
Triglycerides to total lipids ratio in small LDL	Relative lipoprotein lipid
Phospholipids to total lipids ratio in very large HDL	Relative lipoprotein lipid
Cholesterol to total lipids ratio in very large HDL	Relative lipoprotein lipid
Cholesteryl esters to total lipids ratio in very large HDL	Relative lipoprotein lipid
Free cholesterol to total lipids ratio in very large HDL	Relative lipoprotein lipid
Triglycerides to total lipids ratio in very large HDL	Relative lipoprotein lipid
Phospholipids to total lipids ratio in large HDL	Relative lipoprotein lipid
Cholesterol to total lipids ratio in large HDL	Relative lipoprotein lipid
Cholesteryl esters to total lipids ratio in large HDL	Relative lipoprotein lipid
Free cholesterol to total lipids ratio in large HDL	Relative lipoprotein lipid
Triglycerides to total lipids ratio in large HDL	Relative lipoprotein lipid
Phospholipids to total lipids ratio in medium HDL	Relative lipoprotein lipid
Cholesterol to total lipids ratio in medium HDL	Relative lipoprotein lipid
Cholesteryl esters to total lipids ratio in medium HDL	Relative lipoprotein lipid
Free cholesterol to total lipids ratio in medium HDL	Relative lipoprotein lipid
Triglycerides to total lipids ratio in medium HDL	Relative lipoprotein lipid
Phospholipids to total lipids ratio in small HDL	Relative lipoprotein lipid
Cholesterol to total lipids ratio in small HDL	Relative lipoprotein lipid
Cholesteryl esters to total lipids ratio in small HDL	Relative lipoprotein lipid
Free cholesterol to total lipids ratio in small HDL	Relative lipoprotein lipid
Triglycerides to total lipids ratio in small HDL	Relative lipoprotein lipid
Average diameter for VLDL particles	Lipoprotein particle sizes
Average diameter for LDL particles	Lipoprotein particle sizes
Average diameter for HDL particles	Lipoprotein particle sizes
Total cholesterol	Cholesterol
VLDL cholesterol	Cholesterol
Remnant cholesterol (non-HDL, non-LDL -cholesterol)	Cholesterol
LDL cholesterol	Cholesterol
**HDL cholesterol**	Cholesterol
**HDL2 cholesterol**	Cholesterol
HDL3 cholesterol	Cholesterol
Total esterified cholesterol	Cholesteryl esters
Total free cholesterol	Free cholesterol
Total triglycerides	Triglycerides
Triglycerides in VLDL	Triglycerides
Triglycerides in LDL	Triglycerides
Triglycerides in HDL	Triglycerides
Phosphoglycerides	Other lipids
Ratio of triglycerides to phosphoglycerides	Other lipids
Phosphatidylcholines	Other lipids

Sphingomyelins	Other lipids
Total cholines	Other lipids
Apolipoprotein A1	Apolipoproteins
Apolipoprotein B	Apolipoproteins
Ratio of apolipoprotein B to apolipoprotein A1	Apolipoproteins
Total fatty acids	Fatty acids
Degree of unsaturation	Fatty acids
Docosahexaenoic acid	Fatty acids
Linoleic acid	Fatty acids
Omega-3 fatty acids	Fatty acids
Omega-6 fatty acids	Fatty acids
Polyunsaturated fatty acids	Fatty acids
Monounsaturated fatty acids	Fatty acids
Saturated fatty acids	Fatty acids
Ratio of docosahexaenoic acid to total fatty acids	Fatty acids
Ratio of linoleic acid to total fatty acids	Fatty acids
Ratio of omega-3 fatty acids to total fatty acids	Fatty acids
Ratio of omega-6 fatty acids to total fatty acids	Fatty acids
Ratio of polyunsaturated fatty acids to total fatty acids	Fatty acids
Ratio of monounsaturated fatty acids to total fatty acids	Fatty acids
Ratio of saturated fatty acids to total fatty acids	Fatty acids
Glucose	Glycolysis related metabo
Lactate	Glycolysis related metabo
Citrate	Glycolysis related metabo
Alanine	Amino acids
Glutamine	Amino acids
Histidine	Amino acids
Isoleucine	Amino acids
Leucine	Amino acids
Valine	Amino acids
Phenylalanine	Amino acids
Tyrosine	Amino acids
Acetate	Ketone bodies
Acetoacetate	Ketone bodies
**3-Hydroxybutyrate**	Ketone bodies
Creatinine	Fluid balance
Albumin	Fluid balance
Glycoprotein acetyls	Inflammation

Models were adjusted for age, sex, regions, levels of education, smoking status, alcohol consumption, and

CI, confidence interval; LDL, Low Density Lipoprotein; HDL, High Density Lipoprotein; VLDL, Very L

ementary Table 6. Association of all metabolites with sleep duration in the CKB

Male		Female	
Adjusted β (95% CI)	P value	Adjusted β (95% CI)	P value
-0.02 (-0.07, 0.02)	7.16E-01	0.06 (0.01, 0.10)	**4.84E-02**
-0.02 (-0.07, 0.02)	7.16E-01	0.06 (0.01, 0.10)	**4.84E-02**
-0.02 (-0.07, 0.02)	7.16E-01	0.05 (0.01, 0.10)	8.19E-02
-0.01 (-0.06, 0.03)	8.35E-01	0.06 (0.02, 0.11)	**3.17E-02**
0.00 (-0.05, 0.04)	9.77E-01	0.08 (0.03, 0.12)	**1.02E-02**
-0.02 (-0.07, 0.02)	7.16E-01	0.05 (0.00, 0.09)	1.01E-01
-0.03 (-0.07, 0.02)	6.67E-01	0.06 (0.01, 0.10)	**4.97E-02**
-0.02 (-0.07, 0.02)	7.16E-01	0.05 (0.00, 0.09)	9.38E-02
-0.02 (-0.07, 0.02)	7.16E-01	0.05 (0.00, 0.10)	8.93E-02
-0.02 (-0.07, 0.03)	7.43E-01	0.05 (0.01, 0.10)	7.43E-02
-0.01 (-0.06, 0.03)	7.74E-01	0.06 (0.01, 0.11)	**4.13E-02**
-0.01 (-0.06, 0.03)	8.07E-01	0.06 (0.02, 0.11)	**3.30E-02**
-0.02 (-0.06, 0.03)	7.72E-01	0.05 (0.01, 0.10)	7.66E-02
-0.03 (-0.07, 0.02)	6.84E-01	0.05 (0.00, 0.09)	1.15E-01
-0.02 (-0.07, 0.02)	7.29E-01	0.05 (0.00, 0.09)	9.38E-02
-0.02 (-0.07, 0.03)	7.38E-01	0.05 (0.00, 0.09)	9.38E-02
-0.02 (-0.06, 0.03)	7.43E-01	0.05 (0.00, 0.09)	9.38E-02
-0.01 (-0.06, 0.03)	8.07E-01	0.06 (0.01, 0.10)	6.16E-02
-0.01 (-0.05, 0.04)	9.00E-01	0.06 (0.02, 0.11)	**3.50E-02**
-0.02 (-0.06, 0.03)	7.43E-01	0.05 (0.00, 0.09)	1.21E-01
-0.02 (-0.07, 0.02)	7.16E-01	0.05 (0.00, 0.09)	1.08E-01
-0.01 (-0.06, 0.03)	8.26E-01	0.05 (0.01, 0.10)	5.92E-02
-0.01 (-0.06, 0.04)	8.36E-01	0.06 (0.01, 0.10)	5.77E-02
-0.01 (-0.05, 0.04)	9.00E-01	0.06 (0.01, 0.10)	5.77E-02
0.00 (-0.04, 0.05)	9.39E-01	0.07 (0.02, 0.11)	**3.11E-02**
0.02 (-0.03, 0.06)	7.43E-01	0.08 (0.03, 0.12)	**1.09E-02**
-0.01 (-0.06, 0.04)	8.36E-01	0.05 (0.01, 0.10)	8.24E-02
-0.02 (-0.06, 0.03)	7.72E-01	0.05 (0.01, 0.09)	8.24E-02
0.01 (-0.04, 0.06)	8.36E-01	0.05 (0.01, 0.10)	8.24E-02
0.01 (-0.03, 0.06)	7.97E-01	0.05 (0.01, 0.10)	8.24E-02
0.01 (-0.04, 0.06)	8.36E-01	0.04 (0.00, 0.09)	1.53E-01
0.04 (0.00, 0.09)	3.77E-01	0.06 (0.01, 0.10)	5.77E-02
0.06 (-0.01, 0.10)	3.77E-01	0.06 (0.02, 0.11)	**3.78E-02**
0.02 (-0.03, 0.06)	7.72E-01	0.04 (-0.01, 0.09)	1.93E-01
0.00 (-0.05, 0.04)	9.28E-01	0.05 (0.00, 0.09)	1.17E-01
0.04 (-0.01, 0.08)	4.02E-01	0.04 (-0.01, 0.08)	2.34E-01
0.04 (-0.01, 0.09)	3.77E-01	0.03 (-0.01, 0.08)	2.49E-01
0.04 (-0.01, 0.08)	3.95E-01	0.02 (-0.02, 0.07)	4.22E-01
0.05 (0.00, 0.09)	3.77E-01	0.03 (-0.01, 0.08)	2.32E-01
0.05 (0.00, 0.09)	3.77E-01	0.04 (0.00, 0.09)	1.28E-01
0.05 (0.00, 0.09)	3.77E-01	0.01 (-0.04, 0.06)	7.40E-01
0.01 (-0.04, 0.05)	9.00E-01	0.02 (-0.02, 0.07)	4.42E-01
0.04 (-0.01, 0.09)	3.77E-01	0.04 (-0.01, 0.08)	2.31E-01

0.04 (0.00, 0.09)	3.77E-01	0.04 (-0.01, 0.08)	2.31E-01
0.04 (0.00, 0.09)	3.77E-01	0.03 (-0.01, 0.08)	2.52E-01
0.04 (0.00, 0.09)	3.77E-01	0.04 (-0.01, 0.08)	2.31E-01
0.04 (-0.01, 0.08)	3.77E-01	0.04 (0.00, 0.09)	1.54E-01
0.04 (0.00, 0.09)	3.77E-01	0.02 (-0.03, 0.06)	5.81E-01
0.01 (-0.03, 0.05)	8.36E-01	0.00 (-0.05, 0.05)	9.79E-01
0.04 (0.00, 0.09)	3.77E-01	0.04 (-0.01, 0.08)	2.26E-01
0.04 (0.00, 0.09)	3.77E-01	0.04 (-0.01, 0.08)	2.29E-01
0.04 (0.00, 0.09)	3.77E-01	0.04 (-0.01, 0.09)	2.14E-01
0.04 (0.00, 0.09)	3.77E-01	0.04 (-0.01, 0.09)	2.06E-01
0.04 (0.00, 0.09)	3.77E-01	0.04 (0.00, 0.09)	1.54E-01
0.04 (0.00, 0.09)	3.77E-01	0.02 (-0.02, 0.07)	4.22E-01
0.02 (-0.03, 0.06)	7.72E-01	0.00 (-0.05, 0.04)	8.89E-01
0.05 (0.00, 0.09)	3.77E-01	0.04 (-0.01, 0.08)	2.31E-01
0.05 (0.00, 0.09)	3.77E-01	0.04 (-0.01, 0.08)	2.32E-01
0.04 (0.00, 0.09)	3.77E-01	0.04 (0.00, 0.09)	1.45E-01
0.05 (0.00, 0.09)	3.77E-01	0.03 (-0.01, 0.08)	2.45E-01
0.05 (0.00, 0.09)	3.77E-01	0.04 (-0.01, 0.08)	2.31E-01
0.05 (0.00, 0.09)	3.77E-01	0.03 (-0.02, 0.07)	3.84E-01
0.02 (-0.02, 0.07)	7.27E-01	0.00 (-0.05, 0.05)	9.38E-01
0.04 (0.00, 0.09)	3.77E-01	0.03 (-0.02, 0.08)	3.00E-01
0.04 (0.00, 0.09)	3.77E-01	0.03 (-0.02, 0.08)	3.24E-01
0.04 (-0.01, 0.08)	4.24E-01	0.03 (-0.02, 0.07)	3.68E-01
0.04 (0.00, 0.09)	3.77E-01	0.03 (-0.02, 0.07)	3.56E-01
0.04 (0.00, 0.09)	3.77E-01	0.02 (-0.02, 0.07)	4.07E-01
0.04 (0.00, 0.09)	3.77E-01	0.01 (-0.03, 0.06)	6.45E-01
0.02 (-0.02, 0.07)	7.29E-01	0.03 (-0.02, 0.07)	3.53E-01
-0.01 (-0.06, 0.03)	7.85E-01	-0.05 (-0.10, -0.01)	5.77E-02
-0.01 (-0.06, 0.03)	7.74E-01	-0.05 (-0.10, -0.01)	5.77E-02
-0.01 (-0.06, 0.03)	8.26E-01	-0.06 (-0.11, -0.02)	**3.30E-02**
-0.02 (-0.06, 0.03)	7.43E-01	-0.05 (-0.09, 0.00)	9.75E-02
-0.02 (-0.06, 0.03)	7.43E-01	-0.04 (-0.09, 0.00)	1.28E-01
-0.02 (-0.06, 0.03)	7.43E-01	-0.06 (-0.10, -0.01)	**4.97E-02**
0.00 (-0.05, 0.04)	9.73E-01	0.03 (-0.02, 0.07)	3.43E-01
-0.02 (-0.06, 0.03)	7.72E-01	-0.08 (-0.12, -0.03)	**6.59E-03**
-0.02 (-0.06, 0.03)	7.72E-01	-0.08 (-0.12, -0.04)	**6.59E-03**
-0.01 (-0.06, 0.03)	7.85E-01	-0.08 (-0.12, -0.04)	**6.59E-03**
-0.02 (-0.06, 0.03)	7.47E-01	-0.08 (-0.13, -0.04)	**6.52E-03**
-0.02 (-0.07, 0.03)	7.43E-01	-0.08 (-0.13, -0.04)	**6.52E-03**
-0.01 (-0.06, 0.03)	7.81E-01	-0.08 (-0.12, -0.04)	**6.59E-03**
0.01 (-0.04, 0.05)	9.00E-01	-0.01 (-0.05, 0.04)	8.34E-01
-0.02 (-0.07, 0.02)	7.16E-01	-0.07 (-0.11, -0.02)	**2.57E-02**
-0.02 (-0.07, 0.02)	7.16E-01	-0.07 (-0.11, -0.03)	**1.86E-02**
-0.02 (-0.07, 0.03)	7.29E-01	-0.07 (-0.11, -0.02)	**2.48E-02**
-0.02 (-0.07, 0.02)	7.16E-01	-0.08 (-0.12, -0.04)	**6.59E-03**
-0.03 (-0.07, 0.02)	6.84E-01	-0.08 (-0.12, -0.03)	**6.59E-03**
-0.02 (-0.06, 0.03)	7.72E-01	-0.08 (-0.13, -0.04)	**6.52E-03**
0.00 (-0.04, 0.05)	9.28E-01	0.05 (0.00, 0.10)	9.51E-02

-0.01 (-0.05, 0.04)	9.00E-01	-0.03 (-0.08, 0.01)	2.40E-01
-0.01 (-0.05, 0.04)	9.14E-01	-0.04 (-0.08, 0.01)	2.05E-01
-0.02 (-0.07, 0.02)	7.16E-01	-0.04 (-0.08, 0.01)	2.14E-01
0.02 (-0.03, 0.06)	7.43E-01	-0.03 (-0.07, 0.02)	3.00E-01
0.02 (-0.02, 0.07)	7.16E-01	-0.02 (-0.06, 0.03)	5.79E-01
-0.02 (-0.06, 0.03)	7.72E-01	-0.06 (-0.11, -0.02)	**3.04E-02**
0.00 (-0.05, 0.04)	9.68E-01	0.04 (0.00, 0.09)	1.53E-01
0.01 (-0.04, 0.05)	9.00E-01	0.00 (-0.05, 0.04)	9.74E-01
0.03 (-0.02, 0.07)	6.37E-01	0.04 (-0.01, 0.08)	1.98E-01
0.02 (-0.03, 0.06)	7.43E-01	0.04 (0.00, 0.09)	1.25E-01
0.03 (-0.02, 0.07)	5.77E-01	0.00 (-0.05, 0.04)	9.40E-01
-0.03 (-0.07, 0.02)	6.67E-01	-0.03 (-0.08, 0.01)	2.31E-01
0.04 (-0.01, 0.08)	4.24E-01	0.05 (0.00, 0.10)	9.38E-02
0.07 (0.02, 0.11)	3.77E-01	0.07 (0.02, 0.12)	**2.48E-02**
0.04 (-0.01, 0.08)	4.24E-01	0.04 (-0.01, 0.08)	1.95E-01
0.06 (0.01, 0.10)	3.77E-01	0.06 (0.02, 0.11)	**4.01E-02**
-0.07 (-0.12, -0.02)	3.77E-01	-0.08 (-0.13, -0.04)	**6.59E-03**
0.01 (-0.03, 0.06)	7.85E-01	0.04 (0.00, 0.09)	1.56E-01
0.03 (-0.01, 0.08)	4.24E-01	0.06 (0.02, 0.11)	**3.71E-02**
0.03 (-0.02, 0.08)	5.77E-01	0.05 (0.00, 0.10)	9.38E-02
0.01 (-0.03, 0.06)	7.72E-01	0.03 (-0.01, 0.08)	2.74E-01
-0.03 (-0.08, 0.01)	5.22E-01	-0.06 (-0.11, -0.02)	**3.99E-02**
0.02 (-0.03, 0.06)	7.72E-01	-0.02 (-0.06, 0.03)	5.56E-01
0.05 (0.01, 0.10)	3.77E-01	0.05 (0.01, 0.10)	7.66E-02
0.05 (0.00, 0.10)	3.77E-01	0.05 (0.00, 0.09)	1.25E-01
0.00 (-0.04, 0.05)	9.50E-01	0.02 (-0.02, 0.07)	4.61E-01
-0.05 (-0.10, 0.00)	3.77E-01	-0.05 (-0.09, 0.00)	1.22E-01
-0.05 (-0.09, 0.00)	3.77E-01	-0.09 (-0.14, -0.05)	**2.58E-03**
0.05 (0.00, 0.09)	3.77E-01	0.01 (-0.03, 0.06)	7.17E-01
0.05 (0.01, 0.10)	3.77E-01	0.02 (-0.02, 0.07)	3.87E-01
0.00 (-0.04, 0.05)	9.79E-01	-0.09 (-0.13, -0.04)	**3.70E-03**
-0.04 (-0.08, 0.01)	4.02E-01	0.01 (-0.03, 0.05)	7.42E-01
0.01 (-0.03, 0.06)	7.97E-01	-0.01 (-0.05, 0.04)	7.87E-01
0.04 (0.00, 0.09)	3.77E-01	0.02 (-0.03, 0.06)	5.88E-01
0.03 (-0.01, 0.08)	4.24E-01	0.03 (-0.01, 0.08)	2.31E-01
0.04 (0.00, 0.09)	3.77E-01	-0.04 (-0.08, 0.01)	2.06E-01
-0.04 (-0.08, 0.01)	3.77E-01	-0.01 (-0.05, 0.04)	7.80E-01
0.00 (-0.04, 0.05)	9.46E-01	0.00 (-0.05, 0.04)	8.89E-01
0.04 (0.00, 0.09)	3.77E-01	0.04 (-0.01, 0.08)	2.06E-01
0.02 (-0.03, 0.06)	7.68E-01	0.06 (0.02, 0.10)	**3.56E-02**
0.04 (-0.01, 0.08)	3.77E-01	-0.02 (-0.06, 0.03)	5.79E-01
-0.04 (-0.08, 0.00)	3.77E-01	-0.04 (-0.08, 0.01)	2.31E-01
-0.04 (-0.09, 0.00)	3.77E-01	-0.03 (-0.08, 0.02)	2.94E-01
0.04 (0.00, 0.09)	3.77E-01	0.04 (0.00, 0.09)	1.45E-01
0.04 (-0.01, 0.09)	3.77E-01	0.06 (0.02, 0.11)	**3.97E-02**
0.02 (-0.03, 0.06)	7.43E-01	-0.04 (-0.09, 0.00)	1.25E-01
-0.04 (-0.08, 0.01)	3.95E-01	-0.04 (-0.09, 0.00)	1.45E-01
-0.04 (-0.08, 0.01)	3.95E-01	-0.02 (-0.06, 0.03)	5.79E-01

0.04 (-0.01, 0.08)	3.77E-01	0.03 (-0.02, 0.07)	3.24E-01
0.04 (-0.01, 0.09)	3.77E-01	0.04 (-0.01, 0.08)	2.14E-01
-0.04 (-0.08, 0.01)	4.24E-01	-0.05 (-0.10, -0.01)	7.32E-02
-0.03 (-0.08, 0.01)	4.24E-01	-0.05 (-0.09, 0.00)	1.28E-01
-0.04 (-0.08, 0.01)	4.02E-01	-0.03 (-0.08, 0.02)	2.94E-01
0.03 (-0.01, 0.08)	4.24E-01	0.02 (-0.02, 0.07)	4.22E-01
0.03 (-0.01, 0.08)	4.24E-01	0.03 (-0.01, 0.08)	2.52E-01
-0.03 (-0.07, 0.02)	6.26E-01	-0.07 (-0.11, -0.02)	**3.11E-02**
-0.02 (-0.07, 0.02)	7.16E-01	-0.01 (-0.05, 0.04)	8.48E-01
0.02 (-0.03, 0.06)	7.72E-01	-0.03 (-0.08, 0.01)	2.31E-01
-0.02 (-0.07, 0.03)	7.43E-01	0.02 (-0.03, 0.06)	5.57E-01
-0.02 (-0.06, 0.03)	7.69E-01	0.03 (-0.01, 0.07)	2.84E-01
-0.02 (-0.06, 0.03)	7.43E-01	-0.04 (-0.09, 0.00)	1.28E-01
0.00 (-0.04, 0.05)	9.28E-01	0.05 (0.01, 0.10)	6.92E-02
0.01 (-0.03, 0.06)	8.07E-01	0.05 (0.00, 0.09)	1.08E-01
-0.02 (-0.06, 0.03)	7.47E-01	-0.08 (-0.13, -0.03)	**7.48E-03**
-0.03 (-0.08, 0.01)	4.24E-01	-0.08 (-0.12, -0.03)	**1.02E-02**
0.01 (-0.04, 0.06)	8.36E-01	-0.07 (-0.11, -0.02)	**2.49E-02**
0.01 (-0.03, 0.06)	8.07E-01	0.07 (0.02, 0.12)	**2.49E-02**
0.01 (-0.04, 0.05)	9.00E-01	0.03 (-0.02, 0.07)	3.36E-01
-0.01 (-0.06, 0.03)	8.07E-01	-0.07 (-0.12, -0.03)	**1.86E-02**
-0.01 (-0.06, 0.03)	7.72E-01	-0.06 (-0.10, -0.01)	5.92E-02
0.00 (-0.04, 0.05)	9.45E-01	-0.10 (-0.14, -0.05)	**2.58E-03**
0.01 (-0.03, 0.06)	7.97E-01	0.09 (0.04, 0.13)	**5.23E-03**
-0.03 (-0.08, 0.01)	4.24E-01	-0.01 (-0.06, 0.03)	7.29E-01
0.03 (-0.02, 0.07)	5.77E-01	-0.01 (-0.05, 0.04)	7.99E-01
0.03 (-0.02, 0.07)	5.80E-01	0.00 (-0.04, 0.05)	9.79E-01
-0.02 (-0.07, 0.03)	7.43E-01	-0.07 (-0.11, -0.02)	**3.01E-02**
0.00 (-0.04, 0.04)	9.96E-01	0.06 (0.01, 0.10)	5.77E-02
-0.01 (-0.06, 0.03)	8.26E-01	0.04 (0.00, 0.09)	1.28E-01
0.03 (-0.02, 0.08)	5.80E-01	0.01 (-0.04, 0.05)	8.48E-01
0.00 (-0.05, 0.04)	9.45E-01	-0.06 (-0.11, -0.02)	**3.56E-02**
0.03 (-0.01, 0.08)	4.69E-01	0.02 (-0.03, 0.06)	6.16E-01
0.02 (-0.02, 0.07)	7.16E-01	0.07 (0.02, 0.11)	**3.11E-02**
0.04 (-0.01, 0.08)	4.24E-01	0.07 (0.02, 0.11)	**3.50E-02**
0.04 (-0.01, 0.09)	3.77E-01	0.04 (-0.01, 0.08)	2.31E-01
-0.01 (-0.06, 0.03)	7.85E-01	-0.09 (-0.14, -0.05)	**2.58E-03**
-0.02 (-0.06, 0.03)	7.72E-01	-0.09 (-0.14, -0.05)	**2.58E-03**
0.00 (-0.04, 0.05)	9.45E-01	-0.07 (-0.11, -0.02)	**2.68E-02**
0.03 (-0.02, 0.07)	6.84E-01	0.02 (-0.03, 0.07)	5.81E-01
0.03 (-0.01, 0.08)	4.24E-01	0.02 (-0.03, 0.07)	5.05E-01
-0.01 (-0.06, 0.03)	8.07E-01	0.04 (0.00, 0.09)	1.35E-01
-0.01 (-0.06, 0.03)	7.72E-01	0.05 (0.00, 0.09)	1.08E-01
0.01 (-0.04, 0.05)	9.00E-01	0.00 (-0.05, 0.05)	9.39E-01
0.00 (-0.04, 0.05)	9.70E-01	0.04 (-0.01, 0.08)	2.26E-01
0.01 (-0.04, 0.06)	8.36E-01	-0.01 (-0.06, 0.04)	7.36E-01
0.00 (-0.05, 0.04)	9.28E-01	0.06 (0.01, 0.10)	5.77E-02
0.01 (-0.04, 0.05)	9.00E-01	-0.02 (-0.06, 0.03)	5.81E-01

0.01 (-0.03, 0.06)	8.07E-01	0.00 (-0.04, 0.05)	8.82E-01
0.01 (-0.04, 0.05)	9.00E-01	-0.02 (-0.06, 0.03)	5.81E-01
-0.01 (-0.05, 0.04)	9.00E-01	-0.06 (-0.11, -0.02)	**3.11E-02**
0.02 (-0.02, 0.07)	7.16E-01	0.06 (0.02, 0.11)	**4.19E-02**
0.03 (-0.02, 0.07)	6.94E-01	0.09 (0.05, 0.14)	**3.70E-03**
0.00 (-0.04, 0.05)	9.77E-01	0.03 (-0.02, 0.08)	3.00E-01
0.03 (-0.02, 0.08)	5.80E-01	0.01 (-0.03, 0.06)	6.84E-01
0.03 (-0.02, 0.08)	5.58E-01	0.03 (-0.01, 0.08)	2.23E-01
0.00 (-0.04, 0.05)	9.46E-01	0.03 (-0.02, 0.08)	3.43E-01
0.02 (-0.02, 0.07)	7.16E-01	0.05 (0.00, 0.09)	1.03E-01
0.01 (-0.04, 0.05)	9.00E-01	0.03 (-0.02, 0.08)	3.82E-01
0.01 (-0.04, 0.06)	8.26E-01	0.03 (-0.02, 0.08)	2.84E-01
-0.01 (-0.05, 0.04)	9.00E-01	0.02 (-0.02, 0.07)	4.43E-01
0.00 (-0.04, 0.05)	9.70E-01	0.03 (-0.01, 0.08)	2.70E-01
0.04 (-0.00, 0.09)	3.77E-01	0.01 (-0.03, 0.06)	6.96E-01
0.00 (-0.04, 0.05)	9.45E-01	0.00 (-0.04, 0.05)	8.89E-01
0.05 (-0.01, 0.10)	3.77E-01	0.04 (-0.01, 0.08)	1.93E-01
0.01 (-0.04, 0.05)	9.00E-01	-0.01 (-0.05, 0.04)	8.48E-01
0.02 (-0.02, 0.07)	7.16E-01	0.01 (-0.04, 0.05)	8.34E-01
-0.02 (-0.06, 0.03)	7.43E-01	-0.01 (-0.06, 0.03)	7.17E-01
-0.01 (-0.06, 0.04)	8.26E-01	0.00 (-0.04, 0.05)	8.74E-01
0.00 (-0.05, 0.04)	9.90E-01	0.01 (-0.04, 0.05)	7.99E-01
-0.02 (-0.07, 0.02)	7.29E-01	0.01 (-0.03, 0.06)	6.43E-01
-0.01 (-0.05, 0.04)	9.00E-01	-0.02 (-0.06, 0.03)	6.05E-01
0.03 (-0.01, 0.08)	4.24E-01	0.00 (-0.05, 0.04)	9.68E-01
0.02 (-0.02, 0.07)	7.16E-01	-0.01 (-0.06, 0.03)	6.76E-01
0.04 (-0.00, 0.09)	3.77E-01	0.03 (-0.02, 0.07)	3.27E-01
0.00 (-0.04, 0.05)	9.87E-01	0.04 (0.00, 0.09)	1.28E-01
0.01 (-0.03, 0.06)	8.26E-01	0.03 (-0.01, 0.08)	2.23E-01
0.01 (-0.04, 0.05)	9.00E-01	0.03 (-0.02, 0.07)	3.38E-01
0.02 (-0.03, 0.06)	7.43E-01	0.06 (0.02, 0.10)	**3.56E-02**
0.00 (-0.04, 0.04)	9.96E-01	0.05 (0.01, 0.10)	7.40E-02
0.00 (-0.05, 0.04)	9.46E-01	0.02 (-0.02, 0.07)	4.50E-01
-0.06 (-0.10, -0.01)	3.77E-01	0.01 (-0.04, 0.05)	7.76E-01
-0.08 (-0.12, -0.03)	2.80E-01	-0.02 (-0.06, 0.03)	5.65E-01
-0.01 (-0.06, 0.03)	8.26E-01	0.01 (-0.04, 0.05)	8.20E-01
0.00 (-0.04, 0.05)	9.96E-01	-0.02 (-0.07, 0.02)	4.22E-01
-0.02 (-0.06, 0.03)	7.72E-01	0.06 (0.02, 0.10)	**3.97E-02**

self-rated health status. P values are corrected for multiple testing using a false discovery rat
ow Density Lipoprotein; IDL, Intermediate Density Lipoprotein..

in subgroups analysis

Age < 60y		Age >= 60y	
**Adjusted β (95% CI)**	**P value**	**Adjusted β (95% CI)**	**P value**
0.01 (-0.02, 0.04)	7.13E-01	0.02 (-0.06, 0.11)	8.59E-01
0.01 (-0.02, 0.04)	6.99E-01	0.02 (-0.06, 0.12)	8.59E-01
0.01 (-0.03, 0.04)	7.64E-01	0.02 (-0.07, 0.10)	9.00E-01
0.02 (-0.01, 0.05)	4.40E-01	0.03 (-0.06, 0.12)	8.02E-01
0.03 (-0.00, 0.07)	2.66E-01	0.04 (-0.05, 0.13)	7.82E-01
0.01 (-0.03, 0.04)	7.78E-01	0.01 (-0.08, 0.10)	9.33E-01
0.01 (-0.03, 0.04)	7.56E-01	0.02 (-0.06, 0.11)	8.66E-01
0.01 (-0.03, 0.04)	7.79E-01	0.01 (-0.07, 0.10)	9.11E-01
0.01 (-0.03, 0.04)	7.56E-01	0.01 (-0.07, 0.10)	9.08E-01
0.01 (-0.02, 0.05)	6.85E-01	0.01 (-0.07, 0.10)	9.08E-01
0.02 (-0.02, 0.05)	5.17E-01	0.02 (-0.07, 0.11)	8.75E-01
0.02 (-0.01, 0.05)	4.40E-01	0.02 (-0.06, 0.11)	8.58E-01
0.01 (-0.02, 0.05)	6.15E-01	0.01 (-0.08, 0.10)	9.33E-01
0.00 (-0.03, 0.04)	8.32E-01	0.01 (-0.08, 0.10)	9.33E-01
0.01 (-0.03, 0.04)	8.08E-01	0.02 (-0.06, 0.11)	8.54E-01
0.01 (-0.03, 0.04)	8.05E-01	0.03 (-0.06, 0.11)	8.54E-01
0.01 (-0.03, 0.04)	7.91E-01	0.03 (-0.06, 0.11)	8.54E-01
0.01 (-0.02, 0.05)	6.30E-01	0.03 (-0.05, 0.12)	7.95E-01
0.02 (-0.01, 0.05)	4.45E-01	0.04 (-0.04, 0.13)	7.76E-01
0.01 (-0.03, 0.04)	8.08E-01	0.02 (-0.07, 0.11)	8.66E-01
0.00 (-0.03, 0.04)	8.61E-01	0.02 (-0.06, 0.11)	8.59E-01
0.01 (-0.02, 0.04)	6.85E-01	0.04 (-0.04, 0.13)	7.68E-01
0.01 (-0.02, 0.05)	6.55E-01	0.04 (-0.04, 0.13)	7.68E-01
0.01 (-0.02, 0.05)	6.30E-01	0.04 (-0.04, 0.13)	7.68E-01
0.02 (-0.01, 0.06)	3.61E-01	0.05 (-0.04, 0.14)	7.40E-01
0.04 (-0.00, 0.07)	2.07E-01	0.06 (-0.03, 0.15)	6.07E-01
0.01 (-0.02, 0.04)	6.91E-01	0.04 (-0.05, 0.12)	7.95E-01
0.01 (-0.03, 0.04)	8.08E-01	0.04 (-0.05, 0.13)	7.95E-01
0.02 (-0.01, 0.05)	4.59E-01	0.04 (-0.04, 0.13)	7.76E-01
0.02 (-0.01, 0.06)	4.27E-01	0.04 (-0.04, 0.13)	7.70E-01
0.02 (-0.02, 0.05)	5.74E-01	0.04 (-0.05, 0.12)	7.95E-01
0.04 (0.00, 0.07)	2.05E-01	0.06 (-0.03, 0.15)	7.15E-01
0.05 (0.01, 0.08)	1.59E-01	0.07 (-0.02, 0.16)	5.42E-01
0.02 (-0.02, 0.05)	5.17E-01	0.03 (-0.06, 0.12)	8.37E-01
0.01 (-0.02, 0.04)	6.85E-01	0.03 (-0.05, 0.12)	7.95E-01
0.03 (-0.01, 0.06)	2.88E-01	0.02 (-0.07, 0.11)	8.66E-01
0.03 (0.00, 0.06)	2.68E-01	0.02 (-0.07, 0.11)	8.66E-01
0.02 (-0.01, 0.06)	3.95E-01	0.02 (-0.07, 0.11)	8.59E-01
0.04 (0.00, 0.07)	2.05E-01	0.01 (-0.07, 0.10)	9.08E-01
0.04 (0.01, 0.08)	1.69E-01	0.01 (-0.07, 0.10)	9.08E-01
0.02 (-0.01, 0.06)	3.95E-01	0.01 (-0.07, 0.10)	9.08E-01
0.01 (-0.03, 0.04)	7.91E-01	0.01 (-0.08, 0.10)	9.24E-01
0.03 (0.00, 0.07)	2.40E-01	0.01 (-0.08, 0.10)	9.14E-01

0.03 (0.00, 0.07)	2.40E-01	0.01 (-0.08, 0.10)	9.11E-01
0.03 (0.00, 0.07)	2.57E-01	0.02 (-0.07, 0.11)	8.66E-01
0.03 (0.00, 0.08)	2.40E-01	0.02 (-0.07, 0.11)	8.99E-01
0.04 (0.00, 0.09)	2.14E-01	0.02 (-0.07, 0.11)	8.66E-01
0.03 (-0.01, 0.06)	3.29E-01	0.01 (-0.08, 0.10)	9.44E-01
0.00 (-0.03, 0.04)	8.69E-01	-0.02 (-0.11, 0.07)	8.66E-01
0.03 (0.00, 0.07)	2.57E-01	0.03 (-0.06, 0.12)	8.47E-01
0.03 (0.00, 0.07)	2.57E-01	0.03 (-0.06, 0.12)	8.45E-01
0.03 (0.00, 0.07)	2.57E-01	0.03 (-0.06, 0.12)	8.40E-01
0.03 (0.00, 0.07)	2.57E-01	0.04 (-0.05, 0.13)	7.95E-01
0.03 (0.00, 0.07)	2.40E-01	0.04 (-0.05, 0.13)	7.95E-01
0.03 (-0.01, 0.06)	2.88E-01	0.02 (-0.07, 0.11)	8.66E-01
0.01 (-0.02, 0.04)	7.42E-01	-0.04 (-0.13, 0.05)	7.95E-01
0.03 (0.00, 0.07)	2.68E-01	0.04 (-0.05, 0.13)	7.95E-01
0.03 (0.00, 0.07)	2.68E-01	0.04 (-0.05, 0.13)	7.95E-01
0.04 (0.00, 0.07)	2.07E-01	0.02 (-0.07, 0.11)	8.66E-01
0.03 (-0.01, 0.06)	3.02E-01	0.05 (-0.04, 0.14)	7.68E-01
0.03 (-0.01, 0.06)	3.14E-01	0.06 (-0.03, 0.15)	7.33E-01
0.03 (0.00, 0.07)	2.68E-01	0.01 (-0.08, 0.10)	9.11E-01
0.02 (-0.02, 0.05)	5.58E-01	-0.05 (-0.14, 0.04)	7.55E-01
0.03 (-0.01, 0.06)	3.30E-01	0.03 (-0.06, 0.12)	7.95E-01
0.03 (-0.01, 0.06)	3.39E-01	0.03 (-0.06, 0.12)	7.95E-01
0.03 (-0.01, 0.06)	3.02E-01	0.00 (-0.09, 0.09)	9.70E-01
0.02 (-0.01, 0.06)	4.08E-01	0.05 (-0.04, 0.14)	7.68E-01
0.02 (-0.01, 0.05)	4.60E-01	0.05 (-0.04, 0.14)	7.39E-01
0.02 (-0.01, 0.06)	3.98E-01	0.01 (-0.08, 0.10)	9.44E-01
0.03 (-0.01, 0.06)	3.33E-01	-0.02 (-0.11, 0.06)	8.58E-01
-0.01 (-0.05, 0.02)	6.16E-01	-0.12 (-0.20, -0.03)	1.19E-01
-0.01 (-0.05, 0.02)	6.05E-01	-0.12 (-0.20, -0.03)	1.19E-01
-0.01 (-0.05, 0.02)	5.74E-01	-0.12 (-0.20, -0.03)	1.19E-01
-0.01 (-0.05, 0.02)	5.74E-01	-0.11 (-0.19, -0.03)	1.39E-01
-0.01 (-0.05, 0.02)	6.11E-01	-0.10 (-0.19, -0.02)	1.67E-01
-0.02 (-0.05, 0.02)	5.43E-01	-0.12 (-0.21, -0.04)	1.19E-01
0.02 (-0.01, 0.06)	4.20E-01	-0.05 (-0.14, 0.04)	7.60E-01
-0.02 (-0.06, 0.01)	3.54E-01	-0.13 (-0.22, -0.04)	1.19E-01
-0.03 (-0.06, 0.01)	3.43E-01	-0.13 (-0.22, -0.04)	1.19E-01
-0.03 (-0.06, 0.01)	3.35E-01	-0.13 (-0.21, -0.04)	1.19E-01
-0.03 (-0.06, 0.01)	3.02E-01	-0.13 (-0.22, -0.04)	1.19E-01
-0.03 (-0.06, 0.01)	3.02E-01	-0.13 (-0.22, -0.05)	1.19E-01
-0.03 (-0.06, 0.01)	3.34E-01	-0.13 (-0.21, -0.04)	1.19E-01
0.02 (-0.02, 0.05)	5.17E-01	-0.08 (-0.17, 0.01)	4.34E-01
-0.04 (-0.07, 0.00)	2.05E-01	-0.07 (-0.16, 0.02)	4.73E-01
-0.04 (-0.07, 0.00)	2.04E-01	-0.08 (-0.16, 0.01)	4.38E-01
-0.04 (-0.07, 0.00)	2.14E-01	-0.08 (-0.17, 0.01)	4.34E-01
-0.05 (-0.08, -0.01)	1.67E-01	-0.08 (-0.17, 0.01)	4.34E-01
-0.05 (-0.08, -0.01)	1.59E-01	-0.08 (-0.16, 0.01)	4.34E-01
-0.04 (-0.08, -0.01)	2.03E-01	-0.09 (-0.17, 0.00)	3.69E-01
0.02 (-0.01, 0.05)	4.45E-01	0.04 (-0.05, 0.13)	7.95E-01

-0.03 (-0.06, 0.01)	3.02E-01	0.00 (-0.09, 0.08)	9.70E-01
-0.03 (-0.06, 0.01)	2.88E-01	-0.01 (-0.09, 0.08)	9.58E-01
-0.03 (-0.06, 0.00)	2.68E-01	-0.03 (-0.12, 0.06)	8.47E-01
-0.01 (-0.05, 0.02)	6.30E-01	0.02 (-0.07, 0.10)	8.98E-01
-0.01 (-0.04, 0.03)	8.23E-01	0.03 (-0.06, 0.11)	8.47E-01
-0.04 (-0.07, 0.00)	2.04E-01	-0.05 (-0.14, 0.04)	7.40E-01
0.01 (-0.02, 0.05)	6.63E-01	0.03 (-0.05, 0.12)	7.95E-01
-0.01 (-0.04, 0.03)	8.23E-01	0.01 (-0.08, 0.10)	9.34E-01
0.03 (0.00, 0.06)	2.86E-01	0.05 (-0.04, 0.13)	7.68E-01
0.03 (0.00, 0.07)	2.68E-01	0.05 (-0.04, 0.13)	7.68E-01
0.01 (-0.03, 0.04)	7.56E-01	0.02 (-0.07, 0.10)	9.08E-01
-0.02 (-0.06, 0.01)	3.71E-01	-0.04 (-0.13, 0.04)	7.68E-01
0.04 (0.00, 0.07)	2.07E-01	0.03 (-0.06, 0.12)	8.48E-01
0.06 (0.02, 0.09)	8.91E-02	0.10 (-0.01, 0.19)	2.74E-01
0.03 (-0.01, 0.06)	3.02E-01	0.08 (-0.01, 0.16)	4.34E-01
0.05 (0.02, 0.09)	1.57E-01	0.07 (-0.02, 0.16)	5.20E-01
-0.06 (-0.10, -0.03)	**4.68E-02**	-0.10 (-0.19, -0.01)	2.66E-01
0.02 (-0.02, 0.05)	5.61E-01	0.04 (-0.05, 0.13)	7.95E-01
0.04 (0.01, 0.08)	1.69E-01	0.05 (-0.04, 0.14)	7.68E-01
0.04 (0.01, 0.08)	1.88E-01	0.03 (-0.06, 0.12)	8.48E-01
0.02 (-0.02, 0.05)	5.74E-01	0.03 (-0.06, 0.11)	8.47E-01
-0.04 (-0.07, 0.00)	2.04E-01	-0.05 (-0.14, 0.04)	7.68E-01
0.00 (-0.03, 0.04)	9.00E-01	-0.02 (-0.11, 0.06)	8.58E-01
0.05 (0.02, 0.09)	1.27E-01	0.03 (-0.06, 0.12)	8.48E-01
0.05 (0.02, 0.09)	1.52E-01	0.02 (-0.07, 0.11)	8.59E-01
0.01 (-0.03, 0.04)	7.91E-01	0.01 (-0.08, 0.10)	9.44E-01
-0.05 (-0.08, -0.02)	1.57E-01	-0.02 (-0.11, 0.07)	8.66E-01
-0.06 (-0.09, -0.03)	**4.68E-02**	-0.09 (-0.18, -0.01)	2.90E-01
0.03 (0.00, 0.06)	2.66E-01	0.02 (-0.07, 0.11)	8.66E-01
0.04 (0.00, 0.07)	2.05E-01	0.03 (-0.05, 0.12)	7.95E-01
-0.03 (-0.07, 0.00)	2.14E-01	-0.09 (-0.18, 0.00)	2.90E-01
-0.02 (-0.05, 0.02)	5.52E-01	0.00 (-0.08, 0.09)	9.90E-01
0.00 (-0.03, 0.03)	9.59E-01	0.01 (-0.08, 0.10)	9.33E-01
0.04 (0.00, 0.07)	2.05E-01	-0.01 (-0.09, 0.08)	9.58E-01
0.04 (0.01, 0.08)	1.69E-01	-0.01 (-0.09, 0.08)	9.58E-01
0.00 (-0.03, 0.04)	8.32E-01	0.00 (-0.09, 0.08)	9.75E-01
-0.03 (-0.06, 0.00)	2.86E-01	0.00 (-0.09, 0.08)	9.98E-01
0.00 (-0.04, 0.03)	8.32E-01	0.05 (-0.04, 0.14)	7.40E-01
0.04 (0.01, 0.08)	1.69E-01	0.00 (-0.08, 0.09)	9.70E-01
0.04 (0.01, 0.07)	2.03E-01	0.01 (-0.08, 0.10)	9.31E-01
0.02 (-0.02, 0.05)	5.45E-01	-0.01 (-0.09, 0.08)	9.44E-01
-0.04 (-0.07, -0.01)	2.03E-01	-0.02 (-0.11, 0.07)	8.66E-01
-0.04 (-0.07, -0.01)	2.03E-01	0.01 (-0.08, 0.10)	9.33E-01
0.04 (0.01, 0.08)	1.90E-01	0.03 (-0.06, 0.12)	7.95E-01
0.05 (0.01, 0.08)	1.59E-01	0.04 (-0.05, 0.13)	7.95E-01
-0.01 (-0.04, 0.03)	8.19E-01	-0.02 (-0.11, 0.07)	8.66E-01
-0.03 (-0.07, 0.00)	2.33E-01	-0.06 (-0.15, 0.03)	6.51E-01
-0.02 (-0.05, 0.02)	5.43E-01	-0.06 (-0.15, 0.03)	6.51E-01

0.02 (-0.01, 0.05)	4.45E-01	0.08 (-0.01, 0.17)	4.20E-01
0.02 (-0.01, 0.06)	3.75E-01	0.09 (-0.00, 0.17)	3.69E-01
-0.03 (-0.06, 0.00)	2.86E-01	-0.09 (-0.18, 0.00)	3.53E-01
-0.02 (-0.06, 0.01)	3.97E-01	-0.10 (-0.19, -0.01)	2.35E-01
-0.02 (-0.05, 0.02)	5.17E-01	-0.09 (-0.18, 0.00)	2.90E-01
0.01 (-0.02, 0.05)	6.30E-01	0.09 (-0.00, 0.18)	2.90E-01
0.02 (-0.02, 0.05)	5.43E-01	0.10 (-0.01, 0.19)	2.40E-01
-0.03 (-0.06, 0.01)	2.88E-01	-0.11 (-0.20, -0.02)	1.67E-01
0.00 (-0.04, 0.03)	9.12E-01	-0.08 (-0.16, 0.01)	4.34E-01
0.00 (-0.04, 0.03)	8.89E-01	0.00 (-0.09, 0.08)	9.70E-01
0.00 (-0.04, 0.03)	8.34E-01	-0.02 (-0.10, 0.07)	8.96E-01
0.00 (-0.04, 0.03)	9.09E-01	0.01 (-0.07, 0.10)	9.11E-01
-0.01 (-0.05, 0.02)	6.30E-01	-0.11 (-0.20, -0.03)	1.32E-01
0.02 (-0.01, 0.05)	4.27E-01	0.05 (-0.04, 0.13)	7.55E-01
0.01 (-0.03, 0.04)	8.14E-01	0.12 (-0.03, 0.20)	1.34E-01
-0.02 (-0.06, 0.01)	3.61E-01	-0.13 (-0.21, -0.04)	1.19E-01
-0.03 (-0.07, 0.00)	2.58E-01	-0.13 (-0.22, -0.04)	1.19E-01
-0.01 (-0.04, 0.03)	7.56E-01	-0.10 (-0.19, -0.01)	2.35E-01
0.03 (0.00, 0.07)	2.26E-01	0.07 (-0.02, 0.15)	5.75E-01
0.02 (-0.01, 0.05)	4.45E-01	0.00 (-0.09, 0.08)	9.70E-01
-0.04 (-0.07, 0.00)	2.07E-01	-0.05 (-0.14, 0.04)	7.42E-01
-0.03 (-0.07, 0.00)	2.57E-01	-0.03 (-0.12, 0.05)	7.95E-01
-0.04 (-0.07, 0.00)	2.08E-01	-0.08 (-0.17, 0.01)	3.83E-01
0.04 (0.01, 0.07)	2.03E-01	0.08 (-0.01, 0.16)	4.34E-01
-0.01 (-0.05, 0.02)	5.77E-01	-0.05 (-0.14, 0.04)	7.68E-01
0.01 (-0.03, 0.04)	8.08E-01	0.03 (-0.06, 0.12)	8.47E-01
0.01 (-0.03, 0.04)	7.50E-01	0.04 (-0.05, 0.12)	7.95E-01
-0.03 (-0.07, 0.00)	2.68E-01	-0.09 (-0.18, 0.00)	2.98E-01
0.02 (-0.01, 0.06)	4.08E-01	0.04 (-0.05, 0.12)	7.95E-01
0.01 (-0.03, 0.04)	7.56E-01	0.03 (-0.05, 0.12)	7.95E-01
0.03 (0.00, 0.07)	2.40E-01	-0.04 (-0.12, 0.05)	7.95E-01
-0.01 (-0.05, 0.02)	6.41E-01	-0.11 (-0.19, -0.02)	1.67E-01
0.02 (-0.01, 0.06)	4.20E-01	-0.01 (-0.10, 0.08)	9.50E-01
0.03 (0.00, 0.07)	2.34E-01	0.05 (-0.04, 0.14)	7.68E-01
0.04 (0.01, 0.08)	2.03E-01	0.04 (-0.05, 0.13)	7.95E-01
0.03 (-0.01, 0.06)	2.88E-01	0.03 (-0.05, 0.12)	7.95E-01
-0.04 (-0.07, 0.00)	2.07E-01	-0.12 (-0.20, -0.03)	1.19E-01
-0.04 (-0.07, 0.00)	2.05E-01	-0.12 (-0.20, -0.03)	1.19E-01
-0.02 (-0.05, 0.01)	4.40E-01	-0.09 (-0.17, 0.00)	2.90E-01
0.02 (-0.02, 0.05)	5.45E-01	0.00 (-0.09, 0.09)	9.75E-01
0.03 (-0.01, 0.06)	3.40E-01	-0.01 (-0.10, 0.08)	9.44E-01
0.01 (-0.03, 0.04)	7.91E-01	0.02 (-0.06, 0.11)	8.54E-01
0.01 (-0.03, 0.04)	8.08E-01	0.03 (-0.05, 0.12)	7.95E-01
0.00 (-0.03, 0.04)	8.95E-01	-0.03 (-0.12, 0.05)	7.95E-01
0.02 (-0.01, 0.05)	4.45E-01	-0.01 (-0.10, 0.09)	9.58E-01
0.01 (-0.03, 0.04)	7.79E-01	-0.06 (-0.15, 0.02)	5.99E-01
0.02 (-0.02, 0.05)	5.17E-01	0.03 (-0.05, 0.12)	7.95E-01
0.00 (-0.03, 0.04)	8.71E-01	-0.07 (-0.16, 0.02)	4.73E-01

0.01 (-0.03, 0.04)	8.19E-01	-0.01 (-0.10, 0.08)	9.33E-01
0.00 (-0.03, 0.04)	8.58E-01	-0.07 (-0.16, 0.02)	4.85E-01
-0.02 (-0.06, 0.01)	4.00E-01	-0.10 (-0.19, -0.01)	2.40E-01
0.03 (0.00, 0.07)	2.58E-01	0.04 (-0.05, 0.13)	7.95E-01
0.04 (0.01, 0.08)	1.94E-01	0.09 (0.00, 0.18)	3.69E-01
0.01 (-0.02, 0.05)	6.36E-01	-0.01 (-0.10, 0.08)	9.11E-01
0.03 (-0.01, 0.06)	3.34E-01	0.00 (-0.09, 0.09)	9.75E-01
0.02 (-0.01, 0.06)	3.47E-01	0.05 (-0.04, 0.14)	7.39E-01
0.02 (-0.02, 0.05)	5.43E-01	-0.03 (-0.13, 0.06)	7.95E-01
0.03 (-0.01, 0.06)	3.02E-01	0.04 (-0.05, 0.13)	7.95E-01
0.02 (-0.02, 0.05)	5.52E-01	-0.03 (-0.12, 0.07)	8.54E-01
0.02 (-0.02, 0.06)	4.69E-01	-0.02 (-0.11, 0.08)	9.08E-01
0.01 (-0.03, 0.04)	8.08E-01	-0.02 (-0.11, 0.07)	8.66E-01
0.01 (-0.02, 0.05)	6.85E-01	0.00 (-0.09, 0.09)	9.75E-01
0.02 (-0.02, 0.05)	5.23E-01	0.08 (-0.01, 0.17)	3.83E-01
0.02 (-0.02, 0.05)	5.74E-01	-0.05 (-0.14, 0.04)	7.39E-01
0.04 (0.00, 0.07)	2.14E-01	0.08 (0.00, 0.17)	3.69E-01
0.01 (-0.03, 0.04)	7.56E-01	-0.04 (-0.12, 0.05)	7.95E-01
0.02 (-0.02, 0.06)	4.62E-01	-0.01 (-0.10, 0.08)	9.33E-01
-0.01 (-0.04, 0.02)	6.85E-01	-0.03 (-0.12, 0.06)	8.47E-01
-0.02 (-0.05, 0.02)	5.43E-01	0.05 (-0.03, 0.14)	7.39E-01
0.01 (-0.02, 0.05)	6.15E-01	-0.05 (-0.13, 0.04)	7.68E-01
-0.02 (-0.05, 0.02)	5.61E-01	0.05 (-0.03, 0.14)	7.15E-01
-0.03 (-0.06, 0.01)	3.02E-01	0.06 (-0.03, 0.15)	6.51E-01
0.00 (-0.03, 0.03)	9.42E-01	0.07 (-0.01, 0.16)	4.36E-01
-0.01 (-0.04, 0.03)	7.79E-01	0.03 (-0.05, 0.12)	7.95E-01
0.03 (-0.01, 0.06)	3.20E-01	0.06 (-0.02, 0.15)	5.94E-01
0.02 (-0.02, 0.05)	5.74E-01	0.03 (-0.05, 0.12)	7.95E-01
0.01 (-0.03, 0.05)	7.24E-01	0.07 (-0.02, 0.15)	5.20E-01
0.01 (-0.03, 0.04)	8.08E-01	0.04 (-0.04, 0.13)	7.86E-01
0.05 (0.01, 0.08)	1.59E-01	0.02 (-0.07, 0.11)	8.66E-01
0.02 (-0.01, 0.06)	3.54E-01	0.03 (-0.06, 0.11)	8.47E-01
-0.01 (-0.05, 0.02)	6.36E-01	0.08 (-0.01, 0.17)	4.34E-01
-0.02 (-0.06, 0.01)	4.40E-01	-0.06 (-0.14, 0.03)	6.20E-01
-0.05 (-0.08, -0.01)	1.59E-01	-0.05 (-0.14, 0.03)	7.15E-01
0.00 (-0.04, 0.03)	8.58E-01	-0.01 (-0.10, 0.08)	9.50E-01
-0.03 (-0.06, 0.01)	2.88E-01	0.05 (-0.03, 0.14)	7.39E-01
0.02 (-0.01, 0.06)	4.20E-01	0.01 (-0.08, 0.10)	9.43E-01

e (FDR) adjustment.

Rural		Urban	
Adjusted β (95% CI)	P value	Adjusted β (95% CI)	P value
0.00 (-0.04, 0.04)	9.88E-01	0.02 (-0.03, 0.07)	9.70E-01
0.00 (-0.04, 0.04)	9.88E-01	0.02 (-0.03, 0.07)	9.70E-01
0.00 (-0.05, 0.04)	9.25E-01	0.02 (-0.03, 0.07)	9.70E-01
0.01 (-0.03, 0.05)	8.69E-01	0.03 (-0.03, 0.08)	9.70E-01
0.02 (-0.02, 0.06)	4.89E-01	0.03 (-0.02, 0.08)	9.70E-01
-0.01 (-0.05, 0.04)	9.23E-01	0.01 (-0.04, 0.07)	9.70E-01
0.00 (-0.04, 0.04)	9.74E-01	0.02 (-0.03, 0.07)	9.70E-01
-0.01 (-0.05, 0.04)	9.23E-01	0.02 (-0.03, 0.07)	9.70E-01
0.00 (-0.04, 0.04)	9.30E-01	0.02 (-0.03, 0.07)	9.70E-01
0.00 (-0.04, 0.04)	9.74E-01	0.02 (-0.03, 0.07)	9.70E-01
0.01 (-0.04, 0.05)	9.23E-01	0.02 (-0.03, 0.07)	9.70E-01
0.01 (-0.03, 0.05)	8.88E-01	0.03 (-0.02, 0.08)	9.70E-01
0.00 (-0.04, 0.04)	9.88E-01	0.02 (-0.03, 0.07)	9.70E-01
-0.01 (-0.05, 0.03)	9.04E-01	0.02 (-0.04, 0.07)	9.70E-01
-0.01 (-0.05, 0.03)	9.23E-01	0.02 (-0.03, 0.07)	9.70E-01
-0.01 (-0.05, 0.03)	9.23E-01	0.02 (-0.03, 0.07)	9.70E-01
0.00 (-0.04, 0.04)	9.23E-01	0.02 (-0.03, 0.07)	9.70E-01
0.00 (-0.04, 0.04)	9.88E-01	0.02 (-0.03, 0.07)	9.70E-01
0.01 (-0.03, 0.05)	8.88E-01	0.03 (-0.02, 0.08)	9.70E-01
-0.01 (-0.05, 0.03)	9.23E-01	0.02 (-0.04, 0.07)	9.70E-01
-0.01 (-0.05, 0.03)	9.04E-01	0.01 (-0.04, 0.06)	9.70E-01
0.01 (-0.03, 0.05)	9.23E-01	0.01 (-0.04, 0.06)	9.70E-01
0.01 (-0.03, 0.05)	9.23E-01	0.01 (-0.04, 0.06)	9.70E-01
0.01 (-0.03, 0.05)	9.23E-01	0.01 (-0.04, 0.06)	9.70E-01
0.02 (-0.02, 0.06)	5.71E-01	0.02 (-0.03, 0.07)	9.70E-01
0.03 (-0.01, 0.07)	2.45E-01	0.03 (-0.02, 0.08)	9.70E-01
0.00 (-0.04, 0.04)	9.37E-01	0.01 (-0.04, 0.06)	9.70E-01
0.00 (-0.04, 0.04)	9.88E-01	0.01 (-0.04, 0.06)	9.70E-01
0.02 (-0.02, 0.06)	6.53E-01	0.01 (-0.04, 0.06)	9.70E-01
0.02 (-0.02, 0.06)	5.68E-01	0.01 (-0.04, 0.06)	9.70E-01
0.01 (-0.03, 0.05)	8.44E-01	0.00 (-0.05, 0.05)	9.70E-01
0.05 (-0.01, 0.09)	7.04E-02	0.00 (-0.05, 0.05)	9.70E-01
0.07 (-0.03, 0.11)	**2.22E-02**	0.00 (-0.05, 0.05)	9.70E-01
0.02 (-0.02, 0.06)	6.53E-01	0.00 (-0.05, 0.05)	9.71E-01
0.00 (-0.04, 0.04)	9.88E-01	0.01 (-0.04, 0.06)	9.70E-01
0.03 (-0.01, 0.07)	2.60E-01	-0.01 (-0.06, 0.04)	9.70E-01
0.04 (0.00, 0.08)	2.03E-01	-0.01 (-0.06, 0.04)	9.70E-01
0.03 (-0.01, 0.07)	3.55E-01	0.00 (-0.05, 0.05)	9.70E-01
0.05 (0.01, 0.10)	6.03E-02	-0.02 (-0.07, 0.03)	9.70E-01
0.06 (0.02, 0.10)	**3.07E-02**	-0.02 (-0.07, 0.03)	9.70E-01
0.03 (-0.01, 0.07)	2.22E-01	-0.02 (-0.07, 0.03)	9.70E-01
-0.01 (-0.05, 0.03)	9.23E-01	0.01 (-0.04, 0.06)	9.70E-01
0.03 (-0.01, 0.07)	2.69E-01	0.01 (-0.04, 0.06)	9.70E-01

0.03 (-0.01, 0.07)	2.42E-01	0.01 (-0.04, 0.06)	9.70E-01
0.04 (0.00, 0.08)	2.03E-01	0.01 (-0.04, 0.06)	9.70E-01
0.04 (0.00, 0.08)	1.74E-01	0.00 (-0.05, 0.05)	9.81E-01
0.04 (0.00, 0.08)	1.67E-01	0.00 (-0.05, 0.05)	9.70E-01
0.03 (-0.01, 0.08)	2.22E-01	0.00 (-0.06, 0.05)	9.70E-01
-0.02 (-0.06, 0.02)	5.72E-01	0.01 (-0.04, 0.06)	9.70E-01
0.04 (0.00, 0.08)	2.03E-01	0.01 (-0.04, 0.06)	9.70E-01
0.04 (0.00, 0.08)	1.95E-01	0.01 (-0.04, 0.06)	9.70E-01
0.04 (0.00, 0.08)	2.11E-01	0.01 (-0.04, 0.06)	9.70E-01
0.04 (0.00, 0.08)	1.51E-01	0.00 (-0.05, 0.05)	9.70E-01
0.04 (0.00, 0.08)	1.43E-01	0.01 (-0.04, 0.06)	9.70E-01
0.04 (0.00, 0.08)	1.91E-01	0.00 (-0.05, 0.05)	9.80E-01
-0.02 (-0.06, 0.03)	6.53E-01	0.01 (-0.04, 0.06)	9.70E-01
0.04 (0.00, 0.08)	1.97E-01	0.01 (-0.04, 0.06)	9.70E-01
0.04 (0.00, 0.08)	1.95E-01	0.01 (-0.04, 0.06)	9.70E-01
0.03 (-0.01, 0.07)	2.89E-01	0.02 (-0.03, 0.07)	9.70E-01
0.04 (0.00, 0.08)	1.60E-01	0.00 (-0.05, 0.05)	9.71E-01
0.04 (0.00, 0.08)	1.43E-01	0.00 (-0.05, 0.05)	9.80E-01
0.03 (-0.01, 0.07)	2.69E-01	0.01 (-0.04, 0.06)	9.70E-01
-0.01 (-0.05, 0.03)	8.44E-01	0.01 (-0.04, 0.06)	9.70E-01
0.03 (-0.01, 0.07)	2.52E-01	0.00 (-0.05, 0.05)	9.81E-01
0.03 (-0.01, 0.07)	2.45E-01	0.00 (-0.05, 0.05)	9.99E-01
0.02 (-0.02, 0.06)	6.16E-01	0.01 (-0.04, 0.06)	9.70E-01
0.04 (0.00, 0.08)	1.91E-01	0.00 (-0.05, 0.05)	9.70E-01
0.04 (0.00, 0.08)	1.74E-01	-0.01 (-0.06, 0.04)	9.70E-01
0.02 (-0.02, 0.06)	4.52E-01	0.00 (-0.05, 0.05)	9.70E-01
0.00 (-0.04, 0.04)	9.74E-01	0.01 (-0.04, 0.06)	9.70E-01
-0.03 (-0.07, 0.01)	4.00E-01	-0.04 (-0.09, 0.01)	9.70E-01
-0.03 (-0.07, 0.01)	3.95E-01	-0.04 (-0.09, 0.01)	9.70E-01
-0.03 (-0.07, 0.01)	3.31E-01	-0.04 (-0.09, 0.01)	9.70E-01
-0.02 (-0.06, 0.02)	4.80E-01	-0.05 (-0.10, 0.00)	9.70E-01
-0.02 (-0.06, 0.02)	5.71E-01	-0.05 (-0.10, 0.00)	9.70E-01
-0.03 (-0.07, 0.01)	3.00E-01	-0.05 (-0.09, 0.00)	9.70E-01
0.01 (-0.04, 0.05)	9.23E-01	0.00 (-0.05, 0.05)	9.71E-01
-0.04 (-0.08, 0.00)	1.72E-01	-0.04 (-0.09, 0.01)	9.70E-01
-0.04 (-0.08, 0.00)	1.69E-01	-0.04 (-0.09, 0.01)	9.70E-01
-0.04 (-0.08, 0.00)	1.92E-01	-0.04 (-0.09, 0.01)	9.70E-01
-0.04 (-0.08, 0.00)	1.43E-01	-0.04 (-0.09, 0.01)	9.70E-01
-0.04 (-0.09, 0.00)	1.30E-01	-0.04 (-0.09, 0.01)	9.70E-01
-0.04 (-0.08, 0.00)	1.80E-01	-0.04 (-0.09, 0.01)	9.70E-01
-0.01 (-0.05, 0.03)	9.04E-01	0.01 (-0.04, 0.07)	9.70E-01
-0.05 (-0.09, -0.01)	9.09E-02	-0.04 (-0.09, 0.02)	9.70E-01
-0.05 (-0.09, -0.01)	8.03E-02	-0.04 (-0.09, 0.02)	9.70E-01
-0.05 (-0.09, -0.01)	9.94E-02	-0.04 (-0.09, 0.01)	9.70E-01
-0.05 (-0.10, -0.01)	6.03E-02	-0.04 (-0.09, 0.01)	9.70E-01
-0.06 (-0.10, -0.01)	6.03E-02	-0.04 (-0.09, 0.01)	9.70E-01
-0.05 (-0.09, -0.01)	8.03E-02	-0.04 (-0.09, 0.01)	9.70E-01
0.01 (-0.03, 0.05)	8.79E-01	0.02 (-0.03, 0.07)	9.70E-01

-0.03 (-0.08, 0.01)	2.42E-01	-0.01 (-0.06, 0.04)	9.70E-01
-0.03 (-0.08, 0.01)	2.42E-01	-0.02 (-0.07, 0.03)	9.70E-01
-0.04 (-0.08, 0.00)	1.51E-01	-0.01 (-0.07, 0.04)	9.70E-01
0.00 (-0.04, 0.04)	9.23E-01	-0.02 (-0.07, 0.03)	9.70E-01
0.01 (-0.03, 0.05)	9.23E-01	-0.01 (-0.06, 0.04)	9.70E-01
-0.05 (-0.09, -0.01)	6.85E-02	-0.03 (-0.08, 0.02)	9.70E-01
0.00 (-0.05, 0.04)	9.30E-01	0.03 (-0.02, 0.08)	9.70E-01
-0.02 (-0.06, 0.02)	5.43E-01	0.02 (-0.04, 0.07)	9.70E-01
0.03 (-0.01, 0.07)	2.80E-01	0.03 (-0.02, 0.08)	9.70E-01
0.04 (0.00, 0.08)	1.62E-01	0.01 (-0.04, 0.07)	9.70E-01
-0.01 (-0.05, 0.03)	8.26E-01	0.04 (-0.01, 0.09)	9.70E-01
-0.02 (-0.06, 0.02)	5.38E-01	-0.03 (-0.08, 0.02)	9.70E-01
0.04 (0.00, 0.08)	1.97E-01	0.01 (-0.04, 0.07)	9.70E-01
0.09 (0.05, 0.13)	**3.05E-03**	0.01 (-0.04, 0.07)	9.70E-01
0.06 (0.02, 0.10)	6.03E-02	0.01 (-0.05, 0.06)	9.70E-01
0.07 (0.03, 0.11)	**2.84E-02**	0.01 (-0.04, 0.06)	9.70E-01
-0.09 (-0.13, -0.05)	**3.05E-03**	-0.02 (-0.07, 0.04)	9.70E-01
0.01 (-0.03, 0.06)	7.33E-01	0.01 (-0.04, 0.06)	9.70E-01
0.05 (0.01, 0.09)	1.06E-01	0.02 (-0.03, 0.07)	9.70E-01
0.06 (0.01, 0.10)	6.03E-02	0.01 (-0.04, 0.06)	9.70E-01
0.01 (-0.03, 0.05)	9.23E-01	0.02 (-0.03, 0.07)	9.70E-01
-0.04 (-0.08, 0.00)	1.62E-01	-0.02 (-0.07, 0.03)	9.70E-01
0.00 (-0.04, 0.04)	9.88E-01	0.00 (-0.05, 0.05)	9.71E-01
0.06 (0.02, 0.11)	**3.07E-02**	0.01 (-0.04, 0.06)	9.70E-01
0.07 (0.03, 0.11)	**2.84E-02**	0.01 (-0.04, 0.06)	9.70E-01
-0.01 (-0.05, 0.03)	9.23E-01	0.00 (-0.05, 0.05)	9.80E-01
-0.06 (-0.10, -0.02)	5.14E-02	-0.01 (-0.06, 0.04)	9.70E-01
-0.08 (-0.12, -0.04)	**5.29E-03**	-0.02 (-0.07, 0.03)	9.70E-01
0.07 (0.03, 0.11)	**1.81E-02**	-0.04 (-0.09, 0.01)	9.70E-01
0.08 (0.04, 0.12)	**5.29E-03**	-0.03 (-0.08, 0.02)	9.70E-01
-0.03 (-0.07, 0.01)	3.34E-01	-0.08 (-0.13, -0.03)	3.78E-01
-0.05 (-0.09, -0.01)	7.92E-02	0.04 (-0.01, 0.09)	9.70E-01
-0.01 (-0.05, 0.03)	9.23E-01	0.03 (-0.02, 0.08)	9.70E-01
0.07 (0.03, 0.11)	**2.84E-02**	-0.02 (-0.07, 0.03)	9.70E-01
0.07 (0.03, 0.11)	**1.81E-02**	-0.02 (-0.07, 0.03)	9.70E-01
0.02 (-0.02, 0.06)	6.26E-01	-0.02 (-0.07, 0.03)	9.70E-01
-0.05 (-0.09, -0.01)	9.43E-02	0.01 (-0.04, 0.06)	9.70E-01
0.01 (-0.03, 0.05)	8.69E-01	0.02 (-0.03, 0.07)	9.70E-01
0.06 (0.02, 0.10)	**4.49E-02**	-0.01 (-0.06, 0.04)	9.70E-01
0.05 (0.01, 0.09)	1.09E-01	0.00 (-0.05, 0.05)	9.70E-01
0.03 (-0.01, 0.07)	2.97E-01	-0.01 (-0.06, 0.04)	9.70E-01
-0.06 (-0.10, -0.02)	**3.83E-02**	0.00 (-0.05, 0.05)	9.71E-01
-0.05 (-0.09, -0.01)	9.60E-02	0.02 (-0.03, 0.07)	9.70E-01
0.06 (0.02, 0.10)	**2.84E-02**	-0.01 (-0.06, 0.04)	9.70E-01
0.06 (0.02, 0.10)	**2.84E-02**	-0.01 (-0.06, 0.04)	9.70E-01
0.01 (-0.03, 0.05)	9.23E-01	-0.01 (-0.06, 0.04)	9.70E-01
-0.06 (-0.10, -0.02)	**3.07E-02**	0.01 (-0.04, 0.06)	9.70E-01
-0.04 (-0.09, 0.00)	1.21E-01	0.02 (-0.03, 0.07)	9.70E-01

0.05 (0.01, 0.09)	6.03E-02	-0.01 (-0.06, 0.04)	9.70E-01
0.06 (0.02, 0.10)	5.34E-02	-0.01 (-0.06, 0.04)	9.70E-01
-0.05 (-0.09, -0.01)	6.03E-02	0.01 (-0.04, 0.06)	9.70E-01
-0.06 (-0.10, -0.02)	**3.83E-02**	0.01 (-0.04, 0.06)	9.70E-01
-0.05 (-0.09, -0.01)	6.70E-02	0.02 (-0.03, 0.07)	9.70E-01
0.05 (0.01, 0.09)	8.03E-02	-0.01 (-0.06, 0.04)	9.70E-01
0.05 (0.01, 0.09)	6.03E-02	-0.01 (-0.06, 0.04)	9.70E-01
-0.06 (-0.10, -0.02)	5.14E-02	0.00 (-0.05, 0.05)	9.99E-01
-0.04 (-0.08, 0.00)	2.05E-01	0.01 (-0.04, 0.06)	9.70E-01
-0.02 (-0.06, 0.03)	6.70E-01	0.04 (-0.01, 0.09)	9.70E-01
0.01 (-0.03, 0.05)	8.26E-01	-0.06 (-0.11, 0.00)	9.70E-01
0.02 (-0.02, 0.06)	5.71E-01	-0.05 (-0.11, 0.00)	9.70E-01
-0.03 (-0.07, 0.01)	3.51E-01	-0.05 (-0.10, 0.00)	9.70E-01
0.02 (-0.02, 0.06)	6.11E-01	0.02 (-0.03, 0.07)	9.70E-01
0.03 (-0.01, 0.07)	3.34E-01	0.01 (-0.04, 0.06)	9.70E-01
-0.04 (-0.08, 0.00)	1.87E-01	-0.03 (-0.08, 0.02)	9.70E-01
-0.05 (-0.09, -0.01)	6.03E-02	-0.03 (-0.08, 0.02)	9.70E-01
-0.01 (-0.05, 0.03)	8.73E-01	-0.04 (-0.09, 0.01)	9.70E-01
0.02 (-0.02, 0.07)	4.79E-01	0.06 (-0.00, 0.11)	9.70E-01
0.02 (-0.02, 0.06)	4.83E-01	-0.01 (-0.06, 0.04)	9.70E-01
-0.04 (-0.08, 0.00)	2.03E-01	-0.03 (-0.08, 0.02)	9.70E-01
-0.03 (-0.07, 0.01)	3.15E-01	-0.02 (-0.07, 0.03)	9.70E-01
-0.04 (-0.08, 0.00)	1.52E-01	-0.04 (-0.09, 0.01)	9.70E-01
0.04 (0.00, 0.08)	1.91E-01	0.04 (-0.01, 0.09)	9.70E-01
-0.03 (-0.07, 0.01)	3.08E-01	0.00 (-0.05, 0.05)	9.80E-01
0.02 (-0.02, 0.06)	4.79E-01	-0.01 (-0.06, 0.04)	9.70E-01
0.03 (-0.01, 0.07)	3.85E-01	0.00 (-0.05, 0.05)	9.70E-01
-0.05 (-0.09, -0.01)	9.94E-02	-0.03 (-0.08, 0.02)	9.70E-01
0.01 (-0.03, 0.05)	8.88E-01	0.03 (-0.02, 0.08)	9.70E-01
0.00 (-0.04, 0.04)	9.88E-01	0.02 (-0.03, 0.07)	9.70E-01
0.01 (-0.03, 0.05)	8.21E-01	0.06 (-0.01, 0.11)	9.70E-01
-0.03 (-0.07, 0.01)	3.94E-01	-0.03 (-0.08, 0.02)	9.70E-01
0.02 (-0.02, 0.06)	5.19E-01	-0.01 (-0.06, 0.04)	9.70E-01
0.04 (0.00, 0.08)	2.03E-01	0.01 (-0.04, 0.06)	9.70E-01
0.04 (0.00, 0.08)	1.44E-01	0.01 (-0.04, 0.06)	9.70E-01
0.04 (0.00, 0.08)	1.97E-01	0.00 (-0.05, 0.05)	9.70E-01
-0.05 (-0.09, -0.01)	8.36E-02	-0.05 (-0.10, 0.00)	9.70E-01
-0.05 (-0.09, -0.01)	7.73E-02	-0.04 (-0.10, 0.01)	9.70E-01
-0.02 (-0.06, 0.02)	4.58E-01	-0.06 (-0.11, -0.01)	9.70E-01
0.01 (-0.04, 0.05)	9.23E-01	0.01 (-0.04, 0.06)	9.70E-01
0.01 (-0.03, 0.05)	7.36E-01	0.01 (-0.04, 0.06)	9.70E-01
0.00 (-0.04, 0.04)	9.46E-01	0.01 (-0.04, 0.06)	9.70E-01
0.00 (-0.04, 0.04)	9.88E-01	0.01 (-0.04, 0.06)	9.70E-01
-0.02 (-0.06, 0.02)	6.40E-01	0.00 (-0.06, 0.05)	9.70E-01
0.00 (-0.04, 0.04)	9.96E-01	0.02 (-0.03, 0.07)	9.70E-01
-0.01 (-0.06, 0.03)	7.36E-01	-0.01 (-0.06, 0.04)	9.70E-01
0.02 (-0.02, 0.06)	6.53E-01	0.01 (-0.04, 0.06)	9.70E-01
-0.03 (-0.07, 0.01)	3.18E-01	0.01 (-0.04, 0.06)	9.70E-01

0.00 (-0.05, 0.04)	9.23E-01	0.01 (-0.05, 0.06)	9.70E-01
-0.02 (-0.06, 0.02)	4.92E-01	0.00 (-0.06, 0.05)	9.70E-01
-0.04 (-0.08, 0.00)	1.97E-01	-0.04 (-0.09, 0.01)	9.70E-01
0.03 (-0.01, 0.07)	3.18E-01	0.01 (-0.04, 0.06)	9.70E-01
0.05 (0.01, 0.09)	1.10E-01	0.03 (-0.02, 0.08)	9.70E-01
0.00 (-0.04, 0.04)	9.71E-01	0.00 (-0.05, 0.05)	9.99E-01
0.01 (-0.03, 0.05)	8.08E-01	0.04 (-0.01, 0.09)	9.70E-01
0.06 (0.02, 0.10)	5.34E-02	-0.03 (-0.08, 0.02)	9.70E-01
-0.01 (-0.05, 0.03)	9.23E-01	0.01 (-0.04, 0.06)	9.70E-01
0.04 (0.00, 0.08)	1.62E-01	-0.01 (-0.06, 0.04)	9.70E-01
-0.01 (-0.05, 0.04)	9.23E-01	0.01 (-0.04, 0.06)	9.70E-01
0.00 (-0.04, 0.04)	9.46E-01	0.01 (-0.04, 0.06)	9.70E-01
-0.01 (-0.05, 0.03)	8.64E-01	0.00 (-0.05, 0.05)	9.70E-01
0.00 (-0.04, 0.04)	9.96E-01	0.00 (-0.05, 0.05)	9.80E-01
0.07 (0.03, 0.11)	**2.58E-02**	-0.05 (-0.10, 0.01)	9.70E-01
0.00 (-0.05, 0.04)	9.23E-01	0.03 (-0.02, 0.09)	9.70E-01
0.08 (0.03, 0.12)	**1.22E-02**	-0.03 (-0.08, 0.03)	9.70E-01
-0.01 (-0.05, 0.03)	9.23E-01	0.03 (-0.02, 0.08)	9.70E-01
0.02 (-0.02, 0.06)	6.67E-01	0.02 (-0.03, 0.07)	9.70E-01
-0.02 (-0.06, 0.02)	5.57E-01	-0.02 (-0.07, 0.03)	9.70E-01
0.00 (-0.04, 0.04)	9.88E-01	-0.01 (-0.06, 0.04)	9.70E-01
0.01 (-0.04, 0.05)	9.23E-01	-0.01 (-0.06, 0.04)	9.70E-01
-0.02 (-0.06, 0.02)	6.11E-01	0.01 (-0.04, 0.06)	9.70E-01
-0.02 (-0.07, 0.02)	4.79E-01	0.01 (-0.04, 0.07)	9.70E-01
0.00 (-0.04, 0.04)	9.30E-01	0.01 (-0.04, 0.06)	9.70E-01
0.00 (-0.04, 0.05)	9.30E-01	-0.01 (-0.06, 0.04)	9.70E-01
0.05 (0.01, 0.09)	7.92E-02	0.00 (-0.06, 0.05)	9.70E-01
0.00 (-0.04, 0.04)	9.88E-01	0.03 (-0.02, 0.08)	9.70E-01
0.02 (-0.02, 0.06)	5.16E-01	0.00 (-0.05, 0.05)	9.87E-01
0.00 (-0.05, 0.04)	9.23E-01	0.03 (-0.02, 0.08)	9.70E-01
0.04 (0.00, 0.08)	1.45E-01	0.03 (-0.02, 0.08)	9.70E-01
0.01 (-0.03, 0.05)	9.01E-01	0.04 (-0.00, 0.09)	9.70E-01
0.01 (-0.04, 0.05)	9.23E-01	-0.01 (-0.06, 0.04)	9.70E-01
-0.04 (-0.08, 0.00)	1.64E-01	-0.02 (-0.07, 0.04)	9.70E-01
-0.06 (-0.11, -0.02)	**3.07E-02**	-0.03 (-0.08, 0.02)	9.70E-01
-0.01 (-0.05, 0.04)	9.01E-01	0.00 (-0.05, 0.05)	9.80E-01
-0.02 (-0.06, 0.02)	4.66E-01	-0.01 (-0.06, 0.04)	9.70E-01
0.03 (-0.01, 0.07)	3.70E-01	-0.01 (-0.06, 0.04)	9.70E-01

Never/former smoker		Current smoker	
Adjusted β (95% CI)	P value	Adjusted β (95% CI)	P value
0.04 (0.00, 0.08)	1.92E-01	-0.04 (-0.10, 0.01)	3.89E-01
0.04 (0.00, 0.09)	1.92E-01	-0.04 (-0.09, 0.02)	3.98E-01
0.03 (-0.01, 0.07)	2.31E-01	-0.04 (-0.10, 0.01)	3.75E-01
0.04 (0.00, 0.08)	1.25E-01	-0.02 (-0.08, 0.03)	6.56E-01
0.05 (0.01, 0.09)	5.71E-02	-0.01 (-0.07, 0.04)	9.14E-01
0.03 (-0.01, 0.07)	2.64E-01	-0.04 (-0.09, 0.02)	3.99E-01
0.04 (0.00, 0.08)	2.00E-01	-0.04 (-0.10, 0.01)	3.61E-01
0.03 (-0.01, 0.07)	2.31E-01	-0.04 (-0.10, 0.01)	3.58E-01
0.03 (0.00, 0.07)	2.31E-01	-0.04 (-0.10, 0.01)	3.58E-01
0.04 (0.00, 0.08)	2.12E-01	-0.04 (-0.09, 0.02)	3.99E-01
0.04 (0.00, 0.08)	1.45E-01	-0.03 (-0.09, 0.02)	5.03E-01
0.05 (0.01, 0.08)	1.16E-01	-0.03 (-0.08, 0.03)	5.62E-01
0.04 (0.00, 0.08)	2.12E-01	-0.03 (-0.09, 0.02)	4.77E-01
0.03 (-0.01, 0.07)	2.55E-01	-0.05 (-0.10, 0.01)	3.06E-01
0.03 (-0.01, 0.07)	2.31E-01	-0.04 (-0.10, 0.01)	3.58E-01
0.03 (-0.01, 0.07)	2.31E-01	-0.04 (-0.10, 0.01)	3.64E-01
0.03 (-0.01, 0.07)	2.31E-01	-0.04 (-0.10, 0.01)	3.81E-01
0.04 (0.00, 0.08)	1.91E-01	-0.03 (-0.09, 0.02)	5.03E-01
0.04 (0.01, 0.08)	1.18E-01	-0.02 (-0.08, 0.03)	6.66E-01
0.03 (-0.01, 0.07)	2.56E-01	-0.04 (-0.09, 0.01)	3.89E-01
0.03 (-0.01, 0.07)	2.55E-01	-0.05 (-0.10, 0.01)	3.19E-01
0.04 (0.00, 0.08)	1.92E-01	-0.03 (-0.09, 0.02)	5.03E-01
0.04 (0.00, 0.08)	1.91E-01	-0.03 (-0.09, 0.02)	5.35E-01
0.04 (0.00, 0.08)	1.91E-01	-0.03 (-0.08, 0.03)	5.82E-01
0.05 (0.01, 0.08)	1.14E-01	-0.01 (-0.07, 0.04)	9.14E-01
0.05 (0.02, 0.09)	5.35E-02	0.01 (-0.05, 0.06)	9.17E-01
0.03 (0.00, 0.07)	2.31E-01	-0.03 (-0.09, 0.02)	5.35E-01
0.03 (-0.01, 0.07)	2.31E-01	-0.04 (-0.09, 0.02)	3.99E-01
0.03 (-0.01, 0.07)	2.31E-01	0.00 (-0.06, 0.05)	9.91E-01
0.03 (-0.01, 0.07)	2.31E-01	0.00 (-0.05, 0.06)	9.91E-01
0.03 (-0.01, 0.07)	3.75E-01	0.00 (-0.06, 0.05)	9.91E-01
0.04 (0.00, 0.08)	1.71E-01	0.04 (-0.01, 0.10)	3.58E-01
0.05 (0.01, 0.09)	1.08E-01	0.06 (0.01, 0.12)	1.50E-01
0.02 (-0.02, 0.06)	3.98E-01	0.01 (-0.05, 0.06)	9.61E-01
0.03 (-0.01, 0.07)	2.64E-01	-0.02 (-0.08, 0.03)	6.66E-01
0.02 (-0.02, 0.06)	4.42E-01	0.04 (-0.02, 0.09)	4.13E-01
0.02 (-0.02, 0.06)	4.60E-01	0.04 (-0.01, 0.10)	3.58E-01
0.01 (-0.03, 0.05)	6.80E-01	0.05 (0.00, 0.11)	2.45E-01
0.02 (-0.02, 0.06)	3.98E-01	0.06 (0.00, 0.11)	1.77E-01
0.03 (-0.01, 0.07)	2.31E-01	0.05 (0.00, 0.11)	1.87E-01
0.00 (-0.04, 0.04)	8.76E-01	0.06 (0.00, 0.11)	1.77E-01
0.02 (-0.02, 0.06)	5.89E-01	-0.01 (-0.07, 0.04)	8.43E-01
0.02 (-0.02, 0.06)	4.88E-01	0.05 (0.00, 0.11)	1.87E-01

0.02 (-0.02, 0.06)	4.88E-01	0.06 (0.00, 0.11)	1.77E-01
0.02 (-0.02, 0.06)	5.06E-01	0.06 (0.01, 0.12)	1.59E-01
0.02 (-0.02, 0.06)	4.88E-01	0.06 (0.00, 0.11)	1.63E-01
0.03 (-0.01, 0.07)	3.80E-01	0.06 (0.00, 0.11)	1.87E-01
0.01 (-0.03, 0.05)	8.38E-01	0.06 (0.01, 0.12)	1.50E-01
0.00 (-0.04, 0.04)	9.59E-01	0.00 (-0.06, 0.05)	9.61E-01
0.02 (-0.02, 0.06)	4.88E-01	0.06 (0.01, 0.12)	1.60E-01
0.02 (-0.02, 0.06)	4.88E-01	0.06 (0.01, 0.12)	1.50E-01
0.02 (-0.02, 0.06)	4.83E-01	0.06 (0.01, 0.12)	1.50E-01
0.02 (-0.02, 0.06)	4.66E-01	0.06 (0.01, 0.12)	1.50E-01
0.03 (-0.02, 0.07)	3.96E-01	0.06 (0.01, 0.12)	1.50E-01
0.01 (-0.03, 0.05)	6.89E-01	0.07 (0.01, 0.12)	1.50E-01
-0.01 (-0.05, 0.04)	8.47E-01	0.01 (-0.05, 0.06)	9.17E-01
0.02 (-0.02, 0.06)	4.95E-01	0.06 (0.01, 0.12)	1.50E-01
0.02 (-0.02, 0.06)	5.02E-01	0.06 (0.01, 0.12)	1.50E-01
0.03 (-0.02, 0.07)	3.87E-01	0.06 (0.00, 0.11)	1.87E-01
0.02 (-0.02, 0.06)	5.06E-01	0.07 (0.01, 0.12)	1.50E-01
0.02 (-0.02, 0.06)	4.88E-01	0.07 (0.01, 0.12)	1.50E-01
0.01 (-0.03, 0.05)	6.83E-01	0.07 (0.01, 0.12)	1.50E-01
-0.01 (-0.05, 0.04)	8.38E-01	0.02 (-0.04, 0.07)	7.56E-01
0.01 (-0.03, 0.06)	6.19E-01	0.06 (0.00, 0.12)	1.64E-01
0.01 (-0.03, 0.05)	6.53E-01	0.06 (0.01, 0.12)	1.60E-01
0.01 (-0.03, 0.05)	7.56E-01	0.05 (-0.01, 0.10)	2.74E-01
0.01 (-0.03, 0.05)	6.66E-01	0.06 (0.01, 0.12)	1.50E-01
0.01 (-0.03, 0.05)	6.82E-01	0.06 (0.01, 0.12)	1.50E-01
0.00 (-0.04, 0.04)	9.89E-01	0.06 (0.01, 0.12)	1.50E-01
0.02 (-0.02, 0.06)	5.56E-01	0.01 (-0.04, 0.07)	9.14E-01
-0.05 (-0.09, -0.01)	8.18E-02	0.00 (-0.06, 0.05)	9.91E-01
-0.05 (-0.09, -0.01)	8.04E-02	0.00 (-0.06, 0.05)	9.91E-01
-0.05 (-0.09, -0.02)	5.28E-02	0.01 (-0.05, 0.06)	9.54E-01
-0.04 (-0.08, -0.01)	1.15E-01	-0.01 (-0.06, 0.04)	9.17E-01
-0.04 (-0.08, 0.00)	1.47E-01	-0.01 (-0.06, 0.04)	9.14E-01
-0.05 (-0.09, -0.01)	6.06E-02	-0.01 (-0.06, 0.05)	9.60E-01
0.02 (-0.02, 0.06)	5.10E-01	-0.01 (-0.06, 0.04)	9.14E-01
-0.07 (-0.11, -0.03)	**9.26E-03**	0.00 (-0.06, 0.06)	9.91E-01
-0.07 (-0.11, -0.03)	**9.19E-03**	0.00 (-0.06, 0.05)	9.91E-01
-0.07 (-0.11, -0.03)	**9.19E-03**	0.00 (-0.06, 0.06)	9.91E-01
-0.07 (-0.11, -0.03)	**9.19E-03**	0.00 (-0.06, 0.05)	9.61E-01
-0.07 (-0.11, -0.03)	**9.19E-03**	-0.01 (-0.06, 0.05)	9.50E-01
-0.07 (-0.11, -0.03)	**9.19E-03**	0.00 (-0.06, 0.06)	9.91E-01
-0.01 (-0.05, 0.03)	7.59E-01	0.01 (-0.04, 0.07)	8.43E-01
-0.06 (-0.10, -0.02)	**1.63E-02**	-0.02 (-0.08, 0.04)	7.30E-01
-0.07 (-0.11, -0.03)	**1.15E-02**	-0.02 (-0.08, 0.04)	7.44E-01
-0.06 (-0.10, -0.02)	**1.48E-02**	-0.02 (-0.08, 0.04)	7.52E-01
-0.07 (-0.11, -0.03)	**6.91E-03**	-0.02 (-0.08, 0.04)	7.44E-01
-0.07 (-0.11, -0.03)	**8.00E-03**	-0.02 (-0.08, 0.04)	6.87E-01
-0.08 (-0.11, -0.04)	**5.24E-03**	-0.01 (-0.07, 0.05)	9.17E-01
0.03 (-0.01, 0.07)	2.31E-01	-0.01 (-0.06, 0.05)	9.61E-01

-0.03 (-0.07, 0.01)	2.64E-01	-0.02 (-0.08, 0.04)	7.56E-01
-0.03 (-0.07, 0.01)	2.31E-01	-0.02 (-0.07, 0.04)	8.43E-01
-0.03 (-0.07, 0.01)	2.91E-01	-0.05 (-0.10, 0.01)	3.58E-01
-0.03 (-0.07, 0.01)	2.79E-01	0.04 (-0.02, 0.09)	4.21E-01
-0.02 (-0.06, 0.02)	4.92E-01	0.04 (-0.01, 0.10)	3.58E-01
-0.05 (-0.09, -0.01)	6.09E-02	-0.03 (-0.09, 0.02)	5.03E-01
0.03 (-0.01, 0.07)	2.41E-01	-0.03 (-0.08, 0.03)	5.82E-01
0.00 (-0.04, 0.04)	9.16E-01	-0.01 (-0.06, 0.05)	9.54E-01
0.03 (-0.01, 0.07)	2.31E-01	0.03 (-0.02, 0.09)	5.00E-01
0.03 (0.00, 0.07)	2.31E-01	0.03 (-0.02, 0.09)	4.74E-01
0.01 (-0.03, 0.05)	7.30E-01	0.01 (-0.05, 0.06)	9.17E-01
-0.03 (-0.07, 0.01)	2.98E-01	-0.03 (-0.08, 0.03)	6.07E-01
0.05 (0.01, 0.09)	9.18E-02	0.01 (-0.05, 0.07)	9.17E-01
0.07 (0.03, 0.11)	**9.19E-03**	0.06 (0.01, 0.12)	1.50E-01
0.03 (-0.01, 0.07)	2.47E-01	0.06 (0.00, 0.12)	1.77E-01
0.07 (0.03, 0.11)	**9.19E-03**	0.03 (-0.03, 0.08)	5.87E-01
-0.08 (-0.12, -0.04)	**2.76E-03**	-0.05 (-0.11, 0.00)	2.33E-01
0.04 (-0.01, 0.08)	2.31E-01	0.00 (-0.06, 0.05)	9.64E-01
0.05 (0.01, 0.09)	7.11E-02	0.03 (-0.02, 0.09)	4.74E-01
0.04 (0.00, 0.08)	2.31E-01	0.06 (0.00, 0.11)	1.77E-01
0.03 (-0.01, 0.07)	2.64E-01	-0.01 (-0.06, 0.04)	9.14E-01
-0.05 (-0.09, -0.01)	7.91E-02	-0.03 (-0.08, 0.03)	6.07E-01
-0.02 (-0.06, 0.02)	4.67E-01	0.04 (-0.01, 0.10)	3.69E-01
0.04 (0.00, 0.08)	1.91E-01	0.08 (0.02, 0.13)	1.50E-01
0.03 (-0.01, 0.07)	2.31E-01	0.08 (0.03, 0.14)	1.50E-01
0.01 (-0.02, 0.05)	6.05E-01	-0.02 (-0.07, 0.04)	7.83E-01
-0.03 (-0.07, 0.01)	2.47E-01	-0.08 (-0.13, -0.02)	1.50E-01
-0.09 (-0.12, -0.05)	**2.48E-03**	-0.03 (-0.09, 0.02)	4.82E-01
0.01 (-0.03, 0.05)	7.41E-01	0.08 (0.02, 0.13)	1.50E-01
0.02 (-0.02, 0.06)	4.80E-01	0.08 (0.03, 0.13)	1.50E-01
-0.08 (-0.12, -0.04)	**2.76E-03**	0.02 (-0.04, 0.07)	7.51E-01
0.01 (-0.03, 0.05)	7.05E-01	-0.07 (-0.12, -0.01)	1.50E-01
-0.02 (-0.06, 0.02)	5.62E-01	0.04 (-0.01, 0.10)	3.58E-01
0.02 (-0.02, 0.06)	5.10E-01	0.06 (0.01, 0.12)	1.50E-01
0.03 (-0.01, 0.07)	2.31E-01	0.05 (-0.01, 0.10)	2.92E-01
-0.03 (-0.07, 0.01)	3.08E-01	0.06 (0.01, 0.12)	1.50E-01
-0.01 (-0.05, 0.03)	8.16E-01	-0.07 (-0.12, -0.01)	1.50E-01
0.00 (-0.04, 0.04)	9.16E-01	0.02 (-0.03, 0.08)	6.46E-01
0.02 (-0.02, 0.06)	4.13E-01	0.07 (0.01, 0.12)	1.50E-01
0.04 (0.00, 0.08)	1.19E-01	0.02 (-0.03, 0.08)	6.64E-01
-0.02 (-0.05, 0.02)	5.80E-01	0.07 (0.02, 0.12)	1.50E-01
-0.02 (-0.06, 0.02)	4.60E-01	-0.07 (-0.13, -0.02)	1.50E-01
-0.02 (-0.06, 0.02)	5.62E-01	-0.06 (-0.12, -0.01)	1.50E-01
0.03 (-0.01, 0.07)	3.80E-01	0.07 (0.02, 0.13)	1.50E-01
0.04 (0.00, 0.08)	1.91E-01	0.07 (0.01, 0.12)	1.50E-01
-0.03 (-0.07, 0.01)	2.64E-01	0.04 (-0.01, 0.10)	3.58E-01
-0.03 (-0.07, 0.01)	3.59E-01	-0.07 (-0.13, -0.02)	1.50E-01
-0.01 (-0.04, 0.03)	8.49E-01	-0.07 (-0.12, -0.01)	1.50E-01

0.01 (-0.03, 0.05)	6.11E-01	0.07 (0.02, 0.13)	1.50E-01
0.02 (-0.02, 0.06)	4.88E-01	0.07 (0.02, 0.13)	1.50E-01
-0.03 (-0.07, 0.01)	2.55E-01	-0.06 (-0.11, 0.00)	1.76E-01
-0.03 (-0.07, 0.01)	2.92E-01	-0.07 (-0.12, -0.01)	1.50E-01
-0.02 (-0.06, 0.02)	5.55E-01	-0.07 (-0.12, -0.01)	1.50E-01
0.01 (-0.03, 0.05)	6.81E-01	0.06 (0.01, 0.12)	1.50E-01
0.02 (-0.02, 0.06)	5.06E-01	0.06 (0.01, 0.12)	1.50E-01
-0.05 (-0.09, -0.01)	1.18E-01	-0.04 (-0.10, 0.01)	3.58E-01
0.00 (-0.04, 0.04)	9.89E-01	-0.05 (-0.11, 0.00)	1.87E-01
-0.02 (-0.06, 0.02)	5.31E-01	0.03 (-0.03, 0.08)	6.07E-01
0.00 (-0.04, 0.04)	9.00E-01	-0.03 (-0.09, 0.03)	5.87E-01
0.01 (-0.03, 0.05)	6.33E-01	-0.03 (-0.08, 0.03)	6.37E-01
-0.04 (-0.08, 0.00)	1.89E-01	-0.02 (-0.08, 0.03)	6.38E-01
0.04 (0.00, 0.08)	1.48E-01	-0.01 (-0.06, 0.05)	9.17E-01
0.02 (-0.02, 0.06)	4.42E-01	0.04 (-0.02, 0.09)	4.74E-01
-0.05 (-0.09, -0.01)	8.73E-02	-0.04 (-0.09, 0.02)	4.48E-01
-0.05 (-0.09, -0.01)	1.08E-01	-0.06 (-0.12, -0.01)	1.63E-01
-0.04 (-0.08, 0.00)	1.31E-01	0.01 (-0.05, 0.06)	9.17E-01
0.06 (0.02, 0.10)	5.47E-02	0.01 (-0.05, 0.06)	9.17E-01
0.02 (-0.02, 0.06)	4.13E-01	0.00 (-0.05, 0.05)	9.91E-01
-0.06 (-0.10, -0.02)	**3.39E-02**	0.00 (-0.06, 0.05)	9.91E-01
-0.04 (-0.08, 0.00)	1.32E-01	-0.01 (-0.06, 0.05)	9.17E-01
-0.08 (-0.12, -0.04)	**2.48E-03**	0.02 (-0.03, 0.08)	6.72E-01
0.07 (0.03, 0.11)	**9.19E-03**	0.00 (-0.05, 0.06)	9.91E-01
-0.01 (-0.05, 0.03)	8.49E-01	-0.06 (-0.11, 0.00)	1.77E-01
-0.01 (-0.05, 0.03)	7.30E-01	0.05 (0.00, 0.11)	1.87E-01
0.00 (-0.04, 0.04)	9.21E-01	0.05 (0.00, 0.11)	1.97E-01
-0.05 (-0.09, -0.01)	8.18E-02	-0.03 (-0.09, 0.02)	4.74E-01
0.05 (0.01, 0.09)	1.08E-01	-0.02 (-0.08, 0.03)	6.66E-01
0.04 (0.00, 0.07)	2.12E-01	-0.04 (-0.09, 0.01)	3.89E-01
0.02 (-0.02, 0.06)	4.92E-01	0.03 (-0.03, 0.09)	5.54E-01
-0.05 (-0.09, -0.01)	9.46E-02	0.00 (-0.05, 0.06)	9.67E-01
0.00 (-0.04, 0.04)	9.51E-01	0.05 (-0.01, 0.11)	2.55E-01
0.05 (0.01, 0.09)	1.08E-01	0.01 (-0.04, 0.07)	9.09E-01
0.04 (0.00, 0.08)	1.30E-01	0.04 (-0.02, 0.09)	4.21E-01
0.02 (-0.02, 0.06)	4.88E-01	0.06 (0.00, 0.11)	1.76E-01
-0.08 (-0.12, -0.04)	**2.48E-03**	0.00 (-0.05, 0.06)	9.91E-01
-0.08 (-0.12, -0.04)	**2.48E-03**	0.00 (-0.06, 0.06)	9.91E-01
-0.06 (-0.10, -0.02)	**3.27E-02**	0.01 (-0.04, 0.06)	9.14E-01
0.00 (-0.05, 0.04)	8.79E-01	0.05 (0.00, 0.11)	1.93E-01
0.01 (-0.03, 0.05)	6.80E-01	0.04 (-0.02, 0.09)	3.99E-01
0.03 (-0.01, 0.07)	3.08E-01	-0.03 (-0.09, 0.02)	4.82E-01
0.03 (-0.01, 0.07)	2.56E-01	-0.04 (-0.09, 0.02)	4.21E-01
-0.01 (-0.05, 0.03)	7.59E-01	0.00 (-0.05, 0.05)	9.91E-01
0.02 (-0.02, 0.06)	4.20E-01	-0.01 (-0.06, 0.05)	9.17E-01
-0.02 (-0.06, 0.02)	5.72E-01	0.01 (-0.05, 0.06)	9.17E-01
0.04 (0.01, 0.08)	1.14E-01	-0.03 (-0.09, 0.02)	5.03E-01
-0.02 (-0.07, 0.02)	4.19E-01	0.01 (-0.04, 0.07)	9.14E-01

0.00 (-0.05, 0.04)	8.77E-01	0.02 (-0.04, 0.07)	7.68E-01
-0.02 (-0.06, 0.02)	4.66E-01	0.01 (-0.04, 0.07)	9.14E-01
-0.06 (-0.10, -0.02)	**2.09E-02**	0.00 (-0.05, 0.06)	9.67E-01
0.04 (0.00, 0.08)	1.89E-01	0.02 (-0.03, 0.08)	6.64E-01
0.07 (0.03, 0.11)	**1.03E-02**	0.02 (-0.04, 0.08)	7.15E-01
0.02 (-0.02, 0.06)	5.62E-01	-0.01 (-0.07, 0.04)	9.14E-01
0.02 (-0.02, 0.06)	5.10E-01	0.03 (-0.03, 0.09)	5.55E-01
0.03 (-0.01, 0.07)	2.55E-01	0.02 (-0.04, 0.08)	7.52E-01
0.02 (-0.02, 0.06)	5.06E-01	-0.01 (-0.07, 0.05)	9.17E-01
0.04 (0.00, 0.08)	1.92E-01	0.01 (-0.05, 0.07)	9.17E-01
0.01 (-0.03, 0.06)	6.19E-01	0.00 (-0.06, 0.06)	9.91E-01
0.02 (-0.02, 0.06)	4.95E-01	0.00 (-0.05, 0.06)	9.91E-01
0.01 (-0.03, 0.05)	7.30E-01	-0.02 (-0.08, 0.03)	6.65E-01
0.02 (-0.02, 0.06)	5.32E-01	-0.01 (-0.07, 0.04)	9.14E-01
0.02 (-0.02, 0.06)	4.65E-01	0.04 (-0.02, 0.09)	3.99E-01
0.01 (-0.03, 0.05)	7.16E-01	0.00 (-0.05, 0.06)	9.83E-01
0.04 (0.00, 0.08)	1.25E-01	0.04 (-0.02, 0.10)	3.99E-01
0.00 (-0.05, 0.04)	8.77E-01	0.02 (-0.03, 0.08)	6.73E-01
0.01 (-0.03, 0.05)	7.30E-01	0.03 (-0.02, 0.09)	4.74E-01
-0.01 (-0.05, 0.03)	6.80E-01	-0.03 (-0.09, 0.02)	4.74E-01
0.00 (-0.04, 0.04)	9.59E-01	-0.01 (-0.07, 0.05)	9.17E-01
0.01 (-0.03, 0.05)	8.05E-01	-0.01 (-0.06, 0.05)	9.61E-01
0.01 (-0.03, 0.05)	7.69E-01	-0.03 (-0.08, 0.02)	5.35E-01
-0.01 (-0.05, 0.03)	6.66E-01	-0.01 (-0.07, 0.05)	9.14E-01
0.01 (-0.03, 0.05)	6.80E-01	0.01 (-0.05, 0.06)	9.60E-01
0.00 (-0.04, 0.04)	9.89E-01	0.00 (-0.06, 0.05)	9.91E-01
0.03 (-0.01, 0.07)	2.79E-01	0.04 (-0.01, 0.10)	3.75E-01
0.04 (0.00, 0.08)	1.92E-01	-0.03 (-0.08, 0.03)	5.97E-01
0.04 (0.00, 0.08)	1.95E-01	-0.02 (-0.08, 0.03)	6.47E-01
0.03 (-0.01, 0.07)	3.23E-01	-0.02 (-0.08, 0.03)	6.38E-01
0.06 (0.02, 0.10)	**1.48E-02**	-0.01 (-0.06, 0.05)	9.17E-01
0.04 (0.00, 0.08)	1.72E-01	-0.01 (-0.07, 0.04)	8.59E-01
0.00 (-0.04, 0.04)	9.57E-01	0.00 (-0.05, 0.06)	9.91E-01
-0.01 (-0.05, 0.03)	6.80E-01	-0.06 (-0.11, 0.00)	1.87E-01
-0.03 (-0.07, 0.01)	2.79E-01	-0.08 (-0.13, -0.02)	1.50E-01
-0.01 (-0.05, 0.03)	6.28E-01	0.02 (-0.03, 0.07)	7.15E-01
-0.01 (-0.05, 0.02)	6.19E-01	-0.02 (-0.07, 0.04)	7.44E-01
0.04 (0.00, 0.08)	1.48E-01	-0.03 (-0.09, 0.03)	5.39E-01

Never/former drinker		Current drinker	
**Adjusted β (95% CI)**	**P value**	**Adjusted β (95% CI)**	**P value**
0.04 (0.01, 0.08)	5.28E-02	-0.12 (-0.20, -0.05)	**2.90E-02**
0.04 (0.01, 0.09)	5.28E-02	-0.12 (-0.20, -0.06)	**2.90E-02**
0.04 (0.01, 0.10)	6.98E-02	-0.13 (-0.20, -0.05)	**2.90E-02**
0.05 (0.02, 0.09)	**3.62E-02**	-0.10 (-0.18, -0.03)	**4.60E-02**
0.06 (0.02, 0.09)	**1.56E-02**	-0.08 (-0.16, -0.01)	9.80E-02
0.04 (0.00, 0.07)	8.90E-02	-0.12 (-0.20, -0.04)	**2.90E-02**
0.04 (0.01, 0.08)	5.57E-02	-0.13 (-0.20, -0.05)	**2.90E-02**
0.04 (0.00, 0.07)	9.51E-02	-0.12 (-0.19, -0.04)	**3.13E-02**
0.04 (0.00, 0.07)	8.90E-02	-0.12 (-0.19, -0.04)	**3.13E-02**
0.04 (0.01, 0.08)	6.98E-02	-0.12 (-0.19, -0.04)	**3.13E-02**
0.05 (0.01, 0.08)	**4.96E-02**	-0.11 (-0.18, -0.03)	**4.32E-02**
0.05 (0.01, 0.08)	**4.20E-02**	-0.10 (-0.18, -0.02)	5.50E-02
0.04 (0.01, 0.08)	6.54E-02	-0.11 (-0.19, -0.03)	**3.85E-02**
0.03 (0.00, 0.07)	1.18E-01	-0.12 (-0.20, -0.04)	**2.90E-02**
0.04 (0.00, 0.07)	8.90E-02	-0.12 (-0.20, -0.04)	**2.90E-02**
0.04 (0.00, 0.07)	8.80E-02	-0.12 (-0.19, -0.04)	**2.90E-02**
0.04 (0.00, 0.07)	8.73E-02	-0.12 (-0.19, -0.04)	**2.90E-02**
0.04 (0.01, 0.08)	5.90E-02	-0.11 (-0.18, -0.03)	**3.85E-02**
0.05 (0.01, 0.08)	**4.05E-02**	-0.10 (-0.17, -0.02)	6.51E-02
0.04 (0.00, 0.07)	9.62E-02	-0.12 (-0.19, -0.04)	**2.90E-02**
0.04 (0.00, 0.07)	1.01E-01	-0.12 (-0.20, -0.05)	**2.90E-02**
0.04 (0.01, 0.08)	5.17E-02	-0.11 (-0.19, -0.03)	**3.75E-02**
0.05 (0.01, 0.08)	**4.96E-02**	-0.11 (-0.18, -0.03)	**3.85E-02**
0.05 (0.01, 0.08)	**4.96E-02**	-0.11 (-0.18, -0.03)	**4.23E-02**
0.05 (0.02, 0.09)	**2.55E-02**	-0.09 (-0.16, -0.01)	9.57E-02
0.06 (0.03, 0.10)	**1.56E-02**	-0.06 (-0.13, 0.02)	2.42E-01
0.04 (0.01, 0.08)	5.94E-02	-0.11 (-0.19, -0.03)	**3.85E-02**
0.04 (0.01, 0.08)	6.39E-02	-0.12 (-0.19, -0.04)	**2.90E-02**
0.04 (0.01, 0.08)	5.31E-02	-0.08 (-0.16, 0.00)	1.17E-01
0.04 (0.01, 0.08)	5.28E-02	-0.07 (-0.15, 0.00)	1.38E-01
0.04 (0.00, 0.07)	9.60E-02	-0.08 (-0.16, 0.00)	1.08E-01
0.05 (0.01, 0.08)	**4.20E-02**	0.00 (-0.08, 0.08)	9.69E-01
0.05 (0.02, 0.09)	**3.25E-02**	0.04 (-0.04, 0.12)	4.08E-01
0.04 (0.00, 0.07)	1.01E-01	-0.07 (-0.15, 0.01)	1.50E-01
0.04 (0.00, 0.07)	7.50E-02	-0.10 (-0.18, -0.03)	5.14E-02
0.03 (-0.01, 0.06)	2.86E-01	0.03 (-0.05, 0.10)	6.23E-01
0.02 (-0.01, 0.06)	2.97E-01	0.04 (-0.04, 0.12)	4.42E-01
0.01 (-0.02, 0.05)	5.73E-01	0.06 (-0.01, 0.14)	1.99E-01
0.02 (-0.01, 0.06)	2.96E-01	0.07 (0.00, 0.15)	1.38E-01
0.03 (-0.01, 0.07)	1.90E-01	0.07 (0.00, 0.15)	1.38E-01
0.01 (-0.03, 0.04)	7.02E-01	0.07 (-0.01, 0.14)	1.64E-01
0.02 (-0.01, 0.06)	3.38E-01	-0.07 (-0.14, 0.01)	1.66E-01
0.02 (-0.01, 0.06)	3.13E-01	0.06 (-0.02, 0.14)	2.15E-01

0.02 (-0.01, 0.06)	3.15E-01	0.06 (-0.01, 0.14)	1.81E-01
0.02 (-0.02, 0.06)	3.77E-01	0.08 (0.00, 0.16)	1.17E-01
0.02 (-0.01, 0.06)	3.38E-01	0.07 (0.00, 0.15)	1.38E-01
0.03 (-0.01, 0.06)	2.57E-01	0.06 (-0.01, 0.14)	2.02E-01
0.01 (-0.03, 0.04)	7.40E-01	0.09 (0.02, 0.17)	6.68E-02
0.00 (-0.04, 0.03)	8.82E-01	-0.01 (-0.09, 0.06)	8.49E-01
0.02 (-0.01, 0.06)	3.15E-01	0.07 (-0.01, 0.15)	1.57E-01
0.02 (-0.01, 0.06)	3.15E-01	0.07 (0.00, 0.15)	1.41E-01
0.02 (-0.01, 0.06)	3.01E-01	0.07 (-0.01, 0.15)	1.60E-01
0.02 (-0.01, 0.06)	2.96E-01	0.08 (0.00, 0.15)	1.27E-01
0.03 (-0.01, 0.06)	2.40E-01	0.07 (-0.01, 0.15)	1.56E-01
0.01 (-0.02, 0.05)	5.90E-01	0.10 (0.02, 0.17)	6.51E-02
-0.01 (-0.04, 0.03)	7.93E-01	0.01 (-0.07, 0.08)	9.19E-01
0.02 (-0.01, 0.06)	2.96E-01	0.07 (-0.01, 0.14)	1.79E-01
0.02 (-0.01, 0.06)	2.97E-01	0.07 (-0.01, 0.14)	1.66E-01
0.03 (-0.01, 0.07)	2.12E-01	0.05 (-0.03, 0.12)	3.69E-01
0.02 (-0.01, 0.06)	3.02E-01	0.07 (0.00, 0.15)	1.38E-01
0.03 (-0.01, 0.06)	2.86E-01	0.07 (0.00, 0.15)	1.42E-01
0.02 (-0.02, 0.05)	4.79E-01	0.07 (0.00, 0.15)	1.42E-01
0.00 (-0.04, 0.03)	8.47E-01	0.01 (-0.06, 0.09)	8.26E-01
0.02 (-0.01, 0.06)	3.38E-01	0.05 (-0.03, 0.13)	2.89E-01
0.02 (-0.02, 0.06)	3.67E-01	0.05 (-0.02, 0.13)	2.67E-01
0.02 (-0.02, 0.05)	4.21E-01	0.02 (-0.05, 0.10)	6.94E-01
0.02 (-0.02, 0.06)	4.14E-01	0.07 (-0.01, 0.15)	1.60E-01
0.02 (-0.02, 0.05)	4.48E-01	0.07 (-0.01, 0.15)	1.52E-01
0.01 (-0.03, 0.05)	6.98E-01	0.06 (-0.02, 0.13)	2.64E-01
0.02 (-0.01, 0.06)	2.97E-01	-0.03 (-0.11, 0.04)	5.27E-01
-0.04 (-0.08, -0.01)	6.13E-02	-0.01 (-0.08, 0.07)	8.94E-01
-0.04 (-0.08, -0.01)	5.94E-02	-0.01 (-0.08, 0.07)	8.85E-01
-0.05 (-0.08, -0.01)	**4.05E-02**	0.02 (-0.06, 0.09)	7.65E-01
-0.04 (-0.07, 0.00)	9.81E-02	-0.03 (-0.11, 0.04)	5.73E-01
-0.03 (-0.07, 0.00)	1.53E-01	-0.04 (-0.11, 0.04)	4.72E-01
-0.05 (-0.08, -0.01)	**4.96E-02**	-0.01 (-0.09, 0.06)	8.38E-01
0.03 (-0.01, 0.06)	2.13E-01	-0.07 (-0.15, 0.00)	1.38E-01
-0.06 (-0.09, -0.02)	**1.69E-02**	-0.01 (-0.09, 0.07)	8.52E-01
-0.06 (-0.09, -0.02)	**1.56E-02**	-0.01 (-0.09, 0.07)	8.67E-01
-0.06 (-0.10, -0.02)	**1.56E-02**	-0.01 (-0.09, 0.07)	8.73E-01
-0.06 (-0.10, -0.03)	**1.56E-02**	-0.01 (-0.09, 0.07)	8.73E-01
-0.06 (-0.10, -0.03)	**1.56E-02**	-0.01 (-0.09, 0.06)	8.30E-01
-0.06 (-0.09, -0.02)	**1.56E-02**	0.00 (-0.08, 0.08)	9.86E-01
0.00 (-0.03, 0.04)	9.05E-01	-0.03 (-0.11, 0.05)	6.56E-01
-0.05 (-0.08, -0.01)	**4.96E-02**	-0.08 (-0.15, 0.00)	1.27E-01
-0.05 (-0.09, -0.01)	**4.05E-02**	-0.07 (-0.15, 0.00)	1.38E-01
-0.05 (-0.08, -0.01)	**4.20E-02**	-0.07 (-0.14, 0.01)	1.52E-01
-0.06 (-0.09, -0.02)	**1.86E-02**	-0.07 (-0.14, 0.01)	1.66E-01
-0.06 (-0.09, -0.02)	**2.06E-02**	-0.07 (-0.14, 0.01)	1.51E-01
-0.06 (-0.09, -0.02)	**1.56E-02**	-0.05 (-0.13, 0.02)	2.89E-01
0.04 (0.00, 0.08)	7.50E-02	-0.08 (-0.15, 0.00)	1.27E-01

-0.02 (-0.06, 0.01)	2.86E-01	-0.06 (-0.14, 0.01)	2.04E-01
-0.03 (-0.06, 0.01)	2.37E-01	-0.06 (-0.13, 0.02)	2.55E-01
-0.02 (-0.06, 0.01)	2.86E-01	-0.09 (-0.17, -0.02)	6.99E-02
-0.02 (-0.06, 0.01)	2.92E-01	0.04 (-0.03, 0.12)	3.78E-01
-0.02 (-0.05, 0.02)	4.79E-01	0.06 (-0.02, 0.14)	2.15E-01
-0.04 (-0.08, -0.01)	5.94E-02	-0.08 (-0.15, 0.00)	1.12E-01
0.03 (0.00, 0.07)	1.67E-01	-0.07 (-0.15, 0.00)	1.36E-01
0.00 (-0.03, 0.04)	8.50E-01	-0.07 (-0.14, 0.01)	1.88E-01
0.04 (0.00, 0.07)	9.24E-02	0.02 (-0.06, 0.10)	7.36E-01
0.04 (0.00, 0.07)	7.66E-02	0.03 (-0.05, 0.10)	6.76E-01
0.01 (-0.03, 0.05)	6.86E-01	-0.01 (-0.08, 0.07)	9.26E-01
-0.04 (-0.07, 0.00)	1.15E-01	0.01 (-0.07, 0.08)	9.17E-01
0.05 (0.01, 0.08)	**4.96E-02**	-0.03 (-0.11, 0.05)	6.09E-01
0.06 (0.02, 0.09)	**2.06E-02**	0.13 (0.05, 0.21)	**2.90E-02**
0.03 (-0.01, 0.06)	2.70E-01	0.14 (0.07, 0.22)	**2.90E-02**
0.06 (0.02, 0.09)	**2.29E-02**	0.04 (-0.04, 0.12)	4.25E-01
-0.07 (-0.11, -0.04)	**1.11E-02**	-0.08 (-0.16, 0.00)	1.36E-01
0.03 (-0.01, 0.06)	2.29E-01	-0.02 (-0.10, 0.05)	6.94E-01
0.05 (0.01, 0.08)	**4.35E-02**	0.04 (-0.04, 0.11)	5.13E-01
0.03 (0.00, 0.07)	1.66E-01	0.11 (0.03, 0.19)	**3.85E-02**
0.03 (-0.01, 0.06)	2.12E-01	-0.05 (-0.13, 0.02)	2.84E-01
-0.05 (-0.08, -0.01)	**4.96E-02**	-0.02 (-0.10, 0.05)	6.95E-01
-0.02 (-0.06, 0.01)	2.91E-01	0.08 (0.01, 0.16)	1.01E-01
0.04 (0.00, 0.08)	9.24E-02	0.10 (0.03, 0.18)	**4.60E-02**
0.03 (0.00, 0.07)	1.68E-01	0.13 (0.05, 0.20)	**2.90E-02**
0.02 (-0.02, 0.05)	4.25E-01	-0.05 (-0.13, 0.02)	2.65E-01
-0.03 (-0.07, 0.00)	1.71E-01	-0.11 (-0.18, -0.03)	**3.96E-02**
-0.08 (-0.12, -0.05)	**4.28E-04**	-0.02 (-0.10, 0.05)	6.76E-01
0.01 (-0.02, 0.05)	5.77E-01	0.11 (0.04, 0.19)	**3.13E-02**
0.02 (-0.01, 0.06)	3.15E-01	0.12 (0.05, 0.20)	**2.90E-02**
-0.06 (-0.10, -0.03)	**1.56E-02**	0.00 (-0.07, 0.07)	9.99E-01
0.01 (-0.03, 0.04)	8.03E-01	-0.10 (-0.18, -0.03)	**4.37E-02**
-0.01 (-0.05, 0.02)	5.00E-01	0.07 (0.00, 0.15)	1.40E-01
0.01 (-0.02, 0.05)	5.02E-01	0.11 (0.03, 0.18)	**3.85E-02**
0.03 (-0.01, 0.06)	2.66E-01	0.09 (0.02, 0.17)	6.99E-02
-0.02 (-0.05, 0.02)	4.21E-01	0.08 (0.01, 0.16)	9.64E-02
0.00 (-0.04, 0.03)	8.42E-01	-0.11 (-0.19, -0.04)	**3.13E-02**
0.00 (-0.04, 0.03)	9.08E-01	0.05 (-0.02, 0.13)	2.67E-01
0.03 (-0.01, 0.06)	2.41E-01	0.09 (0.01, 0.17)	7.47E-02
0.04 (0.01, 0.08)	5.17E-02	0.00 (-0.08, 0.07)	9.69E-01
-0.01 (-0.05, 0.02)	5.93E-01	0.12 (0.05, 0.20)	**2.90E-02**
-0.03 (-0.06, 0.01)	2.74E-01	-0.10 (-0.18, -0.03)	**4.32E-02**
-0.02 (-0.05, 0.02)	3.81E-01	-0.08 (-0.16, -0.01)	1.07E-01
0.03 (-0.01, 0.06)	2.09E-01	0.10 (0.02, 0.17)	6.51E-02
0.04 (0.01, 0.08)	5.40E-02	0.06 (-0.01, 0.14)	2.05E-01
-0.03 (-0.07, 0.00)	1.17E-01	0.12 (0.04, 0.19)	**2.90E-02**
-0.03 (-0.07, 0.01)	1.90E-01	-0.09 (-0.17, -0.02)	7.10E-02
-0.01 (-0.05, 0.02)	5.58E-01	-0.09 (-0.16, -0.01)	8.50E-02

0.02 (-0.01, 0.06)	3.15E-01	0.09 (0.01, 0.16)	8.17E-02
0.03 (-0.01, 0.06)	2.22E-01	0.08 (0.01, 0.16)	1.07E-01
-0.04 (-0.08, 0.00)	7.66E-02	-0.05 (-0.12, 0.03)	3.63E-01
-0.03 (-0.07, 0.00)	1.21E-01	-0.08 (-0.15, 0.00)	1.28E-01
-0.02 (-0.06, 0.01)	3.17E-01	-0.09 (-0.16, -0.01)	9.35E-02
0.02 (-0.02, 0.05)	4.79E-01	0.09 (0.02, 0.17)	6.86E-02
0.02 (-0.01, 0.06)	3.11E-01	0.09 (0.01, 0.16)	9.16E-02
-0.05 (-0.08, -0.01)	**4.96E-02**	-0.04 (-0.11, 0.04)	4.92E-01
0.00 (-0.04, 0.03)	9.42E-01	-0.09 (-0.17, -0.02)	6.68E-02
-0.02 (-0.06, 0.01)	3.17E-01	0.09 (0.01, 0.17)	8.30E-02
0.01 (-0.03, 0.04)	7.37E-01	-0.09 (-0.17, -0.01)	1.07E-01
0.02 (-0.02, 0.06)	3.91E-01	-0.09 (-0.17, -0.01)	8.62E-02
-0.04 (-0.08, -0.01)	6.98E-02	-0.02 (-0.09, 0.06)	7.65E-01
0.04 (0.01, 0.08)	5.28E-02	-0.06 (-0.14, 0.01)	1.99E-01
0.03 (-0.01, 0.06)	2.13E-01	0.01 (-0.07, 0.09)	9.05E-01
-0.05 (-0.09, -0.02)	**2.34E-02**	0.01 (-0.07, 0.09)	8.73E-01
-0.06 (-0.09, -0.02)	**2.19E-02**	-0.02 (-0.10, 0.06)	6.94E-01
-0.04 (-0.08, -0.01)	6.54E-02	0.05 (-0.02, 0.13)	2.89E-01
0.06 (0.02, 0.09)	**2.34E-02**	-0.03 (-0.11, 0.05)	5.76E-01
0.01 (-0.02, 0.05)	6.04E-01	0.04 (-0.04, 0.11)	4.74E-01
-0.05 (-0.08, -0.01)	5.20E-02	-0.01 (-0.09, 0.06)	8.67E-01
-0.04 (-0.07, 0.00)	1.17E-01	-0.01 (-0.09, 0.07)	8.73E-01
-0.06 (-0.10, -0.03)	**1.56E-02**	-0.01 (-0.08, 0.07)	9.05E-01
0.06 (0.03, 0.10)	**1.56E-02**	-0.02 (-0.09, 0.06)	7.59E-01
-0.01 (-0.04, 0.03)	8.24E-01	-0.09 (-0.17, -0.02)	6.51E-02
-0.01 (-0.04, 0.03)	7.40E-01	0.09 (0.02, 0.17)	6.51E-02
0.00 (-0.04, 0.03)	9.05E-01	0.09 (0.02, 0.17)	6.51E-02
-0.04 (-0.07, 0.00)	9.24E-02	-0.08 (-0.16, -0.01)	1.08E-01
0.04 (0.01, 0.08)	6.98E-02	-0.05 (-0.13, 0.02)	2.81E-01
0.04 (0.00, 0.07)	9.51E-02	-0.10 (-0.17, -0.02)	6.08E-02
0.00 (-0.03, 0.04)	8.66E-01	0.10 (0.02, 0.18)	6.51E-02
-0.04 (-0.08, 0.00)	7.50E-02	0.00 (-0.07, 0.08)	9.63E-01
0.01 (-0.02, 0.05)	5.88E-01	0.02 (-0.05, 0.10)	6.76E-01
0.05 (0.02, 0.09)	**2.33E-02**	-0.06 (-0.13, 0.02)	2.65E-01
0.05 (0.01, 0.09)	**4.05E-02**	-0.01 (-0.08, 0.07)	9.12E-01
0.02 (-0.01, 0.06)	2.96E-01	0.06 (-0.01, 0.14)	1.90E-01
-0.07 (-0.10, -0.03)	**1.11E-02**	-0.02 (-0.10, 0.05)	6.85E-01
-0.07 (-0.10, -0.03)	**1.11E-02**	-0.02 (-0.10, 0.05)	6.94E-01
-0.04 (-0.08, -0.01)	5.94E-02	-0.02 (-0.10, 0.05)	6.94E-01
0.01 (-0.03, 0.05)	7.37E-01	0.03 (-0.05, 0.10)	6.41E-01
0.02 (-0.02, 0.05)	4.58E-01	0.02 (-0.05, 0.10)	6.79E-01
0.04 (0.00, 0.07)	9.62E-02	-0.11 (-0.19, -0.04)	**3.13E-02**
0.04 (0.00, 0.07)	7.50E-02	-0.12 (-0.19, -0.04)	**2.90E-02**
0.00 (-0.04, 0.03)	9.08E-01	-0.03 (-0.11, 0.04)	5.30E-01
0.03 (0.00, 0.07)	1.43E-01	-0.08 (-0.15, 0.00)	1.10E-01
0.00 (-0.04, 0.03)	9.41E-01	-0.04 (-0.12, 0.03)	3.78E-01
0.04 (0.01, 0.08)	5.17E-02	-0.09 (-0.17, -0.01)	7.73E-02
-0.01 (-0.05, 0.03)	6.81E-01	-0.03 (-0.11, 0.04)	5.30E-01

0.00 (-0.04, 0.03)	8.82E-01	0.01 (-0.06, 0.09)	8.26E-01
-0.01 (-0.05, 0.02)	6.25E-01	-0.02 (-0.10, 0.06)	7.09E-01
-0.04 (-0.08, -0.01)	7.15E-02	-0.05 (-0.13, 0.02)	2.59E-01
0.05 (0.01, 0.08)	**4.96E-02**	-0.03 (-0.11, 0.05)	5.62E-01
0.06 (0.03, 0.10)	**1.56E-02**	0.00 (-0.07, 0.08)	9.35E-01
0.03 (-0.01, 0.06)	2.55E-01	-0.08 (-0.16, -0.01)	1.07E-01
0.02 (-0.02, 0.06)	4.21E-01	0.03 (-0.04, 0.11)	5.30E-01
0.04 (0.01, 0.08)	5.94E-02	-0.06 (-0.14, 0.03)	2.89E-01
0.03 (-0.01, 0.06)	2.86E-01	-0.05 (-0.13, 0.02)	2.88E-01
0.05 (0.02, 0.09)	**3.31E-02**	-0.09 (-0.17, -0.01)	9.80E-02
0.02 (-0.01, 0.06)	3.15E-01	-0.05 (-0.13, 0.02)	2.88E-01
0.03 (-0.01, 0.07)	2.12E-01	-0.06 (-0.14, 0.02)	2.15E-01
0.02 (-0.01, 0.06)	3.38E-01	-0.10 (-0.18, -0.02)	5.37E-02
0.03 (-0.01, 0.06)	2.66E-01	-0.07 (-0.15, 0.00)	1.38E-01
0.03 (-0.01, 0.06)	2.13E-01	0.02 (-0.06, 0.10)	7.48E-01
0.00 (-0.03, 0.04)	8.50E-01	0.02 (-0.05, 0.10)	6.78E-01
0.05 (0.02, 0.09)	**2.33E-02**	-0.03 (-0.11, 0.06)	6.78E-01
-0.01 (-0.04, 0.03)	8.04E-01	0.05 (-0.03, 0.13)	3.12E-01
0.01 (-0.03, 0.05)	6.25E-01	0.05 (-0.03, 0.12)	3.69E-01
0.00 (-0.04, 0.03)	8.47E-01	-0.08 (-0.16, -0.01)	9.89E-02
-0.01 (-0.05, 0.02)	6.31E-01	0.04 (-0.04, 0.12)	4.29E-01
0.00 (-0.03, 0.04)	8.47E-01	0.00 (-0.08, 0.08)	9.99E-01
0.01 (-0.02, 0.05)	6.09E-01	-0.08 (-0.15, 0.00)	1.32E-01
-0.01 (-0.04, 0.03)	7.78E-01	-0.05 (-0.13, 0.03)	3.31E-01
0.02 (-0.01, 0.05)	3.64E-01	-0.02 (-0.10, 0.05)	6.87E-01
0.00 (-0.04, 0.03)	9.58E-01	0.00 (-0.07, 0.08)	9.69E-01
0.02 (-0.01, 0.06)	2.86E-01	0.07 (-0.01, 0.14)	1.60E-01
0.03 (0.00, 0.07)	1.17E-01	-0.06 (-0.14, 0.02)	2.38E-01
0.03 (-0.01, 0.06)	2.12E-01	-0.04 (-0.11, 0.04)	5.00E-01
0.02 (-0.02, 0.05)	4.47E-01	-0.01 (-0.08, 0.07)	9.05E-01
0.04 (0.00, 0.07)	7.66E-02	0.06 (-0.01, 0.14)	1.90E-01
0.03 (-0.01, 0.06)	2.55E-01	0.02 (-0.06, 0.09)	7.59E-01
-0.01 (-0.05, 0.02)	5.13E-01	0.00 (-0.08, 0.08)	9.99E-01
-0.03 (-0.06, 0.01)	2.66E-01	-0.06 (-0.13, 0.02)	2.42E-01
-0.05 (-0.09, -0.01)	**4.05E-02**	-0.07 (-0.15, 0.01)	1.66E-01
-0.01 (-0.04, 0.03)	7.52E-01	0.02 (-0.06, 0.09)	7.65E-01
-0.01 (-0.05, 0.02)	4.95E-01	-0.04 (-0.12, 0.03)	3.78E-01
0.04 (0.01, 0.08)	5.40E-02	-0.09 (-0.17, -0.01)	9.64E-02

**Metabolites**	**Group**
Concentration of chylomicrons and extremely large VLDL particles	Lipoprotein subclasses
Total lipids in chylomicrons and extremely large VLDL	Lipoprotein subclasses
Phospholipids in chylomicrons and extremely large VLDL	Lipoprotein subclasses
Cholesterol in chylomicrons and extremely large VLDL	Lipoprotein subclasses
Cholesteryl esters in chylomicrons and extremely large VLDL	Lipoprotein subclasses
Free cholesterol in chylomicrons and extremely large VLDL	Lipoprotein subclasses
Triglycerides in chylomicrons and extremely large VLDL	Lipoprotein subclasses
Concentration of very large VLDL particles	Lipoprotein subclasses
Total lipids in very large VLDL	Lipoprotein subclasses
Phospholipids in very large VLDL	Lipoprotein subclasses
Cholesterol in very large VLDL	Lipoprotein subclasses
Cholesteryl esters in very large VLDL	Lipoprotein subclasses
Free cholesterol in very large VLDL	Lipoprotein subclasses
Triglycerides in very large VLDL	Lipoprotein subclasses
Concentration of large VLDL particles	Lipoprotein subclasses
Total lipids in large VLDL	Lipoprotein subclasses
Phospholipids in large VLDL	Lipoprotein subclasses
Cholesterol in large VLDL	Lipoprotein subclasses
Cholesteryl esters in large VLDL	Lipoprotein subclasses
Free cholesterol in large VLDL	Lipoprotein subclasses
Triglycerides in large VLDL	Lipoprotein subclasses
Concentration of medium VLDL particles	Lipoprotein subclasses
Total lipids in medium VLDL	Lipoprotein subclasses
Phospholipids in medium VLDL	Lipoprotein subclasses
Cholesterol in medium VLDL	Lipoprotein subclasses
Cholesteryl esters in medium VLDL	Lipoprotein subclasses
Free cholesterol in medium VLDL	Lipoprotein subclasses
Triglycerides in medium VLDL	Lipoprotein subclasses
Concentration of small VLDL particles	Lipoprotein subclasses
Total lipids in small VLDL	Lipoprotein subclasses
Phospholipids in small VLDL	Lipoprotein subclasses
Cholesterol in small VLDL	Lipoprotein subclasses
Cholesteryl esters in small VLDL	Lipoprotein subclasses
Free cholesterol in small VLDL	Lipoprotein subclasses
Triglycerides in small VLDL	Lipoprotein subclasses
Concentration of very small VLDL particles	Lipoprotein subclasses
Total lipids in very small VLDL	Lipoprotein subclasses
Phospholipids in very small VLDL	Lipoprotein subclasses
Cholesterol in very small VLDL	Lipoprotein subclasses
Cholesteryl esters in very small VLDL	Lipoprotein subclasses
Free cholesterol in very small VLDL	Lipoprotein subclasses
Triglycerides in very small VLDL	Lipoprotein subclasses
Concentration of IDL particles	Lipoprotein subclasses

Total lipids in IDL	Lipoprotein subclasses
Phospholipids in IDL	Lipoprotein subclasses
Cholesterol in IDL	Lipoprotein subclasses
Cholesteryl esters in IDL	Lipoprotein subclasses
Free cholesterol in IDL	Lipoprotein subclasses
Triglycerides in IDL	Lipoprotein subclasses
Concentration of large LDL particles	Lipoprotein subclasses
Total lipids in large LDL	Lipoprotein subclasses
Phospholipids in large LDL	Lipoprotein subclasses
Cholesterol in large LDL	Lipoprotein subclasses
Cholesteryl esters in large LDL	Lipoprotein subclasses
Free cholesterol in large LDL	Lipoprotein subclasses
Triglycerides in large LDL	Lipoprotein subclasses
Concentration of medium LDL particles	Lipoprotein subclasses
Total lipids in medium LDL	Lipoprotein subclasses
Phospholipids in medium LDL	Lipoprotein subclasses
Cholesterol in medium LDL	Lipoprotein subclasses
Cholesteryl esters in medium LDL	Lipoprotein subclasses
Free cholesterol in medium LDL	Lipoprotein subclasses
Triglycerides in medium LDL	Lipoprotein subclasses
Concentration of small LDL particles	Lipoprotein subclasses
Total lipids in small LDL	Lipoprotein subclasses
Phospholipids in small LDL	Lipoprotein subclasses
Cholesterol in small LDL	Lipoprotein subclasses
Cholesteryl esters in small LDL	Lipoprotein subclasses
Free cholesterol in small LDL	Lipoprotein subclasses
Triglycerides in small LDL	Lipoprotein subclasses
Concentration of very large HDL particles	Lipoprotein subclasses
Total lipids in very large HDL	Lipoprotein subclasses
Phospholipids in very large HDL	Lipoprotein subclasses
Cholesterol in very large HDL	Lipoprotein subclasses
Cholesteryl esters in very large HDL	Lipoprotein subclasses
Free cholesterol in very large HDL	Lipoprotein subclasses
Triglycerides in very large HDL	Lipoprotein subclasses
Concentration of large HDL particles	Lipoprotein subclasses
Total lipids in large HDL	Lipoprotein subclasses
Phospholipids in large HDL	Lipoprotein subclasses
Cholesterol in large HDL	Lipoprotein subclasses
Cholesteryl esters in large HDL	Lipoprotein subclasses
Free cholesterol in large HDL	Lipoprotein subclasses
Triglycerides in large HDL	Lipoprotein subclasses
**Concentration of medium HDL particles**	Lipoprotein subclasses
**Total lipids in medium HDL**	Lipoprotein subclasses
**Phospholipids in medium HDL**	Lipoprotein subclasses
**Cholesterol in medium HDL**	Lipoprotein subclasses
**Cholesteryl esters in medium HDL**	Lipoprotein subclasses
**Free cholesterol in medium HDL**	Lipoprotein subclasses
Triglycerides in medium HDL	Lipoprotein subclasses

Concentration of small HDL particles	Lipoprotein subclasses
Total lipids in small HDL	Lipoprotein subclasses
Phospholipids in small HDL	Lipoprotein subclasses
Cholesterol in small HDL	Lipoprotein subclasses
Cholesteryl esters in small HDL	Lipoprotein subclasses
**Free cholesterol in small HDL**	Lipoprotein subclasses
Triglycerides in small HDL	Lipoprotein subclasses
Phospholipids to total lipids ratio in chylomicrons and extremely large	Relative lipoprotein lipid co
Cholesterol to total lipids ratio in chylomicrons and extremely large VI	Relative lipoprotein lipid co
Cholesteryl esters to total lipids ratio in chylomicrons and extremely la	Relative lipoprotein lipid co
Free cholesterol to total lipids ratio in chylomicrons and extremely larg	Relative lipoprotein lipid co
Triglycerides to total lipids ratio in chylomicrons and extremely large V	Relative lipoprotein lipid co
Phospholipids to total lipids ratio in very large VLDL /(lipds in ckb)	Relative lipoprotein lipid co
**Cholesterol to total lipids ratio in very large VLDL**	Relative lipoprotein lipid co
Cholesteryl esters to total lipids ratio in very large VLDL	Relative lipoprotein lipid co
**Free cholesterol to total lipids ratio in very large VLDL**	Relative lipoprotein lipid co
**Triglycerides to total lipids ratio in very large VLDL**	Relative lipoprotein lipid co
Phospholipids to total lipids ratio in large VLDL	Relative lipoprotein lipid co
Cholesterol to total lipids ratio in large VLDL	Relative lipoprotein lipid co
Cholesteryl esters to total lipids ratio in large VLDL	Relative lipoprotein lipid co
Free cholesterol to total lipids ratio in large VLDL	Relative lipoprotein lipid co
Triglycerides to total lipids ratio in large VLDL	Relative lipoprotein lipid co
Phospholipids to total lipids ratio in medium VLDL	Relative lipoprotein lipid co
**Cholesterol to total lipids ratio in medium VLDL**	Relative lipoprotein lipid co
**Cholesteryl esters to total lipids ratio in medium VLDL**	Relative lipoprotein lipid co
Free cholesterol to total lipids ratio in medium VLDL	Relative lipoprotein lipid co
Triglycerides to total lipids ratio in medium VLDL	Relative lipoprotein lipid co
**Phospholipids to total lipids ratio in small VLDL**	Relative lipoprotein lipid co
Cholesterol to total lipids ratio in small VLDL	Relative lipoprotein lipid co
Cholesteryl esters to total lipids ratio in small VLDL	Relative lipoprotein lipid co
**Free cholesterol to total lipids ratio in small VLDL**	Relative lipoprotein lipid co
Triglycerides to total lipids ratio in small VLDL	Relative lipoprotein lipid co
Phospholipids to total lipids ratio in very small VLDL	Relative lipoprotein lipid co
Cholesterol to total lipids ratio in very small VLDL	Relative lipoprotein lipid co
Cholesteryl esters to total lipids ratio in very small VLDL	Relative lipoprotein lipid co
Free cholesterol to total lipids ratio in very small VLDL	Relative lipoprotein lipid co
Triglycerides to total lipids ratio in very small VLDL	Relative lipoprotein lipid co
Phospholipids to total lipids ratio in IDL	Relative lipoprotein lipid co
Cholesterol to total lipids ratio in IDL	Relative lipoprotein lipid co
Cholesteryl esters to total lipids ratio in IDL	Relative lipoprotein lipid co
Free cholesterol to total lipids ratio in IDL	Relative lipoprotein lipid co
Triglycerides to total lipids ratio in IDL	Relative lipoprotein lipid co
Phospholipids to total lipids ratio in large LDL	Relative lipoprotein lipid co
Cholesterol to total lipids ratio in large LDL	Relative lipoprotein lipid co
Cholesteryl esters to total lipids ratio in large LDL	Relative lipoprotein lipid co
Free cholesterol to total lipids ratio in large LDL	Relative lipoprotein lipid co
Triglycerides to total lipids ratio in large LDL	Relative lipoprotein lipid co
Phospholipids to total lipids ratio in medium LDL	Relative lipoprotein lipid co

Cholesterol to total lipids ratio in medium LDL	Relative lipoprotein lipid co
Cholesteryl esters to total lipids ratio in medium LDL	Relative lipoprotein lipid co
Free cholesterol to total lipids ratio in medium LDL	Relative lipoprotein lipid co
Triglycerides to total lipids ratio in medium LDL	Relative lipoprotein lipid co
Phospholipids to total lipids ratio in small LDL	Relative lipoprotein lipid co
Cholesterol to total lipids ratio in small LDL	Relative lipoprotein lipid co
Cholesteryl esters to total lipids ratio in small LDL	Relative lipoprotein lipid co
Free cholesterol to total lipids ratio in small LDL	Relative lipoprotein lipid co
Triglycerides to total lipids ratio in small LDL	Relative lipoprotein lipid co
Phospholipids to total lipids ratio in very large HDL	Relative lipoprotein lipid co
Cholesterol to total lipids ratio in very large HDL	Relative lipoprotein lipid co
Cholesteryl esters to total lipids ratio in very large HDL	Relative lipoprotein lipid co
Free cholesterol to total lipids ratio in very large HDL	Relative lipoprotein lipid co
Triglycerides to total lipids ratio in very large HDL	Relative lipoprotein lipid co
Phospholipids to total lipids ratio in large HDL	Relative lipoprotein lipid co
Cholesterol to total lipids ratio in large HDL	Relative lipoprotein lipid co
Cholesteryl esters to total lipids ratio in large HDL	Relative lipoprotein lipid co
Free cholesterol to total lipids ratio in large HDL	Relative lipoprotein lipid co
Triglycerides to total lipids ratio in large HDL	Relative lipoprotein lipid co
Phospholipids to total lipids ratio in medium HDL	Relative lipoprotein lipid co
Cholesterol to total lipids ratio in medium HDL	Relative lipoprotein lipid co
Cholesteryl esters to total lipids ratio in medium HDL	Relative lipoprotein lipid co
Free cholesterol to total lipids ratio in medium HDL	Relative lipoprotein lipid co
Triglycerides to total lipids ratio in medium HDL	Relative lipoprotein lipid co
Phospholipids to total lipids ratio in small HDL	Relative lipoprotein lipid co
Cholesterol to total lipids ratio in small HDL	Relative lipoprotein lipid co
Cholesteryl esters to total lipids ratio in small HDL	Relative lipoprotein lipid co
Free cholesterol to total lipids ratio in small HDL	Relative lipoprotein lipid co
Triglycerides to total lipids ratio in small HDL	Relative lipoprotein lipid co
Average diameter for VLDL particles	Lipoprotein particle sizes
Average diameter for LDL particles	Lipoprotein particle sizes
Average diameter for HDL particles	Lipoprotein particle sizes
Total cholesterol	Cholesterol
VLDL cholesterol	Cholesterol
Remnant cholesterol (non-HDL, non-LDL -cholesterol)	Cholesterol
LDL cholesterol	Cholesterol
**HDL cholesterol**	Cholesterol
Total esterified cholesterol	Cholesteryl esters
Total free cholesterol	Free cholesterol
Total triglycerides	Triglycerides
Triglycerides in VLDL	Triglycerides
Triglycerides in LDL	Triglycerides
Triglycerides in HDL	Triglycerides
Phosphoglycerides	Other lipids
Ratio of triglycerides to phosphoglycerides	Other lipids
Phosphatidylcholines	Other lipids
Sphingomyelins	Other lipids
Total cholines	Other lipids

Apolipoprotein A1	Apolipoproteins
Apolipoprotein B	Apolipoproteins
Ratio of apolipoprotein B to apolipoprotein A1	Apolipoproteins
Total fatty acids	Fatty acids
Degree of unsaturation	Fatty acids
Docosahexaenoic acid	Fatty acids
Linoleic acid	Fatty acids
Omega-3 fatty acids	Fatty acids
Omega-6 fatty acids	Fatty acids
Polyunsaturated fatty acids	Fatty acids
Monounsaturated fatty acids	Fatty acids
Saturated fatty acids	Fatty acids
Ratio of docosahexaenoic acid to total fatty acids	Fatty acids
Ratio of linoleic acid to total fatty acids	Fatty acids
Ratio of omega-3 fatty acids to total fatty acids	Fatty acids
Ratio of omega-6 fatty acids to total fatty acids	Fatty acids
Ratio of polyunsaturated fatty acids to total fatty acids	Fatty acids
Ratio of monounsaturated fatty acids to total fatty acids	Fatty acids
Ratio of saturated fatty acids to total fatty acids	Fatty acids
Glucose	Glycolysis related metaboli
Lactate	Glycolysis related metaboli
Citrate	Glycolysis related metaboli
Alanine	Amino acids
Glutamine	Amino acids
Histidine	Amino acids
Isoleucine	Amino acids
Leucine	Amino acids
Valine	Amino acids
Phenylalanine	Amino acids
Tyrosine	Amino acids
Acetate	Ketone bodies
Acetoacetate	Ketone bodies
**3-Hydroxybutyrate**	Ketone bodies
Creatinine	Fluid balance
Albumin	Fluid balance
Glycoprotein acetyls	Inflammation
Acetone	Ketone bodies
Cholesteryl esters in HDL	Cholesteryl esters
Cholesteryl esters in LDL	Cholesteryl esters
Cholesteryl esters in VLDL/ (Cholesterol in ckb)	Cholesteryl esters
Clinical LDL cholesterol	Cholesterol
Concentration of HDL particles	Lipoprotein particle concen
Concentration of LDL particles	Lipoprotein particle concen
Concentration of VLDL particles	Lipoprotein particle concen
Free cholesterol in HDL	Free cholesterol
Free cholesterol in LDL	Free cholesterol
Free cholesterol in VLDL	Free cholesterol
Glycine	Amino acids

Phospholipids in HDL	Phospholipids
Phospholipids in LDL	Phospholipids
Phospholipids in VLDL	Phospholipids
Pyruvate	Glycolysis related metaboli
Ratio of omega-6 fatty acids to omega-3 fatty acids	Fatty acids
Ratio of polyunsaturated fatty acids to monounsaturated fatty acids	Fatty acids
Total cholesterol minus HDL-C	Cholesterol
Total concentration of branched-chain amino acids (leucine + isoleucin	Amino acids
Total concentration of lipoprotein particles	Lipoprotein particle concen
Total lipids in HDL	Total lipids
Total lipids in LDL	Total lipids
Total lipids in lipoprotein particles	Total lipids
Total lipids in VLDL	Total lipids
Total phospholipids in lipoprotein particles	Phospholipids

Models were adjusted for age, sex, ethnicity, levels of education, smoking status, alcohol consumption, and s

CI, confidence interval; LDL, Low Density Lipoprotein; HDL, High Density Lipoprotein; VLDL, Very Low

ry Table 7. Association of all metabolites with sleep duration in the UKB in subgroups analysis

Male		Female		Age < 60y
Adjusted β (95% CI)	P value	Adjusted β (95% CI)	P value	Adjusted β (95% CI)
0.03 (0.02, 0.03)	**2.07E-19**	0.03 (0.02, 0.03)	**2.87E-17**	0.02 (0.01, 0.02)
0.03 (0.02, 0.03)	**5.53E-18**	0.03 (0.02, 0.03)	**6.02E-16**	0.02 (0.01, 0.02)
0.03 (0.02, 0.03)	**1.10E-19**	0.03 (0.02, 0.03)	**4.62E-18**	0.02 (0.01, 0.02)
0.03 (0.02, 0.03)	**1.03E-19**	0.03 (0.02, 0.03)	**1.10E-16**	0.02 (0.01, 0.03)
0.03 (0.02, 0.03)	**5.87E-19**	0.03 (0.02, 0.03)	**3.11E-15**	0.02 (0.01, 0.02)
0.03 (0.02, 0.03)	**3.51E-20**	0.03 (0.02, 0.03)	**4.62E-18**	0.02 (0.01, 0.03)
0.03 (0.02, 0.03)	**1.59E-16**	0.02 (0.02, 0.03)	**7.44E-15**	0.02 (0.01, 0.02)
0.03 (0.02, 0.03)	**1.95E-18**	0.02 (0.02, 0.03)	**5.37E-15**	0.02 (0.01, 0.02)
0.03 (0.02, 0.03)	**4.85E-18**	0.02 (0.02, 0.03)	**2.54E-14**	0.02 (0.01, 0.02)
0.03 (0.02, 0.03)	**2.07E-19**	0.02 (0.02, 0.03)	**8.92E-15**	0.02 (0.01, 0.03)
0.03 (0.02, 0.03)	**9.42E-18**	0.02 (0.01, 0.03)	**7.25E-11**	0.02 (0.01, 0.02)
0.02 (0.02, 0.03)	**4.59E-15**	0.02 (0.01, 0.03)	**4.82E-07**	0.02 (0.01, 0.02)
0.03 (0.02, 0.03)	**1.01E-19**	0.02 (0.02, 0.03)	**1.15E-14**	0.02 (0.01, 0.03)
0.03 (0.02, 0.03)	**6.91E-17**	0.02 (0.02, 0.03)	**9.84E-15**	0.02 (0.01, 0.02)
0.03 (0.02, 0.03)	**9.42E-18**	0.02 (0.02, 0.03)	**4.87E-14**	0.02 (0.01, 0.03)
0.02 (0.02, 0.03)	**5.81E-16**	0.02 (0.02, 0.03)	**1.57E-12**	0.02 (0.01, 0.02)
0.03 (0.02, 0.03)	**1.95E-18**	0.02 (0.02, 0.03)	**3.50E-14**	0.02 (0.01, 0.03)
0.03 (0.02, 0.03)	**7.09E-18**	0.02 (0.02, 0.03)	**1.29E-11**	0.02 (0.01, 0.03)
0.03 (0.02, 0.03)	**1.87E-17**	0.02 (0.01, 0.03)	**1.11E-09**	0.02 (0.01, 0.03)
0.03 (0.02, 0.03)	**1.07E-17**	0.02 (0.02, 0.03)	**6.32E-13**	0.02 (0.01, 0.02)
0.02 (0.02, 0.03)	**4.68E-13**	0.02 (0.02, 0.03)	**1.17E-11**	0.02 (0.01, 0.02)
0.02 (0.01, 0.02)	**3.09E-09**	0.01 (0.00, 0.02)	**1.87E-03**	0.01 (0.01, 0.02)
0.02 (0.01, 0.03)	**8.57E-11**	0.01 (0.01, 0.03)	**1.92E-05**	0.02 (0.01, 0.02)
0.02 (0.01, 0.02)	**5.84E-09**	0.01 (0.00, 0.02)	**4.98E-03**	0.01 (0.01, 0.02)
0.01 (0.00, 0.02)	**1.36E-03**	0.00 (-0.01, 0.00)	3.04E-01	0.01 (0.00, 0.01)
0.00 (0.00, 0.01)	1.36E-01	-0.01 (-0.02, 0.00)	**1.59E-03**	0.00 (0.00, 0.01)
0.02 (0.01, 0.02)	**1.94E-07**	0.01 (0.00, 0.01)	8.64E-02	0.01 (0.01, 0.02)
0.02 (0.02, 0.03)	**7.25E-14**	0.02 (0.02, 0.03)	**8.08E-12**	0.02 (0.01, 0.02)
0.03 (0.02, 0.03)	**3.08E-19**	0.02 (0.02, 0.03)	**5.42E-14**	0.02 (0.02, 0.03)
0.03 (0.02, 0.03)	**8.29E-18**	0.02 (0.02, 0.03)	**6.26E-12**	0.02 (0.02, 0.03)
0.02 (0.01, 0.03)	**2.97E-11**	0.01 (0.01, 0.02)	**1.25E-04**	0.02 (0.01, 0.02)
0.02 (0.01, 0.03)	**1.31E-10**	0.01 (0.00, 0.02)	**4.92E-03**	0.01 (0.01, 0.02)
0.02 (0.02, 0.03)	**5.46E-13**	0.01 (0.01, 0.02)	**1.97E-04**	0.02 (0.01, 0.02)
0.01 (0.01, 0.02)	**1.20E-06**	0.00 (0.00, 0.01)	2.26E-01	0.01 (0.01, 0.02)
0.03 (0.03, 0.04)	**2.15E-23**	0.03 (0.03, 0.04)	**7.58E-24**	0.03 (0.02, 0.03)
0.02 (0.02, 0.03)	**1.43E-13**	0.02 (0.01, 0.02)	**1.95E-06**	0.02 (0.01, 0.02)
0.02 (0.02, 0.03)	**5.94E-15**	0.02 (0.01, 0.02)	**3.85E-08**	0.02 (0.02, 0.03)
0.03 (0.02, 0.03)	**2.13E-18**	0.02 (0.01, 0.03)	**2.87E-11**	0.02 (0.02, 0.03)
0.01 (0.01, 0.02)	**1.17E-06**	0.00 (0.00, 0.01)	2.81E-01	0.01 (0.01, 0.02)
0.01 (0.01, 0.02)	**1.61E-04**	0.00 (-0.01, 0.01)	8.72E-01	0.01 (0.01, 0.02)
0.02 (0.02, 0.03)	**7.98E-13**	0.01 (0.01, 0.02)	**4.28E-05**	0.02 (0.01, 0.02)
0.03 (0.03, 0.04)	**3.53E-26**	0.04 (0.03, 0.04)	**2.07E-28**	0.03 (0.02, 0.03)
0.00 (0.00, 0.01)	5.49E-01	-0.01 (-0.02, 0.00)	**4.22E-03**	0.00 (-0.01, 0.01)

0.00 (-0.01, 0.00)	6.06E-01	-0.01 (-0.02, 0.00)	**1.34E-03**	0.00 (-0.01, 0.01)
0.00 (0.00, 0.01)	2.31E-01	-0.01 (-0.01, 0.00)	**4.62E-02**	0.00 (0.00, 0.01)
-0.01 (-0.01, 0.00)	**2.54E-02**	-0.02 (-0.02, -0.01)	**4.74E-07**	-0.01 (-0.01, 0.00)
-0.01 (-0.01, 0.00)	**1.03E-02**	-0.02 (-0.02, -0.01)	**2.48E-07**	-0.01 (-0.01, 0.00)
0.00 (-0.01, 0.00)	2.10E-01	-0.01 (-0.02, -0.01)	**5.14E-06**	0.00 (-0.01, 0.00)
0.03 (0.02, 0.04)	**1.05E-20**	0.03 (0.03, 0.04)	**4.66E-23**	0.02 (0.02, 0.03)
0.01 (0.00, 0.01)	**9.84E-03**	0.00 (-0.01, 0.00)	3.85E-01	0.01 (0.00, 0.01)
0.00 (-0.01, 0.00)	3.95E-01	-0.01 (-0.02, -0.01)	**1.94E-04**	0.00 (-0.01, 0.00)
0.00 (-0.01, 0.00)	2.43E-01	-0.01 (-0.02, -0.01)	**4.98E-06**	-0.01 (-0.01, 0.00)
0.00 (-0.01, 0.00)	1.39E-01	-0.01 (-0.02, -0.01)	**7.38E-06**	0.00 (-0.01, 0.00)
0.00 (-0.01, 0.00)	3.71E-01	-0.01 (-0.02, -0.01)	**1.24E-04**	0.00 (-0.01, 0.00)
-0.01 (-0.02, 0.00)	**1.85E-03**	-0.02 (-0.03, -0.01)	**9.81E-10**	-0.01 (-0.01, 0.00)
0.02 (0.02, 0.03)	**1.18E-15**	0.03 (0.02, 0.03)	**2.67E-18**	0.02 (0.01, 0.02)
0.01 (0.00, 0.02)	**8.98E-04**	0.00 (-0.01, 0.01)	9.13E-01	0.01 (0.00, 0.01)
0.00 (0.00, 0.01)	3.57E-01	-0.01 (-0.01, 0.00)	8.53E-02	0.00 (-0.01, 0.00)
0.00 (-0.01, 0.01)	8.82E-01	-0.01 (-0.01, 0.00)	**1.33E-02**	0.00 (-0.01, 0.00)
0.00 (0.00, 0.01)	5.56E-01	-0.01 (-0.01, 0.00)	**1.78E-02**	0.00 (-0.01, 0.00)
0.01 (0.00, 0.01)	9.53E-02	0.00 (-0.01, 0.00)	2.86E-01	0.00 (0.00, 0.01)
-0.01 (-0.01, 0.00)	**1.87E-02**	-0.02 (-0.02, -0.01)	**7.26E-09**	-0.01 (-0.01, 0.00)
0.03 (0.02, 0.03)	**6.25E-17**	0.03 (0.02, 0.03)	**7.79E-19**	0.02 (0.01, 0.02)
0.01 (0.01, 0.02)	**4.20E-05**	0.00 (0.00, 0.01)	3.85E-01	0.01 (0.00, 0.01)
0.00 (0.00, 0.01)	1.48E-01	0.00 (-0.01, 0.00)	3.58E-01	0.00 (0.00, 0.01)
0.00 (0.00, 0.01)	5.13E-01	0.00 (-0.01, 0.00)	1.66E-01	0.00 (0.00, 0.01)
0.00 (0.00, 0.01)	4.38E-01	-0.01 (-0.01, 0.00)	**4.01E-02**	0.00 (-0.01, 0.01)
0.01 (0.00, 0.01)	**4.72E-02**	0.00 (-0.01, 0.00)	6.29E-01	0.00 (0.00, 0.01)
-0.01 (-0.01, 0.00)	**1.67E-02**	-0.02 (-0.03, -0.01)	**1.38E-09**	-0.01 (-0.01, 0.00)
0.03 (0.02, 0.04)	**5.86E-21**	0.03 (0.02, 0.04)	**7.00E-21**	0.02 (0.02, 0.03)
-0.01 (-0.01, 0.00)	**3.06E-02**	0.00 (0.00, 0.01)	1.42E-01	0.01 (0.00, 0.02)
-0.01 (-0.01, 0.00)	**7.84E-03**	0.00 (0.00, 0.01)	3.66E-01	0.01 (0.00, 0.01)
-0.01 (-0.01, 0.00)	**1.38E-02**	0.00 (0.00, 0.01)	1.69E-01	0.01 (0.00, 0.02)
-0.01 (-0.02, -0.01)	**2.72E-04**	0.00 (-0.01, 0.00)	4.16E-01	0.01 (0.00, 0.01)
-0.01 (-0.02, -0.01)	**2.44E-05**	0.00 (-0.01, 0.00)	1.99E-01	0.00 (0.00, 0.01)
0.00 (-0.01, 0.00)	1.69E-01	0.00 (0.00, 0.01)	3.69E-01	0.01 (0.00, 0.02)
0.03 (0.02, 0.03)	**8.76E-21**	0.03 (0.03, 0.04)	**1.10E-27**	0.03 (0.02, 0.03)
-0.02 (-0.02, -0.01)	**1.91E-08**	-0.01 (-0.01, 0.00)	5.22E-02	0.00 (-0.01, 0.00)
-0.02 (-0.02, -0.01)	**5.86E-09**	-0.01 (-0.01, 0.00)	5.67E-02	0.00 (-0.01, 0.00)
-0.02 (-0.02, -0.01)	**5.21E-09**	-0.01 (-0.01, 0.00)	1.26E-01	0.00 (-0.01, 0.00)
-0.02 (-0.03, -0.01)	**1.56E-10**	-0.01 (-0.02, 0.00)	**1.61E-03**	0.00 (-0.01, 0.00)
-0.02 (-0.03, -0.01)	**2.08E-11**	-0.01 (-0.02, -0.01)	**3.06E-04**	-0.01 (-0.01, 0.00)
-0.02 (-0.02, -0.01)	**1.46E-07**	-0.01 (-0.01, 0.00)	1.29E-01	0.00 (-0.01, 0.01)
0.02 (0.01, 0.03)	**1.61E-10**	0.03 (0.02, 0.04)	**6.94E-21**	0.02 (0.02, 0.03)
-0.02 (-0.03, -0.02)	**9.68E-16**	-0.01 (-0.02, -0.01)	**1.38E-04**	-0.01 (-0.02, -0.01)
-0.02 (-0.03, -0.02)	**7.25E-14**	-0.01 (-0.02, 0.00)	**2.82E-03**	-0.01 (-0.02, -0.01)
-0.02 (-0.03, -0.01)	**7.09E-11**	-0.01 (-0.01, 0.00)	7.82E-02	-0.01 (-0.02, 0.00)
-0.03 (-0.04, -0.02)	**1.02E-21**	-0.02 (-0.02, -0.01)	**1.00E-08**	-0.02 (-0.02, -0.01)
-0.03 (-0.04, -0.02)	**1.86E-23**	-0.02 (-0.03, -0.01)	**4.09E-10**	-0.02 (-0.03, -0.01)
-0.02 (-0.03, -0.02)	**1.91E-14**	-0.01 (-0.02, 0.00)	**6.47E-04**	-0.01 (-0.02, -0.01)
0.02 (0.02, 0.03)	**6.67E-15**	0.03 (0.02, 0.03)	**1.66E-18**	0.02 (0.01, 0.02)

-0.02 (-0.03, -0.02)	**3.96E-12**	-0.02 (-0.03, -0.01)	**1.90E-10**	-0.02 (-0.03, -0.02)
-0.01 (-0.02, -0.01)	**2.06E-06**	-0.01 (-0.02, 0.00)	**2.36E-03**	-0.01 (-0.02, -0.01)
-0.01 (-0.02, -0.01)	**1.21E-05**	-0.01 (-0.01, 0.00)	**3.70E-02**	-0.01 (-0.02, -0.01)
-0.02 (-0.03, -0.02)	**3.87E-16**	-0.02 (-0.03, -0.02)	**3.58E-13**	-0.02 (-0.03, -0.02)
-0.03 (-0.03, -0.02)	**2.07E-19**	-0.03 (-0.03, -0.02)	**1.75E-18**	-0.03 (-0.03, -0.02)
-0.01 (-0.02, -0.01)	**2.61E-05**	-0.01 (-0.01, 0.00)	6.42E-02	-0.01 (-0.01, 0.00)
0.03 (0.02, 0.03)	**1.15E-19**	0.03 (0.02, 0.04)	**7.74E-20**	0.02 (0.01, 0.02)
0.02 (0.02, 0.03)	**8.29E-15**	0.01 (0.01, 0.02)	**1.25E-04**	0.01 (0.01, 0.02)
-0.01 (-0.01, 0.00)	**2.69E-02**	-0.02 (-0.03, -0.01)	**4.04E-10**	-0.01 (-0.02, 0.00)
-0.01 (-0.01, 0.00)	**1.95E-02**	-0.02 (-0.03, -0.01)	**1.28E-09**	-0.01 (-0.02, -0.01)
0.00 (-0.01, 0.00)	1.88E-01	-0.02 (-0.02, -0.01)	**4.69E-07**	-0.01 (-0.01, 0.00)
0.00 (-0.01, 0.00)	6.58E-01	0.02 (0.01, 0.02)	**2.52E-06**	0.00 (0.00, 0.01)
-0.01 (-0.02, -0.01)	**2.25E-06**	-0.03 (-0.03, -0.02)	**5.36E-16**	-0.01 (-0.01, 0.00)
-0.02 (-0.02, -0.01)	**4.82E-08**	-0.03 (-0.04, -0.02)	**9.60E-20**	-0.01 (-0.02, -0.01)
0.00 (-0.01, 0.00)	6.30E-01	-0.01 (-0.01, 0.00)	6.16E-02	0.01 (0.00, 0.01)
0.01 (0.00, 0.01)	**3.64E-02**	0.02 (0.01, 0.03)	**1.72E-10**	0.00 (0.00, 0.01)
0.03 (0.02, 0.04)	**5.42E-22**	0.02 (0.02, 0.03)	**6.83E-12**	0.02 (0.01, 0.02)
0.01 (0.00, 0.01)	**2.38E-02**	-0.01 (-0.02, -0.01)	**2.95E-04**	0.00 (0.00, 0.01)
0.00 (-0.01, 0.01)	9.56E-01	-0.02 (-0.02, -0.01)	**1.42E-06**	0.00 (0.00, 0.01)
0.02 (0.01, 0.03)	**6.30E-12**	0.00 (0.00, 0.01)	5.93E-01	0.01 (0.00, 0.01)
-0.02 (-0.03, -0.02)	**5.58E-13**	0.00 (-0.01, 0.00)	6.67E-01	-0.01 (-0.02, -0.01)
-0.01 (-0.01, 0.00)	**1.63E-02**	-0.02 (-0.03, -0.02)	**5.06E-13**	-0.01 (-0.01, 0.00)
-0.02 (-0.03, -0.01)	**3.48E-10**	-0.03 (-0.04, -0.03)	**5.54E-23**	-0.02 (-0.02, -0.01)
-0.02 (-0.03, -0.02)	**2.59E-12**	-0.03 (-0.04, -0.03)	**4.05E-25**	-0.02 (-0.02, -0.01)
-0.01 (-0.02, -0.01)	**1.29E-04**	-0.03 (-0.03, -0.02)	**3.34E-16**	-0.01 (-0.02, 0.00)
0.02 (0.01, 0.02)	**3.00E-08**	0.03 (0.02, 0.04)	**4.10E-21**	0.01 (0.01, 0.02)
-0.03 (-0.03, -0.02)	**1.05E-20**	-0.04 (-0.05, -0.03)	**4.44E-34**	-0.02 (-0.03, -0.02)
-0.02 (-0.02, -0.01)	**3.38E-07**	-0.03 (-0.04, -0.03)	**5.54E-25**	-0.02 (-0.02, -0.01)
0.00 (-0.01, 0.00)	3.02E-01	-0.03 (-0.03, -0.02)	**3.91E-16**	-0.01 (-0.02, -0.01)
-0.03 (-0.03, -0.02)	**4.73E-21**	-0.04 (-0.05, -0.03)	**4.44E-34**	-0.02 (-0.03, -0.02)
0.02 (0.01, 0.03)	**5.24E-11**	0.04 (0.03, 0.04)	**3.05E-28**	0.02 (0.01, 0.02)
0.03 (0.03, 0.04)	**8.66E-27**	0.04 (0.03, 0.04)	**3.84E-28**	0.03 (0.02, 0.03)
-0.03 (-0.03, -0.02)	**1.78E-20**	-0.04 (-0.05, -0.03)	**8.06E-34**	-0.02 (-0.03, -0.02)
-0.03 (-0.04, -0.02)	**1.86E-22**	-0.04 (-0.05, -0.03)	**6.21E-35**	-0.02 (-0.03, -0.02)
-0.01 (-0.02, 0.00)	**1.93E-03**	-0.03 (-0.03, -0.02)	**2.18E-15**	-0.01 (-0.02, -0.01)
0.02 (0.02, 0.03)	**2.89E-15**	0.04 (0.03, 0.04)	**1.23E-28**	0.02 (0.01, 0.03)
0.03 (0.03, 0.04)	**1.05E-27**	0.03 (0.02, 0.03)	**1.51E-17**	0.03 (0.02, 0.03)
-0.04 (-0.04, -0.03)	**3.98E-35**	-0.05 (-0.05, -0.04)	**1.04E-44**	-0.03 (-0.04, -0.03)
-0.04 (-0.05, -0.03)	**9.84E-38**	-0.04 (-0.05, -0.04)	**1.54E-42**	-0.03 (-0.04, -0.03)
-0.01 (-0.02, -0.01)	**1.78E-04**	-0.03 (-0.03, -0.02)	**4.67E-16**	-0.02 (-0.02, -0.01)
0.03 (0.03, 0.04)	**5.54E-27**	0.04 (0.04, 0.05)	**5.96E-42**	0.03 (0.02, 0.03)
0.00 (-0.01, 0.00)	1.95E-01	-0.01 (-0.02, -0.01)	**2.09E-05**	-0.01 (-0.02, -0.01)
-0.03 (-0.03, -0.02)	**9.42E-18**	-0.03 (-0.04, -0.03)	**9.81E-27**	-0.02 (-0.02, -0.01)
0.00 (-0.01, 0.00)	1.85E-01	-0.01 (-0.02, -0.01)	**3.80E-05**	0.00 (-0.01, 0.00)
-0.03 (-0.04, -0.02)	**3.64E-23**	-0.04 (-0.05, -0.03)	**2.28E-34**	-0.03 (-0.03, -0.02)
0.03 (0.02, 0.04)	**3.22E-21**	0.04 (0.04, 0.05)	**8.71E-38**	0.02 (0.02, 0.03)
-0.02 (-0.03, -0.01)	**3.72E-11**	-0.02 (-0.02, -0.01)	**3.26E-07**	-0.02 (-0.02, -0.01)
-0.01 (-0.02, -0.01)	**1.08E-05**	-0.03 (-0.04, -0.03)	**1.35E-22**	-0.01 (-0.02, -0.01)

0.02 (0.01, 0.02)	**8.77E-09**	0.01 (0.00, 0.02)	**9.48E-04**	0.01 (0.01, 0.02)
-0.03 (-0.04, -0.03)	**1.56E-25**	-0.04 (-0.04, -0.03)	**1.31E-33**	-0.03 (-0.03, -0.02)
0.03 (0.02, 0.03)	**3.16E-18**	0.04 (0.04, 0.05)	**5.83E-38**	0.02 (0.02, 0.03)
-0.01 (-0.02, 0.00)	**5.83E-04**	0.00 (-0.01, 0.00)	1.66E-01	0.00 (-0.01, 0.00)
-0.01 (-0.02, -0.01)	**1.17E-06**	-0.03 (-0.04, -0.03)	**1.80E-26**	-0.02 (-0.02, -0.01)
0.01 (0.01, 0.02)	**3.04E-04**	0.01 (0.00, 0.01)	6.50E-02	0.00 (0.00, 0.01)
-0.03 (-0.04, -0.02)	**3.74E-21**	-0.04 (-0.04, -0.03)	**1.45E-33**	-0.02 (-0.03, -0.02)
0.03 (0.02, 0.04)	**3.14E-22**	0.04 (0.03, 0.05)	**1.85E-35**	0.02 (0.02, 0.03)
0.00 (0.00, 0.01)	4.21E-01	0.01 (0.00, 0.02)	**1.72E-03**	0.02 (0.01, 0.02)
-0.02 (-0.02, -0.01)	**3.77E-08**	-0.03 (-0.04, -0.02)	**7.08E-20**	-0.03 (-0.03, -0.02)
-0.03 (-0.03, -0.02)	**9.01E-22**	-0.04 (-0.04, -0.03)	**4.56E-31**	-0.03 (-0.03, -0.02)
0.01 (0.00, 0.01)	**3.08E-02**	0.00 (-0.01, 0.00)	2.66E-01	-0.01 (-0.02, -0.01)
0.02 (0.01, 0.02)	**1.24E-08**	0.02 (0.01, 0.03)	**1.97E-10**	0.01 (0.00, 0.02)
0.01 (0.00, 0.01)	**8.17E-03**	0.01 (0.01, 0.02)	**7.09E-06**	0.00 (-0.01, 0.00)
-0.02 (-0.03, -0.01)	**2.77E-11**	-0.03 (-0.03, -0.02)	**6.38E-15**	-0.01 (-0.01, 0.00)
-0.02 (-0.03, -0.02)	**2.35E-14**	-0.03 (-0.03, -0.02)	**4.39E-19**	-0.01 (-0.02, -0.01)
0.00 (-0.01, 0.01)	8.76E-01	0.00 (-0.01, 0.01)	8.50E-01	0.01 (0.01, 0.02)
0.03 (0.02, 0.03)	**2.54E-18**	0.03 (0.02, 0.03)	**4.99E-18**	0.02 (0.01, 0.02)
0.03 (0.03, 0.04)	**1.73E-26**	0.03 (0.03, 0.04)	**5.40E-26**	0.02 (0.02, 0.03)
-0.04 (-0.04, -0.03)	**3.52E-33**	-0.04 (-0.04, -0.03)	**1.73E-32**	-0.03 (-0.03, -0.02)
-0.04 (-0.04, -0.03)	**3.60E-33**	-0.04 (-0.05, -0.03)	**3.69E-34**	-0.03 (-0.03, -0.02)
-0.02 (-0.03, -0.02)	**5.45E-12**	-0.01 (-0.02, -0.01)	**1.26E-04**	-0.01 (-0.01, 0.00)
0.04 (0.03, 0.04)	**3.52E-33**	0.04 (0.03, 0.04)	**5.19E-32**	0.03 (0.02, 0.03)
0.00 (0.00, 0.01)	1.60E-01	0.02 (0.01, 0.02)	**1.80E-07**	0.01 (0.01, 0.02)
-0.03 (-0.04, -0.02)	**5.42E-23**	-0.04 (-0.04, -0.03)	**8.27E-31**	-0.03 (-0.03, -0.02)
-0.03 (-0.04, -0.03)	**9.51E-24**	-0.04 (-0.05, -0.03)	**4.52E-35**	-0.03 (-0.03, -0.02)
0.00 (0.00, 0.01)	4.85E-01	0.01 (0.00, 0.02)	**7.42E-04**	0.01 (0.01, 0.02)
0.04 (0.03, 0.05)	**1.18E-37**	0.04 (0.03, 0.05)	**6.98E-36**	0.03 (0.02, 0.04)
0.02 (0.02, 0.03)	**9.47E-12**	0.02 (0.02, 0.03)	**1.54E-11**	0.01 (0.01, 0.02)
-0.01 (-0.02, -0.01)	**3.29E-04**	-0.01 (-0.02, -0.01)	**1.28E-05**	0.00 (-0.01, 0.00)
-0.01 (-0.02, 0.00)	**8.98E-04**	0.00 (-0.01, 0.01)	7.82E-01	0.01 (0.00, 0.01)
-0.01 (-0.01, 0.00)	**4.39E-02**	-0.01 (-0.02, -0.01)	**5.48E-04**	0.00 (-0.01, 0.00)
0.02 (0.02, 0.03)	**7.39E-13**	0.01 (0.01, 0.02)	**4.47E-05**	0.02 (0.01, 0.02)
0.00 (-0.01, 0.00)	4.83E-01	-0.01 (-0.02, -0.01)	**1.95E-04**	0.00 (-0.01, 0.00)
0.01 (0.00, 0.02)	**1.87E-03**	0.00 (-0.01, 0.01)	9.26E-01	0.01 (0.00, 0.01)
-0.01 (-0.02, 0.00)	**2.98E-03**	-0.01 (-0.02, -0.01)	**2.87E-05**	0.00 (-0.01, 0.00)
0.00 (0.00, 0.01)	7.81E-01	-0.01 (-0.01, 0.00)	1.11E-01	0.00 (0.00, 0.01)
0.03 (0.02, 0.03)	**3.41E-19**	0.03 (0.02, 0.03)	**5.37E-18**	0.02 (0.02, 0.03)
0.03 (0.02, 0.03)	**8.04E-18**	0.03 (0.02, 0.03)	**3.96E-16**	0.02 (0.01, 0.03)
0.03 (0.02, 0.03)	**4.43E-17**	0.03 (0.02, 0.03)	**3.48E-19**	0.02 (0.01, 0.03)
0.03 (0.02, 0.03)	**4.85E-18**	0.03 (0.02, 0.04)	**6.10E-22**	0.02 (0.02, 0.03)
0.00 (-0.01, 0.00)	3.77E-01	0.00 (0.00, 0.01)	3.24E-01	0.00 (0.00, 0.01)
0.03 (0.03, 0.04)	**5.23E-25**	0.03 (0.03, 0.04)	**7.14E-25**	0.02 (0.02, 0.03)
0.00 (-0.01, 0.00)	1.34E-01	0.00 (-0.01, 0.01)	8.98E-01	0.00 (0.00, 0.01)
-0.01 (-0.02, 0.00)	**1.47E-03**	-0.01 (-0.01, 0.00)	**8.11E-03**	0.00 (-0.01, 0.00)
-0.01 (-0.01, 0.00)	5.25E-02	0.00 (-0.01, 0.00)	7.16E-01	0.00 (0.00, 0.01)
-0.02 (-0.03, -0.02)	**1.78E-14**	-0.01 (-0.02, -0.01)	**3.76E-04**	-0.01 (-0.02, -0.01)
0.01 (0.00, 0.02)	**1.13E-03**	0.00 (-0.01, 0.01)	8.68E-01	0.01 (0.00, 0.01)

0.02 (0.01, 0.03)	**2.90E-11**	0.01 (0.00, 0.01)	**4.77E-02**	0.01 (0.01, 0.02)
0.02 (0.01, 0.02)	**6.29E-08**	0.02 (0.01, 0.02)	**2.66E-09**	0.01 (0.01, 0.02)
0.00 (-0.01, 0.01)	9.78E-01	-0.01 (-0.01, 0.00)	5.76E-02	0.01 (0.01, 0.02)
0.02 (0.01, 0.02)	**1.12E-07**	0.02 (0.02, 0.03)	**1.04E-13**	0.03 (0.03, 0.04)
0.00 (0.00, 0.01)	1.24E-01	0.00 (-0.01, 0.01)	8.11E-01	0.01 (0.00, 0.01)
0.03 (0.02, 0.03)	**1.66E-17**	0.03 (0.03, 0.04)	**1.86E-27**	0.04 (0.03, 0.04)
0.00 (0.00, 0.01)	1.54E-01	0.00 (-0.01, 0.01)	9.36E-01	0.01 (0.00, 0.01)
0.01 (0.01, 0.02)	**3.25E-04**	0.01 (0.00, 0.02)	**3.48E-03**	0.02 (0.01, 0.02)
0.02 (0.02, 0.03)	**4.47E-12**	0.02 (0.02, 0.03)	**2.72E-14**	0.01 (0.01, 0.02)
0.01 (0.01, 0.02)	**2.11E-06**	0.02 (0.01, 0.02)	**5.75E-09**	0.01 (0.01, 0.02)
0.01 (0.00, 0.01)	**8.22E-03**	0.01 (0.01, 0.02)	**1.01E-04**	0.02 (0.02, 0.03)
-0.02 (-0.03, -0.01)	**1.12E-10**	-0.04 (-0.04, -0.03)	**9.45E-27**	-0.01 (-0.02, -0.01)
0.02 (0.02, 0.03)	**8.80E-13**	0.03 (0.02, 0.04)	**5.91E-21**	0.04 (0.03, 0.04)
-0.03 (-0.03, -0.02)	**1.20E-18**	-0.04 (-0.05, -0.03)	**4.08E-33**	-0.02 (-0.03, -0.01)
-0.02 (-0.02, -0.01)	**2.92E-07**	-0.03 (-0.03, -0.02)	**3.69E-16**	-0.01 (-0.01, 0.00)
0.02 (0.02, 0.03)	**3.33E-15**	0.03 (0.02, 0.04)	**1.10E-21**	0.01 (0.01, 0.02)
0.00 (-0.01, 0.00)	3.09E-01	0.01 (0.00, 0.02)	**2.42E-03**	-0.01 (-0.01, 0.00)
0.02 (0.01, 0.02)	**3.84E-08**	0.02 (0.01, 0.02)	**2.03E-07**	0.01 (0.01, 0.02)
0.00 (-0.01, 0.01)	8.47E-01	0.01 (0.00, 0.01)	**4.25E-02**	0.00 (0.00, 0.01)
-0.01 (-0.01, 0.00)	**3.46E-02**	0.01 (0.00, 0.01)	**2.65E-02**	-0.01 (-0.01, 0.00)
0.00 (0.00, 0.01)	1.85E-01	0.02 (0.01, 0.03)	**5.97E-11**	0.01 (0.00, 0.01)
-0.03 (-0.03, -0.02)	**2.13E-18**	-0.01 (-0.02, -0.01)	**2.80E-06**	-0.02 (-0.03, -0.02)
0.00 (-0.01, 0.01)	8.47E-01	0.00 (-0.01, 0.01)	9.60E-01	0.00 (-0.01, 0.01)
0.01 (0.01, 0.02)	**1.36E-04**	0.01 (0.00, 0.01)	**4.54E-02**	0.00 (0.00, 0.01)
0.01 (0.01, 0.02)	**3.04E-04**	0.00 (0.00, 0.01)	1.28E-01	0.00 (0.00, 0.01)
0.02 (0.01, 0.03)	**6.08E-12**	0.02 (0.01, 0.03)	**1.92E-10**	0.01 (0.01, 0.02)
0.01 (0.00, 0.02)	**1.62E-03**	0.00 (-0.01, 0.00)	3.10E-01	0.01 (0.00, 0.01)
0.01 (0.00, 0.01)	6.76E-02	0.00 (0.00, 0.01)	4.64E-01	0.00 (-0.01, 0.00)
0.00 (-0.01, 0.00)	6.53E-01	-0.01 (-0.02, 0.00)	**4.22E-03**	-0.01 (-0.01, 0.00)
0.01 (0.00, 0.01)	5.65E-02	0.00 (0.00, 0.01)	6.52E-01	0.01 (0.00, 0.01)
0.01 (0.00, 0.01)	8.09E-02	0.00 (0.00, 0.01)	4.53E-01	0.01 (0.00, 0.01)
0.02 (0.02, 0.03)	**5.43E-14**	0.03 (0.03, 0.04)	**7.52E-25**	0.02 (0.01, 0.02)
-0.01 (-0.01, 0.00)	6.29E-02	0.01 (0.00, 0.01)	**2.57E-02**	0.00 (0.00, 0.01)
0.02 (0.02, 0.03)	**4.65E-15**	0.03 (0.02, 0.04)	**4.56E-20**	0.01 (0.01, 0.02)
-0.02 (-0.03, -0.01)	**1.62E-11**	-0.01 (-0.01, 0.00)	**4.94E-02**	-0.01 (-0.02, -0.01)
0.02 (0.01, 0.02)	**4.89E-08**	0.01 (0.01, 0.02)	**4.88E-05**	0.01 (0.00, 0.01)
0.00 (0.00, 0.01)	2.58E-01	-0.01 (-0.01, 0.00)	**4.72E-02**	0.00 (0.00, 0.01)
0.00 (-0.01, 0.00)	2.10E-01	-0.02 (-0.02, -0.01)	**1.50E-06**	-0.01 (-0.01, 0.00)
0.00 (-0.01, 0.01)	9.54E-01	-0.01 (-0.02, 0.00)	**4.98E-03**	0.00 (-0.01, 0.00)
-0.03 (-0.04, -0.02)	**9.01E-22**	-0.02 (-0.03, -0.01)	**1.06E-10**	-0.02 (-0.02, -0.01)
-0.02 (-0.03, -0.02)	**1.30E-12**	-0.03 (-0.04, -0.02)	**1.13E-19**	-0.01 (-0.02, 0.00)
-0.03 (-0.04, -0.03)	**7.46E-23**	-0.03 (-0.04, -0.03)	**3.10E-23**	-0.03 (-0.04, -0.03)
0.02 (0.02, 0.03)	**2.39E-15**	0.02 (0.01, 0.02)	**1.85E-08**	0.02 (0.01, 0.02)
-0.01 (-0.01, 0.00)	**4.12E-03**	-0.02 (-0.03, -0.01)	**1.27E-09**	-0.01 (-0.01, 0.00)
-0.02 (-0.03, -0.01)	**1.08E-10**	-0.01 (-0.01, 0.00)	**1.58E-02**	-0.01 (-0.01, 0.00)
0.00 (0.00, 0.01)	7.62E-01	0.00 (0.00, 0.01)	1.99E-01	0.00 (0.00, 0.01)
0.00 (-0.01, 0.00)	4.78E-01	-0.01 (-0.01, 0.00)	**2.30E-02**	0.00 (0.00, 0.01)
-0.02 (-0.03, -0.02)	**9.08E-16**	-0.02 (-0.02, -0.01)	**1.04E-07**	-0.02 (-0.02, -0.01)

0.03 (0.02, 0.03)	**1.54E-16**	0.02 (0.01, 0.03)	**6.15E-10**	0.02 (0.01, 0.03)
0.01 (0.00, 0.02)	**2.32E-03**	0.00 (-0.01, 0.00)	6.60E-01	0.01 (0.00, 0.01)
-0.03 (-0.03, -0.02)	**1.08E-18**	-0.02 (-0.02, -0.01)	**2.50E-08**	-0.02 (-0.02, -0.01)
0.00 (-0.01, 0.00)	2.68E-01	0.00 (-0.01, 0.01)	8.88E-01	0.00 (0.00, 0.01)
0.03 (0.02, 0.03)	**1.07E-17**	0.02 (0.02, 0.03)	**2.02E-11**	0.02 (0.01, 0.03)
0.00 (-0.01, 0.00)	5.94E-01	-0.01 (-0.02, -0.01)	**3.29E-04**	0.00 (-0.01, 0.00)
-0.02 (-0.03, -0.01)	**3.55E-11**	-0.01 (-0.01, 0.00)	7.05E-02	-0.01 (-0.01, 0.00)
0.00 (0.00, 0.01)	1.49E-01	0.00 (0.00, 0.01)	2.09E-01	0.01 (0.00, 0.01)
0.03 (0.02, 0.03)	**6.68E-18**	0.02 (0.02, 0.03)	**4.73E-13**	0.02 (0.01, 0.03)
0.00 (-0.01, 0.01)	9.05E-01	-0.01 (-0.02, 0.00)	**3.69E-03**	0.00 (-0.01, 0.00)
-0.02 (-0.03, -0.02)	**5.05E-13**	-0.01 (-0.02, 0.00)	**4.80E-03**	-0.01 (-0.02, 0.00)
0.02 (0.01, 0.03)	**8.88E-11**	0.01 (0.00, 0.02)	**5.36E-03**	0.01 (0.01, 0.02)
-0.03 (-0.03, -0.02)	**2.90E-19**	-0.02 (-0.02, -0.01)	**1.62E-08**	-0.01 (-0.02, -0.01)
0.02 (0.02, 0.03)	**1.89E-12**	0.01 (0.01, 0.02)	**5.52E-06**	0.02 (0.01, 0.02)

self-rated health status. P values are corrected for multiple testing using a false discovery rate (FDR) adjustment.

Density Lipoprotein; IDL, Intermediate Density Lipoprotein..

Age >= 60y			White	
**P value**	**Adjusted β (95% CI)**	**P value**	**Adjusted β (95% CI)**	**P value**
**2.88E-10**	0.04 (0.03, 0.04)	**9.91E-28**	0.02 (0.01, 0.02)	**3.65E-13**
**2.58E-09**	0.04 (0.03, 0.04)	**4.95E-26**	0.02 (0.01, 0.02)	**5.91E-12**
**1.78E-10**	0.04 (0.03, 0.04)	**2.20E-28**	0.02 (0.01, 0.02)	**1.30E-13**
**9.52E-11**	0.04 (0.03, 0.04)	**5.54E-26**	0.02 (0.01, 0.02)	**4.75E-11**
**3.07E-10**	0.03 (0.03, 0.04)	**7.44E-24**	0.01 (0.01, 0.02)	**2.35E-09**
**4.20E-11**	0.04 (0.03, 0.04)	**7.24E-28**	0.02 (0.01, 0.02)	**5.20E-13**
**2.14E-08**	0.04 (0.03, 0.04)	**8.46E-25**	0.02 (0.01, 0.02)	**1.43E-11**
**3.30E-10**	0.03 (0.03, 0.04)	**6.48E-24**	0.01 (0.01, 0.02)	**7.83E-09**
**5.79E-10**	0.03 (0.03, 0.04)	**4.27E-23**	0.01 (0.01, 0.02)	**1.71E-08**
**9.45E-11**	0.03 (0.03, 0.04)	**6.59E-24**	0.01 (0.01, 0.02)	**1.03E-08**
**5.40E-10**	0.03 (0.02, 0.04)	**2.30E-18**	0.01 (0.00, 0.01)	**1.11E-04**
**4.11E-08**	0.02 (0.02, 0.03)	**2.39E-13**	0.00 (0.00, 0.01)	**3.31E-02**
**2.11E-11**	0.03 (0.03, 0.04)	**4.00E-23**	0.01 (0.01, 0.02)	**3.39E-08**
**3.13E-09**	0.03 (0.03, 0.04)	**2.33E-23**	0.01 (0.01, 0.02)	**1.20E-09**
**6.32E-11**	0.03 (0.03, 0.04)	**1.11E-20**	0.01 (0.01, 0.02)	**3.33E-07**
**3.30E-10**	0.03 (0.02, 0.04)	**3.67E-18**	0.01 (0.01, 0.01)	**6.35E-06**
**8.35E-11**	0.03 (0.03, 0.04)	**5.57E-22**	0.01 (0.01, 0.02)	**1.09E-07**
**4.20E-11**	0.03 (0.02, 0.04)	**9.25E-18**	0.01 (0.00, 0.01)	**6.38E-05**
**1.68E-11**	0.03 (0.02, 0.03)	**1.51E-14**	0.01 (0.00, 0.01)	**9.41E-04**
**2.88E-10**	0.03 (0.02, 0.04)	**3.05E-20**	0.01 (0.01, 0.01)	**5.11E-06**
**6.07E-09**	0.03 (0.02, 0.03)	**8.29E-16**	0.01 (0.01, 0.01)	**1.75E-05**
**1.11E-06**	0.02 (0.01, 0.02)	**8.89E-06**	0.00 (-0.01, 0.00)	4.84E-01
**4.61E-08**	0.02 (0.01, 0.03)	**4.54E-08**	0.00 (0.00, 0.01)	6.32E-01
**1.82E-06**	0.01 (0.01, 0.02)	**2.27E-05**	0.00 (-0.01, 0.00)	3.78E-01
**9.18E-03**	0.00 (-0.01, 0.01)	7.76E-01	-0.01 (-0.01, -0.01)	**7.67E-06**
2.62E-01	-0.01 (-0.01, 0.00)	6.23E-02	-0.01 (-0.02, -0.01)	**3.37E-10**
**1.76E-05**	0.01 (0.00, 0.02)	**1.91E-03**	0.00 (-0.01, 0.00)	6.29E-02
**3.30E-10**	0.03 (0.02, 0.03)	**4.64E-15**	0.01 (0.00, 0.01)	**1.46E-04**
**2.70E-14**	0.03 (0.02, 0.04)	**1.63E-18**	0.01 (0.01, 0.02)	**1.51E-06**
**9.51E-13**	0.03 (0.02, 0.03)	**1.01E-16**	0.01 (0.00, 0.01)	**7.75E-05**
**6.32E-08**	0.02 (0.01, 0.03)	**6.77E-08**	0.00 (0.00, 0.01)	6.67E-01
**1.92E-06**	0.02 (0.01, 0.02)	**1.11E-06**	0.00 (0.00, 0.00)	9.35E-01
**1.46E-07**	0.02 (0.01, 0.03)	**2.20E-09**	0.00 (0.00, 0.01)	2.69E-01
**1.48E-04**	0.01 (0.00, 0.02)	**4.45E-03**	0.00 (-0.01, 0.00)	**2.79E-02**
**4.41E-18**	0.04 (0.03, 0.04)	**2.57E-28**	0.02 (0.01, 0.02)	**3.52E-17**
**5.43E-11**	0.02 (0.02, 0.03)	**6.96E-10**	0.01 (0.00, 0.01)	**5.81E-03**
**7.25E-13**	0.02 (0.02, 0.03)	**3.31E-11**	0.01 (0.00, 0.01)	**3.70E-04**
**1.94E-15**	0.03 (0.02, 0.03)	**4.31E-15**	0.01 (0.01, 0.02)	**4.11E-07**
**3.94E-06**	0.01 (0.00, 0.02)	**2.17E-02**	0.00 (-0.01, 0.00)	4.90E-01
**1.99E-04**	0.00 (0.00, 0.01)	3.43E-01	0.00 (-0.01, 0.00)	**4.77E-02**
**1.14E-10**	0.02 (0.01, 0.03)	**6.76E-08**	0.01 (0.00, 0.01)	**2.56E-02**
**5.63E-20**	0.04 (0.04, 0.05)	**2.09E-35**	0.02 (0.02, 0.03)	**1.14E-23**
9.31E-01	0.00 (-0.01, 0.00)	3.44E-01	-0.02 (-0.02, -0.01)	**1.43E-11**

9.03E-01	-0.01 (-0.02, 0.00)	**1.87E-02**	-0.02 (-0.02, -0.01)	**5.86E-14**
1.38E-01	0.00 (-0.01, 0.00)	2.51E-01	-0.01 (-0.02, -0.01)	**2.41E-08**
9.59E-02	-0.02 (-0.02, -0.01)	**2.38E-05**	-0.02 (-0.03, -0.02)	**2.20E-21**
6.65E-02	-0.02 (-0.02, -0.01)	**1.03E-05**	-0.02 (-0.03, -0.02)	**1.96E-22**
2.62E-01	-0.01 (-0.02, -0.01)	**3.39E-04**	-0.02 (-0.02, -0.02)	**1.22E-17**
**1.70E-15**	0.04 (0.03, 0.05)	**4.52E-30**	0.02 (0.01, 0.02)	**1.37E-16**
**3.10E-02**	0.00 (-0.01, 0.01)	7.76E-01	-0.01 (-0.01, -0.01)	**2.48E-06**
2.84E-01	-0.01 (-0.02, 0.00)	**1.04E-02**	-0.02 (-0.02, -0.02)	**7.20E-19**
8.13E-02	-0.01 (-0.02, 0.00)	**3.41E-03**	-0.02 (-0.03, -0.02)	**1.64E-21**
1.31E-01	-0.01 (-0.02, -0.01)	**4.46E-04**	-0.02 (-0.03, -0.02)	**6.06E-22**
3.07E-01	-0.01 (-0.02, 0.00)	**4.61E-03**	-0.02 (-0.02, -0.02)	**2.80E-19**
**3.80E-03**	-0.02 (-0.03, -0.01)	**1.50E-07**	-0.03 (-0.03, -0.02)	**7.44E-29**
**1.54E-10**	0.04 (0.03, 0.04)	**5.21E-26**	0.01 (0.01, 0.02)	**7.23E-10**
5.05E-02	0.01 (0.00, 0.01)	1.02E-01	-0.01 (-0.01, -0.01)	**1.31E-05**
8.63E-01	0.00 (-0.01, 0.01)	9.56E-01	-0.02 (-0.02, -0.01)	**1.30E-12**
3.94E-01	0.00 (-0.01, 0.00)	5.34E-01	-0.02 (-0.02, -0.01)	**4.35E-15**
6.29E-01	0.00 (-0.01, 0.00)	5.59E-01	-0.02 (-0.02, -0.01)	**1.11E-14**
7.10E-01	0.00 (0.00, 0.01)	4.80E-01	-0.01 (-0.02, -0.01)	**1.54E-10**
**2.80E-03**	-0.01 (-0.02, -0.01)	**2.37E-05**	-0.02 (-0.03, -0.02)	**2.89E-27**
**7.87E-11**	0.04 (0.03, 0.04)	**2.40E-27**	0.01 (0.01, 0.02)	**5.10E-10**
**2.80E-03**	0.01 (0.00, 0.02)	**1.13E-02**	-0.01 (-0.01, 0.00)	**2.05E-03**
4.04E-01	0.00 (-0.01, 0.01)	6.62E-01	-0.01 (-0.02, -0.01)	**1.20E-09**
5.28E-01	0.00 (-0.01, 0.01)	7.09E-01	-0.01 (-0.02, -0.01)	**5.76E-10**
9.75E-01	0.00 (-0.01, 0.00)	5.80E-01	-0.02 (-0.02, -0.01)	**3.27E-13**
2.96E-01	0.00 (0.00, 0.01)	3.33E-01	-0.01 (-0.02, -0.01)	**4.64E-09**
**6.70E-03**	-0.02 (-0.02, -0.01)	**3.59E-06**	-0.02 (-0.03, -0.02)	**7.45E-25**
**9.74E-14**	0.04 (0.03, 0.05)	**1.27E-29**	0.02 (0.01, 0.02)	**2.58E-13**
**2.01E-03**	-0.01 (-0.02, -0.01)	**9.66E-05**	0.00 (-0.01, 0.00)	5.82E-01
**6.92E-03**	-0.02 (-0.02, -0.01)	**6.09E-06**	0.00 (-0.01, 0.00)	7.06E-01
**3.20E-03**	-0.02 (-0.02, -0.01)	**2.13E-05**	0.00 (0.00, 0.01)	7.06E-01
1.31E-01	-0.02 (-0.03, -0.01)	**8.92E-09**	-0.01 (-0.01, 0.00)	**2.82E-02**
2.67E-01	-0.02 (-0.03, -0.02)	**1.79E-10**	-0.01 (-0.01, 0.00)	**3.66E-03**
**3.10E-03**	-0.01 (-0.02, 0.00)	**1.42E-03**	0.00 (0.00, 0.01)	6.67E-01
**2.06E-22**	0.04 (0.03, 0.04)	**3.40E-25**	0.02 (0.02, 0.03)	**1.80E-22**
7.22E-01	-0.03 (-0.03, -0.02)	**1.27E-12**	-0.01 (-0.02, -0.01)	**2.27E-05**
5.52E-01	-0.03 (-0.03, -0.02)	**2.17E-12**	-0.01 (-0.01, -0.01)	**4.60E-05**
5.09E-01	-0.03 (-0.03, -0.02)	**2.81E-11**	-0.01 (-0.01, 0.00)	**1.46E-04**
2.54E-01	-0.03 (-0.04, -0.02)	**8.39E-16**	-0.01 (-0.02, -0.01)	**3.94E-07**
1.39E-01	-0.03 (-0.04, -0.02)	**3.04E-17**	-0.01 (-0.02, -0.01)	**4.14E-08**
9.75E-01	-0.02 (-0.03, -0.02)	**4.64E-11**	-0.01 (-0.01, 0.00)	**3.42E-04**
**2.81E-14**	0.03 (0.02, 0.03)	**5.13E-14**	0.02 (0.01, 0.02)	**5.73E-17**
**9.14E-06**	-0.03 (-0.03, -0.02)	**1.53E-12**	-0.02 (-0.03, -0.02)	**5.98E-19**
**4.73E-05**	-0.02 (-0.03, -0.02)	**1.25E-09**	-0.02 (-0.02, -0.01)	**8.15E-16**
**9.86E-04**	-0.02 (-0.02, -0.01)	**3.89E-06**	-0.02 (-0.02, -0.01)	**1.98E-11**
**3.73E-09**	-0.03 (-0.04, -0.03)	**1.68E-19**	-0.03 (-0.03, -0.02)	**2.32E-26**
**1.66E-10**	-0.03 (-0.04, -0.03)	**2.46E-21**	-0.03 (-0.03, -0.02)	**1.35E-28**
**2.08E-04**	-0.03 (-0.03, -0.02)	**3.32E-12**	-0.02 (-0.03, -0.02)	**3.28E-17**
**3.30E-10**	0.03 (0.03, 0.04)	**1.45E-23**	0.02 (0.01, 0.02)	**2.67E-14**

**3.09E-13**	-0.02 (-0.03, -0.01)	**1.07E-07**	-0.03 (-0.03, -0.02)	**6.35E-40**
**8.18E-07**	-0.01 (-0.01, 0.00)	**2.01E-02**	-0.02 (-0.03, -0.02)	**7.26E-21**
**2.75E-05**	-0.01 (-0.01, 0.00)	8.01E-02	-0.02 (-0.02, -0.01)	**5.53E-16**
**8.01E-16**	-0.02 (-0.03, -0.02)	**3.55E-11**	-0.03 (-0.04, -0.03)	**4.78E-45**
**9.46E-21**	-0.03 (-0.03, -0.02)	**1.09E-14**	-0.03 (-0.04, -0.03)	**2.02E-52**
**2.24E-03**	-0.01 (-0.02, 0.00)	**1.89E-02**	-0.02 (-0.02, -0.02)	**7.73E-18**
**3.36E-10**	0.04 (0.03, 0.05)	**5.39E-31**	0.02 (0.01, 0.02)	**7.42E-15**
**5.69E-07**	0.02 (0.01, 0.03)	**6.07E-10**	0.02 (0.01, 0.02)	**1.43E-11**
**4.38E-04**	-0.02 (-0.02, -0.01)	**1.33E-05**	-0.01 (-0.01, 0.00)	**3.58E-04**
**1.09E-04**	-0.01 (-0.02, -0.01)	**8.13E-05**	-0.01 (-0.01, 0.00)	**8.00E-05**
8.69E-02	-0.01 (-0.02, -0.01)	**5.70E-05**	0.00 (-0.01, 0.00)	7.55E-02
1.00E-01	0.01 (0.00, 0.01)	**3.98E-02**	0.00 (0.00, 0.01)	2.42E-01
**2.75E-03**	-0.03 (-0.04, -0.02)	**5.01E-17**	-0.01 (-0.02, -0.01)	**1.14E-07**
**6.01E-05**	-0.03 (-0.04, -0.03)	**1.41E-19**	-0.01 (-0.02, -0.01)	**2.03E-09**
6.61E-02	-0.01 (-0.02, -0.01)	**1.46E-04**	0.00 (-0.01, 0.00)	2.93E-01
2.94E-01	0.02 (0.01, 0.03)	**1.09E-09**	0.01 (0.00, 0.01)	**7.83E-05**
**6.66E-09**	0.04 (0.03, 0.04)	**6.68E-27**	0.02 (0.01, 0.02)	**1.55E-13**
2.43E-01	-0.01 (-0.01, 0.00)	7.76E-02	0.00 (-0.01, 0.00)	4.95E-01
6.60E-01	-0.02 (-0.02, -0.01)	**5.40E-06**	-0.01 (-0.01, 0.00)	**2.47E-02**
**1.01E-02**	0.02 (0.01, 0.03)	**2.25E-08**	0.01 (0.00, 0.01)	**1.33E-03**
**4.63E-05**	-0.02 (-0.02, -0.01)	**5.04E-06**	-0.01 (-0.01, 0.00)	**3.58E-04**
**7.58E-03**	-0.02 (-0.03, -0.01)	**2.65E-09**	-0.02 (-0.02, -0.01)	**1.15E-16**
**2.81E-07**	-0.04 (-0.04, -0.03)	**3.23E-23**	-0.02 (-0.03, -0.02)	**6.34E-25**
**1.61E-08**	-0.04 (-0.05, -0.03)	**3.28E-26**	-0.03 (-0.03, -0.02)	**2.00E-26**
**6.61E-04**	-0.03 (-0.03, -0.02)	**1.64E-14**	-0.02 (-0.03, -0.02)	**3.55E-19**
**2.67E-06**	0.03 (0.03, 0.04)	**3.46E-20**	0.02 (0.02, 0.03)	**1.04E-23**
**1.94E-15**	-0.04 (-0.05, -0.04)	**5.32E-35**	-0.03 (-0.04, -0.03)	**5.83E-49**
**3.14E-09**	-0.03 (-0.04, -0.02)	**1.46E-17**	-0.02 (-0.03, -0.02)	**5.03E-28**
**1.99E-04**	-0.02 (-0.02, -0.01)	**2.80E-06**	-0.02 (-0.02, -0.01)	**2.60E-12**
**2.14E-15**	-0.04 (-0.05, -0.04)	**3.13E-35**	-0.03 (-0.04, -0.03)	**1.43E-48**
**3.12E-11**	0.03 (0.03, 0.04)	**6.61E-23**	0.03 (0.02, 0.03)	**5.61E-35**
**2.99E-19**	0.04 (0.04, 0.05)	**1.58E-33**	0.03 (0.03, 0.04)	**2.24E-47**
**2.91E-15**	-0.05 (-0.05, -0.04)	**1.46E-36**	-0.03 (-0.04, -0.03)	**9.22E-46**
**4.21E-16**	-0.05 (-0.05, -0.04)	**1.41E-38**	-0.03 (-0.04, -0.03)	**3.38E-47**
**3.64E-05**	-0.02 (-0.03, -0.02)	**6.00E-13**	-0.02 (-0.02, -0.02)	**9.22E-20**
**1.95E-11**	0.04 (0.03, 0.05)	**1.39E-30**	0.03 (0.03, 0.03)	**1.48E-36**
**5.43E-23**	0.03 (0.02, 0.04)	**9.62E-18**	0.03 (0.03, 0.04)	**2.67E-45**
**5.12E-28**	-0.05 (-0.06, -0.05)	**7.26E-48**	-0.04 (-0.05, -0.04)	**1.67E-75**
**3.55E-27**	-0.05 (-0.06, -0.04)	**1.10E-47**	-0.04 (-0.05, -0.04)	**1.67E-75**
**8.45E-08**	-0.02 (-0.03, -0.02)	**4.97E-13**	-0.02 (-0.02, -0.01)	**3.52E-17**
**9.46E-21**	0.05 (0.05, 0.06)	**2.13E-47**	0.04 (0.03, 0.04)	**4.48E-63**
**2.32E-05**	0.00 (-0.01, 0.00)	2.86E-01	0.00 (-0.01, 0.00)	9.00E-02
**5.59E-09**	-0.05 (-0.05, -0.04)	**2.61E-40**	-0.03 (-0.04, -0.03)	**1.43E-48**
8.57E-01	-0.02 (-0.03, -0.01)	**1.31E-08**	-0.02 (-0.02, -0.01)	**1.93E-12**
**1.68E-17**	-0.05 (-0.05, -0.04)	**6.25E-39**	-0.03 (-0.04, -0.03)	**1.90E-43**
**1.32E-15**	0.05 (0.04, 0.06)	**1.09E-45**	0.04 (0.03, 0.04)	**2.70E-57**
**1.47E-08**	-0.02 (-0.02, -0.01)	**2.91E-07**	-0.01 (-0.01, 0.00)	**2.64E-04**
**2.30E-06**	-0.03 (-0.04, -0.03)	**5.49E-22**	-0.03 (-0.03, -0.02)	**3.18E-36**

**2.95E-05**	0.01 (0.01, 0.02)	**1.46E-05**	0.00 (0.00, 0.01)	6.50E-01
**1.09E-17**	-0.05 (-0.05, -0.04)	**3.77E-41**	-0.03 (-0.03, -0.02)	**1.12E-32**
**5.97E-16**	0.05 (0.04, 0.05)	**4.80E-40**	0.04 (0.03, 0.04)	**7.54E-55**
2.96E-01	-0.01 (-0.02, 0.00)	**1.71E-03**	0.00 (0.00, 0.01)	6.94E-02
**3.36E-11**	-0.03 (-0.04, -0.02)	**4.32E-19**	-0.03 (-0.04, -0.03)	**5.56E-45**
9.84E-02	0.01 (0.00, 0.02)	**1.90E-03**	-0.01 (-0.01, 0.00)	**2.06E-02**
**3.38E-17**	-0.04 (-0.05, -0.04)	**4.39E-36**	-0.03 (-0.03, -0.02)	**1.89E-32**
**3.34E-17**	0.05 (0.04, 0.05)	**3.89E-40**	0.03 (0.03, 0.04)	**4.60E-45**
**8.60E-08**	0.00 (-0.01, 0.00)	5.73E-01	0.01 (0.01, 0.02)	**5.10E-10**
**2.77E-20**	-0.02 (-0.03, -0.01)	**1.39E-09**	-0.03 (-0.03, -0.02)	**1.76E-35**
**8.43E-21**	-0.04 (-0.05, -0.03)	**6.26E-32**	-0.04 (-0.04, -0.03)	**1.32E-57**
**6.64E-05**	0.01 (0.01, 0.02)	**2.23E-04**	0.00 (-0.01, 0.00)	2.60E-01
**8.89E-04**	0.03 (0.02, 0.03)	**6.04E-16**	0.01 (0.01, 0.02)	**9.49E-08**
7.34E-01	0.03 (0.02, 0.04)	**1.99E-15**	0.01 (0.01, 0.02)	**5.31E-09**
**4.12E-03**	-0.04 (-0.05, -0.03)	**6.92E-29**	-0.02 (-0.02, -0.02)	**1.30E-17**
**2.20E-05**	-0.04 (-0.05, -0.04)	**1.87E-34**	-0.02 (-0.03, -0.02)	**4.23E-23**
**7.20E-05**	-0.01 (-0.02, 0.00)	**3.95E-03**	0.00 (0.00, 0.01)	7.07E-01
**5.37E-07**	0.04 (0.03, 0.05)	**7.73E-31**	0.02 (0.02, 0.02)	**1.61E-18**
**9.74E-14**	0.05 (0.04, 0.06)	**6.25E-42**	0.03 (0.03, 0.04)	**4.38E-42**
**1.00E-18**	-0.05 (-0.06, -0.04)	**8.87E-48**	-0.03 (-0.04, -0.03)	**4.53E-43**
**3.62E-21**	-0.05 (-0.06, -0.04)	**1.10E-47**	-0.03 (-0.04, -0.03)	**2.41E-42**
1.21E-01	-0.03 (-0.04, -0.02)	**4.67E-16**	-0.02 (-0.02, -0.01)	**4.59E-14**
**2.35E-19**	0.05 (0.04, 0.06)	**6.95E-46**	0.03 (0.02, 0.03)	**2.55E-37**
**3.08E-04**	0.01 (0.00, 0.02)	**1.71E-03**	0.01 (0.01, 0.02)	**5.39E-11**
**8.44E-19**	-0.04 (-0.05, -0.04)	**3.30E-35**	-0.03 (-0.03, -0.03)	**1.35E-42**
**1.10E-23**	-0.04 (-0.05, -0.04)	**4.74E-35**	-0.03 (-0.04, -0.03)	**1.38E-44**
**1.54E-06**	0.00 (-0.01, 0.01)	6.85E-01	0.00 (0.00, 0.01)	2.43E-01
**2.17E-22**	0.05 (0.05, 0.06)	**2.87E-50**	0.03 (0.03, 0.04)	**1.21E-43**
**3.91E-06**	0.03 (0.02, 0.04)	**1.32E-16**	0.01 (0.00, 0.01)	**1.59E-04**
1.54E-01	-0.02 (-0.03, -0.01)	**1.18E-09**	-0.01 (-0.02, -0.01)	**3.21E-06**
**4.23E-02**	-0.02 (-0.03, -0.01)	**2.96E-07**	0.00 (0.00, 0.01)	8.10E-01
4.09E-01	-0.01 (-0.02, -0.01)	**2.71E-04**	-0.02 (-0.03, -0.02)	**4.11E-21**
**1.47E-08**	0.02 (0.01, 0.03)	**3.46E-09**	0.00 (0.00, 0.01)	3.67E-01
2.74E-01	-0.01 (-0.02, 0.00)	**1.38E-02**	-0.02 (-0.02, -0.02)	**2.19E-19**
**1.14E-02**	0.00 (0.00, 0.01)	1.99E-01	-0.01 (-0.01, 0.00)	**3.50E-05**
1.13E-01	-0.02 (-0.02, -0.01)	**6.82E-06**	-0.02 (-0.03, -0.02)	**4.52E-25**
2.96E-01	-0.01 (-0.01, 0.00)	1.55E-01	-0.02 (-0.02, -0.01)	**3.09E-12**
**2.61E-12**	0.04 (0.03, 0.04)	**1.69E-25**	0.02 (0.01, 0.02)	**2.89E-11**
**2.78E-11**	0.03 (0.03, 0.04)	**5.20E-23**	0.01 (0.01, 0.02)	**9.33E-10**
**3.10E-11**	0.04 (0.03, 0.04)	**1.67E-27**	0.01 (0.01, 0.02)	**1.42E-10**
**4.97E-13**	0.04 (0.03, 0.04)	**6.26E-27**	0.02 (0.01, 0.02)	**1.38E-17**
1.79E-01	0.00 (-0.01, 0.00)	5.20E-01	-0.01 (-0.02, -0.01)	**2.18E-06**
**1.57E-14**	0.04 (0.04, 0.05)	**2.32E-35**	0.02 (0.02, 0.03)	**9.45E-23**
2.62E-01	-0.01 (-0.02, 0.00)	**3.92E-02**	-0.01 (-0.02, -0.01)	**4.51E-08**
2.43E-01	-0.01 (-0.02, -0.01)	**1.19E-04**	-0.02 (-0.02, -0.02)	**4.42E-17**
5.94E-01	-0.01 (-0.02, 0.00)	**2.80E-02**	-0.02 (-0.02, -0.01)	**9.45E-11**
**9.35E-05**	-0.03 (-0.03, -0.02)	**3.52E-12**	-0.02 (-0.03, -0.02)	**3.07E-19**
**1.50E-02**	0.00 (0.00, 0.01)	1.85E-01	-0.01 (-0.01, -0.01)	**2.57E-05**

**4.57E-05**	0.02 (0.01, 0.02)	**2.57E-06**	0.00 (0.00, 0.01)	4.27E-01
**2.67E-07**	0.02 (0.02, 0.03)	**8.01E-11**	0.00 (0.00, 0.01)	2.81E-01
**4.57E-05**	-0.02 (-0.03, -0.02)	**1.84E-10**	0.00 (-0.01, 0.00)	6.02E-01
**3.55E-27**	0.00 (0.00, 0.01)	2.15E-01	0.01 (0.01, 0.02)	**2.20E-10**
**2.42E-03**	0.00 (-0.01, 0.00)	3.64E-01	-0.01 (-0.02, -0.01)	**6.81E-08**
**6.57E-36**	0.02 (0.01, 0.03)	**2.18E-08**	0.02 (0.02, 0.02)	**3.80E-19**
**8.19E-03**	0.00 (-0.01, 0.01)	6.71E-01	-0.01 (-0.02, -0.01)	**2.97E-08**
**2.15E-08**	0.00 (0.00, 0.01)	2.38E-01	-0.01 (-0.01, 0.00)	**2.37E-02**
**6.80E-07**	0.03 (0.03, 0.04)	**1.79E-22**	0.01 (0.00, 0.01)	**1.38E-04**
**4.59E-05**	0.02 (0.02, 0.03)	**4.63E-12**	0.00 (0.00, 0.01)	1.70E-01
**1.32E-15**	-0.01 (-0.01, 0.00)	5.00E-02	0.02 (0.01, 0.02)	**4.99E-11**
**1.03E-04**	-0.04 (-0.05, -0.04)	**2.32E-35**	-0.03 (-0.03, -0.02)	**1.90E-29**
**1.63E-33**	0.01 (0.00, 0.02)	**1.78E-03**	0.02 (0.02, 0.03)	**9.05E-26**
**2.12E-11**	-0.05 (-0.05, -0.04)	**5.04E-43**	-0.02 (-0.03, -0.02)	**4.44E-26**
1.04E-01	-0.04 (-0.05, -0.03)	**3.59E-32**	-0.01 (-0.02, -0.01)	**4.17E-09**
**1.59E-04**	0.05 (0.04, 0.05)	**1.48E-40**	0.02 (0.01, 0.02)	**1.17E-14**
8.18E-02	0.02 (0.01, 0.02)	**3.89E-06**	0.00 (0.00, 0.01)	4.19E-01
**4.73E-05**	0.02 (0.02, 0.03)	**1.92E-12**	0.02 (0.01, 0.02)	**1.02E-12**
6.57E-01	0.01 (0.00, 0.01)	1.57E-01	0.00 (0.00, 0.01)	6.78E-01
6.54E-02	0.00 (0.00, 0.01)	2.94E-01	0.00 (-0.01, 0.00)	6.61E-01
**3.10E-03**	0.02 (0.01, 0.02)	**8.76E-06**	0.01 (0.00, 0.01)	**3.45E-05**
**3.57E-13**	-0.02 (-0.03, -0.02)	**1.30E-11**	-0.03 (-0.03, -0.02)	**2.03E-31**
9.77E-01	0.00 (0.00, 0.01)	6.43E-01	0.00 (-0.01, 0.00)	5.69E-01
1.60E-01	0.02 (0.01, 0.02)	**2.10E-06**	0.01 (0.00, 0.01)	**6.06E-03**
2.65E-01	0.02 (0.01, 0.02)	**1.83E-05**	0.00 (0.00, 0.01)	**3.42E-02**
**7.29E-05**	0.03 (0.03, 0.04)	**9.28E-22**	0.02 (0.01, 0.02)	**1.46E-11**
8.17E-02	0.00 (0.00, 0.01)	5.27E-01	0.00 (0.00, 0.01)	6.71E-02
7.38E-01	0.01 (0.00, 0.02)	**1.91E-03**	0.00 (0.00, 0.00)	8.10E-01
**5.19E-03**	0.00 (-0.01, 0.00)	5.61E-01	-0.01 (-0.01, 0.00)	**3.56E-03**
8.29E-02	0.00 (0.00, 0.01)	4.19E-01	0.00 (0.00, 0.01)	1.49E-01
**1.23E-02**	0.00 (-0.01, 0.01)	9.81E-01	0.00 (0.00, 0.01)	**3.70E-02**
**5.64E-07**	0.04 (0.04, 0.05)	**5.57E-32**	0.03 (0.03, 0.04)	**2.56E-39**
2.68E-01	0.00 (-0.01, 0.00)	1.70E-01	-0.01 (-0.01, 0.00)	**1.91E-02**
**3.34E-06**	0.04 (0.04, 0.05)	**5.38E-33**	0.02 (0.01, 0.02)	**4.21E-12**
**5.83E-05**	-0.02 (-0.03, -0.01)	**2.86E-07**	-0.02 (-0.02, -0.01)	**8.69E-11**
**9.03E-03**	0.02 (0.02, 0.03)	**6.14E-13**	0.01 (0.01, 0.01)	**3.48E-06**
5.36E-01	0.00 (-0.01, 0.00)	5.41E-01	-0.02 (-0.02, -0.01)	**1.29E-11**
7.05E-02	-0.01 (-0.02, 0.00)	**1.21E-03**	-0.02 (-0.03, -0.02)	**1.74E-22**
6.71E-01	-0.01 (-0.01, 0.00)	1.36E-01	-0.02 (-0.02, -0.01)	**4.51E-16**
**2.16E-07**	-0.04 (-0.05, -0.03)	**4.89E-25**	-0.03 (-0.03, -0.02)	**1.22E-26**
**3.20E-03**	-0.05 (-0.05, -0.04)	**1.86E-38**	-0.02 (-0.02, -0.01)	**2.30E-11**
**1.54E-32**	-0.02 (-0.03, -0.02)	**6.79E-11**	-0.03 (-0.03, -0.02)	**6.75E-37**
**4.41E-10**	0.03 (0.02, 0.03)	**1.02E-13**	0.01 (0.00, 0.01)	**1.76E-02**
**3.35E-03**	-0.02 (-0.02, -0.01)	**7.00E-07**	-0.02 (-0.03, -0.02)	**1.57E-28**
8.29E-02	-0.03 (-0.03, -0.02)	**2.11E-11**	-0.02 (-0.02, -0.01)	**7.84E-12**
8.08E-01	0.01 (0.00, 0.01)	1.29E-01	0.00 (0.00, 0.01)	2.08E-01
7.71E-01	-0.01 (-0.02, -0.01)	**4.48E-04**	0.00 (-0.01, 0.00)	7.64E-02
**1.59E-07**	-0.03 (-0.03, -0.02)	**1.30E-13**	-0.03 (-0.03, -0.02)	**3.66E-35**

**6.65E-12**	0.03 (0.02, 0.03)	**1.36E-14**	0.01 (0.00, 0.01)	**1.49E-03**
**2.28E-02**	0.00 (0.00, 0.01)	3.53E-01	-0.01 (-0.01, -0.01)	**6.23E-06**
**4.52E-09**	-0.03 (-0.04, -0.02)	**1.60E-15**	-0.03 (-0.03, -0.02)	**3.56E-34**
3.52E-01	0.00 (-0.01, 0.00)	2.75E-01	-0.01 (-0.02, -0.01)	**5.64E-10**
**2.04E-11**	0.03 (0.02, 0.04)	**9.25E-18**	0.01 (0.00, 0.01)	**1.72E-04**
2.43E-01	-0.01 (-0.01, 0.00)	5.33E-02	-0.02 (-0.02, -0.02)	**2.38E-18**
**2.05E-02**	-0.02 (-0.03, -0.01)	**5.70E-09**	-0.02 (-0.02, -0.01)	**7.97E-11**
**2.51E-02**	0.00 (0.00, 0.01)	2.40E-01	-0.01 (-0.01, -0.01)	**3.92E-06**
**1.66E-11**	0.03 (0.02, 0.04)	**1.37E-19**	0.01 (0.01, 0.01)	**7.91E-06**
5.40E-01	-0.01 (-0.01, 0.00)	1.30E-01	-0.02 (-0.02, -0.01)	**2.65E-16**
**5.19E-03**	-0.03 (-0.03, -0.02)	**3.93E-12**	-0.02 (-0.02, -0.01)	**3.96E-14**
**3.46E-07**	0.02 (0.01, 0.02)	**3.89E-06**	0.00 (0.00, 0.00)	8.69E-01
**7.98E-06**	-0.04 (-0.04, -0.03)	**6.51E-22**	-0.02 (-0.03, -0.02)	**3.36E-23**
**1.19E-07**	0.02 (0.02, 0.03)	**1.53E-10**	0.01 (0.00, 0.01)	**2.58E-04**

Non-white		Never/former smoker		Current smok
**Adjusted β (95% CI)**	**P value**	**Adjusted β (95% CI)**	**P value**	**Adjusted β (95% CI)**
0.08 (0.06, 0.10)	**2.23E-10**	0.02 (0.01, 0.02)	**1.54E-16**	0.02 (0.01, 0.04)
0.08 (0.06, 0.10)	**3.31E-10**	0.02 (0.01, 0.02)	**1.01E-14**	0.02 (0.01, 0.04)
0.08 (0.06, 0.11)	**8.48E-11**	0.02 (0.02, 0.02)	**2.34E-17**	0.02 (0.02, 0.04)
0.08 (0.06, 0.11)	**5.41E-11**	0.02 (0.01, 0.02)	**1.76E-14**	0.02 (0.01, 0.04)
0.08 (0.06, 0.10)	**1.24E-10**	0.02 (0.01, 0.02)	**2.02E-12**	0.02 (0.01, 0.04)
0.08 (0.06, 0.11)	**3.39E-11**	0.02 (0.01, 0.02)	**8.40E-17**	0.02 (0.01, 0.04)
0.07 (0.05, 0.10)	**1.49E-09**	0.02 (0.01, 0.02)	**7.66E-14**	0.02 (0.01, 0.04)
0.08 (0.06, 0.10)	**4.09E-10**	0.02 (0.01, 0.02)	**1.28E-12**	0.02 (0.00, 0.03)
0.08 (0.05, 0.10)	**4.41E-10**	0.02 (0.01, 0.02)	**5.27E-12**	0.02 (0.00, 0.03)
0.08 (0.05, 0.10)	**4.30E-10**	0.02 (0.01, 0.02)	**1.92E-12**	0.02 (0.00, 0.03)
0.07 (0.05, 0.10)	**5.38E-09**	0.01 (0.01, 0.02)	**1.40E-07**	0.01 (0.00, 0.03)
0.06 (0.04, 0.09)	**1.84E-07**	0.01 (0.00, 0.01)	**4.19E-04**	0.01 (-0.01, 0.02)
0.08 (0.05, 0.10)	**4.14E-10**	0.02 (0.01, 0.02)	**5.70E-12**	0.02 (0.00, 0.03)
0.08 (0.05, 0.10)	**4.79E-10**	0.02 (0.01, 0.02)	**4.46E-13**	0.02 (0.00, 0.03)
0.08 (0.05, 0.10)	**4.41E-10**	0.02 (0.01, 0.02)	**4.34E-11**	0.01 (0.00, 0.03)
0.08 (0.05, 0.10)	**9.20E-10**	0.01 (0.01, 0.02)	**2.41E-09**	0.01 (0.00, 0.03)
0.08 (0.06, 0.10)	**4.19E-10**	0.02 (0.01, 0.02)	**1.13E-11**	0.01 (0.00, 0.03)
0.07 (0.05, 0.10)	**8.10E-09**	0.01 (0.01, 0.02)	**2.90E-08**	0.01 (0.00, 0.03)
0.07 (0.04, 0.09)	**9.95E-08**	0.01 (0.01, 0.02)	**1.12E-06**	0.01 (-0.01, 0.02)
0.08 (0.05, 0.10)	**1.49E-09**	0.01 (0.01, 0.02)	**1.29E-09**	0.01 (0.00, 0.03)
0.08 (0.05, 0.10)	**1.37E-09**	0.01 (0.01, 0.02)	**1.68E-08**	0.01 (0.00, 0.03)
0.05 (0.02, 0.07)	**1.24E-04**	0.00 (0.00, 0.01)	6.10E-01	0.00 (-0.01, 0.02)
0.06 (0.03, 0.08)	**4.33E-06**	0.00 (0.00, 0.01)	6.36E-02	0.01 (-0.01, 0.02)
0.04 (0.02, 0.07)	**4.46E-04**	0.00 (0.00, 0.00)	8.00E-01	0.00 (-0.01, 0.02)
0.01 (-0.01, 0.04)	3.25E-01	-0.01 (-0.01, 0.00)	**2.10E-04**	0.00 (-0.02, 0.01)
0.00 (-0.03, 0.02)	7.32E-01	-0.01 (-0.02, -0.01)	**6.64E-09**	-0.01 (-0.02, 0.01)
0.03 (0.01, 0.06)	**5.69E-03**	0.00 (-0.01, 0.00)	4.27E-01	0.00 (-0.01, 0.02)
0.08 (0.05, 0.10)	**1.37E-09**	0.01 (0.01, 0.02)	**9.25E-08**	0.01 (0.00, 0.03)
0.07 (0.05, 0.09)	**2.91E-08**	0.01 (0.01, 0.02)	**1.53E-10**	0.01 (0.00, 0.03)
0.06 (0.04, 0.09)	**1.47E-07**	0.01 (0.01, 0.02)	**5.07E-08**	0.01 (0.00, 0.03)
0.05 (0.02, 0.07)	**1.86E-04**	0.00 (0.00, 0.01)	8.72E-02	0.00 (-0.01, 0.02)
0.04 (0.01, 0.06)	**2.78E-03**	0.00 (0.00, 0.01)	2.79E-01	0.00 (-0.01, 0.02)
0.04 (0.02, 0.06)	**7.56E-04**	0.01 (0.00, 0.01)	**1.79E-02**	0.00 (-0.01, 0.02)
0.03 (0.01, 0.05)	**2.05E-02**	0.00 (-0.01, 0.00)	2.49E-01	0.00 (-0.02, 0.01)
0.08 (0.06, 0.11)	**2.71E-11**	0.02 (0.02, 0.03)	**1.53E-23**	0.02 (0.00, 0.03)
0.03 (0.01, 0.05)	**1.66E-02**	0.01 (0.00, 0.01)	**5.48E-04**	0.01 (0.00, 0.03)
0.03 (0.01, 0.06)	**5.41E-03**	0.01 (0.01, 0.01)	**2.06E-05**	0.01 (0.00, 0.03)
0.04 (0.02, 0.07)	**4.16E-04**	0.01 (0.01, 0.02)	**1.90E-09**	0.02 (0.00, 0.03)
0.01 (-0.02, 0.03)	6.81E-01	0.00 (-0.01, 0.00)	6.03E-01	0.01 (-0.01, 0.02)
0.00 (-0.03, 0.02)	7.73E-01	0.00 (-0.01, 0.00)	**4.45E-02**	0.01 (-0.01, 0.02)
0.03 (0.00, 0.05)	**3.09E-02**	0.01 (0.00, 0.01)	**3.55E-03**	0.01 (0.00, 0.03)
0.07 (0.05, 0.10)	**2.11E-09**	0.03 (0.02, 0.03)	**1.07E-29**	0.02 (0.01, 0.04)
-0.01 (-0.03, 0.01)	4.89E-01	-0.01 (-0.02, -0.01)	**8.56E-11**	-0.01 (-0.02, 0.01)

-0.02 (-0.04, 0.00)	1.08E-01	-0.02 (-0.02, -0.01)	**2.73E-14**	-0.01 (-0.02, 0.01)
-0.01 (-0.03, 0.01)	3.96E-01	-0.01 (-0.02, -0.01)	**3.83E-08**	0.00 (-0.02, 0.01)
-0.03 (-0.06, -0.01)	**9.57E-03**	-0.02 (-0.03, -0.02)	**1.55E-22**	-0.01 (-0.03, 0.01)
-0.03 (-0.06, -0.01)	**1.08E-02**	-0.02 (-0.03, -0.02)	**1.12E-23**	-0.01 (-0.02, 0.01)
-0.03 (-0.06, -0.01)	**7.65E-03**	-0.02 (-0.03, -0.02)	**1.96E-18**	-0.01 (-0.03, 0.00)
0.06 (0.04, 0.08)	**1.37E-06**	0.02 (0.02, 0.03)	**1.31E-20**	0.02 (0.00, 0.03)
0.01 (-0.02, 0.03)	5.80E-01	-0.01 (-0.01, 0.00)	**7.13E-05**	-0.01 (-0.02, 0.01)
-0.02 (-0.04, 0.01)	2.31E-01	-0.02 (-0.02, -0.02)	**6.20E-18**	-0.01 (-0.03, 0.00)
-0.02 (-0.04, 0.00)	1.22E-01	-0.02 (-0.03, -0.02)	**7.74E-21**	-0.01 (-0.03, 0.00)
-0.02 (-0.04, 0.00)	1.20E-01	-0.02 (-0.03, -0.02)	**3.71E-21**	-0.01 (-0.03, 0.00)
-0.01 (-0.04, 0.01)	2.76E-01	-0.02 (-0.02, -0.02)	**2.74E-18**	-0.01 (-0.03, 0.00)
-0.04 (-0.06, -0.01)	**4.92E-03**	-0.03 (-0.03, -0.02)	**4.98E-29**	-0.02 (-0.03, 0.00)
0.05 (0.03, 0.08)	**1.52E-05**	0.02 (0.01, 0.02)	**3.12E-13**	0.01 (0.00, 0.03)
0.02 (0.00, 0.04)	1.16E-01	-0.01 (-0.01, 0.00)	**4.53E-04**	0.00 (-0.02, 0.01)
0.01 (-0.01, 0.03)	5.36E-01	-0.01 (-0.02, -0.01)	**2.20E-10**	-0.01 (-0.03, 0.00)
0.00 (-0.02, 0.02)	8.78E-01	-0.02 (-0.02, -0.01)	**8.26E-13**	-0.01 (-0.03, 0.00)
0.01 (-0.02, 0.03)	6.93E-01	-0.02 (-0.02, -0.01)	**2.06E-12**	-0.01 (-0.03, 0.00)
0.02 (-0.01, 0.04)	1.91E-01	-0.01 (-0.02, -0.01)	**2.85E-08**	-0.01 (-0.02, 0.01)
-0.03 (-0.05, 0.00)	**4.19E-02**	-0.02 (-0.03, -0.02)	**5.51E-26**	-0.02 (-0.03, 0.00)
0.07 (0.04, 0.09)	**1.47E-07**	0.02 (0.01, 0.02)	**2.54E-14**	0.01 (0.00, 0.03)
0.03 (0.00, 0.05)	**4.45E-02**	0.00 (-0.01, 0.00)	**3.48E-02**	0.00 (-0.02, 0.01)
0.01 (-0.01, 0.04)	2.76E-01	-0.01 (-0.02, -0.01)	**2.36E-07**	-0.01 (-0.02, 0.01)
0.01 (-0.01, 0.03)	5.35E-01	-0.01 (-0.02, -0.01)	**1.42E-07**	-0.01 (-0.02, 0.00)
0.01 (-0.02, 0.03)	5.81E-01	-0.01 (-0.02, -0.01)	**7.53E-11**	-0.01 (-0.02, 0.00)
0.02 (0.00, 0.04)	1.18E-01	-0.01 (-0.02, -0.01)	**8.52E-07**	-0.01 (-0.02, 0.01)
-0.03 (-0.05, 0.00)	**3.53E-02**	-0.02 (-0.03, -0.02)	**1.45E-23**	-0.02 (-0.03, 0.00)
0.08 (0.06, 0.10)	**2.85E-10**	0.02 (0.02, 0.02)	**2.19E-18**	0.02 (0.00, 0.03)
-0.02 (-0.04, 0.01)	2.19E-01	0.00 (-0.01, 0.00)	2.78E-01	0.01 (0.00, 0.03)
-0.02 (-0.05, 0.00)	1.14E-01	0.00 (-0.01, 0.00)	2.86E-01	0.01 (0.00, 0.03)
-0.02 (-0.04, 0.00)	1.52E-01	0.00 (-0.01, 0.00)	7.29E-01	0.01 (0.00, 0.03)
-0.03 (-0.06, -0.01)	**2.03E-02**	-0.01 (-0.01, 0.00)	**3.67E-03**	0.01 (-0.01, 0.02)
-0.03 (-0.06, -0.01)	**1.62E-02**	-0.01 (-0.01, 0.00)	**2.45E-04**	0.01 (-0.01, 0.02)
-0.02 (-0.05, 0.00)	5.95E-02	0.00 (0.00, 0.00)	9.86E-01	0.01 (-0.01, 0.02)
0.06 (0.04, 0.09)	**1.47E-07**	0.02 (0.02, 0.03)	**9.55E-27**	0.03 (0.01, 0.04)
-0.04 (-0.06, -0.01)	**7.63E-03**	-0.01 (-0.02, -0.01)	**9.29E-08**	0.01 (-0.01, 0.02)
-0.04 (-0.06, -0.01)	**5.30E-03**	-0.01 (-0.02, -0.01)	**1.23E-07**	0.01 (-0.01, 0.02)
-0.04 (-0.06, -0.01)	**7.47E-03**	-0.01 (-0.02, -0.01)	**3.67E-07**	0.01 (-0.01, 0.02)
-0.04 (-0.07, -0.02)	**1.10E-03**	-0.02 (-0.02, -0.01)	**4.17E-10**	0.00 (-0.01, 0.02)
-0.05 (-0.07, -0.02)	**6.65E-04**	-0.02 (-0.02, -0.01)	**2.70E-11**	0.00 (-0.01, 0.02)
-0.04 (-0.06, -0.01)	**6.43E-03**	-0.01 (-0.02, -0.01)	**2.19E-06**	0.01 (-0.01, 0.02)
0.04 (0.02, 0.06)	**1.60E-03**	0.02 (0.02, 0.02)	**1.27E-17**	0.02 (0.01, 0.04)
-0.03 (-0.06, -0.01)	**1.59E-02**	-0.03 (-0.03, -0.02)	**3.71E-25**	0.00 (-0.02, 0.01)
-0.03 (-0.05, 0.00)	**4.97E-02**	-0.02 (-0.03, -0.02)	**1.87E-21**	0.00 (-0.01, 0.02)
-0.02 (-0.04, 0.01)	1.88E-01	-0.02 (-0.02, -0.02)	**4.10E-16**	0.00 (-0.01, 0.02)
-0.05 (-0.07, -0.02)	**5.75E-04**	-0.03 (-0.03, -0.03)	**2.15E-34**	-0.01 (-0.02, 0.01)
-0.05 (-0.07, -0.02)	**2.87E-04**	-0.03 (-0.04, -0.03)	**3.47E-37**	-0.01 (-0.02, 0.01)
-0.04 (-0.06, -0.01)	**1.02E-02**	-0.02 (-0.03, -0.02)	**5.63E-23**	0.00 (-0.02, 0.02)
0.06 (0.03, 0.08)	**3.75E-06**	0.02 (0.01, 0.02)	**6.47E-16**	0.02 (0.01, 0.04)

-0.02 (-0.04, 0.00)	1.23E-01	-0.03 (-0.04, -0.03)	**1.71E-42**	-0.02 (-0.03, 0.00)
0.00 (-0.02, 0.02)	9.21E-01	-0.02 (-0.03, -0.02)	**7.16E-23**	-0.01 (-0.02, 0.01)
0.00 (-0.02, 0.03)	7.93E-01	-0.02 (-0.02, -0.02)	**3.61E-18**	0.00 (-0.02, 0.01)
-0.03 (-0.05, -0.01)	**2.30E-02**	-0.03 (-0.04, -0.03)	**2.65E-49**	-0.02 (-0.03, 0.00)
-0.03 (-0.06, -0.01)	**5.41E-03**	-0.04 (-0.04, -0.03)	**2.01E-57**	-0.02 (-0.04, -0.01)
-0.01 (-0.03, 0.02)	6.93E-01	-0.02 (-0.03, -0.02)	**2.87E-19**	-0.01 (-0.02, 0.01)
0.07 (0.05, 0.10)	**6.10E-09**	0.02 (0.02, 0.03)	**4.27E-19**	0.02 (0.00, 0.03)
0.03 (0.01, 0.05)	**2.25E-02**	0.02 (0.01, 0.02)	**1.37E-14**	0.00 (-0.01, 0.02)
-0.06 (-0.08, -0.03)	**3.89E-06**	-0.01 (-0.02, -0.01)	**5.50E-06**	-0.01 (-0.03, 0.00)
-0.05 (-0.08, -0.03)	**1.45E-05**	-0.01 (-0.02, -0.01)	**7.33E-07**	-0.01 (-0.02, 0.01)
-0.05 (-0.07, -0.02)	**1.86E-04**	-0.01 (-0.01, 0.00)	**1.61E-02**	-0.01 (-0.03, 0.00)
0.04 (0.02, 0.07)	**8.92E-04**	0.00 (0.00, 0.01)	8.24E-02	0.01 (0.00, 0.03)
-0.08 (-0.10, -0.06)	**1.24E-10**	-0.02 (-0.02, -0.01)	**1.17E-11**	-0.01 (-0.03, 0.00)
-0.09 (-0.11, -0.06)	**1.21E-11**	-0.02 (-0.02, -0.01)	**2.50E-14**	-0.02 (-0.03, 0.00)
-0.03 (-0.05, 0.00)	**3.53E-02**	0.00 (-0.01, 0.00)	1.40E-01	0.00 (-0.02, 0.01)
0.06 (0.04, 0.09)	**2.93E-07**	0.01 (0.01, 0.02)	**6.49E-07**	0.01 (0.00, 0.03)
0.06 (0.04, 0.09)	**2.36E-07**	0.02 (0.02, 0.02)	**1.30E-17**	0.02 (0.01, 0.04)
-0.05 (-0.07, -0.02)	**1.86E-04**	0.00 (-0.01, 0.00)	2.31E-01	-0.01 (-0.02, 0.01)
-0.06 (-0.08, -0.04)	**1.24E-06**	-0.01 (-0.01, 0.00)	**2.58E-03**	-0.01 (-0.02, 0.01)
0.00 (-0.03, 0.02)	7.85E-01	0.01 (0.00, 0.01)	**6.67E-04**	0.00 (-0.01, 0.02)
0.00 (-0.03, 0.02)	7.56E-01	-0.01 (-0.01, 0.00)	**9.51E-05**	0.00 (-0.02, 0.01)
-0.06 (-0.08, -0.03)	**6.77E-06**	-0.02 (-0.03, -0.02)	**4.27E-19**	-0.01 (-0.03, 0.00)
-0.08 (-0.11, -0.06)	**5.41E-11**	-0.03 (-0.03, -0.02)	**3.26E-30**	-0.02 (-0.04, -0.01)
-0.09 (-0.11, -0.06)	**1.21E-11**	-0.03 (-0.03, -0.02)	**2.03E-32**	-0.02 (-0.04, -0.01)
-0.07 (-0.09, -0.04)	**4.88E-08**	-0.02 (-0.03, -0.02)	**2.88E-22**	-0.02 (-0.03, 0.00)
0.08 (0.06, 0.10)	**3.54E-10**	0.03 (0.02, 0.03)	**1.69E-28**	0.02 (0.00, 0.03)
-0.08 (-0.11, -0.06)	**2.71E-11**	-0.04 (-0.04, -0.03)	**1.75E-55**	-0.03 (-0.04, -0.01)
-0.07 (-0.10, -0.05)	**2.12E-09**	-0.03 (-0.03, -0.02)	**1.00E-31**	-0.02 (-0.04, -0.01)
-0.06 (-0.08, -0.03)	**3.89E-06**	-0.02 (-0.02, -0.01)	**9.17E-14**	-0.02 (-0.03, 0.00)
-0.09 (-0.11, -0.06)	**1.21E-11**	-0.04 (-0.04, -0.03)	**3.56E-55**	-0.03 (-0.05, -0.01)
0.08 (0.06, 0.10)	**3.04E-10**	0.03 (0.03, 0.04)	**1.26E-39**	0.03 (0.01, 0.04)
0.09 (0.07, 0.12)	**5.25E-13**	0.04 (0.03, 0.04)	**2.30E-62**	0.02 (0.00, 0.03)
-0.10 (-0.12, -0.07)	**4.12E-13**	-0.04 (-0.04, -0.03)	**2.75E-55**	-0.02 (-0.04, -0.01)
-0.10 (-0.12, -0.07)	**1.51E-13**	-0.04 (-0.04, -0.03)	**9.40E-58**	-0.02 (-0.04, -0.01)
-0.05 (-0.07, -0.03)	**6.60E-05**	-0.02 (-0.03, -0.02)	**5.19E-20**	-0.02 (-0.04, -0.01)
0.08 (0.06, 0.11)	**1.24E-10**	0.03 (0.03, 0.04)	**5.52E-43**	0.02 (0.01, 0.04)
0.06 (0.04, 0.09)	**1.19E-07**	0.03 (0.03, 0.04)	**1.22E-51**	0.03 (0.01, 0.04)
-0.09 (-0.12, -0.07)	**3.87E-13**	-0.05 (-0.05, -0.04)	**1.55E-85**	-0.03 (-0.05, -0.02)
-0.08 (-0.11, -0.06)	**4.96E-11**	-0.05 (-0.05, -0.04)	**8.13E-86**	-0.03 (-0.04, -0.01)
-0.06 (-0.09, -0.04)	**3.19E-07**	-0.02 (-0.02, -0.02)	**8.77E-19**	-0.03 (-0.04, -0.01)
0.08 (0.06, 0.11)	**3.39E-11**	0.04 (0.04, 0.05)	**3.94E-71**	0.03 (0.02, 0.05)
-0.03 (-0.05, -0.01)	**1.36E-02**	-0.01 (-0.01, 0.00)	**1.31E-02**	0.00 (-0.02, 0.01)
-0.05 (-0.07, -0.02)	**2.72E-04**	-0.04 (-0.04, -0.03)	**6.12E-51**	-0.03 (-0.04, -0.01)
0.01 (-0.01, 0.03)	5.57E-01	-0.02 (-0.02, -0.01)	**3.20E-11**	-0.01 (-0.02, 0.01)
-0.09 (-0.11, -0.06)	**3.28E-11**	-0.04 (-0.04, -0.03)	**1.97E-49**	-0.03 (-0.05, -0.01)
0.06 (0.04, 0.09)	**5.37E-07**	0.04 (0.03, 0.04)	**1.30E-62**	0.03 (0.01, 0.04)
-0.05 (-0.08, -0.03)	**8.59E-06**	-0.01 (-0.01, -0.01)	**7.28E-06**	-0.01 (-0.03, 0.00)
-0.03 (-0.06, -0.01)	**7.47E-03**	-0.03 (-0.03, -0.02)	**1.07E-37**	-0.02 (-0.04, -0.01)

0.06 (0.04, 0.09)	**2.67E-07**	0.00 (0.00, 0.01)	1.12E-01	0.01 (0.00, 0.02)
-0.10 (-0.12, -0.07)	**1.65E-13**	-0.03 (-0.04, -0.03)	**9.64E-40**	-0.03 (-0.05, -0.02)
0.06 (0.04, 0.08)	**5.27E-07**	0.04 (0.03, 0.04)	**9.05E-60**	0.03 (0.01, 0.04)
-0.02 (-0.04, 0.00)	1.13E-01	0.00 (0.00, 0.01)	8.60E-02	0.00 (-0.02, 0.01)
-0.05 (-0.07, -0.03)	**7.68E-05**	-0.03 (-0.04, -0.03)	**2.15E-47**	-0.03 (-0.04, -0.01)
0.04 (0.02, 0.06)	**1.23E-03**	0.00 (-0.01, 0.00)	8.88E-02	0.00 (-0.01, 0.02)
-0.09 (-0.11, -0.06)	**4.62E-12**	-0.03 (-0.03, -0.03)	**2.63E-38**	-0.03 (-0.05, -0.02)
0.07 (0.05, 0.09)	**2.38E-08**	0.03 (0.03, 0.04)	**8.36E-49**	0.03 (0.02, 0.05)
-0.01 (-0.03, 0.02)	5.74E-01	0.01 (0.01, 0.02)	**3.62E-08**	0.02 (0.01, 0.04)
-0.03 (-0.05, -0.01)	**2.34E-02**	-0.03 (-0.03, -0.02)	**8.33E-32**	-0.04 (-0.06, -0.03)
-0.04 (-0.07, -0.02)	**3.92E-04**	-0.04 (-0.04, -0.03)	**3.95E-56**	-0.04 (-0.06, -0.03)
0.00 (-0.02, 0.03)	8.92E-01	0.00 (-0.01, 0.00)	6.07E-01	-0.02 (-0.03, 0.00)
0.06 (0.04, 0.09)	**7.26E-07**	0.01 (0.01, 0.02)	**7.29E-09**	0.02 (0.00, 0.03)
0.02 (0.00, 0.05)	7.26E-02	0.01 (0.01, 0.02)	**2.97E-08**	0.01 (0.00, 0.03)
-0.05 (-0.07, -0.03)	**6.05E-05**	-0.02 (-0.03, -0.02)	**5.02E-20**	-0.01 (-0.03, 0.00)
-0.05 (-0.08, -0.03)	**2.29E-05**	-0.03 (-0.03, -0.02)	**6.88E-26**	-0.02 (-0.03, 0.00)
-0.02 (-0.04, 0.00)	1.33E-01	0.00 (0.00, 0.00)	9.82E-01	0.01 (-0.01, 0.02)
0.06 (0.04, 0.08)	**2.35E-06**	0.02 (0.02, 0.03)	**3.84E-24**	0.01 (0.00, 0.03)
0.07 (0.05, 0.10)	**1.03E-08**	0.03 (0.03, 0.04)	**1.23E-47**	0.03 (0.01, 0.04)
-0.08 (-0.11, -0.06)	**1.85E-10**	-0.04 (-0.04, -0.03)	**7.84E-52**	-0.02 (-0.04, -0.01)
-0.08 (-0.10, -0.05)	**7.04E-10**	-0.03 (-0.04, -0.03)	**4.87E-50**	-0.03 (-0.04, -0.01)
-0.05 (-0.07, -0.02)	**3.11E-04**	-0.02 (-0.03, -0.02)	**6.87E-19**	0.00 (-0.02, 0.01)
0.08 (0.06, 0.10)	**2.46E-10**	0.03 (0.03, 0.04)	**1.45E-47**	0.02 (0.01, 0.04)
0.03 (0.01, 0.06)	**9.91E-03**	0.01 (0.01, 0.02)	**4.67E-09**	0.03 (0.01, 0.04)
-0.08 (-0.10, -0.05)	**5.69E-10**	-0.03 (-0.04, -0.03)	**5.50E-47**	-0.03 (-0.05, -0.02)
-0.07 (-0.10, -0.05)	**2.66E-09**	-0.03 (-0.04, -0.03)	**9.53E-50**	-0.03 (-0.05, -0.02)
-0.01 (-0.03, 0.01)	4.52E-01	0.00 (0.00, 0.01)	1.21E-01	0.00 (-0.02, 0.01)
0.09 (0.07, 0.11)	**4.90E-12**	0.04 (0.03, 0.04)	**2.75E-55**	0.02 (0.01, 0.04)
0.09 (0.07, 0.12)	**4.90E-12**	0.01 (0.01, 0.02)	**6.45E-08**	0.01 (0.00, 0.03)
-0.04 (-0.07, -0.02)	**5.35E-04**	-0.01 (-0.02, -0.01)	**2.05E-07**	-0.01 (-0.03, 0.01)
-0.02 (-0.05, 0.00)	8.98E-02	0.00 (-0.01, 0.00)	3.88E-01	0.02 (0.00, 0.03)
-0.01 (-0.04, 0.01)	2.95E-01	-0.02 (-0.03, -0.02)	**3.71E-21**	-0.01 (-0.02, 0.01)
0.05 (0.02, 0.07)	**1.26E-04**	0.00 (0.00, 0.01)	**4.29E-02**	0.01 (-0.01, 0.02)
-0.01 (-0.03, 0.01)	4.29E-01	-0.02 (-0.02, -0.02)	**6.29E-18**	-0.01 (-0.03, 0.00)
0.01 (-0.01, 0.03)	3.63E-01	-0.01 (-0.01, 0.00)	**2.84E-04**	0.00 (-0.01, 0.02)
-0.02 (-0.04, 0.00)	1.30E-01	-0.02 (-0.03, -0.02)	**1.36E-25**	-0.01 (-0.02, 0.01)
0.00 (-0.02, 0.02)	9.71E-01	-0.02 (-0.02, -0.01)	**1.71E-11**	0.00 (-0.02, 0.01)
0.08 (0.06, 0.10)	**3.04E-10**	0.02 (0.01, 0.02)	**2.36E-15**	0.02 (0.00, 0.03)
0.08 (0.06, 0.10)	**1.85E-10**	0.02 (0.01, 0.02)	**1.34E-13**	0.02 (0.00, 0.03)
0.06 (0.04, 0.08)	**9.78E-07**	0.02 (0.01, 0.02)	**1.77E-14**	0.01 (0.00, 0.03)
0.06 (0.04, 0.08)	**5.82E-07**	0.02 (0.02, 0.03)	**2.46E-20**	0.02 (0.01, 0.04)
0.01 (-0.01, 0.03)	4.29E-01	-0.01 (-0.02, -0.01)	**3.51E-07**	0.01 (-0.01, 0.02)
0.10 (0.07, 0.12)	**4.69E-13**	0.03 (0.02, 0.03)	**9.68E-31**	0.02 (0.01, 0.04)
0.01 (-0.02, 0.03)	6.73E-01	-0.01 (-0.02, -0.01)	**4.17E-09**	0.01 (-0.01, 0.02)
-0.03 (-0.05, 0.00)	**4.43E-02**	-0.02 (-0.03, -0.02)	**1.34E-18**	-0.01 (-0.02, 0.01)
0.00 (-0.02, 0.03)	7.73E-01	-0.02 (-0.02, -0.01)	**1.30E-11**	0.00 (-0.01, 0.02)
-0.03 (-0.06, -0.01)	**2.40E-02**	-0.03 (-0.03, -0.02)	**8.93E-25**	0.00 (-0.02, 0.01)
0.01 (-0.01, 0.04)	2.61E-01	-0.01 (-0.01, 0.00)	**6.32E-04**	0.00 (-0.02, 0.01)

0.03 (0.00, 0.05)	**3.19E-02**	0.01 (0.00, 0.01)	**1.69E-02**	0.00 (-0.02, 0.01)
0.05 (0.03, 0.08)	**1.16E-05**	0.00 (0.00, 0.01)	6.30E-02	0.01 (0.00, 0.03)
-0.06 (-0.09, -0.04)	**5.34E-07**	-0.01 (-0.01, 0.00)	**2.94E-02**	0.00 (-0.02, 0.01)
-0.01 (-0.03, 0.02)	5.76E-01	0.01 (0.01, 0.02)	**2.09E-06**	0.03 (0.02, 0.05)
0.04 (0.02, 0.07)	**5.75E-04**	-0.01 (-0.01, 0.00)	**1.63E-04**	-0.01 (-0.02, 0.01)
0.02 (-0.01, 0.04)	2.22E-01	0.02 (0.01, 0.02)	**1.56E-14**	0.04 (0.02, 0.05)
0.03 (0.01, 0.06)	**5.93E-03**	-0.01 (-0.01, -0.01)	**1.67E-05**	0.00 (-0.02, 0.01)
0.03 (0.01, 0.06)	**5.76E-03**	0.00 (-0.01, 0.00)	1.18E-01	0.01 (-0.01, 0.02)
0.07 (0.05, 0.10)	**9.20E-10**	0.01 (0.01, 0.02)	**3.49E-07**	0.01 (0.00, 0.03)
0.05 (0.02, 0.07)	**2.47E-04**	0.00 (0.00, 0.01)	8.88E-02	0.02 (0.00, 0.03)
-0.04 (-0.07, -0.02)	**5.57E-04**	0.01 (0.01, 0.01)	**3.61E-05**	0.03 (0.01, 0.04)
-0.01 (-0.03, 0.01)	4.50E-01	-0.02 (-0.03, -0.02)	**3.72E-23**	-0.03 (-0.05, -0.02)
-0.02 (-0.04, 0.01)	1.79E-01	0.02 (0.01, 0.02)	**4.43E-16**	0.04 (0.03, 0.06)
-0.06 (-0.08, -0.04)	**6.77E-07**	-0.02 (-0.03, -0.02)	**9.18E-26**	-0.04 (-0.05, -0.02)
-0.07 (-0.09, -0.05)	**2.36E-08**	-0.02 (-0.02, -0.01)	**2.10E-11**	-0.02 (-0.04, -0.01)
0.11 (0.08, 0.13)	**1.44E-16**	0.02 (0.02, 0.03)	**1.06E-22**	0.02 (0.00, 0.03)
0.00 (-0.03, 0.02)	7.56E-01	0.00 (-0.01, 0.00)	8.00E-01	0.02 (0.00, 0.03)
0.02 (0.00, 0.05)	7.65E-02	0.02 (0.01, 0.02)	**5.00E-12**	0.02 (0.00, 0.03)
0.04 (0.02, 0.06)	**7.56E-04**	0.01 (0.00, 0.01)	**2.74E-02**	-0.01 (-0.03, 0.00)
-0.02 (-0.04, 0.01)	1.63E-01	0.00 (-0.01, 0.00)	7.37E-01	-0.01 (-0.03, 0.00)
0.04 (0.01, 0.06)	**3.49E-03**	0.01 (0.01, 0.01)	**1.50E-05**	0.01 (0.00, 0.03)
-0.02 (-0.04, 0.00)	9.49E-02	-0.02 (-0.03, -0.02)	**6.08E-25**	-0.04 (-0.05, -0.02)
0.02 (-0.01, 0.04)	2.44E-01	0.00 (-0.01, 0.00)	7.18E-01	0.00 (-0.02, 0.01)
0.00 (-0.02, 0.03)	7.34E-01	0.01 (0.00, 0.01)	**1.92E-03**	-0.01 (-0.02, 0.01)
0.00 (-0.03, 0.02)	7.56E-01	0.00 (0.00, 0.01)	**3.56E-02**	-0.01 (-0.02, 0.01)
0.02 (0.00, 0.05)	7.20E-02	0.02 (0.01, 0.02)	**2.19E-12**	0.00 (-0.01, 0.02)
0.02 (0.00, 0.05)	6.19E-02	0.01 (0.00, 0.01)	**9.33E-03**	-0.01 (-0.03, 0.01)
0.00 (-0.02, 0.02)	8.81E-01	0.00 (0.00, 0.01)	5.26E-01	-0.01 (-0.03, 0.00)
-0.01 (-0.03, 0.02)	6.72E-01	-0.01 (-0.01, 0.00)	**4.50E-03**	-0.01 (-0.02, 0.01)
0.01 (-0.02, 0.03)	5.85E-01	0.00 (0.00, 0.01)	2.16E-01	0.01 (-0.01, 0.02)
0.02 (-0.01, 0.04)	1.81E-01	0.01 (0.00, 0.01)	**1.61E-02**	0.00 (-0.01, 0.02)
0.00 (-0.02, 0.02)	9.79E-01	0.03 (0.03, 0.04)	**1.76E-33**	0.02 (0.00, 0.04)
0.00 (-0.03, 0.02)	7.73E-01	-0.01 (-0.01, 0.00)	**1.16E-02**	0.00 (-0.02, 0.01)
0.07 (0.05, 0.09)	**3.67E-08**	0.02 (0.02, 0.03)	**1.52E-19**	0.00 (-0.01, 0.02)
-0.02 (-0.04, 0.00)	1.30E-01	-0.01 (-0.02, -0.01)	**5.57E-09**	-0.02 (-0.04, -0.01)
0.01 (-0.01, 0.03)	4.16E-01	0.01 (0.01, 0.02)	**1.17E-06**	0.00 (-0.02, 0.01)
0.00 (-0.02, 0.02)	9.79E-01	-0.01 (-0.02, -0.01)	**2.51E-10**	-0.01 (-0.02, 0.01)
-0.02 (-0.04, 0.00)	1.25E-01	-0.02 (-0.03, -0.02)	**1.96E-21**	-0.01 (-0.03, 0.00)
0.00 (-0.03, 0.02)	8.50E-01	-0.02 (-0.02, -0.01)	**2.30E-14**	-0.01 (-0.03, 0.00)
-0.06 (-0.08, -0.03)	**5.09E-05**	-0.03 (-0.04, -0.03)	**4.47E-34**	-0.01 (-0.02, 0.01)
-0.10 (-0.12, -0.07)	**3.55E-14**	-0.02 (-0.02, -0.02)	**2.18E-17**	-0.02 (-0.03, 0.00)
0.01 (-0.02, 0.03)	5.76E-01	-0.02 (-0.03, -0.02)	**1.96E-23**	-0.04 (-0.06, -0.03)
0.06 (0.04, 0.08)	**1.24E-06**	0.01 (0.00, 0.01)	**2.41E-04**	0.01 (0.00, 0.03)
-0.03 (-0.05, -0.01)	**1.03E-02**	-0.03 (-0.03, -0.02)	**3.94E-28**	-0.02 (-0.03, 0.00)
-0.04 (-0.06, -0.01)	**9.92E-03**	-0.02 (-0.03, -0.02)	**7.53E-16**	0.00 (-0.01, 0.02)
0.03 (0.01, 0.06)	**7.63E-03**	0.00 (0.00, 0.01)	**4.80E-02**	0.00 (-0.01, 0.02)
-0.01 (-0.03, 0.01)	4.03E-01	-0.01 (-0.01, 0.00)	**1.87E-02**	0.00 (-0.01, 0.02)
-0.03 (-0.05, -0.01)	**2.30E-02**	-0.03 (-0.04, -0.03)	**1.13E-40**	-0.01 (-0.02, 0.01)

0.06 (0.03, 0.08)	**2.42E-06**	0.01 (0.01, 0.01)	**7.28E-06**	0.01 (0.00, 0.03)
0.01 (-0.01, 0.04)	3.08E-01	-0.01 (-0.01, 0.00)	**2.15E-04**	0.00 (-0.02, 0.01)
-0.03 (-0.06, -0.01)	**9.33E-03**	-0.03 (-0.04, -0.03)	**8.71E-41**	-0.01 (-0.02, 0.01)
0.01 (-0.02, 0.03)	5.66E-01	-0.02 (-0.02, -0.01)	**1.80E-10**	0.00 (-0.01, 0.02)
0.07 (0.04, 0.09)	**7.11E-08**	0.01 (0.01, 0.02)	**3.36E-07**	0.01 (0.00, 0.03)
-0.01 (-0.03, 0.01)	4.74E-01	-0.02 (-0.02, -0.01)	**9.18E-17**	-0.01 (-0.03, 0.00)
-0.02 (-0.05, 0.00)	8.44E-02	-0.02 (-0.02, -0.01)	**3.61E-15**	0.01 (-0.01, 0.02)
0.02 (0.00, 0.04)	9.52E-02	-0.01 (-0.01, -0.01)	**1.91E-05**	0.00 (-0.01, 0.02)
0.07 (0.05, 0.10)	**5.38E-09**	0.01 (0.01, 0.02)	**7.37E-09**	0.02 (0.00, 0.03)
-0.01 (-0.03, 0.02)	6.75E-01	-0.02 (-0.02, -0.01)	**1.13E-14**	-0.01 (-0.03, 0.00)
-0.03 (-0.06, -0.01)	**2.18E-02**	-0.02 (-0.03, -0.02)	**4.54E-19**	0.00 (-0.01, 0.02)
0.04 (0.01, 0.06)	**2.90E-03**	0.00 (0.00, 0.01)	4.43E-01	0.01 (-0.01, 0.02)
-0.05 (-0.08, -0.03)	**1.68E-04**	-0.03 (-0.03, -0.02)	**6.91E-30**	0.00 (-0.02, 0.01)
0.05 (0.02, 0.07)	**1.57E-04**	0.01 (0.01, 0.02)	**7.54E-07**	0.01 (0.00, 0.03)

er	Never/former drinker		Current drinker	
P value	Adjusted β (95% CI)	P value	Adjusted β (95% CI)	P value
**2.10E-02**	0.03 (0.01, 0.05)	**1.07E-02**	0.02 (0.01, 0.02)	**2.19E-16**
**1.79E-02**	0.03 (0.01, 0.04)	**1.74E-02**	0.02 (0.01, 0.02)	**4.87E-15**
**2.14E-02**	0.03 (0.01, 0.05)	**1.01E-02**	0.02 (0.01, 0.02)	**4.56E-17**
**2.10E-02**	0.03 (0.01, 0.05)	**9.37E-03**	0.02 (0.01, 0.02)	**3.27E-14**
**2.23E-02**	0.03 (0.01, 0.05)	**1.31E-02**	0.02 (0.01, 0.02)	**3.00E-12**
**2.10E-02**	0.03 (0.01, 0.05)	**6.94E-03**	0.02 (0.01, 0.02)	**2.19E-16**
**1.78E-02**	0.02 (0.00, 0.04)	**2.62E-02**	0.02 (0.01, 0.02)	**1.90E-14**
1.02E-01	0.03 (0.01, 0.05)	**4.08E-03**	0.02 (0.01, 0.02)	**5.60E-11**
8.71E-02	0.03 (0.01, 0.05)	**5.47E-03**	0.01 (0.01, 0.02)	**1.07E-10**
8.71E-02	0.03 (0.01, 0.05)	**4.03E-03**	0.01 (0.01, 0.02)	**7.72E-11**
1.88E-01	0.03 (0.01, 0.05)	**3.83E-03**	0.01 (0.01, 0.01)	**6.35E-06**
3.38E-01	0.03 (0.01, 0.05)	**5.55E-03**	0.01 (0.00, 0.01)	**8.46E-03**
8.95E-02	0.03 (0.01, 0.05)	**3.08E-03**	0.01 (0.01, 0.02)	**3.20E-10**
7.24E-02	0.03 (0.01, 0.05)	**8.63E-03**	0.02 (0.01, 0.02)	**3.73E-12**
1.71E-01	0.03 (0.01, 0.05)	**2.86E-03**	0.01 (0.01, 0.02)	**4.57E-09**
1.88E-01	0.03 (0.01, 0.05)	**4.58E-03**	0.01 (0.01, 0.02)	**1.24E-07**
1.61E-01	0.03 (0.01, 0.05)	**2.23E-03**	0.01 (0.01, 0.02)	**1.55E-09**
2.69E-01	0.03 (0.02, 0.05)	**1.90E-03**	0.01 (0.01, 0.01)	**5.06E-06**
3.38E-01	0.03 (0.02, 0.05)	**1.66E-03**	0.01 (0.00, 0.01)	**1.89E-04**
2.16E-01	0.03 (0.01, 0.05)	**2.60E-03**	0.01 (0.01, 0.02)	**1.64E-07**
1.79E-01	0.03 (0.01, 0.05)	**1.26E-02**	0.01 (0.01, 0.02)	**2.27E-07**
7.04E-01	0.03 (0.01, 0.04)	**1.57E-02**	0.00 (-0.01, 0.00)	6.14E-01
5.37E-01	0.03 (0.01, 0.05)	**9.20E-03**	0.00 (0.00, 0.01)	3.87E-01
7.04E-01	0.03 (0.01, 0.04)	**1.79E-02**	0.00 (-0.01, 0.00)	4.55E-01
7.58E-01	0.02 (0.00, 0.04)	1.52E-01	-0.01 (-0.01, -0.01)	**1.76E-06**
5.06E-01	0.01 (-0.01, 0.03)	4.48E-01	-0.02 (-0.02, -0.01)	**1.11E-11**
8.84E-01	0.02 (0.00, 0.04)	**2.71E-02**	0.00 (-0.01, 0.00)	5.98E-02
2.62E-01	0.03 (0.01, 0.05)	**5.32E-03**	0.01 (0.01, 0.01)	**4.50E-06**
2.57E-01	0.04 (0.02, 0.06)	**2.17E-04**	0.01 (0.01, 0.02)	**3.39E-07**
2.77E-01	0.04 (0.02, 0.06)	**4.26E-04**	0.01 (0.01, 0.01)	**2.36E-05**
6.66E-01	0.03 (0.01, 0.05)	**3.95E-03**	0.00 (0.00, 0.01)	6.33E-01
8.54E-01	0.03 (0.01, 0.05)	**5.52E-03**	0.00 (0.00, 0.00)	8.50E-01
7.29E-01	0.03 (0.01, 0.05)	**2.56E-03**	0.00 (0.00, 0.01)	3.01E-01
9.45E-01	0.02 (0.01, 0.04)	**2.53E-02**	-0.01 (-0.01, 0.00)	**1.83E-02**
5.13E-02	0.04 (0.02, 0.06)	**1.29E-04**	0.02 (0.02, 0.02)	**1.18E-19**
2.36E-01	0.03 (0.01, 0.05)	**2.56E-03**	0.01 (0.00, 0.01)	**1.39E-02**
1.49E-01	0.04 (0.02, 0.05)	**1.95E-03**	0.01 (0.00, 0.01)	**8.02E-04**
1.20E-01	0.04 (0.02, 0.06)	**7.72E-04**	0.01 (0.01, 0.02)	**6.12E-07**
4.51E-01	0.02 (0.00, 0.04)	6.44E-02	0.00 (-0.01, 0.00)	2.36E-01
5.35E-01	0.02 (0.00, 0.04)	1.79E-01	-0.01 (-0.01, 0.00)	**1.12E-02**
2.77E-01	0.03 (0.01, 0.05)	**4.48E-03**	0.00 (0.00, 0.01)	5.18E-02
**2.18E-02**	0.05 (0.03, 0.07)	**4.54E-05**	0.02 (0.02, 0.03)	**3.92E-25**
5.16E-01	0.00 (-0.02, 0.02)	7.49E-01	-0.02 (-0.02, -0.01)	**5.74E-13**

5.68E-01	0.00 (-0.02, 0.02)	9.47E-01	-0.02 (-0.02, -0.01)	**3.96E-16**
8.22E-01	0.01 (-0.01, 0.03)	6.07E-01	-0.01 (-0.02, -0.01)	**8.48E-10**
3.20E-01	-0.01 (-0.03, 0.01)	6.33E-01	-0.02 (-0.03, -0.02)	**2.06E-24**
3.36E-01	-0.01 (-0.03, 0.01)	5.78E-01	-0.02 (-0.03, -0.02)	**2.39E-25**
2.83E-01	0.00 (-0.02, 0.02)	8.16E-01	-0.02 (-0.03, -0.02)	**9.11E-21**
**4.90E-02**	0.04 (0.02, 0.06)	**2.17E-04**	0.02 (0.01, 0.02)	**3.00E-17**
5.93E-01	0.01 (-0.01, 0.03)	3.22E-01	-0.01 (-0.02, -0.01)	**6.35E-07**
2.14E-01	0.00 (-0.02, 0.02)	9.58E-01	-0.02 (-0.03, -0.02)	**7.20E-21**
1.79E-01	0.00 (-0.02, 0.02)	9.10E-01	-0.02 (-0.03, -0.02)	**4.47E-24**
1.70E-01	0.00 (-0.02, 0.02)	7.67E-01	-0.02 (-0.03, -0.02)	**3.73E-24**
2.14E-01	0.00 (-0.02, 0.02)	8.99E-01	-0.02 (-0.03, -0.02)	**4.65E-21**
6.96E-02	-0.01 (-0.03, 0.01)	4.14E-01	-0.03 (-0.03, -0.02)	**2.43E-32**
2.03E-01	0.04 (0.02, 0.06)	**1.09E-03**	0.01 (0.01, 0.02)	**2.78E-10**
7.16E-01	0.02 (0.00, 0.04)	9.26E-02	-0.01 (-0.01, -0.01)	**3.82E-06**
2.81E-01	0.01 (-0.01, 0.03)	4.97E-01	-0.02 (-0.02, -0.01)	**2.02E-13**
1.93E-01	0.01 (-0.01, 0.02)	6.74E-01	-0.02 (-0.02, -0.01)	**3.81E-16**
2.53E-01	0.01 (-0.01, 0.03)	5.96E-01	-0.02 (-0.02, -0.01)	**1.20E-15**
4.18E-01	0.01 (-0.01, 0.03)	3.48E-01	-0.01 (-0.02, -0.01)	**6.41E-11**
**4.03E-02**	-0.01 (-0.02, 0.01)	6.74E-01	-0.03 (-0.03, -0.02)	**2.55E-30**
2.22E-01	0.04 (0.02, 0.06)	**1.04E-03**	0.01 (0.01, 0.02)	**4.61E-11**
9.34E-01	0.02 (0.00, 0.04)	5.88E-02	-0.01 (-0.01, 0.00)	**1.25E-03**
4.18E-01	0.01 (-0.01, 0.03)	3.28E-01	-0.01 (-0.02, -0.01)	**3.93E-10**
3.10E-01	0.01 (-0.01, 0.03)	3.75E-01	-0.01 (-0.02, -0.01)	**1.09E-10**
2.89E-01	0.01 (-0.01, 0.03)	5.41E-01	-0.02 (-0.02, -0.01)	**5.29E-14**
5.21E-01	0.01 (-0.01, 0.03)	3.20E-01	-0.01 (-0.02, -0.01)	**3.30E-09**
5.06E-02	0.00 (-0.02, 0.02)	7.92E-01	-0.02 (-0.03, -0.02)	**5.02E-28**
6.55E-02	0.04 (0.02, 0.06)	**9.21E-04**	0.02 (0.01, 0.02)	**1.22E-15**
2.74E-01	0.00 (-0.02, 0.02)	7.92E-01	0.00 (-0.01, 0.00)	4.51E-01
2.35E-01	-0.01 (-0.03, 0.01)	5.67E-01	0.00 (-0.01, 0.00)	6.00E-01
1.62E-01	-0.01 (-0.03, 0.01)	5.74E-01	0.00 (0.00, 0.01)	7.91E-01
5.30E-01	-0.01 (-0.03, 0.01)	3.34E-01	-0.01 (-0.01, 0.00)	**1.41E-02**
5.71E-01	-0.01 (-0.03, 0.01)	2.50E-01	-0.01 (-0.01, 0.00)	**1.60E-03**
3.92E-01	0.00 (-0.02, 0.02)	7.58E-01	0.00 (0.00, 0.01)	8.61E-01
**6.02E-03**	0.04 (0.02, 0.06)	**2.17E-04**	0.02 (0.02, 0.03)	**7.11E-24**
6.09E-01	-0.02 (-0.04, 0.00)	6.07E-02	-0.01 (-0.02, -0.01)	**9.57E-06**
5.65E-01	-0.02 (-0.05, 0.00)	**3.93E-02**	-0.01 (-0.02, -0.01)	**2.20E-05**
5.06E-01	-0.03 (-0.05, 0.00)	**3.08E-02**	-0.01 (-0.01, 0.00)	**9.28E-05**
7.69E-01	-0.03 (-0.05, -0.01)	**2.49E-02**	-0.01 (-0.02, -0.01)	**1.24E-07**
8.67E-01	-0.03 (-0.05, -0.01)	**1.79E-02**	-0.01 (-0.02, -0.01)	**1.11E-08**
4.88E-01	-0.02 (-0.04, 0.00)	7.29E-02	-0.01 (-0.01, 0.00)	**1.59E-04**
**1.01E-02**	0.03 (0.01, 0.05)	**6.76E-03**	0.02 (0.01, 0.02)	**2.02E-17**
9.45E-01	-0.04 (-0.06, -0.02)	**2.02E-03**	-0.02 (-0.03, -0.02)	**1.31E-18**
9.21E-01	-0.04 (-0.06, -0.01)	**3.38E-03**	-0.02 (-0.02, -0.01)	**2.92E-15**
6.61E-01	-0.03 (-0.05, -0.01)	**8.07E-03**	-0.02 (-0.02, -0.01)	**1.03E-10**
6.21E-01	-0.05 (-0.07, -0.02)	**2.19E-04**	-0.03 (-0.03, -0.02)	**2.57E-26**
5.35E-01	-0.05 (-0.07, -0.03)	**1.33E-04**	-0.03 (-0.03, -0.02)	**1.58E-28**
9.89E-01	-0.03 (-0.06, -0.01)	**4.08E-03**	-0.02 (-0.03, -0.02)	**3.05E-17**
**1.78E-02**	0.03 (0.01, 0.05)	**7.13E-03**	0.02 (0.01, 0.02)	**4.92E-16**

5.53E-02	-0.03 (-0.04, -0.01)	**2.00E-02**	-0.03 (-0.03, -0.02)	**4.01E-40**
4.85E-01	-0.02 (-0.04, 0.00)	1.09E-01	-0.02 (-0.02, -0.02)	**5.68E-20**
7.98E-01	-0.02 (-0.04, 0.00)	1.13E-01	-0.02 (-0.02, -0.01)	**8.78E-15**
**3.98E-02**	-0.03 (-0.05, -0.01)	**5.25E-03**	-0.03 (-0.04, -0.03)	**1.05E-45**
**1.53E-02**	-0.04 (-0.06, -0.02)	**1.31E-03**	-0.03 (-0.04, -0.03)	**3.78E-53**
5.28E-01	-0.01 (-0.03, 0.01)	4.17E-01	-0.02 (-0.02, -0.02)	**3.90E-18**
8.24E-02	0.04 (0.02, 0.06)	**1.09E-03**	0.02 (0.01, 0.02)	**7.87E-17**
9.91E-01	0.04 (0.02, 0.06)	**8.81E-04**	0.01 (0.01, 0.02)	**1.58E-10**
2.73E-01	-0.02 (-0.03, 0.00)	1.78E-01	-0.01 (-0.01, -0.01)	**5.63E-06**
3.36E-01	-0.02 (-0.04, 0.00)	1.21E-01	-0.01 (-0.02, -0.01)	**1.64E-06**
2.63E-01	-0.01 (-0.03, 0.01)	6.16E-01	-0.01 (-0.01, 0.00)	**6.46E-03**
3.04E-01	0.00 (-0.02, 0.02)	8.90E-01	0.00 (0.00, 0.01)	**2.97E-02**
1.40E-01	-0.03 (-0.05, -0.01)	**4.55E-03**	-0.01 (-0.02, -0.01)	**1.17E-10**
7.42E-02	-0.04 (-0.06, -0.02)	**8.81E-04**	-0.02 (-0.02, -0.01)	**1.04E-12**
9.45E-01	0.00 (-0.02, 0.02)	8.25E-01	0.00 (-0.01, 0.00)	6.77E-02
2.73E-01	0.02 (0.00, 0.04)	1.56E-01	0.01 (0.01, 0.02)	**3.86E-07**
**3.10E-02**	0.05 (0.03, 0.07)	**9.10E-05**	0.02 (0.01, 0.02)	**9.37E-15**
4.85E-01	0.01 (-0.01, 0.03)	5.32E-01	0.00 (-0.01, 0.00)	**3.94E-02**
3.33E-01	0.00 (-0.02, 0.02)	8.96E-01	-0.01 (-0.01, 0.00)	**2.76E-04**
9.41E-01	0.03 (0.01, 0.05)	**5.52E-03**	0.00 (0.00, 0.01)	**2.78E-02**
6.42E-01	-0.03 (-0.05, -0.01)	**4.97E-03**	-0.01 (-0.01, 0.00)	**5.25E-03**
1.79E-01	-0.02 (-0.04, 0.00)	1.78E-01	-0.02 (-0.03, -0.02)	**2.80E-20**
**3.45E-02**	-0.03 (-0.05, -0.01)	**1.34E-02**	-0.03 (-0.03, -0.02)	**9.89E-31**
**2.88E-02**	-0.03 (-0.05, -0.01)	**6.36E-03**	-0.03 (-0.03, -0.02)	**2.03E-32**
6.55E-02	-0.02 (-0.04, 0.00)	1.17E-01	-0.02 (-0.03, -0.02)	**5.29E-24**
**4.80E-02**	0.03 (0.01, 0.05)	**2.26E-02**	0.03 (0.02, 0.03)	**3.30E-29**
**1.74E-03**	-0.04 (-0.06, -0.02)	**2.17E-04**	-0.04 (-0.04, -0.03)	**1.19E-54**
**1.03E-02**	-0.03 (-0.05, -0.01)	**7.75E-03**	-0.03 (-0.03, -0.02)	**9.93E-33**
5.53E-02	-0.02 (-0.04, 0.00)	1.44E-01	-0.02 (-0.02, -0.01)	**3.80E-15**
**1.64E-03**	-0.04 (-0.06, -0.02)	**2.37E-04**	-0.04 (-0.04, -0.03)	**1.42E-54**
**5.01E-03**	0.03 (0.01, 0.05)	**2.56E-03**	0.03 (0.03, 0.03)	**3.54E-40**
7.03E-02	0.04 (0.02, 0.06)	**1.62E-04**	0.04 (0.03, 0.04)	**4.35E-55**
**1.53E-02**	-0.05 (-0.07, -0.02)	**1.62E-04**	-0.04 (-0.04, -0.03)	**5.63E-52**
**1.99E-02**	-0.05 (-0.07, -0.03)	**1.14E-04**	-0.04 (-0.04, -0.03)	**1.71E-53**
**1.45E-02**	-0.02 (-0.04, 0.00)	1.08E-01	-0.02 (-0.03, -0.02)	**1.80E-22**
**2.10E-02**	0.04 (0.02, 0.06)	**6.13E-04**	0.03 (0.03, 0.04)	**5.48E-41**
**4.27E-03**	0.04 (0.02, 0.06)	**1.09E-03**	0.03 (0.03, 0.04)	**2.61E-49**
**2.52E-04**	-0.05 (-0.07, -0.03)	**4.54E-05**	-0.04 (-0.05, -0.04)	**1.53E-82**
**1.74E-03**	-0.05 (-0.07, -0.03)	**4.53E-05**	-0.04 (-0.05, -0.04)	**1.08E-80**
**3.84E-03**	-0.02 (-0.04, 0.00)	5.61E-02	-0.02 (-0.03, -0.02)	**5.72E-21**
**9.61E-04**	0.05 (0.03, 0.07)	**1.29E-04**	0.04 (0.04, 0.05)	**3.10E-69**
7.89E-01	0.00 (-0.02, 0.02)	9.86E-01	-0.01 (-0.01, 0.00)	**1.20E-02**
**4.91E-03**	-0.04 (-0.06, -0.02)	**1.84E-03**	-0.03 (-0.04, -0.03)	**2.54E-50**
3.42E-01	-0.02 (-0.03, 0.00)	1.78E-01	-0.01 (-0.02, -0.01)	**1.07E-10**
**1.61E-03**	-0.04 (-0.06, -0.02)	**6.15E-04**	-0.03 (-0.04, -0.03)	**3.14E-49**
**2.25E-03**	0.04 (0.02, 0.06)	**9.50E-04**	0.04 (0.03, 0.04)	**1.39E-61**
1.88E-01	-0.02 (-0.04, 0.00)	5.31E-02	-0.01 (-0.01, -0.01)	**2.14E-05**
**3.10E-02**	-0.02 (-0.04, 0.00)	**2.61E-02**	-0.03 (-0.03, -0.02)	**6.95E-38**

3.04E-01	0.02 (0.00, 0.04)	1.07E-01	0.00 (0.00, 0.01)	1.82E-01
**9.61E-04**	-0.04 (-0.06, -0.02)	**2.17E-04**	-0.03 (-0.03, -0.03)	**1.06E-38**
**1.74E-03**	0.04 (0.02, 0.06)	**1.09E-03**	0.04 (0.03, 0.04)	**3.67E-59**
7.14E-01	-0.01 (-0.03, 0.01)	6.02E-01	0.00 (0.00, 0.01)	9.44E-02
**3.65E-03**	-0.03 (-0.05, -0.01)	**1.61E-02**	-0.03 (-0.04, -0.03)	**6.23E-49**
6.87E-01	0.01 (-0.01, 0.03)	6.15E-01	0.00 (-0.01, 0.00)	8.66E-02
**9.61E-04**	-0.03 (-0.05, -0.01)	**2.23E-03**	-0.03 (-0.03, -0.03)	**5.29E-39**
**4.97E-04**	0.04 (0.02, 0.06)	**1.14E-03**	0.03 (0.03, 0.04)	**9.74E-50**
**3.57E-02**	0.00 (-0.02, 0.02)	8.58E-01	0.01 (0.01, 0.02)	**2.58E-10**
**7.14E-06**	-0.02 (-0.04, 0.00)	**4.79E-02**	-0.03 (-0.03, -0.02)	**6.46E-37**
**7.14E-06**	-0.03 (-0.05, -0.01)	**4.70E-03**	-0.04 (-0.04, -0.03)	**1.51E-60**
1.20E-01	0.00 (-0.02, 0.02)	9.08E-01	0.00 (-0.01, 0.00)	2.31E-01
7.14E-02	0.03 (0.01, 0.05)	**4.58E-03**	0.01 (0.01, 0.02)	**1.84E-08**
2.53E-01	0.00 (-0.01, 0.02)	7.34E-01	0.01 (0.01, 0.02)	**2.20E-10**
1.79E-01	-0.02 (-0.04, 0.00)	**2.71E-02**	-0.02 (-0.03, -0.02)	**1.61E-19**
7.62E-02	-0.03 (-0.05, -0.01)	**1.45E-02**	-0.02 (-0.03, -0.02)	**2.17E-25**
4.85E-01	0.00 (-0.02, 0.02)	7.34E-01	0.00 (0.00, 0.01)	8.47E-01
2.72E-01	0.04 (0.02, 0.06)	**1.66E-03**	0.02 (0.02, 0.03)	**8.18E-20**
**4.02E-03**	0.04 (0.02, 0.06)	**1.84E-03**	0.03 (0.03, 0.04)	**7.99E-47**
**9.88E-03**	-0.05 (-0.07, -0.03)	**1.29E-04**	-0.03 (-0.04, -0.03)	**3.61E-47**
**3.94E-03**	-0.05 (-0.06, -0.03)	**1.29E-04**	-0.03 (-0.04, -0.03)	**3.96E-46**
9.34E-01	-0.02 (-0.04, 0.00)	5.31E-02	-0.02 (-0.02, -0.02)	**9.12E-16**
**3.70E-02**	0.05 (0.03, 0.07)	**6.56E-05**	0.03 (0.03, 0.03)	**2.32E-40**
**1.82E-03**	0.00 (-0.02, 0.02)	9.16E-01	0.02 (0.01, 0.02)	**6.94E-14**
**2.52E-04**	-0.03 (-0.05, -0.02)	**1.89E-03**	-0.03 (-0.04, -0.03)	**1.30E-48**
**2.96E-04**	-0.04 (-0.06, -0.02)	**5.52E-04**	-0.03 (-0.04, -0.03)	**1.98E-49**
9.45E-01	0.02 (0.00, 0.04)	1.46E-01	0.00 (0.00, 0.01)	6.20E-01
**2.12E-02**	0.05 (0.03, 0.07)	**1.65E-05**	0.03 (0.03, 0.04)	**4.44E-47**
2.02E-01	0.03 (0.01, 0.05)	**2.00E-02**	0.01 (0.01, 0.02)	**4.89E-07**
3.28E-01	-0.02 (-0.04, 0.00)	**2.81E-02**	-0.01 (-0.02, -0.01)	**1.34E-06**
1.42E-01	-0.01 (-0.03, 0.01)	2.83E-01	0.00 (0.00, 0.01)	8.47E-01
4.72E-01	0.00 (-0.02, 0.02)	7.80E-01	-0.02 (-0.03, -0.02)	**9.20E-23**
3.92E-01	0.03 (0.01, 0.05)	**6.74E-03**	0.00 (0.00, 0.01)	2.85E-01
1.89E-01	0.00 (-0.02, 0.02)	9.97E-01	-0.02 (-0.03, -0.02)	**4.12E-21**
9.68E-01	0.01 (0.00, 0.03)	2.04E-01	-0.01 (-0.01, -0.01)	**1.04E-05**
3.46E-01	-0.01 (-0.03, 0.01)	5.48E-01	-0.02 (-0.03, -0.02)	**5.34E-27**
7.69E-01	0.01 (-0.01, 0.03)	6.74E-01	-0.02 (-0.02, -0.01)	**3.07E-13**
5.53E-02	0.03 (0.01, 0.05)	**2.25E-03**	0.02 (0.01, 0.02)	**1.12E-13**
6.68E-02	0.03 (0.01, 0.05)	**4.02E-03**	0.02 (0.01, 0.02)	**3.33E-12**
1.79E-01	0.04 (0.02, 0.06)	**9.50E-04**	0.02 (0.01, 0.02)	**2.25E-11**
**1.79E-02**	0.03 (0.02, 0.05)	**1.95E-03**	0.02 (0.02, 0.02)	**2.50E-19**
4.85E-01	0.00 (-0.02, 0.02)	9.86E-01	-0.01 (-0.02, -0.01)	**3.29E-06**
**3.70E-02**	0.04 (0.02, 0.06)	**2.70E-04**	0.03 (0.02, 0.03)	**7.07E-27**
5.35E-01	0.00 (-0.02, 0.02)	9.08E-01	-0.01 (-0.02, -0.01)	**5.68E-08**
5.77E-01	0.00 (-0.03, 0.02)	7.39E-01	-0.02 (-0.03, -0.02)	**1.92E-19**
7.69E-01	0.00 (-0.02, 0.02)	8.12E-01	-0.02 (-0.02, -0.01)	**1.07E-10**
9.35E-01	-0.03 (-0.06, -0.01)	**4.78E-03**	-0.02 (-0.03, -0.02)	**4.30E-19**
7.53E-01	0.02 (0.00, 0.03)	1.59E-01	-0.01 (-0.01, -0.01)	**8.06E-06**

6.31E-01	0.03 (0.01, 0.05)	**1.26E-02**	0.00 (0.00, 0.01)	5.30E-01
1.79E-01	0.02 (0.00, 0.04)	**2.62E-02**	0.00 (0.00, 0.01)	1.26E-01
9.45E-01	0.01 (-0.01, 0.03)	5.19E-01	-0.01 (-0.01, 0.00)	**1.96E-02**
**4.97E-04**	0.04 (0.02, 0.06)	**2.17E-04**	0.01 (0.01, 0.02)	**1.68E-06**
6.31E-01	0.02 (0.00, 0.03)	1.79E-01	-0.01 (-0.02, -0.01)	**7.63E-07**
**1.22E-05**	0.05 (0.03, 0.07)	**4.54E-05**	0.02 (0.01, 0.02)	**4.87E-15**
6.49E-01	0.01 (-0.01, 0.03)	2.87E-01	-0.01 (-0.02, -0.01)	**1.64E-07**
5.72E-01	0.02 (0.00, 0.04)	**2.94E-02**	-0.01 (-0.01, 0.00)	**2.10E-02**
1.50E-01	0.03 (0.01, 0.05)	**1.39E-02**	0.01 (0.01, 0.01)	**3.42E-06**
8.89E-02	0.02 (0.00, 0.04)	1.08E-01	0.00 (0.00, 0.01)	6.77E-02
**4.38E-03**	0.03 (0.01, 0.05)	**6.71E-03**	0.01 (0.01, 0.01)	**9.72E-06**
**4.97E-04**	-0.02 (-0.04, 0.00)	1.37E-01	-0.03 (-0.03, -0.02)	**4.65E-29**
**2.07E-06**	0.04 (0.03, 0.06)	**1.29E-04**	0.02 (0.02, 0.02)	**9.20E-18**
**1.15E-04**	-0.03 (-0.05, -0.01)	**2.59E-03**	-0.03 (-0.03, -0.02)	**1.77E-28**
**3.70E-02**	-0.01 (-0.03, 0.01)	2.44E-01	-0.02 (-0.02, -0.01)	**9.88E-13**
1.20E-01	0.03 (0.01, 0.05)	**5.53E-03**	0.02 (0.02, 0.03)	**4.55E-20**
8.89E-02	-0.01 (-0.03, 0.01)	2.83E-01	0.00 (0.00, 0.01)	2.17E-01
8.56E-02	0.03 (0.01, 0.05)	**5.33E-03**	0.01 (0.01, 0.02)	**3.54E-11**
2.77E-01	0.00 (-0.02, 0.02)	9.83E-01	0.00 (0.00, 0.01)	1.39E-01
2.14E-01	-0.01 (-0.03, 0.01)	4.89E-01	0.00 (-0.01, 0.00)	5.78E-01
2.35E-01	0.03 (0.01, 0.05)	**6.92E-03**	0.01 (0.00, 0.01)	**7.23E-05**
**4.34E-05**	-0.02 (-0.04, 0.00)	**3.37E-02**	-0.03 (-0.03, -0.02)	**2.78E-31**
9.31E-01	0.01 (-0.01, 0.03)	3.73E-01	0.00 (-0.01, 0.00)	4.65E-01
5.35E-01	0.03 (0.01, 0.05)	**4.65E-03**	0.00 (0.00, 0.01)	7.39E-02
6.31E-01	0.02 (0.00, 0.04)	**4.32E-02**	0.00 (0.00, 0.01)	2.14E-01
6.65E-01	0.04 (0.02, 0.06)	**1.09E-03**	0.01 (0.01, 0.02)	**9.84E-10**
3.68E-01	0.01 (-0.01, 0.02)	6.92E-01	0.00 (0.00, 0.01)	**3.02E-02**
2.01E-01	0.00 (-0.02, 0.02)	9.95E-01	0.00 (0.00, 0.00)	8.14E-01
5.76E-01	0.00 (-0.02, 0.01)	7.43E-01	-0.01 (-0.01, 0.00)	**2.44E-03**
4.71E-01	0.01 (-0.01, 0.03)	6.15E-01	0.00 (0.00, 0.01)	1.75E-01
7.14E-01	0.00 (-0.02, 0.02)	7.92E-01	0.01 (0.00, 0.01)	**2.23E-02**
6.90E-02	0.05 (0.03, 0.07)	**1.53E-04**	0.03 (0.02, 0.03)	**2.93E-29**
8.54E-01	-0.01 (-0.03, 0.01)	6.00E-01	-0.01 (-0.01, 0.00)	**2.12E-02**
9.31E-01	0.05 (0.03, 0.07)	**1.29E-04**	0.02 (0.01, 0.02)	**1.17E-12**
**2.79E-02**	-0.02 (-0.03, 0.00)	1.91E-01	-0.02 (-0.02, -0.01)	**5.78E-11**
9.35E-01	0.03 (0.01, 0.05)	**3.57E-03**	0.01 (0.00, 0.01)	**1.34E-04**
5.28E-01	0.01 (-0.01, 0.03)	5.74E-01	-0.02 (-0.02, -0.01)	**9.88E-13**
1.40E-01	0.00 (-0.02, 0.02)	8.58E-01	-0.02 (-0.03, -0.02)	**6.20E-25**
2.74E-01	0.00 (-0.02, 0.02)	8.16E-01	-0.02 (-0.02, -0.01)	**2.98E-17**
5.62E-01	-0.04 (-0.07, -0.02)	**3.95E-04**	-0.03 (-0.03, -0.02)	**2.50E-27**
6.68E-02	-0.03 (-0.05, -0.01)	**1.41E-02**	-0.02 (-0.02, -0.01)	**1.76E-16**
**1.80E-06**	-0.05 (-0.07, -0.03)	**1.14E-04**	-0.02 (-0.03, -0.02)	**2.08E-27**
2.74E-01	0.03 (0.01, 0.05)	**3.57E-03**	0.01 (0.00, 0.01)	**6.25E-03**
5.53E-02	-0.01 (-0.03, 0.01)	5.19E-01	-0.03 (-0.03, -0.02)	**6.31E-32**
7.98E-01	-0.03 (-0.05, -0.01)	**2.61E-02**	-0.02 (-0.02, -0.01)	**2.76E-12**
9.66E-01	0.01 (-0.01, 0.03)	5.10E-01	0.00 (0.00, 0.01)	7.39E-02
7.72E-01	-0.01 (-0.03, 0.01)	5.46E-01	0.00 (-0.01, 0.00)	5.98E-02
3.68E-01	-0.03 (-0.05, -0.01)	**6.94E-03**	-0.03 (-0.03, -0.02)	**1.78E-35**

2.61E-01	0.04 (0.02, 0.06)	**1.20E-03**	0.01 (0.00, 0.01)	**6.89E-04**
6.61E-01	0.01 (0.00, 0.03)	1.83E-01	-0.01 (-0.01, -0.01)	**1.77E-06**
3.89E-01	-0.04 (-0.06, -0.02)	**2.13E-03**	-0.03 (-0.03, -0.02)	**3.09E-34**
6.57E-01	0.00 (-0.02, 0.02)	9.86E-01	-0.01 (-0.02, -0.01)	**7.01E-10**
1.85E-01	0.03 (0.01, 0.05)	**2.08E-03**	0.01 (0.01, 0.01)	**2.33E-05**
1.89E-01	0.00 (-0.02, 0.02)	8.78E-01	-0.02 (-0.02, -0.02)	**3.43E-20**
6.06E-01	-0.03 (-0.05, -0.01)	**1.04E-02**	-0.02 (-0.02, -0.01)	**1.95E-10**
7.02E-01	0.01 (-0.01, 0.03)	4.32E-01	-0.01 (-0.01, -0.01)	**5.14E-06**
1.20E-01	0.03 (0.01, 0.05)	**2.54E-03**	0.01 (0.01, 0.02)	**3.78E-07**
2.50E-01	0.00 (-0.02, 0.02)	8.16E-01	-0.02 (-0.02, -0.01)	**9.02E-18**
8.07E-01	-0.03 (-0.05, -0.01)	**6.73E-03**	-0.02 (-0.02, -0.01)	**4.94E-14**
5.29E-01	0.03 (0.01, 0.05)	**1.39E-02**	0.00 (0.00, 0.00)	8.61E-01
6.88E-01	-0.04 (-0.06, -0.02)	**1.05E-03**	-0.02 (-0.03, -0.02)	**7.25E-24**
2.89E-01	0.04 (0.02, 0.06)	**2.70E-04**	0.01 (0.00, 0.01)	**2.08E-04**

Supplementary Table 8. Associati

		CKB
		Male
**Metabolites**	**Group**	**Adjusted HR (95% CI)**
Concentration of chylomicrons and extremely l	Lipoprotein subclasses	0.92 (0.86, 0.99)
Total lipids in chylomicrons and extremely larg	Lipoprotein subclasses	0.93 (0.87, 0.99)
Phospholipids in chylomicrons and extremely l	Lipoprotein subclasses	0.94 (0.88, 1.00)
Cholesterol in chylomicrons and extremely larg	Lipoprotein subclasses	0.96 (0.90, 1.03)
Cholesteryl esters in chylomicrons and extreme	Lipoprotein subclasses	0.98 (0.92, 1.04)
Free cholesterol in chylomicrons and extremely	Lipoprotein subclasses	0.95 (0.89, 1.01)
Triglycerides in chylomicrons and extremely la	Lipoprotein subclasses	0.91 (0.85, 0.98)
Concentration of very large VLDL particles	Lipoprotein subclasses	0.94 (0.88, 1.01)
Total lipids in very large VLDL	Lipoprotein subclasses	0.95 (0.88, 1.01)
Phospholipids in very large VLDL	Lipoprotein subclasses	0.95 (0.89, 1.02)
Cholesterol in very large VLDL	Lipoprotein subclasses	0.96 (0.90, 1.03)
Cholesteryl esters in very large VLDL	Lipoprotein subclasses	0.96 (0.90, 1.03)
Free cholesterol in very large VLDL	Lipoprotein subclasses	0.96 (0.90, 1.03)
Triglycerides in very large VLDL	Lipoprotein subclasses	0.94 (0.88, 1.00)
Concentration of large VLDL particles	Lipoprotein subclasses	0.94 (0.88, 1.01)
Total lipids in large VLDL	Lipoprotein subclasses	0.94 (0.88, 1.01)
Phospholipids in large VLDL	Lipoprotein subclasses	0.95 (0.89, 1.01)
Cholesterol in large VLDL	Lipoprotein subclasses	0.95 (0.89, 1.01)
Cholesteryl esters in large VLDL	Lipoprotein subclasses	0.94 (0.88, 1.00)
Free cholesterol in large VLDL	Lipoprotein subclasses	0.95 (0.89, 1.02)
Triglycerides in large VLDL	Lipoprotein subclasses	0.94 (0.88, 1.01)
Concentration of medium VLDL particles	Lipoprotein subclasses	0.94 (0.88, 1.00)
Total lipids in medium VLDL	Lipoprotein subclasses	0.94 (0.88, 1.00)
Phospholipids in medium VLDL	Lipoprotein subclasses	0.94 (0.88, 1.00)
Cholesterol in medium VLDL	Lipoprotein subclasses	0.95 (0.89, 1.01)
Cholesteryl esters in medium VLDL	Lipoprotein subclasses	0.96 (0.90, 1.02)
Free cholesterol in medium VLDL	Lipoprotein subclasses	0.95 (0.89, 1.01)
Triglycerides in medium VLDL	Lipoprotein subclasses	0.93 (0.88, 1.00)
Concentration of small VLDL particles	Lipoprotein subclasses	0.95 (0.89, 1.01)
Total lipids in small VLDL	Lipoprotein subclasses	0.96 (0.90, 1.02)
Phospholipids in small VLDL	Lipoprotein subclasses	0.95 (0.89, 1.01)
Cholesterol in small VLDL	Lipoprotein subclasses	1.02 (0.96, 1.09)
Cholesteryl esters in small VLDL	Lipoprotein subclasses	1.05 (0.98, 1.11)
Free cholesterol in small VLDL	Lipoprotein subclasses	0.98 (0.93, 1.05)
Triglycerides in small VLDL	Lipoprotein subclasses	0.93 (0.87, 0.99)
Concentration of very small VLDL particles	Lipoprotein subclasses	1.05 (0.99, 1.12)
Total lipids in very small VLDL	Lipoprotein subclasses	1.06 (1.00, 1.13)
Phospholipids in very small VLDL	Lipoprotein subclasses	1.03 (0.96, 1.09)
Cholesterol in very small VLDL	Lipoprotein subclasses	1.11 (1.05, 1.18)
Cholesteryl esters in very small VLDL	Lipoprotein subclasses	1.12 (1.06, 1.19)

Free cholesterol in very small VLDL	Lipoprotein subclasses	1.08 (1.02, 1.15)
Triglycerides in very small VLDL	Lipoprotein subclasses	0.98 (0.93, 1.05)
Concentration of IDL particles	Lipoprotein subclasses	1.02 (0.96, 1.09)
Total lipids in IDL	Lipoprotein subclasses	1.02 (0.96, 1.09)
Phospholipids in IDL	Lipoprotein subclasses	1.01 (0.95, 1.07)
Cholesterol in IDL	Lipoprotein subclasses	1.02 (0.96, 1.08)
Cholesteryl esters in IDL	Lipoprotein subclasses	1.01 (0.95, 1.07)
Free cholesterol in IDL	Lipoprotein subclasses	1.04 (0.98, 1.11)
Triglycerides in IDL	Lipoprotein subclasses	1.07 (1.00, 1.13)
Concentration of large LDL particles	Lipoprotein subclasses	1.00 (0.94, 1.06)
Total lipids in large LDL	Lipoprotein subclasses	1.00 (0.94, 1.06)
Phospholipids in large LDL	Lipoprotein subclasses	0.98 (0.92, 1.04)
Cholesterol in large LDL	Lipoprotein subclasses	0.99 (0.93, 1.06)
Cholesteryl esters in large LDL	Lipoprotein subclasses	0.99 (0.93, 1.05)
Free cholesterol in large LDL	Lipoprotein subclasses	1.01 (0.95, 1.08)
Triglycerides in large LDL	Lipoprotein subclasses	1.10 (1.04, 1.17)
Concentration of medium LDL particles	Lipoprotein subclasses	0.96 (0.90, 1.02)
Total lipids in medium LDL	Lipoprotein subclasses	0.96 (0.90, 1.02)
Phospholipids in medium LDL	Lipoprotein subclasses	0.91 (0.86, 0.97)
Cholesterol in medium LDL	Lipoprotein subclasses	0.96 (0.90, 1.03)
Cholesteryl esters in medium LDL	Lipoprotein subclasses	0.97 (0.91, 1.03)
Free cholesterol in medium LDL	Lipoprotein subclasses	0.95 (0.89, 1.01)
Triglycerides in medium LDL	Lipoprotein subclasses	1.07 (1.00, 1.13)
Concentration of small LDL particles	Lipoprotein subclasses	0.95 (0.89, 1.01)
Total lipids in small LDL	Lipoprotein subclasses	0.95 (0.89, 1.01)
Phospholipids in small LDL	Lipoprotein subclasses	0.91 (0.85, 0.96)
Cholesterol in small LDL	Lipoprotein subclasses	0.97 (0.91, 1.03)
Cholesteryl esters in small LDL	Lipoprotein subclasses	0.97 (0.92, 1.04)
Free cholesterol in small LDL	Lipoprotein subclasses	0.94 (0.88, 1.00)
Triglycerides in small LDL	Lipoprotein subclasses	1.00 (0.94, 1.07)
Concentration of very large HDL particles	Lipoprotein subclasses	1.20 (1.14, 1.26)
Total lipids in very large HDL	Lipoprotein subclasses	1.20 (1.13, 1.26)
Phospholipids in very large HDL	Lipoprotein subclasses	1.20 (1.13, 1.26)
Cholesterol in very large HDL	Lipoprotein subclasses	1.18 (1.12, 1.25)
Cholesteryl esters in very large HDL	Lipoprotein subclasses	1.18 (1.12, 1.25)
Free cholesterol in very large HDL	Lipoprotein subclasses	1.18 (1.12, 1.24)
Triglycerides in very large HDL	Lipoprotein subclasses	1.14 (1.08, 1.21)
Concentration of large HDL particles	Lipoprotein subclasses	1.15 (1.09, 1.22)
Total lipids in large HDL	Lipoprotein subclasses	1.15 (1.09, 1.22)
Phospholipids in large HDL	Lipoprotein subclasses	1.15 (1.08, 1.21)
Cholesterol in large HDL	Lipoprotein subclasses	1.15 (1.09, 1.22)
Cholesteryl esters in large HDL	Lipoprotein subclasses	1.15 (1.09, 1.22)
Free cholesterol in large HDL	Lipoprotein subclasses	1.16 (1.10, 1.23)
Triglycerides in large HDL	Lipoprotein subclasses	1.17 (1.10, 1.23)
**Concentration of medium HDL particles**	Lipoprotein subclasses	0.92 (0.86, 0.98)
**Total lipids in medium HDL**	Lipoprotein subclasses	0.92 (0.86, 0.98)
**Phospholipids in medium HDL**	Lipoprotein subclasses	0.96 (0.90, 1.02)
**Cholesterol in medium HDL**	Lipoprotein subclasses	0.90 (0.85, 0.96)

**Cholesteryl esters in medium HDL**	Lipoprotein subclasses	0.89 (0.84, 0.95)
**Free cholesterol in medium HDL**	Lipoprotein subclasses	0.95 (0.89, 1.02)
Triglycerides in medium HDL	Lipoprotein subclasses	0.91 (0.85, 0.97)
Concentration of small HDL particles	Lipoprotein subclasses	0.84 (0.79, 0.88)
Total lipids in small HDL	Lipoprotein subclasses	0.84 (0.79, 0.88)
Phospholipids in small HDL	Lipoprotein subclasses	0.86 (0.81, 0.92)
Cholesterol in small HDL	Lipoprotein subclasses	0.90 (0.84, 0.95)
Cholesteryl esters in small HDL	Lipoprotein subclasses	0.92 (0.87, 0.97)
**Free cholesterol in small HDL**	Lipoprotein subclasses	0.88 (0.82, 0.93)
Triglycerides in small HDL	Lipoprotein subclasses	0.92 (0.87, 0.98)
Phospholipids to total lipids ratio in chylomicr	Relative lipoprotein lipi	0.95 (0.89, 1.00)
Cholesterol to total lipids ratio in chylomicrons	Relative lipoprotein lipi	1.17 (1.12, 1.23)
Cholesteryl esters to total lipids ratio in chylon	Relative lipoprotein lipi	1.18 (1.12, 1.23)
Free cholesterol to total lipids ratio in chylomi	Relative lipoprotein lipi	1.06 (1.00, 1.13)
Triglycerides to total lipids ratio in chylomicro	Relative lipoprotein lipi	0.87 (0.82, 0.93)
Phospholipids to total lipids ratio in very large	Relative lipoprotein lipi	0.98 (0.92, 1.04)
**Cholesterol to total lipids ratio in very large**	Relative lipoprotein lipi	1.11 (1.07, 1.16)
Cholesteryl esters to total lipids ratio in very la	Relative lipoprotein lipi	1.14 (1.08, 1.21)
**Free cholesterol to total lipids ratio in very l**	Relative lipoprotein lipi	1.04 (0.99, 1.11)
**Triglycerides to total lipids ratio in very lar**	Relative lipoprotein lipi	0.91 (0.85, 0.96)
Phospholipids to total lipids ratio in large VLD	Relative lipoprotein lipi	0.97 (0.92, 1.03)
Cholesterol to total lipids ratio in large VLDL	Relative lipoprotein lipi	0.99 (0.93, 1.05)
Cholesteryl esters to total lipids ratio in large V	Relative lipoprotein lipi	1.03 (0.96, 1.09)
Free cholesterol to total lipids ratio in large VI	Relative lipoprotein lipi	0.97 (0.91, 1.02)
Triglycerides to total lipids ratio in large VLDI	Relative lipoprotein lipi	1.02 (0.96, 1.08)
Phospholipids to total lipids ratio in medium V	Relative lipoprotein lipi	0.98 (0.92, 1.04)
**Cholesterol to total lipids ratio in medium V**	Relative lipoprotein lipi	1.07 (1.01, 1.14)
**Cholesteryl esters to total lipids ratio in mec**	Relative lipoprotein lipi	1.07 (1.00, 1.13)
Free cholesterol to total lipids ratio in medium	Relative lipoprotein lipi	1.01 (0.95, 1.07)
Triglycerides to total lipids ratio in medium VI	Relative lipoprotein lipi	0.94 (0.89, 1.00)
**Phospholipids to total lipids ratio in small V**	Relative lipoprotein lipi	0.97 (0.92, 1.03)
Cholesterol to total lipids ratio in small VLDL	Relative lipoprotein lipi	1.13 (1.06, 1.20)
Cholesteryl esters to total lipids ratio in small V	Relative lipoprotein lipi	1.12 (1.05, 1.18)
**Free cholesterol to total lipids ratio in small**	Relative lipoprotein lipi	1.16 (1.09, 1.23)
Triglycerides to total lipids ratio in small VLD	Relative lipoprotein lipi	0.89 (0.84, 0.95)
Phospholipids to total lipids ratio in very small	Relative lipoprotein lipi	0.93 (0.88, 0.98)
Cholesterol to total lipids ratio in very small V	Relative lipoprotein lipi	1.15 (1.08, 1.22)
Cholesteryl esters to total lipids ratio in very sr	Relative lipoprotein lipi	1.15 (1.08, 1.22)
Free cholesterol to total lipids ratio in very sma	Relative lipoprotein lipi	1.07 (1.00, 1.13)
Triglycerides to total lipids ratio in very small	Relative lipoprotein lipi	0.93 (0.88, 0.99)
Phospholipids to total lipids ratio in IDL	Relative lipoprotein lipi	0.93 (0.87, 0.98)
Cholesterol to total lipids ratio in IDL	Relative lipoprotein lipi	0.98 (0.93, 1.03)
Cholesteryl esters to total lipids ratio in IDL	Relative lipoprotein lipi	0.94 (0.89, 0.99)
Free cholesterol to total lipids ratio in IDL	Relative lipoprotein lipi	1.04 (0.98, 1.11)
Triglycerides to total lipids ratio in IDL	Relative lipoprotein lipi	1.05 (0.99, 1.11)
Phospholipids to total lipids ratio in large LDL	Relative lipoprotein lipi	0.99 (0.93, 1.05)
Cholesterol to total lipids ratio in large LDL	Relative lipoprotein lipi	0.95 (0.90, 1.00)
Cholesteryl esters to total lipids ratio in large L	Relative lipoprotein lipi	0.93 (0.88, 0.98)

Free cholesterol to total lipids ratio in large LD	Relative lipoprotein lipi	1.08 (1.01, 1.15)
Triglycerides to total lipids ratio in large LDL	Relative lipoprotein lipi	1.09 (1.04, 1.15)
Phospholipids to total lipids ratio in medium L	Relative lipoprotein lipi	0.99 (0.93, 1.05)
Cholesterol to total lipids ratio in medium LDI	Relative lipoprotein lipi	0.97 (0.91, 1.03)
Cholesteryl esters to total lipids ratio in mediu	Relative lipoprotein lipi	0.97 (0.91, 1.02)
Free cholesterol to total lipids ratio in medium	Relative lipoprotein lipi	1.04 (0.98, 1.11)
Triglycerides to total lipids ratio in medium LI	Relative lipoprotein lipi	1.11 (1.05, 1.18)
Phospholipids to total lipids ratio in small LDI	Relative lipoprotein lipi	0.99 (0.93, 1.05)
Cholesterol to total lipids ratio in small LDL	Relative lipoprotein lipi	0.99 (0.93, 1.05)
Cholesteryl esters to total lipids ratio in small I	Relative lipoprotein lipi	0.98 (0.93, 1.05)
Free cholesterol to total lipids ratio in small LI	Relative lipoprotein lipi	1.03 (0.96, 1.09)
Triglycerides to total lipids ratio in small LDL	Relative lipoprotein lipi	1.05 (0.99, 1.12)
Phospholipids to total lipids ratio in very large	Relative lipoprotein lipi	1.05 (0.98, 1.11)
Cholesterol to total lipids ratio in very large HI	Relative lipoprotein lipi	0.97 (0.91, 1.03)
Cholesteryl esters to total lipids ratio in very la	Relative lipoprotein lipi	0.96 (0.90, 1.02)
Free cholesterol to total lipids ratio in very larg	Relative lipoprotein lipi	1.02 (0.96, 1.08)
Triglycerides to total lipids ratio in very large l	Relative lipoprotein lipi	0.95 (0.90, 1.01)
Phospholipids to total lipids ratio in large HDL	Relative lipoprotein lipi	0.86 (0.80, 0.91)
Cholesterol to total lipids ratio in large HDL	Relative lipoprotein lipi	1.12 (1.06, 1.20)
Cholesteryl esters to total lipids ratio in large H	Relative lipoprotein lipi	1.08 (1.01, 1.15)
Free cholesterol to total lipids ratio in large HD	Relative lipoprotein lipi	1.16 (1.09, 1.24)
Triglycerides to total lipids ratio in large HDL	Relative lipoprotein lipi	1.03 (0.97, 1.09)
Phospholipids to total lipids ratio in medium H	Relative lipoprotein lipi	1.23 (1.18, 1.29)
Cholesterol to total lipids ratio in medium HDI	Relative lipoprotein lipi	0.87 (0.82, 0.92)
Cholesteryl esters to total lipids ratio in mediu	Relative lipoprotein lipi	0.86 (0.81, 0.91)
Free cholesterol to total lipids ratio in medium	Relative lipoprotein lipi	0.98 (0.92, 1.04)
Triglycerides to total lipids ratio in medium HI	Relative lipoprotein lipi	0.98 (0.92, 1.04)
Phospholipids to total lipids ratio in small HDI	Relative lipoprotein lipi	0.98 (0.93, 1.05)
Cholesterol to total lipids ratio in small HDL	Relative lipoprotein lipi	1.02 (0.95, 1.08)
Cholesteryl esters to total lipids ratio in small I	Relative lipoprotein lipi	1.01 (0.95, 1.08)
Free cholesterol to total lipids ratio in small HI	Relative lipoprotein lipi	1.02 (0.96, 1.09)
Triglycerides to total lipids ratio in small HDL	Relative lipoprotein lipi	0.99 (0.93, 1.06)
Average diameter for VLDL particles	Lipoprotein particle size	0.91 (0.86, 0.97)
Average diameter for LDL particles	Lipoprotein particle size	1.19 (1.12, 1.26)
Average diameter for HDL particles	Lipoprotein particle size	1.20 (1.13, 1.27)
Total cholesterol	Cholesterol	1.03 (0.97, 1.09)
VLDL cholesterol	Cholesterol	1.00 (0.94, 1.06)
Remnant cholesterol (non-HDL, non-LDL -cho	Cholesterol	1.01 (0.95, 1.07)
LDL cholesterol	Cholesterol	0.98 (0.92, 1.05)
**HDL cholesterol**	Cholesterol	1.09 (1.02, 1.15)
**HDL2 cholesterol**	Cholesterol	1.07 (1.01, 1.14)
HDL3 cholesterol	Cholesterol	1.16 (1.09, 1.23)
Total esterified cholesterol	Cholesteryl esters	0.94 (0.88, 1.00)
Total free cholesterol	Free cholesterol	0.91 (0.85, 0.97)
Total triglycerides	Triglycerides	0.95 (0.90, 1.02)
Triglycerides in VLDL	Triglycerides	0.94 (0.88, 1.00)
Triglycerides in LDL	Triglycerides	1.08 (1.02, 1.15)
Triglycerides in HDL	Triglycerides	1.02 (0.96, 1.08)

Phosphoglycerides	Other lipids	0.96 (0.90, 1.02)
Ratio of triglycerides to phosphoglycerides	Other lipids	0.94 (0.88, 1.00)
Phosphatidylcholines	Other lipids	0.86 (0.81, 0.92)
Sphingomyelins	Other lipids	0.86 (0.80, 0.91)
Total cholines	Other lipids	0.94 (0.88, 1.00)
Apolipoprotein A1	Apolipoproteins	1.07 (1.01, 1.14)
Apolipoprotein B	Apolipoproteins	0.97 (0.91, 1.03)
Ratio of apolipoprotein B to apolipoprotein A1	Apolipoproteins	0.94 (0.88, 1.00)
Total fatty acids	Fatty acids	0.89 (0.83, 0.95)
Degree of unsaturation	Fatty acids	0.78 (0.74, 0.82)
Docosahexaenoic acid	Fatty acids	1.06 (1.00, 1.13)
Linoleic acid	Fatty acids	0.85 (0.80, 0.91)
Omega-3 fatty acids	Fatty acids	0.95 (0.89, 1.01)
Omega-6 fatty acids	Fatty acids	0.85 (0.79, 0.90)
Polyunsaturated fatty acids	Fatty acids	0.85 (0.80, 0.91)
Monounsaturated fatty acids	Fatty acids	0.92 (0.86, 0.98)
Saturated fatty acids	Fatty acids	0.91 (0.85, 0.97)
Ratio of docosahexaenoic acid to total fatty aci	Fatty acids	1.18 (1.11, 1.25)
Ratio of linoleic acid to total fatty acids	Fatty acids	0.86 (0.81, 0.91)
Ratio of omega-3 fatty acids to total fatty acids	Fatty acids	1.04 (0.98, 1.10)
Ratio of omega-6 fatty acids to total fatty acids	Fatty acids	0.88 (0.83, 0.93)
Ratio of polyunsaturated fatty acids to total fat	Fatty acids	0.88 (0.83, 0.94)
Ratio of monounsaturated fatty acids to total fa	Fatty acids	1.05 (0.99, 1.12)
Ratio of saturated fatty acids to total fatty acid	Fatty acids	1.14 (1.07, 1.21)
Glucose	Glycolysis related metab	1.14 (1.08, 1.20)
Lactate	Glycolysis related metab	0.97 (0.91, 1.03)
Citrate	Glycolysis related metab	1.02 (0.96, 1.09)
Alanine	Amino acids	0.89 (0.83, 0.94)
Glutamine	Amino acids	0.87 (0.82, 0.92)
Histidine	Amino acids	0.96 (0.91, 1.02)
Isoleucine	Amino acids	0.92 (0.86, 0.98)
Leucine	Amino acids	0.95 (0.90, 1.02)
Valine	Amino acids	0.82 (0.77, 0.87)
Phenylalanine	Amino acids	1.13 (1.08, 1.17)
Tyrosine	Amino acids	1.05 (0.98, 1.11)
Acetate	Ketone bodies	1.10 (1.04, 1.15)
Acetoacetate	Ketone bodies	1.05 (0.99, 1.11)
**3-Hydroxybutyrate**	Ketone bodies	1.09 (1.03, 1.14)
Creatinine	Fluid balance	1.03 (0.99, 1.08)
Albumin	Fluid balance	0.84 (0.80, 0.89)
Glycoprotein acetyls	Inflammation	1.12 (1.05, 1.19)
Acetone	Ketone bodies	
Cholesteryl esters in HDL	Cholesteryl esters	
Cholesteryl esters in LDL	Cholesteryl esters	
Cholesteryl esters in VLDL/ (Cholesterol in ch	Cholesteryl esters	
Clinical LDL cholesterol	Cholesterol	
Concentration of HDL particles	Lipoprotein particle concentrations	
Concentration of LDL particles	Lipoprotein particle concentrations	

Concentration of VLDL particles	Lipoprotein particle concentrations
Free cholesterol in HDL	Free cholesterol
Free cholesterol in LDL	Free cholesterol
Free cholesterol in VLDL	Free cholesterol
Glycine	Amino acids
Phospholipids in HDL	Phospholipids
Phospholipids in LDL	Phospholipids
Phospholipids in VLDL	Phospholipids
Pyruvate	Glycolysis related metabolites
Ratio of omega-6 fatty acids to omega-3 fatty a	Fatty acids
Ratio of polyunsaturated fatty acids to monoun	Fatty acids
Total cholesterol minus HDL-C	Cholesterol
Total concentration of branched-chain amino a	Amino acids
Total concentration of lipoprotein particles	Lipoprotein particle concentrations
Total lipids in HDL	Total lipids
Total lipids in LDL	Total lipids
Total lipids in lipoprotein particles	Total lipids
Total lipids in VLDL	Total lipids
Total phospholipids in lipoprotein particles	Phospholipids

Models were adjusted for age, regions (CKB only), ethnicity (UKB only), levels of education, smoking statu

HR, hazard ratio; CI, confidence interval; LDL, Low Density Lipoprotein; HDL, High Density Lipoprotein;

ion of all metabolites with all-cause and cause-specific mortality in the CKB and UKB by s

All causes mortality			Ischaemic heart c	
	Female		Male	
P value	Adjusted HR (95% CI)	P value	Adjusted HR (95% CI)	P value
**4.90E-02**	0.95 (0.89, 1.02)	3.51E-01	0.94 (0.84, 1.06)	5.59E-01
5.91E-02	0.96 (0.89, 1.02)	3.84E-01	0.95 (0.84, 1.07)	5.73E-01
1.09E-01	0.96 (0.90, 1.03)	4.51E-01	0.96 (0.85, 1.08)	6.91E-01
3.49E-01	0.99 (0.93, 1.05)	8.25E-01	0.99 (0.88, 1.10)	8.91E-01
5.68E-01	1.00 (0.94, 1.07)	9.92E-01	1.00 (0.90, 1.12)	9.75E-01
1.86E-01	0.97 (0.91, 1.04)	6.42E-01	0.96 (0.86, 1.08)	7.04E-01
**2.84E-02**	0.95 (0.89, 1.01)	2.63E-01	0.94 (0.83, 1.05)	4.81E-01
1.56E-01	0.98 (0.91, 1.04)	7.03E-01	0.95 (0.84, 1.07)	5.73E-01
1.68E-01	0.98 (0.92, 1.04)	7.03E-01	0.95 (0.85, 1.07)	5.93E-01
2.16E-01	0.98 (0.92, 1.05)	7.08E-01	0.96 (0.85, 1.08)	6.91E-01
3.17E-01	0.99 (0.93, 1.06)	9.37E-01	0.97 (0.87, 1.09)	7.76E-01
3.19E-01	1.00 (0.94, 1.07)	9.99E-01	0.97 (0.87, 1.09)	7.76E-01
3.05E-01	0.99 (0.93, 1.06)	8.44E-01	0.97 (0.87, 1.09)	7.66E-01
1.28E-01	0.97 (0.91, 1.04)	6.49E-01	0.94 (0.84, 1.06)	5.23E-01
1.45E-01	0.97 (0.91, 1.04)	6.42E-01	0.96 (0.86, 1.08)	7.04E-01
1.48E-01	0.97 (0.91, 1.04)	6.42E-01	0.96 (0.86, 1.08)	7.04E-01
1.65E-01	0.97 (0.91, 1.04)	6.28E-01	0.96 (0.86, 1.08)	7.04E-01
1.67E-01	0.97 (0.91, 1.04)	6.30E-01	0.96 (0.85, 1.07)	6.75E-01
1.28E-01	0.97 (0.91, 1.03)	6.01E-01	0.94 (0.84, 1.06)	5.57E-01
2.20E-01	0.97 (0.91, 1.04)	6.61E-01	0.97 (0.86, 1.09)	7.51E-01
1.38E-01	0.97 (0.91, 1.04)	6.49E-01	0.97 (0.86, 1.08)	7.30E-01
1.03E-01	0.98 (0.92, 1.05)	7.34E-01	0.99 (0.88, 1.11)	8.91E-01
1.11E-01	0.98 (0.92, 1.05)	7.34E-01	0.99 (0.88, 1.11)	8.91E-01
1.00E-01	0.98 (0.92, 1.04)	7.03E-01	0.98 (0.87, 1.10)	8.57E-01
2.07E-01	0.98 (0.92, 1.05)	7.82E-01	0.99 (0.88, 1.10)	8.91E-01
2.33E-01	0.98 (0.92, 1.05)	7.39E-01	0.98 (0.87, 1.10)	8.43E-01
1.97E-01	0.99 (0.93, 1.05)	8.25E-01	0.99 (0.89, 1.11)	9.51E-01
9.26E-02	0.98 (0.92, 1.05)	7.34E-01	0.99 (0.88, 1.11)	8.94E-01
2.04E-01	1.00 (0.93, 1.06)	9.62E-01	1.00 (0.90, 1.13)	9.73E-01
2.69E-01	1.00 (0.94, 1.07)	9.80E-01	1.02 (0.91, 1.14)	8.65E-01
1.50E-01	0.98 (0.92, 1.05)	7.34E-01	0.98 (0.88, 1.10)	8.80E-01
5.36E-01	1.06 (0.99, 1.12)	2.09E-01	1.15 (1.03, 1.28)	**4.09E-02**
2.27E-01	1.07 (1.01, 1.14)	8.71E-02	1.19 (1.07, 1.33)	**7.05E-03**
6.83E-01	1.02 (0.96, 1.09)	6.94E-01	1.06 (0.94, 1.18)	5.57E-01
6.11E-02	0.98 (0.91, 1.04)	6.78E-01	0.95 (0.85, 1.07)	6.26E-01
1.50E-01	1.03 (0.97, 1.10)	6.03E-01	1.14 (1.02, 1.27)	5.07E-02
9.63E-02	1.04 (0.98, 1.10)	4.51E-01	1.16 (1.04, 1.29)	**2.05E-02**
5.11E-01	1.00 (0.94, 1.07)	9.92E-01	1.05 (0.94, 1.18)	5.73E-01
**1.89E-03**	1.08 (1.02, 1.14)	**4.38E-02**	1.28 (1.15, 1.42)	**3.47E-05**
**5.71E-04**	1.08 (1.01, 1.14)	**4.72E-02**	1.30 (1.18, 1.44)	**5.91E-06**

**3.01E-02**	1.08 (1.01, 1.14)	**4.72E-02**	1.21 (1.09, 1.35)	**1.98E-03**
6.74E-01	0.98 (0.92, 1.05)	7.60E-01	0.98 (0.87, 1.10)	8.43E-01
5.17E-01	0.99 (0.93, 1.06)	8.71E-01	1.06 (0.95, 1.18)	5.57E-01
5.20E-01	0.99 (0.93, 1.06)	9.09E-01	1.06 (0.95, 1.18)	5.17E-01
7.92E-01	0.98 (0.92, 1.04)	7.27E-01	1.03 (0.92, 1.16)	7.41E-01
5.81E-01	1.00 (0.94, 1.06)	9.62E-01	1.07 (0.96, 1.20)	4.03E-01
7.53E-01	0.99 (0.93, 1.05)	8.24E-01	1.07 (0.96, 1.19)	4.47E-01
2.70E-01	1.02 (0.96, 1.08)	7.34E-01	1.08 (0.97, 1.21)	3.48E-01
8.67E-02	0.99 (0.93, 1.06)	8.76E-01	1.01 (0.90, 1.13)	9.10E-01
9.99E-01	0.98 (0.92, 1.04)	7.03E-01	1.03 (0.92, 1.15)	7.66E-01
9.66E-01	0.98 (0.92, 1.04)	7.03E-01	1.03 (0.92, 1.15)	7.66E-01
5.54E-01	0.96 (0.90, 1.02)	4.51E-01	1.00 (0.89, 1.12)	9.75E-01
8.36E-01	0.98 (0.92, 1.05)	7.34E-01	1.03 (0.92, 1.15)	7.51E-01
7.04E-01	0.98 (0.92, 1.04)	7.03E-01	1.03 (0.92, 1.15)	7.68E-01
7.02E-01	0.99 (0.93, 1.06)	8.73E-01	1.04 (0.93, 1.16)	6.91E-01
**4.86E-03**	1.01 (0.95, 1.08)	8.71E-01	1.05 (0.94, 1.17)	5.77E-01
2.76E-01	0.97 (0.91, 1.04)	6.15E-01	0.99 (0.88, 1.11)	9.19E-01
2.60E-01	0.97 (0.91, 1.04)	6.15E-01	0.99 (0.88, 1.11)	9.21E-01
**1.51E-02**	0.92 (0.86, 0.98)	**4.72E-02**	0.92 (0.82, 1.02)	2.68E-01
3.28E-01	0.98 (0.92, 1.05)	7.36E-01	1.01 (0.90, 1.13)	8.94E-01
3.81E-01	0.99 (0.93, 1.05)	8.22E-01	1.02 (0.91, 1.14)	8.41E-01
1.40E-01	0.96 (0.90, 1.03)	4.51E-01	0.97 (0.86, 1.09)	7.51E-01
9.14E-02	1.00 (0.94, 1.07)	9.83E-01	1.00 (0.89, 1.12)	9.68E-01
2.06E-01	0.97 (0.91, 1.04)	6.78E-01	1.00 (0.89, 1.12)	1.00E+00
2.06E-01	0.98 (0.91, 1.04)	6.86E-01	1.00 (0.89, 1.12)	9.75E-01
**5.83E-03**	0.92 (0.86, 0.98)	**4.88E-02**	0.91 (0.82, 1.02)	2.31E-01
3.75E-01	0.99 (0.93, 1.06)	8.73E-01	1.03 (0.92, 1.16)	7.51E-01
5.07E-01	0.99 (0.93, 1.06)	8.88E-01	1.04 (0.93, 1.17)	6.87E-01
9.64E-02	0.97 (0.91, 1.04)	6.30E-01	0.99 (0.88, 1.10)	8.91E-01
9.60E-01	1.00 (0.94, 1.07)	9.92E-01	1.00 (0.89, 1.12)	9.75E-01
**2.94E-09**	1.18 (1.11, 1.25)	**7.28E-07**	1.25 (1.14, 1.38)	**4.94E-05**
**2.94E-09**	1.18 (1.11, 1.25)	**7.28E-07**	1.26 (1.14, 1.38)	**4.61E-05**
**4.51E-09**	1.15 (1.09, 1.22)	**2.38E-05**	1.18 (1.07, 1.31)	**4.76E-03**
**3.69E-08**	1.20 (1.13, 1.27)	**9.65E-08**	1.32 (1.20, 1.46)	**4.04E-07**
**3.77E-08**	1.21 (1.14, 1.28)	**3.01E-08**	1.35 (1.23, 1.49)	**3.21E-08**
**4.70E-08**	1.17 (1.10, 1.24)	**1.85E-06**	1.24 (1.13, 1.37)	**1.19E-04**
**1.64E-05**	1.13 (1.06, 1.20)	**7.01E-04**	1.21 (1.10, 1.34)	**1.02E-03**
**5.42E-06**	1.11 (1.05, 1.18)	**3.45E-03**	1.11 (1.00, 1.24)	1.14E-01
**5.42E-06**	1.11 (1.05, 1.18)	**3.01E-03**	1.12 (1.00, 1.24)	1.08E-01
**1.96E-05**	1.10 (1.04, 1.17)	**7.83E-03**	1.11 (1.00, 1.24)	1.26E-01
**5.19E-06**	1.12 (1.05, 1.19)	**1.64E-03**	1.12 (1.01, 1.24)	9.60E-02
**6.67E-06**	1.11 (1.05, 1.19)	**2.57E-03**	1.11 (1.00, 1.24)	1.26E-01
**1.98E-06**	1.14 (1.07, 1.21)	**3.04E-04**	1.14 (1.03, 1.27)	**3.62E-02**
**8.26E-07**	1.07 (1.00, 1.14)	1.15E-01	1.07 (0.96, 1.19)	3.83E-01
**2.88E-02**	0.87 (0.82, 0.93)	**1.85E-04**	0.82 (0.73, 0.93)	**6.57E-03**
**3.15E-02**	0.88 (0.83, 0.94)	**3.74E-04**	0.83 (0.73, 0.93)	**7.65E-03**
2.69E-01	0.89 (0.83, 0.95)	**1.50E-03**	0.87 (0.77, 0.98)	5.88E-02
**7.14E-03**	0.90 (0.85, 0.95)	**2.57E-03**	0.83 (0.74, 0.93)	**7.62E-03**

**1.99E-03**	0.89 (0.84, 0.95)	**1.64E-03**	0.82 (0.73, 0.92)	**4.76E-03**
2.08E-01	0.91 (0.86, 0.97)	**2.09E-02**	0.87 (0.77, 0.98)	6.57E-02
**6.95E-03**	0.84 (0.79, 0.91)	**2.33E-05**	0.78 (0.69, 0.88)	**3.65E-04**
**1.04E-08**	0.81 (0.76, 0.86)	**2.75E-09**	0.75 (0.69, 0.82)	**1.85E-09**
**1.24E-08**	0.81 (0.76, 0.86)	**4.01E-09**	0.75 (0.69, 0.82)	**3.92E-09**
**4.29E-05**	0.84 (0.79, 0.89)	**5.72E-07**	0.79 (0.70, 0.88)	**3.15E-04**
**1.09E-03**	0.90 (0.84, 0.96)	**4.60E-03**	0.82 (0.74, 0.90)	**7.61E-04**
**1.45E-02**	0.92 (0.87, 0.98)	**4.75E-02**	0.85 (0.76, 0.94)	**7.05E-03**
**2.31E-04**	0.87 (0.82, 0.93)	**7.35E-05**	0.81 (0.72, 0.91)	**1.37E-03**
**2.91E-02**	0.88 (0.82, 0.94)	**1.56E-03**	0.82 (0.73, 0.93)	**4.81E-03**
1.28E-01	0.95 (0.89, 1.01)	2.63E-01	0.99 (0.89, 1.10)	8.94E-01
**2.94E-09**	1.14 (1.09, 1.20)	**1.75E-06**	1.15 (1.05, 1.26)	**7.12E-03**
**2.94E-09**	1.14 (1.09, 1.20)	**1.85E-06**	1.16 (1.06, 1.26)	**4.40E-03**
9.80E-02	1.07 (1.00, 1.15)	1.22E-01	1.03 (0.92, 1.15)	7.51E-01
**2.65E-05**	0.88 (0.83, 0.94)	**2.44E-04**	0.88 (0.79, 0.97)	**3.95E-02**
5.07E-01	0.92 (0.86, 0.99)	5.67E-02	1.03 (0.92, 1.15)	7.51E-01
**3.34E-06**	1.08 (1.03, 1.14)	**1.03E-02**	1.12 (1.05, 1.20)	**4.23E-03**
**6.29E-06**	1.09 (1.04, 1.14)	**2.57E-03**	1.15 (1.05, 1.26)	**8.95E-03**
2.13E-01	1.00 (0.93, 1.07)	9.62E-01	1.06 (0.97, 1.17)	3.94E-01
**6.21E-03**	0.96 (0.90, 1.03)	4.75E-01	0.87 (0.78, 0.96)	**1.91E-02**
4.57E-01	0.93 (0.87, 1.00)	1.14E-01	0.93 (0.83, 1.03)	3.01E-01
7.42E-01	0.97 (0.90, 1.03)	5.68E-01	0.93 (0.84, 1.03)	3.50E-01
5.22E-01	1.00 (0.94, 1.06)	9.65E-01	0.93 (0.83, 1.04)	3.50E-01
3.28E-01	0.96 (0.90, 1.03)	4.70E-01	0.98 (0.88, 1.09)	8.34E-01
6.44E-01	1.05 (0.98, 1.12)	3.66E-01	1.08 (0.97, 1.20)	2.99E-01
5.78E-01	0.91 (0.85, 0.98)	**3.09E-02**	0.82 (0.73, 0.92)	**4.40E-03**
6.30E-02	1.02 (0.95, 1.09)	7.35E-01	0.95 (0.85, 1.06)	5.78E-01
8.90E-02	1.01 (0.94, 1.07)	8.90E-01	0.93 (0.83, 1.04)	4.03E-01
7.53E-01	1.04 (0.97, 1.12)	5.68E-01	1.05 (0.94, 1.18)	5.73E-01
1.13E-01	1.00 (0.93, 1.06)	9.62E-01	1.08 (0.97, 1.21)	3.31E-01
4.32E-01	0.89 (0.83, 0.95)	**3.77E-03**	0.79 (0.70, 0.89)	**1.05E-03**
**2.63E-04**	1.12 (1.05, 1.19)	**2.57E-03**	1.25 (1.12, 1.40)	**4.31E-04**
**1.18E-03**	1.10 (1.04, 1.18)	**8.25E-03**	1.24 (1.11, 1.39)	**8.36E-04**
**3.71E-06**	1.17 (1.10, 1.25)	**7.24E-06**	1.21 (1.09, 1.34)	**1.20E-03**
**7.71E-04**	0.91 (0.86, 0.97)	**2.17E-02**	0.84 (0.76, 0.94)	**8.24E-03**
**2.88E-02**	0.93 (0.87, 0.98)	**4.57E-02**	0.84 (0.76, 0.92)	**7.61E-04**
**5.88E-05**	1.16 (1.09, 1.24)	**5.76E-05**	1.52 (1.34, 1.73)	**3.92E-09**
**3.48E-05**	1.12 (1.06, 1.20)	**1.76E-03**	1.47 (1.31, 1.65)	**3.92E-09**
9.66E-02	1.18 (1.10, 1.27)	**4.44E-05**	1.24 (1.09, 1.41)	**4.40E-03**
7.28E-02	0.92 (0.87, 0.98)	**4.77E-02**	0.82 (0.72, 0.93)	**7.34E-03**
**3.35E-02**	0.94 (0.88, 1.00)	1.16E-01	0.87 (0.78, 0.97)	**2.87E-02**
5.22E-01	1.03 (0.97, 1.10)	5.18E-01	1.10 (0.98, 1.24)	2.29E-01
6.11E-02	0.97 (0.91, 1.03)	5.72E-01	1.05 (0.94, 1.17)	5.73E-01
2.76E-01	1.10 (1.03, 1.18)	**2.67E-02**	1.08 (0.96, 1.22)	3.50E-01
1.84E-01	0.99 (0.93, 1.05)	7.98E-01	0.96 (0.86, 1.07)	6.90E-01
7.30E-01	0.95 (0.89, 1.02)	3.49E-01	0.99 (0.88, 1.11)	8.94E-01
1.30E-01	1.01 (0.95, 1.07)	8.73E-01	0.98 (0.88, 1.09)	7.76E-01
**1.98E-02**	0.98 (0.92, 1.05)	7.36E-01	0.95 (0.86, 1.05)	5.48E-01

6.30E-02	1.08 (1.01, 1.16)	6.69E-02	1.09 (0.97, 1.22)	3.19E-01
**3.54E-03**	1.02 (0.96, 1.09)	7.03E-01	1.05 (0.95, 1.16)	5.73E-01
7.26E-01	0.94 (0.88, 1.01)	1.85E-01	0.94 (0.84, 1.06)	5.17E-01
3.81E-01	1.03 (0.97, 1.10)	6.15E-01	1.03 (0.92, 1.16)	7.51E-01
3.23E-01	1.02 (0.96, 1.09)	7.34E-01	1.01 (0.91, 1.13)	8.91E-01
2.60E-01	1.02 (0.95, 1.09)	7.74E-01	1.04 (0.93, 1.16)	7.03E-01
**7.71E-04**	1.03 (0.97, 1.10)	5.98E-01	1.03 (0.92, 1.15)	7.51E-01
8.07E-01	0.95 (0.89, 1.01)	2.49E-01	0.92 (0.82, 1.04)	3.27E-01
7.22E-01	1.03 (0.97, 1.10)	5.97E-01	1.06 (0.94, 1.19)	5.57E-01
6.70E-01	1.02 (0.96, 1.09)	7.03E-01	1.04 (0.93, 1.17)	6.85E-01
5.08E-01	1.02 (0.96, 1.09)	7.03E-01	1.03 (0.92, 1.15)	7.68E-01
1.46E-01	1.01 (0.95, 1.08)	7.82E-01	1.01 (0.90, 1.12)	9.66E-01
2.20E-01	0.94 (0.88, 1.00)	1.03E-01	0.87 (0.78, 0.97)	**2.88E-02**
3.46E-01	1.11 (1.04, 1.18)	**7.63E-03**	1.23 (1.10, 1.38)	**1.63E-03**
2.20E-01	1.11 (1.04, 1.18)	**5.72E-03**	1.22 (1.09, 1.36)	**1.82E-03**
5.54E-01	1.05 (0.98, 1.11)	3.23E-01	1.14 (1.01, 1.29)	7.36E-02
2.04E-01	0.94 (0.88, 1.01)	1.85E-01	0.92 (0.82, 1.03)	3.19E-01
**1.27E-05**	0.89 (0.84, 0.95)	**2.84E-03**	0.86 (0.77, 0.97)	**3.67E-02**
**1.12E-03**	1.13 (1.06, 1.21)	**1.64E-03**	1.18 (1.05, 1.33)	**2.21E-02**
5.07E-02	1.08 (1.01, 1.15)	6.61E-02	1.05 (0.94, 1.18)	5.85E-01
**2.30E-05**	1.18 (1.10, 1.27)	**2.33E-05**	1.35 (1.19, 1.53)	**4.94E-05**
4.04E-01	0.96 (0.90, 1.02)	4.03E-01	0.95 (0.85, 1.06)	5.73E-01
**1.96E-19**	1.09 (1.03, 1.15)	**6.31E-03**	1.22 (1.13, 1.31)	**2.84E-06**
**1.31E-05**	0.99 (0.93, 1.05)	8.34E-01	0.93 (0.83, 1.03)	3.30E-01
**6.17E-07**	0.99 (0.93, 1.05)	8.11E-01	0.92 (0.83, 1.02)	2.76E-01
6.23E-01	1.00 (0.94, 1.07)	9.80E-01	0.98 (0.88, 1.09)	8.12E-01
5.75E-01	0.93 (0.87, 1.00)	1.06E-01	0.88 (0.78, 0.98)	7.16E-02
6.83E-01	0.97 (0.91, 1.03)	6.01E-01	1.02 (0.91, 1.14)	8.46E-01
6.83E-01	1.04 (0.98, 1.11)	4.51E-01	1.01 (0.90, 1.13)	9.45E-01
7.53E-01	1.03 (0.96, 1.10)	6.42E-01	1.00 (0.89, 1.11)	9.66E-01
5.54E-01	1.07 (1.00, 1.14)	9.86E-02	1.11 (0.99, 1.24)	1.75E-01
8.26E-01	0.96 (0.90, 1.02)	3.84E-01	0.92 (0.82, 1.03)	3.02E-01
**7.08E-03**	0.94 (0.88, 1.00)	1.76E-01	0.96 (0.86, 1.07)	6.91E-01
**1.42E-07**	1.00 (0.93, 1.06)	9.62E-01	1.10 (0.99, 1.23)	1.78E-01
**6.81E-09**	1.17 (1.10, 1.25)	**3.78E-06**	1.26 (1.14, 1.40)	**1.02E-04**
4.86E-01	1.03 (0.97, 1.09)	6.42E-01	1.09 (0.98, 1.22)	2.29E-01
9.50E-01	1.03 (0.96, 1.09)	6.42E-01	1.10 (0.98, 1.23)	2.07E-01
8.26E-01	1.02 (0.96, 1.08)	7.36E-01	1.10 (0.99, 1.23)	1.84E-01
6.70E-01	0.99 (0.93, 1.06)	8.90E-01	1.04 (0.93, 1.17)	6.75E-01
**1.78E-02**	1.07 (1.01, 1.14)	8.33E-02	1.07 (0.96, 1.19)	4.47E-01
**4.29E-02**	1.06 (0.99, 1.12)	2.03E-01	1.04 (0.93, 1.16)	7.04E-01
**4.05E-06**	1.17 (1.11, 1.24)	**1.86E-06**	1.31 (1.18, 1.45)	**6.74E-06**
9.66E-02	0.89 (0.83, 0.95)	**3.03E-03**	0.86 (0.77, 0.97)	**3.72E-02**
**9.91E-03**	0.97 (0.90, 1.03)	5.72E-01	0.88 (0.78, 0.99)	1.03E-01
2.27E-01	0.98 (0.92, 1.04)	7.03E-01	0.98 (0.88, 1.10)	8.65E-01
1.00E-01	0.98 (0.92, 1.04)	7.03E-01	0.98 (0.88, 1.10)	8.65E-01
**2.17E-02**	1.01 (0.95, 1.08)	8.44E-01	1.04 (0.93, 1.16)	7.04E-01
6.42E-01	0.95 (0.89, 1.02)	3.28E-01	0.92 (0.82, 1.03)	3.04E-01

2.70E-01	1.04 (0.98, 1.11)	3.84E-01	0.95 (0.85, 1.06)	5.77E-01
1.00E-01	1.00 (0.94, 1.07)	9.62E-01	1.00 (0.89, 1.12)	9.75E-01
**2.39E-05**	0.93 (0.87, 0.99)	8.75E-02	0.78 (0.69, 0.87)	**7.88E-05**
**7.97E-06**	0.87 (0.81, 0.93)	**6.82E-04**	0.79 (0.70, 0.88)	**2.38E-04**
1.00E-01	1.01 (0.95, 1.08)	8.34E-01	0.88 (0.78, 0.98)	6.46E-02
**4.74E-02**	1.08 (1.02, 1.15)	**4.72E-02**	1.10 (0.99, 1.23)	1.88E-01
3.49E-01	0.99 (0.93, 1.06)	8.90E-01	1.04 (0.93, 1.16)	7.04E-01
9.04E-02	0.95 (0.89, 1.02)	3.44E-01	0.98 (0.88, 1.10)	8.43E-01
**1.63E-03**	0.99 (0.92, 1.05)	7.94E-01	0.87 (0.77, 0.98)	6.99E-02
**1.56E-18**	0.84 (0.78, 0.90)	**9.87E-06**	0.70 (0.64, 0.76)	**1.64E-14**
1.28E-01	1.16 (1.10, 1.23)	**4.37E-06**	1.42 (1.29, 1.56)	**1.56E-11**
**6.29E-06**	1.00 (0.93, 1.07)	9.65E-01	0.80 (0.71, 0.89)	**8.02E-04**
1.94E-01	1.07 (1.01, 1.14)	7.72E-02	1.17 (1.04, 1.30)	**2.18E-02**
**4.25E-06**	0.96 (0.89, 1.02)	4.10E-01	0.76 (0.68, 0.86)	**9.70E-05**
**1.79E-05**	0.98 (0.91, 1.05)	7.03E-01	0.81 (0.72, 0.92)	**4.40E-03**
**2.65E-02**	0.98 (0.92, 1.05)	7.27E-01	0.93 (0.83, 1.05)	4.40E-01
**9.85E-03**	1.00 (0.94, 1.07)	9.86E-01	0.88 (0.78, 0.99)	1.02E-01
**4.54E-07**	1.24 (1.16, 1.33)	**4.06E-09**	1.74 (1.58, 1.91)	**7.75E-29**
**2.53E-06**	1.01 (0.95, 1.08)	8.25E-01	0.78 (0.72, 0.86)	**2.90E-06**
3.34E-01	1.13 (1.06, 1.20)	**6.12E-04**	1.46 (1.32, 1.61)	**1.21E-11**
**1.11E-04**	0.93 (0.87, 1.00)	1.28E-01	0.73 (0.66, 0.81)	**7.00E-08**
**2.08E-04**	0.97 (0.91, 1.04)	6.90E-01	0.81 (0.73, 0.90)	**6.87E-04**
1.51E-01	0.97 (0.91, 1.04)	6.01E-01	1.19 (1.07, 1.32)	**4.81E-03**
**1.01E-04**	1.09 (1.02, 1.17)	**4.16E-02**	1.12 (1.00, 1.25)	1.19E-01
**1.98E-06**	1.11 (1.06, 1.17)	**5.28E-05**	1.15 (1.05, 1.25)	**7.05E-03**
4.10E-01	0.97 (0.90, 1.03)	5.72E-01	1.08 (0.97, 1.19)	3.04E-01
5.53E-01	0.96 (0.90, 1.03)	4.51E-01	0.80 (0.71, 0.90)	**1.19E-03**
**3.47E-04**	0.97 (0.91, 1.04)	6.42E-01	0.97 (0.87, 1.08)	7.59E-01
**3.46E-05**	0.95 (0.89, 1.01)	2.43E-01	0.91 (0.81, 1.01)	1.94E-01
3.12E-01	1.02 (0.96, 1.09)	6.90E-01	1.23 (1.12, 1.36)	**2.81E-04**
**2.88E-02**	0.93 (0.87, 0.99)	8.71E-02	0.96 (0.86, 1.08)	7.11E-01
2.20E-01	0.95 (0.89, 1.02)	3.28E-01	1.09 (0.98, 1.22)	2.29E-01
**3.50E-08**	0.82 (0.77, 0.88)	**1.21E-06**	0.83 (0.74, 0.93)	**7.05E-03**
**9.39E-08**	1.10 (1.05, 1.16)	**8.58E-04**	1.19 (1.13, 1.26)	**1.40E-09**
2.20E-01	1.02 (0.96, 1.08)	7.34E-01	1.10 (0.99, 1.22)	1.97E-01
**8.38E-04**	0.98 (0.93, 1.03)	6.86E-01	1.12 (1.02, 1.23)	**4.81E-02**
2.04E-01	0.96 (0.90, 1.03)	4.70E-01	0.83 (0.71, 0.97)	5.25E-02
**5.11E-03**	0.94 (0.88, 1.02)	3.10E-01	0.86 (0.72, 1.03)	2.21E-01
2.06E-01	1.06 (1.02, 1.10)	**2.37E-02**	0.94 (0.75, 1.17)	7.31E-01
**5.07E-09**	0.85 (0.80, 0.90)	**1.75E-06**	0.74 (0.69, 0.81)	**1.56E-11**
**2.34E-03**	1.19 (1.12, 1.27)	**3.50E-07**	1.31 (1.18, 1.46)	**8.97E-06**

s, alcohol consumption, and self-rated health status. P values are corrected for multiple testing using a fa
VLDL, Very Low Density Lipoprotein; IDL, Intermediate Density Lipoprotein..

sex

iseases mortality		Cerebrovascular diseases mortality		
Female		Male		Female
Adjusted HR (95% CI)	P value	Adjusted HR (95% CI)	P value	Adjusted HR (95% CI)
1.03 (0.92, 1.16)	7.85E-01	0.94 (0.83, 1.06)	5.93E-01	0.81 (0.69, 0.94)
1.03 (0.92, 1.17)	7.69E-01	0.94 (0.84, 1.07)	6.05E-01	0.81 (0.70, 0.94)
1.05 (0.93, 1.18)	6.71E-01	0.95 (0.85, 1.08)	6.79E-01	0.82 (0.70, 0.95)
1.06 (0.94, 1.19)	6.34E-01	0.96 (0.86, 1.09)	7.24E-01	0.86 (0.74, 0.99)
1.07 (0.96, 1.21)	4.64E-01	0.97 (0.86, 1.09)	7.24E-01	0.87 (0.76, 1.01)
1.03 (0.91, 1.16)	8.13E-01	0.96 (0.85, 1.08)	7.19E-01	0.83 (0.72, 0.97)
1.03 (0.91, 1.16)	8.13E-01	0.94 (0.83, 1.06)	5.63E-01	0.80 (0.68, 0.93)
1.03 (0.92, 1.17)	7.70E-01	0.96 (0.85, 1.08)	7.02E-01	0.84 (0.73, 0.97)
1.04 (0.92, 1.17)	7.50E-01	0.96 (0.85, 1.08)	7.02E-01	0.84 (0.73, 0.97)
1.04 (0.93, 1.17)	6.98E-01	0.96 (0.85, 1.08)	7.02E-01	0.83 (0.72, 0.96)
1.06 (0.94, 1.19)	6.03E-01	0.97 (0.86, 1.09)	7.24E-01	0.86 (0.75, 0.99)
1.07 (0.95, 1.20)	5.31E-01	0.97 (0.86, 1.09)	7.40E-01	0.88 (0.76, 1.01)
1.05 (0.93, 1.18)	6.71E-01	0.96 (0.85, 1.09)	7.24E-01	0.84 (0.73, 0.97)
1.03 (0.91, 1.16)	8.13E-01	0.95 (0.85, 1.08)	6.79E-01	0.84 (0.72, 0.97)
1.05 (0.94, 1.19)	6.48E-01	0.97 (0.86, 1.09)	7.24E-01	0.86 (0.75, 0.99)
1.05 (0.93, 1.19)	6.50E-01	0.97 (0.86, 1.09)	7.24E-01	0.86 (0.74, 0.99)
1.05 (0.93, 1.18)	6.71E-01	0.97 (0.86, 1.09)	7.24E-01	0.86 (0.74, 0.99)
1.04 (0.93, 1.18)	6.94E-01	0.96 (0.85, 1.09)	7.24E-01	0.85 (0.74, 0.98)
1.04 (0.92, 1.18)	6.99E-01	0.95 (0.84, 1.07)	6.17E-01	0.84 (0.73, 0.97)
1.05 (0.93, 1.18)	6.80E-01	0.98 (0.87, 1.10)	7.82E-01	0.86 (0.74, 0.99)
1.06 (0.94, 1.19)	6.34E-01	0.97 (0.86, 1.09)	7.24E-01	0.86 (0.75, 1.00)
1.07 (0.95, 1.20)	5.63E-01	0.96 (0.85, 1.09)	7.24E-01	0.87 (0.76, 1.01)
1.07 (0.95, 1.20)	5.71E-01	0.97 (0.85, 1.09)	7.24E-01	0.87 (0.75, 1.00)
1.06 (0.94, 1.20)	6.24E-01	0.96 (0.85, 1.09)	7.19E-01	0.86 (0.75, 1.00)
1.06 (0.93, 1.19)	6.48E-01	0.97 (0.85, 1.09)	7.24E-01	0.87 (0.75, 1.00)
1.04 (0.92, 1.18)	6.98E-01	0.95 (0.84, 1.08)	6.72E-01	0.86 (0.74, 0.99)
1.06 (0.94, 1.20)	5.77E-01	0.98 (0.87, 1.10)	8.00E-01	0.88 (0.76, 1.02)
1.07 (0.95, 1.21)	5.10E-01	0.97 (0.86, 1.09)	7.24E-01	0.88 (0.76, 1.01)
1.10 (0.97, 1.24)	3.10E-01	0.96 (0.85, 1.09)	7.17E-01	0.91 (0.79, 1.05)
1.11 (0.99, 1.26)	2.16E-01	0.97 (0.85, 1.09)	7.24E-01	0.92 (0.80, 1.06)
1.09 (0.96, 1.23)	3.70E-01	0.95 (0.84, 1.08)	6.79E-01	0.89 (0.77, 1.03)
1.21 (1.08, 1.35)	**9.19E-03**	1.02 (0.90, 1.15)	8.58E-01	1.06 (0.92, 1.21)
1.22 (1.09, 1.37)	**4.67E-03**	1.02 (0.91, 1.16)	7.93E-01	1.10 (0.97, 1.26)
1.15 (1.02, 1.30)	7.10E-02	1.00 (0.89, 1.13)	9.98E-01	0.97 (0.84, 1.12)
1.04 (0.92, 1.18)	6.99E-01	0.94 (0.83, 1.07)	6.05E-01	0.86 (0.75, 1.00)
1.12 (1.00, 1.26)	1.31E-01	1.03 (0.91, 1.16)	7.46E-01	1.05 (0.92, 1.20)
1.14 (1.02, 1.28)	8.05E-02	1.04 (0.92, 1.17)	7.24E-01	1.07 (0.94, 1.23)
1.05 (0.93, 1.19)	6.71E-01	0.97 (0.86, 1.09)	7.40E-01	1.00 (0.88, 1.15)
1.21 (1.09, 1.35)	**4.39E-03**	1.09 (0.97, 1.23)	3.45E-01	1.17 (1.04, 1.33)
1.21 (1.09, 1.35)	**4.27E-03**	1.11 (0.99, 1.25)	2.34E-01	1.17 (1.03, 1.32)

1.19 (1.07, 1.33)	**9.70E-03**	1.05 (0.93, 1.18)	6.52E-01	1.17 (1.04, 1.33)
1.02 (0.90, 1.16)	8.33E-01	0.99 (0.88, 1.11)	8.86E-01	0.91 (0.79, 1.06)
1.01 (0.89, 1.14)	9.58E-01	0.98 (0.86, 1.10)	7.80E-01	0.97 (0.85, 1.12)
1.01 (0.90, 1.14)	9.09E-01	0.97 (0.86, 1.10)	7.66E-01	0.98 (0.85, 1.12)
0.99 (0.88, 1.13)	9.61E-01	0.95 (0.84, 1.08)	6.72E-01	0.97 (0.84, 1.11)
1.02 (0.91, 1.15)	8.33E-01	0.97 (0.86, 1.09)	7.24E-01	0.99 (0.86, 1.13)
1.02 (0.90, 1.15)	8.63E-01	0.96 (0.85, 1.08)	6.97E-01	0.96 (0.84, 1.10)
1.04 (0.92, 1.17)	7.60E-01	0.99 (0.88, 1.11)	8.86E-01	1.05 (0.92, 1.20)
0.97 (0.86, 1.11)	8.16E-01	1.05 (0.94, 1.18)	5.98E-01	0.98 (0.85, 1.13)
1.02 (0.90, 1.15)	8.63E-01	0.95 (0.84, 1.07)	6.17E-01	0.96 (0.84, 1.11)
1.02 (0.90, 1.15)	8.63E-01	0.95 (0.84, 1.07)	6.05E-01	0.97 (0.84, 1.11)
0.99 (0.88, 1.13)	9.61E-01	0.91 (0.81, 1.03)	3.54E-01	0.92 (0.80, 1.06)
1.03 (0.91, 1.16)	8.13E-01	0.94 (0.83, 1.06)	5.85E-01	0.98 (0.85, 1.12)
1.03 (0.91, 1.17)	8.06E-01	0.94 (0.83, 1.06)	5.60E-01	0.96 (0.84, 1.11)
1.01 (0.90, 1.14)	9.09E-01	0.96 (0.85, 1.08)	7.02E-01	1.01 (0.88, 1.15)
1.00 (0.88, 1.13)	9.79E-01	1.09 (0.98, 1.21)	3.24E-01	1.01 (0.88, 1.16)
1.03 (0.91, 1.16)	8.13E-01	0.93 (0.82, 1.04)	4.47E-01	0.96 (0.83, 1.10)
1.03 (0.91, 1.16)	8.13E-01	0.92 (0.82, 1.04)	4.29E-01	0.96 (0.83, 1.10)
0.94 (0.83, 1.08)	6.48E-01	0.89 (0.79, 0.99)	1.40E-01	0.85 (0.74, 0.98)
1.05 (0.93, 1.19)	6.74E-01	0.93 (0.82, 1.05)	4.47E-01	0.99 (0.86, 1.14)
1.06 (0.94, 1.20)	6.34E-01	0.93 (0.82, 1.05)	4.68E-01	1.00 (0.87, 1.15)
1.00 (0.88, 1.13)	9.78E-01	0.92 (0.82, 1.03)	3.54E-01	0.95 (0.83, 1.09)
1.00 (0.88, 1.13)	9.76E-01	1.08 (0.97, 1.21)	3.83E-01	1.00 (0.87, 1.15)
1.06 (0.93, 1.20)	6.34E-01	0.93 (0.82, 1.04)	4.47E-01	0.97 (0.84, 1.11)
1.06 (0.93, 1.20)	6.34E-01	0.92 (0.82, 1.04)	4.43E-01	0.97 (0.84, 1.12)
0.97 (0.85, 1.11)	8.13E-01	0.89 (0.80, 1.00)	1.52E-01	0.86 (0.75, 0.98)
1.08 (0.95, 1.22)	4.64E-01	0.93 (0.83, 1.06)	5.37E-01	1.01 (0.88, 1.16)
1.08 (0.96, 1.22)	4.33E-01	0.94 (0.83, 1.06)	5.75E-01	1.02 (0.89, 1.17)
1.04 (0.92, 1.18)	6.98E-01	0.92 (0.82, 1.03)	3.70E-01	0.97 (0.85, 1.11)
1.06 (0.94, 1.20)	6.34E-01	1.03 (0.92, 1.16)	7.40E-01	0.94 (0.82, 1.09)
1.20 (1.08, 1.35)	**8.52E-03**	1.26 (1.13, 1.40)	**6.42E-04**	1.23 (1.09, 1.39)
1.21 (1.08, 1.35)	**7.15E-03**	1.26 (1.13, 1.40)	**6.42E-04**	1.23 (1.09, 1.39)
1.11 (0.99, 1.24)	2.11E-01	1.23 (1.10, 1.37)	**2.20E-03**	1.19 (1.06, 1.35)
1.31 (1.17, 1.47)	**3.65E-05**	1.26 (1.13, 1.41)	**7.55E-04**	1.27 (1.12, 1.44)
1.36 (1.22, 1.52)	**1.90E-06**	1.28 (1.14, 1.43)	**5.21E-04**	1.29 (1.14, 1.46)
1.19 (1.06, 1.33)	**1.35E-02**	1.22 (1.10, 1.37)	**3.23E-03**	1.21 (1.07, 1.36)
1.14 (1.02, 1.28)	7.34E-02	1.21 (1.09, 1.33)	**3.31E-03**	1.11 (0.98, 1.26)
1.04 (0.93, 1.18)	6.88E-01	1.19 (1.07, 1.33)	**1.23E-02**	1.14 (1.01, 1.29)
1.05 (0.93, 1.18)	6.72E-01	1.19 (1.07, 1.33)	**1.23E-02**	1.15 (1.01, 1.30)
1.06 (0.94, 1.19)	6.48E-01	1.18 (1.05, 1.32)	**2.25E-02**	1.13 (1.00, 1.28)
1.05 (0.93, 1.19)	6.55E-01	1.19 (1.06, 1.33)	**1.31E-02**	1.16 (1.02, 1.31)
1.04 (0.92, 1.17)	7.21E-01	1.19 (1.06, 1.33)	**1.47E-02**	1.14 (1.01, 1.30)
1.09 (0.97, 1.23)	3.44E-01	1.20 (1.07, 1.34)	**9.92E-03**	1.19 (1.05, 1.35)
0.93 (0.82, 1.06)	5.31E-01	1.23 (1.11, 1.37)	**1.19E-03**	1.07 (0.95, 1.22)
0.83 (0.74, 0.93)	**1.10E-02**	0.91 (0.80, 1.04)	3.70E-01	0.78 (0.69, 0.88)
0.83 (0.74, 0.94)	**1.53E-02**	0.91 (0.80, 1.04)	3.74E-01	0.79 (0.70, 0.89)
0.85 (0.75, 0.96)	**3.66E-02**	0.97 (0.86, 1.10)	7.40E-01	0.81 (0.71, 0.92)
0.86 (0.76, 0.96)	**3.70E-02**	0.89 (0.78, 1.00)	1.85E-01	0.82 (0.72, 0.92)

0.85 (0.76, 0.96)	**3.04E-02**	0.87 (0.77, 0.99)	1.15E-01	0.81 (0.72, 0.91)
0.87 (0.77, 0.98)	7.80E-02	0.95 (0.84, 1.07)	6.17E-01	0.85 (0.75, 0.97)
0.77 (0.67, 0.89)	**2.90E-03**	0.90 (0.79, 1.02)	2.59E-01	0.67 (0.58, 0.78)
0.81 (0.72, 0.90)	**2.60E-03**	0.83 (0.75, 0.93)	**6.21E-03**	0.73 (0.65, 0.81)
0.81 (0.72, 0.91)	**4.23E-03**	0.83 (0.75, 0.92)	**5.98E-03**	0.73 (0.66, 0.81)
0.83 (0.74, 0.93)	**8.85E-03**	0.88 (0.78, 1.00)	1.60E-01	0.74 (0.66, 0.84)
0.92 (0.81, 1.04)	3.56E-01	0.87 (0.78, 0.97)	5.47E-02	0.87 (0.76, 0.98)
0.95 (0.84, 1.07)	6.34E-01	0.89 (0.79, 0.99)	1.22E-01	0.91 (0.80, 1.03)
0.86 (0.77, 0.96)	**4.33E-02**	0.89 (0.79, 1.01)	2.44E-01	0.79 (0.70, 0.89)
0.84 (0.74, 0.96)	5.20E-02	0.95 (0.84, 1.07)	6.05E-01	0.76 (0.66, 0.88)
1.03 (0.90, 1.18)	8.13E-01	0.99 (0.88, 1.12)	9.17E-01	1.01 (0.88, 1.16)
1.12 (1.02, 1.23)	7.04E-02	1.15 (1.04, 1.27)	**4.05E-02**	1.19 (1.10, 1.28)
1.12 (1.03, 1.23)	**4.71E-02**	1.16 (1.04, 1.28)	**2.94E-02**	1.18 (1.10, 1.27)
1.03 (0.90, 1.17)	8.33E-01	1.04 (0.93, 1.17)	7.02E-01	1.10 (0.99, 1.23)
0.88 (0.79, 0.98)	7.10E-02	0.89 (0.79, 0.99)	1.31E-01	0.82 (0.75, 0.90)
1.01 (0.87, 1.16)	9.61E-01	0.89 (0.80, 1.00)	1.60E-01	0.89 (0.79, 1.01)
1.10 (1.00, 1.22)	1.66E-01	1.12 (1.03, 1.22)	**4.27E-02**	1.16 (1.04, 1.29)
1.09 (0.99, 1.20)	2.28E-01	1.23 (1.11, 1.36)	**1.19E-03**	1.17 (1.07, 1.29)
1.05 (0.91, 1.22)	6.80E-01	0.94 (0.83, 1.06)	5.75E-01	0.96 (0.84, 1.10)
0.90 (0.79, 1.03)	3.16E-01	0.97 (0.85, 1.09)	7.24E-01	0.94 (0.82, 1.08)
0.90 (0.79, 1.02)	2.73E-01	0.96 (0.85, 1.08)	7.02E-01	0.97 (0.85, 1.11)
0.87 (0.78, 0.98)	7.10E-02	0.94 (0.84, 1.05)	5.48E-01	0.96 (0.84, 1.09)
0.85 (0.75, 0.97)	5.20E-02	0.92 (0.81, 1.05)	4.43E-01	0.95 (0.83, 1.09)
0.98 (0.86, 1.12)	8.49E-01	0.99 (0.88, 1.11)	8.86E-01	0.99 (0.87, 1.14)
1.15 (1.02, 1.29)	7.10E-02	1.06 (0.95, 1.19)	5.61E-01	1.05 (0.92, 1.19)
0.79 (0.69, 0.91)	**7.15E-03**	0.89 (0.78, 1.00)	1.85E-01	0.89 (0.78, 1.02)
0.87 (0.77, 0.99)	1.14E-01	1.00 (0.89, 1.13)	9.71E-01	0.99 (0.87, 1.14)
0.87 (0.76, 0.99)	1.01E-01	0.99 (0.87, 1.12)	8.86E-01	0.97 (0.85, 1.11)
1.03 (0.89, 1.19)	8.13E-01	1.04 (0.92, 1.18)	7.02E-01	1.09 (0.94, 1.27)
1.17 (1.03, 1.34)	6.05E-02	1.02 (0.90, 1.15)	8.33E-01	1.02 (0.89, 1.17)
0.82 (0.72, 0.94)	**2.37E-02**	0.97 (0.86, 1.10)	7.40E-01	0.81 (0.71, 0.93)
1.22 (1.08, 1.38)	**1.10E-02**	1.14 (1.01, 1.28)	1.38E-01	1.25 (1.09, 1.43)
1.19 (1.05, 1.35)	**2.63E-02**	1.11 (0.99, 1.25)	2.58E-01	1.22 (1.07, 1.39)
1.29 (1.15, 1.45)	**2.33E-04**	1.23 (1.10, 1.37)	**3.23E-03**	1.30 (1.15, 1.47)
0.86 (0.76, 0.97)	5.20E-02	0.89 (0.79, 1.00)	1.84E-01	0.84 (0.74, 0.96)
0.85 (0.76, 0.95)	**1.76E-02**	0.87 (0.79, 0.97)	**4.05E-02**	0.87 (0.77, 0.97)
1.38 (1.20, 1.58)	**4.91E-05**	1.23 (1.08, 1.40)	**1.27E-02**	1.40 (1.21, 1.62)
1.30 (1.14, 1.48)	**8.36E-04**	1.23 (1.09, 1.40)	**7.85E-03**	1.28 (1.12, 1.47)
1.35 (1.17, 1.56)	**6.93E-04**	1.07 (0.95, 1.20)	5.52E-01	1.41 (1.21, 1.65)
0.86 (0.76, 0.98)	7.52E-02	0.94 (0.83, 1.06)	5.75E-01	0.85 (0.73, 0.97)
0.88 (0.78, 0.99)	1.14E-01	0.92 (0.82, 1.03)	3.45E-01	0.94 (0.82, 1.07)
1.08 (0.96, 1.22)	4.33E-01	0.95 (0.85, 1.06)	5.75E-01	1.05 (0.93, 1.20)
1.02 (0.91, 1.15)	8.39E-01	0.92 (0.82, 1.02)	3.26E-01	0.93 (0.82, 1.05)
1.10 (0.97, 1.25)	3.37E-01	1.02 (0.90, 1.14)	8.53E-01	1.21 (1.04, 1.41)
0.97 (0.86, 1.09)	7.67E-01	1.08 (0.97, 1.20)	3.54E-01	0.97 (0.85, 1.10)
0.90 (0.79, 1.04)	3.36E-01	0.95 (0.84, 1.07)	6.40E-01	0.91 (0.79, 1.04)
1.05 (0.93, 1.19)	6.75E-01	0.95 (0.86, 1.06)	6.05E-01	1.04 (0.91, 1.18)
1.05 (0.92, 1.19)	6.74E-01	0.93 (0.84, 1.03)	4.07E-01	0.99 (0.87, 1.12)

1.01 (0.89, 1.15)	9.13E-01	1.07 (0.95, 1.21)	5.37E-01	1.19 (1.03, 1.37)
1.00 (0.89, 1.13)	9.89E-01	1.11 (1.01, 1.23)	1.22E-01	1.01 (0.90, 1.15)
0.86 (0.75, 0.99)	1.11E-01	0.99 (0.88, 1.11)	8.86E-01	0.86 (0.74, 0.99)
1.11 (0.97, 1.26)	3.16E-01	0.95 (0.85, 1.06)	6.05E-01	1.10 (0.96, 1.27)
1.10 (0.96, 1.25)	3.62E-01	0.94 (0.84, 1.06)	5.75E-01	1.07 (0.94, 1.23)
0.94 (0.83, 1.08)	6.50E-01	1.07 (0.95, 1.20)	5.02E-01	1.00 (0.88, 1.14)
1.00 (0.88, 1.13)	9.78E-01	1.15 (1.04, 1.27)	**2.54E-02**	1.02 (0.90, 1.16)
0.86 (0.75, 0.98)	8.43E-02	0.99 (0.88, 1.12)	9.21E-01	0.86 (0.75, 0.99)
1.11 (0.97, 1.26)	3.16E-01	0.97 (0.87, 1.09)	7.40E-01	1.12 (0.97, 1.29)
1.10 (0.96, 1.25)	3.53E-01	0.97 (0.86, 1.08)	7.24E-01	1.09 (0.95, 1.26)
0.95 (0.83, 1.08)	6.74E-01	1.05 (0.94, 1.19)	6.05E-01	1.02 (0.90, 1.17)
1.01 (0.90, 1.14)	9.13E-01	1.09 (0.98, 1.22)	3.10E-01	0.97 (0.85, 1.11)
0.75 (0.67, 0.83)	**1.98E-06**	0.98 (0.87, 1.11)	8.29E-01	0.88 (0.78, 1.00)
1.53 (1.36, 1.73)	**3.53E-10**	1.03 (0.91, 1.17)	7.37E-01	1.23 (1.08, 1.41)
1.53 (1.37, 1.71)	**7.78E-12**	1.04 (0.92, 1.18)	7.24E-01	1.25 (1.10, 1.42)
1.12 (0.99, 1.28)	1.90E-01	0.99 (0.88, 1.12)	9.04E-01	1.06 (0.92, 1.21)
0.90 (0.79, 1.03)	3.01E-01	0.98 (0.87, 1.10)	7.96E-01	0.86 (0.75, 1.00)
1.01 (0.89, 1.14)	9.61E-01	0.86 (0.76, 0.97)	7.51E-02	0.85 (0.74, 0.98)
1.09 (0.95, 1.24)	4.33E-01	1.10 (0.97, 1.26)	3.50E-01	1.27 (1.09, 1.49)
0.97 (0.86, 1.10)	8.13E-01	1.05 (0.93, 1.19)	6.72E-01	1.11 (0.96, 1.28)
1.27 (1.10, 1.46)	**8.52E-03**	1.15 (1.01, 1.32)	1.29E-01	1.50 (1.26, 1.78)
0.85 (0.74, 0.98)	7.80E-02	1.08 (0.96, 1.20)	4.29E-01	0.87 (0.75, 1.01)
1.13 (1.03, 1.24)	**3.48E-02**	1.25 (1.17, 1.34)	**7.52E-09**	1.22 (1.12, 1.32)
0.98 (0.87, 1.11)	8.63E-01	0.84 (0.77, 0.93)	**3.43E-03**	0.94 (0.83, 1.06)
0.99 (0.88, 1.11)	9.13E-01	0.84 (0.77, 0.91)	**1.19E-03**	0.93 (0.82, 1.04)
0.97 (0.86, 1.09)	8.13E-01	0.95 (0.85, 1.07)	6.72E-01	1.02 (0.90, 1.17)
0.89 (0.78, 1.01)	1.90E-01	0.98 (0.87, 1.11)	8.41E-01	0.84 (0.72, 0.98)
0.95 (0.84, 1.08)	6.71E-01	1.02 (0.91, 1.15)	8.08E-01	0.90 (0.79, 1.03)
1.07 (0.94, 1.21)	5.65E-01	0.98 (0.87, 1.10)	7.95E-01	1.13 (0.99, 1.30)
1.06 (0.93, 1.20)	6.50E-01	0.97 (0.87, 1.09)	7.40E-01	1.11 (0.97, 1.27)
1.05 (0.93, 1.19)	6.54E-01	1.07 (0.95, 1.21)	4.80E-01	1.07 (0.94, 1.22)
0.92 (0.81, 1.05)	4.45E-01	1.02 (0.91, 1.15)	8.00E-01	0.90 (0.78, 1.03)
0.98 (0.87, 1.11)	8.63E-01	0.94 (0.84, 1.06)	5.98E-01	0.90 (0.79, 1.03)
0.79 (0.69, 0.90)	**4.39E-03**	1.14 (1.02, 1.28)	9.20E-02	0.96 (0.84, 1.09)
1.15 (1.03, 1.30)	6.72E-02	1.26 (1.13, 1.42)	**1.12E-03**	1.28 (1.13, 1.45)
1.10 (0.98, 1.24)	2.73E-01	1.00 (0.89, 1.13)	9.99E-01	1.01 (0.88, 1.16)
1.15 (1.03, 1.30)	6.59E-02	1.01 (0.89, 1.14)	9.10E-01	0.97 (0.84, 1.11)
1.12 (0.99, 1.26)	1.86E-01	0.99 (0.88, 1.12)	9.10E-01	0.97 (0.85, 1.12)
1.06 (0.93, 1.19)	6.48E-01	0.94 (0.83, 1.06)	5.64E-01	0.99 (0.87, 1.14)
1.06 (0.94, 1.19)	6.24E-01	1.11 (0.99, 1.24)	2.55E-01	1.07 (0.94, 1.21)
1.03 (0.91, 1.16)	8.13E-01	1.09 (0.97, 1.22)	3.60E-01	1.04 (0.91, 1.18)
1.33 (1.19, 1.49)	**1.61E-05**	1.24 (1.11, 1.40)	**2.93E-03**	1.29 (1.14, 1.46)
0.83 (0.72, 0.95)	**3.04E-02**	0.91 (0.80, 1.03)	3.51E-01	0.76 (0.66, 0.89)
0.96 (0.84, 1.10)	7.61E-01	0.81 (0.71, 0.92)	**7.50E-03**	0.92 (0.79, 1.06)
1.04 (0.92, 1.18)	6.94E-01	0.99 (0.88, 1.11)	8.86E-01	0.87 (0.75, 1.01)
1.06 (0.94, 1.20)	6.17E-01	0.97 (0.86, 1.10)	7.43E-01	0.87 (0.75, 1.00)
1.02 (0.90, 1.15)	8.63E-01	1.10 (0.98, 1.22)	2.66E-01	0.99 (0.86, 1.14)
0.87 (0.76, 0.99)	1.15E-01	1.06 (0.95, 1.19)	5.61E-01	0.84 (0.72, 0.97)

1.07 (0.94, 1.21)	5.72E-01	0.90 (0.80, 1.02)	2.66E-01	0.96 (0.84, 1.10)
1.08 (0.96, 1.22)	4.29E-01	0.96 (0.85, 1.09)	7.24E-01	0.93 (0.81, 1.06)
0.86 (0.75, 0.97)	6.99E-02	0.80 (0.71, 0.90)	**2.20E-03**	0.81 (0.70, 0.93)
0.82 (0.72, 0.94)	**2.63E-02**	0.76 (0.67, 0.85)	**8.93E-05**	0.82 (0.71, 0.95)
1.00 (0.88, 1.13)	9.89E-01	0.87 (0.77, 0.97)	6.86E-02	0.94 (0.82, 1.08)
1.12 (0.99, 1.26)	1.78E-01	1.10 (0.98, 1.24)	2.94E-01	1.05 (0.92, 1.19)
1.08 (0.96, 1.22)	4.33E-01	0.95 (0.84, 1.07)	6.05E-01	0.92 (0.79, 1.06)
1.02 (0.90, 1.15)	8.67E-01	0.91 (0.80, 1.03)	3.45E-01	0.90 (0.78, 1.03)
1.02 (0.90, 1.16)	8.63E-01	0.85 (0.75, 0.96)	5.24E-02	0.84 (0.72, 0.97)
0.72 (0.63, 0.83)	**3.65E-05**	0.73 (0.67, 0.79)	**1.69E-10**	0.77 (0.67, 0.88)
1.51 (1.36, 1.68)	**3.31E-12**	1.07 (0.95, 1.21)	5.37E-01	1.38 (1.22, 1.56)
1.03 (0.90, 1.18)	8.13E-01	0.82 (0.73, 0.92)	**6.71E-03**	0.86 (0.74, 1.00)
1.28 (1.15, 1.43)	**1.54E-04**	0.94 (0.83, 1.07)	6.05E-01	1.11 (0.98, 1.27)
0.95 (0.83, 1.09)	6.71E-01	0.80 (0.71, 0.91)	**4.23E-03**	0.78 (0.67, 0.92)
1.01 (0.89, 1.16)	9.09E-01	0.81 (0.71, 0.92)	**9.49E-03**	0.83 (0.71, 0.97)
1.02 (0.90, 1.16)	8.39E-01	0.91 (0.80, 1.03)	3.26E-01	0.87 (0.75, 1.00)
1.02 (0.90, 1.16)	8.63E-01	0.84 (0.74, 0.96)	**4.40E-02**	0.83 (0.72, 0.96)
1.80 (1.58, 2.06)	**3.10E-16**	1.29 (1.14, 1.45)	**6.42E-04**	1.75 (1.51, 2.01)
1.01 (0.88, 1.15)	9.58E-01	0.85 (0.77, 0.94)	**8.42E-03**	1.02 (0.89, 1.17)
1.47 (1.31, 1.64)	**5.42E-10**	1.09 (0.97, 1.23)	3.74E-01	1.38 (1.23, 1.56)
0.85 (0.75, 0.97)	5.22E-02	0.85 (0.76, 0.95)	**3.00E-02**	0.89 (0.78, 1.02)
0.97 (0.85, 1.11)	8.33E-01	0.86 (0.77, 0.97)	5.75E-02	0.99 (0.87, 1.14)
1.01 (0.89, 1.15)	8.97E-01	1.13 (1.01, 1.27)	1.40E-01	1.03 (0.90, 1.17)
1.03 (0.90, 1.17)	8.13E-01	1.08 (0.96, 1.22)	4.29E-01	0.98 (0.86, 1.12)
1.16 (1.07, 1.26)	**2.60E-03**	1.28 (1.19, 1.38)	**7.78E-09**	1.11 (1.01, 1.22)
1.19 (1.07, 1.33)	**8.52E-03**	1.09 (0.98, 1.22)	3.06E-01	0.99 (0.87, 1.13)
0.82 (0.72, 0.94)	**2.37E-02**	0.96 (0.85, 1.10)	7.24E-01	0.71 (0.61, 0.82)
1.12 (0.99, 1.26)	1.90E-01	0.93 (0.82, 1.05)	4.61E-01	1.02 (0.90, 1.16)
1.03 (0.91, 1.16)	8.13E-01	0.93 (0.82, 1.05)	4.68E-01	1.14 (1.00, 1.29)
1.19 (1.06, 1.35)	**2.40E-02**	1.03 (0.92, 1.16)	7.28E-01	1.28 (1.13, 1.45)
1.02 (0.90, 1.16)	8.49E-01	0.98 (0.87, 1.11)	8.47E-01	0.80 (0.70, 0.93)
1.17 (1.05, 1.31)	**2.65E-02**	1.00 (0.89, 1.13)	9.69E-01	0.90 (0.79, 1.03)
0.86 (0.75, 0.98)	8.43E-02	0.78 (0.69, 0.89)	**2.84E-03**	0.74 (0.64, 0.85)
1.19 (1.13, 1.26)	**3.19E-08**	1.15 (1.08, 1.22)	**2.57E-04**	1.17 (1.10, 1.25)
1.12 (0.99, 1.25)	1.83E-01	1.14 (1.01, 1.27)	1.22E-01	1.10 (0.97, 1.25)
1.03 (0.95, 1.11)	6.99E-01	1.20 (1.12, 1.29)	**2.25E-05**	1.00 (0.91, 1.09)
0.85 (0.74, 0.98)	8.67E-02	1.09 (1.00, 1.19)	1.56E-01	0.74 (0.63, 0.87)
0.97 (0.84, 1.12)	8.13E-01	1.09 (0.99, 1.21)	2.37E-01	0.80 (0.66, 0.97)
1.09 (1.04, 1.15)	**2.43E-03**	1.08 (1.03, 1.13)	**8.42E-03**	1.05 (0.96, 1.15)
0.72 (0.65, 0.79)	**1.26E-09**	0.76 (0.69, 0.83)	**2.02E-07**	0.78 (0.70, 0.87)
1.46 (1.32, 1.62)	**2.18E-11**	1.21 (1.07, 1.35)	**9.84E-03**	1.16 (1.03, 1.32)

	Cancer mortality			
	Male		Female	
P value	Adjusted HR (95% CI)	P value	Adjusted HR (95% CI)	P value
**2.17E-02**	0.89 (0.78, 1.01)	2.46E-01	0.98 (0.88, 1.09)	9.80E-01
**2.42E-02**	0.89 (0.78, 1.02)	2.58E-01	0.98 (0.88, 1.09)	9.80E-01
**3.79E-02**	0.90 (0.79, 1.03)	3.31E-01	0.97 (0.87, 1.08)	9.80E-01
8.93E-02	0.93 (0.82, 1.05)	5.06E-01	1.02 (0.92, 1.13)	9.80E-01
1.29E-01	0.95 (0.84, 1.07)	5.41E-01	1.03 (0.93, 1.14)	9.80E-01
5.44E-02	0.92 (0.81, 1.05)	4.50E-01	1.01 (0.91, 1.12)	9.80E-01
**1.57E-02**	0.88 (0.77, 1.00)	1.97E-01	0.97 (0.87, 1.08)	9.80E-01
6.03E-02	0.94 (0.82, 1.07)	5.38E-01	1.03 (0.93, 1.14)	9.80E-01
6.11E-02	0.94 (0.82, 1.07)	5.38E-01	1.03 (0.93, 1.14)	9.80E-01
**4.99E-02**	0.94 (0.83, 1.07)	5.41E-01	1.02 (0.92, 1.14)	9.80E-01
9.68E-02	0.94 (0.83, 1.07)	5.41E-01	1.04 (0.94, 1.15)	9.80E-01
1.33E-01	0.94 (0.83, 1.07)	5.38E-01	1.04 (0.94, 1.15)	9.80E-01
6.26E-02	0.95 (0.83, 1.07)	5.58E-01	1.04 (0.94, 1.15)	9.80E-01
5.71E-02	0.94 (0.82, 1.06)	5.38E-01	1.02 (0.92, 1.14)	9.80E-01
9.38E-02	0.91 (0.80, 1.03)	3.20E-01	1.00 (0.90, 1.10)	9.80E-01
9.28E-02	0.91 (0.80, 1.03)	3.29E-01	1.00 (0.90, 1.10)	9.80E-01
8.93E-02	0.91 (0.80, 1.03)	3.45E-01	1.00 (0.90, 1.10)	9.80E-01
7.42E-02	0.92 (0.82, 1.04)	4.25E-01	1.01 (0.91, 1.11)	9.80E-01
6.26E-02	0.93 (0.82, 1.05)	4.92E-01	1.01 (0.92, 1.12)	9.80E-01
9.28E-02	0.92 (0.81, 1.04)	3.98E-01	1.00 (0.91, 1.11)	9.80E-01
1.02E-01	0.90 (0.79, 1.02)	3.00E-01	0.99 (0.90, 1.10)	9.80E-01
1.20E-01	0.88 (0.78, 0.99)	1.45E-01	1.01 (0.92, 1.12)	9.80E-01
1.18E-01	0.88 (0.78, 0.99)	1.55E-01	1.01 (0.92, 1.12)	9.80E-01
1.04E-01	0.88 (0.79, 0.99)	1.59E-01	1.01 (0.91, 1.12)	9.80E-01
1.10E-01	0.91 (0.81, 1.02)	3.07E-01	1.03 (0.93, 1.14)	9.80E-01
9.48E-02	0.93 (0.83, 1.04)	4.58E-01	1.04 (0.94, 1.14)	9.80E-01
1.51E-01	0.89 (0.79, 1.00)	2.02E-01	1.02 (0.92, 1.13)	9.80E-01
1.33E-01	0.87 (0.77, 0.98)	1.19E-01	1.01 (0.91, 1.11)	9.80E-01
3.03E-01	0.91 (0.81, 1.01)	2.58E-01	1.01 (0.92, 1.12)	9.80E-01
4.00E-01	0.91 (0.81, 1.02)	2.80E-01	1.01 (0.92, 1.11)	9.80E-01
1.99E-01	0.91 (0.82, 1.02)	3.08E-01	1.00 (0.90, 1.10)	9.80E-01
5.86E-01	0.95 (0.85, 1.05)	5.38E-01	1.01 (0.92, 1.11)	9.80E-01
2.48E-01	0.96 (0.87, 1.07)	6.39E-01	1.01 (0.92, 1.11)	9.80E-01
8.03E-01	0.92 (0.83, 1.03)	3.81E-01	1.01 (0.92, 1.11)	9.80E-01
1.04E-01	0.90 (0.80, 1.00)	2.02E-01	1.02 (0.92, 1.12)	9.80E-01
6.61E-01	0.99 (0.89, 1.10)	9.26E-01	0.99 (0.90, 1.09)	9.80E-01
4.37E-01	0.99 (0.89, 1.10)	9.31E-01	0.99 (0.90, 1.09)	9.80E-01
9.70E-01	1.02 (0.92, 1.14)	8.13E-01	0.99 (0.89, 1.09)	9.80E-01
**4.05E-02**	0.99 (0.89, 1.10)	9.26E-01	0.97 (0.88, 1.07)	9.80E-01
**4.62E-02**	0.99 (0.89, 1.10)	9.26E-01	0.97 (0.88, 1.06)	9.80E-01

**4.51E-02**	0.99 (0.89, 1.10)	9.38E-01	0.98 (0.90, 1.08)	9.80E-01
3.43E-01	0.96 (0.86, 1.07)	6.39E-01	1.02 (0.93, 1.13)	9.80E-01
8.04E-01	1.02 (0.92, 1.13)	8.50E-01	1.00 (0.90, 1.10)	9.80E-01
8.43E-01	1.02 (0.92, 1.13)	8.58E-01	1.00 (0.90, 1.10)	9.80E-01
7.74E-01	1.02 (0.91, 1.13)	8.58E-01	0.99 (0.90, 1.09)	9.80E-01
9.14E-01	1.01 (0.91, 1.13)	8.99E-01	1.00 (0.91, 1.09)	9.80E-01
7.25E-01	1.00 (0.90, 1.12)	9.85E-01	0.99 (0.90, 1.09)	9.80E-01
6.53E-01	1.04 (0.93, 1.16)	6.33E-01	1.00 (0.91, 1.10)	9.80E-01
8.27E-01	1.06 (0.95, 1.18)	5.38E-01	1.02 (0.92, 1.12)	9.80E-01
7.51E-01	1.01 (0.91, 1.12)	9.38E-01	0.98 (0.88, 1.08)	9.80E-01
7.74E-01	1.01 (0.90, 1.12)	9.45E-01	0.98 (0.88, 1.08)	9.80E-01
4.15E-01	1.01 (0.90, 1.12)	9.49E-01	0.98 (0.88, 1.08)	9.80E-01
8.26E-01	1.00 (0.90, 1.11)	9.98E-01	0.97 (0.88, 1.07)	9.80E-01
7.59E-01	0.99 (0.89, 1.10)	9.27E-01	0.97 (0.88, 1.07)	9.80E-01
9.57E-01	1.03 (0.93, 1.15)	7.00E-01	0.98 (0.89, 1.08)	9.80E-01
9.14E-01	1.09 (0.98, 1.22)	3.07E-01	1.02 (0.92, 1.12)	9.80E-01
7.12E-01	0.96 (0.86, 1.07)	6.39E-01	0.96 (0.87, 1.07)	9.80E-01
7.18E-01	0.96 (0.86, 1.07)	6.39E-01	0.96 (0.87, 1.07)	9.80E-01
6.40E-02	0.95 (0.85, 1.06)	5.41E-01	0.97 (0.87, 1.07)	9.80E-01
9.33E-01	0.96 (0.86, 1.07)	6.14E-01	0.96 (0.87, 1.06)	9.80E-01
9.90E-01	0.96 (0.86, 1.07)	6.01E-01	0.96 (0.87, 1.06)	9.80E-01
6.03E-01	0.96 (0.86, 1.08)	6.39E-01	0.97 (0.87, 1.07)	9.80E-01
9.70E-01	1.07 (0.95, 1.19)	5.06E-01	1.01 (0.92, 1.12)	9.80E-01
7.74E-01	0.94 (0.85, 1.06)	5.38E-01	0.96 (0.86, 1.06)	9.80E-01
8.03E-01	0.94 (0.85, 1.06)	5.38E-01	0.95 (0.86, 1.06)	9.80E-01
6.34E-02	0.94 (0.84, 1.05)	5.38E-01	0.96 (0.86, 1.07)	9.80E-01
9.14E-01	0.95 (0.85, 1.06)	5.38E-01	0.95 (0.86, 1.05)	9.46E-01
8.45E-01	0.95 (0.85, 1.06)	5.41E-01	0.95 (0.86, 1.05)	8.57E-01
8.03E-01	0.94 (0.84, 1.05)	5.06E-01	0.95 (0.86, 1.05)	9.80E-01
5.85E-01	0.96 (0.86, 1.08)	6.83E-01	1.02 (0.92, 1.13)	9.80E-01
**4.62E-03**	1.12 (1.02, 1.23)	1.11E-01	1.12 (1.02, 1.22)	2.03E-01
**4.62E-03**	1.11 (1.01, 1.22)	1.20E-01	1.12 (1.02, 1.22)	2.11E-01
**2.13E-02**	1.17 (1.07, 1.28)	**9.22E-03**	1.14 (1.04, 1.24)	1.26E-01
**1.37E-03**	1.05 (0.96, 1.16)	5.38E-01	1.08 (0.99, 1.19)	4.56E-01
**5.65E-04**	1.03 (0.94, 1.13)	7.00E-01	1.07 (0.97, 1.17)	6.42E-01
**1.39E-02**	1.11 (1.01, 1.22)	1.20E-01	1.12 (1.03, 1.23)	1.73E-01
1.90E-01	1.00 (0.90, 1.12)	9.85E-01	1.11 (1.01, 1.22)	2.68E-01
9.12E-02	1.15 (1.05, 1.27)	**2.82E-02**	1.11 (1.01, 1.22)	2.68E-01
8.44E-02	1.15 (1.05, 1.27)	**2.82E-02**	1.11 (1.01, 1.22)	2.68E-01
1.08E-01	1.15 (1.04, 1.27)	**3.31E-02**	1.09 (0.99, 1.20)	4.48E-01
6.34E-02	1.15 (1.05, 1.27)	**2.82E-02**	1.11 (1.01, 1.23)	2.49E-01
8.93E-02	1.15 (1.05, 1.27)	**2.82E-02**	1.12 (1.01, 1.23)	2.49E-01
**2.41E-02**	1.15 (1.04, 1.26)	**3.31E-02**	1.11 (1.01, 1.22)	2.63E-01
4.00E-01	1.13 (1.02, 1.24)	9.30E-02	1.11 (1.01, 1.23)	2.49E-01
**8.55E-04**	1.06 (0.95, 1.18)	5.38E-01	0.98 (0.89, 1.09)	9.80E-01
**1.48E-03**	1.06 (0.95, 1.18)	5.38E-01	0.98 (0.89, 1.09)	9.80E-01
**7.04E-03**	1.07 (0.96, 1.18)	4.93E-01	0.97 (0.88, 1.08)	9.80E-01
**6.54E-03**	1.05 (0.94, 1.17)	5.49E-01	0.99 (0.90, 1.10)	9.80E-01

**4.11E-03**	1.04 (0.93, 1.17)	6.30E-01	0.99 (0.90, 1.10)	9.80E-01
**4.62E-02**	1.08 (0.97, 1.20)	4.11E-01	0.99 (0.90, 1.09)	9.80E-01
**1.28E-05**	1.01 (0.91, 1.12)	9.49E-01	1.01 (0.91, 1.12)	9.80E-01
**4.69E-07**	1.00 (0.90, 1.12)	9.85E-01	0.91 (0.83, 1.01)	4.54E-01
**9.25E-07**	1.01 (0.90, 1.12)	9.63E-01	0.91 (0.83, 1.01)	4.54E-01
**2.73E-05**	0.98 (0.87, 1.09)	8.13E-01	0.93 (0.85, 1.03)	6.47E-01
7.42E-02	1.05 (0.94, 1.18)	5.41E-01	0.94 (0.85, 1.04)	7.95E-01
2.40E-01	1.05 (0.94, 1.18)	5.41E-01	0.95 (0.85, 1.05)	8.49E-01
**1.07E-03**	0.99 (0.89, 1.11)	9.60E-01	0.96 (0.87, 1.06)	9.80E-01
**2.24E-03**	0.96 (0.86, 1.07)	6.39E-01	0.99 (0.89, 1.09)	9.80E-01
9.14E-01	0.85 (0.78, 0.94)	**9.22E-03**	0.88 (0.80, 0.98)	2.03E-01
**1.32E-04**	1.20 (1.10, 1.30)	**7.53E-04**	1.13 (1.03, 1.24)	1.84E-01
**1.72E-04**	1.20 (1.10, 1.30)	**8.03E-04**	1.12 (1.01, 1.23)	2.49E-01
1.47E-01	1.08 (0.97, 1.20)	4.10E-01	1.09 (0.97, 1.22)	5.95E-01
**1.82E-04**	0.90 (0.80, 1.00)	1.93E-01	0.93 (0.84, 1.04)	7.68E-01
1.40E-01	0.95 (0.86, 1.05)	5.38E-01	0.92 (0.82, 1.02)	5.11E-01
**3.46E-02**	1.09 (1.01, 1.19)	1.53E-01	1.03 (0.95, 1.11)	9.80E-01
**5.46E-03**	1.08 (0.98, 1.20)	3.08E-01	1.04 (0.96, 1.12)	9.80E-01
7.51E-01	1.06 (0.96, 1.18)	4.92E-01	0.98 (0.88, 1.10)	9.80E-01
5.44E-01	0.95 (0.85, 1.06)	5.38E-01	1.01 (0.91, 1.12)	9.80E-01
8.03E-01	0.98 (0.88, 1.09)	7.98E-01	0.92 (0.82, 1.04)	6.56E-01
6.69E-01	1.06 (0.94, 1.19)	5.38E-01	1.05 (0.93, 1.18)	9.80E-01
6.17E-01	1.20 (1.08, 1.33)	**9.12E-03**	1.10 (1.00, 1.21)	3.42E-01
9.60E-01	0.91 (0.82, 1.00)	2.02E-01	0.94 (0.84, 1.05)	8.33E-01
6.70E-01	0.96 (0.86, 1.08)	6.52E-01	0.98 (0.87, 1.11)	9.80E-01
1.96E-01	1.17 (1.07, 1.28)	**6.66E-03**	0.99 (0.89, 1.10)	9.80E-01
9.57E-01	1.21 (1.09, 1.34)	**4.75E-03**	1.12 (1.01, 1.24)	2.49E-01
7.77E-01	1.22 (1.10, 1.34)	**1.64E-03**	1.10 (1.00, 1.21)	3.21E-01
3.70E-01	0.95 (0.87, 1.04)	5.14E-01	1.02 (0.91, 1.14)	9.80E-01
8.27E-01	0.82 (0.74, 0.91)	**1.69E-03**	0.90 (0.81, 1.00)	3.07E-01
**1.57E-02**	1.10 (1.01, 1.21)	1.28E-01	0.96 (0.86, 1.07)	9.80E-01
**5.90E-03**	1.06 (0.96, 1.17)	4.94E-01	1.01 (0.92, 1.11)	9.80E-01
**1.57E-02**	1.05 (0.95, 1.16)	5.38E-01	1.01 (0.91, 1.11)	9.80E-01
**2.70E-04**	1.09 (0.98, 1.20)	2.81E-01	1.03 (0.93, 1.14)	9.80E-01
**3.80E-02**	0.91 (0.83, 1.01)	2.56E-01	0.99 (0.90, 1.10)	9.80E-01
**4.98E-02**	1.09 (0.97, 1.22)	3.39E-01	1.05 (0.94, 1.17)	9.80E-01
**1.32E-04**	0.94 (0.86, 1.04)	5.01E-01	0.96 (0.87, 1.05)	9.80E-01
**2.52E-03**	0.94 (0.86, 1.04)	5.03E-01	0.95 (0.86, 1.04)	8.45E-01
**1.82E-04**	0.97 (0.87, 1.07)	6.39E-01	1.00 (0.91, 1.11)	9.80E-01
6.01E-02	1.01 (0.91, 1.12)	9.27E-01	1.02 (0.92, 1.12)	9.80E-01
5.10E-01	1.00 (0.90, 1.12)	9.85E-01	0.96 (0.86, 1.06)	9.80E-01
5.86E-01	0.95 (0.87, 1.04)	4.93E-01	1.01 (0.92, 1.11)	9.80E-01
3.51E-01	0.90 (0.82, 0.99)	1.45E-01	0.98 (0.89, 1.08)	9.80E-01
**4.62E-02**	1.03 (0.92, 1.15)	7.25E-01	1.03 (0.93, 1.15)	9.80E-01
7.74E-01	1.06 (0.96, 1.17)	4.93E-01	1.00 (0.91, 1.11)	9.80E-01
2.70E-01	1.01 (0.90, 1.12)	9.49E-01	1.00 (0.89, 1.11)	9.80E-01
7.18E-01	0.95 (0.86, 1.05)	5.41E-01	0.98 (0.89, 1.09)	9.80E-01
9.14E-01	0.94 (0.85, 1.03)	4.24E-01	0.97 (0.87, 1.07)	9.80E-01

6.11E-02	1.07 (0.95, 1.20)	5.06E-01	1.04 (0.93, 1.16)	9.80E-01
9.07E-01	1.07 (0.97, 1.18)	4.11E-01	1.03 (0.93, 1.13)	9.80E-01
9.28E-02	1.02 (0.92, 1.14)	7.98E-01	1.02 (0.92, 1.12)	9.80E-01
2.74E-01	0.95 (0.85, 1.05)	5.38E-01	0.97 (0.88, 1.08)	9.80E-01
4.37E-01	0.95 (0.85, 1.06)	5.38E-01	0.97 (0.88, 1.08)	9.80E-01
9.70E-01	1.03 (0.92, 1.15)	7.27E-01	1.03 (0.93, 1.16)	9.80E-01
8.56E-01	1.12 (1.01, 1.24)	1.57E-01	1.04 (0.94, 1.15)	9.80E-01
9.28E-02	1.05 (0.95, 1.17)	5.41E-01	1.03 (0.93, 1.14)	9.80E-01
1.99E-01	0.95 (0.86, 1.06)	5.41E-01	0.97 (0.87, 1.07)	9.80E-01
3.23E-01	0.96 (0.86, 1.06)	5.58E-01	0.96 (0.87, 1.07)	9.80E-01
8.27E-01	1.02 (0.91, 1.14)	8.62E-01	1.03 (0.92, 1.15)	9.80E-01
8.13E-01	1.04 (0.93, 1.16)	6.26E-01	1.04 (0.94, 1.15)	9.80E-01
9.68E-02	1.26 (1.13, 1.39)	**5.41E-04**	1.10 (1.00, 1.21)	3.42E-01
**1.04E-02**	0.81 (0.74, 0.89)	**5.41E-04**	0.90 (0.82, 0.99)	2.49E-01
**4.80E-03**	0.78 (0.70, 0.86)	**3.71E-05**	0.88 (0.80, 0.96)	1.26E-01
5.85E-01	1.00 (0.91, 1.10)	9.98E-01	1.03 (0.94, 1.13)	9.80E-01
1.13E-01	0.91 (0.82, 1.02)	3.16E-01	1.00 (0.91, 1.10)	9.87E-01
6.34E-02	0.89 (0.80, 1.00)	1.70E-01	0.89 (0.81, 0.99)	2.49E-01
**1.29E-02**	1.10 (0.99, 1.22)	2.74E-01	1.07 (0.97, 1.18)	5.99E-01
2.69E-01	1.11 (0.99, 1.24)	2.25E-01	1.09 (0.98, 1.20)	4.89E-01
**9.05E-05**	1.06 (0.96, 1.18)	4.93E-01	1.05 (0.95, 1.15)	9.80E-01
1.46E-01	1.00 (0.90, 1.11)	9.96E-01	1.03 (0.94, 1.13)	9.80E-01
**8.88E-05**	1.15 (1.02, 1.28)	1.09E-01	0.92 (0.82, 1.03)	5.80E-01
4.62E-01	0.92 (0.82, 1.03)	3.81E-01	1.03 (0.93, 1.13)	9.80E-01
3.35E-01	0.89 (0.79, 1.00)	1.66E-01	1.03 (0.93, 1.14)	9.80E-01
8.26E-01	1.05 (0.94, 1.17)	5.41E-01	1.00 (0.90, 1.11)	9.94E-01
6.34E-02	1.01 (0.91, 1.13)	9.26E-01	1.02 (0.92, 1.12)	9.80E-01
2.22E-01	0.94 (0.84, 1.06)	5.38E-01	1.01 (0.91, 1.11)	9.80E-01
1.51E-01	1.06 (0.94, 1.19)	5.38E-01	0.99 (0.89, 1.09)	9.80E-01
2.37E-01	1.06 (0.94, 1.19)	5.38E-01	0.98 (0.89, 1.09)	9.80E-01
4.36E-01	0.96 (0.86, 1.07)	6.25E-01	1.08 (0.97, 1.20)	5.91E-01
2.17E-01	0.98 (0.88, 1.09)	8.30E-01	1.02 (0.92, 1.12)	9.80E-01
1.99E-01	0.84 (0.75, 0.93)	**9.91E-03**	0.95 (0.86, 1.06)	9.80E-01
6.67E-01	1.23 (1.11, 1.36)	**1.64E-03**	1.13 (1.02, 1.25)	2.03E-01
**1.07E-03**	1.11 (1.01, 1.22)	1.45E-01	1.11 (1.01, 1.22)	2.68E-01
9.44E-01	1.00 (0.90, 1.11)	9.93E-01	1.01 (0.92, 1.11)	9.80E-01
7.74E-01	0.91 (0.81, 1.01)	2.73E-01	1.01 (0.92, 1.11)	9.80E-01
8.04E-01	0.94 (0.84, 1.05)	5.14E-01	1.00 (0.91, 1.11)	9.80E-01
9.60E-01	0.97 (0.87, 1.08)	6.78E-01	0.97 (0.88, 1.07)	9.80E-01
4.62E-01	1.12 (1.01, 1.24)	1.21E-01	1.08 (0.98, 1.18)	5.81E-01
7.18E-01	1.13 (1.02, 1.24)	1.07E-01	1.08 (0.98, 1.19)	5.45E-01
**7.27E-04**	1.04 (0.94, 1.14)	6.25E-01	1.04 (0.95, 1.14)	9.80E-01
**2.77E-03**	1.01 (0.91, 1.13)	9.26E-01	0.99 (0.90, 1.10)	9.80E-01
3.70E-01	0.99 (0.88, 1.11)	9.27E-01	1.00 (0.91, 1.11)	9.80E-01
1.23E-01	0.90 (0.80, 1.01)	2.26E-01	1.01 (0.91, 1.12)	9.80E-01
1.12E-01	0.87 (0.77, 0.98)	1.20E-01	1.00 (0.91, 1.11)	9.80E-01
9.57E-01	1.06 (0.95, 1.18)	5.38E-01	1.02 (0.92, 1.12)	9.80E-01
5.63E-02	1.03 (0.92, 1.14)	7.43E-01	1.06 (0.96, 1.17)	8.45E-01

7.28E-01	1.03 (0.93, 1.15)	7.02E-01	1.09 (0.99, 1.20)	4.66E-01
4.28E-01	0.88 (0.78, 0.98)	1.20E-01	1.02 (0.92, 1.13)	9.80E-01
**1.74E-02**	1.02 (0.91, 1.13)	8.79E-01	1.04 (0.94, 1.15)	9.80E-01
**3.97E-02**	1.00 (0.90, 1.12)	9.85E-01	0.92 (0.82, 1.02)	5.45E-01
5.85E-01	1.06 (0.95, 1.18)	5.38E-01	1.06 (0.96, 1.17)	8.40E-01
6.45E-01	1.07 (0.97, 1.18)	4.50E-01	1.08 (0.98, 1.19)	5.11E-01
3.64E-01	0.92 (0.82, 1.03)	3.41E-01	1.00 (0.91, 1.11)	9.80E-01
2.17E-01	0.90 (0.81, 1.00)	1.93E-01	0.97 (0.87, 1.07)	9.80E-01
5.93E-02	0.95 (0.85, 1.06)	5.41E-01	1.06 (0.96, 1.17)	7.99E-01
**1.85E-03**	0.98 (0.87, 1.09)	8.16E-01	0.99 (0.89, 1.11)	9.80E-01
**1.14E-05**	0.84 (0.75, 0.94)	**1.37E-02**	0.95 (0.86, 1.04)	8.24E-01
1.04E-01	0.93 (0.83, 1.04)	4.49E-01	1.07 (0.97, 1.19)	6.42E-01
1.99E-01	0.81 (0.72, 0.92)	**9.22E-03**	0.98 (0.89, 1.08)	9.80E-01
**1.19E-02**	0.96 (0.85, 1.07)	6.16E-01	1.07 (0.97, 1.19)	6.47E-01
6.03E-02	0.93 (0.82, 1.04)	4.36E-01	1.06 (0.95, 1.17)	8.49E-01
1.10E-01	0.93 (0.83, 1.05)	5.03E-01	1.03 (0.93, 1.14)	9.80E-01
**4.66E-02**	0.99 (0.89, 1.11)	9.45E-01	1.09 (0.99, 1.20)	4.58E-01
**3.17E-12**	0.82 (0.74, 0.91)	**2.12E-03**	0.87 (0.78, 0.96)	1.25E-01
8.34E-01	0.98 (0.86, 1.11)	8.30E-01	1.03 (0.92, 1.14)	9.80E-01
**6.17E-06**	0.76 (0.68, 0.84)	**2.48E-05**	0.87 (0.79, 0.96)	1.28E-01
1.54E-01	1.06 (0.95, 1.18)	5.38E-01	1.03 (0.93, 1.15)	9.80E-01
9.57E-01	0.97 (0.87, 1.08)	6.97E-01	0.98 (0.88, 1.10)	9.80E-01
8.21E-01	0.91 (0.82, 1.01)	2.57E-01	0.91 (0.82, 1.01)	4.54E-01
8.31E-01	1.18 (1.07, 1.31)	**1.06E-02**	1.18 (1.05, 1.32)	1.19E-01
9.28E-02	0.86 (0.76, 0.98)	1.19E-01	1.08 (0.99, 1.17)	4.66E-01
9.44E-01	0.77 (0.68, 0.87)	**5.41E-04**	0.79 (0.70, 0.89)	**8.63E-03**
**5.28E-05**	1.26 (1.13, 1.40)	**6.13E-04**	1.26 (1.14, 1.41)	**8.98E-04**
8.26E-01	0.76 (0.68, 0.85)	**3.71E-05**	0.85 (0.77, 0.95)	1.19E-01
9.91E-02	0.81 (0.72, 0.90)	**1.45E-03**	0.78 (0.70, 0.87)	**7.72E-04**
**1.13E-03**	0.73 (0.66, 0.82)	**1.95E-05**	0.75 (0.67, 0.84)	**1.67E-04**
**1.39E-02**	0.85 (0.76, 0.95)	**3.31E-02**	0.94 (0.85, 1.05)	8.49E-01
2.30E-01	0.82 (0.73, 0.92)	**9.47E-03**	0.87 (0.78, 0.96)	1.28E-01
**3.98E-04**	0.81 (0.72, 0.91)	**4.73E-03**	0.85 (0.76, 0.95)	1.22E-01
**3.49E-05**	0.89 (0.79, 1.00)	1.77E-01	0.83 (0.74, 0.93)	6.77E-02
2.17E-01	0.85 (0.76, 0.95)	**3.31E-02**	0.90 (0.81, 1.00)	2.78E-01
9.70E-01	0.97 (0.86, 1.08)	7.10E-01	0.88 (0.74, 1.05)	5.99E-01
**1.53E-03**	1.15 (1.07, 1.25)	**4.30E-03**	1.15 (1.05, 1.25)	6.88E-02
6.34E-02	1.14 (1.07, 1.22)	**8.36E-04**	0.97 (0.87, 1.09)	9.80E-01
3.93E-01	0.64 (0.48, 0.84)	**1.06E-02**	0.85 (0.71, 1.01)	3.42E-01
**1.82E-04**	1.11 (0.99, 1.23)	2.26E-01	1.08 (0.97, 1.20)	6.35E-01
5.71E-02	0.94 (0.84, 1.06)	5.38E-01	1.07 (0.97, 1.19)	6.56E-01

Other mortality			
Male		Female	
Adjusted HR (95% CI)	P value	Adjusted HR (95% CI)	P value
0.96 (0.78, 1.17)	9.96E-01	1.08 (0.88, 1.32)	8.22E-01
0.96 (0.79, 1.18)	9.96E-01	1.08 (0.88, 1.32)	8.22E-01
0.97 (0.80, 1.19)	9.96E-01	1.08 (0.89, 1.32)	8.22E-01
1.04 (0.86, 1.26)	9.96E-01	1.06 (0.87, 1.30)	8.84E-01
1.07 (0.89, 1.29)	8.79E-01	1.03 (0.84, 1.27)	9.31E-01
1.01 (0.83, 1.23)	9.96E-01	1.09 (0.89, 1.33)	8.22E-01
0.95 (0.77, 1.16)	9.66E-01	1.08 (0.88, 1.32)	8.22E-01
0.98 (0.80, 1.20)	9.96E-01	1.05 (0.86, 1.29)	8.86E-01
0.98 (0.80, 1.20)	9.96E-01	1.05 (0.86, 1.29)	8.86E-01
1.00 (0.82, 1.22)	9.96E-01	1.07 (0.87, 1.31)	8.80E-01
1.01 (0.83, 1.22)	9.96E-01	1.04 (0.85, 1.28)	9.31E-01
1.01 (0.83, 1.23)	9.96E-01	1.02 (0.83, 1.26)	9.56E-01
1.01 (0.83, 1.23)	9.96E-01	1.06 (0.86, 1.30)	8.84E-01
0.97 (0.79, 1.19)	9.96E-01	1.06 (0.86, 1.29)	8.85E-01
1.00 (0.82, 1.22)	9.96E-01	1.01 (0.82, 1.25)	9.62E-01
1.00 (0.82, 1.22)	9.96E-01	1.01 (0.82, 1.24)	9.62E-01
1.01 (0.83, 1.23)	9.96E-01	1.01 (0.83, 1.25)	9.59E-01
1.01 (0.84, 1.23)	9.96E-01	0.99 (0.81, 1.22)	9.66E-01
1.01 (0.83, 1.22)	9.96E-01	0.96 (0.78, 1.19)	9.31E-01
1.02 (0.84, 1.24)	9.96E-01	1.03 (0.84, 1.26)	9.47E-01
0.99 (0.82, 1.21)	9.96E-01	1.02 (0.83, 1.25)	9.56E-01
0.98 (0.80, 1.18)	9.96E-01	0.97 (0.79, 1.20)	9.31E-01
0.98 (0.81, 1.19)	9.96E-01	0.97 (0.78, 1.19)	9.31E-01
0.98 (0.81, 1.19)	9.96E-01	0.96 (0.78, 1.19)	9.31E-01
1.01 (0.83, 1.22)	9.96E-01	0.94 (0.76, 1.17)	8.85E-01
1.01 (0.84, 1.22)	9.96E-01	0.91 (0.74, 1.14)	8.22E-01
1.00 (0.83, 1.21)	9.96E-01	0.98 (0.80, 1.21)	9.56E-01
0.97 (0.79, 1.17)	9.96E-01	0.98 (0.80, 1.21)	9.56E-01
0.99 (0.82, 1.19)	9.96E-01	0.92 (0.74, 1.14)	8.22E-01
0.99 (0.82, 1.19)	9.96E-01	0.91 (0.74, 1.13)	8.22E-01
0.98 (0.81, 1.19)	9.96E-01	0.90 (0.73, 1.12)	8.22E-01
0.99 (0.82, 1.19)	9.96E-01	0.89 (0.72, 1.10)	8.22E-01
0.98 (0.81, 1.18)	9.96E-01	0.88 (0.72, 1.08)	8.22E-01
1.01 (0.83, 1.21)	9.96E-01	0.92 (0.74, 1.13)	8.22E-01
0.99 (0.82, 1.20)	9.96E-01	0.95 (0.77, 1.17)	8.86E-01
1.09 (0.91, 1.31)	7.38E-01	0.90 (0.73, 1.12)	8.22E-01
1.09 (0.90, 1.30)	7.67E-01	0.91 (0.74, 1.12)	8.22E-01
1.07 (0.89, 1.28)	9.02E-01	0.89 (0.73, 1.10)	8.22E-01
1.06 (0.88, 1.27)	9.26E-01	0.95 (0.78, 1.15)	8.84E-01
1.06 (0.88, 1.27)	9.26E-01	0.96 (0.79, 1.16)	9.28E-01

1.06 (0.89, 1.28)	9.26E-01	0.92 (0.76, 1.12)	8.22E-01
1.10 (0.92, 1.31)	7.17E-01	0.91 (0.73, 1.13)	8.22E-01
1.06 (0.88, 1.27)	9.26E-01	0.93 (0.75, 1.14)	8.22E-01
1.05 (0.87, 1.26)	9.96E-01	0.92 (0.75, 1.14)	8.22E-01
1.02 (0.85, 1.23)	9.96E-01	0.90 (0.73, 1.11)	8.22E-01
1.01 (0.84, 1.21)	9.96E-01	0.91 (0.74, 1.12)	8.22E-01
1.00 (0.83, 1.20)	9.96E-01	0.90 (0.74, 1.11)	8.22E-01
1.03 (0.85, 1.24)	9.96E-01	0.93 (0.76, 1.14)	8.61E-01
1.27 (1.09, 1.48)	**3.85E-02**	0.97 (0.79, 1.19)	9.31E-01
1.01 (0.83, 1.22)	9.96E-01	0.88 (0.71, 1.09)	8.22E-01
1.00 (0.82, 1.21)	9.96E-01	0.88 (0.71, 1.09)	8.22E-01
0.97 (0.80, 1.18)	9.96E-01	0.86 (0.69, 1.06)	7.10E-01
0.97 (0.80, 1.17)	9.96E-01	0.88 (0.71, 1.08)	8.22E-01
0.96 (0.80, 1.17)	9.96E-01	0.87 (0.71, 1.08)	8.22E-01
0.99 (0.82, 1.20)	9.96E-01	0.90 (0.73, 1.10)	8.22E-01
1.28 (1.11, 1.49)	**2.80E-02**	0.99 (0.81, 1.21)	9.62E-01
0.94 (0.77, 1.14)	9.26E-01	0.85 (0.68, 1.05)	7.10E-01
0.93 (0.77, 1.13)	8.74E-01	0.85 (0.68, 1.05)	7.10E-01
0.90 (0.75, 1.10)	7.17E-01	0.82 (0.66, 1.03)	7.10E-01
0.92 (0.76, 1.11)	7.67E-01	0.85 (0.69, 1.06)	7.10E-01
0.92 (0.76, 1.12)	7.92E-01	0.85 (0.69, 1.06)	7.10E-01
0.89 (0.74, 1.08)	6.51E-01	0.84 (0.68, 1.04)	7.10E-01
1.24 (1.05, 1.46)	1.29E-01	0.98 (0.79, 1.2)	9.56E-01
0.91 (0.75, 1.10)	7.36E-01	0.84 (0.67, 1.04)	7.10E-01
0.90 (0.74, 1.09)	7.17E-01	0.84 (0.67, 1.04)	7.10E-01
0.87 (0.73, 1.05)	4.52E-01	0.82 (0.66, 1.02)	7.10E-01
0.90 (0.74, 1.09)	7.14E-01	0.85 (0.69, 1.05)	7.10E-01
0.91 (0.75, 1.10)	7.38E-01	0.85 (0.69, 1.05)	7.10E-01
0.87 (0.72, 1.04)	4.52E-01	0.84 (0.68, 1.04)	7.10E-01
1.11 (0.92, 1.33)	6.91E-01	0.94 (0.75, 1.16)	8.84E-01
1.17 (0.99, 1.38)	2.88E-01	1.18 (0.97, 1.43)	7.10E-01
1.16 (0.98, 1.37)	3.40E-01	1.18 (0.97, 1.42)	7.10E-01
1.20 (1.02, 1.42)	1.77E-01	1.17 (0.96, 1.41)	7.10E-01
1.09 (0.92, 1.29)	7.38E-01	1.17 (0.96, 1.41)	7.10E-01
1.07 (0.91, 1.27)	8.00E-01	1.16 (0.96, 1.41)	7.10E-01
1.12 (0.94, 1.32)	5.78E-01	1.17 (0.96, 1.41)	7.10E-01
1.25 (1.06, 1.47)	9.31E-02	1.20 (1.00, 1.45)	7.10E-01
1.21 (1.02, 1.44)	1.77E-01	1.18 (0.97, 1.44)	7.10E-01
1.21 (1.02, 1.44)	1.77E-01	1.18 (0.97, 1.44)	7.10E-01
1.19 (1.00, 1.41)	2.53E-01	1.16 (0.95, 1.41)	7.10E-01
1.20 (1.01, 1.43)	1.89E-01	1.19 (0.97, 1.45)	7.10E-01
1.21 (1.02, 1.43)	1.77E-01	1.19 (0.98, 1.46)	7.10E-01
1.19 (1.00, 1.41)	2.26E-01	1.17 (0.96, 1.43)	7.10E-01
1.39 (1.20, 1.62)	**1.04E-03**	1.19 (0.98, 1.45)	7.10E-01
0.91 (0.74, 1.11)	7.38E-01	0.91 (0.75, 1.12)	8.22E-01
0.90 (0.74, 1.10)	7.29E-01	0.92 (0.75, 1.12)	8.22E-01
0.96 (0.79, 1.18)	9.96E-01	0.94 (0.77, 1.15)	8.84E-01
0.84 (0.69, 1.03)	3.82E-01	0.92 (0.76, 1.12)	8.22E-01

0.83 (0.68, 1.01)	2.79E-01	0.91 (0.75, 1.11)	8.22E-01
0.92 (0.75, 1.12)	7.91E-01	0.95 (0.78, 1.16)	8.86E-01
1.05 (0.88, 1.25)	9.77E-01	0.87 (0.70, 1.09)	8.22E-01
0.82 (0.69, 0.97)	1.63E-01	0.77 (0.63, 0.94)	5.43E-01
0.81 (0.69, 0.96)	1.29E-01	0.77 (0.63, 0.94)	5.43E-01
0.85 (0.70, 1.04)	3.91E-01	0.87 (0.72, 1.05)	7.10E-01
0.83 (0.70, 0.99)	2.19E-01	0.79 (0.64, 0.96)	6.87E-01
0.86 (0.72, 1.03)	3.82E-01	0.81 (0.67, 0.99)	7.10E-01
0.83 (0.68, 1.01)	2.53E-01	0.86 (0.71, 1.04)	7.10E-01
1.09 (0.91, 1.30)	7.38E-01	0.90 (0.73, 1.12)	8.22E-01
1.20 (0.96, 1.50)	3.86E-01	0.93 (0.75, 1.17)	8.84E-01
1.25 (1.09, 1.42)	**2.80E-02**	0.99 (0.79, 1.25)	9.66E-01
1.20 (1.04, 1.39)	1.29E-01	0.98 (0.78, 1.24)	9.57E-01
1.24 (1.06, 1.44)	7.02E-02	1.02 (0.80, 1.30)	9.57E-01
0.76 (0.66, 0.88)	**9.81E-03**	1.03 (0.82, 1.30)	9.31E-01
1.21 (0.97, 1.52)	3.46E-01	0.91 (0.73, 1.13)	8.22E-01
1.11 (0.98, 1.27)	3.82E-01	1.03 (0.85, 1.25)	9.31E-01
1.02 (0.85, 1.24)	9.96E-01	1.04 (0.86, 1.26)	9.31E-01
1.16 (1.02, 1.32)	1.55E-01	1.00 (0.79, 1.25)	9.95E-01
0.82 (0.69, 0.97)	1.55E-01	1.02 (0.82, 1.27)	9.56E-01
1.26 (1.00, 1.57)	2.26E-01	0.96 (0.76, 1.20)	9.31E-01
1.10 (0.90, 1.36)	7.38E-01	0.97 (0.78, 1.21)	9.51E-01
0.96 (0.79, 1.17)	9.96E-01	1.00 (0.81, 1.23)	9.95E-01
1.17 (0.95, 1.44)	4.52E-01	0.96 (0.77, 1.20)	9.31E-01
0.87 (0.70, 1.08)	5.78E-01	1.03 (0.83, 1.29)	9.31E-01
0.97 (0.81, 1.16)	9.96E-01	0.94 (0.76, 1.17)	8.84E-01
1.15 (0.96, 1.38)	4.52E-01	0.99 (0.80, 1.23)	9.62E-01
1.09 (0.91, 1.31)	7.36E-01	0.97 (0.78, 1.20)	9.31E-01
1.14 (0.94, 1.38)	5.69E-01	1.07 (0.84, 1.36)	8.84E-01
0.88 (0.74, 1.06)	5.69E-01	1.02 (0.82, 1.27)	9.56E-01
0.99 (0.83, 1.18)	9.96E-01	1.01 (0.82, 1.25)	9.62E-01
0.99 (0.83, 1.18)	9.96E-01	0.96 (0.79, 1.18)	9.31E-01
0.97 (0.82, 1.16)	9.96E-01	0.96 (0.78, 1.17)	9.23E-01
1.10 (0.92, 1.32)	7.14E-01	1.04 (0.85, 1.28)	9.31E-01
1.02 (0.85, 1.21)	9.96E-01	1.04 (0.85, 1.27)	9.31E-01
0.97 (0.81, 1.16)	9.96E-01	0.92 (0.76, 1.13)	8.22E-01
0.97 (0.82, 1.16)	9.96E-01	1.09 (0.89, 1.34)	8.22E-01
0.98 (0.82, 1.16)	9.96E-01	1.11 (0.90, 1.37)	8.22E-01
0.98 (0.82, 1.17)	9.96E-01	1.01 (0.83, 1.24)	9.62E-01
1.04 (0.87, 1.24)	9.96E-01	0.97 (0.79, 1.18)	9.31E-01
0.88 (0.74, 1.06)	5.63E-01	0.96 (0.78, 1.19)	9.31E-01
0.87 (0.75, 1.00)	2.42E-01	0.96 (0.80, 1.14)	8.86E-01
0.84 (0.72, 0.98)	1.71E-01	0.91 (0.76, 1.1)	8.22E-01
0.96 (0.81, 1.15)	9.96E-01	1.03 (0.84, 1.27)	9.31E-01
1.19 (1.03, 1.38)	1.54E-01	1.06 (0.88, 1.27)	8.84E-01
1.05 (0.87, 1.25)	9.96E-01	1.06 (0.87, 1.28)	8.84E-01
0.86 (0.74, 0.99)	1.77E-01	0.92 (0.77, 1.09)	8.22E-01
0.85 (0.74, 0.98)	1.70E-01	0.88 (0.74, 1.05)	7.10E-01

0.99 (0.82, 1.20)	9.96E-01	1.13 (0.90, 1.43)	8.22E-01
1.23 (1.08, 1.39)	**3.66E-02**	1.10 (0.93, 1.31)	8.22E-01
1.06 (0.89, 1.26)	9.26E-01	1.10 (0.91, 1.32)	8.22E-01
0.88 (0.75, 1.04)	4.38E-01	0.89 (0.74, 1.07)	8.22E-01
0.90 (0.76, 1.06)	5.78E-01	0.88 (0.73, 1.05)	7.10E-01
1.03 (0.85, 1.24)	9.96E-01	1.18 (0.97, 1.44)	7.10E-01
1.26 (1.09, 1.46)	**3.66E-02**	1.15 (0.96, 1.39)	7.10E-01
1.06 (0.89, 1.27)	9.02E-01	1.11 (0.92, 1.34)	8.22E-01
0.90 (0.76, 1.06)	5.78E-01	0.90 (0.75, 1.09)	8.22E-01
0.91 (0.76, 1.08)	7.14E-01	0.89 (0.74, 1.07)	8.22E-01
1.00 (0.82, 1.21)	9.96E-01	1.18 (0.97, 1.44)	7.10E-01
1.19 (1.02, 1.4)	1.77E-01	1.09 (0.90, 1.32)	8.22E-01
1.13 (0.94, 1.36)	5.69E-01	1.07 (0.88, 1.31)	8.39E-01
0.83 (0.70, 0.98)	1.77E-01	0.92 (0.76, 1.11)	8.22E-01
0.82 (0.69, 0.98)	1.77E-01	0.92 (0.76, 1.12)	8.22E-01
0.91 (0.78, 1.06)	6.28E-01	0.93 (0.78, 1.11)	8.22E-01
1.13 (0.97, 1.32)	3.82E-01	1.02 (0.84, 1.23)	9.56E-01
0.76 (0.62, 0.92)	7.02E-02	0.75 (0.60, 0.93)	5.43E-01
1.08 (0.90, 1.30)	8.00E-01	1.22 (0.97, 1.53)	7.10E-01
1.09 (0.90, 1.32)	7.71E-01	1.32 (1.04, 1.67)	6.87E-01
1.06 (0.88, 1.26)	9.26E-01	1.06 (0.86, 1.31)	8.84E-01
1.25 (1.08, 1.44)	**3.85E-02**	1.10 (0.91, 1.33)	8.22E-01
1.33 (1.18, 1.48)	**1.24E-04**	1.11 (0.95, 1.29)	7.97E-01
0.75 (0.65, 0.86)	**3.14E-03**	0.98 (0.81, 1.18)	9.56E-01
0.74 (0.65, 0.85)	**7.18E-04**	0.97 (0.81, 1.17)	9.31E-01
0.92 (0.77, 1.10)	7.67E-01	1.03 (0.83, 1.26)	9.56E-01
1.17 (0.98, 1.39)	3.14E-01	0.92 (0.74, 1.14)	8.22E-01
1.00 (0.83, 1.21)	9.96E-01	1.11 (0.91, 1.35)	8.22E-01
0.95 (0.79, 1.14)	9.38E-01	0.92 (0.75, 1.12)	8.22E-01
0.96 (0.79, 1.15)	9.96E-01	0.91 (0.75, 1.11)	8.22E-01
0.96 (0.79, 1.16)	9.96E-01	1.13 (0.92, 1.39)	8.22E-01
1.19 (1.01, 1.42)	2.12E-01	0.98 (0.80, 1.21)	9.57E-01
0.97 (0.81, 1.17)	9.96E-01	0.99 (0.80, 1.22)	9.62E-01
1.40 (1.19, 1.66)	**3.24E-03**	1.15 (0.94, 1.41)	7.21E-01
1.19 (1.00, 1.41)	2.34E-01	1.18 (0.96, 1.44)	7.10E-01
1.00 (0.83, 1.20)	9.96E-01	0.92 (0.75, 1.13)	8.22E-01
1.02 (0.84, 1.23)	9.96E-01	0.94 (0.77, 1.16)	8.84E-01
1.02 (0.84, 1.23)	9.96E-01	0.93 (0.75, 1.14)	8.28E-01
0.96 (0.79, 1.16)	9.96E-01	0.88 (0.72, 1.08)	8.22E-01
1.03 (0.86, 1.24)	9.96E-01	1.06 (0.87, 1.30)	8.84E-01
1.02 (0.85, 1.23)	9.96E-01	1.06 (0.86, 1.29)	8.84E-01
1.07 (0.90, 1.28)	8.00E-01	1.08 (0.89, 1.31)	8.22E-01
1.01 (0.83, 1.22)	9.96E-01	0.89 (0.71, 1.10)	8.22E-01
1.00 (0.82, 1.21)	9.96E-01	0.91 (0.73, 1.13)	8.22E-01
1.03 (0.86, 1.24)	9.96E-01	1.00 (0.81, 1.23)	9.95E-01
0.98 (0.81, 1.19)	9.96E-01	1.00 (0.81, 1.23)	9.95E-01
1.26 (1.08, 1.47)	5.43E-02	1.01 (0.82, 1.23)	9.66E-01
1.22 (1.03, 1.44)	1.55E-01	1.06 (0.86, 1.31)	8.84E-01

0.99 (0.82, 1.20)	9.96E-01	1.02 (0.83, 1.25)	9.56E-01
0.94 (0.78, 1.14)	9.26E-01	0.98 (0.79, 1.21)	9.56E-01
0.95 (0.78, 1.15)	9.66E-01	0.98 (0.80, 1.21)	9.57E-01
0.97 (0.80, 1.17)	9.96E-01	0.93 (0.74, 1.16)	8.79E-01
1.03 (0.85, 1.25)	9.96E-01	1.02 (0.83, 1.25)	9.57E-01
1.03 (0.86, 1.24)	9.96E-01	1.04 (0.85, 1.27)	9.31E-01
0.98 (0.81, 1.19)	9.96E-01	0.91 (0.73, 1.12)	8.22E-01
1.00 (0.83, 1.21)	9.96E-01	0.89 (0.72, 1.10)	8.22E-01
0.92 (0.76, 1.13)	8.14E-01	0.97 (0.78, 1.20)	9.32E-01
0.79 (0.66, 0.93)	7.02E-02	0.78 (0.62, 0.97)	7.10E-01
0.88 (0.72, 1.07)	5.69E-01	0.90 (0.74, 1.09)	8.22E-01
0.92 (0.75, 1.12)	7.74E-01	0.94 (0.75, 1.17)	8.84E-01
0.87 (0.70, 1.07)	5.63E-01	0.86 (0.69, 1.06)	7.10E-01
0.91 (0.75, 1.11)	7.67E-01	0.91 (0.73, 1.15)	8.22E-01
0.90 (0.73, 1.10)	7.17E-01	0.90 (0.72, 1.13)	8.22E-01
0.94 (0.77, 1.15)	9.26E-01	1.00 (0.81, 1.23)	9.95E-01
0.94 (0.77, 1.14)	9.26E-01	1.01 (0.82, 1.24)	9.66E-01
0.95 (0.79, 1.13)	9.26E-01	0.93 (0.76, 1.13)	8.22E-01
0.88 (0.73, 1.06)	5.52E-01	0.93 (0.75, 1.16)	8.84E-01
0.91 (0.76, 1.09)	7.17E-01	0.82 (0.67, 1.01)	7.10E-01
0.89 (0.74, 1.07)	5.93E-01	0.90 (0.73, 1.12)	8.22E-01
0.86 (0.71, 1.03)	3.86E-01	0.83 (0.67, 1.04)	7.10E-01
1.10 (0.92, 1.32)	7.17E-01	1.06 (0.86, 1.32)	8.84E-01
1.12 (0.93, 1.34)	6.37E-01	1.24 (0.99, 1.54)	7.10E-01
1.32 (1.18, 1.47)	**1.24E-04**	1.17 (1.03, 1.34)	6.87E-01
0.96 (0.80, 1.16)	9.96E-01	0.84 (0.66, 1.06)	7.10E-01
1.13 (0.94, 1.36)	5.78E-01	1.02 (0.82, 1.26)	9.56E-01
0.97 (0.81, 1.17)	9.96E-01	0.95 (0.77, 1.18)	9.28E-01
0.80 (0.66, 0.96)	1.54E-01	0.96 (0.78, 1.19)	9.31E-01
0.83 (0.68, 1.00)	2.26E-01	1.10 (0.89, 1.36)	8.22E-01
0.89 (0.73, 1.08)	6.51E-01	0.97 (0.79, 1.19)	9.31E-01
0.91 (0.75, 1.10)	7.38E-01	0.89 (0.72, 1.10)	8.22E-01
0.90 (0.74, 1.09)	7.17E-01	0.90 (0.73, 1.13)	8.22E-01
1.18 (1.06, 1.30)	**3.31E-02**	1.09 (0.92, 1.29)	8.22E-01
1.30 (1.10, 1.54)	**3.66E-02**	1.05 (0.86, 1.28)	8.86E-01
0.99 (0.81, 1.21)	9.96E-01	0.96 (0.76, 1.21)	9.31E-01
1.00 (0.80, 1.24)	9.96E-01	1.06 (0.88, 1.29)	8.84E-01
1.13 (1.00, 1.28)	2.53E-01	1.07 (0.90, 1.27)	8.22E-01
1.08 (1.02, 1.14)	1.29E-01	1.11 (1.05, 1.18)	5.72E-02
0.84 (0.71, 0.98)	1.77E-01	0.81 (0.67, 0.99)	7.10E-01
0.94 (0.76, 1.15)	9.26E-01	1.09 (0.89, 1.33)	8.22E-01
0.95 (0.92, 0.98)	**1.27E-03**	0.98 (0.94, 1.01)	3.90E-01
0.95 (0.93, 0.98)	**3.07E-03**	0.98 (0.94, 1.01)	3.46E-01
0.93 (0.90, 0.96)	**5.03E-06**	0.97 (0.93, 1.00)	1.98E-01
1.04 (1.02, 1.07)	**1.36E-03**	1.03 (0.99, 1.07)	2.16E-01
1.06 (1.02, 1.09)	**7.31E-04**	1.01 (0.98, 1.05)	5.84E-01
1.08 (1.06, 1.11)	**2.78E-09**	1.01 (0.98, 1.05)	6.44E-01
0.92 (0.89, 0.95)	**4.07E-07**	0.97 (0.94, 1.01)	2.86E-01

0.96 (0.93, 0.99)	**1.46E-02**	0.98 (0.95, 1.02)	4.06E-01
1.05 (1.02, 1.08)	**2.38E-04**	1.04 (1.00, 1.08)	1.20E-01
1.03 (1.01, 1.05)	**6.44E-03**	1.00 (0.98, 1.02)	9.41E-01
1.06 (1.04, 1.08)	**1.18E-07**	1.05 (1.02, 1.08)	**1.74E-02**
0.97 (0.95, 1.00)	5.31E-02	0.99 (0.96, 1.03)	7.90E-01
0.93 (0.90, 0.96)	**8.77E-06**	0.98 (0.94, 1.02)	4.48E-01
0.94 (0.92, 0.97)	**3.90E-04**	0.98 (0.94, 1.02)	3.99E-01
0.98 (0.95, 1.00)	1.24E-01	1.00 (0.96, 1.03)	8.74E-01
0.97 (0.95, 1.00)	7.98E-02	1.00 (0.96, 1.03)	9.43E-01
0.92 (0.89, 0.95)	**1.29E-07**	0.97 (0.94, 1.01)	2.53E-01
0.94 (0.91, 0.97)	**1.22E-04**	0.97 (0.94, 1.01)	2.49E-01
1.03 (1.00, 1.06)	**4.40E-02**	1.03 (0.99, 1.06)	3.11E-01
0.94 (0.92, 0.97)	**2.20E-04**	0.98 (0.95, 1.02)	4.81E-01
0.91 (0.88, 0.94)	**2.49E-09**	0.96 (0.93, 1.00)	1.52E-01
0.94 (0.91, 0.97)	**5.10E-05**	0.97 (0.94, 1.01)	2.65E-01
1.03 (1.01, 1.06)	**1.41E-02**	1.03 (0.99, 1.07)	2.42E-01
0.95 (0.92, 0.98)	**3.89E-03**	0.99 (0.95, 1.02)	6.05E-01
1.05 (1.02, 1.07)	**8.65E-04**	1.03 (1.00, 1.07)	1.86E-01
1.01 (0.99, 1.04)	3.83E-01	1.02 (0.98, 1.07)	4.87E-01

UKB			
**All causes mortality**			
**Male**		**Female**	
**Adjusted HR (95% CI)**	**P value**	**Adjusted HR (95% CI)**	**P value**
0.95 (0.94, 0.97)	**8.90E-07**	1.03 (1.01, 1.05)	**4.08E-03**
0.95 (0.94, 0.97)	**6.43E-07**	1.03 (1.01, 1.05)	**7.41E-03**
0.96 (0.94, 0.97)	**2.34E-06**	1.03 (1.01, 1.05)	**2.74E-03**
0.95 (0.93, 0.96)	**1.67E-08**	1.02 (1.00, 1.05)	**2.63E-02**
0.94 (0.92, 0.96)	**1.36E-09**	1.02 (1.00, 1.04)	5.13E-02
0.95 (0.93, 0.97)	**3.91E-07**	1.03 (1.01, 1.05)	**1.18E-02**
0.96 (0.94, 0.97)	**1.87E-06**	1.03 (1.01, 1.05)	**6.76E-03**
0.91 (0.90, 0.93)	**2.69E-20**	1.01 (0.99, 1.03)	5.89E-01
0.91 (0.90, 0.93)	**7.01E-21**	1.00 (0.98, 1.02)	7.50E-01
0.92 (0.90, 0.93)	**2.43E-19**	1.00 (0.98, 1.03)	7.11E-01
0.90 (0.88, 0.91)	**1.78E-28**	0.98 (0.96, 1.01)	1.64E-01
0.89 (0.87, 0.90)	**3.21E-35**	0.97 (0.95, 0.99)	**7.67E-03**
0.91 (0.90, 0.93)	**7.57E-21**	1.00 (0.98, 1.02)	9.50E-01
0.92 (0.90, 0.94)	**3.24E-18**	1.01 (0.99, 1.03)	3.68E-01
0.90 (0.88, 0.92)	**8.98E-28**	0.99 (0.97, 1.01)	4.89E-01
0.89 (0.87, 0.91)	**3.54E-33**	0.98 (0.96, 1.00)	1.36E-01
0.90 (0.88, 0.92)	**1.43E-26**	1.00 (0.98, 1.02)	7.50E-01
0.89 (0.87, 0.91)	**5.31E-34**	0.98 (0.96, 1.00)	**4.75E-02**
0.89 (0.87, 0.91)	**3.92E-34**	0.97 (0.95, 0.99)	**4.62E-03**
0.89 (0.87, 0.91)	**5.94E-32**	0.99 (0.97, 1.01)	2.61E-01
0.89 (0.87, 0.91)	**1.19E-33**	0.98 (0.96, 1.00)	9.01E-02
0.88 (0.86, 0.89)	**1.25E-42**	0.94 (0.92, 0.96)	**2.37E-08**
0.87 (0.85, 0.89)	**6.56E-46**	0.94 (0.92, 0.96)	**1.87E-07**
0.88 (0.86, 0.90)	**1.41E-39**	0.94 (0.92, 0.96)	**1.77E-08**
0.90 (0.88, 0.91)	**1.61E-30**	0.92 (0.90, 0.94)	**2.03E-14**
0.91 (0.89, 0.93)	**7.74E-23**	0.91 (0.89, 0.93)	**7.88E-17**
0.88 (0.87, 0.90)	**4.17E-37**	0.93 (0.91, 0.95)	**3.17E-10**
0.88 (0.86, 0.90)	**4.63E-39**	0.97 (0.95, 0.99)	**9.40E-03**
0.91 (0.90, 0.93)	**7.53E-21**	0.99 (0.97, 1.01)	2.54E-01
0.91 (0.89, 0.93)	**2.03E-23**	0.98 (0.96, 1.00)	6.87E-02
0.90 (0.88, 0.91)	**4.98E-30**	0.95 (0.93, 0.97)	**2.93E-05**
0.91 (0.89, 0.93)	**1.08E-23**	0.96 (0.94, 0.98)	**1.14E-04**
0.92 (0.90, 0.94)	**4.67E-19**	0.97 (0.95, 0.99)	**6.79E-03**
0.89 (0.88, 0.91)	**8.70E-32**	0.94 (0.92, 0.96)	**6.48E-09**
0.93 (0.92, 0.95)	**7.60E-13**	1.02 (0.99, 1.04)	1.68E-01
1.00 (0.98, 1.02)	9.89E-01	1.00 (0.97, 1.02)	7.30E-01
1.01 (0.99, 1.03)	4.40E-01	1.00 (0.98, 1.02)	9.50E-01
1.02 (1.00, 1.04)	**1.79E-02**	1.02 (1.00, 1.04)	1.10E-01
0.99 (0.98, 1.01)	5.98E-01	0.97 (0.95, 0.99)	**1.80E-03**
0.99 (0.97, 1.01)	3.29E-01	0.95 (0.93, 0.97)	**2.75E-05**

1.01 (0.99, 1.02)	6.06E-01	0.99 (0.97, 1.02)	5.89E-01
1.01 (1.00, 1.03)	1.45E-01	1.06 (1.04, 1.08)	**2.50E-08**
0.93 (0.92, 0.95)	**1.76E-13**	0.93 (0.91, 0.95)	**6.31E-12**
0.94 (0.92, 0.96)	**4.60E-11**	0.92 (0.90, 0.94)	**2.31E-14**
0.95 (0.94, 0.97)	**3.59E-07**	0.93 (0.91, 0.95)	**2.21E-10**
0.93 (0.91, 0.95)	**4.76E-15**	0.90 (0.88, 0.92)	**8.89E-20**
0.92 (0.91, 0.94)	**3.31E-17**	0.90 (0.88, 0.92)	**3.69E-21**
0.95 (0.93, 0.96)	**2.56E-09**	0.92 (0.90, 0.94)	**3.02E-15**
1.04 (1.02, 1.06)	**3.66E-05**	1.07 (1.05, 1.09)	**8.96E-10**
0.89 (0.88, 0.91)	**4.42E-31**	0.92 (0.90, 0.94)	**1.57E-12**
0.88 (0.86, 0.90)	**8.16E-40**	0.90 (0.88, 0.92)	**3.80E-20**
0.88 (0.86, 0.90)	**9.18E-40**	0.90 (0.88, 0.92)	**5.00E-22**
0.87 (0.86, 0.89)	**2.57E-44**	0.89 (0.87, 0.91)	**2.54E-23**
0.87 (0.85, 0.89)	**1.80E-47**	0.89 (0.88, 0.91)	**2.34E-22**
0.89 (0.87, 0.91)	**2.86E-34**	0.89 (0.87, 0.91)	**9.03E-25**
1.02 (1.00, 1.04)	**2.05E-02**	1.06 (1.04, 1.09)	**1.33E-08**
0.87 (0.86, 0.89)	**5.00E-43**	0.92 (0.90, 0.94)	**7.30E-13**
0.85 (0.84, 0.87)	**5.57E-60**	0.91 (0.89, 0.93)	**6.98E-18**
0.85 (0.83, 0.87)	**1.95E-62**	0.90 (0.88, 0.92)	**5.00E-19**
0.85 (0.83, 0.86)	**5.25E-64**	0.90 (0.88, 0.92)	**1.43E-20**
0.85 (0.83, 0.87)	**7.35E-63**	0.91 (0.89, 0.93)	**1.55E-17**
0.86 (0.84, 0.87)	**4.84E-58**	0.88 (0.87, 0.90)	**3.79E-27**
0.98 (0.97, 1.00)	1.17E-01	1.05 (1.03, 1.07)	**8.56E-06**
0.89 (0.87, 0.91)	**3.06E-34**	0.93 (0.91, 0.95)	**1.11E-09**
0.87 (0.85, 0.89)	**2.02E-47**	0.92 (0.90, 0.94)	**5.81E-15**
0.89 (0.87, 0.91)	**6.70E-35**	0.92 (0.90, 0.94)	**9.13E-13**
0.86 (0.84, 0.88)	**2.84E-55**	0.90 (0.89, 0.92)	**6.51E-19**
0.86 (0.84, 0.88)	**3.05E-53**	0.91 (0.89, 0.93)	**1.49E-15**
0.87 (0.85, 0.89)	**1.58E-48**	0.89 (0.87, 0.91)	**4.48E-25**
0.96 (0.94, 0.98)	**8.51E-05**	1.04 (1.01, 1.06)	**1.14E-03**
1.13 (1.12, 1.15)	**3.71E-54**	1.01 (0.99, 1.03)	4.65E-01
1.15 (1.13, 1.17)	**1.20E-68**	1.01 (0.99, 1.04)	2.52E-01
1.16 (1.14, 1.18)	**4.82E-79**	1.02 (1.00, 1.04)	8.51E-02
1.13 (1.12, 1.15)	**6.21E-53**	1.00 (0.98, 1.02)	9.50E-01
1.13 (1.11, 1.14)	**4.30E-46**	0.99 (0.97, 1.02)	6.09E-01
1.16 (1.14, 1.17)	**1.42E-73**	1.03 (1.00, 1.05)	**2.63E-02**
1.04 (1.02, 1.06)	**3.67E-06**	1.06 (1.04, 1.08)	**3.02E-07**
1.11 (1.09, 1.13)	**1.08E-34**	0.98 (0.96, 1.01)	1.75E-01
1.12 (1.10, 1.14)	**1.83E-38**	0.99 (0.97, 1.01)	3.11E-01
1.12 (1.10, 1.14)	**3.37E-39**	0.99 (0.97, 1.01)	4.40E-01
1.11 (1.09, 1.13)	**2.57E-34**	0.98 (0.96, 1.00)	1.10E-01
1.11 (1.09, 1.12)	**1.74E-31**	0.98 (0.96, 1.00)	6.46E-02
1.12 (1.11, 1.14)	**5.25E-44**	0.99 (0.97, 1.01)	4.65E-01
1.08 (1.06, 1.10)	**1.88E-17**	1.05 (1.03, 1.08)	**2.23E-06**
1.00 (0.98, 1.01)	6.74E-01	0.94 (0.92, 0.96)	**3.83E-07**
1.00 (0.98, 1.02)	7.55E-01	0.95 (0.93, 0.97)	**6.99E-06**
1.00 (0.98, 1.02)	9.35E-01	0.96 (0.94, 0.98)	**1.60E-04**
1.00 (0.98, 1.01)	6.34E-01	0.94 (0.91, 0.96)	**1.04E-08**

0.99 (0.97, 1.01)	4.27E-01	0.93 (0.91, 0.95)	**3.30E-09**
1.01 (0.99, 1.03)	4.08E-01	0.94 (0.92, 0.97)	**1.46E-06**
1.00 (0.98, 1.02)	8.70E-01	1.04 (1.02, 1.06)	**4.65E-04**
0.84 (0.82, 0.85)	**1.56E-74**	0.88 (0.86, 0.90)	**2.74E-26**
0.88 (0.86, 0.89)	**1.50E-44**	0.92 (0.90, 0.94)	**4.51E-14**
0.90 (0.88, 0.92)	**8.44E-30**	0.93 (0.91, 0.95)	**2.68E-10**
0.85 (0.83, 0.86)	**1.72E-68**	0.88 (0.86, 0.90)	**1.00E-27**
0.83 (0.82, 0.85)	**1.49E-79**	0.87 (0.85, 0.89)	**3.46E-31**
0.90 (0.88, 0.92)	**2.56E-28**	0.92 (0.90, 0.94)	**1.00E-12**
0.95 (0.94, 0.97)	**5.82E-07**	1.04 (1.02, 1.06)	**5.93E-04**
1.02 (1.00, 1.04)	**2.02E-02**	1.07 (1.04, 1.10)	**9.12E-07**
1.02 (1.00, 1.03)	1.11E-01	1.02 (0.99, 1.04)	2.16E-01
1.01 (0.99, 1.03)	4.27E-01	1.01 (0.99, 1.03)	4.83E-01
1.03 (1.01, 1.04)	**4.41E-03**	1.02 (1.00, 1.05)	**4.75E-02**
0.98 (0.96, 1.00)	**4.08E-02**	0.97 (0.95, 0.99)	**7.41E-03**
1.04 (1.02, 1.06)	**5.24E-06**	0.98 (0.96, 1.01)	1.43E-01
1.03 (1.01, 1.05)	**1.98E-03**	0.97 (0.95, 1.00)	**3.33E-02**
1.08 (1.06, 1.10)	**1.81E-19**	1.02 (0.99, 1.04)	1.71E-01
0.96 (0.94, 0.97)	**2.37E-06**	1.01 (0.98, 1.03)	5.20E-01
0.99 (0.97, 1.01)	3.05E-01	1.06 (1.03, 1.08)	**4.63E-05**
1.07 (1.05, 1.08)	**1.16E-12**	1.03 (1.00, 1.05)	**2.37E-02**
1.07 (1.05, 1.09)	**5.23E-15**	1.01 (0.99, 1.03)	3.76E-01
1.03 (1.01, 1.05)	**1.08E-03**	1.06 (1.04, 1.09)	**1.52E-06**
0.95 (0.94, 0.97)	**2.88E-07**	0.95 (0.93, 0.97)	**8.15E-05**
1.01 (0.99, 1.03)	2.60E-01	0.96 (0.94, 0.98)	**5.93E-04**
1.02 (1.00, 1.04)	7.41E-02	0.94 (0.92, 0.96)	**7.95E-08**
1.02 (1.00, 1.04)	6.78E-02	0.94 (0.92, 0.96)	**1.48E-08**
1.02 (1.00, 1.03)	1.08E-01	0.95 (0.93, 0.97)	**1.65E-05**
0.98 (0.97, 1.00)	9.59E-02	1.06 (1.03, 1.08)	**4.69E-07**
0.95 (0.93, 0.97)	**2.42E-08**	0.90 (0.88, 0.92)	**3.79E-21**
0.98 (0.97, 1.00)	6.96E-02	0.94 (0.92, 0.96)	**1.21E-09**
1.01 (0.99, 1.03)	4.03E-01	0.97 (0.95, 0.99)	**8.31E-03**
0.95 (0.93, 0.97)	**1.17E-07**	0.90 (0.88, 0.92)	**8.97E-22**
1.03 (1.01, 1.05)	**3.09E-03**	1.08 (1.06, 1.11)	**4.39E-13**
1.13 (1.11, 1.16)	**4.42E-42**	1.18 (1.15, 1.20)	**3.29E-46**
0.97 (0.95, 0.98)	**2.21E-04**	0.89 (0.87, 0.91)	**4.68E-28**
0.96 (0.95, 0.98)	**8.23E-05**	0.89 (0.87, 0.90)	**2.95E-30**
0.99 (0.97, 1.00)	1.74E-01	0.95 (0.93, 0.97)	**6.64E-07**
1.01 (0.99, 1.03)	1.95E-01	1.10 (1.08, 1.12)	**4.81E-20**
1.12 (1.10, 1.14)	**5.82E-38**	1.13 (1.10, 1.15)	**8.64E-26**
0.90 (0.88, 0.91)	**6.14E-36**	0.86 (0.85, 0.88)	**3.41E-52**
0.88 (0.86, 0.89)	**5.58E-54**	0.85 (0.83, 0.86)	**2.54E-59**
0.99 (0.98, 1.01)	5.71E-01	0.95 (0.93, 0.97)	**3.87E-06**
1.09 (1.07, 1.11)	**4.61E-25**	1.15 (1.13, 1.17)	**3.19E-48**
1.04 (1.02, 1.05)	**1.21E-05**	0.99 (0.97, 1.01)	3.90E-01
0.92 (0.91, 0.93)	**1.42E-64**	0.92 (0.91, 0.93)	**1.11E-73**
0.92 (0.91, 0.92)	**3.30E-103**	0.92 (0.91, 0.93)	**5.22E-45**
0.97 (0.96, 0.99)	**2.93E-03**	0.91 (0.89, 0.93)	**3.40E-20**

1.11 (1.09, 1.12)	**1.67E-58**	1.11 (1.10, 1.12)	**2.14E-82**
1.03 (1.01, 1.05)	**5.07E-04**	0.99 (0.97, 1.02)	5.45E-01
0.92 (0.91, 0.92)	**1.30E-75**	0.92 (0.91, 0.93)	**1.38E-61**
0.89 (0.88, 0.91)	**1.10E-65**	0.94 (0.92, 0.96)	**1.90E-07**
0.98 (0.97, 1.00)	7.93E-02	0.92 (0.90, 0.94)	**6.92E-16**
1.12 (1.10, 1.13)	**1.40E-75**	1.09 (1.08, 1.10)	**1.83E-75**
1.13 (1.11, 1.14)	**4.22E-60**	1.06 (1.04, 1.09)	**6.19E-08**
0.89 (0.88, 0.90)	**9.20E-97**	0.90 (0.89, 0.91)	**8.87E-60**
0.89 (0.87, 0.90)	**5.56E-52**	0.94 (0.92, 0.96)	**8.27E-08**
0.95 (0.94, 0.97)	**1.84E-07**	0.91 (0.89, 0.93)	**6.15E-20**
1.06 (1.05, 1.08)	**1.47E-13**	1.10 (1.08, 1.12)	**3.01E-30**
1.16 (1.13, 1.18)	**3.26E-42**	1.03 (1.01, 1.05)	**1.40E-02**
0.87 (0.86, 0.89)	**2.59E-43**	0.93 (0.91, 0.95)	**1.82E-10**
0.92 (0.90, 0.93)	**8.29E-25**	0.91 (0.89, 0.92)	**6.26E-24**
0.92 (0.90, 0.94)	**1.79E-16**	1.02 (1.00, 1.04)	1.17E-01
0.95 (0.93, 0.97)	**1.83E-07**	1.03 (1.01, 1.05)	**7.52E-04**
0.98 (0.96, 1.00)	**3.88E-02**	1.05 (1.03, 1.07)	**1.41E-06**
1.02 (1.00, 1.04)	**1.64E-02**	0.94 (0.93, 0.96)	**7.12E-09**
1.01 (0.99, 1.03)	4.01E-01	0.94 (0.92, 0.96)	**1.06E-10**
1.07 (1.05, 1.09)	**3.72E-14**	0.99 (0.97, 1.01)	4.19E-01
0.98 (0.96, 1.00)	**3.64E-02**	1.05 (1.03, 1.07)	**9.92E-08**
1.04 (1.02, 1.06)	**2.84E-05**	1.10 (1.08, 1.12)	**1.23E-18**
0.97 (0.96, 0.99)	**5.05E-03**	0.91 (0.90, 0.93)	**1.60E-16**
0.97 (0.95, 0.99)	**1.41E-03**	0.92 (0.90, 0.94)	**4.24E-15**
1.00 (0.99, 1.02)	7.48E-01	0.93 (0.91, 0.95)	**3.20E-10**
1.02 (1.00, 1.03)	1.11E-01	1.08 (1.06, 1.10)	**1.94E-13**
1.15 (1.13, 1.18)	**4.18E-54**	1.09 (1.06, 1.11)	**5.52E-13**
0.90 (0.88, 0.92)	**2.79E-30**	0.89 (0.87, 0.91)	**1.14E-26**
0.88 (0.87, 0.90)	**3.20E-43**	0.89 (0.87, 0.90)	**7.66E-29**
1.08 (1.06, 1.10)	**6.45E-20**	1.02 (0.99, 1.04)	1.73E-01
1.03 (1.01, 1.04)	**9.16E-03**	1.10 (1.07, 1.12)	**1.71E-17**
0.87 (0.86, 0.89)	**9.00E-51**	0.98 (0.96, 1.00)	**4.86E-02**
1.01 (0.99, 1.03)	4.85E-01	0.96 (0.94, 0.98)	**8.73E-04**
1.16 (1.14, 1.18)	**6.05E-69**	1.02 (1.00, 1.04)	1.03E-01
0.90 (0.89, 0.92)	**9.57E-27**	0.90 (0.88, 0.92)	**1.86E-21**
0.91 (0.89, 0.93)	**2.31E-23**	0.96 (0.94, 0.98)	**2.82E-04**
0.86 (0.85, 0.88)	**1.60E-51**	0.89 (0.88, 0.91)	**1.09E-22**
0.91 (0.89, 0.93)	**1.05E-22**	0.93 (0.91, 0.95)	**1.62E-11**
0.90 (0.89, 0.92)	**4.95E-27**	0.89 (0.88, 0.91)	**6.69E-23**
0.91 (0.89, 0.92)	**3.55E-25**	0.91 (0.89, 0.93)	**2.33E-17**
0.93 (0.91, 0.95)	**2.15E-13**	1.01 (0.99, 1.04)	2.16E-01
0.92 (0.90, 0.93)	**1.19E-19**	1.00 (0.98, 1.02)	7.55E-01
1.00 (0.99, 1.02)	6.57E-01	1.06 (1.04, 1.08)	**2.91E-07**
1.00 (0.99, 1.02)	6.95E-01	1.05 (1.03, 1.07)	**2.93E-05**
0.96 (0.94, 0.97)	**1.20E-06**	0.94 (0.92, 0.96)	**3.99E-08**
0.95 (0.93, 0.96)	**8.02E-09**	1.05 (1.03, 1.07)	**1.73E-05**

0.95 (0.93, 0.97)	**1.06E-07**	0.93 (0.91, 0.95)	**1.02E-10**
0.98 (0.96, 1.00)	**1.91E-02**	0.95 (0.93, 0.97)	**1.35E-06**
0.95 (0.93, 0.97)	**9.88E-08**	0.93 (0.91, 0.95)	**1.60E-10**
1.00 (0.98, 1.01)	6.62E-01	0.94 (0.92, 0.96)	**2.40E-07**
0.90 (0.88, 0.91)	**7.44E-31**	0.93 (0.91, 0.95)	**1.86E-11**
0.92 (0.90, 0.94)	**5.82E-18**	0.97 (0.95, 0.99)	**1.26E-02**
0.94 (0.92, 0.95)	**3.05E-12**	0.98 (0.96, 1.00)	5.24E-02
0.88 (0.86, 0.89)	**8.49E-43**	0.86 (0.84, 0.87)	**1.67E-42**
0.87 (0.86, 0.89)	**5.31E-39**	0.89 (0.87, 0.91)	**6.88E-22**
0.89 (0.88, 0.91)	**2.14E-31**	0.91 (0.89, 0.93)	**2.18E-16**
0.86 (0.84, 0.87)	**5.49E-51**	0.90 (0.88, 0.92)	**2.60E-17**
0.89 (0.88, 0.91)	**2.58E-32**	0.91 (0.89, 0.93)	**1.12E-16**
0.87 (0.86, 0.89)	**7.92E-46**	0.89 (0.87, 0.92)	**3.01E-21**
0.97 (0.96, 0.99)	**5.50E-03**	1.03 (1.01, 1.06)	**3.22E-03**
0.96 (0.95, 0.98)	**1.36E-04**	1.00 (0.98, 1.03)	6.95E-01
0.91 (0.89, 0.93)	**1.51E-21**	0.90 (0.88, 0.92)	**2.52E-17**
0.93 (0.92, 0.95)	**2.76E-13**	0.88 (0.87, 0.90)	**2.13E-27**
0.86 (0.85, 0.88)	**2.32E-47**	0.90 (0.88, 0.92)	**2.76E-18**
0.97 (0.96, 0.99)	**4.03E-03**	0.91 (0.89, 0.92)	**3.02E-19**
0.93 (0.91, 0.94)	**1.15E-16**	0.87 (0.85, 0.89)	**4.93E-36**
1.06 (1.04, 1.08)	**3.73E-09**	1.14 (1.11, 1.16)	**5.41E-30**
1.08 (1.06, 1.10)	**2.02E-15**	1.09 (1.07, 1.12)	**3.69E-16**
1.11 (1.10, 1.12)	**5.34E-56**	1.08 (1.06, 1.10)	**9.39E-20**
1.03 (1.01, 1.05)	**2.56E-03**	1.06 (1.04, 1.08)	**3.13E-07**
1.10 (1.08, 1.12)	**6.54E-27**	1.04 (1.02, 1.06)	**3.04E-04**
0.98 (0.97, 1.00)	1.07E-01	1.00 (0.98, 1.02)	9.38E-01
0.95 (0.93, 0.97)	**3.16E-08**	0.96 (0.94, 0.98)	**2.06E-04**
0.91 (0.89, 0.92)	**2.91E-23**	0.93 (0.91, 0.96)	**7.41E-08**
0.96 (0.95, 0.98)	**9.64E-05**	0.99 (0.97, 1.01)	3.84E-01
0.91 (0.89, 0.93)	**1.50E-22**	0.95 (0.93, 0.98)	**7.06E-05**
0.91 (0.89, 0.93)	**8.95E-24**	0.96 (0.94, 0.98)	**2.26E-04**
1.04 (1.02, 1.05)	**3.29E-06**	1.05 (1.02, 1.07)	**3.27E-05**
1.03 (1.01, 1.04)	**3.76E-03**	1.03 (1.01, 1.05)	**1.64E-02**
1.09 (1.08, 1.10)	**5.19E-80**	1.04 (1.03, 1.05)	**9.44E-11**
1.07 (1.06, 1.08)	**2.31E-32**	1.05 (1.04, 1.07)	**4.75E-16**
1.08 (1.07, 1.10)	**1.38E-39**	1.06 (1.04, 1.08)	**7.95E-11**
1.04 (1.03, 1.05)	**8.47E-16**	1.06 (1.05, 1.07)	**1.84E-30**
0.85 (0.83, 0.86)	**1.80E-71**	0.87 (0.85, 0.89)	**3.66E-35**
1.11 (1.09, 1.13)	**9.84E-29**	1.17 (1.14, 1.19)	**1.43E-42**
1.03 (1.01, 1.05)	**2.35E-03**	0.96 (0.94, 0.99)	**2.67E-03**
0.92 (0.90, 0.94)	**5.84E-19**	0.96 (0.94, 0.98)	**9.58E-04**
0.88 (0.87, 0.90)	**1.86E-37**	0.91 (0.89, 0.93)	**2.60E-17**
0.87 (0.86, 0.89)	**4.28E-44**	0.89 (0.87, 0.91)	**2.30E-23**
0.86 (0.84, 0.88)	**8.83E-54**	0.90 (0.88, 0.92)	**5.19E-21**
1.02 (1.00, 1.04)	**3.53E-02**	0.94 (0.91, 0.96)	**2.12E-08**
0.93 (0.92, 0.95)	**1.06E-12**	0.87 (0.85, 0.89)	**4.98E-31**
1.10 (1.09, 1.11)	**1.81E-58**	1.08 (1.06, 1.09)	**2.03E-20**
0.90 (0.89, 0.92)	**9.92E-26**	0.97 (0.95, 0.99)	**7.53E-03**

0.88 (0.86, 0.89)	**3.42E-42**	0.89 (0.87, 0.91)	**6.21E-26**
1.05 (1.03, 1.07)	**2.40E-08**	0.96 (0.94, 0.98)	**7.42E-04**
1.04 (1.02, 1.05)	**6.29E-08**	1.04 (1.03, 1.06)	**4.69E-07**
1.09 (1.08, 1.10)	**2.76E-51**	1.05 (1.04, 1.07)	**2.83E-12**
0.92 (0.91, 0.94)	**4.85E-18**	0.90 (0.88, 0.92)	**6.34E-20**
0.92 (0.90, 0.94)	**7.98E-18**	0.98 (0.96, 1.00)	5.45E-02
0.89 (0.87, 0.90)	**4.30E-36**	0.92 (0.90, 0.94)	**1.00E-12**
0.94 (0.92, 0.95)	**9.09E-13**	0.91 (0.89, 0.93)	**9.59E-17**
0.94 (0.92, 0.96)	**4.89E-11**	0.93 (0.91, 0.95)	**3.09E-11**
0.91 (0.90, 0.93)	**2.07E-21**	0.98 (0.96, 1.00)	1.45E-01
0.87 (0.85, 0.89)	**8.12E-47**	0.90 (0.88, 0.92)	**2.04E-20**
1.03 (1.02, 1.05)	**1.89E-04**	0.96 (0.94, 0.98)	**1.14E-03**
0.91 (0.89, 0.92)	**2.41E-25**	0.93 (0.91, 0.95)	**1.06E-11**
0.91 (0.89, 0.93)	**2.05E-23**	0.99 (0.97, 1.01)	2.33E-01
0.87 (0.85, 0.89)	**6.86E-47**	0.90 (0.88, 0.92)	**2.17E-19**
1.03 (1.01, 1.05)	**7.69E-04**	0.95 (0.93, 0.98)	**8.82E-05**
0.92 (0.90, 0.93)	**2.67E-20**	0.95 (0.93, 0.97)	**2.12E-05**
1.03 (1.01, 1.05)	**3.76E-03**	0.94 (0.92, 0.96)	**2.58E-07**
1.01 (0.99, 1.03)	2.64E-01	1.03 (1.01, 1.06)	**1.93E-02**

Ischaemic heart diseases mortality			
Male		Female	
**Adjusted HR (95% CI)**	**P value**	**Adjusted HR (95% CI)**	**P value**
1.01 (0.97, 1.06)	6.25E-01	1.09 (1.01, 1.18)	**3.62E-02**
1.01 (0.96, 1.06)	7.03E-01	1.09 (1.01, 1.18)	**4.62E-02**
1.01 (0.97, 1.06)	5.95E-01	1.09 (1.01, 1.18)	**3.68E-02**
1.00 (0.95, 1.05)	9.43E-01	1.08 (0.99, 1.17)	1.05E-01
0.99 (0.94, 1.04)	7.03E-01	1.07 (0.98, 1.16)	1.42E-01
1.01 (0.96, 1.06)	7.92E-01	1.08 (1.00, 1.17)	7.57E-02
1.01 (0.97, 1.06)	6.36E-01	1.09 (1.01, 1.17)	**3.94E-02**
0.99 (0.94, 1.04)	6.94E-01	1.07 (0.99, 1.17)	1.33E-01
0.98 (0.94, 1.03)	5.75E-01	1.06 (0.98, 1.16)	1.75E-01
0.98 (0.94, 1.03)	5.67E-01	1.06 (0.97, 1.16)	2.04E-01
0.95 (0.91, 1.00)	6.77E-02	1.02 (0.93, 1.11)	7.41E-01
0.93 (0.88, 0.98)	**5.38E-03**	0.99 (0.90, 1.08)	7.73E-01
0.98 (0.93, 1.02)	3.94E-01	1.05 (0.96, 1.14)	3.31E-01
1.00 (0.95, 1.04)	8.61E-01	1.08 (1.00, 1.17)	8.44E-02
0.97 (0.93, 1.02)	3.17E-01	1.05 (0.96, 1.14)	3.62E-01
0.96 (0.92, 1.01)	1.57E-01	1.03 (0.94, 1.12)	5.97E-01
0.98 (0.93, 1.02)	3.99E-01	1.06 (0.97, 1.15)	2.66E-01
0.95 (0.90, 1.00)	**4.79E-02**	1.01 (0.92, 1.11)	8.22E-01
0.93 (0.89, 0.98)	**6.82E-03**	0.98 (0.89, 1.08)	7.13E-01
0.97 (0.92, 1.01)	2.02E-01	1.04 (0.95, 1.14)	4.28E-01
0.96 (0.92, 1.01)	1.80E-01	1.02 (0.94, 1.12)	6.56E-01
0.90 (0.85, 0.94)	**3.06E-05**	0.91 (0.83, 1.00)	6.61E-02
0.91 (0.86, 0.95)	**1.81E-04**	0.93 (0.85, 1.02)	1.43E-01
0.90 (0.85, 0.94)	**2.71E-05**	0.91 (0.83, 0.99)	5.07E-02
0.87 (0.83, 0.91)	**1.00E-07**	0.84 (0.77, 0.92)	**7.25E-04**
0.86 (0.82, 0.91)	**2.32E-08**	0.82 (0.75, 0.90)	**7.13E-05**
0.89 (0.84, 0.93)	**5.49E-06**	0.88 (0.81, 0.97)	**1.64E-02**
0.95 (0.91, 1.00)	6.43E-02	1.01 (0.92, 1.10)	9.01E-01
0.97 (0.93, 1.02)	3.49E-01	1.03 (0.94, 1.13)	5.90E-01
0.96 (0.92, 1.01)	1.51E-01	1.01 (0.92, 1.11)	8.05E-01
0.93 (0.88, 0.97)	**3.80E-03**	0.95 (0.87, 1.04)	3.26E-01
0.93 (0.88, 0.97)	**3.49E-03**	0.95 (0.87, 1.04)	3.26E-01
0.94 (0.90, 0.99)	**2.48E-02**	0.98 (0.89, 1.07)	7.05E-01
0.90 (0.86, 0.95)	**6.51E-05**	0.90 (0.82, 0.99)	**3.97E-02**
1.01 (0.97, 1.06)	6.03E-01	1.10 (1.01, 1.19)	**4.91E-02**
0.97 (0.93, 1.02)	3.49E-01	0.98 (0.89, 1.07)	7.05E-01
0.98 (0.94, 1.03)	5.43E-01	0.98 (0.90, 1.08)	7.16E-01
1.01 (0.97, 1.06)	6.70E-01	1.02 (0.93, 1.11)	7.33E-01
0.93 (0.88, 0.97)	**3.09E-03**	0.89 (0.81, 0.98)	**2.16E-02**
0.91 (0.87, 0.95)	**1.95E-04**	0.87 (0.79, 0.95)	**4.03E-03**

0.97 (0.93, 1.02)	2.93E-01	0.96 (0.88, 1.05)	4.28E-01
1.08 (1.03, 1.13)	**1.46E-03**	1.16 (1.07, 1.25)	**1.08E-03**
0.88 (0.84, 0.92)	**7.61E-07**	0.86 (0.79, 0.94)	**2.73E-03**
0.87 (0.83, 0.92)	**1.59E-07**	0.82 (0.75, 0.90)	**7.66E-05**
0.89 (0.85, 0.93)	**5.85E-06**	0.83 (0.76, 0.91)	**3.02E-04**
0.85 (0.81, 0.90)	**1.28E-09**	0.79 (0.72, 0.87)	**5.13E-06**
0.85 (0.81, 0.89)	**2.77E-10**	0.79 (0.72, 0.86)	**2.51E-06**
0.88 (0.83, 0.92)	**2.29E-07**	0.81 (0.74, 0.89)	**4.42E-05**
1.08 (1.04, 1.13)	**8.53E-04**	1.15 (1.06, 1.25)	**2.02E-03**
0.89 (0.85, 0.94)	**2.31E-05**	0.86 (0.79, 0.95)	**3.83E-03**
0.86 (0.82, 0.90)	**7.51E-09**	0.82 (0.75, 0.90)	**1.10E-04**
0.85 (0.81, 0.90)	**1.28E-09**	0.82 (0.74, 0.90)	**6.51E-05**
0.85 (0.81, 0.89)	**5.62E-10**	0.80 (0.73, 0.88)	**2.36E-05**
0.85 (0.81, 0.89)	**1.28E-09**	0.81 (0.74, 0.89)	**5.40E-05**
0.84 (0.80, 0.89)	**1.39E-10**	0.79 (0.72, 0.86)	**2.51E-06**
1.08 (1.03, 1.13)	**1.64E-03**	1.14 (1.06, 1.24)	**2.07E-03**
0.88 (0.84, 0.92)	**8.52E-07**	0.87 (0.80, 0.96)	**8.01E-03**
0.87 (0.83, 0.92)	**1.70E-07**	0.86 (0.79, 0.95)	**4.42E-03**
0.87 (0.83, 0.92)	**1.96E-07**	0.87 (0.79, 0.95)	**5.79E-03**
0.86 (0.82, 0.90)	**1.06E-08**	0.85 (0.77, 0.93)	**1.14E-03**
0.87 (0.83, 0.91)	**7.75E-08**	0.86 (0.79, 0.95)	**3.91E-03**
0.85 (0.81, 0.89)	**4.60E-10**	0.81 (0.74, 0.89)	**4.80E-05**
1.06 (1.01, 1.11)	**1.86E-02**	1.13 (1.05, 1.22)	**3.75E-03**
0.90 (0.85, 0.94)	**2.61E-05**	0.90 (0.82, 0.99)	**3.30E-02**
0.89 (0.85, 0.93)	**8.21E-06**	0.88 (0.80, 0.96)	**8.37E-03**
0.91 (0.87, 0.96)	**3.33E-04**	0.89 (0.81, 0.98)	**1.85E-02**
0.87 (0.83, 0.91)	**1.18E-07**	0.85 (0.78, 0.93)	**1.49E-03**
0.88 (0.84, 0.92)	**7.07E-07**	0.87 (0.79, 0.95)	**4.67E-03**
0.87 (0.83, 0.91)	**3.26E-08**	0.83 (0.76, 0.91)	**1.23E-04**
1.03 (0.99, 1.08)	1.99E-01	1.11 (1.03, 1.20)	**1.48E-02**
1.00 (0.96, 1.05)	8.77E-01	0.84 (0.76, 0.94)	**3.45E-03**
1.02 (0.97, 1.07)	4.98E-01	0.85 (0.76, 0.94)	**4.19E-03**
1.03 (0.98, 1.08)	2.72E-01	0.85 (0.77, 0.95)	**7.51E-03**
1.00 (0.95, 1.05)	9.76E-01	0.83 (0.74, 0.92)	**1.25E-03**
0.99 (0.94, 1.04)	7.27E-01	0.81 (0.73, 0.91)	**5.18E-04**
1.04 (0.99, 1.08)	1.72E-01	0.88 (0.80, 0.98)	**2.48E-02**
1.04 (0.99, 1.09)	1.31E-01	1.07 (0.98, 1.17)	1.36E-01
0.96 (0.92, 1.01)	1.56E-01	0.79 (0.71, 0.89)	**1.35E-04**
0.97 (0.92, 1.02)	2.86E-01	0.81 (0.72, 0.90)	**3.01E-04**
0.98 (0.93, 1.03)	4.41E-01	0.82 (0.73, 0.91)	**5.98E-04**
0.96 (0.91, 1.01)	1.54E-01	0.79 (0.71, 0.88)	**9.83E-05**
0.96 (0.91, 1.00)	1.07E-01	0.79 (0.70, 0.88)	**7.13E-05**
0.98 (0.93, 1.03)	4.47E-01	0.81 (0.72, 0.90)	**3.93E-04**
1.02 (0.97, 1.07)	5.17E-01	1.03 (0.94, 1.13)	5.89E-01
0.91 (0.86, 0.95)	**1.31E-04**	0.80 (0.72, 0.88)	**4.83E-05**
0.92 (0.88, 0.96)	**8.89E-04**	0.82 (0.74, 0.91)	**3.37E-04**
0.93 (0.89, 0.97)	**3.97E-03**	0.85 (0.77, 0.93)	**1.90E-03**
0.90 (0.86, 0.95)	**1.00E-04**	0.79 (0.71, 0.87)	**1.75E-05**

0.90 (0.86, 0.95)	**7.77E-05**	0.79 (0.71, 0.87)	**1.57E-05**
0.91 (0.87, 0.96)	**4.58E-04**	0.79 (0.72, 0.88)	**4.42E-05**
1.01 (0.96, 1.06)	7.03E-01	1.09 (1.00, 1.19)	6.58E-02
0.85 (0.81, 0.89)	**2.84E-10**	0.83 (0.75, 0.92)	**4.34E-04**
0.88 (0.84, 0.93)	**1.09E-06**	0.88 (0.80, 0.97)	**1.40E-02**
0.89 (0.85, 0.94)	**1.40E-05**	0.89 (0.80, 0.97)	**2.00E-02**
0.85 (0.81, 0.89)	**3.19E-10**	0.82 (0.75, 0.90)	**1.89E-04**
0.84 (0.80, 0.88)	**2.02E-11**	0.82 (0.74, 0.90)	**1.18E-04**
0.90 (0.86, 0.94)	**2.38E-05**	0.86 (0.78, 0.95)	**4.64E-03**
1.03 (0.98, 1.08)	2.44E-01	1.15 (1.06, 1.26)	**2.25E-03**
1.07 (1.02, 1.12)	**1.10E-02**	1.34 (1.19, 1.50)	**7.82E-06**
0.95 (0.91, 1.01)	1.22E-01	0.98 (0.88, 1.09)	7.13E-01
0.94 (0.89, 1.00)	6.11E-02	0.95 (0.84, 1.07)	4.28E-01
0.99 (0.94, 1.03)	6.01E-01	1.03 (0.93, 1.14)	5.96E-01
1.02 (0.97, 1.07)	4.65E-01	0.95 (0.86, 1.05)	3.35E-01
0.95 (0.90, 1.00)	8.74E-02	0.81 (0.72, 0.92)	**2.14E-03**
0.95 (0.90, 1.00)	5.71E-02	0.81 (0.71, 0.92)	**2.22E-03**
0.99 (0.94, 1.03)	6.10E-01	0.86 (0.77, 0.97)	**1.70E-02**
1.05 (1.00, 1.11)	5.97E-02	1.19 (1.06, 1.33)	**3.91E-03**
1.04 (0.99, 1.10)	1.47E-01	1.24 (1.08, 1.42)	**4.03E-03**
0.99 (0.95, 1.04)	8.47E-01	0.99 (0.90, 1.09)	8.96E-01
0.97 (0.93, 1.02)	3.37E-01	0.93 (0.84, 1.02)	1.63E-01
1.05 (1.01, 1.10)	**4.45E-02**	1.19 (1.07, 1.32)	**2.07E-03**
0.99 (0.94, 1.03)	6.02E-01	0.92 (0.82, 1.02)	1.36E-01
0.91 (0.87, 0.95)	**8.53E-05**	0.84 (0.77, 0.91)	**4.74E-05**
0.90 (0.86, 0.95)	**3.35E-05**	0.80 (0.73, 0.87)	**1.12E-06**
0.90 (0.86, 0.94)	**2.38E-05**	0.79 (0.72, 0.86)	**6.50E-07**
0.91 (0.87, 0.96)	**1.58E-04**	0.82 (0.75, 0.89)	**1.18E-05**
1.11 (1.06, 1.16)	**3.23E-05**	1.24 (1.14, 1.35)	**1.98E-06**
0.86 (0.82, 0.90)	**3.62E-09**	0.75 (0.68, 0.82)	**9.65E-09**
0.90 (0.86, 0.94)	**1.33E-05**	0.82 (0.75, 0.89)	**2.53E-05**
0.94 (0.89, 0.98)	**7.22E-03**	0.89 (0.82, 0.97)	**1.64E-02**
0.86 (0.82, 0.90)	**9.68E-10**	0.74 (0.67, 0.81)	**1.18E-09**
1.13 (1.08, 1.18)	**1.07E-06**	1.26 (1.15, 1.38)	**2.06E-06**
1.28 (1.23, 1.35)	**1.04E-23**	1.43 (1.31, 1.57)	**3.54E-13**
0.84 (0.80, 0.88)	**1.18E-13**	0.74 (0.69, 0.80)	**6.24E-13**
0.83 (0.79, 0.87)	**3.84E-15**	0.73 (0.68, 0.79)	**1.67E-13**
0.93 (0.89, 0.97)	**1.29E-03**	0.84 (0.78, 0.91)	**4.18E-05**
1.16 (1.11, 1.21)	**7.06E-10**	1.31 (1.21, 1.41)	**8.76E-11**
1.20 (1.15, 1.26)	**7.31E-14**	1.22 (1.11, 1.33)	**9.13E-05**
0.81 (0.78, 0.84)	**1.04E-23**	0.74 (0.69, 0.80)	**4.38E-15**
0.78 (0.75, 0.81)	**2.29E-30**	0.72 (0.67, 0.77)	**4.16E-16**
0.94 (0.90, 0.98)	**8.56E-03**	0.87 (0.81, 0.95)	**2.50E-03**
1.21 (1.16, 1.25)	**3.70E-20**	1.32 (1.24, 1.40)	**5.91E-16**
1.00 (0.95, 1.04)	9.10E-01	0.98 (0.89, 1.07)	6.94E-01
0.91 (0.89, 0.93)	**1.37E-19**	0.90 (0.88, 0.93)	**1.46E-15**
0.92 (0.90, 0.94)	**2.90E-12**	0.91 (0.88, 0.95)	**9.66E-07**
0.87 (0.84, 0.91)	**8.11E-11**	0.80 (0.75, 0.85)	**5.35E-11**

1.14 (1.11, 1.17)	**2.12E-23**	1.15 (1.11, 1.18)	**1.64E-19**
1.06 (1.02, 1.10)	**1.10E-02**	1.11 (1.01, 1.22)	5.08E-02
0.90 (0.89, 0.92)	**3.35E-23**	0.90 (0.88, 0.92)	**1.75E-15**
0.90 (0.87, 0.94)	**3.61E-08**	0.90 (0.83, 0.96)	**4.89E-03**
0.91 (0.87, 0.95)	**2.50E-05**	0.82 (0.76, 0.89)	**4.26E-06**
1.14 (1.11, 1.17)	**3.98E-23**	1.10 (1.08, 1.13)	**4.71E-14**
1.14 (1.10, 1.18)	**3.41E-11**	1.13 (1.03, 1.24)	**1.32E-02**
0.86 (0.84, 0.89)	**8.63E-31**	0.87 (0.84, 0.90)	**3.91E-15**
0.87 (0.84, 0.90)	**2.68E-12**	0.88 (0.82, 0.94)	**1.18E-03**
0.92 (0.88, 0.96)	**1.52E-04**	0.85 (0.79, 0.92)	**8.48E-05**
1.11 (1.07, 1.15)	**6.65E-09**	1.15 (1.10, 1.20)	**1.68E-09**
1.04 (0.99, 1.09)	1.52E-01	0.96 (0.89, 1.03)	3.02E-01
0.95 (0.90, 1.00)	**4.64E-02**	0.99 (0.91, 1.08)	8.14E-01
0.92 (0.88, 0.96)	**1.51E-04**	0.89 (0.83, 0.96)	**3.75E-03**
1.02 (0.98, 1.07)	3.27E-01	1.10 (1.03, 1.17)	**6.83E-03**
1.00 (0.96, 1.04)	9.72E-01	1.06 (1.00, 1.13)	**4.99E-02**
1.09 (1.05, 1.14)	**1.11E-04**	1.18 (1.10, 1.26)	**2.55E-05**
0.92 (0.89, 0.96)	**2.76E-04**	0.86 (0.80, 0.92)	**2.56E-05**
0.92 (0.88, 0.96)	**9.30E-05**	0.85 (0.79, 0.91)	**1.75E-05**
0.97 (0.93, 1.01)	2.36E-01	0.92 (0.86, 0.98)	**2.04E-02**
1.05 (1.01, 1.09)	**1.38E-02**	1.10 (1.04, 1.16)	**5.92E-04**
1.14 (1.09, 1.19)	**8.44E-08**	1.29 (1.19, 1.40)	**2.98E-08**
0.90 (0.86, 0.94)	**8.50E-06**	0.81 (0.75, 0.87)	**1.38E-07**
0.91 (0.87, 0.96)	**1.79E-04**	0.82 (0.76, 0.89)	**5.27E-06**
0.88 (0.85, 0.93)	**4.93E-07**	0.78 (0.71, 0.85)	**9.89E-08**
1.08 (1.04, 1.12)	**1.78E-04**	1.17 (1.11, 1.25)	**6.94E-07**
1.06 (1.01, 1.11)	**2.68E-02**	1.03 (0.94, 1.14)	5.57E-01
0.90 (0.85, 0.94)	**8.45E-06**	0.82 (0.75, 0.89)	**3.18E-05**
0.89 (0.85, 0.93)	**2.05E-06**	0.83 (0.77, 0.91)	**9.83E-05**
1.03 (0.98, 1.07)	3.09E-01	0.95 (0.86, 1.04)	3.07E-01
1.12 (1.07, 1.17)	**3.49E-06**	1.27 (1.17, 1.38)	**8.33E-08**
0.95 (0.91, 1.00)	5.33E-02	1.05 (0.95, 1.15)	3.83E-01
0.99 (0.94, 1.03)	6.01E-01	0.89 (0.81, 0.97)	**9.99E-03**
0.99 (0.94, 1.04)	6.67E-01	0.86 (0.77, 0.95)	**6.19E-03**
0.85 (0.81, 0.89)	**6.37E-10**	0.78 (0.71, 0.86)	**1.74E-06**
0.92 (0.87, 0.96)	**1.17E-03**	0.94 (0.86, 1.03)	2.28E-01
0.85 (0.81, 0.90)	**1.28E-09**	0.82 (0.74, 0.90)	**8.03E-05**
0.88 (0.84, 0.92)	**5.73E-07**	0.86 (0.78, 0.94)	**2.35E-03**
0.85 (0.81, 0.89)	**2.37E-10**	0.77 (0.70, 0.85)	**6.50E-07**
0.86 (0.82, 0.91)	**1.74E-08**	0.81 (0.73, 0.88)	**2.55E-05**
1.00 (0.96, 1.05)	9.51E-01	1.08 (0.99, 1.17)	9.73E-02
0.99 (0.94, 1.04)	7.03E-01	1.06 (0.98, 1.16)	1.80E-01
1.07 (1.02, 1.12)	**6.70E-03**	1.14 (1.05, 1.23)	**2.78E-03**
1.02 (0.98, 1.07)	4.27E-01	1.10 (1.01, 1.20)	**3.62E-02**
0.91 (0.86, 0.95)	**1.13E-04**	0.83 (0.76, 0.92)	**6.05E-04**
1.06 (1.02, 1.11)	**1.35E-02**	1.18 (1.08, 1.28)	**2.98E-04**

0.88 (0.84, 0.93)	**1.23E-06**	0.80 (0.73, 0.89)	**4.97E-05**
0.92 (0.88, 0.96)	**7.86E-04**	0.83 (0.76, 0.91)	**3.02E-04**
0.89 (0.85, 0.93)	**4.64E-06**	0.81 (0.73, 0.89)	**4.76E-05**
0.90 (0.86, 0.95)	**6.56E-05**	0.79 (0.71, 0.87)	**2.14E-05**
0.89 (0.85, 0.93)	**8.21E-06**	0.88 (0.80, 0.96)	**8.37E-03**
0.97 (0.92, 1.02)	2.32E-01	0.99 (0.91, 1.08)	8.64E-01
0.96 (0.92, 1.01)	1.35E-01	0.97 (0.88, 1.06)	5.74E-01
0.86 (0.82, 0.90)	**4.34E-09**	0.74 (0.67, 0.81)	**1.52E-09**
0.85 (0.81, 0.90)	**1.37E-08**	0.76 (0.68, 0.85)	**5.35E-06**
0.86 (0.82, 0.90)	**9.75E-09**	0.79 (0.72, 0.87)	**1.16E-05**
0.88 (0.84, 0.93)	**4.64E-06**	0.84 (0.75, 0.93)	**1.82E-03**
0.87 (0.83, 0.92)	**2.73E-07**	0.80 (0.73, 0.88)	**3.67E-05**
0.86 (0.82, 0.91)	**1.71E-08**	0.79 (0.71, 0.87)	**1.12E-05**
1.03 (0.99, 1.08)	2.26E-01	1.10 (1.01, 1.19)	**4.30E-02**
0.98 (0.94, 1.03)	5.18E-01	1.02 (0.93, 1.11)	7.13E-01
0.88 (0.84, 0.93)	**8.50E-06**	0.80 (0.72, 0.89)	**8.44E-05**
0.84 (0.80, 0.88)	**3.60E-12**	0.72 (0.66, 0.79)	**2.15E-11**
0.89 (0.84, 0.94)	**2.26E-05**	0.84 (0.75, 0.93)	**1.97E-03**
0.90 (0.86, 0.95)	**3.23E-05**	0.79 (0.73, 0.86)	**5.37E-07**
0.87 (0.83, 0.91)	**1.71E-08**	0.75 (0.69, 0.82)	**4.49E-10**
1.19 (1.13, 1.24)	**1.14E-11**	1.37 (1.25, 1.49)	**5.67E-11**
1.05 (1.00, 1.10)	7.61E-02	1.15 (1.05, 1.26)	**3.52E-03**
1.19 (1.16, 1.22)	**4.28E-38**	1.20 (1.15, 1.25)	**4.16E-16**
1.06 (1.01, 1.11)	**2.23E-02**	1.12 (1.02, 1.22)	**2.13E-02**
1.14 (1.09, 1.19)	**7.61E-08**	1.08 (1.02, 1.15)	**9.11E-03**
1.08 (1.03, 1.13)	**1.81E-03**	1.11 (1.01, 1.21)	**3.16E-02**
0.93 (0.88, 0.97)	**2.48E-03**	0.90 (0.82, 0.99)	**4.89E-02**
0.89 (0.85, 0.94)	**2.64E-05**	0.86 (0.77, 0.96)	**1.06E-02**
1.03 (0.99, 1.08)	2.11E-01	1.14 (1.05, 1.24)	**3.75E-03**
1.00 (0.96, 1.05)	9.51E-01	1.11 (1.02, 1.21)	**2.41E-02**
1.03 (0.98, 1.07)	3.49E-01	1.12 (1.02, 1.22)	**2.11E-02**
1.05 (1.02, 1.08)	**1.78E-03**	1.11 (1.02, 1.20)	**1.70E-02**
1.05 (1.01, 1.10)	**3.04E-02**	1.14 (1.05, 1.24)	**4.64E-03**
1.06 (1.03, 1.09)	**3.44E-05**	1.04 (0.99, 1.09)	1.89E-01
1.07 (1.05, 1.10)	**5.02E-09**	1.06 (1.02, 1.11)	**9.99E-03**
1.08 (1.04, 1.11)	**4.20E-06**	1.07 (0.99, 1.15)	8.88E-02
1.06 (1.05, 1.08)	**1.59E-13**	1.08 (1.05, 1.10)	**2.02E-09**
0.86 (0.82, 0.90)	**5.02E-09**	0.76 (0.69, 0.83)	**8.88E-08**
1.24 (1.18, 1.29)	**6.03E-19**	1.28 (1.17, 1.40)	**6.42E-07**
0.94 (0.90, 0.99)	**2.08E-02**	0.84 (0.75, 0.93)	**1.70E-03**
1.02 (0.97, 1.07)	4.51E-01	1.12 (1.03, 1.23)	**1.15E-02**
0.86 (0.82, 0.91)	**1.52E-08**	0.83 (0.76, 0.92)	**3.72E-04**
0.85 (0.81, 0.89)	**5.96E-10**	0.81 (0.74, 0.89)	**4.42E-05**
0.86 (0.82, 0.90)	**4.51E-09**	0.83 (0.75, 0.91)	**2.40E-04**
0.90 (0.86, 0.94)	**5.88E-05**	0.75 (0.68, 0.84)	**1.12E-06**
0.84 (0.80, 0.89)	**2.88E-10**	0.70 (0.64, 0.78)	**8.76E-11**
1.05 (1.00, 1.09)	**4.49E-02**	1.08 (1.01, 1.15)	**4.62E-02**
0.94 (0.89, 0.98)	**1.46E-02**	0.97 (0.89, 1.07)	6.24E-01

0.85 (0.81, 0.89)	**2.62E-10**	0.79 (0.72, 0.87)	**7.54E-06**
0.93 (0.89, 0.98)	**6.90E-03**	0.78 (0.70, 0.87)	**3.84E-05**
1.06 (1.03, 1.09)	**9.24E-05**	1.05 (0.99, 1.12)	1.51E-01
1.09 (1.06, 1.12)	**7.32E-08**	1.03 (0.94, 1.12)	6.02E-01
0.86 (0.82, 0.90)	**4.65E-09**	0.76 (0.69, 0.84)	**4.55E-07**
0.95 (0.91, 1.00)	6.77E-02	0.99 (0.90, 1.08)	8.35E-01
0.89 (0.85, 0.93)	**6.10E-06**	0.87 (0.79, 0.95)	**5.07E-03**
0.87 (0.83, 0.91)	**5.87E-08**	0.77 (0.69, 0.85)	**1.66E-06**
0.89 (0.85, 0.93)	**3.76E-06**	0.81 (0.74, 0.89)	**5.84E-05**
0.96 (0.91, 1.00)	1.07E-01	1.01 (0.92, 1.10)	8.96E-01
0.86 (0.82, 0.91)	**2.14E-08**	0.84 (0.76, 0.92)	**5.17E-04**
0.93 (0.89, 0.98)	**7.24E-03**	0.81 (0.73, 0.90)	**1.62E-04**
0.89 (0.84, 0.93)	**3.37E-06**	0.85 (0.77, 0.93)	**1.13E-03**
0.96 (0.92, 1.01)	1.63E-01	1.02 (0.93, 1.11)	7.06E-01
0.86 (0.82, 0.91)	**2.63E-08**	0.84 (0.76, 0.92)	**4.79E-04**
0.92 (0.88, 0.97)	**1.63E-03**	0.79 (0.71, 0.88)	**3.72E-05**
0.91 (0.86, 0.95)	**1.73E-04**	0.92 (0.84, 1.01)	8.75E-02
0.90 (0.86, 0.95)	**1.53E-04**	0.76 (0.68, 0.84)	**2.06E-06**
0.98 (0.94, 1.03)	4.61E-01	1.03 (0.91, 1.15)	7.13E-01

Cerebrovascular diseases mortality			
Male		Female	
**Adjusted HR (95% CI)**	**P value**	**Adjusted HR (95% CI)**	**P value**
0.88 (0.79, 0.97)	**3.09E-02**	0.99 (0.91, 1.09)	9.15E-01
0.88 (0.79, 0.97)	**3.08E-02**	0.99 (0.90, 1.09)	9.01E-01
0.88 (0.80, 0.98)	**3.54E-02**	1.00 (0.91, 1.10)	9.89E-01
0.88 (0.80, 0.98)	**3.54E-02**	0.99 (0.91, 1.09)	9.18E-01
0.88 (0.80, 0.98)	**3.56E-02**	0.99 (0.90, 1.09)	9.01E-01
0.89 (0.80, 0.98)	**3.66E-02**	1.00 (0.91, 1.09)	9.49E-01
0.88 (0.79, 0.97)	**2.93E-02**	0.99 (0.90, 1.08)	8.70E-01
0.85 (0.77, 0.94)	**8.81E-03**	0.96 (0.87, 1.05)	4.90E-01
0.85 (0.77, 0.94)	**8.81E-03**	0.95 (0.87, 1.05)	4.48E-01
0.86 (0.78, 0.95)	**1.10E-02**	0.95 (0.87, 1.05)	4.66E-01
0.85 (0.77, 0.94)	**8.68E-03**	0.93 (0.85, 1.03)	2.83E-01
0.85 (0.77, 0.94)	**8.43E-03**	0.92 (0.83, 1.01)	1.77E-01
0.86 (0.78, 0.95)	**1.14E-02**	0.95 (0.86, 1.05)	4.28E-01
0.85 (0.77, 0.95)	**1.04E-02**	0.96 (0.87, 1.06)	5.32E-01
0.85 (0.77, 0.94)	**8.43E-03**	0.94 (0.85, 1.04)	3.55E-01
0.85 (0.77, 0.94)	**6.88E-03**	0.93 (0.85, 1.03)	2.89E-01
0.85 (0.77, 0.94)	**8.43E-03**	0.95 (0.86, 1.05)	4.28E-01
0.86 (0.78, 0.94)	**8.81E-03**	0.93 (0.84, 1.03)	2.77E-01
0.87 (0.79, 0.96)	**1.49E-02**	0.93 (0.84, 1.02)	2.27E-01
0.85 (0.77, 0.94)	**6.88E-03**	0.94 (0.85, 1.03)	3.32E-01
0.85 (0.76, 0.93)	**6.72E-03**	0.93 (0.84, 1.02)	2.51E-01
0.86 (0.78, 0.95)	**1.24E-02**	0.88 (0.80, 0.97)	**4.06E-02**
0.86 (0.78, 0.94)	**8.64E-03**	0.89 (0.80, 0.98)	5.22E-02
0.87 (0.79, 0.96)	**1.41E-02**	0.88 (0.80, 0.97)	**4.06E-02**
0.91 (0.83, 1.00)	8.84E-02	0.87 (0.79, 0.96)	**1.76E-02**
0.94 (0.85, 1.03)	2.60E-01	0.87 (0.79, 0.95)	**1.54E-02**
0.88 (0.80, 0.97)	**2.52E-02**	0.88 (0.80, 0.97)	**3.31E-02**
0.85 (0.77, 0.94)	**7.68E-03**	0.92 (0.84, 1.02)	2.15E-01
0.89 (0.81, 0.99)	**4.67E-02**	0.94 (0.86, 1.04)	3.79E-01
0.89 (0.80, 0.98)	**3.44E-02**	0.93 (0.85, 1.03)	2.97E-01
0.89 (0.80, 0.98)	**3.09E-02**	0.90 (0.82, 0.99)	9.79E-02
0.90 (0.82, 0.99)	5.41E-02	0.91 (0.82, 1.00)	1.28E-01
0.90 (0.82, 0.99)	6.50E-02	0.92 (0.84, 1.02)	2.15E-01
0.89 (0.81, 0.98)	**4.23E-02**	0.88 (0.80, 0.97)	**4.06E-02**
0.90 (0.82, 0.99)	6.63E-02	0.99 (0.90, 1.09)	8.63E-01
0.96 (0.88, 1.06)	5.41E-01	0.94 (0.86, 1.04)	3.59E-01
0.97 (0.88, 1.07)	6.21E-01	0.95 (0.87, 1.05)	4.48E-01
0.98 (0.90, 1.08)	7.71E-01	0.97 (0.88, 1.07)	6.40E-01
0.98 (0.89, 1.07)	6.82E-01	0.92 (0.83, 1.01)	1.68E-01
0.98 (0.89, 1.07)	6.71E-01	0.91 (0.83, 1.00)	1.14E-01

0.98 (0.89, 1.07)	7.28E-01	0.95 (0.86, 1.04)	3.75E-01
0.96 (0.87, 1.05)	4.68E-01	1.04 (0.94, 1.14)	5.52E-01
0.90 (0.82, 0.99)	6.33E-02	0.85 (0.78, 0.94)	**7.91E-03**
0.92 (0.84, 1.01)	1.41E-01	0.85 (0.78, 0.94)	**7.23E-03**
0.94 (0.85, 1.03)	2.32E-01	0.87 (0.79, 0.96)	**2.30E-02**
0.92 (0.84, 1.01)	1.25E-01	0.84 (0.76, 0.92)	**2.35E-03**
0.91 (0.83, 1.00)	9.10E-02	0.83 (0.76, 0.92)	**2.11E-03**
0.94 (0.86, 1.04)	3.04E-01	0.86 (0.78, 0.94)	**8.13E-03**
0.97 (0.88, 1.07)	5.92E-01	1.03 (0.94, 1.14)	5.94E-01
0.90 (0.82, 0.99)	5.51E-02	0.87 (0.79, 0.95)	**1.54E-02**
0.87 (0.79, 0.95)	**1.22E-02**	0.84 (0.76, 0.92)	**2.59E-03**
0.87 (0.79, 0.96)	**1.54E-02**	0.83 (0.75, 0.91)	**2.11E-03**
0.86 (0.78, 0.95)	**1.13E-02**	0.83 (0.75, 0.92)	**2.17E-03**
0.86 (0.78, 0.94)	**8.43E-03**	0.83 (0.75, 0.92)	**2.26E-03**
0.89 (0.81, 0.98)	**3.09E-02**	0.83 (0.76, 0.92)	**2.17E-03**
0.95 (0.86, 1.04)	3.48E-01	1.01 (0.92, 1.11)	9.01E-01
0.86 (0.78, 0.95)	**1.09E-02**	0.85 (0.77, 0.94)	**8.39E-03**
0.83 (0.76, 0.92)	**2.29E-03**	0.83 (0.75, 0.92)	**2.26E-03**
0.83 (0.75, 0.92)	**2.29E-03**	0.83 (0.75, 0.91)	**2.11E-03**
0.83 (0.75, 0.92)	**2.29E-03**	0.83 (0.75, 0.91)	**2.09E-03**
0.83 (0.75, 0.91)	**1.91E-03**	0.83 (0.75, 0.92)	**2.26E-03**
0.86 (0.78, 0.94)	**8.43E-03**	0.82 (0.74, 0.90)	**1.59E-03**
0.91 (0.83, 1.01)	1.25E-01	1.00 (0.91, 1.09)	9.38E-01
0.87 (0.79, 0.95)	**1.24E-02**	0.86 (0.78, 0.95)	**1.13E-02**
0.85 (0.77, 0.94)	**7.68E-03**	0.84 (0.76, 0.92)	**2.53E-03**
0.88 (0.80, 0.97)	**2.47E-02**	0.84 (0.76, 0.92)	**2.35E-03**
0.85 (0.77, 0.93)	**5.45E-03**	0.83 (0.75, 0.92)	**2.11E-03**
0.84 (0.76, 0.93)	**3.54E-03**	0.84 (0.76, 0.93)	**3.50E-03**
0.88 (0.80, 0.97)	**2.73E-02**	0.82 (0.75, 0.91)	**1.59E-03**
0.90 (0.81, 0.99)	6.76E-02	0.99 (0.90, 1.09)	9.01E-01
1.17 (1.08, 1.26)	**8.75E-04**	1.01 (0.92, 1.12)	8.63E-01
1.19 (1.10, 1.28)	**2.62E-04**	1.03 (0.93, 1.13)	6.90E-01
1.20 (1.11, 1.29)	**1.70E-04**	1.04 (0.94, 1.15)	5.31E-01
1.18 (1.10, 1.28)	**3.94E-04**	1.01 (0.91, 1.11)	9.12E-01
1.18 (1.09, 1.27)	**5.11E-04**	1.01 (0.91, 1.11)	9.34E-01
1.18 (1.10, 1.28)	**3.94E-04**	1.02 (0.92, 1.12)	8.59E-01
1.00 (0.91, 1.10)	9.76E-01	1.03 (0.94, 1.13)	5.82E-01
1.15 (1.06, 1.25)	**4.12E-03**	1.01 (0.92, 1.11)	9.01E-01
1.15 (1.06, 1.25)	**4.22E-03**	1.01 (0.92, 1.12)	8.63E-01
1.15 (1.05, 1.24)	**7.68E-03**	1.01 (0.92, 1.12)	8.63E-01
1.16 (1.07, 1.26)	**2.43E-03**	1.01 (0.91, 1.11)	9.01E-01
1.16 (1.07, 1.26)	**2.43E-03**	1.01 (0.91, 1.11)	9.01E-01
1.16 (1.07, 1.26)	**2.70E-03**	1.01 (0.91, 1.11)	9.01E-01
1.03 (0.94, 1.13)	6.32E-01	1.06 (0.97, 1.17)	3.37E-01
0.98 (0.90, 1.08)	7.54E-01	0.96 (0.87, 1.06)	4.91E-01
0.97 (0.89, 1.07)	6.50E-01	0.96 (0.87, 1.06)	5.43E-01
0.96 (0.88, 1.06)	5.29E-01	0.97 (0.88, 1.07)	6.14E-01
1.00 (0.91, 1.09)	9.46E-01	0.95 (0.86, 1.05)	4.32E-01

1.00 (0.91, 1.09)	9.41E-01	0.95 (0.86, 1.05)	4.32E-01
1.00 (0.91, 1.09)	9.73E-01	0.95 (0.86, 1.05)	4.32E-01
0.91 (0.83, 1.01)	1.16E-01	1.03 (0.94, 1.14)	5.89E-01
0.81 (0.73, 0.89)	**3.80E-04**	0.85 (0.77, 0.94)	**7.90E-03**
0.82 (0.75, 0.91)	**9.86E-04**	0.89 (0.80, 0.98)	5.90E-02
0.84 (0.76, 0.92)	**2.43E-03**	0.91 (0.82, 1.00)	1.31E-01
0.82 (0.75, 0.90)	**9.30E-04**	0.85 (0.77, 0.94)	**8.34E-03**
0.82 (0.74, 0.90)	**5.50E-04**	0.85 (0.77, 0.94)	**7.23E-03**
0.86 (0.79, 0.95)	**1.03E-02**	0.88 (0.80, 0.97)	**4.26E-02**
0.87 (0.79, 0.96)	**2.07E-02**	1.01 (0.92, 1.11)	8.69E-01
1.09 (1.00, 1.20)	8.50E-02	1.10 (0.98, 1.23)	2.15E-01
1.06 (0.97, 1.16)	2.78E-01	1.11 (1.02, 1.22)	6.32E-02
1.04 (0.94, 1.14)	5.51E-01	1.08 (0.98, 1.19)	2.30E-01
1.09 (1.00, 1.17)	7.01E-02	1.15 (1.05, 1.25)	**8.49E-03**
0.93 (0.85, 1.01)	1.45E-01	0.88 (0.80, 0.97)	**3.53E-02**
1.11 (1.02, 1.21)	**3.47E-02**	1.07 (0.97, 1.18)	3.08E-01
1.09 (1.00, 1.18)	1.02E-01	1.06 (0.96, 1.17)	4.11E-01
1.17 (1.08, 1.26)	**1.24E-03**	1.11 (1.00, 1.22)	1.04E-01
0.90 (0.82, 0.98)	**3.47E-02**	0.94 (0.85, 1.04)	3.32E-01
0.92 (0.85, 0.99)	6.40E-02	1.02 (0.91, 1.13)	8.59E-01
1.11 (1.02, 1.21)	**3.56E-02**	1.06 (0.97, 1.17)	3.37E-01
1.14 (1.04, 1.24)	**1.19E-02**	1.06 (0.97, 1.17)	3.35E-01
1.01 (0.92, 1.11)	8.77E-01	1.05 (0.94, 1.16)	5.26E-01
0.96 (0.88, 1.05)	4.86E-01	0.94 (0.85, 1.05)	4.21E-01
1.03 (0.94, 1.12)	6.21E-01	0.95 (0.87, 1.04)	3.83E-01
1.09 (1.00, 1.19)	1.03E-01	0.95 (0.86, 1.04)	3.71E-01
1.10 (1.00, 1.21)	6.76E-02	0.95 (0.86, 1.04)	3.68E-01
1.06 (0.97, 1.16)	3.04E-01	0.95 (0.87, 1.04)	4.21E-01
0.93 (0.85, 1.02)	1.59E-01	1.06 (0.96, 1.16)	3.69E-01
0.99 (0.90, 1.09)	8.92E-01	0.87 (0.79, 0.95)	**1.54E-02**
1.02 (0.93, 1.12)	7.07E-01	0.92 (0.84, 1.01)	1.60E-01
1.04 (0.95, 1.14)	5.37E-01	0.96 (0.87, 1.05)	5.10E-01
1.00 (0.91, 1.10)	9.73E-01	0.87 (0.79, 0.96)	**2.26E-02**
0.99 (0.90, 1.08)	8.13E-01	1.11 (1.01, 1.22)	7.86E-02
1.11 (1.01, 1.22)	**4.64E-02**	1.18 (1.07, 1.30)	**5.13E-03**
1.01 (0.92, 1.11)	8.78E-01	0.87 (0.80, 0.95)	**1.07E-02**
1.00 (0.91, 1.10)	9.73E-01	0.87 (0.79, 0.95)	**8.99E-03**
1.05 (0.96, 1.15)	3.76E-01	0.92 (0.85, 1.01)	1.65E-01
0.97 (0.88, 1.06)	5.92E-01	1.13 (1.03, 1.23)	**2.64E-02**
1.12 (1.02, 1.22)	**3.33E-02**	1.21 (1.10, 1.34)	**1.61E-03**
0.92 (0.85, 1.00)	1.02E-01	0.82 (0.76, 0.89)	**9.03E-05**
0.88 (0.81, 0.96)	**1.49E-02**	0.80 (0.73, 0.87)	**2.04E-05**
1.05 (0.96, 1.15)	3.95E-01	0.95 (0.86, 1.04)	3.69E-01
1.06 (0.97, 1.16)	2.40E-01	1.19 (1.10, 1.28)	**5.17E-04**
1.08 (1.02, 1.15)	**2.20E-02**	0.98 (0.89, 1.08)	8.25E-01
0.92 (0.87, 0.96)	**2.29E-03**	0.91 (0.88, 0.95)	**3.26E-05**
0.91 (0.88, 0.94)	**8.26E-05**	0.91 (0.88, 0.96)	**1.66E-03**
0.99 (0.91, 1.08)	8.87E-01	0.90 (0.82, 0.98)	**4.11E-02**

1.10 (1.03, 1.17)	**1.50E-02**	1.13 (1.08, 1.17)	**1.54E-05**
1.06 (0.99, 1.15)	1.69E-01	1.00 (0.91, 1.11)	9.55E-01
0.92 (0.88, 0.97)	**1.14E-02**	0.91 (0.88, 0.95)	**1.42E-04**
0.88 (0.83, 0.93)	**2.85E-04**	0.91 (0.83, 0.99)	6.78E-02
1.05 (0.96, 1.15)	3.76E-01	0.93 (0.85, 1.03)	2.74E-01
1.09 (1.02, 1.17)	**3.08E-02**	1.10 (1.06, 1.14)	**3.26E-05**
1.16 (1.09, 1.23)	**1.70E-04**	1.05 (0.95, 1.15)	4.66E-01
0.89 (0.84, 0.95)	**2.21E-03**	0.89 (0.85, 0.95)	**1.94E-03**
0.85 (0.80, 0.91)	**2.62E-04**	0.95 (0.86, 1.04)	3.71E-01
1.02 (0.93, 1.12)	7.28E-01	0.91 (0.83, 0.99)	6.57E-02
1.00 (0.91, 1.10)	9.76E-01	1.11 (1.04, 1.19)	**9.86E-03**
1.22 (1.09, 1.36)	**4.10E-03**	1.05 (0.96, 1.16)	4.25E-01
0.89 (0.80, 0.98)	**3.46E-02**	0.89 (0.81, 0.98)	6.57E-02
0.99 (0.91, 1.08)	8.54E-01	0.89 (0.83, 0.97)	**2.25E-02**
0.85 (0.77, 0.95)	**1.09E-02**	0.99 (0.90, 1.08)	8.73E-01
0.86 (0.76, 0.96)	**2.36E-02**	1.04 (0.96, 1.11)	4.76E-01
0.89 (0.82, 0.97)	**1.67E-02**	1.03 (0.94, 1.12)	6.14E-01
1.13 (1.03, 1.24)	**3.09E-02**	0.95 (0.87, 1.03)	3.37E-01
1.13 (1.02, 1.24)	**3.44E-02**	0.95 (0.87, 1.04)	3.76E-01
1.09 (0.99, 1.20)	1.05E-01	0.95 (0.88, 1.03)	3.30E-01
0.92 (0.83, 1.02)	1.42E-01	1.06 (0.99, 1.14)	2.17E-01
0.95 (0.87, 1.05)	3.95E-01	1.09 (0.99, 1.20)	1.59E-01
1.06 (0.97, 1.17)	2.82E-01	0.92 (0.84, 1.01)	1.54E-01
1.06 (0.97, 1.17)	2.94E-01	0.93 (0.85, 1.02)	2.17E-01
1.03 (0.94, 1.13)	5.65E-01	0.90 (0.82, 0.99)	9.79E-02
0.94 (0.85, 1.03)	2.43E-01	1.08 (0.99, 1.18)	1.77E-01
1.09 (0.99, 1.19)	1.34E-01	1.12 (1.02, 1.23)	6.37E-02
0.98 (0.89, 1.08)	7.71E-01	0.88 (0.80, 0.97)	**2.78E-02**
0.95 (0.87, 1.05)	3.95E-01	0.88 (0.81, 0.97)	**3.41E-02**
1.11 (1.03, 1.21)	**2.62E-02**	0.99 (0.89, 1.09)	8.63E-01
0.95 (0.86, 1.04)	3.67E-01	1.08 (0.99, 1.19)	1.91E-01
0.81 (0.75, 0.89)	**2.62E-04**	0.92 (0.83, 1.01)	1.91E-01
1.10 (1.00, 1.21)	8.03E-02	0.99 (0.90, 1.08)	8.63E-01
1.20 (1.11, 1.31)	**3.77E-04**	1.05 (0.95, 1.16)	4.71E-01
0.90 (0.82, 0.99)	**4.86E-02**	0.84 (0.76, 0.93)	**3.51E-03**
0.89 (0.80, 0.98)	**3.09E-02**	0.91 (0.82, 1.00)	1.19E-01
0.85 (0.77, 0.94)	**7.10E-03**	0.83 (0.75, 0.91)	**2.11E-03**
0.89 (0.81, 0.98)	**3.91E-02**	0.86 (0.78, 0.95)	**1.25E-02**
0.90 (0.82, 0.99)	**4.94E-02**	0.84 (0.76, 0.92)	**2.88E-03**
0.90 (0.82, 0.99)	**4.94E-02**	0.85 (0.77, 0.94)	**7.33E-03**
0.87 (0.79, 0.96)	**2.07E-02**	0.97 (0.88, 1.07)	6.22E-01
0.86 (0.78, 0.95)	**1.26E-02**	0.96 (0.87, 1.05)	4.91E-01
0.93 (0.84, 1.03)	2.19E-01	1.00 (0.91, 1.10)	9.49E-01
0.93 (0.84, 1.02)	1.81E-01	1.04 (0.94, 1.14)	5.70E-01
0.92 (0.84, 1.01)	1.09E-01	0.91 (0.82, 1.00)	1.25E-01
0.89 (0.81, 0.98)	**3.47E-02**	1.02 (0.92, 1.12)	8.59E-01

0.92 (0.84, 1.01)	1.17E-01	0.90 (0.81, 1.00)	1.04E-01
0.98 (0.90, 1.08)	7.71E-01	0.89 (0.81, 0.98)	**4.90E-02**
0.92 (0.84, 1.01)	1.41E-01	0.90 (0.81, 0.99)	9.59E-02
0.98 (0.90, 1.08)	7.71E-01	0.95 (0.86, 1.05)	4.25E-01
0.88 (0.80, 0.97)	**2.85E-02**	0.86 (0.78, 0.95)	**1.17E-02**
0.92 (0.83, 1.01)	1.16E-01	0.92 (0.84, 1.01)	1.91E-01
0.88 (0.80, 0.97)	**3.08E-02**	0.92 (0.83, 1.01)	1.91E-01
0.98 (0.89, 1.08)	7.42E-01	0.88 (0.80, 0.97)	**3.88E-02**
0.96 (0.87, 1.06)	5.05E-01	0.87 (0.79, 0.97)	**3.53E-02**
0.89 (0.81, 0.98)	**3.71E-02**	0.89 (0.81, 0.98)	5.56E-02
0.90 (0.81, 0.99)	5.74E-02	0.88 (0.79, 0.97)	**4.06E-02**
0.88 (0.80, 0.97)	**2.44E-02**	0.87 (0.79, 0.96)	**2.53E-02**
0.87 (0.79, 0.96)	**1.49E-02**	0.86 (0.77, 0.95)	**1.13E-02**
0.92 (0.83, 1.01)	1.19E-01	0.98 (0.89, 1.08)	7.50E-01
0.89 (0.80, 0.98)	**3.47E-02**	0.94 (0.85, 1.04)	3.32E-01
1.03 (0.94, 1.13)	5.92E-01	0.93 (0.85, 1.03)	3.19E-01
1.02 (0.93, 1.13)	6.98E-01	0.93 (0.85, 1.03)	2.97E-01
0.95 (0.86, 1.04)	3.45E-01	0.91 (0.82, 1.01)	1.68E-01
1.08 (0.98, 1.18)	1.87E-01	0.96 (0.87, 1.05)	4.88E-01
1.05 (0.96, 1.16)	3.79E-01	0.92 (0.84, 1.01)	1.91E-01
0.98 (0.89, 1.08)	7.71E-01	1.09 (0.99, 1.20)	1.79E-01
0.93 (0.85, 1.02)	1.91E-01	1.04 (0.95, 1.14)	5.30E-01
1.06 (0.98, 1.14)	2.20E-01	1.10 (1.03, 1.18)	**2.53E-02**
1.04 (0.94, 1.13)	5.51E-01	0.97 (0.88, 1.07)	6.17E-01
1.09 (0.99, 1.19)	1.13E-01	1.05 (0.96, 1.14)	4.11E-01
0.97 (0.89, 1.06)	6.23E-01	1.06 (0.97, 1.17)	3.20E-01
1.12 (1.02, 1.22)	**3.47E-02**	1.10 (1.00, 1.22)	1.14E-01
0.97 (0.88, 1.07)	6.73E-01	1.00 (0.92, 1.10)	9.38E-01
0.96 (0.87, 1.05)	4.22E-01	0.96 (0.87, 1.06)	5.82E-01
0.86 (0.78, 0.95)	**1.09E-02**	0.92 (0.83, 1.01)	1.79E-01
0.84 (0.76, 0.93)	**3.54E-03**	0.94 (0.85, 1.04)	3.68E-01
1.01 (0.92, 1.12)	8.18E-01	1.03 (0.93, 1.12)	6.94E-01
0.95 (0.87, 1.04)	3.61E-01	1.05 (0.96, 1.15)	4.25E-01
1.09 (1.05, 1.14)	**5.50E-04**	1.04 (1.00, 1.09)	1.14E-01
1.07 (1.02, 1.14)	**2.77E-02**	1.06 (1.01, 1.12)	**4.06E-02**
1.09 (1.03, 1.16)	**1.28E-02**	1.07 (0.99, 1.15)	1.59E-01
1.05 (1.01, 1.09)	**2.20E-02**	1.07 (1.04, 1.11)	**1.61E-03**
0.89 (0.81, 0.98)	**3.08E-02**	0.92 (0.83, 1.02)	2.15E-01
1.05 (0.96, 1.16)	3.56E-01	1.10 (1.00, 1.22)	1.31E-01
1.18 (1.08, 1.29)	**2.13E-03**	1.02 (0.92, 1.12)	8.59E-01
0.86 (0.78, 0.95)	**1.19E-02**	0.94 (0.85, 1.03)	3.20E-01
0.87 (0.79, 0.96)	**1.41E-02**	0.84 (0.76, 0.93)	**3.71E-03**
0.87 (0.79, 0.96)	**1.31E-02**	0.83 (0.75, 0.91)	**1.94E-03**
0.84 (0.77, 0.93)	**4.48E-03**	0.83 (0.75, 0.92)	**2.17E-03**
1.05 (0.96, 1.15)	3.45E-01	0.95 (0.86, 1.05)	4.79E-01
1.05 (0.95, 1.15)	4.01E-01	0.92 (0.83, 1.02)	2.12E-01
1.09 (1.02, 1.16)	**2.58E-02**	1.06 (0.97, 1.15)	3.19E-01
0.87 (0.79, 0.96)	**1.58E-02**	0.92 (0.83, 1.01)	1.68E-01

0.88 (0.80, 0.97)	**2.07E-02**	0.83 (0.75, 0.91)	**1.94E-03**
1.06 (0.97, 1.16)	2.79E-01	0.96 (0.87, 1.06)	5.54E-01
1.02 (0.94, 1.11)	7.24E-01	0.98 (0.88, 1.10)	8.63E-01
1.11 (1.05, 1.17)	**1.17E-03**	1.07 (1.01, 1.12)	5.59E-02
0.91 (0.83, 1.00)	8.24E-02	0.88 (0.80, 0.97)	**4.06E-02**
0.89 (0.81, 0.98)	**3.69E-02**	0.92 (0.84, 1.02)	2.15E-01
0.88 (0.80, 0.97)	**2.76E-02**	0.86 (0.78, 0.95)	**1.07E-02**
0.92 (0.84, 1.01)	1.47E-01	0.90 (0.81, 1.00)	1.02E-01
0.91 (0.83, 1.00)	8.03E-02	0.88 (0.80, 0.97)	**3.66E-02**
0.87 (0.79, 0.96)	**1.67E-02**	0.93 (0.84, 1.02)	2.60E-01
0.86 (0.78, 0.95)	**8.81E-03**	0.83 (0.75, 0.91)	**2.09E-03**
1.02 (0.93, 1.12)	7.54E-01	0.97 (0.88, 1.08)	6.94E-01
0.87 (0.80, 0.96)	**1.71E-02**	0.86 (0.78, 0.95)	**1.54E-02**
0.86 (0.78, 0.95)	**1.19E-02**	0.93 (0.85, 1.03)	2.94E-01
0.85 (0.77, 0.94)	**7.68E-03**	0.83 (0.75, 0.92)	**2.26E-03**
1.03 (0.94, 1.13)	6.08E-01	0.97 (0.87, 1.07)	6.14E-01
0.90 (0.82, 0.99)	5.84E-02	0.90 (0.82, 0.99)	1.00E-01
1.05 (0.96, 1.15)	3.24E-01	0.96 (0.86, 1.06)	4.90E-01
1.02 (0.93, 1.12)	7.71E-01	0.99 (0.89, 1.11)	9.12E-01

Cancer mortality				
Male		Female		Male
**Adjusted HR (95% CI)**	**P value**	**djusted HR (95% C**	**P value**	**Adjusted HR (95% CI)**
1.00 (0.97, 1.03)	9.05E-01	1.06 (1.03, 1.08)	**3.04E-04**	0.88 (0.86, 0.91)
1.00 (0.97, 1.02)	8.61E-01	1.05 (1.02, 1.08)	**4.88E-04**	0.88 (0.86, 0.91)
1.00 (0.97, 1.03)	9.99E-01	1.06 (1.03, 1.08)	**3.04E-04**	0.88 (0.86, 0.91)
1.00 (0.97, 1.02)	8.53E-01	1.05 (1.02, 1.08)	**8.02E-04**	0.87 (0.94, 0.90)
0.99 (0.97, 1.02)	7.69E-01	1.05 (1.02, 1.08)	**1.26E-03**	0.87 (0.94, 0.90)
1.00 (0.97, 1.03)	9.43E-01	1.05 (1.03, 1.08)	**5.38E-04**	0.88 (0.85, 0.91)
1.00 (0.97, 1.02)	8.36E-01	1.05 (1.02, 1.08)	**5.47E-04**	0.89 (0.86, 0.92)
0.97 (0.95, 1.00)	6.90E-02	1.04 (1.00, 1.07)	**4.71E-03**	0.82 (0.79, 0.85)
0.97 (0.94, 1.00)	5.57E-02	1.04 (1.00, 1.07)	**6.19E-03**	0.82 (0.79, 0.85)
0.98 (0.95, 1.00)	1.06E-01	1.04 (1.00, 1.07)	**5.11E-03**	0.83 (0.80, 0.85)
0.97 (0.94, 0.99)	**2.03E-02**	1.03 (0.99, 1.06)	**4.15E-02**	0.80 (0.78, 0.83)
0.96 (0.93, 0.98)	**3.71E-03**	1.02 (0.98, 1.05)	1.52E-01	0.79 (0.77, 0.82)
0.97 (0.95, 1.00)	8.67E-02	1.04 (1.00, 1.07)	**8.50E-03**	0.82 (0.80, 0.85)
0.97 (0.95, 1.00)	6.87E-02	1.04 (1.00, 1.07)	**3.99E-03**	0.83 (0.80, 0.86)
0.96 (0.94, 0.99)	**1.03E-02**	1.04 (0.99, 1.07)	**1.94E-02**	0.80 (0.78, 0.83)
0.95 (0.93, 0.98)	**1.72E-03**	1.03 (0.98, 1.06)	**4.90E-02**	0.79 (0.76, 0.82)
0.96 (0.94, 0.99)	**1.87E-02**	1.04 (0.99, 1.07)	**1.22E-02**	0.80 (0.78, 0.83)
0.96 (0.93, 0.98)	**4.54E-03**	1.03 (0.98, 1.06)	6.88E-02	0.79 (0.76, 0.81)
0.96 (0.93, 0.99)	**5.24E-03**	1.02 (0.99, 1.05)	1.47E-01	0.79 (0.77, 0.82)
0.96 (0.93, 0.99)	**5.25E-03**	1.03 (0.99, 1.06)	**3.40E-02**	0.79 (0.76, 0.82)
0.95 (0.92, 0.98)	**4.73E-04**	1.03 (0.98, 1.06)	7.82E-02	0.79 (0.76, 0.82)
0.94 (0.91, 0.97)	**2.80E-05**	1.00 (0.97, 1.03)	9.41E-01	0.79 (0.77, 0.82)
0.94 (0.91, 0.96)	**1.05E-05**	1.00 (0.96, 1.03)	8.68E-01	0.78 (0.76, 0.81)
0.94 (0.92, 0.97)	**6.14E-05**	1.00 (0.97, 1.03)	8.90E-01	0.80 (0.78, 0.83)
0.94 (0.92, 0.97)	**1.04E-04**	0.97 (0.97, 1.00)	9.20E-02	0.85 (0.82, 0.87)
0.95 (0.92, 0.97)	**2.95E-04**	0.96 (0.97, 0.99)	**1.64E-02**	0.88 (0.85, 0.91)
0.94 (0.92, 0.97)	**9.24E-05**	0.99 (0.97, 1.02)	5.54E-01	0.81 (0.79, 0.84)
0.94 (0.92, 0.97)	**9.51E-05**	1.02 (0.97, 1.05)	1.69E-01	0.78 (0.76, 0.81)
0.97 (0.94, 1.00)	**4.22E-02**	1.03 (1.00, 1.06)	**4.77E-02**	0.83 (0.80, 0.85)
0.97 (0.94, 0.99)	**2.28E-02**	1.03 (0.99, 1.06)	9.10E-02	0.82 (0.80, 0.85)
0.95 (0.93, 0.98)	**1.31E-03**	1.01 (0.98, 1.04)	7.14E-01	0.82 (0.79, 0.85)
0.96 (0.94, 0.99)	**1.39E-02**	1.01 (0.99, 1.04)	6.95E-01	0.84 (0.81, 0.86)
0.97 (0.95, 1.00)	7.61E-02	1.02 (1.00, 1.05)	3.36E-01	0.84 (0.82, 0.87)
0.95 (0.92, 0.97)	**2.41E-04**	0.99 (0.97, 1.02)	5.95E-01	0.83 (0.80, 0.85)
0.98 (0.95, 1.01)	2.23E-01	1.05 (1.01, 1.08)	**1.76E-03**	0.85 (0.82, 0.88)
1.03 (1.00, 1.05)	6.92E-02	1.03 (1.05, 1.06)	5.65E-02	0.98 (0.95, 1.01)
1.03 (1.00, 1.06)	**3.13E-02**	1.04 (1.06, 1.07)	**2.97E-02**	0.99 (0.96, 1.02)
1.04 (1.02, 1.07)	**2.97E-03**	1.05 (1.07, 1.08)	**2.94E-03**	1.00 (0.98, 1.03)
1.02 (0.99, 1.04)	2.63E-01	1.00 (1.04, 1.03)	7.79E-01	1.00 (0.97, 1.03)
1.01 (0.98, 1.04)	4.65E-01	0.99 (1.04, 1.02)	7.42E-01	1.00 (0.97, 1.03)

1.03 (1.00, 1.06)	**4.60E-02**	1.03 (1.06, 1.06)	6.61E-02	0.99 (0.96, 1.02)
1.04 (1.01, 1.06)	**1.55E-02**	1.08 (1.06, 1.11)	**1.91E-06**	0.97 (0.94, 1.00)
0.97 (0.94, 1.00)	**3.36E-02**	0.97 (1.00, 1.00)	5.65E-02	0.91 (0.89, 0.94)
0.96 (0.94, 0.99)	**1.39E-02**	0.96 (0.99, 0.99)	**1.15E-02**	0.94 (0.91, 0.97)
0.98 (0.95, 1.00)	1.06E-01	0.97 (1.00, 1.00)	9.03E-02	0.95 (0.92, 0.98)
0.95 (0.93, 0.98)	**1.00E-03**	0.95 (0.98, 0.97)	**4.95E-04**	0.93 (0.90, 0.96)
0.95 (0.92, 0.97)	**2.95E-04**	0.94 (0.97, 0.97)	**2.47E-04**	0.93 (0.90, 0.95)
0.97 (0.94, 0.99)	**2.07E-02**	0.96 (0.99, 0.98)	**4.17E-03**	0.95 (0.92, 0.98)
1.05 (1.02, 1.08)	**6.37E-04**	1.08 (1.08, 1.11)	**1.57E-06**	1.01 (0.98, 1.05)
0.94 (0.91, 0.96)	**8.10E-06**	0.97 (0.96, 1.00)	8.73E-02	0.84 (0.82, 0.87)
0.92 (0.90, 0.95)	**3.63E-08**	0.95 (0.95, 0.98)	**2.12E-03**	0.84 (0.82, 0.87)
0.92 (0.90, 0.95)	**6.72E-08**	0.95 (0.95, 0.98)	**9.29E-04**	0.84 (0.82, 0.87)
0.91 (0.89, 0.94)	**2.11E-09**	0.94 (0.94, 0.97)	**4.34E-04**	0.84 (0.81, 0.86)
0.91 (0.89, 0.94)	**9.31E-10**	0.95 (0.94, 0.98)	**1.03E-03**	0.83 (0.80, 0.85)
0.92 (0.90, 0.95)	**4.54E-08**	0.94 (0.95, 0.96)	**4.45E-05**	0.87 (0.84, 0.90)
1.03 (1.01, 1.06)	**2.18E-02**	1.07 (1.06, 1.10)	**9.89E-06**	0.99 (0.96, 1.02)
0.93 (0.91, 0.96)	**3.32E-06**	0.98 (0.96, 1.01)	2.45E-01	0.81 (0.78, 0.83)
0.91 (0.88, 0.94)	**2.29E-10**	0.96 (0.94, 0.99)	**2.24E-02**	0.78 (0.76, 0.81)
0.90 (0.88, 0.93)	**1.99E-11**	0.96 (0.93, 0.99)	**7.55E-03**	0.78 (0.76, 0.81)
0.91 (0.88, 0.93)	**4.79E-11**	0.96 (0.93, 0.99)	**9.95E-03**	0.78 (0.75, 0.80)
0.91 (0.89, 0.94)	**4.19E-10**	0.97 (0.94, 1.00)	**4.97E-02**	0.77 (0.75, 0.80)
0.90 (0.88, 0.93)	**2.46E-12**	0.94 (0.93, 0.96)	**3.99E-05**	0.81 (0.78, 0.83)
1.01 (0.98, 1.04)	5.97E-01	1.06 (1.04, 1.09)	**6.53E-05**	0.93 (0.90, 0.96)
0.94 (0.91, 0.97)	**2.28E-05**	0.99 (0.97, 1.02)	4.53E-01	0.83 (0.80, 0.86)
0.92 (0.89, 0.94)	**1.25E-08**	0.97 (0.94, 1.00)	**4.98E-02**	0.81 (0.78, 0.83)
0.93 (0.90, 0.95)	**2.86E-07**	0.97 (0.95, 1.00)	**4.90E-02**	0.84 (0.81, 0.86)
0.91 (0.89, 0.94)	**4.55E-10**	0.96 (0.94, 0.99)	**1.25E-02**	0.80 (0.77, 0.82)
0.92 (0.89, 0.94)	**7.62E-09**	0.97 (0.94, 1.00)	6.92E-02	0.79 (0.77, 0.82)
0.91 (0.88, 0.93)	**2.42E-11**	0.94 (0.93, 0.97)	**4.89E-05**	0.83 (0.80, 0.85)
1.00 (0.97, 1.03)	8.80E-01	1.06 (1.03, 1.09)	**1.88E-04**	0.90 (0.87, 0.93)
1.06 (1.03, 1.09)	**2.19E-05**	0.99 (1.09, 1.02)	6.52E-01	1.25 (1.22, 1.28)
1.07 (1.04, 1.10)	**5.59E-07**	0.99 (1.10, 1.02)	6.69E-01	1.28 (1.25, 1.30)
1.08 (1.05, 1.11)	**1.36E-08**	1.00 (1.11, 1.03)	8.43E-01	1.29 (1.26, 1.32)
1.05 (1.03, 1.08)	**1.22E-04**	0.98 (1.08, 1.01)	2.88E-01	1.26 (1.23, 1.29)
1.04 (1.02, 1.07)	**1.71E-03**	0.98 (1.07, 1.01)	1.50E-01	1.25 (1.22, 1.28)
1.08 (1.06, 1.11)	**1.52E-09**	1.01 (1.11, 1.04)	7.77E-01	1.27 (1.24, 1.30)
1.05 (1.02, 1.07)	**1.84E-03**	1.06 (1.07, 1.09)	**4.86E-05**	1.05 (1.02, 1.08)
1.03 (1.01, 1.06)	**2.08E-02**	0.97 (1.06, 1.00)	**4.32E-02**	1.25 (1.22, 1.28)
1.04 (1.01, 1.07)	**7.61E-03**	0.97 (1.07, 1.00)	5.37E-02	1.25 (1.22, 1.29)
1.04 (1.02, 1.07)	**3.71E-03**	0.97 (1.07, 1.00)	7.29E-02	1.26 (1.22, 1.29)
1.03 (1.00, 1.06)	**3.90E-02**	0.96 (1.06, 0.99)	**2.05E-02**	1.25 (1.22, 1.28)
1.03 (1.00, 1.05)	7.74E-02	0.96 (1.05, 0.99)	**1.25E-02**	1.24 (1.21, 1.28)
1.04 (1.02, 1.07)	**1.83E-03**	0.97 (1.07, 1.00)	9.03E-02	1.26 (1.23, 1.29)
1.07 (1.04, 1.10)	**1.46E-06**	1.05 (1.10, 1.08)	**1.61E-03**	1.12 (1.09, 1.15)
0.98 (0.95, 1.00)	9.39E-02	0.95 (1.00, 0.98)	**1.04E-03**	1.06 (1.03, 1.09)
0.98 (0.95, 1.01)	1.71E-01	0.95 (1.01, 0.98)	**4.32E-03**	1.05 (1.02, 1.08)
0.99 (0.96, 1.01)	3.73E-01	0.96 (1.01, 0.99)	**2.08E-02**	1.05 (1.02, 1.08)
0.97 (0.94, 0.99)	**2.78E-02**	0.94 (0.99, 0.97)	**8.28E-05**	1.07 (1.04, 1.10)

0.97 (0.94, 0.99)	**1.69E-02**	0.93 (0.99, 0.96)	**3.95E-05**	1.06 (1.03, 1.09)
0.98 (0.95, 1.01)	1.87E-01	0.95 (1.01, 0.98)	**1.69E-03**	1.08 (1.05, 1.11)
1.03 (1.01, 1.06)	**2.86E-02**	1.06 (1.06, 1.09)	**4.19E-04**	0.96 (0.93, 0.99)
0.89 (0.86, 0.91)	**7.73E-16**	0.93 (0.91, 0.96)	**4.57E-06**	0.78 (0.76, 0.81)
0.92 (0.90, 0.95)	**1.25E-08**	0.95 (0.95, 0.98)	**4.61E-03**	0.83 (0.80, 0.85)
0.94 (0.91, 0.96)	**1.26E-05**	0.96 (0.96, 0.99)	**2.37E-02**	0.86 (0.83, 0.89)
0.89 (0.86, 0.91)	**5.47E-16**	0.92 (0.91, 0.95)	**3.90E-07**	0.80 (0.77, 0.82)
0.88 (0.85, 0.90)	**2.13E-18**	0.91 (0.90, 0.94)	**2.28E-08**	0.78 (0.76, 0.81)
0.93 (0.90, 0.95)	**4.86E-07**	0.95 (0.95, 0.98)	**4.83E-03**	0.87 (0.85, 0.90)
1.00 (0.98, 1.03)	8.22E-01	1.06 (1.03, 1.09)	**1.31E-04**	0.87 (0.84, 0.90)
1.03 (1.01, 1.06)	**2.73E-02**	1.05 (1.06, 1.08)	**9.95E-03**	0.98 (0.96, 1.02)
1.01 (0.98, 1.03)	7.31E-01	1.00 (1.03, 1.03)	9.41E-01	1.04 (1.01, 1.08)
1.00 (0.97, 1.03)	8.87E-01	1.00 (1.03, 1.03)	9.80E-01	1.04 (1.00, 1.07)
1.01 (0.99, 1.04)	4.52E-01	1.01 (1.04, 1.04)	7.79E-01	1.05 (1.02, 1.08)
0.99 (0.96, 1.01)	3.68E-01	0.98 (1.01, 1.02)	3.76E-01	0.97 (0.94, 0.99)
1.00 (0.98, 1.03)	7.93E-01	0.97 (1.03, 1.00)	5.56E-02	1.11 (1.08, 1.15)
0.99 (0.97, 1.02)	7.33E-01	0.96 (1.02, 0.99)	**2.04E-02**	1.10 (1.07, 1.13)
1.04 (1.02, 1.07)	**2.96E-03**	1.00 (1.07, 1.03)	8.78E-01	1.15 (1.12, 1.18)
0.98 (0.96, 1.01)	2.85E-01	1.02 (1.01, 1.05)	3.76E-01	0.90 (0.88, 0.93)
1.06 (1.03, 1.09)	**4.84E-04**	1.08 (1.09, 1.12)	**2.55E-05**	0.92 (0.90, 0.95)
1.05 (1.02, 1.07)	**1.83E-03**	1.02 (1.07, 1.05)	3.51E-01	1.11 (1.08, 1.15)
1.04 (1.01, 1.07)	**5.70E-03**	1.00 (1.07, 1.03)	9.98E-01	1.14 (1.11, 1.17)
1.05 (1.02, 1.08)	**1.28E-03**	1.05 (1.08, 1.09)	**4.32E-03**	1.00 (0.98, 1.04)
0.94 (0.91, 0.97)	**2.19E-05**	0.95 (0.97, 0.98)	**2.08E-03**	0.96 (0.93, 0.98)
1.01 (0.98, 1.03)	6.22E-01	0.98 (1.03, 1.01)	1.82E-01	1.05 (1.03, 1.09)
0.99 (0.97, 1.02)	6.78E-01	0.95 (1.02, 0.98)	**1.21E-03**	1.09 (1.06, 1.12)
0.99 (0.96, 1.02)	5.02E-01	0.95 (1.02, 0.97)	**4.61E-04**	1.09 (1.06, 1.13)
1.00 (0.98, 1.03)	8.42E-01	0.96 (1.03, 0.99)	**1.89E-02**	1.07 (1.04, 1.10)
1.00 (0.98, 1.03)	8.36E-01	1.05 (1.03, 1.08)	**4.17E-03**	0.92 (0.90, 0.95)
0.95 (0.92, 0.97)	**1.84E-04**	0.92 (0.97, 0.95)	**9.79E-08**	0.98 (0.95, 1.01)
0.98 (0.96, 1.01)	2.85E-01	0.95 (1.01, 0.98)	**1.92E-03**	1.01 (0.98, 1.04)
1.01 (0.99, 1.04)	4.32E-01	0.98 (1.04, 1.01)	3.03E-01	1.03 (1.00, 1.06)
0.95 (0.92, 0.97)	**3.33E-04**	0.92 (0.97, 0.94)	**7.46E-08**	0.99 (0.96, 1.02)
1.03 (1.00, 1.06)	6.03E-02	1.06 (1.06, 1.09)	**1.25E-04**	1.00 (0.97, 1.02)
1.11 (1.08, 1.14)	**7.11E-13**	1.13 (1.14, 1.17)	**4.32E-15**	1.11 (1.08, 1.15)
0.97 (0.94, 0.99)	**2.39E-02**	0.92 (0.99, 0.94)	**1.15E-08**	1.02 (0.99, 1.05)
0.96 (0.94, 0.99)	**1.44E-02**	0.91 (0.99, 0.94)	**1.48E-09**	1.02 (0.99, 1.05)
0.99 (0.97, 1.02)	6.09E-01	0.97 (1.02, 1.00)	7.72E-02	1.00 (0.97, 1.03)
1.02 (0.99, 1.04)	3.01E-01	1.07 (1.04, 1.10)	**4.38E-06**	0.96 (0.93, 0.99)
1.10 (1.07, 1.13)	**3.10E-12**	1.10 (1.13, 1.13)	**1.11E-09**	1.12 (1.09, 1.15)
0.92 (0.89, 0.94)	**7.22E-11**	0.89 (0.94, 0.92)	**1.69E-15**	0.92 (0.90, 0.95)
0.90 (0.87, 0.92)	**1.61E-15**	0.88 (0.92, 0.91)	**4.48E-17**	0.89 (0.87, 0.92)
0.99 (0.97, 1.02)	6.21E-01	0.96 (1.02, 0.99)	**1.55E-02**	1.02 (0.99, 1.04)
1.07 (1.05, 1.10)	**1.16E-07**	1.11 (1.10, 1.14)	**1.82E-13**	1.06 (1.04, 1.09)
1.03 (1.01, 1.05)	**2.46E-02**	0.99 (1.05, 1.02)	3.99E-01	1.05 (1.02, 1.07)
0.93 (0.92, 0.95)	**5.27E-16**	0.93 (0.95, 0.95)	**1.09E-16**	0.92 (0.90, 0.93)
0.93 (0.92, 0.94)	**1.33E-20**	0.95 (0.94, 0.97)	**2.72E-06**	0.91 (0.90, 0.92)
0.97 (0.94, 0.99)	**1.72E-02**	0.92 (0.99, 0.95)	**9.40E-08**	1.03 (1.00, 1.06)

1.09 (1.06, 1.11)	**6.60E-14**	1.09 (1.11, 1.11)	**3.35E-18**	1.11 (1.09, 1.13)
0.99 (0.96, 1.02)	4.51E-01	0.95 (1.02, 0.98)	**4.75E-03**	1.06 (1.03, 1.09)
0.93 (0.92, 0.95)	**8.33E-13**	0.94 (0.95, 0.96)	**6.55E-09**	0.91 (0.90, 0.92)
0.95 (0.93, 0.97)	**7.65E-05**	1.01 (0.97, 1.04)	4.78E-01	0.86 (0.85, 0.87)
0.96 (0.94, 0.99)	**6.17E-03**	0.91 (0.99, 0.94)	**7.11E-09**	1.04 (1.01, 1.07)
1.09 (1.07, 1.12)	**5.10E-16**	1.08 (1.12, 1.09)	**6.84E-17**	1.13 (1.11, 1.15)
1.06 (1.04, 1.09)	**1.07E-05**	1.02 (1.09, 1.05)	3.30E-01	1.17 (1.15, 1.19)
0.91 (0.89, 0.93)	**1.61E-15**	0.92 (0.93, 0.94)	**1.43E-10**	0.87 (0.86, 0.89)
0.95 (0.92, 0.97)	**2.11E-04**	0.99 (0.97, 1.02)	5.88E-01	0.85 (0.83, 0.86)
0.95 (0.92, 0.97)	**6.12E-05**	0.92 (0.97, 0.95)	**1.01E-08**	0.98 (0.95, 1.00)
1.06 (1.03, 1.09)	**1.04E-05**	1.08 (1.09, 1.11)	**1.02E-09**	1.05 (1.02, 1.08)
1.09 (1.06, 1.13)	**1.60E-08**	1.00 (1.13, 1.03)	7.79E-01	1.31 (1.26, 1.36)
0.91 (0.88, 0.93)	**1.14E-10**	0.95 (0.93, 0.98)	**4.64E-03**	0.81 (0.78, 0.83)
0.92 (0.90, 0.94)	**8.99E-11**	0.92 (0.94, 0.95)	**6.78E-09**	0.91 (0.89, 0.93)
0.97 (0.94, 0.99)	**2.96E-02**	1.03 (0.99, 1.06)	**4.62E-02**	0.83 (0.80, 0.86)
0.98 (0.96, 1.01)	1.98E-01	1.04 (1.01, 1.06)	**3.86E-03**	0.89 (0.86, 0.93)
1.01 (0.99, 1.04)	4.35E-01	1.05 (1.04, 1.08)	**2.43E-03**	0.92 (0.89, 0.94)
0.99 (0.96, 1.01)	3.64E-01	0.94 (1.01, 0.97)	**3.59E-05**	1.11 (1.08, 1.14)
0.97 (0.95, 1.00)	6.20E-02	0.94 (1.00, 0.96)	**4.33E-06**	1.09 (1.05, 1.12)
1.05 (1.02, 1.07)	**2.75E-03**	0.99 (1.07, 1.02)	5.20E-01	1.16 (1.13, 1.20)
1.01 (0.99, 1.04)	4.32E-01	1.06 (1.04, 1.08)	**2.34E-05**	0.91 (0.88, 0.94)
1.08 (1.05, 1.10)	**3.93E-07**	1.10 (1.10, 1.13)	**1.44E-09**	0.97 (0.94, 1.00)
0.93 (0.91, 0.96)	**2.66E-06**	0.91 (0.96, 0.94)	**1.51E-09**	1.05 (1.02, 1.08)
0.93 (0.91, 0.96)	**2.81E-06**	0.91 (0.96, 0.94)	**3.27E-09**	1.03 (1.00, 1.06)
0.97 (0.94, 0.99)	**2.73E-02**	0.94 (0.99, 0.97)	**1.12E-04**	1.10 (1.07, 1.13)
1.06 (1.03, 1.09)	**3.26E-05**	1.09 (1.09, 1.12)	**1.47E-08**	0.94 (0.91, 0.97)
1.12 (1.09, 1.15)	**4.51E-15**	1.06 (1.15, 1.09)	**2.47E-04**	1.25 (1.21, 1.28)
0.90 (0.87, 0.92)	**2.35E-14**	0.90 (0.92, 0.93)	**9.82E-12**	0.90 (0.87, 0.93)
0.89 (0.87, 0.91)	**5.10E-16**	0.90 (0.91, 0.93)	**7.71E-12**	0.86 (0.84, 0.89)
1.03 (1.00, 1.05)	7.52E-02	1.00 (1.05, 1.03)	9.41E-01	1.16 (1.13, 1.19)
1.06 (1.03, 1.09)	**4.17E-05**	1.10 (1.09, 1.13)	**8.95E-10**	0.95 (0.93, 0.98)
0.94 (0.92, 0.97)	**3.96E-05**	1.02 (0.97, 1.05)	1.71E-01	0.78 (0.76, 0.80)
0.99 (0.96, 1.01)	3.39E-01	0.96 (1.01, 0.99)	**8.00E-03**	1.03 (1.00, 1.06)
1.07 (1.04, 1.10)	**7.90E-07**	0.99 (1.10, 1.02)	6.23E-01	1.33 (1.30, 1.36)
0.93 (0.91, 0.96)	**2.62E-06**	0.95 (0.96, 0.97)	**6.38E-04**	0.89 (0.86, 0.92)
0.97 (0.94, 0.99)	**2.97E-02**	1.01 (0.99, 1.04)	4.39E-01	0.84 (0.81, 0.86)
0.91 (0.89, 0.94)	**4.19E-10**	0.95 (0.94, 0.98)	**1.30E-03**	0.81 (0.79, 0.84)
0.96 (0.93, 0.98)	**3.22E-03**	0.98 (0.98, 1.01)	2.10E-01	0.87 (0.84, 0.90)
0.93 (0.91, 0.96)	**9.62E-07**	0.94 (0.96, 0.97)	**1.83E-04**	0.89 (0.87, 0.92)
0.94 (0.92, 0.97)	**3.50E-05**	0.96 (0.97, 0.99)	**1.17E-02**	0.88 (0.86, 0.91)
0.98 (0.95, 1.01)	2.22E-01	1.05 (1.01, 1.08)	**1.95E-03**	0.85 (0.82, 0.88)
0.97 (0.94, 1.00)	**4.57E-02**	1.04 (1.00, 1.07)	**7.34E-03**	0.83 (0.80, 0.85)
1.02 (1.00, 1.05)	1.25E-01	1.07 (1.05, 1.10)	**1.90E-05**	0.96 (0.93, 0.99)
1.03 (1.01, 1.06)	**2.36E-02**	1.06 (1.06, 1.09)	**1.08E-04**	0.97 (0.94, 1.00)
0.97 (0.94, 1.00)	**3.12E-02**	0.97 (1.00, 1.00)	9.58E-02	0.96 (0.94, 0.99)
1.00 (0.97, 1.03)	9.26E-01	1.07 (1.03, 1.10)	**1.82E-05**	0.85 (0.82, 0.88)

0.96 (0.94, 0.99)	**8.90E-03**	0.96 (0.99, 0.99)	**2.47E-02**	0.97 (0.94, 1.00)
0.98 (0.96, 1.01)	2.15E-01	0.97 (1.01, 1.00)	7.82E-02	1.00 (0.97, 1.03)
0.96 (0.94, 0.99)	**1.44E-02**	0.97 (0.99, 0.99)	**3.40E-02**	0.96 (0.94, 0.99)
0.97 (0.95, 1.00)	8.04E-02	0.95 (1.00, 0.98)	**1.26E-03**	1.06 (1.03, 1.09)
0.94 (0.92, 0.97)	**7.65E-05**	0.98 (0.97, 1.01)	2.14E-01	0.84 (0.82, 0.87)
0.97 (0.94, 1.00)	5.55E-02	1.01 (1.00, 1.04)	5.74E-01	0.84 (0.82, 0.87)
0.97 (0.95, 1.00)	6.67E-02	1.02 (1.00, 1.05)	2.47E-01	0.89 (0.86, 0.91)
0.88 (0.85, 0.90)	**5.89E-19**	0.89 (0.90, 0.92)	**5.07E-13**	0.88 (0.85, 0.90)
0.88 (0.86, 0.91)	**1.06E-15**	0.92 (0.91, 0.95)	**1.98E-06**	0.87 (0.84, 0.89)
0.94 (0.91, 0.96)	**9.65E-06**	0.97 (0.96, 1.00)	**4.50E-02**	0.86 (0.83, 0.88)
0.88 (0.86, 0.91)	**1.90E-15**	0.94 (0.91, 0.97)	**5.34E-04**	0.81 (0.78, 0.84)
0.93 (0.91, 0.96)	**2.81E-06**	0.97 (0.96, 1.00)	**4.90E-02**	0.85 (0.83, 0.88)
0.91 (0.89, 0.94)	**6.00E-10**	0.96 (0.94, 0.99)	**7.78E-03**	0.83 (0.80, 0.85)
1.01 (0.98, 1.03)	7.50E-01	1.06 (1.03, 1.09)	**4.64E-04**	0.92 (0.89, 0.95)
1.00 (0.97, 1.02)	8.68E-01	1.04 (1.02, 1.07)	**2.56E-02**	0.93 (0.90, 0.95)
0.89 (0.86, 0.92)	**4.01E-14**	0.91 (0.92, 0.94)	**9.79E-08**	0.93 (0.90, 0.96)
0.94 (0.91, 0.97)	**2.98E-05**	0.91 (0.97, 0.94)	**2.51E-08**	0.96 (0.93, 0.99)
0.87 (0.85, 0.90)	**1.53E-18**	0.93 (0.90, 0.95)	**6.18E-06**	0.83 (0.81, 0.86)
0.95 (0.93, 0.98)	**1.49E-03**	0.92 (0.98, 0.94)	**3.44E-08**	1.02 (0.99, 1.05)
0.91 (0.89, 0.94)	**8.50E-11**	0.89 (0.94, 0.92)	**1.21E-13**	0.96 (0.93, 0.99)
1.08 (1.05, 1.11)	**1.58E-07**	1.12 (1.11, 1.15)	**4.81E-12**	0.99 (0.96, 1.02)
1.08 (1.05, 1.11)	**5.09E-08**	1.07 (1.11, 1.10)	**8.35E-06**	1.10 (1.07, 1.13)
1.07 (1.05, 1.09)	**1.60E-08**	1.04 (1.09, 1.07)	**1.95E-03**	1.11 (1.09, 1.14)
1.02 (0.99, 1.05)	2.35E-01	1.04 (1.05, 1.07)	**2.18E-02**	1.03 (1.00, 1.06)
1.06 (1.04, 1.09)	**1.69E-05**	1.02 (1.09, 1.05)	2.15E-01	1.14 (1.10, 1.17)
0.98 (0.95, 1.00)	1.08E-01	0.98 (1.00, 1.01)	3.22E-01	0.96 (0.93, 0.99)
0.94 (0.91, 0.96)	**1.03E-05**	0.96 (0.96, 0.99)	**1.15E-02**	0.96 (0.93, 0.99)
0.94 (0.91, 0.96)	**1.74E-05**	0.97 (0.96, 1.00)	7.30E-02	0.87 (0.84, 0.90)
0.98 (0.95, 1.00)	9.56E-02	1.02 (1.00, 1.05)	2.46E-01	0.92 (0.89, 0.95)
0.94 (0.92, 0.97)	**7.44E-05**	1.00 (0.97, 1.03)	9.62E-01	0.84 (0.81, 0.87)
0.94 (0.92, 0.97)	**1.15E-04**	1.01 (0.97, 1.04)	7.26E-01	0.83 (0.80, 0.85)
1.03 (1.00, 1.05)	**3.36E-02**	1.04 (1.05, 1.07)	**1.17E-02**	1.04 (1.02, 1.06)
1.01 (0.99, 1.04)	3.22E-01	1.03 (1.04, 1.06)	1.06E-01	1.04 (1.01, 1.07)
1.05 (1.03, 1.07)	**5.68E-07**	1.03 (1.07, 1.04)	**1.28E-02**	1.12 (1.10, 1.13)
1.05 (1.03, 1.07)	**2.06E-06**	1.04 (1.07, 1.06)	**1.78E-04**	1.07 (1.06, 1.09)
1.07 (1.04, 1.09)	**5.80E-09**	1.03 (1.09, 1.06)	**3.78E-02**	1.10 (1.08, 1.12)
1.02 (1.00, 1.04)	5.55E-02	1.03 (1.04, 1.05)	**8.00E-03**	1.04 (1.02, 1.05)
0.88 (0.86, 0.91)	**2.24E-17**	0.92 (0.91, 0.95)	**1.18E-06**	0.79 (0.77, 0.82)
1.14 (1.11, 1.17)	**3.05E-19**	1.16 (1.17, 1.19)	**3.22E-19**	1.03 (1.00, 1.06)
1.00 (0.97, 1.03)	9.12E-01	0.97 (1.03, 1.00)	5.95E-02	1.08 (1.05, 1.12)
0.95 (0.92, 0.98)	**4.51E-04**	1.01 (0.98, 1.04)	6.59E-01	0.85 (0.82, 0.87)
0.93 (0.91, 0.96)	**2.09E-06**	0.96 (0.96, 0.99)	**2.13E-02**	0.84 (0.81, 0.87)
0.92 (0.89, 0.94)	**9.26E-09**	0.95 (0.94, 0.97)	**5.57E-04**	0.83 (0.81, 0.86)
0.91 (0.89, 0.94)	**4.55E-10**	0.96 (0.94, 0.98)	**4.64E-03**	0.81 (0.78, 0.83)
0.97 (0.95, 1.00)	7.00E-02	0.93 (1.00, 0.96)	**3.68E-05**	1.12 (1.09, 1.15)
0.91 (0.88, 0.93)	**1.62E-10**	0.89 (0.93, 0.92)	**7.75E-13**	0.99 (0.96, 1.02)
1.09 (1.07, 1.11)	**8.09E-18**	1.05 (1.11, 1.08)	**3.38E-04**	1.12 (1.10, 1.14)
0.97 (0.94, 0.99)	**2.08E-02**	1.02 (0.99, 1.05)	1.71E-01	0.82 (0.80, 0.85)

0.91 (0.89, 0.94)	**1.37E-09**	0.94 (0.94, 0.96)	**3.68E-05**	0.85 (0.82, 0.87)
1.00 (0.98, 1.03)	8.68E-01	0.96 (1.03, 0.99)	**1.10E-02**	1.16 (1.13, 1.19)
1.02 (1.00, 1.05)	1.15E-01	1.03 (1.05, 1.05)	6.61E-02	1.05 (1.03, 1.07)
1.05 (1.02, 1.07)	**2.13E-04**	1.03 (1.07, 1.06)	**1.37E-02**	1.12 (1.10, 1.14)
0.93 (0.91, 0.96)	**8.59E-07**	0.93 (0.96, 0.96)	**5.30E-06**	0.94 (0.91, 0.97)
0.98 (0.95, 1.00)	1.06E-01	1.03 (1.00, 1.06)	9.20E-02	0.85 (0.82, 0.87)
0.93 (0.91, 0.96)	**4.18E-06**	0.98 (0.96, 1.01)	1.40E-01	0.83 (0.80, 0.86)
0.94 (0.91, 0.96)	**7.00E-06**	0.93 (0.96, 0.96)	**6.81E-06**	0.96 (0.93, 0.99)
0.96 (0.94, 0.99)	**7.48E-03**	0.97 (0.99, 1.00)	**4.97E-02**	0.94 (0.91, 0.97)
0.97 (0.95, 1.00)	5.94E-02	1.03 (1.00, 1.06)	**4.90E-02**	0.83 (0.80, 0.86)
0.92 (0.89, 0.94)	**4.71E-09**	0.95 (0.94, 0.98)	**2.77E-03**	0.82 (0.80, 0.85)
1.00 (0.97, 1.03)	9.31E-01	0.96 (1.03, 0.99)	**2.02E-02**	1.12 (1.09, 1.15)
0.95 (0.92, 0.97)	**1.44E-04**	0.98 (0.97, 1.01)	1.55E-01	0.87 (0.84, 0.90)
0.97 (0.94, 1.00)	**3.13E-02**	1.03 (1.00, 1.06)	**3.40E-02**	0.82 (0.79, 0.85)
0.92 (0.89, 0.94)	**5.62E-09**	0.96 (0.94, 0.98)	**5.50E-03**	0.82 (0.79, 0.85)
0.99 (0.97, 1.02)	6.95E-01	0.95 (1.02, 0.98)	**5.05E-03**	1.12 (1.09, 1.15)
0.97 (0.94, 1.00)	**4.60E-02**	1.01 (1.00, 1.04)	7.33E-01	0.86 (0.83, 0.88)
0.98 (0.95, 1.01)	1.70E-01	0.94 (1.01, 0.97)	**1.42E-04**	1.13 (1.10, 1.16)
1.04 (1.01, 1.07)	**9.86E-03**	1.06 (1.07, 1.09)	**4.66E-03**	0.99 (0.96, 1.02)

Other mortality		
	Female	
P value	Adjusted HR (95% CI)	P value
**6.58E-14**	0.99 (0.96, 1.03)	6.63E-01
**1.09E-13**	0.99 (0.96, 1.02)	5.96E-01
**1.32E-13**	0.99 (0.96, 1.03)	7.66E-01
**1.94E-16**	0.98 (0.95, 1.02)	3.43E-01
**1.12E-17**	0.98 (0.94, 1.01)	2.55E-01
**1.27E-14**	0.99 (0.95, 1.02)	4.76E-01
**1.32E-12**	0.99 (0.96, 1.03)	6.57E-01
**6.97E-32**	0.95 (0.91, 0.98)	**5.80E-03**
**7.10E-32**	0.94 (0.91, 0.98)	**3.30E-03**
**1.28E-30**	0.94 (0.91, 0.98)	**3.95E-03**
**6.26E-40**	0.92 (0.88, 0.95)	**9.81E-06**
**2.10E-45**	0.90 (0.86, 0.93)	**7.73E-08**
**2.96E-32**	0.94 (0.90, 0.97)	**1.03E-03**
**2.17E-28**	0.95 (0.92, 0.99)	**1.92E-02**
**6.37E-40**	0.92 (0.89, 0.96)	**8.15E-05**
**7.57E-45**	0.91 (0.88, 0.95)	**3.87E-06**
**1.56E-39**	0.93 (0.90, 0.97)	**2.68E-04**
**1.18E-46**	0.90 (0.87, 0.94)	**3.64E-07**
**4.16E-45**	0.89 (0.86, 0.93)	**1.03E-08**
**2.10E-45**	0.92 (0.88, 0.95)	**1.51E-05**
**1.50E-43**	0.91 (0.88, 0.95)	**3.82E-06**
**8.30E-45**	0.86 (0.83, 0.90)	**3.67E-13**
**3.79E-50**	0.86 (0.83, 0.90)	**5.38E-13**
**6.86E-41**	0.87 (0.83, 0.90)	**5.38E-13**
**4.12E-25**	0.86 (0.83, 0.89)	**5.04E-15**
**1.75E-15**	0.86 (0.83, 0.89)	**9.40E-15**
**1.20E-36**	0.86 (0.83, 0.90)	**9.82E-14**
**5.79E-49**	0.90 (0.86, 0.93)	**5.23E-08**
**2.19E-31**	0.92 (0.89, 0.96)	**4.16E-05**
**5.32E-33**	0.91 (0.88, 0.95)	**3.70E-06**
**9.46E-35**	0.89 (0.85, 0.92)	**1.28E-09**
**3.41E-28**	0.89 (0.86, 0.93)	**1.77E-08**
**1.33E-25**	0.91 (0.87, 0.94)	**1.36E-06**
**8.36E-32**	0.87 (0.84, 0.91)	**4.20E-12**
**7.81E-24**	0.96 (0.92, 0.99)	**3.13E-02**
2.37E-01	0.96 (0.92, 1.00)	**3.42E-02**
5.85E-01	0.96 (0.93, 1.00)	6.18E-02
7.63E-01	0.99 (0.95, 1.02)	4.66E-01
8.61E-01	0.93 (0.90, 0.96)	**1.77E-04**
9.93E-01	0.92 (0.89, 0.95)	**1.39E-05**

5.77E-01	0.96 (0.92, 0.99)	**2.67E-02**
**3.24E-02**	1.03 (1.00, 1.07)	9.04E-02
**9.22E-09**	0.89 (0.85, 0.92)	**3.39E-10**
**6.25E-05**	0.89 (0.85, 0.92)	**2.96E-10**
**1.88E-03**	0.90 (0.87, 0.93)	**2.14E-08**
**5.31E-06**	0.87 (0.84, 0.91)	**1.19E-12**
**7.70E-07**	0.87 (0.84, 0.90)	**1.97E-13**
**8.01E-04**	0.89 (0.86, 0.92)	**4.15E-10**
3.69E-01	1.05 (1.02, 1.09)	**7.49E-03**
**1.04E-25**	0.87 (0.84, 0.91)	**2.36E-12**
**7.80E-27**	0.85 (0.82, 0.88)	**4.52E-16**
**1.36E-26**	0.85 (0.82, 0.88)	**3.24E-17**
**9.02E-29**	0.84 (0.81, 0.88)	**7.80E-18**
**1.78E-32**	0.84 (0.81, 0.87)	**5.51E-18**
**1.18E-18**	0.85 (0.82, 0.88)	**1.63E-16**
6.23E-01	1.05 (1.01, 1.09)	**8.44E-03**
**2.79E-39**	0.85 (0.82, 0.89)	**2.67E-15**
**2.90E-51**	0.84 (0.81, 0.88)	**1.12E-17**
**3.20E-52**	0.84 (0.81, 0.88)	**1.15E-17**
**2.59E-53**	0.83 (0.80, 0.87)	**1.21E-19**
**1.72E-55**	0.84 (0.81, 0.87)	**1.35E-18**
**1.09E-40**	0.83 (0.80, 0.87)	**3.58E-20**
**1.79E-05**	1.03 (0.99, 1.06)	1.88E-01
**1.05E-30**	0.87 (0.84, 0.91)	**6.12E-12**
**1.08E-40**	0.86 (0.83, 0.89)	**9.28E-15**
**3.43E-29**	0.88 (0.85, 0.91)	**1.19E-11**
**2.13E-45**	0.84 (0.81, 0.88)	**1.28E-17**
**2.03E-46**	0.85 (0.82, 0.88)	**2.91E-16**
**2.29E-33**	0.85 (0.82, 0.88)	**5.15E-18**
**7.15E-11**	1.00 (0.96, 1.03)	9.49E-01
**1.10E-83**	1.06 (1.02, 1.10)	**3.22E-03**
**6.47E-101**	1.07 (1.03, 1.11)	**4.94E-04**
**3.02E-111**	1.08 (1.04, 1.12)	**7.14E-05**
**5.39E-85**	1.06 (1.02, 1.10)	**7.28E-03**
**6.41E-79**	1.05 (1.01, 1.09)	**2.36E-02**
**6.84E-98**	1.08 (1.04, 1.12)	**3.18E-05**
**3.22E-03**	1.05 (1.01, 1.09)	**1.22E-02**
**7.82E-68**	1.04 (1.00, 1.08)	9.04E-02
**1.04E-71**	1.04 (1.00, 1.08)	**4.39E-02**
**1.76E-70**	1.05 (1.01, 1.09)	**3.08E-02**
**8.81E-69**	1.04 (1.00, 1.08)	9.27E-02
**1.41E-65**	1.03 (0.99, 1.07)	1.26E-01
**2.33E-78**	1.05 (1.01, 1.09)	**2.84E-02**
**7.60E-15**	1.06 (1.02, 1.10)	**1.80E-03**
**1.36E-04**	0.96 (0.92, 0.99)	**2.81E-02**
**6.71E-04**	0.96 (0.92, 1.00)	**4.39E-02**
**2.41E-03**	0.96 (0.93, 1.00)	7.49E-02
**1.86E-05**	0.95 (0.92, 0.99)	**2.19E-02**

**6.12E-05**	0.95 (0.92, 0.99)	**1.73E-02**
**1.41E-07**	0.96 (0.92, 1.00)	6.08E-02
**1.31E-02**	1.01 (0.97, 1.05)	6.90E-01
**1.84E-53**	0.83 (0.80, 0.86)	**2.57E-19**
**1.49E-33**	0.87 (0.84, 0.90)	**4.85E-12**
**6.55E-22**	0.89 (0.85, 0.92)	**4.89E-09**
**1.57E-45**	0.83 (0.80, 0.87)	**9.16E-19**
**1.29E-53**	0.83 (0.80, 0.86)	**8.47E-21**
**5.72E-18**	0.88 (0.85, 0.92)	**1.77E-09**
**1.80E-17**	0.99 (0.96, 1.03)	7.45E-01
3.44E-01	1.07 (1.02, 1.12)	**7.49E-03**
**4.12E-03**	1.03 (0.99, 1.07)	1.84E-01
**2.76E-02**	1.02 (0.98, 1.07)	2.99E-01
**4.36E-04**	1.03 (0.99, 1.07)	1.27E-01
**2.04E-02**	0.96 (0.92, 1.00)	**4.09E-02**
**2.50E-14**	1.01 (0.97, 1.06)	5.22E-01
**2.18E-10**	1.00 (0.96, 1.05)	8.64E-01
**1.55E-26**	1.05 (1.01, 1.09)	**1.99E-02**
**1.99E-12**	0.98 (0.94, 1.02)	4.65E-01
**9.34E-10**	1.00 (0.96, 1.05)	8.53E-01
**7.64E-14**	1.05 (1.01, 1.09)	**2.23E-02**
**4.85E-21**	1.03 (1.00, 1.07)	9.04E-02
7.62E-01	1.07 (1.03, 1.11)	**2.76E-03**
**3.49E-03**	0.96 (0.92, 1.00)	8.25E-02
**3.35E-04**	0.97 (0.93, 1.00)	7.97E-02
**2.24E-08**	0.96 (0.93, 0.99)	**2.79E-02**
**2.81E-09**	0.96 (0.92, 0.99)	**2.08E-02**
**8.96E-06**	0.97 (0.93, 1.00)	7.37E-02
**1.71E-07**	1.04 (1.01, 1.08)	**3.24E-02**
3.15E-01	0.90 (0.87, 0.93)	**5.53E-08**
3.91E-01	0.93 (0.90, 0.97)	**3.86E-04**
**4.75E-02**	0.97 (0.93, 1.00)	9.79E-02
5.19E-01	0.90 (0.86, 0.93)	**3.12E-08**
7.60E-01	1.08 (1.05, 1.12)	**2.68E-05**
**3.91E-12**	1.21 (1.16, 1.25)	**2.18E-21**
2.15E-01	0.89 (0.86, 0.92)	**8.71E-12**
1.66E-01	0.88 (0.85, 0.91)	**1.83E-12**
9.12E-01	0.94 (0.91, 0.97)	**5.12E-04**
**5.25E-03**	1.10 (1.07, 1.14)	**2.87E-08**
**5.45E-14**	1.14 (1.09, 1.18)	**5.89E-11**
**2.36E-09**	0.85 (0.82, 0.88)	**3.32E-23**
**7.26E-16**	0.83 (0.80, 0.86)	**5.26E-27**
3.11E-01	0.95 (0.92, 0.98)	**5.07E-03**
**8.07E-06**	1.16 (1.13, 1.20)	**3.45E-22**
**1.06E-04**	1.00 (0.96, 1.04)	9.49E-01
**4.03E-30**	0.92 (0.90, 0.93)	**1.83E-41**
**8.45E-73**	0.91 (0.90, 0.92)	**1.56E-38**
**3.94E-02**	0.92 (0.89, 0.95)	**7.52E-07**

**4.23E-26**	1.12 (1.10, 1.14)	**1.56E-46**
**3.14E-06**	1.04 (1.00, 1.08)	7.97E-02
**6.99E-47**	0.91 (0.90, 0.92)	**1.86E-43**
**3.55E-82**	0.89 (0.86, 0.91)	**1.09E-16**
**1.03E-02**	0.93 (0.90, 0.97)	**2.53E-04**
**3.97E-43**	1.09 (1.08, 1.11)	**1.64E-43**
**1.18E-64**	1.13 (1.09, 1.17)	**4.07E-10**
**8.61E-63**	0.89 (0.87, 0.90)	**1.86E-42**
**1.56E-55**	0.89 (0.87, 0.92)	**5.78E-11**
9.53E-02	0.91 (0.88, 0.94)	**4.24E-08**
**6.40E-04**	1.10 (1.08, 1.13)	**2.25E-14**
**1.81E-43**	1.08 (1.04, 1.12)	**2.54E-04**
**1.17E-40**	0.89 (0.86, 0.92)	**2.94E-09**
**4.15E-12**	0.90 (0.87, 0.92)	**1.65E-12**
**7.90E-27**	0.98 (0.95, 1.02)	4.23E-01
**3.80E-10**	1.01 (0.98, 1.04)	4.11E-01
**1.67E-09**	1.04 (1.00, 1.07)	**4.40E-02**
**4.31E-11**	0.97 (0.93, 1.00)	**4.10E-02**
**1.14E-07**	0.96 (0.93, 0.99)	**9.88E-03**
**5.27E-20**	1.02 (0.98, 1.05)	3.47E-01
**2.60E-08**	1.03 (0.99, 1.06)	1.18E-01
5.12E-02	1.08 (1.04, 1.12)	**6.46E-05**
**1.88E-03**	0.94 (0.91, 0.97)	**9.05E-04**
**4.40E-02**	0.94 (0.91, 0.98)	**1.60E-03**
**1.09E-09**	0.95 (0.92, 0.99)	**1.25E-02**
**1.93E-04**	1.05 (1.01, 1.09)	**8.15E-03**
**6.44E-48**	1.13 (1.09, 1.17)	**1.28E-09**
**1.42E-12**	0.88 (0.85, 0.92)	**8.51E-11**
**2.51E-23**	0.87 (0.84, 0.90)	**1.71E-13**
**1.86E-30**	1.06 (1.02, 1.10)	**4.62E-03**
**3.20E-03**	1.07 (1.03, 1.11)	**3.14E-04**
**1.28E-65**	0.91 (0.88, 0.95)	**2.13E-06**
**4.12E-02**	0.98 (0.95, 1.02)	3.52E-01
**6.08E-109**	1.09 (1.05, 1.13)	**3.14E-05**
**8.74E-14**	0.86 (0.83, 0.89)	**3.39E-15**
**8.71E-28**	0.89 (0.86, 0.93)	**1.77E-08**
**5.02E-37**	0.84 (0.81, 0.87)	**1.11E-18**
**3.28E-18**	0.87 (0.84, 0.91)	**4.19E-12**
**5.17E-13**	0.86 (0.82, 0.89)	**2.34E-15**
**2.39E-15**	0.86 (0.83, 0.89)	**3.05E-14**
**1.16E-22**	0.96 (0.93, 1.00)	**3.78E-02**
**7.03E-30**	0.94 (0.91, 0.98)	**3.45E-03**
**2.04E-02**	1.04 (1.00, 1.08)	**4.85E-02**
**4.40E-02**	1.02 (0.98, 1.06)	3.43E-01
**1.62E-02**	0.91 (0.87, 0.94)	**2.24E-06**
**1.12E-24**	1.00 (0.97, 1.04)	8.64E-01

**4.53E-02**	0.90 (0.86, 0.93)	**1.05E-07**
8.61E-01	0.94 (0.90, 0.97)	**1.58E-03**
**1.59E-02**	0.90 (0.86, 0.93)	**1.16E-07**
**7.63E-05**	0.95 (0.92, 0.99)	**2.38E-02**
**7.85E-27**	0.87 (0.84, 0.90)	**1.50E-12**
**4.68E-26**	0.92 (0.89, 0.96)	**5.24E-05**
**6.46E-14**	0.93 (0.89, 0.97)	**2.70E-04**
**3.95E-17**	0.82 (0.79, 0.85)	**5.27E-24**
**3.10E-17**	0.86 (0.83, 0.90)	**2.33E-12**
**7.96E-22**	0.85 (0.82, 0.88)	**3.67E-16**
**1.82E-33**	0.85 (0.82, 0.89)	**4.12E-13**
**3.46E-23**	0.85 (0.81, 0.88)	**1.16E-16**
**6.94E-32**	0.83 (0.80, 0.86)	**1.06E-19**
**1.25E-07**	1.00 (0.96, 1.04)	9.77E-01
**1.34E-06**	0.97 (0.93, 1.01)	1.14E-01
**1.03E-05**	0.90 (0.86, 0.93)	**1.39E-07**
**4.53E-03**	0.86 (0.83, 0.89)	**7.76E-15**
**1.98E-26**	0.86 (0.83, 0.90)	**1.28E-11**
3.02E-01	0.90 (0.87, 0.93)	**1.89E-08**
**3.97E-03**	0.86 (0.83, 0.89)	**5.14E-17**
5.88E-01	1.14 (1.10, 1.19)	**2.33E-12**
**8.69E-10**	1.13 (1.09, 1.17)	**2.06E-10**
**2.45E-25**	1.09 (1.06, 1.12)	**1.07E-10**
5.99E-02	1.10 (1.06, 1.14)	**5.54E-07**
**6.16E-18**	1.05 (1.02, 1.08)	**2.30E-03**
**7.20E-03**	1.00 (0.97, 1.04)	9.13E-01
**4.30E-03**	0.94 (0.91, 0.98)	**4.91E-03**
**1.91E-17**	0.88 (0.84, 0.91)	**3.02E-09**
**4.88E-07**	0.92 (0.89, 0.96)	**8.22E-05**
**3.81E-26**	0.86 (0.83, 0.90)	**1.65E-12**
**2.73E-31**	0.86 (0.83, 0.90)	**6.24E-13**
**1.45E-03**	1.05 (1.01, 1.08)	**1.36E-02**
**1.12E-02**	1.01 (0.97, 1.05)	6.23E-01
**1.74E-95**	1.05 (1.04, 1.07)	**2.96E-10**
**4.53E-20**	1.06 (1.05, 1.08)	**1.25E-11**
**1.28E-28**	1.09 (1.06, 1.11)	**8.22E-14**
**4.52E-06**	1.07 (1.05, 1.08)	**1.87E-24**
**5.75E-52**	0.79 (0.76, 0.82)	**1.08E-31**
6.87E-02	1.19 (1.14, 1.23)	**2.75E-18**
**1.42E-07**	0.97 (0.93, 1.01)	1.38E-01
**2.46E-24**	0.87 (0.83, 0.90)	**1.45E-11**
**4.46E-28**	0.85 (0.82, 0.89)	**8.69E-16**
**5.39E-30**	0.84 (0.81, 0.87)	**3.28E-18**
**8.73E-41**	0.84 (0.81, 0.87)	**1.52E-18**
**2.32E-15**	0.97 (0.93, 1.00)	9.29E-02
5.27E-01	0.86 (0.83, 0.90)	**5.38E-13**
**2.96E-48**	1.10 (1.08, 1.12)	**3.15E-22**
**3.16E-33**	0.90 (0.87, 0.94)	**2.45E-07**

**1.28E-25**	0.84 (0.81, 0.88)	**6.23E-18**
**2.38E-25**	0.99 (0.95, 1.03)	7.02E-01
**5.08E-06**	1.06 (1.04, 1.09)	**3.18E-08**
**1.09E-57**	1.07 (1.05, 1.09)	**1.04E-14**
**3.68E-05**	0.88 (0.85, 0.91)	**3.91E-10**
**9.11E-25**	0.91 (0.88, 0.95)	**7.36E-06**
**5.05E-31**	0.86 (0.83, 0.90)	**1.65E-13**
**1.16E-02**	0.90 (0.86, 0.93)	**1.53E-07**
**5.22E-05**	0.89 (0.86, 0.93)	**1.07E-08**
**3.26E-30**	0.92 (0.88, 0.95)	**1.74E-05**
**9.36E-35**	0.85 (0.82, 0.88)	**4.75E-17**
**1.02E-14**	0.98 (0.95, 1.02)	4.76E-01
**1.21E-18**	0.87 (0.84, 0.91)	**1.07E-11**
**5.90E-33**	0.92 (0.89, 0.96)	**2.68E-05**
**5.06E-35**	0.85 (0.82, 0.88)	**1.02E-16**
**1.53E-15**	0.98 (0.94, 1.02)	3.27E-01
**2.12E-22**	0.89 (0.86, 0.93)	**4.80E-09**
**1.12E-17**	0.97 (0.93, 1.01)	1.61E-01
4.77E-01	1.01 (0.96, 1.05)	7.70E-01

Supplementary Table 9. Association of

		CKB	
		All causes	
		Age < 60y	
**Metabolites**	**Group**	**Adjusted HR (95% CI)**	**P value**
Concentration of chylomicrons and	Lipoprotein subclasses	0.96 (0.90, 1.01)	2.29E-01
Total lipids in chylomicrons and e:	Lipoprotein subclasses	0.96 (0.91, 1.01)	2.55E-01
Phospholipids in chylomicrons and	Lipoprotein subclasses	0.97 (0.91, 1.02)	3.81E-01
Cholesterol in chylomicrons and e:	Lipoprotein subclasses	0.99 (0.93, 1.04)	7.26E-01
Cholesteryl esters in chylomicrons	Lipoprotein subclasses	1.00 (0.94, 1.05)	9.05E-01
Free cholesterol in chylomicrons a	Lipoprotein subclasses	0.97 (0.92, 1.03)	5.51E-01
Triglycerides in chylomicrons and	Lipoprotein subclasses	0.95 (0.90, 1.01)	1.65E-01
Concentration of very large VLDL	Lipoprotein subclasses	0.98 (0.93, 1.03)	5.75E-01
Total lipids in very large VLDL	Lipoprotein subclasses	0.98 (0.93, 1.03)	5.92E-01
Phospholipids in very large VLDL	Lipoprotein subclasses	0.98 (0.93, 1.04)	6.28E-01
Cholesterol in very large VLDL	Lipoprotein subclasses	0.99 (0.94, 1.05)	7.83E-01
Cholesteryl esters in very large VL	Lipoprotein subclasses	0.99 (0.94, 1.05)	8.46E-01
Free cholesterol in very large VLD	Lipoprotein subclasses	0.99 (0.94, 1.04)	7.39E-01
Triglycerides in very large VLDL	Lipoprotein subclasses	0.97 (0.92, 1.03)	5.51E-01
Concentration of large VLDL parti	Lipoprotein subclasses	0.98 (0.92, 1.03)	5.51E-01
Total lipids in large VLDL	Lipoprotein subclasses	0.98 (0.92, 1.03)	5.51E-01
Phospholipids in large VLDL	Lipoprotein subclasses	0.98 (0.92, 1.03)	5.57E-01
Cholesterol in large VLDL	Lipoprotein subclasses	0.98 (0.92, 1.03)	5.51E-01
Cholesteryl esters in large VLDL	Lipoprotein subclasses	0.97 (0.92, 1.02)	4.48E-01
Free cholesterol in large VLDL	Lipoprotein subclasses	0.98 (0.93, 1.04)	6.21E-01
Triglycerides in large VLDL	Lipoprotein subclasses	0.98 (0.92, 1.03)	5.51E-01
Concentration of medium VLDL p	Lipoprotein subclasses	0.98 (0.93, 1.03)	5.68E-01
Total lipids in medium VLDL	Lipoprotein subclasses	0.98 (0.93, 1.03)	5.75E-01
Phospholipids in medium VLDL	Lipoprotein subclasses	0.98 (0.92, 1.03)	5.51E-01
Cholesterol in medium VLDL	Lipoprotein subclasses	0.98 (0.93, 1.04)	6.50E-01
Cholesteryl esters in medium VLD	Lipoprotein subclasses	0.98 (0.93, 1.03)	5.97E-01
Free cholesterol in medium VLDL	Lipoprotein subclasses	0.99 (0.93, 1.04)	7.22E-01
Triglycerides in medium VLDL	Lipoprotein subclasses	0.98 (0.93, 1.03)	5.64E-01
Concentration of small VLDL part	Lipoprotein subclasses	0.99 (0.94, 1.04)	7.59E-01
Total lipids in small VLDL	Lipoprotein subclasses	0.99 (0.94, 1.05)	8.78E-01
Phospholipids in small VLDL	Lipoprotein subclasses	0.98 (0.93, 1.03)	6.01E-01
Cholesterol in small VLDL	Lipoprotein subclasses	1.04 (0.99, 1.10)	2.66E-01
Cholesteryl esters in small VLDL	Lipoprotein subclasses	1.05 (1.00, 1.11)	1.32E-01
Free cholesterol in small VLDL	Lipoprotein subclasses	1.01 (0.96, 1.07)	7.07E-01
Triglycerides in small VLDL	Lipoprotein subclasses	0.97 (0.92, 1.03)	5.23E-01
Concentration of very small VLDI	Lipoprotein subclasses	1.05 (0.99, 1.10)	2.28E-01
Total lipids in very small VLDL	Lipoprotein subclasses	1.05 (1.00, 1.11)	1.59E-01
Phospholipids in very small VLDI	Lipoprotein subclasses	1.01 (0.96, 1.07)	7.66E-01
Cholesterol in very small VLDL	Lipoprotein subclasses	1.08 (1.03, 1.14)	**9.34E-03**
Cholesteryl esters in very small VI	Lipoprotein subclasses	1.09 (1.03, 1.15)	**6.14E-03**

Free cholesterol in very small VLI	Lipoprotein subclasses	1.07 (1.01, 1.13)	**3.54E-02**
Triglycerides in very small VLDL	Lipoprotein subclasses	1.00 (0.95, 1.06)	9.05E-01
Concentration of IDL particles	Lipoprotein subclasses	1.00 (0.95, 1.06)	9.59E-01
Total lipids in IDL	Lipoprotein subclasses	1.00 (0.95, 1.06)	9.68E-01
Phospholipids in IDL	Lipoprotein subclasses	0.99 (0.94, 1.04)	7.39E-01
Cholesterol in IDL	Lipoprotein subclasses	1.00 (0.95, 1.05)	9.54E-01
Cholesteryl esters in IDL	Lipoprotein subclasses	0.99 (0.94, 1.04)	7.64E-01
Free cholesterol in IDL	Lipoprotein subclasses	1.02 (0.96, 1.07)	6.50E-01
Triglycerides in IDL	Lipoprotein subclasses	1.05 (0.99, 1.11)	1.72E-01
Concentration of large LDL particl	Lipoprotein subclasses	0.99 (0.93, 1.04)	7.27E-01
Total lipids in large LDL	Lipoprotein subclasses	0.99 (0.93, 1.04)	7.07E-01
Phospholipids in large LDL	Lipoprotein subclasses	0.96 (0.91, 1.02)	3.44E-01
Cholesterol in large LDL	Lipoprotein subclasses	0.98 (0.93, 1.04)	6.50E-01
Cholesteryl esters in large LDL	Lipoprotein subclasses	0.98 (0.93, 1.03)	5.83E-01
Free cholesterol in large LDL	Lipoprotein subclasses	0.99 (0.94, 1.05)	8.73E-01
Triglycerides in large LDL	Lipoprotein subclasses	1.07 (1.02, 1.13)	**2.84E-02**
Concentration of medium LDL par	Lipoprotein subclasses	0.96 (0.91, 1.02)	3.41E-01
Total lipids in medium LDL	Lipoprotein subclasses	0.96 (0.91, 1.02)	3.10E-01
Phospholipids in medium LDL	Lipoprotein subclasses	0.92 (0.87, 0.97)	**1.26E-02**
Cholesterol in medium LDL	Lipoprotein subclasses	0.97 (0.92, 1.02)	4.24E-01
Cholesteryl esters in medium LDL	Lipoprotein subclasses	0.97 (0.92, 1.03)	5.14E-01
Free cholesterol in medium LDL	Lipoprotein subclasses	0.95 (0.90, 1.00)	1.42E-01
Triglycerides in medium LDL	Lipoprotein subclasses	1.05 (1.00, 1.11)	1.64E-01
Concentration of small LDL partic	Lipoprotein subclasses	0.96 (0.91, 1.02)	3.04E-01
Total lipids in small LDL	Lipoprotein subclasses	0.96 (0.91, 1.02)	2.99E-01
Phospholipids in small LDL	Lipoprotein subclasses	0.92 (0.87, 0.97)	**9.03E-03**
Cholesterol in small LDL	Lipoprotein subclasses	0.97 (0.92, 1.03)	5.22E-01
Cholesteryl esters in small LDL	Lipoprotein subclasses	0.98 (0.93, 1.03)	5.77E-01
Free cholesterol in small LDL	Lipoprotein subclasses	0.95 (0.90, 1.00)	1.51E-01
Triglycerides in small LDL	Lipoprotein subclasses	1.02 (0.96, 1.07)	6.86E-01
Concentration of very large HDL p	Lipoprotein subclasses	1.16 (1.10, 1.22)	**2.22E-07**
Total lipids in very large HDL	Lipoprotein subclasses	1.16 (1.10, 1.21)	**2.69E-07**
Phospholipids in very large HDL	Lipoprotein subclasses	1.14 (1.08, 1.20)	**3.45E-06**
Cholesterol in very large HDL	Lipoprotein subclasses	1.16 (1.10, 1.22)	**3.10E-07**
Cholesteryl esters in very large HI	Lipoprotein subclasses	1.16 (1.10, 1.22)	**1.74E-07**
Free cholesterol in very large HDI	Lipoprotein subclasses	1.14 (1.09, 1.20)	**3.08E-06**
Triglycerides in very large HDL	Lipoprotein subclasses	1.12 (1.07, 1.18)	**3.61E-05**
Concentration of large HDL partic	Lipoprotein subclasses	1.10 (1.04, 1.15)	**2.26E-03**
Total lipids in large HDL	Lipoprotein subclasses	1.10 (1.04, 1.15)	**2.19E-03**
Phospholipids in large HDL	Lipoprotein subclasses	1.08 (1.03, 1.14)	**7.53E-03**
Cholesterol in large HDL	Lipoprotein subclasses	1.10 (1.04, 1.16)	**1.42E-03**
Cholesteryl esters in large HDL	Lipoprotein subclasses	1.10 (1.04, 1.15)	**2.06E-03**
Free cholesterol in large HDL	Lipoprotein subclasses	1.11 (1.05, 1.17)	**5.00E-04**
Triglycerides in large HDL	Lipoprotein subclasses	1.11 (1.05, 1.17)	**4.63E-04**
**Concentration of medium HDL p**	Lipoprotein subclasses	0.89 (0.84, 0.94)	**2.78E-04**
**Total lipids in medium HDL**	Lipoprotein subclasses	0.89 (0.84, 0.94)	**3.70E-04**
**Phospholipids in medium HDL**	Lipoprotein subclasses	0.92 (0.87, 0.97)	**9.98E-03**
**Cholesterol in medium HDL**	Lipoprotein subclasses	0.89 (0.84, 0.94)	**1.14E-04**

**Cholesteryl esters in medium HI**	Lipoprotein subclasses	0.88 (0.83, 0.93)	**2.98E-05**
**Free cholesterol in medium HDL**	Lipoprotein subclasses	0.92 (0.87, 0.97)	**1.13E-02**
Triglycerides in medium HDL	Lipoprotein subclasses	0.90 (0.85, 0.95)	**1.42E-03**
Concentration of small HDL partic	Lipoprotein subclasses	0.84 (0.80, 0.88)	**3.91E-10**
Total lipids in small HDL	Lipoprotein subclasses	0.84 (0.80, 0.88)	**5.42E-10**
Phospholipids in small HDL	Lipoprotein subclasses	0.87 (0.82, 0.91)	**3.08E-06**
Cholesterol in small HDL	Lipoprotein subclasses	0.91 (0.86, 0.95)	**8.78E-04**
Cholesteryl esters in small HDL	Lipoprotein subclasses	0.93 (0.88, 0.98)	**1.14E-02**
**Free cholesterol in small HDL**	Lipoprotein subclasses	0.88 (0.84, 0.93)	**6.00E-05**
Triglycerides in small HDL	Lipoprotein subclasses	0.94 (0.89, 0.99)	5.61E-02
Phospholipids to total lipids ratio i	Relative lipoprotein lipi	0.95 (0.90, 1.01)	1.91E-01
Cholesterol to total lipids ratio in c	Relative lipoprotein lipi	1.16 (1.11, 1.20)	**2.39E-11**
Cholesteryl esters to total lipids ra	Relative lipoprotein lipi	1.15 (1.11, 1.20)	**2.39E-11**
Free cholesterol to total lipids ratio	Relative lipoprotein lipi	1.07 (1.01, 1.13)	**3.94E-02**
Triglycerides to total lipids ratio ir	Relative lipoprotein lipi	0.87 (0.83, 0.92)	**7.80E-07**
Phospholipids to total lipids ratio i	Relative lipoprotein lipi	0.96 (0.91, 1.01)	2.28E-01
**Cholesterol to total lipids ratio i**	Relative lipoprotein lipi	1.10 (1.06, 1.14)	**2.98E-05**
Cholesteryl esters to total lipids ra	Relative lipoprotein lipi	1.13 (1.08, 1.19)	**3.08E-06**
**Free cholesterol to total lipids ra**	Relative lipoprotein lipi	1.01 (0.96, 1.07)	7.59E-01
**Triglycerides to total lipids ratio**	Relative lipoprotein lipi	0.94 (0.89, 0.99)	5.69E-02
Phospholipids to total lipids ratio i	Relative lipoprotein lipi	0.97 (0.92, 1.03)	5.51E-01
Cholesterol to total lipids ratio in l	Relative lipoprotein lipi	0.98 (0.93, 1.03)	5.87E-01
Cholesteryl esters to total lipids ra	Relative lipoprotein lipi	1.00 (0.95, 1.06)	9.68E-01
Free cholesterol to total lipids ratio	Relative lipoprotein lipi	0.97 (0.92, 1.03)	5.29E-01
Triglycerides to total lipids ratio ir	Relative lipoprotein lipi	1.02 (0.97, 1.08)	5.51E-01
Phospholipids to total lipids ratio i	Relative lipoprotein lipi	0.96 (0.91, 1.01)	2.66E-01
**Cholesterol to total lipids ratio i**	Relative lipoprotein lipi	1.03 (0.97, 1.09)	5.51E-01
**Cholesteryl esters to total lipids**	Relative lipoprotein lipi	1.01 (0.96, 1.07)	7.26E-01
Free cholesterol to total lipids ratio	Relative lipoprotein lipi	1.03 (0.98, 1.10)	4.06E-01
Triglycerides to total lipids ratio ir	Relative lipoprotein lipi	0.98 (0.93, 1.04)	6.80E-01
**Phospholipids to total lipids rati**	Relative lipoprotein lipi	0.94 (0.89, 1.00)	8.09E-02
Cholesterol to total lipids ratio in s	Relative lipoprotein lipi	1.09 (1.03, 1.15)	**8.13E-03**
Cholesteryl esters to total lipids ra	Relative lipoprotein lipi	1.08 (1.02, 1.14)	**2.54E-02**
**Free cholesterol to total lipids ra**	Relative lipoprotein lipi	1.13 (1.07, 1.19)	**4.35E-05**
Triglycerides to total lipids ratio ir	Relative lipoprotein lipi	0.93 (0.89, 0.99)	**3.54E-02**
Phospholipids to total lipids ratio i	Relative lipoprotein lipi	0.93 (0.88, 0.97)	**9.70E-03**
Cholesterol to total lipids ratio in v	Relative lipoprotein lipi	1.11 (1.05, 1.18)	**6.43E-04**
Cholesteryl esters to total lipids ra	Relative lipoprotein lipi	1.11 (1.05, 1.17)	**1.42E-03**
Free cholesterol to total lipids ratio	Relative lipoprotein lipi	1.07 (1.01, 1.13)	**3.92E-02**
Triglycerides to total lipids ratio ir	Relative lipoprotein lipi	0.96 (0.91, 1.01)	2.66E-01
Phospholipids to total lipids ratio i	Relative lipoprotein lipi	0.94 (0.90, 0.99)	6.74E-02
Cholesterol to total lipids ratio in I	Relative lipoprotein lipi	0.98 (0.93, 1.03)	5.57E-01
Cholesteryl esters to total lipids ra	Relative lipoprotein lipi	0.93 (0.89, 0.98)	**3.03E-02**
Free cholesterol to total lipids ratio	Relative lipoprotein lipi	1.04 (0.99, 1.10)	2.68E-01
Triglycerides to total lipids ratio ir	Relative lipoprotein lipi	1.04 (0.99, 1.09)	2.39E-01
Phospholipids to total lipids ratio i	Relative lipoprotein lipi	0.98 (0.93, 1.04)	6.28E-01
Cholesterol to total lipids ratio in l	Relative lipoprotein lipi	0.97 (0.92, 1.02)	3.44E-01
Cholesteryl esters to total lipids ra	Relative lipoprotein lipi	0.95 (0.91, 1.00)	9.49E-02

Free cholesterol to total lipids ratic	Relative lipoprotein lipi	1.05 (1.00, 1.11)	1.65E-01
Triglycerides to total lipids ratio ir	Relative lipoprotein lipi	1.06 (1.02, 1.11)	**2.48E-02**
Phospholipids to total lipids ratio i	Relative lipoprotein lipi	0.98 (0.93, 1.04)	6.50E-01
Cholesterol to total lipids ratio in r	Relative lipoprotein lipi	0.98 (0.93, 1.03)	6.28E-01
Cholesteryl esters to total lipids rat	Relative lipoprotein lipi	0.98 (0.93, 1.03)	5.51E-01
Free cholesterol to total lipids ratic	Relative lipoprotein lipi	1.03 (0.98, 1.09)	3.81E-01
Triglycerides to total lipids ratio ir	Relative lipoprotein lipi	1.08 (1.03, 1.14)	**5.54E-03**
Phospholipids to total lipids ratio i	Relative lipoprotein lipi	0.99 (0.93, 1.04)	7.15E-01
Cholesterol to total lipids ratio in s	Relative lipoprotein lipi	0.99 (0.94, 1.05)	8.12E-01
Cholesteryl esters to total lipids rat	Relative lipoprotein lipi	0.99 (0.94, 1.04)	7.39E-01
Free cholesterol to total lipids ratic	Relative lipoprotein lipi	1.03 (0.97, 1.09)	5.21E-01
Triglycerides to total lipids ratio ir	Relative lipoprotein lipi	1.05 (1.00, 1.10)	1.49E-01
Phospholipids to total lipids ratio i	Relative lipoprotein lipi	0.99 (0.94, 1.04)	7.66E-01
Cholesterol to total lipids ratio in v	Relative lipoprotein lipi	1.02 (0.97, 1.08)	5.92E-01
Cholesteryl esters to total lipids rat	Relative lipoprotein lipi	1.02 (0.97, 1.08)	5.57E-01
Free cholesterol to total lipids ratic	Relative lipoprotein lipi	1.00 (0.95, 1.06)	9.68E-01
Triglycerides to total lipids ratio ir	Relative lipoprotein lipi	0.98 (0.93, 1.03)	5.57E-01
Phospholipids to total lipids ratio i	Relative lipoprotein lipi	0.89 (0.84, 0.94)	**4.77E-04**
Cholesterol to total lipids ratio in l	Relative lipoprotein lipi	1.09 (1.03, 1.15)	**1.05E-02**
Cholesteryl esters to total lipids rat	Relative lipoprotein lipi	1.05 (0.99, 1.11)	1.74E-01
Free cholesterol to total lipids ratic	Relative lipoprotein lipi	1.12 (1.06, 1.19)	**5.87E-04**
Triglycerides to total lipids ratio ir	Relative lipoprotein lipi	1.03 (0.98, 1.09)	4.48E-01
Phospholipids to total lipids ratio i	Relative lipoprotein lipi	1.18 (1.13, 1.22)	**3.21E-15**
Cholesterol to total lipids ratio in r	Relative lipoprotein lipi	0.90 (0.86, 0.94)	**1.05E-04**
Cholesteryl esters to total lipids rat	Relative lipoprotein lipi	0.89 (0.85, 0.94)	**3.61E-05**
Free cholesterol to total lipids ratic	Relative lipoprotein lipi	0.96 (0.91, 1.01)	2.40E-01
Triglycerides to total lipids ratio ir	Relative lipoprotein lipi	0.99 (0.94, 1.04)	7.42E-01
Phospholipids to total lipids ratio i	Relative lipoprotein lipi	0.98 (0.93, 1.03)	5.75E-01
Cholesterol to total lipids ratio in s	Relative lipoprotein lipi	1.02 (0.96, 1.07)	6.50E-01
Cholesteryl esters to total lipids rat	Relative lipoprotein lipi	1.01 (0.96, 1.07)	7.39E-01
Free cholesterol to total lipids ratic	Relative lipoprotein lipi	1.03 (0.98, 1.09)	4.14E-01
Triglycerides to total lipids ratio ir	Relative lipoprotein lipi	1.00 (0.95, 1.06)	9.05E-01
Average diameter for VLDL partic	Lipoprotein particle size	0.95 (0.90, 1.00)	1.03E-01
Average diameter for LDL particle	Lipoprotein particle size	1.07 (1.02, 1.13)	**2.84E-02**
Average diameter for HDL particle	Lipoprotein particle size	1.15 (1.09, 1.21)	**1.85E-06**
Total cholesterol	Cholesterol	1.01 (0.96, 1.07)	7.76E-01
VLDL cholesterol	Cholesterol	1.02 (0.96, 1.07)	6.50E-01
Remnant cholesterol (non-HDL, nc	Cholesterol	1.01 (0.96, 1.07)	7.59E-01
LDL cholesterol	Cholesterol	0.98 (0.93, 1.04)	6.28E-01
**HDL cholesterol**	Cholesterol	1.04 (0.99, 1.10)	2.57E-01
**HDL2 cholesterol**	Cholesterol	1.03 (0.98, 1.09)	4.46E-01
HDL3 cholesterol	Cholesterol	1.13 (1.07, 1.19)	**4.03E-05**
Total esterified cholesterol	Cholesteryl esters	0.92 (0.87, 0.97)	**9.96E-03**
Total free cholesterol	Free cholesterol	0.92 (0.86, 0.97)	**9.98E-03**
Total triglycerides	Triglycerides	0.99 (0.93, 1.04)	7.26E-01
Triglycerides in VLDL	Triglycerides	0.98 (0.93, 1.03)	5.66E-01
Triglycerides in LDL	Triglycerides	1.06 (1.01, 1.12)	5.06E-02
Triglycerides in HDL	Triglycerides	1.00 (0.95, 1.06)	9.08E-01

Phosphoglycerides	Other lipids	0.97 (0.92, 1.03)	5.51E-01
Ratio of triglycerides to phosphogl	Other lipids	0.98 (0.93, 1.04)	6.80E-01
Phosphatidylcholines	Other lipids	0.89 (0.84, 0.94)	**9.30E-05**
Sphingomyelins	Other lipids	0.86 (0.81, 0.90)	**5.83E-07**
Total cholines	Other lipids	0.95 (0.90, 1.01)	1.78E-01
Apolipoprotein A1	Apolipoproteins	1.04 (0.99, 1.10)	2.50E-01
Apolipoprotein B	Apolipoproteins	0.98 (0.93, 1.04)	6.50E-01
Ratio of apolipoprotein B to apolip	Apolipoproteins	0.97 (0.91, 1.02)	3.81E-01
Total fatty acids	Fatty acids	0.93 (0.88, 0.98)	**3.54E-02**
Degree of unsaturation	Fatty acids	0.81 (0.77, 0.85)	**1.51E-15**
Docosahexaenoic acid	Fatty acids	1.09 (1.04, 1.15)	**4.02E-03**
Linoleic acid	Fatty acids	0.90 (0.86, 0.96)	**2.12E-03**
Omega-3 fatty acids	Fatty acids	1.00 (0.95, 1.06)	9.05E-01
Omega-6 fatty acids	Fatty acids	0.89 (0.84, 0.94)	**3.90E-04**
Polyunsaturated fatty acids	Fatty acids	0.90 (0.85, 0.96)	**2.30E-03**
Monounsaturated fatty acids	Fatty acids	0.95 (0.90, 1.01)	1.74E-01
Saturated fatty acids	Fatty acids	0.94 (0.89, 1.00)	9.49E-02
Ratio of docosahexaenoic acid to t	Fatty acids	1.18 (1.12, 1.25)	**7.80E-09**
Ratio of linoleic acid to total fatty	Fatty acids	0.91 (0.86, 0.95)	**5.87E-04**
Ratio of omega-3 fatty acids to tota	Fatty acids	1.08 (1.02, 1.14)	**1.41E-02**
Ratio of omega-6 fatty acids to tota	Fatty acids	0.90 (0.85, 0.95)	**6.25E-04**
Ratio of polyunsaturated fatty acid	Fatty acids	0.92 (0.87, 0.97)	**6.84E-03**
Ratio of monounsaturated fatty aci	Fatty acids	1.04 (0.98, 1.09)	3.03E-01
Ratio of saturated fatty acids to tot	Fatty acids	1.10 (1.04, 1.16)	**3.64E-03**
Glucose	Glycolysis related metab	1.14 (1.10, 1.19)	**3.50E-10**
Lactate	Glycolysis related metab	0.99 (0.94, 1.04)	7.47E-01
Citrate	Glycolysis related metab	0.99 (0.93, 1.04)	7.39E-01
Alanine	Amino acids	0.96 (0.91, 1.01)	2.05E-01
Glutamine	Amino acids	0.94 (0.89, 0.99)	**4.79E-02**
Histidine	Amino acids	0.99 (0.94, 1.05)	8.72E-01
Isoleucine	Amino acids	0.94 (0.89, 1.00)	8.80E-02
Leucine	Amino acids	0.96 (0.91, 1.02)	2.81E-01
Valine	Amino acids	0.83 (0.78, 0.88)	**1.00E-08**
Phenylalanine	Amino acids	1.11 (1.07, 1.15)	**2.04E-08**
Tyrosine	Amino acids	1.07 (1.01, 1.13)	**4.85E-02**
Acetate	Ketone bodies	1.07 (1.03, 1.12)	**1.42E-03**
Acetoacetate	Ketone bodies	1.03 (0.98, 1.09)	4.29E-01
**3-Hydroxybutyrate**	Ketone bodies	1.06 (1.01, 1.11)	5.06E-02
Creatinine	Fluid balance	1.05 (1.02, 1.08)	**1.25E-03**
Albumin	Fluid balance	0.84 (0.80, 0.88)	**3.51E-11**
Glycoprotein acetyls	Inflammation	1.14 (1.08, 1.20)	**1.76E-05**
Acetone	Ketone bodies		
Cholesteryl esters in HDL	Cholesteryl esters		
Cholesteryl esters in LDL	Cholesteryl esters		
Cholesteryl esters in VLDL/ (Chol	Cholesteryl esters		
Clinical LDL cholesterol	Cholesterol		
Concentration of HDL particles	Lipoprotein particle concentrations		
Concentration of LDL particles	Lipoprotein particle concentrations		

Concentration of VLDL particles	Lipoprotein particle concentrations
Free cholesterol in HDL	Free cholesterol
Free cholesterol in LDL	Free cholesterol
Free cholesterol in VLDL	Free cholesterol
Glycine	Amino acids
Phospholipids in HDL	Phospholipids
Phospholipids in LDL	Phospholipids
Phospholipids in VLDL	Phospholipids
Pyruvate	Glycolysis related metabolites
Ratio of omega-6 fatty acids to om	Fatty acids
Ratio of polyunsaturated fatty acid	Fatty acids
Total cholesterol minus HDL-C	Cholesterol
Total concentration of branched-cl	Amino acids
Total concentration of lipoprotein	Lipoprotein particle concentrations
Total lipids in HDL	Total lipids
Total lipids in LDL	Total lipids
Total lipids in lipoprotein particles	Total lipids
Total lipids in VLDL	Total lipids
Total phospholipids in lipoprotein	Phospholipids

Models were adjusted for age, sex, regions (CKB only), ethnicity (UKB only), levels of education, smoking

HR, hazard ratio; CI, confidence interval; LDL, Low Density Lipoprotein; HDL, High Density Lipoprotein

all metabolites with all-cause and cause-specific mortality in the CKB and UKB by age

mortality		Ischaemic heart diseases mortality		
Age >= 60y		Age < 60y		Age >= 60y
**Adjusted HR (95% CI)**	**P value**	**Adjusted HR (95% CI)**	**P value**	**Adjusted HR (95% CI)**
0.88 (0.81, 0.96)	**1.58E-02**	0.98 (0.90, 1.08)	8.78E-01	0.89 (0.75, 1.06)
0.88 (0.81, 0.96)	**1.92E-02**	0.99 (0.90, 1.08)	8.92E-01	0.90 (0.75, 1.07)
0.89 (0.82, 0.97)	**2.83E-02**	1.00 (0.91, 1.10)	9.72E-01	0.90 (0.76, 1.07)
0.93 (0.86, 1.01)	1.85E-01	1.01 (0.92, 1.11)	9.16E-01	0.95 (0.80, 1.13)
0.96 (0.88, 1.04)	3.87E-01	1.02 (0.93, 1.12)	7.73E-01	0.99 (0.84, 1.17)
0.91 (0.83, 0.99)	6.68E-02	0.99 (0.90, 1.09)	9.22E-01	0.89 (0.75, 1.07)
0.87 (0.80, 0.95)	**8.03E-03**	0.98 (0.89, 1.08)	7.93E-01	0.88 (0.74, 1.05)
0.90 (0.83, 0.99)	6.04E-02	0.99 (0.90, 1.09)	9.16E-01	0.87 (0.73, 1.04)
0.91 (0.83, 0.99)	6.70E-02	0.99 (0.90, 1.09)	9.22E-01	0.88 (0.73, 1.05)
0.91 (0.84, 0.99)	7.52E-02	1.00 (0.91, 1.10)	9.80E-01	0.88 (0.74, 1.06)
0.93 (0.86, 1.01)	1.70E-01	1.01 (0.92, 1.11)	9.22E-01	0.93 (0.78, 1.10)
0.94 (0.86, 1.02)	2.09E-01	1.01 (0.92, 1.11)	9.16E-01	0.94 (0.79, 1.12)
0.92 (0.85, 1.01)	1.29E-01	1.00 (0.92, 1.10)	9.52E-01	0.91 (0.76, 1.08)
0.90 (0.83, 0.98)	**4.43E-02**	0.99 (0.90, 1.08)	8.80E-01	0.86 (0.72, 1.03)
0.91 (0.84, 0.99)	7.40E-02	1.01 (0.92, 1.11)	9.22E-01	0.91 (0.76, 1.08)
0.91 (0.84, 0.99)	7.43E-02	1.01 (0.92, 1.11)	9.22E-01	0.91 (0.76, 1.08)
0.91 (0.84, 0.99)	7.21E-02	1.01 (0.92, 1.11)	9.22E-01	0.90 (0.76, 1.08)
0.92 (0.84, 0.99)	8.16E-02	1.00 (0.91, 1.10)	9.68E-01	0.91 (0.76, 1.08)
0.92 (0.84, 1.00)	8.39E-02	1.00 (0.91, 1.10)	9.68E-01	0.91 (0.76, 1.08)
0.92 (0.84, 0.99)	8.25E-02	1.01 (0.92, 1.11)	9.22E-01	0.90 (0.76, 1.08)
0.91 (0.84, 0.99)	7.21E-02	1.01 (0.92, 1.11)	9.16E-01	0.91 (0.77, 1.08)
0.92 (0.84, 0.99)	8.16E-02	1.03 (0.94, 1.13)	7.54E-01	0.94 (0.79, 1.11)
0.92 (0.85, 0.99)	8.25E-02	1.03 (0.94, 1.13)	7.54E-01	0.94 (0.79, 1.11)
0.91 (0.84, 0.99)	7.39E-02	1.02 (0.93, 1.12)	7.73E-01	0.93 (0.78, 1.10)
0.93 (0.86, 1.01)	1.41E-01	1.02 (0.93, 1.13)	7.73E-01	0.93 (0.78, 1.11)
0.93 (0.86, 1.01)	1.85E-01	1.02 (0.93, 1.12)	8.56E-01	0.93 (0.78, 1.12)
0.93 (0.85, 1.00)	1.16E-01	1.03 (0.94, 1.13)	7.18E-01	0.94 (0.79, 1.11)
0.91 (0.84, 0.99)	7.52E-02	1.03 (0.94, 1.13)	7.40E-01	0.94 (0.79, 1.12)
0.94 (0.86, 1.01)	1.87E-01	1.06 (0.97, 1.17)	4.09E-01	0.94 (0.79, 1.13)
0.94 (0.87, 1.02)	2.57E-01	1.08 (0.98, 1.18)	2.97E-01	0.96 (0.80, 1.14)
0.92 (0.85, 1.00)	9.89E-02	1.05 (0.96, 1.16)	5.19E-01	0.91 (0.76, 1.10)
1.02 (0.94, 1.11)	6.82E-01	1.18 (1.07, 1.29)	**2.74E-03**	1.11 (0.94, 1.31)
1.05 (0.97, 1.14)	3.23E-01	1.20 (1.10, 1.31)	**7.99E-04**	1.17 (0.99, 1.38)
0.97 (0.90, 1.05)	5.65E-01	1.11 (1.01, 1.22)	8.59E-02	0.99 (0.83, 1.18)
0.91 (0.84, 0.99)	6.04E-02	1.02 (0.93, 1.12)	8.66E-01	0.89 (0.74, 1.06)
1.02 (0.94, 1.10)	7.04E-01	1.15 (1.05, 1.26)	**1.34E-02**	1.07 (0.91, 1.27)
1.03 (0.96, 1.11)	5.17E-01	1.16 (1.06, 1.27)	**6.68E-03**	1.10 (0.94, 1.30)
1.00 (0.93, 1.08)	9.96E-01	1.07 (0.98, 1.18)	3.37E-01	1.00 (0.84, 1.18)
1.09 (1.02, 1.18)	5.01E-02	1.23 (1.13, 1.34)	**6.02E-05**	1.26 (1.08, 1.46)
1.10 (1.02, 1.18)	**3.66E-02**	1.23 (1.13, 1.35)	**3.97E-05**	1.29 (1.11, 1.50)

1.08 (1.00, 1.16)	9.87E-02	1.20 (1.10, 1.32)	**6.24E-04**	1.18 (1.01, 1.38)
0.94 (0.87, 1.02)	2.23E-01	1.04 (0.94, 1.14)	6.43E-01	0.88 (0.74, 1.06)
1.00 (0.92, 1.07)	9.27E-01	1.05 (0.95, 1.15)	5.64E-01	0.98 (0.83, 1.17)
1.00 (0.93, 1.08)	9.96E-01	1.05 (0.95, 1.15)	5.57E-01	0.99 (0.83, 1.18)
0.99 (0.91, 1.07)	7.95E-01	1.03 (0.94, 1.13)	7.40E-01	0.97 (0.82, 1.15)
1.00 (0.93, 1.08)	9.27E-01	1.05 (0.96, 1.16)	5.05E-01	1.01 (0.85, 1.20)
0.99 (0.92, 1.07)	9.07E-01	1.05 (0.95, 1.15)	5.61E-01	1.00 (0.84, 1.18)
1.03 (0.96, 1.11)	5.33E-01	1.06 (0.97, 1.17)	4.17E-01	1.04 (0.88, 1.23)
0.98 (0.91, 1.06)	6.77E-01	1.03 (0.94, 1.14)	6.76E-01	0.90 (0.75, 1.07)
0.98 (0.90, 1.06)	6.53E-01	1.04 (0.95, 1.15)	5.65E-01	0.96 (0.80, 1.14)
0.98 (0.90, 1.06)	6.77E-01	1.04 (0.95, 1.15)	5.76E-01	0.96 (0.81, 1.14)
0.96 (0.89, 1.04)	4.09E-01	1.02 (0.93, 1.12)	8.37E-01	0.92 (0.77, 1.10)
0.98 (0.91, 1.06)	7.28E-01	1.05 (0.95, 1.15)	5.64E-01	0.98 (0.82, 1.16)
0.97 (0.90, 1.05)	6.33E-01	1.05 (0.95, 1.15)	5.64E-01	0.97 (0.82, 1.16)
1.00 (0.93, 1.08)	9.52E-01	1.04 (0.95, 1.15)	5.76E-01	0.99 (0.83, 1.17)
1.00 (0.93, 1.08)	9.56E-01	1.06 (0.96, 1.16)	4.75E-01	0.93 (0.78, 1.11)
0.95 (0.88, 1.03)	3.56E-01	1.03 (0.94, 1.13)	7.40E-01	0.93 (0.78, 1.12)
0.95 (0.88, 1.03)	3.60E-01	1.03 (0.93, 1.13)	7.43E-01	0.94 (0.78, 1.12)
0.89 (0.82, 0.97)	**2.19E-02**	0.96 (0.87, 1.05)	5.64E-01	0.82 (0.68, 0.99)
0.97 (0.90, 1.05)	5.66E-01	1.05 (0.95, 1.15)	5.64E-01	0.97 (0.82, 1.16)
0.97 (0.90, 1.06)	6.44E-01	1.06 (0.96, 1.16)	4.87E-01	0.99 (0.83, 1.18)
0.95 (0.87, 1.03)	2.86E-01	1.00 (0.91, 1.10)	9.85E-01	0.91 (0.76, 1.09)
0.98 (0.91, 1.06)	7.41E-01	1.03 (0.93, 1.13)	7.66E-01	0.90 (0.75, 1.08)
0.95 (0.88, 1.03)	3.33E-01	1.04 (0.95, 1.15)	5.76E-01	0.95 (0.79, 1.13)
0.95 (0.88, 1.03)	3.56E-01	1.04 (0.95, 1.15)	5.65E-01	0.95 (0.80, 1.14)
0.88 (0.81, 0.96)	**1.04E-02**	0.96 (0.87, 1.06)	5.89E-01	0.82 (0.68, 0.98)
0.98 (0.90, 1.06)	6.77E-01	1.07 (0.97, 1.17)	3.92E-01	1.00 (0.84, 1.19)
0.98 (0.91, 1.06)	7.39E-01	1.07 (0.98, 1.18)	3.22E-01	1.01 (0.85, 1.21)
0.95 (0.87, 1.03)	2.81E-01	1.02 (0.93, 1.13)	7.84E-01	0.94 (0.78, 1.12)
0.95 (0.88, 1.03)	3.47E-01	1.04 (0.95, 1.15)	5.89E-01	0.92 (0.77, 1.10)
1.22 (1.14, 1.31)	**1.06E-06**	1.18 (1.08, 1.28)	**1.85E-03**	1.29 (1.11, 1.50)
1.22 (1.14, 1.31)	**1.06E-06**	1.18 (1.08, 1.28)	**1.56E-03**	1.29 (1.11, 1.51)
1.21 (1.12, 1.29)	**4.72E-06**	1.11 (1.01, 1.21)	7.80E-02	1.19 (1.02, 1.40)
1.22 (1.14, 1.31)	**9.79E-07**	1.24 (1.14, 1.36)	**1.68E-05**	1.40 (1.20, 1.63)
1.23 (1.14, 1.32)	**8.59E-07**	1.27 (1.17, 1.39)	**1.85E-06**	1.45 (1.25, 1.69)
1.21 (1.13, 1.30)	**2.51E-06**	1.17 (1.07, 1.28)	**2.59E-03**	1.27 (1.09, 1.48)
1.11 (1.04, 1.20)	**1.28E-02**	1.14 (1.05, 1.24)	**1.29E-02**	1.17 (1.00, 1.36)
1.16 (1.07, 1.25)	**6.50E-04**	1.05 (0.95, 1.15)	5.61E-01	1.06 (0.89, 1.25)
1.16 (1.08, 1.25)	**5.46E-04**	1.05 (0.96, 1.15)	5.42E-01	1.06 (0.90, 1.25)
1.15 (1.07, 1.24)	**1.33E-03**	1.05 (0.96, 1.15)	5.59E-01	1.05 (0.89, 1.24)
1.17 (1.08, 1.26)	**3.23E-04**	1.05 (0.96, 1.15)	4.95E-01	1.07 (0.91, 1.27)
1.16 (1.08, 1.25)	**4.96E-04**	1.04 (0.95, 1.14)	5.64E-01	1.06 (0.90, 1.25)
1.18 (1.10, 1.27)	**6.38E-05**	1.08 (0.99, 1.18)	2.52E-01	1.12 (0.95, 1.32)
1.08 (1.00, 1.16)	9.83E-02	1.00 (0.90, 1.10)	9.52E-01	0.96 (0.80, 1.14)
0.87 (0.80, 0.94)	**1.83E-03**	0.83 (0.76, 0.92)	**1.99E-03**	0.66 (0.56, 0.78)
0.87 (0.81, 0.94)	**3.38E-03**	0.84 (0.76, 0.92)	**2.46E-03**	0.67 (0.56, 0.79)
0.89 (0.82, 0.96)	**1.28E-02**	0.86 (0.78, 0.95)	**1.30E-02**	0.70 (0.58, 0.83)
0.89 (0.82, 0.96)	**1.16E-02**	0.85 (0.77, 0.93)	**3.47E-03**	0.70 (0.59, 0.82)

0.88 (0.82, 0.95)	**7.14E-03**	0.84 (0.77, 0.92)	**2.27E-03**	0.70 (0.59, 0.82)
0.92 (0.85, 0.99)	7.21E-02	0.88 (0.80, 0.96)	**2.75E-02**	0.72 (0.60, 0.85)
0.82 (0.75, 0.89)	**2.61E-05**	0.83 (0.75, 0.91)	**1.37E-03**	0.65 (0.54, 0.79)
0.77 (0.71, 0.83)	**2.41E-09**	0.80 (0.73, 0.87)	**6.33E-06**	0.58 (0.49, 0.68)
0.77 (0.71, 0.83)	**3.00E-09**	0.81 (0.74, 0.88)	**1.68E-05**	0.58 (0.49, 0.68)
0.80 (0.74, 0.87)	**8.59E-07**	0.82 (0.75, 0.91)	**7.97E-04**	0.65 (0.55, 0.76)
0.88 (0.81, 0.95)	**4.36E-03**	0.90 (0.82, 0.98)	6.74E-02	0.74 (0.63, 0.87)
0.91 (0.85, 0.98)	5.23E-02	0.92 (0.84, 1.01)	2.27E-01	0.81 (0.69, 0.94)
0.83 (0.77, 0.90)	**2.73E-05**	0.85 (0.77, 0.94)	**5.06E-03**	0.65 (0.56, 0.77)
0.84 (0.77, 0.91)	**1.75E-04**	0.88 (0.80, 0.97)	**3.42E-02**	0.73 (0.61, 0.88)
0.92 (0.85, 0.99)	7.43E-02	1.02 (0.92, 1.12)	8.80E-01	0.86 (0.74, 1.00)
1.16 (1.08, 1.23)	**1.84E-04**	1.13 (1.05, 1.21)	**6.83E-03**	1.21 (1.07, 1.37)
1.16 (1.09, 1.24)	**1.06E-04**	1.12 (1.04, 1.21)	**7.61E-03**	1.26 (1.12, 1.41)
1.05 (0.97, 1.14)	3.27E-01	1.06 (0.96, 1.16)	4.92E-01	0.90 (0.76, 1.07)
0.90 (0.83, 0.97)	**1.52E-02**	0.88 (0.81, 0.96)	**1.70E-02**	0.86 (0.74, 1.01)
0.92 (0.86, 0.99)	8.16E-02	1.02 (0.93, 1.13)	7.84E-01	0.89 (0.76, 1.05)
1.11 (1.04, 1.19)	**9.28E-03**	1.08 (1.01, 1.15)	9.99E-02	1.28 (1.13, 1.45)
1.10 (1.04, 1.17)	**7.45E-03**	1.09 (1.00, 1.19)	1.60E-01	1.28 (1.15, 1.43)
1.03 (0.94, 1.12)	6.31E-01	1.04 (0.95, 1.14)	5.65E-01	1.00 (0.82, 1.21)
0.94 (0.87, 1.02)	2.53E-01	0.92 (0.83, 1.00)	1.76E-01	0.80 (0.66, 0.97)
0.91 (0.84, 0.98)	**3.59E-02**	0.93 (0.85, 1.02)	3.22E-01	0.88 (0.76, 1.01)
0.98 (0.90, 1.06)	6.82E-01	0.92 (0.84, 1.00)	1.66E-01	0.95 (0.82, 1.10)
1.04 (0.96, 1.12)	5.05E-01	0.90 (0.81, 0.99)	1.07E-01	1.01 (0.84, 1.21)
0.94 (0.88, 1.01)	1.97E-01	0.98 (0.89, 1.08)	8.77E-01	0.93 (0.80, 1.08)
1.04 (0.97, 1.13)	3.82E-01	1.09 (1.00, 1.19)	1.57E-01	1.08 (0.93, 1.24)
0.93 (0.86, 1.00)	1.18E-01	0.86 (0.78, 0.95)	**1.41E-02**	0.73 (0.61, 0.88)
1.06 (0.98, 1.15)	1.97E-01	0.94 (0.85, 1.03)	3.92E-01	0.91 (0.76, 1.08)
1.07 (0.99, 1.15)	1.70E-01	0.92 (0.83, 1.02)	2.48E-01	0.92 (0.77, 1.10)
0.98 (0.91, 1.06)	7.47E-01	1.05 (0.95, 1.16)	5.65E-01	0.96 (0.81, 1.13)
0.96 (0.88, 1.03)	3.60E-01	1.09 (0.99, 1.20)	2.27E-01	1.16 (0.97, 1.38)
0.92 (0.85, 1.00)	8.30E-02	0.84 (0.76, 0.92)	**2.46E-03**	0.73 (0.60, 0.89)
1.19 (1.10, 1.29)	**1.02E-04**	1.18 (1.07, 1.30)	**3.30E-03**	1.39 (1.17, 1.66)
1.18 (1.09, 1.27)	**2.92E-04**	1.17 (1.06, 1.28)	**7.70E-03**	1.39 (1.17, 1.66)
1.20 (1.11, 1.29)	**2.46E-05**	1.19 (1.09, 1.30)	**8.75E-04**	1.26 (1.07, 1.47)
0.86 (0.80, 0.93)	**4.96E-04**	0.89 (0.81, 0.97)	**4.15E-02**	0.77 (0.65, 0.91)
0.93 (0.87, 1.00)	1.02E-01	0.87 (0.80, 0.94)	**3.82E-03**	0.82 (0.72, 0.93)
1.22 (1.13, 1.32)	**1.06E-05**	1.33 (1.19, 1.47)	**4.20E-06**	1.79 (1.48, 2.18)
1.19 (1.10, 1.29)	**9.68E-05**	1.28 (1.16, 1.41)	**1.87E-05**	1.72 (1.43, 2.07)
1.19 (1.10, 1.30)	**4.04E-04**	1.20 (1.08, 1.33)	**2.92E-03**	1.48 (1.20, 1.82)
0.88 (0.81, 0.95)	**7.92E-03**	0.89 (0.80, 0.98)	6.74E-02	0.72 (0.60, 0.87)
0.93 (0.86, 1.01)	1.70E-01	0.90 (0.83, 0.99)	8.10E-02	0.89 (0.75, 1.06)
1.04 (0.96, 1.12)	4.86E-01	1.05 (0.96, 1.16)	4.92E-01	1.16 (0.95, 1.40)
0.97 (0.90, 1.04)	4.99E-01	1.01 (0.92, 1.10)	9.16E-01	1.04 (0.88, 1.22)
1.12 (1.03, 1.22)	**2.98E-02**	1.06 (0.97, 1.17)	4.23E-01	1.21 (1.00, 1.47)
0.98 (0.91, 1.06)	7.59E-01	0.99 (0.90, 1.08)	8.94E-01	0.91 (0.76, 1.08)
0.96 (0.88, 1.04)	3.82E-01	0.96 (0.87, 1.06)	5.89E-01	0.93 (0.77, 1.11)
1.00 (0.93, 1.08)	9.68E-01	1.00 (0.92, 1.10)	9.52E-01	1.04 (0.87, 1.23)
0.96 (0.89, 1.03)	3.82E-01	0.99 (0.91, 1.08)	9.16E-01	0.98 (0.83, 1.16)

1.15 (1.06, 1.25)	**4.41E-03**	1.04 (0.95, 1.14)	5.89E-01	1.18 (0.98, 1.40)
1.03 (0.95, 1.11)	5.60E-01	1.02 (0.94, 1.11)	7.66E-01	1.01 (0.85, 1.19)
0.93 (0.86, 1.01)	1.32E-01	0.92 (0.83, 1.01)	2.27E-01	0.86 (0.72, 1.04)
1.04 (0.96, 1.12)	4.74E-01	1.06 (0.96, 1.17)	4.86E-01	1.11 (0.93, 1.32)
1.02 (0.94, 1.11)	6.82E-01	1.05 (0.95, 1.15)	5.64E-01	1.07 (0.90, 1.27)
1.03 (0.95, 1.11)	6.21E-01	0.99 (0.90, 1.09)	9.16E-01	1.02 (0.87, 1.20)
1.04 (0.96, 1.12)	4.66E-01	1.01 (0.92, 1.11)	9.16E-01	1.00 (0.84, 1.18)
0.93 (0.86, 1.01)	1.76E-01	0.90 (0.81, 1.00)	1.29E-01	0.84 (0.70, 1.01)
1.05 (0.97, 1.14)	3.27E-01	1.07 (0.97, 1.19)	3.55E-01	1.14 (0.95, 1.36)
1.04 (0.96, 1.13)	4.56E-01	1.07 (0.97, 1.18)	4.23E-01	1.11 (0.93, 1.33)
1.02 (0.94, 1.10)	7.25E-01	0.98 (0.89, 1.08)	8.56E-01	1.01 (0.86, 1.20)
0.99 (0.92, 1.07)	9.07E-01	1.00 (0.91, 1.10)	9.68E-01	0.97 (0.82, 1.15)
0.99 (0.92, 1.07)	8.58E-01	0.84 (0.77, 0.92)	**9.92E-04**	0.81 (0.70, 0.93)
1.05 (0.98, 1.14)	2.76E-01	1.27 (1.16, 1.41)	**1.87E-05**	1.45 (1.24, 1.71)
1.04 (0.96, 1.12)	4.39E-01	1.29 (1.17, 1.42)	**6.33E-06**	1.40 (1.21, 1.61)
1.09 (1.00, 1.18)	8.30E-02	1.09 (0.99, 1.20)	2.26E-01	1.22 (1.00, 1.49)
0.89 (0.82, 0.97)	**2.70E-02**	0.96 (0.87, 1.06)	5.89E-01	0.77 (0.63, 0.94)
0.85 (0.78, 0.92)	**5.34E-04**	0.95 (0.87, 1.05)	5.64E-01	0.83 (0.70, 1.00)
1.20 (1.11, 1.31)	**1.91E-04**	1.08 (0.98, 1.19)	2.97E-01	1.31 (1.07, 1.59)
1.14 (1.04, 1.24)	**1.28E-02**	0.99 (0.90, 1.08)	8.80E-01	1.12 (0.93, 1.36)
1.27 (1.16, 1.38)	**2.11E-06**	1.22 (1.10, 1.36)	**1.89E-03**	1.55 (1.26, 1.91)
0.92 (0.85, 0.99)	8.16E-02	0.94 (0.85, 1.04)	4.46E-01	0.83 (0.68, 1.00)
1.13 (1.06, 1.20)	**1.47E-03**	1.17 (1.09, 1.25)	**8.24E-05**	1.27 (1.13, 1.41)
0.99 (0.92, 1.07)	8.71E-01	0.93 (0.85, 1.02)	3.11E-01	0.95 (0.81, 1.11)
0.98 (0.91, 1.06)	7.62E-01	0.94 (0.85, 1.02)	3.28E-01	0.97 (0.82, 1.14)
1.02 (0.94, 1.10)	7.64E-01	0.95 (0.87, 1.04)	5.28E-01	0.91 (0.78, 1.07)
0.91 (0.84, 0.98)	**4.95E-02**	0.93 (0.84, 1.03)	3.46E-01	0.81 (0.68, 0.98)
0.96 (0.89, 1.04)	4.51E-01	0.96 (0.88, 1.06)	6.43E-01	0.96 (0.81, 1.14)
1.06 (0.98, 1.15)	2.40E-01	1.05 (0.95, 1.15)	5.64E-01	1.08 (0.91, 1.27)
1.05 (0.97, 1.13)	3.60E-01	1.04 (0.94, 1.14)	6.53E-01	1.06 (0.90, 1.26)
1.05 (0.97, 1.14)	3.18E-01	1.05 (0.96, 1.15)	5.34E-01	1.03 (0.86, 1.22)
0.92 (0.85, 1.00)	9.18E-02	0.95 (0.86, 1.05)	5.38E-01	0.88 (0.74, 1.04)
0.88 (0.82, 0.95)	**7.14E-03**	0.98 (0.89, 1.07)	7.73E-01	0.90 (0.77, 1.06)
1.13 (1.05, 1.22)	**7.14E-03**	0.94 (0.86, 1.04)	4.86E-01	1.06 (0.90, 1.26)
1.22 (1.13, 1.32)	**2.40E-06**	1.15 (1.05, 1.26)	**1.26E-02**	1.29 (1.10, 1.52)
1.02 (0.95, 1.10)	6.77E-01	1.09 (0.99, 1.20)	1.81E-01	1.03 (0.87, 1.21)
0.98 (0.91, 1.07)	7.64E-01	1.12 (1.02, 1.22)	6.74E-02	1.06 (0.90, 1.26)
0.99 (0.92, 1.07)	9.07E-01	1.11 (1.01, 1.21)	9.99E-02	1.05 (0.89, 1.24)
0.98 (0.91, 1.06)	7.64E-01	1.06 (0.96, 1.16)	4.55E-01	1.01 (0.85, 1.20)
1.10 (1.02, 1.19)	**3.66E-02**	1.03 (0.94, 1.13)	7.40E-01	1.00 (0.85, 1.19)
1.09 (1.01, 1.17)	7.87E-02	1.01 (0.92, 1.10)	9.49E-01	0.96 (0.81, 1.14)
1.20 (1.11, 1.29)	**1.76E-05**	1.23 (1.13, 1.35)	**7.76E-05**	1.37 (1.17, 1.61)
0.88 (0.81, 0.95)	**7.08E-03**	0.89 (0.81, 0.99)	8.59E-02	0.71 (0.59, 0.85)
0.94 (0.87, 1.02)	2.57E-01	0.94 (0.85, 1.04)	4.23E-01	0.84 (0.70, 1.01)
0.92 (0.85, 0.99)	8.16E-02	1.02 (0.93, 1.12)	8.30E-01	0.92 (0.77, 1.09)
0.91 (0.84, 0.99)	7.21E-02	1.02 (0.93, 1.12)	7.88E-01	0.93 (0.78, 1.11)
0.99 (0.92, 1.07)	9.07E-01	1.06 (0.96, 1.16)	4.86E-01	0.93 (0.78, 1.11)
0.92 (0.85, 1.00)	8.61E-02	0.92 (0.83, 1.01)	2.43E-01	0.79 (0.66, 0.95)

0.99 (0.92, 1.07)	9.07E-01	0.99 (0.90, 1.09)	9.14E-01	0.89 (0.75, 1.05)
0.94 (0.87, 1.02)	2.33E-01	1.05 (0.96, 1.15)	5.18E-01	0.94 (0.79, 1.11)
0.88 (0.81, 0.95)	**7.04E-03**	0.84 (0.76, 0.92)	**2.27E-03**	0.68 (0.57, 0.81)
0.86 (0.79, 0.93)	**1.01E-03**	0.84 (0.76, 0.92)	**2.05E-03**	0.68 (0.57, 0.82)
0.97 (0.90, 1.05)	5.78E-01	0.93 (0.84, 1.02)	3.23E-01	0.84 (0.71, 1.01)
1.08 (1.00, 1.16)	8.89E-02	1.07 (0.98, 1.17)	3.23E-01	1.00 (0.84, 1.18)
0.96 (0.88, 1.04)	3.76E-01	1.07 (0.97, 1.18)	3.58E-01	0.97 (0.82, 1.16)
0.92 (0.84, 0.99)	8.06E-02	1.03 (0.94, 1.13)	7.15E-01	0.95 (0.79, 1.13)
0.92 (0.84, 1.00)	8.28E-02	0.96 (0.87, 1.06)	6.25E-01	0.78 (0.65, 0.95)
0.80 (0.74, 0.86)	**3.26E-07**	0.76 (0.70, 0.82)	**7.14E-10**	0.67 (0.59, 0.75)
1.11 (1.03, 1.20)	**1.84E-02**	1.36 (1.25, 1.47)	**8.06E-12**	1.49 (1.28, 1.73)
0.92 (0.84, 1.00)	9.89E-02	0.93 (0.85, 1.03)	3.79E-01	0.74 (0.61, 0.90)
0.98 (0.90, 1.06)	6.82E-01	1.19 (1.08, 1.30)	**1.37E-03**	1.12 (0.94, 1.32)
0.89 (0.81, 0.96)	**1.84E-02**	0.89 (0.81, 0.99)	9.53E-02	0.67 (0.56, 0.82)
0.90 (0.82, 0.98)	**3.66E-02**	0.94 (0.85, 1.04)	4.69E-01	0.73 (0.60, 0.88)
0.92 (0.85, 1.00)	8.48E-02	0.99 (0.90, 1.09)	9.16E-01	0.82 (0.68, 0.99)
0.94 (0.87, 1.02)	2.31E-01	0.96 (0.87, 1.05)	5.65E-01	0.83 (0.69, 1.00)
1.23 (1.14, 1.33)	**2.20E-06**	1.55 (1.42, 1.69)	**1.07E-20**	2.13 (1.80, 2.51)
0.97 (0.89, 1.05)	5.17E-01	0.88 (0.81, 0.96)	**1.29E-02**	0.83 (0.71, 0.97)
1.05 (0.97, 1.13)	3.60E-01	1.35 (1.24, 1.47)	**3.66E-10**	1.53 (1.31, 1.78)
0.92 (0.85, 1.00)	8.30E-02	0.83 (0.76, 0.91)	**7.97E-04**	0.73 (0.63, 0.85)
0.93 (0.86, 1.01)	1.38E-01	0.91 (0.83, 1.00)	1.38E-01	0.82 (0.70, 0.97)
0.97 (0.89, 1.05)	5.17E-01	1.10 (1.01, 1.21)	1.07E-01	1.03 (0.86, 1.22)
1.17 (1.08, 1.26)	**5.83E-04**	1.02 (0.93, 1.13)	7.90E-01	1.32 (1.12, 1.56)
1.06 (0.99, 1.14)	1.52E-01	1.14 (1.07, 1.21)	**7.97E-04**	1.12 (0.97, 1.29)
0.94 (0.86, 1.01)	1.84E-01	1.06 (0.97, 1.16)	4.51E-01	1.37 (1.20, 1.56)
1.01 (0.94, 1.09)	8.58E-01	0.86 (0.78, 0.95)	**1.03E-02**	0.76 (0.65, 0.90)
0.85 (0.79, 0.92)	**3.97E-04**	1.01 (0.92, 1.10)	9.52E-01	1.04 (0.89, 1.22)
0.86 (0.79, 0.92)	**5.34E-04**	0.97 (0.88, 1.06)	7.05E-01	0.92 (0.78, 1.09)
0.99 (0.92, 1.07)	8.58E-01	1.16 (1.06, 1.27)	**7.01E-03**	1.30 (1.11, 1.52)
0.90 (0.83, 0.97)	**2.57E-02**	0.99 (0.90, 1.09)	9.16E-01	0.97 (0.82, 1.14)
0.96 (0.89, 1.03)	3.71E-01	1.09 (0.99, 1.20)	1.81E-01	1.17 (1.01, 1.35)
0.83 (0.76, 0.90)	**6.38E-05**	0.85 (0.77, 0.94)	**8.29E-03**	0.85 (0.72, 1.01)
1.10 (1.02, 1.19)	**3.66E-02**	1.16 (1.11, 1.22)	**1.09E-08**	1.41 (1.24, 1.60)
0.96 (0.89, 1.03)	3.60E-01	1.05 (0.95, 1.15)	5.64E-01	1.20 (1.03, 1.40)
1.00 (0.93, 1.08)	9.68E-01	1.06 (0.99, 1.14)	2.68E-01	1.12 (0.98, 1.27)
0.97 (0.90, 1.06)	6.44E-01	0.89 (0.79, 1.00)	1.57E-01	0.64 (0.51, 0.80)
1.01 (0.93, 1.09)	9.07E-01	0.97 (0.86, 1.09)	7.54E-01	0.84 (0.66, 1.07)
1.03 (0.95, 1.11)	6.08E-01	1.05 (1.00, 1.10)	1.19E-01	1.05 (0.88, 1.26)
0.86 (0.79, 0.92)	**3.75E-04**	0.79 (0.73, 0.85)	**8.59E-08**	0.60 (0.53, 0.67)
1.17 (1.08, 1.26)	**4.96E-04**	1.33 (1.22, 1.45)	**1.72E-09**	1.34 (1.16, 1.56)

status, alcohol consumption, and self-rated health status. P values are corrected for mult

; VLDL, Very Low Density Lipoprotein; IDL, Intermediate Density Lipoprotein..

	Cerebrovascular diseases mortality			
	Age < 60y		Age >= 60y	
**P value**	**Adjusted HR (95% CI)**	**P value**	**Adjusted HR (95% CI)**	**P value**
4.12E-01	0.89 (0.80, 0.98)	5.67E-02	0.92 (0.70, 1.19)	9.71E-01
4.21E-01	0.89 (0.81, 0.98)	6.19E-02	0.92 (0.70, 1.20)	9.71E-01
4.47E-01	0.90 (0.82, 1.00)	1.04E-01	0.91 (0.70, 1.19)	9.71E-01
7.08E-01	0.92 (0.84, 1.01)	1.86E-01	0.94 (0.72, 1.22)	9.71E-01
9.60E-01	0.93 (0.84, 1.02)	2.14E-01	0.95 (0.73, 1.24)	9.71E-01
4.15E-01	0.91 (0.83, 1.01)	1.42E-01	0.91 (0.70, 1.19)	9.71E-01
3.54E-01	0.88 (0.80, 0.97)	**4.09E-02**	0.91 (0.70, 1.19)	9.71E-01
3.08E-01	0.91 (0.83, 1.00)	1.34E-01	0.94 (0.72, 1.22)	9.71E-01
3.32E-01	0.91 (0.83, 1.01)	1.35E-01	0.94 (0.72, 1.22)	9.71E-01
3.65E-01	0.91 (0.82, 1.00)	1.21E-01	0.93 (0.71, 1.21)	9.71E-01
5.91E-01	0.92 (0.84, 1.02)	2.06E-01	0.95 (0.73, 1.24)	9.71E-01
6.45E-01	0.93 (0.85, 1.03)	2.45E-01	0.96 (0.74, 1.25)	9.71E-01
4.59E-01	0.91 (0.83, 1.01)	1.47E-01	0.94 (0.72, 1.22)	9.71E-01
2.33E-01	0.91 (0.82, 1.00)	1.25E-01	0.93 (0.72, 1.22)	9.71E-01
4.66E-01	0.92 (0.84, 1.02)	2.06E-01	0.96 (0.74, 1.25)	9.71E-01
4.66E-01	0.92 (0.84, 1.02)	2.06E-01	0.96 (0.74, 1.25)	9.71E-01
4.56E-01	0.92 (0.84, 1.02)	2.06E-01	0.96 (0.74, 1.25)	9.71E-01
4.59E-01	0.92 (0.83, 1.01)	1.70E-01	0.95 (0.73, 1.24)	9.71E-01
4.66E-01	0.91 (0.82, 1.00)	1.17E-01	0.94 (0.72, 1.23)	9.71E-01
4.56E-01	0.93 (0.84, 1.02)	2.33E-01	0.96 (0.74, 1.24)	9.71E-01
4.66E-01	0.93 (0.84, 1.02)	2.14E-01	0.96 (0.74, 1.25)	9.71E-01
6.34E-01	0.93 (0.84, 1.02)	2.33E-01	0.96 (0.74, 1.25)	9.71E-01
6.34E-01	0.93 (0.84, 1.02)	2.33E-01	0.96 (0.74, 1.25)	9.71E-01
5.91E-01	0.92 (0.84, 1.02)	2.06E-01	0.96 (0.74, 1.25)	9.71E-01
6.34E-01	0.93 (0.84, 1.02)	2.14E-01	0.96 (0.74, 1.25)	9.71E-01
6.34E-01	0.91 (0.83, 1.01)	1.46E-01	0.95 (0.72, 1.24)	9.71E-01
6.34E-01	0.94 (0.85, 1.04)	3.14E-01	0.97 (0.75, 1.26)	9.71E-01
6.45E-01	0.93 (0.85, 1.03)	2.56E-01	0.97 (0.75, 1.25)	9.71E-01
6.65E-01	0.94 (0.86, 1.04)	3.20E-01	0.97 (0.75, 1.27)	9.71E-01
7.57E-01	0.95 (0.86, 1.05)	4.08E-01	0.98 (0.75, 1.28)	9.71E-01
5.30E-01	0.93 (0.85, 1.03)	2.45E-01	0.95 (0.73, 1.24)	9.71E-01
4.42E-01	1.04 (0.94, 1.14)	5.55E-01	1.04 (0.80, 1.34)	9.71E-01
1.69E-01	1.06 (0.96, 1.16)	3.17E-01	1.05 (0.82, 1.36)	9.71E-01
9.73E-01	0.99 (0.90, 1.09)	9.05E-01	1.00 (0.77, 1.30)	9.97E-01
3.78E-01	0.92 (0.83, 1.01)	1.71E-01	0.96 (0.74, 1.25)	9.71E-01
6.15E-01	1.03 (0.94, 1.13)	5.86E-01	1.06 (0.83, 1.34)	9.71E-01
4.47E-01	1.05 (0.95, 1.15)	4.23E-01	1.07 (0.84, 1.35)	9.71E-01
9.84E-01	0.98 (0.89, 1.07)	6.85E-01	1.02 (0.80, 1.31)	9.71E-01
**1.31E-02**	1.12 (1.03, 1.23)	**3.55E-02**	1.12 (0.89, 1.41)	9.71E-01
**5.22E-03**	1.13 (1.03, 1.24)	**2.26E-02**	1.12 (0.89, 1.41)	9.71E-01

1.09E-01	1.10 (1.00, 1.20)	1.08E-01	1.11 (0.88, 1.39)	9.71E-01
3.72E-01	0.96 (0.87, 1.06)	5.34E-01	0.98 (0.76, 1.27)	9.71E-01
8.96E-01	0.96 (0.87, 1.05)	4.70E-01	1.04 (0.82, 1.32)	9.71E-01
9.67E-01	0.96 (0.87, 1.06)	4.83E-01	1.04 (0.82, 1.32)	9.71E-01
8.38E-01	0.94 (0.86, 1.04)	3.17E-01	1.03 (0.81, 1.31)	9.71E-01
9.60E-01	0.96 (0.87, 1.05)	4.60E-01	1.04 (0.82, 1.33)	9.71E-01
9.84E-01	0.94 (0.85, 1.04)	3.14E-01	1.03 (0.81, 1.32)	9.71E-01
7.72E-01	1.00 (0.91, 1.10)	9.86E-01	1.06 (0.84, 1.35)	9.71E-01
4.40E-01	1.03 (0.94, 1.13)	6.43E-01	1.02 (0.80, 1.29)	9.71E-01
7.50E-01	0.94 (0.85, 1.03)	2.90E-01	1.05 (0.83, 1.34)	9.71E-01
7.72E-01	0.94 (0.85, 1.03)	2.90E-01	1.06 (0.83, 1.35)	9.71E-01
5.80E-01	0.90 (0.82, 0.99)	8.26E-02	1.03 (0.80, 1.32)	9.71E-01
8.66E-01	0.94 (0.85, 1.03)	2.92E-01	1.06 (0.83, 1.36)	9.71E-01
8.56E-01	0.93 (0.85, 1.03)	2.42E-01	1.06 (0.83, 1.35)	9.71E-01
9.59E-01	0.96 (0.87, 1.06)	4.83E-01	1.08 (0.85, 1.37)	9.71E-01
6.34E-01	1.06 (0.97, 1.16)	3.16E-01	1.05 (0.83, 1.33)	9.71E-01
6.34E-01	0.92 (0.84, 1.02)	2.01E-01	1.06 (0.83, 1.36)	9.71E-01
6.42E-01	0.92 (0.84, 1.02)	1.92E-01	1.06 (0.83, 1.37)	9.71E-01
1.16E-01	0.87 (0.79, 0.95)	**6.11E-03**	0.99 (0.76, 1.29)	9.90E-01
8.62E-01	0.94 (0.85, 1.03)	2.73E-01	1.08 (0.84, 1.38)	9.71E-01
9.60E-01	0.94 (0.86, 1.04)	3.16E-01	1.08 (0.84, 1.38)	9.71E-01
5.06E-01	0.91 (0.83, 1.00)	1.17E-01	1.08 (0.85, 1.39)	9.71E-01
4.56E-01	1.05 (0.95, 1.14)	4.23E-01	1.05 (0.83, 1.34)	9.71E-01
7.08E-01	0.93 (0.84, 1.02)	2.10E-01	1.07 (0.84, 1.38)	9.71E-01
7.38E-01	0.93 (0.84, 1.02)	2.13E-01	1.08 (0.84, 1.38)	9.71E-01
8.48E-02	0.87 (0.80, 0.95)	**6.02E-03**	1.00 (0.77, 1.31)	9.92E-01
9.84E-01	0.95 (0.86, 1.05)	3.79E-01	1.10 (0.86, 1.40)	9.71E-01
9.38E-01	0.96 (0.87, 1.06)	4.74E-01	1.09 (0.85, 1.39)	9.71E-01
6.34E-01	0.92 (0.84, 1.01)	1.49E-01	1.12 (0.87, 1.43)	9.71E-01
5.78E-01	0.99 (0.90, 1.09)	9.19E-01	1.02 (0.79, 1.32)	9.71E-01
**4.93E-03**	1.22 (1.12, 1.33)	**3.12E-05**	1.19 (0.94, 1.50)	9.71E-01
**4.34E-03**	1.22 (1.12, 1.33)	**3.41E-05**	1.19 (0.95, 1.51)	9.71E-01
8.58E-02	1.19 (1.10, 1.30)	**3.58E-04**	1.14 (0.89, 1.44)	9.71E-01
**1.45E-04**	1.24 (1.13, 1.35)	**1.83E-05**	1.24 (0.99, 1.57)	6.12E-01
**2.18E-05**	1.26 (1.15, 1.37)	**5.00E-06**	1.27 (1.01, 1.60)	5.39E-01
**1.02E-02**	1.19 (1.09, 1.30)	**4.23E-04**	1.19 (0.94, 1.50)	9.71E-01
1.31E-01	1.15 (1.06, 1.25)	**4.54E-03**	1.16 (0.92, 1.46)	9.71E-01
6.73E-01	1.15 (1.05, 1.25)	**6.94E-03**	1.07 (0.83, 1.37)	9.71E-01
6.45E-01	1.15 (1.05, 1.25)	**6.69E-03**	1.07 (0.83, 1.37)	9.71E-01
7.08E-01	1.14 (1.04, 1.24)	**1.29E-02**	1.04 (0.81, 1.34)	9.71E-01
5.96E-01	1.15 (1.06, 1.26)	**6.02E-03**	1.08 (0.84, 1.39)	9.71E-01
6.45E-01	1.15 (1.05, 1.25)	**8.07E-03**	1.08 (0.84, 1.39)	9.71E-01
3.53E-01	1.17 (1.07, 1.28)	**1.97E-03**	1.11 (0.87, 1.42)	9.71E-01
7.52E-01	1.15 (1.05, 1.26)	**6.11E-03**	1.08 (0.85, 1.37)	9.71E-01
**3.62E-05**	0.85 (0.77, 0.93)	**3.36E-03**	0.73 (0.57, 0.94)	3.17E-01
**6.06E-05**	0.85 (0.78, 0.94)	**4.67E-03**	0.74 (0.57, 0.95)	3.17E-01
**4.89E-04**	0.89 (0.81, 0.98)	6.01E-02	0.77 (0.59, 0.99)	5.39E-01
**1.72E-04**	0.85 (0.78, 0.93)	**3.26E-03**	0.75 (0.59, 0.96)	3.26E-01

**1.29E-04**	0.84 (0.77, 0.92)	**1.26E-03**	0.75 (0.59, 0.95)	3.17E-01
**9.92E-04**	0.90 (0.82, 0.99)	7.72E-02	0.78 (0.60, 1.00)	5.80E-01
**8.67E-05**	0.81 (0.73, 0.89)	**2.18E-04**	0.76 (0.58, 1.00)	5.39E-01
**4.93E-09**	0.79 (0.73, 0.85)	**1.01E-07**	0.73 (0.57, 0.92)	3.17E-01
**5.32E-09**	0.79 (0.73, 0.85)	**1.44E-07**	0.73 (0.57, 0.93)	3.17E-01
**3.52E-06**	0.82 (0.74, 0.90)	**1.76E-04**	0.76 (0.60, 0.95)	3.17E-01
**1.33E-03**	0.87 (0.79, 0.95)	**6.94E-03**	0.85 (0.67, 1.09)	9.71E-01
**2.75E-02**	0.90 (0.82, 0.98)	**4.15E-02**	0.90 (0.71, 1.15)	9.71E-01
**5.15E-06**	0.85 (0.77, 0.93)	**2.23E-03**	0.79 (0.62, 1.00)	5.39E-01
**3.69E-03**	0.87 (0.79, 0.96)	**2.12E-02**	0.89 (0.69, 1.16)	9.71E-01
1.46E-01	1.00 (0.91, 1.11)	9.54E-01	1.01 (0.77, 1.32)	9.89E-01
**1.06E-02**	1.18 (1.12, 1.25)	**2.27E-07**	0.97 (0.74, 1.26)	9.71E-01
**5.02E-04**	1.18 (1.11, 1.24)	**3.00E-07**	0.99 (0.76, 1.28)	9.87E-01
4.39E-01	1.10 (1.01, 1.19)	7.72E-02	0.94 (0.73, 1.21)	9.71E-01
1.69E-01	0.83 (0.78, 0.89)	**5.00E-06**	1.03 (0.80, 1.32)	9.71E-01
3.54E-01	0.89 (0.81, 0.97)	**2.23E-02**	0.94 (0.73, 1.20)	9.71E-01
**5.02E-04**	1.14 (1.07, 1.21)	**3.74E-04**	1.06 (0.82, 1.38)	9.71E-01
**1.38E-04**	1.23 (1.14, 1.32)	**1.20E-06**	1.08 (0.85, 1.36)	9.71E-01
9.92E-01	0.95 (0.86, 1.04)	3.45E-01	0.98 (0.74, 1.29)	9.71E-01
6.74E-02	0.95 (0.86, 1.05)	3.97E-01	0.99 (0.76, 1.29)	9.87E-01
1.74E-01	0.97 (0.88, 1.06)	5.59E-01	0.98 (0.76, 1.26)	9.71E-01
6.45E-01	0.94 (0.86, 1.03)	3.14E-01	1.01 (0.79, 1.31)	9.77E-01
9.71E-01	0.93 (0.84, 1.03)	2.45E-01	0.96 (0.74, 1.25)	9.71E-01
5.15E-01	0.99 (0.90, 1.09)	8.58E-01	1.06 (0.81, 1.39)	9.71E-01
4.94E-01	1.06 (0.96, 1.16)	3.20E-01	1.00 (0.77, 1.28)	9.92E-01
**3.74E-03**	0.91 (0.82, 1.00)	1.08E-01	0.79 (0.61, 1.02)	6.60E-01
4.59E-01	1.00 (0.90, 1.09)	9.36E-01	0.96 (0.75, 1.25)	9.71E-01
5.75E-01	0.97 (0.89, 1.07)	6.70E-01	0.94 (0.72, 1.22)	9.71E-01
7.39E-01	1.06 (0.96, 1.17)	3.17E-01	1.07 (0.83, 1.40)	9.71E-01
2.64E-01	1.02 (0.93, 1.12)	7.15E-01	1.07 (0.83, 1.39)	9.71E-01
**8.50E-03**	0.91 (0.83, 1.00)	1.18E-01	0.81 (0.61, 1.07)	9.71E-01
**9.52E-04**	1.19 (1.08, 1.30)	**2.23E-03**	1.08 (0.84, 1.39)	9.71E-01
**1.08E-03**	1.16 (1.05, 1.27)	**9.13E-03**	1.08 (0.84, 1.38)	9.71E-01
**1.56E-02**	1.26 (1.16, 1.37)	**2.46E-06**	1.08 (0.85, 1.38)	9.71E-01
**9.35E-03**	0.87 (0.79, 0.95)	**1.06E-02**	0.97 (0.76, 1.24)	9.71E-01
**1.07E-02**	0.87 (0.80, 0.94)	**2.23E-03**	0.90 (0.73, 1.11)	9.71E-01
**1.45E-07**	1.29 (1.16, 1.43)	**2.06E-05**	1.21 (0.93, 1.56)	9.71E-01
**4.37E-07**	1.25 (1.13, 1.38)	**8.86E-05**	1.16 (0.90, 1.49)	9.71E-01
**1.08E-03**	1.17 (1.06, 1.29)	**8.26E-03**	1.22 (0.93, 1.60)	9.71E-01
**4.25E-03**	0.91 (0.83, 1.00)	1.34E-01	0.92 (0.71, 1.18)	9.71E-01
3.73E-01	0.93 (0.85, 1.02)	2.09E-01	0.94 (0.73, 1.21)	9.71E-01
3.11E-01	0.97 (0.89, 1.06)	6.24E-01	1.03 (0.81, 1.31)	9.71E-01
7.89E-01	0.90 (0.83, 0.98)	5.83E-02	0.99 (0.79, 1.26)	9.92E-01
1.33E-01	1.08 (0.98, 1.19)	2.06E-01	1.06 (0.82, 1.36)	9.71E-01
4.66E-01	1.05 (0.96, 1.14)	3.45E-01	0.99 (0.77, 1.26)	9.77E-01
5.98E-01	0.94 (0.85, 1.03)	3.02E-01	0.86 (0.65, 1.14)	9.71E-01
8.05E-01	0.98 (0.90, 1.07)	6.87E-01	1.09 (0.83, 1.44)	9.71E-01
8.92E-01	0.95 (0.88, 1.03)	3.14E-01	1.04 (0.79, 1.36)	9.71E-01

1.81E-01	1.11 (1.00, 1.22)	1.06E-01	1.15 (0.88, 1.51)	9.71E-01
9.80E-01	1.08 (1.00, 1.16)	1.34E-01	0.98 (0.76, 1.27)	9.71E-01
2.64E-01	0.94 (0.85, 1.04)	3.14E-01	0.87 (0.66, 1.14)	9.71E-01
4.59E-01	1.00 (0.91, 1.10)	9.98E-01	1.10 (0.85, 1.44)	9.71E-01
6.20E-01	0.99 (0.90, 1.08)	8.58E-01	1.07 (0.83, 1.38)	9.71E-01
8.76E-01	1.04 (0.95, 1.14)	4.74E-01	1.02 (0.80, 1.30)	9.71E-01
9.84E-01	1.11 (1.02, 1.20)	**4.15E-02**	0.99 (0.77, 1.28)	9.92E-01
1.74E-01	0.94 (0.85, 1.04)	3.14E-01	0.88 (0.68, 1.15)	9.71E-01
3.36E-01	1.02 (0.93, 1.12)	6.92E-01	1.11 (0.85, 1.44)	9.71E-01
4.42E-01	1.01 (0.92, 1.11)	8.48E-01	1.07 (0.83, 1.39)	9.71E-01
9.37E-01	1.03 (0.94, 1.13)	5.51E-01	1.07 (0.84, 1.36)	9.71E-01
8.21E-01	1.05 (0.96, 1.15)	3.45E-01	0.97 (0.75, 1.25)	9.71E-01
**1.41E-02**	0.95 (0.86, 1.04)	3.27E-01	0.79 (0.63, 1.00)	5.39E-01
**7.79E-05**	1.10 (1.00, 1.21)	1.34E-01	1.38 (1.06, 1.78)	3.17E-01
**6.50E-05**	1.11 (1.01, 1.22)	9.49E-02	1.37 (1.09, 1.73)	3.17E-01
1.31E-01	1.01 (0.92, 1.11)	9.19E-01	1.08 (0.83, 1.41)	9.71E-01
**3.65E-02**	0.94 (0.85, 1.03)	2.90E-01	0.95 (0.73, 1.22)	9.71E-01
1.33E-01	0.86 (0.78, 0.95)	**9.43E-03**	0.94 (0.73, 1.23)	9.71E-01
**2.75E-02**	1.16 (1.04, 1.28)	**1.79E-02**	1.10 (0.83, 1.45)	9.71E-01
4.21E-01	1.06 (0.96, 1.17)	3.17E-01	1.05 (0.80, 1.38)	9.71E-01
**3.48E-04**	1.27 (1.14, 1.42)	**1.86E-04**	1.15 (0.88, 1.52)	9.71E-01
1.28E-01	1.00 (0.91, 1.10)	9.89E-01	0.92 (0.71, 1.20)	9.71E-01
**2.33E-04**	1.23 (1.16, 1.29)	**1.74E-11**	1.27 (1.10, 1.47)	1.49E-01
6.73E-01	0.88 (0.82, 0.95)	**6.94E-03**	0.86 (0.71, 1.04)	9.71E-01
7.91E-01	0.87 (0.81, 0.94)	**2.47E-03**	0.87 (0.72, 1.04)	9.71E-01
4.56E-01	0.98 (0.89, 1.07)	6.61E-01	0.89 (0.70, 1.14)	9.71E-01
8.29E-02	0.93 (0.84, 1.02)	2.15E-01	0.97 (0.75, 1.25)	9.71E-01
7.72E-01	0.97 (0.89, 1.07)	6.42E-01	0.90 (0.71, 1.15)	9.71E-01
5.98E-01	1.03 (0.94, 1.13)	5.88E-01	1.09 (0.85, 1.41)	9.71E-01
6.42E-01	1.02 (0.93, 1.12)	7.15E-01	1.08 (0.84, 1.39)	9.71E-01
8.60E-01	1.07 (0.98, 1.17)	2.36E-01	1.02 (0.79, 1.32)	9.71E-01
3.17E-01	0.97 (0.88, 1.07)	6.31E-01	1.01 (0.79, 1.30)	9.77E-01
4.12E-01	0.92 (0.84, 1.01)	1.78E-01	1.00 (0.79, 1.27)	9.97E-01
6.42E-01	1.07 (0.98, 1.17)	2.42E-01	0.87 (0.67, 1.13)	9.71E-01
**7.93E-03**	1.25 (1.14, 1.37)	**1.19E-05**	1.15 (0.90, 1.47)	9.71E-01
8.62E-01	0.98 (0.89, 1.08)	7.93E-01	1.05 (0.82, 1.34)	9.71E-01
6.42E-01	0.99 (0.90, 1.09)	8.58E-01	1.02 (0.79, 1.32)	9.71E-01
7.16E-01	0.97 (0.88, 1.07)	6.42E-01	1.04 (0.81, 1.33)	9.71E-01
9.84E-01	0.95 (0.86, 1.04)	3.32E-01	1.06 (0.83, 1.36)	9.71E-01
9.84E-01	1.07 (0.98, 1.17)	2.27E-01	1.00 (0.77, 1.29)	9.92E-01
7.72E-01	1.05 (0.96, 1.15)	3.68E-01	0.97 (0.75, 1.26)	9.71E-01
**6.36E-04**	1.24 (1.14, 1.36)	**1.99E-05**	1.19 (0.94, 1.52)	9.71E-01
**1.37E-03**	0.83 (0.75, 0.92)	**1.31E-03**	0.92 (0.71, 1.19)	9.71E-01
1.67E-01	0.83 (0.75, 0.92)	**1.58E-03**	1.00 (0.77, 1.29)	9.92E-01
5.15E-01	0.94 (0.86, 1.04)	3.17E-01	0.97 (0.75, 1.26)	9.71E-01
6.20E-01	0.93 (0.85, 1.03)	2.45E-01	0.97 (0.75, 1.25)	9.71E-01
6.34E-01	1.05 (0.96, 1.15)	3.27E-01	1.05 (0.82, 1.33)	9.71E-01
**3.54E-02**	0.96 (0.88, 1.06)	5.34E-01	0.93 (0.72, 1.20)	9.71E-01

3.65E-01	0.91 (0.83, 1.00)	1.27E-01	0.92 (0.71, 1.20)	9.71E-01
6.34E-01	0.96 (0.87, 1.05)	4.62E-01	0.99 (0.77, 1.28)	9.92E-01
**1.57E-04**	0.79 (0.72, 0.87)	**1.19E-05**	0.87 (0.67, 1.13)	9.71E-01
**2.95E-04**	0.77 (0.70, 0.84)	**6.13E-07**	0.91 (0.70, 1.19)	9.71E-01
1.51E-01	0.88 (0.80, 0.97)	**2.26E-02**	0.95 (0.73, 1.23)	9.71E-01
9.84E-01	1.06 (0.97, 1.16)	3.16E-01	1.00 (0.77, 1.28)	9.92E-01
8.60E-01	0.92 (0.84, 1.02)	2.09E-01	1.02 (0.79, 1.32)	9.71E-01
6.91E-01	0.90 (0.82, 1.00)	1.03E-01	1.03 (0.80, 1.33)	9.71E-01
**3.93E-02**	0.84 (0.76, 0.93)	**2.66E-03**	0.92 (0.70, 1.21)	9.71E-01
**2.92E-09**	0.75 (0.70, 0.81)	**4.26E-13**	0.82 (0.63, 1.06)	9.71E-01
**7.97E-06**	1.19 (1.09, 1.30)	**8.18E-04**	1.15 (0.90, 1.48)	9.71E-01
**8.25E-03**	0.83 (0.75, 0.91)	**4.23E-04**	0.97 (0.74, 1.28)	9.71E-01
4.12E-01	1.00 (0.91, 1.11)	9.36E-01	1.02 (0.78, 1.34)	9.71E-01
**4.91E-04**	0.79 (0.71, 0.87)	**2.38E-05**	0.92 (0.69, 1.22)	9.71E-01
**5.80E-03**	0.81 (0.73, 0.90)	**3.40E-04**	0.93 (0.70, 1.24)	9.71E-01
1.09E-01	0.89 (0.81, 0.98)	5.78E-02	0.91 (0.70, 1.19)	9.71E-01
1.31E-01	0.83 (0.75, 0.92)	**1.91E-03**	0.93 (0.71, 1.21)	9.71E-01
**1.06E-16**	1.45 (1.32, 1.59)	**2.86E-13**	1.27 (0.99, 1.62)	6.11E-01
5.76E-02	0.89 (0.83, 0.97)	**1.67E-02**	1.09 (0.82, 1.44)	9.71E-01
**2.79E-06**	1.21 (1.11, 1.33)	**1.86E-04**	1.14 (0.88, 1.47)	9.71E-01
**5.74E-04**	0.86 (0.78, 0.94)	**3.52E-03**	0.99 (0.76, 1.28)	9.77E-01
6.38E-02	0.90 (0.82, 0.99)	7.17E-02	1.03 (0.78, 1.35)	9.71E-01
8.60E-01	1.10 (1.01, 1.21)	8.23E-02	0.91 (0.70, 1.18)	9.71E-01
**4.75E-03**	1.04 (0.94, 1.14)	5.32E-01	1.09 (0.84, 1.41)	9.71E-01
2.89E-01	1.19 (1.12, 1.27)	**2.27E-07**	1.10 (0.89, 1.36)	9.71E-01
**3.59E-05**	1.05 (0.97, 1.15)	3.19E-01	1.08 (0.85, 1.37)	9.71E-01
**6.83E-03**	0.87 (0.78, 0.96)	**1.52E-02**	0.74 (0.58, 0.96)	3.56E-01
7.38E-01	0.97 (0.89, 1.07)	6.39E-01	0.97 (0.75, 1.24)	9.71E-01
5.59E-01	1.01 (0.92, 1.10)	9.24E-01	1.21 (0.94, 1.55)	9.71E-01
**5.22E-03**	1.09 (0.99, 1.19)	1.36E-01	1.52 (1.20, 1.93)	1.40E-01
8.11E-01	0.90 (0.81, 0.99)	7.72E-02	1.04 (0.81, 1.32)	9.71E-01
1.23E-01	0.94 (0.85, 1.04)	3.16E-01	1.15 (0.92, 1.45)	9.71E-01
1.58E-01	0.75 (0.68, 0.83)	**1.10E-06**	0.95 (0.73, 1.22)	9.71E-01
**1.69E-06**	1.15 (1.10, 1.20)	**8.45E-08**	1.23 (0.99, 1.54)	6.11E-01
6.06E-02	1.11 (1.01, 1.22)	6.21E-02	1.14 (0.91, 1.44)	9.71E-01
2.16E-01	1.13 (1.06, 1.19)	**3.14E-04**	1.17 (1.03, 1.34)	3.17E-01
**5.02E-04**	1.01 (0.92, 1.10)	9.19E-01	0.92 (0.70, 1.22)	9.71E-01
3.52E-01	1.02 (0.93, 1.13)	6.85E-01	0.95 (0.70, 1.30)	9.71E-01
7.08E-01	1.07 (1.03, 1.11)	**2.23E-03**	1.22 (1.04, 1.43)	3.17E-01
**1.59E-15**	0.78 (0.72, 0.84)	**1.15E-08**	0.72 (0.57, 0.90)	3.01E-01
**7.23E-04**	1.20 (1.10, 1.30)	**3.74E-04**	1.07 (0.82, 1.39)	9.71E-01

Cancer mortality				
Age < 60y		Age >= 60y		Age < 60y
**Adjusted HR (95% CI)**	**P value**	**Adjusted HR (95% CI)**	**P value**	**Adjusted HR (95% CI**
1.00 (0.89, 1.12)	1.00E+00	0.85 (0.76, 0.96)	**4.73E-02**	1.01 (0.85, 1.20)
1.00 (0.90, 1.12)	9.99E-01	0.86 (0.76, 0.97)	5.37E-02	1.01 (0.85, 1.20)
1.00 (0.89, 1.12)	9.99E-01	0.87 (0.77, 0.98)	7.16E-02	1.03 (0.86, 1.22)
1.03 (0.92, 1.14)	9.30E-01	0.91 (0.81, 1.02)	2.56E-01	1.05 (0.89, 1.24)
1.04 (0.94, 1.16)	8.90E-01	0.93 (0.83, 1.04)	3.58E-01	1.04 (0.88, 1.23)
1.01 (0.91, 1.13)	9.60E-01	0.90 (0.80, 1.01)	1.90E-01	1.06 (0.90, 1.25)
1.00 (0.89, 1.12)	9.99E-01	0.84 (0.74, 0.95)	**2.63E-02**	1.00 (0.84, 1.19)
1.04 (0.93, 1.16)	8.90E-01	0.91 (0.81, 1.02)	2.49E-01	1.04 (0.88, 1.23)
1.04 (0.93, 1.16)	8.90E-01	0.91 (0.81, 1.02)	2.54E-01	1.04 (0.88, 1.23)
1.04 (0.93, 1.16)	8.90E-01	0.91 (0.81, 1.03)	2.67E-01	1.06 (0.89, 1.25)
1.05 (0.94, 1.17)	8.14E-01	0.93 (0.83, 1.04)	3.58E-01	1.04 (0.88, 1.24)
1.05 (0.94, 1.17)	8.14E-01	0.93 (0.83, 1.04)	3.61E-01	1.03 (0.87, 1.22)
1.05 (0.94, 1.17)	8.14E-01	0.93 (0.83, 1.04)	3.58E-01	1.06 (0.89, 1.25)
1.04 (0.93, 1.16)	8.90E-01	0.91 (0.81, 1.02)	2.32E-01	1.04 (0.88, 1.23)
0.99 (0.89, 1.10)	9.61E-01	0.90 (0.81, 1.01)	1.81E-01	1.03 (0.87, 1.22)
0.99 (0.89, 1.11)	9.69E-01	0.90 (0.81, 1.01)	1.81E-01	1.03 (0.87, 1.22)
0.99 (0.89, 1.11)	9.87E-01	0.90 (0.81, 1.01)	1.81E-01	1.04 (0.87, 1.23)
1.01 (0.90, 1.12)	9.74E-01	0.91 (0.81, 1.02)	2.26E-01	1.02 (0.86, 1.21)
1.02 (0.92, 1.14)	9.40E-01	0.91 (0.82, 1.02)	2.32E-01	0.99 (0.83, 1.17)
1.00 (0.89, 1.11)	9.93E-01	0.91 (0.81, 1.02)	2.30E-01	1.05 (0.89, 1.24)
0.98 (0.88, 1.10)	9.49E-01	0.90 (0.80, 1.01)	1.78E-01	1.03 (0.87, 1.22)
0.98 (0.88, 1.09)	9.40E-01	0.90 (0.81, 1.00)	1.63E-01	0.99 (0.83, 1.18)
0.98 (0.88, 1.10)	9.49E-01	0.90 (0.81, 1.00)	1.71E-01	0.99 (0.83, 1.18)
0.99 (0.88, 1.10)	9.49E-01	0.90 (0.81, 1.00)	1.60E-01	0.99 (0.83, 1.18)
1.01 (0.91, 1.13)	9.60E-01	0.92 (0.83, 1.03)	2.73E-01	0.98 (0.82, 1.17)
1.04 (0.93, 1.15)	9.11E-01	0.93 (0.83, 1.04)	3.55E-01	0.95 (0.79, 1.14)
0.99 (0.89, 1.10)	9.60E-01	0.91 (0.82, 1.02)	2.30E-01	1.01 (0.85, 1.21)
0.97 (0.87, 1.08)	9.30E-01	0.89 (0.80, 1.00)	1.38E-01	1.00 (0.84, 1.19)
0.98 (0.88, 1.09)	9.40E-01	0.93 (0.83, 1.03)	3.35E-01	0.95 (0.79, 1.14)
0.97 (0.87, 1.08)	9.30E-01	0.94 (0.84, 1.04)	3.80E-01	0.95 (0.79, 1.13)
0.97 (0.87, 1.09)	9.30E-01	0.92 (0.83, 1.03)	2.79E-01	0.94 (0.78, 1.13)
0.94 (0.84, 1.05)	6.37E-01	1.00 (0.90, 1.11)	1.00E+00	0.91 (0.76, 1.09)
0.93 (0.84, 1.04)	6.02E-01	1.02 (0.92, 1.13)	8.00E-01	0.89 (0.74, 1.06)
0.95 (0.85, 1.06)	8.14E-01	0.96 (0.87, 1.07)	6.48E-01	0.96 (0.80, 1.15)
1.00 (0.89, 1.11)	9.97E-01	0.91 (0.81, 1.01)	1.81E-01	0.98 (0.82, 1.17)
0.97 (0.87, 1.08)	9.30E-01	0.99 (0.90, 1.10)	9.64E-01	0.95 (0.79, 1.14)
0.96 (0.85, 1.07)	8.61E-01	1.00 (0.91, 1.11)	9.88E-01	0.95 (0.79, 1.14)
1.01 (0.91, 1.13)	9.60E-01	0.99 (0.89, 1.10)	9.23E-01	0.92 (0.76, 1.10)
0.90 (0.81, 1.01)	2.87E-01	1.04 (0.94, 1.15)	6.27E-01	0.96 (0.80, 1.14)
0.90 (0.81, 1.01)	2.87E-01	1.03 (0.94, 1.14)	6.52E-01	0.96 (0.81, 1.15)

0.91 (0.82, 1.02)	3.61E-01	1.04 (0.95, 1.15)	5.82E-01	0.96 (0.80, 1.14)
1.03 (0.92, 1.14)	9.31E-01	0.95 (0.86, 1.05)	5.22E-01	1.00 (0.83, 1.19)
1.03 (0.93, 1.15)	9.30E-01	0.98 (0.89, 1.09)	8.50E-01	0.92 (0.77, 1.11)
1.03 (0.92, 1.15)	9.30E-01	0.99 (0.89, 1.09)	8.93E-01	0.91 (0.76, 1.10)
1.04 (0.93, 1.16)	9.07E-01	0.97 (0.88, 1.08)	7.13E-01	0.87 (0.73, 1.05)
1.02 (0.92, 1.14)	9.40E-01	0.99 (0.90, 1.09)	9.23E-01	0.89 (0.74, 1.06)
1.02 (0.92, 1.14)	9.40E-01	0.98 (0.89, 1.08)	8.00E-01	0.88 (0.73, 1.06)
1.02 (0.92, 1.14)	9.40E-01	1.02 (0.92, 1.12)	8.61E-01	0.91 (0.76, 1.08)
1.07 (0.96, 1.19)	5.68E-01	0.99 (0.89, 1.09)	9.19E-01	1.13 (0.97, 1.33)
1.02 (0.92, 1.14)	9.40E-01	0.96 (0.87, 1.07)	6.27E-01	0.87 (0.73, 1.05)
1.02 (0.91, 1.14)	9.40E-01	0.96 (0.87, 1.07)	6.31E-01	0.87 (0.72, 1.04)
1.03 (0.92, 1.15)	9.30E-01	0.95 (0.86, 1.06)	5.39E-01	0.84 (0.70, 1.01)
1.01 (0.90, 1.13)	9.69E-01	0.96 (0.87, 1.07)	6.31E-01	0.85 (0.71, 1.02)
1.00 (0.90, 1.12)	9.93E-01	0.95 (0.86, 1.06)	5.50E-01	0.85 (0.70, 1.02)
1.02 (0.92, 1.14)	9.40E-01	0.99 (0.89, 1.09)	9.23E-01	0.87 (0.73, 1.04)
1.08 (0.97, 1.20)	5.10E-01	1.01 (0.92, 1.12)	9.19E-01	1.16 (0.99, 1.36)
0.99 (0.88, 1.10)	9.60E-01	0.94 (0.84, 1.04)	3.80E-01	0.83 (0.69, 1.00)
0.98 (0.88, 1.10)	9.49E-01	0.94 (0.84, 1.04)	3.83E-01	0.83 (0.69, 0.99)
1.02 (0.91, 1.14)	9.49E-01	0.90 (0.80, 1.00)	1.58E-01	0.83 (0.70, 0.99)
0.97 (0.87, 1.08)	9.30E-01	0.94 (0.85, 1.05)	4.69E-01	0.82 (0.68, 0.98)
0.97 (0.87, 1.08)	9.30E-01	0.95 (0.85, 1.05)	4.78E-01	0.81 (0.68, 0.98)
0.98 (0.88, 1.10)	9.49E-01	0.94 (0.85, 1.05)	4.27E-01	0.84 (0.71, 0.99)
1.07 (0.95, 1.19)	6.45E-01	1.00 (0.90, 1.10)	9.77E-01	1.12 (0.95, 1.33)
0.96 (0.86, 1.08)	9.07E-01	0.93 (0.84, 1.04)	3.55E-01	0.82 (0.69, 0.98)
0.96 (0.86, 1.07)	8.90E-01	0.93 (0.84, 1.04)	3.58E-01	0.82 (0.69, 0.98)
1.00 (0.89, 1.12)	9.99E-01	0.89 (0.80, 1.00)	1.38E-01	0.84 (0.72, 0.99)
0.95 (0.85, 1.06)	7.62E-01	0.95 (0.85, 1.05)	4.92E-01	0.81 (0.68, 0.97)
0.94 (0.85, 1.06)	7.25E-01	0.95 (0.85, 1.05)	5.12E-01	0.81 (0.68, 0.97)
0.95 (0.85, 1.06)	8.04E-01	0.93 (0.84, 1.04)	3.58E-01	0.84 (0.71, 0.99)
1.02 (0.91, 1.14)	9.49E-01	0.95 (0.86, 1.06)	5.28E-01	1.03 (0.86, 1.23)
1.01 (0.91, 1.12)	9.61E-01	1.22 (1.12, 1.34)	**4.15E-04**	1.24 (1.06, 1.46)
1.01 (0.90, 1.12)	9.93E-01	1.22 (1.12, 1.34)	**4.15E-04**	1.24 (1.05, 1.45)
1.08 (0.97, 1.20)	5.34E-01	1.24 (1.13, 1.36)	**2.07E-04**	1.23 (1.05, 1.45)
0.93 (0.84, 1.04)	5.66E-01	1.20 (1.09, 1.31)	**2.30E-03**	1.21 (1.02, 1.42)
0.91 (0.81, 1.01)	2.87E-01	1.18 (1.07, 1.30)	**5.00E-03**	1.20 (1.01, 1.41)
1.01 (0.91, 1.12)	9.61E-01	1.23 (1.12, 1.35)	**4.15E-04**	1.22 (1.04, 1.43)
1.02 (0.91, 1.13)	9.49E-01	1.09 (0.98, 1.20)	2.30E-01	1.26 (1.09, 1.47)
1.05 (0.94, 1.17)	8.14E-01	1.22 (1.11, 1.34)	**8.70E-04**	1.21 (1.03, 1.43)
1.04 (0.94, 1.16)	8.46E-01	1.22 (1.11, 1.34)	**8.09E-04**	1.21 (1.03, 1.42)
1.03 (0.92, 1.15)	9.30E-01	1.21 (1.10, 1.33)	**1.06E-03**	1.18 (1.00, 1.39)
1.05 (0.94, 1.17)	8.14E-01	1.22 (1.11, 1.34)	**6.21E-04**	1.22 (1.03, 1.43)
1.05 (0.95, 1.17)	7.72E-01	1.22 (1.11, 1.34)	**7.34E-04**	1.22 (1.04, 1.44)
1.03 (0.92, 1.15)	9.30E-01	1.23 (1.12, 1.35)	**4.15E-04**	1.20 (1.02, 1.41)
1.15 (1.03, 1.28)	7.01E-02	1.10 (1.00, 1.21)	1.71E-01	1.29 (1.11, 1.51)
1.03 (0.92, 1.15)	9.30E-01	1.00 (0.91, 1.11)	9.88E-01	0.93 (0.78, 1.12)
1.02 (0.92, 1.14)	9.40E-01	1.01 (0.91, 1.12)	9.23E-01	0.93 (0.78, 1.12)
1.02 (0.92, 1.14)	9.40E-01	1.01 (0.91, 1.12)	9.23E-01	0.99 (0.82, 1.18)
1.00 (0.90, 1.12)	9.99E-01	1.03 (0.93, 1.14)	6.98E-01	0.91 (0.76, 1.08)

1.00 (0.89, 1.12)	1.00E+00	1.03 (0.93, 1.14)	7.45E-01	0.89 (0.75, 1.07)
1.01 (0.90, 1.12)	9.93E-01	1.05 (0.95, 1.16)	5.07E-01	0.97 (0.81, 1.16)
1.14 (1.03, 1.26)	7.01E-02	0.88 (0.79, 0.98)	7.57E-02	0.93 (0.77, 1.11)
1.04 (0.93, 1.17)	8.90E-01	0.87 (0.78, 0.96)	**4.04E-02**	0.80 (0.68, 0.93)
1.04 (0.92, 1.16)	9.30E-01	0.88 (0.79, 0.97)	5.41E-02	0.79 (0.68, 0.93)
1.01 (0.90, 1.13)	9.61E-01	0.89 (0.80, 0.99)	9.71E-02	0.88 (0.73, 1.05)
1.02 (0.92, 1.14)	9.40E-01	0.96 (0.87, 1.06)	6.16E-01	0.80 (0.68, 0.94)
1.02 (0.92, 1.14)	9.40E-01	0.97 (0.88, 1.08)	7.13E-01	0.82 (0.70, 0.96)
1.00 (0.89, 1.12)	9.97E-01	0.94 (0.85, 1.05)	4.62E-01	0.88 (0.74, 1.06)
1.09 (0.99, 1.21)	3.30E-01	0.85 (0.77, 0.95)	**2.09E-02**	0.99 (0.83, 1.18)
0.81 (0.74, 0.89)	**2.87E-04**	0.91 (0.82, 1.00)	1.71E-01	1.07 (0.88, 1.31)
1.14 (1.04, 1.26)	**4.73E-02**	1.18 (1.08, 1.29)	**2.01E-03**	1.17 (1.02, 1.33)
1.16 (1.06, 1.27)	**1.05E-02**	1.17 (1.06, 1.28)	**8.95E-03**	1.13 (0.97, 1.31)
0.99 (0.88, 1.12)	9.78E-01	1.14 (1.03, 1.26)	5.43E-02	1.18 (1.03, 1.35)
0.98 (0.87, 1.11)	9.40E-01	0.88 (0.80, 0.97)	5.37E-02	0.84 (0.72, 0.97)
0.97 (0.86, 1.09)	9.30E-01	0.90 (0.82, 0.99)	1.22E-01	1.02 (0.84, 1.23)
1.06 (0.96, 1.17)	6.45E-01	1.07 (0.98, 1.18)	2.54E-01	1.12 (0.99, 1.27)
1.07 (0.96, 1.20)	6.26E-01	1.07 (0.98, 1.16)	2.73E-01	1.08 (0.91, 1.29)
1.01 (0.91, 1.14)	9.60E-01	1.03 (0.92, 1.16)	7.13E-01	1.13 (0.97, 1.32)
0.97 (0.86, 1.09)	9.30E-01	0.99 (0.89, 1.10)	9.05E-01	0.88 (0.73, 1.06)
1.04 (0.92, 1.18)	8.90E-01	0.88 (0.80, 0.97)	**4.97E-02**	1.03 (0.86, 1.25)
1.19 (1.05, 1.36)	5.87E-02	0.96 (0.86, 1.07)	6.25E-01	0.95 (0.80, 1.13)
1.33 (1.19, 1.48)	**6.96E-06**	1.05 (0.95, 1.17)	5.38E-01	0.87 (0.72, 1.06)
0.92 (0.83, 1.03)	5.08E-01	0.91 (0.83, 1.00)	1.71E-01	1.05 (0.87, 1.26)
0.86 (0.75, 0.98)	1.22E-01	1.07 (0.96, 1.18)	3.66E-01	1.04 (0.87, 1.24)
1.18 (1.06, 1.30)	**1.42E-02**	1.03 (0.93, 1.13)	7.13E-01	0.91 (0.76, 1.10)
1.24 (1.11, 1.39)	**2.74E-03**	1.13 (1.02, 1.25)	7.16E-02	0.99 (0.83, 1.19)
1.24 (1.11, 1.39)	**1.36E-03**	1.13 (1.03, 1.25)	5.79E-02	0.95 (0.79, 1.14)
0.96 (0.86, 1.07)	8.90E-01	0.97 (0.88, 1.07)	6.90E-01	1.14 (0.94, 1.39)
0.80 (0.71, 0.89)	**1.30E-03**	0.89 (0.80, 0.98)	8.58E-02	1.02 (0.85, 1.23)
1.11 (1.00, 1.22)	2.47E-01	1.01 (0.91, 1.12)	9.23E-01	1.02 (0.86, 1.21)
0.92 (0.83, 1.02)	4.38E-01	1.17 (1.05, 1.29)	**1.89E-02**	0.95 (0.80, 1.12)
0.93 (0.83, 1.03)	5.34E-01	1.15 (1.04, 1.27)	**4.04E-02**	0.93 (0.78, 1.10)
0.87 (0.78, 0.98)	9.95E-02	1.24 (1.12, 1.37)	**4.15E-04**	1.12 (0.94, 1.32)
1.06 (0.95, 1.18)	6.72E-01	0.86 (0.77, 0.94)	**1.60E-02**	1.05 (0.88, 1.25)
1.17 (1.04, 1.31)	7.01E-02	1.00 (0.90, 1.10)	9.80E-01	0.93 (0.79, 1.09)
0.83 (0.75, 0.91)	**8.29E-04**	1.11 (1.01, 1.23)	1.32E-01	1.04 (0.87, 1.24)
0.83 (0.75, 0.91)	**2.24E-03**	1.08 (0.98, 1.20)	2.64E-01	1.04 (0.87, 1.24)
0.87 (0.79, 0.95)	**2.48E-02**	1.15 (1.03, 1.29)	5.82E-02	1.02 (0.86, 1.20)
1.10 (1.00, 1.21)	2.47E-01	0.92 (0.83, 1.02)	2.32E-01	1.01 (0.85, 1.20)
1.05 (0.94, 1.18)	8.04E-01	0.93 (0.84, 1.03)	3.50E-01	0.87 (0.74, 1.02)
0.93 (0.85, 1.03)	5.34E-01	1.02 (0.92, 1.13)	8.16E-01	0.88 (0.76, 1.02)
0.92 (0.83, 1.03)	4.84E-01	0.95 (0.87, 1.05)	5.22E-01	0.85 (0.73, 1.01)
0.98 (0.89, 1.09)	9.40E-01	1.11 (0.99, 1.24)	1.81E-01	0.97 (0.83, 1.14)
1.05 (0.95, 1.16)	7.57E-01	1.00 (0.90, 1.11)	1.00E+00	1.17 (1.02, 1.35)
1.05 (0.94, 1.16)	8.14E-01	0.96 (0.86, 1.07)	6.31E-01	1.05 (0.9, 1.23)
0.95 (0.86, 1.04)	6.45E-01	1.00 (0.90, 1.11)	9.96E-01	0.88 (0.77, 1.00)
0.94 (0.85, 1.04)	6.26E-01	0.96 (0.87, 1.06)	5.48E-01	0.86 (0.76, 0.98)

0.99 (0.89, 1.10)	9.63E-01	1.15 (1.03, 1.29)	5.41E-02	1.04 (0.87, 1.25)
1.06 (0.96, 1.16)	6.53E-01	1.03 (0.93, 1.14)	7.13E-01	1.18 (1.04, 1.33)
1.07 (0.97, 1.19)	5.34E-01	0.96 (0.86, 1.07)	6.16E-01	1.13 (0.97, 1.31)
0.93 (0.84, 1.02)	4.84E-01	1.01 (0.91, 1.12)	9.40E-01	0.85 (0.73, 0.98)
0.93 (0.84, 1.04)	5.50E-01	1.00 (0.90, 1.10)	9.77E-01	0.84 (0.73, 0.98)
1.03 (0.91, 1.15)	9.40E-01	1.04 (0.94, 1.16)	6.16E-01	1.18 (1.01, 1.39)
1.08 (0.97, 1.19)	5.00E-01	1.06 (0.95, 1.17)	4.81E-01	1.24 (1.09, 1.42)
1.10 (0.99, 1.22)	2.87E-01	0.98 (0.88, 1.08)	7.88E-01	1.15 (0.98, 1.35)
0.92 (0.83, 1.01)	3.34E-01	1.02 (0.92, 1.13)	8.50E-01	0.86 (0.74, 1.00)
0.92 (0.83, 1.02)	4.51E-01	1.01 (0.91, 1.12)	9.36E-01	0.85 (0.73, 0.99)
1.02 (0.91, 1.15)	9.40E-01	1.03 (0.93, 1.14)	7.13E-01	1.18 (1.00, 1.39)
1.06 (0.96, 1.18)	6.00E-01	1.00 (0.90, 1.10)	9.80E-01	1.18 (1.01, 1.36)
1.28 (1.15, 1.43)	**2.30E-04**	1.12 (1.01, 1.24)	9.95E-02	1.08 (0.91, 1.29)
0.77 (0.69, 0.84)	**3.77E-06**	0.92 (0.84, 1.02)	2.30E-01	0.88 (0.75, 1.05)
0.75 (0.67, 0.82)	**7.99E-07**	0.88 (0.80, 0.98)	5.82E-02	0.87 (0.74, 1.04)
0.92 (0.84, 1.01)	3.33E-01	1.11 (1.00, 1.23)	1.71E-01	0.96 (0.81, 1.14)
1.01 (0.92, 1.12)	9.49E-01	0.89 (0.79, 1.00)	1.38E-01	1.08 (0.93, 1.26)
0.93 (0.83, 1.04)	5.50E-01	0.85 (0.76, 0.94)	**1.60E-02**	0.73 (0.60, 0.88)
1.00 (0.90, 1.11)	9.99E-01	1.20 (1.07, 1.34)	**1.10E-02**	1.16 (0.96, 1.40)
1.06 (0.95, 1.19)	6.53E-01	1.16 (1.04, 1.30)	**4.76E-02**	1.23 (1.02, 1.47)
0.93 (0.84, 1.02)	4.86E-01	1.23 (1.09, 1.37)	**4.18E-03**	1.02 (0.86, 1.22)
1.11 (1.01, 1.23)	1.87E-01	0.92 (0.83, 1.03)	2.86E-01	1.22 (1.06, 1.41)
1.03 (0.91, 1.16)	9.30E-01	1.00 (0.90, 1.11)	9.88E-01	1.24 (1.12, 1.38)
0.89 (0.81, 0.99)	1.71E-01	1.08 (0.97, 1.20)	3.34E-01	0.85 (0.74, 0.99)
0.90 (0.81, 1.00)	1.97E-01	1.05 (0.95, 1.17)	5.36E-01	0.83 (0.73, 0.96)
0.93 (0.83, 1.03)	5.10E-01	1.13 (1.01, 1.25)	9.95E-02	1.02 (0.85, 1.21)
1.15 (1.04, 1.27)	5.43E-02	0.90 (0.81, 1.00)	1.54E-01	1.00 (0.83, 1.19)
0.97 (0.87, 1.08)	9.30E-01	0.97 (0.88, 1.08)	7.31E-01	1.09 (0.92, 1.28)
1.00 (0.90, 1.11)	9.99E-01	1.06 (0.95, 1.18)	4.78E-01	0.91 (0.78, 1.08)
1.01 (0.90, 1.12)	9.93E-01	1.04 (0.94, 1.15)	6.27E-01	0.91 (0.77, 1.07)
0.93 (0.83, 1.04)	5.34E-01	1.10 (0.99, 1.22)	1.80E-01	1.14 (0.96, 1.35)
1.10 (0.99, 1.22)	3.08E-01	0.90 (0.81, 1.00)	1.38E-01	1.08 (0.91, 1.28)
0.93 (0.83, 1.04)	5.34E-01	0.85 (0.76, 0.94)	**1.08E-02**	1.00 (0.84, 1.19)
1.20 (1.07, 1.34)	**1.30E-02**	1.18 (1.07, 1.31)	**6.20E-03**	1.27 (1.08, 1.50)
0.99 (0.89, 1.10)	9.61E-01	1.24 (1.12, 1.36)	**4.15E-04**	1.25 (1.05, 1.48)
0.98 (0.88, 1.09)	9.40E-01	1.02 (0.93, 1.13)	7.77E-01	0.91 (0.76, 1.09)
0.95 (0.85, 1.07)	8.14E-01	0.95 (0.86, 1.06)	5.48E-01	0.97 (0.82, 1.17)
0.97 (0.87, 1.09)	9.30E-01	0.96 (0.87, 1.07)	6.52E-01	0.93 (0.78, 1.12)
0.98 (0.88, 1.09)	9.40E-01	0.96 (0.86, 1.06)	5.64E-01	0.84 (0.70, 1.01)
1.00 (0.90, 1.11)	1.00E+00	1.19 (1.08, 1.31)	**4.14E-03**	1.09 (0.93, 1.30)
1.02 (0.92, 1.13)	9.40E-01	1.19 (1.08, 1.31)	**5.00E-03**	1.08 (0.91, 1.28)
0.87 (0.78, 0.97)	6.20E-02	1.19 (1.08, 1.31)	**4.14E-03**	1.18 (1.00, 1.40)
1.10 (0.99, 1.23)	3.02E-01	0.92 (0.83, 1.02)	2.45E-01	0.87 (0.72, 1.05)
1.03 (0.92, 1.16)	9.30E-01	0.96 (0.86, 1.07)	6.20E-01	0.88 (0.73, 1.06)
0.99 (0.89, 1.10)	9.69E-01	0.90 (0.81, 1.01)	1.81E-01	1.04 (0.88, 1.24)
0.97 (0.87, 1.08)	9.30E-01	0.89 (0.80, 1.00)	1.38E-01	1.02 (0.85, 1.21)
1.06 (0.95, 1.18)	6.65E-01	1.00 (0.90, 1.10)	9.88E-01	1.15 (0.98, 1.35)
1.12 (1.02, 1.25)	1.32E-01	0.95 (0.86, 1.06)	5.34E-01	1.15 (0.97, 1.35)

1.04 (0.93, 1.16)	8.90E-01	1.06 (0.96, 1.18)	3.93E-01	1.03 (0.86, 1.22)
0.95 (0.85, 1.06)	7.91E-01	0.93 (0.84, 1.03)	3.55E-01	0.96 (0.80, 1.14)
1.10 (0.99, 1.23)	3.12E-01	0.96 (0.87, 1.06)	6.16E-01	0.93 (0.78, 1.11)
1.03 (0.92, 1.16)	9.30E-01	0.89 (0.80, 0.99)	1.21E-01	0.87 (0.72, 1.04)
1.08 (0.97, 1.21)	5.10E-01	1.03 (0.93, 1.14)	7.13E-01	1.02 (0.85, 1.22)
0.97 (0.87, 1.08)	9.30E-01	1.16 (1.05, 1.28)	**1.72E-02**	1.09 (0.92, 1.29)
0.98 (0.88, 1.10)	9.49E-01	0.93 (0.84, 1.04)	3.58E-01	0.90 (0.75, 1.08)
1.00 (0.90, 1.12)	9.99E-01	0.86 (0.77, 0.96)	**3.81E-02**	0.88 (0.73, 1.06)
1.03 (0.92, 1.15)	9.30E-01	0.97 (0.87, 1.08)	7.06E-01	0.93 (0.77, 1.12)
1.17 (1.04, 1.32)	6.20E-02	0.85 (0.77, 0.95)	**2.06E-02**	0.73 (0.63, 0.84)
0.76 (0.69, 0.85)	**1.87E-05**	1.02 (0.92, 1.13)	7.82E-01	0.88 (0.74, 1.06)
1.02 (0.91, 1.15)	9.40E-01	0.97 (0.87, 1.08)	7.14E-01	0.86 (0.72, 1.04)
0.86 (0.76, 0.96)	6.12E-02	0.93 (0.83, 1.04)	3.61E-01	0.82 (0.67, 0.99)
1.08 (0.96, 1.20)	5.68E-01	0.95 (0.86, 1.06)	5.82E-01	0.84 (0.69, 1.02)
1.03 (0.92, 1.16)	9.30E-01	0.95 (0.85, 1.06)	5.24E-01	0.82 (0.68, 1.00)
0.99 (0.89, 1.10)	9.61E-01	0.96 (0.87, 1.07)	6.27E-01	0.97 (0.82, 1.16)
1.07 (0.96, 1.20)	5.50E-01	1.00 (0.90, 1.11)	9.88E-01	0.99 (0.83, 1.19)
0.71 (0.64, 0.78)	**1.14E-09**	1.03 (0.93, 1.14)	7.60E-01	0.95 (0.81, 1.13)
1.01 (0.89, 1.13)	9.93E-01	1.00 (0.90, 1.12)	1.00E+00	0.87 (0.74, 1.01)
0.74 (0.67, 0.83)	**1.32E-06**	0.88 (0.79, 0.98)	7.39E-02	0.83 (0.69, 0.98)
1.13 (1.01, 1.27)	1.85E-01	0.98 (0.88, 1.09)	8.50E-01	0.82 (0.69, 0.97)
1.03 (0.91, 1.15)	9.40E-01	0.94 (0.85, 1.05)	4.61E-01	0.77 (0.65, 0.91)
0.85 (0.76, 0.95)	**3.41E-02**	0.95 (0.86, 1.06)	5.31E-01	1.13 (0.95, 1.33)
1.21 (1.08, 1.36)	**9.86E-03**	1.17 (1.05, 1.30)	**2.08E-02**	1.33 (1.12, 1.57)
0.97 (0.86, 1.08)	9.30E-01	1.00 (0.90, 1.11)	1.00E+00	1.27 (1.15, 1.40)
0.80 (0.70, 0.91)	**5.48E-03**	0.76 (0.67, 0.85)	**1.33E-04**	0.95 (0.80, 1.14)
1.37 (1.24, 1.52)	**8.37E-08**	1.16 (1.05, 1.28)	**2.59E-02**	1.02 (0.85, 1.22)
0.84 (0.75, 0.94)	**1.80E-02**	0.76 (0.69, 0.85)	**6.49E-05**	1.04 (0.88, 1.24)
0.84 (0.75, 0.93)	**1.48E-02**	0.77 (0.69, 0.85)	**6.49E-05**	0.85 (0.72, 1.02)
0.69 (0.62, 0.77)	**2.73E-08**	0.81 (0.73, 0.90)	**1.55E-03**	0.97 (0.81, 1.15)
0.92 (0.82, 1.03)	5.10E-01	0.87 (0.78, 0.96)	**4.04E-02**	1.00 (0.84, 1.20)
0.81 (0.72, 0.92)	**9.74E-03**	0.86 (0.77, 0.96)	**2.96E-02**	0.97 (0.81, 1.16)
0.86 (0.76, 0.97)	7.01E-02	0.80 (0.72, 0.89)	**1.06E-03**	0.97 (0.81, 1.17)
0.77 (0.68, 0.88)	**1.30E-03**	0.92 (0.83, 1.03)	2.81E-01	1.15 (1.05, 1.26)
0.93 (0.82, 1.04)	5.50E-01	0.83 (0.75, 0.92)	**5.00E-03**	1.34 (1.14, 1.58)
1.02 (0.93, 1.12)	9.40E-01	0.89 (0.78, 1.02)	2.08E-01	1.02 (0.87, 1.19)
1.16 (1.09, 1.23)	**1.87E-05**	1.07 (0.97, 1.19)	3.10E-01	0.99 (0.81, 1.2)
1.13 (1.07, 1.20)	**1.04E-03**	1.03 (0.94, 1.14)	6.86E-01	1.10 (0.97, 1.25)
0.76 (0.58, 1.00)	2.47E-01	0.81 (0.70, 0.94)	**2.63E-02**	1.09 (1.03, 1.14)
1.07 (0.95, 1.19)	6.39E-01	1.10 (0.99, 1.23)	2.11E-01	0.83 (0.71, 0.97)
0.86 (0.77, 0.97)	8.91E-02	1.14 (1.03, 1.26)	5.43E-02	1.00 (0.84, 1.20)

			UKB	
Other mortality			All causes	
	Age >= 60y		Age < 60y	
P value	Adjusted HR (95% CI)	P value	Adjusted HR (95% CI)	P value
9.48E-01	0.99 (0.78, 1.26)	9.98E-01	1.01 (0.98, 1.03)	6.55E-01
9.45E-01	1.00 (0.78, 1.26)	9.98E-01	1.00 (0.98, 1.03)	7.41E-01
9.14E-01	0.99 (0.78, 1.26)	9.98E-01	1.01 (0.98, 1.03)	6.09E-01
8.28E-01	1.01 (0.80, 1.28)	9.98E-01	1.00 (0.97, 1.02)	9.09E-01
8.59E-01	1.04 (0.82, 1.31)	9.98E-01	0.99 (0.97, 1.02)	6.70E-01
7.82E-01	0.98 (0.77, 1.25)	9.98E-01	1.00 (0.98, 1.03)	7.73E-01
9.82E-01	0.99 (0.78, 1.26)	9.98E-01	1.01 (0.98, 1.03)	6.78E-01
8.59E-01	0.94 (0.73, 1.20)	9.98E-01	0.97 (0.95, 1.00)	**4.30E-02**
8.59E-01	0.94 (0.73, 1.21)	9.98E-01	0.97 (0.94, 0.99)	**2.39E-02**
8.13E-01	0.95 (0.74, 1.22)	9.98E-01	0.97 (0.95, 1.00)	**3.59E-02**
8.59E-01	0.95 (0.74, 1.22)	9.98E-01	0.95 (0.92, 0.97)	**1.13E-04**
8.92E-01	0.96 (0.75, 1.22)	9.98E-01	0.93 (0.91, 0.96)	**2.96E-07**
8.13E-01	0.96 (0.75, 1.23)	9.98E-01	0.97 (0.94, 0.99)	**1.44E-02**
8.62E-01	0.94 (0.73, 1.20)	9.98E-01	0.98 (0.95, 1.00)	8.55E-02
8.92E-01	0.96 (0.75, 1.22)	9.98E-01	0.95 (0.93, 0.98)	**1.00E-03**
8.92E-01	0.96 (0.75, 1.22)	9.98E-01	0.94 (0.92, 0.97)	**2.81E-05**
8.67E-01	0.96 (0.75, 1.22)	9.98E-01	0.96 (0.93, 0.98)	**2.46E-03**
9.36E-01	0.96 (0.76, 1.23)	9.98E-01	0.94 (0.91, 0.96)	**7.35E-06**
9.45E-01	0.97 (0.76, 1.23)	9.98E-01	0.93 (0.91, 0.96)	**6.84E-07**
8.31E-01	0.96 (0.75, 1.22)	9.98E-01	0.95 (0.92, 0.97)	**9.24E-05**
8.92E-01	0.95 (0.75, 1.21)	9.98E-01	0.94 (0.92, 0.97)	**1.19E-05**
9.52E-01	0.94 (0.74, 1.19)	9.98E-01	0.91 (0.89, 0.93)	**7.75E-12**
9.52E-01	0.94 (0.74, 1.19)	9.98E-01	0.91 (0.89, 0.94)	**1.36E-11**
9.45E-01	0.94 (0.74, 1.19)	9.98E-01	0.91 (0.89, 0.94)	**2.25E-11**
9.36E-01	0.96 (0.76, 1.21)	9.98E-01	0.90 (0.88, 0.93)	**2.88E-14**
8.31E-01	0.97 (0.76, 1.23)	9.98E-01	0.90 (0.88, 0.93)	**1.85E-14**
9.45E-01	0.95 (0.75, 1.20)	9.98E-01	0.91 (0.89, 0.93)	**3.23E-12**
9.82E-01	0.94 (0.74, 1.19)	9.98E-01	0.93 (0.91, 0.96)	**5.92E-07**
8.31E-01	0.95 (0.75, 1.20)	9.98E-01	0.97 (0.94, 0.99)	**1.82E-02**
8.28E-01	0.94 (0.75, 1.20)	9.98E-01	0.96 (0.94, 0.99)	**3.35E-03**
8.03E-01	0.93 (0.73, 1.18)	9.98E-01	0.93 (0.91, 0.96)	**6.06E-07**
5.86E-01	0.95 (0.75, 1.20)	9.98E-01	0.94 (0.92, 0.97)	**6.22E-06**
4.04E-01	0.97 (0.77, 1.22)	9.98E-01	0.95 (0.93, 0.98)	**5.61E-04**
8.70E-01	0.93 (0.74, 1.18)	9.98E-01	0.92 (0.90, 0.94)	**3.40E-10**
9.36E-01	0.95 (0.75, 1.21)	9.98E-01	1.00 (0.97, 1.02)	8.60E-01
8.31E-01	1.02 (0.82, 1.27)	9.98E-01	1.02 (1.00, 1.05)	9.95E-02
8.31E-01	1.02 (0.82, 1.27)	9.98E-01	1.03 (1.01, 1.06)	**2.30E-02**
6.15E-01	1.03 (0.83, 1.28)	9.98E-01	1.05 (1.02, 1.07)	**3.49E-04**
8.59E-01	1.03 (0.83, 1.27)	9.98E-01	0.99 (0.97, 1.02)	7.16E-01
8.62E-01	1.04 (0.83, 1.28)	9.98E-01	0.98 (0.96, 1.01)	2.10E-01

8.59E-01	1.00 (0.81, 1.25)	9.98E-01	1.02 (1.00, 1.05)	1.12E-01
9.82E-01	0.99 (0.79, 1.24)	9.98E-01	1.07 (1.05, 1.10)	**1.57E-08**
6.74E-01	1.04 (0.84, 1.29)	9.98E-01	0.93 (0.90, 0.95)	**1.17E-08**
6.14E-01	1.04 (0.83, 1.29)	9.98E-01	0.93 (0.91, 0.96)	**4.60E-07**
3.41E-01	1.04 (0.84, 1.30)	9.98E-01	0.95 (0.93, 0.97)	**1.31E-04**
4.04E-01	1.02 (0.82, 1.27)	9.98E-01	0.91 (0.89, 0.94)	**3.83E-11**
3.77E-01	1.01 (0.81, 1.26)	9.98E-01	0.91 (0.89, 0.93)	**2.35E-12**
5.45E-01	1.05 (0.85, 1.30)	9.98E-01	0.93 (0.91, 0.96)	**1.28E-07**
3.25E-01	1.06 (0.86, 1.30)	9.98E-01	1.09 (1.07, 1.12)	**1.02E-12**
3.49E-01	1.01 (0.81, 1.26)	9.98E-01	0.91 (0.89, 0.93)	**1.23E-12**
3.25E-01	1.01 (0.81, 1.26)	9.98E-01	0.89 (0.87, 0.92)	**3.46E-17**
1.99E-01	1.00 (0.80, 1.25)	9.98E-01	0.89 (0.87, 0.91)	**7.21E-19**
2.50E-01	1.01 (0.81, 1.26)	9.98E-01	0.88 (0.86, 0.91)	**2.00E-20**
2.29E-01	1.00 (0.80, 1.26)	9.98E-01	0.88 (0.86, 0.91)	**3.30E-20**
3.33E-01	1.02 (0.82, 1.28)	9.98E-01	0.89 (0.86, 0.91)	**5.44E-20**
1.99E-01	1.06 (0.86, 1.3)	9.98E-01	1.08 (1.05, 1.11)	**4.94E-10**
1.70E-01	0.98 (0.78, 1.23)	9.98E-01	0.90 (0.88, 0.93)	**1.63E-14**
1.56E-01	0.98 (0.78, 1.23)	9.98E-01	0.88 (0.86, 0.91)	**1.80E-20**
1.46E-01	0.92 (0.72, 1.17)	9.98E-01	0.88 (0.85, 0.90)	**2.26E-22**
1.44E-01	0.99 (0.79, 1.24)	9.98E-01	0.87 (0.85, 0.90)	**3.32E-23**
1.44E-01	1.00 (0.80, 1.26)	9.98E-01	0.88 (0.86, 0.90)	**9.71E-21**
1.52E-01	0.93 (0.73, 1.17)	9.98E-01	0.87 (0.84, 0.89)	**3.65E-27**
3.77E-01	1.03 (0.83, 1.27)	9.98E-01	1.05 (1.02, 1.07)	**1.55E-04**
1.45E-01	0.95 (0.75, 1.2)	9.98E-01	0.91 (0.89, 0.94)	**3.30E-11**
1.44E-01	0.95 (0.75, 1.2)	9.98E-01	0.89 (0.87, 0.92)	**1.11E-16**
1.46E-01	0.86 (0.67, 1.09)	9.98E-01	0.91 (0.89, 0.93)	**6.13E-13**
1.41E-01	0.97 (0.77, 1.23)	9.98E-01	0.88 (0.86, 0.90)	**2.35E-21**
1.41E-01	0.99 (0.79, 1.25)	9.98E-01	0.89 (0.87, 0.91)	**2.32E-18**
1.46E-01	0.89 (0.70, 1.13)	9.98E-01	0.87 (0.85, 0.90)	**4.57E-25**
8.92E-01	0.98 (0.78, 1.23)	9.98E-01	1.02 (1.00, 1.05)	6.47E-02
9.99E-02	1.02 (0.82, 1.27)	9.98E-01	1.11 (1.08, 1.14)	**8.12E-13**
1.03E-01	1.02 (0.81, 1.27)	9.98E-01	1.12 (1.09, 1.15)	**9.06E-15**
1.03E-01	1.05 (0.84, 1.30)	9.98E-01	1.13 (1.10, 1.17)	**2.90E-17**
1.44E-01	0.98 (0.78, 1.22)	9.98E-01	1.10 (1.07, 1.13)	**5.74E-10**
1.46E-01	0.97 (0.78, 1.21)	9.98E-01	1.09 (1.06, 1.12)	**3.59E-08**
1.41E-01	0.98 (0.79, 1.23)	9.98E-01	1.12 (1.09, 1.15)	**8.78E-17**
5.27E-02	1.08 (0.87, 1.34)	9.98E-01	1.08 (1.06, 1.11)	**2.52E-11**
1.41E-01	1.08 (0.86, 1.35)	9.98E-01	1.08 (1.05, 1.12)	**4.30E-07**
1.41E-01	1.07 (0.85, 1.35)	9.98E-01	1.09 (1.06, 1.12)	**4.02E-08**
1.70E-01	1.06 (0.84, 1.33)	9.98E-01	1.10 (1.06, 1.13)	**3.53E-09**
1.41E-01	1.07 (0.85, 1.35)	9.98E-01	1.07 (1.04, 1.11)	**7.25E-06**
1.38E-01	1.07 (0.85, 1.35)	9.98E-01	1.07 (1.03, 1.10)	**5.17E-05**
1.44E-01	1.07 (0.85, 1.34)	9.98E-01	1.10 (1.06, 1.13)	**2.79E-09**
**3.53E-02**	1.19 (0.97, 1.45)	9.98E-01	1.11 (1.08, 1.13)	**1.67E-15**
7.38E-01	0.81 (0.64, 1.02)	9.98E-01	1.00 (0.97, 1.02)	8.17E-01
7.38E-01	0.81 (0.64, 1.02)	9.98E-01	1.00 (0.98, 1.03)	8.60E-01
9.45E-01	0.83 (0.66, 1.05)	9.98E-01	1.01 (0.98, 1.04)	4.90E-01
5.43E-01	0.80 (0.64, 0.99)	9.98E-01	0.99 (0.96, 1.01)	3.64E-01

4.13E-01	0.79 (0.64, 0.99)	9.98E-01	0.98 (0.95, 1.01)	2.07E-01
8.92E-01	0.82 (0.65, 1.03)	9.98E-01	1.01 (0.98, 1.04)	6.80E-01
6.77E-01	0.98 (0.78, 1.23)	9.98E-01	1.05 (1.02, 1.08)	**1.44E-04**
6.67E-02	0.78 (0.62, 0.97)	9.98E-01	0.87 (0.85, 0.89)	**4.57E-25**
5.75E-02	0.77 (0.61, 0.97)	9.98E-01	0.91 (0.89, 0.94)	**2.56E-11**
3.77E-01	0.81 (0.65, 1.01)	9.98E-01	0.94 (0.91, 0.96)	**9.38E-07**
9.63E-02	0.84 (0.67, 1.05)	9.98E-01	0.87 (0.85, 0.89)	**1.21E-24**
1.38E-01	0.89 (0.71, 1.12)	9.98E-01	0.85 (0.83, 0.88)	**3.37E-31**
3.77E-01	0.76 (0.61, 0.95)	9.98E-01	0.94 (0.91, 0.96)	**1.78E-06**
9.52E-01	1.00 (0.79, 1.25)	9.98E-01	1.02 (1.00, 1.05)	1.12E-01
8.05E-01	1.05 (0.82, 1.34)	9.98E-01	1.07 (1.04, 1.11)	**1.77E-05**
1.41E-01	1.05 (0.84, 1.32)	9.98E-01	1.01 (0.98, 1.04)	7.16E-01
2.90E-01	1.04 (0.83, 1.31)	9.98E-01	1.00 (0.97, 1.03)	9.86E-01
1.41E-01	1.04 (0.83, 1.31)	9.98E-01	1.02 (0.99, 1.05)	2.62E-01
1.41E-01	0.94 (0.75, 1.18)	9.98E-01	0.98 (0.95, 1.00)	1.15E-01
9.45E-01	1.10 (0.85, 1.42)	9.98E-01	0.99 (0.96, 1.02)	7.16E-01
2.02E-01	0.98 (0.76, 1.27)	9.98E-01	0.98 (0.95, 1.01)	2.56E-01
6.36E-01	0.94 (0.72, 1.23)	9.98E-01	1.04 (1.01, 1.07)	**5.66E-03**
3.24E-01	1.07 (0.83, 1.38)	9.98E-01	1.00 (0.97, 1.02)	7.73E-01
3.77E-01	0.97 (0.76, 1.23)	9.98E-01	1.05 (1.02, 1.09)	**4.21E-03**
8.92E-01	1.19 (0.92, 1.55)	9.98E-01	1.05 (1.02, 1.07)	**1.12E-03**
8.21E-01	1.18 (0.91, 1.53)	9.98E-01	1.04 (1.01, 1.07)	**9.32E-03**
3.74E-01	1.08 (0.86, 1.36)	9.98E-01	1.06 (1.03, 1.09)	**8.65E-05**
8.59E-01	1.10 (0.87, 1.41)	9.98E-01	0.94 (0.92, 0.97)	**7.04E-05**
8.67E-01	0.84 (0.64, 1.09)	9.98E-01	0.98 (0.95, 1.00)	1.09E-01
6.14E-01	0.98 (0.78, 1.21)	9.98E-01	0.97 (0.94, 0.99)	**1.38E-02**
9.52E-01	1.12 (0.90, 1.40)	9.98E-01	0.96 (0.94, 0.99)	**7.68E-03**
8.31E-01	1.11 (0.89, 1.38)	9.98E-01	0.98 (0.95, 1.00)	7.30E-02
4.03E-01	1.03 (0.83, 1.30)	9.98E-01	1.03 (1.01, 1.06)	**2.08E-02**
9.36E-01	0.90 (0.72, 1.13)	9.98E-01	0.90 (0.87, 0.92)	**6.81E-16**
9.36E-01	0.93 (0.73, 1.18)	9.98E-01	0.94 (0.92, 0.96)	**2.46E-06**
8.21E-01	1.01 (0.81, 1.26)	9.98E-01	0.98 (0.95, 1.00)	7.67E-02
6.60E-01	1.01 (0.81, 1.27)	9.98E-01	0.90 (0.87, 0.92)	**1.46E-15**
4.06E-01	0.95 (0.75, 1.19)	9.98E-01	1.08 (1.05, 1.11)	**6.19E-09**
8.31E-01	1.01 (0.81, 1.26)	9.98E-01	1.16 (1.13, 1.19)	**2.84E-28**
6.15E-01	0.98 (0.79, 1.23)	9.98E-01	0.91 (0.89, 0.93)	**2.34E-14**
8.64E-01	1.00 (0.80, 1.25)	9.98E-01	0.90 (0.88, 0.93)	**2.06E-15**
8.64E-01	1.03 (0.82, 1.29)	9.98E-01	0.95 (0.93, 0.98)	**1.11E-04**
9.41E-01	0.94 (0.77, 1.15)	9.98E-01	1.08 (1.05, 1.11)	**1.09E-09**
9.45E-01	1.01 (0.81, 1.25)	9.98E-01	1.13 (1.10, 1.16)	**1.80E-20**
2.60E-01	1.06 (0.85, 1.32)	9.98E-01	0.86 (0.84, 0.88)	**4.78E-38**
2.51E-01	0.92 (0.76, 1.12)	9.98E-01	0.84 (0.82, 0.86)	**2.33E-48**
1.98E-01	0.89 (0.73, 1.07)	9.98E-01	0.96 (0.94, 0.99)	**2.57E-03**
8.92E-01	1.02 (0.81, 1.28)	9.98E-01	1.14 (1.11, 1.16)	**1.99E-32**
1.44E-01	1.07 (0.87, 1.32)	9.98E-01	1.01 (0.98, 1.03)	6.55E-01
8.23E-01	1.07 (0.87, 1.32)	9.98E-01	0.92 (0.91, 0.93)	**1.39E-48**
1.79E-01	0.90 (0.74, 1.10)	9.98E-01	0.92 (0.91, 0.93)	**2.33E-48**
1.41E-01	0.88 (0.73, 1.07)	9.98E-01	0.93 (0.90, 0.95)	**4.21E-11**

8.59E-01	1.07 (0.84, 1.36)	9.98E-01	1.11 (1.10, 1.13)	**9.17E-55**
9.99E-02	1.12 (0.92, 1.37)	9.98E-01	1.00 (0.97, 1.02)	8.61E-01
3.25E-01	0.98 (0.78, 1.23)	9.98E-01	0.92 (0.91, 0.93)	**9.21E-56**
1.44E-01	0.98 (0.79, 1.22)	9.98E-01	0.91 (0.89, 0.92)	**4.52E-20**
1.41E-01	1.00 (0.80, 1.25)	9.98E-01	0.93 (0.91, 0.96)	**1.22E-07**
1.56E-01	0.96 (0.76, 1.22)	9.98E-01	1.12 (1.10, 1.13)	**2.60E-67**
**3.53E-02**	1.09 (0.87, 1.35)	9.98E-01	1.12 (1.09, 1.15)	**7.84E-18**
2.47E-01	0.97 (0.77, 1.22)	9.98E-01	0.87 (0.86, 0.89)	**8.56E-73**
1.75E-01	1.00 (0.80, 1.25)	9.98E-01	0.90 (0.87, 0.92)	**1.14E-17**
1.56E-01	1.01 (0.81, 1.27)	9.98E-01	0.92 (0.90, 0.94)	**8.38E-13**
1.70E-01	0.92 (0.72, 1.17)	9.98E-01	1.09 (1.07, 1.11)	**1.25E-21**
1.46E-01	1.06 (0.85, 1.31)	9.98E-01	1.11 (1.08, 1.15)	**1.03E-12**
6.60E-01	1.08 (0.86, 1.34)	9.98E-01	0.88 (0.86, 0.90)	**6.20E-21**
3.70E-01	0.89 (0.72, 1.09)	9.98E-01	0.91 (0.89, 0.93)	**1.42E-18**
3.25E-01	0.91 (0.73, 1.13)	9.98E-01	0.93 (0.91, 0.96)	**5.38E-06**
8.62E-01	0.87 (0.72, 1.04)	9.98E-01	1.00 (0.98, 1.03)	8.29E-01
5.88E-01	1.10 (0.90, 1.35)	9.98E-01	1.02 (1.00, 1.05)	8.54E-02
**3.53E-02**	0.85 (0.66, 1.08)	9.98E-01	0.98 (0.95, 1.00)	8.06E-02
3.25E-01	1.08 (0.85, 1.38)	9.98E-01	0.96 (0.94, 0.99)	**2.53E-03**
1.44E-01	1.07 (0.84, 1.36)	9.98E-01	1.06 (1.03, 1.09)	**3.76E-05**
9.36E-01	1.09 (0.86, 1.37)	9.98E-01	1.02 (0.99, 1.04)	2.07E-01
9.38E-02	1.09 (0.88, 1.34)	9.98E-01	1.08 (1.05, 1.11)	**4.84E-08**
**4.68E-03**	1.17 (0.99, 1.38)	9.98E-01	0.93 (0.91, 0.96)	**1.64E-07**
1.44E-01	0.86 (0.72, 1.03)	9.98E-01	0.93 (0.90, 0.95)	**9.21E-09**
9.99E-02	0.88 (0.74, 1.05)	9.98E-01	1.00 (0.97, 1.03)	8.33E-01
9.41E-01	0.84 (0.69, 1.04)	9.98E-01	1.06 (1.04, 1.08)	**1.17E-06**
9.82E-01	1.12 (0.91, 1.38)	9.98E-01	1.16 (1.13, 1.19)	**3.43E-27**
6.12E-01	0.97 (0.77, 1.21)	9.98E-01	0.87 (0.85, 0.89)	**3.59E-28**
5.51E-01	1.00 (0.79, 1.25)	9.98E-01	0.86 (0.84, 0.88)	**5.20E-37**
5.07E-01	1.02 (0.81, 1.28)	9.98E-01	1.09 (1.07, 1.12)	**1.55E-11**
3.33E-01	0.86 (0.68, 1.09)	9.98E-01	1.09 (1.06, 1.12)	**5.44E-10**
6.45E-01	1.10 (0.89, 1.37)	9.98E-01	0.90 (0.88, 0.93)	**3.31E-12**
9.82E-01	0.97 (0.77, 1.21)	9.98E-01	0.98 (0.95, 1.00)	6.85E-02
5.75E-02	1.24 (1.00, 1.54)	9.98E-01	1.15 (1.12, 1.19)	**6.42E-21**
1.03E-01	1.02 (0.82, 1.28)	9.98E-01	0.91 (0.88, 0.93)	**3.19E-13**
6.02E-01	0.97 (0.77, 1.21)	9.98E-01	0.94 (0.92, 0.97)	**1.30E-05**
9.14E-01	0.96 (0.76, 1.22)	9.98E-01	0.88 (0.86, 0.90)	**9.35E-22**
7.49E-01	0.99 (0.79, 1.24)	9.98E-01	0.92 (0.90, 0.95)	**3.53E-09**
1.99E-01	1.02 (0.81, 1.27)	9.98E-01	0.90 (0.88, 0.93)	**5.31E-14**
5.57E-01	0.89 (0.71, 1.13)	9.98E-01	0.92 (0.89, 0.94)	**4.75E-11**
6.26E-01	0.90 (0.71, 1.13)	9.98E-01		
1.87E-01	0.90 (0.72, 1.13)	9.98E-01		
3.33E-01	1.00 (0.80, 1.25)	9.99E-01	0.99 (0.97, 1.02)	5.96E-01
3.81E-01	1.01 (0.81, 1.27)	9.98E-01	0.97 (0.95, 1.00)	5.40E-02
8.59E-01	0.96 (0.76, 1.21)	9.98E-01	1.07 (1.04, 1.09)	**4.18E-07**
9.42E-01	0.94 (0.74, 1.19)	9.98E-01	1.06 (1.03, 1.08)	**9.91E-06**
2.31E-01	1.05 (0.85, 1.3)	9.98E-01	0.98 (0.96, 1.01)	2.00E-01
2.79E-01	1.06 (0.85, 1.32)	9.98E-01	1.00 (0.97, 1.02)	8.29E-01

9.14E-01	0.90 (0.71, 1.13)	9.98E-01	0.97 (0.95, 1.00)	5.40E-02
8.60E-01	0.97 (0.78, 1.22)	9.98E-01	0.98 (0.95, 1.01)	1.81E-01
6.82E-01	0.93 (0.74, 1.16)	9.98E-01	0.97 (0.94, 1.00)	**3.40E-02**
3.25E-01	1.02 (0.81, 1.28)	9.98E-01	1.00 (0.97, 1.02)	8.14E-01
9.36E-01	0.94 (0.75, 1.18)	9.98E-01	0.91 (0.89, 0.94)	**1.36E-11**
6.15E-01	0.88 (0.70, 1.10)	9.98E-01	0.93 (0.91, 0.96)	**4.46E-07**
5.31E-01	0.99 (0.78, 1.24)	9.98E-01	0.99 (0.96, 1.01)	3.74E-01
3.77E-01	1.08 (0.85, 1.35)	9.98E-01	0.83 (0.81, 0.85)	**4.07E-39**
7.25E-01	0.91 (0.72, 1.16)	9.98E-01	0.88 (0.86, 0.91)	**8.30E-17**
**1.30E-03**	0.94 (0.74, 1.19)	9.98E-01	0.91 (0.89, 0.93)	**2.40E-12**
3.77E-01	0.86 (0.68, 1.08)	9.98E-01	0.89 (0.87, 0.92)	**1.53E-15**
3.19E-01	0.99 (0.77, 1.26)	9.98E-01	0.91 (0.89, 0.94)	**1.87E-11**
1.56E-01	0.88 (0.69, 1.13)	9.98E-01	0.90 (0.87, 0.92)	**4.70E-15**
2.22E-01	0.97 (0.76, 1.24)	9.98E-01	1.04 (1.01, 1.06)	**6.86E-03**
1.75E-01	0.95 (0.75, 1.22)	9.98E-01	1.02 (0.99, 1.04)	1.88E-01
9.14E-01	0.91 (0.72, 1.16)	9.98E-01	0.89 (0.86, 0.92)	**6.51E-15**
9.52E-01	0.89 (0.70, 1.13)	9.98E-01	0.88 (0.86, 0.90)	**1.78E-21**
8.40E-01	0.92 (0.73, 1.15)	9.98E-01	0.86 (0.84, 0.89)	**4.44E-22**
2.09E-01	1.03 (0.81, 1.31)	9.98E-01	0.91 (0.89, 0.93)	**9.75E-13**
1.44E-01	0.91 (0.72, 1.15)	9.98E-01	0.87 (0.85, 0.89)	**1.97E-26**
1.41E-01	1.05 (0.83, 1.33)	9.98E-01	1.13 (1.10, 1.16)	**5.01E-19**
5.27E-02	1.02 (0.81, 1.30)	9.98E-01	1.11 (1.08, 1.14)	**1.15E-16**
3.77E-01	1.01 (0.80, 1.28)	9.98E-01	1.10 (1.09, 1.12)	**1.94E-32**
**3.53E-02**	0.95 (0.75, 1.20)	9.98E-01	1.06 (1.04, 1.09)	**6.05E-06**
**3.63E-04**	1.19 (1.01, 1.39)	9.98E-01	1.09 (1.06, 1.12)	**8.86E-11**
8.59E-01	0.86 (0.68, 1.10)	9.98E-01	0.98 (0.95, 1.00)	9.17E-02
9.36E-01	1.15 (0.92, 1.42)	9.98E-01	0.93 (0.90, 0.95)	**1.50E-08**
8.59E-01	0.83 (0.66, 1.04)	9.98E-01	0.89 (0.86, 0.91)	**2.15E-16**
2.31E-01	0.94 (0.75, 1.18)	9.98E-01	0.97 (0.94, 0.99)	**2.39E-02**
8.92E-01	0.93 (0.74, 1.16)	9.98E-01	0.91 (0.89, 0.94)	**1.27E-10**
9.82E-01	0.82 (0.65, 1.05)	9.98E-01	0.92 (0.89, 0.94)	**3.53E-09**
8.93E-01	0.86 (0.68, 1.08)	9.98E-01	1.03 (1.01, 1.05)	**5.53E-04**
8.99E-01	0.85 (0.67, 1.08)	9.98E-01	1.04 (1.02, 1.07)	**1.98E-03**
5.75E-02	1.09 (0.88, 1.36)	9.98E-01	1.08 (1.07, 1.09)	**1.53E-70**
**1.74E-02**	0.96 (0.77, 1.20)	9.98E-01	1.06 (1.05, 1.07)	**6.38E-26**
9.41E-01	0.87 (0.61, 1.24)	9.98E-01	1.08 (1.06, 1.09)	**1.23E-28**
9.45E-01	1.13 (0.92, 1.38)	9.98E-01	1.05 (1.04, 1.07)	**5.73E-16**
3.25E-01	1.15 (0.98, 1.35)	9.98E-01	0.82 (0.80, 0.84)	**2.33E-52**
**3.53E-02**	1.27 (1.16, 1.40)	**2.18E-04**	1.16 (1.14, 1.19)	**6.59E-31**
1.41E-01	0.81 (0.66, 1.01)	9.98E-01	0.97 (0.94, 1.00)	5.85E-02
9.82E-01	1.00 (0.79, 1.27)	9.98E-01	0.93 (0.90, 0.95)	**1.14E-07**
			0.90 (0.88, 0.92)	**1.53E-15**
			0.88 (0.86, 0.90)	**1.50E-21**
			0.88 (0.86, 0.90)	**7.23E-21**
			0.99 (0.96, 1.02)	6.55E-01
			0.86 (0.84, 0.89)	**7.06E-23**
			1.11 (1.09, 1.13)	**8.40E-29**
			0.95 (0.92, 0.97)	**8.28E-05**

0.88 (0.86, 0.90)	**1.14E-22**
1.04 (1.01, 1.07)	**8.89E-03**
1.05 (1.03, 1.07)	**5.65E-06**
1.07 (1.06, 1.08)	**4.07E-39**
0.93 (0.90, 0.95)	**1.11E-07**
0.97 (0.94, 0.99)	**1.10E-02**
0.91 (0.88, 0.93)	**1.67E-13**
0.94 (0.91, 0.96)	**1.15E-05**
0.96 (0.93, 0.98)	**2.42E-03**
0.96 (0.94, 0.99)	**3.98E-03**
0.89 (0.86, 0.91)	**1.12E-19**
1.03 (1.01, 1.06)	**2.51E-02**
0.93 (0.91, 0.96)	**4.95E-07**
0.96 (0.94, 0.99)	**3.54E-03**
0.89 (0.87, 0.91)	**1.86E-18**
1.03 (1.00, 1.06)	1.10E-01
0.94 (0.92, 0.96)	**5.38E-06**
1.00 (0.97, 1.03)	8.60E-01
1.04 (1.00, 1.07)	**3.43E-02**

mortality		Ischaemic heart di	
Age >= 60y		Age < 60y	
Adjusted HR (95% CI)	P value	Adjusted HR (95% CI)	P value
0.97 (0.96, 0.99)	**2.55E-03**	1.11 (1.04, 1.19)	**4.36E-03**
0.97 (0.96, 0.99)	**1.64E-03**	1.11 (1.04, 1.18)	**6.06E-03**
0.98 (0.96, 0.99)	**5.81E-03**	1.11 (1.04, 1.19)	**4.36E-03**
0.97 (0.95, 0.99)	**2.90E-04**	1.11 (1.04, 1.19)	**6.39E-03**
0.97 (0.95, 0.98)	**8.82E-05**	1.11 (1.04, 1.19)	**7.40E-03**
0.97 (0.96, 0.99)	**1.35E-03**	1.11 (1.04, 1.19)	**5.92E-03**
0.97 (0.96, 0.99)	**2.30E-03**	1.10 (1.03, 1.17)	**6.88E-03**
0.94 (0.93, 0.96)	**6.00E-12**	1.13 (1.05, 1.21)	**2.16E-03**
0.94 (0.92, 0.96)	**1.53E-12**	1.12 (1.05, 1.21)	**3.90E-03**
0.94 (0.93, 0.96)	**2.09E-11**	1.13 (1.05, 1.21)	**3.92E-03**
0.93 (0.91, 0.94)	**5.74E-18**	1.11 (1.03, 1.20)	**1.67E-02**
0.92 (0.90, 0.93)	**6.40E-23**	1.09 (1.00, 1.18)	6.59E-02
0.94 (0.92, 0.96)	**1.25E-12**	1.12 (1.04, 1.21)	**5.49E-03**
0.95 (0.93, 0.96)	**1.03E-10**	1.12 (1.05, 1.20)	**2.75E-03**
0.93 (0.91, 0.94)	**1.51E-17**	1.13 (1.05, 1.21)	**3.92E-03**
0.92 (0.90, 0.94)	**1.15E-21**	1.12 (1.04, 1.20)	**6.88E-03**
0.93 (0.92, 0.95)	**6.82E-16**	1.14 (1.05, 1.22)	**2.60E-03**
0.92 (0.90, 0.93)	**7.29E-22**	1.12 (1.03, 1.21)	**1.16E-02**
0.92 (0.90, 0.93)	**6.12E-23**	1.10 (1.01, 1.19)	**4.19E-02**
0.92 (0.91, 0.94)	**9.20E-20**	1.13 (1.05, 1.22)	**4.81E-03**
0.92 (0.90, 0.93)	**8.42E-23**	1.11 (1.03, 1.19)	**1.00E-02**
0.90 (0.88, 0.91)	**2.74E-34**	1.05 (0.97, 1.13)	3.10E-01
0.90 (0.88, 0.91)	**5.03E-36**	1.07 (0.99, 1.15)	1.38E-01
0.90 (0.88, 0.92)	**1.57E-32**	1.04 (0.97, 1.13)	3.54E-01
0.90 (0.89, 0.92)	**8.48E-30**	0.97 (0.90, 1.05)	5.09E-01
0.91 (0.89, 0.93)	**2.97E-25**	0.93 (0.86, 1.01)	1.13E-01
0.90 (0.88, 0.92)	**4.41E-32**	1.02 (0.95, 1.10)	6.54E-01
0.91 (0.89, 0.92)	**1.76E-27**	1.11 (1.03, 1.20)	**9.16E-03**
0.93 (0.92, 0.95)	**6.24E-15**	1.14 (1.06, 1.23)	**1.89E-03**
0.93 (0.91, 0.94)	**2.19E-17**	1.13 (1.05, 1.22)	**4.69E-03**
0.91 (0.90, 0.93)	**1.89E-24**	1.08 (1.00, 1.17)	7.37E-02
0.92 (0.91, 0.94)	**2.04E-19**	1.06 (0.99, 1.15)	1.63E-01
0.93 (0.92, 0.95)	**6.79E-15**	1.08 (1.00, 1.17)	6.82E-02
0.91 (0.89, 0.92)	**6.04E-28**	1.03 (0.96, 1.11)	5.09E-01
0.95 (0.94, 0.97)	**4.26E-08**	1.16 (1.09, 1.25)	**9.73E-05**
0.99 (0.97, 1.00)	1.65E-01	1.08 (1.00, 1.17)	6.60E-02
0.99 (0.98, 1.01)	4.43E-01	1.09 (1.01, 1.18)	**4.45E-02**
1.01 (0.99, 1.03)	3.23E-01	1.12 (1.04, 1.20)	**7.28E-03**
0.97 (0.96, 0.99)	**5.69E-03**	0.99 (0.92, 1.08)	9.19E-01
0.97 (0.95, 0.99)	**5.99E-04**	0.96 (0.89, 1.04)	4.14E-01

0.99 (0.97, 1.01)	2.98E-01	1.07 (0.99, 1.15)	1.23E-01
1.02 (1.00, 1.03)	7.84E-02	1.20 (1.12, 1.28)	**8.72E-07**
0.93 (0.91, 0.94)	**1.17E-16**	0.97 (0.89, 1.04)	4.62E-01
0.92 (0.91, 0.94)	**1.10E-17**	0.94 (0.87, 1.02)	2.05E-01
0.94 (0.92, 0.95)	**1.85E-12**	0.95 (0.88, 1.03)	2.93E-01
0.91 (0.90, 0.93)	**9.23E-23**	0.91 (0.84, 0.99)	**4.17E-02**
0.91 (0.89, 0.92)	**3.46E-25**	0.91 (0.84, 0.98)	**3.47E-02**
0.93 (0.91, 0.94)	**8.77E-16**	0.92 (0.85, 1.00)	7.06E-02
1.03 (1.01, 1.05)	**2.46E-04**	1.19 (1.11, 1.27)	**1.70E-06**
0.90 (0.89, 0.92)	**1.16E-29**	0.99 (0.92, 1.07)	9.21E-01
0.88 (0.87, 0.90)	**1.05E-41**	0.96 (0.89, 1.03)	3.16E-01
0.88 (0.87, 0.90)	**4.11E-42**	0.95 (0.88, 1.02)	2.12E-01
0.88 (0.86, 0.89)	**3.64E-46**	0.94 (0.87, 1.01)	1.43E-01
0.87 (0.86, 0.89)	**3.52E-48**	0.95 (0.88, 1.02)	2.37E-01
0.89 (0.87, 0.90)	**1.23E-38**	0.91 (0.84, 0.98)	**2.83E-02**
1.02 (1.00, 1.04)	**2.35E-02**	1.19 (1.12, 1.27)	**9.51E-07**
0.89 (0.88, 0.91)	**1.16E-38**	1.01 (0.93, 1.09)	8.92E-01
0.87 (0.86, 0.89)	**1.95E-53**	1.00 (0.93, 1.08)	9.26E-01
0.87 (0.85, 0.89)	**1.03E-54**	1.00 (0.93, 1.08)	9.32E-01
0.87 (0.85, 0.88)	**1.56E-57**	0.99 (0.91, 1.06)	7.99E-01
0.87 (0.85, 0.88)	**2.78E-55**	1.00 (0.93, 1.08)	9.31E-01
0.87 (0.85, 0.88)	**2.77E-56**	0.94 (0.87, 1.01)	1.65E-01
0.99 (0.98, 1.01)	4.27E-01	1.18 (1.10, 1.25)	**3.39E-06**
0.90 (0.89, 0.92)	**3.08E-30**	1.02 (0.95, 1.10)	6.55E-01
0.89 (0.87, 0.90)	**7.23E-43**	1.02 (0.94, 1.10)	7.20E-01
0.90 (0.88, 0.92)	**5.94E-33**	1.03 (0.95, 1.11)	5.69E-01
0.88 (0.86, 0.89)	**8.41E-50**	0.99 (0.92, 1.07)	8.61E-01
0.88 (0.86, 0.90)	**6.72E-47**	1.01 (0.94, 1.09)	8.42E-01
0.88 (0.86, 0.89)	**1.70E-47**	0.94 (0.88, 1.02)	1.73E-01
0.98 (0.96, 0.99)	**6.08E-03**	1.15 (1.08, 1.23)	**8.24E-05**
1.08 (1.06, 1.10)	**1.18E-16**	0.90 (0.81, 1.01)	1.08E-01
1.10 (1.08, 1.12)	**8.63E-23**	0.88 (0.79, 0.99)	5.65E-02
1.11 (1.09, 1.13)	**8.97E-28**	0.89 (0.79, 0.99)	6.60E-02
1.08 (1.06, 1.10)	**3.27E-16**	0.86 (0.77, 0.96)	**1.67E-02**
1.07 (1.05, 1.09)	**4.76E-13**	0.85 (0.75, 0.95)	**9.93E-03**
1.11 (1.09, 1.12)	**7.25E-29**	0.91 (0.83, 1.01)	1.23E-01
1.03 (1.01, 1.05)	**2.77E-04**	1.12 (1.05, 1.20)	**2.41E-03**
1.05 (1.03, 1.07)	**6.78E-08**	0.83 (0.73, 0.93)	**4.69E-03**
1.06 (1.04, 1.08)	**2.03E-09**	0.83 (0.74, 0.94)	**6.20E-03**
1.06 (1.04, 1.08)	**7.75E-10**	0.86 (0.77, 0.96)	**1.56E-02**
1.05 (1.03, 1.07)	**4.75E-08**	0.80 (0.71, 0.90)	**1.30E-03**
1.05 (1.03, 1.07)	**3.36E-07**	0.79 (0.70, 0.89)	**7.26E-04**
1.07 (1.05, 1.09)	**3.02E-11**	0.84 (0.75, 0.95)	**8.71E-03**
1.05 (1.04, 1.07)	**2.88E-09**	1.07 (0.99, 1.16)	1.06E-01
0.96 (0.94, 0.98)	**5.52E-05**	0.90 (0.82, 0.99)	**4.34E-02**
0.97 (0.95, 0.98)	**1.80E-04**	0.92 (0.85, 1.01)	1.21E-01
0.97 (0.95, 0.99)	**8.48E-04**	0.95 (0.87, 1.03)	2.83E-01
0.96 (0.94, 0.98)	**3.15E-05**	0.88 (0.80, 0.96)	**1.04E-02**

0.96 (0.94, 0.98)	**1.26E-05**	0.87 (0.79, 0.95)	**7.04E-03**
0.97 (0.95, 0.99)	**1.36E-03**	0.91 (0.82, 0.99)	6.11E-02
1.00 (0.98, 1.02)	9.64E-01	1.11 (1.04, 1.20)	**6.88E-03**
0.85 (0.84, 0.87)	**2.13E-71**	0.95 (0.88, 1.03)	3.16E-01
0.88 (0.87, 0.90)	**7.28E-45**	1.00 (0.92, 1.08)	9.32E-01
0.90 (0.89, 0.92)	**2.24E-32**	1.00 (0.92, 1.08)	9.64E-01
0.86 (0.84, 0.87)	**3.36E-68**	0.94 (0.87, 1.02)	1.88E-01
0.85 (0.84, 0.86)	**1.84E-75**	0.92 (0.85, 1.00)	6.59E-02
0.90 (0.88, 0.91)	**1.14E-34**	1.01 (0.94, 1.09)	8.13E-01
0.97 (0.96, 0.99)	**2.74E-03**	1.18 (1.10, 1.27)	**4.61E-05**
1.03 (1.01, 1.05)	**1.39E-03**	1.19 (1.06, 1.33)	**6.46E-03**
1.02 (1.00, 1.04)	**3.03E-02**	0.92 (0.83, 1.03)	1.95E-01
1.01 (0.99, 1.03)	2.06E-01	0.93 (0.84, 1.04)	2.62E-01
1.03 (1.01, 1.05)	**8.01E-04**	0.92 (0.83, 1.02)	1.86E-01
0.98 (0.96, 0.99)	**4.02E-03**	1.03 (0.93, 1.13)	6.40E-01
1.03 (1.01, 1.05)	**4.02E-03**	0.81 (0.72, 0.90)	**8.94E-04**
1.02 (1.00, 1.03)	1.01E-01	0.80 (0.72, 0.91)	**1.16E-03**
1.06 (1.05, 1.08)	**7.35E-12**	0.85 (0.77, 0.95)	**6.88E-03**
0.97 (0.95, 0.99)	**1.03E-03**	1.20 (1.08, 1.32)	**1.83E-03**
1.01 (0.99, 1.03)	4.06E-01	1.26 (1.10, 1.44)	**3.17E-03**
1.05 (1.04, 1.07)	**2.29E-09**	0.92 (0.84, 1.00)	8.81E-02
1.05 (1.04, 1.07)	**2.47E-09**	0.87 (0.80, 0.95)	**6.06E-03**
1.04 (1.02, 1.06)	**2.34E-05**	1.08 (0.98, 1.19)	1.58E-01
0.96 (0.94, 0.97)	**6.93E-07**	1.00 (0.91, 1.09)	9.32E-01
1.00 (0.98, 1.01)	7.55E-01	0.87 (0.81, 0.93)	**1.97E-04**
1.00 (0.98, 1.01)	6.00E-01	0.83 (0.77, 0.89)	**3.93E-06**
0.99 (0.98, 1.01)	5.57E-01	0.82 (0.76, 0.88)	**2.02E-06**
1.00 (0.98, 1.01)	7.41E-01	0.85 (0.79, 0.91)	**4.97E-05**
1.00 (0.99, 1.02)	6.25E-01	1.20 (1.12, 1.29)	**7.18E-06**
0.94 (0.92, 0.96)	**4.16E-12**	0.81 (0.75, 0.88)	**1.25E-06**
0.97 (0.96, 0.99)	**1.87E-03**	0.85 (0.79, 0.92)	**8.78E-05**
1.00 (0.98, 1.02)	9.64E-01	0.89 (0.83, 0.96)	**6.01E-03**
0.94 (0.92, 0.96)	**7.64E-12**	0.81 (0.75, 0.87)	**3.44E-07**
1.04 (1.02, 1.06)	**1.68E-05**	1.19 (1.11, 1.28)	**2.16E-05**
1.15 (1.13, 1.17)	**5.46E-57**	1.30 (1.20, 1.40)	**9.43E-10**
0.95 (0.93, 0.96)	**2.35E-10**	0.80 (0.75, 0.85)	**4.09E-10**
0.94 (0.93, 0.96)	**1.38E-11**	0.78 (0.73, 0.84)	**7.91E-11**
0.98 (0.97, 1.00)	**3.44E-02**	0.91 (0.85, 0.97)	**5.82E-03**
1.03 (1.02, 1.05)	**2.52E-04**	1.22 (1.15, 1.30)	**1.77E-08**
1.12 (1.10, 1.14)	**1.69E-42**	1.08 (1.00, 1.17)	6.55E-02
0.89 (0.88, 0.91)	**4.03E-46**	0.81 (0.77, 0.86)	**2.18E-10**
0.87 (0.86, 0.89)	**8.76E-64**	0.80 (0.75, 0.85)	**1.66E-10**
0.99 (0.97, 1.00)	8.60E-02	0.89 (0.83, 0.95)	**1.03E-03**
1.10 (1.08, 1.12)	**3.11E-34**	1.22 (1.15, 1.28)	**5.52E-11**
1.02 (1.01, 1.04)	**1.92E-03**	0.96 (0.89, 1.04)	3.71E-01
0.92 (0.92, 0.93)	**8.81E-77**	0.92 (0.90, 0.95)	**5.44E-08**
0.92 (0.92, 0.93)	**9.01E-99**	0.94 (0.91, 0.98)	**3.90E-03**
0.96 (0.94, 0.98)	**5.31E-07**	0.85 (0.80, 0.89)	**1.29E-08**

1.10 (1.09, 1.11)	**2.53E-71**	1.12 (1.08, 1.15)	**1.75E-10**
1.03 (1.01, 1.04)	**2.99E-03**	1.02 (0.94, 1.10)	7.17E-01
0.92 (0.91, 0.93)	**8.84E-79**	0.92 (0.89, 0.95)	**4.03E-08**
0.91 (0.90, 0.92)	**2.08E-41**	0.97 (0.90, 1.05)	5.29E-01
0.97 (0.95, 0.98)	**1.51E-04**	0.85 (0.80, 0.91)	**5.95E-06**
1.10 (1.09, 1.11)	**2.09E-86**	1.11 (1.08, 1.15)	**3.39E-09**
1.10 (1.09, 1.12)	**3.02E-38**	1.06 (0.97, 1.14)	2.56E-01
0.90 (0.89, 0.91)	**9.16E-89**	0.87 (0.84, 0.91)	**1.01E-09**
0.91 (0.90, 0.92)	**2.74E-34**	0.95 (0.87, 1.02)	2.12E-01
0.95 (0.93, 0.96)	**2.65E-11**	0.88 (0.82, 0.93)	**1.05E-04**
1.07 (1.05, 1.08)	**1.73E-18**	1.10 (1.06, 1.15)	**1.25E-06**
1.10 (1.09, 1.12)	**7.54E-27**	0.99 (0.92, 1.06)	7.68E-01
0.90 (0.89, 0.92)	**3.46E-32**	0.96 (0.90, 1.03)	3.71E-01
0.92 (0.91, 0.94)	**8.36E-28**	0.92 (0.87, 0.98)	**1.99E-02**
0.96 (0.94, 0.98)	**5.03E-06**	1.04 (0.97, 1.12)	2.93E-01
0.97 (0.96, 0.99)	**7.56E-04**	1.05 (1.00, 1.10)	8.61E-02
1.00 (0.98, 1.02)	9.72E-01	1.13 (1.06, 1.20)	**7.25E-04**
1.00 (0.98, 1.01)	9.18E-01	0.88 (0.83, 0.93)	**9.14E-05**
0.99 (0.97, 1.01)	2.06E-01	0.87 (0.82, 0.92)	**2.46E-05**
1.04 (1.02, 1.06)	**5.09E-06**	0.97 (0.91, 1.03)	3.41E-01
1.00 (0.99, 1.02)	8.82E-01	1.09 (1.04, 1.14)	**8.02E-04**
1.06 (1.04, 1.08)	**2.91E-11**	1.19 (1.11, 1.28)	**1.73E-05**
0.96 (0.94, 0.97)	**2.81E-07**	0.83 (0.77, 0.89)	**2.61E-06**
0.96 (0.95, 0.98)	**1.49E-06**	0.84 (0.78, 0.90)	**5.58E-06**
0.96 (0.95, 0.98)	**7.17E-05**	0.89 (0.82, 0.97)	**1.43E-02**
1.03 (1.02, 1.05)	**2.08E-04**	1.11 (1.07, 1.15)	**2.83E-08**
1.11 (1.10, 1.13)	**5.56E-37**	1.01 (0.94, 1.10)	8.13E-01
0.91 (0.90, 0.93)	**1.97E-29**	0.87 (0.81, 0.93)	**3.52E-04**
0.90 (0.89, 0.92)	**5.99E-37**	0.86 (0.81, 0.92)	**1.22E-04**
1.04 (1.03, 1.06)	**2.29E-06**	1.05 (0.97, 1.14)	3.26E-01
1.04 (1.02, 1.06)	**3.02E-06**	1.22 (1.14, 1.30)	**8.09E-09**
0.91 (0.89, 0.93)	**4.84E-28**	1.09 (1.00, 1.18)	8.64E-02
1.00 (0.98, 1.01)	6.36E-01	0.93 (0.86, 1.00)	7.06E-02
1.10 (1.08, 1.12)	**3.91E-25**	0.86 (0.77, 0.96)	**1.24E-02**
0.89 (0.88, 0.91)	**2.32E-34**	0.94 (0.87, 1.01)	1.39E-01
0.92 (0.91, 0.94)	**4.74E-19**	1.06 (0.98, 1.15)	1.89E-01
0.87 (0.86, 0.89)	**1.19E-50**	0.96 (0.89, 1.03)	3.10E-01
0.91 (0.90, 0.93)	**1.62E-24**	0.99 (0.92, 1.07)	8.61E-01
0.89 (0.87, 0.91)	**3.27E-35**	0.93 (0.86, 1.00)	8.18E-02
0.90 (0.88, 0.91)	**4.13E-31**	0.96 (0.89, 1.04)	4.09E-01
0.95 (0.94, 0.97)	**1.73E-08**	1.14 (1.06, 1.22)	**8.94E-04**
0.94 (0.92, 0.96)	**1.58E-12**	1.13 (1.05, 1.21)	**2.41E-03**
1.01 (0.99, 1.02)	4.36E-01	1.18 (1.11, 1.26)	**1.91E-06**
1.00 (0.99, 1.02)	6.08E-01	1.13 (1.05, 1.21)	**2.32E-03**
0.93 (0.91, 0.95)	**1.54E-14**	1.00 (0.93, 1.09)	9.37E-01
0.98 (0.96, 1.00)	**1.52E-02**	1.19 (1.10, 1.28)	**2.92E-05**

0.92 (0.91, 0.94)	**2.97E-17**	0.97 (0.90, 1.06)	5.86E-01
0.95 (0.94, 0.97)	**1.33E-06**	0.97 (0.90, 1.05)	5.85E-01
0.92 (0.91, 0.94)	**1.69E-16**	0.98 (0.90, 1.06)	6.38E-01
0.96 (0.94, 0.98)	**3.45E-05**	0.90 (0.82, 0.99)	**4.90E-02**
0.90 (0.89, 0.92)	**3.37E-29**	1.01 (0.93, 1.09)	9.05E-01
0.94 (0.93, 0.96)	**2.90E-11**	1.06 (0.98, 1.14)	1.89E-01
0.94 (0.92, 0.95)	**1.23E-13**	1.10 (1.02, 1.18)	**1.72E-02**
0.88 (0.86, 0.89)	**4.87E-47**	0.79 (0.72, 0.85)	**6.16E-08**
0.87 (0.86, 0.89)	**4.25E-43**	0.87 (0.79, 0.95)	**4.81E-03**
0.89 (0.88, 0.91)	**7.26E-35**	0.98 (0.90, 1.05)	6.25E-01
0.87 (0.85, 0.88)	**1.83E-49**	0.95 (0.87, 1.03)	2.81E-01
0.89 (0.87, 0.91)	**5.99E-37**	0.99 (0.92, 1.07)	8.79E-01
0.87 (0.85, 0.88)	**4.94E-51**	0.98 (0.91, 1.06)	6.89E-01
0.98 (0.96, 1.00)	**1.73E-02**	1.15 (1.08, 1.23)	**7.70E-05**
0.96 (0.95, 0.98)	**2.34E-05**	1.11 (1.04, 1.19)	**6.06E-03**
0.91 (0.89, 0.93)	**3.37E-24**	0.81 (0.74, 0.89)	**2.92E-05**
0.93 (0.91, 0.94)	**7.49E-18**	0.81 (0.75, 0.87)	**2.55E-07**
0.88 (0.86, 0.90)	**3.93E-42**	0.87 (0.79, 0.95)	**5.16E-03**
0.97 (0.95, 0.98)	**2.27E-05**	0.83 (0.77, 0.89)	**1.20E-06**
0.92 (0.91, 0.94)	**1.75E-22**	0.80 (0.74, 0.85)	**5.24E-09**
1.07 (1.05, 1.09)	**2.09E-15**	1.31 (1.21, 1.41)	**1.58E-10**
1.07 (1.05, 1.08)	**1.87E-15**	1.11 (1.03, 1.19)	**8.52E-03**
1.09 (1.08, 1.11)	**3.20E-45**	1.20 (1.16, 1.24)	**1.64E-28**
1.03 (1.01, 1.05)	**2.65E-04**	1.10 (1.02, 1.18)	**3.29E-02**
1.07 (1.05, 1.08)	**1.07E-17**	1.10 (1.01, 1.19)	**4.45E-02**
1.00 (0.98, 1.01)	7.75E-01	1.09 (1.01, 1.17)	**4.34E-02**
0.97 (0.95, 0.98)	**3.01E-05**	0.88 (0.82, 0.96)	**5.05E-03**
0.93 (0.91, 0.95)	**1.23E-14**	0.82 (0.76, 0.90)	**5.24E-05**
0.98 (0.96, 0.99)	**7.35E-03**	1.04 (0.97, 1.13)	3.35E-01
0.93 (0.92, 0.95)	**8.58E-15**	0.99 (0.92, 1.08)	9.19E-01
0.93 (0.92, 0.95)	**7.48E-16**	1.03 (0.96, 1.12)	4.84E-01
1.04 (1.03, 1.06)	**3.10E-07**	0.99 (0.91, 1.07)	8.18E-01
1.02 (1.01, 1.04)	**9.51E-03**	1.04 (0.96, 1.12)	3.97E-01
1.05 (1.04, 1.06)	**2.28E-26**	1.04 (1.01, 1.08)	**1.32E-02**
1.07 (1.05, 1.08)	**4.30E-20**	1.06 (1.03, 1.09)	**1.34E-03**
1.07 (1.06, 1.09)	**1.75E-21**	1.07 (1.02, 1.12)	**1.04E-02**
1.05 (1.04, 1.06)	**2.93E-24**	1.07 (1.04, 1.09)	**7.78E-07**
0.87 (0.86, 0.89)	**3.57E-56**	0.81 (0.75, 0.87)	**2.85E-07**
1.12 (1.10, 1.13)	**1.35E-38**	1.35 (1.26, 1.45)	**5.90E-14**
1.01 (0.99, 1.03)	5.23E-01	0.84 (0.76, 0.94)	**5.25E-03**
0.94 (0.92, 0.95)	**1.07E-12**	1.03 (0.95, 1.11)	5.91E-01
0.89 (0.87, 0.91)	**5.22E-38**	0.97 (0.90, 1.05)	5.41E-01
0.88 (0.86, 0.90)	**5.90E-45**	0.94 (0.87, 1.01)	1.65E-01
0.87 (0.86, 0.89)	**1.41E-51**	0.97 (0.90, 1.05)	5.04E-01
0.98 (0.96, 1.00)	**2.42E-02**	0.82 (0.74, 0.91)	**7.44E-04**
0.93 (0.91, 0.94)	**2.35E-18**	0.74 (0.68, 0.80)	**1.58E-10**
1.07 (1.06, 1.09)	**9.03E-48**	1.03 (0.96, 1.10)	4.66E-01
0.92 (0.91, 0.94)	**2.73E-19**	1.09 (1.01, 1.18)	**4.27E-02**

0.88 (0.86, 0.90)	**8.79E-45**	0.92 (0.85, 0.99)	5.65E-02
1.00 (0.98, 1.02)	7.70E-01	0.89 (0.80, 0.99)	**4.65E-02**
1.04 (1.03, 1.05)	**3.71E-09**	1.08 (1.03, 1.12)	**1.33E-03**
1.07 (1.06, 1.08)	**3.95E-24**	1.07 (1.02, 1.11)	**4.83E-03**
0.90 (0.88, 0.92)	**3.29E-29**	0.91 (0.84, 0.99)	**4.81E-02**
0.94 (0.92, 0.95)	**2.89E-14**	1.11 (1.03, 1.19)	**1.56E-02**
0.90 (0.88, 0.91)	**2.05E-33**	1.00 (0.93, 1.08)	9.68E-01
0.91 (0.89, 0.93)	**9.62E-23**	0.90 (0.83, 0.98)	**3.63E-02**
0.92 (0.90, 0.94)	**3.11E-19**	0.99 (0.91, 1.07)	8.15E-01
0.93 (0.92, 0.95)	**2.81E-15**	1.11 (1.03, 1.20)	**1.03E-02**
0.88 (0.86, 0.89)	**7.71E-46**	0.97 (0.90, 1.05)	5.69E-01
0.99 (0.98, 1.01)	5.22E-01	0.92 (0.84, 1.01)	1.13E-01
0.90 (0.89, 0.92)	**6.84E-30**	1.02 (0.94, 1.09)	7.62E-01
0.93 (0.92, 0.95)	**2.14E-16**	1.12 (1.04, 1.20)	**7.15E-03**
0.88 (0.86, 0.89)	**5.88E-46**	0.98 (0.90, 1.05)	6.01E-01
0.99 (0.97, 1.01)	2.60E-01	0.89 (0.81, 0.98)	**4.19E-02**
0.93 (0.91, 0.94)	**6.55E-18**	1.03 (0.96, 1.12)	4.79E-01
0.98 (0.96, 1.00)	9.09E-02	0.83 (0.75, 0.92)	**1.92E-03**
1.01 (1.00, 1.03)	1.36E-01	1.01 (0.91, 1.13)	8.61E-01

iseases mortality		Cerebrovascular diseases mortality		
Age >= 60y		Age < 60y		Age >= 60
**Adjusted HR (95% CI)**	**P value**	**Adjusted HR (95% CI)**	**P value**	**Adjusted HR (95% CI)**
0.99 (0.95, 1.04)	8.69E-01	0.90 (0.77, 1.05)	3.55E-01	0.94 (0.87, 1.02)
0.99 (0.95, 1.04)	7.81E-01	0.90 (0.77, 1.04)	3.33E-01	0.94 (0.87, 1.01)
1.00 (0.95, 1.05)	9.03E-01	0.91 (0.78, 1.06)	3.94E-01	0.94 (0.87, 1.02)
0.98 (0.93, 1.03)	3.68E-01	0.90 (0.77, 1.05)	3.81E-01	0.94 (0.87, 1.02)
0.97 (0.92, 1.02)	2.11E-01	0.90 (0.78, 1.06)	3.94E-01	0.94 (0.87, 1.02)
0.99 (0.94, 1.04)	6.41E-01	0.90 (0.77, 1.05)	3.73E-01	0.95 (0.88, 1.02)
1.00 (0.95, 1.04)	8.93E-01	0.89 (0.76, 1.04)	3.10E-01	0.94 (0.87, 1.01)
0.96 (0.91, 1.01)	1.18E-01	0.85 (0.73, 1.00)	1.97E-01	0.91 (0.84, 0.99)
0.95 (0.91, 1.00)	8.18E-02	0.85 (0.72, 1.00)	1.97E-01	0.91 (0.84, 0.98)
0.95 (0.91, 1.00)	7.62E-02	0.85 (0.73, 1.00)	1.97E-01	0.91 (0.84, 0.99)
0.91 (0.87, 0.96)	**1.41E-03**	0.84 (0.72, 0.99)	1.97E-01	0.90 (0.83, 0.97)
0.89 (0.84, 0.94)	**3.58E-05**	0.84 (0.72, 0.98)	1.97E-01	0.89 (0.83, 0.96)
0.94 (0.90, 0.99)	**3.30E-02**	0.85 (0.73, 1.00)	1.97E-01	0.91 (0.84, 0.99)
0.97 (0.92, 1.02)	2.29E-01	0.85 (0.73, 1.00)	1.97E-01	0.91 (0.85, 0.99)
0.94 (0.89, 0.98)	**1.58E-02**	0.84 (0.72, 0.99)	1.97E-01	0.90 (0.84, 0.98)
0.92 (0.88, 0.97)	**3.39E-03**	0.83 (0.70, 0.97)	1.97E-01	0.90 (0.83, 0.97)
0.94 (0.89, 0.99)	**2.96E-02**	0.85 (0.72, 0.99)	1.97E-01	0.91 (0.84, 0.98)
0.91 (0.86, 0.96)	**4.76E-04**	0.84 (0.72, 0.98)	1.97E-01	0.90 (0.84, 0.98)
0.89 (0.84, 0.93)	**2.05E-05**	0.84 (0.72, 0.98)	1.97E-01	0.91 (0.84, 0.98)
0.93 (0.88, 0.98)	**7.59E-03**	0.84 (0.71, 0.98)	1.97E-01	0.90 (0.83, 0.97)
0.93 (0.88, 0.97)	**4.14E-03**	0.82 (0.70, 0.96)	1.97E-01	0.90 (0.83, 0.97)
0.84 (0.80, 0.89)	**2.03E-09**	0.83 (0.71, 0.97)	1.97E-01	0.88 (0.81, 0.95)
0.85 (0.81, 0.90)	**1.55E-08**	0.82 (0.70, 0.96)	1.97E-01	0.88 (0.81, 0.95)
0.84 (0.80, 0.89)	**1.77E-09**	0.84 (0.72, 0.97)	1.97E-01	0.88 (0.81, 0.95)
0.82 (0.77, 0.86)	**5.11E-12**	0.87 (0.75, 1.00)	1.97E-01	0.89 (0.82, 0.96)
0.81 (0.77, 0.86)	**3.59E-12**	0.89 (0.77, 1.03)	2.49E-01	0.90 (0.83, 0.97)
0.83 (0.79, 0.88)	**2.42E-10**	0.84 (0.73, 0.98)	1.97E-01	0.88 (0.81, 0.95)
0.91 (0.86, 0.95)	**3.43E-04**	0.82 (0.70, 0.97)	1.97E-01	0.90 (0.83, 0.97)
0.93 (0.88, 0.98)	**9.24E-03**	0.88 (0.75, 1.02)	2.45E-01	0.93 (0.86, 1.00)
0.92 (0.87, 0.97)	**1.68E-03**	0.87 (0.75, 1.01)	2.44E-01	0.92 (0.85, 0.99)
0.88 (0.83, 0.92)	**3.44E-06**	0.86 (0.74, 1.00)	1.97E-01	0.90 (0.83, 0.97)
0.88 (0.83, 0.93)	**1.19E-05**	0.88 (0.76, 1.02)	2.45E-01	0.90 (0.83, 0.98)
0.90 (0.85, 0.95)	**3.44E-04**	0.89 (0.77, 1.03)	2.85E-01	0.91 (0.84, 0.99)
0.85 (0.80, 0.90)	**1.38E-08**	0.86 (0.74, 1.00)	1.97E-01	0.89 (0.82, 0.96)
0.98 (0.93, 1.03)	4.44E-01	0.90 (0.77, 1.04)	3.30E-01	0.95 (0.88, 1.03)
0.93 (0.88, 0.99)	**2.06E-02**	0.97 (0.84, 1.12)	8.58E-01	0.94 (0.87, 1.02)
0.94 (0.89, 0.99)	**3.77E-02**	0.97 (0.84, 1.12)	8.49E-01	0.95 (0.88, 1.03)
0.97 (0.92, 1.03)	3.68E-01	0.97 (0.84, 1.12)	8.35E-01	0.97 (0.90, 1.05)
0.88 (0.83, 0.93)	**2.54E-05**	0.97 (0.84, 1.12)	8.53E-01	0.93 (0.86, 1.01)
0.86 (0.81, 0.91)	**1.22E-06**	0.97 (0.84, 1.13)	8.93E-01	0.92 (0.85, 1.00)

0.93 (0.88, 0.98)	**1.22E-02**	0.96 (0.83, 1.11)	7.68E-01	0.95 (0.88, 1.03)
1.05 (1.00, 1.11)	**4.39E-02**	0.98 (0.85, 1.13)	9.25E-01	1.00 (0.93, 1.07)
0.83 (0.79, 0.88)	**4.54E-10**	0.92 (0.79, 1.06)	4.32E-01	0.86 (0.79, 0.93)
0.82 (0.77, 0.86)	**1.32E-11**	0.93 (0.80, 1.07)	5.01E-01	0.86 (0.79, 0.94)
0.83 (0.79, 0.88)	**2.07E-09**	0.94 (0.81, 1.09)	6.07E-01	0.88 (0.81, 0.96)
0.80 (0.75, 0.84)	**5.22E-14**	0.92 (0.79, 1.06)	4.50E-01	0.85 (0.78, 0.93)
0.79 (0.74, 0.83)	**6.52E-15**	0.92 (0.79, 1.06)	4.32E-01	0.84 (0.78, 0.92)
0.82 (0.78, 0.87)	**1.07E-10**	0.93 (0.80, 1.07)	5.14E-01	0.88 (0.81, 0.95)
1.06 (1.01, 1.11)	**3.00E-02**	1.01 (0.87, 1.16)	9.51E-01	0.99 (0.92, 1.07)
0.84 (0.80, 0.89)	**3.80E-09**	0.86 (0.74, 1.00)	1.97E-01	0.88 (0.81, 0.95)
0.80 (0.76, 0.85)	**1.31E-13**	0.88 (0.76, 1.01)	2.44E-01	0.83 (0.77, 0.90)
0.80 (0.76, 0.85)	**2.80E-14**	0.88 (0.76, 1.01)	2.44E-01	0.83 (0.77, 0.90)
0.79 (0.75, 0.84)	**7.72E-15**	0.87 (0.75, 1.01)	2.31E-01	0.83 (0.76, 0.90)
0.80 (0.75, 0.84)	**1.56E-14**	0.86 (0.75, 1.00)	1.97E-01	0.83 (0.76, 0.90)
0.79 (0.75, 0.84)	**4.53E-15**	0.90 (0.78, 1.04)	2.97E-01	0.84 (0.77, 0.91)
1.05 (1.00, 1.10)	6.08E-02	0.99 (0.86, 1.14)	9.38E-01	0.97 (0.90, 1.05)
0.83 (0.78, 0.87)	**2.67E-11**	0.86 (0.74, 1.00)	1.97E-01	0.85 (0.78, 0.92)
0.82 (0.78, 0.87)	**2.69E-12**	0.84 (0.72, 0.97)	1.97E-01	0.82 (0.76, 0.89)
0.82 (0.78, 0.87)	**5.15E-12**	0.84 (0.72, 0.97)	1.97E-01	0.82 (0.76, 0.89)
0.81 (0.77, 0.85)	**7.32E-14**	0.84 (0.72, 0.97)	1.97E-01	0.82 (0.76, 0.89)
0.82 (0.77, 0.86)	**7.38E-13**	0.83 (0.72, 0.97)	1.97E-01	0.82 (0.76, 0.89)
0.80 (0.76, 0.84)	**8.80E-15**	0.86 (0.74, 0.99)	1.97E-01	0.82 (0.76, 0.89)
1.03 (0.98, 1.08)	2.84E-01	0.95 (0.82, 1.10)	7.25E-01	0.95 (0.88, 1.03)
0.85 (0.80, 0.89)	**5.04E-09**	0.85 (0.73, 0.99)	1.97E-01	0.86 (0.79, 0.93)
0.84 (0.79, 0.88)	**2.33E-10**	0.84 (0.72, 0.97)	1.97E-01	0.84 (0.78, 0.91)
0.86 (0.81, 0.91)	**5.11E-08**	0.85 (0.73, 0.98)	1.97E-01	0.86 (0.79, 0.92)
0.82 (0.78, 0.86)	**1.51E-12**	0.84 (0.72, 0.97)	1.97E-01	0.83 (0.77, 0.90)
0.82 (0.78, 0.87)	**8.34E-12**	0.83 (0.72, 0.97)	1.97E-01	0.83 (0.77, 0.90)
0.82 (0.78, 0.87)	**3.55E-12**	0.86 (0.74, 0.99)	1.97E-01	0.84 (0.78, 0.91)
1.00 (0.96, 1.05)	9.01E-01	0.92 (0.79, 1.07)	4.93E-01	0.95 (0.88, 1.02)
0.96 (0.90, 1.03)	2.74E-01	1.15 (0.98, 1.34)	2.45E-01	1.09 (1.01, 1.18)
0.99 (0.93, 1.05)	7.10E-01	1.17 (1.00, 1.36)	1.97E-01	1.11 (1.03, 1.20)
1.00 (0.94, 1.07)	9.52E-01	1.18 (1.02, 1.38)	1.97E-01	1.12 (1.04, 1.22)
0.97 (0.91, 1.03)	3.08E-01	1.14 (0.98, 1.34)	2.45E-01	1.10 (1.01, 1.19)
0.95 (0.89, 1.01)	1.46E-01	1.14 (0.97, 1.33)	2.49E-01	1.10 (1.01, 1.19)
1.02 (0.96, 1.08)	6.39E-01	1.15 (0.99, 1.33)	2.31E-01	1.10 (1.02, 1.19)
1.01 (0.96, 1.06)	7.15E-01	1.03 (0.90, 1.18)	8.53E-01	1.01 (0.94, 1.09)
0.92 (0.86, 0.98)	**1.45E-02**	1.15 (0.97, 1.35)	2.49E-01	1.08 (0.99, 1.17)
0.93 (0.87, 0.99)	**4.09E-02**	1.15 (0.98, 1.35)	2.45E-01	1.08 (1.00, 1.17)
0.94 (0.88, 1.00)	7.39E-02	1.15 (0.98, 1.35)	2.45E-01	1.08 (0.99, 1.17)
0.92 (0.86, 0.98)	**2.11E-02**	1.14 (0.97, 1.34)	2.57E-01	1.09 (1.00, 1.18)
0.92 (0.86, 0.98)	**1.49E-02**	1.14 (0.97, 1.34)	2.77E-01	1.09 (1.00, 1.18)
0.94 (0.88, 1.00)	6.96E-02	1.15 (0.98, 1.36)	2.45E-01	1.08 (1.00, 1.17)
1.00 (0.95, 1.05)	9.72E-01	1.11 (0.97, 1.27)	2.85E-01	1.03 (0.95, 1.11)
0.86 (0.81, 0.91)	**6.95E-07**	1.02 (0.88, 1.20)	9.19E-01	0.95 (0.88, 1.03)
0.87 (0.83, 0.93)	**9.11E-06**	1.02 (0.87, 1.19)	9.25E-01	0.95 (0.88, 1.03)
0.89 (0.84, 0.94)	**6.24E-05**	1.02 (0.87, 1.18)	9.28E-01	0.95 (0.88, 1.03)
0.86 (0.81, 0.91)	**1.01E-06**	1.02 (0.87, 1.19)	9.25E-01	0.96 (0.88, 1.04)

0.86 (0.81, 0.91)	**1.06E-06**	1.02 (0.87, 1.19)	9.28E-01	0.96 (0.88, 1.04)
0.86 (0.81, 0.91)	**1.57E-06**	1.03 (0.88, 1.21)	8.87E-01	0.96 (0.88, 1.04)
0.99 (0.95, 1.05)	8.71E-01	1.01 (0.87, 1.16)	9.45E-01	0.96 (0.89, 1.04)
0.81 (0.77, 0.85)	**2.32E-14**	0.85 (0.73, 0.98)	1.97E-01	0.82 (0.76, 0.89)
0.84 (0.80, 0.89)	**6.61E-10**	0.88 (0.76, 1.02)	2.45E-01	0.84 (0.78, 0.91)
0.86 (0.81, 0.90)	**2.00E-08**	0.90 (0.78, 1.05)	3.61E-01	0.86 (0.79, 0.93)
0.81 (0.77, 0.85)	**4.14E-14**	0.85 (0.74, 0.99)	1.97E-01	0.83 (0.76, 0.89)
0.81 (0.77, 0.85)	**1.37E-14**	0.84 (0.73, 0.98)	1.97E-01	0.82 (0.76, 0.89)
0.84 (0.80, 0.89)	**1.49E-09**	0.91 (0.78, 1.05)	3.94E-01	0.86 (0.79, 0.93)
1.01 (0.96, 1.07)	6.28E-01	0.94 (0.81, 1.09)	6.10E-01	0.94 (0.87, 1.01)
1.13 (1.06, 1.19)	**9.97E-05**	0.96 (0.81, 1.14)	8.35E-01	1.13 (1.04, 1.22)
0.97 (0.91, 1.03)	3.17E-01	1.02 (0.87, 1.20)	9.19E-01	1.10 (1.03, 1.18)
0.95 (0.89, 1.01)	9.54E-02	0.99 (0.84, 1.17)	9.45E-01	1.07 (1.00, 1.15)
1.01 (0.96, 1.07)	6.43E-01	1.09 (0.94, 1.26)	4.50E-01	1.13 (1.06, 1.20)
1.00 (0.94, 1.05)	9.01E-01	0.99 (0.84, 1.16)	9.43E-01	0.89 (0.83, 0.95)
0.95 (0.89, 1.00)	8.78E-02	1.18 (1.02, 1.36)	1.97E-01	1.07 (0.99, 1.16)
0.94 (0.88, 1.00)	5.80E-02	1.16 (1.01, 1.34)	1.97E-01	1.05 (0.97, 1.14)
0.98 (0.93, 1.04)	6.39E-01	1.19 (1.03, 1.37)	1.97E-01	1.13 (1.05, 1.22)
1.06 (1.00, 1.12)	7.16E-02	0.85 (0.73, 0.98)	1.97E-01	0.93 (0.86, 1.01)
1.07 (1.00, 1.14)	6.18E-02	0.92 (0.79, 1.08)	5.14E-01	0.97 (0.90, 1.05)
1.02 (0.97, 1.08)	4.23E-01	1.11 (0.97, 1.28)	2.89E-01	1.08 (1.00, 1.17)
1.00 (0.94, 1.05)	8.93E-01	1.11 (0.96, 1.29)	3.01E-01	1.10 (1.02, 1.19)
1.10 (1.04, 1.16)	**1.14E-03**	1.10 (0.93, 1.29)	4.50E-01	1.01 (0.93, 1.09)
0.95 (0.90, 1.01)	1.09E-01	0.95 (0.81, 1.12)	7.74E-01	0.96 (0.88, 1.03)
0.91 (0.86, 0.95)	**1.18E-04**	1.01 (0.88, 1.17)	9.28E-01	0.98 (0.91, 1.06)
0.90 (0.85, 0.94)	**4.45E-05**	1.06 (0.92, 1.24)	6.07E-01	1.01 (0.93, 1.09)
0.89 (0.85, 0.94)	**3.02E-05**	1.07 (0.92, 1.25)	5.77E-01	1.01 (0.94, 1.10)
0.91 (0.86, 0.95)	**2.25E-04**	1.04 (0.90, 1.21)	7.75E-01	1.00 (0.92, 1.08)
1.11 (1.06, 1.17)	**4.37E-05**	0.95 (0.82, 1.10)	6.96E-01	1.00 (0.92, 1.08)
0.84 (0.80, 0.89)	**2.64E-10**	0.95 (0.82, 1.10)	6.96E-01	0.92 (0.85, 1.00)
0.89 (0.85, 0.94)	**2.05E-05**	1.00 (0.86, 1.15)	9.87E-01	0.96 (0.89, 1.04)
0.94 (0.90, 0.99)	**2.89E-02**	1.02 (0.89, 1.18)	9.00E-01	0.99 (0.92, 1.07)
0.84 (0.79, 0.88)	**5.41E-11**	0.96 (0.83, 1.12)	8.04E-01	0.93 (0.86, 1.00)
1.14 (1.08, 1.20)	**8.04E-07**	1.02 (0.88, 1.18)	9.25E-01	1.05 (0.98, 1.14)
1.31 (1.25, 1.38)	**2.55E-25**	0.99 (0.85, 1.14)	9.28E-01	1.19 (1.11, 1.29)
0.83 (0.79, 0.87)	**3.31E-14**	0.99 (0.86, 1.15)	9.45E-01	0.93 (0.86, 1.00)
0.82 (0.78, 0.86)	**2.74E-15**	1.00 (0.87, 1.16)	9.92E-01	0.92 (0.85, 0.99)
0.92 (0.88, 0.96)	**4.07E-04**	0.95 (0.83, 1.08)	6.02E-01	1.00 (0.93, 1.08)
1.17 (1.11, 1.22)	**3.35E-10**	1.01 (0.88, 1.17)	9.38E-01	1.05 (0.97, 1.13)
1.25 (1.19, 1.31)	**8.88E-18**	1.13 (0.98, 1.31)	2.45E-01	1.17 (1.09, 1.26)
0.80 (0.76, 0.83)	**1.79E-25**	0.92 (0.81, 1.04)	3.73E-01	0.86 (0.80, 0.92)
0.76 (0.73, 0.79)	**1.74E-33**	0.89 (0.78, 1.02)	2.45E-01	0.83 (0.77, 0.88)
0.95 (0.91, 0.99)	**3.82E-02**	1.01 (0.88, 1.17)	9.28E-01	1.00 (0.93, 1.07)
1.21 (1.17, 1.26)	**8.95E-21**	1.06 (0.94, 1.21)	5.38E-01	1.14 (1.06, 1.22)
1.01 (0.96, 1.05)	8.69E-01	1.06 (0.93, 1.20)	5.87E-01	1.05 (0.98, 1.12)
0.91 (0.89, 0.92)	**2.89E-23**	0.94 (0.86, 1.01)	2.49E-01	0.91 (0.88, 0.94)
0.92 (0.90, 0.94)	**2.74E-15**	0.91 (0.86, 0.97)	7.79E-02	0.91 (0.89, 0.94)
0.87 (0.83, 0.90)	**3.53E-11**	1.07 (0.92, 1.24)	5.93E-01	0.92 (0.86, 0.99)

1.14 (1.11, 1.17)	**2.68E-27**	1.07 (0.97, 1.18)	3.81E-01	1.13 (1.08, 1.18)
1.08 (1.03, 1.12)	**6.84E-04**	1.08 (0.95, 1.23)	4.32E-01	1.03 (0.96, 1.11)
0.90 (0.88, 0.92)	**5.48E-28**	0.92 (0.86, 0.99)	1.97E-01	0.91 (0.88, 0.95)
0.88 (0.85, 0.92)	**8.78E-12**	0.86 (0.78, 0.93)	6.83E-02	0.89 (0.84, 0.95)
0.91 (0.87, 0.95)	**7.71E-05**	1.09 (0.94, 1.27)	4.50E-01	0.98 (0.91, 1.05)
1.13 (1.10, 1.15)	**3.12E-29**	1.08 (0.99, 1.19)	2.45E-01	1.11 (1.06, 1.15)
1.15 (1.11, 1.19)	**1.83E-13**	1.13 (0.98, 1.31)	2.45E-01	1.13 (1.06, 1.20)
0.87 (0.85, 0.89)	**1.74E-33**	0.91 (0.81, 1.01)	2.44E-01	0.89 (0.85, 0.93)
0.86 (0.83, 0.89)	**6.69E-16**	0.87 (0.76, 1.00)	1.97E-01	0.89 (0.83, 0.95)
0.92 (0.88, 0.96)	**4.39E-04**	1.02 (0.88, 1.17)	9.25E-01	0.95 (0.89, 1.02)
1.12 (1.08, 1.17)	**4.00E-09**	1.02 (0.89, 1.16)	9.25E-01	1.07 (1.00, 1.14)
1.03 (0.98, 1.08)	2.28E-01	1.16 (0.97, 1.39)	2.45E-01	1.14 (1.05, 1.25)
0.96 (0.92, 1.01)	1.25E-01	0.87 (0.74, 1.01)	2.44E-01	0.90 (0.83, 0.97)
0.93 (0.89, 0.96)	**2.18E-04**	0.90 (0.80, 1.02)	2.49E-01	0.96 (0.90, 1.03)
1.04 (0.99, 1.08)	1.06E-01	0.93 (0.78, 1.10)	5.93E-01	0.89 (0.82, 0.98)
1.00 (0.96, 1.04)	8.93E-01	0.97 (0.83, 1.12)	8.33E-01	0.93 (0.86, 1.01)
1.09 (1.05, 1.14)	**8.49E-05**	0.99 (0.86, 1.13)	9.28E-01	0.93 (0.86, 1.00)
0.93 (0.89, 0.97)	**8.19E-04**	0.96 (0.84, 1.11)	8.02E-01	1.07 (0.99, 1.16)
0.92 (0.89, 0.96)	**4.79E-04**	0.96 (0.84, 1.10)	7.72E-01	1.07 (0.99, 1.16)
0.96 (0.92, 1.00)	9.54E-02	1.00 (0.87, 1.14)	9.67E-01	1.04 (0.96, 1.12)
1.04 (1.00, 1.08)	5.38E-02	1.06 (0.95, 1.20)	4.93E-01	0.96 (0.88, 1.03)
1.16 (1.10, 1.21)	**6.99E-09**	1.02 (0.88, 1.18)	9.25E-01	1.01 (0.94, 1.09)
0.89 (0.85, 0.94)	**9.13E-06**	0.98 (0.84, 1.14)	9.25E-01	1.00 (0.92, 1.07)
0.92 (0.88, 0.96)	**7.14E-04**	0.98 (0.85, 1.14)	9.25E-01	1.00 (0.93, 1.08)
0.83 (0.79, 0.88)	**8.01E-11**	0.99 (0.84, 1.17)	9.45E-01	0.96 (0.88, 1.05)
1.08 (1.03, 1.14)	**1.63E-03**	1.02 (0.88, 1.18)	9.25E-01	1.00 (0.93, 1.08)
1.07 (1.02, 1.12)	**1.54E-02**	1.13 (0.98, 1.31)	2.49E-01	1.10 (1.02, 1.18)
0.89 (0.85, 0.93)	**4.96E-06**	0.93 (0.81, 1.07)	5.38E-01	0.93 (0.87, 1.01)
0.89 (0.85, 0.94)	**6.16E-06**	0.92 (0.80, 1.05)	4.05E-01	0.92 (0.86, 0.99)
0.99 (0.94, 1.05)	7.76E-01	1.09 (0.94, 1.26)	4.50E-01	1.05 (0.97, 1.13)
1.13 (1.07, 1.18)	**7.44E-06**	0.99 (0.85, 1.16)	9.45E-01	1.02 (0.94, 1.10)
0.93 (0.89, 0.98)	**1.17E-02**	0.77 (0.66, 0.91)	7.77E-02	0.87 (0.81, 0.94)
0.98 (0.94, 1.03)	5.36E-01	1.00 (0.87, 1.16)	9.87E-01	1.06 (0.98, 1.14)
0.97 (0.92, 1.03)	4.23E-01	1.19 (1.01, 1.40)	1.97E-01	1.13 (1.05, 1.23)
0.78 (0.74, 0.83)	**1.03E-15**	0.89 (0.77, 1.03)	2.68E-01	0.85 (0.78, 0.92)
0.87 (0.83, 0.92)	**1.44E-06**	0.87 (0.75, 1.01)	2.29E-01	0.90 (0.83, 0.97)
0.80 (0.76, 0.84)	**1.21E-14**	0.86 (0.74, 0.99)	1.97E-01	0.82 (0.76, 0.89)
0.82 (0.78, 0.87)	**2.55E-11**	0.88 (0.76, 1.02)	2.45E-01	0.87 (0.80, 0.94)
0.78 (0.74, 0.83)	**3.59E-16**	0.89 (0.77, 1.03)	2.85E-01	0.85 (0.78, 0.92)
0.80 (0.75, 0.84)	**3.38E-14**	0.88 (0.76, 1.02)	2.45E-01	0.86 (0.79, 0.93)
0.97 (0.92, 1.02)	2.44E-01	0.88 (0.75, 1.03)	2.49E-01	0.93 (0.86, 1.00)
0.96 (0.91, 1.01)	9.69E-02	0.85 (0.73, 1.00)	1.97E-01	0.92 (0.85, 0.99)
1.04 (0.99, 1.09)	1.46E-01	0.97 (0.84, 1.12)	8.58E-01	0.96 (0.89, 1.04)
1.00 (0.95, 1.06)	9.01E-01	1.01 (0.88, 1.16)	9.39E-01	0.97 (0.90, 1.04)
0.84 (0.79, 0.89)	**2.72E-09**	0.94 (0.80, 1.09)	5.93E-01	0.89 (0.82, 0.97)
1.05 (1.00, 1.10)	7.10E-02	0.88 (0.75, 1.03)	2.57E-01	0.96 (0.89, 1.04)

0.81 (0.77, 0.86)	**5.15E-12**	0.94 (0.81, 1.10)	6.51E-01	0.89 (0.82, 0.97)
0.86 (0.81, 0.91)	**3.79E-07**	0.98 (0.85, 1.15)	9.28E-01	0.91 (0.84, 0.99)
0.82 (0.77, 0.87)	**2.55E-11**	0.93 (0.80, 1.09)	5.93E-01	0.89 (0.82, 0.97)
0.85 (0.80, 0.90)	**1.01E-07**	1.02 (0.87, 1.19)	9.25E-01	0.95 (0.87, 1.03)
0.84 (0.79, 0.88)	**8.10E-10**	0.87 (0.75, 1.00)	1.98E-01	0.86 (0.80, 0.94)
0.94 (0.89, 0.99)	**2.80E-02**	0.89 (0.76, 1.04)	2.87E-01	0.92 (0.85, 0.99)
0.91 (0.86, 0.96)	**4.24E-04**	0.89 (0.77, 1.03)	2.85E-01	0.90 (0.83, 0.97)
0.84 (0.80, 0.89)	**6.91E-10**	0.92 (0.78, 1.07)	4.50E-01	0.94 (0.87, 1.01)
0.80 (0.75, 0.85)	**1.94E-11**	0.91 (0.77, 1.07)	4.50E-01	0.91 (0.84, 0.99)
0.79 (0.74, 0.83)	**1.03E-15**	0.86 (0.74, 1.00)	1.97E-01	0.89 (0.82, 0.96)
0.83 (0.79, 0.89)	**6.21E-09**	0.87 (0.75, 1.03)	2.49E-01	0.88 (0.81, 0.96)
0.80 (0.76, 0.85)	**5.15E-14**	0.85 (0.73, 0.99)	1.97E-01	0.87 (0.80, 0.95)
0.79 (0.74, 0.83)	**1.25E-15**	0.84 (0.72, 0.98)	1.97E-01	0.86 (0.79, 0.93)
1.00 (0.95, 1.05)	9.01E-01	0.92 (0.80, 1.07)	4.81E-01	0.95 (0.88, 1.02)
0.94 (0.89, 0.99)	**2.26E-02**	0.92 (0.79, 1.07)	4.50E-01	0.90 (0.84, 0.98)
0.88 (0.83, 0.93)	**1.69E-05**	1.01 (0.87, 1.18)	9.35E-01	0.98 (0.91, 1.06)
0.82 (0.78, 0.86)	**1.70E-14**	0.96 (0.83, 1.11)	7.75E-01	0.99 (0.92, 1.07)
0.88 (0.83, 0.93)	**1.03E-05**	0.93 (0.80, 1.09)	5.94E-01	0.93 (0.86, 1.00)
0.91 (0.87, 0.95)	**9.97E-05**	1.02 (0.88, 1.18)	9.25E-01	1.02 (0.95, 1.10)
0.87 (0.83, 0.91)	**2.70E-08**	1.00 (0.86, 1.16)	9.67E-01	0.99 (0.92, 1.07)
1.18 (1.13, 1.24)	**2.55E-11**	0.98 (0.84, 1.14)	9.19E-01	1.04 (0.97, 1.12)
1.05 (1.00, 1.10)	7.74E-02	1.04 (0.90, 1.20)	8.04E-01	0.96 (0.89, 1.03)
1.17 (1.14, 1.20)	**3.30E-26**	0.99 (0.87, 1.13)	9.38E-01	1.10 (1.04, 1.16)
1.06 (1.01, 1.11)	**2.47E-02**	1.05 (0.91, 1.21)	7.25E-01	0.99 (0.92, 1.07)
1.12 (1.08, 1.16)	**1.07E-10**	1.09 (0.94, 1.26)	4.50E-01	1.06 (0.99, 1.13)
1.08 (1.03, 1.13)	**1.74E-03**	0.98 (0.84, 1.13)	9.00E-01	1.03 (0.95, 1.10)
0.94 (0.90, 0.99)	**1.92E-02**	1.09 (0.94, 1.26)	4.50E-01	1.12 (1.04, 1.20)
0.91 (0.86, 0.96)	**1.82E-03**	1.00 (0.86, 1.16)	9.92E-01	0.99 (0.91, 1.07)
1.06 (1.01, 1.11)	**2.06E-02**	1.02 (0.88, 1.18)	9.23E-01	0.94 (0.87, 1.02)
1.04 (0.99, 1.09)	1.46E-01	0.91 (0.77, 1.06)	4.32E-01	0.88 (0.81, 0.95)
1.05 (1.00, 1.10)	6.32E-02	0.93 (0.79, 1.08)	5.24E-01	0.88 (0.81, 0.95)
1.11 (1.06, 1.16)	**7.65E-06**	1.02 (0.90, 1.16)	9.00E-01	1.02 (0.95, 1.10)
1.09 (1.04, 1.14)	**9.07E-04**	1.00 (0.87, 1.16)	9.92E-01	1.00 (0.93, 1.08)
1.05 (1.02, 1.08)	**5.70E-04**	1.09 (1.05, 1.14)	**9.41E-03**	1.06 (1.01, 1.10)
1.10 (1.06, 1.14)	**2.33E-07**	1.06 (1.02, 1.12)	1.97E-01	1.07 (1.01, 1.14)
1.09 (1.04, 1.13)	**9.51E-05**	1.09 (1.03, 1.15)	7.77E-02	1.07 (1.00, 1.14)
1.07 (1.05, 1.09)	**3.05E-14**	1.05 (0.97, 1.14)	4.50E-01	1.07 (1.04, 1.11)
0.86 (0.82, 0.90)	**6.48E-09**	0.90 (0.78, 1.05)	3.45E-01	0.91 (0.84, 0.98)
1.21 (1.15, 1.26)	**1.74E-13**	1.03 (0.89, 1.20)	8.53E-01	1.08 (1.00, 1.16)
0.91 (0.85, 0.97)	**4.36E-03**	1.07 (0.91, 1.25)	6.10E-01	1.09 (1.01, 1.18)
1.05 (1.00, 1.10)	5.89E-02	0.94 (0.80, 1.09)	6.04E-01	0.88 (0.82, 0.96)
0.81 (0.76, 0.85)	**1.93E-13**	0.87 (0.75, 1.00)	2.00E-01	0.84 (0.78, 0.91)
0.80 (0.75, 0.84)	**1.21E-14**	0.87 (0.75, 1.00)	1.97E-01	0.83 (0.77, 0.90)
0.80 (0.76, 0.85)	**3.16E-14**	0.85 (0.73, 0.99)	1.97E-01	0.82 (0.76, 0.89)
0.85 (0.80, 0.90)	**5.27E-07**	1.05 (0.89, 1.23)	7.97E-01	0.99 (0.91, 1.08)
0.84 (0.80, 0.89)	**4.93E-10**	1.02 (0.87, 1.20)	9.19E-01	0.98 (0.91, 1.06)
1.05 (1.01, 1.09)	**1.14E-02**	1.03 (0.89, 1.18)	8.90E-01	1.07 (1.03, 1.12)
0.89 (0.85, 0.94)	**4.23E-05**	0.85 (0.73, 0.99)	1.97E-01	0.90 (0.83, 0.97)

0.80 (0.75, 0.84)	**6.52E-15**	0.88 (0.76, 1.02)	2.45E-01	0.83 (0.77, 0.90)
0.87 (0.82, 0.93)	**3.54E-05**	1.07 (0.91, 1.26)	6.00E-01	1.00 (0.91, 1.09)
1.05 (1.02, 1.08)	**6.23E-03**	0.94 (0.79, 1.13)	7.25E-01	1.02 (0.95, 1.09)
1.08 (1.04, 1.12)	**4.71E-05**	1.08 (1.03, 1.13)	7.77E-02	1.09 (1.03, 1.15)
0.80 (0.75, 0.84)	**3.58E-14**	0.93 (0.80, 1.09)	5.69E-01	0.88 (0.80, 0.95)
0.91 (0.86, 0.95)	**4.24E-04**	0.88 (0.75, 1.02)	2.45E-01	0.91 (0.84, 0.98)
0.84 (0.79, 0.88)	**4.65E-10**	0.86 (0.74, 0.99)	1.97E-01	0.87 (0.80, 0.94)
0.81 (0.77, 0.86)	**4.73E-12**	0.95 (0.81, 1.11)	7.31E-01	0.89 (0.82, 0.97)
0.82 (0.77, 0.86)	**8.46E-12**	0.92 (0.79, 1.07)	4.50E-01	0.88 (0.81, 0.95)
0.91 (0.87, 0.96)	**1.22E-03**	0.86 (0.74, 1.00)	1.97E-01	0.91 (0.84, 0.98)
0.81 (0.77, 0.86)	**3.78E-13**	0.86 (0.74, 0.99)	1.97E-01	0.83 (0.76, 0.90)
0.88 (0.83, 0.94)	**6.24E-05**	1.05 (0.90, 1.24)	7.25E-01	0.98 (0.90, 1.07)
0.82 (0.78, 0.87)	**7.31E-12**	0.88 (0.76, 1.01)	2.44E-01	0.86 (0.79, 0.93)
0.92 (0.88, 0.97)	**3.00E-03**	0.85 (0.73, 0.99)	1.97E-01	0.90 (0.84, 0.98)
0.81 (0.77, 0.86)	**3.57E-13**	0.86 (0.74, 1.00)	1.97E-01	0.83 (0.77, 0.90)
0.87 (0.81, 0.92)	**8.85E-06**	1.05 (0.90, 1.24)	7.25E-01	0.99 (0.91, 1.07)
0.86 (0.81, 0.91)	**1.84E-07**	0.88 (0.76, 1.02)	2.49E-01	0.90 (0.83, 0.97)
0.85 (0.80, 0.91)	**1.09E-06**	1.05 (0.89, 1.24)	7.52E-01	0.99 (0.91, 1.08)
0.99 (0.93, 1.04)	6.44E-01	0.99 (0.83, 1.18)	9.45E-01	1.00 (0.93, 1.08)

	Cancer mortality			
y	Age < 60y		Age >= 60y	
P value	Adjusted HR (95% CI)	P value	Adjusted HR (95% CI)	P value
1.67E-01	1.04 (1.00, 1.07)	8.08E-02	1.01 (0.99, 1.04)	2.86E-01
1.55E-01	1.03 (1.00, 1.07)	9.03E-02	1.01 (0.99, 1.04)	3.49E-01
2.07E-01	1.04 (1.00, 1.07)	8.06E-02	1.02 (0.99, 1.04)	2.24E-01
1.88E-01	1.03 (1.00, 1.07)	1.01E-01	1.01 (0.99, 1.04)	3.69E-01
1.75E-01	1.03 (1.00, 1.07)	1.27E-01	1.01 (0.98, 1.03)	4.49E-01
2.10E-01	1.04 (1.00, 1.07)	8.27E-02	1.01 (0.99, 1.04)	2.97E-01
1.43E-01	1.03 (1.00, 1.07)	9.37E-02	1.01 (0.99, 1.04)	3.81E-01
**3.92E-02**	1.02 (0.98, 1.05)	4.40E-01	1.00 (0.96, 1.02)	7.38E-01
**3.64E-02**	1.02 (0.98, 1.05)	5.01E-01	0.99 (0.96, 1.02)	6.65E-01
**4.32E-02**	1.02 (0.98, 1.06)	3.68E-01	1.00 (0.96, 1.02)	7.97E-01
**2.03E-02**	1.01 (0.97, 1.05)	7.05E-01	0.99 (0.94, 1.01)	2.84E-01
**1.18E-02**	1.00 (0.96, 1.04)	9.87E-01	0.98 (0.93, 1.00)	8.64E-02
**4.15E-02**	1.02 (0.98, 1.06)	4.04E-01	0.99 (0.96, 1.02)	6.85E-01
**4.76E-02**	1.02 (0.98, 1.05)	4.86E-01	1.00 (0.96, 1.02)	7.81E-01
**2.62E-02**	1.01 (0.97, 1.04)	7.52E-01	0.99 (0.94, 1.01)	3.02E-01
**1.97E-02**	1.00 (0.96, 1.04)	9.73E-01	0.98 (0.94, 1.00)	1.25E-01
**3.56E-02**	1.01 (0.97, 1.05)	6.47E-01	0.99 (0.95, 1.01)	4.34E-01
**2.20E-02**	1.00 (0.97, 1.04)	9.24E-01	0.98 (0.93, 1.00)	1.46E-01
**2.77E-02**	1.00 (0.97, 1.04)	9.73E-01	0.98 (0.93, 1.00)	1.04E-01
**2.03E-02**	1.00 (0.97, 1.04)	8.84E-01	0.98 (0.94, 1.01)	2.15E-01
**1.78E-02**	0.99 (0.96, 1.03)	7.46E-01	0.98 (0.93, 1.00)	6.57E-02
**4.58E-03**	0.98 (0.94, 1.01)	2.78E-01	0.96 (0.91, 0.98)	**7.06E-04**
**4.58E-03**	0.98 (0.94, 1.01)	2.61E-01	0.96 (0.91, 0.98)	**8.62E-04**
**4.72E-03**	0.98 (0.94, 1.01)	2.78E-01	0.96 (0.92, 0.98)	**1.00E-03**
**8.98E-03**	0.97 (0.93, 1.00)	7.99E-02	0.95 (0.92, 0.97)	**6.41E-05**
**1.89E-02**	0.96 (0.93, 0.99)	**3.89E-02**	0.94 (0.93, 0.97)	**3.10E-05**
**5.57E-03**	0.97 (0.94, 1.01)	2.20E-01	0.95 (0.92, 0.98)	**4.87E-04**
**1.74E-02**	0.99 (0.95, 1.02)	5.79E-01	0.97 (0.92, 0.99)	**2.26E-02**
8.37E-02	1.01 (0.98, 1.05)	5.36E-01	0.99 (0.95, 1.01)	3.14E-01
**4.78E-02**	1.01 (0.97, 1.05)	6.96E-01	0.98 (0.94, 1.01)	1.84E-01
**1.66E-02**	0.99 (0.96, 1.03)	6.91E-01	0.97 (0.93, 0.99)	**9.72E-03**
**2.42E-02**	1.00 (0.96, 1.03)	9.09E-01	0.97 (0.94, 1.00)	**3.20E-02**
**4.27E-02**	1.01 (0.97, 1.04)	7.20E-01	0.98 (0.95, 1.00)	1.54E-01
**9.48E-03**	0.98 (0.94, 1.01)	2.78E-01	0.96 (0.92, 0.98)	**8.66E-04**
2.73E-01	1.03 (0.99, 1.06)	2.07E-01	1.00 (0.97, 1.03)	8.82E-01
1.86E-01	1.04 (1.01, 1.08)	**3.09E-02**	1.02 (1.00, 1.04)	2.63E-01
2.99E-01	1.05 (1.01, 1.09)	**1.45E-02**	1.02 (1.01, 1.05)	1.41E-01
5.78E-01	1.06 (1.03, 1.10)	**1.29E-03**	1.03 (1.03, 1.06)	**1.74E-02**
1.20E-01	1.02 (0.99, 1.06)	3.01E-01	1.00 (0.99, 1.03)	9.72E-01
7.85E-02	1.01 (0.98, 1.05)	6.35E-01	0.99 (0.99, 1.02)	6.68E-01

3.28E-01	1.05 (1.01, 1.08)	**2.42E-02**	1.02 (1.01, 1.04)	2.44E-01
9.53E-01	1.07 (1.04, 1.11)	**2.85E-04**	1.04 (1.03, 1.07)	**1.50E-03**
**9.21E-04**	0.96 (0.93, 1.00)	6.47E-02	0.96 (0.94, 0.99)	**9.13E-03**
**1.90E-03**	0.96 (0.92, 0.99)	**3.46E-02**	0.95 (0.94, 0.98)	**1.09E-03**
**8.45E-03**	0.98 (0.94, 1.01)	3.13E-01	0.96 (0.95, 0.99)	**1.17E-02**
**8.42E-04**	0.94 (0.91, 0.97)	**1.77E-03**	0.94 (0.93, 0.97)	**3.84E-05**
**4.08E-04**	0.93 (0.90, 0.97)	**5.68E-04**	0.94 (0.92, 0.97)	**1.55E-05**
**6.62E-03**	0.96 (0.93, 0.99)	**4.15E-02**	0.95 (0.94, 0.98)	**6.04E-04**
9.22E-01	1.08 (1.04, 1.11)	**8.21E-05**	1.05 (1.05, 1.07)	**1.22E-04**
**5.71E-03**	0.96 (0.93, 1.00)	6.16E-02	0.94 (0.92, 0.97)	**7.59E-06**
**9.61E-05**	0.94 (0.90, 0.97)	**9.42E-04**	0.93 (0.90, 0.95)	**1.78E-08**
**8.07E-05**	0.93 (0.90, 0.97)	**4.73E-04**	0.93 (0.90, 0.95)	**2.01E-08**
**7.12E-05**	0.93 (0.90, 0.96)	**2.33E-04**	0.92 (0.89, 0.94)	**8.11E-10**
**6.76E-05**	0.93 (0.90, 0.97)	**3.96E-04**	0.92 (0.89, 0.94)	**7.49E-10**
**1.61E-04**	0.92 (0.89, 0.96)	**6.50E-05**	0.92 (0.90, 0.95)	**3.52E-09**
5.01E-01	1.06 (1.03, 1.10)	**1.21E-03**	1.04 (1.04, 1.06)	**2.75E-03**
**3.19E-04**	0.97 (0.93, 1.00)	9.03E-02	0.94 (0.91, 0.97)	**1.22E-05**
**2.98E-05**	0.94 (0.91, 0.97)	**1.52E-03**	0.93 (0.89, 0.95)	**5.29E-09**
**2.79E-05**	0.93 (0.90, 0.96)	**2.84E-04**	0.92 (0.89, 0.95)	**8.33E-10**
**2.79E-05**	0.93 (0.90, 0.97)	**7.18E-04**	0.92 (0.88, 0.94)	**7.77E-10**
**2.79E-05**	0.94 (0.91, 0.98)	**3.98E-03**	0.93 (0.88, 0.95)	**1.21E-08**
**3.46E-05**	0.92 (0.88, 0.95)	**8.32E-06**	0.91 (0.88, 0.93)	**2.59E-12**
2.58E-01	1.05 (1.01, 1.08)	**1.84E-02**	1.02 (1.01, 1.04)	1.18E-01
**8.42E-04**	0.97 (0.94, 1.01)	1.77E-01	0.95 (0.92, 0.97)	**1.15E-04**
**1.20E-04**	0.95 (0.92, 0.98)	**9.08E-03**	0.93 (0.90, 0.96)	**9.51E-08**
**5.84E-04**	0.95 (0.92, 0.99)	**1.95E-02**	0.94 (0.92, 0.96)	**6.44E-07**
**6.67E-05**	0.94 (0.91, 0.97)	**1.47E-03**	0.93 (0.89, 0.95)	**3.69E-09**
**7.12E-05**	0.95 (0.91, 0.98)	**8.33E-03**	0.93 (0.90, 0.96)	**1.19E-07**
**1.72E-04**	0.92 (0.89, 0.96)	**3.57E-05**	0.91 (0.89, 0.94)	**7.45E-12**
2.05E-01	1.04 (1.00, 1.08)	5.21E-02	1.01 (0.99, 1.04)	3.52E-01
5.32E-02	1.03 (0.99, 1.08)	1.52E-01	1.03 (1.10, 1.05)	9.10E-02
**1.95E-02**	1.04 (1.00, 1.08)	9.37E-02	1.03 (1.12, 1.06)	**2.89E-02**
**9.64E-03**	1.05 (1.01, 1.09)	**4.93E-02**	1.04 (1.13, 1.07)	**6.53E-03**
**4.27E-02**	1.02 (0.98, 1.06)	3.34E-01	1.02 (1.10, 1.05)	2.24E-01
**4.78E-02**	1.01 (0.97, 1.06)	5.99E-01	1.01 (1.09, 1.04)	4.95E-01
**3.38E-02**	1.06 (1.02, 1.10)	**1.24E-02**	1.05 (1.12, 1.07)	**1.45E-03**
8.40E-01	1.07 (1.03, 1.10)	**6.09E-04**	1.04 (1.05, 1.07)	**6.25E-04**
1.06E-01	1.00 (0.96, 1.04)	9.09E-01	1.00 (1.07, 1.03)	8.92E-01
1.00E-01	1.00 (0.96, 1.04)	9.87E-01	1.01 (1.08, 1.03)	6.83E-01
1.26E-01	1.00 (0.96, 1.04)	9.54E-01	1.01 (1.08, 1.04)	5.05E-01
8.17E-02	0.99 (0.95, 1.03)	7.39E-01	1.00 (1.07, 1.03)	8.99E-01
7.85E-02	0.99 (0.95, 1.03)	6.07E-01	0.99 (1.07, 1.02)	7.31E-01
1.01E-01	1.01 (0.97, 1.05)	7.80E-01	1.01 (1.09, 1.04)	4.98E-01
6.02E-01	1.06 (1.03, 1.10)	**2.06E-03**	1.06 (1.07, 1.09)	**5.78E-06**
3.24E-01	0.95 (0.91, 0.98)	**1.35E-02**	0.96 (0.98, 0.99)	**1.29E-02**
3.10E-01	0.95 (0.92, 0.99)	**3.12E-02**	0.97 (0.98, 1.00)	**3.79E-02**
2.82E-01	0.96 (0.93, 1.00)	9.03E-02	0.98 (0.99, 1.00)	1.30E-01
4.01E-01	0.93 (0.90, 0.97)	**2.19E-03**	0.96 (0.98, 0.98)	**1.94E-03**

4.10E-01	0.93 (0.90, 0.97)	**1.19E-03**	0.95 (0.98, 0.98)	**1.05E-03**
3.76E-01	0.95 (0.91, 0.99)	**2.87E-02**	0.97 (0.99, 0.99)	**2.23E-02**
3.70E-01	1.05 (1.01, 1.08)	**2.42E-02**	1.04 (1.02, 1.06)	**2.75E-03**
**2.21E-05**	0.89 (0.86, 0.93)	**9.35E-09**	0.91 (0.87, 0.93)	**2.55E-13**
**1.56E-04**	0.92 (0.89, 0.96)	**8.10E-05**	0.94 (0.90, 0.96)	**7.34E-07**
**7.60E-04**	0.94 (0.90, 0.97)	**1.22E-03**	0.95 (0.92, 0.98)	**1.95E-04**
**3.46E-05**	0.89 (0.86, 0.92)	**2.57E-09**	0.90 (0.87, 0.93)	**3.81E-14**
**2.79E-05**	0.88 (0.85, 0.91)	**9.03E-11**	0.90 (0.86, 0.92)	**3.06E-16**
**7.60E-04**	0.93 (0.90, 0.97)	**6.58E-04**	0.94 (0.91, 0.96)	**1.77E-06**
1.52E-01	1.04 (1.00, 1.08)	5.86E-02	1.02 (0.99, 1.05)	9.27E-02
**1.13E-02**	1.06 (1.02, 1.10)	**1.60E-02**	1.03 (1.05, 1.06)	**2.65E-02**
**1.78E-02**	1.02 (0.98, 1.05)	5.28E-01	1.00 (1.04, 1.02)	9.72E-01
9.59E-02	1.02 (0.98, 1.06)	5.03E-01	0.99 (1.03, 1.02)	6.91E-01
**1.47E-03**	1.01 (0.97, 1.05)	7.09E-01	1.01 (1.05, 1.04)	4.77E-01
**4.12E-03**	0.97 (0.94, 1.01)	1.87E-01	0.99 (0.99, 1.02)	5.58E-01
1.14E-01	0.99 (0.95, 1.03)	5.89E-01	0.99 (1.05, 1.01)	3.69E-01
2.73E-01	0.98 (0.94, 1.02)	3.34E-01	0.98 (1.03, 1.00)	1.29E-01
**3.10E-03**	1.03 (0.99, 1.07)	2.58E-01	1.02 (1.08, 1.05)	1.03E-01
1.19E-01	0.99 (0.96, 1.03)	7.53E-01	1.00 (0.99, 1.03)	8.88E-01
5.46E-01	1.10 (1.06, 1.15)	**8.10E-05**	1.05 (1.03, 1.08)	**5.78E-04**
7.09E-02	1.04 (1.01, 1.08)	**4.02E-02**	1.03 (1.07, 1.05)	6.38E-02
**2.43E-02**	1.03 (0.99, 1.07)	1.60E-01	1.02 (1.07, 1.04)	2.02E-01
9.12E-01	1.07 (1.03, 1.11)	**2.62E-03**	1.04 (1.06, 1.07)	**5.61E-03**
3.28E-01	0.92 (0.89, 0.96)	**2.35E-04**	0.96 (0.97, 0.98)	**1.01E-03**
7.23E-01	1.00 (0.97, 1.04)	9.78E-01	0.99 (1.01, 1.01)	4.52E-01
8.55E-01	0.98 (0.94, 1.01)	2.55E-01	0.97 (1.01, 1.00)	**2.99E-02**
7.87E-01	0.97 (0.94, 1.01)	1.63E-01	0.97 (1.01, 0.99)	**1.61E-02**
9.52E-01	0.99 (0.96, 1.03)	7.05E-01	0.98 (1.01, 1.01)	1.59E-01
9.52E-01	1.02 (0.98, 1.06)	3.71E-01	1.03 (1.02, 1.05)	6.06E-02
7.09E-02	0.92 (0.89, 0.96)	**1.01E-04**	0.93 (0.96, 0.96)	**3.25E-07**
3.83E-01	0.97 (0.94, 1.00)	1.37E-01	0.97 (0.99, 0.99)	**1.06E-02**
8.44E-01	1.01 (0.97, 1.04)	7.21E-01	0.99 (1.02, 1.02)	5.74E-01
1.03E-01	0.92 (0.89, 0.96)	**8.21E-05**	0.94 (0.96, 0.96)	**6.67E-07**
2.47E-01	1.05 (1.01, 1.08)	**2.52E-02**	1.05 (1.06, 1.07)	**7.91E-04**
**6.76E-05**	1.15 (1.10, 1.19)	**1.20E-11**	1.11 (1.17, 1.14)	**2.02E-16**
7.63E-02	0.94 (0.90, 0.97)	**7.18E-04**	0.95 (0.96, 0.97)	**2.66E-05**
**4.57E-02**	0.93 (0.90, 0.96)	**2.01E-04**	0.94 (0.96, 0.97)	**9.19E-06**
9.52E-01	0.99 (0.96, 1.03)	7.91E-01	0.98 (1.00, 1.00)	8.90E-02
2.70E-01	1.05 (1.01, 1.08)	**2.44E-02**	1.04 (1.05, 1.06)	**3.46E-03**
**1.82E-04**	1.15 (1.11, 1.19)	**2.13E-13**	1.08 (1.14, 1.11)	**1.66E-10**
**1.02E-04**	0.89 (0.86, 0.92)	**1.14E-11**	0.91 (0.91, 0.93)	**2.50E-14**
**4.67E-06**	0.86 (0.83, 0.89)	**1.43E-16**	0.90 (0.89, 0.92)	**4.51E-17**
9.53E-01	1.00 (0.96, 1.03)	8.84E-01	0.97 (1.00, 1.00)	**3.18E-02**
**9.75E-04**	1.10 (1.06, 1.13)	**6.65E-08**	1.09 (1.12, 1.11)	**7.39E-12**
2.07E-01	1.00 (0.97, 1.04)	9.92E-01	1.02 (1.04, 1.04)	1.58E-01
**1.43E-06**	0.94 (0.92, 0.95)	**2.57E-09**	0.93 (0.93, 0.94)	**4.23E-22**
**1.43E-06**	0.94 (0.92, 0.96)	**1.03E-06**	0.93 (0.93, 0.95)	**8.89E-22**
**3.64E-02**	0.94 (0.91, 0.97)	**6.09E-04**	0.95 (0.98, 0.97)	**1.04E-04**

**3.87E-06**	1.08 (1.06, 1.11)	**6.18E-10**	1.09 (1.11, 1.11)	**3.58E-20**
4.64E-01	0.94 (0.91, 0.98)	**3.98E-03**	0.99 (1.04, 1.01)	4.81E-01
**2.79E-05**	0.94 (0.92, 0.97)	**7.38E-06**	0.93 (0.93, 0.95)	**8.80E-17**
**8.61E-04**	0.99 (0.96, 1.03)	7.21E-01	0.96 (0.92, 0.99)	**3.46E-03**
5.85E-01	0.93 (0.90, 0.97)	**5.68E-04**	0.95 (0.98, 0.97)	**1.13E-05**
**1.78E-05**	1.09 (1.06, 1.11)	**1.32E-10**	1.09 (1.11, 1.10)	**2.43E-22**
**1.41E-03**	1.05 (1.02, 1.09)	**1.21E-02**	1.04 (1.12, 1.07)	**1.01E-03**
**8.05E-06**	0.91 (0.88, 0.93)	**3.23E-10**	0.92 (0.91, 0.94)	**3.12E-17**
**1.75E-03**	0.96 (0.93, 0.99)	**4.38E-02**	0.97 (0.92, 0.99)	**6.55E-03**
2.49E-01	0.93 (0.90, 0.97)	**2.60E-04**	0.94 (0.96, 0.96)	**5.02E-08**
7.85E-02	1.07 (1.04, 1.10)	**6.00E-05**	1.07 (1.08, 1.09)	**2.91E-09**
**7.75E-03**	1.08 (1.04, 1.13)	**6.29E-04**	1.05 (1.12, 1.08)	**2.78E-04**
**1.65E-02**	0.91 (0.87, 0.94)	**6.68E-06**	0.93 (0.92, 0.96)	**6.81E-08**
3.28E-01	0.91 (0.89, 0.94)	**3.54E-07**	0.92 (0.94, 0.94)	**2.36E-12**
**2.67E-02**	0.97 (0.93, 1.01)	2.02E-01	1.00 (0.98, 1.02)	8.92E-01
1.52E-01	1.00 (0.97, 1.04)	9.39E-01	1.00 (0.99, 1.03)	7.76E-01
9.63E-02	1.01 (0.98, 1.05)	5.12E-01	1.03 (1.02, 1.06)	**1.54E-02**
1.35E-01	0.98 (0.94, 1.01)	2.91E-01	0.96 (1.01, 0.99)	**3.69E-03**
1.21E-01	0.96 (0.93, 1.00)	6.30E-02	0.96 (1.01, 0.98)	**3.22E-04**
4.40E-01	1.06 (1.02, 1.10)	**7.81E-03**	1.01 (1.06, 1.03)	4.52E-01
3.34E-01	1.02 (0.99, 1.06)	2.78E-01	1.03 (1.02, 1.05)	**8.54E-03**
8.24E-01	1.10 (1.06, 1.14)	**6.44E-06**	1.08 (1.08, 1.11)	**6.53E-10**
9.39E-01	0.92 (0.88, 0.95)	**1.69E-05**	0.93 (0.97, 0.95)	**3.69E-09**
9.52E-01	0.92 (0.89, 0.95)	**1.69E-05**	0.93 (0.98, 0.95)	**1.00E-08**
4.26E-01	0.96 (0.92, 0.99)	**4.52E-02**	0.95 (0.98, 0.97)	**1.85E-04**
9.87E-01	1.07 (1.04, 1.10)	**4.96E-05**	1.07 (1.05, 1.09)	**1.31E-07**
**3.68E-02**	1.09 (1.05, 1.13)	**1.50E-05**	1.10 (1.13, 1.12)	**5.60E-13**
1.14E-01	0.89 (0.86, 0.93)	**2.57E-09**	0.90 (0.93, 0.92)	**2.02E-16**
6.08E-02	0.89 (0.86, 0.92)	**2.16E-10**	0.90 (0.92, 0.92)	**4.51E-17**
3.30E-01	1.03 (1.00, 1.07)	1.17E-01	1.01 (1.06, 1.03)	7.31E-01
7.51E-01	1.10 (1.06, 1.14)	**5.36E-06**	1.07 (1.06, 1.10)	**1.31E-07**
**1.97E-03**	0.97 (0.94, 1.01)	2.61E-01	0.97 (0.93, 1.00)	**4.17E-02**
1.97E-01	0.97 (0.94, 1.01)	1.66E-01	0.97 (1.01, 1.00)	**4.99E-02**
**7.49E-03**	1.05 (1.01, 1.10)	**3.12E-02**	1.03 (1.12, 1.06)	**2.73E-02**
**7.57E-04**	0.94 (0.90, 0.97)	**1.13E-03**	0.93 (0.91, 0.95)	**1.67E-07**
**1.74E-02**	1.00 (0.97, 1.04)	9.64E-01	0.98 (0.94, 1.00)	8.53E-02
**4.51E-05**	0.93 (0.90, 0.96)	**3.14E-04**	0.92 (0.89, 0.94)	**6.19E-10**
**1.84E-03**	0.97 (0.94, 1.01)	1.51E-01	0.96 (0.93, 0.98)	**1.90E-03**
**5.96E-04**	0.93 (0.90, 0.97)	**3.96E-04**	0.93 (0.91, 0.95)	**4.83E-08**
**1.47E-03**	0.95 (0.92, 0.99)	**1.57E-02**	0.94 (0.91, 0.96)	**5.85E-06**
8.17E-02	1.02 (0.99, 1.06)	2.61E-01	1.00 (0.97, 1.03)	9.04E-01
5.42E-02	1.01 (0.98, 1.05)	5.33E-01	0.99 (0.96, 1.02)	5.94E-01
3.74E-01	1.06 (1.02, 1.09)	**3.98E-03**	1.03 (1.02, 1.06)	**1.75E-02**
4.75E-01	1.05 (1.02, 1.09)	**1.10E-02**	1.04 (1.02, 1.07)	**1.74E-03**
**2.03E-02**	0.97 (0.93, 1.00)	1.37E-01	0.96 (0.95, 0.99)	**1.15E-02**
3.61E-01	1.04 (1.00, 1.08)	8.06E-02	1.02 (1.00, 1.05)	1.06E-01

**1.74E-02**	0.96 (0.92, 0.99)	**4.08E-02**	0.96 (0.94, 0.98)	**2.28E-03**
5.50E-02	0.97 (0.94, 1.01)	2.35E-01	0.97 (0.97, 1.00)	**4.47E-02**
**2.03E-02**	0.96 (0.93, 1.00)	6.84E-02	0.96 (0.94, 0.98)	**2.83E-03**
2.90E-01	0.95 (0.91, 0.98)	**1.36E-02**	0.96 (0.98, 0.99)	**1.10E-02**
**1.59E-03**	0.97 (0.93, 1.00)	1.14E-01	0.95 (0.92, 0.97)	**1.15E-04**
6.11E-02	1.00 (0.96, 1.03)	9.09E-01	0.98 (0.96, 1.00)	1.26E-01
**1.89E-02**	1.00 (0.97, 1.04)	9.09E-01	0.98 (0.95, 1.01)	1.99E-01
1.49E-01	0.86 (0.82, 0.89)	**1.35E-13**	0.89 (0.89, 0.91)	**1.54E-18**
6.11E-02	0.89 (0.86, 0.93)	**9.04E-08**	0.90 (0.89, 0.92)	**7.50E-14**
**1.25E-02**	0.95 (0.92, 0.98)	**9.08E-03**	0.94 (0.91, 0.97)	**2.74E-05**
**1.10E-02**	0.91 (0.88, 0.95)	**1.78E-05**	0.91 (0.88, 0.93)	**1.82E-12**
**4.12E-03**	0.95 (0.91, 0.98)	**7.98E-03**	0.94 (0.91, 0.97)	**1.13E-05**
**1.41E-03**	0.93 (0.90, 0.97)	**6.09E-04**	0.92 (0.88, 0.95)	**7.14E-09**
2.24E-01	1.04 (1.01, 1.08)	**3.41E-02**	1.01 (1.00, 1.04)	2.74E-01
**2.59E-02**	1.02 (0.99, 1.06)	2.48E-01	1.00 (0.98, 1.03)	7.90E-01
7.31E-01	0.88 (0.85, 0.92)	**1.73E-08**	0.91 (0.93, 0.93)	**9.32E-13**
8.44E-01	0.91 (0.88, 0.95)	**9.07E-06**	0.94 (0.94, 0.96)	**2.73E-07**
1.05E-01	0.88 (0.85, 0.92)	**4.95E-09**	0.90 (0.90, 0.92)	**2.64E-14**
5.97E-01	0.93 (0.89, 0.96)	**9.16E-05**	0.95 (0.98, 0.97)	**2.88E-05**
9.25E-01	0.89 (0.85, 0.92)	**6.18E-10**	0.91 (0.94, 0.93)	**3.54E-13**
3.61E-01	1.12 (1.08, 1.16)	**1.66E-08**	1.08 (1.09, 1.11)	**8.05E-10**
3.52E-01	1.08 (1.05, 1.12)	**4.96E-05**	1.07 (1.08, 1.10)	**2.01E-08**
**3.79E-03**	1.04 (1.01, 1.07)	**2.91E-02**	1.07 (1.11, 1.09)	**6.07E-10**
8.50E-01	1.02 (0.98, 1.06)	3.34E-01	1.03 (1.05, 1.05)	**2.72E-02**
1.13E-01	1.07 (1.03, 1.11)	**9.86E-04**	1.03 (1.08, 1.06)	**1.20E-02**
5.78E-01	0.99 (0.95, 1.03)	6.36E-01	0.98 (1.01, 1.00)	6.06E-02
**9.48E-03**	0.94 (0.91, 0.97)	**1.61E-03**	0.95 (0.98, 0.97)	**7.54E-05**
8.24E-01	0.94 (0.90, 0.97)	**1.99E-03**	0.96 (0.95, 0.98)	**2.04E-03**
2.04E-01	0.99 (0.95, 1.02)	6.12E-01	1.00 (0.99, 1.02)	8.35E-01
**5.54E-03**	0.96 (0.93, 1.00)	6.76E-02	0.97 (0.95, 0.99)	**2.39E-02**
**4.03E-03**	0.97 (0.93, 1.01)	1.55E-01	0.97 (0.95, 1.00)	**3.06E-02**
7.10E-01	1.04 (1.01, 1.06)	**5.55E-03**	1.03 (1.06, 1.05)	**2.95E-02**
9.87E-01	1.04 (1.01, 1.08)	**3.12E-02**	1.01 (1.04, 1.03)	5.32E-01
**2.16E-02**	1.05 (1.03, 1.07)	**2.80E-07**	1.03 (1.06, 1.05)	**3.46E-03**
**4.48E-02**	1.05 (1.03, 1.07)	**6.41E-08**	1.04 (1.08, 1.06)	**7.06E-04**
7.09E-02	1.06 (1.04, 1.09)	**5.30E-07**	1.04 (1.09, 1.06)	**6.81E-04**
**8.07E-05**	1.03 (1.01, 1.06)	**3.61E-02**	1.03 (1.06, 1.05)	**2.01E-02**
**2.64E-02**	0.87 (0.84, 0.90)	**2.65E-12**	0.92 (0.89, 0.94)	**7.39E-12**
7.55E-02	1.16 (1.12, 1.20)	**7.32E-14**	1.13 (1.13, 1.16)	**6.15E-23**
5.21E-02	0.97 (0.93, 1.01)	1.83E-01	0.99 (1.03, 1.02)	4.49E-01
**8.59E-03**	0.97 (0.94, 1.01)	1.78E-01	0.98 (0.95, 1.00)	6.73E-02
**2.01E-04**	0.95 (0.92, 0.98)	**9.08E-03**	0.94 (0.91, 0.96)	**1.67E-06**
**7.12E-05**	0.93 (0.90, 0.96)	**2.60E-04**	0.92 (0.90, 0.95)	**3.69E-09**
**4.21E-05**	0.94 (0.90, 0.97)	**7.97E-04**	0.92 (0.89, 0.95)	**1.36E-09**
9.39E-01	0.94 (0.90, 0.97)	**3.98E-03**	0.96 (1.00, 0.98)	**2.15E-03**
7.41E-01	0.87 (0.84, 0.91)	**3.53E-10**	0.91 (0.94, 0.93)	**3.33E-12**
**5.57E-03**	1.10 (1.07, 1.13)	**6.18E-10**	1.06 (1.09, 1.08)	**2.18E-11**
**1.74E-02**	1.00 (0.97, 1.04)	8.46E-01	0.98 (0.94, 1.00)	1.30E-01

**8.72E-05**	0.92 (0.89, 0.95)	**3.01E-05**	0.92 (0.90, 0.94)	**2.29E-10**
9.52E-01	0.97 (0.93, 1.01)	2.40E-01	0.98 (1.02, 1.01)	2.23E-01
7.31E-01	1.02 (0.99, 1.05)	3.34E-01	1.03 (1.05, 1.05)	**2.23E-02**
**6.31E-03**	1.04 (1.02, 1.07)	**7.18E-04**	1.04 (1.08, 1.06)	**2.75E-03**
**6.31E-03**	0.91 (0.88, 0.95)	**2.08E-05**	0.93 (0.92, 0.95)	**5.98E-08**
**3.35E-02**	1.01 (0.98, 1.05)	5.33E-01	0.99 (0.95, 1.01)	3.41E-01
**1.73E-03**	0.96 (0.93, 1.00)	7.14E-02	0.94 (0.91, 0.97)	**7.59E-06**
**2.03E-02**	0.92 (0.88, 0.95)	**3.43E-05**	0.93 (0.93, 0.96)	**6.35E-07**
**6.16E-03**	0.96 (0.93, 1.00)	7.99E-02	0.96 (0.94, 0.98)	**1.66E-03**
**2.64E-02**	1.01 (0.98, 1.05)	5.68E-01	0.99 (0.95, 1.01)	3.59E-01
**6.58E-05**	0.93 (0.90, 0.97)	**5.68E-04**	0.93 (0.89, 0.95)	**7.33E-09**
7.42E-01	0.97 (0.93, 1.01)	2.08E-01	0.99 (1.01, 1.01)	3.37E-01
**1.01E-03**	0.96 (0.93, 1.00)	7.28E-02	0.95 (0.92, 0.97)	**1.85E-04**
**2.49E-02**	1.01 (0.98, 1.05)	6.32E-01	0.99 (0.95, 1.01)	3.27E-01
**6.67E-05**	0.94 (0.91, 0.97)	**1.19E-03**	0.93 (0.89, 0.95)	**1.00E-08**
8.01E-01	0.96 (0.92, 1.00)	9.37E-02	0.98 (1.01, 1.00)	1.17E-01
**1.95E-02**	1.00 (0.96, 1.03)	9.64E-01	0.98 (0.94, 1.00)	6.57E-02
9.39E-01	0.94 (0.91, 0.98)	**1.20E-02**	0.96 (1.00, 0.99)	**7.39E-03**
9.53E-01	1.07 (1.03, 1.12)	**5.22E-03**	1.03 (1.03, 1.06)	**2.23E-02**

Other mortality			
Age < 60y		Age >= 60y	
**Adjusted HR (95% CI)**	**P value**	**Adjusted HR (95% CI)**	**P value**
0.94 (0.90, 0.98)	**7.17E-03**	0.92 (0.90, 0.95)	**3.41E-08**
0.94 (0.90, 0.98)	**5.69E-03**	0.92 (0.90, 0.95)	**3.74E-08**
0.94 (0.90, 0.98)	**9.37E-03**	0.92 (0.90, 0.95)	**8.52E-08**
0.92 (0.88, 0.96)	**4.67E-04**	0.92 (0.89, 0.94)	**3.23E-09**
0.91 (0.87, 0.95)	**7.98E-05**	0.91 (0.89, 0.94)	**1.62E-09**
0.93 (0.89, 0.98)	**3.62E-03**	0.92 (0.89, 0.95)	**1.25E-08**
0.94 (0.90, 0.99)	**1.10E-02**	0.93 (0.90, 0.95)	**9.83E-08**
0.87 (0.83, 0.91)	**6.84E-09**	0.87 (0.85, 0.90)	**1.47E-19**
0.86 (0.82, 0.91)	**2.63E-09**	0.87 (0.85, 0.90)	**8.45E-20**
0.86 (0.82, 0.91)	**2.63E-09**	0.88 (0.85, 0.90)	**1.89E-18**
0.82 (0.78, 0.86)	**4.63E-15**	0.86 (0.84, 0.89)	**1.09E-23**
0.80 (0.76, 0.84)	**2.08E-19**	0.85 (0.83, 0.88)	**1.23E-26**
0.85 (0.81, 0.90)	**1.77E-10**	0.87 (0.85, 0.90)	**1.79E-19**
0.88 (0.84, 0.92)	**2.42E-07**	0.88 (0.85, 0.90)	**5.17E-18**
0.84 (0.80, 0.88)	**1.01E-12**	0.86 (0.83, 0.88)	**5.13E-25**
0.82 (0.78, 0.86)	**2.74E-15**	0.85 (0.82, 0.87)	**1.36E-28**
0.84 (0.80, 0.88)	**2.87E-12**	0.86 (0.84, 0.89)	**7.12E-24**
0.81 (0.77, 0.85)	**1.33E-17**	0.85 (0.82, 0.87)	**2.72E-28**
0.80 (0.76, 0.84)	**2.35E-19**	0.85 (0.82, 0.87)	**1.09E-27**
0.82 (0.78, 0.86)	**3.48E-15**	0.85 (0.83, 0.87)	**1.93E-27**
0.82 (0.79, 0.86)	**1.17E-14**	0.84 (0.82, 0.87)	**5.47E-29**
0.78 (0.75, 0.82)	**3.71E-23**	0.84 (0.82, 0.87)	**1.82E-30**
0.78 (0.75, 0.82)	**2.27E-23**	0.83 (0.81, 0.86)	**2.49E-34**
0.79 (0.76, 0.83)	**5.85E-22**	0.85 (0.82, 0.87)	**2.26E-28**
0.80 (0.76, 0.84)	**5.24E-21**	0.87 (0.85, 0.90)	**1.61E-19**
0.82 (0.78, 0.86)	**4.59E-18**	0.89 (0.87, 0.92)	**1.78E-13**
0.79 (0.76, 0.83)	**4.77E-22**	0.85 (0.83, 0.88)	**5.23E-26**
0.81 (0.77, 0.85)	**3.47E-17**	0.83 (0.81, 0.86)	**6.29E-33**
0.86 (0.82, 0.90)	**3.80E-10**	0.87 (0.85, 0.90)	**1.09E-20**
0.85 (0.81, 0.89)	**1.93E-11**	0.87 (0.84, 0.89)	**3.78E-22**
0.82 (0.78, 0.86)	**1.59E-16**	0.86 (0.84, 0.89)	**1.02E-23**
0.83 (0.79, 0.87)	**1.15E-14**	0.88 (0.85, 0.90)	**7.86E-19**
0.84 (0.81, 0.88)	**3.44E-12**	0.88 (0.86, 0.91)	**2.41E-16**
0.81 (0.77, 0.85)	**6.50E-19**	0.86 (0.84, 0.89)	**1.04E-22**
0.91 (0.87, 0.95)	**1.17E-04**	0.89 (0.86, 0.91)	**1.71E-16**
0.98 (0.94, 1.02)	4.08E-01	0.97 (0.94, 1.00)	6.59E-02
0.99 (0.95, 1.04)	8.15E-01	0.98 (0.95, 1.01)	1.37E-01
1.01 (0.97, 1.06)	6.11E-01	0.99 (0.97, 1.02)	7.05E-01
0.96 (0.92, 1.00)	7.35E-02	0.97 (0.95, 1.00)	9.62E-02
0.95 (0.91, 0.99)	**3.03E-02**	0.97 (0.94, 1.00)	7.98E-02

0.98 (0.94, 1.02)	4.05E-01	0.98 (0.95, 1.01)	1.68E-01
1.05 (1.00, 1.09)	**4.08E-02**	0.97 (0.95, 1.00)	7.09E-02
0.87 (0.83, 0.90)	**4.25E-10**	0.92 (0.89, 0.94)	**1.17E-08**
0.90 (0.86, 0.94)	**5.15E-06**	0.92 (0.89, 0.95)	**1.93E-07**
0.91 (0.87, 0.95)	**3.67E-05**	0.94 (0.91, 0.97)	**2.86E-05**
0.88 (0.84, 0.92)	**4.75E-08**	0.91 (0.89, 0.94)	**7.89E-09**
0.88 (0.84, 0.92)	**1.54E-08**	0.91 (0.88, 0.94)	**6.06E-10**
0.90 (0.86, 0.94)	**1.86E-06**	0.93 (0.91, 0.96)	**8.16E-06**
1.09 (1.05, 1.14)	**3.44E-05**	1.01 (0.98, 1.03)	6.87E-01
0.81 (0.78, 0.85)	**2.20E-18**	0.87 (0.85, 0.90)	**4.66E-19**
0.82 (0.78, 0.85)	**2.68E-18**	0.86 (0.83, 0.88)	**2.68E-23**
0.81 (0.77, 0.85)	**1.73E-19**	0.86 (0.83, 0.89)	**2.85E-23**
0.80 (0.77, 0.84)	**9.22E-21**	0.85 (0.83, 0.88)	**6.33E-25**
0.80 (0.76, 0.84)	**1.11E-21**	0.85 (0.82, 0.87)	**1.93E-27**
0.82 (0.79, 0.86)	**2.69E-17**	0.88 (0.85, 0.90)	**1.05E-17**
1.08 (1.03, 1.12)	**4.67E-04**	0.99 (0.97, 1.02)	6.53E-01
0.78 (0.75, 0.82)	**3.39E-24**	0.85 (0.82, 0.87)	**3.16E-28**
0.77 (0.74, 0.81)	**3.17E-27**	0.83 (0.80, 0.85)	**2.51E-37**
0.77 (0.73, 0.81)	**5.70E-28**	0.82 (0.80, 0.85)	**2.51E-37**
0.76 (0.73, 0.80)	**5.98E-30**	0.82 (0.80, 0.85)	**3.32E-39**
0.76 (0.73, 0.80)	**2.71E-29**	0.82 (0.80, 0.84)	**3.72E-40**
0.78 (0.74, 0.81)	**1.42E-27**	0.84 (0.81, 0.86)	**6.54E-32**
1.02 (0.98, 1.06)	4.84E-01	0.95 (0.93, 0.98)	**9.79E-04**
0.81 (0.77, 0.85)	**2.61E-19**	0.87 (0.84, 0.89)	**9.33E-22**
0.79 (0.75, 0.83)	**9.97E-24**	0.85 (0.82, 0.87)	**5.61E-29**
0.82 (0.78, 0.85)	**1.67E-18**	0.87 (0.85, 0.90)	**6.28E-21**
0.77 (0.74, 0.81)	**5.70E-28**	0.84 (0.81, 0.86)	**1.57E-32**
0.78 (0.74, 0.81)	**2.48E-26**	0.84 (0.81, 0.86)	**6.29E-33**
0.79 (0.76, 0.82)	**3.95E-26**	0.86 (0.83, 0.88)	**1.99E-25**
0.97 (0.93, 1.01)	1.78E-01	0.93 (0.90, 0.95)	**2.74E-07**
1.28 (1.22, 1.33)	**1.07E-25**	1.18 (1.15, 1.21)	**7.26E-29**
1.30 (1.24, 1.36)	**1.98E-29**	1.20 (1.17, 1.23)	**5.11E-36**
1.32 (1.26, 1.38)	**4.36E-33**	1.21 (1.18, 1.25)	**1.99E-40**
1.26 (1.21, 1.32)	**4.94E-23**	1.18 (1.15, 1.22)	**3.02E-30**
1.26 (1.20, 1.32)	**4.69E-21**	1.18 (1.14, 1.21)	**2.45E-27**
1.27 (1.22, 1.33)	**2.62E-28**	1.20 (1.17, 1.24)	**2.63E-39**
1.10 (1.06, 1.14)	**2.33E-06**	1.02 (1.00, 1.05)	1.22E-01
1.28 (1.22, 1.34)	**4.67E-23**	1.15 (1.12, 1.19)	**2.16E-20**
1.30 (1.23, 1.36)	**2.93E-25**	1.16 (1.13, 1.19)	**5.12E-22**
1.31 (1.25, 1.37)	**5.17E-27**	1.16 (1.12, 1.19)	**1.75E-21**
1.27 (1.21, 1.33)	**3.25E-21**	1.16 (1.12, 1.19)	**1.12E-21**
1.26 (1.20, 1.32)	**1.03E-19**	1.15 (1.12, 1.19)	**9.86E-21**
1.30 (1.24, 1.36)	**3.44E-26**	1.17 (1.14, 1.20)	**1.33E-24**
1.18 (1.13, 1.23)	**1.44E-15**	1.06 (1.03, 1.09)	**3.58E-05**
1.10 (1.05, 1.16)	**7.73E-05**	0.99 (0.96, 1.02)	6.03E-01
1.10 (1.05, 1.16)	**4.21E-05**	0.99 (0.96, 1.02)	4.28E-01
1.10 (1.06, 1.16)	**2.90E-05**	0.98 (0.96, 1.01)	3.34E-01
1.10 (1.05, 1.15)	**1.42E-04**	1.00 (0.97, 1.03)	8.60E-01

1.09 (1.04, 1.15)	**3.92E-04**	0.99 (0.97, 1.03)	7.58E-01
1.13 (1.07, 1.18)	**2.18E-06**	1.01 (0.98, 1.04)	7.11E-01
1.04 (1.00, 1.09)	5.83E-02	0.96 (0.93, 0.98)	**2.36E-03**
0.81 (0.78, 0.85)	**2.88E-18**	0.80 (0.78, 0.83)	**6.40E-48**
0.88 (0.84, 0.92)	**7.79E-08**	0.83 (0.81, 0.86)	**4.73E-34**
0.92 (0.88, 0.96)	**5.20E-04**	0.86 (0.83, 0.88)	**2.12E-25**
0.82 (0.79, 0.86)	**3.77E-16**	0.81 (0.79, 0.84)	**4.05E-43**
0.80 (0.77, 0.84)	**6.36E-21**	0.81 (0.78, 0.83)	**2.04E-47**
0.92 (0.88, 0.97)	**8.38E-04**	0.86 (0.84, 0.89)	**1.64E-22**
0.96 (0.92, 1.00)	8.28E-02	0.91 (0.88, 0.93)	**4.39E-11**
1.10 (1.04, 1.17)	**1.68E-03**	0.99 (0.96, 1.02)	7.22E-01
1.01 (0.96, 1.06)	7.30E-01	1.05 (1.02, 1.08)	**1.14E-03**
0.99 (0.94, 1.05)	8.15E-01	1.04 (1.02, 1.07)	**3.25E-03**
1.05 (1.00, 1.10)	7.70E-02	1.04 (1.02, 1.07)	**2.58E-03**
0.97 (0.92, 1.02)	2.28E-01	0.96 (0.93, 0.99)	**5.18E-03**
1.03 (0.98, 1.09)	2.88E-01	1.09 (1.06, 1.12)	**7.12E-10**
1.01 (0.96, 1.06)	7.64E-01	1.08 (1.05, 1.11)	**1.82E-07**
1.10 (1.05, 1.16)	**1.14E-04**	1.13 (1.09, 1.16)	**1.26E-16**
0.97 (0.92, 1.02)	3.18E-01	0.92 (0.89, 0.94)	**3.03E-09**
0.96 (0.91, 1.01)	1.52E-01	0.95 (0.93, 0.98)	**8.38E-04**
1.08 (1.04, 1.13)	**6.17E-04**	1.10 (1.07, 1.13)	**1.99E-10**
1.09 (1.04, 1.14)	**2.33E-04**	1.11 (1.08, 1.14)	**1.60E-13**
1.05 (1.00, 1.10)	8.04E-02	1.03 (1.00, 1.06)	5.80E-02
0.95 (0.91, 1.00)	7.67E-02	0.96 (0.93, 0.98)	**2.99E-03**
0.99 (0.95, 1.03)	5.99E-01	1.04 (1.01, 1.07)	**8.07E-03**
1.00 (0.95, 1.04)	8.83E-01	1.06 (1.03, 1.09)	**1.46E-04**
1.00 (0.95, 1.04)	8.91E-01	1.06 (1.03, 1.09)	**6.70E-05**
0.99 (0.95, 1.04)	8.48E-01	1.05 (1.02, 1.08)	**1.62E-03**
1.01 (0.96, 1.05)	8.15E-01	0.95 (0.92, 0.98)	**3.20E-04**
0.88 (0.84, 0.92)	**2.24E-08**	0.98 (0.95, 1.01)	2.28E-01
0.92 (0.89, 0.97)	**4.81E-04**	1.01 (0.98, 1.04)	5.45E-01
0.96 (0.92, 1.00)	6.76E-02	1.03 (1.00, 1.06)	5.09E-02
0.88 (0.85, 0.93)	**1.47E-07**	0.98 (0.95, 1.01)	2.48E-01
1.10 (1.05, 1.15)	**3.03E-05**	1.00 (0.97, 1.03)	9.29E-01
1.16 (1.11, 1.22)	**1.65E-10**	1.14 (1.11, 1.17)	**7.85E-20**
0.91 (0.87, 0.94)	**5.02E-06**	0.99 (0.97, 1.02)	6.53E-01
0.91 (0.87, 0.94)	**6.91E-06**	0.99 (0.96, 1.02)	5.62E-01
0.93 (0.89, 0.96)	**8.46E-05**	1.01 (0.98, 1.03)	6.12E-01
1.08 (1.04, 1.13)	**2.07E-04**	0.98 (0.95, 1.01)	1.86E-01
1.11 (1.06, 1.16)	**5.49E-06**	1.13 (1.10, 1.16)	**1.80E-17**
0.85 (0.82, 0.88)	**4.49E-18**	0.91 (0.89, 0.94)	**4.00E-12**
0.83 (0.80, 0.86)	**1.71E-21**	0.89 (0.86, 0.91)	**1.07E-19**
0.94 (0.91, 0.98)	**6.26E-03**	1.01 (0.99, 1.04)	3.81E-01
1.16 (1.12, 1.20)	**1.68E-17**	1.07 (1.04, 1.10)	**1.36E-07**
1.03 (0.99, 1.07)	2.09E-01	1.03 (1.01, 1.06)	**6.17E-03**
0.91 (0.90, 0.93)	**3.43E-34**	0.92 (0.91, 0.94)	**7.67E-29**
0.91 (0.90, 0.92)	**1.18E-44**	0.91 (0.90, 0.92)	**1.95E-60**
0.93 (0.90, 0.97)	**5.89E-04**	1.01 (0.99, 1.04)	4.27E-01

1.13 (1.11, 1.15)	**1.13E-39**	1.10 (1.08, 1.12)	**3.93E-25**
1.06 (1.02, 1.10)	**3.33E-03**	1.05 (1.02, 1.07)	**2.86E-04**
0.90 (0.89, 0.92)	**1.48E-50**	0.92 (0.90, 0.93)	**4.24E-37**
0.85 (0.83, 0.87)	**5.56E-46**	0.88 (0.86, 0.89)	**2.02E-45**
0.95 (0.92, 1.00)	**3.77E-02**	1.02 (0.99, 1.05)	2.11E-01
1.14 (1.12, 1.15)	**5.52E-56**	1.10 (1.08, 1.12)	**1.92E-36**
1.24 (1.19, 1.30)	**2.44E-23**	1.14 (1.12, 1.17)	**5.33E-36**
0.85 (0.83, 0.86)	**1.28E-70**	0.89 (0.88, 0.91)	**1.40E-44**
0.82 (0.79, 0.85)	**1.98E-29**	0.88 (0.86, 0.90)	**1.29E-30**
0.91 (0.87, 0.94)	**6.08E-07**	0.97 (0.95, 1.00)	**3.44E-02**
1.10 (1.08, 1.13)	**3.73E-15**	1.05 (1.02, 1.07)	**4.65E-04**
1.22 (1.16, 1.29)	**5.54E-13**	1.21 (1.17, 1.25)	**7.58E-30**
0.82 (0.78, 0.85)	**8.46E-19**	0.86 (0.83, 0.88)	**5.25E-29**
0.89 (0.86, 0.92)	**4.28E-10**	0.92 (0.90, 0.94)	**1.10E-12**
0.84 (0.80, 0.89)	**2.24E-10**	0.89 (0.86, 0.92)	**1.42E-13**
0.99 (0.95, 1.03)	5.49E-01	0.92 (0.90, 0.95)	**2.40E-07**
1.00 (0.96, 1.04)	9.23E-01	0.94 (0.92, 0.97)	**1.23E-05**
1.02 (0.98, 1.07)	3.18E-01	1.07 (1.04, 1.09)	**6.71E-06**
1.00 (0.96, 1.05)	8.86E-01	1.05 (1.02, 1.08)	**6.60E-04**
1.10 (1.06, 1.16)	**2.89E-05**	1.11 (1.08, 1.15)	**4.22E-13**
0.96 (0.92, 1.01)	1.14E-01	0.95 (0.92, 0.98)	**4.13E-04**
1.02 (0.97, 1.07)	4.56E-01	1.01 (0.98, 1.03)	7.26E-01
0.99 (0.94, 1.03)	6.30E-01	1.01 (0.98, 1.04)	4.10E-01
0.97 (0.93, 1.01)	1.71E-01	1.01 (0.98, 1.04)	6.12E-01
1.10 (1.05, 1.16)	**2.43E-04**	1.03 (1.00, 1.06)	8.63E-02
1.01 (0.96, 1.05)	8.04E-01	0.98 (0.95, 1.00)	1.00E-01
1.31 (1.26, 1.37)	**1.11E-32**	1.15 (1.12, 1.19)	**3.82E-24**
0.83 (0.80, 0.87)	**7.97E-18**	0.92 (0.90, 0.95)	**1.17E-08**
0.81 (0.78, 0.84)	**3.01E-26**	0.90 (0.88, 0.92)	**1.12E-14**
1.19 (1.14, 1.24)	**1.06E-16**	1.11 (1.08, 1.14)	**2.08E-12**
1.04 (0.99, 1.09)	1.23E-01	0.99 (0.96, 1.01)	3.34E-01
0.77 (0.74, 0.81)	**1.17E-24**	0.83 (0.81, 0.86)	**5.44E-37**
1.00 (0.95, 1.04)	9.20E-01	1.02 (0.99, 1.05)	1.84E-01
1.40 (1.34, 1.47)	**2.45E-43**	1.22 (1.19, 1.26)	**1.73E-41**
0.86 (0.82, 0.89)	**2.38E-11**	0.88 (0.86, 0.91)	**4.50E-16**
0.83 (0.79, 0.87)	**2.01E-14**	0.88 (0.85, 0.90)	**6.64E-19**
0.79 (0.75, 0.82)	**2.34E-24**	0.84 (0.82, 0.87)	**8.20E-30**
0.84 (0.81, 0.88)	**2.60E-13**	0.88 (0.86, 0.91)	**3.93E-16**
0.86 (0.82, 0.90)	**5.38E-11**	0.88 (0.86, 0.91)	**1.06E-15**
0.85 (0.82, 0.89)	**7.35E-12**	0.88 (0.85, 0.91)	**1.81E-16**
0.91 (0.87, 0.95)	**6.73E-05**	0.89 (0.86, 0.92)	**1.97E-15**
0.88 (0.84, 0.92)	**5.00E-08**	0.87 (0.85, 0.90)	**8.45E-20**
1.05 (1.01, 1.09)	**3.39E-02**	0.97 (0.95, 1.00)	6.89E-02
1.05 (1.01, 1.10)	**2.18E-02**	0.96 (0.94, 0.99)	**1.29E-02**
1.00 (0.96, 1.05)	9.97E-01	0.92 (0.89, 0.95)	**9.65E-08**
0.89 (0.85, 0.93)	**1.99E-06**	0.91 (0.88, 0.93)	**4.05E-11**

1.00 (0.95, 1.05)	9.95E-01	0.92 (0.89, 0.95)	**4.99E-08**
0.99 (0.95, 1.04)	8.13E-01	0.97 (0.94, 1.00)	**4.96E-02**
0.99 (0.94, 1.03)	5.74E-01	0.92 (0.89, 0.95)	**8.72E-08**
1.10 (1.05, 1.16)	**9.26E-05**	0.99 (0.96, 1.02)	6.58E-01
0.81 (0.78, 0.85)	**2.23E-18**	0.87 (0.85, 0.90)	**5.23E-20**
0.81 (0.77, 0.85)	**6.24E-18**	0.90 (0.88, 0.93)	**2.64E-12**
0.94 (0.90, 0.98)	**7.39E-03**	0.90 (0.87, 0.92)	**3.83E-14**
0.80 (0.76, 0.83)	**1.02E-20**	0.87 (0.84, 0.89)	**2.02E-21**
0.87 (0.82, 0.91)	**7.03E-08**	0.85 (0.83, 0.88)	**1.49E-21**
0.84 (0.80, 0.88)	**6.44E-14**	0.86 (0.84, 0.89)	**1.36E-22**
0.83 (0.79, 0.88)	**3.51E-12**	0.82 (0.80, 0.85)	**1.57E-32**
0.85 (0.81, 0.88)	**1.34E-12**	0.85 (0.83, 0.88)	**6.47E-25**
0.83 (0.79, 0.87)	**3.65E-15**	0.83 (0.80, 0.85)	**6.38E-34**
1.00 (0.96, 1.04)	8.93E-01	0.94 (0.91, 0.96)	**4.15E-06**
0.99 (0.95, 1.03)	6.21E-01	0.93 (0.90, 0.96)	**5.34E-07**
0.91 (0.86, 0.95)	**1.68E-04**	0.91 (0.89, 0.94)	**5.73E-09**
0.85 (0.82, 0.89)	**1.22E-12**	0.95 (0.92, 0.97)	**1.77E-04**
0.82 (0.78, 0.86)	**3.49E-13**	0.85 (0.82, 0.87)	**2.53E-25**
0.91 (0.88, 0.95)	**3.75E-05**	1.00 (0.97, 1.02)	8.51E-01
0.86 (0.83, 0.90)	**1.89E-11**	0.94 (0.92, 0.97)	**8.81E-06**
1.10 (1.06, 1.16)	**2.43E-05**	1.03 (1.00, 1.05)	7.40E-02
1.16 (1.12, 1.21)	**1.90E-12**	1.08 (1.06, 1.11)	**2.55E-09**
1.13 (1.10, 1.15)	**1.89E-22**	1.09 (1.07, 1.11)	**3.91E-17**
1.12 (1.07, 1.17)	**6.10E-07**	1.03 (1.00, 1.06)	**2.83E-02**
1.13 (1.08, 1.18)	**3.39E-07**	1.08 (1.06, 1.10)	**1.15E-13**
0.92 (0.88, 0.97)	**7.81E-04**	1.00 (0.97, 1.02)	8.31E-01
0.91 (0.87, 0.95)	**2.81E-05**	0.97 (0.95, 1.00)	5.96E-02
0.82 (0.78, 0.86)	**3.36E-14**	0.89 (0.86, 0.92)	**4.97E-13**
0.91 (0.87, 0.95)	**1.27E-04**	0.93 (0.90, 0.96)	**8.89E-07**
0.81 (0.77, 0.85)	**1.20E-15**	0.86 (0.84, 0.89)	**4.65E-22**
0.81 (0.77, 0.85)	**1.03E-16**	0.85 (0.83, 0.88)	**1.10E-25**
1.04 (1.01, 1.07)	**1.12E-02**	1.04 (1.02, 1.07)	**1.51E-03**
1.04 (1.00, 1.09)	7.35E-02	1.02 (1.00, 1.05)	1.00E-01
1.10 (1.09, 1.11)	**4.52E-77**	1.07 (1.06, 1.08)	**5.94E-28**
1.06 (1.05, 1.08)	**2.91E-15**	1.08 (1.06, 1.11)	**1.16E-13**
1.09 (1.07, 1.11)	**5.13E-22**	1.10 (1.08, 1.12)	**1.70E-18**
1.06 (1.04, 1.08)	**2.18E-11**	1.05 (1.04, 1.07)	**5.90E-12**
0.74 (0.71, 0.77)	**2.68E-42**	0.82 (0.80, 0.84)	**1.34E-43**
1.13 (1.09, 1.19)	**2.54E-08**	1.07 (1.04, 1.10)	**6.84E-07**
0.99 (0.94, 1.04)	7.67E-01	1.04 (1.01, 1.08)	**8.47E-03**
0.83 (0.79, 0.87)	**2.41E-13**	0.87 (0.84, 0.89)	**3.11E-21**
0.81 (0.77, 0.85)	**2.61E-19**	0.86 (0.83, 0.88)	**2.29E-23**
0.80 (0.76, 0.83)	**7.99E-23**	0.85 (0.83, 0.88)	**7.91E-25**
0.78 (0.75, 0.82)	**6.08E-25**	0.84 (0.81, 0.86)	**3.69E-32**
1.13 (1.08, 1.19)	**2.78E-06**	1.04 (1.01, 1.08)	**1.06E-02**
0.88 (0.83, 0.92)	**2.18E-07**	0.96 (0.93, 0.99)	**6.82E-03**
1.14 (1.11, 1.16)	**2.17E-26**	1.09 (1.08, 1.11)	**3.23E-41**
0.83 (0.79, 0.87)	**4.57E-14**	0.87 (0.84, 0.89)	**8.54E-22**

0.80 (0.77, 0.84)	**4.37E-21**	0.86 (0.84, 0.89)	**3.03E-22**
1.20 (1.14, 1.26)	**1.53E-12**	1.07 (1.04, 1.10)	**2.99E-05**
1.07 (1.04, 1.10)	**6.20E-07**	1.05 (1.03, 1.07)	**1.40E-07**
1.09 (1.08, 1.10)	**1.65E-45**	1.10 (1.08, 1.12)	**9.21E-24**
0.95 (0.91, 1.00)	5.44E-02	0.90 (0.87, 0.93)	**1.30E-11**
0.86 (0.82, 0.90)	**8.59E-10**	0.88 (0.86, 0.91)	**3.56E-17**
0.80 (0.77, 0.84)	**6.51E-21**	0.86 (0.84, 0.89)	**1.54E-22**
0.99 (0.94, 1.03)	6.11E-01	0.92 (0.89, 0.95)	**3.74E-08**
0.95 (0.90, 0.99)	**2.27E-02**	0.91 (0.88, 0.94)	**1.34E-09**
0.85 (0.81, 0.89)	**3.26E-11**	0.87 (0.85, 0.90)	**1.31E-19**
0.80 (0.76, 0.83)	**1.89E-22**	0.85 (0.82, 0.87)	**1.32E-27**
1.17 (1.12, 1.23)	**1.78E-10**	1.04 (1.01, 1.07)	**2.04E-02**
0.88 (0.84, 0.92)	**9.35E-09**	0.87 (0.85, 0.90)	**1.38E-19**
0.85 (0.81, 0.89)	**3.32E-11**	0.87 (0.84, 0.89)	**8.26E-22**
0.80 (0.76, 0.84)	**1.06E-21**	0.85 (0.82, 0.87)	**4.64E-28**
1.16 (1.11, 1.22)	**1.33E-09**	1.04 (1.01, 1.07)	**1.49E-02**
0.84 (0.80, 0.88)	**5.94E-14**	0.89 (0.86, 0.91)	**5.89E-16**
1.15 (1.09, 1.21)	**1.37E-07**	1.05 (1.02, 1.08)	**3.27E-03**
1.00 (0.94, 1.05)	9.20E-01	1.00 (0.97, 1.03)	9.38E-01

Supplementary Table 10. Association of all meta

		CKB	
			**All causes**
		**Urban**	
**Metabolites**	**Group**	**Adjusted HR (95% CI)**	**P value**
Concentration of chylomicrons ar	Lipoprotein subclasses	1.05 (0.97, 1.14)	3.45E-01
Total lipids in chylomicrons and (	Lipoprotein subclasses	1.05 (0.97, 1.14)	3.28E-01
Phospholipids in chylomicrons ar	Lipoprotein subclasses	1.06 (0.98, 1.14)	2.85E-01
Cholesterol in chylomicrons and (	Lipoprotein subclasses	1.09 (1.01, 1.17)	1.14E-01
Cholesteryl esters in chylomicron	Lipoprotein subclasses	1.10 (1.02, 1.18)	7.61E-02
Free cholesterol in chylomicrons	Lipoprotein subclasses	1.07 (0.99, 1.15)	1.87E-01
Triglycerides in chylomicrons and	Lipoprotein subclasses	1.05 (0.97, 1.13)	4.11E-01
Concentration of very large VLD	Lipoprotein subclasses	1.07 (0.99, 1.16)	1.69E-01
Total lipids in very large VLDL	Lipoprotein subclasses	1.08 (1.00, 1.16)	1.62E-01
Phospholipids in very large VLDI	Lipoprotein subclasses	1.08 (1.00, 1.17)	1.52E-01
Cholesterol in very large VLDL	Lipoprotein subclasses	1.09 (1.01, 1.17)	1.11E-01
Cholesteryl esters in very large V	Lipoprotein subclasses	1.09 (1.01, 1.18)	9.42E-02
Free cholesterol in very large VL	Lipoprotein subclasses	1.08 (1.00, 1.17)	1.31E-01
Triglycerides in very large VLDL	Lipoprotein subclasses	1.07 (0.99, 1.16)	1.80E-01
Concentration of large VLDL par	Lipoprotein subclasses	1.07 (0.99, 1.16)	1.70E-01
Total lipids in large VLDL	Lipoprotein subclasses	1.07 (0.99, 1.16)	1.70E-01
Phospholipids in large VLDL	Lipoprotein subclasses	1.07 (0.99, 1.16)	1.70E-01
Cholesterol in large VLDL	Lipoprotein subclasses	1.07 (1.00, 1.16)	1.61E-01
Cholesteryl esters in large VLDL	Lipoprotein subclasses	1.07 (1.00, 1.16)	1.61E-01
Free cholesterol in large VLDL	Lipoprotein subclasses	1.07 (1.00, 1.16)	1.61E-01
Triglycerides in large VLDL	Lipoprotein subclasses	1.07 (0.99, 1.16)	1.74E-01
Concentration of medium VLDL	Lipoprotein subclasses	1.07 (0.99, 1.15)	1.72E-01
Total lipids in medium VLDL	Lipoprotein subclasses	1.07 (0.99, 1.15)	1.70E-01
Phospholipids in medium VLDL	Lipoprotein subclasses	1.07 (0.99, 1.15)	1.76E-01
Cholesterol in medium VLDL	Lipoprotein subclasses	1.08 (1.00, 1.16)	1.31E-01
Cholesteryl esters in medium VLI	Lipoprotein subclasses	1.08 (1.00, 1.16)	1.30E-01
Free cholesterol in medium VLDI	Lipoprotein subclasses	1.08 (1.00, 1.16)	1.52E-01
Triglycerides in medium VLDL	Lipoprotein subclasses	1.07 (0.99, 1.15)	1.87E-01
Concentration of small VLDL pa	Lipoprotein subclasses	1.08 (1.00, 1.16)	1.52E-01
Total lipids in small VLDL	Lipoprotein subclasses	1.08 (1.00, 1.16)	1.31E-01
Phospholipids in small VLDL	Lipoprotein subclasses	1.06 (0.99, 1.15)	1.89E-01
Cholesterol in small VLDL	Lipoprotein subclasses	1.10 (1.03, 1.19)	**4.90E-02**
Cholesteryl esters in small VLDL	Lipoprotein subclasses	1.10 (1.03, 1.19)	**4.90E-02**
Free cholesterol in small VLDL	Lipoprotein subclasses	1.09 (1.01, 1.17)	8.57E-02
Triglycerides in small VLDL	Lipoprotein subclasses	1.06 (0.98, 1.14)	2.56E-01
Concentration of very small VLD	Lipoprotein subclasses	1.08 (1.01, 1.16)	1.04E-01
Total lipids in very small VLDL	Lipoprotein subclasses	1.08 (1.01, 1.16)	1.04E-01
Phospholipids in very small VLD	Lipoprotein subclasses	1.04 (0.96, 1.12)	4.66E-01
Cholesterol in very small VLDL	Lipoprotein subclasses	1.09 (1.02, 1.17)	7.72E-02
Cholesteryl esters in very small V	Lipoprotein subclasses	1.10 (1.02, 1.18)	5.97E-02

Free cholesterol in very small VL	Lipoprotein subclasses	1.07 (1.00, 1.16)	1.52E-01
Triglycerides in very small VLDI	Lipoprotein subclasses	1.07 (0.99, 1.15)	1.70E-01
Concentration of IDL particles	Lipoprotein subclasses	1.04 (0.97, 1.12)	4.31E-01
Total lipids in IDL	Lipoprotein subclasses	1.04 (0.96, 1.12)	4.65E-01
Phospholipids in IDL	Lipoprotein subclasses	1.02 (0.95, 1.10)	7.20E-01
Cholesterol in IDL	Lipoprotein subclasses	1.04 (0.96, 1.11)	4.89E-01
Cholesteryl esters in IDL	Lipoprotein subclasses	1.04 (0.97, 1.12)	4.24E-01
Free cholesterol in IDL	Lipoprotein subclasses	1.02 (0.95, 1.10)	7.05E-01
Triglycerides in IDL	Lipoprotein subclasses	1.07 (1.00, 1.15)	1.52E-01
Concentration of large LDL partic	Lipoprotein subclasses	1.03 (0.95, 1.11)	6.37E-01
Total lipids in large LDL	Lipoprotein subclasses	1.02 (0.95, 1.10)	6.92E-01
Phospholipids in large LDL	Lipoprotein subclasses	1.01 (0.94, 1.09)	8.34E-01
Cholesterol in large LDL	Lipoprotein subclasses	1.02 (0.95, 1.10)	7.53E-01
Cholesteryl esters in large LDL	Lipoprotein subclasses	1.02 (0.95, 1.10)	7.20E-01
Free cholesterol in large LDL	Lipoprotein subclasses	1.01 (0.94, 1.09)	8.62E-01
Triglycerides in large LDL	Lipoprotein subclasses	1.09 (1.01, 1.17)	8.99E-02
Concentration of medium LDL pa	Lipoprotein subclasses	1.01 (0.94, 1.09)	9.10E-01
Total lipids in medium LDL	Lipoprotein subclasses	1.01 (0.93, 1.09)	9.40E-01
Phospholipids in medium LDL	Lipoprotein subclasses	1.00 (0.92, 1.08)	9.63E-01
Cholesterol in medium LDL	Lipoprotein subclasses	1.00 (0.93, 1.08)	9.60E-01
Cholesteryl esters in medium LDI	Lipoprotein subclasses	1.00 (0.93, 1.08)	9.53E-01
Free cholesterol in medium LDL	Lipoprotein subclasses	0.99 (0.92, 1.07)	9.53E-01
Triglycerides in medium LDL	Lipoprotein subclasses	1.07 (1.00, 1.16)	1.52E-01
Concentration of small LDL parti	Lipoprotein subclasses	1.01 (0.94, 1.09)	8.67E-01
Total lipids in small LDL	Lipoprotein subclasses	1.01 (0.94, 1.09)	9.08E-01
Phospholipids in small LDL	Lipoprotein subclasses	1.00 (0.92, 1.08)	9.53E-01
Cholesterol in small LDL	Lipoprotein subclasses	1.00 (0.93, 1.08)	9.53E-01
Cholesteryl esters in small LDL	Lipoprotein subclasses	1.00 (0.93, 1.08)	9.53E-01
Free cholesterol in small LDL	Lipoprotein subclasses	1.00 (0.92, 1.07)	9.53E-01
Triglycerides in small LDL	Lipoprotein subclasses	1.08 (1.01, 1.17)	1.11E-01
Concentration of very large HDL	Lipoprotein subclasses	1.11 (1.04, 1.19)	**3.61E-02**
Total lipids in very large HDL	Lipoprotein subclasses	1.11 (1.03, 1.19)	**3.61E-02**
Phospholipids in very large HDL	Lipoprotein subclasses	1.09 (1.02, 1.17)	7.96E-02
Cholesterol in very large HDL	Lipoprotein subclasses	1.11 (1.04, 1.19)	**3.49E-02**
Cholesteryl esters in very large H	Lipoprotein subclasses	1.12 (1.04, 1.20)	**3.05E-02**
Free cholesterol in very large HD	Lipoprotein subclasses	1.10 (1.03, 1.18)	**4.24E-02**
Triglycerides in very large HDL	Lipoprotein subclasses	1.17 (1.10, 1.26)	**2.34E-04**
Concentration of large HDL parti	Lipoprotein subclasses	1.05 (0.97, 1.13)	3.75E-01
Total lipids in large HDL	Lipoprotein subclasses	1.04 (0.97, 1.12)	3.90E-01
Phospholipids in large HDL	Lipoprotein subclasses	1.04 (0.96, 1.12)	4.87E-01
Cholesterol in large HDL	Lipoprotein subclasses	1.04 (0.97, 1.12)	4.11E-01
Cholesteryl esters in large HDL	Lipoprotein subclasses	1.04 (0.97, 1.12)	4.24E-01
Free cholesterol in large HDL	Lipoprotein subclasses	1.05 (0.97, 1.12)	3.81E-01
Triglycerides in large HDL	Lipoprotein subclasses	1.13 (1.05, 1.21)	**2.20E-02**
**Concentration of medium HDL**	Lipoprotein subclasses	0.93 (0.86, 1.00)	1.61E-01
**Total lipids in medium HDL**	Lipoprotein subclasses	0.93 (0.86, 1.00)	1.52E-01
**Phospholipids in medium HDL**	Lipoprotein subclasses	0.95 (0.88, 1.03)	3.28E-01
**Cholesterol in medium HDL**	Lipoprotein subclasses	0.90 (0.84, 0.98)	6.87E-02

**Cholesteryl esters in medium H**	Lipoprotein subclasses	0.90 (0.83, 0.97)	**4.90E-02**
**Free cholesterol in medium HD**	Lipoprotein subclasses	0.93 (0.86, 1.01)	1.76E-01
Triglycerides in medium HDL	Lipoprotein subclasses	1.01 (0.94, 1.09)	8.43E-01
Concentration of small HDL parti	Lipoprotein subclasses	0.90 (0.84, 0.97)	**4.17E-02**
Total lipids in small HDL	Lipoprotein subclasses	0.90 (0.83, 0.96)	**3.64E-02**
Phospholipids in small HDL	Lipoprotein subclasses	0.92 (0.85, 1.00)	1.31E-01
Cholesterol in small HDL	Lipoprotein subclasses	0.91 (0.84, 0.98)	8.01E-02
Cholesteryl esters in small HDL	Lipoprotein subclasses	0.92 (0.86, 1.00)	1.31E-01
**Free cholesterol in small HDL**	Lipoprotein subclasses	0.93 (0.86, 1.01)	1.69E-01
Triglycerides in small HDL	Lipoprotein subclasses	1.03 (0.95, 1.11)	6.71E-01
Phospholipids to total lipids ratio	Relative lipoprotein lipi	1.01 (0.93, 1.10)	8.67E-01
Cholesterol to total lipids ratio in	Relative lipoprotein lipi	1.22 (1.12, 1.32)	**1.06E-04**
Cholesteryl esters to total lipids ra	Relative lipoprotein lipi	1.18 (1.09, 1.28)	**8.74E-04**
Free cholesterol to total lipids rati	Relative lipoprotein lipi	1.11 (1.02, 1.21)	6.91E-02
Triglycerides to total lipids ratio i	Relative lipoprotein lipi	0.84 (0.77, 0.92)	**3.45E-03**
Phospholipids to total lipids ratio	Relative lipoprotein lipi	1.04 (0.96, 1.13)	5.03E-01
**Cholesterol to total lipids ratio**	Relative lipoprotein lipi	1.07 (1.02, 1.13)	6.87E-02
Cholesteryl esters to total lipids ra	Relative lipoprotein lipi	1.05 (0.99, 1.11)	2.56E-01
**Free cholesterol to total lipids r**	Relative lipoprotein lipi	1.11 (1.02, 1.21)	8.57E-02
**Triglycerides to total lipids rati**	Relative lipoprotein lipi	0.91 (0.84, 0.98)	6.87E-02
Phospholipids to total lipids ratio	Relative lipoprotein lipi	1.05 (0.96, 1.14)	4.06E-01
Cholesterol to total lipids ratio in	Relative lipoprotein lipi	1.05 (0.96, 1.14)	4.44E-01
Cholesteryl esters to total lipids ra	Relative lipoprotein lipi	0.99 (0.92, 1.08)	9.46E-01
Free cholesterol to total lipids rati	Relative lipoprotein lipi	1.06 (0.97, 1.14)	3.28E-01
Triglycerides to total lipids ratio i	Relative lipoprotein lipi	0.95 (0.87, 1.04)	4.03E-01
Phospholipids to total lipids ratio	Relative lipoprotein lipi	0.92 (0.85, 1.00)	1.36E-01
**Cholesterol to total lipids ratio**	Relative lipoprotein lipi	1.08 (1.00, 1.16)	1.61E-01
**Cholesteryl esters to total lipids**	Relative lipoprotein lipi	1.04 (0.96, 1.12)	5.08E-01
Free cholesterol to total lipids rati	Relative lipoprotein lipi	1.10 (1.02, 1.20)	8.57E-02
Triglycerides to total lipids ratio i	Relative lipoprotein lipi	0.95 (0.87, 1.02)	3.00E-01
**Phospholipids to total lipids rat**	Relative lipoprotein lipi	0.89 (0.82, 0.96)	**3.61E-02**
Cholesterol to total lipids ratio in	Relative lipoprotein lipi	1.06 (0.99, 1.15)	2.12E-01
Cholesteryl esters to total lipids ra	Relative lipoprotein lipi	1.05 (0.98, 1.14)	3.00E-01
**Free cholesterol to total lipids r**	Relative lipoprotein lipi	1.11 (1.03, 1.19)	5.68E-02
Triglycerides to total lipids ratio i	Relative lipoprotein lipi	0.96 (0.89, 1.04)	5.02E-01
Phospholipids to total lipids ratio	Relative lipoprotein lipi	0.90 (0.83, 0.97)	**4.24E-02**
Cholesterol to total lipids ratio in	Relative lipoprotein lipi	1.06 (0.98, 1.14)	2.82E-01
Cholesteryl esters to total lipids ra	Relative lipoprotein lipi	1.07 (0.99, 1.15)	1.87E-01
Free cholesterol to total lipids rati	Relative lipoprotein lipi	1.01 (0.93, 1.09)	9.10E-01
Triglycerides to total lipids ratio i	Relative lipoprotein lipi	1.00 (0.93, 1.08)	9.72E-01
Phospholipids to total lipids ratio	Relative lipoprotein lipi	0.88 (0.82, 0.95)	**1.12E-02**
Cholesterol to total lipids ratio in	Relative lipoprotein lipi	1.00 (0.93, 1.07)	9.60E-01
Cholesteryl esters to total lipids ra	Relative lipoprotein lipi	1.03 (0.96, 1.11)	6.06E-01
Free cholesterol to total lipids rati	Relative lipoprotein lipi	0.96 (0.89, 1.04)	4.99E-01
Triglycerides to total lipids ratio i	Relative lipoprotein lipi	1.05 (0.97, 1.12)	3.78E-01
Phospholipids to total lipids ratio	Relative lipoprotein lipi	0.94 (0.87, 1.02)	3.08E-01
Cholesterol to total lipids ratio in	Relative lipoprotein lipi	0.99 (0.92, 1.06)	8.48E-01
Cholesteryl esters to total lipids ra	Relative lipoprotein lipi	1.00 (0.93, 1.08)	9.53E-01

Free cholesterol to total lipids rati	Relative lipoprotein lipi	0.95 (0.88, 1.03)	3.41E-01
Triglycerides to total lipids ratio i	Relative lipoprotein lipi	1.06 (0.99, 1.13)	1.97E-01
Phospholipids to total lipids ratio	Relative lipoprotein lipi	0.99 (0.92, 1.07)	8.82E-01
Cholesterol to total lipids ratio in	Relative lipoprotein lipi	0.98 (0.91, 1.06)	7.53E-01
Cholesteryl esters to total lipids ra	Relative lipoprotein lipi	0.99 (0.92, 1.07)	9.08E-01
Free cholesterol to total lipids rati	Relative lipoprotein lipi	0.98 (0.90, 1.06)	7.04E-01
Triglycerides to total lipids ratio i	Relative lipoprotein lipi	1.07 (1.00, 1.15)	1.47E-01
Phospholipids to total lipids ratio	Relative lipoprotein lipi	0.99 (0.91, 1.07)	8.58E-01
Cholesterol to total lipids ratio in	Relative lipoprotein lipi	0.98 (0.91, 1.06)	7.47E-01
Cholesteryl esters to total lipids ra	Relative lipoprotein lipi	0.99 (0.92, 1.07)	8.67E-01
Free cholesterol to total lipids rati	Relative lipoprotein lipi	0.97 (0.90, 1.05)	6.12E-01
Triglycerides to total lipids ratio i	Relative lipoprotein lipi	1.08 (1.00, 1.16)	1.31E-01
Phospholipids to total lipids ratio	Relative lipoprotein lipi	0.97 (0.90, 1.05)	6.37E-01
Cholesterol to total lipids ratio in	Relative lipoprotein lipi	1.02 (0.94, 1.10)	8.28E-01
Cholesteryl esters to total lipids ra	Relative lipoprotein lipi	1.01 (0.94, 1.10)	8.67E-01
Free cholesterol to total lipids rati	Relative lipoprotein lipi	1.02 (0.95, 1.10)	7.18E-01
Triglycerides to total lipids ratio i	Relative lipoprotein lipi	1.04 (0.97, 1.12)	4.47E-01
Phospholipids to total lipids ratio	Relative lipoprotein lipi	0.95 (0.88, 1.03)	3.28E-01
Cholesterol to total lipids ratio in	Relative lipoprotein lipi	1.00 (0.93, 1.08)	9.53E-01
Cholesteryl esters to total lipids ra	Relative lipoprotein lipi	0.99 (0.92, 1.06)	8.48E-01
Free cholesterol to total lipids rati	Relative lipoprotein lipi	1.03 (0.96, 1.11)	6.11E-01
Triglycerides to total lipids ratio i	Relative lipoprotein lipi	1.07 (1.00, 1.15)	1.61E-01
Phospholipids to total lipids ratio	Relative lipoprotein lipi	1.16 (1.09, 1.23)	**1.06E-04**
Cholesterol to total lipids ratio in	Relative lipoprotein lipi	0.88 (0.82, 0.95)	**8.44E-03**
Cholesteryl esters to total lipids ra	Relative lipoprotein lipi	0.88 (0.82, 0.94)	**6.46E-03**
Free cholesterol to total lipids rati	Relative lipoprotein lipi	0.94 (0.87, 1.01)	1.87E-01
Triglycerides to total lipids ratio i	Relative lipoprotein lipi	1.07 (0.99, 1.15)	1.87E-01
Phospholipids to total lipids ratio	Relative lipoprotein lipi	1.00 (0.92, 1.08)	9.92E-01
Cholesterol to total lipids ratio in	Relative lipoprotein lipi	0.98 (0.91, 1.06)	7.30E-01
Cholesteryl esters to total lipids ra	Relative lipoprotein lipi	0.98 (0.90, 1.06)	7.18E-01
Free cholesterol to total lipids rati	Relative lipoprotein lipi	1.03 (0.96, 1.12)	5.72E-01
Triglycerides to total lipids ratio i	Relative lipoprotein lipi	1.07 (0.99, 1.16)	1.66E-01
Average diameter for VLDL parti	Lipoprotein particle size	1.02 (0.95, 1.11)	7.02E-01
Average diameter for LDL particl	Lipoprotein particle size	1.07 (0.99, 1.16)	1.73E-01
Average diameter for HDL partic	Lipoprotein particle size	1.09 (1.01, 1.17)	8.99E-02
Total cholesterol	Cholesterol	1.05 (0.98, 1.13)	3.28E-01
VLDL cholesterol	Cholesterol	1.10 (1.02, 1.18)	5.97E-02
Remnant cholesterol (non-HDL, r	Cholesterol	1.09 (1.01, 1.17)	9.42E-02
LDL cholesterol	Cholesterol	1.01 (0.94, 1.09)	8.62E-01
**HDL cholesterol**	Cholesterol	1.02 (0.94, 1.09)	8.28E-01
**HDL2 cholesterol**	Cholesterol	1.00 (0.93, 1.08)	9.53E-01
HDL3 cholesterol	Cholesterol	1.10 (1.02, 1.18)	6.87E-02
Total esterified cholesterol	Cholesteryl esters	0.97 (0.90, 1.05)	6.62E-01
Total free cholesterol	Free cholesterol	0.99 (0.92, 1.07)	9.10E-01
Total triglycerides	Triglycerides	1.07 (0.99, 1.15)	1.70E-01
Triglycerides in VLDL	Triglycerides	1.06 (0.99, 1.15)	2.01E-01
Triglycerides in LDL	Triglycerides	1.09 (1.01, 1.17)	8.71E-02
Triglycerides in HDL	Triglycerides	1.09 (1.01, 1.17)	1.04E-01

Phosphoglycerides	Other lipids	1.05 (0.97, 1.13)	3.65E-01
Ratio of triglycerides to phospho	Other lipids	1.07 (1.00, 1.16)	1.61E-01
Phosphatidylcholines	Other lipids	0.95 (0.88, 1.03)	3.28E-01
Sphingomyelins	Other lipids	0.89 (0.83, 0.97)	**4.24E-02**
Total cholines	Other lipids	1.02 (0.95, 1.10)	7.20E-01
Apolipoprotein A1	Apolipoproteins	1.05 (0.98, 1.13)	2.87E-01
Apolipoprotein B	Apolipoproteins	1.07 (0.99, 1.15)	1.73E-01
Ratio of apolipoprotein B to apol	Apolipoproteins	1.04 (0.97, 1.13)	3.97E-01
Total fatty acids	Fatty acids	1.04 (0.96, 1.12)	4.75E-01
Degree of unsaturation	Fatty acids	0.82 (0.76, 0.88)	**3.08E-05**
Docosahexaenoic acid	Fatty acids	1.09 (1.02, 1.18)	6.91E-02
Linoleic acid	Fatty acids	1.00 (0.93, 1.08)	9.60E-01
Omega-3 fatty acids	Fatty acids	1.04 (0.97, 1.12)	3.99E-01
Omega-6 fatty acids	Fatty acids	0.99 (0.92, 1.07)	9.40E-01
Polyunsaturated fatty acids	Fatty acids	1.00 (0.93, 1.08)	9.53E-01
Monounsaturated fatty acids	Fatty acids	1.05 (0.98, 1.13)	3.28E-01
Saturated fatty acids	Fatty acids	1.05 (0.98, 1.13)	3.27E-01
Ratio of docosahexaenoic acid to	Fatty acids	1.11 (1.03, 1.19)	5.97E-02
Ratio of linoleic acid to total fatty	Fatty acids	0.91 (0.84, 0.98)	7.72E-02
Ratio of omega-3 fatty acids to to	Fatty acids	1.03 (0.96, 1.11)	5.16E-01
Ratio of omega-6 fatty acids to to	Fatty acids	0.89 (0.83, 0.96)	**3.61E-02**
Ratio of polyunsaturated fatty aci	Fatty acids	0.89 (0.83, 0.97)	**4.17E-02**
Ratio of monounsaturated fatty ac	Fatty acids	1.08 (1.00, 1.16)	1.58E-01
Ratio of saturated fatty acids to tc	Fatty acids	1.08 (1.00, 1.17)	1.31E-01
Glucose	Glycolysis related metal	1.12 (1.06, 1.19)	**3.45E-03**
Lactate	Glycolysis related metal	0.96 (0.89, 1.04)	4.47E-01
Citrate	Glycolysis related metal	1.07 (0.98, 1.16)	2.56E-01
Alanine	Amino acids	0.95 (0.88, 1.03)	3.45E-01
Glutamine	Amino acids	0.89 (0.83, 0.96)	**3.49E-02**
Histidine	Amino acids	0.91 (0.84, 0.99)	8.71E-02
Isoleucine	Amino acids	1.02 (0.95, 1.11)	7.18E-01
Leucine	Amino acids	1.01 (0.93, 1.09)	8.95E-01
Valine	Amino acids	0.91 (0.84, 0.98)	8.57E-02
Phenylalanine	Amino acids	1.11 (1.04, 1.19)	**3.61E-02**
Tyrosine	Amino acids	1.01 (0.94, 1.09)	8.67E-01
Acetate	Ketone bodies	1.07 (1.01, 1.13)	8.71E-02
Acetoacetate	Ketone bodies	1.11 (1.04, 1.18)	**3.03E-02**
**3-Hydroxybutyrate**	Ketone bodies	1.08 (1.02, 1.16)	6.87E-02
Creatinine	Fluid balance	1.06 (1.02, 1.11)	**4.17E-02**
Albumin	Fluid balance	0.92 (0.86, 0.99)	1.03E-01
Glycoprotein acetyls	Inflammation	1.20 (1.11, 1.30)	**1.06E-04**
Acetone	Ketone bodies		
Cholesteryl esters in HDL	Cholesteryl esters		
Cholesteryl esters in LDL	Cholesteryl esters		
Cholesteryl esters in VLDL/ (Chc	Cholesteryl esters		
Clinical LDL cholesterol	Cholesterol		
Concentration of HDL particles	Lipoprotein particle concentrations		
Concentration of LDL particles	Lipoprotein particle concentrations		

Concentration of VLDL particles	Lipoprotein particle concentrations
Free cholesterol in HDL	Free cholesterol
Free cholesterol in LDL	Free cholesterol
Free cholesterol in VLDL	Free cholesterol
Glycine	Amino acids
Phospholipids in HDL	Phospholipids
Phospholipids in LDL	Phospholipids
Phospholipids in VLDL	Phospholipids
Pyruvate	Glycolysis related metabolites
Ratio of omega-6 fatty acids to or	Fatty acids
Ratio of polyunsaturated fatty aci	Fatty acids
Total cholesterol minus HDL-C	Cholesterol
Total concentration of branched-c	Amino acids
Total concentration of lipoprotein	Lipoprotein particle concentrations
Total lipids in HDL	Total lipids
Total lipids in LDL	Total lipids
Total lipids in lipoprotein particle	Total lipids
Total lipids in VLDL	Total lipids
Total phospholipids in lipoprotein	Phospholipids

Models were adjusted for age, sex, levels of education, smoking status, alcohol consumption, and self-rated heal

HR, hazard ratio; CI, confidence interval; LDL, Low Density Lipoprotein; HDL, High Density Lipoprotein; VL

abolites with all-cause and cause-specific mortality in the CKB and UKB by reg

mortality		Ischaemic heart	
Rural		Urban	
Adjusted HR (95% CI)	P value	Adjusted HR (95% CI)	P value
0.89 (0.84, 0.95)	**4.40E-04**	1.17 (1.01,1.36)	6.80E-02
0.89 (0.84, 0.95)	**5.81E-04**	1.17 (1.01,1.36)	6.51E-02
0.90 (0.85, 0.96)	**1.82E-03**	1.17 (1.00,1.36)	7.01E-02
0.93 (0.88, 0.98)	**1.34E-02**	1.22 (1.05,1.41)	**2.15E-02**
0.94 (0.89, 0.99)	**2.98E-02**	1.26 (1.09,1.45)	**6.01E-03**
0.92 (0.86, 0.97)	**5.43E-03**	1.15 (0.99,1.34)	1.04E-01
0.89 (0.84, 0.94)	**1.85E-04**	1.16 (1.00,1.35)	8.30E-02
0.92 (0.86, 0.97)	**4.96E-03**	1.15 (0.99,1.34)	9.97E-02
0.92 (0.87, 0.97)	**5.60E-03**	1.16 (0.99,1.35)	9.08E-02
0.92 (0.87, 0.97)	**7.13E-03**	1.16 (1.00,1.35)	7.47E-02
0.93 (0.88, 0.98)	**1.95E-02**	1.19 (1.03,1.38)	**4.18E-02**
0.93 (0.88, 0.99)	**2.12E-02**	1.21 (1.04,1.40)	**3.02E-02**
0.93 (0.88, 0.98)	**1.74E-02**	1.17 (1.01,1.36)	6.23E-02
0.91 (0.86, 0.97)	**3.48E-03**	1.14 (0.98,1.33)	1.30E-01
0.91 (0.86, 0.97)	**3.23E-03**	1.18 (1.02,1.38)	5.31E-02
0.91 (0.86, 0.97)	**3.23E-03**	1.18 (1.02,1.38)	5.25E-02
0.91 (0.86, 0.97)	**3.24E-03**	1.18 (1.02,1.38)	5.25E-02
0.91 (0.86, 0.96)	**2.19E-03**	1.20 (1.03,1.39)	**3.83E-02**
0.90 (0.85, 0.95)	**7.87E-04**	1.22 (1.05,1.41)	**2.42E-02**
0.92 (0.87, 0.97)	**6.06E-03**	1.17 (1.01,1.37)	6.23E-02
0.91 (0.86, 0.97)	**3.57E-03**	1.18 (1.01,1.37)	5.93E-02
0.91 (0.86, 0.96)	**2.15E-03**	1.24 (1.06,1.43)	**1.59E-02**
0.91 (0.86, 0.96)	**2.18E-03**	1.24 (1.07,1.44)	**1.55E-02**
0.91 (0.86, 0.96)	**1.55E-03**	1.24 (1.07,1.44)	**1.55E-02**
0.91 (0.86, 0.96)	**2.52E-03**	1.28 (1.10,1.48)	**5.09E-03**
0.91 (0.86, 0.96)	**1.55E-03**	1.31 (1.14,1.52)	**1.95E-03**
0.92 (0.87, 0.97)	**6.01E-03**	1.23 (1.06,1.43)	**1.80E-02**
0.91 (0.86, 0.96)	**2.66E-03**	1.22 (1.05,1.41)	**2.41E-02**
0.92 (0.87, 0.97)	**5.38E-03**	1.30 (1.12,1.51)	**3.68E-03**
0.93 (0.88, 0.98)	**1.03E-02**	1.32 (1.14,1.54)	**1.97E-03**
0.91 (0.86, 0.96)	**1.71E-03**	1.30 (1.12,1.51)	**4.12E-03**
0.99 (0.94, 1.04)	8.03E-01	1.48 (1.28,1.71)	**3.73E-06**
1.02 (0.97, 1.07)	6.13E-01	1.52 (1.31,1.75)	**7.08E-07**
0.95 (0.90, 1.00)	1.03E-01	1.35 (1.17,1.57)	**6.94E-04**
0.90 (0.85, 0.95)	**7.06E-04**	1.20 (1.03,1.40)	**4.03E-02**
1.00 (0.95, 1.06)	9.15E-01	1.39 (1.20,1.61)	**1.24E-04**
1.02 (0.97, 1.07)	6.13E-01	1.40 (1.21,1.62)	**8.08E-05**
0.98 (0.94, 1.04)	6.15E-01	1.29 (1.10,1.50)	**5.91E-03**
1.07 (1.02, 1.12)	**1.05E-02**	1.48 (1.28,1.71)	**5.63E-06**
1.07 (1.02, 1.13)	**7.32E-03**	1.50 (1.30,1.74)	**2.35E-06**

1.06 (1.01, 1.11)	**3.27E-02**	1.41 (1.21,1.63)	**1.02E-04**
0.94 (0.89, 0.99)	**3.06E-02**	1.19 (1.03,1.39)	**4.38E-02**
0.97 (0.93, 1.03)	3.82E-01	1.26 (1.08,1.47)	**1.08E-02**
0.98 (0.93, 1.03)	4.39E-01	1.26 (1.08,1.47)	**1.09E-02**
0.96 (0.92, 1.02)	2.15E-01	1.23 (1.05,1.44)	**2.24E-02**
0.98 (0.93, 1.03)	4.41E-01	1.27 (1.09,1.48)	**9.41E-03**
0.96 (0.91, 1.01)	1.87E-01	1.28 (1.11,1.49)	**5.09E-03**
1.01 (0.96, 1.07)	6.51E-01	1.22 (1.04,1.43)	**3.54E-02**
1.00 (0.94, 1.05)	9.15E-01	1.13 (0.97,1.32)	1.63E-01
0.96 (0.91, 1.01)	1.31E-01	1.25 (1.07,1.46)	**1.42E-02**
0.96 (0.91, 1.01)	1.37E-01	1.25 (1.07,1.46)	**1.55E-02**
0.93 (0.89, 0.98)	**1.83E-02**	1.24 (1.06,1.45)	**1.87E-02**
0.96 (0.91, 1.01)	1.47E-01	1.25 (1.07,1.46)	**1.55E-02**
0.95 (0.90, 1.00)	8.46E-02	1.26 (1.08,1.47)	**1.15E-02**
0.98 (0.93, 1.03)	5.70E-01	1.20 (1.03,1.41)	**4.57E-02**
1.03 (0.97, 1.08)	4.00E-01	1.16 (1.00,1.34)	8.53E-02
0.93 (0.89, 0.98)	**1.91E-02**	1.24 (1.06,1.45)	**1.89E-02**
0.93 (0.89, 0.98)	**1.87E-02**	1.24 (1.06,1.45)	**2.05E-02**
0.88 (0.83, 0.93)	**6.45E-06**	1.23 (1.05,1.44)	**2.74E-02**
0.95 (0.90, 1.00)	6.90E-02	1.23 (1.05,1.45)	**2.30E-02**
0.95 (0.91, 1.00)	1.03E-01	1.24 (1.06,1.45)	**1.99E-02**
0.93 (0.88, 0.98)	**7.32E-03**	1.20 (1.02,1.41)	**4.98E-02**
1.00 (0.95, 1.06)	9.57E-01	1.17 (1.00,1.36)	7.47E-02
0.93 (0.88, 0.98)	**1.51E-02**	1.26 (1.08,1.47)	**1.28E-02**
0.93 (0.89, 0.98)	**1.74E-02**	1.26 (1.07,1.47)	**1.42E-02**
0.88 (0.84, 0.93)	**3.43E-06**	1.22 (1.03,1.43)	**3.88E-02**
0.96 (0.91, 1.01)	1.25E-01	1.25 (1.06,1.46)	**1.79E-02**
0.96 (0.91, 1.01)	1.92E-01	1.25 (1.07,1.47)	**1.59E-02**
0.93 (0.88, 0.98)	**9.61E-03**	1.21 (1.03,1.42)	**4.18E-02**
0.95 (0.90, 1.01)	1.17E-01	1.25 (1.08,1.45)	**1.28E-02**
1.20 (1.15, 1.26)	**1.03E-12**	1.17 (1.01,1.36)	6.81E-02
1.20 (1.15, 1.26)	**1.06E-12**	1.17 (1.01,1.36)	6.61E-02
1.19 (1.13, 1.25)	**3.62E-11**	1.07 (0.91,1.25)	4.44E-01
1.21 (1.15, 1.27)	**9.46E-13**	1.26 (1.08,1.47)	**1.08E-02**
1.21 (1.16, 1.27)	**3.28E-13**	1.30 (1.12,1.52)	**3.74E-03**
1.19 (1.13, 1.25)	**3.62E-11**	1.16 (0.99,1.35)	9.08E-02
1.10 (1.05, 1.16)	**4.36E-04**	1.29 (1.13,1.48)	**1.80E-03**
1.15 (1.10, 1.21)	**1.43E-07**	0.94 (0.80,1.12)	5.31E-01
1.15 (1.10, 1.21)	**1.05E-07**	0.94 (0.79,1.11)	5.15E-01
1.14 (1.09, 1.20)	**6.34E-07**	0.95 (0.80,1.13)	5.93E-01
1.16 (1.11, 1.22)	**2.50E-08**	0.92 (0.78,1.10)	4.13E-01
1.16 (1.10, 1.22)	**6.77E-08**	0.91 (0.77,1.09)	3.71E-01
1.18 (1.12, 1.24)	**1.27E-09**	0.95 (0.80,1.13)	5.98E-01
1.09 (1.03, 1.15)	**2.80E-03**	1.08 (0.91,1.27)	4.27E-01
0.88 (0.83, 0.93)	**2.56E-05**	0.83 (0.70,0.98)	5.68E-02
0.89 (0.84, 0.94)	**6.21E-05**	0.82 (0.69,0.98)	**4.77E-02**
0.91 (0.86, 0.96)	**2.15E-03**	0.85 (0.72,1.01)	9.42E-02
0.90 (0.85, 0.95)	**2.83E-04**	0.78 (0.66,0.93)	**1.62E-02**

0.89 (0.84, 0.94)	**7.89E-05**	0.77 (0.65,0.92)	**1.29E-02**
0.93 (0.88, 0.98)	**1.56E-02**	0.82 (0.69,0.98)	5.34E-02
0.81 (0.77, 0.86)	**1.26E-11**	1.07 (0.91,1.26)	4.61E-01
0.79 (0.75, 0.83)	**2.65E-16**	0.82 (0.72,0.94)	**1.55E-02**
0.80 (0.76, 0.84)	**9.36E-16**	0.82 (0.72,0.94)	**1.55E-02**
0.83 (0.79, 0.88)	**1.79E-10**	0.81 (0.69,0.96)	**3.02E-02**
0.89 (0.85, 0.94)	**4.90E-05**	0.88 (0.75,1.03)	1.63E-01
0.92 (0.87, 0.97)	**2.45E-03**	0.92 (0.78,1.08)	3.71E-01
0.86 (0.81, 0.91)	**1.43E-07**	0.80 (0.69,0.94)	**1.59E-02**
0.85 (0.81, 0.90)	**1.31E-07**	1.02 (0.87,1.21)	7.95E-01
0.93 (0.88, 0.98)	**1.01E-02**	1.10 (0.91,1.33)	3.76E-01
1.15 (1.10, 1.19)	**2.03E-09**	1.35 (1.15,1.59)	**2.69E-03**
1.15 (1.10, 1.20)	**4.52E-10**	1.34 (1.15,1.56)	**1.95E-03**
1.05 (0.99, 1.11)	1.08E-01	1.08 (0.90,1.28)	4.60E-01
0.89 (0.85, 0.94)	**2.65E-05**	0.74 (0.61,0.89)	**6.01E-03**
0.92 (0.87, 0.97)	**3.36E-03**	1.52 (1.19,1.95)	**4.62E-03**
1.10 (1.06, 1.15)	**1.84E-05**	1.16 (1.05,1.28)	**1.02E-02**
1.14 (1.09, 1.20)	**9.49E-08**	1.07 (0.95,1.22)	3.36E-01
0.99 (0.93, 1.04)	7.27E-01	1.55 (1.23,1.96)	**1.97E-03**
0.96 (0.91, 1.01)	1.77E-01	0.72 (0.62,0.83)	**8.57E-05**
0.92 (0.87, 0.97)	**3.57E-03**	1.22 (1.00,1.50)	8.00E-02
0.94 (0.89, 0.99)	**4.05E-02**	1.24 (1.01,1.52)	6.61E-02
1.00 (0.95, 1.06)	1.00E+00	1.01 (0.85,1.20)	9.48E-01
0.93 (0.88, 0.98)	**1.03E-02**	1.26 (1.03,1.54)	**4.49E-02**
1.07 (1.02, 1.13)	**1.54E-02**	0.80 (0.65,0.98)	5.93E-02
0.96 (0.91, 1.01)	1.57E-01	0.76 (0.63,0.91)	**1.04E-02**
1.01 (0.95, 1.06)	8.18E-01	1.19 (1.00,1.42)	7.14E-02
1.01 (0.96, 1.07)	6.53E-01	1.09 (0.92,1.29)	3.98E-01
0.98 (0.93, 1.04)	5.88E-01	1.31 (1.07,1.60)	**2.08E-02**
1.00 (0.95, 1.06)	1.00E+00	0.89 (0.75,1.06)	2.38E-01
0.96 (0.91, 1.02)	2.08E-01	0.61 (0.49,0.74)	**3.97E-05**
1.14 (1.08, 1.20)	**6.94E-06**	1.28 (1.08,1.52)	**1.37E-02**
1.12 (1.06, 1.18)	**6.21E-05**	1.28 (1.08,1.52)	**1.35E-02**
1.17 (1.11, 1.23)	**8.21E-09**	1.16 (0.99,1.36)	1.03E-01
0.89 (0.84, 0.94)	**3.69E-05**	0.86 (0.72,1.01)	1.04E-01
0.93 (0.89, 0.98)	**1.08E-02**	0.81 (0.70,0.95)	**1.90E-02**
1.19 (1.13, 1.26)	**4.63E-09**	1.38 (1.15,1.67)	**4.12E-03**
1.16 (1.10, 1.22)	**2.66E-07**	1.39 (1.16,1.66)	**2.71E-03**
1.14 (1.08, 1.21)	**1.60E-05**	1.17 (0.96,1.41)	1.52E-01
0.91 (0.86, 0.96)	**1.55E-03**	0.87 (0.72,1.04)	1.75E-01
0.96 (0.91, 1.02)	2.03E-01	0.78 (0.68,0.90)	**4.34E-03**
1.00 (0.95, 1.05)	9.57E-01	1.27 (1.05,1.54)	**3.39E-02**
0.92 (0.88, 0.97)	**3.36E-03**	1.37 (1.15,1.63)	**2.87E-03**
1.10 (1.04, 1.17)	**1.89E-03**	1.00 (0.84,1.18)	9.62E-01
1.01 (0.96, 1.07)	6.37E-01	0.89 (0.74,1.07)	2.76E-01
0.99 (0.94, 1.05)	7.88E-01	0.77 (0.62,0.94)	**2.76E-02**
0.97 (0.92, 1.02)	2.29E-01	1.20 (0.98,1.46)	1.04E-01
0.93 (0.89, 0.98)	**7.32E-03**	1.35 (1.09,1.66)	**1.55E-02**

1.14 (1.07, 1.20)	**5.39E-05**	0.87 (0.75,1.01)	9.42E-02
1.06 (1.01, 1.11)	**2.69E-02**	0.92 (0.77,1.10)	4.14E-01
0.96 (0.91, 1.02)	2.05E-01	0.87 (0.72,1.05)	1.98E-01
1.00 (0.95, 1.05)	1.00E+00	1.12 (0.93,1.35)	2.76E-01
0.98 (0.94, 1.04)	6.27E-01	1.17 (0.97,1.42)	1.37E-01
1.06 (1.00, 1.11)	5.96E-02	0.77 (0.64,0.94)	**2.59E-02**
1.07 (1.02, 1.13)	**1.29E-02**	0.95 (0.79,1.13)	5.72E-01
0.97 (0.92, 1.02)	3.16E-01	0.81 (0.67,0.99)	6.51E-02
1.02 (0.96, 1.07)	6.29E-01	1.12 (0.93,1.35)	2.70E-01
1.00 (0.95, 1.06)	9.41E-01	1.17 (0.97,1.42)	1.39E-01
1.05 (1.00, 1.11)	9.03E-02	0.73 (0.60,0.89)	**9.41E-03**
1.02 (0.97, 1.07)	5.95E-01	1.04 (0.89,1.22)	6.48E-01
0.99 (0.94, 1.04)	7.02E-01	0.77 (0.66,0.90)	**5.78E-03**
1.05 (1.00, 1.11)	1.00E-01	1.38 (1.16,1.64)	**2.69E-03**
1.05 (0.99, 1.11)	1.17E-01	1.37 (1.16,1.62)	**1.74E-03**
1.04 (0.99, 1.09)	1.92E-01	1.14 (0.95,1.36)	2.14E-01
0.91 (0.86, 0.96)	**2.53E-03**	1.04 (0.89,1.22)	6.28E-01
0.85 (0.81, 0.90)	**6.77E-08**	1.05 (0.89,1.23)	6.17E-01
1.20 (1.13, 1.27)	**5.19E-09**	0.93 (0.80,1.09)	4.27E-01
1.13 (1.07, 1.19)	**3.89E-05**	0.86 (0.74,0.99)	6.31E-02
1.24 (1.17, 1.32)	**1.00E-11**	1.09 (0.92,1.30)	3.71E-01
0.95 (0.90, 1.00)	7.16E-02	1.08 (0.92,1.26)	4.16E-01
1.17 (1.12, 1.22)	**2.20E-12**	1.22 (1.09,1.36)	**4.23E-03**
0.95 (0.90, 1.00)	1.00E-01	0.81 (0.71,0.92)	**7.56E-03**
0.94 (0.90, 0.99)	**4.39E-02**	0.82 (0.72,0.93)	**9.63E-03**
1.01 (0.96, 1.06)	8.12E-01	0.86 (0.74,1.00)	7.78E-02
0.90 (0.85, 0.95)	**8.04E-04**	1.17 (1.00,1.37)	7.12E-02
0.98 (0.93, 1.03)	4.20E-01	0.93 (0.78,1.11)	4.60E-01
1.04 (0.99, 1.10)	1.74E-01	1.03 (0.87,1.22)	7.53E-01
1.03 (0.98, 1.09)	3.16E-01	1.04 (0.88,1.24)	6.76E-01
1.06 (1.00, 1.11)	5.70E-02	0.90 (0.75,1.07)	2.86E-01
0.93 (0.88, 0.99)	**2.11E-02**	1.10 (0.94,1.30)	2.79E-01
0.89 (0.85, 0.94)	**1.18E-04**	1.15 (0.98,1.36)	1.30E-01
1.09 (1.03, 1.15)	**3.29E-03**	0.92 (0.77,1.10)	3.98E-01
1.21 (1.15, 1.28)	**1.79E-12**	1.09 (0.93,1.28)	3.71E-01
1.00 (0.95, 1.05)	9.15E-01	1.30 (1.12,1.51)	**3.74E-03**
0.96 (0.91, 1.01)	1.64E-01	1.41 (1.22,1.63)	**3.99E-05**
0.96 (0.91, 1.01)	1.92E-01	1.42 (1.23,1.64)	**3.97E-05**
0.96 (0.92, 1.01)	2.03E-01	1.25 (1.07,1.46)	**1.66E-02**
1.09 (1.04, 1.15)	**1.90E-03**	0.95 (0.81,1.13)	6.01E-01
1.08 (1.02, 1.13)	**7.32E-03**	0.92 (0.78,1.09)	3.98E-01
1.18 (1.12, 1.24)	**4.52E-10**	1.23 (1.05,1.43)	**2.74E-02**
0.88 (0.83, 0.92)	**8.69E-06**	1.04 (0.88,1.23)	6.32E-01
0.89 (0.84, 0.94)	**2.54E-04**	1.15 (0.98,1.34)	1.13E-01
0.92 (0.87, 0.97)	**5.65E-03**	1.21 (1.04,1.40)	**3.08E-02**
0.91 (0.86, 0.96)	**2.70E-03**	1.21 (1.04,1.40)	**3.02E-02**
1.02 (0.96, 1.07)	6.37E-01	1.17 (1.01,1.36)	5.78E-02
0.93 (0.88, 0.98)	**1.74E-02**	1.12 (0.95,1.31)	2.29E-01

0.96 (0.91, 1.01)	1.57E-01	1.16 (0.99,1.36)	1.04E-01
0.93 (0.88, 0.98)	**1.08E-02**	1.20 (1.03,1.41)	**4.15E-02**
0.86 (0.82, 0.91)	**4.44E-07**	0.93 (0.79,1.10)	4.42E-01
0.84 (0.80, 0.89)	**1.01E-08**	0.98 (0.82,1.16)	7.95E-01
0.93 (0.88, 0.99)	**2.09E-02**	1.09 (0.93,1.28)	3.52E-01
1.07 (1.01, 1.12)	**2.04E-02**	1.11 (0.94,1.30)	2.70E-01
0.93 (0.88, 0.98)	**1.05E-02**	1.36 (1.18,1.58)	**5.43E-04**
0.90 (0.86, 0.95)	**4.87E-04**	1.31 (1.11,1.53)	**5.09E-03**
0.88 (0.83, 0.93)	**2.20E-05**	1.17 (1.00,1.37)	7.84E-02
0.80 (0.76, 0.84)	**3.91E-18**	0.69 (0.60,0.80)	**1.13E-05**
1.11 (1.05, 1.16)	**2.28E-04**	1.54 (1.34,1.76)	**6.15E-08**
0.87 (0.82, 0.92)	**1.53E-06**	1.12 (0.95,1.32)	2.39E-01
0.98 (0.93, 1.03)	4.96E-01	1.32 (1.14,1.52)	**1.56E-03**
0.84 (0.80, 0.89)	**1.67E-08**	1.06 (0.90,1.25)	5.11E-01
0.86 (0.81, 0.91)	**4.21E-07**	1.12 (0.95,1.32)	2.13E-01
0.90 (0.85, 0.95)	**3.34E-04**	1.21 (1.04,1.41)	**3.07E-02**
0.89 (0.85, 0.95)	**2.77E-04**	1.15 (0.98,1.34)	1.19E-01
1.26 (1.19, 1.33)	**9.36E-16**	1.65 (1.42,1.92)	**1.78E-08**
0.91 (0.87, 0.96)	**1.21E-03**	0.86 (0.73,1.00)	8.14E-02
1.10 (1.04, 1.15)	**1.38E-03**	1.40 (1.21,1.63)	**1.37E-04**
0.91 (0.86, 0.95)	**5.67E-04**	0.75 (0.64,0.88)	**2.81E-03**
0.93 (0.88, 0.98)	**9.61E-03**	0.82 (0.70,0.97)	**4.18E-02**
1.00 (0.95, 1.05)	1.00E+00	1.32 (1.13,1.55)	**3.74E-03**
1.13 (1.07, 1.20)	**2.80E-05**	0.91 (0.77,1.08)	3.58E-01
1.12 (1.08, 1.17)	**1.66E-07**	1.24 (1.11,1.37)	**7.90E-04**
0.98 (0.92, 1.03)	4.26E-01	1.18 (1.01,1.38)	6.12E-02
0.95 (0.90, 1.01)	1.47E-01	0.84 (0.70,1.01)	9.08E-02
0.91 (0.86, 0.96)	**1.92E-03**	1.21 (1.03,1.42)	**4.40E-02**
0.93 (0.88, 0.98)	**1.10E-02**	1.00 (0.85,1.17)	9.64E-01
1.04 (0.98, 1.09)	2.10E-01	1.21 (1.02,1.43)	5.25E-02
0.89 (0.84, 0.94)	**7.93E-05**	1.22 (1.04,1.42)	**3.10E-02**
0.94 (0.89, 0.99)	**4.80E-02**	1.31 (1.13,1.52)	**2.71E-03**
0.80 (0.76, 0.85)	**6.65E-13**	1.09 (0.93,1.29)	3.46E-01
1.12 (1.08, 1.16)	**1.64E-08**	1.38 (1.21,1.58)	**2.76E-05**
1.04 (0.98, 1.10)	2.03E-01	1.19 (1.01,1.39)	5.93E-02
1.06 (1.01, 1.11)	**2.09E-02**	1.06 (0.94,1.20)	3.82E-01
0.96 (0.91, 1.02)	2.39E-01	1.08 (0.93,1.24)	3.71E-01
1.00 (0.95, 1.06)	9.15E-01	1.01 (0.85,1.20)	9.19E-01
1.06 (1.01, 1.12)	**3.15E-02**	1.09 (1.02,1.16)	**3.17E-02**
0.82 (0.78, 0.86)	**3.79E-15**	0.79 (0.69,0.90)	**3.67E-03**
1.12 (1.06, 1.18)	**6.08E-05**	1.51 (1.31,1.74)	**7.08E-07**

th status. P values are corrected for multiple testing using a false discovery rate (FDR) adjus
DL, Very Low Density Lipoprotein; IDL, Intermediate Density Lipoprotein..

gion or ethnicity

diseases mortality		Cerebrovascular diseases mortality		
Rural		Urban		Rural
Adjusted HR (95% CI)	P value	Adjusted HR (95% CI)	P value	Adjusted HR (95% CI)
0.93 (0.84, 1.03)	2.59E-01	0.95 (0.79, 1.14)	8.60E-01	0.88 (0.79, 0.98)
0.93 (0.84, 1.03)	2.75E-01	0.95 (0.79, 1.14)	8.66E-01	0.88 (0.79, 0.98)
0.95 (0.86, 1.05)	4.04E-01	0.95 (0.79, 1.14)	8.60E-01	0.90 (0.81, 1.00)
0.95 (0.87, 1.05)	4.44E-01	0.99 (0.83, 1.18)	9.79E-01	0.91 (0.82, 1.01)
0.96 (0.87, 1.05)	4.67E-01	1.01 (0.85, 1.21)	9.79E-01	0.91 (0.82, 1.01)
0.95 (0.86, 1.04)	3.93E-01	0.96 (0.80, 1.15)	8.71E-01	0.91 (0.82, 1.01)
0.92 (0.84, 1.02)	2.21E-01	0.94 (0.78, 1.14)	8.46E-01	0.88 (0.79, 0.98)
0.94 (0.85, 1.03)	3.31E-01	0.97 (0.81, 1.16)	9.33E-01	0.91 (0.82, 1.01)
0.94 (0.85, 1.04)	3.49E-01	0.97 (0.81, 1.17)	9.41E-01	0.91 (0.82, 1.01)
0.95 (0.86, 1.04)	3.92E-01	0.97 (0.81, 1.16)	9.33E-01	0.90 (0.81, 1.00)
0.96 (0.87, 1.05)	4.74E-01	0.99 (0.83, 1.19)	9.79E-01	0.92 (0.83, 1.02)
0.96 (0.87, 1.05)	4.74E-01	1.00 (0.84, 1.20)	9.94E-01	0.92 (0.84, 1.02)
0.96 (0.87, 1.05)	4.61E-01	0.98 (0.82, 1.17)	9.79E-01	0.91 (0.82, 1.01)
0.93 (0.85, 1.03)	2.87E-01	0.97 (0.80, 1.16)	9.08E-01	0.91 (0.82, 1.01)
0.95 (0.87, 1.05)	4.26E-01	0.98 (0.82, 1.18)	9.79E-01	0.92 (0.83, 1.02)
0.95 (0.86, 1.05)	4.11E-01	0.98 (0.82, 1.18)	9.79E-01	0.92 (0.83, 1.02)
0.95 (0.86, 1.04)	3.94E-01	0.99 (0.82, 1.18)	9.79E-01	0.92 (0.83, 1.02)
0.93 (0.85, 1.03)	2.81E-01	0.98 (0.82, 1.18)	9.79E-01	0.91 (0.82, 1.01)
0.92 (0.83, 1.01)	1.53E-01	0.98 (0.81, 1.17)	9.79E-01	0.90 (0.81, 0.99)
0.95 (0.87, 1.05)	4.33E-01	0.98 (0.82, 1.18)	9.79E-01	0.93 (0.83, 1.03)
0.96 (0.87, 1.05)	4.68E-01	0.98 (0.82, 1.18)	9.79E-01	0.92 (0.83, 1.02)
0.96 (0.87, 1.05)	4.67E-01	0.99 (0.83, 1.19)	9.79E-01	0.92 (0.83, 1.02)
0.96 (0.87, 1.05)	4.46E-01	1.00 (0.83, 1.19)	9.79E-01	0.92 (0.83, 1.02)
0.95 (0.86, 1.04)	3.98E-01	0.99 (0.83, 1.19)	9.79E-01	0.92 (0.83, 1.02)
0.93 (0.85, 1.02)	2.49E-01	1.01 (0.84, 1.20)	9.79E-01	0.91 (0.83, 1.01)
0.90 (0.82, 1.00)	9.01E-02	1.01 (0.84, 1.21)	9.79E-01	0.90 (0.81, 1.00)
0.96 (0.88, 1.06)	5.06E-01	1.00 (0.84, 1.20)	9.84E-01	0.93 (0.84, 1.03)
0.97 (0.88, 1.06)	5.90E-01	0.99 (0.83, 1.19)	9.79E-01	0.93 (0.84, 1.03)
0.96 (0.88, 1.05)	4.83E-01	1.01 (0.84, 1.21)	9.79E-01	0.93 (0.84, 1.03)
0.97 (0.88, 1.06)	5.90E-01	1.02 (0.85, 1.22)	9.79E-01	0.94 (0.85, 1.04)
0.94 (0.86, 1.03)	3.31E-01	0.99 (0.83, 1.20)	9.79E-01	0.92 (0.84, 1.02)
1.06 (0.97, 1.15)	3.55E-01	1.10 (0.92, 1.32)	7.37E-01	1.02 (0.92, 1.12)
1.08 (0.99, 1.18)	1.59E-01	1.13 (0.94, 1.35)	6.07E-01	1.04 (0.94, 1.14)
1.00 (0.92, 1.10)	9.37E-01	1.05 (0.87, 1.26)	8.66E-01	0.98 (0.89, 1.08)
0.93 (0.85, 1.02)	2.45E-01	0.98 (0.82, 1.18)	9.79E-01	0.91 (0.82, 1.01)
1.03 (0.95, 1.13)	5.51E-01	1.10 (0.92, 1.32)	7.27E-01	1.02 (0.92, 1.12)
1.05 (0.96, 1.15)	3.85E-01	1.12 (0.93, 1.34)	6.64E-01	1.03 (0.94, 1.14)
0.97 (0.88, 1.06)	5.47E-01	1.05 (0.87, 1.26)	8.68E-01	0.97 (0.88, 1.07)
1.15 (1.06, 1.25)	**3.22E-03**	1.19 (0.99, 1.42)	2.79E-01	1.10 (1.01, 1.21)
1.16 (1.07, 1.26)	**1.55E-03**	1.22 (1.02, 1.45)	1.92E-01	1.11 (1.01, 1.21)

1.12 (1.03, 1.21)	**2.61E-02**	1.12 (0.94, 1.34)	6.16E-01	1.09 (0.99, 1.20)
0.93 (0.85, 1.03)	2.81E-01	1.00 (0.84, 1.21)	9.79E-01	0.96 (0.87, 1.06)
0.95 (0.87, 1.04)	3.85E-01	1.08 (0.90, 1.30)	8.12E-01	0.94 (0.85, 1.04)
0.96 (0.87, 1.05)	4.33E-01	1.08 (0.91, 1.30)	8.12E-01	0.94 (0.85, 1.04)
0.94 (0.85, 1.03)	2.75E-01	1.07 (0.89, 1.28)	8.42E-01	0.93 (0.84, 1.02)
0.97 (0.88, 1.06)	5.28E-01	1.08 (0.90, 1.30)	8.12E-01	0.94 (0.85, 1.04)
0.95 (0.87, 1.05)	4.28E-01	1.08 (0.90, 1.29)	8.27E-01	0.92 (0.84, 1.02)
0.99 (0.91, 1.09)	9.12E-01	1.09 (0.91, 1.30)	8.12E-01	0.98 (0.89, 1.08)
0.94 (0.86, 1.04)	3.85E-01	1.08 (0.91, 1.30)	8.12E-01	1.01 (0.91, 1.12)
0.94 (0.86, 1.03)	3.31E-01	1.07 (0.89, 1.28)	8.42E-01	0.92 (0.84, 1.02)
0.94 (0.86, 1.03)	3.41E-01	1.07 (0.89, 1.28)	8.44E-01	0.92 (0.84, 1.02)
0.91 (0.83, 1.00)	1.24E-01	1.03 (0.86, 1.24)	9.33E-01	0.89 (0.80, 0.98)
0.95 (0.87, 1.04)	3.98E-01	1.07 (0.89, 1.28)	8.42E-01	0.92 (0.84, 1.02)
0.95 (0.87, 1.04)	3.85E-01	1.07 (0.89, 1.28)	8.44E-01	0.92 (0.83, 1.01)
0.96 (0.88, 1.05)	4.80E-01	1.07 (0.90, 1.29)	8.38E-01	0.95 (0.86, 1.04)
0.97 (0.88, 1.07)	6.74E-01	1.12 (0.94, 1.33)	6.16E-01	1.04 (0.95, 1.15)
0.93 (0.85, 1.02)	2.31E-01	1.06 (0.88, 1.27)	8.46E-01	0.91 (0.83, 1.01)
0.93 (0.85, 1.02)	2.43E-01	1.06 (0.88, 1.27)	8.46E-01	0.91 (0.83, 1.00)
0.85 (0.78, 0.93)	**1.64E-03**	1.01 (0.84, 1.21)	9.79E-01	0.85 (0.78, 0.93)
0.96 (0.88, 1.05)	4.74E-01	1.06 (0.89, 1.27)	8.45E-01	0.93 (0.84, 1.02)
0.97 (0.89, 1.06)	5.90E-01	1.07 (0.89, 1.28)	8.44E-01	0.93 (0.84, 1.03)
0.92 (0.84, 1.00)	1.19E-01	1.05 (0.88, 1.26)	8.60E-01	0.90 (0.82, 0.99)
0.93 (0.84, 1.03)	2.84E-01	1.12 (0.94, 1.33)	6.16E-01	1.03 (0.93, 1.14)
0.95 (0.87, 1.04)	3.85E-01	1.06 (0.88, 1.27)	8.55E-01	0.92 (0.83, 1.01)
0.95 (0.87, 1.04)	4.01E-01	1.06 (0.88, 1.27)	8.55E-01	0.92 (0.83, 1.01)
0.87 (0.80, 0.95)	**5.63E-03**	0.99 (0.83, 1.19)	9.79E-01	0.86 (0.79, 0.94)
0.98 (0.90, 1.08)	7.95E-01	1.07 (0.89, 1.28)	8.42E-01	0.94 (0.85, 1.04)
0.99 (0.91, 1.09)	9.09E-01	1.07 (0.89, 1.28)	8.42E-01	0.95 (0.86, 1.05)
0.95 (0.87, 1.04)	3.85E-01	1.05 (0.88, 1.26)	8.64E-01	0.92 (0.83, 1.01)
0.95 (0.86, 1.04)	3.85E-01	1.06 (0.89, 1.27)	8.46E-01	0.98 (0.89, 1.09)
1.22 (1.12, 1.32)	**2.94E-05**	1.20 (1.01, 1.43)	1.99E-01	1.23 (1.12, 1.34)
1.22 (1.12, 1.33)	**2.14E-05**	1.20 (1.01, 1.43)	2.00E-01	1.23 (1.12, 1.34)
1.14 (1.04, 1.24)	**9.95E-03**	1.15 (0.97, 1.37)	4.24E-01	1.20 (1.09, 1.31)
1.31 (1.20, 1.42)	**5.00E-09**	1.23 (1.04, 1.47)	1.40E-01	1.25 (1.14, 1.37)
1.35 (1.24, 1.46)	**9.75E-11**	1.26 (1.05, 1.50)	9.67E-02	1.27 (1.16, 1.39)
1.21 (1.11, 1.31)	**5.76E-05**	1.18 (0.99, 1.40)	3.13E-01	1.20 (1.09, 1.31)
1.13 (1.03, 1.23)	**2.04E-02**	1.23 (1.05, 1.43)	8.63E-02	1.14 (1.04, 1.25)
1.09 (1.00, 1.19)	1.06E-01	1.06 (0.89, 1.27)	8.44E-01	1.16 (1.06, 1.27)
1.10 (1.00, 1.20)	9.01E-02	1.06 (0.89, 1.27)	8.45E-01	1.16 (1.06, 1.28)
1.09 (1.00, 1.20)	1.01E-01	1.04 (0.87, 1.25)	8.68E-01	1.15 (1.05, 1.27)
1.11 (1.01, 1.21)	5.42E-02	1.06 (0.89, 1.27)	8.46E-01	1.17 (1.07, 1.28)
1.10 (1.01, 1.20)	8.69E-02	1.05 (0.88, 1.26)	8.56E-01	1.16 (1.06, 1.28)
1.14 (1.04, 1.24)	**1.03E-02**	1.08 (0.90, 1.29)	8.37E-01	1.19 (1.08, 1.30)
0.96 (0.87, 1.06)	4.90E-01	1.24 (1.05, 1.47)	9.67E-02	1.12 (1.02, 1.23)
0.81 (0.74, 0.89)	**1.26E-04**	0.78 (0.64, 0.93)	8.03E-02	0.86 (0.78, 0.95)
0.82 (0.75, 0.90)	**2.74E-04**	0.77 (0.64, 0.93)	8.03E-02	0.87 (0.78, 0.96)
0.85 (0.77, 0.93)	**3.16E-03**	0.84 (0.69, 1.00)	2.77E-01	0.90 (0.81, 1.00)
0.85 (0.77, 0.93)	**1.85E-03**	0.74 (0.62, 0.90)	**4.12E-02**	0.87 (0.79, 0.96)

0.84 (0.77, 0.92)	**1.23E-03**	0.73 (0.60, 0.88)	**2.24E-02**	0.86 (0.78, 0.95)
0.87 (0.79, 0.96)	**1.22E-02**	0.81 (0.67, 0.97)	1.74E-01	0.92 (0.83, 1.01)
0.70 (0.63, 0.77)	**1.57E-10**	0.87 (0.72, 1.05)	5.27E-01	0.78 (0.70, 0.87)
0.75 (0.68, 0.82)	**1.48E-09**	0.82 (0.72, 0.94)	7.62E-02	0.77 (0.70, 0.84)
0.75 (0.69, 0.82)	**5.00E-09**	0.83 (0.72, 0.95)	7.62E-02	0.77 (0.70, 0.85)
0.81 (0.73, 0.89)	**4.24E-05**	0.80 (0.68, 0.94)	8.03E-02	0.82 (0.74, 0.91)
0.85 (0.78, 0.93)	**1.32E-03**	0.92 (0.77, 1.09)	7.61E-01	0.85 (0.78, 0.94)
0.88 (0.80, 0.96)	**1.00E-02**	0.95 (0.80, 1.13)	8.60E-01	0.88 (0.80, 0.96)
0.85 (0.77, 0.93)	**1.62E-03**	0.82 (0.70, 0.96)	1.29E-01	0.86 (0.77, 0.95)
0.79 (0.71, 0.87)	**2.58E-05**	0.98 (0.81, 1.18)	9.79E-01	0.85 (0.77, 0.94)
0.98 (0.90, 1.08)	7.87E-01	0.93 (0.77, 1.12)	8.38E-01	1.03 (0.93, 1.15)
1.11 (1.02, 1.20)	**2.85E-02**	1.32 (1.09, 1.61)	7.62E-02	1.17 (1.09, 1.25)
1.12 (1.03, 1.21)	**1.47E-02**	1.36 (1.14, 1.62)	**2.24E-02**	1.16 (1.08, 1.25)
1.01 (0.92, 1.12)	8.17E-01	1.00 (0.82, 1.20)	9.79E-01	1.09 (1.00, 1.19)
0.91 (0.83, 0.99)	8.52E-02	0.82 (0.67, 1.02)	3.35E-01	0.85 (0.78, 0.92)
0.94 (0.86, 1.04)	3.47E-01	0.87 (0.74, 1.03)	4.24E-01	0.91 (0.83, 1.00)
1.09 (1.02, 1.17)	**4.24E-02**	1.16 (1.01, 1.34)	2.08E-01	1.13 (1.05, 1.22)
1.13 (1.04, 1.23)	**7.69E-03**	1.19 (1.05, 1.34)	7.62E-02	1.19 (1.10, 1.30)
0.98 (0.89, 1.08)	7.95E-01	0.95 (0.80, 1.14)	8.66E-01	0.96 (0.87, 1.06)
0.95 (0.86, 1.05)	4.30E-01	0.96 (0.79, 1.16)	8.71E-01	0.95 (0.86, 1.05)
0.86 (0.79, 0.94)	**2.65E-03**	0.98 (0.82, 1.17)	9.79E-01	0.97 (0.88, 1.08)
0.85 (0.78, 0.92)	**3.46E-04**	0.96 (0.80, 1.14)	8.66E-01	0.95 (0.86, 1.05)
0.85 (0.77, 0.94)	**3.19E-03**	0.93 (0.77, 1.14)	8.45E-01	0.93 (0.84, 1.03)
0.92 (0.85, 1.01)	1.59E-01	1.00 (0.83, 1.20)	9.94E-01	1.00 (0.91, 1.11)
1.18 (1.09, 1.28)	**2.25E-04**	1.04 (0.87, 1.25)	8.71E-01	1.05 (0.95, 1.16)
0.83 (0.75, 0.91)	**6.23E-04**	0.86 (0.71, 1.04)	4.24E-01	0.90 (0.81, 0.99)
0.83 (0.76, 0.92)	**7.33E-04**	1.06 (0.87, 1.29)	8.46E-01	0.97 (0.88, 1.07)
0.84 (0.76, 0.92)	**1.10E-03**	1.01 (0.83, 1.23)	9.79E-01	0.95 (0.86, 1.06)
0.98 (0.90, 1.08)	7.80E-01	1.13 (0.93, 1.37)	6.64E-01	1.05 (0.95, 1.17)
1.22 (1.11, 1.34)	**2.20E-04**	0.97 (0.80, 1.18)	9.79E-01	1.05 (0.95, 1.16)
0.86 (0.78, 0.95)	**7.97E-03**	0.81 (0.67, 0.99)	2.02E-01	0.92 (0.83, 1.02)
1.20 (1.09, 1.31)	**5.18E-04**	1.16 (0.96, 1.40)	4.35E-01	1.17 (1.06, 1.29)
1.18 (1.07, 1.29)	**1.89E-03**	1.14 (0.95, 1.37)	5.39E-01	1.14 (1.03, 1.26)
1.24 (1.14, 1.36)	**7.69E-06**	1.21 (1.02, 1.44)	1.92E-01	1.25 (1.14, 1.37)
0.87 (0.79, 0.95)	**5.72E-03**	0.91 (0.76, 1.09)	7.61E-01	0.88 (0.79, 0.97)
0.84 (0.78, 0.91)	**9.79E-05**	0.87 (0.74, 1.02)	3.99E-01	0.87 (0.80, 0.94)
1.45 (1.31, 1.61)	**9.09E-11**	1.30 (1.06, 1.59)	1.05E-01	1.27 (1.14, 1.42)
1.37 (1.25, 1.51)	**2.69E-09**	1.33 (1.08, 1.62)	7.82E-02	1.22 (1.10, 1.35)
1.30 (1.17, 1.45)	**2.22E-05**	1.10 (0.91, 1.33)	7.88E-01	1.20 (1.08, 1.34)
0.85 (0.77, 0.94)	**3.59E-03**	0.89 (0.74, 1.08)	6.87E-01	0.92 (0.83, 1.02)
0.92 (0.84, 1.00)	1.18E-01	0.92 (0.78, 1.10)	8.12E-01	0.92 (0.84, 1.02)
1.05 (0.95, 1.14)	4.46E-01	1.04 (0.86, 1.25)	8.82E-01	0.97 (0.89, 1.07)
0.98 (0.89, 1.07)	6.74E-01	0.99 (0.82, 1.19)	9.79E-01	0.91 (0.83, 1.00)
1.11 (1.00, 1.22)	1.06E-01	1.06 (0.88, 1.28)	8.45E-01	1.08 (0.97, 1.20)
0.99 (0.90, 1.08)	8.25E-01	0.99 (0.83, 1.19)	9.79E-01	1.05 (0.96, 1.15)
1.01 (0.92, 1.10)	9.23E-01	0.79 (0.64, 0.98)	1.99E-01	0.97 (0.87, 1.07)
0.97 (0.89, 1.05)	5.28E-01	1.11 (0.91, 1.34)	7.63E-01	0.96 (0.88, 1.05)
0.94 (0.86, 1.02)	2.22E-01	1.08 (0.89, 1.31)	8.37E-01	0.93 (0.86, 1.02)

1.12 (1.01, 1.23)	7.27E-02	1.08 (0.89, 1.30)	8.37E-01	1.10 (0.99, 1.22)
1.05 (0.97, 1.15)	3.75E-01	1.01 (0.85, 1.20)	9.79E-01	1.09 (1.00, 1.18)
0.92 (0.83, 1.02)	1.92E-01	0.85 (0.69, 1.05)	4.43E-01	0.96 (0.86, 1.06)
1.05 (0.95, 1.15)	4.33E-01	1.10 (0.91, 1.33)	8.03E-01	0.99 (0.90, 1.09)
1.02 (0.93, 1.12)	7.41E-01	1.09 (0.90, 1.32)	8.12E-01	0.98 (0.89, 1.07)
1.05 (0.96, 1.15)	3.77E-01	0.94 (0.78, 1.14)	8.46E-01	1.06 (0.96, 1.16)
1.03 (0.94, 1.12)	6.71E-01	1.04 (0.87, 1.23)	8.86E-01	1.11 (1.02, 1.22)
0.92 (0.83, 1.01)	1.64E-01	0.85 (0.69, 1.04)	4.24E-01	0.96 (0.87, 1.06)
1.07 (0.97, 1.17)	3.27E-01	1.12 (0.92, 1.37)	6.87E-01	1.01 (0.91, 1.11)
1.04 (0.95, 1.14)	5.06E-01	1.12 (0.92, 1.36)	7.27E-01	0.99 (0.90, 1.09)
1.06 (0.97, 1.17)	2.92E-01	0.95 (0.79, 1.15)	8.66E-01	1.06 (0.96, 1.17)
1.00 (0.91, 1.09)	9.37E-01	0.98 (0.82, 1.18)	9.79E-01	1.07 (0.97, 1.17)
0.81 (0.74, 0.88)	**1.06E-05**	0.90 (0.75, 1.08)	6.99E-01	0.92 (0.84, 1.02)
1.38 (1.25, 1.51)	**7.71E-10**	1.14 (0.94, 1.39)	6.08E-01	1.14 (1.03, 1.26)
1.38 (1.26, 1.51)	**1.22E-10**	1.17 (0.97, 1.43)	4.14E-01	1.14 (1.03, 1.26)
1.13 (1.02, 1.24)	**3.53E-02**	0.94 (0.79, 1.12)	8.45E-01	1.04 (0.94, 1.16)
0.88 (0.80, 0.98)	**4.06E-02**	1.01 (0.84, 1.21)	9.79E-01	0.92 (0.83, 1.02)
0.91 (0.83, 1.00)	9.71E-02	0.96 (0.80, 1.16)	9.08E-01	0.85 (0.77, 0.94)
1.20 (1.09, 1.32)	**1.32E-03**	0.98 (0.81, 1.18)	9.79E-01	1.20 (1.08, 1.33)
1.08 (0.98, 1.18)	2.23E-01	0.92 (0.77, 1.09)	7.88E-01	1.11 (1.00, 1.23)
1.35 (1.22, 1.51)	**5.14E-07**	1.11 (0.91, 1.36)	7.61E-01	1.29 (1.15, 1.45)
0.85 (0.76, 0.94)	**4.73E-03**	1.10 (0.91, 1.33)	7.61E-01	0.96 (0.86, 1.06)
1.18 (1.10, 1.26)	**1.56E-05**	1.33 (1.22, 1.45)	**1.71E-08**	1.22 (1.14, 1.30)
1.00 (0.91, 1.11)	9.37E-01	0.79 (0.69, 0.91)	**2.24E-02**	0.90 (0.83, 0.99)
1.01 (0.91, 1.11)	9.23E-01	0.79 (0.70, 0.90)	**2.13E-02**	0.89 (0.82, 0.97)
0.99 (0.91, 1.09)	9.24E-01	0.88 (0.74, 1.04)	4.43E-01	1.00 (0.90, 1.10)
0.80 (0.72, 0.89)	**9.79E-05**	1.02 (0.85, 1.24)	9.79E-01	0.91 (0.81, 1.01)
1.01 (0.92, 1.11)	8.69E-01	0.89 (0.74, 1.07)	6.16E-01	1.00 (0.90, 1.10)
1.03 (0.94, 1.13)	6.32E-01	1.09 (0.90, 1.31)	8.12E-01	1.02 (0.92, 1.12)
1.01 (0.92, 1.11)	8.78E-01	1.08 (0.90, 1.30)	8.17E-01	1.00 (0.91, 1.11)
1.14 (1.04, 1.24)	**1.31E-02**	0.95 (0.79, 1.15)	8.66E-01	1.10 (1.00, 1.22)
0.88 (0.79, 0.97)	**2.10E-02**	1.07 (0.89, 1.28)	8.42E-01	0.95 (0.86, 1.05)
0.94 (0.85, 1.03)	2.81E-01	0.98 (0.82, 1.17)	9.79E-01	0.93 (0.84, 1.02)
0.95 (0.87, 1.05)	4.46E-01	1.06 (0.88, 1.28)	8.46E-01	1.04 (0.94, 1.14)
1.21 (1.11, 1.32)	**1.07E-04**	1.16 (0.97, 1.39)	4.07E-01	1.26 (1.14, 1.38)
1.01 (0.93, 1.10)	8.61E-01	1.08 (0.90, 1.29)	8.37E-01	0.98 (0.89, 1.08)
1.02 (0.93, 1.11)	7.70E-01	1.07 (0.89, 1.28)	8.42E-01	0.98 (0.89, 1.08)
1.00 (0.91, 1.09)	9.46E-01	1.09 (0.91, 1.31)	8.03E-01	0.96 (0.87, 1.06)
0.98 (0.90, 1.07)	7.17E-01	1.07 (0.89, 1.28)	8.42E-01	0.94 (0.85, 1.03)
1.07 (0.98, 1.17)	2.75E-01	0.99 (0.83, 1.19)	9.79E-01	1.09 (0.99, 1.20)
1.04 (0.95, 1.14)	5.11E-01	0.96 (0.80, 1.16)	8.86E-01	1.07 (0.97, 1.18)
1.31 (1.20, 1.42)	**6.34E-09**	1.27 (1.06, 1.51)	8.63E-02	1.23 (1.12, 1.36)
0.79 (0.71, 0.87)	**1.21E-05**	0.96 (0.79, 1.16)	8.71E-01	0.82 (0.74, 0.91)
0.83 (0.76, 0.92)	**1.33E-03**	0.90 (0.74, 1.09)	7.27E-01	0.84 (0.76, 0.93)
0.95 (0.86, 1.04)	4.01E-01	1.00 (0.84, 1.20)	9.79E-01	0.94 (0.85, 1.04)
0.96 (0.88, 1.06)	5.06E-01	0.99 (0.83, 1.18)	9.79E-01	0.93 (0.84, 1.03)
0.97 (0.88, 1.07)	6.44E-01	1.12 (0.95, 1.33)	5.88E-01	1.04 (0.94, 1.15)
0.82 (0.75, 0.91)	**8.49E-04**	1.05 (0.88, 1.26)	8.56E-01	0.94 (0.85, 1.04)

0.93 (0.85, 1.02)	2.38E-01	0.86 (0.71, 1.04)	4.24E-01	0.94 (0.85, 1.03)
0.99 (0.91, 1.09)	9.11E-01	1.00 (0.83, 1.19)	9.90E-01	0.96 (0.87, 1.06)
0.77 (0.70, 0.84)	**4.95E-07**	0.75 (0.62, 0.90)	**4.73E-02**	0.82 (0.74, 0.90)
0.75 (0.68, 0.82)	**2.87E-08**	0.77 (0.64, 0.94)	8.53E-02	0.79 (0.71, 0.87)
0.86 (0.79, 0.95)	**7.26E-03**	0.83 (0.69, 1.00)	2.77E-01	0.91 (0.82, 1.00)
1.08 (0.98, 1.18)	2.14E-01	1.02 (0.85, 1.22)	9.79E-01	1.07 (0.97, 1.18)
0.96 (0.87, 1.05)	4.51E-01	1.05 (0.87, 1.26)	8.66E-01	0.92 (0.83, 1.01)
0.92 (0.84, 1.01)	1.75E-01	1.04 (0.87, 1.25)	8.73E-01	0.89 (0.81, 0.99)
0.86 (0.78, 0.95)	**6.53E-03**	0.85 (0.70, 1.03)	4.04E-01	0.86 (0.77, 0.95)
0.72 (0.67, 0.78)	**7.03E-16**	0.68 (0.59, 0.79)	**2.30E-05**	0.76 (0.70, 0.82)
1.37 (1.27, 1.48)	**3.41E-15**	1.08 (0.90, 1.29)	8.17E-01	1.22 (1.12, 1.33)
0.82 (0.74, 0.90)	**2.01E-04**	0.80 (0.67, 0.97)	1.73E-01	0.85 (0.77, 0.94)
1.15 (1.06, 1.25)	**4.56E-03**	0.93 (0.77, 1.12)	8.41E-01	1.04 (0.95, 1.15)
0.77 (0.70, 0.85)	**3.14E-06**	0.80 (0.66, 0.97)	1.74E-01	0.81 (0.73, 0.90)
0.82 (0.75, 0.91)	**5.20E-04**	0.82 (0.67, 0.99)	2.17E-01	0.84 (0.76, 0.93)
0.89 (0.81, 0.98)	**4.76E-02**	0.90 (0.75, 1.09)	7.27E-01	0.90 (0.81, 1.00)
0.87 (0.79, 0.96)	**1.80E-02**	0.85 (0.70, 1.03)	3.99E-01	0.85 (0.77, 0.94)
1.79 (1.64, 1.95)	**3.93E-37**	1.32 (1.11, 1.58)	**4.12E-02**	1.49 (1.34, 1.65)
0.84 (0.77, 0.91)	**1.85E-04**	0.84 (0.72, 0.98)	1.74E-01	0.91 (0.84, 1.00)
1.46 (1.34, 1.58)	**3.13E-17**	1.08 (0.91, 1.29)	8.12E-01	1.25 (1.14, 1.38)
0.79 (0.72, 0.86)	**1.58E-06**	0.86 (0.72, 1.03)	3.99E-01	0.87 (0.79, 0.95)
0.88 (0.81, 0.97)	**1.86E-02**	0.87 (0.73, 1.05)	4.86E-01	0.92 (0.84, 1.01)
1.06 (0.97, 1.16)	3.51E-01	1.13 (0.95, 1.36)	5.54E-01	1.07 (0.97, 1.18)
1.14 (1.04, 1.26)	**1.47E-02**	1.05 (0.87, 1.26)	8.66E-01	1.04 (0.94, 1.15)
1.11 (1.04, 1.19)	**7.36E-03**	1.14 (0.99, 1.32)	3.35E-01	1.21 (1.13, 1.29)
1.12 (1.03, 1.22)	**2.08E-02**	0.93 (0.77, 1.12)	8.37E-01	1.08 (0.99, 1.19)
0.80 (0.72, 0.88)	**5.25E-05**	0.86 (0.69, 1.05)	4.87E-01	0.83 (0.74, 0.93)
0.98 (0.90, 1.08)	7.91E-01	0.78 (0.65, 0.95)	1.00E-01	1.03 (0.93, 1.13)
0.97 (0.88, 1.06)	5.45E-01	1.08 (0.90, 1.30)	8.12E-01	1.02 (0.93, 1.13)
1.24 (1.13, 1.35)	**1.90E-05**	0.97 (0.80, 1.17)	9.33E-01	1.19 (1.08, 1.30)
0.93 (0.85, 1.02)	2.44E-01	0.89 (0.73, 1.08)	6.64E-01	0.93 (0.84, 1.03)
1.09 (1.00, 1.19)	1.22E-01	0.96 (0.79, 1.16)	8.82E-01	0.98 (0.89, 1.08)
0.79 (0.72, 0.88)	**3.51E-05**	0.72 (0.58, 0.88)	**3.18E-02**	0.81 (0.72, 0.89)
1.19 (1.14, 1.24)	**3.44E-12**	1.25 (1.07, 1.47)	7.62E-02	1.16 (1.10, 1.22)
1.08 (0.98, 1.18)	2.06E-01	1.11 (0.93, 1.33)	6.72E-01	1.12 (1.02, 1.24)
1.10 (1.02, 1.19)	**3.49E-02**	1.20 (1.13, 1.29)	**5.81E-06**	1.09 (1.02, 1.17)
0.75 (0.66, 0.85)	**3.24E-05**	0.95 (0.78, 1.15)	8.60E-01	1.02 (0.93, 1.12)
0.88 (0.78, 0.99)	8.52E-02	1.07 (0.92, 1.24)	8.12E-01	0.98 (0.88, 1.09)
0.98 (0.88, 1.09)	7.91E-01	1.09 (1.01, 1.18)	1.92E-01	1.19 (1.13, 1.27)
0.73 (0.68, 0.78)	**6.59E-18**	0.73 (0.65, 0.84)	**1.55E-04**	0.79 (0.73, 0.86)
1.31 (1.20, 1.42)	**2.73E-09**	1.17 (0.99, 1.39)	2.80E-01	1.18 (1.08, 1.29)

	Cancer mortality			
	Urban		Rural	
P value	Adjusted HR (95% CI)	P value	Adjusted HR (95% CI)	P value
6.22E-02	1.07 (0.95, 1.20)	4.94E-01	0.81 (0.71, 0.91)	**2.31E-03**
6.82E-02	1.07 (0.96, 1.20)	4.87E-01	0.81 (0.71, 0.91)	**2.53E-03**
1.17E-01	1.08 (0.97, 1.21)	4.54E-01	0.80 (0.71, 0.91)	**2.12E-03**
1.52E-01	1.09 (0.98, 1.22)	4.14E-01	0.86 (0.77, 0.96)	**2.48E-02**
1.54E-01	1.09 (0.97, 1.21)	4.27E-01	0.89 (0.80, 0.99)	8.19E-02
1.47E-01	1.11 (0.99, 1.24)	4.14E-01	0.83 (0.74, 0.94)	**8.25E-03**
**4.98E-02**	1.07 (0.95, 1.20)	5.17E-01	0.80 (0.70, 0.90)	**1.50E-03**
1.46E-01	1.13 (1.01, 1.27)	3.14E-01	0.85 (0.75, 0.95)	**1.51E-02**
1.46E-01	1.13 (1.01, 1.27)	3.14E-01	0.85 (0.75, 0.95)	**1.58E-02**
1.33E-01	1.13 (1.01, 1.26)	3.14E-01	0.85 (0.76, 0.96)	**2.06E-02**
1.87E-01	1.13 (1.01, 1.26)	3.14E-01	0.87 (0.77, 0.97)	**3.31E-02**
2.09E-01	1.13 (1.01, 1.26)	3.14E-01	0.87 (0.77, 0.97)	**3.21E-02**
1.49E-01	1.13 (1.01, 1.26)	3.14E-01	0.87 (0.77, 0.98)	**4.29E-02**
1.40E-01	1.13 (1.01, 1.27)	3.14E-01	0.84 (0.75, 0.95)	**1.32E-02**
1.90E-01	1.09 (0.98, 1.22)	4.14E-01	0.81 (0.72, 0.91)	**2.19E-03**
1.88E-01	1.09 (0.98, 1.22)	4.14E-01	0.82 (0.73, 0.92)	**2.33E-03**
1.87E-01	1.09 (0.98, 1.22)	4.17E-01	0.82 (0.73, 0.92)	**2.58E-03**
1.52E-01	1.09 (0.98, 1.22)	4.14E-01	0.84 (0.75, 0.94)	**6.19E-03**
1.02E-01	1.09 (0.98, 1.21)	4.17E-01	0.85 (0.76, 0.95)	**1.14E-02**
2.15E-01	1.10 (0.98, 1.23)	4.14E-01	0.82 (0.73, 0.93)	**3.69E-03**
1.97E-01	1.09 (0.98, 1.22)	4.14E-01	0.81 (0.72, 0.91)	**1.62E-03**
1.97E-01	1.08 (0.97, 1.20)	4.58E-01	0.81 (0.73, 0.90)	**9.56E-04**
1.97E-01	1.07 (0.97, 1.20)	4.58E-01	0.81 (0.73, 0.91)	**1.23E-03**
1.79E-01	1.07 (0.96, 1.19)	4.72E-01	0.82 (0.73, 0.91)	**1.27E-03**
1.64E-01	1.07 (0.96, 1.19)	4.87E-01	0.86 (0.77, 0.96)	**1.41E-02**
1.02E-01	1.05 (0.95, 1.17)	5.50E-01	0.90 (0.81, 1.00)	8.48E-02
2.64E-01	1.08 (0.97, 1.20)	4.31E-01	0.82 (0.74, 0.92)	**2.09E-03**
2.33E-01	1.08 (0.97, 1.20)	4.54E-01	0.79 (0.71, 0.89)	**3.67E-04**
2.57E-01	1.06 (0.95, 1.17)	5.29E-01	0.84 (0.76, 0.94)	**4.19E-03**
3.24E-01	1.05 (0.95, 1.17)	5.50E-01	0.85 (0.77, 0.94)	**6.05E-03**
1.99E-01	1.05 (0.94, 1.16)	5.92E-01	0.84 (0.76, 0.94)	**4.19E-03**
7.61E-01	1.01 (0.91, 1.12)	9.09E-01	0.92 (0.84, 1.02)	1.88E-01
5.24E-01	0.99 (0.89, 1.10)	9.10E-01	0.96 (0.88, 1.06)	6.09E-01
7.61E-01	1.05 (0.95, 1.16)	5.68E-01	0.86 (0.78, 0.96)	**1.34E-02**
1.46E-01	1.08 (0.97, 1.19)	4.54E-01	0.82 (0.74, 0.92)	**1.53E-03**
7.61E-01	0.99 (0.89, 1.09)	9.00E-01	0.97 (0.88, 1.06)	6.42E-01
5.90E-01	0.97 (0.88, 1.08)	8.09E-01	0.98 (0.89, 1.07)	7.40E-01
5.76E-01	0.95 (0.86, 1.06)	5.79E-01	1.03 (0.93, 1.13)	7.30E-01
1.00E-01	0.94 (0.85, 1.04)	4.94E-01	1.00 (0.91, 1.10)	9.93E-01
9.07E-02	0.94 (0.85, 1.04)	4.75E-01	1.00 (0.91, 1.10)	9.91E-01

1.42E-01	0.95 (0.86, 1.06)	5.68E-01	1.00 (0.91, 1.10)	9.91E-01
4.86E-01	1.07 (0.97, 1.19)	4.54E-01	0.89 (0.80, 0.98)	**4.49E-02**
3.16E-01	0.95 (0.86, 1.05)	5.50E-01	1.04 (0.95, 1.14)	5.82E-01
3.25E-01	0.94 (0.85, 1.05)	5.21E-01	1.05 (0.95, 1.15)	5.08E-01
1.97E-01	0.93 (0.84, 1.03)	4.58E-01	1.05 (0.96, 1.16)	4.16E-01
3.02E-01	0.94 (0.85, 1.04)	4.91E-01	1.05 (0.96, 1.15)	4.27E-01
1.96E-01	0.95 (0.85, 1.05)	5.28E-01	1.03 (0.94, 1.13)	6.42E-01
7.68E-01	0.93 (0.83, 1.03)	4.31E-01	1.09 (1.00, 1.20)	1.01E-01
8.68E-01	1.05 (0.95, 1.16)	5.50E-01	0.98 (0.89, 1.09)	8.13E-01
1.97E-01	0.94 (0.85, 1.04)	4.94E-01	1.02 (0.93, 1.13)	7.40E-01
1.97E-01	0.94 (0.84, 1.04)	4.87E-01	1.03 (0.93, 1.13)	7.20E-01
6.02E-02	0.94 (0.84, 1.04)	4.87E-01	1.03 (0.93, 1.13)	7.29E-01
1.97E-01	0.93 (0.84, 1.03)	4.54E-01	1.03 (0.93, 1.13)	7.20E-01
1.66E-01	0.93 (0.84, 1.04)	4.62E-01	1.01 (0.92, 1.11)	8.69E-01
3.62E-01	0.92 (0.83, 1.02)	4.27E-01	1.07 (0.98, 1.18)	2.24E-01
4.76E-01	1.04 (0.94, 1.15)	6.21E-01	1.02 (0.92, 1.13)	7.63E-01
1.41E-01	0.92 (0.83, 1.03)	4.31E-01	0.98 (0.89, 1.09)	8.42E-01
1.38E-01	0.92 (0.83, 1.03)	4.27E-01	0.99 (0.89, 1.09)	8.55E-01
**4.10E-03**	0.94 (0.84, 1.04)	4.91E-01	0.96 (0.87, 1.06)	5.99E-01
1.97E-01	0.91 (0.82, 1.02)	4.14E-01	0.99 (0.90, 1.09)	9.08E-01
2.33E-01	0.91 (0.82, 1.02)	4.14E-01	0.99 (0.90, 1.09)	8.84E-01
8.82E-02	0.91 (0.82, 1.02)	4.14E-01	1.00 (0.90, 1.10)	9.91E-01
6.46E-01	1.03 (0.93, 1.14)	8.08E-01	1.01 (0.91, 1.12)	8.91E-01
1.64E-01	0.93 (0.83, 1.03)	4.54E-01	0.96 (0.87, 1.06)	5.82E-01
1.66E-01	0.92 (0.83, 1.03)	4.31E-01	0.96 (0.87, 1.06)	6.05E-01
**5.58E-03**	0.95 (0.85, 1.06)	5.50E-01	0.93 (0.84, 1.03)	3.13E-01
3.10E-01	0.91 (0.82, 1.01)	4.14E-01	0.98 (0.89, 1.08)	7.76E-01
3.80E-01	0.91 (0.82, 1.01)	4.07E-01	0.98 (0.89, 1.08)	8.13E-01
1.47E-01	0.92 (0.83, 1.02)	4.21E-01	0.96 (0.86, 1.06)	5.82E-01
7.68E-01	1.06 (0.95, 1.17)	5.19E-01	0.90 (0.81, 1.00)	8.48E-02
**1.18E-04**	1.02 (0.93, 1.13)	8.19E-01	1.21 (1.11, 1.32)	**1.44E-04**
**1.18E-04**	1.02 (0.93, 1.12)	8.29E-01	1.21 (1.11, 1.32)	**1.84E-04**
**9.53E-04**	1.03 (0.93, 1.14)	7.49E-01	1.28 (1.17, 1.40)	**1.13E-06**
**4.27E-05**	1.01 (0.91, 1.11)	9.43E-01	1.13 (1.03, 1.24)	**1.96E-02**
**1.28E-05**	1.00 (0.90, 1.10)	9.51E-01	1.10 (1.00, 1.20)	8.29E-02
**8.71E-04**	1.03 (0.94, 1.13)	7.48E-01	1.21 (1.10, 1.32)	**1.84E-04**
**1.73E-02**	1.09 (0.98, 1.20)	4.17E-01	1.02 (0.92, 1.12)	8.31E-01
**6.26E-03**	1.02 (0.92, 1.13)	8.35E-01	1.24 (1.13, 1.36)	**3.21E-05**
**5.64E-03**	1.02 (0.92, 1.13)	8.36E-01	1.24 (1.13, 1.36)	**3.19E-05**
**9.91E-03**	1.01 (0.91, 1.12)	9.09E-01	1.23 (1.12, 1.35)	**7.61E-05**
**4.64E-03**	1.02 (0.93, 1.13)	8.19E-01	1.24 (1.14, 1.36)	**2.31E-05**
**6.08E-03**	1.03 (0.93, 1.14)	8.03E-01	1.24 (1.14, 1.36)	**2.31E-05**
**1.40E-03**	1.02 (0.92, 1.12)	9.00E-01	1.24 (1.14, 1.36)	**2.31E-05**
5.32E-02	1.05 (0.95, 1.16)	5.71E-01	1.17 (1.07, 1.29)	**3.42E-03**
**1.29E-02**	1.02 (0.92, 1.14)	8.35E-01	1.02 (0.92, 1.13)	7.89E-01
**1.87E-02**	1.02 (0.92, 1.14)	8.60E-01	1.03 (0.93, 1.13)	7.40E-01
1.09E-01	1.02 (0.91, 1.13)	9.00E-01	1.03 (0.93, 1.13)	7.40E-01
**2.30E-02**	1.01 (0.91, 1.13)	9.09E-01	1.04 (0.94, 1.15)	6.42E-01

**1.18E-02**	1.01 (0.91, 1.13)	9.09E-01	1.03 (0.93, 1.14)	7.12E-01
1.64E-01	1.01 (0.91, 1.12)	9.17E-01	1.06 (0.96, 1.17)	3.82E-01
**1.18E-04**	1.07 (0.96, 1.18)	4.87E-01	0.94 (0.85, 1.03)	3.17E-01
**9.37E-07**	1.01 (0.91, 1.13)	9.10E-01	0.91 (0.82, 1.00)	1.03E-01
**1.08E-06**	1.01 (0.90, 1.12)	9.51E-01	0.91 (0.83, 1.01)	1.44E-01
**9.52E-04**	1.05 (0.94, 1.17)	5.85E-01	0.88 (0.80, 0.97)	**2.97E-02**
**5.00E-03**	0.93 (0.83, 1.04)	4.87E-01	1.04 (0.94, 1.15)	6.05E-01
**2.21E-02**	0.92 (0.82, 1.03)	4.31E-01	1.06 (0.96, 1.18)	3.76E-01
**9.98E-03**	1.07 (0.96, 1.19)	4.94E-01	0.91 (0.82, 1.00)	1.17E-01
**9.91E-03**	1.07 (0.96, 1.19)	4.82E-01	0.87 (0.79, 0.97)	**2.06E-02**
6.22E-01	1.00 (0.90, 1.12)	9.79E-01	0.78 (0.72, 0.85)	**5.35E-07**
**2.32E-04**	1.13 (1.01, 1.27)	3.14E-01	1.17 (1.09, 1.27)	**3.32E-04**
**4.43E-04**	1.06 (0.95, 1.19)	5.17E-01	1.19 (1.10, 1.28)	**4.48E-05**
1.05E-01	1.17 (1.03, 1.31)	3.02E-01	1.01 (0.90, 1.13)	8.78E-01
**4.43E-04**	0.90 (0.80, 1.02)	4.14E-01	0.93 (0.84, 1.03)	3.00E-01
1.27E-01	1.02 (0.91, 1.14)	8.71E-01	0.87 (0.79, 0.95)	**1.10E-02**
**7.82E-03**	1.01 (0.93, 1.10)	9.00E-01	1.10 (1.02, 1.19)	**4.14E-02**
**3.85E-04**	0.99 (0.91, 1.09)	9.10E-01	1.14 (1.04, 1.24)	**1.15E-02**
5.24E-01	1.06 (0.94, 1.19)	5.50E-01	0.99 (0.89, 1.11)	9.38E-01
4.16E-01	0.98 (0.88, 1.08)	8.33E-01	0.99 (0.89, 1.10)	9.28E-01
6.55E-01	1.01 (0.90, 1.14)	9.17E-01	0.90 (0.81, 0.99)	6.85E-02
3.92E-01	1.01 (0.89, 1.13)	9.42E-01	1.07 (0.96, 1.19)	3.91E-01
2.33E-01	0.99 (0.89, 1.11)	9.37E-01	1.26 (1.15, 1.38)	**7.36E-06**
9.74E-01	1.01 (0.91, 1.13)	9.09E-01	0.84 (0.76, 0.92)	**1.44E-03**
4.18E-01	0.99 (0.88, 1.12)	9.17E-01	0.98 (0.88, 1.09)	7.99E-01
1.02E-01	1.00 (0.90, 1.11)	9.81E-01	1.16 (1.07, 1.27)	**2.60E-03**
6.24E-01	1.02 (0.91, 1.13)	9.00E-01	1.28 (1.17, 1.41)	**6.95E-06**
4.54E-01	1.00 (0.90, 1.11)	9.66E-01	1.31 (1.20, 1.43)	**2.15E-07**
4.03E-01	1.05 (0.94, 1.18)	5.71E-01	0.89 (0.81, 0.98)	**3.22E-02**
4.70E-01	0.98 (0.88, 1.10)	9.00E-01	0.78 (0.71, 0.86)	**3.54E-06**
1.96E-01	0.99 (0.89, 1.09)	9.00E-01	1.11 (1.02, 1.22)	**4.20E-02**
**7.82E-03**	0.95 (0.86, 1.05)	5.29E-01	1.13 (1.03, 1.25)	**2.86E-02**
**2.75E-02**	0.94 (0.85, 1.04)	4.94E-01	1.13 (1.02, 1.24)	**3.25E-02**
**3.98E-05**	1.01 (0.91, 1.12)	9.09E-01	1.09 (0.99, 1.20)	1.43E-01
**2.52E-02**	1.06 (0.95, 1.18)	5.17E-01	0.86 (0.78, 0.95)	**6.54E-03**
**4.64E-03**	0.95 (0.85, 1.06)	5.85E-01	1.17 (1.05, 1.31)	**1.14E-02**
**1.58E-04**	0.89 (0.80, 0.98)	3.14E-01	1.02 (0.93, 1.12)	7.63E-01
**1.20E-03**	0.89 (0.81, 0.98)	3.14E-01	1.01 (0.92, 1.11)	8.75E-01
**5.58E-03**	0.92 (0.82, 1.02)	4.14E-01	1.03 (0.94, 1.14)	6.42E-01
1.87E-01	1.12 (1.02, 1.25)	3.14E-01	0.91 (0.83, 1.01)	1.40E-01
1.90E-01	0.91 (0.82, 1.02)	4.14E-01	1.06 (0.96, 1.16)	3.90E-01
6.23E-01	0.91 (0.83, 1.00)	3.57E-01	1.04 (0.95, 1.14)	6.03E-01
1.05E-01	0.96 (0.87, 1.06)	5.79E-01	0.93 (0.85, 1.02)	1.89E-01
2.33E-01	0.90 (0.81, 1.00)	3.20E-01	1.19 (1.06, 1.34)	**8.73E-03**
3.44E-01	1.12 (1.02, 1.24)	3.14E-01	0.94 (0.85, 1.04)	3.70E-01
5.73E-01	1.05 (0.95, 1.17)	5.33E-01	0.97 (0.87, 1.08)	7.06E-01
4.70E-01	0.92 (0.84, 1.00)	3.57E-01	1.03 (0.93, 1.15)	7.25E-01
1.97E-01	0.94 (0.86, 1.02)	4.31E-01	0.96 (0.87, 1.06)	6.26E-01

1.40E-01	0.91 (0.82, 1.02)	4.14E-01	1.25 (1.11, 1.40)	**8.43E-04**
1.12E-01	1.11 (1.01, 1.21)	3.14E-01	0.98 (0.88, 1.09)	7.63E-01
4.66E-01	1.09 (0.99, 1.20)	4.14E-01	0.96 (0.86, 1.07)	6.05E-01
8.43E-01	0.90 (0.82, 0.99)	3.14E-01	1.02 (0.92, 1.13)	7.63E-01
6.68E-01	0.91 (0.83, 1.00)	3.81E-01	1.01 (0.91, 1.12)	9.01E-01
3.16E-01	1.05 (0.94, 1.17)	5.71E-01	1.03 (0.93, 1.14)	7.12E-01
5.30E-02	1.13 (1.03, 1.24)	3.02E-01	1.01 (0.91, 1.12)	8.75E-01
4.90E-01	1.10 (1.00, 1.21)	3.57E-01	0.99 (0.89, 1.09)	8.69E-01
9.24E-01	0.89 (0.81, 0.98)	3.14E-01	1.04 (0.94, 1.15)	6.42E-01
9.05E-01	0.90 (0.81, 0.99)	3.14E-01	1.03 (0.93, 1.14)	7.29E-01
3.18E-01	1.05 (0.95, 1.17)	5.68E-01	1.01 (0.91, 1.13)	8.58E-01
2.61E-01	1.15 (1.04, 1.26)	2.11E-01	0.92 (0.83, 1.02)	2.22E-01
1.87E-01	1.07 (0.96, 1.19)	4.94E-01	1.32 (1.19, 1.47)	**1.71E-06**
**4.23E-02**	0.92 (0.83, 1.01)	4.14E-01	0.79 (0.72, 0.87)	**7.72E-06**
**2.95E-02**	0.89 (0.80, 0.99)	3.14E-01	0.75 (0.68, 0.83)	**2.46E-07**
4.85E-01	1.02 (0.93, 1.12)	8.33E-01	1.01 (0.92, 1.11)	8.55E-01
1.99E-01	1.05 (0.95, 1.16)	5.50E-01	0.86 (0.77, 0.96)	**1.58E-02**
**7.82E-03**	0.95 (0.85, 1.06)	5.50E-01	0.83 (0.75, 0.92)	**2.09E-03**
**4.99E-03**	1.02 (0.92, 1.12)	8.86E-01	1.20 (1.08, 1.33)	**2.53E-03**
1.06E-01	1.05 (0.95, 1.16)	5.68E-01	1.18 (1.06, 1.30)	**5.65E-03**
**1.93E-04**	0.97 (0.88, 1.07)	7.69E-01	1.18 (1.06, 1.31)	**6.57E-03**
5.03E-01	1.05 (0.94, 1.16)	5.85E-01	0.96 (0.87, 1.06)	6.26E-01
**8.20E-08**	0.98 (0.87, 1.09)	8.35E-01	1.02 (0.92, 1.14)	7.63E-01
6.31E-02	0.97 (0.87, 1.08)	7.48E-01	1.02 (0.92, 1.13)	8.13E-01
**2.75E-02**	0.97 (0.87, 1.08)	7.69E-01	1.00 (0.90, 1.11)	9.80E-01
9.53E-01	0.98 (0.88, 1.08)	8.12E-01	1.09 (0.98, 1.21)	1.79E-01
1.40E-01	1.06 (0.96, 1.17)	5.17E-01	0.95 (0.86, 1.05)	4.87E-01
9.34E-01	1.08 (0.97, 1.21)	4.45E-01	0.90 (0.81, 0.99)	6.69E-02
7.61E-01	0.91 (0.82, 1.02)	4.14E-01	1.13 (1.02, 1.26)	**4.20E-02**
9.34E-01	0.91 (0.82, 1.01)	4.14E-01	1.13 (1.02, 1.25)	5.11E-02
1.05E-01	1.12 (1.01, 1.24)	3.14E-01	0.94 (0.85, 1.04)	4.16E-01
4.44E-01	1.08 (0.97, 1.20)	4.31E-01	0.91 (0.82, 1.01)	1.28E-01
2.15E-01	1.03 (0.92, 1.15)	7.69E-01	0.78 (0.70, 0.86)	**2.17E-05**
5.39E-01	1.09 (0.98, 1.21)	4.21E-01	1.25 (1.14, 1.38)	**4.48E-05**
**4.01E-05**	1.02 (0.93, 1.13)	8.22E-01	1.22 (1.11, 1.34)	**3.45E-04**
6.85E-01	0.96 (0.87, 1.06)	6.11E-01	1.04 (0.95, 1.14)	6.05E-01
7.12E-01	1.03 (0.93, 1.14)	7.61E-01	0.87 (0.79, 0.97)	**2.06E-02**
4.80E-01	0.99 (0.90, 1.10)	9.18E-01	0.93 (0.85, 1.03)	3.00E-01
2.52E-01	0.92 (0.83, 1.02)	4.27E-01	1.00 (0.91, 1.10)	9.91E-01
1.64E-01	1.01 (0.91, 1.12)	9.17E-01	1.19 (1.09, 1.30)	**1.04E-03**
2.52E-01	1.01 (0.91, 1.12)	9.09E-01	1.20 (1.09, 1.31)	**6.25E-04**
**1.33E-04**	0.99 (0.89, 1.09)	9.09E-01	1.08 (0.99, 1.19)	1.58E-01
**1.17E-03**	0.95 (0.85, 1.05)	5.50E-01	1.04 (0.94, 1.14)	6.42E-01
**5.58E-03**	0.95 (0.85, 1.05)	5.29E-01	1.03 (0.93, 1.14)	7.25E-01
2.85E-01	1.08 (0.97, 1.20)	4.54E-01	0.82 (0.74, 0.92)	**2.08E-03**
2.33E-01	1.08 (0.97, 1.20)	4.58E-01	0.80 (0.71, 0.89)	**4.58E-04**
5.33E-01	1.04 (0.94, 1.15)	6.22E-01	0.99 (0.90, 1.10)	9.38E-01
3.02E-01	1.08 (0.98, 1.20)	4.27E-01	0.98 (0.89, 1.09)	8.13E-01

2.70E-01	1.07 (0.97, 1.18)	4.75E-01	1.04 (0.94, 1.14)	6.42E-01
4.65E-01	1.10 (0.99, 1.23)	4.14E-01	0.79 (0.71, 0.89)	**2.78E-04**
**5.95E-04**	1.02 (0.92, 1.13)	8.63E-01	1.03 (0.93, 1.14)	7.40E-01
**4.01E-05**	0.87 (0.78, 0.97)	3.02E-01	1.05 (0.94, 1.16)	5.63E-01
1.24E-01	1.04 (0.94, 1.15)	7.05E-01	1.06 (0.96, 1.18)	3.70E-01
2.40E-01	1.02 (0.93, 1.13)	8.15E-01	1.12 (1.02, 1.23)	**4.14E-02**
1.64E-01	1.00 (0.90, 1.11)	9.98E-01	0.90 (0.82, 1.00)	9.20E-02
6.93E-02	0.99 (0.89, 1.10)	9.10E-01	0.86 (0.78, 0.95)	**8.91E-03**
**1.32E-02**	1.07 (0.96, 1.19)	4.72E-01	0.92 (0.83, 1.02)	2.14E-01
**8.96E-10**	0.93 (0.84, 1.04)	4.69E-01	1.04 (0.93, 1.15)	6.81E-01
**1.10E-04**	0.96 (0.87, 1.07)	6.37E-01	0.82 (0.74, 0.90)	**3.99E-04**
**6.79E-03**	1.03 (0.93, 1.15)	7.49E-01	0.95 (0.86, 1.05)	4.84E-01
4.59E-01	1.00 (0.90, 1.11)	9.70E-01	0.78 (0.70, 0.87)	**7.84E-05**
**4.57E-04**	1.03 (0.93, 1.15)	7.48E-01	0.97 (0.88, 1.08)	7.29E-01
**4.64E-03**	1.03 (0.93, 1.14)	7.86E-01	0.93 (0.84, 1.04)	3.21E-01
1.05E-01	1.07 (0.96, 1.19)	4.72E-01	0.88 (0.79, 0.98)	**4.43E-02**
**9.91E-03**	1.10 (0.99, 1.22)	3.94E-01	0.96 (0.87, 1.06)	5.97E-01
**2.77E-12**	0.89 (0.80, 0.99)	3.14E-01	0.81 (0.73, 0.89)	**1.30E-04**
1.02E-01	0.94 (0.84, 1.05)	5.29E-01	1.06 (0.95, 1.20)	4.51E-01
**4.54E-05**	0.91 (0.82, 1.01)	4.14E-01	0.72 (0.66, 0.80)	**2.89E-08**
**1.24E-02**	0.94 (0.84, 1.04)	4.87E-01	1.17 (1.05, 1.30)	**1.12E-02**
1.75E-01	0.90 (0.81, 1.01)	3.81E-01	1.06 (0.95, 1.18)	4.74E-01
2.27E-01	1.01 (0.91, 1.13)	9.09E-01	0.82 (0.74, 0.91)	**7.75E-04**
5.07E-01	1.16 (1.04, 1.28)	2.11E-01	1.20 (1.08, 1.32)	**2.31E-03**
**2.93E-07**	1.04 (0.94, 1.14)	6.21E-01	0.92 (0.82, 1.04)	3.35E-01
1.66E-01	0.89 (0.79, 1.00)	3.49E-01	0.67 (0.59, 0.76)	**6.35E-08**
**4.64E-03**	1.23 (1.10, 1.38)	**1.63E-02**	1.28 (1.16, 1.41)	**2.24E-05**
6.34E-01	0.94 (0.85, 1.05)	4.94E-01	0.69 (0.62, 0.77)	**3.75E-09**
7.25E-01	0.80 (0.72, 0.89)	**2.64E-03**	0.79 (0.72, 0.88)	**1.44E-04**
**1.62E-03**	0.78 (0.70, 0.88)	**2.64E-03**	0.72 (0.65, 0.80)	**6.35E-08**
2.49E-01	1.03 (0.92, 1.15)	8.05E-01	0.75 (0.67, 0.85)	**2.17E-05**
7.61E-01	0.95 (0.85, 1.06)	5.68E-01	0.72 (0.64, 0.81)	**1.14E-06**
**4.57E-04**	0.92 (0.82, 1.02)	4.21E-01	0.75 (0.67, 0.84)	**7.36E-06**
**2.39E-07**	0.96 (0.86, 1.07)	6.21E-01	0.76 (0.67, 0.86)	**1.22E-04**
5.05E-02	0.92 (0.83, 1.03)	4.27E-01	0.83 (0.74, 0.92)	**2.38E-03**
**4.49E-02**	0.93 (0.81, 1.07)	5.50E-01	0.98 (0.89, 1.09)	8.00E-01
7.61E-01	1.19 (1.11, 1.28)	**6.00E-04**	1.09 (0.99, 1.19)	1.44E-01
7.61E-01	1.10 (1.00, 1.20)	3.49E-01	1.10 (1.01, 1.18)	**4.73E-02**
**2.19E-07**	0.85 (0.64, 1.13)	5.12E-01	0.74 (0.64, 0.85)	**1.53E-04**
**9.69E-07**	1.09 (0.98, 1.21)	4.21E-01	1.08 (0.97, 1.19)	2.83E-01
**1.88E-03**	1.12 (1.01, 1.26)	3.14E-01	0.88 (0.79, 0.98)	**4.27E-02**

				UKB
Other mortality				
Urban		Rural		White
Adjusted HR (95% CI)	P value	Adjusted HR (95% CI)	P value	Adjusted HR (95% CI)
0.92 (0.70, 1.21)	9.15E-01	1.04 (0.89, 1.23)	7.72E-01	0.98 (0.97, 1.00)
0.92 (0.70, 1.21)	9.15E-01	1.04 (0.89, 1.23)	7.54E-01	0.98 (0.97, 1.00)
0.94 (0.71, 1.23)	9.62E-01	1.05 (0.89, 1.23)	7.37E-01	0.99 (0.97, 1.00)
0.95 (0.73, 1.23)	9.66E-01	1.08 (0.92, 1.27)	6.18E-01	0.98 (0.96, 0.99)
0.95 (0.74, 1.23)	9.66E-01	1.08 (0.92, 1.27)	6.18E-01	0.97 (0.96, 0.99)
0.95 (0.72, 1.25)	9.66E-01	1.07 (0.92, 1.26)	6.54E-01	0.98 (0.97, 1.00)
0.92 (0.69, 1.21)	9.15E-01	1.04 (0.88, 1.22)	7.84E-01	0.98 (0.97, 1.00)
0.88 (0.66, 1.17)	8.58E-01	1.06 (0.91, 1.25)	7.05E-01	0.95 (0.94, 0.96)
0.88 (0.67, 1.17)	8.58E-01	1.07 (0.91, 1.25)	7.02E-01	0.95 (0.93, 0.96)
0.92 (0.70, 1.21)	9.15E-01	1.07 (0.91, 1.25)	6.83E-01	0.95 (0.94, 0.96)
0.89 (0.67, 1.17)	8.58E-01	1.07 (0.91, 1.26)	6.57E-01	0.93 (0.92, 0.95)
0.88 (0.67, 1.16)	8.58E-01	1.07 (0.91, 1.25)	6.89E-01	0.92 (0.91, 0.93)
0.91 (0.69, 1.20)	8.85E-01	1.08 (0.92, 1.26)	6.42E-01	0.95 (0.93, 0.96)
0.88 (0.66, 1.17)	8.58E-01	1.06 (0.90, 1.25)	7.12E-01	0.95 (0.94, 0.97)
0.90 (0.69, 1.18)	8.58E-01	1.05 (0.90, 1.24)	7.19E-01	0.93 (0.92, 0.95)
0.90 (0.69, 1.18)	8.58E-01	1.05 (0.90, 1.24)	7.19E-01	0.92 (0.91, 0.94)
0.91 (0.69, 1.19)	8.58E-01	1.06 (0.90, 1.24)	7.13E-01	0.94 (0.92, 0.95)
0.90 (0.69, 1.17)	8.58E-01	1.05 (0.89, 1.23)	7.40E-01	0.92 (0.91, 0.94)
0.88 (0.68, 1.15)	8.58E-01	1.03 (0.88, 1.21)	8.16E-01	0.92 (0.91, 0.93)
0.92 (0.70, 1.2)	9.15E-01	1.07 (0.91, 1.25)	6.96E-01	0.93 (0.91, 0.94)
0.90 (0.69, 1.18)	8.58E-01	1.05 (0.90, 1.24)	7.19E-01	0.92 (0.91, 0.94)
0.84 (0.65, 1.10)	8.58E-01	1.04 (0.88, 1.22)	7.81E-01	0.90 (0.89, 0.91)
0.85 (0.65, 1.10)	8.58E-01	1.04 (0.88, 1.22)	7.81E-01	0.90 (0.89, 0.91)
0.84 (0.65, 1.10)	8.58E-01	1.04 (0.88, 1.22)	7.81E-01	0.90 (0.89, 0.92)
0.86 (0.67, 1.11)	8.58E-01	1.03 (0.88, 1.21)	8.16E-01	0.90 (0.89, 0.92)
0.86 (0.67, 1.11)	8.58E-01	1.01 (0.85, 1.19)	9.67E-01	0.91 (0.90, 0.92)
0.86 (0.66, 1.12)	8.58E-01	1.06 (0.90, 1.24)	7.19E-01	0.90 (0.89, 0.92)
0.84 (0.64, 1.10)	8.58E-01	1.04 (0.89, 1.22)	7.73E-01	0.91 (0.90, 0.93)
0.84 (0.65, 1.07)	8.58E-01	1.02 (0.86, 1.2)	9.17E-01	0.94 (0.93, 0.96)
0.84 (0.66, 1.08)	8.58E-01	1.01 (0.86, 1.19)	9.52E-01	0.94 (0.92, 0.95)
0.84 (0.65, 1.07)	8.58E-01	1.00 (0.85, 1.18)	9.87E-01	0.92 (0.91, 0.93)
0.88 (0.70, 1.11)	8.58E-01	0.96 (0.81, 1.13)	7.54E-01	0.93 (0.91, 0.94)
0.90 (0.72, 1.13)	8.58E-01	0.93 (0.79, 1.1)	6.57E-01	0.94 (0.92, 0.95)
0.86 (0.68, 1.10)	8.58E-01	1.01 (0.86, 1.19)	9.52E-01	0.91 (0.90, 0.92)
0.84 (0.65, 1.08)	8.58E-01	1.05 (0.89, 1.23)	7.40E-01	0.97 (0.95, 0.98)
0.95 (0.76, 1.19)	9.66E-01	0.99 (0.85, 1.17)	9.67E-01	1.00 (0.98, 1.01)
0.96 (0.77, 1.20)	9.66E-01	0.99 (0.84, 1.16)	9.31E-01	1.00 (0.99, 1.02)
0.96 (0.76, 1.20)	9.66E-01	0.97 (0.82, 1.13)	7.81E-01	1.02 (1.00, 1.03)
1.01 (0.81, 1.26)	9.85E-01	0.96 (0.82, 1.13)	7.81E-01	0.98 (0.97, 0.99)
1.03 (0.83, 1.27)	9.84E-01	0.96 (0.82, 1.13)	7.72E-01	0.97 (0.96, 0.99)

0.98 (0.78, 1.22)	9.84E-01	0.97 (0.83, 1.14)	8.39E-01	1.00 (0.98, 1.01)
0.88 (0.69, 1.13)	8.58E-01	1.06 (0.91, 1.25)	7.06E-01	1.03 (1.02, 1.05)
0.99 (0.79, 1.24)	9.85E-01	0.95 (0.81, 1.12)	7.40E-01	0.93 (0.92, 0.94)
0.99 (0.79, 1.24)	9.88E-01	0.94 (0.80, 1.11)	7.13E-01	0.93 (0.91, 0.94)
0.99 (0.78, 1.24)	9.85E-01	0.91 (0.77, 1.08)	5.60E-01	0.94 (0.93, 0.96)
0.99 (0.79, 1.24)	9.89E-01	0.91 (0.77, 1.07)	5.44E-01	0.92 (0.90, 0.93)
0.98 (0.79, 1.23)	9.84E-01	0.90 (0.76, 1.06)	5.21E-01	0.91 (0.90, 0.92)
1.02 (0.81, 1.28)	9.84E-01	0.93 (0.79, 1.09)	6.32E-01	0.93 (0.92, 0.94)
1.01 (0.81, 1.27)	9.85E-01	1.15 (0.99, 1.33)	3.03E-01	1.05 (1.03, 1.06)
0.96 (0.76, 1.20)	9.66E-01	0.91 (0.76, 1.07)	5.44E-01	0.91 (0.89, 0.92)
0.96 (0.76, 1.20)	9.66E-01	0.90 (0.76, 1.06)	5.07E-01	0.89 (0.88, 0.90)
0.95 (0.75, 1.19)	9.62E-01	0.87 (0.73, 1.03)	3.53E-01	0.89 (0.87, 0.90)
0.95 (0.76, 1.20)	9.66E-01	0.88 (0.75, 1.05)	4.13E-01	0.88 (0.87, 0.89)
0.94 (0.75, 1.18)	9.15E-01	0.88 (0.75, 1.04)	4.10E-01	0.88 (0.87, 0.89)
0.99 (0.79, 1.25)	9.89E-01	0.89 (0.75, 1.05)	4.39E-01	0.89 (0.88, 0.90)
1.06 (0.85, 1.32)	9.15E-01	1.15 (0.99, 1.33)	3.06E-01	1.04 (1.02, 1.05)
0.89 (0.71, 1.13)	8.58E-01	0.88 (0.74, 1.04)	3.70E-01	0.89 (0.88, 0.91)
0.89 (0.71, 1.13)	8.58E-01	0.87 (0.74, 1.03)	3.53E-01	0.88 (0.86, 0.89)
0.85 (0.67, 1.08)	8.58E-01	0.87 (0.74, 1.02)	3.19E-01	0.87 (0.86, 0.89)
0.90 (0.71, 1.14)	8.58E-01	0.86 (0.73, 1.02)	3.14E-01	0.87 (0.86, 0.88)
0.91 (0.72, 1.14)	8.58E-01	0.86 (0.73, 1.02)	3.36E-01	0.87 (0.86, 0.89)
0.89 (0.70, 1.13)	8.58E-01	0.85 (0.72, 1.00)	2.47E-01	0.87 (0.86, 0.88)
1.01 (0.80, 1.26)	9.89E-01	1.12 (0.96, 1.32)	4.04E-01	1.01 (0.99, 1.02)
0.88 (0.70, 1.12)	8.58E-01	0.86 (0.73, 1.01)	3.03E-01	0.91 (0.89, 0.92)
0.88 (0.70, 1.12)	8.58E-01	0.85 (0.72, 1.01)	2.96E-01	0.89 (0.88, 0.90)
0.84 (0.66, 1.06)	8.58E-01	0.85 (0.73, 0.99)	2.19E-01	0.90 (0.89, 0.92)
0.91 (0.72, 1.14)	8.58E-01	0.85 (0.72, 1.00)	2.79E-01	0.88 (0.87, 0.89)
0.91 (0.72, 1.15)	8.58E-01	0.85 (0.72, 1.01)	2.96E-01	0.88 (0.87, 0.90)
0.88 (0.70, 1.12)	8.58E-01	0.84 (0.71, 0.98)	1.65E-01	0.88 (0.87, 0.89)
0.90 (0.71, 1.15)	8.58E-01	1.07 (0.91, 1.26)	6.83E-01	0.99 (0.98, 1.00)
1.25 (1.03, 1.53)	4.43E-01	1.09 (0.93, 1.27)	6.03E-01	1.09 (1.07, 1.11)
1.25 (1.02, 1.52)	4.43E-01	1.08 (0.92, 1.27)	6.18E-01	1.10 (1.09, 1.12)
1.28 (1.05, 1.56)	3.29E-01	1.09 (0.93, 1.28)	6.02E-01	1.11 (1.10, 1.13)
1.20 (0.97, 1.47)	8.58E-01	1.05 (0.90, 1.23)	7.31E-01	1.09 (1.07, 1.10)
1.19 (0.96, 1.46)	8.58E-01	1.05 (0.89, 1.23)	7.40E-01	1.08 (1.06, 1.09)
1.22 (1.00, 1.49)	6.90E-01	1.06 (0.90, 1.24)	7.13E-01	1.11 (1.09, 1.13)
1.17 (0.95, 1.45)	8.58E-01	1.21 (1.04, 1.40)	1.46E-01	1.05 (1.03, 1.06)
1.31 (1.06, 1.61)	3.29E-01	1.10 (0.93, 1.29)	5.50E-01	1.06 (1.05, 1.08)
1.30 (1.06, 1.61)	3.29E-01	1.09 (0.93, 1.28)	5.60E-01	1.07 (1.05, 1.09)
1.28 (1.03, 1.59)	4.43E-01	1.07 (0.91, 1.26)	6.65E-01	1.07 (1.06, 1.09)
1.30 (1.06, 1.60)	3.29E-01	1.10 (0.94, 1.29)	5.44E-01	1.06 (1.04, 1.08)
1.31 (1.06, 1.61)	3.29E-01	1.10 (0.94, 1.29)	5.33E-01	1.06 (1.04, 1.07)
1.29 (1.05, 1.59)	3.33E-01	1.09 (0.92, 1.27)	6.06E-01	1.08 (1.06, 1.09)
1.34 (1.11, 1.63)	3.29E-01	1.21 (1.03, 1.41)	1.53E-01	1.07 (1.05, 1.08)
0.99 (0.77, 1.26)	9.85E-01	0.85 (0.72, 1.01)	3.03E-01	0.97 (0.96, 0.99)
0.99 (0.78, 1.26)	9.88E-01	0.85 (0.72, 1.01)	3.03E-01	0.98 (0.96, 0.99)
1.04 (0.82, 1.33)	9.66E-01	0.89 (0.75, 1.06)	4.54E-01	0.98 (0.97, 1.00)
0.97 (0.76, 1.24)	9.84E-01	0.83 (0.70, 0.98)	1.95E-01	0.97 (0.95, 0.98)

0.95 (0.75, 1.22)	9.66E-01	0.82 (0.70, 0.97)	1.64E-01	0.97 (0.95, 0.98)
1.02 (0.80, 1.30)	9.84E-01	0.87 (0.73, 1.04)	3.57E-01	0.98 (0.96, 1.00)
0.90 (0.71, 1.15)	8.58E-01	0.97 (0.82, 1.15)	8.42E-01	1.01 (1.00, 1.03)
0.84 (0.68, 1.03)	8.58E-01	0.77 (0.65, 0.91)	**4.21E-02**	0.86 (0.84, 0.87)
0.84 (0.68, 1.03)	8.58E-01	0.76 (0.65, 0.90)	**3.28E-02**	0.89 (0.88, 0.90)
0.87 (0.68, 1.11)	8.58E-01	0.85 (0.72, 1.01)	2.96E-01	0.91 (0.90, 0.92)
0.87 (0.69, 1.11)	8.58E-01	0.79 (0.67, 0.92)	**4.93E-02**	0.86 (0.85, 0.87)
0.90 (0.71, 1.14)	8.58E-01	0.82 (0.71, 0.95)	1.15E-01	0.85 (0.84, 0.86)
0.87 (0.69, 1.11)	8.58E-01	0.83 (0.70, 0.98)	1.65E-01	0.91 (0.89, 0.92)
0.91 (0.71, 1.16)	8.58E-01	1.04 (0.88, 1.22)	7.81E-01	0.99 (0.97, 1.00)
1.09 (0.83, 1.43)	9.15E-01	1.06 (0.87, 1.28)	7.49E-01	1.04 (1.03, 1.06)
1.13 (0.87, 1.47)	8.58E-01	1.13 (0.98, 1.31)	3.53E-01	1.01 (1.00, 1.03)
1.07 (0.83, 1.38)	9.15E-01	1.10 (0.94, 1.29)	5.25E-01	1.01 (0.99, 1.02)
1.15 (0.88, 1.51)	8.58E-01	1.13 (0.97, 1.32)	3.53E-01	1.03 (1.01, 1.04)
0.87 (0.66, 1.14)	8.58E-01	0.87 (0.75, 1.02)	3.40E-01	0.98 (0.96, 0.99)
0.94 (0.75, 1.18)	9.15E-01	1.11 (0.91, 1.35)	6.03E-01	1.02 (1.00, 1.03)
1.03 (0.85, 1.25)	9.81E-01	1.09 (0.94, 1.26)	5.50E-01	1.00 (0.99, 1.02)
1.02 (0.83, 1.24)	9.84E-01	1.02 (0.86, 1.22)	8.80E-01	1.06 (1.04, 1.07)
1.05 (0.81, 1.35)	9.66E-01	1.12 (0.96, 1.31)	3.90E-01	0.98 (0.96, 0.99)
1.00 (0.80, 1.27)	9.89E-01	0.88 (0.74, 1.05)	4.27E-01	1.02 (1.00, 1.04)
1.04 (0.80, 1.36)	9.83E-01	1.10 (0.91, 1.34)	6.03E-01	1.05 (1.03, 1.06)
1.02 (0.78, 1.34)	9.84E-01	1.00 (0.84, 1.20)	9.69E-01	1.05 (1.03, 1.06)
0.97 (0.76, 1.24)	9.84E-01	0.95 (0.80, 1.13)	7.35E-01	1.04 (1.03, 1.06)
1.05 (0.81, 1.36)	9.66E-01	1.06 (0.89, 1.28)	7.19E-01	0.95 (0.94, 0.97)
0.97 (0.74, 1.28)	9.84E-01	0.97 (0.81, 1.16)	8.42E-01	0.99 (0.98, 1.01)
0.95 (0.74, 1.20)	9.66E-01	0.94 (0.80, 1.11)	7.13E-01	0.99 (0.97, 1.00)
1.08 (0.85, 1.36)	9.15E-01	1.01 (0.85, 1.20)	9.52E-01	0.99 (0.97, 1.00)
1.08 (0.86, 1.36)	8.85E-01	0.96 (0.81, 1.14)	7.81E-01	0.99 (0.98, 1.00)
0.98 (0.77, 1.25)	9.84E-01	1.16 (0.97, 1.39)	3.56E-01	1.01 (1.00, 1.03)
0.94 (0.74, 1.19)	9.15E-01	1.00 (0.84, 1.19)	9.87E-01	0.93 (0.91, 0.94)
1.09 (0.88, 1.36)	8.58E-01	0.95 (0.80, 1.12)	7.31E-01	0.96 (0.95, 0.98)
1.08 (0.86, 1.36)	8.74E-01	0.91 (0.78, 1.06)	5.33E-01	0.99 (0.98, 1.01)
1.06 (0.85, 1.34)	9.15E-01	0.90 (0.77, 1.06)	5.06E-01	0.93 (0.91, 0.94)
1.22 (0.97, 1.53)	8.58E-01	0.98 (0.83, 1.16)	9.17E-01	1.05 (1.04, 1.07)
0.90 (0.72, 1.14)	8.58E-01	1.11 (0.95, 1.30)	4.75E-01	1.15 (1.14, 1.17)
0.96 (0.75, 1.22)	9.66E-01	0.95 (0.81, 1.10)	7.13E-01	0.94 (0.92, 0.95)
1.19 (0.93, 1.52)	8.58E-01	0.94 (0.81, 1.10)	7.12E-01	0.93 (0.92, 0.95)
1.21 (0.96, 1.54)	8.58E-01	0.94 (0.80, 1.10)	7.12E-01	0.97 (0.96, 0.99)
1.03 (0.80, 1.33)	9.84E-01	0.98 (0.84, 1.14)	8.39E-01	1.04 (1.03, 1.06)
0.88 (0.68, 1.14)	8.58E-01	1.07 (0.92, 1.24)	6.40E-01	1.12 (1.11, 1.14)
0.92 (0.73, 1.16)	8.58E-01	0.94 (0.8, 1.11)	7.13E-01	0.89 (0.88, 0.90)
1.02 (0.81, 1.28)	9.84E-01	0.87 (0.77, 0.98)	1.53E-01	0.87 (0.86, 0.88)
0.96 (0.77, 1.20)	9.66E-01	0.84 (0.74, 0.96)	1.08E-01	0.98 (0.96, 0.99)
1.09 (0.83, 1.43)	9.15E-01	0.96 (0.82, 1.11)	7.40E-01	1.11 (1.09, 1.12)
1.01 (0.80, 1.27)	9.85E-01	1.18 (1.04, 1.34)	1.15E-01	1.02 (1.01, 1.03)
1.00 (0.79, 1.27)	9.89E-01	1.08 (0.93, 1.26)	6.02E-01	0.92 (0.92, 0.93)
0.94 (0.77, 1.16)	9.15E-01	0.87 (0.77, 0.98)	1.53E-01	0.92 (0.92, 0.93)
0.90 (0.75, 1.09)	8.58E-01	0.86 (0.76, 0.97)	1.28E-01	0.95 (0.94, 0.96)

1.19 (0.91, 1.56)	8.58E-01	1.00 (0.84, 1.18)	9.84E-01	1.11 (1.10, 1.11)
1.09 (0.90, 1.33)	8.58E-01	1.19 (1.06, 1.35)	5.37E-02	1.02 (1.00, 1.03)
0.98 (0.77, 1.26)	9.84E-01	1.12 (0.97, 1.3)	3.63E-01	0.92 (0.91, 0.93)
0.98 (0.77, 1.23)	9.84E-01	0.85 (0.74, 0.98)	1.65E-01	0.91 (0.90, 0.92)
0.96 (0.76, 1.2)	9.66E-01	0.86 (0.74, 0.99)	2.19E-01	0.96 (0.95, 0.97)
1.10 (0.87, 1.39)	8.58E-01	1.11 (0.95, 1.30)	4.82E-01	1.10 (1.09, 1.11)
1.11 (0.89, 1.38)	8.58E-01	1.24 (1.09, 1.42)	**3.28E-02**	1.11 (1.10, 1.12)
1.00 (0.79, 1.28)	9.89E-01	1.13 (0.97, 1.31)	3.56E-01	0.89 (0.88, 0.90)
1.00 (0.79, 1.28)	9.90E-01	0.86 (0.74, 0.99)	2.19E-01	0.91 (0.89, 0.92)
0.98 (0.78, 1.25)	9.84E-01	0.86 (0.74, 1.00)	2.79E-01	0.94 (0.93, 0.95)
1.10 (0.87, 1.38)	8.58E-01	1.08 (0.92, 1.27)	6.32E-01	1.08 (1.06, 1.09)
0.99 (0.77, 1.26)	9.85E-01	1.22 (1.06, 1.4)	8.46E-02	1.11 (1.09, 1.13)
1.14 (0.90, 1.44)	8.58E-01	1.06 (0.9, 1.25)	7.13E-01	0.89 (0.88, 0.90)
0.87 (0.70, 1.08)	8.58E-01	0.89 (0.76, 1.05)	4.47E-01	0.92 (0.91, 0.93)
0.85 (0.68, 1.07)	8.58E-01	0.90 (0.76, 1.07)	5.33E-01	0.95 (0.94, 0.96)
0.96 (0.79, 1.16)	9.66E-01	0.89 (0.77, 1.03)	3.53E-01	0.98 (0.97, 0.99)
1.02 (0.82, 1.27)	9.84E-01	1.11 (0.97, 1.28)	3.90E-01	1.00 (0.99, 1.02)
0.79 (0.62, 1.02)	8.40E-01	0.76 (0.64, 0.90)	**3.82E-02**	1.00 (0.98, 1.01)
1.10 (0.87, 1.39)	8.58E-01	1.15 (0.97, 1.37)	3.56E-01	0.98 (0.97, 1.00)
1.09 (0.87, 1.38)	8.58E-01	1.22 (1.03, 1.44)	1.65E-01	1.05 (1.03, 1.06)
1.09 (0.86, 1.38)	8.58E-01	1.04 (0.88, 1.22)	7.81E-01	1.00 (0.99, 1.02)
1.13 (0.92, 1.39)	8.58E-01	1.17 (1.01, 1.35)	2.21E-01	1.06 (1.05, 1.08)
1.24 (1.07, 1.45)	3.29E-01	1.22 (1.08, 1.37)	**3.28E-02**	0.95 (0.94, 0.96)
0.89 (0.72, 1.11)	8.58E-01	0.84 (0.74, 0.96)	1.28E-01	0.95 (0.94, 0.96)
0.87 (0.71, 1.07)	8.58E-01	0.84 (0.74, 0.96)	1.15E-01	0.97 (0.96, 0.99)
1.03 (0.82, 1.31)	9.83E-01	0.91 (0.78, 1.07)	5.44E-01	1.04 (1.03, 1.05)
0.96 (0.76, 1.22)	9.66E-01	1.09 (0.93, 1.27)	6.03E-01	1.13 (1.12, 1.15)
1.00 (0.78, 1.29)	9.89E-01	1.07 (0.92, 1.25)	6.57E-01	0.90 (0.89, 0.91)
1.00 (0.78, 1.29)	9.89E-01	0.91 (0.78, 1.06)	5.25E-01	0.89 (0.87, 0.90)
1.00 (0.78, 1.28)	9.90E-01	0.91 (0.78, 1.06)	5.44E-01	1.06 (1.04, 1.07)
1.03 (0.81, 1.31)	9.84E-01	1.04 (0.88, 1.22)	7.81E-01	1.05 (1.04, 1.07)
0.98 (0.77, 1.24)	9.84E-01	1.14 (0.98, 1.34)	3.40E-01	0.91 (0.89, 0.92)
0.87 (0.68, 1.12)	8.58E-01	1.04 (0.88, 1.22)	7.81E-01	0.99 (0.98, 1.01)
1.26 (1.01, 1.56)	5.18E-01	1.26 (1.07, 1.47)	6.76E-02	1.12 (1.10, 1.14)
1.27 (1.03, 1.56)	4.43E-01	1.10 (0.93, 1.29)	5.50E-01	0.90 (0.89, 0.91)
0.98 (0.79, 1.23)	9.84E-01	0.91 (0.77, 1.07)	5.44E-01	0.93 (0.92, 0.94)
0.88 (0.70, 1.12)	8.58E-01	1.02 (0.86, 1.19)	9.17E-01	0.88 (0.86, 0.89)
0.92 (0.73, 1.16)	8.58E-01	0.97 (0.83, 1.14)	8.31E-01	0.92 (0.90, 0.93)
0.94 (0.75, 1.18)	9.15E-01	0.89 (0.76, 1.05)	4.48E-01	0.90 (0.88, 0.91)
1.18 (0.95, 1.47)	8.58E-01	0.95 (0.80, 1.12)	7.19E-01	0.91 (0.89, 0.92)
1.18 (0.95, 1.47)	8.58E-01	0.94 (0.79, 1.11)	7.12E-01	
1.14 (0.91, 1.41)	8.58E-01	1.01 (0.87, 1.19)	9.22E-01	
0.97 (0.77, 1.22)	9.83E-01	0.90 (0.76, 1.06)	5.06E-01	0.96 (0.95, 0.98)
1.03 (0.83, 1.30)	9.83E-01	0.87 (0.73, 1.04)	3.90E-01	0.95 (0.94, 0.96)
0.88 (0.68, 1.14)	8.58E-01	1.08 (0.92, 1.26)	6.18E-01	1.02 (1.01, 1.04)
0.85 (0.65, 1.11)	8.58E-01	1.06 (0.90, 1.24)	7.13E-01	1.02 (1.01, 1.03)
1.03 (0.83, 1.29)	9.83E-01	1.15 (0.99, 1.34)	2.96E-01	0.95 (0.93, 0.96)
1.07 (0.85, 1.35)	9.15E-01	1.14 (0.97, 1.34)	3.53E-01	0.98 (0.97, 1.00)

1.01 (0.81, 1.27)	9.85E-01	0.96 (0.81, 1.13)	7.73E-01	0.94 (0.92, 0.95)
0.84 (0.64, 1.08)	8.58E-01	1.03 (0.88, 1.21)	8.16E-01	0.96 (0.95, 0.98)
0.98 (0.78, 1.22)	9.84E-01	0.92 (0.77, 1.09)	6.03E-01	0.94 (0.92, 0.95)
1.09 (0.87, 1.37)	8.58E-01	0.85 (0.71, 1.01)	2.96E-01	0.97 (0.96, 0.99)
1.10 (0.88, 1.37)	8.58E-01	0.94 (0.79, 1.11)	7.12E-01	0.91 (0.90, 0.92)
1.12 (0.90, 1.40)	8.58E-01	0.95 (0.80, 1.12)	7.19E-01	0.94 (0.93, 0.95)
0.88 (0.70, 1.12)	8.58E-01	0.96 (0.82, 1.14)	7.81E-01	0.95 (0.94, 0.96)
0.87 (0.68, 1.10)	8.58E-01	0.99 (0.84, 1.17)	9.52E-01	0.87 (0.85, 0.88)
0.93 (0.73, 1.18)	9.15E-01	0.93 (0.78, 1.10)	6.57E-01	0.87 (0.86, 0.89)
0.95 (0.75, 1.21)	9.66E-01	0.75 (0.65, 0.86)	**3.39E-03**	0.90 (0.89, 0.91)
0.92 (0.73, 1.15)	8.58E-01	0.87 (0.74, 1.02)	3.20E-01	0.87 (0.86, 0.88)
0.99 (0.78, 1.26)	9.88E-01	0.87 (0.74, 1.03)	3.56E-01	0.90 (0.89, 0.91)
0.85 (0.67, 1.08)	8.58E-01	0.86 (0.72, 1.03)	3.53E-01	0.88 (0.87, 0.89)
0.98 (0.77, 1.24)	9.84E-01	0.85 (0.72, 1.01)	3.03E-01	1.00 (0.98, 1.01)
0.95 (0.75, 1.20)	9.66E-01	0.84 (0.71, 1.01)	2.96E-01	0.98 (0.96, 0.99)
0.89 (0.70, 1.14)	8.58E-01	0.99 (0.84, 1.17)	9.42E-01	0.90 (0.89, 0.92)
0.95 (0.75, 1.21)	9.66E-01	0.95 (0.80, 1.13)	7.40E-01	0.92 (0.91, 0.93)
0.98 (0.77, 1.23)	9.84E-01	0.93 (0.79, 1.09)	6.55E-01	0.87 (0.86, 0.89)
1.13 (0.87, 1.48)	8.58E-01	0.85 (0.73, 0.98)	1.65E-01	0.95 (0.94, 0.97)
0.87 (0.69, 1.09)	8.58E-01	0.87 (0.74, 1.03)	3.53E-01	0.91 (0.90, 0.92)
1.12 (0.87, 1.43)	8.58E-01	0.81 (0.69, 0.95)	1.08E-01	1.09 (1.07, 1.10)
1.07 (0.84, 1.37)	9.15E-01	0.77 (0.66, 0.91)	**3.28E-02**	1.08 (1.06, 1.09)
0.86 (0.68, 1.09)	8.58E-01	1.21 (1.03, 1.41)	1.53E-01	1.09 (1.08, 1.10)
1.12 (0.89, 1.40)	8.58E-01	1.18 (1.00, 1.41)	2.81E-01	1.04 (1.03, 1.06)
1.23 (1.06, 1.42)	3.29E-01	1.26 (1.14, 1.39)	**1.01E-03**	1.07 (1.06, 1.08)
0.90 (0.70, 1.16)	8.58E-01	0.93 (0.78, 1.11)	7.05E-01	0.99 (0.98, 1.01)
1.13 (0.88, 1.44)	8.58E-01	1.03 (0.87, 1.24)	8.13E-01	0.95 (0.94, 0.97)
0.84 (0.66, 1.07)	8.58E-01	1.03 (0.87, 1.22)	8.26E-01	0.92 (0.90, 0.93)
0.87 (0.69, 1.09)	8.58E-01	0.89 (0.75, 1.06)	4.82E-01	0.97 (0.96, 0.99)
0.87 (0.68, 1.11)	8.58E-01	0.99 (0.83, 1.17)	9.52E-01	0.93 (0.91, 0.94)
0.85 (0.66, 1.10)	8.58E-01	0.98 (0.83, 1.16)	8.80E-01	0.93 (0.91, 0.94)
0.79 (0.60, 1.03)	8.58E-01	0.99 (0.84, 1.18)	9.67E-01	1.04 (1.03, 1.05)
0.86 (0.67, 1.11)	8.58E-01	0.95 (0.80, 1.13)	7.54E-01	1.03 (1.01, 1.04)
1.03 (0.83, 1.29)	9.83E-01	1.16 (1.06, 1.26)	**3.28E-02**	1.07 (1.06, 1.07)
0.98 (0.77, 1.23)	9.84E-01	1.29 (1.10, 1.50)	**3.28E-02**	1.06 (1.05, 1.07)
0.69 (0.30, 1.59)	8.58E-01	1.01 (0.87, 1.17)	9.42E-01	1.08 (1.06, 1.09)
0.98 (0.73, 1.31)	9.84E-01	1.07 (0.93, 1.23)	6.03E-01	1.04 (1.04, 1.05)
1.18 (1.04, 1.35)	3.29E-01	1.06 (0.91, 1.22)	7.12E-01	0.86 (0.84, 0.87)
1.11 (1.04, 1.19)	3.29E-01	1.24 (1.15, 1.35)	**5.33E-05**	1.13 (1.11, 1.14)
0.98 (0.78, 1.22)	9.84E-01	0.77 (0.67, 0.88)	**1.18E-02**	1.00 (0.98, 1.01)
0.93 (0.71, 1.20)	9.15E-01	1.05 (0.89, 1.24)	7.40E-01	0.93 (0.92, 0.95)
				0.89 (0.88, 0.91)
				0.88 (0.87, 0.90)
				0.88 (0.86, 0.89)
				0.98 (0.97, 1.00)
				0.91 (0.90, 0.92)
				1.09 (1.08, 1.10)
				0.93 (0.92, 0.94)

0.88 (0.87, 0.89)
1.01 (1.00, 1.03)
1.04 (1.03, 1.05)
1.07 (1.06, 1.08)
0.91 (0.89, 0.92)
0.94 (0.93, 0.96)
0.90 (0.89, 0.91)
0.92 (0.91, 0.93)
0.93 (0.92, 0.95)
0.94 (0.93, 0.95)
0.88 (0.87, 0.90)
1.01 (0.99, 1.02)
0.91 (0.90, 0.93)
0.94 (0.93, 0.95)
0.88 (0.87, 0.90)
1.00 (0.98, 1.02)
0.93 (0.92, 0.94)
0.99 (0.97, 1.01)
1.02 (1.00, 1.04)

All causes mortality			Ischaemic heart	
	Non-white		White	
**P value**	**Adjusted HR (95% CI)**	**P value**	**Adjusted HR (95% CI)**	**P value**
**2.67E-02**	0.95 (0.87, 1.03)	2.60E-01	1.04 (1.00, 1.08)	1.00E-01
**1.64E-02**	0.94 (0.87, 1.02)	2.42E-01	1.03 (0.99, 1.07)	1.37E-01
5.36E-02	0.94 (0.86, 1.02)	2.26E-01	1.04 (1.00, 1.08)	8.78E-02
**2.40E-03**	0.93 (0.86, 1.01)	1.65E-01	1.02 (0.98, 1.07)	3.27E-01
**4.57E-04**	0.93 (0.86, 1.01)	1.62E-01	1.02 (0.97, 1.06)	4.94E-01
**1.48E-02**	0.93 (0.86, 1.02)	1.78E-01	1.03 (0.99, 1.07)	1.83E-01
**2.26E-02**	0.95 (0.87, 1.03)	2.86E-01	1.03 (0.99, 1.07)	1.17E-01
**4.01E-12**	0.93 (0.85, 1.01)	1.47E-01	1.01 (0.97, 1.06)	5.35E-01
**5.48E-13**	0.93 (0.85, 1.01)	1.27E-01	1.01 (0.97, 1.05)	7.06E-01
**1.03E-11**	0.92 (0.85, 1.00)	1.06E-01	1.01 (0.97, 1.05)	7.17E-01
**8.64E-21**	0.90 (0.82, 0.98)	**3.36E-02**	0.98 (0.93, 1.02)	3.27E-01
**2.31E-28**	0.88 (0.81, 0.96)	**1.49E-02**	0.95 (0.91, 1.00)	**4.09E-02**
**2.73E-13**	0.91 (0.84, 1.00)	7.76E-02	1.00 (0.96, 1.04)	9.76E-01
**1.31E-10**	0.94 (0.86, 1.02)	2.16E-01	1.02 (0.98, 1.06)	4.09E-01
**2.34E-19**	0.92 (0.84, 1.00)	9.08E-02	1.00 (0.95, 1.04)	8.68E-01
**5.58E-25**	0.91 (0.83, 0.99)	5.61E-02	0.98 (0.94, 1.03)	4.96E-01
**2.51E-17**	0.92 (0.84, 1.00)	9.08E-02	1.00 (0.96, 1.05)	9.38E-01
**7.08E-26**	0.90 (0.82, 0.98)	**3.52E-02**	0.97 (0.93, 1.02)	2.32E-01
**4.77E-28**	0.89 (0.82, 0.97)	**2.52E-02**	0.95 (0.91, 0.99)	**3.64E-02**
**1.17E-22**	0.91 (0.83, 0.99)	5.19E-02	0.99 (0.95, 1.04)	7.18E-01
**1.78E-26**	0.91 (0.84, 0.99)	6.77E-02	0.98 (0.94, 1.03)	4.68E-01
**4.53E-44**	0.87 (0.80, 0.94)	**3.19E-03**	0.91 (0.87, 0.95)	**5.81E-05**
**1.86E-45**	0.87 (0.80, 0.95)	**4.25E-03**	0.92 (0.88, 0.96)	**4.50E-04**
**7.07E-42**	0.87 (0.80, 0.94)	**3.31E-03**	0.91 (0.87, 0.95)	**4.13E-05**
**7.07E-42**	0.86 (0.79, 0.93)	**1.44E-03**	0.87 (0.83, 0.91)	**7.57E-09**
**3.04E-37**	0.86 (0.79, 0.93)	**1.35E-03**	0.86 (0.82, 0.90)	**2.53E-10**
**2.07E-42**	0.87 (0.80, 0.94)	**3.48E-03**	0.90 (0.86, 0.94)	**3.89E-06**
**2.49E-32**	0.90 (0.83, 0.98)	**3.91E-02**	0.97 (0.93, 1.01)	2.06E-01
**1.90E-15**	0.93 (0.86, 1.01)	1.55E-01	1.00 (0.95, 1.04)	8.45E-01
**1.22E-18**	0.92 (0.85, 1.00)	9.69E-02	0.98 (0.94, 1.03)	4.68E-01
**1.03E-29**	0.89 (0.82, 0.97)	**2.33E-02**	0.94 (0.90, 0.98)	**1.21E-02**
**5.67E-24**	0.90 (0.83, 0.98)	**3.76E-02**	0.94 (0.90, 0.99)	**1.29E-02**
**1.54E-17**	0.92 (0.84, 1.00)	7.76E-02	0.96 (0.92, 1.01)	1.08E-01
**1.46E-36**	0.88 (0.81, 0.96)	**8.97E-03**	0.91 (0.87, 0.95)	**7.70E-05**
**1.85E-06**	0.97 (0.89, 1.05)	4.85E-01	1.04 (0.99, 1.08)	1.15E-01
6.32E-01	0.98 (0.90, 1.06)	6.73E-01	0.98 (0.94, 1.03)	4.85E-01
7.06E-01	0.99 (0.91, 1.07)	7.66E-01	0.99 (0.95, 1.03)	6.64E-01
**1.04E-02**	1.00 (0.93, 1.09)	9.33E-01	1.02 (0.98, 1.07)	4.43E-01
**8.19E-03**	0.95 (0.88, 1.03)	2.89E-01	0.92 (0.88, 0.96)	**7.43E-04**
**2.75E-04**	0.94 (0.86, 1.01)	1.66E-01	0.90 (0.86, 0.94)	**1.65E-05**

8.03E-01	0.98 (0.91, 1.07)	7.58E-01	0.97 (0.93, 1.02)	3.09E-01
**1.40E-05**	1.04 (0.97, 1.13)	3.70E-01	1.10 (1.06, 1.15)	**6.30E-06**
**1.14E-23**	0.88 (0.81, 0.96)	**7.54E-03**	0.88 (0.84, 0.92)	**1.35E-07**
**2.85E-23**	0.89 (0.82, 0.96)	**1.30E-02**	0.87 (0.83, 0.91)	**2.19E-09**
**1.15E-15**	0.90 (0.83, 0.98)	**2.70E-02**	0.88 (0.84, 0.92)	**1.45E-07**
**4.86E-32**	0.87 (0.80, 0.95)	**4.25E-03**	0.84 (0.81, 0.88)	**2.33E-12**
**1.83E-35**	0.86 (0.79, 0.94)	**1.99E-03**	0.84 (0.80, 0.88)	**2.50E-13**
**3.42E-22**	0.90 (0.83, 0.98)	**3.39E-02**	0.87 (0.83, 0.91)	**2.13E-09**
**1.69E-11**	1.05 (0.98, 1.14)	2.59E-01	1.10 (1.06, 1.15)	**3.89E-06**
**2.94E-40**	0.86 (0.79, 0.94)	**1.94E-03**	0.90 (0.86, 0.94)	**5.40E-06**
**5.47E-57**	0.85 (0.79, 0.93)	**9.00E-04**	0.86 (0.82, 0.90)	**4.17E-10**
**5.27E-59**	0.85 (0.78, 0.92)	**6.24E-04**	0.86 (0.82, 0.90)	**5.17E-11**
**1.96E-64**	0.85 (0.78, 0.92)	**4.96E-04**	0.85 (0.81, 0.89)	**8.74E-12**
**3.03E-66**	0.84 (0.78, 0.92)	**4.65E-04**	0.85 (0.82, 0.89)	**4.28E-11**
**1.94E-56**	0.85 (0.79, 0.93)	**8.36E-04**	0.84 (0.80, 0.88)	**2.83E-13**
**4.97E-07**	1.04 (0.96, 1.13)	3.94E-01	1.10 (1.06, 1.14)	**5.40E-06**
**1.92E-51**	0.86 (0.80, 0.94)	**2.68E-03**	0.89 (0.85, 0.93)	**6.10E-07**
**9.20E-72**	0.84 (0.77, 0.91)	**3.89E-04**	0.89 (0.85, 0.93)	**1.63E-07**
**5.69E-75**	0.84 (0.77, 0.91)	**2.90E-04**	0.89 (0.85, 0.93)	**2.53E-07**
**1.27E-78**	0.84 (0.77, 0.91)	**2.80E-04**	0.87 (0.83, 0.91)	**4.37E-09**
**9.64E-74**	0.84 (0.77, 0.91)	**3.10E-04**	0.88 (0.84, 0.92)	**6.78E-08**
**2.90E-81**	0.84 (0.77, 0.91)	**2.86E-04**	0.85 (0.82, 0.89)	**1.69E-11**
2.46E-01	1.01 (0.93, 1.09)	8.98E-01	1.08 (1.04, 1.12)	**1.65E-04**
**9.02E-40**	0.88 (0.81, 0.95)	**5.68E-03**	0.91 (0.87, 0.95)	**2.83E-05**
**2.77E-57**	0.85 (0.78, 0.92)	**5.05E-04**	0.90 (0.86, 0.94)	**5.40E-06**
**8.72E-44**	0.86 (0.79, 0.93)	**1.51E-03**	0.92 (0.88, 0.96)	**2.00E-04**
**9.33E-69**	0.84 (0.77, 0.91)	**2.80E-04**	0.88 (0.84, 0.92)	**3.74E-08**
**8.15E-63**	0.84 (0.77, 0.91)	**2.90E-04**	0.89 (0.85, 0.93)	**4.44E-07**
**3.09E-70**	0.85 (0.79, 0.92)	**5.90E-04**	0.87 (0.83, 0.91)	**1.21E-09**
1.92E-01	0.97 (0.89, 1.05)	5.54E-01	1.05 (1.01, 1.10)	**1.10E-02**
**1.31E-27**	1.06 (0.97, 1.15)	2.62E-01	0.95 (0.90, 1.00)	6.86E-02
**4.38E-36**	1.07 (0.99, 1.17)	1.61E-01	0.96 (0.91, 1.01)	1.63E-01
**1.23E-43**	1.09 (1.00, 1.18)	9.69E-02	0.97 (0.92, 1.03)	3.23E-01
**6.42E-25**	1.05 (0.97, 1.15)	3.03E-01	0.94 (0.89, 0.99)	**3.38E-02**
**4.44E-20**	1.04 (0.96, 1.14)	4.02E-01	0.92 (0.87, 0.98)	**8.98E-03**
**4.58E-44**	1.09 (1.00, 1.18)	9.14E-02	0.99 (0.94, 1.04)	7.23E-01
**2.54E-11**	1.03 (0.95, 1.11)	5.19E-01	1.05 (1.01, 1.09)	**3.64E-02**
**1.73E-13**	1.03 (0.95, 1.13)	5.39E-01	0.89 (0.84, 0.95)	**2.19E-04**
**5.82E-16**	1.04 (0.96, 1.14)	4.15E-01	0.90 (0.85, 0.96)	**1.00E-03**
**3.61E-17**	1.05 (0.96, 1.15)	3.59E-01	0.92 (0.87, 0.97)	**3.73E-03**
**9.69E-13**	1.03 (0.94, 1.13)	5.54E-01	0.89 (0.84, 0.94)	**1.68E-04**
**3.69E-11**	1.03 (0.94, 1.13)	5.98E-01	0.88 (0.83, 0.94)	**7.35E-05**
**7.01E-19**	1.05 (0.96, 1.14)	3.93E-01	0.91 (0.86, 0.97)	**2.76E-03**
**1.43E-20**	1.06 (0.98, 1.15)	1.99E-01	1.02 (0.97, 1.07)	4.49E-01
**4.43E-04**	0.94 (0.86, 1.03)	2.87E-01	0.87 (0.83, 0.92)	**2.45E-07**
**2.43E-03**	0.95 (0.87, 1.04)	3.36E-01	0.89 (0.85, 0.94)	**7.31E-06**
**1.71E-02**	0.96 (0.88, 1.04)	3.93E-01	0.91 (0.87, 0.95)	**1.14E-04**
**6.63E-05**	0.94 (0.86, 1.03)	2.59E-01	0.87 (0.82, 0.91)	**7.33E-08**

**1.38E-05**	0.94 (0.86, 1.03)	2.34E-01	0.87 (0.82, 0.91)	**5.16E-08**
**1.48E-02**	0.96 (0.87, 1.05)	4.04E-01	0.87 (0.83, 0.92)	**7.01E-07**
5.55E-02	1.01 (0.93, 1.09)	8.58E-01	1.03 (0.99, 1.08)	1.76E-01
**6.41E-95**	0.83 (0.77, 0.91)	**2.80E-04**	0.86 (0.82, 0.90)	**8.20E-11**
**3.34E-54**	0.87 (0.80, 0.94)	**3.55E-03**	0.89 (0.86, 0.93)	**1.45E-06**
**6.88E-37**	0.88 (0.81, 0.96)	**1.30E-02**	0.90 (0.86, 0.94)	**1.23E-05**
**2.50E-91**	0.84 (0.77, 0.91)	**3.10E-04**	0.86 (0.82, 0.90)	**4.29E-11**
**4.72E-105**	0.83 (0.76, 0.90)	**1.52E-04**	0.85 (0.81, 0.89)	**2.51E-12**
**6.45E-39**	0.90 (0.82, 0.97)	**2.54E-02**	0.90 (0.86, 0.94)	**7.05E-06**
7.37E-02	0.99 (0.91, 1.08)	8.58E-01	1.07 (1.02, 1.11)	**4.69E-03**
**2.71E-07**	1.02 (0.93, 1.12)	7.14E-01	1.14 (1.08, 1.20)	**8.52E-06**
8.76E-02	1.09 (1.01, 1.18)	7.37E-02	0.95 (0.90, 1.00)	9.43E-02
5.30E-01	1.10 (1.02, 1.19)	**4.31E-02**	0.94 (0.89, 0.99)	**3.72E-02**
**5.49E-04**	1.04 (0.96, 1.12)	4.31E-01	0.99 (0.94, 1.04)	6.64E-01
**2.56E-03**	0.92 (0.85, 1.00)	9.08E-02	1.01 (0.96, 1.06)	7.33E-01
5.56E-02	1.06 (0.97, 1.15)	2.58E-01	0.91 (0.86, 0.96)	**1.42E-03**
6.37E-01	1.05 (0.97, 1.14)	3.39E-01	0.90 (0.85, 0.96)	**8.81E-04**
**1.33E-12**	1.08 (0.99, 1.17)	1.50E-01	0.95 (0.90, 1.00)	6.96E-02
**8.49E-03**	0.94 (0.87, 1.02)	2.34E-01	1.09 (1.04, 1.15)	**1.45E-03**
**2.99E-02**	1.01 (0.92, 1.10)	8.77E-01	1.10 (1.04, 1.17)	**2.76E-03**
**1.98E-10**	1.11 (1.03, 1.21)	**2.34E-02**	1.00 (0.95, 1.04)	8.45E-01
**9.01E-10**	1.10 (1.01, 1.19)	5.19E-02	0.96 (0.92, 1.01)	1.07E-01
**4.14E-07**	1.13 (1.04, 1.24)	**1.43E-02**	1.10 (1.05, 1.16)	**1.76E-04**
**2.56E-09**	0.92 (0.84, 1.00)	9.14E-02	0.97 (0.92, 1.01)	1.90E-01
2.32E-01	0.98 (0.90, 1.05)	5.98E-01	0.90 (0.87, 0.94)	**4.43E-07**
8.21E-02	0.97 (0.90, 1.05)	5.59E-01	0.88 (0.84, 0.92)	**1.04E-08**
6.32E-02	0.97 (0.89, 1.05)	4.96E-01	0.88 (0.84, 0.91)	**3.97E-09**
1.81E-01	0.99 (0.91, 1.07)	7.98E-01	0.90 (0.86, 0.93)	**3.82E-07**
9.93E-02	1.03 (0.95, 1.11)	5.59E-01	1.13 (1.09, 1.18)	**1.57E-08**
**4.19E-24**	0.90 (0.83, 0.98)	**3.82E-02**	0.84 (0.80, 0.88)	**3.53E-14**
**1.64E-07**	0.96 (0.88, 1.03)	3.39E-01	0.89 (0.85, 0.92)	**3.75E-08**
2.46E-01	1.00 (0.92, 1.08)	9.46E-01	0.93 (0.89, 0.97)	**1.30E-03**
**1.47E-23**	0.91 (0.83, 0.98)	**4.31E-02**	0.83 (0.80, 0.87)	**2.29E-15**
**1.03E-11**	1.07 (0.98, 1.15)	1.88E-01	1.15 (1.10, 1.20)	**5.83E-10**
**2.13E-83**	1.15 (1.06, 1.25)	**4.99E-03**	1.30 (1.24, 1.35)	**7.17E-32**
**4.25E-20**	0.90 (0.83, 0.97)	**1.92E-02**	0.82 (0.79, 0.86)	**2.29E-21**
**4.58E-22**	0.89 (0.82, 0.96)	**1.07E-02**	0.81 (0.78, 0.85)	**1.55E-23**
**6.87E-05**	0.98 (0.91, 1.06)	6.49E-01	0.92 (0.89, 0.95)	**1.32E-05**
**1.01E-09**	1.10 (1.01, 1.18)	**4.88E-02**	1.18 (1.13, 1.22)	**8.89E-16**
**3.52E-61**	1.13 (1.05, 1.23)	**6.22E-03**	1.19 (1.14, 1.24)	**2.50E-15**
**2.27E-77**	0.86 (0.81, 0.92)	**2.90E-04**	0.81 (0.78, 0.84)	**1.73E-32**
**1.07E-102**	0.82 (0.77, 0.88)	**1.58E-06**	0.78 (0.75, 0.81)	**4.86E-40**
**8.10E-04**	1.03 (0.95, 1.11)	5.59E-01	0.94 (0.90, 0.97)	**6.77E-04**
**4.92E-60**	1.13 (1.06, 1.21)	**8.69E-04**	1.21 (1.17, 1.25)	**2.57E-29**
**5.59E-03**	1.04 (0.97, 1.12)	2.96E-01	0.99 (0.95, 1.03)	7.37E-01
**4.80E-121**	0.91 (0.87, 0.95)	**9.46E-05**	0.91 (0.90, 0.93)	**1.25E-28**
**2.45E-143**	0.91 (0.88, 0.94)	**1.33E-05**	0.93 (0.91, 0.94)	**1.26E-15**
**2.83E-14**	0.94 (0.88, 1.01)	1.61E-01	0.86 (0.83, 0.89)	**3.69E-17**

**2.00E-120**	1.12 (1.07, 1.18)	**1.52E-04**	1.13 (1.11, 1.15)	**3.73E-35**
**1.48E-02**	1.03 (0.95, 1.11)	5.53E-01	1.06 (1.02, 1.10)	**1.81E-03**
**2.05E-130**	0.92 (0.88, 0.95)	**4.64E-05**	0.91 (0.89, 0.92)	**1.08E-33**
**6.31E-59**	0.89 (0.84, 0.94)	**7.69E-04**	0.90 (0.87, 0.93)	**5.15E-09**
**2.47E-09**	0.96 (0.89, 1.04)	4.17E-01	0.89 (0.86, 0.93)	**1.11E-08**
**6.76E-150**	1.11 (1.06, 1.16)	**4.54E-05**	1.12 (1.10, 1.14)	**5.02E-36**
**1.87E-56**	1.10 (1.02, 1.19)	**4.31E-02**	1.13 (1.09, 1.17)	**1.26E-10**
**3.17E-154**	0.88 (0.82, 0.93)	**2.02E-04**	0.87 (0.86, 0.89)	**7.79E-40**
**2.05E-50**	0.88 (0.81, 0.95)	**4.57E-03**	0.88 (0.85, 0.91)	**1.02E-12**
**2.44E-21**	0.96 (0.90, 1.04)	4.07E-01	0.91 (0.88, 0.94)	**4.64E-07**
**6.61E-35**	1.07 (1.01, 1.13)	**3.76E-02**	1.11 (1.09, 1.14)	**1.71E-14**
**1.70E-39**	1.06 (0.98, 1.16)	2.32E-01	1.01 (0.97, 1.06)	5.36E-01
**4.42E-53**	0.95 (0.87, 1.02)	2.32E-01	0.97 (0.93, 1.01)	1.20E-01
**3.34E-44**	0.93 (0.87, 1.00)	7.37E-02	0.93 (0.90, 0.96)	**2.38E-05**
**4.09E-11**	1.00 (0.93, 1.08)	9.63E-01	1.04 (1.00, 1.08)	**3.76E-02**
**3.33E-03**	0.99 (0.93, 1.05)	7.35E-01	1.01 (0.98, 1.05)	4.46E-01
6.72E-01	1.07 (1.00, 1.15)	1.08E-01	1.10 (1.06, 1.14)	**4.94E-07**
5.70E-01	0.94 (0.88, 1.01)	1.63E-01	0.92 (0.88, 0.95)	**1.31E-06**
**2.40E-02**	0.94 (0.87, 1.01)	1.27E-01	0.91 (0.88, 0.94)	**2.72E-07**
**1.67E-10**	0.98 (0.91, 1.05)	6.79E-01	0.97 (0.93, 1.00)	6.65E-02
5.67E-01	1.02 (0.97, 1.08)	5.57E-01	1.06 (1.03, 1.10)	**3.48E-04**
**7.27E-17**	1.09 (1.00, 1.18)	7.37E-02	1.16 (1.12, 1.21)	**2.51E-12**
**4.37E-12**	0.93 (0.86, 1.00)	1.01E-01	0.87 (0.84, 0.91)	**8.07E-10**
**3.84E-12**	0.93 (0.86, 1.01)	1.40E-01	0.89 (0.86, 0.93)	**1.49E-07**
**6.99E-04**	0.94 (0.86, 1.02)	2.06E-01	0.85 (0.81, 0.89)	**2.60E-11**
**5.39E-08**	1.07 (0.99, 1.16)	1.62E-01	1.10 (1.07, 1.13)	**7.34E-09**
**1.91E-62**	1.10 (1.02, 1.20)	**4.31E-02**	1.04 (1.00, 1.09)	6.95E-02
**1.55E-53**	0.90 (0.83, 0.97)	**2.19E-02**	0.89 (0.85, 0.92)	**2.79E-08**
**2.75E-68**	0.88 (0.82, 0.95)	**4.43E-03**	0.89 (0.85, 0.92)	**1.72E-08**
**3.72E-13**	1.10 (1.02, 1.19)	**4.09E-02**	1.01 (0.96, 1.05)	8.45E-01
**1.71E-12**	1.08 (1.00, 1.17)	1.04E-01	1.16 (1.11, 1.21)	**4.28E-11**
**2.47E-38**	0.88 (0.81, 0.96)	**1.01E-02**	0.98 (0.93, 1.02)	3.29E-01
2.46E-01	0.94 (0.87, 1.02)	2.22E-01	0.97 (0.93, 1.01)	1.75E-01
**6.07E-44**	1.09 (1.00, 1.19)	9.74E-02	0.94 (0.89, 0.99)	**2.63E-02**
**1.47E-45**	0.85 (0.79, 0.93)	**1.01E-03**	0.84 (0.80, 0.88)	**9.40E-13**
**5.85E-23**	0.89 (0.82, 0.97)	**1.77E-02**	0.93 (0.89, 0.98)	**3.73E-03**
**3.09E-70**	0.84 (0.77, 0.91)	**3.10E-04**	0.86 (0.82, 0.90)	**6.56E-11**
**5.63E-32**	0.87 (0.80, 0.95)	**4.25E-03**	0.88 (0.84, 0.92)	**1.30E-07**
**3.58E-47**	0.85 (0.78, 0.93)	**9.09E-04**	0.84 (0.80, 0.88)	**1.43E-13**
**3.66E-40**	0.86 (0.79, 0.93)	**1.66E-03**	0.86 (0.82, 0.90)	**1.55E-10**
**3.26E-07**	0.95 (0.87, 1.03)	3.05E-01	1.02 (0.98, 1.07)	3.02E-01
**2.08E-12**	0.93 (0.86, 1.01)	1.62E-01	1.01 (0.97, 1.05)	6.20E-01
**1.39E-03**	1.02 (0.95, 1.11)	6.36E-01	1.09 (1.05, 1.13)	**3.66E-05**
**6.25E-03**	1.02 (0.94, 1.10)	7.22E-01	1.04 (1.00, 1.09)	6.41E-02
**4.07E-13**	0.92 (0.84, 1.00)	8.57E-02	0.89 (0.85, 0.94)	**3.81E-06**
**2.40E-02**	0.98 (0.90, 1.07)	7.14E-01	1.09 (1.05, 1.14)	**1.05E-04**

**3.54E-16**	0.90 (0.82, 0.98)	**3.03E-02**	0.86 (0.82, 0.91)	**4.76E-09**
**3.53E-07**	0.96 (0.88, 1.04)	4.23E-01	0.90 (0.86, 0.94)	**1.74E-05**
**3.31E-16**	0.90 (0.83, 0.98)	**4.31E-02**	0.87 (0.83, 0.91)	**2.79E-08**
**3.01E-04**	0.94 (0.86, 1.03)	2.49E-01	0.87 (0.82, 0.91)	**6.50E-08**
**5.60E-39**	0.87 (0.80, 0.94)	**3.19E-03**	0.90 (0.86, 0.94)	**4.06E-06**
**6.00E-17**	0.92 (0.85, 1.00)	9.57E-02	0.98 (0.94, 1.03)	4.58E-01
**8.83E-12**	0.93 (0.86, 1.01)	1.40E-01	0.97 (0.93, 1.01)	2.30E-01
**8.89E-82**	0.91 (0.84, 0.99)	5.36E-02	0.83 (0.79, 0.87)	**1.82E-15**
**5.49E-61**	0.96 (0.88, 1.04)	4.07E-01	0.82 (0.78, 0.87)	**8.89E-13**
**1.02E-43**	0.83 (0.76, 0.91)	**3.10E-04**	0.85 (0.81, 0.89)	**1.50E-11**
**2.82E-66**	0.95 (0.88, 1.04)	3.39E-01	0.87 (0.83, 0.91)	**6.02E-08**
**1.74E-45**	0.84 (0.77, 0.92)	**5.90E-04**	0.87 (0.83, 0.91)	**2.03E-09**
**1.34E-63**	0.85 (0.78, 0.92)	**9.09E-04**	0.85 (0.81, 0.89)	**4.28E-11**
5.22E-01	0.98 (0.91, 1.06)	6.96E-01	1.05 (1.01, 1.09)	**2.27E-02**
**2.72E-03**	0.97 (0.90, 1.05)	5.62E-01	1.00 (0.96, 1.04)	9.76E-01
**9.20E-39**	1.00 (0.92, 1.08)	1.00E+00	0.86 (0.82, 0.90)	**3.22E-09**
**6.11E-31**	0.83 (0.76, 0.90)	**7.65E-05**	0.82 (0.78, 0.85)	**1.81E-20**
**7.04E-66**	0.99 (0.91, 1.07)	8.35E-01	0.87 (0.83, 0.92)	**1.30E-07**
**7.82E-12**	0.89 (0.83, 0.96)	**9.00E-03**	0.88 (0.85, 0.92)	**2.11E-09**
**1.40E-41**	0.89 (0.82, 0.96)	**7.53E-03**	0.85 (0.81, 0.88)	**4.70E-15**
**5.73E-29**	1.10 (1.01, 1.19)	**4.90E-02**	1.22 (1.17, 1.27)	**3.48E-19**
**4.85E-27**	1.11 (1.03, 1.20)	**2.08E-02**	1.07 (1.03, 1.11)	**1.49E-03**
**6.21E-70**	1.14 (1.08, 1.20)	**2.71E-05**	1.18 (1.15, 1.20)	**1.06E-48**
**2.03E-08**	1.01 (0.93, 1.10)	7.83E-01	1.07 (1.03, 1.12)	**1.54E-03**
**3.15E-26**	1.09 (1.01, 1.18)	6.53E-02	1.11 (1.08, 1.15)	**1.99E-10**
3.84E-01	0.92 (0.85, 1.00)	9.08E-02	1.08 (1.04, 1.13)	**1.87E-04**
**2.17E-10**	0.90 (0.83, 0.98)	**2.87E-02**	0.92 (0.88, 0.96)	**1.17E-04**
**1.67E-26**	0.85 (0.78, 0.93)	**1.60E-03**	0.88 (0.84, 0.92)	**4.18E-07**
**2.04E-04**	0.99 (0.91, 1.07)	7.88E-01	1.05 (1.01, 1.10)	**2.11E-02**
**7.22E-24**	0.91 (0.83, 0.99)	6.53E-02	1.02 (0.98, 1.07)	3.54E-01
**8.98E-24**	0.90 (0.83, 0.98)	**4.27E-02**	1.04 (1.00, 1.09)	8.78E-02
**8.56E-11**	1.04 (0.96, 1.13)	3.84E-01	1.05 (1.02, 1.08)	**2.34E-04**
**2.56E-04**	1.03 (0.95, 1.12)	5.25E-01	1.07 (1.03, 1.11)	**2.58E-03**
**8.52E-85**	1.08 (1.05, 1.12)	**3.46E-05**	1.05 (1.03, 1.07)	**1.88E-05**
**8.99E-46**	1.04 (1.02, 1.07)	**6.22E-03**	1.08 (1.06, 1.10)	**1.00E-11**
**4.29E-46**	1.06 (1.03, 1.10)	**1.51E-03**	1.08 (1.05, 1.12)	**8.64E-07**
**6.44E-27**	1.11 (1.08, 1.14)	**1.65E-14**	1.06 (1.05, 1.08)	**9.78E-16**
**3.57E-100**	0.80 (0.74, 0.87)	**1.19E-05**	0.84 (0.81, 0.88)	**6.33E-14**
**2.61E-61**	1.20 (1.11, 1.30)	**1.23E-04**	1.25 (1.20, 1.30)	**9.55E-25**
6.03E-01	1.00 (0.92, 1.09)	9.67E-01	0.88 (0.83, 0.93)	**1.34E-05**
**2.84E-19**	0.92 (0.84, 1.00)	9.37E-02	1.04 (1.00, 1.08)	9.62E-02
**1.62E-51**	0.85 (0.79, 0.93)	**8.69E-04**	0.87 (0.83, 0.91)	**1.73E-09**
**2.78E-64**	0.84 (0.78, 0.92)	**3.92E-04**	0.85 (0.82, 0.89)	**1.69E-11**
**3.09E-70**	0.84 (0.77, 0.91)	**3.10E-04**	0.86 (0.83, 0.90)	**4.94E-10**
**3.63E-02**	0.95 (0.87, 1.04)	3.39E-01	0.84 (0.80, 0.89)	**2.59E-09**
**8.58E-35**	0.90 (0.83, 0.97)	**2.34E-02**	0.81 (0.77, 0.85)	**5.52E-18**
**5.29E-84**	1.04 (0.97, 1.12)	3.38E-01	1.05 (1.01, 1.09)	**8.24E-03**
**2.84E-22**	0.90 (0.82, 0.97)	**2.71E-02**	0.96 (0.91, 1.00)	6.08E-02

**3.44E-65**	0.85 (0.78, 0.92)	**5.05E-04**	0.85 (0.81, 0.88)	**1.40E-12**
9.68E-02	0.98 (0.90, 1.08)	7.83E-01	0.88 (0.83, 0.93)	**6.57E-06**
**9.06E-15**	1.00 (0.93, 1.07)	9.81E-01	1.06 (1.03, 1.08)	**4.96E-05**
**1.54E-62**	1.06 (1.03, 1.10)	**3.31E-03**	1.07 (1.04, 1.10)	**6.15E-07**
**6.76E-35**	0.87 (0.80, 0.95)	**6.37E-03**	0.84 (0.80, 0.88)	**1.40E-12**
**3.74E-15**	0.92 (0.85, 1.00)	9.40E-02	0.97 (0.93, 1.01)	2.05E-01
**5.89E-45**	0.86 (0.79, 0.94)	**1.94E-03**	0.90 (0.86, 0.94)	**2.27E-06**
**1.94E-26**	0.89 (0.81, 0.97)	**2.07E-02**	0.84 (0.80, 0.89)	**2.90E-11**
**3.09E-20**	0.89 (0.82, 0.96)	**1.43E-02**	0.88 (0.84, 0.92)	**4.02E-08**
**1.80E-16**	0.91 (0.84, 0.99)	5.34E-02	0.98 (0.94, 1.02)	3.55E-01
**2.03E-63**	0.85 (0.78, 0.92)	**4.65E-04**	0.87 (0.83, 0.91)	**4.11E-09**
5.14E-01	0.97 (0.89, 1.07)	6.43E-01	0.89 (0.85, 0.94)	**2.81E-05**
**1.24E-34**	0.87 (0.80, 0.95)	**4.25E-03**	0.89 (0.85, 0.93)	**3.78E-07**
**1.67E-17**	0.91 (0.84, 0.99)	6.23E-02	0.98 (0.94, 1.03)	5.08E-01
**2.00E-62**	0.85 (0.78, 0.92)	**5.90E-04**	0.87 (0.83, 0.91)	**4.52E-09**
9.40E-01	0.97 (0.88, 1.06)	5.49E-01	0.87 (0.83, 0.92)	**1.63E-06**
**2.63E-22**	0.89 (0.82, 0.96)	**1.48E-02**	0.92 (0.88, 0.96)	**3.54E-04**
2.04E-01	0.96 (0.87, 1.05)	4.10E-01	0.85 (0.80, 0.90)	**1.16E-08**
**1.96E-02**	1.01 (0.92, 1.10)	8.98E-01	0.99 (0.94, 1.04)	7.29E-01

diseases mortality		Cerebrovascular	
Non-white		White	
Adjusted HR (95% CI)	P value	Adjusted HR (95% CI)	P value
1.09 (0.89, 1.32)	5.99E-01	0.93 (0.87, 1.00)	7.18E-02
1.07 (0.88, 1.30)	6.47E-01	0.93 (0.86, 1.00)	6.34E-02
1.09 (0.89, 1.33)	5.96E-01	0.94 (0.87, 1.00)	1.00E-01
1.07 (0.87, 1.32)	6.55E-01	0.93 (0.87, 1.00)	8.79E-02
1.06 (0.86, 1.31)	7.03E-01	0.93 (0.87, 1.00)	8.43E-02
1.08 (0.89, 1.32)	6.17E-01	0.94 (0.87, 1.00)	9.89E-02
1.06 (0.88, 1.29)	6.62E-01	0.92 (0.86, 0.99)	5.32E-02
1.09 (0.89, 1.34)	5.99E-01	0.90 (0.84, 0.96)	**7.82E-03**
1.08 (0.88, 1.33)	6.29E-01	0.90 (0.83, 0.96)	**6.43E-03**
1.08 (0.87, 1.33)	6.41E-01	0.90 (0.84, 0.97)	**8.76E-03**
1.03 (0.83, 1.29)	8.24E-01	0.89 (0.83, 0.95)	**3.27E-03**
1.00 (0.80, 1.25)	9.87E-01	0.88 (0.82, 0.95)	**1.64E-03**
1.07 (0.86, 1.32)	6.78E-01	0.90 (0.84, 0.97)	**8.39E-03**
1.10 (0.90, 1.34)	5.61E-01	0.90 (0.84, 0.97)	**8.90E-03**
1.09 (0.88, 1.34)	6.17E-01	0.89 (0.83, 0.96)	**3.98E-03**
1.08 (0.87, 1.33)	6.47E-01	0.88 (0.82, 0.95)	**2.19E-03**
1.09 (0.88, 1.35)	5.99E-01	0.90 (0.83, 0.96)	**6.05E-03**
1.05 (0.84, 1.30)	7.74E-01	0.89 (0.83, 0.95)	**3.38E-03**
1.03 (0.83, 1.28)	8.53E-01	0.89 (0.83, 0.96)	**4.84E-03**
1.06 (0.86, 1.32)	7.03E-01	0.89 (0.82, 0.95)	**3.04E-03**
1.08 (0.88, 1.33)	6.31E-01	0.88 (0.82, 0.95)	**1.64E-03**
0.93 (0.75, 1.17)	6.80E-01	0.87 (0.81, 0.93)	**4.07E-04**
0.97 (0.78, 1.21)	8.53E-01	0.87 (0.81, 0.93)	**3.26E-04**
0.93 (0.75, 1.16)	6.57E-01	0.87 (0.81, 0.94)	**4.84E-04**
0.83 (0.66, 1.04)	2.44E-01	0.89 (0.83, 0.95)	**1.75E-03**
0.79 (0.63, 1.00)	1.53E-01	0.90 (0.84, 0.96)	**5.62E-03**
0.90 (0.72, 1.12)	5.29E-01	0.88 (0.82, 0.94)	**6.90E-04**
1.08 (0.87, 1.33)	6.47E-01	0.88 (0.82, 0.95)	**1.64E-03**
1.08 (0.88, 1.34)	6.30E-01	0.92 (0.85, 0.98)	**2.38E-02**
1.06 (0.86, 1.32)	7.03E-01	0.91 (0.84, 0.97)	**1.10E-02**
0.98 (0.78, 1.21)	8.61E-01	0.89 (0.83, 0.95)	**2.84E-03**
0.95 (0.76, 1.18)	7.35E-01	0.90 (0.84, 0.96)	**6.85E-03**
0.97 (0.78, 1.21)	8.55E-01	0.91 (0.85, 0.98)	**1.48E-02**
0.90 (0.72, 1.13)	5.55E-01	0.88 (0.83, 0.95)	**1.73E-03**
1.18 (0.97, 1.44)	2.38E-01	0.94 (0.87, 1.01)	1.03E-01
1.04 (0.84, 1.28)	8.16E-01	0.95 (0.89, 1.02)	1.75E-01
1.06 (0.86, 1.31)	7.05E-01	0.96 (0.89, 1.03)	2.80E-01
1.12 (0.91, 1.38)	4.89E-01	0.97 (0.91, 1.04)	5.16E-01
0.92 (0.74, 1.15)	6.31E-01	0.94 (0.88, 1.01)	1.19E-01
0.88 (0.70, 1.10)	4.39E-01	0.93 (0.87, 1.00)	8.65E-02

1.03 (0.83, 1.28)	8.47E-01	0.96 (0.89, 1.03)	2.84E-01
1.25 (1.04, 1.50)	8.50E-02	0.99 (0.93, 1.06)	8.72E-01
0.81 (0.65, 1.02)	2.00E-01	0.87 (0.82, 0.94)	**5.70E-04**
0.79 (0.63, 0.99)	1.42E-01	0.88 (0.82, 0.94)	**1.09E-03**
0.82 (0.65, 1.03)	2.15E-01	0.90 (0.84, 0.96)	**5.62E-03**
0.74 (0.59, 0.94)	6.77E-02	0.87 (0.81, 0.93)	**3.97E-04**
0.74 (0.59, 0.93)	6.50E-02	0.86 (0.80, 0.93)	**1.79E-04**
0.76 (0.60, 0.95)	8.33E-02	0.89 (0.83, 0.96)	**3.69E-03**
1.22 (1.01, 1.48)	1.41E-01	0.99 (0.93, 1.06)	9.05E-01
0.83 (0.67, 1.04)	2.44E-01	0.88 (0.82, 0.94)	**8.09E-04**
0.76 (0.61, 0.95)	8.33E-02	0.84 (0.79, 0.91)	**1.94E-05**
0.74 (0.59, 0.93)	6.50E-02	0.84 (0.79, 0.91)	**1.87E-05**
0.74 (0.59, 0.93)	6.50E-02	0.84 (0.78, 0.90)	**1.17E-05**
0.76 (0.60, 0.95)	7.93E-02	0.84 (0.78, 0.90)	**8.33E-06**
0.72 (0.57, 0.90)	**3.77E-02**	0.85 (0.80, 0.91)	**5.20E-05**
1.18 (0.97, 1.43)	2.35E-01	0.97 (0.91, 1.04)	4.64E-01
0.85 (0.68, 1.06)	2.93E-01	0.85 (0.80, 0.92)	**6.10E-05**
0.81 (0.65, 1.01)	1.88E-01	0.83 (0.77, 0.89)	**2.56E-06**
0.79 (0.63, 0.99)	1.42E-01	0.82 (0.77, 0.88)	**2.15E-06**
0.79 (0.63, 0.99)	1.43E-01	0.83 (0.77, 0.88)	**2.15E-06**
0.82 (0.66, 1.03)	2.23E-01	0.83 (0.77, 0.89)	**2.53E-06**
0.73 (0.58, 0.91)	**4.66E-02**	0.83 (0.78, 0.89)	**4.30E-06**
1.16 (0.95, 1.40)	2.81E-01	0.95 (0.88, 1.02)	1.75E-01
0.88 (0.71, 1.10)	4.59E-01	0.86 (0.80, 0.92)	**9.51E-05**
0.83 (0.66, 1.04)	2.35E-01	0.84 (0.78, 0.90)	**1.13E-05**
0.84 (0.67, 1.04)	2.45E-01	0.86 (0.80, 0.92)	**5.22E-05**
0.80 (0.64, 1.00)	1.65E-01	0.83 (0.78, 0.90)	**5.18E-06**
0.83 (0.67, 1.04)	2.44E-01	0.84 (0.78, 0.90)	**6.83E-06**
0.75 (0.61, 0.93)	6.50E-02	0.85 (0.79, 0.91)	**1.99E-05**
1.14 (0.94, 1.38)	3.21E-01	0.94 (0.87, 1.01)	1.02E-01
1.01 (0.79, 1.28)	9.84E-01	1.10 (1.02, 1.18)	**1.74E-02**
1.01 (0.79, 1.29)	9.56E-01	1.12 (1.04, 1.20)	**4.39E-03**
1.04 (0.82, 1.32)	8.16E-01	1.13 (1.06, 1.21)	**1.68E-03**
0.95 (0.74, 1.22)	7.95E-01	1.10 (1.03, 1.18)	**1.37E-02**
0.95 (0.74, 1.22)	7.64E-01	1.10 (1.02, 1.18)	**1.75E-02**
0.98 (0.78, 1.24)	8.99E-01	1.11 (1.03, 1.18)	**8.34E-03**
1.19 (0.98, 1.43)	2.03E-01	1.01 (0.95, 1.08)	7.82E-01
0.94 (0.72, 1.21)	7.35E-01	1.09 (1.01, 1.17)	**4.94E-02**
0.94 (0.73, 1.22)	7.49E-01	1.09 (1.01, 1.17)	**4.37E-02**
0.95 (0.74, 1.23)	7.98E-01	1.08 (1.01, 1.17)	5.88E-02
0.91 (0.70, 1.18)	6.39E-01	1.09 (1.01, 1.17)	**3.83E-02**
0.90 (0.69, 1.17)	6.17E-01	1.09 (1.01, 1.17)	**3.83E-02**
0.94 (0.73, 1.22)	7.45E-01	1.09 (1.01, 1.17)	**4.16E-02**
1.19 (0.97, 1.45)	2.30E-01	1.04 (0.97, 1.12)	3.03E-01
0.84 (0.65, 1.07)	3.04E-01	0.96 (0.89, 1.03)	3.38E-01
0.86 (0.67, 1.10)	4.17E-01	0.96 (0.89, 1.03)	3.10E-01
0.89 (0.70, 1.13)	5.31E-01	0.96 (0.89, 1.03)	2.85E-01
0.80 (0.62, 1.04)	2.30E-01	0.96 (0.89, 1.04)	4.01E-01

0.80 (0.62, 1.03)	2.12E-01	0.96 (0.89, 1.04)	4.02E-01
0.84 (0.66, 1.09)	3.42E-01	0.96 (0.89, 1.04)	4.02E-01
1.16 (0.95, 1.41)	2.97E-01	0.97 (0.90, 1.04)	4.43E-01
0.74 (0.59, 0.93)	6.50E-02	0.82 (0.76, 0.88)	**1.75E-06**
0.82 (0.65, 1.03)	2.23E-01	0.85 (0.79, 0.91)	**2.33E-05**
0.85 (0.68, 1.07)	3.05E-01	0.86 (0.80, 0.92)	**1.68E-04**
0.73 (0.58, 0.91)	5.91E-02	0.83 (0.77, 0.89)	**2.56E-06**
0.70 (0.56, 0.88)	**3.16E-02**	0.82 (0.77, 0.88)	**2.15E-06**
0.85 (0.68, 1.07)	3.04E-01	0.86 (0.80, 0.92)	**1.67E-04**
1.17 (0.96, 1.43)	2.60E-01	0.94 (0.87, 1.01)	1.02E-01
1.38 (1.08, 1.78)	6.50E-02	1.11 (1.03, 1.20)	**1.45E-02**
0.97 (0.76, 1.25)	8.61E-01	1.09 (1.02, 1.16)	**2.18E-02**
0.93 (0.71, 1.23)	7.35E-01	1.06 (0.99, 1.13)	1.62E-01
1.04 (0.85, 1.28)	7.92E-01	1.12 (1.06, 1.19)	**6.61E-04**
0.94 (0.75, 1.17)	7.03E-01	0.90 (0.85, 0.96)	**5.63E-03**
0.78 (0.58, 1.04)	2.23E-01	1.10 (1.03, 1.18)	**1.34E-02**
0.76 (0.56, 1.03)	2.03E-01	1.08 (1.01, 1.16)	5.84E-02
0.91 (0.72, 1.16)	6.31E-01	1.15 (1.08, 1.23)	**9.09E-05**
1.20 (0.93, 1.55)	3.01E-01	0.91 (0.85, 0.97)	**1.09E-02**
1.45 (1.02, 2.07)	1.42E-01	0.97 (0.90, 1.04)	4.16E-01
0.95 (0.75, 1.19)	7.36E-01	1.10 (1.03, 1.17)	**1.30E-02**
0.93 (0.74, 1.17)	6.59E-01	1.11 (1.04, 1.19)	**4.35E-03**
1.00 (0.79, 1.27)	9.87E-01	1.03 (0.96, 1.11)	4.86E-01
0.91 (0.72, 1.16)	6.17E-01	0.95 (0.88, 1.02)	1.81E-01
0.84 (0.70, 1.01)	1.99E-01	0.99 (0.93, 1.06)	9.03E-01
0.78 (0.64, 0.96)	8.33E-02	1.03 (0.96, 1.10)	5.53E-01
0.78 (0.64, 0.96)	8.33E-02	1.03 (0.96, 1.10)	4.68E-01
0.80 (0.66, 0.97)	1.02E-01	1.01 (0.94, 1.08)	8.36E-01
1.26 (1.03, 1.54)	9.40E-02	0.98 (0.92, 1.05)	6.78E-01
0.71 (0.57, 0.88)	**2.56E-02**	0.93 (0.87, 1.00)	7.02E-02
0.77 (0.63, 0.95)	7.45E-02	0.98 (0.91, 1.04)	5.64E-01
0.85 (0.69, 1.03)	2.38E-01	1.01 (0.94, 1.08)	9.03E-01
0.70 (0.57, 0.87)	**2.46E-02**	0.94 (0.88, 1.01)	1.13E-01
1.33 (1.08, 1.64)	5.91E-02	1.04 (0.97, 1.11)	3.40E-01
1.63 (1.29, 2.05)	**1.92E-03**	1.17 (1.09, 1.25)	**6.10E-05**
0.71 (0.59, 0.86)	**1.22E-02**	0.94 (0.88, 1.01)	1.03E-01
0.70 (0.58, 0.85)	**8.83E-03**	0.93 (0.87, 1.00)	7.12E-02
0.84 (0.70, 1.01)	1.87E-01	1.00 (0.94, 1.06)	9.61E-01
1.34 (1.11, 1.61)	**3.16E-02**	1.04 (0.97, 1.11)	3.56E-01
1.38 (1.13, 1.69)	**2.56E-02**	1.17 (1.09, 1.25)	**3.60E-05**
0.74 (0.64, 0.85)	**1.80E-03**	0.87 (0.82, 0.93)	**5.15E-05**
0.71 (0.62, 0.83)	**1.80E-03**	0.84 (0.79, 0.89)	**1.75E-06**
0.83 (0.70, 0.99)	1.41E-01	1.00 (0.94, 1.07)	9.72E-01
1.29 (1.13, 1.47)	**4.82E-03**	1.12 (1.05, 1.19)	**7.33E-04**
0.92 (0.73, 1.15)	6.24E-01	1.05 (1.00, 1.12)	9.67E-02
0.88 (0.81, 0.96)	**3.17E-02**	0.91 (0.89, 0.94)	**3.82E-07**
0.91 (0.82, 1.01)	2.00E-01	0.91 (0.89, 0.94)	**1.06E-08**
0.80 (0.69, 0.93)	**3.17E-02**	0.95 (0.89, 1.01)	1.31E-01

1.19 (1.08, 1.31)	**1.28E-02**	1.11 (1.07, 1.16)	**2.15E-06**
0.88 (0.71, 1.10)	4.64E-01	1.03 (0.97, 1.10)	3.80E-01
0.90 (0.84, 0.98)	6.50E-02	0.92 (0.89, 0.95)	**5.18E-06**
1.00 (0.81, 1.24)	9.87E-01	0.89 (0.84, 0.93)	**3.72E-05**
0.80 (0.68, 0.95)	6.50E-02	0.99 (0.93, 1.06)	9.12E-01
1.14 (1.05, 1.24)	**2.55E-02**	1.10 (1.06, 1.14)	**4.36E-06**
1.10 (0.89, 1.35)	5.96E-01	1.13 (1.06, 1.20)	**2.57E-04**
0.82 (0.73, 0.92)	**2.46E-02**	0.89 (0.86, 0.93)	**2.15E-06**
0.93 (0.75, 1.15)	6.47E-01	0.89 (0.84, 0.94)	**3.47E-04**
0.83 (0.71, 0.97)	9.40E-02	0.96 (0.90, 1.03)	3.40E-01
1.12 (1.03, 1.23)	6.50E-02	1.06 (1.00, 1.12)	1.03E-01
1.07 (0.87, 1.33)	6.57E-01	1.15 (1.06, 1.25)	**1.64E-03**
0.88 (0.73, 1.06)	3.19E-01	0.88 (0.82, 0.95)	**2.75E-03**
0.89 (0.77, 1.03)	2.56E-01	0.95 (0.89, 1.01)	1.35E-01
0.96 (0.78, 1.19)	8.09E-01	0.90 (0.83, 0.97)	**1.09E-02**
1.03 (0.93, 1.14)	7.05E-01	0.94 (0.87, 1.01)	1.27E-01
1.15 (0.97, 1.38)	2.45E-01	0.94 (0.88, 1.00)	6.95E-02
0.87 (0.75, 1.01)	2.00E-01	1.05 (0.98, 1.13)	1.98E-01
0.85 (0.73, 1.00)	1.61E-01	1.05 (0.98, 1.13)	1.98E-01
0.95 (0.80, 1.11)	6.50E-01	1.03 (0.97, 1.10)	4.27E-01
1.05 (0.96, 1.14)	5.06E-01	0.98 (0.92, 1.05)	6.54E-01
1.26 (1.04, 1.51)	8.22E-02	1.01 (0.94, 1.08)	8.41E-01
0.78 (0.64, 0.94)	6.50E-02	0.99 (0.93, 1.07)	9.05E-01
0.79 (0.66, 0.95)	7.45E-02	1.00 (0.93, 1.07)	9.88E-01
0.85 (0.69, 1.05)	2.82E-01	0.97 (0.90, 1.04)	4.56E-01
1.28 (1.05, 1.55)	7.22E-02	1.00 (0.94, 1.08)	9.61E-01
1.22 (0.98, 1.51)	2.00E-01	1.11 (1.03, 1.19)	**9.46E-03**
0.74 (0.61, 0.90)	**3.17E-02**	0.93 (0.87, 1.00)	6.95E-02
0.73 (0.61, 0.88)	**2.46E-02**	0.92 (0.86, 0.98)	**2.67E-02**
1.11 (0.90, 1.37)	5.40E-01	1.05 (0.98, 1.12)	2.10E-01
1.35 (1.10, 1.64)	**3.73E-02**	1.01 (0.94, 1.09)	8.27E-01
1.01 (0.81, 1.26)	9.50E-01	0.85 (0.79, 0.91)	**4.52E-05**
0.85 (0.69, 1.05)	2.60E-01	1.05 (0.98, 1.12)	2.31E-01
1.04 (0.82, 1.33)	7.98E-01	1.15 (1.07, 1.23)	**9.61E-04**
0.77 (0.61, 0.96)	8.91E-02	0.86 (0.80, 0.92)	**1.52E-04**
0.95 (0.76, 1.19)	7.55E-01	0.89 (0.83, 0.96)	**3.79E-03**
0.76 (0.61, 0.95)	8.22E-02	0.83 (0.78, 0.89)	**5.18E-06**
0.84 (0.67, 1.05)	2.60E-01	0.87 (0.81, 0.94)	**4.66E-04**
0.75 (0.60, 0.94)	7.45E-02	0.86 (0.80, 0.92)	**1.29E-04**
0.80 (0.64, 1.01)	1.77E-01	0.87 (0.81, 0.93)	**3.02E-04**
1.12 (0.92, 1.37)	4.42E-01	0.91 (0.85, 0.98)	**2.19E-02**
1.10 (0.90, 1.35)	5.40E-01	0.90 (0.84, 0.97)	**9.71E-03**
1.17 (0.96, 1.42)	2.45E-01	0.96 (0.89, 1.03)	3.08E-01
1.18 (0.97, 1.43)	2.40E-01	0.98 (0.91, 1.04)	5.53E-01
0.91 (0.73, 1.14)	5.99E-01	0.90 (0.84, 0.97)	**8.39E-03**
1.21 (0.99, 1.49)	1.96E-01	0.94 (0.88, 1.01)	1.34E-01

0.90 (0.71, 1.13)	5.40E-01	0.90 (0.83, 0.97)	**8.39E-03**
0.84 (0.67, 1.05)	2.66E-01	0.92 (0.86, 0.99)	**4.94E-02**
0.87 (0.69, 1.10)	4.25E-01	0.90 (0.83, 0.97)	**8.39E-03**
0.84 (0.65, 1.07)	3.04E-01	0.95 (0.89, 1.03)	2.97E-01
0.85 (0.68, 1.06)	3.00E-01	0.87 (0.81, 0.93)	**2.82E-04**
0.95 (0.76, 1.19)	7.64E-01	0.91 (0.85, 0.98)	**2.11E-02**
1.03 (0.83, 1.26)	8.55E-01	0.89 (0.83, 0.96)	**4.20E-03**
0.81 (0.65, 1.00)	1.53E-01	0.94 (0.87, 1.00)	9.12E-02
0.90 (0.71, 1.13)	5.43E-01	0.91 (0.85, 0.98)	**2.55E-02**
0.89 (0.71, 1.12)	5.29E-01	0.88 (0.82, 0.95)	**1.89E-03**
0.96 (0.77, 1.20)	7.98E-01	0.88 (0.82, 0.95)	**2.15E-03**
0.89 (0.71, 1.11)	4.97E-01	0.87 (0.81, 0.93)	**4.01E-04**
0.89 (0.71, 1.12)	5.12E-01	0.85 (0.80, 0.92)	**1.07E-04**
1.15 (0.95, 1.40)	2.90E-01	0.94 (0.87, 1.01)	1.02E-01
1.03 (0.84, 1.26)	8.53E-01	0.90 (0.84, 0.97)	**9.24E-03**
0.88 (0.70, 1.10)	4.42E-01	0.99 (0.92, 1.06)	8.42E-01
0.84 (0.68, 1.03)	2.23E-01	0.99 (0.92, 1.06)	8.21E-01
0.92 (0.74, 1.16)	6.47E-01	0.93 (0.87, 1.00)	7.05E-02
0.84 (0.69, 1.02)	2.03E-01	1.03 (0.96, 1.10)	4.68E-01
0.82 (0.68, 0.99)	1.42E-01	1.00 (0.93, 1.07)	9.97E-01
1.37 (1.12, 1.67)	**2.59E-02**	1.02 (0.95, 1.10)	5.88E-01
1.00 (0.82, 1.22)	9.87E-01	0.97 (0.91, 1.04)	4.74E-01
1.24 (1.12, 1.37)	**1.80E-03**	1.06 (1.01, 1.12)	**3.28E-02**
1.06 (0.85, 1.31)	7.25E-01	1.00 (0.94, 1.07)	9.72E-01
1.19 (0.96, 1.47)	2.43E-01	1.06 (1.00, 1.12)	8.79E-02
1.11 (0.91, 1.35)	4.95E-01	1.02 (0.96, 1.09)	5.58E-01
1.13 (0.91, 1.40)	4.42E-01	1.11 (1.04, 1.19)	**5.63E-03**
1.00 (0.79, 1.25)	9.87E-01	0.99 (0.92, 1.06)	8.36E-01
1.18 (0.97, 1.43)	2.35E-01	0.95 (0.89, 1.02)	2.10E-01
1.16 (0.95, 1.42)	3.00E-01	0.87 (0.81, 0.94)	**1.14E-03**
1.24 (1.01, 1.52)	1.46E-01	0.88 (0.82, 0.94)	**1.64E-03**
1.28 (1.06, 1.55)	6.50E-02	1.01 (0.95, 1.08)	7.82E-01
1.30 (1.07, 1.57)	6.11E-02	0.99 (0.93, 1.06)	8.36E-01
1.07 (0.99, 1.16)	2.23E-01	1.07 (1.04, 1.10)	**5.92E-05**
1.03 (0.96, 1.11)	5.89E-01	1.06 (1.02, 1.11)	**1.13E-02**
1.03 (0.93, 1.15)	6.80E-01	1.07 (1.02, 1.13)	**9.73E-03**
1.12 (1.06, 1.18)	**1.80E-03**	1.06 (1.03, 1.10)	**1.52E-04**
0.84 (0.67, 1.04)	2.45E-01	0.90 (0.84, 0.97)	**8.90E-03**
1.39 (1.13, 1.69)	**2.55E-02**	1.07 (1.00, 1.15)	7.61E-02
1.10 (0.86, 1.42)	6.17E-01	1.09 (1.01, 1.17)	**3.54E-02**
1.21 (0.99, 1.48)	1.90E-01	0.89 (0.82, 0.95)	**3.43E-03**
0.79 (0.63, 0.99)	1.46E-01	0.85 (0.79, 0.91)	**3.72E-05**
0.74 (0.59, 0.93)	6.50E-02	0.84 (0.78, 0.90)	**1.17E-05**
0.78 (0.62, 0.97)	1.13E-01	0.83 (0.78, 0.89)	**4.36E-06**
0.78 (0.60, 1.02)	2.03E-01	1.00 (0.92, 1.08)	9.57E-01
0.75 (0.61, 0.94)	6.50E-02	1.00 (0.93, 1.07)	9.73E-01
0.95 (0.73, 1.24)	7.98E-01	1.07 (1.02, 1.13)	**9.32E-03**
1.00 (0.80, 1.24)	9.84E-01	0.89 (0.83, 0.95)	**2.98E-03**

0.72 (0.58, 0.90)	**3.96E-02**	0.85 (0.79, 0.91)	**1.95E-05**
0.87 (0.67, 1.12)	4.64E-01	1.00 (0.93, 1.08)	9.69E-01
1.06 (0.94, 1.19)	5.22E-01	1.01 (0.94, 1.08)	8.72E-01
1.07 (1.00, 1.15)	1.99E-01	1.08 (1.04, 1.12)	**1.23E-04**
0.76 (0.60, 0.97)	1.04E-01	0.88 (0.82, 0.95)	**2.19E-03**
1.04 (0.84, 1.28)	8.16E-01	0.90 (0.84, 0.97)	**8.76E-03**
0.84 (0.67, 1.05)	2.62E-01	0.87 (0.81, 0.93)	**2.36E-04**
0.77 (0.60, 0.98)	1.36E-01	0.90 (0.83, 0.97)	**9.32E-03**
0.86 (0.68, 1.07)	3.19E-01	0.88 (0.82, 0.95)	**2.04E-03**
1.04 (0.84, 1.29)	7.98E-01	0.90 (0.84, 0.96)	**5.52E-03**
0.77 (0.61, 0.96)	8.81E-02	0.84 (0.78, 0.90)	**7.60E-06**
0.89 (0.69, 1.14)	5.57E-01	0.99 (0.92, 1.07)	8.31E-01
0.87 (0.70, 1.08)	3.76E-01	0.86 (0.80, 0.92)	**1.56E-04**
1.05 (0.85, 1.30)	7.35E-01	0.89 (0.83, 0.96)	**4.25E-03**
0.78 (0.62, 0.97)	1.13E-01	0.84 (0.78, 0.90)	**7.97E-06**
0.86 (0.67, 1.11)	4.42E-01	0.99 (0.92, 1.07)	8.64E-01
0.92 (0.74, 1.15)	6.30E-01	0.90 (0.84, 0.96)	**5.63E-03**
0.80 (0.61, 1.04)	2.35E-01	1.00 (0.92, 1.08)	9.71E-01
1.12 (0.85, 1.48)	6.01E-01	1.02 (0.94, 1.10)	7.34E-01

diseases mortality			Cancer mo
Non-white		White	
Adjusted HR (95% CI)	P value	Adjusted HR (95% CI)	P value
0.99 (0.65, 1.51)	9.95E-01	1.02 (1.00, 1.04)	6.73E-02
1.01 (0.67, 1.52)	9.95E-01	1.02 (1.00, 1.04)	9.20E-02
0.98 (0.64, 1.50)	9.95E-01	1.02 (1.00, 1.04)	**4.85E-02**
0.99 (0.64, 1.51)	9.95E-01	1.02 (1.00, 1.04)	1.10E-01
1.00 (0.65, 1.52)	9.95E-01	1.02 (1.00, 1.04)	1.67E-01
0.97 (0.63, 1.49)	9.95E-01	1.02 (1.00, 1.04)	6.97E-02
1.02 (0.68, 1.52)	9.95E-01	1.02 (1.00, 1.04)	1.03E-01
0.98 (0.64, 1.51)	9.95E-01	1.00 (0.98, 1.02)	9.74E-01
0.98 (0.64, 1.51)	9.95E-01	1.00 (0.98, 1.02)	8.74E-01
0.96 (0.62, 1.49)	9.95E-01	1.00 (0.98, 1.02)	9.28E-01
0.94 (0.61, 1.47)	9.95E-01	0.99 (0.97, 1.01)	3.59E-01
0.93 (0.60, 1.45)	9.95E-01	0.98 (0.96, 1.00)	7.97E-02
0.96 (0.62, 1.48)	9.95E-01	1.00 (0.98, 1.02)	9.50E-01
1.00 (0.65, 1.52)	9.95E-01	1.00 (0.98, 1.02)	9.98E-01
0.98 (0.63, 1.51)	9.95E-01	0.99 (0.97, 1.01)	3.57E-01
0.96 (0.62, 1.49)	9.95E-01	0.98 (0.96, 1.00)	1.09E-01
0.96 (0.62, 1.49)	9.95E-01	0.99 (0.97, 1.01)	5.45E-01
0.94 (0.60, 1.46)	9.95E-01	0.98 (0.96, 1.00)	1.51E-01
0.93 (0.60, 1.44)	9.95E-01	0.98 (0.96, 1.00)	1.01E-01
0.96 (0.61, 1.49)	9.95E-01	0.99 (0.97, 1.01)	2.27E-01
0.97 (0.63, 1.50)	9.95E-01	0.98 (0.96, 1.00)	**4.32E-02**
0.95 (0.62, 1.46)	9.95E-01	0.96 (0.94, 0.98)	**1.32E-04**
0.97 (0.63, 1.49)	9.95E-01	0.96 (0.94, 0.98)	**1.46E-04**
0.96 (0.62, 1.46)	9.95E-01	0.96 (0.94, 0.98)	**1.89E-04**
0.94 (0.62, 1.43)	9.95E-01	0.95 (0.93, 0.97)	**3.01E-06**
0.93 (0.61, 1.42)	9.95E-01	0.95 (0.93, 0.97)	**7.90E-07**
0.96 (0.63, 1.46)	9.95E-01	0.96 (0.94, 0.98)	**6.47E-05**
0.99 (0.65, 1.53)	9.95E-01	0.97 (0.95, 0.99)	**1.06E-02**
1.01 (0.66, 1.53)	9.95E-01	0.99 (0.97, 1.01)	4.75E-01
1.01 (0.67, 1.54)	9.95E-01	0.99 (0.97, 1.01)	2.57E-01
0.98 (0.65, 1.50)	9.95E-01	0.97 (0.95, 0.99)	**6.27E-03**
0.96 (0.63, 1.46)	9.95E-01	0.98 (0.96, 1.00)	**3.13E-02**
0.95 (0.63, 1.46)	9.95E-01	0.99 (0.97, 1.01)	2.17E-01
0.97 (0.64, 1.47)	9.95E-01	0.96 (0.94, 0.98)	**1.71E-04**
1.07 (0.71, 1.60)	9.95E-01	1.01 (0.99, 1.03)	5.06E-01
1.06 (0.71, 1.59)	9.95E-01	1.02 (1.00, 1.04)	**4.35E-02**
1.05 (0.70, 1.58)	9.95E-01	1.03 (1.01, 1.05)	**1.28E-02**
1.03 (0.68, 1.54)	9.95E-01	1.04 (1.02, 1.06)	**1.99E-04**
1.02 (0.68, 1.54)	9.95E-01	1.01 (0.98, 1.03)	6.61E-01
1.02 (0.67, 1.54)	9.95E-01	1.00 (0.98, 1.02)	8.29E-01

1.02 (0.68, 1.54)	9.95E-01	1.02 (1.00, 1.04)	**3.60E-02**
1.11 (0.76, 1.63)	9.95E-01	1.05 (1.03, 1.07)	**2.82E-06**
0.92 (0.60, 1.39)	9.95E-01	0.96 (0.94, 0.98)	**4.58E-04**
1.05 (0.69, 1.58)	9.95E-01	0.96 (0.94, 0.97)	**2.75E-05**
1.04 (0.69, 1.57)	9.95E-01	0.97 (0.95, 0.99)	**3.30E-03**
1.03 (0.68, 1.56)	9.95E-01	0.94 (0.92, 0.96)	**4.95E-08**
1.03 (0.68, 1.56)	9.95E-01	0.94 (0.92, 0.96)	**6.77E-09**
1.02 (0.67, 1.54)	9.95E-01	0.95 (0.93, 0.97)	**1.49E-05**
1.17 (0.80, 1.70)	9.95E-01	1.06 (1.04, 1.08)	**5.58E-08**
1.02 (0.67, 1.54)	9.95E-01	0.95 (0.93, 0.97)	**3.19E-07**
1.06 (0.70, 1.60)	9.95E-01	0.93 (0.91, 0.95)	**4.41E-12**
1.01 (0.67, 1.53)	9.95E-01	0.93 (0.91, 0.95)	**2.58E-12**
1.06 (0.70, 1.60)	9.95E-01	0.92 (0.90, 0.94)	**4.03E-14**
1.07 (0.71, 1.62)	9.95E-01	0.92 (0.90, 0.94)	**5.88E-14**
1.03 (0.68, 1.56)	9.95E-01	0.92 (0.90, 0.94)	**5.48E-14**
1.19 (0.82, 1.73)	9.95E-01	1.05 (1.03, 1.07)	**2.11E-05**
0.93 (0.61, 1.43)	9.95E-01	0.95 (0.93, 0.97)	**4.36E-07**
0.98 (0.64, 1.49)	9.95E-01	0.93 (0.91, 0.95)	**1.86E-12**
1.01 (0.66, 1.53)	9.95E-01	0.92 (0.90, 0.94)	**4.75E-14**
0.95 (0.62, 1.46)	9.95E-01	0.92 (0.91, 0.94)	**1.01E-13**
0.93 (0.61, 1.43)	9.95E-01	0.93 (0.91, 0.95)	**1.18E-11**
1.01 (0.66, 1.53)	9.95E-01	0.91 (0.89, 0.93)	**1.86E-18**
1.15 (0.79, 1.67)	9.95E-01	1.03 (1.01, 1.05)	**1.10E-02**
1.02 (0.67, 1.54)	9.95E-01	0.95 (0.94, 0.97)	**1.09E-05**
0.98 (0.65, 1.49)	9.95E-01	0.94 (0.92, 0.95)	**3.24E-10**
0.99 (0.66, 1.50)	9.95E-01	0.94 (0.92, 0.96)	**7.41E-09**
0.97 (0.63, 1.47)	9.95E-01	0.93 (0.91, 0.95)	**1.45E-12**
0.96 (0.63, 1.47)	9.95E-01	0.94 (0.92, 0.95)	**3.24E-10**
0.98 (0.65, 1.48)	9.95E-01	0.92 (0.90, 0.93)	**3.99E-17**
1.10 (0.75, 1.61)	9.95E-01	1.02 (1.00, 1.04)	7.38E-02
1.27 (0.86, 1.87)	9.95E-01	1.03 (1.01, 1.05)	**1.66E-02**
1.25 (0.84, 1.86)	9.95E-01	1.04 (1.01, 1.06)	**2.69E-03**
1.25 (0.84, 1.86)	9.95E-01	1.04 (1.02, 1.07)	**2.38E-04**
1.24 (0.83, 1.84)	9.95E-01	1.02 (1.00, 1.04)	7.97E-02
1.25 (0.84, 1.86)	9.95E-01	1.01 (0.99, 1.04)	2.91E-01
1.17 (0.78, 1.75)	9.95E-01	1.05 (1.03, 1.07)	**2.11E-05**
1.17 (0.82, 1.69)	9.95E-01	1.05 (1.03, 1.07)	**1.49E-06**
1.32 (0.89, 1.97)	9.95E-01	1.00 (0.98, 1.03)	8.53E-01
1.33 (0.90, 1.99)	9.95E-01	1.01 (0.98, 1.03)	6.03E-01
1.35 (0.91, 2.01)	9.95E-01	1.01 (0.99, 1.03)	4.24E-01
1.30 (0.87, 1.95)	9.95E-01	1.00 (0.98, 1.02)	8.99E-01
1.30 (0.87, 1.94)	9.95E-01	0.99 (0.97, 1.02)	6.61E-01
1.32 (0.89, 1.97)	9.95E-01	1.01 (0.99, 1.04)	3.50E-01
1.20 (0.82, 1.74)	9.95E-01	1.06 (1.04, 1.08)	**2.11E-08**
1.31 (0.87, 1.96)	9.95E-01	0.96 (0.94, 0.98)	**4.92E-04**
1.30 (0.87, 1.94)	9.95E-01	0.97 (0.95, 0.99)	**3.28E-03**
1.29 (0.87, 1.93)	9.95E-01	0.97 (0.95, 1.00)	**2.87E-02**
1.29 (0.86, 1.94)	9.95E-01	0.95 (0.93, 0.97)	**1.78E-05**

1.28 (0.85, 1.92)	9.95E-01	0.95 (0.93, 0.97)	**5.58E-06**
1.34 (0.89, 2.00)	9.95E-01	0.96 (0.94, 0.98)	**1.58E-03**
1.09 (0.73, 1.61)	9.95E-01	1.04 (1.02, 1.06)	**1.46E-04**
1.09 (0.72, 1.65)	9.95E-01	0.90 (0.88, 0.92)	**5.82E-22**
1.12 (0.74, 1.69)	9.95E-01	0.93 (0.91, 0.95)	**4.23E-11**
1.13 (0.75, 1.70)	9.95E-01	0.95 (0.93, 0.97)	**3.10E-07**
1.11 (0.73, 1.68)	9.95E-01	0.90 (0.88, 0.92)	**2.69E-23**
1.06 (0.70, 1.61)	9.95E-01	0.89 (0.87, 0.91)	**6.07E-27**
1.23 (0.82, 1.85)	9.95E-01	0.93 (0.92, 0.95)	**6.77E-10**
1.03 (0.68, 1.56)	9.95E-01	1.03 (1.01, 1.05)	**1.72E-02**
0.78 (0.52, 1.18)	9.95E-01	1.04 (1.02, 1.07)	**2.84E-04**
1.08 (0.71, 1.65)	9.95E-01	1.00 (0.98, 1.03)	7.71E-01
1.10 (0.71, 1.68)	9.95E-01	1.00 (0.98, 1.02)	9.00E-01
1.02 (0.69, 1.52)	9.95E-01	1.01 (0.99, 1.03)	2.48E-01
1.01 (0.65, 1.58)	9.95E-01	0.99 (0.96, 1.01)	1.88E-01
0.96 (0.60, 1.54)	9.95E-01	0.99 (0.96, 1.01)	2.27E-01
1.01 (0.64, 1.59)	9.95E-01	0.98 (0.95, 1.00)	**4.84E-02**
0.80 (0.51, 1.26)	9.95E-01	1.02 (1.00, 1.05)	**4.41E-02**
1.22 (0.76, 1.95)	9.95E-01	1.00 (0.98, 1.02)	9.54E-01
0.87 (0.59, 1.27)	9.95E-01	1.07 (1.05, 1.10)	**9.76E-08**
0.84 (0.55, 1.29)	9.95E-01	1.03 (1.01, 1.05)	**6.95E-03**
0.84 (0.54, 1.30)	9.95E-01	1.02 (1.00, 1.04)	5.92E-02
0.89 (0.59, 1.33)	9.95E-01	1.05 (1.02, 1.07)	**8.74E-05**
1.20 (0.83, 1.74)	9.95E-01	0.95 (0.93, 0.97)	**8.20E-07**
0.87 (0.61, 1.25)	9.95E-01	0.99 (0.97, 1.01)	5.02E-01
0.89 (0.59, 1.33)	9.95E-01	0.97 (0.95, 0.99)	**1.19E-02**
0.89 (0.59, 1.33)	9.95E-01	0.97 (0.95, 0.99)	**4.51E-03**
0.89 (0.60, 1.31)	9.95E-01	0.98 (0.96, 1.00)	1.25E-01
1.14 (0.77, 1.69)	9.95E-01	1.02 (1.00, 1.04)	**3.21E-02**
0.83 (0.54, 1.26)	9.95E-01	0.93 (0.91, 0.95)	**3.32E-11**
0.77 (0.51, 1.15)	9.95E-01	0.97 (0.95, 0.99)	**1.74E-03**
0.75 (0.51, 1.09)	9.95E-01	1.00 (0.98, 1.02)	7.51E-01
0.83 (0.54, 1.28)	9.95E-01	0.93 (0.91, 0.95)	**6.81E-11**
1.28 (0.85, 1.93)	9.95E-01	1.05 (1.03, 1.07)	**2.24E-05**
0.73 (0.57, 0.95)	8.89E-01	1.12 (1.10, 1.15)	**4.79E-28**
0.93 (0.62, 1.39)	9.95E-01	0.94 (0.92, 0.96)	**3.51E-08**
0.96 (0.63, 1.45)	9.95E-01	0.94 (0.92, 0.96)	**3.96E-09**
0.80 (0.56, 1.14)	9.95E-01	0.98 (0.96, 1.00)	7.66E-02
1.19 (0.81, 1.75)	9.95E-01	1.04 (1.02, 1.06)	**1.61E-04**
0.98 (0.66, 1.47)	9.95E-01	1.11 (1.08, 1.13)	**4.40E-22**
0.93 (0.66, 1.32)	9.95E-01	0.90 (0.89, 0.92)	**2.20E-25**
0.92 (0.64, 1.32)	9.95E-01	0.89 (0.87, 0.91)	**4.08E-32**
0.98 (0.67, 1.43)	9.95E-01	0.98 (0.96, 1.00)	**3.46E-02**
1.08 (0.78, 1.50)	9.95E-01	1.09 (1.07, 1.11)	**3.43E-19**
0.79 (0.51, 1.22)	9.95E-01	1.01 (1.00, 1.03)	1.68E-01
0.97 (0.69, 1.35)	9.95E-01	0.93 (0.92, 0.94)	**2.32E-32**
1.01 (0.67, 1.53)	9.95E-01	0.94 (0.93, 0.95)	**2.52E-29**
0.93 (0.65, 1.34)	9.95E-01	0.95 (0.93, 0.97)	**8.77E-08**

1.09 (0.82, 1.45)	9.95E-01	1.09 (1.07, 1.10)	**8.27E-31**
1.25 (0.95, 1.63)	9.95E-01	0.98 (0.96, 1.00)	**4.92E-02**
0.89 (0.76, 1.04)	9.95E-01	0.94 (0.92, 0.95)	**1.18E-23**
0.81 (0.66, 0.99)	9.95E-01	0.97 (0.95, 0.99)	**2.57E-03**
1.08 (0.70, 1.65)	9.95E-01	0.94 (0.92, 0.96)	**1.23E-08**
1.12 (0.89, 1.41)	9.95E-01	1.09 (1.07, 1.10)	**2.57E-34**
0.99 (0.66, 1.49)	9.95E-01	1.05 (1.03, 1.07)	**3.01E-06**
0.89 (0.64, 1.25)	9.95E-01	0.91 (0.90, 0.93)	**3.62E-29**
0.88 (0.59, 1.33)	9.95E-01	0.96 (0.94, 0.98)	**1.18E-04**
0.99 (0.67, 1.45)	9.95E-01	0.94 (0.92, 0.95)	**1.28E-11**
1.11 (0.85, 1.44)	9.95E-01	1.07 (1.05, 1.09)	**1.13E-13**
1.03 (0.69, 1.53)	9.95E-01	1.06 (1.04, 1.09)	**7.44E-08**
0.98 (0.67, 1.43)	9.95E-01	0.92 (0.90, 0.94)	**5.55E-14**
0.90 (0.66, 1.22)	9.95E-01	0.92 (0.90, 0.94)	**1.14E-19**
1.10 (0.79, 1.52)	9.95E-01	0.99 (0.97, 1.01)	2.91E-01
0.99 (0.77, 1.28)	9.95E-01	1.00 (0.98, 1.02)	8.45E-01
1.19 (0.85, 1.68)	9.95E-01	1.03 (1.01, 1.05)	**1.25E-02**
0.89 (0.65, 1.22)	9.95E-01	0.97 (0.95, 0.99)	**2.43E-03**
0.88 (0.64, 1.22)	9.95E-01	0.96 (0.94, 0.98)	**4.05E-05**
0.93 (0.68, 1.28)	9.95E-01	1.02 (1.00, 1.05)	**2.71E-02**
1.02 (0.82, 1.26)	9.95E-01	1.03 (1.01, 1.05)	**5.47E-03**
1.15 (0.78, 1.70)	9.95E-01	1.09 (1.07, 1.11)	**2.57E-15**
0.89 (0.60, 1.33)	9.95E-01	0.92 (0.91, 0.94)	**1.13E-13**
0.89 (0.60, 1.31)	9.95E-01	0.93 (0.91, 0.95)	**3.56E-13**
1.00 (0.64, 1.57)	9.95E-01	0.95 (0.93, 0.97)	**1.09E-05**
1.09 (0.73, 1.63)	9.95E-01	1.07 (1.05, 1.09)	**1.82E-11**
1.05 (0.68, 1.63)	9.95E-01	1.10 (1.08, 1.12)	**4.01E-19**
0.93 (0.62, 1.39)	9.95E-01	0.90 (0.88, 0.92)	**1.01E-25**
0.88 (0.59, 1.31)	9.95E-01	0.90 (0.88, 0.91)	**1.97E-27**
1.23 (0.84, 1.81)	9.95E-01	1.01 (0.99, 1.03)	3.03E-01
1.07 (0.71, 1.63)	9.95E-01	1.08 (1.06, 1.10)	**3.08E-12**
0.93 (0.60, 1.44)	9.95E-01	0.97 (0.95, 0.99)	**8.43E-03**
0.97 (0.63, 1.48)	9.95E-01	0.98 (0.96, 1.00)	**2.52E-02**
1.10 (0.70, 1.73)	9.95E-01	1.04 (1.02, 1.07)	**7.62E-04**
1.10 (0.72, 1.66)	9.95E-01	0.93 (0.91, 0.95)	**7.88E-11**
0.96 (0.63, 1.47)	9.95E-01	0.98 (0.96, 1.00)	9.20E-02
1.02 (0.68, 1.55)	9.95E-01	0.92 (0.90, 0.94)	**3.64E-14**
0.99 (0.65, 1.50)	9.95E-01	0.96 (0.94, 0.98)	**1.83E-04**
1.11 (0.73, 1.68)	9.95E-01	0.93 (0.91, 0.95)	**6.90E-12**
1.06 (0.70, 1.60)	9.95E-01	0.94 (0.92, 0.96)	**4.63E-08**
1.04 (0.69, 1.56)	9.95E-01	1.01 (0.99, 1.03)	5.82E-01
1.01 (0.67, 1.54)	9.95E-01	1.00 (0.98, 1.02)	8.02E-01
1.17 (0.81, 1.69)	9.95E-01	1.04 (1.02, 1.06)	**3.95E-04**
1.10 (0.75, 1.63)	9.95E-01	1.04 (1.02, 1.06)	**5.08E-05**
1.26 (0.83, 1.90)	9.95E-01	0.96 (0.94, 0.99)	**1.68E-03**
1.01 (0.66, 1.56)	9.95E-01	1.03 (1.00, 1.05)	**2.68E-02**

1.21 (0.79, 1.84)	9.95E-01	0.96 (0.94, 0.98)	**1.32E-04**
1.26 (0.84, 1.90)	9.95E-01	0.97 (0.95, 0.99)	**6.95E-03**
1.25 (0.83, 1.91)	9.95E-01	0.96 (0.94, 0.98)	**1.89E-04**
1.31 (0.87, 1.96)	9.95E-01	0.96 (0.94, 0.98)	**3.77E-04**
0.98 (0.65, 1.49)	9.95E-01	0.95 (0.93, 0.97)	**7.99E-06**
0.99 (0.64, 1.53)	9.95E-01	0.98 (0.96, 1.00)	9.53E-02
1.14 (0.77, 1.69)	9.95E-01	0.99 (0.97, 1.01)	2.17E-01
0.84 (0.55, 1.27)	9.95E-01	0.88 (0.86, 0.90)	**4.47E-32**
1.03 (0.68, 1.55)	9.95E-01	0.89 (0.87, 0.91)	**5.11E-22**
1.02 (0.67, 1.55)	9.95E-01	0.94 (0.93, 0.96)	**1.77E-07**
1.06 (0.71, 1.58)	9.95E-01	0.91 (0.89, 0.92)	**1.56E-18**
1.05 (0.69, 1.59)	9.95E-01	0.94 (0.92, 0.96)	**4.82E-08**
1.06 (0.70, 1.61)	9.95E-01	0.92 (0.91, 0.94)	**1.25E-12**
1.16 (0.80, 1.68)	9.95E-01	1.02 (1.00, 1.04)	**4.63E-02**
1.17 (0.81, 1.70)	9.95E-01	1.01 (0.99, 1.03)	5.05E-01
0.91 (0.59, 1.40)	9.95E-01	0.90 (0.88, 0.92)	**5.38E-21**
0.81 (0.54, 1.21)	9.95E-01	0.93 (0.91, 0.95)	**2.02E-11**
0.95 (0.62, 1.45)	9.95E-01	0.89 (0.87, 0.91)	**7.08E-24**
0.80 (0.55, 1.17)	9.95E-01	0.94 (0.92, 0.96)	**3.13E-08**
0.79 (0.54, 1.15)	9.95E-01	0.91 (0.89, 0.92)	**1.55E-21**
1.27 (0.86, 1.88)	9.95E-01	1.09 (1.07, 1.12)	**1.11E-16**
1.17 (0.79, 1.72)	9.95E-01	1.07 (1.05, 1.09)	**3.28E-12**
1.28 (1.05, 1.55)	8.49E-01	1.06 (1.04, 1.08)	**4.20E-12**
1.04 (0.69, 1.56)	9.95E-01	1.03 (1.01, 1.05)	**1.45E-02**
1.33 (0.92, 1.93)	9.95E-01	1.04 (1.02, 1.06)	**4.44E-05**
0.70 (0.45, 1.11)	9.95E-01	0.98 (0.96, 1.00)	1.03E-01
1.19 (0.79, 1.80)	9.95E-01	0.95 (0.93, 0.97)	**4.53E-07**
1.01 (0.66, 1.55)	9.95E-01	0.95 (0.93, 0.97)	**2.24E-05**
1.27 (0.90, 1.79)	9.95E-01	0.99 (0.97, 1.01)	6.28E-01
1.23 (0.84, 1.78)	9.95E-01	0.97 (0.95, 0.99)	**2.85E-03**
1.09 (0.72, 1.65)	9.95E-01	0.97 (0.95, 0.99)	**8.05E-03**
1.25 (0.87, 1.80)	9.95E-01	1.03 (1.02, 1.05)	**3.31E-04**
1.38 (0.98, 1.94)	9.95E-01	1.02 (1.00, 1.04)	7.97E-02
1.13 (1.00, 1.28)	9.95E-01	1.04 (1.03, 1.05)	**2.47E-08**
1.09 (1.03, 1.16)	8.49E-01	1.05 (1.03, 1.06)	**1.29E-09**
1.11 (1.03, 1.21)	8.49E-01	1.05 (1.03, 1.07)	**1.42E-08**
1.10 (0.97, 1.25)	9.95E-01	1.03 (1.01, 1.05)	**6.47E-04**
0.95 (0.63, 1.44)	9.95E-01	0.90 (0.88, 0.92)	**5.50E-23**
1.09 (0.72, 1.65)	9.95E-01	1.14 (1.12, 1.16)	**2.57E-34**
1.02 (0.66, 1.59)	9.95E-01	0.98 (0.96, 1.01)	1.91E-01
1.18 (0.80, 1.75)	9.95E-01	0.97 (0.95, 0.99)	**1.98E-02**
1.01 (0.66, 1.52)	9.95E-01	0.94 (0.92, 0.96)	**5.77E-09**
0.99 (0.66, 1.50)	9.95E-01	0.92 (0.91, 0.94)	**2.08E-13**
1.02 (0.67, 1.55)	9.95E-01	0.92 (0.91, 0.94)	**2.38E-13**
1.30 (0.87, 1.96)	9.95E-01	0.95 (0.93, 0.97)	**3.32E-05**
0.73 (0.47, 1.13)	9.95E-01	0.90 (0.88, 0.92)	**1.02E-20**
1.09 (0.78, 1.51)	9.95E-01	1.07 (1.06, 1.09)	**1.02E-21**
0.96 (0.63, 1.48)	9.95E-01	0.98 (0.97, 1.00)	1.61E-01

1.02 (0.67, 1.54)	9.95E-01	0.92 (0.90, 0.94)	**1.07E-15**
1.36 (0.91, 2.03)	9.95E-01	0.98 (0.96, 1.00)	9.51E-02
0.86 (0.51, 1.48)	9.95E-01	1.03 (1.01, 1.04)	**5.98E-03**
1.12 (1.03, 1.23)	8.49E-01	1.04 (1.02, 1.06)	**2.89E-06**
1.25 (0.83, 1.89)	9.95E-01	0.92 (0.90, 0.94)	**1.44E-12**
1.00 (0.66, 1.52)	9.95E-01	0.99 (0.97, 1.01)	5.05E-01
1.00 (0.66, 1.51)	9.95E-01	0.95 (0.93, 0.97)	**2.97E-07**
1.26 (0.84, 1.90)	9.95E-01	0.93 (0.91, 0.95)	**3.32E-11**
1.19 (0.79, 1.81)	9.95E-01	0.96 (0.94, 0.98)	**1.26E-04**
0.97 (0.63, 1.49)	9.95E-01	0.99 (0.97, 1.01)	5.16E-01
1.00 (0.66, 1.52)	9.95E-01	0.93 (0.91, 0.95)	**1.07E-12**
1.34 (0.89, 2.00)	9.95E-01	0.98 (0.96, 1.00)	1.44E-01
1.12 (0.74, 1.68)	9.95E-01	0.95 (0.93, 0.97)	**8.69E-06**
0.99 (0.65, 1.51)	9.95E-01	0.99 (0.97, 1.01)	4.52E-01
1.03 (0.68, 1.56)	9.95E-01	0.93 (0.91, 0.95)	**3.08E-12**
1.34 (0.90, 2.01)	9.95E-01	0.97 (0.95, 1.00)	**2.71E-02**
0.96 (0.63, 1.46)	9.95E-01	0.98 (0.96, 1.00)	6.51E-02
1.32 (0.88, 1.97)	9.95E-01	0.96 (0.93, 0.98)	**2.71E-04**
0.76 (0.56, 1.03)	9.95E-01	1.05 (1.02, 1.07)	**5.10E-04**

ortality		Other mortality		
Non-white		White		Non-white
**Adjusted HR (95% CI)**	**P value**	**Adjusted HR (95% CI)**	**P value**	**Adjusted HR (95% CI)**
0.97 (0.85, 1.10)	9.97E-01	0.93 (0.90, 0.95)	**1.80E-09**	0.88 (0.76, 1.01)
0.97 (0.85, 1.10)	9.97E-01	0.93 (0.90, 0.95)	**1.49E-09**	0.87 (0.76, 1.00)
0.96 (0.84, 1.09)	9.97E-01	0.93 (0.91, 0.95)	**6.49E-09**	0.87 (0.76, 1.00)
0.97 (0.85, 1.10)	9.97E-01	0.92 (0.90, 0.94)	**1.85E-11**	0.85 (0.74, 0.98)
0.98 (0.86, 1.11)	9.97E-01	0.91 (0.89, 0.94)	**2.01E-12**	0.84 (0.73, 0.97)
0.96 (0.84, 1.09)	9.97E-01	0.92 (0.90, 0.95)	**4.36E-10**	0.86 (0.75, 0.99)
0.97 (0.85, 1.10)	9.97E-01	0.93 (0.91, 0.95)	**6.36E-09**	0.88 (0.77, 1.01)
0.99 (0.87, 1.12)	9.97E-01	0.87 (0.85, 0.89)	**2.76E-26**	0.82 (0.71, 0.95)
0.99 (0.87, 1.12)	9.97E-01	0.87 (0.85, 0.89)	**7.11E-27**	0.82 (0.71, 0.94)
0.99 (0.87, 1.12)	9.97E-01	0.87 (0.85, 0.90)	**1.94E-25**	0.81 (0.71, 0.94)
0.99 (0.87, 1.12)	9.97E-01	0.85 (0.83, 0.87)	**1.80E-35**	0.77 (0.67, 0.89)
0.99 (0.87, 1.13)	9.97E-01	0.84 (0.82, 0.86)	**6.57E-42**	0.76 (0.66, 0.87)
0.98 (0.86, 1.12)	9.97E-01	0.87 (0.85, 0.89)	**2.16E-27**	0.80 (0.70, 0.92)
0.99 (0.87, 1.12)	9.97E-01	0.88 (0.86, 0.90)	**2.06E-23**	0.84 (0.73, 0.96)
0.99 (0.87, 1.13)	9.97E-01	0.85 (0.83, 0.87)	**4.33E-35**	0.79 (0.69, 0.91)
1.00 (0.88, 1.14)	9.97E-01	0.84 (0.82, 0.86)	**8.08E-41**	0.77 (0.66, 0.89)
0.99 (0.87, 1.13)	9.97E-01	0.85 (0.83, 0.88)	**1.58E-33**	0.79 (0.69, 0.92)
0.99 (0.87, 1.13)	9.97E-01	0.84 (0.81, 0.86)	**2.02E-42**	0.77 (0.67, 0.88)
1.00 (0.88, 1.13)	9.97E-01	0.84 (0.81, 0.86)	**6.17E-43**	0.76 (0.66, 0.87)
0.99 (0.87, 1.13)	9.97E-01	0.84 (0.82, 0.86)	**1.06E-39**	0.78 (0.68, 0.90)
1.01 (0.88, 1.14)	9.97E-01	0.84 (0.82, 0.86)	**1.21E-40**	0.76 (0.66, 0.88)
0.99 (0.87, 1.12)	9.97E-01	0.83 (0.81, 0.85)	**8.52E-49**	0.73 (0.64, 0.84)
0.99 (0.87, 1.12)	9.97E-01	0.82 (0.80, 0.84)	**4.33E-53**	0.73 (0.63, 0.83)
0.98 (0.87, 1.12)	9.97E-01	0.83 (0.81, 0.86)	**1.57E-45**	0.74 (0.64, 0.85)
0.98 (0.87, 1.11)	9.97E-01	0.86 (0.84, 0.88)	**8.76E-35**	0.75 (0.66, 0.86)
0.97 (0.86, 1.10)	9.97E-01	0.88 (0.86, 0.90)	**8.98E-26**	0.77 (0.68, 0.88)
0.99 (0.87, 1.12)	9.97E-01	0.84 (0.82, 0.86)	**4.41E-43**	0.75 (0.65, 0.86)
1.00 (0.88, 1.13)	9.97E-01	0.83 (0.81, 0.85)	**1.17E-46**	0.75 (0.65, 0.87)
1.00 (0.88, 1.13)	9.97E-01	0.87 (0.85, 0.89)	**1.51E-28**	0.83 (0.72, 0.95)
0.99 (0.87, 1.13)	9.97E-01	0.86 (0.84, 0.88)	**4.14E-31**	0.81 (0.71, 0.93)
0.99 (0.87, 1.12)	9.97E-01	0.85 (0.83, 0.87)	**8.02E-37**	0.78 (0.68, 0.90)
0.99 (0.87, 1.12)	9.97E-01	0.86 (0.84, 0.89)	**2.27E-30**	0.81 (0.71, 0.93)
0.99 (0.87, 1.12)	9.97E-01	0.87 (0.85, 0.90)	**5.97E-26**	0.84 (0.73, 0.95)
0.99 (0.87, 1.12)	9.97E-01	0.85 (0.83, 0.87)	**1.45E-37**	0.78 (0.68, 0.89)
1.00 (0.88, 1.13)	9.97E-01	0.89 (0.87, 0.92)	**7.14E-19**	0.86 (0.75, 0.99)
0.99 (0.88, 1.13)	9.97E-01	0.97 (0.95, 1.00)	**3.77E-02**	0.95 (0.84, 1.08)
1.00 (0.88, 1.13)	9.97E-01	0.98 (0.96, 1.01)	1.47E-01	0.95 (0.84, 1.08)
1.00 (0.88, 1.13)	9.97E-01	1.00 (0.98, 1.02)	9.17E-01	0.98 (0.87, 1.11)
1.00 (0.88, 1.13)	9.97E-01	0.97 (0.95, 0.99)	**1.98E-02**	0.92 (0.81, 1.04)
0.99 (0.88, 1.12)	9.97E-01	0.97 (0.94, 0.99)	**1.08E-02**	0.90 (0.79, 1.02)

1.00 (0.89, 1.14)	9.97E-01	0.98 (0.96, 1.00)	8.81E-02	0.96 (0.84, 1.09)
0.99 (0.88, 1.13)	9.97E-01	0.99 (0.97, 1.02)	5.92E-01	1.02 (0.90, 1.16)
0.97 (0.86, 1.10)	9.97E-01	0.91 (0.88, 0.93)	**1.51E-15**	0.82 (0.72, 0.94)
0.97 (0.86, 1.10)	9.97E-01	0.92 (0.90, 0.94)	**2.28E-11**	0.83 (0.73, 0.95)
0.98 (0.86, 1.11)	9.97E-01	0.93 (0.91, 0.96)	**2.52E-08**	0.85 (0.74, 0.96)
0.97 (0.85, 1.10)	9.97E-01	0.91 (0.89, 0.93)	**2.74E-14**	0.82 (0.72, 0.93)
0.96 (0.85, 1.09)	9.97E-01	0.90 (0.88, 0.93)	**1.01E-15**	0.80 (0.70, 0.91)
0.98 (0.87, 1.12)	9.97E-01	0.92 (0.90, 0.95)	**3.84E-10**	0.87 (0.77, 0.99)
0.99 (0.87, 1.12)	9.97E-01	1.03 (1.01, 1.05)	**1.66E-02**	1.06 (0.94, 1.20)
0.96 (0.85, 1.09)	9.97E-01	0.86 (0.84, 0.88)	**9.46E-33**	0.77 (0.68, 0.88)
0.97 (0.86, 1.10)	9.97E-01	0.85 (0.83, 0.87)	**8.58E-38**	0.77 (0.67, 0.88)
0.97 (0.85, 1.10)	9.97E-01	0.85 (0.83, 0.87)	**8.54E-39**	0.77 (0.68, 0.88)
0.97 (0.86, 1.10)	9.97E-01	0.84 (0.82, 0.86)	**3.13E-41**	0.76 (0.66, 0.86)
0.97 (0.86, 1.10)	9.97E-01	0.84 (0.82, 0.86)	**1.13E-44**	0.75 (0.66, 0.85)
0.97 (0.86, 1.10)	9.97E-01	0.87 (0.84, 0.89)	**1.05E-30**	0.78 (0.69, 0.89)
0.98 (0.86, 1.11)	9.97E-01	1.02 (0.99, 1.04)	2.14E-01	1.05 (0.93, 1.19)
1.00 (0.89, 1.13)	9.97E-01	0.83 (0.81, 0.85)	**2.13E-47**	0.75 (0.65, 0.86)
0.97 (0.85, 1.10)	9.97E-01	0.81 (0.79, 0.83)	**3.05E-60**	0.74 (0.65, 0.84)
0.96 (0.85, 1.09)	9.97E-01	0.81 (0.79, 0.83)	**6.83E-61**	0.73 (0.64, 0.84)
0.97 (0.86, 1.10)	9.97E-01	0.81 (0.79, 0.83)	**2.69E-64**	0.73 (0.64, 0.83)
0.97 (0.86, 1.10)	9.97E-01	0.81 (0.79, 0.83)	**9.29E-65**	0.73 (0.63, 0.83)
0.97 (0.86, 1.10)	9.97E-01	0.82 (0.80, 0.84)	**1.37E-54**	0.75 (0.66, 0.85)
0.97 (0.86, 1.11)	9.97E-01	0.97 (0.95, 0.99)	**1.40E-02**	0.98 (0.87, 1.11)
1.00 (0.88, 1.13)	9.97E-01	0.85 (0.83, 0.88)	**2.36E-36**	0.76 (0.66, 0.87)
0.96 (0.85, 1.09)	9.97E-01	0.83 (0.81, 0.85)	**8.08E-48**	0.74 (0.65, 0.85)
0.97 (0.85, 1.09)	9.97E-01	0.86 (0.84, 0.88)	**5.31E-35**	0.77 (0.68, 0.88)
0.97 (0.85, 1.09)	9.97E-01	0.82 (0.80, 0.84)	**6.69E-55**	0.73 (0.64, 0.83)
0.96 (0.85, 1.09)	9.97E-01	0.82 (0.80, 0.84)	**4.96E-54**	0.72 (0.63, 0.83)
0.98 (0.87, 1.11)	9.97E-01	0.84 (0.82, 0.86)	**5.03E-46**	0.77 (0.68, 0.87)
0.97 (0.85, 1.10)	9.97E-01	0.94 (0.92, 0.96)	**5.94E-07**	0.91 (0.79, 1.04)
1.00 (0.87, 1.14)	9.97E-01	1.21 (1.18, 1.24)	**2.40E-51**	1.11 (0.98, 1.27)
1.00 (0.88, 1.15)	9.97E-01	1.23 (1.20, 1.26)	**2.58E-62**	1.14 (1.01, 1.30)
1.00 (0.87, 1.15)	9.97E-01	1.24 (1.21, 1.27)	**3.60E-70**	1.17 (1.03, 1.33)
1.00 (0.88, 1.15)	9.97E-01	1.21 (1.18, 1.24)	**7.38E-51**	1.12 (0.98, 1.27)
1.00 (0.87, 1.14)	9.97E-01	1.20 (1.17, 1.23)	**5.03E-46**	1.10 (0.96, 1.26)
1.03 (0.91, 1.17)	9.97E-01	1.22 (1.20, 1.25)	**5.41E-65**	1.16 (1.03, 1.32)
1.00 (0.88, 1.13)	9.97E-01	1.05 (1.02, 1.07)	**5.38E-05**	1.00 (0.89, 1.14)
0.97 (0.84, 1.12)	9.97E-01	1.19 (1.16, 1.22)	**4.42E-39**	1.10 (0.96, 1.26)
0.97 (0.84, 1.12)	9.97E-01	1.20 (1.17, 1.23)	**2.44E-42**	1.12 (0.98, 1.28)
0.97 (0.84, 1.12)	9.97E-01	1.20 (1.17, 1.23)	**1.04E-42**	1.13 (0.99, 1.30)
0.97 (0.84, 1.12)	9.97E-01	1.19 (1.16, 1.22)	**3.01E-39**	1.10 (0.96, 1.26)
0.97 (0.84, 1.12)	9.97E-01	1.18 (1.15, 1.21)	**3.79E-37**	1.10 (0.96, 1.26)
0.98 (0.85, 1.12)	9.97E-01	1.21 (1.17, 1.24)	**5.03E-46**	1.12 (0.98, 1.29)
1.00 (0.88, 1.13)	9.97E-01	1.10 (1.07, 1.12)	**2.76E-15**	1.08 (0.95, 1.21)
0.93 (0.81, 1.06)	9.97E-01	1.02 (0.99, 1.05)	1.39E-01	0.97 (0.84, 1.11)
0.93 (0.81, 1.06)	9.97E-01	1.02 (0.99, 1.04)	1.86E-01	0.97 (0.85, 1.12)
0.93 (0.81, 1.06)	9.97E-01	1.02 (0.99, 1.04)	2.21E-01	0.98 (0.85, 1.12)
0.93 (0.81, 1.06)	9.97E-01	1.02 (1.00, 1.05)	8.78E-02	0.97 (0.84, 1.12)

0.93 (0.81, 1.06)	9.97E-01	1.02 (0.99, 1.05)	1.45E-01	0.97 (0.84, 1.11)
0.94 (0.81, 1.08)	9.97E-01	1.04 (1.01, 1.07)	**7.02E-03**	0.98 (0.85, 1.13)
0.98 (0.87, 1.11)	9.97E-01	0.98 (0.96, 1.00)	1.20E-01	0.99 (0.87, 1.12)
0.92 (0.81, 1.05)	9.97E-01	0.80 (0.78, 0.82)	**8.65E-66**	0.77 (0.67, 0.88)
0.93 (0.82, 1.05)	9.97E-01	0.84 (0.82, 0.87)	**1.20E-40**	0.81 (0.71, 0.93)
0.93 (0.82, 1.06)	9.97E-01	0.87 (0.85, 0.89)	**2.09E-27**	0.84 (0.74, 0.96)
0.93 (0.81, 1.05)	9.97E-01	0.81 (0.79, 0.83)	**1.18E-58**	0.78 (0.68, 0.89)
0.92 (0.81, 1.05)	9.97E-01	0.80 (0.78, 0.82)	**6.47E-68**	0.77 (0.67, 0.87)
0.94 (0.83, 1.07)	9.97E-01	0.88 (0.86, 0.90)	**2.35E-24**	0.85 (0.74, 0.97)
0.99 (0.87, 1.12)	9.97E-01	0.92 (0.90, 0.94)	**4.21E-11**	0.94 (0.83, 1.07)
0.90 (0.79, 1.03)	9.97E-01	1.02 (0.99, 1.05)	2.50E-01	1.11 (0.96, 1.29)
1.04 (0.92, 1.19)	9.97E-01	1.03 (1.01, 1.06)	**1.08E-02**	1.18 (1.04, 1.33)
1.09 (0.97, 1.23)	9.97E-01	1.03 (1.00, 1.05)	5.71E-02	1.16 (1.03, 1.31)
0.91 (0.79, 1.05)	9.97E-01	1.04 (1.02, 1.07)	**1.33E-03**	1.15 (1.03, 1.29)
0.99 (0.87, 1.13)	9.97E-01	0.97 (0.94, 0.99)	**8.51E-03**	0.84 (0.74, 0.95)
1.05 (0.93, 1.20)	9.97E-01	1.07 (1.05, 1.10)	**5.77E-08**	1.15 (1.02, 1.30)
1.05 (0.93, 1.19)	9.97E-01	1.06 (1.03, 1.09)	**2.59E-05**	1.13 (1.00, 1.28)
1.05 (0.92, 1.20)	9.97E-01	1.12 (1.09, 1.15)	**1.74E-18**	1.18 (1.04, 1.35)
0.94 (0.83, 1.07)	9.97E-01	0.93 (0.91, 0.96)	**2.07E-07**	0.87 (0.76, 0.98)
0.97 (0.85, 1.10)	9.97E-01	0.95 (0.93, 0.97)	**1.29E-04**	1.00 (0.87, 1.15)
1.04 (0.92, 1.18)	9.97E-01	1.08 (1.06, 1.11)	**6.78E-11**	1.27 (1.14, 1.43)
1.04 (0.91, 1.18)	9.97E-01	1.10 (1.07, 1.13)	**1.37E-14**	1.24 (1.10, 1.40)
1.04 (0.91, 1.19)	9.97E-01	1.02 (1.00, 1.05)	1.08E-01	1.33 (1.17, 1.51)
0.99 (0.87, 1.12)	9.97E-01	0.96 (0.94, 0.99)	**4.22E-03**	0.81 (0.70, 0.93)
0.99 (0.87, 1.12)	9.97E-01	1.02 (1.00, 1.05)	7.05E-02	1.03 (0.91, 1.17)
0.99 (0.87, 1.12)	9.97E-01	1.04 (1.01, 1.06)	**2.16E-03**	1.04 (0.92, 1.18)
0.98 (0.86, 1.11)	9.97E-01	1.04 (1.02, 1.07)	**1.05E-03**	1.04 (0.91, 1.18)
1.00 (0.89, 1.14)	9.97E-01	1.03 (1.01, 1.05)	**1.73E-02**	1.06 (0.94, 1.21)
1.01 (0.89, 1.15)	9.97E-01	0.97 (0.94, 0.99)	**4.65E-03**	0.96 (0.85, 1.09)
0.98 (0.86, 1.11)	9.97E-01	0.95 (0.93, 0.97)	**6.78E-05**	0.92 (0.81, 1.04)
0.99 (0.87, 1.12)	9.97E-01	0.98 (0.96, 1.01)	1.42E-01	1.01 (0.89, 1.15)
1.01 (0.89, 1.14)	9.97E-01	1.00 (0.98, 1.03)	7.12E-01	1.08 (0.95, 1.22)
0.97 (0.86, 1.11)	9.97E-01	0.95 (0.93, 0.98)	**1.51E-04**	0.93 (0.82, 1.06)
1.01 (0.89, 1.15)	9.97E-01	1.03 (1.00, 1.05)	**2.49E-02**	1.02 (0.90, 1.15)
1.02 (0.89, 1.16)	9.97E-01	1.14 (1.12, 1.17)	**4.58E-28**	1.23 (1.07, 1.41)
0.98 (0.86, 1.11)	9.97E-01	0.97 (0.95, 0.99)	**1.12E-02**	0.90 (0.80, 1.02)
0.97 (0.85, 1.10)	9.97E-01	0.97 (0.95, 0.99)	**1.04E-02**	0.89 (0.79, 1.01)
1.03 (0.91, 1.16)	9.97E-01	0.98 (0.96, 1.00)	7.64E-02	1.02 (0.90, 1.14)
1.02 (0.90, 1.16)	9.97E-01	1.01 (0.98, 1.03)	5.86E-01	1.07 (0.95, 1.21)
1.05 (0.93, 1.19)	9.97E-01	1.12 (1.10, 1.15)	**2.15E-22**	1.14 (1.01, 1.29)
0.97 (0.86, 1.10)	9.97E-01	0.90 (0.88, 0.92)	**4.04E-24**	0.85 (0.76, 0.94)
0.95 (0.84, 1.07)	9.97E-01	0.88 (0.86, 0.89)	**7.52E-34**	0.78 (0.70, 0.86)
1.04 (0.92, 1.17)	9.97E-01	0.99 (0.97, 1.01)	3.13E-01	1.12 (0.99, 1.26)
1.02 (0.90, 1.15)	9.97E-01	1.09 (1.07, 1.12)	**7.58E-18**	1.16 (1.05, 1.27)
0.98 (0.86, 1.11)	9.97E-01	1.03 (1.01, 1.05)	**1.16E-02**	1.12 (1.04, 1.21)
1.01 (0.89, 1.15)	9.97E-01	0.92 (0.91, 0.93)	**2.47E-56**	0.88 (0.84, 0.93)
1.00 (0.89, 1.13)	9.97E-01	0.91 (0.91, 0.92)	**4.14E-99**	0.88 (0.85, 0.91)
1.01 (0.89, 1.15)	9.97E-01	0.99 (0.97, 1.01)	3.78E-01	0.95 (0.85, 1.06)

1.00 (0.88, 1.12)	9.97E-01	1.11 (1.10, 1.13)	**1.03E-55**	1.16 (1.09, 1.23)
0.96 (0.85, 1.09)	9.97E-01	1.05 (1.03, 1.07)	**1.08E-05**	1.11 (1.00, 1.25)
1.02 (0.90, 1.16)	9.97E-01	0.91 (0.90, 0.92)	**2.29E-79**	0.89 (0.86, 0.93)
1.02 (0.89, 1.15)	9.97E-01	0.87 (0.85, 0.88)	**2.05E-83**	0.83 (0.78, 0.88)
1.01 (0.89, 1.15)	9.97E-01	1.00 (0.98, 1.02)	9.56E-01	0.98 (0.87, 1.11)
0.99 (0.88, 1.12)	9.97E-01	1.11 (1.10, 1.12)	**3.71E-83**	1.15 (1.09, 1.21)
0.98 (0.87, 1.11)	9.97E-01	1.16 (1.14, 1.18)	**7.86E-61**	1.23 (1.08, 1.38)
1.02 (0.90, 1.16)	9.97E-01	0.88 (0.87, 0.89)	**4.45E-96**	0.82 (0.77, 0.89)
1.00 (0.88, 1.13)	9.97E-01	0.87 (0.85, 0.88)	**4.33E-53**	0.77 (0.69, 0.87)
1.02 (0.90, 1.16)	9.97E-01	0.95 (0.93, 0.97)	**6.40E-06**	0.97 (0.87, 1.09)
0.99 (0.88, 1.13)	9.97E-01	1.07 (1.05, 1.09)	**1.43E-11**	1.09 (1.01, 1.18)
0.99 (0.87, 1.12)	9.97E-01	1.22 (1.19, 1.26)	**1.04E-42**	1.14 (0.99, 1.31)
1.02 (0.90, 1.16)	9.97E-01	0.84 (0.82, 0.86)	**1.66E-47**	0.91 (0.81, 1.03)
1.00 (0.89, 1.12)	9.97E-01	0.91 (0.90, 0.93)	**2.19E-20**	0.91 (0.82, 1.01)
1.03 (0.92, 1.16)	9.97E-01	0.87 (0.85, 0.90)	**2.76E-23**	0.98 (0.87, 1.11)
0.99 (0.88, 1.11)	9.97E-01	0.94 (0.92, 0.96)	**8.68E-07**	0.95 (0.85, 1.07)
1.01 (0.90, 1.13)	9.97E-01	0.95 (0.93, 0.97)	**5.72E-06**	1.09 (0.97, 1.21)
0.99 (0.88, 1.12)	9.97E-01	1.06 (1.04, 1.08)	**8.68E-07**	0.95 (0.85, 1.05)
0.99 (0.88, 1.12)	9.97E-01	1.04 (1.02, 1.07)	**4.74E-04**	0.94 (0.84, 1.04)
0.99 (0.88, 1.12)	9.97E-01	1.12 (1.09, 1.14)	**1.49E-18**	1.00 (0.89, 1.11)
1.00 (0.90, 1.12)	9.97E-01	0.95 (0.93, 0.97)	**3.65E-05**	1.01 (0.93, 1.10)
1.01 (0.89, 1.14)	9.97E-01	1.01 (0.98, 1.03)	6.58E-01	1.10 (0.97, 1.24)
0.99 (0.87, 1.12)	9.97E-01	1.01 (0.98, 1.03)	5.18E-01	0.93 (0.82, 1.05)
0.99 (0.87, 1.12)	9.97E-01	1.00 (0.98, 1.02)	9.16E-01	0.94 (0.84, 1.06)
0.99 (0.86, 1.13)	9.97E-01	1.05 (1.02, 1.08)	**3.39E-04**	0.92 (0.80, 1.05)
1.02 (0.89, 1.15)	9.97E-01	0.98 (0.96, 1.01)	1.66E-01	1.05 (0.93, 1.19)
0.99 (0.87, 1.12)	9.97E-01	1.20 (1.17, 1.23)	**1.45E-50**	1.18 (1.04, 1.34)
1.00 (0.88, 1.13)	9.97E-01	0.90 (0.88, 0.92)	**7.55E-21**	0.87 (0.78, 0.98)
0.98 (0.87, 1.12)	9.97E-01	0.87 (0.85, 0.89)	**5.73E-34**	0.85 (0.76, 0.95)
1.05 (0.93, 1.19)	9.97E-01	1.13 (1.10, 1.15)	**2.82E-24**	1.14 (1.01, 1.29)
1.02 (0.89, 1.16)	9.97E-01	1.00 (0.97, 1.02)	8.96E-01	1.07 (0.94, 1.21)
0.99 (0.87, 1.13)	9.97E-01	0.82 (0.80, 0.84)	**3.52E-57**	0.75 (0.66, 0.86)
0.91 (0.80, 1.03)	9.97E-01	1.01 (0.99, 1.04)	2.56E-01	1.01 (0.89, 1.14)
1.00 (0.87, 1.15)	9.97E-01	1.27 (1.24, 1.31)	**5.30E-79**	1.18 (1.03, 1.35)
0.96 (0.84, 1.09)	9.97E-01	0.88 (0.86, 0.90)	**2.16E-24**	0.78 (0.68, 0.89)
0.98 (0.87, 1.11)	9.97E-01	0.87 (0.84, 0.89)	**8.02E-30**	0.79 (0.69, 0.90)
0.97 (0.86, 1.10)	9.97E-01	0.83 (0.81, 0.85)	**2.23E-49**	0.74 (0.65, 0.85)
0.98 (0.86, 1.10)	9.97E-01	0.88 (0.86, 0.90)	**6.07E-26**	0.79 (0.69, 0.90)
0.96 (0.84, 1.08)	9.97E-01	0.88 (0.86, 0.90)	**9.16E-24**	0.78 (0.68, 0.89)
0.96 (0.85, 1.09)	9.97E-01	0.88 (0.86, 0.90)	**3.92E-25**	0.78 (0.68, 0.89)
0.99 (0.87, 1.12)	9.97E-01	0.89 (0.87, 0.92)	**2.52E-18**	0.85 (0.74, 0.98)
0.99 (0.87, 1.13)	9.97E-01	0.87 (0.85, 0.90)	**1.43E-25**	0.82 (0.71, 0.94)
0.98 (0.86, 1.11)	9.97E-01	0.99 (0.97, 1.02)	5.93E-01	1.01 (0.90, 1.15)
0.99 (0.87, 1.12)	9.97E-01	0.99 (0.97, 1.01)	3.91E-01	0.99 (0.88, 1.13)
0.94 (0.83, 1.07)	9.97E-01	0.94 (0.92, 0.97)	**6.13E-06**	0.87 (0.77, 1.00)
1.01 (0.89, 1.15)	9.97E-01	0.90 (0.88, 0.92)	**1.07E-15**	0.88 (0.77, 1.01)

0.92 (0.81, 1.05)	9.97E-01	0.94 (0.92, 0.97)	**5.59E-06**	0.85 (0.74, 0.97)
1.02 (0.90, 1.16)	9.97E-01	0.97 (0.95, 1.00)	5.43E-02	0.93 (0.81, 1.06)
0.95 (0.83, 1.08)	9.97E-01	0.94 (0.92, 0.96)	**1.40E-06**	0.85 (0.74, 0.97)
0.93 (0.81, 1.07)	9.97E-01	1.02 (1.00, 1.05)	1.20E-01	0.96 (0.83, 1.10)
0.98 (0.86, 1.11)	9.97E-01	0.86 (0.84, 0.88)	**6.91E-34**	0.77 (0.68, 0.88)
1.01 (0.89, 1.15)	9.97E-01	0.88 (0.86, 0.90)	**6.14E-25**	0.83 (0.72, 0.95)
0.97 (0.86, 1.10)	9.97E-01	0.91 (0.89, 0.93)	**7.74E-15**	0.85 (0.75, 0.97)
0.97 (0.85, 1.10)	9.97E-01	0.85 (0.83, 0.87)	**3.14E-38**	0.90 (0.80, 1.03)
0.97 (0.85, 1.10)	9.97E-01	0.85 (0.83, 0.88)	**2.71E-29**	0.97 (0.85, 1.11)
0.95 (0.84, 1.08)	9.97E-01	0.86 (0.84, 0.88)	**1.69E-31**	0.70 (0.60, 0.80)
0.96 (0.84, 1.09)	9.97E-01	0.82 (0.80, 0.84)	**6.94E-45**	0.94 (0.83, 1.07)
0.95 (0.84, 1.08)	9.97E-01	0.86 (0.84, 0.88)	**8.49E-34**	0.72 (0.63, 0.83)
0.95 (0.84, 1.08)	9.97E-01	0.83 (0.81, 0.85)	**9.18E-46**	0.74 (0.64, 0.85)
0.98 (0.87, 1.11)	9.97E-01	0.95 (0.93, 0.98)	**7.63E-05**	0.91 (0.80, 1.04)
0.99 (0.88, 1.12)	9.97E-01	0.95 (0.92, 0.97)	**4.03E-06**	0.93 (0.82, 1.05)
0.98 (0.87, 1.12)	9.97E-01	0.91 (0.89, 0.93)	**3.52E-13**	1.07 (0.94, 1.20)
0.94 (0.83, 1.07)	9.97E-01	0.93 (0.91, 0.95)	**3.50E-09**	0.73 (0.65, 0.82)
0.98 (0.86, 1.11)	9.97E-01	0.83 (0.81, 0.86)	**3.40E-39**	1.03 (0.91, 1.16)
0.96 (0.85, 1.08)	9.97E-01	0.98 (0.96, 1.00)	7.99E-02	0.85 (0.76, 0.96)
0.95 (0.84, 1.08)	9.97E-01	0.92 (0.90, 0.94)	**6.54E-12**	0.87 (0.77, 0.97)
1.03 (0.91, 1.17)	9.97E-01	1.05 (1.02, 1.07)	**3.82E-04**	1.06 (0.94, 1.20)
1.06 (0.93, 1.20)	9.97E-01	1.10 (1.07, 1.12)	**1.69E-16**	1.20 (1.07, 1.35)
0.93 (0.82, 1.07)	9.97E-01	1.10 (1.08, 1.11)	**4.40E-30**	1.19 (1.12, 1.28)
0.99 (0.87, 1.13)	9.97E-01	1.06 (1.03, 1.08)	**3.81E-06**	1.03 (0.90, 1.16)
0.98 (0.86, 1.11)	9.97E-01	1.09 (1.07, 1.11)	**2.69E-19**	1.15 (1.02, 1.30)
0.88 (0.77, 1.00)	9.97E-01	0.98 (0.96, 1.00)	7.03E-02	0.92 (0.81, 1.05)
0.89 (0.78, 1.01)	9.97E-01	0.96 (0.94, 0.98)	**7.95E-04**	0.82 (0.72, 0.93)
0.89 (0.78, 1.03)	9.97E-01	0.87 (0.85, 0.90)	**9.94E-23**	0.75 (0.65, 0.86)
0.95 (0.83, 1.08)	9.97E-01	0.92 (0.90, 0.94)	**1.63E-10**	0.93 (0.82, 1.07)
0.91 (0.80, 1.05)	9.97E-01	0.85 (0.82, 0.87)	**2.64E-35**	0.79 (0.69, 0.92)
0.91 (0.80, 1.04)	9.97E-01	0.84 (0.82, 0.86)	**3.48E-40**	0.78 (0.67, 0.89)
1.01 (0.89, 1.14)	9.97E-01	1.04 (1.02, 1.06)	**9.13E-06**	0.97 (0.86, 1.10)
0.98 (0.86, 1.11)	9.97E-01	1.03 (1.01, 1.05)	**1.77E-02**	0.97 (0.86, 1.10)
1.04 (0.95, 1.13)	9.97E-01	1.09 (1.08, 1.10)	**5.27E-98**	1.11 (1.06, 1.15)
1.04 (0.99, 1.10)	9.97E-01	1.07 (1.06, 1.09)	**8.80E-32**	1.04 (0.99, 1.09)
1.06 (0.99, 1.12)	9.97E-01	1.10 (1.08, 1.11)	**7.10E-43**	1.06 (1.01, 1.11)
0.93 (0.76, 1.13)	9.97E-01	1.04 (1.03, 1.06)	**4.66E-11**	1.14 (1.11, 1.17)
0.90 (0.79, 1.02)	9.97E-01	0.80 (0.78, 0.82)	**4.93E-77**	0.70 (0.62, 0.80)
1.19 (1.05, 1.34)	9.97E-01	1.09 (1.06, 1.11)	**2.28E-11**	1.17 (1.04, 1.32)
0.96 (0.84, 1.10)	9.97E-01	1.03 (1.00, 1.06)	**4.44E-02**	1.01 (0.88, 1.16)
0.92 (0.80, 1.05)	9.97E-01	0.85 (0.83, 0.88)	**1.04E-32**	0.80 (0.69, 0.93)
0.97 (0.86, 1.10)	9.97E-01	0.85 (0.83, 0.87)	**2.42E-38**	0.76 (0.66, 0.87)
0.97 (0.86, 1.10)	9.97E-01	0.84 (0.82, 0.86)	**8.89E-43**	0.76 (0.67, 0.86)
0.97 (0.86, 1.10)	9.97E-01	0.82 (0.80, 0.85)	**2.34E-52**	0.74 (0.65, 0.84)
0.94 (0.81, 1.08)	9.97E-01	1.07 (1.04, 1.09)	**3.89E-06**	0.98 (0.85, 1.13)
0.95 (0.84, 1.08)	9.97E-01	0.94 (0.92, 0.96)	**2.37E-06**	0.91 (0.80, 1.03)
1.08 (0.98, 1.19)	9.97E-01	1.11 (1.10, 1.13)	**1.18E-77**	1.02 (0.90, 1.15)
0.98 (0.87, 1.12)	9.97E-01	0.86 (0.84, 0.88)	**8.49E-33**	0.79 (0.69, 0.90)

0.97 (0.86, 1.10)	9.97E-01	0.85 (0.83, 0.87)	**1.15E-38**	0.77 (0.68, 0.88)
0.95 (0.83, 1.09)	9.97E-01	1.10 (1.07, 1.13)	**6.23E-13**	1.02 (0.89, 1.18)
0.95 (0.82, 1.10)	9.97E-01	1.06 (1.04, 1.07)	**1.16E-13**	1.02 (0.92, 1.12)
0.99 (0.88, 1.12)	9.97E-01	1.09 (1.08, 1.10)	**2.20E-75**	1.07 (1.03, 1.12)
0.92 (0.80, 1.05)	9.97E-01	0.91 (0.89, 0.94)	**1.02E-11**	0.83 (0.73, 0.96)
0.99 (0.87, 1.12)	9.97E-01	0.88 (0.86, 0.90)	**1.30E-24**	0.82 (0.72, 0.94)
0.98 (0.86, 1.11)	9.97E-01	0.85 (0.83, 0.87)	**1.77E-38**	0.76 (0.67, 0.87)
0.92 (0.80, 1.05)	9.97E-01	0.93 (0.91, 0.96)	**5.02E-07**	0.87 (0.75, 1.00)
0.94 (0.83, 1.07)	9.97E-01	0.92 (0.90, 0.95)	**3.63E-10**	0.83 (0.72, 0.94)
0.98 (0.87, 1.12)	9.97E-01	0.87 (0.85, 0.89)	**3.75E-28**	0.80 (0.70, 0.92)
0.97 (0.85, 1.09)	9.97E-01	0.84 (0.82, 0.86)	**9.98E-46**	0.76 (0.66, 0.86)
0.94 (0.81, 1.08)	9.97E-01	1.07 (1.05, 1.10)	**1.35E-07**	1.01 (0.88, 1.16)
0.96 (0.84, 1.09)	9.97E-01	0.88 (0.86, 0.90)	**6.07E-26**	0.78 (0.69, 0.89)
0.99 (0.87, 1.12)	9.97E-01	0.86 (0.84, 0.88)	**2.07E-30**	0.79 (0.69, 0.91)
0.97 (0.86, 1.10)	9.97E-01	0.84 (0.82, 0.86)	**1.45E-45**	0.76 (0.66, 0.86)
0.94 (0.81, 1.08)	9.97E-01	1.07 (1.05, 1.10)	**1.91E-07**	1.00 (0.87, 1.15)
0.98 (0.87, 1.11)	9.97E-01	0.87 (0.85, 0.90)	**3.65E-26**	0.79 (0.69, 0.90)
0.94 (0.82, 1.08)	9.97E-01	1.08 (1.05, 1.10)	**1.65E-07**	0.99 (0.86, 1.14)
1.03 (0.90, 1.18)	9.97E-01	1.00 (0.97, 1.03)	8.68E-01	1.00 (0.87, 1.15)

P value
1.00E-01
8.79E-02
8.84E-02
**4.46E-02**
**3.45E-02**
6.41E-02
1.13E-01
**1.48E-02**
**1.19E-02**
**1.04E-02**
**1.37E-03**
**4.43E-04**
**6.24E-03**
**2.78E-02**
**3.91E-03**
**1.23E-03**
**4.54E-03**
**9.22E-04**
**5.28E-04**
**2.03E-03**
**1.20E-03**
**9.56E-05**
**7.60E-05**
**1.21E-04**
**1.96E-04**
**4.62E-04**
**1.78E-04**
**5.28E-04**
**1.32E-02**
**6.70E-03**
**1.29E-03**
**6.24E-03**
**1.77E-02**
**8.72E-04**
5.35E-02
5.05E-01
5.74E-01
8.46E-01
2.47E-01
1.58E-01

5.93E-01
8.03E-01
8.47E-03
1.49E-02
2.40E-02
6.70E-03
2.82E-03
6.29E-02
4.35E-01
5.28E-04
4.16E-04
4.62E-04
1.95E-04
1.36E-04
7.73E-04
5.15E-01
1.71E-04
7.22E-05
6.40E-05
3.80E-05
3.80E-05
1.21E-04
8.42E-01
2.64E-04
8.65E-05
3.78E-04
3.80E-05
3.42E-05
2.56E-04
2.17E-01
1.67E-01
6.99E-02
3.48E-02
1.54E-01
2.25E-01
3.43E-02
9.68E-01
2.49E-01
1.55E-01
1.13E-01
2.34E-01
2.66E-01
1.42E-01
3.26E-01
7.51E-01
7.89E-01
8.26E-01
7.69E-01

7.58E-01
8.42E-01
8.92E-01
4.16E-04
6.43E-03
2.33E-02
8.08E-04
3.93E-04
2.92E-02
4.66E-01
2.39E-01
1.78E-02
3.39E-02
2.96E-02
1.40E-02
4.86E-02
8.95E-02
2.03E-02
4.86E-02
9.79E-01
2.25E-04
1.20E-03
1.45E-04
9.22E-03
6.92E-01
6.07E-01
6.92E-01
4.21E-01
6.15E-01
2.60E-01
8.80E-01
3.16E-01
3.47E-01
8.49E-01
7.59E-03
1.53E-01
1.01E-01
8.52E-01
3.29E-01
5.68E-02
3.63E-03
1.22E-05
1.23E-01
7.21E-03
5.32E-03
7.94E-06
1.46E-08
4.80E-01

6.74E-05
9.22E-02
2.67E-06
4.48E-07
8.42E-01
7.94E-06
3.51E-03
8.91E-06
1.45E-04
7.03E-01
5.29E-02
1.13E-01
1.87E-01
1.03E-01
8.26E-01
5.00E-01
2.02E-01
3.98E-01
3.16E-01
9.74E-01
8.92E-01
2.03E-01
3.39E-01
4.29E-01
2.93E-01
5.01E-01
2.03E-02
4.82E-02
1.42E-02
5.93E-02
4.21E-01
1.78E-04
9.27E-01
2.92E-02
6.66E-04
1.54E-03
9.47E-05
1.20E-03
7.45E-04
6.61E-04

4.44E-02
1.24E-02
8.66E-01
9.44E-01
8.39E-02
1.08E-01

3.67E-02
3.39E-01
3.55E-02
6.21E-01
5.28E-04
1.31E-02
3.15E-02
1.75E-01
7.61E-01
8.85E-06
4.61E-01
4.07E-05
1.14E-04
2.47E-01
3.26E-01
3.93E-01
8.85E-06
7.69E-01
1.77E-02
3.15E-02
4.27E-01
5.66E-03
7.94E-06
7.88E-01
4.31E-02
2.84E-01
5.76E-03
2.58E-04
4.16E-01
5.32E-03
1.40E-03
7.51E-01
7.69E-01
1.22E-05
1.48E-01
2.56E-02
2.76E-17
1.86E-06
2.47E-02
8.92E-01
6.83E-03
2.39E-04
2.13E-04
6.74E-05
8.48E-01
2.09E-01
8.52E-01
1.84E-03

**3.81E-04**
8.26E-01
8.26E-01
**6.55E-03**
**2.24E-02**
**1.02E-02**
**2.73E-04**
7.39E-02
**1.13E-02**
**4.28E-03**
**1.96E-04**
9.14E-01
**9.82E-04**
**3.70E-03**
**1.86E-04**
9.96E-01
**1.84E-03**
9.15E-01
9.93E-01

Supplementary Table 11. Association of all meta

		CKB	
		All causes	
		Never/former smoker	
**Metabolites**	**Group**	**Adjusted HR (95% CI)**	**P value**
Concentration of chylomicrons and	Lipoprotein subclasses	0.93 (0.88, 0.99)	**4.83E-02**
Total lipids in chylomicrons and ex	Lipoprotein subclasses	0.93 (0.88, 0.99)	5.77E-02
Phospholipids in chylomicrons and	Lipoprotein subclasses	0.94 (0.89, 1.00)	7.89E-02
Cholesterol in chylomicrons and ex	Lipoprotein subclasses	0.97 (0.92, 1.03)	3.59E-01
Cholesteryl esters in chylomicrons a	Lipoprotein subclasses	0.98 (0.93, 1.04)	6.27E-01
Free cholesterol in chylomicrons an	Lipoprotein subclasses	0.95 (0.90, 1.01)	1.50E-01
Triglycerides in chylomicrons and e	Lipoprotein subclasses	0.93 (0.87, 0.98)	**2.72E-02**
Concentration of very large VLDL	Lipoprotein subclasses	0.95 (0.90, 1.01)	1.61E-01
Total lipids in very large VLDL	Lipoprotein subclasses	0.95 (0.90, 1.01)	1.79E-01
Phospholipids in very large VLDL	Lipoprotein subclasses	0.96 (0.90, 1.01)	1.92E-01
Cholesterol in very large VLDL	Lipoprotein subclasses	0.97 (0.92, 1.03)	4.09E-01
Cholesteryl esters in very large VLI	Lipoprotein subclasses	0.98 (0.92, 1.03)	5.07E-01
Free cholesterol in very large VLD	Lipoprotein subclasses	0.97 (0.91, 1.02)	3.23E-01
Triglycerides in very large VLDL	Lipoprotein subclasses	0.95 (0.89, 1.00)	1.27E-01
Concentration of large VLDL partic	Lipoprotein subclasses	0.95 (0.90, 1.00)	1.22E-01
Total lipids in large VLDL	Lipoprotein subclasses	0.95 (0.90, 1.00)	1.22E-01
Phospholipids in large VLDL	Lipoprotein subclasses	0.95 (0.89, 1.00)	1.18E-01
Cholesterol in large VLDL	Lipoprotein subclasses	0.95 (0.90, 1.00)	1.26E-01
Cholesteryl esters in large VLDL	Lipoprotein subclasses	0.95 (0.90, 1.00)	1.18E-01
Free cholesterol in large VLDL	Lipoprotein subclasses	0.95 (0.90, 1.01)	1.46E-01
Triglycerides in large VLDL	Lipoprotein subclasses	0.95 (0.90, 1.00)	1.22E-01
Concentration of medium VLDL pa	Lipoprotein subclasses	0.95 (0.90, 1.00)	1.26E-01
Total lipids in medium VLDL	Lipoprotein subclasses	0.95 (0.90, 1.00)	1.28E-01
Phospholipids in medium VLDL	Lipoprotein subclasses	0.95 (0.90, 1.00)	1.08E-01
Cholesterol in medium VLDL	Lipoprotein subclasses	0.96 (0.91, 1.01)	1.99E-01
Cholesteryl esters in medium VLDI	Lipoprotein subclasses	0.96 (0.91, 1.01)	2.18E-01
Free cholesterol in medium VLDL	Lipoprotein subclasses	0.96 (0.90, 1.01)	1.86E-01
Triglycerides in medium VLDL	Lipoprotein subclasses	0.95 (0.90, 1.00)	1.22E-01
Concentration of small VLDL parti	Lipoprotein subclasses	0.96 (0.91, 1.02)	2.35E-01
Total lipids in small VLDL	Lipoprotein subclasses	0.97 (0.92, 1.02)	3.34E-01
Phospholipids in small VLDL	Lipoprotein subclasses	0.95 (0.90, 1.00)	1.12E-01
Cholesterol in small VLDL	Lipoprotein subclasses	1.03 (0.98, 1.09)	3.09E-01
Cholesteryl esters in small VLDL	Lipoprotein subclasses	1.06 (1.00, 1.11)	7.89E-02
Free cholesterol in small VLDL	Lipoprotein subclasses	0.99 (0.94, 1.04)	7.34E-01
Triglycerides in small VLDL	Lipoprotein subclasses	0.94 (0.89, 0.99)	6.73E-02
Concentration of very small VLDL	Lipoprotein subclasses	1.03 (0.97, 1.08)	4.31E-01
Total lipids in very small VLDL	Lipoprotein subclasses	1.04 (0.98, 1.09)	2.47E-01
Phospholipids in very small VLDL	Lipoprotein subclasses	1.00 (0.95, 1.06)	9.68E-01
Cholesterol in very small VLDL	Lipoprotein subclasses	1.09 (1.04, 1.15)	**2.83E-03**
Cholesteryl esters in very small VL	Lipoprotein subclasses	1.10 (1.04, 1.15)	**1.72E-03**

Free cholesterol in very small VLD	Lipoprotein subclasses	1.08 (1.02, 1.13)	**1.47E-02**
Triglycerides in very small VLDL	Lipoprotein subclasses	0.96 (0.91, 1.01)	2.14E-01
Concentration of IDL particles	Lipoprotein subclasses	0.99 (0.94, 1.05)	8.86E-01
Total lipids in IDL	Lipoprotein subclasses	1.00 (0.95, 1.05)	9.61E-01
Phospholipids in IDL	Lipoprotein subclasses	0.99 (0.93, 1.04)	6.60E-01
Cholesterol in IDL	Lipoprotein subclasses	1.00 (0.95, 1.06)	9.56E-01
Cholesteryl esters in IDL	Lipoprotein subclasses	0.99 (0.94, 1.05)	8.07E-01
Free cholesterol in IDL	Lipoprotein subclasses	1.03 (0.97, 1.08)	4.02E-01
Triglycerides in IDL	Lipoprotein subclasses	0.99 (0.93, 1.04)	6.78E-01
Concentration of large LDL particl	Lipoprotein subclasses	0.98 (0.92, 1.03)	5.00E-01
Total lipids in large LDL	Lipoprotein subclasses	0.98 (0.93, 1.03)	5.21E-01
Phospholipids in large LDL	Lipoprotein subclasses	0.96 (0.91, 1.01)	2.08E-01
Cholesterol in large LDL	Lipoprotein subclasses	0.98 (0.93, 1.04)	6.04E-01
Cholesteryl esters in large LDL	Lipoprotein subclasses	0.98 (0.92, 1.03)	5.00E-01
Free cholesterol in large LDL	Lipoprotein subclasses	1.00 (0.95, 1.05)	9.61E-01
Triglycerides in large LDL	Lipoprotein subclasses	1.01 (0.95, 1.06)	8.37E-01
Concentration of medium LDL part	Lipoprotein subclasses	0.96 (0.91, 1.01)	2.14E-01
Total lipids in medium LDL	Lipoprotein subclasses	0.96 (0.91, 1.01)	2.16E-01
Phospholipids in medium LDL	Lipoprotein subclasses	0.90 (0.85, 0.96)	**1.86E-03**
Cholesterol in medium LDL	Lipoprotein subclasses	0.97 (0.92, 1.03)	4.18E-01
Cholesteryl esters in medium LDL	Lipoprotein subclasses	0.98 (0.92, 1.03)	5.00E-01
Free cholesterol in medium LDL	Lipoprotein subclasses	0.95 (0.90, 1.01)	1.36E-01
Triglycerides in medium LDL	Lipoprotein subclasses	0.99 (0.94, 1.05)	8.07E-01
Concentration of small LDL particl	Lipoprotein subclasses	0.96 (0.90, 1.01)	2.03E-01
Total lipids in small LDL	Lipoprotein subclasses	0.96 (0.91, 1.01)	2.16E-01
Phospholipids in small LDL	Lipoprotein subclasses	0.90 (0.85, 0.95)	**7.60E-04**
Cholesterol in small LDL	Lipoprotein subclasses	0.98 (0.93, 1.04)	5.46E-01
Cholesteryl esters in small LDL	Lipoprotein subclasses	0.98 (0.93, 1.04)	6.27E-01
Free cholesterol in small LDL	Lipoprotein subclasses	0.95 (0.90, 1.01)	1.53E-01
Triglycerides in small LDL	Lipoprotein subclasses	0.97 (0.92, 1.02)	3.59E-01
Concentration of very large HDL p	Lipoprotein subclasses	1.18 (1.12, 1.24)	**1.51E-09**
Total lipids in very large HDL	Lipoprotein subclasses	1.18 (1.12, 1.24)	**1.51E-09**
Phospholipids in very large HDL	Lipoprotein subclasses	1.16 (1.10, 1.22)	**7.89E-08**
Cholesterol in very large HDL	Lipoprotein subclasses	1.19 (1.13, 1.25)	**3.32E-10**
Cholesteryl esters in very large HD	Lipoprotein subclasses	1.19 (1.14, 1.26)	**1.26E-10**
Free cholesterol in very large HDL	Lipoprotein subclasses	1.17 (1.11, 1.23)	**7.43E-09**
Triglycerides in very large HDL	Lipoprotein subclasses	1.11 (1.06, 1.17)	**2.52E-04**
Concentration of large HDL particl	Lipoprotein subclasses	1.11 (1.05, 1.17)	**4.41E-04**
Total lipids in large HDL	Lipoprotein subclasses	1.11 (1.05, 1.17)	**3.58E-04**
Phospholipids in large HDL	Lipoprotein subclasses	1.10 (1.04, 1.16)	**1.58E-03**
Cholesterol in large HDL	Lipoprotein subclasses	1.12 (1.06, 1.18)	**1.25E-04**
Cholesteryl esters in large HDL	Lipoprotein subclasses	1.11 (1.06, 1.17)	**2.56E-04**
Free cholesterol in large HDL	Lipoprotein subclasses	1.13 (1.08, 1.19)	**1.13E-05**
Triglycerides in large HDL	Lipoprotein subclasses	1.07 (1.02, 1.13)	**3.38E-02**
**Concentration of medium HDL p**	Lipoprotein subclasses	0.86 (0.81, 0.91)	**3.35E-07**
**Total lipids in medium HDL**	Lipoprotein subclasses	0.86 (0.82, 0.91)	**9.63E-07**
**Phospholipids in medium HDL**	Lipoprotein subclasses	0.88 (0.83, 0.93)	**2.48E-05**
**Cholesterol in medium HDL**	Lipoprotein subclasses	0.88 (0.83, 0.93)	**1.05E-05**

**Cholesteryl esters in medium HD**	Lipoprotein subclasses	0.87 (0.83, 0.92)	**3.25E-06**
**Free cholesterol in medium HDL**	Lipoprotein subclasses	0.90 (0.85, 0.95)	**6.76E-04**
Triglycerides in medium HDL	Lipoprotein subclasses	0.84 (0.79, 0.89)	**5.22E-08**
Concentration of small HDL particl	Lipoprotein subclasses	0.79 (0.75, 0.83)	**4.19E-17**
Total lipids in small HDL	Lipoprotein subclasses	0.79 (0.75, 0.83)	**8.35E-17**
Phospholipids in small HDL	Lipoprotein subclasses	0.82 (0.78, 0.86)	**8.79E-12**
Cholesterol in small HDL	Lipoprotein subclasses	0.89 (0.84, 0.94)	**9.27E-05**
Cholesteryl esters in small HDL	Lipoprotein subclasses	0.92 (0.87, 0.97)	**7.40E-03**
**Free cholesterol in small HDL**	Lipoprotein subclasses	0.84 (0.80, 0.89)	**3.52E-09**
Triglycerides in small HDL	Lipoprotein subclasses	0.87 (0.83, 0.92)	**1.57E-05**
Phospholipids to total lipids ratio ir	Relative lipoprotein lipid	0.93 (0.88, 0.98)	**1.67E-02**
Cholesterol to total lipids ratio in cl	Relative lipoprotein lipid	1.15 (1.10, 1.20)	**1.55E-09**
Cholesteryl esters to total lipids rati	Relative lipoprotein lipid	1.15 (1.10, 1.20)	**3.32E-10**
Free cholesterol to total lipids ratio	Relative lipoprotein lipid	1.05 (0.99, 1.11)	1.59E-01
Triglycerides to total lipids ratio in	Relative lipoprotein lipid	0.89 (0.85, 0.94)	**4.53E-05**
Phospholipids to total lipids ratio ir	Relative lipoprotein lipid	0.92 (0.87, 0.97)	**7.48E-03**
**Cholesterol to total lipids ratio in**	Relative lipoprotein lipid	1.12 (1.07, 1.16)	**1.44E-06**
Cholesteryl esters to total lipids rati	Relative lipoprotein lipid	1.12 (1.08, 1.17)	**8.92E-08**
**Free cholesterol to total lipids rat**	Relative lipoprotein lipid	1.01 (0.95, 1.07)	8.83E-01
**Triglycerides to total lipids ratio**	Relative lipoprotein lipid	0.94 (0.88, 0.99)	6.58E-02
Phospholipids to total lipids ratio ir	Relative lipoprotein lipid	0.94 (0.88, 0.99)	6.45E-02
Cholesterol to total lipids ratio in la	Relative lipoprotein lipid	0.98 (0.92, 1.04)	5.99E-01
Cholesteryl esters to total lipids rati	Relative lipoprotein lipid	1.03 (0.97, 1.09)	3.98E-01
Free cholesterol to total lipids ratio	Relative lipoprotein lipid	0.95 (0.90, 1.01)	1.50E-01
Triglycerides to total lipids ratio in	Relative lipoprotein lipid	1.03 (0.98, 1.10)	3.34E-01
Phospholipids to total lipids ratio ir	Relative lipoprotein lipid	0.93 (0.88, 0.99)	**4.64E-02**
**Cholesterol to total lipids ratio in**	Relative lipoprotein lipid	1.04 (0.99, 1.11)	2.16E-01
**Cholesteryl esters to total lipids r**	Relative lipoprotein lipid	1.04 (0.98, 1.10)	2.35E-01
Free cholesterol to total lipids ratio	Relative lipoprotein lipid	1.00 (0.95, 1.06)	9.30E-01
Triglycerides to total lipids ratio in	Relative lipoprotein lipid	0.97 (0.92, 1.03)	4.12E-01
**Phospholipids to total lipids ratio**	Relative lipoprotein lipid	0.90 (0.86, 0.96)	**1.63E-03**
Cholesterol to total lipids ratio in sı	Relative lipoprotein lipid	1.14 (1.08, 1.20)	**1.10E-05**
Cholesteryl esters to total lipids rati	Relative lipoprotein lipid	1.13 (1.07, 1.19)	**4.74E-05**
**Free cholesterol to total lipids rat**	Relative lipoprotein lipid	1.15 (1.09, 1.21)	**1.44E-06**
Triglycerides to total lipids ratio in	Relative lipoprotein lipid	0.90 (0.85, 0.95)	**3.31E-04**
Phospholipids to total lipids ratio ir	Relative lipoprotein lipid	0.93 (0.89, 0.98)	**2.34E-02**
Cholesterol to total lipids ratio in v	Relative lipoprotein lipid	1.19 (1.12, 1.26)	**1.97E-08**
Cholesteryl esters to total lipids rati	Relative lipoprotein lipid	1.16 (1.10, 1.23)	**9.63E-07**
Free cholesterol to total lipids ratio	Relative lipoprotein lipid	1.17 (1.10, 1.24)	**3.25E-06**
Triglycerides to total lipids ratio in	Relative lipoprotein lipid	0.90 (0.85, 0.95)	**1.45E-03**
Phospholipids to total lipids ratio ir	Relative lipoprotein lipid	0.94 (0.89, 0.99)	6.73E-02
Cholesterol to total lipids ratio in II	Relative lipoprotein lipid	1.04 (0.99, 1.10)	1.99E-01
Cholesteryl esters to total lipids rati	Relative lipoprotein lipid	0.97 (0.92, 1.02)	3.30E-01
Free cholesterol to total lipids ratio	Relative lipoprotein lipid	1.11 (1.05, 1.18)	**1.68E-03**
Triglycerides to total lipids ratio in	Relative lipoprotein lipid	0.98 (0.93, 1.03)	5.00E-01
Phospholipids to total lipids ratio ir	Relative lipoprotein lipid	0.95 (0.89, 1.00)	1.22E-01
Cholesterol to total lipids ratio in la	Relative lipoprotein lipid	1.01 (0.96, 1.07)	6.60E-01
Cholesteryl esters to total lipids rati	Relative lipoprotein lipid	0.98 (0.93, 1.03)	5.29E-01

Free cholesterol to total lipids ratio	Relative lipoprotein lipid (	1.11 (1.05, 1.17)	**1.76E-03**
Triglycerides to total lipids ratio in	Relative lipoprotein lipid (	1.02 (0.96, 1.07)	6.04E-01
Phospholipids to total lipids ratio in	Relative lipoprotein lipid (	0.94 (0.89, 1.00)	7.89E-02
Cholesterol to total lipids ratio in m	Relative lipoprotein lipid (	1.03 (0.97, 1.09)	3.75E-01
Cholesteryl esters to total lipids rati	Relative lipoprotein lipid (	1.02 (0.96, 1.08)	6.22E-01
Free cholesterol to total lipids ratio	Relative lipoprotein lipid (	1.03 (0.97, 1.09)	4.70E-01
Triglycerides to total lipids ratio in	Relative lipoprotein lipid (	1.03 (0.98, 1.09)	3.34E-01
Phospholipids to total lipids ratio in	Relative lipoprotein lipid (	0.94 (0.89, 1.00)	1.05E-01
Cholesterol to total lipids ratio in sı	Relative lipoprotein lipid (	1.04 (0.98, 1.10)	2.43E-01
Cholesteryl esters to total lipids rati	Relative lipoprotein lipid (	1.03 (0.97, 1.09)	3.98E-01
Free cholesterol to total lipids ratio	Relative lipoprotein lipid (	1.02 (0.97, 1.08)	5.00E-01
Triglycerides to total lipids ratio in	Relative lipoprotein lipid (	1.00 (0.95, 1.06)	9.99E-01
Phospholipids to total lipids ratio in	Relative lipoprotein lipid (	0.97 (0.92, 1.03)	4.07E-01
Cholesterol to total lipids ratio in v	Relative lipoprotein lipid (	1.07 (1.01, 1.13)	5.56E-02
Cholesteryl esters to total lipids rati	Relative lipoprotein lipid (	1.06 (1.01, 1.12)	7.65E-02
Free cholesterol to total lipids ratio	Relative lipoprotein lipid (	1.05 (1.00, 1.11)	1.08E-01
Triglycerides to total lipids ratio in	Relative lipoprotein lipid (	0.92 (0.87, 0.97)	**1.34E-02**
Phospholipids to total lipids ratio in	Relative lipoprotein lipid (	0.88 (0.83, 0.93)	**5.46E-05**
Cholesterol to total lipids ratio in la	Relative lipoprotein lipid (	1.14 (1.08, 1.21)	**3.14E-05**
Cholesteryl esters to total lipids rati	Relative lipoprotein lipid (	1.09 (1.03, 1.16)	**7.48E-03**
Free cholesterol to total lipids ratio	Relative lipoprotein lipid (	1.19 (1.12, 1.26)	**9.81E-08**
Triglycerides to total lipids ratio in	Relative lipoprotein lipid (	0.96 (0.91, 1.01)	2.01E-01
Phospholipids to total lipids ratio in	Relative lipoprotein lipid (	1.13 (1.08, 1.17)	**3.20E-07**
Cholesterol to total lipids ratio in m	Relative lipoprotein lipid (	0.97 (0.92, 1.02)	2.57E-01
Cholesteryl esters to total lipids rati	Relative lipoprotein lipid (	0.96 (0.91, 1.01)	2.35E-01
Free cholesterol to total lipids ratio	Relative lipoprotein lipid (	0.99 (0.93, 1.04)	6.59E-01
Triglycerides to total lipids ratio in	Relative lipoprotein lipid (	0.93 (0.88, 0.99)	**4.73E-02**
Phospholipids to total lipids ratio in	Relative lipoprotein lipid (	0.96 (0.91, 1.02)	2.28E-01
Cholesterol to total lipids ratio in sı	Relative lipoprotein lipid (	1.05 (0.99, 1.11)	1.50E-01
Cholesteryl esters to total lipids rati	Relative lipoprotein lipid (	1.04 (0.98, 1.10)	2.35E-01
Free cholesterol to total lipids ratio	Relative lipoprotein lipid (	1.04 (0.99, 1.10)	2.18E-01
Triglycerides to total lipids ratio in	Relative lipoprotein lipid (	0.95 (0.90, 1.01)	1.53E-01
Average diameter for VLDL particl	Lipoprotein particle sizes	0.92 (0.87, 0.97)	**7.48E-03**
Average diameter for LDL particles	Lipoprotein particle sizes	1.06 (1.00, 1.12)	1.16E-01
Average diameter for HDL particle	Lipoprotein particle sizes	1.18 (1.12, 1.24)	**6.29E-09**
Total cholesterol	Cholesterol	1.02 (0.96, 1.07)	5.99E-01
VLDL cholesterol	Cholesterol	1.00 (0.95, 1.06)	9.11E-01
Remnant cholesterol (non-HDL, no	Cholesterol	1.00 (0.95, 1.06)	9.00E-01
LDL cholesterol	Cholesterol	0.99 (0.94, 1.04)	7.56E-01
**HDL cholesterol**	Cholesterol	1.06 (1.01, 1.12)	6.58E-02
**HDL2 cholesterol**	Cholesterol	1.05 (0.99, 1.10)	1.50E-01
HDL3 cholesterol	Cholesterol	1.16 (1.10, 1.22)	**9.81E-08**
Total esterified cholesterol	Cholesteryl esters	0.89 (0.84, 0.94)	**2.52E-04**
Total free cholesterol	Free cholesterol	0.94 (0.89, 1.00)	1.01E-01
Total triglycerides	Triglycerides	0.95 (0.90, 1.00)	1.27E-01
Triglycerides in VLDL	Triglycerides	0.95 (0.90, 1.00)	1.12E-01
Triglycerides in LDL	Triglycerides	1.00 (0.95, 1.06)	9.61E-01
Triglycerides in HDL	Triglycerides	0.94 (0.89, 1.00)	8.54E-02

Phosphoglycerides	Other lipids	1.01 (0.95, 1.06)	8.88E-01
Ratio of triglycerides to phosphogly	Other lipids	0.97 (0.92, 1.02)	3.34E-01
Phosphatidylcholines	Other lipids	0.90 (0.85, 0.95)	**6.66E-04**
Sphingomyelins	Other lipids	0.87 (0.82, 0.92)	**1.48E-05**
Total cholines	Other lipids	0.98 (0.93, 1.04)	6.04E-01
Apolipoprotein A1	Apolipoproteins	1.05 (1.00, 1.11)	9.96E-02
Apolipoprotein B	Apolipoproteins	0.97 (0.92, 1.03)	3.91E-01
Ratio of apolipoprotein B to apolip	Apolipoproteins	0.94 (0.89, 1.00)	9.72E-02
Total fatty acids	Fatty acids	0.94 (0.89, 1.00)	7.89E-02
Degree of unsaturation	Fatty acids	0.83 (0.79, 0.88)	**1.19E-09**
Docosahexaenoic acid	Fatty acids	1.13 (1.08, 1.19)	**8.08E-06**
Linoleic acid	Fatty acids	0.94 (0.88, 0.99)	6.45E-02
Omega-3 fatty acids	Fatty acids	1.02 (0.97, 1.08)	4.56E-01
Omega-6 fatty acids	Fatty acids	0.91 (0.86, 0.96)	**4.16E-03**
Polyunsaturated fatty acids	Fatty acids	0.93 (0.87, 0.98)	**2.66E-02**
Monounsaturated fatty acids	Fatty acids	0.94 (0.89, 1.00)	7.89E-02
Saturated fatty acids	Fatty acids	0.96 (0.91, 1.02)	2.34E-01
Ratio of docosahexaenoic acid to to	Fatty acids	1.24 (1.17, 1.31)	**3.44E-12**
Ratio of linoleic acid to total fatty a	Fatty acids	0.97 (0.91, 1.02)	3.32E-01
Ratio of omega-3 fatty acids to tota	Fatty acids	1.10 (1.04, 1.16)	**1.76E-03**
Ratio of omega-6 fatty acids to tota	Fatty acids	0.93 (0.87, 0.98)	**2.43E-02**
Ratio of polyunsaturated fatty acids	Fatty acids	0.95 (0.90, 1.01)	1.91E-01
Ratio of monounsaturated fatty acid	Fatty acids	0.97 (0.92, 1.02)	3.46E-01
Ratio of saturated fatty acids to tota	Fatty acids	1.13 (1.06, 1.19)	**1.76E-04**
Glucose	Glycolysis related metabo	1.12 (1.07, 1.17)	**7.25E-07**
Lactate	Glycolysis related metabo	0.96 (0.91, 1.02)	2.60E-01
Citrate	Glycolysis related metabo	0.99 (0.93, 1.05)	8.00E-01
Alanine	Amino acids	0.92 (0.87, 0.97)	**1.09E-02**
Glutamine	Amino acids	0.92 (0.87, 0.97)	**1.26E-02**
Histidine	Amino acids	1.00 (0.94, 1.06)	9.61E-01
Isoleucine	Amino acids	0.92 (0.87, 0.98)	**2.11E-02**
Leucine	Amino acids	0.95 (0.90, 1.01)	1.61E-01
Valine	Amino acids	0.82 (0.78, 0.88)	**4.45E-09**
Phenylalanine	Amino acids	1.13 (1.08, 1.18)	**3.86E-08**
Tyrosine	Amino acids	1.04 (0.99, 1.10)	2.05E-01
Acetate	Ketone bodies	1.03 (0.99, 1.07)	1.86E-01
Acetoacetate	Ketone bodies	0.94 (0.88, 0.99)	6.45E-02
**3-Hydroxybutyrate**	Ketone bodies	0.98 (0.92, 1.04)	5.07E-01
Creatinine	Fluid balance	1.04 (1.01, 1.07)	5.74E-02
Albumin	Fluid balance	0.83 (0.79, 0.88)	**4.52E-11**
Glycoprotein acetyls	Inflammation	1.16 (1.10, 1.22)	**2.73E-07**
Acetone	Ketone bodies		
Cholesteryl esters in HDL	Cholesteryl esters		
Cholesteryl esters in LDL	Cholesteryl esters		
Cholesteryl esters in VLDL/ (Chole	Cholesteryl esters		
Clinical LDL cholesterol	Cholesterol		
Concentration of HDL particles	Lipoprotein particle concentrations		
Concentration of LDL particles	Lipoprotein particle concentrations		

Concentration of VLDL particles	Lipoprotein particle concentrations
Free cholesterol in HDL	Free cholesterol
Free cholesterol in LDL	Free cholesterol
Free cholesterol in VLDL	Free cholesterol
Glycine	Amino acids
Phospholipids in HDL	Phospholipids
Phospholipids in LDL	Phospholipids
Phospholipids in VLDL	Phospholipids
Pyruvate	Glycolysis related metabolites
Ratio of omega-6 fatty acids to ome	Fatty acids
Ratio of polyunsaturated fatty acids	Fatty acids
Total cholesterol minus HDL-C	Cholesterol
Total concentration of branched-ch	Amino acids
Total concentration of lipoprotein p	Lipoprotein particle concentrations
Total lipids in HDL	Total lipids
Total lipids in LDL	Total lipids
Total lipids in lipoprotein particles	Total lipids
Total lipids in VLDL	Total lipids
Total phospholipids in lipoprotein p	Phospholipids

Models were adjusted for age, sex, regions (CKB only), ethnicity (UKB only), levels of education, smoking sta

HR, hazard ratio; CI, confidence interval; LDL, Low Density Lipoprotein; HDL, High Density Lipoprotein; V

abolites with all-cause and cause-specific mortality in the CKB and UKB by smoking status

s mortality		Ischaemic heart diseases mortality		
Current smoker		Never/former smoker		Current smok
Adjusted HR (95% CI)	P value	Adjusted HR (95% CI)	P value	Adjusted HR (95% CI
0.95 (0.88, 1.03)	4.10E-01	1.00 (0.90, 1.12)	9.48E-01	0.96 (0.84, 1.10)
0.95 (0.88, 1.03)	4.33E-01	1.01 (0.90, 1.12)	9.48E-01	0.96 (0.84, 1.11)
0.96 (0.89, 1.04)	6.24E-01	1.02 (0.92, 1.13)	8.95E-01	0.98 (0.85, 1.12)
0.99 (0.91, 1.07)	8.52E-01	1.04 (0.93, 1.15)	7.67E-01	1.00 (0.87, 1.14)
1.00 (0.92, 1.07)	9.37E-01	1.05 (0.95, 1.17)	5.84E-01	1.01 (0.88, 1.15)
0.98 (0.90, 1.05)	7.93E-01	1.01 (0.91, 1.12)	9.48E-01	0.97 (0.85, 1.12)
0.94 (0.87, 1.02)	3.03E-01	1.00 (0.90, 1.11)	9.48E-01	0.96 (0.83, 1.10)
0.98 (0.90, 1.06)	8.08E-01	1.00 (0.90, 1.12)	9.48E-01	0.97 (0.85, 1.11)
0.98 (0.90, 1.06)	8.15E-01	1.01 (0.91, 1.12)	9.48E-01	0.97 (0.85, 1.11)
0.98 (0.91, 1.06)	8.15E-01	1.01 (0.91, 1.13)	9.29E-01	0.98 (0.86, 1.12)
0.99 (0.91, 1.07)	8.52E-01	1.03 (0.93, 1.15)	7.68E-01	0.99 (0.87, 1.13)
0.99 (0.91, 1.07)	8.52E-01	1.04 (0.94, 1.15)	7.37E-01	0.99 (0.87, 1.13)
0.99 (0.91, 1.07)	8.53E-01	1.02 (0.92, 1.14)	8.65E-01	0.99 (0.86, 1.13)
0.97 (0.90, 1.05)	7.67E-01	1.00 (0.90, 1.11)	9.48E-01	0.96 (0.84, 1.11)
0.98 (0.91, 1.06)	8.15E-01	1.02 (0.92, 1.13)	8.95E-01	0.99 (0.87, 1.14)
0.98 (0.91, 1.06)	8.15E-01	1.02 (0.92, 1.13)	8.95E-01	0.99 (0.87, 1.14)
0.98 (0.91, 1.06)	8.15E-01	1.02 (0.91, 1.13)	9.19E-01	0.99 (0.87, 1.13)
0.98 (0.91, 1.06)	8.15E-01	1.01 (0.91, 1.12)	9.44E-01	0.98 (0.86, 1.12)
0.97 (0.90, 1.05)	7.46E-01	1.01 (0.91, 1.12)	9.48E-01	0.97 (0.85, 1.11)
0.99 (0.92, 1.07)	8.63E-01	1.02 (0.92, 1.13)	9.09E-01	0.99 (0.87, 1.14)
0.98 (0.91, 1.06)	8.15E-01	1.02 (0.92, 1.13)	8.95E-01	1.00 (0.87, 1.14)
0.98 (0.91, 1.06)	8.15E-01	1.03 (0.93, 1.15)	7.89E-01	1.02 (0.89, 1.17)
0.98 (0.91, 1.06)	8.15E-01	1.03 (0.93, 1.14)	7.93E-01	1.02 (0.89, 1.17)
0.98 (0.91, 1.06)	8.15E-01	1.03 (0.92, 1.14)	8.65E-01	1.02 (0.89, 1.16)
0.99 (0.92, 1.07)	8.52E-01	1.03 (0.92, 1.14)	8.65E-01	1.01 (0.88, 1.16)
0.98 (0.91, 1.06)	8.15E-01	1.01 (0.91, 1.13)	9.24E-01	1.00 (0.88, 1.14)
0.99 (0.92, 1.07)	9.30E-01	1.04 (0.93, 1.15)	7.67E-01	1.02 (0.90, 1.17)
0.98 (0.91, 1.06)	8.15E-01	1.04 (0.93, 1.15)	7.61E-01	1.03 (0.90, 1.17)
1.00 (0.92, 1.08)	9.58E-01	1.05 (0.95, 1.17)	6.00E-01	1.05 (0.91, 1.20)
1.00 (0.93, 1.08)	9.58E-01	1.06 (0.96, 1.18)	4.60E-01	1.06 (0.93, 1.21)
0.99 (0.92, 1.07)	9.30E-01	1.04 (0.94, 1.15)	7.43E-01	1.02 (0.89, 1.17)
1.04 (0.97, 1.12)	5.01E-01	1.16 (1.05, 1.28)	**1.06E-02**	1.18 (1.04, 1.34)
1.04 (0.97, 1.12)	4.56E-01	1.19 (1.07, 1.31)	**3.48E-03**	1.22 (1.07, 1.38)
1.03 (0.96, 1.11)	6.91E-01	1.10 (1.00, 1.22)	1.48E-01	1.09 (0.96, 1.24)
0.98 (0.91, 1.06)	8.15E-01	1.00 (0.90, 1.12)	9.48E-01	0.99 (0.86, 1.14)
1.06 (0.98, 1.13)	3.13E-01	1.12 (1.01, 1.24)	7.13E-02	1.13 (1.00, 1.28)
1.06 (0.98, 1.14)	2.88E-01	1.14 (1.03, 1.25)	**3.36E-02**	1.15 (1.01, 1.30)
1.02 (0.94, 1.09)	8.25E-01	1.04 (0.93, 1.15)	7.61E-01	1.05 (0.93, 1.20)
1.08 (1.00, 1.16)	1.09E-01	1.23 (1.12, 1.35)	**1.32E-04**	1.24 (1.10, 1.40)
1.08 (1.01, 1.16)	8.04E-02	1.24 (1.13, 1.36)	**5.58E-05**	1.26 (1.12, 1.42)

1.06 (0.99, 1.14)	2.47E-01	1.19 (1.08, 1.31)	**2.13E-03**	1.20 (1.06, 1.36)
1.03 (0.96, 1.11)	6.63E-01	1.01 (0.91, 1.12)	9.48E-01	0.99 (0.86, 1.14)
1.01 (0.94, 1.09)	8.72E-01	1.01 (0.91, 1.13)	9.37E-01	1.04 (0.91, 1.18)
1.01 (0.94, 1.08)	8.96E-01	1.02 (0.91, 1.13)	9.09E-01	1.05 (0.92, 1.19)
0.99 (0.92, 1.07)	8.91E-01	0.99 (0.89, 1.10)	9.48E-01	1.03 (0.90, 1.17)
1.00 (0.93, 1.08)	9.80E-01	1.03 (0.92, 1.14)	8.65E-01	1.06 (0.93, 1.21)
0.99 (0.92, 1.07)	8.96E-01	1.02 (0.92, 1.13)	8.95E-01	1.06 (0.93, 1.20)
1.01 (0.94, 1.09)	8.52E-01	1.04 (0.93, 1.15)	7.60E-01	1.06 (0.93, 1.21)
1.09 (1.02, 1.18)	**4.52E-02**	1.00 (0.90, 1.12)	9.48E-01	0.96 (0.84, 1.10)
0.99 (0.92, 1.07)	8.91E-01	1.01 (0.90, 1.12)	9.48E-01	1.03 (0.90, 1.18)
0.99 (0.92, 1.07)	8.52E-01	1.01 (0.90, 1.12)	9.48E-01	1.03 (0.91, 1.18)
0.97 (0.90, 1.05)	6.89E-01	0.98 (0.88, 1.09)	8.95E-01	1.01 (0.89, 1.15)
0.98 (0.91, 1.06)	8.15E-01	1.01 (0.91, 1.13)	9.48E-01	1.04 (0.91, 1.19)
0.98 (0.90, 1.05)	7.79E-01	1.01 (0.91, 1.13)	9.44E-01	1.04 (0.91, 1.19)
0.99 (0.92, 1.07)	8.91E-01	1.01 (0.91, 1.12)	9.48E-01	1.04 (0.91, 1.18)
1.12 (1.05, 1.20)	**5.25E-03**	1.03 (0.93, 1.15)	7.67E-01	0.99 (0.86, 1.13)
0.96 (0.89, 1.04)	5.34E-01	1.00 (0.89, 1.11)	9.49E-01	1.01 (0.88, 1.15)
0.96 (0.89, 1.03)	4.91E-01	1.00 (0.89, 1.11)	9.48E-01	1.01 (0.89, 1.16)
0.92 (0.86, 1.00)	1.15E-01	0.92 (0.82, 1.03)	3.22E-01	0.93 (0.81, 1.06)
0.96 (0.89, 1.03)	5.01E-01	1.01 (0.91, 1.13)	9.37E-01	1.04 (0.91, 1.19)
0.96 (0.89, 1.04)	5.81E-01	1.02 (0.91, 1.14)	8.95E-01	1.06 (0.93, 1.21)
0.94 (0.87, 1.02)	2.80E-01	0.98 (0.88, 1.09)	8.95E-01	0.97 (0.85, 1.11)
1.09 (1.01, 1.18)	6.76E-02	1.02 (0.92, 1.13)	8.95E-01	0.93 (0.81, 1.07)
0.96 (0.89, 1.03)	5.01E-01	1.02 (0.92, 1.14)	8.95E-01	1.02 (0.90, 1.17)
0.96 (0.89, 1.03)	4.75E-01	1.02 (0.92, 1.14)	8.95E-01	1.03 (0.90, 1.17)
0.92 (0.86, 1.00)	1.09E-01	0.94 (0.84, 1.05)	5.52E-01	0.92 (0.81, 1.05)
0.96 (0.89, 1.04)	5.73E-01	1.04 (0.93, 1.16)	7.47E-01	1.06 (0.93, 1.21)
0.97 (0.90, 1.05)	6.63E-01	1.04 (0.94, 1.16)	7.34E-01	1.08 (0.95, 1.23)
0.94 (0.88, 1.02)	2.84E-01	1.02 (0.91, 1.14)	8.95E-01	0.99 (0.87, 1.13)
1.05 (0.97, 1.13)	4.56E-01	1.05 (0.94, 1.16)	6.41E-01	0.98 (0.86, 1.12)
1.17 (1.10, 1.25)	**7.22E-05**	1.25 (1.14, 1.37)	**3.85E-05**	1.15 (1.03, 1.30)
1.17 (1.10, 1.25)	**8.63E-05**	1.25 (1.14, 1.38)	**2.79E-05**	1.16 (1.03, 1.30)
1.17 (1.09, 1.25)	**1.69E-04**	1.16 (1.05, 1.28)	**1.21E-02**	1.08 (0.96, 1.22)
1.16 (1.09, 1.24)	**1.98E-04**	1.35 (1.23, 1.49)	**9.97E-09**	1.22 (1.09, 1.37)
1.16 (1.09, 1.24)	**1.69E-04**	1.40 (1.27, 1.54)	**2.02E-10**	1.26 (1.12, 1.42)
1.15 (1.08, 1.23)	**3.58E-04**	1.24 (1.13, 1.37)	**6.49E-05**	1.14 (1.01, 1.28)
1.15 (1.08, 1.23)	**3.58E-04**	1.19 (1.08, 1.31)	**2.63E-03**	1.14 (1.01, 1.29)
1.14 (1.07, 1.23)	**1.10E-03**	1.08 (0.98, 1.20)	2.83E-01	1.03 (0.91, 1.18)
1.14 (1.07, 1.23)	**1.10E-03**	1.08 (0.98, 1.20)	2.62E-01	1.03 (0.91, 1.18)
1.14 (1.06, 1.22)	**2.02E-03**	1.08 (0.98, 1.20)	2.83E-01	1.04 (0.91, 1.18)
1.14 (1.06, 1.22)	**1.22E-03**	1.09 (0.99, 1.21)	2.14E-01	1.03 (0.91, 1.18)
1.14 (1.06, 1.22)	**1.35E-03**	1.08 (0.98, 1.20)	2.83E-01	1.03 (0.90, 1.17)
1.15 (1.07, 1.23)	**9.87E-04**	1.13 (1.02, 1.24)	6.04E-02	1.06 (0.93, 1.20)
1.16 (1.08, 1.24)	**2.16E-04**	0.99 (0.89, 1.11)	9.48E-01	0.97 (0.85, 1.11)
0.95 (0.88, 1.03)	4.10E-01	0.80 (0.72, 0.89)	**1.83E-04**	0.84 (0.72, 0.97)
0.95 (0.88, 1.03)	4.10E-01	0.81 (0.73, 0.89)	**3.21E-04**	0.84 (0.73, 0.97)
0.99 (0.91, 1.07)	8.52E-01	0.83 (0.75, 0.93)	**4.07E-03**	0.86 (0.75, 1.00)
0.93 (0.86, 1.00)	1.64E-01	0.82 (0.74, 0.91)	**6.74E-04**	0.85 (0.74, 0.98)

0.92 (0.85, 0.99)	9.44E-02	0.81 (0.74, 0.90)	**3.54E-04**	0.85 (0.74, 0.98)
0.98 (0.90, 1.05)	7.86E-01	0.85 (0.76, 0.94)	**7.56E-03**	0.87 (0.76, 1.01)
0.95 (0.88, 1.02)	2.98E-01	0.76 (0.68, 0.86)	**4.18E-05**	0.80 (0.70, 0.92)
0.88 (0.82, 0.95)	**4.13E-03**	0.75 (0.68, 0.82)	**2.79E-08**	0.79 (0.71, 0.89)
0.88 (0.82, 0.95)	**4.25E-03**	0.75 (0.68, 0.83)	**5.28E-08**	0.80 (0.71, 0.89)
0.92 (0.85, 0.99)	1.07E-01	0.79 (0.71, 0.87)	**5.06E-05**	0.83 (0.72, 0.95)
0.91 (0.85, 0.98)	**4.00E-02**	0.84 (0.76, 0.93)	**4.58E-03**	0.87 (0.77, 0.99)
0.92 (0.86, 0.99)	8.36E-02	0.87 (0.79, 0.97)	**3.52E-02**	0.90 (0.79, 1.02)
0.94 (0.87, 1.01)	2.47E-01	0.82 (0.74, 0.90)	**5.28E-04**	0.85 (0.74, 0.98)
0.97 (0.90, 1.04)	6.61E-01	0.84 (0.75, 0.94)	**9.23E-03**	0.84 (0.73, 0.96)
0.99 (0.92, 1.06)	8.52E-01	0.97 (0.87, 1.08)	8.48E-01	1.05 (0.92, 1.20)
1.16 (1.09, 1.23)	**7.22E-05**	1.13 (1.04, 1.22)	**9.49E-03**	1.12 (1.01, 1.25)
1.15 (1.08, 1.22)	**1.30E-04**	1.14 (1.06, 1.23)	**2.87E-03**	1.12 (1.01, 1.25)
1.09 (1.01, 1.17)	6.77E-02	1.01 (0.90, 1.13)	9.48E-01	1.05 (0.92, 1.19)
0.87 (0.81, 0.93)	**5.90E-04**	0.89 (0.81, 0.98)	**4.22E-02**	0.88 (0.78, 0.99)
1.01 (0.94, 1.08)	8.96E-01	0.98 (0.87, 1.09)	8.95E-01	1.07 (0.94, 1.22)
1.07 (1.01, 1.13)	7.17E-02	1.14 (1.05, 1.24)	**5.25E-03**	1.08 (0.99, 1.18)
1.07 (1.00, 1.14)	1.34E-01	1.14 (1.05, 1.23)	**6.13E-03**	1.08 (0.97, 1.21)
1.04 (0.97, 1.11)	4.47E-01	1.04 (0.93, 1.17)	7.61E-01	1.07 (0.96, 1.19)
0.94 (0.87, 1.00)	1.72E-01	0.88 (0.79, 0.99)	9.64E-02	0.89 (0.79, 1.00)
0.98 (0.91, 1.05)	8.15E-01	0.88 (0.80, 0.98)	6.04E-02	0.95 (0.84, 1.08)
0.97 (0.90, 1.04)	5.61E-01	0.87 (0.79, 0.96)	**2.24E-02**	0.94 (0.84, 1.06)
0.97 (0.90, 1.04)	6.47E-01	0.88 (0.79, 0.98)	7.13E-02	0.89 (0.78, 1.01)
0.98 (0.92, 1.06)	8.15E-01	0.94 (0.85, 1.05)	5.34E-01	1.03 (0.91, 1.17)
1.03 (0.96, 1.11)	5.99E-01	1.15 (1.05, 1.27)	**1.55E-02**	1.06 (0.94, 1.20)
0.97 (0.90, 1.04)	6.63E-01	0.79 (0.71, 0.89)	**6.61E-04**	0.81 (0.71, 0.93)
1.02 (0.95, 1.10)	7.86E-01	0.90 (0.81, 1.00)	1.48E-01	0.91 (0.80, 1.03)
1.01 (0.94, 1.08)	8.96E-01	0.89 (0.80, 1.00)	1.15E-01	0.89 (0.78, 1.01)
1.05 (0.97, 1.12)	4.18E-01	1.03 (0.92, 1.16)	8.02E-01	1.06 (0.93, 1.21)
0.98 (0.92, 1.06)	8.15E-01	1.14 (1.02, 1.28)	5.43E-02	1.13 (0.99, 1.29)
0.99 (0.92, 1.06)	8.52E-01	0.81 (0.73, 0.91)	**2.33E-03**	0.77 (0.66, 0.89)
1.08 (1.01, 1.16)	9.44E-02	1.23 (1.10, 1.36)	**8.80E-04**	1.22 (1.07, 1.39)
1.06 (0.99, 1.14)	2.14E-01	1.20 (1.08, 1.34)	**2.98E-03**	1.21 (1.07, 1.38)
1.16 (1.09, 1.25)	**2.47E-04**	1.28 (1.16, 1.41)	**1.44E-05**	1.16 (1.03, 1.31)
0.93 (0.86, 1.00)	1.15E-01	0.85 (0.77, 0.95)	**1.08E-02**	0.87 (0.76, 0.99)
0.91 (0.86, 0.98)	**2.94E-02**	0.84 (0.77, 0.92)	**6.82E-04**	0.84 (0.76, 0.94)
1.08 (1.01, 1.17)	9.03E-02	1.43 (1.27, 1.61)	**5.28E-08**	1.46 (1.26, 1.70)
1.09 (1.01, 1.17)	6.42E-02	1.36 (1.21, 1.52)	**1.19E-06**	1.41 (1.23, 1.62)
1.03 (0.96, 1.11)	7.10E-01	1.33 (1.17, 1.51)	**8.87E-05**	1.21 (1.05, 1.40)
0.99 (0.92, 1.06)	8.52E-01	0.85 (0.76, 0.95)	**1.47E-02**	0.85 (0.73, 0.98)
0.93 (0.87, 1.00)	1.15E-01	0.85 (0.76, 0.94)	**7.47E-03**	0.92 (0.81, 1.04)
0.94 (0.88, 1.00)	1.30E-01	1.07 (0.97, 1.19)	3.78E-01	1.11 (0.97, 1.28)
0.92 (0.86, 0.99)	6.36E-02	1.02 (0.92, 1.13)	8.95E-01	1.07 (0.94, 1.21)
0.99 (0.92, 1.07)	8.96E-01	1.09 (0.98, 1.22)	2.58E-01	1.07 (0.93, 1.23)
1.09 (1.02, 1.16)	**3.21E-02**	0.99 (0.89, 1.09)	9.20E-01	0.93 (0.81, 1.07)
1.02 (0.95, 1.10)	7.86E-01	0.91 (0.81, 1.02)	2.37E-01	1.02 (0.90, 1.16)
0.92 (0.86, 0.98)	**3.21E-02**	1.02 (0.92, 1.13)	8.95E-01	0.99 (0.87, 1.12)
0.91 (0.85, 0.97)	**1.19E-02**	1.01 (0.91, 1.12)	9.48E-01	0.97 (0.85, 1.10)

1.02 (0.95, 1.11)	8.08E-01	1.04 (0.93, 1.15)	7.68E-01	1.07 (0.93, 1.22)
1.13 (1.06, 1.20)	**9.61E-04**	1.04 (0.94, 1.15)	7.34E-01	1.01 (0.88, 1.14)
1.01 (0.94, 1.09)	8.52E-01	0.91 (0.81, 1.02)	2.23E-01	0.91 (0.79, 1.05)
0.95 (0.88, 1.01)	2.78E-01	1.05 (0.94, 1.17)	6.71E-01	1.09 (0.95, 1.25)
0.95 (0.88, 1.02)	2.84E-01	1.04 (0.93, 1.16)	7.68E-01	1.07 (0.93, 1.22)
1.05 (0.97, 1.12)	4.33E-01	1.00 (0.90, 1.12)	9.48E-01	0.99 (0.87, 1.13)
1.13 (1.06, 1.21)	**1.10E-03**	1.05 (0.95, 1.16)	6.00E-01	0.94 (0.82, 1.08)
1.02 (0.95, 1.09)	8.15E-01	0.89 (0.80, 1.00)	1.48E-01	0.89 (0.77, 1.02)
0.96 (0.89, 1.03)	4.39E-01	1.06 (0.95, 1.19)	5.24E-01	1.10 (0.96, 1.27)
0.96 (0.89, 1.03)	4.42E-01	1.05 (0.94, 1.18)	6.39E-01	1.09 (0.95, 1.25)
1.03 (0.96, 1.11)	6.36E-01	1.01 (0.90, 1.13)	9.48E-01	0.98 (0.86, 1.11)
1.09 (1.02, 1.17)	**4.14E-02**	1.04 (0.93, 1.15)	7.61E-01	0.96 (0.84, 1.10)
1.01 (0.94, 1.09)	8.52E-01	0.79 (0.72, 0.87)	**9.01E-06**	0.82 (0.72, 0.93)
0.98 (0.91, 1.06)	8.15E-01	1.43 (1.28, 1.59)	**1.44E-09**	1.30 (1.14, 1.49)
0.98 (0.91, 1.06)	8.15E-01	1.41 (1.27, 1.55)	**3.59E-10**	1.32 (1.16, 1.50)
0.98 (0.92, 1.06)	8.15E-01	1.17 (1.04, 1.31)	**2.77E-02**	1.08 (0.95, 1.24)
1.01 (0.94, 1.08)	8.61E-01	0.90 (0.80, 1.00)	1.44E-01	0.96 (0.84, 1.09)
0.88 (0.82, 0.96)	**8.58E-03**	0.91 (0.81, 1.01)	2.01E-01	0.98 (0.86, 1.12)
1.08 (1.00, 1.17)	1.24E-01	1.16 (1.03, 1.30)	**3.36E-02**	1.07 (0.94, 1.23)
1.04 (0.96, 1.12)	5.62E-01	1.05 (0.94, 1.17)	6.58E-01	0.95 (0.84, 1.08)
1.12 (1.04, 1.21)	**1.73E-02**	1.31 (1.16, 1.49)	**1.09E-04**	1.26 (1.09, 1.46)
1.05 (0.98, 1.13)	2.95E-01	0.90 (0.80, 1.00)	1.45E-01	0.91 (0.79, 1.04)
1.23 (1.17, 1.30)	**3.54E-12**	1.20 (1.12, 1.28)	**1.44E-06**	1.14 (1.02, 1.27)
0.87 (0.81, 0.93)	**3.53E-04**	0.93 (0.85, 1.02)	3.01E-01	0.99 (0.87, 1.13)
0.85 (0.80, 0.91)	**7.22E-05**	0.93 (0.85, 1.03)	3.12E-01	1.00 (0.87, 1.15)
0.98 (0.91, 1.05)	8.15E-01	0.95 (0.86, 1.05)	6.00E-01	0.97 (0.85, 1.11)
1.00 (0.93, 1.08)	9.58E-01	0.89 (0.79, 0.99)	9.94E-02	0.88 (0.77, 1.01)
1.01 (0.94, 1.09)	8.91E-01	0.99 (0.89, 1.10)	9.23E-01	1.00 (0.87, 1.14)
0.99 (0.92, 1.06)	8.52E-01	1.03 (0.93, 1.15)	7.93E-01	1.03 (0.90, 1.18)
0.98 (0.91, 1.06)	8.15E-01	1.02 (0.92, 1.13)	8.95E-01	1.02 (0.89, 1.16)
1.05 (0.98, 1.13)	3.73E-01	1.09 (0.98, 1.21)	2.83E-01	1.08 (0.95, 1.23)
1.02 (0.95, 1.10)	8.15E-01	0.94 (0.84, 1.05)	4.95E-01	0.90 (0.79, 1.03)
0.95 (0.88, 1.02)	2.98E-01	0.96 (0.86, 1.07)	7.38E-01	1.01 (0.89, 1.14)
1.15 (1.07, 1.23)	**1.10E-03**	0.91 (0.82, 1.02)	2.43E-01	1.00 (0.87, 1.14)
1.16 (1.09, 1.25)	**2.64E-04**	1.22 (1.10, 1.35)	**7.44E-04**	1.15 (1.01, 1.30)
1.02 (0.95, 1.09)	8.15E-01	1.08 (0.98, 1.20)	2.83E-01	1.09 (0.96, 1.24)
1.02 (0.95, 1.10)	8.15E-01	1.12 (1.02, 1.24)	6.78E-02	1.12 (0.98, 1.27)
1.01 (0.94, 1.09)	8.52E-01	1.10 (0.99, 1.22)	1.77E-01	1.11 (0.98, 1.26)
0.97 (0.90, 1.05)	6.91E-01	1.03 (0.93, 1.15)	8.10E-01	1.06 (0.93, 1.21)
1.08 (1.00, 1.16)	1.15E-01	1.06 (0.96, 1.17)	4.74E-01	1.01 (0.89, 1.16)
1.07 (0.99, 1.15)	1.99E-01	1.03 (0.93, 1.14)	8.17E-01	0.99 (0.86, 1.13)
1.14 (1.07, 1.22)	**9.61E-04**	1.34 (1.22, 1.48)	**5.28E-08**	1.23 (1.09, 1.38)
0.94 (0.87, 1.01)	2.50E-01	0.81 (0.72, 0.91)	**2.17E-03**	0.87 (0.76, 1.00)
0.90 (0.83, 0.97)	**2.45E-02**	0.93 (0.83, 1.04)	4.30E-01	0.87 (0.76, 1.00)
1.00 (0.93, 1.08)	9.98E-01	1.02 (0.92, 1.13)	8.95E-01	1.00 (0.88, 1.15)
0.98 (0.91, 1.06)	8.15E-01	1.03 (0.93, 1.14)	8.17E-01	1.02 (0.89, 1.16)
1.11 (1.04, 1.20)	**1.13E-02**	1.04 (0.94, 1.15)	7.29E-01	0.99 (0.86, 1.13)
1.05 (0.98, 1.13)	3.88E-01	0.89 (0.79, 1.00)	1.14E-01	0.89 (0.78, 1.02)

0.95 (0.88, 1.03)	4.15E-01	1.05 (0.94, 1.16)	6.41E-01	0.90 (0.78, 1.02)
0.98 (0.91, 1.06)	8.15E-01	1.05 (0.95, 1.17)	6.02E-01	1.03 (0.90, 1.18)
0.86 (0.80, 0.93)	**9.61E-04**	0.85 (0.76, 0.94)	**9.23E-03**	0.74 (0.65, 0.84)
0.83 (0.77, 0.90)	**7.22E-05**	0.83 (0.74, 0.93)	**4.58E-03**	0.75 (0.65, 0.85)
0.92 (0.86, 1.00)	1.11E-01	0.98 (0.88, 1.09)	8.95E-01	0.83 (0.72, 0.94)
1.08 (1.00, 1.16)	1.16E-01	1.10 (1.00, 1.22)	1.43E-01	1.06 (0.93, 1.21)
0.98 (0.91, 1.06)	8.15E-01	1.05 (0.94, 1.17)	6.36E-01	1.07 (0.93, 1.22)
0.95 (0.88, 1.03)	4.15E-01	0.99 (0.89, 1.10)	9.48E-01	1.02 (0.90, 1.17)
0.91 (0.84, 0.98)	5.56E-02	0.98 (0.88, 1.09)	8.95E-01	0.86 (0.74, 0.99)
0.76 (0.71, 0.81)	**3.12E-15**	0.71 (0.64, 0.79)	**7.31E-10**	0.70 (0.63, 0.77)
1.06 (0.98, 1.14)	3.03E-01	1.47 (1.35, 1.60)	**1.97E-17**	1.42 (1.26, 1.60)
0.86 (0.79, 0.93)	**1.10E-03**	0.95 (0.85, 1.06)	6.39E-01	0.79 (0.69, 0.91)
0.97 (0.89, 1.05)	6.63E-01	1.25 (1.14, 1.37)	**4.18E-05**	1.16 (1.01, 1.32)
0.85 (0.79, 0.93)	**9.61E-04**	0.88 (0.79, 0.99)	1.02E-01	0.76 (0.66, 0.88)
0.86 (0.80, 0.94)	**2.49E-03**	0.95 (0.85, 1.06)	6.36E-01	0.81 (0.70, 0.94)
0.95 (0.88, 1.02)	3.53E-01	1.01 (0.91, 1.12)	9.48E-01	0.91 (0.79, 1.05)
0.92 (0.85, 0.99)	9.54E-02	0.99 (0.89, 1.10)	9.42E-01	0.87 (0.75, 1.00)
1.17 (1.09, 1.26)	**1.90E-04**	1.82 (1.64, 2.03)	**1.95E-25**	1.73 (1.55, 1.93)
0.84 (0.79, 0.90)	**7.22E-05**	0.92 (0.82, 1.02)	2.58E-01	0.78 (0.70, 0.87)
1.05 (0.98, 1.13)	3.88E-01	1.47 (1.33, 1.61)	**2.63E-13**	1.47 (1.31, 1.66)
0.86 (0.80, 0.92)	**3.53E-04**	0.80 (0.72, 0.89)	**4.25E-04**	0.74 (0.66, 0.83)
0.86 (0.80, 0.93)	**6.50E-04**	0.91 (0.82, 1.02)	2.45E-01	0.81 (0.72, 0.92)
1.10 (1.02, 1.18)	**3.24E-02**	1.07 (0.96, 1.19)	4.30E-01	1.18 (1.04, 1.33)
1.12 (1.04, 1.21)	**1.39E-02**	1.06 (0.95, 1.18)	5.82E-01	1.13 (0.99, 1.29)
1.13 (1.07, 1.20)	**2.64E-04**	1.20 (1.12, 1.28)	**1.88E-06**	1.06 (0.95, 1.18)
0.99 (0.92, 1.06)	8.81E-01	1.17 (1.07, 1.29)	**4.07E-03**	1.08 (0.97, 1.22)
0.99 (0.92, 1.06)	8.43E-01	0.82 (0.73, 0.92)	**2.98E-03**	0.79 (0.70, 0.90)
0.93 (0.86, 1.00)	1.15E-01	1.04 (0.94, 1.15)	7.43E-01	1.03 (0.91, 1.16)
0.89 (0.83, 0.96)	**1.43E-02**	0.94 (0.84, 1.04)	4.30E-01	1.02 (0.90, 1.16)
1.00 (0.93, 1.07)	9.58E-01	1.18 (1.07, 1.31)	**5.25E-03**	1.30 (1.15, 1.46)
0.94 (0.87, 1.01)	2.52E-01	0.99 (0.89, 1.10)	9.44E-01	1.02 (0.89, 1.16)
0.98 (0.91, 1.06)	8.15E-01	1.15 (1.04, 1.27)	**2.56E-02**	1.15 (1.02, 1.29)
0.83 (0.77, 0.90)	**1.12E-04**	0.84 (0.75, 0.94)	**9.49E-03**	0.88 (0.77, 1.01)
1.10 (1.05, 1.16)	**1.31E-03**	1.21 (1.15, 1.27)	**2.24E-12**	1.18 (1.11, 1.26)
1.00 (0.93, 1.08)	9.58E-01	1.14 (1.03, 1.26)	**4.22E-02**	1.06 (0.93, 1.20)
1.10 (1.04, 1.17)	**6.90E-03**	1.05 (0.97, 1.13)	4.81E-01	1.13 (1.01, 1.27)
1.11 (1.04, 1.18)	**3.88E-03**	0.81 (0.71, 0.92)	**4.94E-03**	0.91 (0.76, 1.09)
1.11 (1.05, 1.18)	**2.14E-03**	0.96 (0.85, 1.09)	7.91E-01	0.83 (0.66, 1.05)
1.11 (1.04, 1.18)	**3.70E-03**	1.04 (0.98, 1.11)	3.78E-01	1.11 (0.99, 1.23)
0.87 (0.81, 0.93)	**2.92E-04**	0.70 (0.65, 0.76)	**7.45E-17**	0.78 (0.71, 0.86)
1.14 (1.05, 1.23)	**4.97E-03**	1.41 (1.29, 1.55)	**4.97E-12**	1.34 (1.18, 1.52)

ıtus, alcohol consumption, and self-rated health status. P values are corrected for multiple t
LDL, Very Low Density Lipoprotein; IDL, Intermediate Density Lipoprotein..

	Cerebrovascular diseases mortality			
er	Never/former smoker		Current smoker	
P value	Adjusted HR (95% CI)	P value	Adjusted HR (95% CI)	P value
8.59E-01	0.84 (0.74, 0.95)	**2.15E-02**	0.97 (0.84, 1.12)	9.20E-01
8.72E-01	0.84 (0.74, 0.95)	**2.38E-02**	0.97 (0.84, 1.12)	9.33E-01
9.11E-01	0.85 (0.75, 0.96)	**3.12E-02**	0.99 (0.86, 1.13)	9.89E-01
9.68E-01	0.87 (0.77, 0.99)	7.13E-02	1.00 (0.87, 1.15)	9.89E-01
9.63E-01	0.89 (0.79, 1.00)	1.04E-01	1.00 (0.87, 1.17)	9.89E-01
9.04E-01	0.86 (0.76, 0.97)	**4.24E-02**	1.00 (0.87, 1.12)	9.89E-01
8.09E-01	0.83 (0.73, 0.95)	**1.75E-02**	0.96 (0.83, 1.11)	8.79E-01
8.89E-01	0.86 (0.76, 0.98)	5.15E-02	0.99 (0.86, 1.12)	9.89E-01
9.01E-01	0.86 (0.77, 0.98)	5.21E-02	0.99 (0.86, 1.12)	9.89E-01
9.18E-01	0.86 (0.76, 0.97)	**3.98E-02**	1.00 (0.86, 1.13)	9.89E-01
9.58E-01	0.88 (0.78, 0.99)	8.51E-02	1.00 (0.87, 1.15)	9.89E-01
9.61E-01	0.89 (0.79, 1.01)	1.21E-01	1.00 (0.87, 1.15)	9.89E-01
9.58E-01	0.86 (0.76, 0.98)	5.17E-02	1.00 (0.87, 1.14)	9.95E-01
8.72E-01	0.86 (0.76, 0.97)	**4.75E-02**	0.99 (0.86, 1.11)	9.89E-01
9.63E-01	0.88 (0.78, 0.99)	8.38E-02	1.00 (0.87, 1.13)	9.89E-01
9.63E-01	0.88 (0.78, 0.99)	8.38E-02	1.00 (0.87, 1.13)	9.89E-01
9.63E-01	0.88 (0.78, 0.99)	7.79E-02	1.01 (0.87, 1.13)	9.89E-01
9.38E-01	0.87 (0.77, 0.98)	6.17E-02	1.00 (0.87, 1.12)	9.89E-01
8.89E-01	0.86 (0.76, 0.97)	**4.66E-02**	0.99 (0.86, 1.12)	9.89E-01
9.63E-01	0.88 (0.78, 0.99)	8.38E-02	1.02 (0.88, 1.13)	9.89E-01
9.77E-01	0.88 (0.78, 1.00)	9.35E-02	1.00 (0.87, 1.13)	9.89E-01
9.18E-01	0.89 (0.79, 1.00)	1.13E-01	1.00 (0.87, 1.15)	9.89E-01
9.18E-01	0.89 (0.79, 1.00)	1.10E-01	1.00 (0.87, 1.14)	9.89E-01
9.38E-01	0.88 (0.78, 1.00)	9.03E-02	1.00 (0.87, 1.14)	9.89E-01
9.58E-01	0.88 (0.78, 1.00)	9.03E-02	1.01 (0.87, 1.14)	9.89E-01
9.91E-01	0.88 (0.78, 0.99)	7.28E-02	0.99 (0.85, 1.13)	9.89E-01
9.14E-01	0.90 (0.79, 1.01)	1.24E-01	1.02 (0.89, 1.15)	9.52E-01
9.04E-01	0.90 (0.80, 1.01)	1.29E-01	1.00 (0.87, 1.15)	9.89E-01
8.09E-01	0.90 (0.80, 1.01)	1.46E-01	1.03 (0.89, 1.17)	9.52E-01
6.68E-01	0.91 (0.81, 1.03)	2.02E-01	1.03 (0.89, 1.18)	9.29E-01
9.16E-01	0.87 (0.77, 0.99)	7.13E-02	1.04 (0.90, 1.15)	8.96E-01
5.23E-02	1.03 (0.92, 1.15)	7.17E-01	1.06 (0.92, 1.28)	7.36E-01
**1.39E-02**	1.08 (0.96, 1.21)	2.92E-01	1.04 (0.90, 1.31)	8.96E-01
4.42E-01	0.94 (0.84, 1.06)	4.61E-01	1.08 (0.94, 1.22)	5.88E-01
9.63E-01	0.87 (0.77, 0.98)	5.21E-02	1.01 (0.87, 1.12)	9.89E-01
1.73E-01	1.01 (0.90, 1.14)	8.66E-01	1.07 (0.93, 1.24)	6.24E-01
1.11E-01	1.04 (0.92, 1.16)	6.32E-01	1.07 (0.94, 1.25)	6.24E-01
7.10E-01	0.98 (0.87, 1.10)	8.15E-01	0.99 (0.86, 1.15)	9.89E-01
**3.70E-03**	1.14 (1.02, 1.27)	**4.75E-02**	1.11 (0.97, 1.35)	3.70E-01
**1.73E-03**	1.14 (1.02, 1.27)	**4.91E-02**	1.13 (0.98, 1.36)	2.50E-01

**2.54E-02**	1.13 (1.02, 1.26)	6.21E-02	1.06 (0.92, 1.31)	7.06E-01
9.63E-01	0.90 (0.79, 1.01)	1.43E-01	1.07 (0.93, 1.12)	6.31E-01
8.51E-01	0.95 (0.84, 1.07)	5.28E-01	0.99 (0.86, 1.13)	9.89E-01
7.97E-01	0.96 (0.85, 1.08)	6.13E-01	0.99 (0.86, 1.13)	9.89E-01
9.04E-01	0.95 (0.84, 1.07)	5.10E-01	0.96 (0.83, 1.10)	8.79E-01
6.57E-01	0.97 (0.86, 1.09)	7.18E-01	0.97 (0.84, 1.14)	9.20E-01
6.69E-01	0.95 (0.84, 1.07)	4.89E-01	0.96 (0.84, 1.13)	8.96E-01
6.42E-01	1.03 (0.92, 1.16)	6.98E-01	0.98 (0.85, 1.15)	9.52E-01
8.55E-01	0.93 (0.82, 1.05)	3.42E-01	1.16 (1.02, 1.12)	8.25E-02
8.87E-01	0.95 (0.84, 1.07)	4.89E-01	0.96 (0.83, 1.12)	8.79E-01
8.87E-01	0.95 (0.84, 1.07)	5.28E-01	0.95 (0.82, 1.12)	8.12E-01
9.58E-01	0.91 (0.81, 1.03)	2.01E-01	0.92 (0.80, 1.09)	5.84E-01
8.49E-01	0.97 (0.86, 1.09)	6.66E-01	0.94 (0.81, 1.13)	6.63E-01
8.31E-01	0.96 (0.85, 1.08)	5.85E-01	0.93 (0.81, 1.13)	6.35E-01
8.72E-01	0.99 (0.88, 1.11)	9.12E-01	0.95 (0.82, 1.12)	7.94E-01
9.58E-01	0.96 (0.85, 1.08)	5.88E-01	1.19 (1.06, 1.15)	**2.44E-02**
9.63E-01	0.94 (0.83, 1.06)	4.53E-01	0.93 (0.81, 1.11)	6.46E-01
9.58E-01	0.94 (0.84, 1.07)	4.66E-01	0.93 (0.81, 1.11)	6.24E-01
5.22E-01	0.83 (0.74, 0.94)	**9.54E-03**	0.92 (0.80, 1.03)	5.58E-01
8.31E-01	0.98 (0.87, 1.11)	8.28E-01	0.91 (0.79, 1.13)	5.15E-01
6.69E-01	1.00 (0.88, 1.12)	9.60E-01	0.91 (0.79, 1.14)	4.77E-01
9.04E-01	0.92 (0.82, 1.04)	2.73E-01	0.94 (0.81, 1.09)	6.35E-01
6.22E-01	0.94 (0.83, 1.06)	3.96E-01	1.20 (1.06, 1.13)	**2.67E-02**
9.11E-01	0.95 (0.84, 1.07)	4.89E-01	0.94 (0.81, 1.14)	6.72E-01
9.04E-01	0.95 (0.84, 1.07)	5.28E-01	0.93 (0.81, 1.14)	6.35E-01
4.58E-01	0.83 (0.75, 0.93)	**5.74E-03**	0.94 (0.82, 1.05)	6.34E-01
6.48E-01	1.00 (0.89, 1.13)	9.65E-01	0.92 (0.80, 1.16)	5.68E-01
5.27E-01	1.02 (0.90, 1.14)	8.28E-01	0.92 (0.79, 1.16)	5.50E-01
9.63E-01	0.94 (0.83, 1.06)	4.07E-01	0.94 (0.82, 1.14)	6.90E-01
9.38E-01	0.91 (0.80, 1.02)	1.75E-01	1.13 (0.99, 1.16)	2.21E-01
7.85E-02	1.20 (1.08, 1.33)	**2.46E-03**	1.26 (1.11, 1.37)	**4.77E-03**
7.51E-02	1.20 (1.09, 1.34)	**2.40E-03**	1.26 (1.11, 1.38)	**5.33E-03**
4.58E-01	1.17 (1.05, 1.29)	**1.71E-02**	1.23 (1.08, 1.28)	**1.65E-02**
**5.82E-03**	1.24 (1.11, 1.38)	**6.39E-04**	1.26 (1.11, 1.49)	**5.67E-03**
**1.30E-03**	1.26 (1.13, 1.40)	**2.18E-04**	1.28 (1.12, 1.54)	**4.15E-03**
1.11E-01	1.18 (1.06, 1.31)	**1.03E-02**	1.23 (1.08, 1.37)	**1.66E-02**
1.11E-01	1.09 (0.98, 1.22)	1.56E-01	1.25 (1.11, 1.31)	**3.45E-03**
8.87E-01	1.10 (0.99, 1.23)	1.26E-01	1.21 (1.06, 1.20)	**2.83E-02**
8.72E-01	1.11 (0.99, 1.23)	1.15E-01	1.21 (1.06, 1.20)	**2.98E-02**
8.51E-01	1.09 (0.98, 1.22)	1.73E-01	1.20 (1.05, 1.20)	**4.03E-02**
8.72E-01	1.12 (1.01, 1.25)	8.38E-02	1.20 (1.05, 1.21)	**3.87E-02**
9.04E-01	1.11 (1.00, 1.23)	1.13E-01	1.20 (1.05, 1.20)	**3.87E-02**
6.48E-01	1.15 (1.04, 1.28)	**2.67E-02**	1.20 (1.05, 1.24)	**4.03E-02**
8.89E-01	1.05 (0.94, 1.17)	5.28E-01	1.30 (1.15, 1.11)	**7.11E-04**
7.18E-02	0.76 (0.68, 0.85)	**1.35E-05**	0.97 (0.84, 0.89)	9.52E-01
7.85E-02	0.77 (0.69, 0.86)	**3.19E-05**	0.97 (0.84, 0.89)	9.42E-01
1.45E-01	0.79 (0.70, 0.88)	**2.61E-04**	1.04 (0.90, 0.93)	8.79E-01
1.08E-01	0.80 (0.72, 0.88)	**2.50E-04**	0.92 (0.79, 0.91)	5.88E-01

9.40E-02	0.79 (0.71, 0.88)	**1.55E-04**	0.90 (0.77, 0.90)	4.13E-01
1.93E-01	0.82 (0.73, 0.92)	**2.36E-03**	1.00 (0.86, 0.94)	9.89E-01
**1.39E-02**	0.69 (0.61, 0.79)	**6.54E-07**	0.97 (0.84, 0.86)	9.41E-01
**6.91E-04**	0.73 (0.67, 0.80)	**1.03E-10**	0.88 (0.77, 0.82)	2.58E-01
**1.30E-03**	0.74 (0.68, 0.80)	**2.40E-10**	0.88 (0.76, 0.83)	2.47E-01
**3.63E-02**	0.74 (0.67, 0.81)	**1.45E-07**	0.96 (0.83, 0.87)	8.79E-01
1.15E-01	0.86 (0.78, 0.96)	**2.82E-02**	0.87 (0.76, 0.93)	1.26E-01
2.46E-01	0.91 (0.82, 1.02)	1.56E-01	0.87 (0.76, 0.97)	1.34E-01
9.40E-02	0.77 (0.70, 0.86)	**1.33E-05**	0.98 (0.84, 0.90)	9.89E-01
5.23E-02	0.78 (0.69, 0.88)	**6.95E-04**	1.03 (0.89, 0.94)	9.52E-01
7.67E-01	0.98 (0.87, 1.09)	7.40E-01	1.07 (0.91, 1.08)	7.04E-01
1.26E-01	1.16 (1.08, 1.25)	**2.49E-04**	1.17 (1.04, 1.22)	**4.03E-02**
1.32E-01	1.17 (1.09, 1.25)	**8.03E-05**	1.15 (1.02, 1.23)	1.00E-01
7.67E-01	1.05 (0.95, 1.17)	4.63E-01	1.14 (0.98, 1.13)	2.58E-01
1.31E-01	0.85 (0.78, 0.93)	**1.71E-03**	0.84 (0.73, 0.98)	**4.28E-02**
6.22E-01	0.86 (0.78, 0.95)	**1.55E-02**	0.96 (0.84, 1.09)	8.79E-01
1.93E-01	1.19 (1.09, 1.28)	**2.61E-04**	1.05 (0.92, 1.24)	7.36E-01
4.22E-01	1.23 (1.14, 1.32)	**1.12E-06**	1.09 (0.95, 1.23)	5.50E-01
4.91E-01	0.94 (0.84, 1.05)	3.69E-01	0.98 (0.85, 1.17)	9.89E-01
1.56E-01	0.93 (0.83, 1.05)	3.76E-01	0.98 (0.85, 0.99)	9.89E-01
7.51E-01	0.95 (0.85, 1.06)	4.99E-01	1.00 (0.87, 0.98)	9.89E-01
6.22E-01	0.95 (0.85, 1.06)	4.99E-01	0.95 (0.83, 0.96)	7.06E-01
2.07E-01	0.96 (0.85, 1.08)	5.85E-01	0.89 (0.77, 0.98)	3.06E-01
8.87E-01	0.98 (0.87, 1.10)	7.91E-01	1.03 (0.89, 1.05)	9.42E-01
6.22E-01	1.05 (0.94, 1.18)	4.63E-01	1.05 (0.91, 1.27)	8.12E-01
**1.93E-02**	0.86 (0.76, 0.97)	**3.98E-02**	0.92 (0.79, 0.89)	5.58E-01
3.53E-01	0.99 (0.88, 1.11)	8.80E-01	0.99 (0.86, 1.00)	9.89E-01
2.01E-01	0.98 (0.87, 1.10)	8.05E-01	0.95 (0.82, 1.00)	7.36E-01
6.57E-01	1.03 (0.91, 1.15)	7.40E-01	1.14 (0.98, 1.16)	2.50E-01
1.93E-01	1.04 (0.92, 1.17)	6.52E-01	1.03 (0.89, 1.28)	9.52E-01
**5.01E-03**	0.81 (0.72, 0.91)	**2.68E-03**	1.02 (0.88, 0.91)	9.89E-01
**1.47E-02**	1.25 (1.11, 1.40)	**1.04E-03**	1.07 (0.93, 1.36)	6.24E-01
**1.93E-02**	1.23 (1.10, 1.38)	**2.36E-03**	1.04 (0.90, 1.34)	8.79E-01
7.18E-02	1.23 (1.10, 1.36)	**1.02E-03**	1.27 (1.12, 1.41)	**4.15E-03**
1.11E-01	0.84 (0.75, 0.95)	**1.47E-02**	0.93 (0.81, 0.95)	6.24E-01
**1.19E-02**	0.88 (0.79, 0.97)	**2.62E-02**	0.86 (0.76, 0.92)	**4.03E-02**
**1.53E-05**	1.36 (1.20, 1.54)	**3.31E-05**	1.19 (1.02, 1.61)	1.07E-01
**1.82E-05**	1.27 (1.13, 1.43)	**6.66E-04**	1.21 (1.04, 1.52)	5.29E-02
**4.94E-02**	1.33 (1.17, 1.52)	**2.35E-04**	1.03 (0.90, 1.51)	9.33E-01
9.40E-02	0.86 (0.76, 0.97)	**4.38E-02**	0.98 (0.85, 0.95)	9.70E-01
4.22E-01	0.97 (0.87, 1.09)	7.02E-01	0.87 (0.76, 0.94)	1.29E-01
3.47E-01	1.07 (0.96, 1.20)	3.34E-01	0.89 (0.80, 1.19)	1.81E-01
6.04E-01	0.94 (0.85, 1.05)	3.95E-01	0.90 (0.79, 1.13)	2.46E-01
6.33E-01	1.21 (1.06, 1.38)	**1.50E-02**	0.95 (0.84, 1.22)	7.36E-01
6.22E-01	0.94 (0.84, 1.06)	4.41E-01	1.16 (1.04, 1.09)	**4.48E-02**
9.18E-01	0.91 (0.81, 1.03)	2.16E-01	0.95 (0.82, 1.02)	8.24E-01
9.58E-01	1.06 (0.94, 1.19)	4.61E-01	0.91 (0.81, 1.13)	3.32E-01
8.72E-01	1.00 (0.90, 1.12)	9.88E-01	0.91 (0.80, 1.12)	2.69E-01

6.54E-01	1.18 (1.05, 1.34)	**2.09E-02**	1.00 (0.87, 1.15)	9.89E-01
9.63E-01	0.98 (0.88, 1.10)	8.28E-01	1.18 (1.06, 1.15)	**1.66E-02**
4.22E-01	0.84 (0.74, 0.95)	**2.11E-02**	1.06 (0.93, 1.02)	6.54E-01
4.58E-01	1.14 (1.01, 1.29)	7.79E-02	0.87 (0.77, 1.17)	1.21E-01
6.22E-01	1.11 (0.99, 1.25)	1.47E-01	0.87 (0.77, 1.16)	1.29E-01
9.63E-01	0.98 (0.88, 1.10)	8.20E-01	1.12 (0.98, 1.12)	2.58E-01
6.77E-01	0.97 (0.87, 1.09)	7.10E-01	1.27 (1.14, 1.16)	**6.38E-04**
2.54E-01	0.84 (0.74, 0.95)	**2.09E-02**	1.07 (0.93, 1.00)	6.34E-01
3.99E-01	1.16 (1.02, 1.31)	5.31E-02	0.90 (0.79, 1.19)	2.58E-01
4.58E-01	1.13 (1.00, 1.28)	9.35E-02	0.89 (0.78, 1.18)	2.46E-01
9.11E-01	0.99 (0.88, 1.11)	8.66E-01	1.12 (0.98, 1.13)	2.69E-01
8.55E-01	0.94 (0.84, 1.06)	4.17E-01	1.19 (1.06, 1.15)	**2.63E-02**
**1.39E-02**	0.88 (0.79, 0.98)	5.21E-02	0.98 (0.85, 0.87)	9.89E-01
**1.31E-03**	1.23 (1.09, 1.38)	**2.68E-03**	1.01 (0.88, 1.59)	9.89E-01
**5.14E-04**	1.24 (1.11, 1.39)	**1.04E-03**	1.02 (0.88, 1.55)	9.89E-01
5.13E-01	1.03 (0.92, 1.16)	6.66E-01	0.99 (0.86, 1.31)	9.89E-01
8.09E-01	0.89 (0.78, 1.00)	1.13E-01	1.02 (0.89, 1.00)	9.89E-01
9.38E-01	0.89 (0.79, 1.00)	9.97E-02	0.84 (0.73, 1.01)	1.07E-01
6.22E-01	1.20 (1.06, 1.37)	**2.00E-02**	1.08 (0.93, 1.30)	6.24E-01
7.51E-01	1.08 (0.96, 1.22)	3.11E-01	1.03 (0.89, 1.17)	9.42E-01
**1.38E-02**	1.35 (1.18, 1.55)	**2.45E-04**	1.14 (0.97, 1.49)	2.76E-01
4.09E-01	0.89 (0.78, 1.01)	1.16E-01	1.13 (1.00, 1.00)	1.74E-01
7.85E-02	1.19 (1.11, 1.28)	**1.35E-05**	1.29 (1.20, 1.28)	**3.87E-09**
9.63E-01	0.93 (0.84, 1.04)	2.98E-01	0.81 (0.73, 1.02)	**1.87E-03**
9.91E-01	0.94 (0.85, 1.04)	3.20E-01	0.80 (0.73, 1.03)	**2.05E-04**
8.89E-01	0.95 (0.85, 1.06)	4.69E-01	1.00 (0.86, 1.05)	9.89E-01
2.02E-01	0.87 (0.77, 0.99)	7.04E-02	1.02 (0.88, 0.99)	9.89E-01
9.71E-01	0.90 (0.80, 1.01)	1.14E-01	1.07 (0.94, 1.10)	6.24E-01
8.87E-01	1.13 (1.01, 1.27)	8.38E-02	0.93 (0.81, 1.15)	5.58E-01
9.25E-01	1.12 (0.99, 1.25)	1.21E-01	0.92 (0.81, 1.13)	5.15E-01
5.21E-01	1.03 (0.92, 1.15)	7.18E-01	1.14 (0.99, 1.21)	2.05E-01
3.53E-01	0.91 (0.81, 1.02)	1.81E-01	1.08 (0.94, 1.05)	5.77E-01
9.63E-01	0.91 (0.82, 1.02)	1.88E-01	0.97 (0.84, 1.07)	9.25E-01
9.71E-01	0.94 (0.84, 1.05)	3.62E-01	1.21 (1.06, 1.02)	**2.55E-02**
1.11E-01	1.23 (1.11, 1.38)	**1.02E-03**	1.26 (1.10, 1.35)	**8.95E-03**
4.40E-01	0.99 (0.88, 1.11)	8.82E-01	1.01 (0.88, 1.20)	9.89E-01
2.32E-01	0.96 (0.86, 1.08)	6.25E-01	1.05 (0.91, 1.24)	7.91E-01
2.72E-01	0.96 (0.85, 1.08)	6.24E-01	1.02 (0.88, 1.22)	9.89E-01
6.47E-01	0.99 (0.88, 1.11)	8.82E-01	0.93 (0.80, 1.15)	5.97E-01
9.58E-01	1.03 (0.92, 1.15)	6.83E-01	1.13 (0.98, 1.17)	2.56E-01
9.58E-01	1.01 (0.90, 1.13)	9.11E-01	1.10 (0.96, 1.14)	3.98E-01
**7.46E-03**	1.22 (1.09, 1.36)	**1.79E-03**	1.30 (1.13, 1.48)	**3.45E-03**
1.62E-01	0.78 (0.69, 0.88)	**6.44E-04**	0.93 (0.81, 0.91)	6.36E-01
1.73E-01	0.88 (0.78, 1.00)	1.03E-01	0.80 (0.69, 1.04)	**2.29E-02**
9.71E-01	0.88 (0.78, 1.00)	9.40E-02	1.04 (0.91, 1.13)	8.54E-01
9.45E-01	0.89 (0.79, 1.00)	1.03E-01	1.01 (0.88, 1.14)	9.89E-01
9.58E-01	0.95 (0.84, 1.07)	5.28E-01	1.20 (1.06, 1.15)	**2.29E-02**
2.46E-01	0.84 (0.75, 0.95)	**2.09E-02**	1.15 (1.01, 1.00)	1.35E-01

2.72E-01	0.93 (0.83, 1.04)	3.13E-01	0.90 (0.78, 1.16)	3.85E-01
8.87E-01	0.94 (0.84, 1.06)	4.53E-01	0.99 (0.86, 1.17)	9.89E-01
**1.21E-04**	0.80 (0.72, 0.90)	**1.45E-03**	0.79 (0.69, 0.94)	**1.05E-02**
**3.49E-04**	0.80 (0.71, 0.90)	**1.52E-03**	0.75 (0.65, 0.93)	**1.03E-03**
**2.80E-02**	0.92 (0.82, 1.03)	2.24E-01	0.85 (0.74, 1.09)	1.00E-01
6.51E-01	1.01 (0.91, 1.13)	8.82E-01	1.13 (0.99, 1.22)	2.33E-01
6.30E-01	0.92 (0.81, 1.03)	2.50E-01	0.97 (0.84, 1.17)	9.42E-01
9.11E-01	0.92 (0.82, 1.03)	2.46E-01	0.92 (0.79, 1.10)	5.50E-01
1.26E-01	0.83 (0.74, 0.95)	**1.75E-02**	0.86 (0.74, 1.09)	1.75E-01
**2.31E-10**	0.80 (0.72, 0.89)	**2.61E-04**	0.70 (0.63, 0.79)	**2.49E-10**
**6.71E-07**	1.29 (1.17, 1.43)	**1.10E-05**	1.05 (0.91, 1.60)	7.96E-01
**9.52E-03**	0.85 (0.75, 0.96)	**3.12E-02**	0.81 (0.70, 1.06)	**2.39E-02**
1.15E-01	1.06 (0.95, 1.19)	3.95E-01	0.95 (0.81, 1.37)	7.91E-01
**1.93E-03**	0.79 (0.69, 0.90)	**1.78E-03**	0.80 (0.69, 0.99)	**2.29E-02**
**2.64E-02**	0.83 (0.73, 0.94)	**1.56E-02**	0.81 (0.69, 1.06)	**3.70E-02**
4.58E-01	0.86 (0.76, 0.97)	**3.98E-02**	0.94 (0.81, 1.12)	7.36E-01
1.53E-01	0.84 (0.74, 0.95)	**1.86E-02**	0.85 (0.73, 1.10)	1.21E-01
**2.32E-20**	1.64 (1.46, 1.85)	**6.08E-14**	1.25 (1.09, 2.03)	**1.57E-02**
**1.21E-04**	1.00 (0.89, 1.12)	9.72E-01	0.82 (0.73, 1.02)	**5.67E-03**
**1.29E-08**	1.31 (1.18, 1.46)	**1.33E-05**	1.09 (0.95, 1.61)	5.17E-01
**2.24E-05**	0.90 (0.80, 1.01)	1.16E-01	0.82 (0.72, 0.89)	**2.28E-02**
**9.13E-03**	0.98 (0.87, 1.10)	8.05E-01	0.83 (0.72, 1.02)	**2.98E-02**
**4.31E-02**	0.99 (0.89, 1.11)	9.32E-01	1.23 (1.08, 1.19)	**1.66E-02**
1.93E-01	1.04 (0.93, 1.17)	5.77E-01	1.04 (0.89, 1.18)	9.14E-01
6.22E-01	1.12 (1.02, 1.22)	**3.60E-02**	1.30 (1.20, 1.28)	**7.08E-09**
4.09E-01	1.00 (0.90, 1.11)	9.73E-01	1.14 (1.01, 1.29)	1.34E-01
**4.74E-03**	0.79 (0.70, 0.90)	**1.45E-03**	0.90 (0.78, 0.92)	4.26E-01
8.87E-01	1.01 (0.91, 1.13)	8.82E-01	0.91 (0.79, 1.15)	5.15E-01
9.18E-01	1.10 (0.98, 1.22)	1.60E-01	0.93 (0.81, 1.04)	6.34E-01
**3.75E-04**	1.22 (1.10, 1.34)	**1.02E-03**	1.03 (0.89, 1.31)	9.42E-01
9.38E-01	0.89 (0.79, 1.00)	9.40E-02	0.96 (0.83, 1.10)	8.96E-01
1.11E-01	0.97 (0.86, 1.08)	6.45E-01	0.99 (0.86, 1.27)	9.89E-01
2.01E-01	0.78 (0.69, 0.88)	**6.39E-04**	0.77 (0.66, 0.94)	**8.95E-03**
**6.75E-06**	1.21 (1.15, 1.27)	**2.37E-11**	1.09 (1.00, 1.27)	1.75E-01
6.62E-01	1.20 (1.08, 1.34)	**2.95E-03**	0.99 (0.86, 1.26)	9.89E-01
1.11E-01	1.07 (1.01, 1.14)	6.21E-02	1.22 (1.12, 1.13)	**3.91E-04**
6.04E-01	0.80 (0.70, 0.91)	**3.67E-03**	1.12 (1.03, 0.92)	**4.28E-02**
3.05E-01	0.84 (0.72, 0.99)	8.38E-02	1.12 (1.01, 1.09)	1.10E-01
1.93E-01	1.07 (1.02, 1.13)	**3.07E-02**	1.19 (1.12, 1.11)	**1.26E-06**
**3.92E-05**	0.77 (0.70, 0.84)	**1.52E-07**	0.78 (0.69, 0.76)	**8.89E-04**
**1.21E-04**	1.17 (1.05, 1.30)	**1.71E-02**	1.23 (1.07, 1.55)	**2.21E-02**

Cancer mortality				
Never/former smoker		Current smoker		Never/former sm
Adjusted HR (95% CI)	P value	Adjusted HR (95% CI)	P value	Adjusted HR (95% CI)
0.92 (0.83, 1.01)	3.73E-01	0.98 (0.84, 1.14)	8.59E-01	1.08 (0.92, 1.28)
0.92 (0.83, 1.02)	3.75E-01	0.98 (0.84, 1.14)	8.75E-01	1.08 (0.92, 1.28)
0.92 (0.83, 1.01)	3.75E-01	0.99 (0.85, 1.15)	9.42E-01	1.08 (0.92, 1.27)
0.97 (0.88, 1.06)	8.06E-01	1.01 (0.87, 1.17)	9.80E-01	1.09 (0.93, 1.28)
0.98 (0.90, 1.08)	9.13E-01	1.01 (0.88, 1.17)	9.42E-01	1.08 (0.91, 1.27)
0.95 (0.86, 1.04)	6.12E-01	1.01 (0.87, 1.17)	9.61E-01	1.10 (0.94, 1.29)
0.91 (0.82, 1.01)	3.16E-01	0.97 (0.83, 1.13)	8.22E-01	1.08 (0.92, 1.27)
0.96 (0.87, 1.06)	7.91E-01	1.04 (0.89, 1.20)	8.22E-01	1.07 (0.91, 1.26)
0.96 (0.88, 1.06)	7.96E-01	1.04 (0.89, 1.20)	8.22E-01	1.07 (0.91, 1.26)
0.96 (0.88, 1.06)	7.96E-01	1.03 (0.89, 1.20)	8.22E-01	1.08 (0.91, 1.27)
0.98 (0.89, 1.08)	9.03E-01	1.03 (0.89, 1.19)	8.37E-01	1.07 (0.90, 1.26)
0.98 (0.90, 1.08)	9.13E-01	1.02 (0.88, 1.19)	8.60E-01	1.06 (0.90, 1.25)
0.98 (0.89, 1.08)	9.05E-01	1.03 (0.89, 1.19)	8.37E-01	1.08 (0.91, 1.27)
0.96 (0.87, 1.05)	7.48E-01	1.04 (0.90, 1.20)	8.19E-01	1.07 (0.91, 1.27)
0.94 (0.85, 1.03)	4.47E-01	1.00 (0.86, 1.16)	9.98E-01	1.04 (0.88, 1.23)
0.94 (0.85, 1.03)	4.56E-01	1.00 (0.86, 1.16)	9.98E-01	1.04 (0.88, 1.23)
0.94 (0.85, 1.03)	4.56E-01	1.00 (0.86, 1.16)	9.98E-01	1.04 (0.88, 1.23)
0.95 (0.87, 1.04)	6.40E-01	1.00 (0.87, 1.16)	9.98E-01	1.02 (0.87, 1.21)
0.96 (0.88, 1.05)	7.91E-01	1.00 (0.86, 1.15)	9.98E-01	0.99 (0.84, 1.18)
0.94 (0.86, 1.03)	5.02E-01	1.01 (0.87, 1.16)	9.85E-01	1.05 (0.89, 1.24)
0.93 (0.85, 1.02)	4.23E-01	1.00 (0.86, 1.16)	9.98E-01	1.05 (0.89, 1.23)
0.94 (0.86, 1.03)	4.47E-01	0.97 (0.84, 1.12)	8.22E-01	0.99 (0.84, 1.17)
0.94 (0.86, 1.03)	4.56E-01	0.97 (0.84, 1.12)	8.22E-01	0.99 (0.83, 1.16)
0.94 (0.86, 1.03)	4.56E-01	0.97 (0.84, 1.12)	8.22E-01	0.98 (0.83, 1.16)
0.97 (0.89, 1.06)	8.06E-01	0.98 (0.85, 1.12)	8.59E-01	0.97 (0.82, 1.14)
0.99 (0.91, 1.08)	9.47E-01	0.98 (0.85, 1.12)	8.41E-01	0.94 (0.79, 1.11)
0.95 (0.87, 1.04)	5.54E-01	0.98 (0.85, 1.13)	9.00E-01	1.00 (0.85, 1.18)
0.93 (0.85, 1.02)	3.75E-01	0.97 (0.84, 1.12)	8.22E-01	1.00 (0.84, 1.18)
0.97 (0.89, 1.05)	7.91E-01	0.95 (0.83, 1.09)	7.04E-01	0.93 (0.78, 1.10)
0.97 (0.89, 1.05)	7.96E-01	0.95 (0.82, 1.09)	6.81E-01	0.92 (0.78, 1.09)
0.96 (0.88, 1.04)	6.84E-01	0.95 (0.83, 1.09)	7.05E-01	0.90 (0.76, 1.07)
1.00 (0.92, 1.09)	9.54E-01	0.92 (0.80, 1.05)	4.58E-01	0.89 (0.75, 1.05)
1.02 (0.94, 1.11)	8.98E-01	0.91 (0.79, 1.03)	3.64E-01	0.88 (0.75, 1.04)
0.98 (0.90, 1.06)	8.81E-01	0.95 (0.83, 1.09)	7.01E-01	0.92 (0.77, 1.08)
0.95 (0.88, 1.04)	6.18E-01	0.97 (0.84, 1.12)	8.29E-01	0.96 (0.81, 1.13)
1.01 (0.93, 1.09)	9.52E-01	0.94 (0.82, 1.08)	6.54E-01	0.92 (0.77, 1.09)
1.01 (0.93, 1.09)	9.49E-01	0.93 (0.82, 1.06)	5.85E-01	0.92 (0.78, 1.09)
1.02 (0.94, 1.11)	8.98E-01	0.95 (0.83, 1.09)	7.01E-01	0.89 (0.75, 1.06)
1.01 (0.94, 1.10)	9.13E-01	0.90 (0.79, 1.02)	3.17E-01	0.95 (0.81, 1.12)
1.01 (0.93, 1.10)	9.13E-01	0.89 (0.79, 1.02)	2.99E-01	0.97 (0.83, 1.14)

1.02 (0.94, 1.10)	8.98E-01	0.91 (0.80, 1.04)	3.66E-01	0.92 (0.78, 1.08)
0.98 (0.90, 1.07)	8.98E-01	1.02 (0.89, 1.17)	8.41E-01	0.94 (0.79, 1.11)
1.02 (0.94, 1.11)	8.78E-01	0.95 (0.83, 1.08)	6.81E-01	0.91 (0.77, 1.08)
1.03 (0.95, 1.11)	8.57E-01	0.94 (0.83, 1.08)	6.54E-01	0.91 (0.77, 1.08)
1.02 (0.94, 1.11)	8.81E-01	0.93 (0.82, 1.07)	6.12E-01	0.88 (0.74, 1.04)
1.03 (0.95, 1.12)	8.27E-01	0.93 (0.82, 1.06)	5.90E-01	0.90 (0.76, 1.06)
1.02 (0.94, 1.11)	8.93E-01	0.93 (0.81, 1.06)	5.61E-01	0.89 (0.75, 1.06)
1.04 (0.96, 1.13)	6.70E-01	0.94 (0.83, 1.08)	6.54E-01	0.91 (0.77, 1.07)
1.01 (0.92, 1.10)	9.52E-01	1.09 (0.96, 1.25)	4.13E-01	1.01 (0.86, 1.19)
1.01 (0.92, 1.09)	9.52E-01	0.94 (0.82, 1.08)	6.62E-01	0.86 (0.72, 1.03)
1.01 (0.93, 1.09)	9.52E-01	0.94 (0.82, 1.07)	6.38E-01	0.86 (0.72, 1.02)
1.01 (0.92, 1.09)	9.52E-01	0.94 (0.82, 1.07)	6.38E-01	0.84 (0.70, 1.00)
1.01 (0.92, 1.09)	9.52E-01	0.92 (0.81, 1.06)	5.05E-01	0.85 (0.72, 1.02)
1.00 (0.92, 1.09)	9.90E-01	0.92 (0.80, 1.05)	4.70E-01	0.85 (0.71, 1.01)
1.02 (0.94, 1.11)	8.84E-01	0.94 (0.82, 1.08)	6.54E-01	0.87 (0.74, 1.04)
1.02 (0.93, 1.11)	9.04E-01	1.12 (0.98, 1.27)	2.86E-01	1.03 (0.88, 1.21)
0.98 (0.90, 1.07)	8.98E-01	0.91 (0.79, 1.04)	4.05E-01	0.82 (0.68, 0.98)
0.98 (0.90, 1.07)	8.98E-01	0.91 (0.79, 1.04)	3.74E-01	0.82 (0.68, 0.98)
0.97 (0.89, 1.06)	8.38E-01	0.91 (0.80, 1.05)	4.53E-01	0.81 (0.68, 0.96)
0.98 (0.90, 1.07)	9.03E-01	0.89 (0.78, 1.02)	3.20E-01	0.82 (0.68, 0.98)
0.98 (0.90, 1.07)	8.98E-01	0.89 (0.78, 1.02)	3.19E-01	0.82 (0.69, 0.98)
0.99 (0.90, 1.08)	9.13E-01	0.90 (0.78, 1.03)	3.52E-01	0.81 (0.68, 0.97)
1.01 (0.92, 1.10)	9.52E-01	1.09 (0.95, 1.25)	4.70E-01	1.00 (0.85, 1.19)
0.97 (0.89, 1.06)	7.96E-01	0.90 (0.78, 1.03)	3.40E-01	0.80 (0.67, 0.96)
0.97 (0.89, 1.06)	7.96E-01	0.90 (0.78, 1.03)	3.27E-01	0.80 (0.67, 0.96)
0.95 (0.87, 1.05)	6.70E-01	0.92 (0.80, 1.06)	5.02E-01	0.80 (0.67, 0.95)
0.97 (0.89, 1.06)	8.59E-01	0.88 (0.77, 1.01)	2.73E-01	0.81 (0.68, 0.97)
0.97 (0.89, 1.06)	8.57E-01	0.89 (0.77, 1.01)	2.79E-01	0.81 (0.68, 0.97)
0.97 (0.88, 1.05)	7.96E-01	0.89 (0.77, 1.02)	2.86E-01	0.80 (0.67, 0.95)
0.97 (0.89, 1.06)	8.60E-01	1.03 (0.89, 1.18)	8.41E-01	0.96 (0.81, 1.14)
1.11 (1.03, 1.20)	7.30E-02	1.13 (1.00, 1.27)	1.81E-01	1.17 (1.01, 1.37)
1.11 (1.03, 1.20)	7.63E-02	1.12 (1.00, 1.26)	2.02E-01	1.17 (1.00, 1.36)
1.14 (1.06, 1.23)	**1.20E-02**	1.18 (1.05, 1.32)	5.92E-02	1.17 (1.00, 1.36)
1.07 (0.99, 1.16)	3.75E-01	1.06 (0.94, 1.20)	6.15E-01	1.15 (0.98, 1.34)
1.05 (0.97, 1.14)	5.43E-01	1.04 (0.92, 1.17)	7.61E-01	1.14 (0.98, 1.34)
1.11 (1.03, 1.20)	5.53E-02	1.12 (1.00, 1.26)	2.12E-01	1.16 (0.99, 1.35)
1.04 (0.96, 1.14)	6.84E-01	1.08 (0.95, 1.23)	4.75E-01	1.23 (1.06, 1.44)
1.11 (1.02, 1.20)	8.63E-02	1.18 (1.04, 1.33)	6.41E-02	1.16 (0.99, 1.36)
1.11 (1.02, 1.20)	8.63E-02	1.18 (1.04, 1.33)	6.41E-02	1.16 (0.99, 1.36)
1.10 (1.01, 1.19)	1.31E-01	1.16 (1.03, 1.32)	9.16E-02	1.12 (0.96, 1.32)
1.11 (1.02, 1.21)	7.63E-02	1.18 (1.04, 1.33)	6.11E-02	1.17 (1.00, 1.37)
1.11 (1.02, 1.21)	7.63E-02	1.18 (1.05, 1.34)	5.92E-02	1.17 (1.00, 1.38)
1.11 (1.02, 1.20)	7.63E-02	1.17 (1.03, 1.32)	8.23E-02	1.15 (0.98, 1.35)
1.09 (1.00, 1.19)	2.24E-01	1.17 (1.04, 1.32)	6.11E-02	1.23 (1.06, 1.44)
1.00 (0.92, 1.09)	9.54E-01	1.06 (0.92, 1.21)	6.81E-01	0.85 (0.72, 1.01)
1.01 (0.92, 1.10)	9.52E-01	1.06 (0.92, 1.21)	6.81E-01	0.85 (0.72, 1.01)
1.00 (0.91, 1.09)	9.54E-01	1.07 (0.94, 1.23)	5.85E-01	0.89 (0.75, 1.05)
1.02 (0.93, 1.11)	9.13E-01	1.03 (0.90, 1.19)	8.22E-01	0.85 (0.72, 0.99)

1.01 (0.93, 1.11)	9.13E-01	1.02 (0.89, 1.18)	8.41E-01	0.84 (0.72, 0.99)
1.02 (0.94, 1.12)	8.81E-01	1.06 (0.92, 1.21)	6.81E-01	0.87 (0.74, 1.03)
0.99 (0.90, 1.08)	9.13E-01	1.06 (0.93, 1.20)	6.54E-01	0.90 (0.76, 1.07)
0.93 (0.85, 1.02)	4.01E-01	1.01 (0.88, 1.16)	9.63E-01	0.73 (0.63, 0.85)
0.94 (0.86, 1.02)	4.35E-01	1.00 (0.88, 1.15)	9.98E-01	0.73 (0.63, 0.84)
0.92 (0.85, 1.01)	3.33E-01	1.03 (0.90, 1.18)	8.22E-01	0.84 (0.71, 0.98)
1.00 (0.92, 1.10)	9.54E-01	0.96 (0.84, 1.10)	7.61E-01	0.73 (0.62, 0.85)
1.01 (0.93, 1.11)	9.13E-01	0.95 (0.83, 1.09)	7.05E-01	0.76 (0.65, 0.89)
0.95 (0.87, 1.04)	6.26E-01	1.04 (0.90, 1.19)	8.18E-01	0.81 (0.69, 0.95)
0.94 (0.87, 1.03)	4.83E-01	1.05 (0.92, 1.20)	6.81E-01	0.95 (0.80, 1.13)
0.87 (0.80, 0.95)	**1.72E-02**	0.86 (0.77, 0.97)	8.29E-02	1.01 (0.84, 1.21)
1.15 (1.06, 1.24)	**9.66E-03**	1.19 (1.07, 1.32)	**2.31E-02**	1.11 (0.95, 1.29)
1.14 (1.05, 1.24)	**1.30E-02**	1.18 (1.06, 1.31)	**2.63E-02**	1.08 (0.92, 1.27)
1.07 (0.97, 1.18)	4.74E-01	1.10 (0.97, 1.25)	3.40E-01	1.13 (0.97, 1.33)
0.93 (0.84, 1.02)	4.33E-01	0.89 (0.78, 1.01)	2.59E-01	0.91 (0.77, 1.07)
0.92 (0.84, 1.01)	3.27E-01	0.96 (0.85, 1.10)	7.79E-01	1.01 (0.84, 1.23)
1.05 (0.98, 1.13)	4.40E-01	1.06 (0.96, 1.18)	4.91E-01	1.10 (0.95, 1.26)
1.05 (0.98, 1.12)	4.35E-01	1.06 (0.94, 1.19)	6.30E-01	1.05 (0.90, 1.23)
1.02 (0.92, 1.12)	9.13E-01	1.04 (0.93, 1.18)	7.07E-01	1.12 (0.92, 1.35)
0.99 (0.90, 1.08)	9.30E-01	0.96 (0.85, 1.09)	7.61E-01	0.91 (0.76, 1.09)
0.96 (0.87, 1.06)	7.96E-01	0.94 (0.83, 1.07)	6.27E-01	1.02 (0.85, 1.24)
1.10 (0.98, 1.22)	3.75E-01	1.00 (0.88, 1.13)	9.98E-01	1.04 (0.86, 1.26)
1.16 (1.06, 1.26)	**9.60E-03**	1.10 (0.98, 1.25)	3.27E-01	1.00 (0.84, 1.19)
0.93 (0.85, 1.02)	4.33E-01	0.91 (0.80, 1.02)	3.19E-01	1.05 (0.87, 1.27)
0.94 (0.84, 1.05)	5.87E-01	1.02 (0.90, 1.15)	8.83E-01	0.96 (0.79, 1.17)
1.08 (1.00, 1.18)	3.16E-01	1.10 (0.98, 1.24)	3.17E-01	0.88 (0.74, 1.06)
1.17 (1.07, 1.28)	**8.80E-03**	1.14 (1.01, 1.29)	1.45E-01	1.04 (0.87, 1.24)
1.15 (1.06, 1.25)	**8.80E-03**	1.15 (1.02, 1.30)	1.11E-01	1.01 (0.85, 1.20)
0.97 (0.88, 1.06)	7.96E-01	0.99 (0.88, 1.11)	9.27E-01	1.10 (0.91, 1.32)
0.86 (0.78, 0.94)	**8.80E-03**	0.87 (0.77, 0.98)	1.13E-01	0.98 (0.82, 1.17)
1.01 (0.93, 1.10)	9.13E-01	1.11 (0.99, 1.25)	2.24E-01	0.95 (0.80, 1.12)
1.07 (0.98, 1.16)	3.91E-01	0.97 (0.86, 1.10)	8.22E-01	0.95 (0.81, 1.11)
1.07 (0.98, 1.16)	4.24E-01	0.96 (0.85, 1.09)	7.61E-01	0.95 (0.80, 1.11)
1.06 (0.97, 1.16)	4.56E-01	1.06 (0.93, 1.20)	6.54E-01	0.98 (0.83, 1.16)
0.93 (0.86, 1.01)	3.75E-01	1.00 (0.88, 1.13)	9.98E-01	1.07 (0.91, 1.25)
1.09 (0.99, 1.20)	3.65E-01	1.02 (0.89, 1.17)	8.59E-01	0.90 (0.77, 1.05)
1.02 (0.94, 1.11)	8.98E-01	0.83 (0.74, 0.93)	**2.55E-02**	1.06 (0.90, 1.25)
1.01 (0.93, 1.09)	9.52E-01	0.84 (0.74, 0.94)	**4.14E-02**	1.09 (0.92, 1.29)
1.05 (0.96, 1.15)	6.30E-01	0.87 (0.77, 0.98)	9.38E-02	0.97 (0.83, 1.13)
0.95 (0.87, 1.04)	5.67E-01	1.15 (1.03, 1.30)	8.87E-02	1.01 (0.86, 1.18)
0.99 (0.90, 1.08)	9.13E-01	0.97 (0.85, 1.11)	8.22E-01	0.90 (0.76, 1.07)
1.04 (0.95, 1.13)	7.68E-01	0.87 (0.78, 0.96)	5.58E-02	0.93 (0.81, 1.07)
0.98 (0.90, 1.07)	8.98E-01	0.87 (0.78, 0.97)	7.92E-02	0.91 (0.78, 1.06)
1.09 (0.99, 1.20)	3.25E-01	0.92 (0.81, 1.05)	4.46E-01	0.97 (0.83, 1.14)
0.97 (0.89, 1.05)	7.96E-01	1.18 (1.05, 1.31)	**4.14E-02**	1.11 (0.96, 1.28)
0.96 (0.87, 1.05)	7.41E-01	1.10 (0.97, 1.26)	3.47E-01	1.07 (0.92, 1.25)
1.03 (0.94, 1.12)	8.81E-01	0.86 (0.77, 0.96)	6.41E-02	0.89 (0.78, 1.02)
0.99 (0.91, 1.09)	9.52E-01	0.87 (0.78, 0.97)	8.08E-02	0.87 (0.76, 0.99)

1.10 (1.00, 1.21)	2.36E-01	0.96 (0.83, 1.10)	7.61E-01	1.06 (0.89, 1.26)
0.99 (0.90, 1.08)	9.42E-01	1.18 (1.06, 1.32)	**4.14E-02**	1.14 (1.00, 1.31)
0.98 (0.89, 1.08)	8.98E-01	1.10 (0.98, 1.25)	3.27E-01	1.13 (0.98, 1.32)
1.01 (0.92, 1.10)	9.52E-01	0.88 (0.78, 0.99)	1.49E-01	0.86 (0.75, 0.99)
1.00 (0.91, 1.10)	9.90E-01	0.89 (0.79, 1.00)	2.16E-01	0.85 (0.74, 0.98)
1.02 (0.92, 1.12)	9.13E-01	1.07 (0.94, 1.22)	6.12E-01	1.20 (1.02, 1.40)
1.02 (0.93, 1.12)	8.98E-01	1.18 (1.05, 1.32)	**4.95E-02**	1.19 (1.04, 1.37)
1.00 (0.91, 1.09)	9.54E-01	1.13 (1.00, 1.28)	1.76E-01	1.15 (0.99, 1.34)
1.01 (0.92, 1.10)	9.52E-01	0.87 (0.77, 0.98)	1.14E-01	0.87 (0.75, 1.00)
1.00 (0.92, 1.10)	9.54E-01	0.88 (0.78, 0.99)	1.73E-01	0.86 (0.74, 1.00)
1.01 (0.92, 1.12)	9.13E-01	1.05 (0.92, 1.20)	7.01E-01	1.18 (1.00, 1.39)
0.99 (0.90, 1.08)	9.13E-01	1.15 (1.02, 1.30)	1.03E-01	1.15 (0.99, 1.33)
1.17 (1.08, 1.28)	**5.31E-03**	1.21 (1.05, 1.39)	5.92E-02	1.02 (0.87, 1.21)
0.87 (0.80, 0.94)	**8.08E-03**	0.81 (0.71, 0.91)	**2.15E-02**	0.95 (0.81, 1.12)
0.83 (0.77, 0.91)	**7.20E-04**	0.78 (0.69, 0.89)	**8.59E-03**	0.95 (0.80, 1.12)
1.03 (0.96, 1.12)	7.91E-01	0.96 (0.85, 1.08)	7.30E-01	0.98 (0.85, 1.14)
0.93 (0.85, 1.02)	3.75E-01	1.04 (0.92, 1.18)	7.30E-01	1.05 (0.91, 1.22)
0.92 (0.84, 1.00)	2.92E-01	0.83 (0.72, 0.96)	7.92E-02	0.70 (0.58, 0.84)
1.07 (0.99, 1.17)	3.75E-01	1.11 (0.97, 1.28)	3.42E-01	1.25 (1.04, 1.51)
1.06 (0.98, 1.16)	4.36E-01	1.18 (1.02, 1.35)	1.11E-01	1.35 (1.12, 1.64)
1.07 (0.99, 1.17)	3.75E-01	1.01 (0.89, 1.15)	9.42E-01	1.10 (0.92, 1.30)
0.99 (0.91, 1.08)	9.47E-01	1.08 (0.95, 1.22)	4.91E-01	1.11 (0.96, 1.30)
0.92 (0.84, 1.02)	4.24E-01	1.22 (1.08, 1.38)	**2.59E-02**	1.19 (1.07, 1.33)
1.05 (0.95, 1.15)	6.84E-01	0.84 (0.73, 0.96)	6.11E-02	0.89 (0.77, 1.02)
1.04 (0.95, 1.14)	7.96E-01	0.82 (0.72, 0.94)	**4.14E-02**	0.89 (0.78, 1.02)
1.06 (0.97, 1.16)	4.72E-01	0.96 (0.84, 1.10)	7.61E-01	0.92 (0.79, 1.08)
0.99 (0.91, 1.07)	9.13E-01	1.09 (0.96, 1.25)	4.19E-01	1.00 (0.85, 1.18)
0.95 (0.87, 1.04)	6.36E-01	1.03 (0.90, 1.18)	8.22E-01	1.13 (0.96, 1.33)
1.05 (0.96, 1.15)	6.12E-01	0.95 (0.83, 1.09)	7.07E-01	0.88 (0.75, 1.04)
1.05 (0.95, 1.15)	6.84E-01	0.95 (0.83, 1.09)	7.09E-01	0.88 (0.75, 1.03)
1.01 (0.93, 1.11)	9.13E-01	1.03 (0.90, 1.18)	8.22E-01	1.12 (0.95, 1.33)
0.97 (0.88, 1.05)	7.96E-01	1.08 (0.95, 1.24)	4.95E-01	1.07 (0.91, 1.26)
0.88 (0.80, 0.96)	**4.55E-02**	0.91 (0.80, 1.04)	4.22E-01	1.03 (0.87, 1.22)
1.19 (1.09, 1.30)	**4.21E-03**	1.19 (1.05, 1.36)	6.11E-02	1.24 (1.06, 1.46)
1.11 (1.02, 1.21)	7.63E-02	1.11 (0.98, 1.26)	3.20E-01	1.19 (1.01, 1.40)
1.03 (0.95, 1.12)	8.06E-01	0.94 (0.82, 1.07)	6.12E-01	0.89 (0.75, 1.05)
0.98 (0.90, 1.06)	8.86E-01	0.92 (0.80, 1.06)	5.04E-01	0.96 (0.81, 1.13)
1.00 (0.92, 1.08)	9.62E-01	0.91 (0.80, 1.04)	4.21E-01	0.93 (0.78, 1.10)
1.00 (0.92, 1.09)	9.81E-01	0.89 (0.78, 1.02)	2.86E-01	0.85 (0.72, 1.01)
1.09 (1.01, 1.19)	1.66E-01	1.10 (0.97, 1.24)	3.66E-01	1.00 (0.85, 1.18)
1.10 (1.01, 1.19)	1.58E-01	1.11 (0.98, 1.26)	3.19E-01	1.00 (0.85, 1.18)
1.06 (0.98, 1.15)	4.36E-01	0.98 (0.87, 1.11)	8.83E-01	1.03 (0.88, 1.21)
1.02 (0.93, 1.11)	9.04E-01	0.95 (0.83, 1.08)	7.01E-01	0.85 (0.71, 1.02)
1.01 (0.93, 1.11)	9.13E-01	0.94 (0.82, 1.08)	6.54E-01	0.85 (0.71, 1.02)
0.94 (0.86, 1.03)	4.56E-01	0.99 (0.86, 1.14)	9.54E-01	1.02 (0.86, 1.20)
0.93 (0.85, 1.02)	3.91E-01	0.97 (0.84, 1.11)	8.22E-01	1.01 (0.86, 1.19)
1.00 (0.92, 1.09)	9.54E-01	1.10 (0.96, 1.25)	3.75E-01	1.03 (0.88, 1.21)
1.01 (0.93, 1.11)	9.13E-01	1.10 (0.98, 1.25)	3.27E-01	1.09 (0.92, 1.28)

1.06 (0.97, 1.15)	4.56E-01	1.04 (0.91, 1.18)	7.84E-01	0.93 (0.79, 1.10)
0.94 (0.86, 1.02)	4.36E-01	0.97 (0.85, 1.12)	8.41E-01	0.99 (0.84, 1.17)
1.02 (0.94, 1.11)	8.98E-01	1.03 (0.90, 1.17)	8.37E-01	0.87 (0.73, 1.03)
0.96 (0.88, 1.05)	7.53E-01	0.94 (0.82, 1.08)	6.65E-01	0.86 (0.72, 1.03)
1.05 (0.96, 1.14)	6.26E-01	1.05 (0.92, 1.21)	7.01E-01	0.94 (0.79, 1.11)
1.08 (0.99, 1.17)	3.27E-01	1.06 (0.93, 1.20)	6.65E-01	0.98 (0.83, 1.15)
0.98 (0.90, 1.07)	8.98E-01	0.91 (0.79, 1.04)	4.10E-01	0.90 (0.76, 1.07)
0.94 (0.86, 1.03)	4.56E-01	0.90 (0.78, 1.04)	3.64E-01	0.92 (0.78, 1.09)
1.00 (0.92, 1.09)	9.54E-01	1.00 (0.87, 1.15)	9.98E-01	0.90 (0.76, 1.07)
1.01 (0.92, 1.11)	9.52E-01	0.94 (0.81, 1.08)	6.38E-01	0.71 (0.6, 0.83)
0.93 (0.85, 1.01)	3.16E-01	0.84 (0.73, 0.96)	6.65E-02	0.88 (0.75, 1.04)
1.01 (0.93, 1.11)	9.13E-01	0.94 (0.81, 1.09)	6.81E-01	0.84 (0.70, 1.01)
0.93 (0.86, 1.02)	3.91E-01	0.84 (0.72, 0.97)	1.03E-01	0.81 (0.68, 0.97)
1.02 (0.94, 1.12)	8.93E-01	0.97 (0.84, 1.12)	8.22E-01	0.83 (0.69, 1.00)
1.01 (0.92, 1.10)	9.52E-01	0.94 (0.82, 1.09)	6.81E-01	0.82 (0.68, 0.99)
0.97 (0.89, 1.06)	8.78E-01	1.00 (0.87, 1.15)	9.98E-01	0.93 (0.78, 1.10)
1.03 (0.95, 1.12)	8.06E-01	1.05 (0.91, 1.20)	7.30E-01	0.96 (0.81, 1.14)
0.87 (0.80, 0.95)	**2.47E-02**	0.80 (0.70, 0.90)	**1.27E-02**	0.96 (0.81, 1.13)
1.02 (0.93, 1.12)	9.03E-01	0.94 (0.81, 1.10)	6.81E-01	0.82 (0.70, 0.97)
0.85 (0.78, 0.93)	**6.79E-03**	0.74 (0.65, 0.84)	**9.15E-04**	0.83 (0.70, 0.98)
1.07 (0.97, 1.17)	4.56E-01	0.99 (0.87, 1.14)	9.72E-01	0.84 (0.70, 0.99)
1.01 (0.92, 1.11)	9.46E-01	0.90 (0.79, 1.03)	3.52E-01	0.78 (0.65, 0.93)
0.89 (0.82, 0.98)	8.99E-02	0.96 (0.84, 1.10)	7.67E-01	1.05 (0.89, 1.25)
1.15 (1.05, 1.26)	**2.21E-02**	1.22 (1.08, 1.39)	**2.83E-02**	1.36 (1.16, 1.60)
1.03 (0.95, 1.11)	8.58E-01	0.88 (0.74, 1.03)	3.27E-01	1.21 (1.09, 1.35)
0.77 (0.70, 0.86)	**8.32E-05**	0.79 (0.68, 0.91)	**2.55E-02**	0.91 (0.76, 1.10)
1.28 (1.17, 1.41)	**2.11E-05**	1.24 (1.09, 1.40)	**2.15E-02**	1.04 (0.87, 1.25)
0.79 (0.72, 0.87)	**3.17E-05**	0.83 (0.73, 0.95)	6.06E-02	0.93 (0.78, 1.10)
0.79 (0.72, 0.86)	**2.36E-05**	0.81 (0.71, 0.92)	**2.59E-02**	1.00 (0.85, 1.19)
0.73 (0.66, 0.80)	**6.71E-08**	0.78 (0.68, 0.89)	**8.59E-03**	1.02 (0.86, 1.22)
0.90 (0.82, 0.99)	1.66E-01	0.87 (0.75, 1.01)	2.22E-01	0.96 (0.81, 1.14)
0.84 (0.76, 0.92)	**5.31E-03**	0.85 (0.74, 0.99)	1.41E-01	0.93 (0.78, 1.11)
0.83 (0.75, 0.92)	**5.31E-03**	0.82 (0.71, 0.94)	**4.95E-02**	0.91 (0.76, 1.09)
0.85 (0.77, 0.94)	**1.30E-02**	0.86 (0.74, 1.00)	2.14E-01	1.13 (0.99, 1.28)
0.87 (0.79, 0.95)	**3.07E-02**	0.87 (0.76, 1.00)	1.85E-01	1.11 (0.94, 1.31)
0.97 (0.89, 1.06)	8.06E-01	0.97 (0.84, 1.12)	8.22E-01	1.05 (0.94, 1.16)
1.09 (1.00, 1.18)	2.36E-01	1.21 (1.12, 1.31)	**2.34E-04**	1.02 (0.86, 1.20)
1.04 (0.96, 1.12)	7.59E-01	1.17 (1.08, 1.26)	**7.74E-03**	1.01 (0.85, 1.20)
0.80 (0.66, 0.98)	1.76E-01	0.68 (0.55, 0.85)	**2.15E-02**	1.09 (1.04, 1.14)
1.08 (0.99, 1.18)	3.75E-01	1.11 (0.97, 1.26)	3.40E-01	0.81 (0.70, 0.95)
1.02 (0.93, 1.12)	8.98E-01	0.97 (0.84, 1.13)	8.41E-01	1.08 (0.92, 1.28)

			UKB	
Other mortality				All causes
oker	Current smoker		Never/former smoker	
P value	Adjusted HR (95% CI)	P value	Adjusted HR (95% CI)	P value
5.71E-01	0.83 (0.62, 1.10)	9.52E-01	1.00 (0.98, 1.01)	5.86E-01
5.71E-01	0.84 (0.63, 1.11)	9.52E-01	0.99 (0.98, 1.01)	4.63E-01
5.77E-01	0.86 (0.65, 1.13)	9.52E-01	1.00 (0.98, 1.01)	7.07E-01
5.54E-01	0.92 (0.71, 1.19)	9.52E-01	0.99 (0.97, 1.00)	1.24E-01
5.99E-01	0.95 (0.74, 1.22)	9.61E-01	0.98 (0.97, 1.00)	**4.29E-02**
4.94E-01	0.89 (0.68, 1.16)	9.52E-01	0.99 (0.98, 1.01)	3.52E-01
5.77E-01	0.81 (0.61, 1.09)	8.83E-01	1.00 (0.98, 1.01)	5.72E-01
6.02E-01	0.85 (0.65, 1.13)	9.52E-01	0.97 (0.95, 0.98)	**1.85E-05**
6.02E-01	0.86 (0.65, 1.13)	9.52E-01	0.96 (0.95, 0.98)	**5.13E-06**
5.99E-01	0.89 (0.67, 1.16)	9.52E-01	0.96 (0.95, 0.98)	**1.41E-05**
6.42E-01	0.89 (0.68, 1.16)	9.52E-01	0.94 (0.93, 0.96)	**7.29E-12**
6.97E-01	0.88 (0.68, 1.15)	9.52E-01	0.93 (0.92, 0.95)	**1.32E-17**
6.02E-01	0.90 (0.69, 1.18)	9.52E-01	0.96 (0.95, 0.98)	**9.93E-07**
6.02E-01	0.84 (0.64, 1.12)	9.52E-01	0.97 (0.95, 0.99)	**1.92E-04**
7.92E-01	0.89 (0.68, 1.17)	9.52E-01	0.95 (0.94, 0.97)	**1.34E-09**
7.95E-01	0.89 (0.69, 1.17)	9.52E-01	0.94 (0.93, 0.96)	**4.82E-13**
7.92E-01	0.91 (0.70, 1.18)	9.52E-01	0.95 (0.94, 0.97)	**1.19E-08**
9.07E-01	0.92 (0.71, 1.19)	9.52E-01	0.94 (0.92, 0.95)	**1.15E-14**
9.77E-01	0.92 (0.71, 1.19)	9.52E-01	0.93 (0.92, 0.95)	**3.09E-17**
7.20E-01	0.92 (0.71, 1.19)	9.52E-01	0.94 (0.93, 0.96)	**8.30E-12**
7.55E-01	0.88 (0.67, 1.16)	9.52E-01	0.94 (0.93, 0.96)	**1.65E-13**
9.42E-01	0.89 (0.69, 1.16)	9.52E-01	0.91 (0.90, 0.93)	**9.77E-30**
9.37E-01	0.90 (0.69, 1.17)	9.52E-01	0.91 (0.90, 0.93)	**4.18E-29**
9.11E-01	0.91 (0.70, 1.17)	9.52E-01	0.91 (0.90, 0.93)	**7.08E-29**
8.27E-01	0.94 (0.73, 1.21)	9.58E-01	0.91 (0.89, 0.92)	**4.72E-33**
6.76E-01	0.96 (0.75, 1.23)	9.61E-01	0.91 (0.90, 0.92)	**7.29E-32**
9.94E-01	0.92 (0.71, 1.19)	9.52E-01	0.91 (0.90, 0.93)	**2.61E-30**
9.85E-01	0.88 (0.68, 1.15)	9.52E-01	0.93 (0.92, 0.95)	**1.96E-17**
5.99E-01	0.96 (0.75, 1.23)	9.62E-01	0.96 (0.94, 0.97)	**2.35E-08**
5.71E-01	0.97 (0.75, 1.24)	9.71E-01	0.95 (0.93, 0.96)	**1.24E-10**
4.94E-01	0.98 (0.77, 1.25)	9.90E-01	0.93 (0.91, 0.94)	**1.49E-19**
4.10E-01	1.00 (0.79, 1.27)	9.99E-01	0.93 (0.92, 0.95)	**1.09E-16**
3.74E-01	1.00 (0.79, 1.27)	9.95E-01	0.95 (0.93, 0.96)	**6.91E-12**
5.71E-01	1.00 (0.79, 1.28)	9.95E-01	0.92 (0.90, 0.93)	**2.48E-26**
7.90E-01	0.95 (0.74, 1.22)	9.60E-01	0.98 (0.97, 1.00)	**1.92E-02**
5.71E-01	1.11 (0.88, 1.39)	9.52E-01	0.99 (0.98, 1.01)	5.50E-01
5.71E-01	1.11 (0.88, 1.39)	9.52E-01	1.00 (0.98, 1.02)	9.48E-01
4.11E-01	1.13 (0.90, 1.42)	9.52E-01	1.02 (1.00, 1.03)	**3.68E-02**
7.27E-01	1.07 (0.85, 1.34)	9.52E-01	0.97 (0.96, 0.99)	**1.18E-03**
8.32E-01	1.04 (0.83, 1.31)	9.61E-01	0.97 (0.95, 0.98)	**1.59E-05**

5.71E-01	1.11 (0.89, 1.40)	9.52E-01	1.00 (0.98, 1.01)	5.72E-01
6.70E-01	1.10 (0.87, 1.39)	9.52E-01	1.04 (1.02, 1.06)	**6.03E-07**
5.54E-01	1.10 (0.88, 1.39)	9.52E-01	0.93 (0.91, 0.94)	**2.43E-20**
5.26E-01	1.09 (0.87, 1.38)	9.52E-01	0.93 (0.91, 0.94)	**5.43E-21**
3.58E-01	1.09 (0.86, 1.37)	9.52E-01	0.94 (0.92, 0.95)	**4.76E-15**
4.46E-01	1.05 (0.83, 1.33)	9.60E-01	0.91 (0.90, 0.93)	**1.56E-28**
4.20E-01	1.03 (0.82, 1.30)	9.71E-01	0.91 (0.89, 0.92)	**1.90E-31**
5.20E-01	1.10 (0.87, 1.39)	9.52E-01	0.93 (0.91, 0.94)	**5.51E-20**
9.37E-01	1.29 (1.06, 1.57)	1.92E-01	1.05 (1.04, 1.07)	**7.57E-11**
2.72E-01	1.07 (0.85, 1.36)	9.52E-01	0.91 (0.90, 0.92)	**2.76E-31**
2.70E-01	1.06 (0.84, 1.35)	9.52E-01	0.89 (0.88, 0.91)	**2.77E-43**
2.18E-01	1.04 (0.82, 1.32)	9.61E-01	0.89 (0.88, 0.91)	**1.45E-45**
2.56E-01	1.04 (0.82, 1.32)	9.61E-01	0.89 (0.87, 0.90)	**3.62E-49**
2.51E-01	1.03 (0.81, 1.31)	9.73E-01	0.89 (0.87, 0.90)	**1.15E-49**
3.25E-01	1.07 (0.84, 1.35)	9.52E-01	0.89 (0.88, 0.91)	**2.93E-45**
8.34E-01	1.30 (1.07, 1.58)	1.79E-01	1.04 (1.03, 1.06)	**1.74E-07**
1.86E-01	1.01 (0.80, 1.29)	9.92E-01	0.90 (0.89, 0.92)	**3.84E-35**
1.81E-01	1.01 (0.79, 1.28)	9.95E-01	0.89 (0.87, 0.90)	**1.09E-49**
1.66E-01	0.94 (0.73, 1.20)	9.52E-01	0.88 (0.87, 0.90)	**3.88E-52**
1.81E-01	1.00 (0.79, 1.28)	9.95E-01	0.88 (0.87, 0.89)	**4.93E-55**
1.81E-01	1.02 (0.80, 1.29)	9.92E-01	0.89 (0.87, 0.90)	**1.14E-50**
1.74E-01	0.95 (0.75, 1.21)	9.61E-01	0.88 (0.86, 0.89)	**6.37E-60**
9.85E-01	1.26 (1.01, 1.56)	4.11E-01	1.02 (1.00, 1.03)	**1.66E-02**
1.66E-01	0.98 (0.77, 1.25)	9.90E-01	0.92 (0.90, 0.93)	**4.16E-27**
1.66E-01	0.98 (0.77, 1.24)	9.85E-01	0.90 (0.88, 0.91)	**2.11E-40**
1.19E-01	0.90 (0.71, 1.14)	9.52E-01	0.91 (0.90, 0.92)	**5.53E-32**
1.72E-01	0.99 (0.78, 1.26)	9.95E-01	0.89 (0.87, 0.90)	**3.91E-49**
1.74E-01	1.01 (0.79, 1.28)	9.95E-01	0.89 (0.88, 0.91)	**5.32E-44**
1.45E-01	0.93 (0.74, 1.19)	9.52E-01	0.89 (0.87, 0.90)	**9.19E-53**
8.05E-01	1.09 (0.85, 1.38)	9.52E-01	1.00 (0.99, 1.02)	7.18E-01
2.12E-01	1.13 (0.91, 1.40)	9.52E-01	1.07 (1.05, 1.09)	**1.48E-13**
2.18E-01	1.12 (0.90, 1.39)	9.52E-01	1.08 (1.06, 1.10)	**1.04E-17**
2.24E-01	1.18 (0.95, 1.47)	7.45E-01	1.09 (1.07, 1.11)	**4.00E-22**
2.56E-01	1.03 (0.83, 1.29)	9.62E-01	1.06 (1.04, 1.08)	**3.13E-11**
2.70E-01	1.02 (0.82, 1.28)	9.79E-01	1.05 (1.03, 1.07)	**1.30E-08**
2.42E-01	1.06 (0.85, 1.32)	9.52E-01	1.09 (1.07, 1.10)	**1.11E-22**
1.16E-01	1.13 (0.91, 1.41)	9.52E-01	1.05 (1.03, 1.07)	**1.69E-10**
2.51E-01	1.23 (0.98, 1.53)	5.89E-01	1.04 (1.02, 1.06)	**2.68E-05**
2.51E-01	1.22 (0.98, 1.53)	5.97E-01	1.05 (1.03, 1.06)	**1.55E-06**
3.78E-01	1.22 (0.97, 1.52)	6.66E-01	1.05 (1.03, 1.07)	**2.73E-07**
2.24E-01	1.21 (0.97, 1.51)	6.66E-01	1.04 (1.02, 1.06)	**9.07E-05**
2.18E-01	1.21 (0.97, 1.51)	6.66E-01	1.03 (1.01, 1.05)	**5.28E-04**
2.59E-01	1.20 (0.96, 1.50)	6.91E-01	1.05 (1.03, 1.07)	**5.61E-08**
1.16E-01	1.34 (1.10, 1.63)	1.47E-01	1.07 (1.05, 1.08)	**9.11E-16**
2.42E-01	0.98 (0.76, 1.27)	9.92E-01	0.96 (0.94, 0.98)	**9.04E-06**
2.42E-01	0.98 (0.76, 1.26)	9.89E-01	0.97 (0.95, 0.98)	**7.99E-05**
4.02E-01	1.03 (0.80, 1.33)	9.71E-01	0.97 (0.96, 0.99)	**1.15E-03**
2.12E-01	0.93 (0.72, 1.2)	9.52E-01	0.96 (0.94, 0.97)	**4.44E-07**

1.86E-01	0.91 (0.70, 1.17)	9.52E-01	0.95 (0.94, 0.97)	**7.38E-08**
3.12E-01	1.02 (0.79, 1.31)	9.92E-01	0.97 (0.95, 0.99)	**3.55E-04**
4.94E-01	1.03 (0.82, 1.29)	9.73E-01	1.02 (1.00, 1.04)	**1.30E-02**
**3.24E-03**	0.93 (0.73, 1.19)	9.52E-01	0.87 (0.85, 0.88)	**9.06E-69**
**2.99E-03**	0.93 (0.73, 1.18)	9.52E-01	0.90 (0.88, 0.91)	**1.82E-39**
1.86E-01	0.89 (0.69, 1.14)	9.52E-01	0.91 (0.90, 0.93)	**5.66E-28**
**5.87E-03**	1.01 (0.80, 1.29)	9.92E-01	0.87 (0.85, 0.88)	**9.77E-68**
**1.70E-02**	1.04 (0.81, 1.32)	9.62E-01	0.86 (0.84, 0.87)	**4.06E-78**
1.19E-01	0.90 (0.70, 1.15)	9.52E-01	0.91 (0.90, 0.93)	**1.59E-28**
7.27E-01	1.06 (0.84, 1.33)	9.58E-01	1.00 (0.99, 1.02)	8.68E-01
9.85E-01	1.17 (0.88, 1.55)	9.52E-01	1.04 (1.02, 1.05)	**1.45E-04**
4.11E-01	1.17 (0.96, 1.43)	7.45E-01	1.01 (0.99, 1.03)	3.30E-01
5.99E-01	1.14 (0.93, 1.40)	9.52E-01	1.00 (0.99, 1.02)	8.07E-01
3.32E-01	1.14 (0.90, 1.45)	9.52E-01	1.02 (1.00, 1.04)	**2.48E-02**
4.94E-01	0.81 (0.65, 1.02)	5.89E-01	0.98 (0.97, 1.00)	**4.42E-02**
9.42E-01	1.11 (0.85, 1.44)	9.52E-01	1.01 (0.99, 1.02)	5.81E-01
4.51E-01	1.04 (0.85, 1.27)	9.61E-01	0.99 (0.98, 1.01)	5.78E-01
6.94E-01	0.97 (0.76, 1.24)	9.73E-01	1.04 (1.03, 1.06)	**8.98E-07**
4.96E-01	1.09 (0.91, 1.32)	9.52E-01	0.99 (0.97, 1.01)	2.44E-01
5.65E-01	0.91 (0.72, 1.16)	9.52E-01	1.02 (1.00, 1.04)	**3.31E-02**
9.11E-01	1.22 (0.92, 1.62)	8.88E-01	1.04 (1.02, 1.05)	**3.17E-05**
8.05E-01	0.99 (0.78, 1.25)	9.92E-01	1.03 (1.01, 1.05)	**2.85E-04**
9.85E-01	0.92 (0.72, 1.18)	9.52E-01	1.04 (1.02, 1.06)	**2.26E-05**
7.52E-01	1.06 (0.83, 1.35)	9.60E-01	0.96 (0.95, 0.98)	**2.09E-05**
8.23E-01	0.97 (0.76, 1.24)	9.71E-01	0.98 (0.96, 0.99)	**5.06E-03**
4.10E-01	1.08 (0.87, 1.35)	9.52E-01	0.97 (0.96, 0.99)	**7.17E-04**
8.07E-01	1.07 (0.85, 1.35)	9.52E-01	0.97 (0.96, 0.99)	**4.05E-04**
9.73E-01	1.05 (0.83, 1.33)	9.60E-01	0.98 (0.96, 0.99)	**4.77E-03**
5.71E-01	1.06 (0.84, 1.34)	9.58E-01	1.03 (1.01, 1.04)	**9.41E-04**
9.15E-01	0.92 (0.73, 1.17)	9.52E-01	0.92 (0.91, 0.94)	**1.80E-23**
7.20E-01	1.09 (0.88, 1.36)	9.52E-01	0.95 (0.94, 0.97)	**2.51E-09**
6.98E-01	1.04 (0.83, 1.31)	9.61E-01	0.98 (0.97, 1.00)	**2.22E-02**
6.94E-01	1.01 (0.81, 1.27)	9.92E-01	0.92 (0.91, 0.94)	**8.30E-24**
9.11E-01	1.23 (0.98, 1.53)	5.97E-01	1.06 (1.04, 1.08)	**3.34E-13**
6.27E-01	0.94 (0.75, 1.18)	9.52E-01	1.15 (1.14, 1.17)	**5.10E-70**
4.17E-01	1.06 (0.83, 1.35)	9.58E-01	0.93 (0.91, 0.94)	**1.56E-23**
6.94E-01	0.97 (0.78, 1.22)	9.73E-01	0.92 (0.91, 0.93)	**7.73E-26**
5.71E-01	0.95 (0.76, 1.18)	9.58E-01	0.97 (0.95, 0.98)	**1.64E-05**
8.21E-01	1.06 (0.83, 1.35)	9.60E-01	1.06 (1.04, 1.07)	**2.04E-13**
9.77E-01	0.99 (0.79, 1.25)	9.95E-01	1.12 (1.10, 1.13)	**8.50E-45**
4.94E-01	1.00 (0.79, 1.26)	9.95E-01	0.88 (0.87, 0.90)	**3.48E-69**
5.54E-01	0.87 (0.72, 1.04)	7.45E-01	0.86 (0.85, 0.88)	**5.46E-91**
4.94E-01	0.81 (0.68, 0.96)	1.92E-01	0.98 (0.96, 0.99)	**9.99E-04**
8.60E-01	1.05 (0.82, 1.35)	9.61E-01	1.12 (1.10, 1.13)	**3.70E-57**
3.82E-01	1.16 (0.96, 1.41)	7.45E-01	1.01 (1.00, 1.03)	9.69E-02
5.99E-01	1.05 (0.83, 1.32)	9.61E-01	0.92 (0.92, 0.93)	**2.57E-96**
2.70E-01	0.86 (0.71, 1.04)	7.45E-01	0.93 (0.92, 0.93)	**7.41E-100**
2.12E-01	0.84 (0.70, 1.01)	5.89E-01	0.94 (0.93, 0.95)	**6.10E-17**

6.98E-01	1.08 (0.83, 1.40)	9.52E-01	1.10 (1.09, 1.11)	**4.06E-101**
2.21E-01	1.22 (1.03, 1.45)	2.91E-01	1.01 (1.00, 1.03)	1.45E-01
2.72E-01	0.95 (0.74, 1.22)	9.61E-01	0.92 (0.91, 0.93)	**1.67E-104**
2.12E-01	0.96 (0.77, 1.20)	9.61E-01	0.92 (0.90, 0.93)	**1.05E-36**
1.81E-01	0.98 (0.78, 1.24)	9.92E-01	0.95 (0.94, 0.96)	**5.68E-11**
1.76E-01	0.94 (0.73, 1.22)	9.60E-01	1.10 (1.09, 1.11)	**2.17E-124**
1.63E-01	1.20 (0.99, 1.47)	5.89E-01	1.09 (1.08, 1.11)	**2.95E-33**
2.51E-01	0.96 (0.75, 1.22)	9.61E-01	0.90 (0.89, 0.90)	**2.32E-115**
2.31E-01	0.99 (0.79, 1.26)	9.95E-01	0.92 (0.90, 0.93)	**7.67E-33**
2.18E-01	1.01 (0.79, 1.28)	9.95E-01	0.94 (0.92, 0.95)	**7.72E-20**
2.21E-01	0.93 (0.72, 1.20)	9.52E-01	1.08 (1.07, 1.10)	**4.55E-34**
2.51E-01	1.10 (0.88, 1.37)	9.52E-01	1.08 (1.06, 1.10)	**2.74E-19**
8.98E-01	1.27 (1.00, 1.60)	5.24E-01	0.91 (0.89, 0.92)	**3.91E-33**
7.27E-01	0.75 (0.61, 0.92)	1.79E-01	0.92 (0.91, 0.93)	**4.10E-37**
7.13E-01	0.75 (0.60, 0.94)	1.92E-01	0.97 (0.96, 0.99)	**5.63E-04**
9.11E-01	0.81 (0.68, 0.96)	1.92E-01	1.00 (0.98, 1.01)	8.07E-01
6.97E-01	1.14 (0.93, 1.39)	9.52E-01	1.02 (1.00, 1.03)	**1.48E-02**
**7.12E-03**	0.88 (0.69, 1.13)	9.52E-01	0.98 (0.96, 0.99)	**1.18E-03**
1.66E-01	0.96 (0.76, 1.20)	9.61E-01	0.97 (0.95, 0.98)	**5.42E-06**
**4.18E-02**	0.94 (0.74, 1.18)	9.52E-01	1.02 (1.01, 1.04)	**2.41E-03**
5.54E-01	1.00 (0.80, 1.25)	9.99E-01	1.02 (1.01, 1.04)	**1.25E-03**
3.99E-01	1.24 (1.04, 1.48)	2.29E-01	1.07 (1.05, 1.09)	**5.80E-18**
**3.12E-02**	1.29 (1.09, 1.52)	1.47E-01	0.94 (0.93, 0.96)	**2.31E-14**
2.70E-01	0.80 (0.65, 0.98)	3.12E-01	0.94 (0.93, 0.96)	**7.92E-14**
2.70E-01	0.77 (0.64, 0.93)	1.79E-01	0.96 (0.94, 0.98)	**6.48E-06**
5.71E-01	1.02 (0.80, 1.30)	9.88E-01	1.05 (1.04, 1.07)	**1.77E-10**
9.94E-01	1.12 (0.89, 1.4)	9.52E-01	1.11 (1.10, 1.13)	**8.01E-39**
3.51E-01	0.88 (0.68, 1.14)	9.52E-01	0.90 (0.89, 0.91)	**1.47E-42**
3.32E-01	1.08 (0.84, 1.39)	9.52E-01	0.89 (0.88, 0.90)	**1.57E-53**
3.32E-01	1.09 (0.85, 1.40)	9.52E-01	1.05 (1.03, 1.07)	**2.36E-09**
4.20E-01	0.87 (0.68, 1.12)	9.52E-01	1.07 (1.05, 1.08)	**1.22E-15**
6.02E-01	1.09 (0.87, 1.38)	9.52E-01	0.93 (0.91, 0.94)	**6.26E-20**
8.27E-01	0.86 (0.68, 1.10)	9.52E-01	0.97 (0.96, 0.99)	**8.61E-04**
1.16E-01	1.34 (1.08, 1.67)	1.79E-01	1.09 (1.07, 1.11)	**5.03E-22**
1.86E-01	1.13 (0.90, 1.41)	9.52E-01	0.90 (0.89, 0.91)	**1.33E-37**
4.10E-01	1.05 (0.83, 1.33)	9.60E-01	0.94 (0.92, 0.95)	**5.54E-16**
7.90E-01	0.97 (0.76, 1.23)	9.67E-01	0.88 (0.87, 0.90)	**6.43E-52**
5.99E-01	1.00 (0.79, 1.27)	9.95E-01	0.92 (0.91, 0.93)	**3.36E-25**
2.51E-01	1.04 (0.82, 1.31)	9.62E-01	0.90 (0.88, 0.91)	**3.55E-39**
9.93E-01	1.09 (0.86, 1.37)	9.52E-01	0.91 (0.89, 0.92)	**1.40E-32**
9.94E-01	1.08 (0.85, 1.36)	9.52E-01		
8.27E-01	1.13 (0.90, 1.40)	9.52E-01		
2.58E-01	1.09 (0.86, 1.37)	9.52E-01	0.98 (0.96, 0.99)	**5.61E-03**
2.65E-01	1.09 (0.86, 1.39)	9.52E-01	0.97 (0.95, 0.98)	**1.44E-05**
9.15E-01	0.96 (0.75, 1.23)	9.61E-01	1.03 (1.02, 1.05)	**9.75E-05**
9.67E-01	0.90 (0.69, 1.17)	9.52E-01	1.03 (1.01, 1.04)	**7.17E-04**
8.32E-01	1.28 (1.05, 1.57)	1.92E-01	0.94 (0.93, 0.96)	**1.67E-11**
5.71E-01	1.17 (0.94, 1.45)	8.83E-01	1.00 (0.99, 1.02)	8.84E-01

6.02E-01	1.05 (0.83, 1.34)	9.60E-01	0.93 (0.92, 0.95)	**6.98E-15**
9.67E-01	0.87 (0.67, 1.12)	9.52E-01	0.96 (0.94, 0.97)	**7.35E-08**
2.85E-01	1.06 (0.84, 1.35)	9.58E-01	0.94 (0.92, 0.95)	**7.29E-15**
3.07E-01	1.04 (0.82, 1.34)	9.61E-01	0.96 (0.94, 0.98)	**6.37E-06**
6.69E-01	1.09 (0.86, 1.40)	9.52E-01	0.91 (0.90, 0.93)	**5.25E-29**
9.11E-01	1.07 (0.84, 1.35)	9.52E-01	0.95 (0.93, 0.96)	**2.29E-11**
4.96E-01	0.98 (0.76, 1.25)	9.89E-01	0.96 (0.95, 0.98)	**5.15E-07**
5.71E-01	0.99 (0.77, 1.27)	9.92E-01	0.88 (0.86, 0.89)	**2.64E-58**
4.96E-01	0.94 (0.73, 1.21)	9.60E-01	0.89 (0.87, 0.90)	**1.13E-41**
**2.99E-03**	0.88 (0.69, 1.12)	9.52E-01	0.91 (0.89, 0.92)	**1.23E-31**
3.38E-01	0.87 (0.68, 1.11)	9.52E-01	0.89 (0.88, 0.91)	**3.84E-40**
2.47E-01	1.01 (0.78, 1.32)	9.95E-01	0.91 (0.89, 0.92)	**1.60E-32**
1.76E-01	0.91 (0.70, 1.18)	9.52E-01	0.89 (0.88, 0.90)	**1.31E-43**
2.24E-01	0.99 (0.77, 1.28)	9.95E-01	1.00 (0.99, 1.02)	5.72E-01
2.02E-01	0.97 (0.75, 1.26)	9.79E-01	0.99 (0.97, 1.00)	6.35E-02
6.02E-01	0.96 (0.75, 1.23)	9.61E-01	0.91 (0.90, 0.93)	**6.51E-28**
8.00E-01	0.91 (0.70, 1.17)	9.52E-01	0.92 (0.90, 0.93)	**3.02E-28**
7.55E-01	0.93 (0.74, 1.16)	9.52E-01	0.89 (0.88, 0.91)	**6.22E-40**
1.66E-01	1.03 (0.78, 1.37)	9.73E-01	0.95 (0.93, 0.96)	**8.65E-13**
1.86E-01	0.94 (0.74, 1.18)	9.52E-01	0.91 (0.89, 0.92)	**3.11E-35**
2.12E-01	1.01 (0.79, 1.29)	9.92E-01	1.09 (1.08, 1.11)	**1.09E-28**
9.29E-02	0.99 (0.78, 1.26)	9.95E-01	1.07 (1.05, 1.09)	**7.47E-19**
7.20E-01	1.09 (0.86, 1.37)	9.52E-01	1.11 (1.10, 1.12)	**1.14E-82**
**7.70E-03**	0.90 (0.70, 1.15)	9.52E-01	1.03 (1.02, 1.05)	**5.44E-05**
**1.08E-02**	1.29 (1.13, 1.47)	**4.33E-02**	1.07 (1.05, 1.08)	**3.82E-22**
5.71E-01	0.92 (0.72, 1.16)	9.52E-01	1.01 (1.00, 1.03)	1.70E-01
7.95E-01	1.11 (0.89, 1.39)	9.52E-01	0.94 (0.93, 0.96)	**1.20E-12**
6.02E-01	1.00 (0.79, 1.25)	9.95E-01	0.93 (0.91, 0.95)	**2.84E-16**
9.85E-01	0.70 (0.55, 0.89)	1.47E-01	0.98 (0.97, 1.00)	**3.99E-02**
9.11E-01	0.84 (0.67, 1.06)	8.40E-01	0.94 (0.93, 0.96)	**5.59E-13**
8.21E-01	0.85 (0.65, 1.10)	9.52E-01	0.95 (0.94, 0.97)	**2.91E-09**
6.15E-01	0.88 (0.68, 1.14)	9.52E-01	1.05 (1.03, 1.06)	**4.55E-16**
5.71E-01	0.91 (0.71, 1.16)	9.52E-01	1.05 (1.03, 1.06)	**3.46E-09**
2.51E-01	1.18 (1.04, 1.33)	1.79E-01	1.05 (1.04, 1.06)	**4.13E-38**
4.53E-01	1.31 (1.06, 1.62)	1.92E-01	1.07 (1.06, 1.08)	**7.50E-26**
6.02E-01	0.90 (0.65, 1.23)	9.52E-01	1.06 (1.05, 1.07)	**2.29E-21**
9.11E-01	1.04 (0.8, 1.35)	9.62E-01	1.06 (1.05, 1.07)	**9.58E-54**
9.67E-01	1.20 (1.06, 1.34)	1.47E-01	0.87 (0.85, 0.88)	**2.34E-69**
**1.08E-02**	1.19 (1.07, 1.32)	1.47E-01	1.14 (1.12, 1.16)	**7.25E-60**
1.16E-01	0.84 (0.68, 1.03)	6.66E-01	0.99 (0.97, 1.01)	1.91E-01
5.77E-01	0.82 (0.62, 1.07)	8.39E-01	0.95 (0.94, 0.97)	**1.43E-08**
			0.90 (0.89, 0.91)	**4.94E-39**
			0.89 (0.87, 0.90)	**1.97E-49**
			0.88 (0.87, 0.90)	**5.06E-51**
			0.97 (0.95, 0.98)	**1.97E-04**
			0.91 (0.89, 0.92)	**1.10E-30**
			1.08 (1.07, 1.09)	**3.62E-49**
			0.94 (0.93, 0.96)	**5.82E-14**

0.89 (0.87, 0.90)	**1.22E-50**
1.00 (0.98, 1.02)	7.90E-01
1.04 (1.03, 1.05)	**1.62E-12**
1.06 (1.05, 1.07)	**2.94E-30**
0.90 (0.89, 0.92)	**4.34E-31**
0.95 (0.94, 0.97)	**2.98E-09**
0.91 (0.89, 0.92)	**3.94E-33**
0.91 (0.90, 0.93)	**1.43E-24**
0.93 (0.92, 0.95)	**7.17E-18**
0.95 (0.94, 0.97)	**1.30E-09**
0.89 (0.88, 0.90)	**4.86E-47**
0.99 (0.97, 1.01)	3.39E-01
0.92 (0.90, 0.93)	**5.92E-26**
0.95 (0.94, 0.97)	**1.53E-09**
0.89 (0.88, 0.91)	**9.96E-46**
0.98 (0.97, 1.00)	8.90E-02
0.93 (0.92, 0.95)	**9.09E-17**
0.97 (0.96, 0.99)	**2.80E-03**
1.02 (1.00, 1.04)	6.05E-02

mortality			Ischaemic heart
Current smoker		Never/former smoker	
Adjusted HR (95% CI)	P value	Adjusted HR (95% CI)	P value
0.93 (0.90, 0.96)	**4.48E-05**	1.07 (1.02, 1.11)	**6.94E-03**
0.93 (0.90, 0.96)	**4.53E-05**	1.06 (1.01, 1.11)	**1.24E-02**
0.93 (0.90, 0.97)	**1.11E-04**	1.07 (1.02, 1.12)	**5.75E-03**
0.93 (0.90, 0.96)	**2.17E-05**	1.05 (1.00, 1.10)	5.11E-02
0.92 (0.89, 0.96)	**9.90E-06**	1.04 (0.99, 1.09)	1.19E-01
0.93 (0.90, 0.96)	**6.69E-05**	1.06 (1.01, 1.11)	**1.73E-02**
0.93 (0.90, 0.96)	**5.68E-05**	1.06 (1.02, 1.11)	**1.05E-02**
0.88 (0.85, 0.91)	**3.42E-13**	1.04 (1.00, 1.09)	8.85E-02
0.88 (0.85, 0.91)	**2.45E-13**	1.04 (0.99, 1.09)	1.43E-01
0.88 (0.85, 0.91)	**1.61E-12**	1.04 (0.99, 1.09)	1.70E-01
0.86 (0.83, 0.89)	**1.78E-16**	1.00 (0.95, 1.05)	8.68E-01
0.85 (0.83, 0.88)	**4.62E-19**	0.96 (0.92, 1.01)	1.86E-01
0.88 (0.85, 0.91)	**3.60E-13**	1.03 (0.98, 1.08)	3.38E-01
0.88 (0.85, 0.91)	**4.70E-12**	1.05 (1.00, 1.10)	**4.40E-02**
0.86 (0.83, 0.89)	**2.53E-17**	1.02 (0.98, 1.07)	3.64E-01
0.85 (0.82, 0.88)	**7.18E-20**	1.01 (0.96, 1.06)	7.10E-01
0.86 (0.84, 0.90)	**4.41E-16**	1.03 (0.98, 1.08)	2.76E-01
0.85 (0.82, 0.88)	**8.78E-20**	0.99 (0.94, 1.04)	7.36E-01
0.85 (0.82, 0.88)	**1.83E-19**	0.96 (0.91, 1.01)	1.65E-01
0.85 (0.82, 0.88)	**4.60E-19**	1.02 (0.97, 1.07)	5.56E-01
0.84 (0.82, 0.88)	**2.24E-20**	1.01 (0.97, 1.06)	6.68E-01
0.84 (0.81, 0.87)	**1.95E-23**	0.91 (0.87, 0.96)	**7.90E-04**
0.83 (0.80, 0.86)	**1.07E-25**	0.93 (0.89, 0.98)	**7.94E-03**
0.84 (0.82, 0.87)	**9.35E-22**	0.91 (0.87, 0.96)	**4.12E-04**
0.87 (0.84, 0.90)	**8.19E-17**	0.86 (0.82, 0.90)	**1.14E-08**
0.89 (0.86, 0.92)	**1.17E-12**	0.84 (0.80, 0.88)	**8.89E-11**
0.85 (0.82, 0.88)	**1.43E-20**	0.89 (0.85, 0.94)	**2.54E-05**
0.84 (0.81, 0.87)	**4.19E-23**	1.00 (0.95, 1.05)	9.20E-01
0.88 (0.85, 0.91)	**5.11E-14**	1.01 (0.96, 1.06)	6.42E-01
0.87 (0.84, 0.90)	**4.66E-15**	1.00 (0.95, 1.05)	9.40E-01
0.86 (0.83, 0.89)	**7.73E-18**	0.95 (0.90, 1.00)	**4.07E-02**
0.88 (0.85, 0.91)	**5.76E-14**	0.94 (0.89, 0.99)	**2.25E-02**
0.89 (0.86, 0.92)	**8.95E-12**	0.96 (0.91, 1.01)	1.56E-01
0.86 (0.83, 0.89)	**8.73E-18**	0.91 (0.86, 0.95)	**2.17E-04**
0.90 (0.87, 0.93)	**8.03E-10**	1.07 (1.02, 1.12)	**7.56E-03**
0.98 (0.95, 1.01)	2.64E-01	0.98 (0.93, 1.03)	3.99E-01
0.99 (0.96, 1.02)	6.00E-01	0.98 (0.94, 1.03)	5.62E-01
1.01 (0.97, 1.04)	7.73E-01	1.02 (0.97, 1.07)	5.28E-01
0.98 (0.95, 1.02)	3.14E-01	0.90 (0.86, 0.95)	**1.54E-04**
0.98 (0.95, 1.01)	2.55E-01	0.88 (0.83, 0.92)	**2.20E-06**

0.99 (0.96, 1.02)	5.09E-01	0.96 (0.92, 1.01)	1.86E-01
1.00 (0.96, 1.03)	7.78E-01	1.13 (1.08, 1.18)	**4.02E-07**
0.91 (0.88, 0.94)	**1.58E-08**	0.86 (0.82, 0.91)	**4.41E-08**
0.92 (0.89, 0.95)	**1.47E-06**	0.85 (0.80, 0.89)	**8.60E-10**
0.94 (0.91, 0.97)	**1.28E-04**	0.86 (0.82, 0.91)	**4.21E-08**
0.91 (0.88, 0.94)	**2.48E-08**	0.82 (0.78, 0.87)	**7.69E-13**
0.90 (0.87, 0.93)	**4.14E-09**	0.81 (0.77, 0.86)	**8.51E-14**
0.92 (0.89, 0.96)	**4.69E-06**	0.85 (0.80, 0.89)	**5.55E-10**
1.03 (1.00, 1.06)	7.82E-02	1.12 (1.07, 1.17)	**1.36E-06**
0.87 (0.84, 0.90)	**9.08E-17**	0.89 (0.84, 0.93)	**6.90E-06**
0.85 (0.82, 0.88)	**1.66E-20**	0.85 (0.81, 0.90)	**2.19E-09**
0.85 (0.83, 0.88)	**2.98E-20**	0.84 (0.80, 0.89)	**1.59E-10**
0.84 (0.82, 0.87)	**1.81E-22**	0.84 (0.80, 0.88)	**7.20E-11**
0.84 (0.81, 0.87)	**4.91E-24**	0.85 (0.81, 0.89)	**5.46E-10**
0.86 (0.84, 0.89)	**1.49E-17**	0.82 (0.78, 0.87)	**4.52E-13**
1.01 (0.98, 1.04)	5.45E-01	1.12 (1.07, 1.17)	**1.59E-06**
0.84 (0.81, 0.86)	**8.88E-25**	0.89 (0.85, 0.93)	**7.52E-06**
0.82 (0.79, 0.84)	**2.53E-31**	0.88 (0.84, 0.93)	**3.04E-06**
0.81 (0.79, 0.84)	**2.76E-32**	0.88 (0.84, 0.93)	**2.94E-06**
0.81 (0.78, 0.84)	**6.79E-33**	0.87 (0.83, 0.91)	**1.14E-07**
0.81 (0.78, 0.84)	**1.63E-32**	0.88 (0.84, 0.93)	**2.42E-06**
0.82 (0.80, 0.85)	**1.91E-29**	0.84 (0.80, 0.89)	**1.01E-10**
0.96 (0.93, 0.99)	**2.48E-02**	1.11 (1.06, 1.15)	**1.06E-05**
0.85 (0.82, 0.88)	**1.73E-21**	0.91 (0.87, 0.96)	**2.51E-04**
0.83 (0.80, 0.86)	**2.35E-26**	0.90 (0.85, 0.94)	**3.19E-05**
0.85 (0.83, 0.88)	**1.51E-20**	0.91 (0.87, 0.96)	**2.45E-04**
0.82 (0.79, 0.85)	**1.70E-29**	0.88 (0.83, 0.92)	**4.03E-07**
0.82 (0.80, 0.85)	**1.55E-28**	0.89 (0.85, 0.93)	**7.52E-06**
0.84 (0.81, 0.87)	**1.36E-25**	0.86 (0.82, 0.90)	**1.53E-09**
0.93 (0.90, 0.96)	**7.84E-05**	1.08 (1.04, 1.13)	**5.00E-04**
1.16 (1.12, 1.19)	**2.24E-20**	0.92 (0.86, 0.98)	**1.00E-02**
1.18 (1.15, 1.22)	**5.96E-27**	0.92 (0.87, 0.98)	**1.83E-02**
1.20 (1.16, 1.24)	**2.97E-31**	0.94 (0.88, 1.00)	5.03E-02
1.16 (1.13, 1.20)	**1.74E-21**	0.90 (0.84, 0.96)	**1.79E-03**
1.16 (1.12, 1.19)	**1.92E-19**	0.89 (0.83, 0.95)	**4.90E-04**
1.18 (1.15, 1.22)	**3.17E-27**	0.95 (0.90, 1.01)	1.01E-01
1.03 (0.99, 1.06)	1.36E-01	1.07 (1.02, 1.12)	**7.37E-03**
1.15 (1.12, 1.19)	**1.25E-17**	0.87 (0.81, 0.93)	**4.41E-05**
1.16 (1.13, 1.20)	**1.19E-19**	0.88 (0.82, 0.94)	**1.81E-04**
1.17 (1.13, 1.21)	**2.02E-20**	0.89 (0.84, 0.95)	**8.38E-04**
1.16 (1.12, 1.19)	**8.56E-18**	0.86 (0.80, 0.92)	**1.92E-05**
1.15 (1.11, 1.19)	**8.40E-17**	0.85 (0.80, 0.91)	**8.36E-06**
1.17 (1.13, 1.21)	**5.87E-21**	0.88 (0.83, 0.94)	**3.36E-04**
1.08 (1.05, 1.12)	**2.23E-06**	1.04 (0.99, 1.09)	1.89E-01
1.04 (1.00, 1.07)	**4.39E-02**	0.87 (0.82, 0.92)	**1.54E-06**
1.04 (1.00, 1.08)	**3.05E-02**	0.89 (0.84, 0.94)	**3.79E-05**
1.04 (1.01, 1.08)	**2.42E-02**	0.91 (0.86, 0.96)	**5.56E-04**
1.04 (1.01, 1.08)	**2.76E-02**	0.85 (0.81, 0.90)	**2.09E-07**

1.04 (1.00, 1.07)	**3.66E-02**	0.85 (0.81, 0.90)	**1.36E-07**
1.05 (1.01, 1.09)	**9.00E-03**	0.87 (0.82, 0.92)	**2.42E-06**
0.99 (0.96, 1.03)	7.73E-01	1.06 (1.01, 1.11)	**2.41E-02**
0.83 (0.81, 0.86)	**2.23E-25**	0.86 (0.82, 0.91)	**4.04E-08**
0.88 (0.85, 0.91)	**1.88E-13**	0.91 (0.86, 0.95)	**1.50E-04**
0.91 (0.88, 0.94)	**9.77E-08**	0.91 (0.87, 0.96)	**6.25E-04**
0.85 (0.82, 0.87)	**4.88E-22**	0.86 (0.82, 0.90)	**1.29E-08**
0.84 (0.81, 0.86)	**3.76E-25**	0.85 (0.81, 0.89)	**9.98E-10**
0.90 (0.87, 0.93)	**5.32E-10**	0.91 (0.86, 0.95)	**2.70E-04**
0.93 (0.90, 0.96)	**2.82E-05**	1.10 (1.05, 1.15)	**1.22E-04**
1.08 (1.04, 1.12)	**8.18E-05**	1.16 (1.09, 1.23)	**3.92E-06**
1.05 (1.01, 1.08)	**9.00E-03**	0.92 (0.87, 0.98)	**1.18E-02**
1.03 (1.00, 1.07)	7.04E-02	0.90 (0.84, 0.96)	**2.15E-03**
1.06 (1.03, 1.10)	**2.25E-04**	0.98 (0.92, 1.03)	4.57E-01
0.94 (0.91, 0.98)	**6.37E-04**	1.03 (0.97, 1.09)	3.44E-01
1.06 (1.03, 1.10)	**3.38E-04**	0.87 (0.82, 0.93)	**8.30E-05**
1.05 (1.02, 1.09)	**5.56E-03**	0.87 (0.81, 0.93)	**6.17E-05**
1.10 (1.07, 1.14)	**7.36E-09**	0.92 (0.87, 0.98)	**9.50E-03**
0.94 (0.91, 0.97)	**4.22E-04**	1.13 (1.07, 1.20)	**8.92E-05**
0.99 (0.96, 1.03)	7.50E-01	1.12 (1.04, 1.20)	**2.21E-03**
1.10 (1.06, 1.13)	**1.01E-08**	0.96 (0.91, 1.01)	1.28E-01
1.10 (1.07, 1.14)	**1.03E-09**	0.92 (0.87, 0.97)	**3.02E-03**
1.06 (1.02, 1.09)	**1.54E-03**	1.08 (1.02, 1.14)	**9.68E-03**
0.93 (0.89, 0.96)	**1.59E-05**	0.99 (0.94, 1.04)	7.14E-01
1.03 (0.99, 1.06)	1.44E-01	0.88 (0.84, 0.92)	**1.31E-08**
1.03 (1.00, 1.06)	9.32E-02	0.85 (0.81, 0.89)	**1.14E-10**
1.03 (1.00, 1.06)	8.25E-02	0.85 (0.81, 0.89)	**4.16E-11**
1.03 (0.99, 1.06)	1.43E-01	0.87 (0.83, 0.91)	**7.18E-09**
0.97 (0.94, 1.00)	9.79E-02	1.17 (1.12, 1.23)	**1.95E-10**
0.94 (0.91, 0.97)	**1.78E-04**	0.81 (0.77, 0.85)	**1.47E-15**
0.98 (0.95, 1.02)	3.60E-01	0.86 (0.82, 0.90)	**7.96E-10**
1.02 (0.98, 1.05)	3.94E-01	0.91 (0.87, 0.95)	**6.17E-05**
0.95 (0.91, 0.98)	**9.36E-04**	0.80 (0.77, 0.84)	**4.59E-17**
1.03 (1.00, 1.06)	7.77E-02	1.19 (1.13, 1.24)	**1.36E-11**
1.13 (1.10, 1.17)	**1.55E-13**	1.33 (1.27, 1.39)	**9.89E-30**
0.97 (0.94, 1.00)	5.89E-02	0.80 (0.76, 0.83)	**1.01E-23**
0.97 (0.94, 1.00)	5.45E-02	0.79 (0.75, 0.82)	**1.16E-25**
0.98 (0.95, 1.01)	2.10E-01	0.90 (0.86, 0.93)	**3.83E-07**
1.01 (0.98, 1.04)	5.72E-01	1.22 (1.17, 1.27)	**1.95E-18**
1.14 (1.11, 1.18)	**1.63E-16**	1.21 (1.16, 1.27)	**7.84E-15**
0.89 (0.87, 0.92)	**1.07E-13**	0.79 (0.76, 0.82)	**1.32E-33**
0.87 (0.85, 0.90)	**4.16E-19**	0.76 (0.73, 0.79)	**5.32E-41**
0.99 (0.96, 1.02)	4.25E-01	0.92 (0.89, 0.96)	**2.45E-04**
1.09 (1.06, 1.12)	**2.48E-09**	1.24 (1.20, 1.28)	**5.99E-31**
1.05 (1.02, 1.09)	**6.11E-04**	0.96 (0.92, 1.01)	1.19E-01
0.90 (0.89, 0.92)	**5.62E-34**	0.91 (0.90, 0.93)	**1.71E-23**
0.89 (0.88, 0.91)	**1.09E-56**	0.93 (0.91, 0.95)	**4.36E-10**
0.98 (0.95, 1.01)	2.16E-01	0.85 (0.82, 0.88)	**4.57E-19**

1.13 (1.10, 1.15)	**3.48E-26**	1.13 (1.11, 1.16)	**1.77E-32**
1.05 (1.02, 1.08)	**3.55E-03**	1.04 (1.00, 1.09)	8.27E-02
0.90 (0.89, 0.92)	**2.38E-34**	0.91 (0.89, 0.92)	**5.64E-30**
0.87 (0.85, 0.89)	**3.82E-33**	0.91 (0.88, 0.95)	**1.48E-05**
0.99 (0.96, 1.02)	6.31E-01	0.87 (0.84, 0.91)	**6.32E-10**
1.13 (1.10, 1.15)	**5.97E-33**	1.13 (1.11, 1.15)	**1.70E-35**
1.18 (1.14, 1.21)	**2.93E-25**	1.11 (1.06, 1.15)	**5.22E-06**
0.85 (0.83, 0.87)	**6.90E-38**	0.88 (0.86, 0.89)	**2.34E-33**
0.86 (0.83, 0.89)	**1.44E-21**	0.89 (0.85, 0.93)	**3.50E-08**
0.95 (0.92, 0.98)	**2.49E-03**	0.89 (0.86, 0.93)	**3.87E-08**
1.06 (1.03, 1.09)	**4.19E-05**	1.14 (1.11, 1.17)	**4.10E-17**
1.26 (1.21, 1.31)	**8.78E-27**	0.99 (0.95, 1.03)	6.95E-01
0.85 (0.82, 0.88)	**1.22E-21**	0.97 (0.93, 1.01)	1.93E-01
0.92 (0.90, 0.95)	**4.38E-08**	0.92 (0.89, 0.96)	**2.03E-05**
0.87 (0.84, 0.90)	**4.75E-13**	1.05 (1.01, 1.10)	**1.24E-02**
0.90 (0.87, 0.94)	**1.64E-07**	1.04 (1.01, 1.07)	**2.91E-02**
0.98 (0.95, 1.01)	1.32E-01	1.12 (1.08, 1.16)	**3.95E-08**
1.06 (1.03, 1.10)	**3.21E-04**	0.89 (0.86, 0.93)	**1.68E-08**
1.04 (1.01, 1.08)	**9.00E-03**	0.89 (0.86, 0.92)	**5.46E-09**
1.11 (1.08, 1.15)	**5.89E-10**	0.94 (0.91, 0.98)	**4.55E-03**
0.92 (0.89, 0.95)	**6.02E-06**	1.08 (1.05, 1.11)	**4.25E-06**
1.03 (0.99, 1.06)	1.48E-01	1.19 (1.13, 1.24)	**1.57E-12**
1.00 (0.97, 1.03)	8.95E-01	0.86 (0.82, 0.89)	**7.17E-11**
0.99 (0.96, 1.02)	6.71E-01	0.87 (0.84, 0.91)	**1.13E-08**
1.02 (0.99, 1.06)	2.02E-01	0.84 (0.80, 0.88)	**9.02E-11**
0.99 (0.95, 1.02)	4.38E-01	1.14 (1.09, 1.19)	**6.78E-09**
1.22 (1.18, 1.26)	**2.41E-31**	1.05 (1.00, 1.10)	9.30E-02
0.89 (0.86, 0.92)	**5.38E-13**	0.87 (0.83, 0.91)	**7.18E-09**
0.88 (0.85, 0.90)	**2.22E-16**	0.87 (0.83, 0.91)	**6.84E-09**
1.07 (1.03, 1.10)	**1.11E-04**	1.00 (0.95, 1.05)	9.00E-01
0.99 (0.96, 1.03)	7.00E-01	1.20 (1.14, 1.25)	**1.34E-12**
0.83 (0.80, 0.86)	**9.55E-28**	1.01 (0.96, 1.06)	7.85E-01
1.05 (1.01, 1.08)	**6.21E-03**	0.94 (0.90, 0.98)	**1.15E-02**
1.22 (1.18, 1.26)	**5.97E-33**	0.92 (0.86, 0.97)	**7.36E-03**
0.88 (0.85, 0.91)	**3.33E-13**	0.83 (0.79, 0.87)	**6.82E-12**
0.88 (0.85, 0.91)	**8.70E-14**	0.94 (0.89, 0.98)	**1.24E-02**
0.83 (0.80, 0.86)	**2.02E-26**	0.85 (0.81, 0.89)	**8.60E-10**
0.88 (0.85, 0.91)	**3.79E-13**	0.87 (0.83, 0.92)	**3.02E-07**
0.88 (0.85, 0.91)	**5.59E-13**	0.82 (0.78, 0.87)	**1.12E-12**
0.88 (0.85, 0.91)	**2.45E-13**	0.85 (0.81, 0.89)	**8.60E-10**
0.90 (0.87, 0.93)	**1.62E-09**	1.05 (1.01, 1.10)	**3.19E-02**
0.88 (0.85, 0.91)	**5.54E-13**	1.04 (0.99, 1.09)	1.00E-01
0.99 (0.96, 1.02)	4.69E-01	1.11 (1.06, 1.16)	**4.83E-06**
0.99 (0.96, 1.03)	7.07E-01	1.07 (1.02, 1.12)	**5.84E-03**
0.95 (0.92, 0.99)	**9.71E-03**	0.90 (0.85, 0.94)	**6.67E-05**
0.91 (0.88, 0.94)	**1.83E-08**	1.12 (1.07, 1.18)	**3.16E-06**

0.95 (0.92, 0.99)	**7.07E-03**	0.86 (0.82, 0.91)	**1.73E-07**
0.98 (0.95, 1.02)	3.60E-01	0.88 (0.84, 0.93)	**7.20E-06**
0.95 (0.92, 0.98)	**5.28E-03**	0.87 (0.82, 0.92)	**4.87E-07**
1.03 (1.00, 1.07)	7.83E-02	0.86 (0.81, 0.91)	**4.18E-07**
0.86 (0.83, 0.89)	**8.73E-18**	0.89 (0.85, 0.94)	**1.03E-05**
0.88 (0.85, 0.91)	**5.65E-13**	0.97 (0.93, 1.02)	3.45E-01
0.91 (0.88, 0.94)	**6.49E-08**	0.99 (0.95, 1.04)	7.56E-01
0.86 (0.83, 0.88)	**2.88E-20**	0.83 (0.79, 0.87)	**2.17E-13**
0.87 (0.84, 0.90)	**1.75E-14**	0.83 (0.78, 0.87)	**1.85E-10**
0.85 (0.82, 0.88)	**9.09E-21**	0.85 (0.81, 0.90)	**4.45E-09**
0.83 (0.80, 0.86)	**4.66E-24**	0.89 (0.84, 0.94)	**3.79E-05**
0.85 (0.82, 0.88)	**8.68E-20**	0.87 (0.83, 0.92)	**3.83E-07**
0.83 (0.80, 0.86)	**2.23E-25**	0.86 (0.82, 0.91)	**4.09E-08**
0.95 (0.92, 0.98)	**4.54E-03**	1.08 (1.03, 1.13)	**1.08E-03**
0.95 (0.92, 0.98)	**3.31E-03**	1.02 (0.97, 1.06)	5.11E-01
0.92 (0.89, 0.95)	**5.46E-06**	0.85 (0.81, 0.90)	**1.37E-08**
0.90 (0.87, 0.93)	**2.43E-10**	0.80 (0.76, 0.84)	**6.48E-20**
0.84 (0.81, 0.87)	**8.87E-21**	0.89 (0.84, 0.93)	**1.50E-05**
0.96 (0.93, 0.99)	**2.11E-02**	0.86 (0.82, 0.90)	**2.73E-10**
0.91 (0.88, 0.94)	**2.51E-08**	0.83 (0.80, 0.87)	**4.65E-15**
1.04 (1.01, 1.08)	**1.40E-02**	1.26 (1.20, 1.32)	**6.93E-21**
1.12 (1.09, 1.16)	**1.14E-12**	1.07 (1.02, 1.12)	**5.13E-03**
1.05 (1.03, 1.08)	**1.92E-04**	1.19 (1.17, 1.22)	**3.09E-52**
1.06 (1.03, 1.09)	**4.40E-04**	1.07 (1.02, 1.12)	**6.02E-03**
1.08 (1.05, 1.12)	**2.68E-06**	1.11 (1.07, 1.15)	**3.39E-09**
0.92 (0.89, 0.95)	**2.26E-06**	1.09 (1.04, 1.14)	**1.83E-04**
0.96 (0.93, 1.00)	**3.30E-02**	0.90 (0.86, 0.95)	**5.71E-05**
0.87 (0.84, 0.90)	**8.08E-16**	0.91 (0.86, 0.96)	**1.13E-03**
0.94 (0.91, 0.97)	**5.52E-04**	1.08 (1.03, 1.13)	**1.21E-03**
0.87 (0.84, 0.90)	**6.10E-14**	1.06 (1.02, 1.12)	**1.23E-02**
0.84 (0.81, 0.87)	**4.88E-22**	1.09 (1.04, 1.15)	**2.96E-04**
1.01 (0.98, 1.04)	6.89E-01	1.06 (1.04, 1.08)	**3.36E-09**
0.97 (0.94, 1.00)	**4.87E-02**	1.11 (1.06, 1.16)	**6.86E-06**
1.13 (1.11, 1.14)	**1.78E-50**	1.05 (1.03, 1.07)	**3.78E-05**
1.07 (1.05, 1.08)	**2.73E-17**	1.10 (1.07, 1.14)	**5.21E-10**
1.12 (1.10, 1.14)	**2.33E-31**	1.08 (1.04, 1.12)	**3.27E-05**
0.94 (0.90, 0.97)	**1.01E-03**	1.07 (1.06, 1.09)	**3.94E-23**
0.81 (0.79, 0.84)	**3.35E-35**	0.86 (0.82, 0.90)	**2.23E-09**
1.08 (1.05, 1.12)	**1.79E-06**	1.28 (1.22, 1.34)	**2.02E-24**
1.00 (0.97, 1.04)	9.24E-01	0.84 (0.78, 0.89)	**2.63E-07**
0.87 (0.84, 0.90)	**1.97E-15**	1.09 (1.04, 1.14)	**9.01E-04**
0.85 (0.83, 0.88)	**4.45E-20**	0.86 (0.82, 0.90)	**8.69E-09**
0.84 (0.82, 0.87)	**1.81E-22**	0.84 (0.80, 0.88)	**6.08E-11**
0.83 (0.80, 0.86)	**1.49E-27**	0.86 (0.82, 0.90)	**9.01E-09**
1.06 (1.03, 1.10)	**5.75E-04**	0.82 (0.77, 0.87)	**1.95E-09**
0.93 (0.90, 0.96)	**2.17E-05**	0.79 (0.75, 0.83)	**6.90E-18**
1.12 (1.09, 1.15)	**8.50E-19**	1.04 (0.99, 1.08)	1.20E-01
0.87 (0.84, 0.90)	**1.84E-15**	0.97 (0.92, 1.02)	2.09E-01

0.85 (0.82, 0.88)	**1.27E-21**	0.83 (0.79, 0.87)	**3.21E-12**
1.09 (1.05, 1.13)	**9.97E-07**	0.86 (0.81, 0.91)	**4.38E-06**
1.04 (1.01, 1.07)	**6.44E-03**	1.06 (1.03, 1.09)	**1.46E-04**
1.10 (1.08, 1.12)	**1.64E-34**	1.07 (1.04, 1.10)	**6.61E-05**
0.93 (0.90, 0.97)	**1.64E-04**	0.83 (0.79, 0.88)	**9.23E-11**
0.89 (0.86, 0.92)	**4.45E-12**	0.98 (0.93, 1.03)	4.79E-01
0.85 (0.82, 0.88)	**3.35E-20**	0.89 (0.85, 0.93)	**7.17E-06**
0.96 (0.92, 0.99)	**1.47E-02**	0.84 (0.79, 0.89)	**1.11E-09**
0.93 (0.90, 0.97)	**9.12E-05**	0.87 (0.83, 0.92)	**4.87E-07**
0.88 (0.85, 0.91)	**1.68E-13**	0.99 (0.95, 1.04)	8.29E-01
0.84 (0.81, 0.87)	**2.15E-24**	0.86 (0.82, 0.91)	**1.56E-08**
1.08 (1.04, 1.12)	**1.43E-05**	0.88 (0.83, 0.94)	**5.35E-05**
0.88 (0.85, 0.91)	**2.45E-13**	0.89 (0.85, 0.94)	**8.55E-06**
0.87 (0.84, 0.90)	**9.68E-15**	1.01 (0.96, 1.06)	8.34E-01
0.84 (0.81, 0.87)	**1.66E-24**	0.86 (0.82, 0.91)	**3.71E-08**
1.08 (1.04, 1.11)	**5.22E-05**	0.86 (0.81, 0.92)	**2.98E-06**
0.89 (0.86, 0.92)	**6.09E-12**	0.91 (0.87, 0.96)	**7.84E-04**
1.07 (1.03, 1.11)	**1.47E-04**	0.83 (0.78, 0.88)	**8.46E-09**
1.01 (0.97, 1.04)	7.85E-01	0.99 (0.93, 1.05)	7.45E-01

diseases mortality			Cerebrovascula
Current smoker		Never/former smoker	
Adjusted HR (95% CI)	P value	Adjusted HR (95% CI)	P value
0.95 (0.87, 1.04)	3.87E-01	0.91 (0.84, 0.98)	**2.94E-02**
0.95 (0.87, 1.04)	3.88E-01	0.91 (0.84, 0.98)	**2.86E-02**
0.95 (0.87, 1.04)	3.94E-01	0.91 (0.84, 0.99)	**4.24E-02**
0.95 (0.86, 1.04)	3.22E-01	0.91 (0.84, 0.98)	**2.65E-02**
0.94 (0.86, 1.04)	3.14E-01	0.90 (0.83, 0.97)	**1.91E-02**
0.95 (0.87, 1.04)	3.45E-01	0.91 (0.84, 0.99)	**4.14E-02**
0.96 (0.88, 1.05)	4.15E-01	0.91 (0.84, 0.98)	**2.86E-02**
0.93 (0.85, 1.02)	2.27E-01	0.87 (0.80, 0.94)	**2.07E-03**
0.93 (0.85, 1.02)	2.02E-01	0.87 (0.80, 0.94)	**1.93E-03**
0.93 (0.85, 1.02)	2.21E-01	0.87 (0.81, 0.95)	**2.49E-03**
0.92 (0.84, 1.01)	1.37E-01	0.86 (0.79, 0.93)	**6.67E-04**
0.91 (0.83, 1.00)	9.77E-02	0.85 (0.79, 0.92)	**2.57E-04**
0.93 (0.85, 1.02)	2.02E-01	0.87 (0.81, 0.95)	**2.29E-03**
0.93 (0.85, 1.02)	2.27E-01	0.88 (0.81, 0.95)	**3.36E-03**
0.92 (0.84, 1.01)	1.46E-01	0.86 (0.80, 0.94)	**1.02E-03**
0.91 (0.83, 1.00)	1.07E-01	0.86 (0.79, 0.93)	**7.24E-04**
0.93 (0.85, 1.02)	1.94E-01	0.87 (0.80, 0.94)	**1.44E-03**
0.91 (0.83, 1.00)	1.28E-01	0.86 (0.80, 0.93)	**7.52E-04**
0.91 (0.83, 1.00)	1.12E-01	0.87 (0.80, 0.94)	**1.00E-03**
0.92 (0.84, 1.01)	1.48E-01	0.86 (0.79, 0.93)	**7.60E-04**
0.90 (0.82, 0.99)	8.46E-02	0.86 (0.79, 0.93)	**7.64E-04**
0.88 (0.81, 0.97)	**3.31E-02**	0.84 (0.78, 0.91)	**7.77E-05**
0.88 (0.80, 0.97)	**3.08E-02**	0.84 (0.78, 0.91)	**7.68E-05**
0.89 (0.81, 0.98)	**4.13E-02**	0.84 (0.78, 0.91)	**8.80E-05**
0.89 (0.81, 0.98)	**4.83E-02**	0.86 (0.80, 0.93)	**6.21E-04**
0.90 (0.82, 0.99)	7.22E-02	0.88 (0.82, 0.95)	**2.46E-03**
0.89 (0.81, 0.98)	**4.31E-02**	0.85 (0.79, 0.92)	**1.68E-04**
0.90 (0.82, 0.99)	6.14E-02	0.86 (0.79, 0.93)	**5.43E-04**
0.95 (0.86, 1.04)	3.22E-01	0.88 (0.82, 0.95)	**3.46E-03**
0.94 (0.85, 1.03)	2.44E-01	0.87 (0.81, 0.94)	**1.74E-03**
0.92 (0.84, 1.01)	1.48E-01	0.86 (0.80, 0.93)	**4.40E-04**
0.93 (0.85, 1.02)	2.29E-01	0.87 (0.80, 0.94)	**1.03E-03**
0.95 (0.87, 1.04)	3.53E-01	0.88 (0.81, 0.95)	**2.17E-03**
0.91 (0.83, 1.00)	1.06E-01	0.86 (0.79, 0.93)	**3.27E-04**
0.96 (0.88, 1.05)	4.62E-01	0.91 (0.84, 0.98)	**3.06E-02**
1.00 (0.91, 1.09)	9.53E-01	0.91 (0.84, 0.98)	**2.86E-02**
1.00 (0.92, 1.10)	9.46E-01	0.92 (0.85, 0.99)	5.24E-02
1.03 (0.94, 1.13)	6.18E-01	0.94 (0.87, 1.01)	1.23E-01
0.97 (0.89, 1.06)	6.03E-01	0.91 (0.85, 0.98)	**3.15E-02**
0.96 (0.87, 1.05)	4.31E-01	0.91 (0.84, 0.98)	**2.59E-02**

1.00 (0.92, 1.10)	9.60E-01	0.92 (0.86, 1.00)	6.45E-02
1.04 (0.96, 1.14)	4.16E-01	0.95 (0.88, 1.03)	2.66E-01
0.92 (0.84, 1.01)	1.42E-01	0.85 (0.79, 0.91)	**9.87E-05**
0.91 (0.83, 0.99)	9.25E-02	0.86 (0.79, 0.92)	**3.11E-04**
0.92 (0.84, 1.01)	1.55E-01	0.87 (0.81, 0.94)	**1.18E-03**
0.89 (0.81, 0.98)	**4.25E-02**	0.85 (0.79, 0.92)	**1.92E-04**
0.89 (0.81, 0.97)	**3.31E-02**	0.84 (0.78, 0.91)	**8.80E-05**
0.91 (0.83, 1.00)	9.77E-02	0.88 (0.81, 0.95)	**1.88E-03**
1.06 (0.98, 1.16)	2.32E-01	0.95 (0.88, 1.02)	2.33E-01
0.91 (0.83, 1.00)	9.76E-02	0.86 (0.79, 0.93)	**3.06E-04**
0.87 (0.79, 0.95)	**1.44E-02**	0.82 (0.76, 0.89)	**5.19E-06**
0.87 (0.80, 0.96)	**1.66E-02**	0.82 (0.76, 0.89)	**5.19E-06**
0.86 (0.78, 0.94)	**8.32E-03**	0.82 (0.76, 0.89)	**4.65E-06**
0.86 (0.78, 0.94)	**8.13E-03**	0.82 (0.76, 0.88)	**3.06E-06**
0.87 (0.79, 0.95)	**1.27E-02**	0.83 (0.77, 0.90)	**2.53E-05**
1.05 (0.97, 1.15)	3.15E-01	0.92 (0.85, 1.00)	6.90E-02
0.88 (0.80, 0.96)	**2.10E-02**	0.82 (0.76, 0.89)	**5.90E-06**
0.87 (0.79, 0.95)	**1.25E-02**	0.80 (0.74, 0.86)	**6.43E-07**
0.87 (0.79, 0.95)	**1.52E-02**	0.80 (0.74, 0.87)	**6.43E-07**
0.86 (0.78, 0.94)	**8.13E-03**	0.80 (0.74, 0.86)	**5.98E-07**
0.86 (0.78, 0.94)	**8.32E-03**	0.80 (0.74, 0.86)	**5.98E-07**
0.86 (0.79, 0.94)	**8.77E-03**	0.82 (0.76, 0.88)	**2.30E-06**
1.02 (0.93, 1.11)	7.82E-01	0.90 (0.83, 0.98)	**2.20E-02**
0.89 (0.81, 0.97)	**3.31E-02**	0.83 (0.77, 0.90)	**1.70E-05**
0.89 (0.81, 0.97)	**3.31E-02**	0.82 (0.76, 0.88)	**2.50E-06**
0.92 (0.84, 1.01)	1.32E-01	0.84 (0.77, 0.90)	**1.96E-05**
0.87 (0.79, 0.95)	**1.39E-02**	0.81 (0.75, 0.87)	**1.69E-06**
0.87 (0.79, 0.95)	**1.43E-02**	0.81 (0.75, 0.87)	**1.57E-06**
0.88 (0.81, 0.97)	**2.81E-02**	0.84 (0.78, 0.90)	**1.58E-05**
0.98 (0.90, 1.07)	7.34E-01	0.90 (0.83, 0.97)	**1.90E-02**
1.03 (0.94, 1.14)	5.76E-01	1.10 (1.02, 1.19)	**3.27E-02**
1.06 (0.96, 1.17)	3.13E-01	1.13 (1.04, 1.22)	**5.44E-03**
1.07 (0.98, 1.18)	2.29E-01	1.14 (1.06, 1.23)	**1.92E-03**
1.05 (0.95, 1.15)	4.31E-01	1.11 (1.03, 1.20)	**1.64E-02**
1.03 (0.94, 1.14)	6.03E-01	1.11 (1.02, 1.20)	**1.92E-02**
1.09 (1.00, 1.19)	1.23E-01	1.11 (1.03, 1.20)	**1.16E-02**
1.00 (0.92, 1.10)	9.45E-01	0.97 (0.90, 1.05)	5.61E-01
0.98 (0.89, 1.09)	8.17E-01	1.09 (1.01, 1.19)	**5.00E-02**
0.99 (0.90, 1.10)	9.42E-01	1.10 (1.01, 1.19)	**4.14E-02**
1.00 (0.90, 1.11)	9.96E-01	1.09 (1.01, 1.19)	5.26E-02
0.99 (0.89, 1.10)	8.96E-01	1.10 (1.02, 1.19)	**3.15E-02**
0.98 (0.89, 1.09)	8.35E-01	1.10 (1.02, 1.20)	**2.95E-02**
1.01 (0.91, 1.12)	9.11E-01	1.10 (1.01, 1.19)	**4.17E-02**
0.99 (0.91, 1.09)	9.41E-01	1.01 (0.93, 1.09)	9.00E-01
0.90 (0.82, 0.99)	9.07E-02	0.96 (0.88, 1.04)	3.48E-01
0.91 (0.83, 1.01)	1.31E-01	0.95 (0.88, 1.03)	3.04E-01
0.92 (0.84, 1.01)	1.56E-01	0.95 (0.88, 1.03)	2.66E-01
0.91 (0.82, 1.00)	1.11E-01	0.97 (0.89, 1.05)	4.91E-01

0.91 (0.82, 1.00)	1.08E-01	0.97 (0.89, 1.05)	5.16E-01
0.91 (0.82, 1.01)	1.35E-01	0.96 (0.88, 1.04)	4.01E-01
0.98 (0.89, 1.07)	6.60E-01	0.93 (0.87, 1.01)	1.23E-01
0.83 (0.76, 0.91)	**8.20E-04**	0.81 (0.75, 0.87)	**1.57E-06**
0.86 (0.78, 0.94)	**8.13E-03**	0.83 (0.77, 0.89)	**1.48E-05**
0.87 (0.79, 0.96)	**1.60E-02**	0.84 (0.78, 0.91)	**1.07E-04**
0.83 (0.76, 0.91)	**1.26E-03**	0.82 (0.76, 0.88)	**3.06E-06**
0.83 (0.75, 0.91)	**7.30E-04**	0.82 (0.76, 0.88)	**3.06E-06**
0.87 (0.80, 0.96)	**1.75E-02**	0.84 (0.77, 0.91)	**5.60E-05**
0.98 (0.90, 1.07)	7.37E-01	0.90 (0.83, 0.98)	**1.93E-02**
1.10 (0.99, 1.22)	1.54E-01	1.06 (0.97, 1.15)	2.33E-01
1.05 (0.96, 1.15)	3.74E-01	1.07 (0.99, 1.15)	1.23E-01
1.05 (0.96, 1.15)	3.73E-01	1.03 (0.95, 1.11)	5.57E-01
1.04 (0.95, 1.14)	5.13E-01	1.12 (1.04, 1.19)	**2.78E-03**
0.94 (0.86, 1.03)	2.42E-01	0.93 (0.86, 1.00)	8.03E-02
0.99 (0.90, 1.10)	9.36E-01	1.09 (1.01, 1.18)	**4.33E-02**
0.99 (0.89, 1.09)	8.42E-01	1.07 (0.99, 1.16)	1.30E-01
1.02 (0.93, 1.13)	6.92E-01	1.15 (1.07, 1.23)	**6.60E-04**
1.01 (0.91, 1.11)	9.42E-01	0.92 (0.86, 1.00)	6.99E-02
1.08 (0.97, 1.21)	2.58E-01	0.93 (0.86, 1.00)	8.13E-02
1.09 (1.00, 1.19)	9.77E-02	1.06 (0.98, 1.14)	1.60E-01
1.07 (0.98, 1.17)	2.07E-01	1.09 (1.01, 1.18)	**4.14E-02**
1.14 (1.05, 1.25)	**1.60E-02**	0.97 (0.90, 1.05)	5.97E-01
0.90 (0.81, 0.98)	6.12E-02	1.00 (0.92, 1.08)	9.42E-01
0.96 (0.88, 1.04)	3.92E-01	0.96 (0.90, 1.04)	3.83E-01
0.95 (0.87, 1.04)	3.56E-01	1.01 (0.94, 1.09)	7.71E-01
0.95 (0.87, 1.04)	3.47E-01	1.02 (0.95, 1.10)	6.43E-01
0.96 (0.88, 1.04)	4.06E-01	0.99 (0.92, 1.07)	8.81E-01
1.05 (0.96, 1.14)	3.56E-01	1.00 (0.93, 1.07)	9.58E-01
0.89 (0.82, 0.98)	**4.23E-02**	0.93 (0.86, 1.00)	7.91E-02
0.95 (0.87, 1.03)	3.03E-01	0.96 (0.89, 1.04)	3.73E-01
0.99 (0.91, 1.08)	8.78E-01	0.98 (0.92, 1.06)	7.46E-01
0.89 (0.82, 0.98)	**4.12E-02**	0.94 (0.87, 1.01)	1.25E-01
1.07 (0.98, 1.17)	1.86E-01	1.05 (0.98, 1.13)	2.38E-01
1.25 (1.14, 1.36)	**3.24E-05**	1.15 (1.07, 1.24)	**6.44E-04**
0.89 (0.82, 0.96)	**1.75E-02**	0.95 (0.88, 1.02)	1.90E-01
0.88 (0.81, 0.95)	**1.16E-02**	0.94 (0.87, 1.01)	1.42E-01
0.97 (0.90, 1.05)	5.74E-01	1.00 (0.93, 1.07)	9.85E-01
1.09 (1.01, 1.19)	8.18E-02	1.03 (0.96, 1.11)	4.87E-01
1.14 (1.05, 1.25)	**1.21E-02**	1.17 (1.08, 1.25)	**1.59E-04**
0.85 (0.79, 0.92)	**5.35E-04**	0.87 (0.82, 0.93)	**1.85E-04**
0.83 (0.77, 0.90)	**6.79E-05**	0.84 (0.78, 0.89)	**2.30E-06**
0.95 (0.88, 1.03)	3.22E-01	1.02 (0.95, 1.09)	7.46E-01
1.15 (1.07, 1.24)	**1.42E-03**	1.12 (1.05, 1.19)	**1.74E-03**
1.08 (1.01, 1.16)	8.85E-02	1.07 (1.01, 1.13)	**4.17E-02**
0.89 (0.86, 0.93)	**2.18E-07**	0.91 (0.89, 0.94)	**5.98E-07**
0.90 (0.87, 0.93)	**2.18E-07**	0.91 (0.89, 0.94)	**5.07E-08**
0.90 (0.84, 0.97)	**3.12E-02**	0.94 (0.88, 1.01)	1.23E-01

1.15 (1.09, 1.20)	**3.36E-06**	1.12 (1.07, 1.16)	**3.06E-06**
1.10 (1.03, 1.16)	**1.38E-02**	1.07 (1.00, 1.14)	7.88E-02
0.90 (0.87, 0.93)	**2.82E-06**	0.91 (0.88, 0.94)	**1.79E-06**
0.88 (0.83, 0.94)	**6.29E-04**	0.87 (0.83, 0.92)	**1.89E-06**
0.94 (0.86, 1.02)	2.06E-01	1.01 (0.94, 1.09)	8.57E-01
1.12 (1.07, 1.18)	**4.73E-05**	1.10 (1.06, 1.15)	**5.28E-06**
1.21 (1.12, 1.32)	**7.16E-05**	1.15 (1.09, 1.22)	**1.14E-05**
0.83 (0.78, 0.88)	**2.18E-07**	0.89 (0.85, 0.92)	**5.98E-07**
0.83 (0.76, 0.90)	**1.37E-04**	0.87 (0.82, 0.92)	**1.74E-05**
0.95 (0.87, 1.03)	2.65E-01	0.97 (0.91, 1.04)	5.11E-01
1.07 (1.00, 1.14)	9.90E-02	1.06 (0.99, 1.13)	1.54E-01
1.11 (1.01, 1.22)	7.70E-02	1.17 (1.07, 1.27)	**1.72E-03**
0.95 (0.87, 1.03)	2.87E-01	0.89 (0.82, 0.96)	**6.14E-03**
0.94 (0.88, 1.02)	2.21E-01	0.95 (0.88, 1.01)	1.32E-01
0.99 (0.91, 1.08)	8.92E-01	0.90 (0.83, 0.98)	**3.42E-02**
0.92 (0.84, 1.01)	1.55E-01	0.92 (0.85, 1.00)	6.90E-02
1.06 (0.98, 1.14)	2.42E-01	0.95 (0.88, 1.02)	1.77E-01
0.97 (0.90, 1.05)	5.82E-01	1.05 (0.98, 1.13)	2.25E-01
0.96 (0.89, 1.04)	4.24E-01	1.06 (0.98, 1.14)	1.90E-01
1.03 (0.95, 1.11)	6.03E-01	1.02 (0.95, 1.09)	6.94E-01
0.99 (0.92, 1.08)	9.13E-01	0.97 (0.89, 1.04)	4.36E-01
1.11 (1.02, 1.21)	**4.55E-02**	1.01 (0.94, 1.09)	7.68E-01
0.93 (0.85, 1.01)	1.56E-01	1.01 (0.94, 1.09)	8.57E-01
0.94 (0.87, 1.03)	2.65E-01	1.02 (0.95, 1.10)	6.51E-01
0.89 (0.82, 0.98)	**4.48E-02**	0.95 (0.88, 1.03)	3.13E-01
1.05 (0.97, 1.13)	3.56E-01	0.98 (0.90, 1.05)	5.91E-01
1.07 (0.98, 1.17)	2.18E-01	1.12 (1.04, 1.21)	**6.75E-03**
0.92 (0.84, 1.00)	9.80E-02	0.94 (0.87, 1.01)	1.51E-01
0.91 (0.83, 0.99)	6.81E-02	0.93 (0.87, 1.01)	1.01E-01
1.04 (0.95, 1.13)	4.79E-01	1.03 (0.96, 1.11)	5.19E-01
1.07 (0.98, 1.17)	1.94E-01	0.98 (0.91, 1.06)	7.51E-01
0.89 (0.81, 0.97)	**3.31E-02**	0.84 (0.78, 0.91)	**7.77E-05**
1.04 (0.95, 1.13)	4.79E-01	1.06 (0.98, 1.14)	1.72E-01
1.02 (0.92, 1.12)	8.22E-01	1.15 (1.06, 1.25)	**1.49E-03**
0.86 (0.79, 0.95)	**1.06E-02**	0.84 (0.78, 0.91)	**5.60E-05**
0.92 (0.84, 1.01)	1.31E-01	0.86 (0.80, 0.93)	**6.24E-04**
0.86 (0.78, 0.94)	**8.13E-03**	0.81 (0.75, 0.88)	**1.89E-06**
0.90 (0.82, 0.98)	5.46E-02	0.85 (0.78, 0.91)	**9.34E-05**
0.86 (0.78, 0.94)	**8.32E-03**	0.84 (0.78, 0.91)	**5.60E-05**
0.87 (0.80, 0.96)	**1.75E-02**	0.84 (0.78, 0.91)	**7.77E-05**
0.95 (0.86, 1.04)	3.20E-01	0.89 (0.82, 0.96)	**5.49E-03**
0.93 (0.85, 1.02)	2.07E-01	0.88 (0.81, 0.95)	**3.32E-03**
1.03 (0.95, 1.13)	5.28E-01	0.91 (0.85, 0.99)	**4.14E-02**
0.98 (0.90, 1.07)	7.60E-01	0.94 (0.87, 1.01)	1.42E-01
0.89 (0.81, 0.98)	**4.16E-02**	0.88 (0.81, 0.95)	**2.94E-03**
1.01 (0.92, 1.10)	8.93E-01	0.93 (0.86, 1.00)	8.06E-02

0.87 (0.79, 0.96)	**1.97E-02**	0.87 (0.81, 0.95)	**2.66E-03**
0.94 (0.86, 1.03)	2.97E-01	0.90 (0.83, 0.98)	**2.25E-02**
0.88 (0.80, 0.97)	**2.84E-02**	0.88 (0.81, 0.95)	**3.04E-03**
0.89 (0.81, 0.99)	7.61E-02	0.95 (0.87, 1.03)	2.73E-01
0.90 (0.82, 0.99)	6.81E-02	0.84 (0.78, 0.91)	**5.60E-05**
0.98 (0.90, 1.07)	7.41E-01	0.89 (0.82, 0.96)	**6.75E-03**
0.92 (0.84, 1.00)	1.31E-01	0.86 (0.80, 0.93)	**6.67E-04**
0.86 (0.79, 0.94)	**8.13E-03**	0.96 (0.89, 1.04)	3.78E-01
0.87 (0.79, 0.96)	**2.33E-02**	0.91 (0.84, 0.99)	**4.14E-02**
0.83 (0.76, 0.91)	**1.56E-03**	0.86 (0.80, 0.93)	**6.98E-04**
0.85 (0.77, 0.94)	**8.32E-03**	0.88 (0.81, 0.95)	**3.49E-03**
0.84 (0.76, 0.92)	**2.89E-03**	0.85 (0.78, 0.92)	**1.69E-04**
0.82 (0.75, 0.91)	**1.01E-03**	0.84 (0.77, 0.91)	**5.69E-05**
0.98 (0.89, 1.07)	6.63E-01	0.90 (0.84, 0.98)	**1.97E-02**
0.95 (0.87, 1.04)	3.22E-01	0.87 (0.80, 0.94)	**1.34E-03**
0.93 (0.85, 1.02)	2.20E-01	1.00 (0.93, 1.08)	9.80E-01
0.86 (0.79, 0.93)	**4.35E-03**	0.99 (0.92, 1.07)	9.30E-01
0.88 (0.80, 0.97)	**3.66E-02**	0.94 (0.87, 1.02)	1.61E-01
0.93 (0.85, 1.01)	1.55E-01	1.06 (0.98, 1.14)	2.18E-01
0.89 (0.82, 0.97)	**3.31E-02**	1.03 (0.95, 1.11)	5.76E-01
1.12 (1.03, 1.22)	**3.78E-02**	1.00 (0.93, 1.08)	9.80E-01
1.07 (0.98, 1.16)	2.21E-01	0.95 (0.89, 1.03)	2.47E-01
1.14 (1.08, 1.20)	**7.87E-05**	1.09 (1.03, 1.15)	**3.32E-03**
1.07 (0.98, 1.16)	2.13E-01	0.98 (0.91, 1.06)	6.45E-01
1.13 (1.04, 1.24)	**1.75E-02**	1.07 (1.01, 1.13)	**4.60E-02**
1.07 (0.98, 1.16)	1.94E-01	1.01 (0.94, 1.09)	7.95E-01
0.97 (0.89, 1.06)	6.06E-01	1.10 (1.03, 1.19)	**1.72E-02**
0.81 (0.74, 0.89)	**2.20E-04**	1.00 (0.92, 1.07)	9.42E-01
0.99 (0.91, 1.08)	8.97E-01	0.93 (0.86, 1.01)	1.23E-01
0.92 (0.84, 1.01)	1.56E-01	0.87 (0.80, 0.94)	**1.85E-03**
0.92 (0.84, 1.01)	1.56E-01	0.88 (0.81, 0.95)	**2.77E-03**
0.97 (0.88, 1.07)	6.03E-01	1.01 (0.94, 1.09)	7.83E-01
0.97 (0.89, 1.06)	6.18E-01	1.03 (0.96, 1.10)	5.35E-01
1.06 (1.00, 1.13)	9.76E-02	1.06 (1.02, 1.09)	**4.97E-03**
1.06 (1.00, 1.11)	8.17E-02	1.07 (1.01, 1.13)	**4.78E-02**
1.08 (1.02, 1.14)	**4.13E-02**	1.06 (1.00, 1.12)	1.04E-01
0.99 (0.91, 1.08)	8.90E-01	1.06 (1.03, 1.10)	**7.66E-04**
0.81 (0.74, 0.88)	**4.86E-05**	0.93 (0.87, 1.01)	1.11E-01
1.18 (1.08, 1.29)	**2.83E-03**	1.05 (0.98, 1.14)	2.20E-01
1.02 (0.92, 1.14)	7.45E-01	1.08 (1.00, 1.17)	8.06E-02
0.93 (0.85, 1.03)	2.31E-01	0.88 (0.81, 0.95)	**3.90E-03**
0.87 (0.80, 0.96)	**1.75E-02**	0.83 (0.77, 0.89)	**8.32E-06**
0.87 (0.79, 0.95)	**1.38E-02**	0.82 (0.76, 0.89)	**4.65E-06**
0.86 (0.78, 0.94)	**8.13E-03**	0.81 (0.75, 0.87)	**1.57E-06**
0.91 (0.82, 1.01)	1.42E-01	1.01 (0.92, 1.09)	9.35E-01
0.87 (0.80, 0.96)	**1.69E-02**	1.02 (0.95, 1.11)	6.39E-01
1.06 (0.98, 1.14)	2.11E-01	1.07 (1.01, 1.13)	**2.95E-02**
0.92 (0.84, 1.01)	1.32E-01	0.86 (0.80, 0.93)	**5.45E-04**

0.87 (0.79, 0.95)	**1.21E-02**	0.83 (0.77, 0.89)	**1.03E-05**
0.94 (0.85, 1.04)	3.22E-01	1.00 (0.92, 1.09)	9.97E-01
1.06 (1.00, 1.14)	1.31E-01	0.99 (0.92, 1.08)	9.30E-01
1.09 (1.04, 1.14)	**5.85E-03**	1.07 (1.03, 1.12)	**1.08E-03**
0.85 (0.77, 0.93)	**7.87E-03**	0.87 (0.80, 0.94)	**1.86E-03**
0.94 (0.85, 1.03)	2.31E-01	0.87 (0.80, 0.94)	**1.10E-03**
0.90 (0.82, 0.98)	5.05E-02	0.84 (0.78, 0.91)	**5.60E-05**
0.86 (0.78, 0.95)	**1.21E-02**	0.89 (0.82, 0.97)	**1.12E-02**
0.88 (0.80, 0.97)	**2.94E-02**	0.86 (0.79, 0.93)	**5.86E-04**
0.93 (0.85, 1.02)	1.94E-01	0.87 (0.80, 0.94)	**8.48E-04**
0.88 (0.80, 0.96)	**1.83E-02**	0.81 (0.75, 0.88)	**2.30E-06**
0.93 (0.84, 1.03)	2.42E-01	0.99 (0.91, 1.07)	7.95E-01
0.87 (0.80, 0.96)	**1.69E-02**	0.84 (0.77, 0.90)	**2.93E-05**
0.92 (0.84, 1.01)	1.56E-01	0.86 (0.80, 0.93)	**8.48E-04**
0.87 (0.79, 0.95)	**1.39E-02**	0.81 (0.75, 0.88)	**2.30E-06**
0.92 (0.83, 1.02)	1.98E-01	0.99 (0.91, 1.08)	8.52E-01
0.92 (0.84, 1.01)	1.41E-01	0.87 (0.80, 0.94)	**8.83E-04**
0.92 (0.83, 1.02)	1.65E-01	1.00 (0.92, 1.09)	9.45E-01
1.00 (0.91, 1.11)	9.70E-01	0.98 (0.90, 1.06)	6.94E-01

r diseases mortality		Cancer r	
Current smoker		Never/former smoker	
Adjusted HR (95% CI)	P value	Adjusted HR (95% CI)	P value
1.02 (0.87, 1.19)	9.76E-01	1.03 (1.01, 1.06)	**4.97E-03**
1.01 (0.86, 1.18)	9.76E-01	1.03 (1.01, 1.05)	**7.71E-03**
1.02 (0.87, 1.19)	9.76E-01	1.03 (1.01, 1.06)	**4.89E-03**
1.04 (0.89, 1.21)	9.42E-01	1.03 (1.01, 1.05)	**2.04E-02**
1.06 (0.90, 1.23)	9.42E-01	1.03 (1.00, 1.05)	**3.89E-02**
1.02 (0.87, 1.19)	9.76E-01	1.03 (1.01, 1.05)	**9.55E-03**
1.00 (0.85, 1.17)	9.95E-01	1.03 (1.01, 1.05)	**6.95E-03**
1.00 (0.85, 1.17)	9.95E-01	1.02 (1.00, 1.04)	1.48E-01
0.99 (0.84, 1.16)	9.76E-01	1.02 (0.99, 1.04)	1.96E-01
0.99 (0.85, 1.16)	9.76E-01	1.02 (1.00, 1.04)	1.43E-01
0.99 (0.85, 1.16)	9.76E-01	1.01 (0.98, 1.03)	6.93E-01
0.99 (0.85, 1.16)	9.76E-01	1.00 (0.97, 1.02)	7.15E-01
0.99 (0.85, 1.16)	9.76E-01	1.02 (0.99, 1.04)	2.08E-01
0.99 (0.84, 1.16)	9.76E-01	1.02 (1.00, 1.04)	1.30E-01
0.99 (0.85, 1.16)	9.76E-01	1.01 (0.99, 1.03)	5.09E-01
0.97 (0.83, 1.14)	9.63E-01	1.00 (0.98, 1.02)	8.95E-01
1.00 (0.85, 1.17)	9.95E-01	1.01 (0.99, 1.03)	3.88E-01
0.99 (0.84, 1.16)	9.76E-01	1.00 (0.98, 1.02)	9.27E-01
0.99 (0.84, 1.16)	9.76E-01	1.00 (0.98, 1.02)	8.49E-01
0.99 (0.84, 1.16)	9.76E-01	1.01 (0.98, 1.03)	7.03E-01
0.96 (0.81, 1.13)	9.42E-01	1.00 (0.98, 1.02)	9.03E-01
0.98 (0.83, 1.14)	9.72E-01	0.97 (0.95, 1.00)	**3.01E-02**
0.97 (0.83, 1.14)	9.42E-01	0.98 (0.96, 1.00)	**4.91E-02**
0.98 (0.84, 1.15)	9.76E-01	0.97 (0.95, 1.00)	**3.05E-02**
0.96 (0.82, 1.12)	9.42E-01	0.96 (0.94, 0.98)	**4.34E-04**
0.96 (0.82, 1.12)	9.42E-01	0.95 (0.93, 0.97)	**4.38E-05**
0.97 (0.83, 1.13)	9.42E-01	0.97 (0.95, 0.99)	**1.09E-02**
0.98 (0.83, 1.15)	9.76E-01	0.99 (0.97, 1.01)	5.55E-01
1.05 (0.90, 1.22)	9.42E-01	1.01 (0.99, 1.03)	5.20E-01
1.04 (0.89, 1.21)	9.42E-01	1.00 (0.98, 1.03)	7.78E-01
1.01 (0.87, 1.18)	9.76E-01	0.98 (0.96, 1.01)	2.07E-01
1.02 (0.87, 1.19)	9.76E-01	0.99 (0.97, 1.01)	3.36E-01
1.03 (0.88, 1.20)	9.42E-01	1.00 (0.98, 1.02)	8.56E-01
0.99 (0.85, 1.16)	9.76E-01	0.97 (0.95, 0.99)	**1.63E-02**
1.06 (0.91, 1.24)	9.42E-01	1.03 (1.00, 1.05)	**3.01E-02**
1.11 (0.95, 1.29)	8.16E-01	1.03 (1.01, 1.05)	**1.70E-02**
1.11 (0.95, 1.29)	8.16E-01	1.03 (1.01, 1.06)	**5.46E-03**
1.12 (0.96, 1.30)	8.16E-01	1.05 (1.02, 1.07)	**9.83E-05**
1.06 (0.91, 1.23)	9.42E-01	1.01 (0.99, 1.03)	5.85E-01
1.04 (0.89, 1.22)	9.42E-01	1.00 (0.98, 1.02)	8.96E-01

1.09 (0.94, 1.27)	8.48E-01	1.03 (1.01, 1.05)	**1.99E-02**
1.16 (1.01, 1.34)	5.73E-01	1.06 (1.04, 1.09)	**8.00E-08**
0.99 (0.84, 1.15)	9.76E-01	0.97 (0.95, 0.99)	**8.98E-03**
1.00 (0.85, 1.17)	9.95E-01	0.96 (0.94, 0.98)	**8.91E-04**
1.02 (0.87, 1.19)	9.76E-01	0.97 (0.95, 0.99)	**2.02E-02**
0.97 (0.83, 1.13)	9.42E-01	0.95 (0.93, 0.97)	**6.91E-06**
0.96 (0.82, 1.13)	9.42E-01	0.94 (0.92, 0.97)	**1.67E-06**
0.98 (0.83, 1.14)	9.63E-01	0.96 (0.94, 0.98)	**3.51E-04**
1.19 (1.03, 1.36)	3.01E-01	1.07 (1.04, 1.09)	**9.59E-09**
0.96 (0.82, 1.12)	9.42E-01	0.96 (0.94, 0.98)	**1.52E-04**
0.97 (0.83, 1.14)	9.42E-01	0.94 (0.92, 0.96)	**9.53E-08**
0.96 (0.82, 1.12)	9.42E-01	0.94 (0.92, 0.96)	**3.35E-08**
0.96 (0.82, 1.12)	9.42E-01	0.93 (0.91, 0.95)	**3.15E-09**
0.96 (0.82, 1.12)	9.42E-01	0.93 (0.91, 0.95)	**6.76E-09**
0.95 (0.81, 1.12)	9.42E-01	0.93 (0.91, 0.95)	**7.88E-10**
1.18 (1.02, 1.35)	3.38E-01	1.06 (1.03, 1.08)	**1.35E-06**
0.99 (0.85, 1.16)	9.76E-01	0.96 (0.94, 0.98)	**7.52E-04**
0.96 (0.82, 1.13)	9.42E-01	0.94 (0.92, 0.96)	**3.36E-07**
0.95 (0.81, 1.11)	9.42E-01	0.94 (0.92, 0.96)	**3.19E-08**
0.95 (0.81, 1.11)	9.42E-01	0.94 (0.92, 0.96)	**3.35E-08**
0.96 (0.82, 1.13)	9.42E-01	0.94 (0.92, 0.97)	**1.19E-06**
0.92 (0.79, 1.08)	9.25E-01	0.92 (0.90, 0.94)	**4.21E-12**
1.14 (0.99, 1.31)	6.14E-01	1.04 (1.02, 1.06)	**2.83E-04**
0.97 (0.83, 1.14)	9.63E-01	0.97 (0.95, 0.99)	**6.53E-03**
0.95 (0.81, 1.12)	9.42E-01	0.95 (0.93, 0.97)	**8.64E-06**
0.94 (0.80, 1.10)	9.33E-01	0.95 (0.93, 0.97)	**3.89E-05**
0.95 (0.81, 1.11)	9.42E-01	0.94 (0.92, 0.96)	**1.55E-07**
0.96 (0.82, 1.13)	9.42E-01	0.95 (0.93, 0.97)	**8.37E-06**
0.91 (0.78, 1.07)	8.48E-01	0.92 (0.90, 0.95)	**1.81E-11**
1.09 (0.94, 1.26)	8.50E-01	1.04 (1.01, 1.06)	**2.12E-03**
1.12 (0.96, 1.30)	8.16E-01	1.02 (0.99, 1.04)	1.70E-01
1.10 (0.95, 1.28)	8.16E-01	1.02 (1.00, 1.05)	9.71E-02
1.10 (0.95, 1.29)	8.16E-01	1.03 (1.00, 1.05)	**2.96E-02**
1.08 (0.92, 1.26)	9.25E-01	1.01 (0.98, 1.03)	5.80E-01
1.08 (0.92, 1.26)	9.25E-01	1.00 (0.98, 1.02)	9.95E-01
1.07 (0.92, 1.25)	9.25E-01	1.04 (1.01, 1.06)	**4.00E-03**
1.18 (1.03, 1.35)	3.36E-01	1.06 (1.04, 1.08)	**8.11E-08**
1.11 (0.95, 1.30)	8.16E-01	0.99 (0.96, 1.01)	4.07E-01
1.11 (0.95, 1.30)	8.16E-01	0.99 (0.97, 1.02)	5.69E-01
1.11 (0.95, 1.30)	8.16E-01	0.99 (0.97, 1.02)	7.40E-01
1.09 (0.93, 1.28)	8.80E-01	0.98 (0.96, 1.01)	2.05E-01
1.08 (0.92, 1.27)	9.25E-01	0.98 (0.95, 1.00)	1.15E-01
1.11 (0.95, 1.29)	8.16E-01	1.00 (0.97, 1.02)	8.56E-01
1.21 (1.05, 1.39)	2.40E-01	1.07 (1.04, 1.09)	**3.82E-08**
1.07 (0.91, 1.26)	9.33E-01	0.95 (0.93, 0.97)	**9.83E-05**
1.08 (0.92, 1.27)	9.25E-01	0.96 (0.93, 0.98)	**7.51E-04**
1.08 (0.92, 1.27)	9.25E-01	0.97 (0.94, 0.99)	**9.20E-03**
1.05 (0.89, 1.24)	9.42E-01	0.94 (0.92, 0.96)	**1.38E-06**

1.04 (0.89, 1.23)	9.42E-01	0.94 (0.91, 0.96)	**3.16E-07**
1.08 (0.92, 1.27)	9.25E-01	0.95 (0.93, 0.98)	**4.09E-04**
1.13 (0.98, 1.31)	6.81E-01	1.05 (1.03, 1.07)	**2.02E-05**
0.95 (0.81, 1.11)	9.42E-01	0.91 (0.89, 0.93)	**3.85E-16**
1.00 (0.85, 1.17)	9.98E-01	0.94 (0.92, 0.96)	**3.95E-08**
1.01 (0.86, 1.19)	9.76E-01	0.95 (0.93, 0.97)	**1.33E-05**
0.95 (0.81, 1.12)	9.42E-01	0.90 (0.88, 0.92)	**7.31E-18**
0.93 (0.79, 1.09)	9.25E-01	0.89 (0.87, 0.91)	**3.16E-21**
1.05 (0.90, 1.23)	9.42E-01	0.94 (0.92, 0.96)	**9.08E-07**
1.09 (0.94, 1.27)	8.42E-01	1.04 (1.02, 1.07)	**4.09E-04**
1.28 (1.09, 1.49)	1.25E-01	1.03 (1.01, 1.06)	**1.80E-02**
1.17 (1.02, 1.34)	3.36E-01	1.00 (0.98, 1.02)	9.97E-01
1.16 (1.02, 1.32)	3.69E-01	1.00 (0.97, 1.02)	8.56E-01
1.14 (1.00, 1.30)	6.06E-01	1.00 (0.98, 1.03)	7.18E-01
0.83 (0.72, 0.94)	1.51E-01	0.99 (0.97, 1.01)	5.23E-01
1.12 (0.97, 1.31)	7.82E-01	0.98 (0.95, 1.00)	1.14E-01
1.12 (0.96, 1.30)	8.16E-01	0.97 (0.95, 0.99)	**2.41E-02**
1.12 (0.97, 1.30)	7.82E-01	1.02 (0.99, 1.04)	1.97E-01
0.87 (0.75, 1.02)	6.14E-01	1.01 (0.98, 1.03)	6.80E-01
1.15 (0.94, 1.40)	8.16E-01	1.07 (1.04, 1.10)	**8.64E-07**
1.18 (1.03, 1.35)	3.06E-01	1.02 (1.00, 1.04)	9.02E-02
1.15 (1.00, 1.32)	6.06E-01	1.01 (0.99, 1.03)	4.01E-01
1.23 (1.08, 1.41)	1.25E-01	1.04 (1.02, 1.07)	**1.16E-03**
0.81 (0.69, 0.95)	2.43E-01	0.95 (0.93, 0.97)	**7.14E-05**
1.09 (0.93, 1.28)	8.50E-01	0.98 (0.96, 1.01)	1.87E-01
1.04 (0.89, 1.22)	9.42E-01	0.96 (0.94, 0.98)	**1.05E-03**
1.03 (0.88, 1.21)	9.42E-01	0.96 (0.94, 0.98)	**3.29E-04**
1.06 (0.90, 1.24)	9.42E-01	0.97 (0.95, 1.00)	**2.44E-02**
0.95 (0.81, 1.11)	9.42E-01	1.04 (1.01, 1.06)	**3.80E-03**
0.93 (0.79, 1.09)	9.25E-01	0.92 (0.90, 0.95)	**1.10E-10**
0.99 (0.84, 1.16)	9.76E-01	0.96 (0.94, 0.98)	**3.95E-04**
1.03 (0.88, 1.21)	9.42E-01	0.99 (0.97, 1.01)	3.28E-01
0.94 (0.80, 1.10)	9.33E-01	0.92 (0.90, 0.95)	**1.14E-10**
1.03 (0.88, 1.21)	9.42E-01	1.05 (1.03, 1.08)	**6.91E-06**
1.08 (0.92, 1.27)	9.25E-01	1.13 (1.10, 1.15)	**5.04E-24**
0.90 (0.78, 1.05)	8.16E-01	0.93 (0.91, 0.95)	**3.78E-09**
0.90 (0.77, 1.05)	8.16E-01	0.93 (0.91, 0.95)	**3.80E-10**
0.93 (0.81, 1.08)	9.25E-01	0.98 (0.96, 1.00)	**3.78E-02**
1.10 (0.94, 1.28)	8.16E-01	1.05 (1.03, 1.08)	**1.10E-05**
1.12 (0.96, 1.30)	8.16E-01	1.09 (1.07, 1.12)	**1.29E-14**
0.88 (0.76, 1.01)	6.11E-01	0.90 (0.88, 0.92)	**3.29E-22**
0.87 (0.75, 1.01)	6.08E-01	0.89 (0.87, 0.91)	**9.13E-27**
0.94 (0.81, 1.09)	9.34E-01	0.97 (0.95, 0.99)	**1.27E-02**
1.13 (0.98, 1.30)	7.03E-01	1.10 (1.08, 1.12)	**1.74E-18**
0.94 (0.80, 1.11)	9.42E-01	1.01 (0.98, 1.03)	6.71E-01
0.91 (0.81, 1.02)	7.38E-01	0.93 (0.92, 0.94)	**1.32E-26**
0.91 (0.82, 1.01)	6.14E-01	0.94 (0.93, 0.95)	**7.89E-20**
0.96 (0.83, 1.12)	9.42E-01	0.94 (0.92, 0.96)	**6.98E-09**

1.12 (0.99, 1.27)	6.08E-01	1.09 (1.07, 1.11)	**2.33E-27**
0.90 (0.76, 1.07)	8.16E-01	0.98 (0.96, 1.00)	5.06E-02
0.93 (0.82, 1.05)	8.16E-01	0.93 (0.92, 0.95)	**5.13E-21**
0.96 (0.82, 1.13)	9.42E-01	0.98 (0.96, 1.00)	**4.61E-02**
0.95 (0.81, 1.11)	9.42E-01	0.93 (0.91, 0.95)	**1.67E-09**
1.12 (1.00, 1.25)	6.08E-01	1.09 (1.07, 1.10)	**2.89E-31**
0.95 (0.81, 1.10)	9.42E-01	1.04 (1.02, 1.06)	**1.50E-03**
0.96 (0.83, 1.12)	9.42E-01	0.92 (0.90, 0.93)	**1.05E-23**
1.03 (0.88, 1.20)	9.42E-01	0.97 (0.95, 0.99)	**5.92E-03**
0.94 (0.81, 1.09)	9.27E-01	0.93 (0.91, 0.95)	**1.43E-11**
1.08 (0.95, 1.24)	8.42E-01	1.08 (1.06, 1.10)	**1.89E-14**
1.06 (0.89, 1.26)	9.42E-01	1.04 (1.02, 1.06)	**2.18E-03**
0.90 (0.76, 1.06)	8.16E-01	0.93 (0.91, 0.95)	**3.42E-09**
0.97 (0.83, 1.12)	9.42E-01	0.92 (0.90, 0.93)	**4.40E-18**
0.89 (0.74, 1.07)	8.16E-01	1.01 (0.98, 1.03)	6.13E-01
1.03 (0.90, 1.17)	9.42E-01	1.02 (1.00, 1.04)	**4.35E-02**
0.95 (0.82, 1.11)	9.42E-01	1.04 (1.02, 1.06)	**4.99E-04**
1.00 (0.85, 1.16)	9.93E-01	0.95 (0.93, 0.97)	**5.02E-06**
0.99 (0.85, 1.15)	9.76E-01	0.94 (0.92, 0.96)	**3.95E-08**
1.04 (0.89, 1.23)	9.42E-01	1.01 (0.99, 1.03)	5.48E-01
1.05 (0.91, 1.20)	9.42E-01	1.05 (1.03, 1.07)	**6.77E-06**
0.99 (0.84, 1.17)	9.76E-01	1.10 (1.07, 1.12)	**2.59E-15**
0.94 (0.80, 1.10)	9.42E-01	0.91 (0.89, 0.93)	**5.77E-15**
0.92 (0.79, 1.08)	9.25E-01	0.92 (0.90, 0.94)	**1.29E-14**
1.06 (0.90, 1.26)	9.42E-01	0.94 (0.92, 0.97)	**9.20E-06**
1.09 (0.97, 1.23)	8.16E-01	1.09 (1.06, 1.11)	**5.23E-13**
1.06 (0.90, 1.25)	9.42E-01	1.09 (1.06, 1.11)	**2.84E-12**
0.90 (0.77, 1.05)	8.16E-01	0.90 (0.88, 0.92)	**3.58E-22**
0.87 (0.74, 1.01)	6.11E-01	0.89 (0.87, 0.91)	**3.60E-24**
1.15 (0.99, 1.32)	6.08E-01	1.02 (1.00, 1.04)	1.55E-01
1.11 (0.95, 1.29)	8.16E-01	1.09 (1.07, 1.12)	**2.30E-14**
0.88 (0.75, 1.04)	8.16E-01	0.99 (0.97, 1.01)	5.02E-01
0.97 (0.83, 1.14)	9.63E-01	0.96 (0.94, 0.98)	**1.32E-03**
1.12 (0.96, 1.32)	8.16E-01	1.02 (1.00, 1.05)	9.07E-02
0.99 (0.85, 1.16)	9.76E-01	0.94 (0.92, 0.96)	**1.08E-07**
1.01 (0.86, 1.18)	9.76E-01	0.99 (0.97, 1.02)	6.01E-01
0.95 (0.81, 1.12)	9.42E-01	0.93 (0.91, 0.95)	**5.67E-09**
0.99 (0.85, 1.15)	9.76E-01	0.97 (0.95, 0.99)	**1.01E-02**
0.99 (0.84, 1.16)	9.76E-01	0.93 (0.91, 0.96)	**1.33E-08**
1.00 (0.85, 1.17)	9.93E-01	0.95 (0.93, 0.97)	**2.07E-05**
1.03 (0.88, 1.21)	9.42E-01	1.02 (1.00, 1.05)	**4.30E-02**
1.00 (0.85, 1.17)	9.98E-01	1.02 (0.99, 1.04)	2.03E-01
1.16 (1.00, 1.33)	5.74E-01	1.05 (1.03, 1.07)	**1.33E-05**
1.15 (0.99, 1.33)	6.08E-01	1.05 (1.03, 1.08)	**3.02E-06**
1.07 (0.91, 1.26)	9.27E-01	0.97 (0.95, 0.99)	**1.23E-02**
0.99 (0.84, 1.17)	9.83E-01	1.05 (1.02, 1.07)	**2.21E-04**

1.07 (0.91, 1.26)	9.33E-01	0.96 (0.94, 0.98)	**1.49E-03**
1.07 (0.91, 1.25)	9.33E-01	0.97 (0.95, 0.99)	**1.28E-02**
1.07 (0.91, 1.25)	9.42E-01	0.96 (0.94, 0.98)	**1.74E-03**
1.08 (0.92, 1.27)	9.25E-01	0.95 (0.93, 0.97)	**1.20E-04**
0.98 (0.84, 1.14)	9.76E-01	0.96 (0.94, 0.99)	**2.14E-03**
0.97 (0.83, 1.14)	9.42E-01	0.99 (0.97, 1.02)	6.31E-01
1.06 (0.91, 1.24)	9.42E-01	1.00 (0.98, 1.02)	9.97E-01
0.86 (0.73, 1.01)	6.08E-01	0.89 (0.87, 0.91)	**1.52E-22**
1.01 (0.86, 1.18)	9.76E-01	0.91 (0.89, 0.93)	**2.59E-13**
0.99 (0.85, 1.17)	9.83E-01	0.96 (0.94, 0.98)	**5.12E-04**
0.99 (0.84, 1.16)	9.76E-01	0.93 (0.91, 0.95)	**2.12E-09**
0.99 (0.84, 1.16)	9.76E-01	0.96 (0.93, 0.98)	**1.52E-04**
0.99 (0.84, 1.16)	9.76E-01	0.94 (0.92, 0.96)	**8.14E-07**
1.09 (0.94, 1.27)	8.42E-01	1.03 (1.01, 1.06)	**5.27E-03**
1.08 (0.93, 1.26)	8.91E-01	1.02 (1.00, 1.04)	1.18E-01
0.98 (0.84, 1.15)	9.76E-01	0.91 (0.89, 0.93)	**1.44E-14**
0.91 (0.77, 1.06)	8.16E-01	0.93 (0.91, 0.95)	**5.88E-09**
0.95 (0.81, 1.12)	9.42E-01	0.91 (0.89, 0.93)	**1.29E-13**
0.89 (0.77, 1.05)	8.16E-01	0.94 (0.92, 0.96)	**1.88E-08**
0.88 (0.75, 1.03)	7.45E-01	0.91 (0.89, 0.93)	**8.33E-18**
1.12 (0.95, 1.31)	8.16E-01	1.10 (1.07, 1.12)	**5.78E-15**
1.09 (0.94, 1.28)	8.48E-01	1.07 (1.05, 1.09)	**3.81E-09**
1.03 (0.90, 1.19)	9.42E-01	1.07 (1.05, 1.09)	**3.66E-12**
1.08 (0.93, 1.26)	8.91E-01	1.02 (0.99, 1.04)	1.81E-01
1.03 (0.88, 1.21)	9.42E-01	1.04 (1.02, 1.06)	**9.94E-04**
1.06 (0.91, 1.22)	9.42E-01	1.00 (0.97, 1.02)	7.72E-01
1.11 (0.95, 1.30)	8.16E-01	0.94 (0.92, 0.96)	**1.72E-08**
0.98 (0.83, 1.15)	9.76E-01	0.96 (0.94, 0.98)	**7.33E-04**
1.09 (0.94, 1.26)	8.62E-01	1.00 (0.98, 1.02)	9.72E-01
0.96 (0.82, 1.14)	9.42E-01	0.98 (0.95, 1.00)	**4.41E-02**
0.95 (0.81, 1.12)	9.42E-01	0.99 (0.97, 1.01)	5.20E-01
1.05 (0.95, 1.16)	9.25E-01	1.04 (1.02, 1.06)	**4.45E-05**
0.92 (0.79, 1.08)	9.25E-01	1.04 (1.02, 1.06)	**1.22E-03**
1.14 (1.06, 1.23)	7.02E-02	1.02 (1.00, 1.04)	5.50E-02
1.07 (1.02, 1.14)	2.43E-01	1.04 (1.02, 1.06)	**2.28E-04**
1.14 (1.06, 1.24)	7.02E-02	1.03 (1.01, 1.05)	**8.10E-03**
1.09 (1.03, 1.15)	1.25E-01	1.04 (1.02, 1.05)	**9.18E-06**
0.81 (0.69, 0.95)	2.43E-01	0.91 (0.89, 0.93)	**1.34E-14**
1.14 (0.97, 1.33)	7.43E-01	1.15 (1.12, 1.17)	**9.96E-33**
1.07 (0.92, 1.26)	9.26E-01	0.97 (0.95, 1.00)	**3.67E-02**
0.99 (0.84, 1.16)	9.76E-01	0.99 (0.97, 1.01)	3.72E-01
0.97 (0.83, 1.13)	9.42E-01	0.95 (0.93, 0.97)	**1.48E-05**
0.94 (0.81, 1.10)	9.42E-01	0.93 (0.91, 0.95)	**5.97E-09**
0.96 (0.82, 1.12)	9.42E-01	0.94 (0.92, 0.96)	**3.45E-08**
1.05 (0.89, 1.24)	9.42E-01	0.94 (0.91, 0.96)	**1.50E-06**
0.88 (0.74, 1.03)	7.63E-01	0.90 (0.88, 0.92)	**3.14E-17**
1.06 (0.91, 1.23)	9.42E-01	1.06 (1.05, 1.08)	**6.71E-12**
1.00 (0.86, 1.17)	9.95E-01	1.00 (0.98, 1.02)	9.39E-01

0.94 (0.80, 1.10)	9.42E-01	0.93 (0.91, 0.95)	**9.40E-11**
1.10 (0.94, 1.30)	8.16E-01	0.97 (0.95, 0.99)	**2.68E-02**
1.06 (0.93, 1.21)	9.27E-01	1.02 (1.00, 1.04)	**2.84E-02**
1.10 (1.03, 1.18)	1.51E-01	1.02 (1.00, 1.04)	1.59E-01
1.03 (0.87, 1.21)	9.63E-01	0.92 (0.90, 0.94)	**5.88E-11**
1.04 (0.89, 1.21)	9.42E-01	1.01 (0.99, 1.03)	5.86E-01
0.97 (0.83, 1.13)	9.42E-01	0.96 (0.94, 0.98)	**2.88E-04**
1.03 (0.88, 1.21)	9.42E-01	0.92 (0.90, 0.95)	**2.67E-10**
1.05 (0.90, 1.24)	9.42E-01	0.96 (0.94, 0.98)	**2.11E-03**
1.01 (0.87, 1.19)	9.76E-01	1.01 (0.99, 1.03)	5.48E-01
0.95 (0.81, 1.12)	9.42E-01	0.94 (0.92, 0.96)	**5.21E-08**
1.09 (0.93, 1.29)	8.74E-01	0.97 (0.95, 1.00)	**3.54E-02**
1.02 (0.87, 1.19)	9.76E-01	0.96 (0.94, 0.99)	**2.13E-03**
1.00 (0.86, 1.18)	9.86E-01	1.01 (0.99, 1.03)	5.20E-01
0.97 (0.83, 1.13)	9.42E-01	0.94 (0.92, 0.96)	**1.56E-07**
1.09 (0.93, 1.29)	8.76E-01	0.96 (0.94, 0.99)	**4.90E-03**
1.01 (0.87, 1.18)	9.76E-01	0.99 (0.97, 1.01)	3.94E-01
1.06 (0.90, 1.25)	9.42E-01	0.94 (0.92, 0.97)	**1.90E-05**
1.09 (0.90, 1.32)	9.25E-01	1.05 (1.02, 1.08)	**7.51E-04**

nortality			Other
Current smoker		Never/former smoker	
Adjusted HR (95% CI)	P value	Adjusted HR (95% CI)	P value
0.96 (0.91, 1.01)	1.19E-01	0.94 (0.91, 0.96)	**2.49E-06**
0.96 (0.91, 1.01)	1.17E-01	0.94 (0.91, 0.96)	**1.91E-06**
0.96 (0.92, 1.01)	1.86E-01	0.94 (0.91, 0.96)	**5.72E-06**
0.97 (0.92, 1.01)	2.11E-01	0.93 (0.90, 0.95)	**1.10E-07**
0.97 (0.92, 1.01)	2.23E-01	0.93 (0.90, 0.95)	**3.22E-08**
0.96 (0.92, 1.01)	2.07E-01	0.93 (0.91, 0.96)	**6.95E-07**
0.95 (0.91, 1.00)	8.93E-02	0.94 (0.91, 0.96)	**4.70E-06**
0.92 (0.87, 0.96)	**1.83E-03**	0.89 (0.86, 0.91)	**3.68E-17**
0.92 (0.87, 0.97)	**1.88E-03**	0.89 (0.86, 0.91)	**1.05E-17**
0.92 (0.88, 0.97)	**3.10E-03**	0.89 (0.86, 0.91)	**5.25E-17**
0.91 (0.87, 0.96)	**9.30E-04**	0.86 (0.84, 0.89)	**2.63E-24**
0.91 (0.87, 0.96)	**4.51E-04**	0.85 (0.83, 0.88)	**5.32E-29**
0.92 (0.88, 0.97)	**2.76E-03**	0.88 (0.86, 0.91)	**1.45E-18**
0.92 (0.88, 0.97)	**2.55E-03**	0.90 (0.87, 0.92)	**4.61E-15**
0.90 (0.86, 0.95)	**2.63E-04**	0.87 (0.84, 0.89)	**3.36E-23**
0.90 (0.85, 0.94)	**9.57E-05**	0.86 (0.83, 0.88)	**2.99E-27**
0.91 (0.87, 0.96)	**6.22E-04**	0.87 (0.85, 0.89)	**3.19E-22**
0.90 (0.86, 0.95)	**1.72E-04**	0.85 (0.83, 0.88)	**8.36E-29**
0.91 (0.86, 0.95)	**2.39E-04**	0.85 (0.83, 0.87)	**4.77E-30**
0.90 (0.86, 0.95)	**1.83E-04**	0.86 (0.83, 0.88)	**3.28E-26**
0.89 (0.85, 0.94)	**4.98E-05**	0.86 (0.83, 0.88)	**5.42E-27**
0.89 (0.84, 0.93)	**8.85E-06**	0.84 (0.82, 0.86)	**2.39E-35**
0.88 (0.84, 0.93)	**2.93E-06**	0.83 (0.81, 0.86)	**1.35E-37**
0.89 (0.85, 0.94)	**1.67E-05**	0.84 (0.82, 0.87)	**1.78E-33**
0.90 (0.86, 0.95)	**1.42E-04**	0.86 (0.84, 0.88)	**1.65E-27**
0.92 (0.87, 0.96)	**7.74E-04**	0.88 (0.85, 0.90)	**1.05E-21**
0.89 (0.85, 0.94)	**3.51E-05**	0.85 (0.83, 0.87)	**4.49E-32**
0.89 (0.84, 0.93)	**1.35E-05**	0.85 (0.82, 0.87)	**4.37E-31**
0.91 (0.87, 0.96)	**5.44E-04**	0.88 (0.86, 0.91)	**2.24E-19**
0.91 (0.86, 0.95)	**3.56E-04**	0.87 (0.85, 0.90)	**1.96E-21**
0.90 (0.86, 0.94)	**9.33E-05**	0.86 (0.84, 0.89)	**2.92E-26**
0.91 (0.87, 0.96)	**7.74E-04**	0.87 (0.85, 0.90)	**3.86E-22**
0.92 (0.88, 0.97)	**2.62E-03**	0.88 (0.86, 0.91)	**9.55E-19**
0.90 (0.86, 0.95)	**9.45E-05**	0.86 (0.84, 0.88)	**8.85E-28**
0.92 (0.88, 0.97)	**3.91E-03**	0.91 (0.88, 0.93)	**4.14E-12**
0.97 (0.93, 1.02)	3.48E-01	0.97 (0.94, 0.99)	**2.18E-02**
0.98 (0.93, 1.03)	4.82E-01	0.97 (0.95, 1.00)	6.28E-02
0.99 (0.94, 1.04)	7.13E-01	0.99 (0.97, 1.02)	5.44E-01
0.98 (0.93, 1.02)	4.19E-01	0.96 (0.93, 0.99)	**2.94E-03**
0.98 (0.93, 1.02)	3.91E-01	0.95 (0.93, 0.98)	**9.70E-04**

0.98 (0.94, 1.03)	5.09E-01	0.97 (0.95, 1.00)	**3.89E-02**
0.98 (0.94, 1.03)	5.55E-01	1.00 (0.97, 1.02)	8.90E-01
0.92 (0.88, 0.97)	**2.18E-03**	0.90 (0.88, 0.93)	**1.57E-13**
0.92 (0.88, 0.97)	**2.62E-03**	0.91 (0.89, 0.94)	**5.62E-11**
0.94 (0.89, 0.98)	**1.74E-02**	0.92 (0.90, 0.95)	**1.39E-08**
0.92 (0.87, 0.96)	**8.92E-04**	0.90 (0.88, 0.93)	**1.94E-13**
0.91 (0.87, 0.96)	**4.78E-04**	0.90 (0.87, 0.92)	**7.94E-15**
0.93 (0.89, 0.98)	**6.53E-03**	0.92 (0.90, 0.95)	**1.83E-09**
1.00 (0.96, 1.05)	9.42E-01	1.03 (1.00, 1.05)	**4.36E-02**
0.89 (0.85, 0.94)	**2.15E-05**	0.86 (0.84, 0.89)	**5.02E-26**
0.88 (0.84, 0.92)	**2.26E-06**	0.86 (0.83, 0.88)	**8.09E-29**
0.89 (0.84, 0.93)	**6.07E-06**	0.86 (0.83, 0.88)	**1.93E-29**
0.88 (0.83, 0.92)	**7.71E-07**	0.85 (0.83, 0.87)	**1.72E-31**
0.87 (0.83, 0.92)	**4.40E-07**	0.84 (0.82, 0.87)	**1.04E-33**
0.89 (0.85, 0.93)	**9.24E-06**	0.87 (0.85, 0.89)	**2.23E-24**
0.98 (0.94, 1.03)	5.91E-01	1.02 (0.99, 1.04)	2.16E-01
0.89 (0.84, 0.93)	**7.07E-06**	0.85 (0.83, 0.87)	**1.41E-32**
0.86 (0.82, 0.91)	**8.09E-08**	0.83 (0.81, 0.85)	**9.49E-42**
0.86 (0.82, 0.90)	**2.27E-08**	0.83 (0.80, 0.85)	**2.04E-42**
0.86 (0.82, 0.91)	**8.09E-08**	0.82 (0.80, 0.85)	**1.41E-44**
0.87 (0.83, 0.91)	**1.63E-07**	0.82 (0.80, 0.85)	**1.64E-44**
0.86 (0.82, 0.91)	**8.09E-08**	0.84 (0.81, 0.86)	**3.63E-39**
0.95 (0.91, 1.00)	8.24E-02	0.98 (0.95, 1.00)	1.17E-01
0.88 (0.84, 0.93)	**4.58E-06**	0.86 (0.84, 0.89)	**3.83E-26**
0.87 (0.83, 0.91)	**1.71E-07**	0.85 (0.82, 0.87)	**1.75E-34**
0.88 (0.83, 0.92)	**5.33E-07**	0.87 (0.84, 0.89)	**1.97E-26**
0.87 (0.83, 0.91)	**1.03E-07**	0.83 (0.81, 0.86)	**3.16E-39**
0.87 (0.83, 0.91)	**2.35E-07**	0.84 (0.81, 0.86)	**2.02E-38**
0.87 (0.83, 0.91)	**1.89E-07**	0.85 (0.83, 0.87)	**1.24E-33**
0.94 (0.89, 0.99)	**2.41E-02**	0.95 (0.93, 0.97)	**1.61E-04**
1.06 (1.01, 1.11)	**3.98E-02**	1.16 (1.13, 1.20)	**2.34E-27**
1.08 (1.03, 1.13)	**3.39E-03**	1.18 (1.15, 1.22)	**1.79E-33**
1.09 (1.04, 1.14)	**7.51E-04**	1.20 (1.17, 1.23)	**3.93E-38**
1.06 (1.02, 1.12)	**1.74E-02**	1.16 (1.13, 1.20)	**7.22E-27**
1.06 (1.01, 1.11)	**2.98E-02**	1.16 (1.12, 1.19)	**3.87E-24**
1.08 (1.03, 1.13)	**3.04E-03**	1.18 (1.15, 1.21)	**3.28E-35**
0.99 (0.94, 1.04)	6.45E-01	1.04 (1.01, 1.07)	**2.42E-03**
1.07 (1.02, 1.12)	**1.77E-02**	1.15 (1.11, 1.18)	**2.06E-20**
1.08 (1.02, 1.13)	**7.68E-03**	1.16 (1.12, 1.19)	**2.19E-22**
1.08 (1.03, 1.14)	**4.40E-03**	1.16 (1.12, 1.19)	**9.38E-23**
1.07 (1.02, 1.12)	**1.50E-02**	1.15 (1.12, 1.18)	**1.37E-20**
1.07 (1.02, 1.12)	**1.77E-02**	1.14 (1.11, 1.18)	**1.88E-19**
1.07 (1.02, 1.13)	**8.36E-03**	1.16 (1.13, 1.19)	**2.88E-24**
1.03 (0.98, 1.08)	2.71E-01	1.08 (1.06, 1.11)	**2.36E-09**
1.02 (0.97, 1.07)	5.09E-01	1.00 (0.98, 1.03)	8.28E-01
1.02 (0.97, 1.08)	4.32E-01	1.00 (0.97, 1.03)	9.11E-01
1.03 (0.98, 1.08)	3.75E-01	1.00 (0.97, 1.03)	9.57E-01
1.02 (0.97, 1.07)	4.70E-01	1.01 (0.98, 1.04)	7.03E-01

1.02 (0.97, 1.07)	4.55E-01	1.00 (0.97, 1.03)	8.39E-01
1.02 (0.97, 1.07)	5.40E-01	1.02 (0.99, 1.05)	2.70E-01
1.00 (0.95, 1.05)	9.48E-01	0.98 (0.96, 1.01)	1.92E-01
0.90 (0.86, 0.94)	**7.37E-05**	0.82 (0.80, 0.84)	**3.29E-45**
0.93 (0.89, 0.98)	**8.36E-03**	0.86 (0.83, 0.88)	**5.07E-29**
0.96 (0.91, 1.00)	9.47E-02	0.88 (0.85, 0.90)	**1.11E-20**
0.91 (0.86, 0.95)	**2.19E-04**	0.83 (0.81, 0.85)	**8.85E-41**
0.90 (0.86, 0.95)	**1.79E-04**	0.82 (0.80, 0.84)	**1.47E-46**
0.92 (0.88, 0.97)	**2.42E-03**	0.89 (0.86, 0.91)	**4.00E-18**
0.95 (0.91, 1.00)	8.71E-02	0.93 (0.91, 0.96)	**1.44E-06**
1.08 (1.02, 1.14)	**7.67E-03**	1.01 (0.98, 1.05)	4.02E-01
1.03 (0.98, 1.08)	3.58E-01	1.04 (1.01, 1.07)	**1.53E-02**
1.02 (0.97, 1.07)	5.36E-01	1.03 (1.00, 1.06)	**3.58E-02**
1.04 (0.99, 1.09)	1.70E-01	1.04 (1.01, 1.06)	**1.50E-02**
0.96 (0.92, 1.01)	1.21E-01	0.97 (0.94, 0.99)	**1.51E-02**
1.02 (0.97, 1.08)	4.31E-01	1.06 (1.03, 1.09)	**4.21E-05**
1.02 (0.97, 1.07)	5.91E-01	1.05 (1.02, 1.08)	**1.43E-03**
1.04 (1.00, 1.10)	1.09E-01	1.10 (1.07, 1.13)	**6.03E-11**
0.98 (0.93, 1.03)	4.19E-01	0.95 (0.92, 0.97)	**1.37E-04**
1.03 (0.97, 1.08)	4.27E-01	0.96 (0.93, 0.98)	**2.45E-03**
1.06 (1.01, 1.11)	**3.35E-02**	1.07 (1.05, 1.10)	**2.04E-07**
1.05 (1.01, 1.11)	**4.45E-02**	1.08 (1.05, 1.11)	**8.49E-09**
1.05 (1.00, 1.10)	6.77E-02	1.03 (1.01, 1.06)	**2.54E-02**
0.94 (0.90, 0.99)	**3.60E-02**	0.97 (0.94, 1.00)	**2.73E-02**
1.01 (0.96, 1.06)	7.01E-01	1.01 (0.98, 1.03)	5.65E-01
1.01 (0.96, 1.06)	7.00E-01	1.02 (1.00, 1.05)	8.12E-02
1.01 (0.96, 1.06)	7.30E-01	1.03 (1.00, 1.05)	6.28E-02
1.01 (0.97, 1.06)	5.96E-01	1.02 (0.99, 1.04)	1.74E-01
0.99 (0.94, 1.04)	6.99E-01	0.98 (0.95, 1.01)	1.29E-01
0.95 (0.91, 1.00)	6.79E-02	0.95 (0.92, 0.98)	**2.08E-04**
0.99 (0.95, 1.04)	7.55E-01	0.98 (0.95, 1.00)	9.61E-02
1.02 (0.97, 1.07)	4.99E-01	1.00 (0.97, 1.03)	9.96E-01
0.96 (0.91, 1.00)	9.95E-02	0.95 (0.92, 0.98)	**2.05E-04**
1.02 (0.97, 1.07)	4.71E-01	1.03 (1.01, 1.06)	**2.12E-02**
1.08 (1.03, 1.14)	**2.49E-03**	1.14 (1.11, 1.17)	**2.51E-22**
0.98 (0.94, 1.03)	5.26E-01	0.96 (0.93, 0.98)	**1.10E-03**
0.98 (0.94, 1.03)	4.91E-01	0.96 (0.93, 0.98)	**6.53E-04**
1.00 (0.95, 1.04)	8.72E-01	0.98 (0.96, 1.00)	1.17E-01
1.00 (0.96, 1.05)	8.95E-01	1.02 (1.00, 1.05)	1.12E-01
1.14 (1.09, 1.19)	**4.40E-07**	1.11 (1.08, 1.14)	**3.53E-16**
0.92 (0.88, 0.96)	**7.74E-04**	0.90 (0.88, 0.92)	**2.25E-20**
0.90 (0.86, 0.94)	**1.33E-05**	0.87 (0.85, 0.89)	**2.05E-29**
1.01 (0.96, 1.06)	7.45E-01	0.99 (0.97, 1.02)	6.83E-01
1.06 (1.01, 1.10)	**2.68E-02**	1.10 (1.07, 1.12)	**5.30E-16**
1.05 (1.01, 1.10)	**3.64E-02**	1.03 (1.01, 1.05)	**2.01E-02**
0.92 (0.89, 0.95)	**7.07E-06**	0.92 (0.91, 0.93)	**3.21E-42**
0.91 (0.88, 0.94)	**1.74E-09**	0.92 (0.91, 0.93)	**7.47E-70**
0.99 (0.95, 1.04)	7.43E-01	0.98 (0.96, 1.01)	1.64E-01

1.08 (1.04, 1.12)	**6.22E-04**	1.11 (1.09, 1.12)	**8.14E-42**
0.98 (0.94, 1.03)	5.91E-01	1.04 (1.01, 1.07)	**2.42E-03**
0.94 (0.91, 0.97)	**2.06E-03**	0.91 (0.90, 0.92)	**1.85E-55**
0.94 (0.90, 0.98)	**8.90E-03**	0.87 (0.86, 0.89)	**3.53E-51**
0.99 (0.94, 1.04)	7.48E-01	0.99 (0.97, 1.02)	6.89E-01
1.08 (1.04, 1.12)	**3.58E-04**	1.11 (1.09, 1.12)	**1.61E-59**
1.08 (1.03, 1.13)	**1.59E-03**	1.14 (1.12, 1.16)	**7.17E-34**
0.91 (0.88, 0.95)	**1.67E-04**	0.89 (0.88, 0.90)	**3.57E-61**
0.94 (0.89, 0.98)	**8.86E-03**	0.88 (0.86, 0.90)	**6.37E-33**
0.97 (0.93, 1.02)	2.83E-01	0.95 (0.93, 0.98)	**1.73E-04**
1.03 (0.98, 1.08)	2.66E-01	1.07 (1.05, 1.09)	**1.35E-09**
1.17 (1.10, 1.24)	**7.33E-07**	1.17 (1.13, 1.20)	**1.04E-23**
0.90 (0.86, 0.95)	**1.52E-04**	0.86 (0.84, 0.88)	**9.17E-28**
0.95 (0.91, 1.00)	**4.90E-02**	0.92 (0.90, 0.94)	**2.60E-14**
0.91 (0.87, 0.96)	**1.83E-03**	0.90 (0.88, 0.93)	**3.27E-12**
0.91 (0.86, 0.96)	**2.06E-03**	0.96 (0.93, 0.98)	**1.34E-03**
0.99 (0.95, 1.04)	7.11E-01	0.97 (0.94, 0.99)	**7.25E-03**
1.05 (1.00, 1.10)	8.00E-02	1.04 (1.01, 1.06)	**7.37E-03**
1.04 (0.99, 1.09)	1.75E-01	1.02 (1.00, 1.05)	1.00E-01
1.07 (1.02, 1.13)	**8.27E-03**	1.08 (1.05, 1.11)	**5.48E-09**
0.93 (0.88, 0.98)	**8.90E-03**	0.97 (0.95, 1.00)	**4.22E-02**
1.03 (0.98, 1.08)	2.54E-01	1.01 (0.99, 1.04)	3.85E-01
0.99 (0.95, 1.04)	7.60E-01	1.00 (0.97, 1.03)	9.66E-01
0.99 (0.95, 1.04)	8.25E-01	0.99 (0.97, 1.02)	6.61E-01
0.99 (0.94, 1.04)	6.35E-01	1.03 (1.00, 1.06)	8.72E-02
0.99 (0.94, 1.04)	7.45E-01	0.99 (0.97, 1.02)	6.04E-01
1.16 (1.10, 1.21)	**8.09E-08**	1.16 (1.13, 1.20)	**1.33E-28**
0.92 (0.88, 0.96)	**9.86E-04**	0.91 (0.89, 0.93)	**9.50E-14**
0.92 (0.88, 0.97)	**2.44E-03**	0.88 (0.86, 0.91)	**4.73E-22**
0.97 (0.93, 1.02)	3.36E-01	1.11 (1.08, 1.14)	**2.55E-15**
0.99 (0.94, 1.04)	7.54E-01	1.01 (0.98, 1.04)	5.33E-01
0.89 (0.85, 0.94)	**2.68E-05**	0.84 (0.82, 0.86)	**1.84E-36**
1.01 (0.97, 1.06)	6.08E-01	0.99 (0.96, 1.02)	4.61E-01
1.11 (1.06, 1.17)	**9.45E-05**	1.22 (1.19, 1.26)	**1.41E-43**
0.90 (0.86, 0.95)	**1.24E-04**	0.88 (0.86, 0.90)	**1.19E-20**
0.92 (0.87, 0.96)	**1.22E-03**	0.87 (0.85, 0.90)	**2.42E-22**
0.87 (0.83, 0.91)	**2.35E-07**	0.84 (0.82, 0.86)	**2.31E-36**
0.91 (0.87, 0.95)	**3.45E-04**	0.88 (0.86, 0.90)	**3.89E-21**
0.90 (0.86, 0.95)	**1.42E-04**	0.88 (0.86, 0.90)	**3.43E-20**
0.90 (0.86, 0.95)	**1.24E-04**	0.88 (0.85, 0.90)	**3.98E-21**
0.93 (0.88, 0.97)	**5.19E-03**	0.91 (0.88, 0.93)	**4.07E-12**
0.92 (0.87, 0.96)	**1.38E-03**	0.89 (0.86, 0.91)	**6.55E-17**
0.97 (0.92, 1.02)	2.83E-01	1.00 (0.97, 1.02)	8.96E-01
0.99 (0.94, 1.04)	7.13E-01	0.99 (0.97, 1.02)	5.40E-01
0.95 (0.91, 1.00)	1.01E-01	0.94 (0.91, 0.96)	**4.08E-06**
0.93 (0.89, 0.98)	**1.21E-02**	0.92 (0.90, 0.95)	**2.97E-09**

0.95 (0.90, 1.00)	5.54E-02	0.93 (0.91, 0.96)	**9.30E-07**
0.98 (0.93, 1.03)	4.27E-01	0.97 (0.94, 0.99)	**1.75E-02**
0.95 (0.91, 1.00)	8.11E-02	0.93 (0.90, 0.96)	**4.64E-07**
1.01 (0.96, 1.06)	7.15E-01	1.00 (0.98, 1.03)	8.28E-01
0.89 (0.85, 0.94)	**2.68E-05**	0.87 (0.84, 0.89)	**7.54E-26**
0.91 (0.87, 0.96)	**7.08E-04**	0.89 (0.86, 0.91)	**9.60E-18**
0.93 (0.88, 0.97)	**5.57E-03**	0.91 (0.89, 0.94)	**5.70E-11**
0.87 (0.83, 0.91)	**1.63E-07**	0.86 (0.84, 0.89)	**2.36E-27**
0.85 (0.81, 0.90)	**8.09E-08**	0.87 (0.84, 0.89)	**2.19E-21**
0.87 (0.83, 0.92)	**8.90E-07**	0.86 (0.84, 0.89)	**1.26E-25**
0.83 (0.79, 0.88)	**7.23E-10**	0.84 (0.82, 0.87)	**3.26E-30**
0.88 (0.84, 0.93)	**6.52E-06**	0.86 (0.84, 0.89)	**2.35E-26**
0.86 (0.82, 0.90)	**6.59E-08**	0.84 (0.82, 0.86)	**1.98E-34**
0.97 (0.92, 1.01)	2.34E-01	0.96 (0.94, 0.99)	**3.27E-03**
0.96 (0.91, 1.01)	1.39E-01	0.95 (0.92, 0.97)	**1.14E-04**
0.90 (0.85, 0.94)	**1.12E-04**	0.92 (0.89, 0.94)	**1.35E-09**
0.91 (0.86, 0.95)	**1.72E-04**	0.92 (0.90, 0.94)	**3.98E-10**
0.84 (0.80, 0.89)	**2.39E-09**	0.86 (0.83, 0.88)	**6.20E-25**
0.97 (0.92, 1.01)	2.07E-01	0.97 (0.95, 1.00)	**2.51E-02**
0.91 (0.87, 0.96)	**5.49E-04**	0.92 (0.90, 0.94)	**1.48E-10**
1.06 (1.01, 1.11)	**3.98E-02**	1.06 (1.03, 1.09)	**4.28E-05**
1.10 (1.05, 1.15)	**2.38E-04**	1.09 (1.06, 1.12)	**2.22E-11**
1.03 (0.99, 1.08)	1.74E-01	1.12 (1.10, 1.14)	**2.39E-40**
1.05 (1.01, 1.10)	**3.95E-02**	1.05 (1.03, 1.08)	**1.32E-04**
1.06 (1.01, 1.11)	**2.98E-02**	1.09 (1.07, 1.11)	**7.23E-18**
0.92 (0.87, 0.96)	**8.84E-04**	1.01 (0.98, 1.03)	6.13E-01
0.97 (0.92, 1.01)	2.30E-01	0.95 (0.93, 0.98)	**2.15E-04**
0.93 (0.88, 0.97)	**4.55E-03**	0.89 (0.86, 0.92)	**7.19E-15**
0.97 (0.92, 1.02)	2.64E-01	0.94 (0.91, 0.96)	**4.43E-06**
0.94 (0.89, 0.98)	**1.74E-02**	0.87 (0.84, 0.89)	**6.30E-22**
0.89 (0.85, 0.94)	**3.70E-05**	0.87 (0.84, 0.89)	**1.16E-22**
1.01 (0.97, 1.06)	6.35E-01	1.05 (1.03, 1.07)	**9.97E-08**
0.95 (0.91, 1.00)	6.25E-02	1.04 (1.02, 1.07)	**1.33E-03**
1.10 (1.07, 1.13)	**2.27E-09**	1.07 (1.06, 1.08)	**6.65E-51**
1.06 (1.04, 1.09)	**3.69E-07**	1.09 (1.07, 1.11)	**2.90E-19**
1.11 (1.08, 1.15)	**5.69E-11**	1.09 (1.07, 1.10)	**6.79E-25**
0.94 (0.89, 1.00)	6.45E-02	1.06 (1.05, 1.07)	**8.86E-31**
0.85 (0.81, 0.90)	**2.66E-09**	0.80 (0.78, 0.83)	**5.24E-59**
1.09 (1.04, 1.15)	**6.90E-04**	1.10 (1.07, 1.13)	**9.11E-13**
1.01 (0.96, 1.06)	7.86E-01	1.03 (1.00, 1.07)	**3.44E-02**
0.92 (0.87, 0.97)	**2.06E-03**	0.88 (0.85, 0.90)	**4.17E-19**
0.89 (0.85, 0.93)	**7.29E-06**	0.86 (0.83, 0.88)	**2.14E-29**
0.88 (0.84, 0.92)	**2.10E-06**	0.85 (0.83, 0.87)	**2.51E-32**
0.87 (0.83, 0.91)	**1.89E-07**	0.84 (0.81, 0.86)	**3.60E-38**
1.03 (0.97, 1.08)	4.14E-01	1.04 (1.01, 1.07)	**1.09E-02**
0.92 (0.87, 0.96)	**1.51E-03**	0.93 (0.91, 0.96)	**1.91E-06**
1.11 (1.07, 1.16)	**5.77E-07**	1.10 (1.09, 1.12)	**7.14E-48**
0.91 (0.87, 0.96)	**6.10E-04**	0.87 (0.85, 0.89)	**1.67E-23**

0.88 (0.84, 0.92)	**1.27E-06**	0.86 (0.83, 0.88)	**2.08E-29**
1.03 (0.98, 1.08)	3.43E-01	1.07 (1.04, 1.10)	**5.28E-06**
1.04 (0.99, 1.08)	1.71E-01	1.06 (1.04, 1.07)	**7.71E-13**
1.09 (1.06, 1.12)	**7.23E-10**	1.08 (1.07, 1.10)	**3.53E-51**
0.95 (0.90, 1.00)	6.54E-02	0.91 (0.88, 0.94)	**7.30E-11**
0.92 (0.87, 0.96)	**1.22E-03**	0.89 (0.86, 0.91)	**7.51E-18**
0.89 (0.85, 0.93)	**7.95E-06**	0.86 (0.84, 0.88)	**6.91E-29**
0.97 (0.92, 1.02)	2.42E-01	0.93 (0.90, 0.96)	**5.67E-07**
0.94 (0.89, 0.99)	**1.96E-02**	0.92 (0.89, 0.94)	**4.23E-10**
0.92 (0.87, 0.96)	**1.14E-03**	0.88 (0.86, 0.90)	**4.97E-20**
0.87 (0.83, 0.92)	**4.67E-07**	0.85 (0.82, 0.87)	**1.17E-33**
1.04 (0.99, 1.09)	1.83E-01	1.05 (1.02, 1.08)	**1.99E-03**
0.90 (0.86, 0.95)	**1.54E-04**	0.88 (0.86, 0.90)	**5.95E-21**
0.91 (0.87, 0.96)	**6.68E-04**	0.88 (0.85, 0.90)	**4.24E-21**
0.87 (0.83, 0.92)	**4.63E-07**	0.85 (0.82, 0.87)	**1.77E-33**
1.03 (0.98, 1.09)	2.83E-01	1.05 (1.02, 1.08)	**2.52E-03**
0.92 (0.88, 0.97)	**2.76E-03**	0.88 (0.86, 0.90)	**2.03E-20**
1.03 (0.98, 1.08)	3.92E-01	1.05 (1.02, 1.08)	**2.52E-03**
1.01 (0.96, 1.06)	8.53E-01	0.99 (0.96, 1.02)	6.46E-01

mortality	
**Current smoker**	
**Adjusted HR (95% CI)**	**P value**
0.88 (0.83, 0.93)	**1.63E-05**
0.88 (0.83, 0.93)	**1.95E-05**
0.88 (0.83, 0.93)	**2.25E-05**
0.86 (0.81, 0.91)	**7.22E-07**
0.85 (0.80, 0.90)	**9.62E-08**
0.87 (0.83, 0.93)	**9.00E-06**
0.89 (0.84, 0.94)	**5.97E-05**
0.79 (0.75, 0.84)	**9.28E-14**
0.79 (0.75, 0.84)	**9.28E-14**
0.80 (0.75, 0.85)	**3.52E-13**
0.77 (0.72, 0.81)	**9.15E-18**
0.75 (0.71, 0.80)	**7.87E-21**
0.79 (0.75, 0.84)	**5.38E-14**
0.80 (0.76, 0.85)	**4.18E-12**
0.76 (0.72, 0.81)	**1.13E-17**
0.75 (0.71, 0.80)	**5.96E-20**
0.77 (0.72, 0.82)	**4.03E-17**
0.75 (0.70, 0.79)	**4.33E-21**
0.75 (0.71, 0.79)	**6.09E-21**
0.75 (0.71, 0.80)	**3.84E-20**
0.75 (0.70, 0.80)	**1.86E-19**
0.74 (0.70, 0.79)	**5.18E-22**
0.73 (0.69, 0.78)	**3.22E-24**
0.76 (0.71, 0.80)	**2.44E-20**
0.80 (0.75, 0.84)	**1.41E-14**
0.83 (0.79, 0.88)	**3.08E-10**
0.76 (0.72, 0.81)	**4.02E-19**
0.73 (0.69, 0.78)	**1.33E-22**
0.79 (0.74, 0.83)	**3.98E-15**
0.78 (0.74, 0.83)	**7.42E-16**
0.77 (0.73, 0.82)	**8.88E-18**
0.79 (0.75, 0.84)	**1.36E-14**
0.80 (0.76, 0.85)	**4.63E-13**
0.78 (0.73, 0.82)	**5.82E-17**
0.82 (0.77, 0.87)	**8.99E-11**
0.97 (0.92, 1.02)	2.84E-01
0.98 (0.93, 1.04)	6.11E-01
1.00 (0.95, 1.06)	9.37E-01
0.98 (0.93, 1.04)	5.86E-01
0.99 (0.93, 1.04)	6.38E-01

0.98 (0.93, 1.03)	4.84E-01
0.97 (0.92, 1.03)	3.62E-01
0.87 (0.83, 0.92)	**3.87E-06**
0.91 (0.86, 0.96)	**1.31E-03**
0.93 (0.88, 0.98)	**9.32E-03**
0.90 (0.85, 0.95)	**2.18E-04**
0.89 (0.84, 0.94)	**1.01E-04**
0.92 (0.87, 0.97)	**2.09E-03**
1.03 (0.98, 1.09)	2.45E-01
0.80 (0.76, 0.85)	**2.19E-13**
0.79 (0.75, 0.84)	**3.26E-15**
0.79 (0.75, 0.84)	**1.33E-15**
0.78 (0.74, 0.83)	**2.13E-16**
0.77 (0.73, 0.82)	**6.18E-18**
0.82 (0.78, 0.87)	**7.82E-12**
1.01 (0.96, 1.06)	8.06E-01
0.74 (0.70, 0.78)	**1.10E-23**
0.72 (0.68, 0.76)	**1.02E-27**
0.72 (0.68, 0.76)	**9.90E-28**
0.72 (0.68, 0.76)	**2.73E-29**
0.71 (0.67, 0.75)	**5.40E-30**
0.75 (0.71, 0.79)	**1.68E-23**
0.93 (0.88, 0.98)	**1.56E-02**
0.77 (0.73, 0.82)	**3.36E-18**
0.75 (0.71, 0.80)	**5.18E-22**
0.79 (0.75, 0.84)	**7.20E-16**
0.74 (0.69, 0.78)	**4.66E-25**
0.73 (0.69, 0.78)	**6.18E-25**
0.77 (0.73, 0.81)	**4.81E-20**
0.89 (0.84, 0.94)	**7.57E-05**
1.32 (1.27, 1.38)	**3.75E-33**
1.35 (1.30, 1.42)	**1.08E-40**
1.38 (1.32, 1.44)	**3.48E-45**
1.33 (1.27, 1.39)	**7.75E-34**
1.32 (1.26, 1.38)	**1.99E-31**
1.34 (1.28, 1.39)	**3.94E-40**
1.06 (1.01, 1.12)	**2.71E-02**
1.33 (1.27, 1.40)	**1.56E-28**
1.34 (1.28, 1.41)	**1.53E-30**
1.35 (1.28, 1.42)	**7.14E-31**
1.33 (1.26, 1.39)	**1.47E-28**
1.32 (1.26, 1.39)	**3.51E-27**
1.35 (1.29, 1.42)	**7.06E-33**
1.16 (1.10, 1.22)	**1.87E-08**
1.11 (1.05, 1.17)	**4.89E-04**
1.11 (1.05, 1.17)	**6.08E-04**
1.10 (1.04, 1.17)	**8.03E-04**
1.12 (1.05, 1.18)	**2.03E-04**

1.11 (1.05, 1.17)	**4.12E-04**
1.14 (1.07, 1.20)	**1.34E-05**
0.98 (0.93, 1.04)	5.39E-01
0.75 (0.71, 0.79)	**2.97E-23**
0.81 (0.77, 0.86)	**1.46E-12**
0.86 (0.81, 0.91)	**3.82E-07**
0.77 (0.72, 0.81)	**8.98E-20**
0.75 (0.71, 0.79)	**1.71E-23**
0.86 (0.81, 0.91)	**2.24E-07**
0.86 (0.81, 0.91)	**5.96E-07**
1.04 (0.98, 1.11)	2.00E-01
1.05 (1.00, 1.11)	8.21E-02
1.03 (0.97, 1.09)	4.11E-01
1.09 (1.04, 1.15)	**1.04E-03**
0.95 (0.90, 1.00)	5.25E-02
1.13 (1.08, 1.20)	**5.26E-06**
1.11 (1.05, 1.17)	**2.60E-04**
1.19 (1.13, 1.25)	**4.93E-12**
0.89 (0.84, 0.93)	**1.68E-05**
0.92 (0.88, 0.97)	**4.51E-03**
1.14 (1.08, 1.20)	**8.37E-07**
1.17 (1.11, 1.23)	**5.42E-10**
1.01 (0.96, 1.07)	7.18E-01
0.93 (0.88, 0.98)	**1.63E-02**
1.06 (1.01, 1.12)	**3.24E-02**
1.08 (1.03, 1.14)	**5.59E-03**
1.09 (1.03, 1.15)	**2.84E-03**
1.06 (1.01, 1.12)	**3.54E-02**
0.93 (0.88, 0.98)	**7.55E-03**
0.94 (0.89, 0.99)	**2.59E-02**
0.99 (0.94, 1.04)	6.93E-01
1.02 (0.96, 1.07)	5.77E-01
0.95 (0.90, 1.00)	8.56E-02
1.03 (0.97, 1.08)	3.58E-01
1.16 (1.10, 1.23)	**1.08E-07**
0.99 (0.94, 1.05)	8.31E-01
1.00 (0.95, 1.05)	9.54E-01
0.97 (0.92, 1.02)	2.44E-01
0.98 (0.93, 1.03)	4.32E-01
1.15 (1.09, 1.22)	**1.82E-07**
0.88 (0.84, 0.92)	**3.33E-07**
0.86 (0.82, 0.90)	**1.35E-09**
0.98 (0.93, 1.03)	4.56E-01
1.11 (1.06, 1.16)	**2.22E-05**
1.06 (1.01, 1.11)	**3.36E-02**
0.89 (0.87, 0.91)	**1.72E-23**
0.88 (0.87, 0.90)	**3.14E-42**
1.00 (0.95, 1.05)	9.49E-01

1.15 (1.12, 1.19)	**2.98E-21**
1.09 (1.06, 1.14)	**2.17E-06**
0.89 (0.87, 0.90)	**3.75E-33**
0.83 (0.81, 0.86)	**1.74E-45**
1.02 (0.97, 1.08)	5.13E-01
1.15 (1.13, 1.18)	**1.81E-31**
1.32 (1.25, 1.38)	**2.72E-28**
0.80 (0.77, 0.82)	**5.89E-44**
0.77 (0.74, 0.81)	**2.32E-25**
0.94 (0.89, 0.98)	**1.05E-02**
1.08 (1.04, 1.13)	**1.15E-04**
1.53 (1.42, 1.66)	**5.49E-26**
0.76 (0.73, 0.80)	**1.42E-25**
0.88 (0.84, 0.92)	**1.69E-08**
0.76 (0.71, 0.81)	**3.99E-15**
0.87 (0.81, 0.93)	**3.82E-05**
0.94 (0.89, 0.98)	**8.01E-03**
1.13 (1.07, 1.19)	**1.95E-05**
1.10 (1.04, 1.16)	**9.96E-04**
1.23 (1.16, 1.30)	**5.58E-11**
0.86 (0.81, 0.92)	**3.99E-06**
0.99 (0.94, 1.04)	7.20E-01
1.04 (0.99, 1.10)	1.78E-01
1.02 (0.96, 1.07)	5.51E-01
1.13 (1.07, 1.20)	**6.27E-05**
0.94 (0.89, 1.00)	**4.89E-02**
1.38 (1.31, 1.46)	**9.86E-32**
0.84 (0.80, 0.89)	**2.26E-10**
0.81 (0.77, 0.85)	**1.03E-15**
1.18 (1.12, 1.24)	**3.95E-11**
0.95 (0.90, 1.01)	1.09E-01
0.73 (0.69, 0.77)	**1.30E-28**
1.11 (1.05, 1.17)	**4.39E-04**
1.44 (1.37, 1.51)	**6.93E-48**
0.85 (0.80, 0.90)	**2.64E-08**
0.80 (0.75, 0.85)	**7.49E-14**
0.76 (0.72, 0.80)	**7.23E-21**
0.83 (0.79, 0.88)	**2.38E-10**
0.85 (0.81, 0.90)	**6.49E-08**
0.84 (0.80, 0.89)	**5.21E-09**
0.83 (0.78, 0.88)	**1.20E-09**
0.80 (0.75, 0.85)	**6.04E-13**
0.97 (0.92, 1.03)	3.33E-01
0.98 (0.93, 1.04)	5.96E-01
0.97 (0.91, 1.02)	2.67E-01
0.82 (0.78, 0.87)	**1.16E-10**

0.98 (0.92, 1.03)	4.41E-01
1.00 (0.94, 1.05)	9.07E-01
0.96 (0.91, 1.02)	2.34E-01
1.11 (1.05, 1.17)	**5.18E-04**
0.79 (0.75, 0.84)	**5.18E-15**
0.80 (0.75, 0.84)	**5.22E-14**
0.87 (0.82, 0.92)	**1.49E-06**
0.83 (0.79, 0.88)	**1.22E-10**
0.86 (0.81, 0.92)	**3.86E-06**
0.80 (0.76, 0.85)	**1.43E-13**
0.79 (0.74, 0.84)	**2.19E-13**
0.80 (0.75, 0.85)	**5.67E-14**
0.78 (0.73, 0.82)	**7.62E-17**
0.91 (0.86, 0.96)	**1.04E-03**
0.92 (0.87, 0.98)	**7.03E-03**
0.94 (0.89, 1.00)	5.25E-02
0.91 (0.86, 0.96)	**5.08E-04**
0.81 (0.76, 0.86)	**4.01E-11**
0.98 (0.93, 1.03)	4.44E-01
0.92 (0.87, 0.97)	**2.43E-03**
0.99 (0.94, 1.05)	7.94E-01
1.18 (1.12, 1.24)	**9.40E-10**
1.04 (0.99, 1.08)	1.55E-01
1.06 (1.01, 1.12)	**3.88E-02**
1.10 (1.04, 1.16)	**1.06E-03**
0.86 (0.82, 0.91)	**3.49E-07**
0.94 (0.89, 0.99)	**3.27E-02**
0.81 (0.76, 0.85)	**3.33E-13**
0.87 (0.82, 0.92)	**3.92E-06**
0.76 (0.71, 0.81)	**2.92E-17**
0.73 (0.69, 0.78)	**4.70E-23**
1.01 (0.95, 1.06)	8.22E-01
0.99 (0.94, 1.04)	7.41E-01
1.16 (1.14, 1.18)	**1.84E-47**
1.07 (1.04, 1.09)	**1.74E-08**
1.13 (1.10, 1.16)	**1.11E-17**
0.87 (0.81, 0.92)	**2.87E-05**
0.76 (0.72, 0.80)	**5.64E-23**
1.03 (0.98, 1.09)	2.64E-01
0.98 (0.92, 1.04)	4.79E-01
0.76 (0.71, 0.81)	**7.33E-18**
0.79 (0.75, 0.84)	**9.20E-16**
0.78 (0.74, 0.82)	**1.67E-17**
0.75 (0.71, 0.79)	**2.26E-22**
1.17 (1.11, 1.24)	**2.79E-08**
0.97 (0.92, 1.02)	3.11E-01
1.16 (1.12, 1.21)	**2.78E-14**
0.78 (0.74, 0.83)	**1.21E-15**

0.79 (0.75, 0.84)	**1.16E-15**
1.23 (1.16, 1.29)	**3.90E-13**
1.04 (0.99, 1.09)	1.56E-01
1.11 (1.09, 1.14)	**2.86E-22**
0.94 (0.88, 0.99)	**3.33E-02**
0.81 (0.77, 0.86)	**3.85E-12**
0.78 (0.74, 0.82)	**3.81E-17**
0.97 (0.92, 1.03)	3.88E-01
0.93 (0.88, 0.99)	**1.87E-02**
0.80 (0.75, 0.85)	**8.08E-14**
0.77 (0.72, 0.81)	**1.59E-19**
1.19 (1.13, 1.26)	**1.39E-09**
0.84 (0.79, 0.89)	**2.28E-09**
0.79 (0.74, 0.84)	**8.41E-15**
0.77 (0.72, 0.81)	**1.63E-19**
1.19 (1.12, 1.26)	**2.14E-09**
0.82 (0.77, 0.86)	**7.13E-12**
1.19 (1.12, 1.26)	**2.11E-09**
1.00 (0.94, 1.06)	9.00E-01

Supplementary Table 12. Association of all metaboli

		CKB	
		All causes	
		Never/former drinker	
**Metabolites**	**Group**	**Adjusted HR (95% CI)**	**P value**
Concentration of chylomicro	Lipoprotein subclasses	0.93 (0.88, 0.98)	**1.08E-02**
Total lipids in chylomicrons a	Lipoprotein subclasses	0.93 (0.88, 0.98)	**1.41E-02**
Phospholipids in chylomicro	Lipoprotein subclasses	0.93 (0.89, 0.98)	**2.57E-02**
Cholesterol in chylomicrons a	Lipoprotein subclasses	0.96 (0.92, 1.01)	2.40E-01
Cholesteryl esters in chylomi	Lipoprotein subclasses	0.98 (0.93, 1.03)	5.07E-01
Free cholesterol in chylomicr	Lipoprotein subclasses	0.94 (0.90, 0.99)	6.45E-02
Triglycerides in chylomicron	Lipoprotein subclasses	0.92 (0.87, 0.97)	**4.80E-03**
Concentration of very large V	Lipoprotein subclasses	0.95 (0.90, 1.00)	7.23E-02
Total lipids in very large VLI	Lipoprotein subclasses	0.95 (0.90, 1.00)	8.20E-02
Phospholipids in very large V	Lipoprotein subclasses	0.95 (0.90, 1.00)	1.01E-01
Cholesterol in very large VLI	Lipoprotein subclasses	0.97 (0.92, 1.02)	2.70E-01
Cholesteryl esters in very larg	Lipoprotein subclasses	0.97 (0.92, 1.02)	3.40E-01
Free cholesterol in very large	Lipoprotein subclasses	0.96 (0.91, 1.01)	1.94E-01
Triglycerides in very large V	Lipoprotein subclasses	0.94 (0.90, 0.99)	5.36E-02
Concentration of large VLDI	Lipoprotein subclasses	0.95 (0.90, 0.99)	6.46E-02
Total lipids in large VLDL	Lipoprotein subclasses	0.95 (0.90, 1.00)	6.46E-02
Phospholipids in large VLDI	Lipoprotein subclasses	0.95 (0.90, 1.00)	6.46E-02
Cholesterol in large VLDL	Lipoprotein subclasses	0.95 (0.90, 1.00)	6.69E-02
Cholesteryl esters in large VI	Lipoprotein subclasses	0.95 (0.90, 0.99)	6.10E-02
Free cholesterol in large VLI	Lipoprotein subclasses	0.95 (0.90, 1.00)	8.19E-02
Triglycerides in large VLDL	Lipoprotein subclasses	0.95 (0.90, 0.99)	6.46E-02
Concentration of medium VI	Lipoprotein subclasses	0.95 (0.91, 1.00)	8.85E-02
Total lipids in medium VLDI	Lipoprotein subclasses	0.95 (0.91, 1.00)	9.22E-02
Phospholipids in medium VL	Lipoprotein subclasses	0.95 (0.90, 1.00)	7.50E-02
Cholesterol in medium VLDI	Lipoprotein subclasses	0.96 (0.91, 1.01)	1.57E-01
Cholesteryl esters in medium	Lipoprotein subclasses	0.96 (0.92, 1.01)	1.84E-01
Free cholesterol in medium V	Lipoprotein subclasses	0.96 (0.91, 1.01)	1.51E-01
Triglycerides in medium VLI	Lipoprotein subclasses	0.95 (0.90, 1.00)	8.24E-02
Concentration of small VLDI	Lipoprotein subclasses	0.97 (0.92, 1.02)	3.24E-01
Total lipids in small VLDL	Lipoprotein subclasses	0.98 (0.93, 1.03)	4.63E-01
Phospholipids in small VLDI	Lipoprotein subclasses	0.96 (0.91, 1.01)	1.36E-01
Cholesterol in small VLDL	Lipoprotein subclasses	1.04 (1.00, 1.09)	1.28E-01
Cholesteryl esters in small VI	Lipoprotein subclasses	1.07 (1.02, 1.12)	**1.83E-02**
Free cholesterol in small VLI	Lipoprotein subclasses	1.00 (0.95, 1.05)	9.82E-01
Triglycerides in small VLDL	Lipoprotein subclasses	0.95 (0.90, 1.00)	6.52E-02
Concentration of very small V	Lipoprotein subclasses	1.04 (0.99, 1.09)	1.87E-01
Total lipids in very small VL	Lipoprotein subclasses	1.05 (1.00, 1.10)	9.19E-02
Phospholipids in very small V	Lipoprotein subclasses	1.02 (0.97, 1.07)	6.33E-01
Cholesterol in very small VL	Lipoprotein subclasses	1.09 (1.04, 1.14)	**5.56E-04**
Cholesteryl esters in very sma	Lipoprotein subclasses	1.09 (1.04, 1.14)	**4.82E-04**

Free cholesterol in very smal	Lipoprotein subclasses	1.08 (1.04, 1.13)	**1.76E-03**
Triglycerides in very small V	Lipoprotein subclasses	0.97 (0.93, 1.02)	3.90E-01
Concentration of IDL particl	Lipoprotein subclasses	1.00 (0.95, 1.05)	9.83E-01
Total lipids in IDL	Lipoprotein subclasses	1.00 (0.96, 1.05)	9.13E-01
Phospholipids in IDL	Lipoprotein subclasses	0.99 (0.95, 1.04)	8.37E-01
Cholesterol in IDL	Lipoprotein subclasses	1.00 (0.96, 1.05)	8.83E-01
Cholesteryl esters in IDL	Lipoprotein subclasses	0.99 (0.95, 1.04)	8.20E-01
Free cholesterol in IDL	Lipoprotein subclasses	1.03 (0.99, 1.08)	2.58E-01
Triglycerides in IDL	Lipoprotein subclasses	1.01 (0.96, 1.06)	7.57E-01
Concentration of large LDL	Lipoprotein subclasses	0.99 (0.94, 1.04)	7.05E-01
Total lipids in large LDL	Lipoprotein subclasses	0.99 (0.94, 1.04)	7.24E-01
Phospholipids in large LDL	Lipoprotein subclasses	0.97 (0.92, 1.02)	2.56E-01
Cholesterol in large LDL	Lipoprotein subclasses	0.99 (0.94, 1.04)	7.71E-01
Cholesteryl esters in large LI	Lipoprotein subclasses	0.99 (0.94, 1.03)	6.48E-01
Free cholesterol in large LDI	Lipoprotein subclasses	1.01 (0.96, 1.06)	8.59E-01
Triglycerides in large LDL	Lipoprotein subclasses	1.03 (0.98, 1.08)	3.34E-01
Concentration of medium LD	Lipoprotein subclasses	0.97 (0.92, 1.02)	3.60E-01
Total lipids in medium LDL	Lipoprotein subclasses	0.97 (0.92, 1.02)	3.56E-01
Phospholipids in medium LD	Lipoprotein subclasses	0.91 (0.87, 0.96)	**2.20E-03**
Cholesterol in medium LDL	Lipoprotein subclasses	0.98 (0.94, 1.03)	6.46E-01
Cholesteryl esters in medium	Lipoprotein subclasses	0.99 (0.94, 1.04)	7.71E-01
Free cholesterol in medium L	Lipoprotein subclasses	0.96 (0.91, 1.01)	1.56E-01
Triglycerides in medium LDI	Lipoprotein subclasses	1.02 (0.97, 1.07)	5.38E-01
Concentration of small LDL	Lipoprotein subclasses	0.97 (0.92, 1.02)	3.45E-01
Total lipids in small LDL	Lipoprotein subclasses	0.97 (0.92, 1.02)	3.57E-01
Phospholipids in small LDL	Lipoprotein subclasses	0.91 (0.86, 0.96)	**6.34E-04**
Cholesterol in small LDL	Lipoprotein subclasses	0.99 (0.94, 1.04)	8.12E-01
Cholesteryl esters in small LI	Lipoprotein subclasses	1.00 (0.95, 1.05)	9.29E-01
Free cholesterol in small LDI	Lipoprotein subclasses	0.96 (0.91, 1.01)	1.56E-01
Triglycerides in small LDL	Lipoprotein subclasses	0.99 (0.95, 1.04)	8.45E-01
Concentration of very large H	Lipoprotein subclasses	1.16 (1.11, 1.22)	**1.46E-10**
Total lipids in very large HD	Lipoprotein subclasses	1.16 (1.11, 1.22)	**1.57E-10**
Phospholipids in very large H	Lipoprotein subclasses	1.15 (1.10, 1.20)	**5.72E-09**
Cholesterol in very large HD	Lipoprotein subclasses	1.17 (1.12, 1.22)	**1.03E-10**
Cholesteryl esters in very lar	Lipoprotein subclasses	1.17 (1.12, 1.23)	**2.71E-11**
Free cholesterol in very large	Lipoprotein subclasses	1.15 (1.10, 1.20)	**4.85E-09**
Triglycerides in very large H	Lipoprotein subclasses	1.11 (1.06, 1.16)	**5.49E-05**
Concentration of large HDL	Lipoprotein subclasses	1.09 (1.04, 1.15)	**6.72E-04**
Total lipids in large HDL	Lipoprotein subclasses	1.10 (1.04, 1.15)	**5.56E-04**
Phospholipids in large HDL	Lipoprotein subclasses	1.08 (1.03, 1.14)	**3.01E-03**
Cholesterol in large HDL	Lipoprotein subclasses	1.10 (1.05, 1.16)	**1.75E-04**
Cholesteryl esters in large HI	Lipoprotein subclasses	1.10 (1.05, 1.15)	**4.18E-04**
Free cholesterol in large HDI	Lipoprotein subclasses	1.12 (1.07, 1.17)	**8.60E-06**
Triglycerides in large HDL	Lipoprotein subclasses	1.07 (1.02, 1.12)	**1.83E-02**
**Concentration of medium H**	Lipoprotein subclasses	0.83 (0.79, 0.87)	**4.52E-12**
**Total lipids in medium HDI**	Lipoprotein subclasses	0.84 (0.80, 0.88)	**1.71E-11**
**Phospholipids in medium H**	Lipoprotein subclasses	0.85 (0.81, 0.89)	**1.77E-09**
**Cholesterol in medium HDI**	Lipoprotein subclasses	0.85 (0.81, 0.89)	**6.37E-10**

**Cholesteryl esters in mediu**	Lipoprotein subclasses	0.85 (0.81, 0.89)	**1.49E-10**
**Free cholesterol in medium**	Lipoprotein subclasses	0.87 (0.83, 0.92)	**3.90E-07**
Triglycerides in medium HD	Lipoprotein subclasses	0.84 (0.80, 0.88)	**4.89E-10**
Concentration of small HDL	Lipoprotein subclasses	0.79 (0.76, 0.83)	**4.47E-25**
Total lipids in small HDL	Lipoprotein subclasses	0.80 (0.76, 0.83)	**1.33E-24**
Phospholipids in small HDL	Lipoprotein subclasses	0.81 (0.77, 0.84)	**1.85E-18**
Cholesterol in small HDL	Lipoprotein subclasses	0.89 (0.85, 0.94)	**1.23E-05**
Cholesteryl esters in small H	Lipoprotein subclasses	0.93 (0.88, 0.97)	**5.43E-03**
**Free cholesterol in small H**	Lipoprotein subclasses	0.83 (0.79, 0.86)	**7.28E-15**
Triglycerides in small HDL	Lipoprotein subclasses	0.88 (0.84, 0.93)	**3.46E-06**
Phospholipids to total lipids r	Relative lipoprotein lip	0.93 (0.89, 0.98)	**1.36E-02**
Cholesterol to total lipids rati	Relative lipoprotein lip	1.16 (1.11, 1.20)	**3.94E-12**
Cholesteryl esters to total lipi	Relative lipoprotein lip	1.16 (1.11, 1.20)	**1.46E-12**
Free cholesterol to total lipid	Relative lipoprotein lip	1.05 (1.00, 1.11)	1.23E-01
Triglycerides to total lipids ra	Relative lipoprotein lip	0.88 (0.84, 0.93)	**1.99E-06**
Phospholipids to total lipids r	Relative lipoprotein lip	0.93 (0.88, 0.98)	**1.07E-02**
**Cholesterol to total lipids ra**	Relative lipoprotein lip	1.10 (1.06, 1.14)	**1.24E-06**
Cholesteryl esters to total lipi	Relative lipoprotein lip	1.12 (1.08, 1.16)	**9.82E-09**
**Free cholesterol to total lipi**	Relative lipoprotein lip	1.00 (0.95, 1.05)	9.02E-01
**Triglycerides to total lipids**	Relative lipoprotein lip	0.94 (0.89, 0.99)	**4.47E-02**
Phospholipids to total lipids r	Relative lipoprotein lip	0.93 (0.89, 0.98)	**1.72E-02**
Cholesterol to total lipids rati	Relative lipoprotein lip	0.97 (0.92, 1.02)	2.79E-01
Cholesteryl esters to total lipi	Relative lipoprotein lip	1.02 (0.98, 1.08)	4.34E-01
Free cholesterol to total lipid	Relative lipoprotein lip	0.94 (0.90, 0.99)	**3.33E-02**
Triglycerides to total lipids ra	Relative lipoprotein lip	1.05 (1.00, 1.10)	1.27E-01
Phospholipids to total lipids r	Relative lipoprotein lip	0.95 (0.90, 1.00)	6.91E-02
**Cholesterol to total lipids ra**	Relative lipoprotein lip	1.03 (0.98, 1.08)	3.21E-01
**Cholesteryl esters to total li**	Relative lipoprotein lip	1.03 (0.98, 1.09)	2.80E-01
Free cholesterol to total lipid	Relative lipoprotein lip	0.99 (0.94, 1.04)	8.13E-01
Triglycerides to total lipids ra	Relative lipoprotein lip	0.98 (0.93, 1.03)	5.38E-01
**Phospholipids to total lipids**	Relative lipoprotein lip	0.90 (0.86, 0.95)	**2.99E-04**
Cholesterol to total lipids rati	Relative lipoprotein lip	1.14 (1.09, 1.20)	**3.29E-07**
Cholesteryl esters to total lipi	Relative lipoprotein lip	1.13 (1.08, 1.19)	**1.99E-06**
**Free cholesterol to total lipi**	Relative lipoprotein lip	1.15 (1.09, 1.20)	**9.03E-08**
Triglycerides to total lipids ra	Relative lipoprotein lip	0.89 (0.85, 0.94)	**2.67E-05**
Phospholipids to total lipids r	Relative lipoprotein lip	0.94 (0.90, 0.99)	**3.33E-02**
Cholesterol to total lipids rati	Relative lipoprotein lip	1.17 (1.11, 1.23)	**5.72E-09**
Cholesteryl esters to total lipi	Relative lipoprotein lip	1.14 (1.08, 1.20)	**1.08E-06**
Free cholesterol to total lipid	Relative lipoprotein lip	1.16 (1.10, 1.23)	**1.34E-07**
Triglycerides to total lipids ra	Relative lipoprotein lip	0.91 (0.86, 0.95)	**5.36E-04**
Phospholipids to total lipids r	Relative lipoprotein lip	0.95 (0.91, 1.00)	8.44E-02
Cholesterol to total lipids rati	Relative lipoprotein lip	1.02 (0.97, 1.07)	4.93E-01
Cholesteryl esters to total lipi	Relative lipoprotein lip	0.94 (0.90, 0.99)	**2.87E-02**
Free cholesterol to total lipid	Relative lipoprotein lip	1.11 (1.05, 1.17)	**3.54E-04**
Triglycerides to total lipids ra	Relative lipoprotein lip	1.00 (0.95, 1.04)	9.02E-01
Phospholipids to total lipids r	Relative lipoprotein lip	0.94 (0.89, 0.99)	**4.00E-02**
Cholesterol to total lipids rati	Relative lipoprotein lip	1.01 (0.96, 1.06)	7.51E-01
Cholesteryl esters to total lipi	Relative lipoprotein lip	0.98 (0.93, 1.02)	4.33E-01

Free cholesterol to total lipid	Relative lipoprotein lip	1.10 (1.05, 1.16)	**6.46E-04**
Triglycerides to total lipids ra	Relative lipoprotein lip	1.03 (0.98, 1.08)	3.24E-01
Phospholipids to total lipids r	Relative lipoprotein lip	0.93 (0.88, 0.98)	**2.07E-02**
Cholesterol to total lipids rati	Relative lipoprotein lip	1.03 (0.98, 1.08)	3.16E-01
Cholesteryl esters to total lipi	Relative lipoprotein lip	1.02 (0.97, 1.07)	4.93E-01
Free cholesterol to total lipid	Relative lipoprotein lip	1.01 (0.96, 1.06)	8.13E-01
Triglycerides to total lipids ra	Relative lipoprotein lip	1.05 (1.00, 1.10)	1.07E-01
Phospholipids to total lipids r	Relative lipoprotein lip	0.94 (0.89, 0.99)	**3.13E-02**
Cholesterol to total lipids rati	Relative lipoprotein lip	1.04 (0.99, 1.09)	1.87E-01
Cholesteryl esters to total lipi	Relative lipoprotein lip	1.03 (0.98, 1.09)	2.76E-01
Free cholesterol to total lipid	Relative lipoprotein lip	1.00 (0.95, 1.05)	9.92E-01
Triglycerides to total lipids ra	Relative lipoprotein lip	1.01 (0.97, 1.06)	6.55E-01
Phospholipids to total lipids r	Relative lipoprotein lip	0.99 (0.94, 1.03)	6.55E-01
Cholesterol to total lipids rati	Relative lipoprotein lip	1.04 (0.99, 1.09)	1.52E-01
Cholesteryl esters to total lipi	Relative lipoprotein lip	1.04 (0.99, 1.10)	1.49E-01
Free cholesterol to total lipid	Relative lipoprotein lip	1.02 (0.98, 1.07)	4.65E-01
Triglycerides to total lipids ra	Relative lipoprotein lip	0.94 (0.89, 0.99)	**2.72E-02**
Phospholipids to total lipids r	Relative lipoprotein lip	0.89 (0.84, 0.93)	**1.60E-05**
Cholesterol to total lipids rati	Relative lipoprotein lip	1.12 (1.07, 1.18)	**2.91E-05**
Cholesteryl esters to total lipi	Relative lipoprotein lip	1.07 (1.02, 1.12)	**2.64E-02**
Free cholesterol to total lipid	Relative lipoprotein lip	1.18 (1.12, 1.24)	**4.06E-09**
Triglycerides to total lipids ra	Relative lipoprotein lip	0.97 (0.93, 1.02)	3.75E-01
Phospholipids to total lipids r	Relative lipoprotein lip	1.15 (1.11, 1.19)	**1.71E-11**
Cholesterol to total lipids rati	Relative lipoprotein lip	0.94 (0.90, 0.99)	**3.70E-02**
Cholesteryl esters to total lipi	Relative lipoprotein lip	0.95 (0.90, 0.99)	**4.52E-02**
Free cholesterol to total lipid	Relative lipoprotein lip	0.96 (0.92, 1.01)	1.36E-01
Triglycerides to total lipids ra	Relative lipoprotein lip	0.95 (0.91, 1.00)	8.85E-02
Phospholipids to total lipids r	Relative lipoprotein lip	0.94 (0.90, 0.99)	**4.00E-02**
Cholesterol to total lipids rati	Relative lipoprotein lip	1.06 (1.01, 1.12)	**3.49E-02**
Cholesteryl esters to total lipi	Relative lipoprotein lip	1.06 (1.01, 1.11)	6.46E-02
Free cholesterol to total lipid	Relative lipoprotein lip	1.01 (0.96, 1.06)	7.63E-01
Triglycerides to total lipids ra	Relative lipoprotein lip	0.97 (0.92, 1.01)	2.36E-01
Average diameter for VLDL	Lipoprotein particle siz	0.91 (0.87, 0.96)	**6.46E-04**
Average diameter for LDL pa	Lipoprotein particle siz	1.07 (1.02, 1.13)	**1.66E-02**
Average diameter for HDL p	Lipoprotein particle siz	1.17 (1.12, 1.22)	**5.40E-10**
Total cholesterol	Cholesterol	1.01 (0.97, 1.06)	6.66E-01
VLDL cholesterol	Cholesterol	1.01 (0.96, 1.06)	8.37E-01
Remnant cholesterol (non-HI	Cholesterol	1.01 (0.96, 1.06)	8.32E-01
LDL cholesterol	Cholesterol	1.00 (0.95, 1.05)	8.83E-01
**HDL cholesterol**	Cholesterol	1.04 (0.99, 1.09)	1.84E-01
**HDL2 cholesterol**	Cholesterol	1.02 (0.98, 1.07)	4.15E-01
HDL3 cholesterol	Cholesterol	1.14 (1.09, 1.19)	**9.66E-08**
Total esterified cholesterol	Cholesteryl esters	0.88 (0.84, 0.93)	**8.78E-06**
Total free cholesterol	Free cholesterol	0.96 (0.91, 1.01)	1.56E-01
Total triglycerides	Triglycerides	0.95 (0.91, 1.00)	9.97E-02
Triglycerides in VLDL	Triglycerides	0.95 (0.90, 1.00)	7.05E-02
Triglycerides in LDL	Triglycerides	1.02 (0.97, 1.07)	4.97E-01
Triglycerides in HDL	Triglycerides	0.94 (0.90, 0.99)	**4.47E-02**

Phosphoglycerides	Other lipids	1.01 (0.96, 1.05)	8.75E-01
Ratio of triglycerides to phos	Other lipids	0.97 (0.92, 1.02)	2.93E-01
Phosphatidylcholines	Other lipids	0.90 (0.86, 0.95)	**2.83E-04**
Sphingomyelins	Other lipids	0.88 (0.83, 0.93)	**6.07E-06**
Total cholines	Other lipids	0.99 (0.95, 1.04)	8.32E-01
Apolipoprotein A1	Apolipoproteins	1.03 (0.98, 1.08)	2.96E-01
Apolipoprotein B	Apolipoproteins	0.98 (0.93, 1.03)	4.57E-01
Ratio of apolipoprotein B to	Apolipoproteins	0.96 (0.91, 1.01)	1.84E-01
Total fatty acids	Fatty acids	0.95 (0.90, 0.99)	6.25E-02
Degree of unsaturation	Fatty acids	0.84 (0.80, 0.88)	**8.86E-11**
Docosahexaenoic acid	Fatty acids	1.12 (1.07, 1.17)	**3.95E-06**
Linoleic acid	Fatty acids	0.96 (0.91, 1.01)	1.46E-01
Omega-3 fatty acids	Fatty acids	1.02 (0.97, 1.07)	5.13E-01
Omega-6 fatty acids	Fatty acids	0.92 (0.87, 0.97)	**4.80E-03**
Polyunsaturated fatty acids	Fatty acids	0.93 (0.89, 0.98)	**2.62E-02**
Monounsaturated fatty acids	Fatty acids	0.95 (0.90, 1.00)	8.19E-02
Saturated fatty acids	Fatty acids	0.96 (0.91, 1.01)	1.44E-01
Ratio of docosahexaenoic aci	Fatty acids	1.21 (1.15, 1.27)	**3.70E-13**
Ratio of linoleic acid to total	Fatty acids	1.01 (0.96, 1.06)	7.45E-01
Ratio of omega-3 fatty acids	Fatty acids	1.08 (1.03, 1.14)	**3.34E-03**
Ratio of omega-6 fatty acids	Fatty acids	0.95 (0.90, 1.00)	6.46E-02
Ratio of polyunsaturated fatty	Fatty acids	0.97 (0.92, 1.02)	3.36E-01
Ratio of monounsaturated fat	Fatty acids	0.98 (0.93, 1.03)	4.93E-01
Ratio of saturated fatty acids	Fatty acids	1.08 (1.03, 1.14)	**8.15E-03**
Glucose	Glycolysis related meta	1.12 (1.08, 1.17)	**4.06E-09**
Lactate	Glycolysis related meta	0.96 (0.91, 1.00)	1.28E-01
Citrate	Glycolysis related meta	0.99 (0.94, 1.04)	7.04E-01
Alanine	Amino acids	0.94 (0.89, 0.98)	**2.03E-02**
Glutamine	Amino acids	0.93 (0.88, 0.98)	**1.05E-02**
Histidine	Amino acids	1.00 (0.95, 1.05)	9.29E-01
Isoleucine	Amino acids	0.92 (0.87, 0.96)	**2.39E-03**
Leucine	Amino acids	0.95 (0.90, 1.00)	6.91E-02
Valine	Amino acids	0.82 (0.78, 0.87)	**1.58E-11**
Phenylalanine	Amino acids	1.16 (1.12, 1.20)	**5.88E-17**
Tyrosine	Amino acids	1.05 (1.00, 1.11)	6.58E-02
Acetate	Ketone bodies	0.99 (0.94, 1.04)	7.81E-01
Acetoacetate	Ketone bodies	0.93 (0.88, 0.98)	**1.19E-02**
**3-Hydroxybutyrate**	Ketone bodies	0.98 (0.93, 1.03)	4.93E-01
Creatinine	Fluid balance	1.05 (1.02, 1.08)	**4.31E-03**
Albumin	Fluid balance	0.82 (0.78, 0.86)	**5.17E-16**
Glycoprotein acetyls	Inflammation	1.16 (1.11, 1.22)	**2.71E-09**
Acetone	Ketone bodies		
Cholesteryl esters in HDL	Cholesteryl esters		
Cholesteryl esters in LDL	Cholesteryl esters		
Cholesteryl esters in VLDL/	Cholesteryl esters		
Clinical LDL cholesterol	Cholesterol		
Concentration of HDL partic	Lipoprotein particle concentrations		
Concentration of LDL particl	Lipoprotein particle concentrations		

Concentration of VLDL parti	Lipoprotein particle concentrations
Free cholesterol in HDL	Free cholesterol
Free cholesterol in LDL	Free cholesterol
Free cholesterol in VLDL	Free cholesterol
Glycine	Amino acids
Phospholipids in HDL	Phospholipids
Phospholipids in LDL	Phospholipids
Phospholipids in VLDL	Phospholipids
Pyruvate	Glycolysis related metabolites
Ratio of omega-6 fatty acids	Fatty acids
Ratio of polyunsaturated fatty	Fatty acids
Total cholesterol minus HDL	Cholesterol
Total concentration of branch	Amino acids
Total concentration of lipopr	Lipoprotein particle concentrations
Total lipids in HDL	Total lipids
Total lipids in LDL	Total lipids
Total lipids in lipoprotein par	Total lipids
Total lipids in VLDL	Total lipids
Total phospholipids in lipopr	Phospholipids

Models were adjusted for age, sex, regions (CKB only), ethnicity (UKB only), levels of education, s

HR, hazard ratio; CI, confidence interval; LDL, Low Density Lipoprotein; HDL, High Density Lipo

ites with all-cause and cause-specific mortality in the CKB and UKB by alcohol consumption sta

mortality		Ischaemic heart diseases mortality		
Current drinker		Never/former drinker		Current drink
Adjusted HR (95% CI)	P value	Adjusted HR (95% CI)	P value	djusted HR (95% CI
0.99 (0.89, 1.10)	9.53E-01	0.96 (0.87, 1.05)	4.90E-01	1.13 (0.93, 1.37)
0.99 (0.89, 1.10)	9.53E-01	0.96 (0.88, 1.05)	5.19E-01	1.13 (0.93, 1.38)
1.00 (0.90, 1.12)	9.72E-01	0.97 (0.89, 1.06)	6.65E-01	1.15 (0.95, 1.39)
1.01 (0.91, 1.13)	9.53E-01	0.99 (0.91, 1.08)	9.23E-01	1.15 (0.95, 1.39)
1.02 (0.91, 1.13)	9.53E-01	1.01 (0.93, 1.11)	8.34E-01	1.14 (0.93, 1.38)
1.02 (0.91, 1.13)	9.53E-01	0.96 (0.88, 1.05)	5.26E-01	1.16 (0.96, 1.40)
0.98 (0.88, 1.09)	9.52E-01	0.95 (0.87, 1.04)	4.21E-01	1.13 (0.93, 1.37)
1.02 (0.91, 1.13)	9.53E-01	0.96 (0.87, 1.05)	4.90E-01	1.15 (0.95, 1.39)
1.02 (0.91, 1.13)	9.53E-01	0.96 (0.88, 1.05)	5.26E-01	1.15 (0.95, 1.39)
1.02 (0.92, 1.13)	9.48E-01	0.97 (0.88, 1.06)	6.31E-01	1.15 (0.95, 1.39)
1.02 (0.92, 1.14)	9.26E-01	0.99 (0.90, 1.08)	8.76E-01	1.15 (0.95, 1.40)
1.02 (0.92, 1.13)	9.53E-01	0.99 (0.91, 1.09)	9.34E-01	1.15 (0.94, 1.39)
1.02 (0.92, 1.14)	9.12E-01	0.98 (0.90, 1.07)	7.51E-01	1.15 (0.95, 1.40)
1.02 (0.91, 1.13)	9.53E-01	0.95 (0.87, 1.04)	4.21E-01	1.15 (0.94, 1.39)
1.01 (0.91, 1.12)	9.55E-01	0.98 (0.89, 1.07)	7.22E-01	1.16 (0.96, 1.41)
1.01 (0.91, 1.12)	9.54E-01	0.98 (0.89, 1.07)	7.19E-01	1.16 (0.96, 1.41)
1.01 (0.91, 1.12)	9.53E-01	0.97 (0.89, 1.06)	6.99E-01	1.16 (0.96, 1.40)
1.01 (0.91, 1.12)	9.61E-01	0.97 (0.89, 1.06)	6.53E-01	1.15 (0.94, 1.39)
0.99 (0.89, 1.10)	9.61E-01	0.97 (0.88, 1.06)	6.17E-01	1.12 (0.92, 1.36)
1.02 (0.92, 1.13)	9.52E-01	0.97 (0.89, 1.06)	6.96E-01	1.17 (0.97, 1.41)
1.01 (0.91, 1.12)	9.53E-01	0.98 (0.89, 1.07)	7.47E-01	1.17 (0.97, 1.42)
0.99 (0.89, 1.09)	9.53E-01	1.00 (0.91, 1.09)	9.54E-01	1.17 (0.97, 1.43)
0.99 (0.89, 1.10)	9.53E-01	1.00 (0.91, 1.09)	9.54E-01	1.17 (0.96, 1.43)
0.98 (0.89, 1.09)	9.53E-01	0.99 (0.91, 1.08)	9.11E-01	1.17 (0.96, 1.42)
0.99 (0.89, 1.10)	9.53E-01	0.99 (0.91, 1.09)	9.30E-01	1.14 (0.94, 1.40)
0.98 (0.88, 1.08)	9.20E-01	0.99 (0.91, 1.08)	9.11E-01	1.10 (0.90, 1.34)
1.00 (0.90, 1.11)	9.90E-01	1.00 (0.91, 1.09)	9.54E-01	1.19 (0.98, 1.44)
0.99 (0.89, 1.10)	9.53E-01	1.00 (0.91, 1.09)	9.71E-01	1.18 (0.98, 1.44)
0.97 (0.87, 1.08)	8.43E-01	1.03 (0.94, 1.13)	6.37E-01	1.13 (0.92, 1.38)
0.97 (0.88, 1.08)	8.69E-01	1.05 (0.96, 1.14)	4.64E-01	1.13 (0.92, 1.38)
0.97 (0.88, 1.08)	8.73E-01	1.02 (0.93, 1.11)	8.28E-01	1.11 (0.91, 1.36)
0.98 (0.89, 1.09)	9.53E-01	1.18 (1.08, 1.28)	**6.36E-04**	1.11 (0.90, 1.36)
0.98 (0.88, 1.08)	8.96E-01	1.22 (1.12, 1.32)	**1.83E-05**	1.07 (0.87, 1.31)
1.00 (0.90, 1.10)	9.75E-01	1.08 (0.99, 1.18)	1.39E-01	1.15 (0.94, 1.41)
0.97 (0.87, 1.08)	8.25E-01	0.97 (0.89, 1.06)	6.85E-01	1.13 (0.92, 1.38)
1.01 (0.92, 1.11)	9.53E-01	1.14 (1.04, 1.23)	**9.39E-03**	1.04 (0.85, 1.28)
1.01 (0.92, 1.12)	9.53E-01	1.16 (1.06, 1.25)	**2.11E-03**	1.04 (0.85, 1.28)
0.96 (0.87, 1.06)	6.92E-01	1.08 (0.99, 1.18)	1.70E-01	0.87 (0.70, 1.07)
1.05 (0.95, 1.15)	6.16E-01	1.25 (1.15, 1.35)	**2.24E-07**	1.12 (0.92, 1.36)
1.07 (0.97, 1.17)	3.65E-01	1.26 (1.16, 1.36)	**1.22E-07**	1.17 (0.97, 1.42)

1.00 (0.91, 1.11)	9.90E-01	1.22 (1.13, 1.32)	**4.74E-06**	1.00 (0.82, 1.23)
1.00 (0.91, 1.11)	9.73E-01	0.98 (0.90, 1.08)	8.28E-01	1.08 (0.89, 1.32)
0.99 (0.90, 1.09)	9.53E-01	1.04 (0.96, 1.14)	4.90E-01	0.93 (0.75, 1.14)
0.99 (0.89, 1.09)	9.53E-01	1.05 (0.96, 1.15)	4.22E-01	0.92 (0.75, 1.13)
0.96 (0.87, 1.06)	7.18E-01	1.04 (0.95, 1.13)	5.80E-01	0.87 (0.70, 1.07)
0.98 (0.89, 1.09)	9.52E-01	1.06 (0.98, 1.16)	2.94E-01	0.92 (0.75, 1.13)
0.99 (0.89, 1.09)	9.53E-01	1.05 (0.96, 1.15)	4.21E-01	0.95 (0.77, 1.17)
0.98 (0.88, 1.08)	8.95E-01	1.09 (1.00, 1.19)	1.08E-01	0.86 (0.69, 1.06)
1.06 (0.96, 1.17)	3.99E-01	0.98 (0.89, 1.07)	7.50E-01	1.04 (0.85, 1.27)
0.96 (0.87, 1.07)	7.22E-01	1.05 (0.96, 1.15)	4.41E-01	0.87 (0.70, 1.08)
0.96 (0.86, 1.06)	6.68E-01	1.05 (0.96, 1.15)	4.21E-01	0.86 (0.69, 1.07)
0.95 (0.86, 1.05)	5.57E-01	1.02 (0.93, 1.12)	7.69E-01	0.86 (0.69, 1.07)
0.95 (0.85, 1.05)	5.05E-01	1.06 (0.97, 1.16)	3.11E-01	0.84 (0.68, 1.05)
0.94 (0.85, 1.04)	4.49E-01	1.06 (0.97, 1.16)	3.16E-01	0.85 (0.69, 1.06)
0.96 (0.86, 1.06)	6.92E-01	1.06 (0.97, 1.16)	3.09E-01	0.83 (0.67, 1.02)
1.11 (1.01, 1.23)	7.53E-02	1.00 (0.92, 1.10)	9.54E-01	1.08 (0.89, 1.32)
0.91 (0.82, 1.01)	1.58E-01	1.05 (0.96, 1.15)	4.46E-01	0.81 (0.66, 1.00)
0.91 (0.82, 1.01)	1.47E-01	1.05 (0.96, 1.15)	4.33E-01	0.81 (0.65, 0.99)
0.90 (0.81, 1.00)	1.09E-01	0.95 (0.87, 1.05)	4.86E-01	0.82 (0.68, 1.00)
0.90 (0.81, 1.00)	1.37E-01	1.08 (0.99, 1.18)	1.87E-01	0.80 (0.65, 0.99)
0.90 (0.81, 1.00)	1.39E-01	1.09 (1.00, 1.19)	1.28E-01	0.81 (0.65, 1.00)
0.91 (0.82, 1.00)	1.43E-01	1.03 (0.94, 1.13)	7.03E-01	0.81 (0.67, 0.97)
1.05 (0.95, 1.16)	6.16E-01	1.00 (0.91, 1.09)	9.95E-01	0.93 (0.75, 1.15)
0.91 (0.82, 1.01)	1.47E-01	1.07 (0.98, 1.17)	2.40E-01	0.82 (0.67, 1.01)
0.91 (0.82, 1.00)	1.44E-01	1.07 (0.98, 1.17)	2.20E-01	0.82 (0.67, 1.00)
0.91 (0.82, 1.01)	1.47E-01	0.96 (0.88, 1.05)	5.67E-01	0.84 (0.70, 1.01)
0.90 (0.82, 1.00)	1.43E-01	1.11 (1.01, 1.21)	5.97E-02	0.81 (0.65, 1.00)
0.91 (0.82, 1.00)	1.43E-01	1.11 (1.02, 1.22)	**4.09E-02**	0.81 (0.65, 1.00)
0.91 (0.82, 1.01)	1.77E-01	1.06 (0.97, 1.16)	3.88E-01	0.83 (0.68, 1.00)
1.00 (0.90, 1.11)	9.83E-01	1.02 (0.94, 1.12)	7.40E-01	1.01 (0.82, 1.25)
1.24 (1.13, 1.36)	**5.87E-05**	1.22 (1.13, 1.32)	**6.79E-06**	1.17 (0.97, 1.41)
1.24 (1.13, 1.35)	**5.87E-05**	1.22 (1.13, 1.32)	**4.74E-06**	1.17 (0.97, 1.41)
1.23 (1.12, 1.34)	**1.42E-04**	1.14 (1.05, 1.24)	**6.65E-03**	1.07 (0.88, 1.30)
1.23 (1.13, 1.35)	**8.10E-05**	1.31 (1.21, 1.42)	**6.89E-10**	1.25 (1.04, 1.51)
1.23 (1.12, 1.35)	**8.85E-05**	1.35 (1.25, 1.46)	**4.16E-12**	1.28 (1.07, 1.54)
1.23 (1.13, 1.35)	**8.10E-05**	1.20 (1.11, 1.30)	**2.98E-05**	1.18 (0.98, 1.42)
1.20 (1.10, 1.32)	**7.77E-04**	1.13 (1.04, 1.22)	**1.22E-02**	1.36 (1.14, 1.62)
1.25 (1.14, 1.37)	**5.87E-05**	1.05 (0.96, 1.14)	4.60E-01	1.13 (0.93, 1.37)
1.25 (1.14, 1.37)	**5.87E-05**	1.05 (0.96, 1.15)	4.21E-01	1.13 (0.93, 1.37)
1.24 (1.13, 1.36)	**7.78E-05**	1.05 (0.96, 1.15)	4.21E-01	1.13 (0.93, 1.37)
1.24 (1.13, 1.36)	**8.10E-05**	1.06 (0.97, 1.15)	3.33E-01	1.11 (0.92, 1.35)
1.24 (1.13, 1.36)	**7.78E-05**	1.05 (0.96, 1.14)	4.64E-01	1.12 (0.92, 1.36)
1.23 (1.12, 1.35)	**1.42E-04**	1.10 (1.01, 1.20)	6.56E-02	1.10 (0.90, 1.34)
1.26 (1.15, 1.38)	**2.59E-05**	0.94 (0.86, 1.03)	3.26E-01	1.22 (1.01, 1.47)
1.11 (1.01, 1.23)	7.53E-02	0.76 (0.70, 0.83)	**1.65E-08**	1.09 (0.90, 1.32)
1.11 (1.01, 1.23)	7.65E-02	0.77 (0.71, 0.84)	**4.98E-08**	1.08 (0.89, 1.31)
1.16 (1.05, 1.27)	**1.07E-02**	0.79 (0.72, 0.86)	**9.70E-07**	1.14 (0.94, 1.37)
1.08 (0.98, 1.19)	2.52E-01	0.80 (0.73, 0.87)	**1.78E-06**	1.03 (0.84, 1.25)

1.06 (0.96, 1.17)	3.98E-01	0.80 (0.73, 0.87)	**9.69E-07**	1.01 (0.83, 1.24)
1.13 (1.03, 1.24)	**4.45E-02**	0.81 (0.75, 0.89)	**3.17E-05**	1.07 (0.88, 1.30)
1.02 (0.93, 1.13)	9.19E-01	0.73 (0.66, 0.80)	**4.28E-09**	1.05 (0.87, 1.28)
1.02 (0.92, 1.12)	9.53E-01	0.75 (0.70, 0.80)	**1.24E-14**	0.97 (0.79, 1.19)
1.01 (0.92, 1.12)	9.53E-01	0.76 (0.70, 0.81)	**9.32E-14**	0.97 (0.79, 1.19)
1.09 (0.99, 1.20)	1.94E-01	0.75 (0.70, 0.82)	**1.03E-10**	1.17 (0.96, 1.42)
0.92 (0.83, 1.01)	1.77E-01	0.88 (0.80, 0.96)	**9.34E-03**	0.76 (0.63, 0.92)
0.90 (0.81, 0.99)	8.45E-02	0.92 (0.85, 1.01)	1.38E-01	0.74 (0.62, 0.90)
1.12 (1.01, 1.23)	8.65E-02	0.78 (0.72, 0.85)	**1.65E-08**	1.18 (0.97, 1.44)
1.01 (0.91, 1.11)	9.53E-01	0.80 (0.72, 0.88)	**1.85E-05**	1.08 (0.88, 1.32)
1.02 (0.92, 1.13)	9.48E-01	1.00 (0.92, 1.10)	9.66E-01	1.02 (0.83, 1.25)
1.15 (1.06, 1.24)	**2.14E-03**	1.10 (1.02, 1.19)	**4.03E-02**	1.23 (1.10, 1.38)
1.13 (1.05, 1.22)	**8.56E-03**	1.11 (1.03, 1.20)	**1.37E-02**	1.21 (1.09, 1.34)
1.12 (1.03, 1.22)	**2.98E-02**	0.99 (0.90, 1.09)	9.21E-01	1.17 (1.01, 1.35)
0.87 (0.80, 0.95)	**7.98E-03**	0.91 (0.83, 0.99)	7.62E-02	0.80 (0.69, 0.91)
1.04 (0.94, 1.14)	7.70E-01	0.99 (0.90, 1.09)	8.94E-01	1.18 (0.94, 1.49)
1.09 (0.99, 1.20)	1.75E-01	1.11 (1.05, 1.18)	**1.63E-03**	1.11 (0.91, 1.36)
1.01 (0.92, 1.12)	9.53E-01	1.13 (1.06, 1.21)	**1.74E-03**	1.00 (0.82, 1.23)
1.13 (1.02, 1.25)	5.23E-02	1.04 (0.95, 1.13)	5.90E-01	1.18 (0.97, 1.43)
0.92 (0.83, 1.02)	2.52E-01	0.89 (0.82, 0.97)	**3.16E-02**	0.85 (0.68, 1.06)
1.03 (0.93, 1.14)	8.27E-01	0.89 (0.82, 0.97)	**2.68E-02**	1.06 (0.86, 1.30)
0.99 (0.90, 1.09)	9.53E-01	0.89 (0.82, 0.96)	**1.22E-02**	1.00 (0.82, 1.22)
0.92 (0.83, 1.02)	2.24E-01	0.90 (0.82, 0.98)	5.00E-02	0.84 (0.70, 1.02)
1.06 (0.96, 1.18)	4.60E-01	0.95 (0.87, 1.03)	3.85E-01	1.21 (0.96, 1.52)
1.00 (0.91, 1.11)	9.90E-01	1.13 (1.05, 1.23)	**8.80E-03**	0.99 (0.81, 1.21)
0.95 (0.86, 1.05)	5.92E-01	0.84 (0.76, 0.92)	**1.17E-03**	0.66 (0.52, 0.84)
1.05 (0.95, 1.16)	5.57E-01	0.91 (0.83, 1.00)	1.03E-01	0.87 (0.71, 1.07)
1.01 (0.91, 1.12)	9.53E-01	0.92 (0.83, 1.01)	1.33E-01	0.77 (0.62, 0.95)
1.11 (1.00, 1.23)	1.18E-01	0.99 (0.90, 1.08)	8.87E-01	1.40 (1.10, 1.78)
0.96 (0.87, 1.06)	7.18E-01	1.12 (1.02, 1.23)	**4.09E-02**	1.21 (0.98, 1.49)
1.04 (0.95, 1.15)	6.16E-01	0.80 (0.72, 0.88)	**2.18E-05**	0.81 (0.65, 1.01)
1.03 (0.93, 1.13)	8.80E-01	1.29 (1.18, 1.41)	**1.92E-07**	0.95 (0.78, 1.15)
1.00 (0.91, 1.10)	9.90E-01	1.28 (1.17, 1.40)	**7.94E-07**	0.93 (0.76, 1.13)
1.19 (1.09, 1.31)	**1.53E-03**	1.26 (1.16, 1.37)	**7.56E-07**	1.11 (0.91, 1.34)
0.96 (0.87, 1.06)	7.18E-01	0.81 (0.74, 0.89)	**3.15E-05**	1.11 (0.91, 1.36)
0.87 (0.80, 0.95)	**8.04E-03**	0.87 (0.81, 0.95)	**3.62E-03**	0.73 (0.63, 0.83)
1.08 (0.98, 1.19)	2.69E-01	1.47 (1.33, 1.62)	**1.75E-12**	1.31 (1.04, 1.64)
1.12 (1.01, 1.24)	7.53E-02	1.37 (1.25, 1.50)	**7.85E-10**	1.44 (1.16, 1.78)
0.96 (0.88, 1.05)	6.65E-01	1.40 (1.26, 1.56)	**7.70E-09**	0.92 (0.77, 1.11)
1.03 (0.93, 1.13)	8.70E-01	0.80 (0.73, 0.88)	**4.46E-05**	1.07 (0.88, 1.29)
0.89 (0.81, 0.98)	6.93E-02	0.90 (0.83, 0.98)	**4.27E-02**	0.80 (0.66, 0.97)
0.93 (0.86, 1.02)	2.44E-01	1.13 (1.03, 1.24)	**2.78E-02**	0.94 (0.79, 1.11)
0.97 (0.88, 1.07)	8.14E-01	1.02 (0.93, 1.11)	8.05E-01	1.14 (0.92, 1.41)
0.93 (0.84, 1.02)	2.39E-01	1.18 (1.07, 1.31)	**4.33E-03**	0.82 (0.70, 0.97)
1.11 (1.02, 1.21)	6.62E-02	0.92 (0.84, 1.01)	1.83E-01	1.14 (0.96, 1.34)
1.10 (1.01, 1.21)	1.02E-01	0.86 (0.78, 0.95)	**9.49E-03**	1.25 (1.09, 1.43)
0.87 (0.80, 0.95)	**6.21E-03**	1.11 (1.00, 1.22)	8.80E-02	0.80 (0.70, 0.91)
0.87 (0.80, 0.94)	**4.43E-03**	1.08 (0.98, 1.18)	2.39E-01	0.81 (0.71, 0.92)

0.99 (0.89, 1.10)	9.53E-01	1.09 (0.99, 1.19)	1.54E-01	0.89 (0.73, 1.08)
1.17 (1.07, 1.27)	**1.53E-03**	0.96 (0.88, 1.05)	5.31E-01	1.24 (1.08, 1.43)
1.10 (1.00, 1.21)	1.18E-01	0.83 (0.75, 0.92)	**1.48E-03**	1.25 (1.05, 1.49)
0.88 (0.81, 0.97)	**3.62E-02**	1.15 (1.04, 1.27)	**1.48E-02**	0.80 (0.67, 0.95)
0.88 (0.80, 0.96)	**2.68E-02**	1.13 (1.03, 1.25)	**3.17E-02**	0.79 (0.66, 0.94)
1.14 (1.03, 1.25)	**3.19E-02**	0.94 (0.86, 1.03)	3.43E-01	1.28 (1.07, 1.54)
1.17 (1.07, 1.27)	**4.43E-03**	0.97 (0.88, 1.06)	5.93E-01	1.20 (1.01, 1.43)
1.11 (1.01, 1.22)	1.02E-01	0.82 (0.74, 0.90)	**4.45E-04**	1.24 (1.03, 1.49)
0.90 (0.82, 0.99)	8.11E-02	1.17 (1.06, 1.29)	**6.65E-03**	0.80 (0.67, 0.96)
0.89 (0.81, 0.98)	5.79E-02	1.16 (1.05, 1.28)	**1.19E-02**	0.79 (0.66, 0.95)
1.14 (1.04, 1.26)	**3.19E-02**	0.94 (0.85, 1.03)	3.09E-01	1.29 (1.07, 1.55)
1.11 (1.01, 1.22)	9.48E-02	0.96 (0.87, 1.05)	4.90E-01	1.24 (1.03, 1.48)
1.00 (0.91, 1.11)	9.72E-01	0.81 (0.74, 0.88)	**2.46E-06**	0.77 (0.63, 0.94)
0.99 (0.89, 1.10)	9.53E-01	1.38 (1.26, 1.52)	**5.52E-11**	1.31 (1.06, 1.63)
0.97 (0.88, 1.08)	8.73E-01	1.38 (1.26, 1.50)	**6.20E-12**	1.32 (1.07, 1.64)
1.09 (0.98, 1.21)	2.44E-01	1.14 (1.04, 1.25)	**1.99E-02**	1.10 (0.88, 1.37)
1.00 (0.91, 1.11)	9.73E-01	0.87 (0.79, 0.96)	**2.07E-02**	1.14 (0.95, 1.35)
0.84 (0.76, 0.94)	**8.56E-03**	0.95 (0.87, 1.04)	4.33E-01	0.86 (0.69, 1.07)
1.12 (1.01, 1.25)	8.43E-02	1.13 (1.03, 1.24)	**3.16E-02**	1.09 (0.88, 1.35)
1.12 (1.01, 1.24)	1.02E-01	1.00 (0.91, 1.09)	9.54E-01	1.08 (0.87, 1.35)
1.11 (1.00, 1.23)	1.43E-01	1.34 (1.20, 1.48)	**4.21E-07**	1.08 (0.87, 1.34)
1.06 (0.97, 1.17)	3.88E-01	0.87 (0.79, 0.96)	**1.37E-02**	1.10 (0.90, 1.33)
1.22 (1.14, 1.30)	**9.85E-07**	1.16 (1.09, 1.24)	**4.95E-05**	1.26 (1.12, 1.41)
0.88 (0.80, 0.96)	**2.00E-02**	0.98 (0.90, 1.07)	8.03E-01	0.83 (0.71, 0.97)
0.84 (0.78, 0.92)	**4.59E-04**	0.99 (0.91, 1.09)	9.30E-01	0.81 (0.71, 0.94)
1.11 (1.00, 1.23)	1.41E-01	0.95 (0.87, 1.04)	4.21E-01	1.01 (0.82, 1.23)
0.97 (0.88, 1.08)	8.63E-01	0.87 (0.79, 0.95)	**9.49E-03**	1.00 (0.82, 1.23)
1.12 (1.02, 1.24)	6.75E-02	0.92 (0.84, 1.01)	1.47E-01	1.33 (1.10, 1.60)
0.90 (0.82, 1.00)	1.12E-01	1.11 (1.01, 1.22)	5.96E-02	0.77 (0.64, 0.92)
0.90 (0.81, 0.99)	7.81E-02	1.10 (1.00, 1.20)	1.00E-01	0.76 (0.63, 0.91)
1.19 (1.08, 1.31)	**2.40E-03**	1.02 (0.93, 1.11)	8.28E-01	1.42 (1.18, 1.70)
1.01 (0.91, 1.12)	9.53E-01	0.89 (0.82, 0.98)	**4.63E-02**	1.10 (0.90, 1.33)
0.99 (0.90, 1.09)	9.53E-01	0.92 (0.85, 1.01)	1.57E-01	1.28 (1.05, 1.57)
1.17 (1.06, 1.29)	**8.32E-03**	0.90 (0.82, 0.99)	7.32E-02	1.19 (0.98, 1.44)
1.22 (1.11, 1.34)	**3.90E-04**	1.20 (1.10, 1.30)	**1.56E-04**	1.15 (0.94, 1.40)
1.03 (0.93, 1.13)	8.44E-01	1.10 (1.01, 1.20)	6.08E-02	0.99 (0.81, 1.21)
1.00 (0.91, 1.11)	9.75E-01	1.11 (1.02, 1.20)	5.06E-02	1.18 (0.97, 1.44)
0.99 (0.90, 1.10)	9.61E-01	1.10 (1.01, 1.20)	5.96E-02	1.09 (0.89, 1.33)
0.93 (0.84, 1.03)	2.96E-01	1.08 (0.99, 1.18)	1.49E-01	0.87 (0.70, 1.07)
1.19 (1.08, 1.31)	**1.67E-03**	1.03 (0.95, 1.13)	5.93E-01	1.08 (0.89, 1.31)
1.18 (1.08, 1.30)	**2.71E-03**	1.00 (0.92, 1.09)	9.54E-01	1.05 (0.86, 1.28)
1.22 (1.11, 1.34)	**3.52E-04**	1.29 (1.19, 1.40)	**7.70E-09**	1.28 (1.05, 1.55)
1.01 (0.91, 1.11)	9.61E-01	0.83 (0.75, 0.91)	**6.85E-04**	0.88 (0.71, 1.09)
0.82 (0.73, 0.91)	**1.20E-03**	0.97 (0.89, 1.07)	7.22E-01	0.67 (0.54, 0.82)
1.01 (0.91, 1.12)	9.53E-01	0.98 (0.89, 1.07)	7.51E-01	1.18 (0.98, 1.43)
0.99 (0.89, 1.10)	9.53E-01	0.99 (0.91, 1.08)	8.93E-01	1.18 (0.97, 1.43)
1.11 (1.01, 1.22)	8.97E-02	1.01 (0.92, 1.10)	9.23E-01	1.08 (0.89, 1.31)
1.13 (1.03, 1.24)	**4.76E-02**	0.83 (0.75, 0.92)	**8.17E-04**	1.19 (0.99, 1.44)

0.92 (0.83, 1.02)	2.57E-01	1.02 (0.94, 1.12)	7.19E-01	0.82 (0.66, 1.01)
0.98 (0.88, 1.08)	8.96E-01	1.01 (0.93, 1.11)	8.48E-01	1.19 (0.97, 1.45)
0.83 (0.74, 0.92)	**2.05E-03**	0.84 (0.77, 0.92)	**7.99E-04**	0.66 (0.54, 0.81)
0.77 (0.70, 0.86)	**3.72E-05**	0.85 (0.77, 0.93)	**2.09E-03**	0.62 (0.51, 0.76)
0.86 (0.77, 0.95)	**1.57E-02**	0.98 (0.90, 1.08)	8.06E-01	0.69 (0.57, 0.84)
1.19 (1.09, 1.31)	**1.22E-03**	1.07 (0.98, 1.16)	2.51E-01	1.17 (0.97, 1.41)
0.95 (0.86, 1.06)	6.16E-01	1.06 (0.97, 1.16)	3.38E-01	1.03 (0.84, 1.26)
0.88 (0.79, 0.97)	**4.82E-02**	1.02 (0.93, 1.11)	8.03E-01	0.94 (0.76, 1.16)
0.87 (0.78, 0.97)	**3.89E-02**	0.96 (0.87, 1.05)	4.90E-01	0.83 (0.66, 1.04)
0.69 (0.64, 0.75)	**1.39E-15**	0.75 (0.69, 0.82)	**4.08E-09**	0.59 (0.51, 0.67)
1.03 (0.93, 1.14)	8.69E-01	1.44 (1.34, 1.55)	**2.55E-21**	1.42 (1.17, 1.73)
0.77 (0.69, 0.85)	**1.28E-05**	0.96 (0.88, 1.06)	5.95E-01	0.65 (0.54, 0.79)
0.94 (0.84, 1.05)	4.44E-01	1.22 (1.12, 1.32)	**1.51E-05**	1.17 (0.95, 1.44)
0.79 (0.71, 0.88)	**1.42E-04**	0.89 (0.81, 0.98)	**4.44E-02**	0.64 (0.52, 0.79)
0.80 (0.71, 0.89)	**5.48E-04**	0.95 (0.86, 1.04)	4.19E-01	0.69 (0.55, 0.86)
0.92 (0.82, 1.02)	2.24E-01	0.97 (0.89, 1.07)	7.03E-01	0.95 (0.77, 1.18)
0.90 (0.80, 1.00)	1.21E-01	0.95 (0.87, 1.05)	4.76E-01	0.87 (0.69, 1.09)
1.21 (1.09, 1.34)	**1.41E-03**	1.73 (1.59, 1.88)	**2.58E-35**	1.92 (1.61, 2.29)
0.74 (0.68, 0.80)	**1.09E-10**	1.00 (0.91, 1.09)	9.54E-01	0.63 (0.55, 0.72)
1.07 (0.96, 1.19)	3.99E-01	1.44 (1.33, 1.56)	**4.42E-17**	1.60 (1.32, 1.93)
0.76 (0.69, 0.84)	**2.90E-06**	0.86 (0.79, 0.94)	**3.38E-03**	0.54 (0.46, 0.64)
0.76 (0.69, 0.84)	**3.09E-06**	0.97 (0.88, 1.06)	6.68E-01	0.57 (0.48, 0.68)
1.15 (1.05, 1.27)	**1.74E-02**	1.03 (0.94, 1.13)	6.37E-01	1.54 (1.28, 1.86)
1.29 (1.17, 1.42)	**6.21E-06**	1.01 (0.92, 1.11)	8.95E-01	1.49 (1.24, 1.79)
1.11 (1.02, 1.20)	6.20E-02	1.15 (1.08, 1.22)	**5.80E-05**	1.12 (0.95, 1.33)
1.03 (0.94, 1.14)	7.62E-01	1.12 (1.03, 1.22)	**1.79E-02**	1.19 (1.00, 1.42)
1.01 (0.91, 1.12)	9.53E-01	0.82 (0.74, 0.90)	**1.30E-04**	0.77 (0.62, 0.96)
0.86 (0.78, 0.95)	**1.53E-02**	1.04 (0.95, 1.13)	5.19E-01	0.99 (0.80, 1.21)
0.86 (0.78, 0.95)	**1.27E-02**	0.99 (0.91, 1.09)	9.23E-01	0.89 (0.73, 1.09)
0.98 (0.88, 1.08)	8.96E-01	1.19 (1.09, 1.29)	**3.49E-04**	1.49 (1.22, 1.81)
0.98 (0.88, 1.09)	9.26E-01	0.96 (0.87, 1.05)	4.87E-01	1.21 (1.00, 1.47)
1.02 (0.92, 1.13)	9.30E-01	1.09 (1.00, 1.19)	8.80E-02	1.37 (1.15, 1.62)
0.82 (0.74, 0.92)	**3.49E-03**	0.83 (0.76, 0.92)	**7.96E-04**	0.97 (0.78, 1.22)
0.93 (0.83, 1.03)	3.30E-01	1.23 (1.18, 1.28)	**1.28E-19**	1.12 (1.00, 1.24)
0.93 (0.84, 1.02)	2.60E-01	1.09 (1.00, 1.19)	9.61E-02	1.14 (0.95, 1.38)
1.18 (1.09, 1.27)	**1.42E-04**	1.01 (0.95, 1.09)	8.06E-01	1.23 (1.07, 1.43)
1.19 (1.11, 1.27)	**1.28E-05**	0.76 (0.69, 0.85)	**4.74E-06**	1.12 (0.96, 1.31)
1.17 (1.09, 1.26)	**1.42E-04**	0.91 (0.82, 1.02)	2.14E-01	0.94 (0.69, 1.27)
0.99 (0.89, 1.11)	9.53E-01	1.05 (1.00, 1.11)	7.79E-02	0.85 (0.66, 1.10)
0.93 (0.85, 1.02)	2.52E-01	0.70 (0.65, 0.75)	**6.97E-23**	0.89 (0.75, 1.06)
1.09 (0.97, 1.21)	2.78E-01	1.36 (1.26, 1.47)	**2.25E-13**	1.42 (1.16, 1.74)

smoking status, and self-rated health status. P values are corrected for multiple testing
oprotein; VLDL, Very Low Density Lipoprotein; IDL, Intermediate Density Lipoprotein..

atus

	Cerebrovascular diseases mortality			
ker	Never/former drinker		Current drinker	
P value	Adjusted HR (95% CI)	P value	Adjusted HR (95% CI)	P value
3.21E-01	0.87 (0.78, 0.97)	**3.57E-02**	0.97 (0.81, 1.17)	9.82E-01
3.21E-01	0.87 (0.78, 0.97)	**3.89E-02**	0.97 (0.81, 1.17)	9.82E-01
2.84E-01	0.88 (0.79, 0.98)	6.02E-02	0.99 (0.82, 1.18)	9.96E-01
2.84E-01	0.90 (0.81, 1.00)	1.34E-01	0.99 (0.83, 1.19)	9.96E-01
3.11E-01	0.91 (0.82, 1.02)	1.81E-01	0.99 (0.82, 1.19)	9.96E-01
2.65E-01	0.89 (0.80, 0.99)	8.31E-02	0.99 (0.83, 1.19)	9.96E-01
3.37E-01	0.86 (0.77, 0.96)	**2.59E-02**	0.97 (0.80, 1.16)	9.71E-01
2.84E-01	0.89 (0.80, 0.99)	9.19E-02	0.99 (0.82, 1.19)	9.96E-01
2.84E-01	0.89 (0.80, 0.99)	9.41E-02	0.99 (0.83, 1.19)	9.96E-01
2.84E-01	0.89 (0.79, 0.99)	7.70E-02	0.99 (0.83, 1.19)	9.96E-01
2.84E-01	0.91 (0.82, 1.01)	1.60E-01	1.00 (0.83, 1.20)	9.96E-01
2.84E-01	0.92 (0.83, 1.02)	2.12E-01	1.00 (0.83, 1.20)	9.96E-01
2.77E-01	0.89 (0.80, 0.99)	9.81E-02	1.00 (0.83, 1.20)	9.96E-01
2.84E-01	0.89 (0.80, 0.99)	8.31E-02	0.99 (0.82, 1.19)	9.96E-01
2.53E-01	0.91 (0.82, 1.01)	1.72E-01	0.98 (0.82, 1.18)	9.96E-01
2.54E-01	0.91 (0.82, 1.01)	1.72E-01	0.99 (0.82, 1.18)	9.96E-01
2.64E-01	0.91 (0.82, 1.01)	1.67E-01	0.99 (0.82, 1.19)	9.96E-01
2.84E-01	0.90 (0.81, 1.00)	1.30E-01	0.98 (0.82, 1.18)	9.96E-01
3.76E-01	0.89 (0.80, 0.99)	9.56E-02	0.96 (0.80, 1.16)	9.59E-01
2.34E-01	0.91 (0.82, 1.01)	1.72E-01	1.00 (0.84, 1.20)	9.96E-01
2.34E-01	0.91 (0.82, 1.02)	1.85E-01	0.98 (0.82, 1.18)	9.96E-01
2.34E-01	0.92 (0.83, 1.02)	2.22E-01	0.97 (0.81, 1.17)	9.82E-01
2.34E-01	0.92 (0.83, 1.02)	2.18E-01	0.97 (0.81, 1.18)	9.82E-01
2.53E-01	0.91 (0.82, 1.02)	1.85E-01	0.97 (0.80, 1.17)	9.82E-01
2.90E-01	0.91 (0.82, 1.01)	1.81E-01	0.98 (0.81, 1.18)	9.96E-01
4.70E-01	0.90 (0.81, 1.00)	1.34E-01	0.96 (0.80, 1.17)	9.59E-01
2.12E-01	0.93 (0.83, 1.03)	2.71E-01	1.00 (0.83, 1.20)	9.96E-01
2.14E-01	0.93 (0.83, 1.03)	2.59E-01	0.97 (0.81, 1.17)	9.82E-01
3.48E-01	0.95 (0.85, 1.05)	4.44E-01	0.93 (0.77, 1.13)	8.66E-01
3.45E-01	0.96 (0.86, 1.07)	5.38E-01	0.94 (0.77, 1.14)	8.90E-01
4.15E-01	0.93 (0.84, 1.03)	2.98E-01	0.94 (0.78, 1.14)	9.07E-01
4.37E-01	1.06 (0.96, 1.17)	3.74E-01	0.95 (0.78, 1.16)	9.51E-01
6.13E-01	1.09 (0.99, 1.21)	1.67E-01	0.94 (0.77, 1.14)	9.07E-01
2.84E-01	1.00 (0.90, 1.11)	9.53E-01	0.98 (0.81, 1.19)	9.96E-01
3.38E-01	0.92 (0.82, 1.02)	1.94E-01	0.93 (0.77, 1.13)	8.66E-01
7.35E-01	1.03 (0.93, 1.14)	6.21E-01	1.04 (0.87, 1.25)	9.57E-01
7.41E-01	1.05 (0.95, 1.16)	4.57E-01	1.05 (0.87, 1.26)	9.33E-01
3.00E-01	0.98 (0.88, 1.09)	7.52E-01	0.99 (0.82, 1.19)	9.96E-01
3.62E-01	1.13 (1.03, 1.25)	**3.80E-02**	1.10 (0.92, 1.32)	6.29E-01
2.34E-01	1.14 (1.03, 1.25)	**3.27E-02**	1.13 (0.94, 1.35)	5.18E-01

9.94E-01	1.12 (1.01, 1.23)	7.54E-02	1.05 (0.87, 1.26)	9.44E-01
5.31E-01	0.95 (0.85, 1.06)	4.44E-01	1.00 (0.83, 1.21)	9.96E-01
5.48E-01	0.95 (0.85, 1.05)	4.33E-01	1.04 (0.87, 1.25)	9.57E-01
5.22E-01	0.95 (0.86, 1.06)	4.59E-01	1.04 (0.86, 1.25)	9.59E-01
2.97E-01	0.94 (0.85, 1.05)	3.74E-01	1.00 (0.83, 1.21)	9.96E-01
5.22E-01	0.95 (0.86, 1.06)	4.83E-01	1.02 (0.85, 1.23)	9.96E-01
6.93E-01	0.93 (0.84, 1.04)	3.32E-01	1.02 (0.85, 1.23)	9.96E-01
2.77E-01	1.00 (0.91, 1.11)	9.53E-01	1.03 (0.85, 1.23)	9.90E-01
7.48E-01	0.99 (0.89, 1.10)	8.93E-01	1.11 (0.95, 1.31)	5.18E-01
3.11E-01	0.94 (0.84, 1.05)	3.74E-01	1.00 (0.83, 1.21)	9.96E-01
2.84E-01	0.94 (0.85, 1.05)	3.84E-01	0.99 (0.82, 1.20)	9.96E-01
2.84E-01	0.90 (0.81, 1.01)	1.40E-01	0.96 (0.79, 1.16)	9.57E-01
2.53E-01	0.95 (0.85, 1.05)	4.33E-01	0.98 (0.81, 1.18)	9.96E-01
2.77E-01	0.94 (0.85, 1.05)	3.74E-01	0.97 (0.80, 1.18)	9.82E-01
2.12E-01	0.97 (0.87, 1.08)	6.40E-01	1.00 (0.83, 1.21)	9.96E-01
5.16E-01	1.02 (0.92, 1.12)	8.18E-01	1.18 (1.01, 1.38)	1.62E-01
1.49E-01	0.94 (0.84, 1.04)	3.53E-01	0.95 (0.79, 1.15)	9.33E-01
1.44E-01	0.94 (0.84, 1.04)	3.52E-01	0.95 (0.78, 1.14)	9.22E-01
1.46E-01	0.85 (0.77, 0.95)	**1.01E-02**	0.93 (0.78, 1.11)	8.26E-01
1.46E-01	0.96 (0.86, 1.07)	5.34E-01	0.94 (0.77, 1.13)	8.74E-01
1.57E-01	0.97 (0.87, 1.08)	6.26E-01	0.93 (0.77, 1.13)	8.66E-01
9.14E-02	0.92 (0.83, 1.02)	2.15E-01	0.96 (0.79, 1.15)	9.51E-01
5.85E-01	1.01 (0.91, 1.12)	8.91E-01	1.16 (0.98, 1.37)	3.05E-01
1.69E-01	0.94 (0.85, 1.04)	3.74E-01	0.96 (0.79, 1.16)	9.57E-01
1.59E-01	0.94 (0.85, 1.05)	3.77E-01	0.96 (0.79, 1.16)	9.57E-01
1.75E-01	0.85 (0.77, 0.94)	**5.21E-03**	0.96 (0.80, 1.16)	9.58E-01
1.55E-01	0.98 (0.88, 1.08)	7.08E-01	0.95 (0.79, 1.15)	9.33E-01
1.59E-01	0.99 (0.89, 1.09)	8.34E-01	0.94 (0.78, 1.14)	9.07E-01
1.57E-01	0.93 (0.84, 1.03)	2.68E-01	0.98 (0.81, 1.18)	9.96E-01
9.32E-01	0.97 (0.88, 1.08)	6.97E-01	1.07 (0.89, 1.28)	8.66E-01
2.33E-01	1.16 (1.06, 1.28)	**7.66E-03**	1.47 (1.26, 1.72)	**5.09E-05**
2.30E-01	1.16 (1.06, 1.28)	**7.68E-03**	1.47 (1.26, 1.72)	**5.09E-05**
5.84E-01	1.13 (1.02, 1.24)	**4.41E-02**	1.43 (1.22, 1.68)	**1.56E-04**
6.73E-02	1.19 (1.08, 1.31)	**2.20E-03**	1.49 (1.26, 1.75)	**5.39E-05**
**4.27E-02**	1.21 (1.10, 1.33)	**6.76E-04**	1.50 (1.27, 1.77)	**5.09E-05**
2.08E-01	1.13 (1.03, 1.25)	**3.62E-02**	1.45 (1.24, 1.71)	**1.42E-04**
**5.58E-03**	1.11 (1.01, 1.22)	7.73E-02	1.31 (1.13, 1.52)	**4.11E-03**
3.21E-01	1.06 (0.96, 1.17)	3.84E-01	1.45 (1.23, 1.70)	**1.42E-04**
3.32E-01	1.06 (0.96, 1.17)	3.74E-01	1.45 (1.23, 1.70)	**1.43E-04**
3.32E-01	1.04 (0.95, 1.15)	4.92E-01	1.44 (1.22, 1.69)	**1.56E-04**
3.76E-01	1.07 (0.97, 1.18)	2.92E-01	1.44 (1.22, 1.69)	**1.56E-04**
3.59E-01	1.06 (0.96, 1.17)	3.74E-01	1.44 (1.22, 1.70)	**1.56E-04**
4.46E-01	1.10 (1.00, 1.21)	1.30E-01	1.43 (1.22, 1.69)	**2.06E-04**
1.34E-01	1.05 (0.95, 1.17)	4.22E-01	1.46 (1.25, 1.70)	**5.09E-05**
5.06E-01	0.74 (0.67, 0.81)	**2.64E-08**	1.19 (1.00, 1.42)	1.71E-01
5.21E-01	0.74 (0.67, 0.82)	**6.41E-08**	1.19 (1.00, 1.42)	1.71E-01
2.96E-01	0.76 (0.69, 0.84)	**1.87E-06**	1.27 (1.07, 1.50)	**3.49E-02**
8.36E-01	0.76 (0.69, 0.84)	**5.11E-07**	1.14 (0.95, 1.36)	4.64E-01

9.26E-01	0.76 (0.69, 0.84)	**2.66E-07**	1.11 (0.93, 1.33)	6.03E-01
5.59E-01	0.78 (0.71, 0.87)	**2.54E-05**	1.23 (1.03, 1.46)	9.50E-02
6.73E-01	0.74 (0.66, 0.82)	**1.12E-06**	1.00 (0.84, 1.21)	9.96E-01
8.35E-01	0.74 (0.69, 0.80)	**1.52E-12**	1.01 (0.84, 1.21)	9.96E-01
8.06E-01	0.75 (0.69, 0.80)	**2.75E-12**	1.00 (0.83, 1.21)	9.96E-01
2.34E-01	0.74 (0.68, 0.82)	**6.02E-09**	1.10 (0.92, 1.32)	6.35E-01
**3.12E-02**	0.85 (0.77, 0.94)	**5.21E-03**	0.90 (0.75, 1.07)	5.70E-01
**1.32E-02**	0.90 (0.81, 0.99)	7.54E-02	0.88 (0.74, 1.04)	4.21E-01
2.30E-01	0.78 (0.71, 0.85)	**4.58E-07**	1.15 (0.96, 1.37)	4.21E-01
5.45E-01	0.84 (0.75, 0.93)	**4.99E-03**	1.00 (0.83, 1.19)	9.96E-01
9.26E-01	0.98 (0.89, 1.08)	7.77E-01	1.14 (0.92, 1.42)	5.70E-01
**2.95E-03**	1.19 (1.12, 1.27)	**4.93E-07**	1.06 (0.88, 1.27)	9.22E-01
**5.17E-03**	1.20 (1.12, 1.28)	**4.58E-07**	1.04 (0.85, 1.26)	9.81E-01
1.30E-01	1.09 (0.99, 1.19)	1.73E-01	1.07 (0.90, 1.27)	8.66E-01
**8.71E-03**	0.83 (0.76, 0.89)	**1.77E-05**	0.92 (0.77, 1.10)	7.25E-01
2.84E-01	0.87 (0.80, 0.96)	**1.55E-02**	1.00 (0.83, 1.20)	9.96E-01
4.02E-01	1.13 (1.06, 1.22)	**2.67E-03**	1.11 (0.93, 1.34)	5.86E-01
9.86E-01	1.20 (1.12, 1.29)	**9.19E-06**	1.10 (0.91, 1.32)	6.66E-01
2.33E-01	0.93 (0.84, 1.03)	2.70E-01	1.05 (0.86, 1.29)	9.33E-01
2.77E-01	0.96 (0.87, 1.07)	5.78E-01	0.93 (0.76, 1.14)	8.66E-01
6.73E-01	0.96 (0.87, 1.06)	5.33E-01	1.01 (0.84, 1.21)	9.96E-01
9.99E-01	0.94 (0.86, 1.04)	3.74E-01	0.95 (0.80, 1.14)	9.32E-01
1.94E-01	0.95 (0.85, 1.05)	4.08E-01	0.86 (0.71, 1.03)	3.49E-01
2.34E-01	0.98 (0.89, 1.08)	7.59E-01	1.07 (0.88, 1.30)	8.66E-01
9.46E-01	1.06 (0.96, 1.17)	3.74E-01	1.04 (0.87, 1.24)	9.59E-01
**5.88E-03**	0.87 (0.79, 0.97)	**3.80E-02**	0.92 (0.76, 1.12)	8.21E-01
3.00E-01	0.97 (0.87, 1.07)	6.12E-01	1.06 (0.88, 1.28)	9.07E-01
6.73E-02	0.95 (0.85, 1.05)	4.44E-01	1.02 (0.85, 1.23)	9.96E-01
**3.84E-02**	1.05 (0.95, 1.17)	4.44E-01	1.12 (0.92, 1.38)	5.86E-01
1.94E-01	1.06 (0.95, 1.17)	4.33E-01	0.96 (0.80, 1.16)	9.57E-01
1.78E-01	0.82 (0.74, 0.91)	**1.62E-03**	1.12 (0.93, 1.34)	5.65E-01
6.59E-01	1.20 (1.09, 1.33)	**2.13E-03**	1.09 (0.90, 1.31)	7.69E-01
5.48E-01	1.18 (1.07, 1.31)	**5.48E-03**	1.04 (0.86, 1.26)	9.57E-01
4.06E-01	1.22 (1.11, 1.34)	**2.90E-04**	1.35 (1.14, 1.59)	**4.38E-03**
4.02E-01	0.87 (0.79, 0.96)	**2.67E-02**	0.90 (0.75, 1.08)	5.86E-01
**1.80E-04**	0.86 (0.79, 0.94)	**2.94E-03**	0.87 (0.74, 1.02)	3.02E-01
7.75E-02	1.33 (1.19, 1.49)	**6.01E-06**	1.16 (0.95, 1.41)	4.27E-01
**7.54E-03**	1.27 (1.14, 1.41)	**8.08E-05**	1.18 (0.97, 1.42)	3.14E-01
4.92E-01	1.25 (1.12, 1.40)	**6.29E-04**	1.03 (0.85, 1.23)	9.82E-01
5.90E-01	0.88 (0.79, 0.98)	5.98E-02	0.98 (0.82, 1.19)	9.96E-01
8.01E-02	0.96 (0.87, 1.05)	4.67E-01	0.85 (0.71, 1.00)	2.07E-01
5.45E-01	1.01 (0.92, 1.12)	8.67E-01	0.92 (0.78, 1.08)	6.44E-01
3.21E-01	0.91 (0.83, 1.00)	1.34E-01	0.94 (0.79, 1.12)	8.66E-01
7.52E-02	1.14 (1.02, 1.28)	5.64E-02	0.94 (0.79, 1.12)	8.82E-01
2.72E-01	1.01 (0.91, 1.11)	9.26E-01	1.14 (0.97, 1.34)	3.35E-01
**1.17E-02**	0.93 (0.84, 1.03)	2.72E-01	0.94 (0.77, 1.16)	9.26E-01
**7.07E-03**	1.01 (0.92, 1.11)	8.85E-01	0.92 (0.78, 1.09)	7.06E-01
**1.17E-02**	0.97 (0.88, 1.06)	5.78E-01	0.91 (0.77, 1.08)	6.22E-01

3.42E-01	1.14 (1.02, 1.27)	5.25E-02	1.02 (0.84, 1.23)	9.96E-01
**1.78E-02**	1.04 (0.95, 1.14)	4.79E-01	1.17 (1.01, 1.35)	1.68E-01
5.23E-02	0.90 (0.81, 1.00)	1.30E-01	1.03 (0.86, 1.24)	9.82E-01
5.53E-02	1.05 (0.95, 1.16)	4.59E-01	0.91 (0.76, 1.08)	6.13E-01
**4.04E-02**	1.03 (0.93, 1.14)	6.26E-01	0.90 (0.76, 1.07)	5.74E-01
**3.85E-02**	1.02 (0.92, 1.12)	7.93E-01	1.12 (0.93, 1.34)	5.65E-01
1.30E-01	1.06 (0.97, 1.17)	3.36E-01	1.21 (1.04, 1.41)	7.74E-02
7.75E-02	0.89 (0.80, 1.00)	1.02E-01	1.06 (0.88, 1.27)	9.07E-01
6.37E-02	1.07 (0.96, 1.19)	3.22E-01	0.93 (0.78, 1.11)	7.85E-01
**4.84E-02**	1.06 (0.95, 1.17)	4.19E-01	0.91 (0.77, 1.09)	6.61E-01
**4.04E-02**	1.01 (0.91, 1.12)	8.83E-01	1.15 (0.96, 1.38)	3.82E-01
7.52E-02	1.02 (0.93, 1.13)	7.13E-01	1.11 (0.93, 1.31)	5.70E-01
**4.84E-02**	0.89 (0.81, 0.98)	6.37E-02	1.03 (0.85, 1.25)	9.82E-01
5.72E-02	1.19 (1.07, 1.32)	**5.21E-03**	0.97 (0.80, 1.17)	9.82E-01
**4.60E-02**	1.21 (1.09, 1.33)	**1.77E-03**	0.95 (0.79, 1.16)	9.57E-01
4.94E-01	1.01 (0.92, 1.12)	8.42E-01	1.05 (0.86, 1.28)	9.51E-01
2.84E-01	0.92 (0.83, 1.03)	2.33E-01	0.99 (0.83, 1.19)	9.96E-01
2.84E-01	0.91 (0.82, 1.01)	1.72E-01	0.73 (0.60, 0.89)	**1.62E-02**
5.22E-01	1.13 (1.01, 1.26)	8.17E-02	1.24 (1.01, 1.53)	1.71E-01
5.45E-01	1.03 (0.92, 1.14)	6.85E-01	1.19 (0.98, 1.45)	2.87E-01
5.55E-01	1.27 (1.13, 1.43)	**6.03E-04**	1.22 (0.99, 1.51)	2.20E-01
4.57E-01	0.94 (0.84, 1.05)	3.75E-01	1.15 (0.97, 1.36)	3.35E-01
**1.11E-03**	1.23 (1.16, 1.31)	**5.93E-10**	1.25 (1.13, 1.38)	**1.46E-04**
7.52E-02	0.89 (0.81, 0.97)	**2.26E-02**	0.86 (0.75, 0.99)	1.71E-01
**3.12E-02**	0.89 (0.82, 0.97)	**2.80E-02**	0.83 (0.73, 0.94)	**1.65E-02**
9.66E-01	0.91 (0.83, 1.00)	1.24E-01	1.22 (1.00, 1.48)	1.89E-01
9.82E-01	0.93 (0.83, 1.03)	2.92E-01	0.91 (0.76, 1.11)	7.26E-01
**1.76E-02**	0.91 (0.82, 1.01)	1.68E-01	1.13 (0.96, 1.35)	4.27E-01
**3.12E-02**	1.09 (0.99, 1.21)	1.82E-01	0.90 (0.76, 1.07)	5.68E-01
**2.11E-02**	1.08 (0.98, 1.20)	2.41E-01	0.89 (0.75, 1.05)	4.69E-01
**2.95E-03**	1.02 (0.92, 1.13)	7.59E-01	1.25 (1.05, 1.48)	6.08E-02
4.59E-01	0.97 (0.87, 1.07)	6.18E-01	0.99 (0.83, 1.19)	9.96E-01
6.30E-02	0.93 (0.84, 1.03)	2.72E-01	0.95 (0.79, 1.14)	9.22E-01
2.14E-01	1.02 (0.93, 1.13)	7.04E-01	1.12 (0.94, 1.35)	5.37E-01
2.84E-01	1.19 (1.08, 1.31)	**2.68E-03**	1.49 (1.25, 1.78)	**1.52E-04**
9.38E-01	0.96 (0.86, 1.06)	5.33E-01	1.13 (0.95, 1.36)	4.69E-01
2.30E-01	0.99 (0.89, 1.10)	8.77E-01	1.01 (0.84, 1.22)	9.96E-01
5.09E-01	0.97 (0.88, 1.08)	6.82E-01	1.02 (0.85, 1.23)	9.96E-01
2.85E-01	0.96 (0.87, 1.07)	5.60E-01	0.96 (0.79, 1.16)	9.57E-01
5.45E-01	0.98 (0.88, 1.08)	7.23E-01	1.38 (1.17, 1.63)	**1.55E-03**
6.73E-01	0.95 (0.86, 1.05)	4.59E-01	1.36 (1.15, 1.61)	**2.74E-03**
5.72E-02	1.20 (1.08, 1.32)	**1.77E-03**	1.46 (1.23, 1.74)	**2.59E-04**
3.39E-01	0.76 (0.68, 0.84)	**9.25E-06**	1.14 (0.96, 1.35)	4.27E-01
**2.01E-03**	0.88 (0.78, 0.98)	5.98E-02	0.77 (0.64, 0.93)	**4.28E-02**
2.17E-01	0.92 (0.83, 1.02)	2.33E-01	1.02 (0.85, 1.22)	9.96E-01
2.19E-01	0.92 (0.83, 1.02)	2.22E-01	0.98 (0.82, 1.19)	9.96E-01
5.19E-01	1.00 (0.90, 1.11)	9.69E-01	1.20 (1.03, 1.40)	8.82E-02
1.87E-01	0.88 (0.79, 0.98)	6.29E-02	1.21 (1.02, 1.42)	1.25E-01

1.75E-01	0.93 (0.84, 1.03)	2.41E-01	0.90 (0.75, 1.09)	6.22E-01
2.27E-01	0.97 (0.88, 1.08)	6.94E-01	0.92 (0.76, 1.11)	7.69E-01
**9.21E-04**	0.80 (0.72, 0.89)	**1.66E-04**	0.80 (0.66, 0.96)	8.82E-02
**7.86E-05**	0.79 (0.71, 0.88)	**1.59E-04**	0.73 (0.61, 0.88)	**6.35E-03**
**2.95E-03**	0.91 (0.82, 1.01)	1.60E-01	0.84 (0.70, 1.01)	2.07E-01
2.34E-01	0.96 (0.87, 1.06)	5.16E-01	1.40 (1.19, 1.65)	**6.85E-04**
8.36E-01	0.93 (0.83, 1.03)	2.79E-01	0.97 (0.80, 1.17)	9.82E-01
6.29E-01	0.95 (0.85, 1.05)	4.32E-01	0.81 (0.67, 0.99)	1.64E-01
2.30E-01	0.86 (0.77, 0.96)	**2.30E-02**	0.81 (0.66, 0.99)	1.71E-01
**1.81E-12**	0.76 (0.69, 0.83)	**3.28E-07**	0.65 (0.57, 0.75)	**1.11E-07**
**4.02E-03**	1.25 (1.14, 1.37)	**2.75E-05**	1.03 (0.84, 1.26)	9.82E-01
**3.36E-04**	0.88 (0.79, 0.99)	8.21E-02	0.72 (0.60, 0.86)	**2.28E-03**
2.77E-01	1.04 (0.94, 1.16)	5.23E-01	0.92 (0.75, 1.14)	8.66E-01
**8.01E-04**	0.81 (0.72, 0.91)	**1.76E-03**	0.75 (0.61, 0.91)	**2.03E-02**
**8.71E-03**	0.84 (0.75, 0.94)	**1.22E-02**	0.76 (0.62, 0.93)	**3.91E-02**
7.19E-01	0.90 (0.81, 1.00)	1.05E-01	0.89 (0.73, 1.08)	5.68E-01
3.21E-01	0.85 (0.76, 0.95)	**1.54E-02**	0.81 (0.66, 1.00)	1.71E-01
**2.92E-11**	1.50 (1.36, 1.66)	**3.28E-13**	1.31 (1.09, 1.58)	**2.66E-02**
**3.59E-10**	1.04 (0.94, 1.15)	5.33E-01	0.72 (0.63, 0.82)	**5.09E-05**
**4.29E-05**	1.24 (1.13, 1.37)	**8.62E-05**	1.15 (0.94, 1.40)	4.64E-01
**5.42E-11**	0.90 (0.81, 1.00)	1.02E-01	0.76 (0.64, 0.91)	**1.62E-02**
**3.21E-08**	0.96 (0.87, 1.07)	5.61E-01	0.77 (0.65, 0.92)	**2.72E-02**
**1.15E-04**	1.05 (0.95, 1.16)	4.44E-01	1.21 (1.01, 1.45)	1.60E-01
**4.70E-04**	0.99 (0.90, 1.10)	9.12E-01	1.21 (1.01, 1.45)	1.64E-01
2.98E-01	1.18 (1.11, 1.26)	**1.05E-05**	1.24 (1.09, 1.40)	**5.77E-03**
1.55E-01	1.02 (0.93, 1.13)	7.00E-01	1.14 (0.96, 1.35)	4.02E-01
7.47E-02	0.83 (0.74, 0.92)	**3.60E-03**	0.87 (0.71, 1.06)	4.64E-01
9.32E-01	1.03 (0.94, 1.14)	5.85E-01	0.75 (0.61, 0.91)	**2.46E-02**
3.55E-01	1.07 (0.97, 1.18)	3.22E-01	0.93 (0.77, 1.12)	8.66E-01
**1.08E-03**	1.20 (1.09, 1.31)	**9.27E-04**	0.96 (0.80, 1.15)	9.57E-01
1.55E-01	0.91 (0.82, 1.01)	1.78E-01	0.92 (0.76, 1.12)	7.85E-01
**3.84E-03**	0.98 (0.89, 1.09)	8.16E-01	0.94 (0.77, 1.14)	9.07E-01
8.61E-01	0.81 (0.72, 0.90)	**6.29E-04**	0.64 (0.52, 0.80)	**6.93E-04**
1.42E-01	1.22 (1.17, 1.28)	**6.17E-16**	0.90 (0.73, 1.11)	7.06E-01
2.84E-01	1.20 (1.09, 1.32)	**7.84E-04**	0.86 (0.72, 1.04)	3.97E-01
**2.66E-02**	1.04 (0.98, 1.10)	3.28E-01	1.37 (1.22, 1.53)	**6.70E-06**
2.72E-01	0.81 (0.72, 0.92)	**2.92E-03**	1.19 (1.07, 1.31)	**6.35E-03**
7.31E-01	0.89 (0.78, 1.01)	1.67E-01	1.19 (1.05, 1.36)	**4.70E-02**
3.21E-01	1.08 (1.03, 1.12)	**1.77E-03**	1.16 (1.03, 1.31)	7.06E-02
3.17E-01	0.76 (0.70, 0.83)	**1.97E-09**	0.79 (0.68, 0.92)	**1.65E-02**
**5.85E-03**	1.21 (1.10, 1.32)	**6.29E-04**	1.13 (0.94, 1.36)	5.18E-01

Cancer mortality				
Never/former drinker		Current drinker		Never/former dri
Adjusted HR (95% CI)	P value	Adjusted HR (95% CI	P value	Adjusted HR (95% CI)
0.93 (0.85, 1.02)	3.61E-01	0.93 (0.76, 1.14)	8.52E-01	1.03 (0.89, 1.19)
0.93 (0.85, 1.02)	3.94E-01	0.93 (0.76, 1.14)	8.52E-01	1.03 (0.89, 1.20)
0.93 (0.85, 1.02)	3.55E-01	0.95 (0.78, 1.17)	8.73E-01	1.04 (0.90, 1.20)
0.97 (0.89, 1.06)	8.23E-01	0.96 (0.79, 1.16)	8.73E-01	1.06 (0.92, 1.22)
0.99 (0.91, 1.07)	9.34E-01	0.97 (0.80, 1.17)	8.96E-01	1.05 (0.91, 1.22)
0.96 (0.88, 1.04)	6.13E-01	0.97 (0.79, 1.18)	8.96E-01	1.06 (0.92, 1.22)
0.92 (0.84, 1.01)	2.98E-01	0.92 (0.75, 1.12)	8.52E-01	1.02 (0.88, 1.19)
0.98 (0.90, 1.07)	8.41E-01	0.98 (0.81, 1.20)	9.14E-01	1.03 (0.89, 1.19)
0.98 (0.90, 1.07)	8.54E-01	0.98 (0.80, 1.20)	9.12E-01	1.03 (0.89, 1.20)
0.98 (0.90, 1.07)	8.41E-01	0.98 (0.81, 1.20)	9.22E-01	1.05 (0.90, 1.21)
0.99 (0.91, 1.08)	9.54E-01	0.98 (0.80, 1.19)	8.96E-01	1.03 (0.89, 1.20)
0.99 (0.91, 1.08)	9.54E-01	0.97 (0.80, 1.18)	8.96E-01	1.02 (0.88, 1.19)
0.99 (0.91, 1.08)	9.54E-01	0.98 (0.81, 1.19)	9.12E-01	1.05 (0.90, 1.21)
0.97 (0.89, 1.06)	8.23E-01	0.99 (0.81, 1.20)	9.33E-01	1.03 (0.89, 1.19)
0.95 (0.87, 1.03)	5.21E-01	0.94 (0.77, 1.14)	8.52E-01	1.01 (0.87, 1.17)
0.95 (0.87, 1.03)	5.31E-01	0.94 (0.78, 1.14)	8.52E-01	1.01 (0.87, 1.17)
0.95 (0.87, 1.03)	5.35E-01	0.94 (0.78, 1.14)	8.52E-01	1.02 (0.88, 1.18)
0.96 (0.88, 1.05)	6.63E-01	0.95 (0.78, 1.14)	8.57E-01	1.00 (0.87, 1.16)
0.97 (0.89, 1.05)	7.90E-01	0.95 (0.79, 1.14)	8.57E-01	0.98 (0.84, 1.14)
0.95 (0.88, 1.04)	5.89E-01	0.95 (0.78, 1.15)	8.57E-01	1.03 (0.89, 1.19)
0.94 (0.87, 1.03)	4.99E-01	0.94 (0.77, 1.14)	8.52E-01	1.01 (0.87, 1.17)
0.95 (0.87, 1.03)	5.04E-01	0.91 (0.76, 1.09)	8.52E-01	0.99 (0.85, 1.14)
0.95 (0.87, 1.03)	5.21E-01	0.91 (0.76, 1.09)	8.52E-01	0.99 (0.85, 1.14)
0.95 (0.87, 1.03)	5.17E-01	0.91 (0.76, 1.09)	8.52E-01	0.98 (0.85, 1.14)
0.97 (0.90, 1.05)	8.06E-01	0.93 (0.78, 1.11)	8.52E-01	0.98 (0.85, 1.14)
0.99 (0.91, 1.07)	9.38E-01	0.94 (0.79, 1.11)	8.52E-01	0.96 (0.83, 1.12)
0.96 (0.88, 1.04)	5.96E-01	0.93 (0.77, 1.11)	8.52E-01	1.00 (0.87, 1.16)
0.94 (0.86, 1.02)	4.05E-01	0.91 (0.75, 1.09)	8.50E-01	0.99 (0.85, 1.15)
0.96 (0.89, 1.04)	6.04E-01	0.93 (0.79, 1.10)	8.52E-01	0.96 (0.82, 1.11)
0.96 (0.89, 1.04)	6.12E-01	0.93 (0.79, 1.10)	8.52E-01	0.95 (0.82, 1.11)
0.95 (0.88, 1.03)	5.24E-01	0.94 (0.80, 1.11)	8.52E-01	0.94 (0.81, 1.09)
0.98 (0.90, 1.06)	8.34E-01	0.95 (0.81, 1.11)	8.52E-01	0.93 (0.80, 1.08)
0.99 (0.91, 1.07)	9.28E-01	0.95 (0.82, 1.12)	8.57E-01	0.92 (0.79, 1.07)
0.97 (0.89, 1.04)	6.87E-01	0.95 (0.81, 1.12)	8.52E-01	0.96 (0.82, 1.11)
0.96 (0.88, 1.04)	5.99E-01	0.93 (0.78, 1.10)	8.52E-01	0.98 (0.84, 1.14)
0.99 (0.91, 1.07)	9.22E-01	0.96 (0.83, 1.12)	8.73E-01	0.98 (0.84, 1.13)
0.99 (0.91, 1.07)	9.22E-01	0.96 (0.82, 1.12)	8.69E-01	0.97 (0.84, 1.13)
1.00 (0.93, 1.09)	9.91E-01	0.98 (0.84, 1.14)	8.96E-01	0.96 (0.83, 1.11)
0.98 (0.91, 1.06)	8.54E-01	0.95 (0.81, 1.11)	8.52E-01	0.98 (0.85, 1.12)
0.98 (0.91, 1.05)	8.23E-01	0.95 (0.81, 1.11)	8.52E-01	0.98 (0.85, 1.13)

0.99 (0.92, 1.07)	9.53E-01	0.96 (0.82, 1.12)	8.57E-01	0.98 (0.85, 1.12)
0.99 (0.91, 1.07)	9.34E-01	0.98 (0.83, 1.15)	8.96E-01	1.00 (0.86, 1.16)
1.01 (0.93, 1.09)	9.54E-01	0.97 (0.84, 1.13)	8.95E-01	0.96 (0.83, 1.12)
1.01 (0.93, 1.09)	9.54E-01	0.97 (0.83, 1.13)	8.87E-01	0.95 (0.82, 1.11)
1.00 (0.93, 1.09)	9.82E-01	0.96 (0.83, 1.13)	8.73E-01	0.93 (0.80, 1.08)
1.01 (0.93, 1.09)	9.69E-01	0.97 (0.83, 1.13)	8.87E-01	0.93 (0.80, 1.08)
1.00 (0.92, 1.08)	9.99E-01	0.96 (0.83, 1.12)	8.73E-01	0.92 (0.79, 1.07)
1.02 (0.94, 1.10)	8.41E-01	0.98 (0.84, 1.15)	9.12E-01	0.96 (0.83, 1.11)
1.02 (0.95, 1.11)	8.23E-01	1.02 (0.87, 1.21)	8.96E-01	1.11 (0.97, 1.27)
0.98 (0.91, 1.07)	9.05E-01	0.98 (0.84, 1.14)	8.96E-01	0.92 (0.79, 1.07)
0.98 (0.91, 1.07)	9.05E-01	0.98 (0.84, 1.14)	8.96E-01	0.92 (0.79, 1.07)
0.98 (0.91, 1.07)	8.85E-01	0.98 (0.84, 1.15)	8.96E-01	0.89 (0.76, 1.04)
0.98 (0.91, 1.06)	8.69E-01	0.97 (0.83, 1.14)	8.95E-01	0.91 (0.78, 1.06)
0.97 (0.90, 1.06)	8.23E-01	0.96 (0.82, 1.13)	8.73E-01	0.90 (0.77, 1.05)
1.00 (0.92, 1.08)	9.99E-01	0.99 (0.85, 1.16)	9.39E-01	0.92 (0.80, 1.07)
1.03 (0.95, 1.12)	7.50E-01	1.06 (0.91, 1.25)	8.52E-01	1.12 (0.98, 1.28)
0.96 (0.88, 1.04)	6.05E-01	0.95 (0.81, 1.12)	8.52E-01	0.89 (0.76, 1.04)
0.96 (0.88, 1.04)	6.04E-01	0.95 (0.81, 1.11)	8.52E-01	0.88 (0.76, 1.03)
0.95 (0.87, 1.03)	5.21E-01	0.95 (0.81, 1.13)	8.57E-01	0.86 (0.74, 1.01)
0.96 (0.88, 1.04)	5.99E-01	0.94 (0.80, 1.11)	8.52E-01	0.88 (0.75, 1.02)
0.96 (0.88, 1.04)	5.99E-01	0.94 (0.80, 1.10)	8.52E-01	0.88 (0.75, 1.03)
0.96 (0.88, 1.04)	6.04E-01	0.95 (0.81, 1.13)	8.57E-01	0.86 (0.74, 1.01)
1.02 (0.94, 1.11)	8.69E-01	1.05 (0.88, 1.24)	8.57E-01	1.11 (0.96, 1.27)
0.94 (0.87, 1.03)	4.99E-01	0.94 (0.79, 1.10)	8.52E-01	0.87 (0.74, 1.01)
0.94 (0.87, 1.03)	4.99E-01	0.94 (0.79, 1.10)	8.52E-01	0.87 (0.74, 1.01)
0.94 (0.86, 1.02)	4.05E-01	0.95 (0.80, 1.13)	8.57E-01	0.84 (0.73, 0.98)
0.95 (0.87, 1.03)	5.17E-01	0.93 (0.79, 1.10)	8.52E-01	0.87 (0.74, 1.01)
0.95 (0.87, 1.03)	5.17E-01	0.93 (0.79, 1.10)	8.52E-01	0.87 (0.75, 1.02)
0.94 (0.86, 1.02)	4.05E-01	0.94 (0.79, 1.11)	8.52E-01	0.85 (0.73, 0.99)
0.98 (0.91, 1.07)	9.05E-01	0.97 (0.81, 1.15)	8.95E-01	1.03 (0.89, 1.20)
1.11 (1.04, 1.20)	**2.79E-02**	1.13 (0.97, 1.32)	6.04E-01	1.15 (1.01, 1.32)
1.11 (1.03, 1.19)	**2.98E-02**	1.13 (0.97, 1.31)	6.05E-01	1.15 (1.00, 1.31)
1.15 (1.07, 1.23)	**1.86E-03**	1.18 (1.01, 1.37)	3.60E-01	1.16 (1.01, 1.33)
1.06 (0.99, 1.14)	3.31E-01	1.07 (0.92, 1.25)	8.52E-01	1.10 (0.96, 1.27)
1.04 (0.97, 1.12)	5.69E-01	1.05 (0.90, 1.23)	8.52E-01	1.10 (0.95, 1.26)
1.11 (1.04, 1.19)	**2.70E-02**	1.13 (0.97, 1.32)	6.04E-01	1.12 (0.98, 1.28)
1.05 (0.98, 1.14)	4.99E-01	1.02 (0.86, 1.21)	9.12E-01	1.23 (1.07, 1.40)
1.12 (1.03, 1.20)	**3.20E-02**	1.20 (1.03, 1.41)	3.09E-01	1.19 (1.03, 1.37)
1.12 (1.03, 1.20)	**3.20E-02**	1.20 (1.03, 1.41)	3.09E-01	1.18 (1.03, 1.36)
1.10 (1.02, 1.19)	6.34E-02	1.21 (1.03, 1.41)	3.09E-01	1.16 (1.00, 1.34)
1.12 (1.04, 1.21)	**2.70E-02**	1.20 (1.03, 1.40)	3.13E-01	1.19 (1.03, 1.37)
1.12 (1.04, 1.21)	**2.70E-02**	1.20 (1.03, 1.40)	3.09E-01	1.19 (1.04, 1.37)
1.12 (1.04, 1.21)	**2.81E-02**	1.19 (1.02, 1.39)	3.20E-01	1.17 (1.02, 1.35)
1.11 (1.03, 1.20)	**4.01E-02**	1.13 (0.96, 1.32)	6.05E-01	1.29 (1.13, 1.48)
0.98 (0.90, 1.07)	8.80E-01	1.13 (0.97, 1.32)	6.04E-01	0.87 (0.75, 1.02)
0.98 (0.91, 1.07)	8.95E-01	1.14 (0.97, 1.32)	6.04E-01	0.87 (0.75, 1.01)
0.98 (0.90, 1.06)	8.34E-01	1.14 (0.98, 1.33)	5.65E-01	0.91 (0.79, 1.06)
0.99 (0.91, 1.08)	9.54E-01	1.13 (0.96, 1.32)	6.05E-01	0.85 (0.74, 0.99)

0.99 (0.91, 1.07)	9.41E-01	1.12 (0.96, 1.32)	6.63E-01	0.84 (0.73, 0.98)
1.00 (0.92, 1.08)	9.95E-01	1.15 (0.99, 1.34)	5.24E-01	0.90 (0.77, 1.05)
0.99 (0.91, 1.07)	9.54E-01	1.04 (0.90, 1.22)	8.57E-01	0.95 (0.81, 1.10)
0.91 (0.84, 0.99)	1.20E-01	1.11 (0.95, 1.30)	7.19E-01	0.79 (0.69, 0.89)
0.91 (0.84, 0.99)	1.29E-01	1.11 (0.95, 1.31)	7.19E-01	0.78 (0.69, 0.88)
0.91 (0.84, 0.99)	1.20E-01	1.10 (0.93, 1.29)	8.08E-01	0.84 (0.73, 0.97)
0.97 (0.89, 1.06)	8.17E-01	1.07 (0.90, 1.27)	8.52E-01	0.79 (0.69, 0.91)
0.99 (0.91, 1.07)	9.28E-01	1.04 (0.87, 1.24)	8.74E-01	0.82 (0.71, 0.95)
0.93 (0.86, 1.01)	3.09E-01	1.14 (0.96, 1.34)	6.05E-01	0.82 (0.72, 0.95)
0.96 (0.89, 1.04)	6.12E-01	1.00 (0.84, 1.18)	9.77E-01	0.99 (0.85, 1.15)
0.85 (0.79, 0.92)	**4.46E-04**	0.95 (0.81, 1.10)	8.52E-01	1.03 (0.87, 1.21)
1.17 (1.09, 1.26)	**3.01E-04**	1.13 (0.98, 1.30)	6.03E-01	1.13 (0.99, 1.30)
1.17 (1.09, 1.25)	**3.05E-04**	1.10 (0.94, 1.29)	7.68E-01	1.09 (0.95, 1.26)
1.06 (0.97, 1.16)	5.35E-01	1.14 (0.99, 1.32)	5.24E-01	1.15 (0.98, 1.34)
0.92 (0.84, 1.01)	2.68E-01	0.91 (0.78, 1.07)	8.08E-01	0.88 (0.76, 1.02)
0.93 (0.85, 1.01)	3.09E-01	0.95 (0.82, 1.09)	8.52E-01	0.99 (0.84, 1.16)
1.05 (0.99, 1.12)	2.98E-01	1.01 (0.85, 1.20)	9.39E-01	1.05 (0.93, 1.20)
1.06 (1.00, 1.13)	1.65E-01	0.93 (0.79, 1.09)	8.52E-01	1.02 (0.89, 1.18)
1.00 (0.91, 1.09)	9.82E-01	1.12 (0.95, 1.32)	7.19E-01	1.07 (0.92, 1.25)
0.97 (0.90, 1.06)	8.23E-01	1.03 (0.88, 1.21)	8.96E-01	0.96 (0.82, 1.12)
0.92 (0.84, 1.01)	2.98E-01	1.02 (0.87, 1.20)	8.96E-01	1.08 (0.91, 1.28)
1.06 (0.97, 1.17)	5.24E-01	1.00 (0.85, 1.18)	9.77E-01	1.02 (0.87, 1.20)
1.16 (1.08, 1.25)	**9.93E-04**	1.03 (0.87, 1.23)	8.96E-01	0.99 (0.85, 1.15)
0.90 (0.83, 0.97)	5.10E-02	0.98 (0.84, 1.15)	8.96E-01	1.04 (0.89, 1.22)
0.97 (0.89, 1.07)	8.39E-01	0.99 (0.84, 1.17)	9.39E-01	0.97 (0.82, 1.14)
1.07 (0.99, 1.16)	2.98E-01	1.16 (1.00, 1.33)	3.67E-01	0.97 (0.83, 1.12)
1.15 (1.06, 1.24)	**9.91E-03**	1.20 (1.02, 1.40)	3.20E-01	1.07 (0.92, 1.25)
1.15 (1.06, 1.24)	**3.73E-03**	1.17 (1.00, 1.37)	3.67E-01	1.04 (0.89, 1.20)
0.94 (0.87, 1.02)	4.77E-01	1.02 (0.89, 1.18)	8.96E-01	1.10 (0.94, 1.30)
0.87 (0.80, 0.95)	**9.91E-03**	0.82 (0.70, 0.96)	3.09E-01	0.94 (0.81, 1.10)
1.04 (0.95, 1.12)	7.23E-01	1.11 (0.97, 1.27)	6.04E-01	0.96 (0.83, 1.12)
1.04 (0.96, 1.12)	6.57E-01	1.04 (0.89, 1.21)	8.73E-01	0.98 (0.85, 1.13)
1.04 (0.96, 1.12)	6.72E-01	1.02 (0.87, 1.19)	8.96E-01	0.97 (0.84, 1.12)
1.03 (0.95, 1.12)	8.06E-01	1.17 (1.00, 1.37)	3.93E-01	1.05 (0.90, 1.21)
0.96 (0.89, 1.04)	5.89E-01	0.92 (0.79, 1.08)	8.52E-01	1.03 (0.89, 1.19)
1.10 (1.01, 1.21)	1.36E-01	1.00 (0.85, 1.18)	9.77E-01	0.95 (0.82, 1.09)
0.97 (0.90, 1.04)	7.23E-01	0.91 (0.78, 1.05)	7.19E-01	1.00 (0.87, 1.15)
0.96 (0.89, 1.03)	5.87E-01	0.92 (0.79, 1.07)	8.33E-01	1.00 (0.87, 1.16)
1.01 (0.93, 1.10)	9.41E-01	0.92 (0.79, 1.07)	8.46E-01	0.99 (0.86, 1.14)
0.99 (0.91, 1.07)	9.34E-01	1.08 (0.92, 1.26)	8.52E-01	1.03 (0.89, 1.18)
1.00 (0.92, 1.08)	9.82E-01	0.97 (0.82, 1.15)	8.96E-01	0.92 (0.79, 1.06)
0.99 (0.92, 1.07)	9.60E-01	0.93 (0.81, 1.06)	8.08E-01	0.88 (0.78, 1.00)
0.94 (0.87, 1.02)	4.15E-01	0.91 (0.79, 1.05)	7.19E-01	0.84 (0.74, 0.96)
1.06 (0.97, 1.16)	4.99E-01	0.97 (0.82, 1.15)	8.96E-01	0.99 (0.86, 1.14)
1.01 (0.93, 1.09)	9.54E-01	1.10 (0.95, 1.27)	7.19E-01	1.16 (1.02, 1.31)
0.99 (0.91, 1.08)	9.54E-01	1.06 (0.89, 1.26)	8.52E-01	1.02 (0.88, 1.19)
0.98 (0.91, 1.06)	8.86E-01	0.91 (0.78, 1.07)	8.08E-01	0.89 (0.79, 1.01)
0.95 (0.88, 1.03)	5.64E-01	0.91 (0.78, 1.07)	8.08E-01	0.87 (0.77, 0.98)

1.09 (1.00, 1.19)	2.58E-01	0.99 (0.83, 1.19)	9.69E-01	1.04 (0.89, 1.22)
1.03 (0.95, 1.11)	7.82E-01	1.11 (0.95, 1.30)	7.19E-01	1.17 (1.05, 1.32)
1.00 (0.92, 1.09)	9.91E-01	1.08 (0.92, 1.27)	8.52E-01	1.09 (0.95, 1.25)
0.98 (0.90, 1.06)	8.41E-01	0.91 (0.78, 1.06)	7.68E-01	0.87 (0.77, 1.00)
0.98 (0.90, 1.06)	8.23E-01	0.91 (0.78, 1.07)	8.08E-01	0.88 (0.77, 1.00)
1.03 (0.94, 1.13)	8.06E-01	1.06 (0.90, 1.25)	8.52E-01	1.11 (0.96, 1.29)
1.05 (0.97, 1.14)	5.17E-01	1.14 (0.97, 1.33)	6.04E-01	1.23 (1.09, 1.40)
1.03 (0.95, 1.12)	8.06E-01	1.09 (0.93, 1.27)	8.52E-01	1.10 (0.96, 1.26)
0.97 (0.90, 1.05)	8.01E-01	0.92 (0.79, 1.08)	8.52E-01	0.89 (0.77, 1.01)
0.97 (0.89, 1.05)	8.01E-01	0.93 (0.79, 1.09)	8.52E-01	0.89 (0.77, 1.02)
1.03 (0.94, 1.12)	8.32E-01	1.04 (0.88, 1.23)	8.73E-01	1.08 (0.93, 1.26)
1.03 (0.95, 1.11)	8.23E-01	1.07 (0.90, 1.26)	8.52E-01	1.17 (1.03, 1.33)
1.20 (1.11, 1.30)	**1.30E-04**	1.14 (0.96, 1.36)	6.06E-01	1.08 (0.93, 1.25)
0.84 (0.78, 0.90)	**7.24E-05**	0.87 (0.74, 1.03)	6.04E-01	0.88 (0.77, 1.02)
0.81 (0.75, 0.87)	**4.45E-06**	0.84 (0.71, 0.99)	3.67E-01	0.89 (0.77, 1.03)
1.00 (0.93, 1.07)	9.82E-01	1.11 (0.93, 1.33)	7.81E-01	0.91 (0.81, 1.02)
0.95 (0.88, 1.03)	5.50E-01	0.95 (0.80, 1.13)	8.57E-01	1.10 (0.97, 1.25)
0.87 (0.80, 0.95)	**1.22E-02**	0.96 (0.81, 1.14)	8.73E-01	0.76 (0.65, 0.90)
1.10 (1.02, 1.20)	7.95E-02	1.04 (0.89, 1.22)	8.73E-01	1.12 (0.96, 1.31)
1.11 (1.02, 1.21)	5.61E-02	1.06 (0.90, 1.26)	8.52E-01	1.17 (1.00, 1.37)
1.07 (0.99, 1.16)	2.86E-01	1.01 (0.86, 1.19)	9.28E-01	1.04 (0.90, 1.20)
1.01 (0.94, 1.09)	9.22E-01	0.98 (0.83, 1.16)	8.96E-01	1.17 (1.03, 1.33)
0.99 (0.91, 1.09)	9.76E-01	1.07 (0.89, 1.28)	8.52E-01	1.23 (1.11, 1.35)
0.99 (0.92, 1.08)	9.76E-01	0.97 (0.81, 1.15)	8.95E-01	0.86 (0.76, 0.97)
0.99 (0.91, 1.08)	9.54E-01	0.92 (0.77, 1.09)	8.52E-01	0.86 (0.76, 0.96)
1.01 (0.93, 1.09)	9.54E-01	1.12 (0.94, 1.33)	7.19E-01	0.93 (0.81, 1.08)
1.01 (0.93, 1.09)	9.41E-01	1.00 (0.85, 1.18)	9.85E-01	1.05 (0.91, 1.21)
0.96 (0.88, 1.04)	6.12E-01	1.03 (0.86, 1.22)	8.96E-01	1.05 (0.91, 1.22)
1.04 (0.95, 1.13)	6.97E-01	0.98 (0.83, 1.17)	9.12E-01	0.93 (0.81, 1.07)
1.03 (0.95, 1.12)	7.60E-01	0.98 (0.82, 1.16)	8.96E-01	0.93 (0.81, 1.08)
1.00 (0.92, 1.09)	9.99E-01	1.07 (0.91, 1.27)	8.52E-01	1.04 (0.89, 1.20)
0.99 (0.92, 1.08)	9.76E-01	0.97 (0.82, 1.16)	8.96E-01	1.10 (0.95, 1.26)
0.88 (0.81, 0.96)	**2.87E-02**	0.89 (0.75, 1.04)	6.43E-01	0.98 (0.85, 1.14)
1.19 (1.10, 1.30)	**4.42E-04**	1.17 (0.99, 1.38)	4.31E-01	1.28 (1.11, 1.47)
1.12 (1.04, 1.21)	**2.87E-02**	1.10 (0.94, 1.28)	8.08E-01	1.16 (1.01, 1.34)
1.00 (0.93, 1.08)	9.82E-01	0.98 (0.85, 1.14)	9.02E-01	0.93 (0.80, 1.08)
0.96 (0.89, 1.04)	6.53E-01	0.92 (0.78, 1.08)	8.52E-01	0.98 (0.84, 1.13)
0.98 (0.90, 1.06)	8.23E-01	0.93 (0.80, 1.09)	8.52E-01	0.95 (0.82, 1.11)
0.97 (0.90, 1.06)	8.23E-01	0.93 (0.79, 1.09)	8.52E-01	0.90 (0.77, 1.05)
1.08 (1.00, 1.17)	1.87E-01	1.17 (1.00, 1.36)	4.11E-01	1.01 (0.88, 1.17)
1.09 (1.01, 1.17)	1.45E-01	1.17 (1.01, 1.37)	3.67E-01	1.01 (0.87, 1.17)
1.03 (0.95, 1.11)	8.05E-01	1.07 (0.91, 1.24)	8.52E-01	1.05 (0.91, 1.21)
1.00 (0.92, 1.09)	9.99E-01	0.98 (0.84, 1.14)	8.96E-01	0.91 (0.78, 1.06)
1.00 (0.92, 1.09)	9.99E-01	0.95 (0.81, 1.12)	8.57E-01	0.95 (0.81, 1.11)
0.95 (0.88, 1.03)	5.35E-01	0.93 (0.78, 1.11)	8.52E-01	1.03 (0.89, 1.19)
0.94 (0.86, 1.02)	4.05E-01	0.91 (0.76, 1.09)	8.52E-01	1.00 (0.87, 1.16)
1.02 (0.94, 1.10)	8.68E-01	1.04 (0.89, 1.22)	8.73E-01	1.12 (0.97, 1.28)
1.03 (0.95, 1.11)	8.06E-01	1.05 (0.89, 1.23)	8.57E-01	1.13 (0.98, 1.31)

1.04 (0.97, 1.13)	5.89E-01	1.06 (0.90, 1.25)	8.52E-01	1.02 (0.89, 1.19)
0.94 (0.87, 1.02)	4.83E-01	0.93 (0.78, 1.11)	8.52E-01	0.98 (0.84, 1.13)
1.02 (0.94, 1.11)	8.68E-01	1.02 (0.87, 1.21)	8.96E-01	0.98 (0.84, 1.14)
0.96 (0.88, 1.04)	6.04E-01	0.95 (0.80, 1.12)	8.53E-01	0.95 (0.81, 1.11)
1.05 (0.97, 1.13)	5.89E-01	1.05 (0.89, 1.25)	8.57E-01	1.05 (0.91, 1.22)
1.06 (0.98, 1.14)	4.54E-01	1.12 (0.96, 1.30)	6.80E-01	1.00 (0.87, 1.16)
0.97 (0.89, 1.05)	7.13E-01	0.91 (0.78, 1.07)	8.08E-01	0.94 (0.80, 1.09)
0.94 (0.86, 1.02)	3.81E-01	0.88 (0.75, 1.04)	6.05E-01	0.95 (0.82, 1.11)
1.00 (0.92, 1.09)	9.99E-01	0.98 (0.82, 1.16)	8.96E-01	0.96 (0.83, 1.12)
1.00 (0.92, 1.09)	9.91E-01	0.94 (0.78, 1.14)	8.57E-01	0.81 (0.69, 0.94)
0.90 (0.83, 0.97)	**3.62E-02**	0.88 (0.74, 1.04)	6.05E-01	0.90 (0.78, 1.03)
1.01 (0.93, 1.10)	9.54E-01	0.94 (0.78, 1.13)	8.52E-01	0.94 (0.80, 1.10)
0.91 (0.84, 0.99)	1.11E-01	0.86 (0.71, 1.03)	6.04E-01	0.88 (0.75, 1.02)
1.02 (0.94, 1.11)	8.85E-01	0.96 (0.81, 1.15)	8.78E-01	0.93 (0.79, 1.09)
1.00 (0.92, 1.08)	9.89E-01	0.94 (0.79, 1.12)	8.52E-01	0.91 (0.78, 1.07)
0.98 (0.90, 1.06)	8.23E-01	0.97 (0.82, 1.15)	8.96E-01	0.99 (0.85, 1.15)
1.03 (0.95, 1.12)	7.82E-01	1.02 (0.86, 1.21)	8.96E-01	0.99 (0.85, 1.15)
0.84 (0.78, 0.91)	**4.79E-04**	0.84 (0.71, 0.99)	3.47E-01	0.92 (0.79, 1.06)
1.02 (0.93, 1.11)	8.72E-01	0.96 (0.78, 1.17)	8.74E-01	0.93 (0.80, 1.08)
0.82 (0.75, 0.89)	**3.23E-05**	0.78 (0.66, 0.93)	1.41E-01	0.85 (0.73, 0.98)
1.07 (0.98, 1.17)	4.05E-01	1.00 (0.83, 1.19)	9.81E-01	0.91 (0.78, 1.06)
1.00 (0.92, 1.09)	9.99E-01	0.92 (0.77, 1.10)	8.52E-01	0.85 (0.73, 0.99)
0.89 (0.82, 0.97)	**3.73E-02**	0.96 (0.82, 1.13)	8.73E-01	1.08 (0.93, 1.25)
1.17 (1.08, 1.28)	**2.68E-03**	1.20 (1.02, 1.41)	3.17E-01	1.16 (0.99, 1.35)
1.00 (0.93, 1.09)	9.82E-01	0.90 (0.75, 1.08)	8.08E-01	1.23 (1.12, 1.34)
0.77 (0.70, 0.85)	**8.97E-06**	0.79 (0.67, 0.94)	2.25E-01	0.84 (0.71, 0.99)
1.27 (1.17, 1.38)	**2.93E-06**	1.28 (1.08, 1.52)	1.44E-01	1.06 (0.91, 1.24)
0.80 (0.74, 0.88)	**2.72E-05**	0.77 (0.66, 0.91)	1.04E-01	0.89 (0.76, 1.03)
0.80 (0.73, 0.87)	**1.97E-05**	0.78 (0.67, 0.91)	9.50E-02	0.86 (0.74, 1.01)
0.74 (0.68, 0.81)	**2.63E-08**	0.74 (0.62, 0.87)	**3.35E-02**	0.93 (0.80, 1.09)
0.89 (0.82, 0.97)	**4.38E-02**	0.88 (0.73, 1.05)	6.80E-01	0.92 (0.79, 1.07)
0.83 (0.76, 0.91)	**5.45E-04**	0.85 (0.71, 1.02)	5.95E-01	0.90 (0.77, 1.05)
0.82 (0.75, 0.90)	**4.46E-04**	0.82 (0.69, 0.98)	3.20E-01	0.87 (0.74, 1.02)
0.88 (0.81, 0.97)	**3.77E-02**	0.74 (0.60, 0.90)	1.17E-01	1.20 (1.09, 1.32)
0.88 (0.81, 0.96)	**3.62E-02**	0.82 (0.70, 0.96)	3.07E-01	1.19 (1.03, 1.37)
0.62 (0.44, 0.87)	**3.62E-02**	0.99 (0.84, 1.16)	9.28E-01	0.92 (0.73, 1.16)
1.10 (1.02, 1.18)	6.34E-02	1.26 (1.14, 1.40)	**1.86E-03**	1.07 (0.93, 1.23)
1.04 (0.96, 1.12)	6.10E-01	1.23 (1.11, 1.37)	**7.61E-03**	1.07 (0.94, 1.21)
0.75 (0.62, 0.91)	**2.70E-02**	0.79 (0.65, 0.97)	3.17E-01	1.09 (1.04, 1.13)
1.08 (0.99, 1.17)	3.06E-01	1.12 (0.96, 1.31)	6.80E-01	0.82 (0.72, 0.95)
1.02 (0.94, 1.11)	8.95E-01	0.93 (0.77, 1.13)	8.52E-01	1.05 (0.91, 1.22)

			UKB	
Other mortality			All caus	
nker	Current drinker		Never/former drinker	
P value	Adjusted HR (95% CI	P value	Adjusted HR (95% CI)	P value
8.81E-01	0.88 (0.59, 1.30)	9.75E-01	0.94 (0.90, 0.98)	**7.60E-03**
8.81E-01	0.88 (0.60, 1.30)	9.75E-01	0.94 (0.90, 0.98)	**6.68E-03**
8.55E-01	0.89 (0.60, 1.31)	9.75E-01	0.94 (0.90, 0.98)	**5.20E-03**
7.60E-01	0.93 (0.64, 1.36)	9.75E-01	0.93 (0.89, 0.97)	**7.70E-04**
7.71E-01	0.95 (0.65, 1.38)	9.75E-01	0.92 (0.88, 0.96)	**3.69E-04**
7.50E-01	0.92 (0.63, 1.34)	9.75E-01	0.93 (0.89, 0.97)	**1.97E-03**
8.85E-01	0.87 (0.59, 1.29)	9.75E-01	0.95 (0.91, 0.99)	**1.49E-02**
8.81E-01	0.89 (0.60, 1.32)	9.75E-01	0.91 (0.87, 0.95)	**2.42E-05**
8.81E-01	0.89 (0.61, 1.32)	9.75E-01	0.91 (0.87, 0.95)	**2.02E-05**
8.06E-01	0.91 (0.62, 1.33)	9.75E-01	0.91 (0.87, 0.95)	**1.73E-05**
8.81E-01	0.92 (0.63, 1.34)	9.75E-01	0.88 (0.84, 0.92)	**4.24E-08**
8.85E-01	0.92 (0.63, 1.34)	9.75E-01	0.87 (0.83, 0.91)	**8.95E-10**
8.06E-01	0.92 (0.63, 1.35)	9.75E-01	0.90 (0.86, 0.94)	**3.90E-06**
8.81E-01	0.88 (0.60, 1.31)	9.75E-01	0.92 (0.88, 0.96)	**2.23E-04**
9.27E-01	0.93 (0.64, 1.37)	9.75E-01	0.89 (0.85, 0.93)	**9.55E-07**
9.27E-01	0.93 (0.64, 1.37)	9.75E-01	0.89 (0.85, 0.93)	**1.76E-07**
9.20E-01	0.94 (0.64, 1.37)	9.75E-01	0.89 (0.86, 0.93)	**1.27E-06**
9.86E-01	0.94 (0.65, 1.37)	9.75E-01	0.88 (0.84, 0.92)	**9.53E-09**
8.97E-01	0.94 (0.65, 1.37)	9.75E-01	0.87 (0.83, 0.91)	**2.67E-09**
8.81E-01	0.94 (0.65, 1.37)	9.75E-01	0.88 (0.85, 0.92)	**8.02E-08**
9.27E-01	0.93 (0.64, 1.36)	9.75E-01	0.89 (0.85, 0.93)	**7.11E-07**
9.23E-01	0.85 (0.58, 1.24)	9.75E-01	0.86 (0.82, 0.89)	**1.18E-11**
9.23E-01	0.85 (0.58, 1.24)	9.75E-01	0.85 (0.82, 0.89)	**9.42E-12**
9.16E-01	0.85 (0.58, 1.24)	9.75E-01	0.86 (0.82, 0.90)	**2.39E-11**
9.09E-01	0.86 (0.59, 1.25)	9.75E-01	0.87 (0.83, 0.90)	**2.22E-10**
8.61E-01	0.87 (0.60, 1.26)	9.75E-01	0.88 (0.84, 0.91)	**4.06E-09**
9.86E-01	0.86 (0.59, 1.26)	9.75E-01	0.86 (0.82, 0.90)	**6.44E-11**
9.27E-01	0.84 (0.57, 1.24)	9.75E-01	0.88 (0.84, 0.91)	**9.32E-09**
8.06E-01	0.86 (0.59, 1.24)	9.75E-01	0.90 (0.86, 0.94)	**2.19E-06**
8.06E-01	0.86 (0.60, 1.25)	9.75E-01	0.89 (0.85, 0.93)	**2.51E-07**
7.18E-01	0.88 (0.61, 1.26)	9.75E-01	0.87 (0.84, 0.91)	**3.25E-09**
6.39E-01	0.89 (0.62, 1.28)	9.75E-01	0.88 (0.84, 0.92)	**2.04E-08**
5.88E-01	0.90 (0.63, 1.29)	9.75E-01	0.89 (0.85, 0.93)	**1.93E-07**
8.07E-01	0.89 (0.62, 1.28)	9.75E-01	0.87 (0.83, 0.91)	**6.06E-10**
8.93E-01	0.85 (0.59, 1.25)	9.75E-01	0.93 (0.89, 0.97)	**8.92E-04**
8.81E-01	0.99 (0.70, 1.39)	9.88E-01	0.95 (0.91, 0.99)	**2.94E-02**
8.81E-01	1.00 (0.71, 1.40)	9.88E-01	0.95 (0.91, 1.00)	**4.30E-02**
8.55E-01	0.98 (0.69, 1.38)	9.88E-01	0.97 (0.93, 1.02)	2.68E-01
8.81E-01	1.05 (0.75, 1.46)	9.81E-01	0.94 (0.90, 0.98)	**4.25E-03**
8.85E-01	1.07 (0.77, 1.49)	9.75E-01	0.93 (0.89, 0.97)	**1.28E-03**

8.81E-01	1.00 (0.71, 1.40)	9.88E-01	0.96 (0.92, 1.00)	6.09E-02
9.95E-01	0.93 (0.65, 1.33)	9.75E-01	0.99 (0.95, 1.03)	6.67E-01
8.55E-01	1.02 (0.73, 1.43)	9.88E-01	0.88 (0.85, 0.92)	**5.00E-08**
8.06E-01	1.02 (0.72, 1.43)	9.88E-01	0.89 (0.85, 0.92)	**1.01E-07**
6.39E-01	1.01 (0.71, 1.43)	9.88E-01	0.90 (0.86, 0.94)	**3.33E-06**
6.43E-01	1.00 (0.71, 1.41)	9.88E-01	0.88 (0.84, 0.92)	**8.21E-09**
6.15E-01	0.99 (0.71, 1.40)	9.88E-01	0.87 (0.83, 0.91)	**8.95E-10**
8.06E-01	1.02 (0.72, 1.44)	9.88E-01	0.90 (0.86, 0.94)	**3.64E-06**
3.93E-01	1.07 (0.77, 1.49)	9.75E-01	1.00 (0.96, 1.05)	9.11E-01
6.31E-01	0.95 (0.66, 1.35)	9.75E-01	0.87 (0.83, 0.90)	**2.77E-10**
5.88E-01	0.95 (0.66, 1.35)	9.75E-01	0.85 (0.81, 0.89)	**5.63E-13**
3.93E-01	0.94 (0.66, 1.35)	9.75E-01	0.85 (0.81, 0.88)	**3.50E-13**
5.04E-01	0.93 (0.65, 1.33)	9.75E-01	0.84 (0.81, 0.88)	**9.82E-14**
4.70E-01	0.92 (0.64, 1.31)	9.75E-01	0.84 (0.80, 0.88)	**2.53E-14**
6.28E-01	0.97 (0.68, 1.39)	9.88E-01	0.86 (0.82, 0.89)	**1.06E-11**
3.14E-01	1.11 (0.80, 1.53)	9.75E-01	0.99 (0.95, 1.04)	8.01E-01
3.85E-01	0.85 (0.60, 1.21)	9.75E-01	0.87 (0.83, 0.90)	**1.60E-10**
3.54E-01	0.85 (0.60, 1.21)	9.75E-01	0.84 (0.80, 0.87)	**4.31E-15**
2.71E-01	0.82 (0.59, 1.13)	9.75E-01	0.83 (0.80, 0.87)	**1.28E-15**
3.14E-01	0.86 (0.60, 1.24)	9.75E-01	0.83 (0.80, 0.87)	**1.24E-15**
3.33E-01	0.87 (0.61, 1.25)	9.75E-01	0.83 (0.80, 0.87)	**2.25E-15**
2.71E-01	0.84 (0.60, 1.17)	9.75E-01	0.84 (0.80, 0.87)	**6.10E-15**
4.13E-01	0.97 (0.67, 1.39)	9.88E-01	0.97 (0.93, 1.01)	1.63E-01
3.00E-01	0.84 (0.59, 1.19)	9.75E-01	0.87 (0.84, 0.91)	**2.14E-09**
2.87E-01	0.84 (0.59, 1.19)	9.75E-01	0.86 (0.82, 0.89)	**3.98E-12**
1.85E-01	0.82 (0.60, 1.11)	9.75E-01	0.88 (0.84, 0.92)	**8.21E-09**
2.97E-01	0.86 (0.60, 1.24)	9.75E-01	0.84 (0.81, 0.88)	**6.23E-14**
3.00E-01	0.87 (0.61, 1.25)	9.75E-01	0.84 (0.81, 0.88)	**3.45E-14**
2.08E-01	0.84 (0.60, 1.18)	9.75E-01	0.87 (0.83, 0.90)	**6.44E-11**
8.61E-01	0.87 (0.61, 1.24)	9.75E-01	0.95 (0.91, 0.99)	**1.94E-02**
2.25E-01	1.21 (0.89, 1.64)	9.75E-01	1.11 (1.06, 1.16)	**4.84E-06**
2.42E-01	1.21 (0.89, 1.64)	9.75E-01	1.13 (1.08, 1.18)	**6.46E-08**
1.92E-01	1.23 (0.90, 1.68)	9.75E-01	1.14 (1.09, 1.19)	**3.44E-09**
4.28E-01	1.17 (0.86, 1.59)	9.75E-01	1.11 (1.07, 1.16)	**2.35E-06**
4.76E-01	1.16 (0.85, 1.59)	9.75E-01	1.10 (1.06, 1.15)	**1.76E-05**
3.33E-01	1.18 (0.87, 1.61)	9.75E-01	1.14 (1.09, 1.18)	**2.26E-09**
**4.66E-02**	1.08 (0.78, 1.49)	9.75E-01	1.01 (0.97, 1.06)	5.42E-01
1.53E-01	1.21 (0.88, 1.66)	9.75E-01	1.08 (1.03, 1.13)	**1.71E-03**
1.53E-01	1.21 (0.88, 1.66)	9.75E-01	1.09 (1.04, 1.14)	**7.18E-04**
2.25E-01	1.20 (0.87, 1.65)	9.75E-01	1.09 (1.04, 1.14)	**7.89E-04**
1.53E-01	1.21 (0.88, 1.66)	9.75E-01	1.08 (1.04, 1.14)	**8.20E-04**
1.53E-01	1.21 (0.88, 1.66)	9.75E-01	1.08 (1.03, 1.13)	**1.25E-03**
1.85E-01	1.20 (0.87, 1.65)	9.75E-01	1.09 (1.05, 1.15)	**1.80E-04**
**5.59E-03**	1.22 (0.89, 1.67)	9.75E-01	1.03 (0.98, 1.07)	2.81E-01
3.00E-01	1.03 (0.74, 1.44)	9.88E-01	0.92 (0.88, 0.96)	**1.00E-03**
3.00E-01	1.03 (0.74, 1.44)	9.88E-01	0.92 (0.88, 0.96)	**9.19E-04**
5.53E-01	1.07 (0.77, 1.49)	9.75E-01	0.92 (0.88, 0.96)	**8.92E-04**
2.08E-01	1.01 (0.72, 1.41)	9.88E-01	0.93 (0.88, 0.97)	**3.40E-03**

1.66E-01	1.00 (0.71, 1.40)	9.88E-01	0.93 (0.88, 0.97)	**2.85E-03**
4.37E-01	1.05 (0.75, 1.47)	9.75E-01	0.93 (0.89, 0.98)	**9.90E-03**
7.71E-01	0.95 (0.68, 1.33)	9.75E-01	0.95 (0.91, 0.99)	**2.61E-02**
**5.32E-03**	0.93 (0.67, 1.30)	9.75E-01	0.79 (0.76, 0.83)	**7.17E-24**
**5.25E-03**	0.93 (0.67, 1.30)	9.75E-01	0.82 (0.78, 0.85)	**3.91E-18**
1.53E-01	0.98 (0.69, 1.38)	9.88E-01	0.83 (0.79, 0.87)	**2.79E-15**
**2.57E-02**	0.93 (0.67, 1.29)	9.75E-01	0.80 (0.77, 0.83)	**2.00E-22**
8.99E-02	0.94 (0.67, 1.30)	9.75E-01	0.80 (0.76, 0.83)	**7.17E-24**
8.99E-02	0.98 (0.69, 1.38)	9.88E-01	0.84 (0.81, 0.88)	**2.87E-13**
9.27E-01	0.96 (0.68, 1.35)	9.82E-01	0.93 (0.89, 0.98)	**3.14E-03**
8.81E-01	1.35 (0.86, 2.10)	9.75E-01	1.04 (0.99, 1.10)	1.13E-01
3.00E-01	1.15 (0.89, 1.50)	9.75E-01	1.02 (0.97, 1.06)	5.42E-01
5.05E-01	1.11 (0.84, 1.48)	9.75E-01	1.00 (0.96, 1.05)	9.42E-01
3.00E-01	1.18 (0.90, 1.54)	9.75E-01	1.04 (0.99, 1.08)	1.06E-01
3.10E-01	0.82 (0.63, 1.08)	9.75E-01	0.98 (0.93, 1.02)	3.16E-01
9.27E-01	1.57 (0.95, 2.60)	9.75E-01	1.04 (1.00, 1.09)	8.77E-02
7.49E-01	1.17 (0.91, 1.50)	9.75E-01	1.03 (0.98, 1.08)	2.23E-01
8.81E-01	1.01 (0.73, 1.41)	9.88E-01	1.07 (1.03, 1.12)	**1.90E-03**
7.10E-01	1.26 (1.00, 1.58)	9.75E-01	0.96 (0.91, 1.00)	5.23E-02
8.55E-01	0.73 (0.54, 0.99)	9.75E-01	0.99 (0.94, 1.03)	6.02E-01
7.14E-01	1.12 (0.77, 1.64)	9.75E-01	1.07 (1.03, 1.11)	**2.72E-03**
9.03E-01	0.97 (0.69, 1.37)	9.88E-01	1.07 (1.03, 1.12)	**2.77E-03**
9.27E-01	0.82 (0.58, 1.17)	9.75E-01	1.05 (1.01, 1.10)	**3.69E-02**
8.61E-01	1.15 (0.78, 1.68)	9.75E-01	0.95 (0.91, 1.00)	**4.23E-02**
8.81E-01	1.00 (0.70, 1.42)	9.88E-01	0.99 (0.95, 1.03)	6.93E-01
8.61E-01	0.89 (0.62, 1.26)	9.75E-01	1.00 (0.96, 1.05)	9.15E-01
6.77E-01	0.92 (0.65, 1.30)	9.75E-01	1.00 (0.96, 1.05)	9.44E-01
8.61E-01	0.91 (0.64, 1.30)	9.75E-01	1.01 (0.96, 1.05)	8.20E-01
5.38E-01	1.02 (0.73, 1.40)	9.88E-01	1.00 (0.96, 1.04)	1.00E+00
7.60E-01	1.11 (0.78, 1.57)	9.75E-01	0.94 (0.90, 0.98)	**5.88E-03**
8.61E-01	1.14 (0.84, 1.53)	9.75E-01	0.96 (0.92, 1.01)	1.06E-01
8.81E-01	0.96 (0.70, 1.33)	9.88E-01	0.99 (0.95, 1.03)	5.42E-01
8.61E-01	0.94 (0.68, 1.30)	9.75E-01	0.94 (0.90, 0.98)	**5.68E-03**
8.06E-01	1.15 (0.83, 1.58)	9.75E-01	1.05 (1.00, 1.09)	**4.80E-02**
8.61E-01	1.00 (0.72, 1.38)	9.88E-01	1.18 (1.13, 1.24)	**1.93E-13**
7.64E-01	0.95 (0.70, 1.30)	9.75E-01	0.94 (0.90, 0.98)	**3.35E-03**
9.89E-01	1.18 (0.83, 1.68)	9.75E-01	0.93 (0.89, 0.97)	**9.08E-04**
9.90E-01	1.21 (0.87, 1.68)	9.75E-01	1.01 (0.97, 1.05)	8.03E-01
9.27E-01	1.01 (0.74, 1.39)	9.88E-01	1.04 (1.00, 1.08)	8.20E-02
8.81E-01	0.91 (0.64, 1.3)	9.75E-01	1.15 (1.10, 1.20)	**1.45E-10**
5.60E-01	1.01 (0.73, 1.4)	9.88E-01	0.88 (0.85, 0.92)	**5.00E-10**
2.42E-01	0.96 (0.72, 1.28)	9.75E-01	0.85 (0.82, 0.88)	**1.22E-15**
1.40E-01	0.93 (0.7, 1.24)	9.75E-01	1.02 (0.98, 1.06)	4.28E-01
9.27E-01	1.02 (0.73, 1.42)	9.88E-01	1.11 (1.07, 1.15)	**2.51E-07**
1.66E-01	1.05 (0.77, 1.43)	9.75E-01	1.02 (0.99, 1.06)	2.47E-01
8.89E-01	1.21 (0.93, 1.56)	9.75E-01	0.93 (0.91, 0.95)	**8.35E-10**
2.71E-01	0.83 (0.65, 1.07)	9.75E-01	0.93 (0.92, 0.95)	**1.29E-13**
1.85E-01	0.81 (0.65, 1.02)	9.75E-01	0.97 (0.93, 1.01)	1.67E-01

8.61E-01	1.17 (0.80, 1.70)	9.75E-01	1.10 (1.07, 1.13)	**1.22E-10**
8.99E-02	1.18 (0.91, 1.53)	9.75E-01	1.02 (0.98, 1.06)	3.51E-01
5.34E-01	1.05 (0.75, 1.47)	9.75E-01	0.94 (0.92, 0.96)	**1.10E-09**
2.25E-01	0.94 (0.67, 1.31)	9.75E-01	0.92 (0.90, 0.94)	**5.50E-10**
2.42E-01	0.92 (0.66, 1.28)	9.75E-01	0.99 (0.95, 1.03)	6.50E-01
4.28E-01	1.14 (0.82, 1.59)	9.75E-01	1.07 (1.05, 1.10)	**3.56E-11**
**1.93E-02**	1.08 (0.78, 1.51)	9.75E-01	1.11 (1.08, 1.14)	**1.11E-13**
4.69E-01	1.06 (0.75, 1.48)	9.75E-01	0.92 (0.90, 0.94)	**2.25E-15**
3.00E-01	0.97 (0.69, 1.36)	9.88E-01	0.90 (0.87, 0.92)	**6.10E-15**
3.10E-01	0.94 (0.67, 1.32)	9.75E-01	0.98 (0.94, 1.02)	4.02E-01
6.39E-01	1.16 (0.83, 1.63)	9.75E-01	1.05 (1.02, 1.09)	**7.26E-03**
1.53E-01	0.99 (0.69, 1.41)	9.88E-01	1.13 (1.08, 1.18)	**5.43E-07**
6.39E-01	1.21 (0.85, 1.72)	9.75E-01	0.89 (0.85, 0.93)	**2.75E-07**
3.00E-01	0.85 (0.61, 1.18)	9.75E-01	0.93 (0.89, 0.96)	**8.02E-05**
3.33E-01	0.82 (0.59, 1.15)	9.75E-01	0.94 (0.90, 0.98)	**8.63E-03**
3.56E-01	1.01 (0.72, 1.41)	9.88E-01	0.96 (0.92, 1.00)	5.04E-02
3.58E-01	0.90 (0.63, 1.29)	9.75E-01	0.99 (0.96, 1.03)	7.45E-01
**1.93E-02**	0.75 (0.51, 1.09)	9.75E-01	1.02 (0.98, 1.06)	4.18E-01
3.87E-01	1.25 (0.86, 1.82)	9.75E-01	1.01 (0.97, 1.05)	6.68E-01
2.39E-01	1.20 (0.83, 1.74)	9.75E-01	1.04 (1.00, 1.09)	**4.36E-02**
8.55E-01	1.27 (0.86, 1.87)	9.75E-01	0.98 (0.94, 1.02)	2.98E-01
1.53E-01	1.07 (0.77, 1.47)	9.75E-01	1.04 (0.99, 1.08)	1.06E-01
**5.25E-03**	1.21 (0.95, 1.53)	9.75E-01	0.98 (0.94, 1.02)	3.45E-01
1.40E-01	0.91 (0.66, 1.25)	9.75E-01	0.98 (0.94, 1.02)	4.28E-01
1.05E-01	0.87 (0.65, 1.16)	9.75E-01	0.97 (0.93, 1.02)	2.66E-01
6.39E-01	1.14 (0.79, 1.63)	9.75E-01	1.01 (0.97, 1.05)	6.78E-01
7.92E-01	0.93 (0.65, 1.32)	9.75E-01	1.11 (1.06, 1.15)	**1.04E-05**
7.72E-01	1.03 (0.73, 1.44)	9.88E-01	0.92 (0.88, 0.96)	**1.72E-04**
6.39E-01	0.98 (0.70, 1.38)	9.88E-01	0.90 (0.86, 0.93)	**4.56E-07**
6.39E-01	0.98 (0.70, 1.38)	9.88E-01	1.10 (1.05, 1.14)	**1.04E-05**
8.61E-01	1.04 (0.74, 1.46)	9.82E-01	1.04 (0.99, 1.08)	1.04E-01
4.94E-01	0.98 (0.69, 1.38)	9.88E-01	0.88 (0.84, 0.91)	**3.98E-09**
9.10E-01	0.94 (0.67, 1.32)	9.75E-01	0.96 (0.93, 1.01)	1.08E-01
**1.93E-02**	1.26 (0.93, 1.71)	9.75E-01	1.16 (1.11, 1.21)	**2.92E-10**
2.16E-01	1.23 (0.89, 1.70)	9.75E-01	0.85 (0.82, 0.89)	**9.98E-12**
6.39E-01	1.00 (0.71, 1.40)	9.88E-01	0.88 (0.84, 0.92)	**9.32E-09**
8.81E-01	0.89 (0.62, 1.28)	9.75E-01	0.84 (0.80, 0.88)	**1.30E-14**
8.06E-01	0.93 (0.66, 1.32)	9.75E-01	0.87 (0.83, 0.91)	**4.66E-10**
4.37E-01	0.95 (0.67, 1.34)	9.75E-01	0.85 (0.82, 0.89)	**6.02E-12**
9.23E-01	1.15 (0.83, 1.58)	9.75E-01	0.86 (0.82, 0.90)	**6.30E-11**
9.27E-01	1.14 (0.82, 1.58)	9.75E-01		
7.80E-01	1.17 (0.84, 1.63)	9.75E-01		
5.05E-01	1.01 (0.72, 1.43)	9.88E-01	0.92 (0.88, 0.96)	**3.69E-04**
7.80E-01	0.88 (0.61, 1.25)	9.75E-01	0.91 (0.87, 0.95)	**4.63E-05**
8.81E-01	0.89 (0.61, 1.29)	9.75E-01	0.98 (0.94, 1.02)	4.08E-01
9.86E-01	0.86 (0.59, 1.26)	9.75E-01	0.96 (0.92, 1.00)	1.02E-01
3.51E-01	1.08 (0.78, 1.50)	9.75E-01	0.88 (0.84, 0.92)	**1.39E-07**
3.10E-01	1.05 (0.75, 1.46)	9.75E-01	0.96 (0.92, 1.00)	6.12E-02

8.81E-01	0.79 (0.55, 1.14)	9.75E-01	0.88 (0.84, 0.92)	**1.01E-07**
8.85E-01	0.84 (0.58, 1.22)	9.75E-01	0.92 (0.88, 0.97)	**9.07E-04**
8.89E-01	0.77 (0.54, 1.09)	9.75E-01	0.87 (0.84, 0.92)	**3.36E-08**
8.06E-01	0.83 (0.58, 1.18)	9.75E-01	0.92 (0.87, 0.96)	**1.01E-03**
8.06E-01	0.79 (0.56, 1.12)	9.75E-01	0.87 (0.83, 0.91)	**3.88E-10**
9.73E-01	1.09 (0.78, 1.51)	9.75E-01	0.92 (0.88, 0.96)	**1.88E-04**
7.14E-01	0.88 (0.62, 1.26)	9.75E-01	0.89 (0.85, 0.93)	**6.74E-07**
8.06E-01	0.86 (0.60, 1.21)	9.75E-01	0.87 (0.84, 0.91)	**9.20E-09**
8.61E-01	0.74 (0.5, 1.09)	9.75E-01	0.87 (0.83, 0.91)	**2.21E-08**
8.99E-02	0.71 (0.53, 0.93)	9.75E-01	0.85 (0.82, 0.89)	**2.39E-11**
3.85E-01	0.83 (0.56, 1.24)	9.75E-01	0.85 (0.81, 0.89)	**3.28E-11**
7.46E-01	0.78 (0.55, 1.11)	9.75E-01	0.84 (0.81, 0.88)	**1.12E-12**
3.10E-01	0.75 (0.49, 1.15)	9.75E-01	0.82 (0.79, 0.86)	**3.29E-15**
6.39E-01	0.75 (0.52, 1.08)	9.75E-01	0.94 (0.90, 0.98)	**6.39E-03**
5.60E-01	0.73 (0.50, 1.07)	9.75E-01	0.92 (0.89, 0.97)	**7.19E-04**
9.27E-01	0.75 (0.51, 1.12)	9.75E-01	0.94 (0.90, 0.98)	**7.26E-03**
9.47E-01	0.76 (0.51, 1.12)	9.75E-01	0.93 (0.89, 0.97)	**1.25E-03**
5.31E-01	1.19 (0.84, 1.68)	9.75E-01	0.88 (0.84, 0.92)	**4.46E-07**
6.39E-01	0.83 (0.60, 1.13)	9.75E-01	0.98 (0.94, 1.02)	3.05E-01
1.85E-01	1.05 (0.73, 1.51)	9.85E-01	0.93 (0.89, 0.97)	**2.22E-03**
5.00E-01	0.84 (0.60, 1.18)	9.75E-01	1.05 (1.01, 1.10)	**3.12E-02**
2.25E-01	0.84 (0.60, 1.19)	9.75E-01	1.07 (1.02, 1.11)	**3.27E-03**
6.39E-01	1.03 (0.74, 1.44)	9.88E-01	1.15 (1.12, 1.19)	**6.69E-21**
2.71E-01	1.26 (0.92, 1.72)	9.75E-01	1.01 (0.97, 1.05)	7.22E-01
**1.86E-03**	1.28 (1.03, 1.59)	9.75E-01	1.07 (1.03, 1.12)	**1.55E-03**
2.25E-01	1.26 (0.96, 1.65)	9.75E-01	1.03 (0.99, 1.07)	2.12E-01
7.71E-01	1.15 (0.81, 1.63)	9.75E-01	0.96 (0.92, 1.00)	6.35E-02
3.56E-01	1.38 (1.00, 1.90)	9.75E-01	0.92 (0.88, 0.97)	**1.88E-03**
2.71E-01	0.96 (0.68, 1.36)	9.88E-01	0.94 (0.90, 0.98)	**1.12E-02**
6.52E-01	1.06 (0.76, 1.48)	9.75E-01	0.89 (0.85, 0.93)	**3.89E-07**
5.88E-01	0.98 (0.69, 1.4)	9.88E-01	0.88 (0.84, 0.92)	**3.42E-08**
4.44E-01	1.04 (0.74, 1.47)	9.88E-01	1.08 (1.04, 1.12)	**3.27E-04**
3.10E-01	1.10 (0.78, 1.56)	9.75E-01	1.04 (1.00, 1.08)	8.41E-02
**5.25E-03**	0.93 (0.65, 1.35)	9.75E-01	1.04 (1.01, 1.07)	**2.84E-02**
1.53E-01	1.12 (0.82, 1.53)	9.75E-01	1.05 (1.01, 1.09)	**1.54E-02**
7.71E-01	1.04 (0.77, 1.42)	9.81E-01	1.05 (1.01, 1.09)	**2.72E-02**
6.39E-01	0.91 (0.55, 1.52)	9.75E-01	1.07 (1.05, 1.09)	**8.21E-15**
6.39E-01	1.28 (1.06, 1.54)	9.75E-01	0.78 (0.74, 0.81)	**4.36E-30**
**5.25E-03**	1.17 (0.91, 1.50)	9.75E-01	1.09 (1.05, 1.14)	**1.03E-04**
8.99E-02	0.83 (0.64, 1.08)	9.75E-01	1.02 (0.98, 1.07)	3.53E-01
7.80E-01	0.72 (0.47, 1.11)	9.75E-01	0.89 (0.85, 0.93)	**5.63E-07**
			0.85 (0.81, 0.89)	**1.24E-12**
			0.85 (0.81, 0.88)	**2.15E-13**
			0.84 (0.80, 0.87)	**5.26E-15**
			0.97 (0.92, 1.02)	2.09E-01
			0.94 (0.90, 0.99)	**1.30E-02**
			1.12 (1.09, 1.16)	**9.82E-12**
			0.88 (0.84, 0.92)	**2.27E-08**

0.85 (0.82, 0.89)	**1.24E-12**
0.99 (0.95, 1.04)	8.03E-01
1.03 (1.00, 1.07)	8.84E-02
1.04 (1.00, 1.08)	8.68E-02
0.84 (0.80, 0.88)	**2.76E-12**
0.90 (0.86, 0.93)	**1.18E-06**
0.87 (0.83, 0.90)	**1.31E-10**
0.86 (0.82, 0.90)	**4.10E-10**
0.87 (0.83, 0.91)	**1.87E-09**
0.89 (0.85, 0.93)	**3.47E-07**
0.85 (0.81, 0.88)	**1.18E-13**
0.96 (0.92, 1.01)	1.45E-01
0.86 (0.82, 0.90)	**2.35E-11**
0.89 (0.85, 0.93)	**4.55E-07**
0.84 (0.81, 0.88)	**1.05E-13**
0.96 (0.92, 1.01)	1.53E-01
0.88 (0.84, 0.92)	**1.20E-08**
0.97 (0.93, 1.02)	2.98E-01
1.03 (0.98, 1.08)	3.26E-01

es mortality		Ischaemic heart d	
Current drinker		Never/former drinker	
Adjusted HR (95% CI)	P value	Adjusted HR (95% CI)	P value
0.99 (0.97, 1.00)	1.40E-01	1.04 (0.92, 1.17)	6.07E-01
0.99 (0.97, 1.00)	9.77E-02	1.04 (0.92, 1.17)	6.22E-01
0.99 (0.98, 1.01)	2.46E-01	1.03 (0.92, 1.17)	6.53E-01
0.98 (0.97, 1.00)	**3.42E-02**	0.99 (0.87, 1.12)	8.62E-01
0.98 (0.97, 1.00)	**1.13E-02**	0.96 (0.85, 1.10)	6.47E-01
0.99 (0.97, 1.00)	1.09E-01	1.01 (0.89, 1.14)	8.95E-01
0.99 (0.97, 1.00)	1.07E-01	1.05 (0.94, 1.18)	4.51E-01
0.95 (0.94, 0.97)	**2.85E-09**	0.99 (0.88, 1.13)	9.31E-01
0.95 (0.94, 0.97)	**4.90E-10**	0.99 (0.87, 1.12)	8.98E-01
0.96 (0.94, 0.97)	**6.03E-09**	0.97 (0.86, 1.11)	7.39E-01
0.94 (0.92, 0.95)	**2.82E-16**	0.89 (0.78, 1.02)	1.22E-01
0.93 (0.91, 0.94)	**1.27E-22**	0.84 (0.73, 0.96)	**1.81E-02**
0.95 (0.94, 0.97)	**3.45E-10**	0.95 (0.84, 1.08)	5.11E-01
0.96 (0.94, 0.97)	**3.35E-08**	1.03 (0.91, 1.16)	7.18E-01
0.94 (0.93, 0.95)	**2.60E-15**	0.96 (0.84, 1.09)	5.88E-01
0.93 (0.92, 0.94)	**2.99E-20**	0.95 (0.84, 1.08)	5.10E-01
0.94 (0.93, 0.96)	**1.45E-13**	0.96 (0.85, 1.09)	6.36E-01
0.93 (0.91, 0.94)	**1.13E-20**	0.89 (0.78, 1.02)	1.22E-01
0.93 (0.91, 0.94)	**1.31E-22**	0.85 (0.74, 0.97)	**2.71E-02**
0.93 (0.92, 0.95)	**5.64E-18**	0.94 (0.82, 1.07)	3.85E-01
0.93 (0.91, 0.94)	**6.70E-22**	0.98 (0.86, 1.11)	7.56E-01
0.91 (0.89, 0.92)	**1.16E-36**	0.78 (0.68, 0.89)	**6.65E-04**
0.90 (0.89, 0.92)	**1.10E-37**	0.81 (0.71, 0.93)	**4.34E-03**
0.91 (0.90, 0.92)	**8.62E-35**	0.77 (0.68, 0.89)	**5.02E-04**
0.91 (0.89, 0.92)	**6.09E-36**	0.71 (0.61, 0.81)	**5.36E-06**
0.91 (0.90, 0.93)	**1.60E-32**	0.69 (0.60, 0.79)	**1.47E-06**
0.91 (0.89, 0.92)	**1.16E-35**	0.75 (0.65, 0.86)	**1.15E-04**
0.92 (0.91, 0.93)	**2.31E-26**	0.93 (0.82, 1.06)	3.29E-01
0.95 (0.93, 0.96)	**6.35E-12**	0.91 (0.80, 1.04)	2.00E-01
0.94 (0.93, 0.96)	**2.04E-14**	0.89 (0.78, 1.01)	1.04E-01
0.92 (0.91, 0.94)	**3.88E-24**	0.81 (0.71, 0.93)	**4.57E-03**
0.93 (0.92, 0.95)	**3.31E-19**	0.80 (0.69, 0.91)	**2.17E-03**
0.94 (0.93, 0.96)	**1.31E-13**	0.82 (0.72, 0.94)	**7.35E-03**
0.91 (0.90, 0.93)	**1.79E-30**	0.76 (0.67, 0.87)	**2.42E-04**
0.97 (0.96, 0.99)	**1.26E-04**	1.03 (0.91, 1.16)	7.18E-01
1.00 (0.99, 1.02)	8.38E-01	0.84 (0.73, 0.96)	**1.85E-02**
1.01 (0.99, 1.02)	2.80E-01	0.84 (0.73, 0.97)	**2.54E-02**
1.02 (1.01, 1.04)	**2.00E-03**	0.88 (0.77, 1.01)	1.03E-01
0.98 (0.97, 1.00)	5.43E-02	0.75 (0.65, 0.87)	**2.32E-04**
0.98 (0.96, 0.99)	**3.95E-03**	0.73 (0.63, 0.84)	**4.79E-05**

1.00 (0.99, 1.02)	7.25E-01	0.82 (0.71, 0.94)	**9.69E-03**
1.04 (1.02, 1.05)	**6.81E-07**	1.08 (0.95, 1.22)	2.85E-01
0.93 (0.92, 0.95)	**2.38E-19**	0.72 (0.63, 0.83)	**1.84E-05**
0.93 (0.92, 0.95)	**9.10E-19**	0.69 (0.60, 0.79)	**1.87E-06**
0.95 (0.93, 0.96)	**1.64E-12**	0.70 (0.61, 0.80)	**3.72E-06**
0.92 (0.91, 0.93)	**1.34E-26**	0.67 (0.59, 0.77)	**5.95E-07**
0.92 (0.90, 0.93)	**2.22E-29**	0.67 (0.58, 0.77)	**4.54E-07**
0.93 (0.92, 0.95)	**1.39E-18**	0.69 (0.60, 0.79)	**1.79E-06**
1.06 (1.04, 1.07)	**2.90E-13**	1.04 (0.92, 1.18)	5.69E-01
0.91 (0.90, 0.92)	**2.82E-34**	0.74 (0.64, 0.84)	**3.59E-05**
0.89 (0.88, 0.91)	**6.35E-48**	0.70 (0.61, 0.80)	**1.62E-06**
0.89 (0.88, 0.90)	**7.64E-50**	0.69 (0.60, 0.79)	**8.24E-07**
0.89 (0.87, 0.90)	**9.55E-55**	0.69 (0.60, 0.79)	**8.21E-07**
0.88 (0.87, 0.90)	**4.95E-56**	0.69 (0.61, 0.79)	**1.33E-06**
0.89 (0.88, 0.91)	**1.52E-48**	0.67 (0.59, 0.77)	**3.42E-07**
1.04 (1.03, 1.06)	**1.62E-08**	1.03 (0.91, 1.17)	6.63E-01
0.90 (0.88, 0.91)	**1.62E-44**	0.74 (0.65, 0.85)	**3.91E-05**
0.88 (0.87, 0.89)	**7.19E-61**	0.74 (0.65, 0.84)	**2.23E-05**
0.88 (0.86, 0.89)	**1.18E-63**	0.74 (0.65, 0.84)	**2.65E-05**
0.87 (0.86, 0.89)	**4.47E-67**	0.72 (0.63, 0.82)	**6.32E-06**
0.88 (0.86, 0.89)	**1.52E-62**	0.73 (0.64, 0.84)	**1.88E-05**
0.87 (0.86, 0.88)	**1.37E-70**	0.70 (0.61, 0.80)	**1.47E-06**
1.01 (1.00, 1.03)	6.51E-02	1.03 (0.92, 1.17)	6.47E-01
0.91 (0.90, 0.92)	**3.90E-34**	0.77 (0.67, 0.88)	**2.59E-04**
0.89 (0.88, 0.91)	**1.42E-49**	0.76 (0.66, 0.86)	**9.68E-05**
0.91 (0.89, 0.92)	**5.27E-39**	0.79 (0.69, 0.90)	**6.91E-04**
0.88 (0.87, 0.90)	**1.68E-59**	0.73 (0.64, 0.83)	**1.21E-05**
0.89 (0.87, 0.90)	**2.45E-53**	0.74 (0.65, 0.84)	**2.60E-05**
0.88 (0.87, 0.89)	**1.40E-63**	0.73 (0.65, 0.84)	**1.21E-05**
1.00 (0.98, 1.01)	5.70E-01	1.03 (0.92, 1.16)	6.63E-01
1.09 (1.07, 1.10)	**3.80E-23**	0.84 (0.72, 0.99)	5.66E-02
1.10 (1.08, 1.12)	**4.81E-30**	0.87 (0.74, 1.02)	1.22E-01
1.11 (1.09, 1.13)	**1.49E-36**	0.90 (0.77, 1.05)	2.28E-01
1.08 (1.06, 1.10)	**2.99E-20**	0.84 (0.72, 0.98)	**4.72E-02**
1.07 (1.05, 1.09)	**3.04E-16**	0.83 (0.70, 0.97)	**3.16E-02**
1.10 (1.09, 1.12)	**2.72E-36**	0.90 (0.78, 1.04)	1.98E-01
1.05 (1.04, 1.07)	**5.35E-12**	1.02 (0.90, 1.15)	8.26E-01
1.06 (1.04, 1.08)	**8.53E-12**	0.79 (0.67, 0.94)	**1.62E-02**
1.07 (1.05, 1.09)	**4.35E-14**	0.81 (0.69, 0.97)	**3.07E-02**
1.07 (1.05, 1.09)	**1.61E-15**	0.83 (0.70, 0.99)	5.18E-02
1.06 (1.04, 1.08)	**8.95E-11**	0.80 (0.67, 0.95)	**1.85E-02**
1.05 (1.04, 1.07)	**2.42E-09**	0.80 (0.67, 0.95)	**1.62E-02**
1.07 (1.06, 1.09)	**2.25E-16**	0.82 (0.69, 0.97)	**3.21E-02**
1.08 (1.06, 1.09)	**3.22E-22**	0.99 (0.86, 1.13)	8.98E-01
0.98 (0.96, 1.00)	**1.83E-02**	0.75 (0.64, 0.87)	**5.59E-04**
0.98 (0.97, 1.00)	6.79E-02	0.78 (0.67, 0.90)	**2.39E-03**
0.99 (0.98, 1.01)	2.58E-01	0.81 (0.69, 0.94)	**8.85E-03**
0.97 (0.96, 0.99)	**2.75E-03**	0.74 (0.63, 0.87)	**4.68E-04**

0.97 (0.96, 0.99)	**8.03E-04**	0.75 (0.64, 0.87)	**5.02E-04**
0.99 (0.97, 1.00)	1.41E-01	0.74 (0.63, 0.87)	**5.07E-04**
1.02 (1.01, 1.04)	**2.13E-03**	1.01 (0.89, 1.16)	8.62E-01
0.87 (0.85, 0.88)	**5.44E-75**	0.74 (0.65, 0.85)	**2.65E-05**
0.90 (0.89, 0.92)	**6.69E-40**	0.79 (0.69, 0.90)	**9.78E-04**
0.92 (0.91, 0.94)	**1.03E-25**	0.80 (0.70, 0.91)	**2.33E-03**
0.87 (0.86, 0.88)	**1.73E-72**	0.74 (0.65, 0.85)	**2.65E-05**
0.86 (0.85, 0.87)	**7.42E-85**	0.75 (0.66, 0.85)	**2.43E-05**
0.92 (0.90, 0.93)	**1.47E-28**	0.77 (0.68, 0.89)	**4.94E-04**
0.99 (0.98, 1.01)	4.81E-01	1.06 (0.94, 1.21)	3.90E-01
1.04 (1.03, 1.06)	**1.75E-06**	1.19 (1.02, 1.39)	**4.61E-02**
1.02 (1.00, 1.03)	5.15E-02	0.85 (0.72, 1.01)	9.77E-02
1.01 (0.99, 1.03)	2.58E-01	0.78 (0.64, 0.95)	**2.58E-02**
1.03 (1.01, 1.04)	**1.63E-03**	0.99 (0.86, 1.14)	9.26E-01
0.97 (0.96, 0.99)	**1.66E-03**	1.08 (0.93, 1.26)	3.71E-01
1.01 (1.00, 1.03)	1.20E-01	0.75 (0.64, 0.89)	**2.57E-03**
1.00 (0.99, 1.02)	8.07E-01	0.74 (0.62, 0.89)	**2.49E-03**
1.05 (1.04, 1.07)	**1.72E-10**	0.83 (0.72, 0.97)	**2.58E-02**
0.98 (0.97, 1.00)	**3.05E-02**	1.31 (1.12, 1.53)	**1.39E-03**
1.02 (1.00, 1.04)	**1.75E-02**	1.01 (0.86, 1.19)	9.09E-01
1.05 (1.03, 1.06)	**4.67E-09**	0.84 (0.74, 0.96)	**2.04E-02**
1.04 (1.03, 1.06)	**3.35E-08**	0.82 (0.72, 0.95)	**1.11E-02**
1.04 (1.03, 1.06)	**6.24E-07**	0.93 (0.81, 1.06)	3.22E-01
0.95 (0.94, 0.97)	**1.33E-08**	1.14 (1.00, 1.30)	7.57E-02
0.99 (0.98, 1.00)	1.92E-01	0.78 (0.70, 0.87)	**4.21E-05**
0.98 (0.97, 1.00)	**3.68E-02**	0.77 (0.68, 0.87)	**9.52E-05**
0.98 (0.97, 1.00)	**2.67E-02**	0.77 (0.68, 0.87)	**9.23E-05**
0.99 (0.97, 1.00)	9.82E-02	0.79 (0.70, 0.88)	**1.66E-04**
1.02 (1.00, 1.03)	5.16E-02	1.30 (1.15, 1.47)	**6.70E-05**
0.92 (0.91, 0.94)	**8.33E-24**	0.73 (0.64, 0.83)	**6.86E-06**
0.96 (0.95, 0.98)	**2.27E-07**	0.77 (0.68, 0.87)	**1.09E-04**
0.99 (0.98, 1.01)	2.58E-01	0.82 (0.72, 0.92)	**2.54E-03**
0.93 (0.91, 0.94)	**4.03E-23**	0.72 (0.64, 0.82)	**4.74E-06**
1.05 (1.04, 1.07)	**1.63E-11**	1.32 (1.17, 1.50)	**3.91E-05**
1.15 (1.13, 1.16)	**7.58E-71**	1.44 (1.27, 1.63)	**4.29E-07**
0.94 (0.92, 0.95)	**2.50E-19**	0.73 (0.66, 0.82)	**1.33E-06**
0.93 (0.92, 0.95)	**8.08E-21**	0.72 (0.64, 0.81)	**6.77E-07**
0.97 (0.96, 0.98)	**6.96E-06**	0.85 (0.76, 0.95)	**5.40E-03**
1.05 (1.03, 1.06)	**5.77E-10**	1.31 (1.17, 1.46)	**1.07E-05**
1.12 (1.10, 1.14)	**9.08E-52**	1.27 (1.13, 1.43)	**2.00E-04**
0.89 (0.88, 0.90)	**9.28E-71**	0.73 (0.66, 0.80)	**1.58E-08**
0.87 (0.86, 0.88)	**1.63E-91**	0.70 (0.63, 0.77)	**7.19E-10**
0.97 (0.96, 0.99)	**1.80E-04**	0.87 (0.78, 0.98)	**2.58E-02**
1.11 (1.10, 1.12)	**2.52E-56**	1.33 (1.21, 1.46)	**4.71E-08**
1.02 (1.00, 1.03)	**1.02E-02**	0.98 (0.87, 1.10)	8.06E-01
0.92 (0.91, 0.93)	**4.31E-119**	0.91 (0.87, 0.95)	**2.65E-05**
0.92 (0.91, 0.93)	**4.00E-137**	0.93 (0.89, 0.98)	**6.03E-03**
0.95 (0.93, 0.96)	**9.01E-15**	0.82 (0.75, 0.89)	**8.30E-06**

1.11 (1.10, 1.12)	**8.81E-116**	1.16 (1.10, 1.22)	**5.15E-07**
1.02 (1.00, 1.03)	**3.05E-02**	1.07 (1.00, 1.14)	9.33E-02
0.91 (0.90, 0.92)	**4.04E-130**	0.92 (0.88, 0.95)	**9.29E-06**
0.90 (0.89, 0.92)	**2.23E-50**	0.90 (0.85, 0.96)	**3.81E-03**
0.95 (0.94, 0.97)	**3.45E-10**	0.86 (0.77, 0.96)	**1.11E-02**
1.12 (1.11, 1.13)	**7.70E-140**	1.11 (1.07, 1.15)	**2.58E-06**
1.11 (1.09, 1.12)	**2.82E-42**	1.13 (1.07, 1.20)	**7.75E-05**
0.88 (0.87, 0.89)	**4.00E-137**	0.89 (0.85, 0.92)	**5.47E-08**
0.91 (0.90, 0.92)	**5.98E-38**	0.86 (0.82, 0.91)	**3.72E-06**
0.93 (0.92, 0.95)	**9.40E-23**	0.88 (0.79, 0.98)	**3.81E-02**
1.08 (1.07, 1.09)	**2.20E-35**	1.15 (1.08, 1.23)	**6.10E-05**
1.11 (1.09, 1.13)	**6.90E-34**	1.01 (0.89, 1.13)	9.31E-01
0.89 (0.88, 0.91)	**1.06E-46**	0.94 (0.83, 1.07)	4.08E-01
0.92 (0.91, 0.93)	**1.09E-40**	0.87 (0.79, 0.96)	**1.19E-02**
0.95 (0.94, 0.97)	**2.20E-09**	1.08 (0.97, 1.20)	1.91E-01
0.98 (0.97, 1.00)	**1.78E-02**	1.04 (0.95, 1.14)	4.39E-01
1.01 (0.99, 1.02)	2.58E-01	1.19 (1.07, 1.31)	**2.10E-03**
0.99 (0.98, 1.00)	2.06E-01	0.85 (0.78, 0.94)	**2.86E-03**
0.98 (0.97, 0.99)	**3.53E-03**	0.85 (0.77, 0.94)	**3.45E-03**
1.04 (1.03, 1.06)	**6.95E-09**	0.90 (0.82, 0.98)	**3.09E-02**
1.01 (0.99, 1.02)	2.79E-01	1.10 (1.02, 1.19)	**2.86E-02**
1.07 (1.05, 1.08)	**4.16E-17**	1.28 (1.14, 1.44)	**8.64E-05**
0.95 (0.93, 0.96)	**8.80E-13**	0.82 (0.73, 0.92)	**1.15E-03**
0.95 (0.93, 0.96)	**3.91E-13**	0.85 (0.76, 0.96)	**1.18E-02**
0.97 (0.96, 0.99)	**1.78E-03**	0.75 (0.67, 0.85)	**1.61E-05**
1.04 (1.03, 1.06)	**7.37E-09**	1.16 (1.04, 1.29)	**1.33E-02**
1.14 (1.12, 1.15)	**1.28E-60**	1.10 (0.96, 1.25)	2.08E-01
0.89 (0.88, 0.91)	**1.49E-52**	0.83 (0.74, 0.94)	**5.46E-03**
0.88 (0.87, 0.90)	**6.70E-65**	0.85 (0.75, 0.96)	**1.30E-02**
1.05 (1.03, 1.07)	**5.41E-10**	0.93 (0.81, 1.06)	3.29E-01
1.06 (1.04, 1.07)	**2.47E-12**	1.22 (1.08, 1.38)	**2.52E-03**
0.91 (0.90, 0.92)	**2.47E-32**	0.98 (0.86, 1.11)	7.99E-01
0.99 (0.98, 1.01)	3.61E-01	0.88 (0.78, 0.99)	5.18E-02
1.11 (1.09, 1.13)	**1.44E-36**	0.90 (0.77, 1.04)	2.04E-01
0.90 (0.89, 0.92)	**1.52E-37**	0.67 (0.58, 0.77)	**3.42E-07**
0.93 (0.92, 0.95)	**3.74E-18**	0.79 (0.69, 0.91)	**1.78E-03**
0.88 (0.87, 0.89)	**7.07E-60**	0.70 (0.61, 0.80)	**1.43E-06**
0.92 (0.91, 0.94)	**6.50E-26**	0.72 (0.62, 0.82)	**1.12E-05**
0.90 (0.89, 0.92)	**7.09E-39**	0.66 (0.58, 0.76)	**2.21E-07**
0.91 (0.90, 0.92)	**4.92E-33**	0.69 (0.60, 0.79)	**1.26E-06**
0.97 (0.95, 0.98)	**3.55E-05**	1.01 (0.89, 1.14)	8.98E-01
0.95 (0.94, 0.97)	**1.39E-09**	1.00 (0.89, 1.14)	9.51E-01
1.03 (1.01, 1.04)	**1.11E-04**	1.03 (0.92, 1.17)	6.57E-01
1.03 (1.01, 1.04)	**1.96E-04**	1.03 (0.90, 1.17)	7.25E-01
0.95 (0.94, 0.97)	**9.33E-09**	0.73 (0.63, 0.85)	**6.97E-05**
0.99 (0.97, 1.00)	7.37E-02	1.15 (1.02, 1.30)	**3.24E-02**

0.95 (0.93, 0.96)	**1.40E-11**	0.70 (0.61, 0.82)	**1.21E-05**
0.97 (0.95, 0.98)	**5.04E-05**	0.72 (0.62, 0.83)	**1.73E-05**
0.95 (0.93, 0.96)	**2.62E-11**	0.70 (0.60, 0.81)	**5.69E-06**
0.98 (0.96, 0.99)	**1.25E-02**	0.72 (0.62, 0.84)	**1.32E-04**
0.91 (0.90, 0.93)	**6.77E-33**	0.74 (0.65, 0.85)	**4.33E-05**
0.94 (0.93, 0.95)	**1.95E-15**	0.87 (0.76, 0.99)	5.31E-02
0.96 (0.95, 0.97)	**6.10E-08**	0.86 (0.76, 0.99)	**4.41E-02**
0.87 (0.85, 0.88)	**5.44E-75**	0.76 (0.66, 0.87)	**1.35E-04**
0.88 (0.87, 0.89)	**5.33E-52**	0.77 (0.66, 0.90)	**2.62E-03**
0.90 (0.89, 0.92)	**3.79E-37**	0.68 (0.59, 0.79)	**1.90E-06**
0.88 (0.86, 0.89)	**1.23E-54**	0.83 (0.72, 0.96)	**2.34E-02**
0.90 (0.89, 0.92)	**4.00E-37**	0.69 (0.60, 0.80)	**2.58E-06**
0.89 (0.87, 0.90)	**4.36E-52**	0.69 (0.60, 0.79)	**2.58E-06**
1.00 (0.99, 1.02)	6.99E-01	1.00 (0.88, 1.12)	9.54E-01
0.99 (0.97, 1.00)	8.01E-02	0.92 (0.81, 1.05)	2.69E-01
0.90 (0.89, 0.92)	**6.54E-36**	0.90 (0.78, 1.04)	1.90E-01
0.91 (0.90, 0.93)	**7.88E-34**	0.72 (0.63, 0.81)	**2.58E-06**
0.88 (0.86, 0.89)	**4.84E-57**	0.91 (0.79, 1.05)	2.30E-01
0.94 (0.93, 0.96)	**3.32E-14**	0.82 (0.73, 0.93)	**2.73E-03**
0.90 (0.89, 0.92)	**3.41E-43**	0.80 (0.71, 0.90)	**5.93E-04**
1.09 (1.08, 1.11)	**9.32E-30**	1.28 (1.14, 1.45)	**1.68E-04**
1.08 (1.07, 1.10)	**2.37E-28**	1.12 (0.99, 1.26)	1.03E-01
1.09 (1.08, 1.10)	**3.92E-55**	1.27 (1.19, 1.35)	**3.31E-11**
1.05 (1.03, 1.06)	**5.64E-09**	1.14 (1.01, 1.29)	**4.39E-02**
1.07 (1.06, 1.08)	**3.75E-24**	1.20 (1.07, 1.35)	**4.72E-03**
0.99 (0.97, 1.00)	6.41E-02	1.22 (1.10, 1.37)	**6.65E-04**
0.95 (0.94, 0.96)	**3.59E-11**	0.84 (0.74, 0.95)	**1.23E-02**
0.91 (0.90, 0.93)	**1.94E-26**	0.86 (0.74, 0.99)	5.64E-02
0.98 (0.96, 0.99)	**3.24E-03**	1.11 (0.98, 1.25)	1.28E-01
0.93 (0.92, 0.95)	**4.65E-19**	1.08 (0.96, 1.22)	2.44E-01
0.93 (0.92, 0.95)	**1.65E-18**	1.12 (0.99, 1.26)	9.33E-02
1.03 (1.02, 1.05)	**4.07E-08**	1.10 (0.98, 1.24)	1.26E-01
1.03 (1.01, 1.04)	**6.66E-04**	1.07 (0.95, 1.20)	3.45E-01
1.07 (1.06, 1.08)	**4.82E-89**	0.85 (0.71, 1.01)	9.33E-02
1.06 (1.05, 1.07)	**1.39E-46**	1.05 (0.94, 1.17)	4.34E-01
1.08 (1.07, 1.09)	**9.44E-49**	1.07 (0.96, 1.20)	2.56E-01
1.05 (1.04, 1.06)	**1.58E-23**	1.07 (1.03, 1.11)	**2.10E-03**
0.86 (0.85, 0.88)	**9.56E-80**	0.79 (0.70, 0.89)	**5.02E-04**
1.14 (1.12, 1.15)	**4.26E-63**	1.18 (1.04, 1.34)	**1.48E-02**
0.99 (0.97, 1.01)	2.41E-01	0.86 (0.74, 1.01)	9.24E-02
0.94 (0.92, 0.95)	**7.66E-15**	1.11 (0.98, 1.25)	1.22E-01
0.90 (0.89, 0.91)	**4.41E-43**	0.70 (0.61, 0.80)	**2.58E-06**
0.89 (0.87, 0.90)	**3.82E-55**	0.69 (0.60, 0.79)	**8.24E-07**
0.88 (0.87, 0.89)	**1.68E-59**	0.71 (0.62, 0.81)	**2.58E-06**
0.99 (0.97, 1.00)	1.02E-01	0.70 (0.59, 0.83)	**1.11E-04**
0.90 (0.89, 0.92)	**4.16E-36**	0.76 (0.67, 0.88)	**3.70E-04**
1.08 (1.07, 1.09)	**8.73E-67**	1.03 (0.91, 1.17)	6.86E-01
0.94 (0.92, 0.95)	**1.47E-17**	0.84 (0.74, 0.96)	**1.89E-02**

0.88 (0.87, 0.90)	**1.43E-56**	0.68 (0.60, 0.78)	**5.95E-07**
1.02 (1.00, 1.04)	**4.43E-02**	0.73 (0.61, 0.86)	**5.27E-04**
1.04 (1.03, 1.05)	**1.32E-13**	1.07 (0.98, 1.15)	1.51E-01
1.07 (1.06, 1.08)	**2.59E-66**	1.00 (0.88, 1.12)	9.53E-01
0.92 (0.90, 0.93)	**3.96E-26**	0.67 (0.58, 0.78)	**1.17E-06**
0.95 (0.93, 0.96)	**1.28E-11**	0.86 (0.75, 0.98)	**3.58E-02**
0.90 (0.89, 0.92)	**1.79E-38**	0.74 (0.64, 0.84)	**3.33E-05**
0.93 (0.91, 0.94)	**2.08E-19**	0.69 (0.60, 0.80)	**6.29E-06**
0.94 (0.93, 0.96)	**1.57E-14**	0.70 (0.61, 0.81)	**6.29E-06**
0.95 (0.93, 0.96)	**1.15E-12**	0.88 (0.77, 1.01)	9.07E-02
0.89 (0.87, 0.90)	**5.21E-54**	0.71 (0.62, 0.81)	**3.81E-06**
1.01 (1.00, 1.03)	1.50E-01	0.76 (0.65, 0.90)	**2.10E-03**
0.92 (0.91, 0.93)	**6.21E-27**	0.74 (0.64, 0.84)	**3.59E-05**
0.94 (0.93, 0.96)	**1.48E-13**	0.92 (0.81, 1.04)	2.41E-01
0.89 (0.87, 0.90)	**8.99E-53**	0.71 (0.62, 0.81)	**3.88E-06**
1.01 (0.99, 1.02)	5.40E-01	0.73 (0.62, 0.86)	**5.41E-04**
0.94 (0.92, 0.95)	**1.08E-17**	0.76 (0.66, 0.87)	**2.92E-04**
0.99 (0.98, 1.01)	3.98E-01	0.70 (0.59, 0.83)	**1.30E-04**
1.02 (1.00, 1.04)	5.15E-02	0.92 (0.81, 1.05)	2.73E-01

liseases mortality		Cerebrovascular diseases mortality		
Current drinker		Never/former drinker		Current drinke
Adjusted HR (95% CI)	P value	Adjusted HR (95% CI)	P value	Adjusted HR (95% CI)
1.04 (1.00, 1.08)	1.13E-01	0.84 (0.67, 1.06)	3.61E-01	0.94 (0.88, 1.01)
1.03 (0.99, 1.08)	1.58E-01	0.84 (0.67, 1.06)	3.61E-01	0.94 (0.87, 1.01)
1.04 (1.00, 1.08)	9.24E-02	0.84 (0.67, 1.06)	3.61E-01	0.95 (0.88, 1.02)
1.03 (0.98, 1.07)	2.50E-01	0.83 (0.66, 1.04)	3.61E-01	0.95 (0.88, 1.02)
1.02 (0.98, 1.07)	3.56E-01	0.82 (0.66, 1.04)	3.61E-01	0.95 (0.88, 1.02)
1.03 (0.99, 1.08)	1.58E-01	0.84 (0.67, 1.05)	3.61E-01	0.95 (0.88, 1.02)
1.03 (0.99, 1.07)	1.58E-01	0.85 (0.67, 1.07)	3.61E-01	0.94 (0.87, 1.01)
1.02 (0.98, 1.07)	4.50E-01	0.84 (0.67, 1.05)	3.61E-01	0.91 (0.84, 0.98)
1.01 (0.97, 1.06)	6.12E-01	0.83 (0.66, 1.04)	3.61E-01	0.91 (0.84, 0.98)
1.01 (0.97, 1.06)	5.82E-01	0.85 (0.68, 1.06)	3.61E-01	0.91 (0.84, 0.98)
0.99 (0.94, 1.03)	6.53E-01	0.85 (0.68, 1.06)	3.61E-01	0.90 (0.83, 0.96)
0.97 (0.92, 1.01)	1.97E-01	0.86 (0.69, 1.06)	3.61E-01	0.89 (0.82, 0.96)
1.01 (0.96, 1.05)	7.48E-01	0.85 (0.68, 1.06)	3.61E-01	0.91 (0.84, 0.98)
1.02 (0.98, 1.07)	4.17E-01	0.83 (0.66, 1.04)	3.61E-01	0.91 (0.85, 0.98)
1.00 (0.96, 1.05)	8.98E-01	0.85 (0.68, 1.06)	3.61E-01	0.90 (0.83, 0.97)
0.99 (0.95, 1.04)	7.15E-01	0.84 (0.67, 1.05)	3.61E-01	0.89 (0.83, 0.96)
1.01 (0.96, 1.06)	7.34E-01	0.85 (0.68, 1.06)	3.61E-01	0.90 (0.84, 0.97)
0.98 (0.94, 1.03)	5.29E-01	0.86 (0.69, 1.07)	3.67E-01	0.89 (0.83, 0.96)
0.97 (0.92, 1.01)	1.77E-01	0.88 (0.71, 1.09)	4.11E-01	0.90 (0.83, 0.97)
1.00 (0.95, 1.05)	9.82E-01	0.85 (0.68, 1.05)	3.61E-01	0.89 (0.83, 0.96)
0.99 (0.94, 1.03)	6.06E-01	0.83 (0.66, 1.04)	3.61E-01	0.89 (0.82, 0.96)
0.93 (0.88, 0.97)	**2.39E-03**	0.87 (0.70, 1.08)	3.92E-01	0.87 (0.81, 0.94)
0.94 (0.89, 0.98)	**8.18E-03**	0.85 (0.69, 1.05)	3.61E-01	0.87 (0.81, 0.94)
0.93 (0.88, 0.97)	**1.97E-03**	0.88 (0.71, 1.08)	4.02E-01	0.87 (0.81, 0.94)
0.89 (0.85, 0.93)	**4.39E-06**	0.93 (0.75, 1.14)	6.34E-01	0.88 (0.82, 0.95)
0.88 (0.84, 0.92)	**3.58E-07**	0.96 (0.78, 1.17)	7.59E-01	0.89 (0.83, 0.96)
0.92 (0.87, 0.96)	**3.70E-04**	0.89 (0.72, 1.10)	4.77E-01	0.88 (0.81, 0.94)
0.98 (0.94, 1.02)	4.05E-01	0.82 (0.66, 1.03)	3.61E-01	0.89 (0.83, 0.96)
1.01 (0.96, 1.06)	7.43E-01	0.90 (0.72, 1.11)	4.92E-01	0.92 (0.86, 0.99)
1.00 (0.95, 1.04)	8.98E-01	0.88 (0.71, 1.09)	4.21E-01	0.91 (0.85, 0.98)
0.96 (0.92, 1.01)	1.11E-01	0.89 (0.72, 1.10)	4.67E-01	0.89 (0.83, 0.96)
0.96 (0.92, 1.01)	1.21E-01	0.92 (0.74, 1.13)	5.71E-01	0.90 (0.84, 0.97)
0.98 (0.93, 1.03)	4.44E-01	0.92 (0.75, 1.14)	5.91E-01	0.91 (0.85, 0.98)
0.93 (0.89, 0.97)	**3.51E-03**	0.91 (0.74, 1.12)	5.30E-01	0.89 (0.82, 0.95)
1.04 (1.00, 1.09)	8.37E-02	0.87 (0.69, 1.08)	3.92E-01	0.95 (0.88, 1.02)
1.00 (0.96, 1.05)	8.98E-01	0.98 (0.80, 1.21)	8.99E-01	0.95 (0.88, 1.02)
1.01 (0.96, 1.06)	7.08E-01	0.97 (0.79, 1.20)	8.66E-01	0.96 (0.89, 1.03)
1.04 (0.99, 1.09)	1.15E-01	1.00 (0.81, 1.24)	9.94E-01	0.97 (0.91, 1.04)
0.94 (0.90, 0.99)	**2.46E-02**	0.98 (0.80, 1.21)	8.99E-01	0.94 (0.87, 1.01)
0.92 (0.88, 0.97)	**1.67E-03**	0.98 (0.79, 1.20)	8.66E-01	0.93 (0.87, 1.00)

1.00 (0.95, 1.04)	9.08E-01	1.00 (0.81, 1.23)	9.94E-01	0.96 (0.89, 1.03)
1.11 (1.06, 1.16)	**3.20E-06**	0.93 (0.75, 1.16)	6.63E-01	1.00 (0.94, 1.08)
0.90 (0.86, 0.94)	**2.72E-05**	0.92 (0.75, 1.13)	5.84E-01	0.87 (0.81, 0.94)
0.89 (0.85, 0.93)	**1.83E-06**	0.90 (0.73, 1.12)	5.16E-01	0.88 (0.82, 0.95)
0.90 (0.86, 0.95)	**6.02E-05**	0.94 (0.76, 1.16)	6.69E-01	0.90 (0.83, 0.97)
0.86 (0.82, 0.91)	**7.55E-09**	0.89 (0.72, 1.11)	4.77E-01	0.87 (0.81, 0.94)
0.86 (0.82, 0.90)	**1.22E-09**	0.88 (0.71, 1.09)	4.17E-01	0.87 (0.81, 0.93)
0.89 (0.85, 0.93)	**1.46E-06**	0.94 (0.76, 1.16)	6.83E-01	0.89 (0.83, 0.96)
1.11 (1.07, 1.16)	**1.05E-06**	0.95 (0.76, 1.18)	7.46E-01	1.01 (0.94, 1.08)
0.92 (0.88, 0.96)	**4.94E-04**	0.93 (0.75, 1.14)	6.34E-01	0.88 (0.82, 0.94)
0.88 (0.84, 0.92)	**3.47E-07**	0.87 (0.70, 1.07)	3.69E-01	0.85 (0.79, 0.91)
0.87 (0.84, 0.92)	**6.79E-08**	0.86 (0.70, 1.07)	3.67E-01	0.85 (0.79, 0.91)
0.87 (0.83, 0.91)	**1.50E-08**	0.87 (0.70, 1.07)	3.72E-01	0.84 (0.78, 0.91)
0.87 (0.83, 0.91)	**5.18E-08**	0.86 (0.69, 1.06)	3.61E-01	0.84 (0.78, 0.91)
0.86 (0.82, 0.90)	**1.06E-09**	0.89 (0.72, 1.10)	4.73E-01	0.85 (0.79, 0.92)
1.11 (1.07, 1.16)	**1.56E-06**	0.93 (0.75, 1.16)	6.63E-01	0.98 (0.91, 1.06)
0.91 (0.87, 0.95)	**8.43E-05**	0.88 (0.71, 1.09)	4.17E-01	0.85 (0.79, 0.92)
0.90 (0.86, 0.94)	**2.50E-05**	0.84 (0.68, 1.03)	3.61E-01	0.83 (0.77, 0.90)
0.90 (0.86, 0.94)	**2.77E-05**	0.84 (0.68, 1.04)	3.61E-01	0.83 (0.77, 0.89)
0.89 (0.85, 0.93)	**1.61E-06**	0.84 (0.68, 1.03)	3.61E-01	0.83 (0.77, 0.89)
0.90 (0.86, 0.94)	**1.43E-05**	0.83 (0.67, 1.02)	3.61E-01	0.83 (0.77, 0.89)
0.87 (0.83, 0.91)	**1.42E-08**	0.87 (0.70, 1.07)	3.67E-01	0.83 (0.78, 0.90)
1.09 (1.05, 1.13)	**7.56E-05**	0.90 (0.73, 1.13)	5.29E-01	0.96 (0.89, 1.03)
0.92 (0.88, 0.97)	**1.37E-03**	0.89 (0.72, 1.10)	4.52E-01	0.86 (0.80, 0.92)
0.92 (0.88, 0.96)	**3.35E-04**	0.88 (0.71, 1.08)	4.02E-01	0.84 (0.78, 0.90)
0.93 (0.89, 0.98)	**3.83E-03**	0.93 (0.76, 1.14)	6.52E-01	0.85 (0.79, 0.91)
0.90 (0.86, 0.94)	**8.50E-06**	0.86 (0.70, 1.06)	3.67E-01	0.84 (0.78, 0.90)
0.91 (0.87, 0.95)	**7.10E-05**	0.84 (0.68, 1.04)	3.61E-01	0.84 (0.78, 0.90)
0.88 (0.84, 0.92)	**2.34E-07**	0.93 (0.76, 1.14)	6.34E-01	0.84 (0.79, 0.91)
1.06 (1.02, 1.10)	**7.65E-03**	0.87 (0.70, 1.09)	4.17E-01	0.95 (0.88, 1.02)
0.97 (0.91, 1.02)	2.74E-01	1.14 (0.93, 1.39)	3.92E-01	1.10 (1.02, 1.18)
0.97 (0.92, 1.03)	4.44E-01	1.16 (0.95, 1.42)	3.61E-01	1.12 (1.04, 1.20)
0.99 (0.93, 1.04)	6.61E-01	1.17 (0.96, 1.43)	3.61E-01	1.13 (1.05, 1.22)
0.96 (0.90, 1.01)	1.50E-01	1.16 (0.95, 1.41)	3.61E-01	1.10 (1.02, 1.18)
0.94 (0.89, 1.00)	5.94E-02	1.14 (0.93, 1.40)	3.88E-01	1.10 (1.02, 1.18)
1.00 (0.95, 1.06)	8.98E-01	1.21 (1.00, 1.45)	2.85E-01	1.09 (1.02, 1.18)
1.06 (1.01, 1.10)	**1.60E-02**	0.96 (0.77, 1.19)	8.02E-01	1.03 (0.96, 1.10)
0.91 (0.86, 0.97)	**3.20E-03**	1.05 (0.84, 1.31)	7.59E-01	1.10 (1.02, 1.19)
0.92 (0.87, 0.98)	**8.91E-03**	1.05 (0.83, 1.31)	7.85E-01	1.10 (1.02, 1.19)
0.93 (0.88, 0.99)	**2.30E-02**	1.02 (0.81, 1.29)	8.86E-01	1.10 (1.02, 1.19)
0.91 (0.85, 0.96)	**2.12E-03**	1.07 (0.86, 1.34)	6.63E-01	1.10 (1.02, 1.19)
0.90 (0.85, 0.96)	**1.05E-03**	1.07 (0.86, 1.34)	6.63E-01	1.10 (1.02, 1.19)
0.93 (0.87, 0.99)	**2.09E-02**	1.07 (0.86, 1.34)	6.63E-01	1.10 (1.02, 1.19)
1.03 (0.98, 1.08)	2.50E-01	0.92 (0.73, 1.16)	6.52E-01	1.06 (0.99, 1.14)
0.89 (0.84, 0.93)	**1.16E-05**	0.74 (0.58, 0.95)	1.66E-01	0.99 (0.92, 1.07)
0.90 (0.86, 0.95)	**1.60E-04**	0.73 (0.57, 0.94)	1.41E-01	0.99 (0.92, 1.07)
0.92 (0.88, 0.97)	**1.26E-03**	0.72 (0.57, 0.92)	1.16E-01	0.99 (0.92, 1.07)
0.88 (0.84, 0.93)	**3.62E-06**	0.77 (0.60, 0.98)	2.36E-01	1.00 (0.92, 1.07)

0.88 (0.83, 0.92)	**2.37E-06**	0.77 (0.61, 0.98)	2.36E-01	1.00 (0.92, 1.07)
0.89 (0.85, 0.94)	**3.47E-05**	0.78 (0.61, 1.00)	2.84E-01	1.00 (0.92, 1.08)
1.04 (0.99, 1.09)	1.19E-01	0.82 (0.65, 1.03)	3.61E-01	0.99 (0.92, 1.06)
0.87 (0.83, 0.91)	**1.28E-08**	0.68 (0.55, 0.84)	**1.90E-02**	0.85 (0.79, 0.91)
0.90 (0.86, 0.95)	**3.34E-05**	0.66 (0.53, 0.83)	**1.90E-02**	0.88 (0.82, 0.94)
0.91 (0.87, 0.96)	**2.06E-04**	0.66 (0.53, 0.82)	**1.90E-02**	0.90 (0.83, 0.96)
0.87 (0.83, 0.91)	**5.91E-09**	0.68 (0.55, 0.84)	**1.90E-02**	0.86 (0.80, 0.92)
0.86 (0.82, 0.90)	**3.02E-10**	0.69 (0.56, 0.85)	**1.90E-02**	0.85 (0.79, 0.91)
0.91 (0.87, 0.96)	**2.51E-04**	0.71 (0.57, 0.89)	6.36E-02	0.89 (0.83, 0.96)
1.07 (1.02, 1.12)	**4.79E-03**	0.84 (0.67, 1.05)	3.61E-01	0.95 (0.89, 1.03)
1.14 (1.08, 1.21)	**1.05E-05**	1.15 (0.91, 1.46)	4.20E-01	1.09 (1.01, 1.18)
0.97 (0.92, 1.02)	2.51E-01	1.05 (0.86, 1.29)	7.18E-01	1.09 (1.02, 1.17)
0.96 (0.90, 1.01)	1.51E-01	0.99 (0.78, 1.24)	9.40E-01	1.07 (0.99, 1.14)
0.99 (0.94, 1.04)	7.75E-01	1.15 (0.97, 1.36)	3.61E-01	1.12 (1.05, 1.19)
1.00 (0.95, 1.05)	9.19E-01	0.92 (0.76, 1.13)	5.87E-01	0.90 (0.84, 0.97)
0.92 (0.87, 0.98)	**1.22E-02**	1.30 (1.10, 1.55)	6.36E-02	1.07 (0.99, 1.15)
0.92 (0.87, 0.97)	**7.73E-03**	1.26 (1.06, 1.50)	1.16E-01	1.05 (0.98, 1.14)
0.97 (0.91, 1.02)	2.50E-01	1.37 (1.16, 1.61)	**1.90E-02**	1.12 (1.04, 1.20)
1.07 (1.01, 1.13)	**1.86E-02**	0.75 (0.63, 0.90)	**4.60E-02**	0.94 (0.87, 1.01)
1.13 (1.06, 1.20)	**6.31E-04**	0.95 (0.77, 1.18)	7.47E-01	0.96 (0.89, 1.04)
1.01 (0.97, 1.06)	6.19E-01	1.29 (1.06, 1.55)	1.16E-01	1.07 (0.99, 1.15)
0.98 (0.93, 1.03)	4.17E-01	1.29 (1.06, 1.56)	1.16E-01	1.08 (1.01, 1.16)
1.12 (1.07, 1.18)	**2.52E-05**	1.22 (0.98, 1.53)	3.45E-01	1.01 (0.93, 1.08)
0.94 (0.89, 0.99)	**3.23E-02**	0.82 (0.66, 1.02)	3.45E-01	0.97 (0.90, 1.05)
0.92 (0.88, 0.95)	**5.20E-05**	1.11 (0.90, 1.37)	4.94E-01	0.98 (0.91, 1.05)
0.89 (0.85, 0.93)	**1.06E-06**	1.18 (0.96, 1.46)	3.61E-01	1.00 (0.93, 1.08)
0.89 (0.85, 0.93)	**4.32E-07**	1.19 (0.96, 1.47)	3.61E-01	1.01 (0.94, 1.08)
0.91 (0.87, 0.95)	**1.97E-05**	1.16 (0.94, 1.43)	3.61E-01	0.99 (0.92, 1.06)
1.12 (1.07, 1.17)	**1.79E-06**	0.86 (0.69, 1.06)	3.61E-01	1.00 (0.93, 1.08)
0.85 (0.81, 0.89)	**1.23E-11**	1.05 (0.85, 1.30)	7.46E-01	0.91 (0.85, 0.98)
0.90 (0.86, 0.94)	**2.11E-06**	1.13 (0.91, 1.39)	4.46E-01	0.95 (0.89, 1.02)
0.94 (0.90, 0.98)	**1.07E-02**	1.17 (0.95, 1.44)	3.61E-01	0.98 (0.91, 1.05)
0.84 (0.80, 0.88)	**1.09E-12**	1.06 (0.86, 1.32)	6.82E-01	0.92 (0.86, 0.99)
1.14 (1.09, 1.19)	**6.70E-08**	0.90 (0.73, 1.12)	5.17E-01	1.06 (0.99, 1.14)
1.29 (1.24, 1.35)	**2.06E-28**	1.30 (1.05, 1.61)	1.49E-01	1.13 (1.05, 1.21)
0.83 (0.80, 0.87)	**4.71E-18**	1.01 (0.82, 1.25)	9.40E-01	0.93 (0.87, 1.00)
0.82 (0.79, 0.85)	**6.15E-20**	0.99 (0.80, 1.22)	9.68E-01	0.93 (0.87, 1.00)
0.93 (0.89, 0.96)	**1.18E-04**	1.16 (0.94, 1.43)	3.67E-01	0.97 (0.91, 1.04)
1.17 (1.12, 1.21)	**3.65E-13**	0.94 (0.76, 1.16)	6.63E-01	1.05 (0.98, 1.13)
1.19 (1.14, 1.24)	**1.04E-13**	1.32 (1.08, 1.62)	1.07E-01	1.14 (1.07, 1.22)
0.82 (0.79, 0.85)	**9.15E-28**	0.88 (0.73, 1.06)	3.68E-01	0.87 (0.82, 0.93)
0.79 (0.76, 0.82)	**3.61E-34**	0.80 (0.67, 0.97)	1.92E-01	0.85 (0.79, 0.90)
0.94 (0.90, 0.98)	**2.14E-03**	1.14 (0.93, 1.40)	3.92E-01	0.99 (0.92, 1.05)
1.20 (1.16, 1.24)	**9.71E-25**	1.07 (0.88, 1.30)	6.53E-01	1.12 (1.06, 1.20)
0.99 (0.95, 1.03)	7.23E-01	1.05 (0.90, 1.21)	6.63E-01	1.05 (0.99, 1.12)
0.91 (0.90, 0.93)	**1.45E-25**	0.94 (0.83, 1.05)	4.50E-01	0.91 (0.88, 0.94)
0.93 (0.91, 0.94)	**2.72E-14**	0.92 (0.85, 0.99)	2.36E-01	0.91 (0.88, 0.94)
0.87 (0.84, 0.90)	**2.95E-14**	1.08 (0.87, 1.33)	6.52E-01	0.93 (0.87, 1.00)

1.13 (1.11, 1.15)	**2.15E-30**	1.08 (0.92, 1.26)	5.16E-01	1.12 (1.07, 1.17)
1.05 (1.01, 1.10)	**2.27E-02**	1.08 (0.95, 1.22)	4.17E-01	1.03 (0.96, 1.11)
0.90 (0.89, 0.92)	**1.73E-29**	0.93 (0.85, 1.03)	3.67E-01	0.91 (0.88, 0.94)
0.91 (0.87, 0.94)	**1.61E-06**	0.88 (0.80, 0.98)	1.51E-01	0.88 (0.83, 0.94)
0.89 (0.86, 0.93)	**7.00E-08**	1.11 (0.89, 1.38)	5.24E-01	0.98 (0.92, 1.06)
1.13 (1.11, 1.16)	**1.03E-29**	1.07 (0.94, 1.21)	4.77E-01	1.12 (1.07, 1.17)
1.13 (1.08, 1.18)	**4.50E-07**	1.16 (1.06, 1.27)	**4.60E-02**	1.11 (1.03, 1.19)
0.86 (0.84, 0.88)	**2.91E-32**	0.91 (0.82, 1.00)	2.91E-01	0.88 (0.84, 0.93)
0.88 (0.85, 0.92)	**3.98E-08**	0.85 (0.77, 0.94)	**4.60E-02**	0.90 (0.84, 0.96)
0.91 (0.87, 0.94)	**1.46E-06**	1.12 (0.89, 1.39)	5.12E-01	0.95 (0.89, 1.01)
1.11 (1.08, 1.14)	**4.22E-12**	0.94 (0.76, 1.17)	7.01E-01	1.07 (1.01, 1.14)
1.02 (0.98, 1.06)	4.44E-01	1.03 (0.84, 1.27)	8.23E-01	1.16 (1.07, 1.27)
0.96 (0.93, 1.01)	1.14E-01	1.00 (0.81, 1.23)	9.94E-01	0.88 (0.81, 0.94)
0.93 (0.90, 0.97)	**1.72E-04**	0.94 (0.78, 1.14)	6.63E-01	0.95 (0.89, 1.01)
1.03 (0.99, 1.07)	1.25E-01	1.06 (0.88, 1.27)	6.82E-01	0.88 (0.81, 0.96)
1.01 (0.98, 1.05)	5.88E-01	0.95 (0.77, 1.17)	7.47E-01	0.94 (0.87, 1.01)
1.09 (1.05, 1.13)	**1.50E-05**	0.94 (0.78, 1.14)	6.69E-01	0.94 (0.88, 1.01)
0.92 (0.89, 0.96)	**2.79E-05**	1.03 (0.85, 1.26)	8.23E-01	1.05 (0.98, 1.12)
0.91 (0.88, 0.95)	**5.13E-06**	1.04 (0.85, 1.27)	7.88E-01	1.05 (0.97, 1.12)
0.98 (0.94, 1.01)	2.50E-01	1.00 (0.82, 1.21)	9.94E-01	1.03 (0.96, 1.11)
1.05 (1.02, 1.08)	**3.00E-03**	1.00 (0.82, 1.22)	9.94E-01	0.98 (0.91, 1.05)
1.15 (1.10, 1.20)	**6.13E-10**	1.03 (0.84, 1.27)	8.23E-01	1.01 (0.94, 1.09)
0.88 (0.84, 0.92)	**2.18E-08**	1.00 (0.81, 1.24)	9.94E-01	0.99 (0.92, 1.06)
0.90 (0.86, 0.93)	**9.27E-07**	1.03 (0.83, 1.27)	8.66E-01	0.99 (0.93, 1.07)
0.87 (0.83, 0.91)	**2.50E-08**	0.91 (0.73, 1.13)	5.56E-01	0.98 (0.91, 1.06)
1.10 (1.06, 1.13)	**4.96E-08**	0.97 (0.78, 1.21)	8.53E-01	1.01 (0.94, 1.09)
1.05 (1.00, 1.10)	7.14E-02	0.96 (0.77, 1.20)	8.18E-01	1.12 (1.04, 1.21)
0.89 (0.85, 0.93)	**1.31E-07**	1.02 (0.82, 1.27)	8.86E-01	0.92 (0.86, 0.99)
0.88 (0.85, 0.92)	**3.53E-08**	0.98 (0.79, 1.21)	8.86E-01	0.91 (0.85, 0.98)
1.02 (0.97, 1.07)	4.43E-01	1.14 (0.95, 1.38)	3.61E-01	1.04 (0.97, 1.12)
1.16 (1.11, 1.21)	**6.20E-10**	1.00 (0.81, 1.24)	9.96E-01	1.01 (0.94, 1.09)
0.98 (0.93, 1.02)	3.56E-01	0.77 (0.63, 0.95)	1.51E-01	0.86 (0.80, 0.93)
0.98 (0.93, 1.02)	3.38E-01	1.10 (0.89, 1.36)	5.18E-01	1.04 (0.97, 1.12)
0.95 (0.90, 1.00)	9.77E-02	1.14 (0.92, 1.41)	4.23E-01	1.15 (1.06, 1.24)
0.86 (0.82, 0.90)	**5.91E-09**	0.85 (0.68, 1.05)	3.61E-01	0.87 (0.81, 0.94)
0.95 (0.91, 1.00)	5.27E-02	0.89 (0.72, 1.10)	4.52E-01	0.90 (0.83, 0.96)
0.88 (0.84, 0.92)	**7.19E-08**	0.86 (0.69, 1.06)	3.61E-01	0.84 (0.78, 0.90)
0.90 (0.86, 0.95)	**3.65E-05**	0.88 (0.72, 1.09)	4.27E-01	0.88 (0.82, 0.94)
0.86 (0.82, 0.90)	**1.22E-09**	0.84 (0.68, 1.05)	3.61E-01	0.87 (0.81, 0.94)
0.88 (0.84, 0.92)	**3.15E-07**	0.86 (0.70, 1.07)	3.67E-01	0.88 (0.81, 0.94)

1.03 (0.99, 1.07)	2.43E-01	**0.84 (0.67, 1.06)**	3.61E-01	**0.93 (0.86, 1.00)**
1.02 (0.97, 1.06)	5.56E-01	**0.84 (0.67, 1.05)**	3.61E-01	**0.91 (0.85, 0.98)**
1.10 (1.06, 1.14)	**1.36E-05**	0.92 (0.74, 1.14)	5.91E-01	0.97 (0.90, 1.04)
1.05 (1.01, 1.10)	**3.89E-02**	0.85 (0.67, 1.06)	3.61E-01	1.00 (0.93, 1.07)
0.91 (0.87, 0.96)	**4.48E-04**	0.78 (0.62, 0.98)	2.36E-01	0.92 (0.86, 1.00)
1.09 (1.04, 1.13)	**4.29E-04**	0.90 (0.72, 1.12)	5.16E-01	0.95 (0.88, 1.02)

0.89 (0.84, 0.93)	**3.22E-06**	0.80 (0.63, 1.00)	3.00E-01	0.92 (0.85, 0.99)
0.92 (0.88, 0.97)	**1.93E-03**	0.87 (0.69, 1.08)	3.88E-01	0.94 (0.87, 1.01)
0.89 (0.85, 0.94)	**1.58E-05**	0.80 (0.63, 1.00)	3.00E-01	0.92 (0.86, 0.99)
0.88 (0.84, 0.93)	**6.33E-06**	0.76 (0.59, 0.97)	2.05E-01	0.99 (0.92, 1.07)
0.92 (0.88, 0.96)	**4.15E-04**	0.91 (0.74, 1.12)	5.18E-01	0.87 (0.81, 0.93)
0.99 (0.95, 1.04)	8.37E-01	1.04 (0.84, 1.27)	8.16E-01	0.90 (0.84, 0.97)
0.99 (0.95, 1.03)	6.33E-01	0.81 (0.64, 1.01)	3.17E-01	0.91 (0.85, 0.98)
0.84 (0.80, 0.88)	**5.63E-13**	1.05 (0.85, 1.29)	7.59E-01	0.92 (0.85, 0.99)
0.83 (0.79, 0.88)	**1.24E-10**	0.93 (0.75, 1.16)	6.63E-01	0.92 (0.85, 0.99)
0.87 (0.83, 0.92)	**1.06E-07**	0.90 (0.73, 1.12)	5.16E-01	0.89 (0.83, 0.96)
0.88 (0.84, 0.92)	**1.55E-06**	0.86 (0.68, 1.08)	3.73E-01	0.89 (0.83, 0.96)
0.89 (0.85, 0.93)	**4.20E-06**	0.85 (0.68, 1.06)	3.61E-01	0.88 (0.81, 0.95)
0.87 (0.83, 0.92)	**1.71E-07**	0.83 (0.66, 1.04)	3.61E-01	0.87 (0.80, 0.93)
1.06 (1.02, 1.10)	**8.39E-03**	0.85 (0.68, 1.06)	3.61E-01	0.96 (0.89, 1.03)
1.01 (0.97, 1.05)	7.14E-01	0.78 (0.62, 0.98)	2.36E-01	0.93 (0.86, 1.00)
0.86 (0.82, 0.90)	**7.51E-09**	1.07 (0.88, 1.30)	6.63E-01	0.98 (0.91, 1.05)
0.83 (0.79, 0.87)	**3.90E-16**	1.18 (0.95, 1.47)	3.61E-01	0.96 (0.89, 1.03)
0.87 (0.83, 0.92)	**3.47E-07**	0.97 (0.78, 1.20)	8.33E-01	0.93 (0.86, 1.00)
0.89 (0.85, 0.92)	**6.70E-08**	1.23 (0.98, 1.55)	3.37E-01	1.00 (0.93, 1.07)
0.85 (0.82, 0.89)	**4.60E-13**	1.20 (0.96, 1.50)	3.61E-01	0.97 (0.90, 1.04)
1.22 (1.16, 1.27)	**1.99E-17**	0.95 (0.77, 1.18)	7.47E-01	1.04 (0.97, 1.12)
1.06 (1.02, 1.10)	**8.41E-03**	0.76 (0.61, 0.95)	1.41E-01	1.01 (0.94, 1.08)
1.17 (1.14, 1.19)	**1.23E-40**	1.19 (1.04, 1.37)	1.41E-01	1.06 (1.00, 1.12)
1.06 (1.02, 1.11)	**9.34E-03**	0.81 (0.65, 1.02)	3.40E-01	1.03 (0.96, 1.10)
1.11 (1.07, 1.15)	**1.42E-08**	1.09 (0.89, 1.33)	5.59E-01	1.06 (1.00, 1.13)
1.07 (1.02, 1.11)	**6.34E-03**	0.92 (0.74, 1.13)	5.78E-01	1.03 (0.96, 1.10)
0.94 (0.90, 0.98)	**6.35E-03**	1.21 (0.99, 1.48)	3.17E-01	1.10 (1.02, 1.18)
0.89 (0.84, 0.93)	**6.85E-06**	1.05 (0.91, 1.21)	6.63E-01	0.98 (0.91, 1.05)
1.05 (1.01, 1.10)	**3.23E-02**	0.85 (0.68, 1.07)	3.61E-01	0.97 (0.90, 1.04)
1.02 (0.98, 1.07)	4.41E-01	0.76 (0.60, 0.96)	1.92E-01	0.90 (0.83, 0.97)
1.04 (0.99, 1.09)	1.39E-01	0.78 (0.62, 0.97)	2.16E-01	0.90 (0.83, 0.97)
1.05 (1.02, 1.08)	**1.92E-04**	1.03 (0.85, 1.26)	8.23E-01	1.02 (0.95, 1.09)
1.08 (1.03, 1.13)	**1.08E-03**	1.19 (0.99, 1.43)	3.25E-01	0.98 (0.91, 1.05)
1.05 (1.03, 1.07)	**5.57E-06**	1.04 (0.91, 1.19)	7.01E-01	1.07 (1.04, 1.11)
1.07 (1.05, 1.09)	**3.16E-10**	1.20 (1.05, 1.36)	1.06E-01	1.06 (1.02, 1.11)
1.08 (1.04, 1.11)	**3.14E-06**	1.19 (1.04, 1.35)	1.41E-01	1.07 (1.02, 1.13)
1.07 (1.06, 1.09)	**5.34E-18**	1.09 (1.03, 1.16)	6.36E-02	1.06 (1.03, 1.10)
0.85 (0.81, 0.89)	**1.23E-11**	0.86 (0.70, 1.06)	3.61E-01	0.91 (0.85, 0.98)
1.27 (1.21, 1.32)	**2.23E-25**	0.93 (0.75, 1.16)	6.63E-01	1.09 (1.02, 1.17)
0.89 (0.84, 0.94)	**2.65E-04**	1.19 (0.97, 1.46)	3.61E-01	1.07 (0.99, 1.16)
1.04 (0.99, 1.08)	1.42E-01	0.78 (0.62, 0.98)	2.36E-01	0.91 (0.84, 0.98)
0.89 (0.85, 0.93)	**1.21E-06**	0.87 (0.70, 1.07)	3.73E-01	0.85 (0.79, 0.92)
0.87 (0.83, 0.91)	**2.50E-08**	0.87 (0.71, 1.08)	3.95E-01	0.84 (0.78, 0.91)
0.88 (0.84, 0.92)	**3.58E-07**	0.85 (0.68, 1.05)	3.61E-01	0.84 (0.78, 0.90)
0.86 (0.81, 0.91)	**3.62E-07**	0.87 (0.68, 1.11)	4.43E-01	1.02 (0.94, 1.10)
0.81 (0.78, 0.85)	**3.90E-16**	1.14 (0.92, 1.41)	4.21E-01	0.97 (0.90, 1.05)
1.05 (1.01, 1.09)	**1.06E-02**	1.17 (1.02, 1.34)	1.76E-01	1.06 (1.00, 1.12)
0.97 (0.93, 1.02)	2.59E-01	0.87 (0.70, 1.08)	3.97E-01	0.89 (0.83, 0.96)

0.86 (0.82, 0.90)	**2.81E-09**	0.89 (0.72, 1.10)	4.50E-01	0.85 (0.79, 0.91)
0.90 (0.85, 0.95)	**3.24E-04**	0.89 (0.70, 1.14)	5.18E-01	1.03 (0.95, 1.11)
1.06 (1.03, 1.09)	**1.04E-04**	0.92 (0.72, 1.19)	6.63E-01	1.01 (0.94, 1.09)
1.08 (1.05, 1.10)	**3.98E-08**	1.13 (0.97, 1.32)	3.61E-01	1.08 (1.04, 1.12)
0.85 (0.81, 0.90)	**2.33E-09**	0.72 (0.57, 0.90)	8.96E-02	0.91 (0.85, 0.99)
0.99 (0.94, 1.03)	6.08E-01	0.90 (0.73, 1.11)	4.92E-01	0.91 (0.84, 0.98)
0.91 (0.87, 0.96)	**2.62E-04**	0.91 (0.74, 1.12)	5.24E-01	0.87 (0.81, 0.93)
0.86 (0.82, 0.90)	**1.43E-08**	0.71 (0.56, 0.90)	8.96E-02	0.93 (0.86, 1.01)
0.90 (0.86, 0.94)	**1.90E-05**	0.79 (0.63, 0.99)	2.52E-01	0.91 (0.84, 0.98)
0.99 (0.95, 1.04)	7.43E-01	0.87 (0.70, 1.08)	3.88E-01	0.90 (0.84, 0.97)
0.89 (0.85, 0.93)	**1.56E-06**	0.86 (0.70, 1.07)	3.67E-01	0.84 (0.78, 0.90)
0.91 (0.86, 0.96)	**6.90E-04**	0.79 (0.62, 1.02)	3.40E-01	1.02 (0.94, 1.10)
0.91 (0.87, 0.95)	**7.10E-05**	0.81 (0.65, 1.00)	3.00E-01	0.88 (0.82, 0.94)
0.99 (0.95, 1.04)	8.29E-01	0.85 (0.68, 1.06)	3.61E-01	0.90 (0.84, 0.97)
0.89 (0.85, 0.93)	**1.88E-06**	0.86 (0.69, 1.06)	3.61E-01	0.84 (0.78, 0.91)
0.89 (0.85, 0.94)	**8.26E-05**	0.82 (0.64, 1.05)	3.61E-01	1.02 (0.94, 1.10)
0.94 (0.90, 0.98)	**1.21E-02**	0.90 (0.73, 1.12)	5.16E-01	0.90 (0.84, 0.97)
0.87 (0.82, 0.92)	**1.53E-06**	0.87 (0.68, 1.11)	4.52E-01	1.02 (0.95, 1.11)
1.01 (0.95, 1.06)	8.78E-01	1.16 (0.88, 1.53)	4.77E-01	0.99 (0.91, 1.07)

	Cancer mortality			
er	Never/former drinker		Current drinker	
P value	Adjusted HR (95% CI)	P value	Adjusted HR (95% CI)	P value
1.67E-01	1.00 (0.94, 1.07)	9.78E-01	1.02 (1.00, 1.04)	7.28E-02
1.53E-01	1.00 (0.94, 1.07)	9.90E-01	1.02 (1.00, 1.04)	9.42E-02
2.27E-01	1.00 (0.94, 1.07)	9.90E-01	1.02 (1.00, 1.04)	5.26E-02
2.23E-01	1.00 (0.94, 1.07)	9.99E-01	1.02 (1.00, 1.04)	1.13E-01
2.23E-01	1.00 (0.94, 1.07)	9.90E-01	1.02 (0.99, 1.04)	1.68E-01
2.27E-01	1.00 (0.94, 1.07)	9.90E-01	1.02 (1.00, 1.04)	7.28E-02
1.27E-01	1.00 (0.94, 1.07)	9.90E-01	1.02 (1.00, 1.04)	1.05E-01
**2.55E-02**	0.99 (0.93, 1.06)	8.91E-01	1.00 (0.98, 1.02)	9.84E-01
**2.26E-02**	0.99 (0.93, 1.06)	8.97E-01	1.00 (0.98, 1.02)	9.20E-01
**2.65E-02**	0.99 (0.93, 1.06)	8.97E-01	1.00 (0.98, 1.02)	8.95E-01
**9.84E-03**	0.98 (0.92, 1.05)	7.65E-01	0.99 (0.97, 1.01)	4.11E-01
**5.34E-03**	0.98 (0.91, 1.04)	6.62E-01	0.98 (0.96, 1.00)	1.03E-01
**2.46E-02**	0.99 (0.92, 1.05)	8.49E-01	1.00 (0.98, 1.02)	9.91E-01
**3.10E-02**	0.99 (0.93, 1.06)	9.40E-01	1.00 (0.98, 1.02)	9.71E-01
**1.29E-02**	0.98 (0.92, 1.05)	7.83E-01	0.99 (0.97, 1.01)	4.18E-01
**8.25E-03**	0.98 (0.92, 1.04)	6.95E-01	0.98 (0.96, 1.00)	1.47E-01
**1.91E-02**	0.98 (0.92, 1.05)	7.98E-01	0.99 (0.97, 1.02)	6.03E-01
**9.74E-03**	0.98 (0.92, 1.04)	6.97E-01	0.98 (0.96, 1.01)	1.84E-01
**1.12E-02**	0.98 (0.91, 1.04)	6.79E-01	0.98 (0.96, 1.00)	1.25E-01
**9.80E-03**	0.98 (0.92, 1.05)	7.30E-01	0.99 (0.97, 1.01)	2.78E-01
**6.83E-03**	0.98 (0.91, 1.04)	6.62E-01	0.98 (0.96, 1.00)	6.60E-02
**1.38E-03**	0.96 (0.90, 1.02)	3.87E-01	0.96 (0.94, 0.98)	**2.92E-04**
**1.46E-03**	0.96 (0.90, 1.02)	3.87E-01	0.96 (0.94, 0.98)	**3.41E-04**
**1.53E-03**	0.96 (0.90, 1.02)	3.97E-01	0.96 (0.94, 0.98)	**3.95E-04**
**2.77E-03**	0.95 (0.89, 1.02)	3.31E-01	0.95 (0.93, 0.97)	**6.84E-06**
**7.02E-03**	0.95 (0.89, 1.02)	2.90E-01	0.95 (0.93, 0.97)	**1.77E-06**
**1.67E-03**	0.96 (0.90, 1.02)	4.16E-01	0.96 (0.94, 0.98)	**1.26E-04**
**7.92E-03**	0.97 (0.91, 1.03)	5.45E-01	0.97 (0.95, 0.99)	**1.86E-02**
**4.90E-02**	0.99 (0.93, 1.06)	8.97E-01	0.99 (0.97, 1.01)	4.77E-01
**2.93E-02**	0.98 (0.92, 1.05)	8.16E-01	0.99 (0.97, 1.01)	2.78E-01
**7.29E-03**	0.97 (0.91, 1.04)	6.08E-01	0.97 (0.95, 0.99)	**8.51E-03**
**1.24E-02**	0.98 (0.91, 1.04)	6.62E-01	0.98 (0.96, 1.00)	**3.80E-02**
**2.61E-02**	0.98 (0.92, 1.05)	7.84E-01	0.99 (0.97, 1.01)	2.33E-01
**3.60E-03**	0.96 (0.90, 1.03)	4.70E-01	0.96 (0.94, 0.98)	**2.91E-04**
2.42E-01	1.00 (0.94, 1.07)	9.78E-01	1.01 (0.99, 1.03)	5.23E-01
2.10E-01	1.01 (0.95, 1.08)	8.44E-01	1.02 (1.00, 1.04)	5.81E-02
3.28E-01	1.02 (0.95, 1.09)	7.98E-01	1.03 (1.01, 1.05)	**1.86E-02**
5.40E-01	1.03 (0.97, 1.10)	5.93E-01	1.04 (1.02, 1.06)	**5.04E-04**
1.36E-01	1.00 (0.93, 1.06)	9.78E-01	1.01 (0.98, 1.03)	6.71E-01
9.89E-02	0.99 (0.92, 1.06)	8.57E-01	1.00 (0.98, 1.02)	8.63E-01

2.87E-01	1.02 (0.96, 1.09)	7.65E-01	1.02 (1.00, 1.04)	5.30E-02
9.32E-01	1.04 (0.97, 1.11)	4.96E-01	1.05 (1.03, 1.07)	**7.96E-06**
**1.02E-03**	0.94 (0.88, 1.01)	1.94E-01	0.96 (0.95, 0.99)	**1.52E-03**
**2.89E-03**	0.95 (0.88, 1.01)	2.28E-01	0.96 (0.94, 0.98)	**9.70E-05**
**9.80E-03**	0.96 (0.90, 1.03)	4.99E-01	0.97 (0.95, 0.99)	**5.42E-03**
**1.29E-03**	0.93 (0.87, 1.00)	1.32E-01	0.94 (0.92, 0.96)	**3.84E-07**
**7.69E-04**	0.93 (0.87, 0.99)	1.15E-01	0.94 (0.92, 0.96)	**7.63E-08**
**6.61E-03**	0.95 (0.89, 1.02)	3.47E-01	0.95 (0.93, 0.98)	**4.18E-05**
9.03E-01	1.04 (0.97, 1.11)	4.48E-01	1.06 (1.04, 1.08)	**1.97E-07**
**1.59E-03**	0.95 (0.89, 1.01)	2.24E-01	0.95 (0.93, 0.97)	**8.89E-07**
**9.34E-05**	0.93 (0.87, 1.00)	1.26E-01	0.93 (0.91, 0.95)	**4.95E-11**
**8.34E-05**	0.93 (0.87, 0.99)	1.25E-01	0.93 (0.91, 0.95)	**2.94E-11**
**6.46E-05**	0.93 (0.87, 0.99)	1.15E-01	0.92 (0.90, 0.94)	**6.56E-13**
**5.19E-05**	0.93 (0.87, 0.99)	1.15E-01	0.92 (0.90, 0.94)	**9.18E-13**
**1.61E-04**	0.93 (0.87, 0.99)	1.15E-01	0.92 (0.90, 0.94)	**8.71E-13**
7.07E-01	1.03 (0.97, 1.10)	5.81E-01	1.04 (1.02, 1.07)	**5.08E-05**
**1.70E-04**	0.97 (0.91, 1.04)	6.38E-01	0.95 (0.93, 0.97)	**5.48E-07**
**2.11E-05**	0.94 (0.88, 1.00)	1.34E-01	0.93 (0.91, 0.95)	**1.49E-11**
**1.79E-05**	0.93 (0.87, 0.99)	1.08E-01	0.92 (0.90, 0.94)	**7.27E-13**
**1.74E-05**	0.93 (0.88, 1.00)	1.32E-01	0.92 (0.90, 0.94)	**9.18E-13**
**2.01E-05**	0.94 (0.88, 1.00)	1.69E-01	0.93 (0.91, 0.95)	**6.95E-11**
**2.11E-05**	0.92 (0.86, 0.98)	9.61E-02	0.91 (0.89, 0.93)	**4.78E-17**
3.42E-01	1.02 (0.95, 1.09)	7.84E-01	1.03 (1.01, 1.05)	**1.70E-02**
**2.79E-04**	0.97 (0.91, 1.03)	5.03E-01	0.95 (0.93, 0.97)	**1.84E-05**
**4.27E-05**	0.94 (0.88, 1.00)	1.86E-01	0.94 (0.92, 0.95)	**1.29E-09**
**8.58E-05**	0.95 (0.89, 1.01)	2.47E-01	0.94 (0.92, 0.96)	**1.82E-08**
**2.69E-05**	0.94 (0.88, 1.00)	1.34E-01	0.93 (0.91, 0.95)	**1.02E-11**
**3.98E-05**	0.94 (0.88, 1.00)	1.59E-01	0.94 (0.92, 0.96)	**1.66E-09**
**3.98E-05**	0.93 (0.87, 1.00)	1.28E-01	0.91 (0.90, 0.93)	**3.84E-16**
2.37E-01	1.01 (0.94, 1.07)	9.24E-01	1.02 (1.00, 1.04)	8.94E-02
**2.80E-02**	1.03 (0.96, 1.11)	5.79E-01	1.03 (1.00, 1.05)	**3.09E-02**
**9.49E-03**	1.04 (0.97, 1.11)	4.85E-01	1.03 (1.01, 1.06)	**6.23E-03**
**3.90E-03**	1.04 (0.97, 1.12)	4.12E-01	1.04 (1.02, 1.07)	**7.10E-04**
**2.80E-02**	1.03 (0.96, 1.10)	6.44E-01	1.02 (1.00, 1.04)	1.21E-01
**2.93E-02**	1.02 (0.95, 1.09)	7.86E-01	1.01 (0.99, 1.04)	3.72E-01
**2.86E-02**	1.06 (0.99, 1.13)	2.03E-01	1.05 (1.02, 1.07)	**1.04E-04**
5.76E-01	1.04 (0.98, 1.11)	3.93E-01	1.05 (1.03, 1.07)	**5.59E-06**
**3.33E-02**	0.99 (0.92, 1.07)	9.08E-01	1.00 (0.98, 1.03)	8.15E-01
**2.93E-02**	0.99 (0.92, 1.07)	9.25E-01	1.01 (0.98, 1.03)	5.58E-01
**3.31E-02**	0.99 (0.92, 1.07)	9.19E-01	1.01 (0.99, 1.04)	3.76E-01
**3.16E-02**	0.99 (0.92, 1.06)	8.97E-01	1.00 (0.98, 1.02)	9.40E-01
**3.16E-02**	0.99 (0.92, 1.06)	8.49E-01	0.99 (0.97, 1.02)	7.16E-01
**3.31E-02**	1.00 (0.93, 1.08)	9.90E-01	1.01 (0.99, 1.04)	3.57E-01
1.41E-01	1.04 (0.97, 1.11)	4.85E-01	1.06 (1.04, 1.08)	**4.24E-08**
9.13E-01	0.91 (0.84, 0.98)	7.83E-02	0.97 (0.94, 0.99)	**4.12E-03**
9.03E-01	0.91 (0.84, 0.98)	9.24E-02	0.97 (0.95, 0.99)	**1.93E-02**
8.92E-01	0.92 (0.85, 0.99)	1.08E-01	0.98 (0.96, 1.00)	1.05E-01
9.32E-01	0.90 (0.84, 0.97)	5.88E-02	0.96 (0.93, 0.98)	**2.39E-04**

9.32E-01	0.90 (0.84, 0.97)	5.70E-02	0.95 (0.93, 0.98)	**9.09E-05**
9.32E-01	0.92 (0.85, 0.99)	1.15E-01	0.97 (0.95, 0.99)	**8.88E-03**
8.71E-01	1.01 (0.94, 1.08)	9.45E-01	1.04 (1.02, 1.06)	**1.07E-04**
**8.34E-05**	0.87 (0.81, 0.93)	**1.82E-03**	0.91 (0.89, 0.93)	**1.59E-18**
**2.04E-03**	0.89 (0.83, 0.95)	**1.27E-02**	0.94 (0.92, 0.96)	**5.84E-09**
**9.69E-03**	0.90 (0.84, 0.96)	**3.12E-02**	0.95 (0.93, 0.97)	**1.26E-05**
**1.96E-04**	0.87 (0.81, 0.93)	**1.40E-03**	0.90 (0.88, 0.92)	**1.26E-19**
**1.02E-04**	0.86 (0.81, 0.92)	**6.41E-04**	0.90 (0.88, 0.92)	**7.94E-23**
**7.02E-03**	0.90 (0.84, 0.97)	5.45E-02	0.94 (0.92, 0.96)	**3.28E-08**
2.64E-01	1.01 (0.94, 1.08)	9.24E-01	1.03 (1.01, 1.05)	**1.77E-02**
5.87E-02	1.05 (0.97, 1.13)	4.35E-01	1.04 (1.01, 1.06)	**2.95E-03**
**2.60E-02**	1.01 (0.95, 1.09)	8.44E-01	1.00 (0.98, 1.03)	7.59E-01
1.25E-01	1.02 (0.95, 1.09)	8.41E-01	1.00 (0.98, 1.02)	9.87E-01
**2.77E-03**	1.01 (0.94, 1.08)	9.14E-01	1.01 (0.99, 1.03)	3.93E-01
**1.03E-02**	0.98 (0.91, 1.04)	6.79E-01	0.99 (0.96, 1.01)	2.54E-01
1.25E-01	1.00 (0.93, 1.08)	9.78E-01	0.99 (0.96, 1.01)	2.65E-01
2.57E-01	0.99 (0.92, 1.07)	9.24E-01	0.98 (0.95, 1.00)	7.28E-02
**8.25E-03**	1.04 (0.98, 1.12)	3.93E-01	1.02 (1.00, 1.04)	7.94E-02
1.57E-01	0.97 (0.91, 1.04)	6.44E-01	1.00 (0.98, 1.02)	8.93E-01
4.19E-01	1.09 (1.01, 1.19)	1.25E-01	1.06 (1.04, 1.09)	**3.20E-06**
1.20E-01	1.07 (1.00, 1.14)	1.29E-01	1.03 (1.00, 1.05)	**3.04E-02**
**4.84E-02**	1.05 (0.99, 1.13)	2.59E-01	1.02 (1.00, 1.04)	1.37E-01
9.09E-01	1.11 (1.03, 1.19)	5.70E-02	1.04 (1.02, 1.06)	**1.21E-03**
5.39E-01	0.91 (0.85, 0.97)	5.84E-02	0.95 (0.93, 0.97)	**3.09E-05**
5.91E-01	1.01 (0.94, 1.07)	9.52E-01	0.99 (0.97, 1.01)	4.01E-01
9.46E-01	0.98 (0.92, 1.05)	7.82E-01	0.97 (0.95, 0.99)	**1.24E-02**
8.86E-01	0.97 (0.91, 1.04)	6.44E-01	0.97 (0.95, 0.99)	**5.58E-03**
8.32E-01	1.00 (0.94, 1.07)	9.61E-01	0.98 (0.96, 1.00)	9.57E-02
9.34E-01	1.01 (0.95, 1.08)	8.44E-01	1.03 (1.00, 1.05)	**3.03E-02**
**3.00E-02**	0.94 (0.88, 1.00)	1.68E-01	0.93 (0.91, 0.95)	**2.31E-10**
2.42E-01	0.97 (0.91, 1.04)	6.31E-01	0.97 (0.95, 0.99)	**2.48E-03**
6.50E-01	1.00 (0.94, 1.07)	9.90E-01	1.00 (0.98, 1.02)	7.16E-01
5.12E-02	0.94 (0.88, 1.01)	1.89E-01	0.93 (0.91, 0.95)	**3.78E-10**
1.36E-01	1.04 (0.97, 1.11)	4.48E-01	1.05 (1.03, 1.07)	**4.46E-05**
**3.77E-03**	1.15 (1.07, 1.23)	**2.54E-03**	1.12 (1.09, 1.14)	**1.47E-23**
7.78E-02	0.94 (0.88, 1.01)	2.02E-01	0.94 (0.92, 0.96)	**1.95E-07**
6.25E-02	0.93 (0.87, 1.00)	1.34E-01	0.94 (0.92, 0.96)	**3.79E-08**
5.40E-01	1.02 (0.96, 1.09)	6.79E-01	0.98 (0.96, 1.00)	**4.27E-02**
1.91E-01	1.04 (0.97, 1.11)	4.70E-01	1.04 (1.02, 1.06)	**2.98E-04**
**9.23E-04**	1.17 (1.10, 1.25)	**1.61E-04**	1.10 (1.07, 1.12)	**1.73E-17**
**1.59E-04**	0.89 (0.84, 0.95)	**5.58E-03**	0.91 (0.89, 0.92)	**8.33E-22**
**1.66E-05**	0.86 (0.81, 0.91)	**1.59E-04**	0.89 (0.88, 0.91)	**6.84E-27**
7.51E-01	1.02 (0.95, 1.08)	7.84E-01	0.98 (0.96, 1.00)	**2.32E-02**
**1.02E-03**	1.09 (1.02, 1.16)	5.84E-02	1.09 (1.07, 1.11)	**4.61E-17**
1.89E-01	1.00 (0.94, 1.06)	9.93E-01	1.02 (1.00, 1.04)	1.71E-01
**3.57E-07**	0.95 (0.91, 0.99)	9.61E-02	0.93 (0.92, 0.94)	**1.47E-30**
**1.52E-07**	0.95 (0.92, 0.99)	1.08E-01	0.93 (0.92, 0.94)	**9.44E-28**
6.46E-02	0.96 (0.90, 1.02)	3.80E-01	0.95 (0.93, 0.97)	**3.92E-07**

**4.18E-06**	1.07 (1.02, 1.13)	5.88E-02	1.09 (1.07, 1.10)	**2.83E-28**
4.98E-01	0.92 (0.86, 0.99)	1.15E-01	0.98 (0.96, 1.00)	1.42E-01
**1.18E-05**	0.97 (0.92, 1.01)	2.78E-01	0.93 (0.92, 0.94)	**6.26E-23**
**2.46E-04**	0.99 (0.93, 1.05)	8.97E-01	0.97 (0.95, 0.99)	**2.97E-03**
7.39E-01	0.96 (0.90, 1.02)	3.80E-01	0.94 (0.92, 0.96)	**5.49E-08**
**1.18E-05**	1.06 (1.02, 1.10)	5.84E-02	1.09 (1.08, 1.11)	**1.47E-30**
**9.74E-03**	1.04 (0.98, 1.10)	3.87E-01	1.05 (1.03, 1.07)	**1.83E-05**
**2.11E-05**	0.94 (0.90, 0.99)	7.83E-02	0.91 (0.89, 0.93)	**7.98E-26**
**8.34E-03**	0.96 (0.90, 1.01)	2.78E-01	0.96 (0.94, 0.98)	**5.95E-04**
1.86E-01	0.96 (0.90, 1.03)	4.48E-01	0.93 (0.92, 0.95)	**5.20E-11**
**4.57E-02**	1.05 (0.99, 1.11)	1.94E-01	1.07 (1.05, 1.09)	**1.17E-12**
**1.85E-03**	1.04 (0.97, 1.11)	5.09E-01	1.06 (1.04, 1.09)	**2.01E-07**
**2.37E-03**	0.94 (0.88, 1.01)	1.94E-01	0.92 (0.90, 0.94)	**1.10E-12**
1.57E-01	0.94 (0.88, 0.99)	1.25E-01	0.92 (0.90, 0.94)	**4.22E-18**
**8.01E-03**	0.99 (0.93, 1.06)	9.61E-01	0.99 (0.97, 1.01)	3.36E-01
1.67E-01	1.01 (0.96, 1.07)	8.64E-01	1.00 (0.98, 1.02)	9.42E-01
1.36E-01	1.02 (0.96, 1.08)	7.86E-01	1.03 (1.01, 1.05)	**1.12E-02**
2.78E-01	0.98 (0.92, 1.04)	6.44E-01	0.97 (0.95, 0.99)	**3.47E-03**
2.85E-01	0.97 (0.91, 1.03)	4.70E-01	0.96 (0.94, 0.98)	**8.51E-05**
4.78E-01	1.02 (0.96, 1.09)	6.65E-01	1.02 (1.00, 1.04)	**4.41E-02**
6.80E-01	1.03 (0.97, 1.09)	6.31E-01	1.03 (1.01, 1.05)	**1.14E-02**
8.50E-01	1.07 (1.00, 1.14)	1.59E-01	1.09 (1.07, 1.11)	**3.83E-14**
8.50E-01	0.94 (0.88, 1.00)	1.63E-01	0.92 (0.91, 0.94)	**1.59E-12**
9.03E-01	0.94 (0.88, 1.01)	1.94E-01	0.93 (0.91, 0.95)	**3.36E-12**
6.74E-01	0.95 (0.88, 1.02)	2.90E-01	0.95 (0.93, 0.97)	**5.78E-05**
8.56E-01	1.06 (0.99, 1.13)	1.99E-01	1.07 (1.05, 1.09)	**1.81E-10**
**5.89E-03**	1.06 (0.99, 1.13)	2.68E-01	1.10 (1.08, 1.13)	**1.58E-18**
**4.26E-02**	0.92 (0.86, 0.98)	9.61E-02	0.90 (0.88, 0.92)	**7.59E-24**
**2.14E-02**	0.91 (0.85, 0.97)	5.70E-02	0.90 (0.88, 0.91)	**4.56E-25**
3.28E-01	1.04 (0.98, 1.11)	3.98E-01	1.01 (0.99, 1.03)	4.84E-01
7.77E-01	1.08 (1.01, 1.15)	1.15E-01	1.08 (1.05, 1.10)	**8.52E-11**
**5.57E-04**	0.97 (0.91, 1.04)	6.31E-01	0.97 (0.95, 0.99)	**1.39E-02**
3.57E-01	0.93 (0.87, 0.99)	1.26E-01	0.98 (0.96, 1.00)	5.60E-02
**2.04E-03**	1.04 (0.97, 1.11)	5.03E-01	1.04 (1.02, 1.07)	**1.49E-03**
**1.08E-03**	0.92 (0.86, 0.99)	1.15E-01	0.93 (0.91, 0.95)	**1.07E-09**
**9.10E-03**	0.98 (0.91, 1.04)	6.62E-01	0.98 (0.96, 1.00)	1.11E-01
**3.42E-05**	0.93 (0.87, 0.99)	1.15E-01	0.92 (0.90, 0.94)	**4.86E-13**
**1.53E-03**	0.95 (0.89, 1.02)	3.07E-01	0.96 (0.94, 0.98)	**4.81E-04**
**1.02E-03**	0.92 (0.86, 0.98)	9.73E-02	0.93 (0.91, 0.95)	**1.30E-10**
**1.53E-03**	0.94 (0.88, 1.00)	1.59E-01	0.94 (0.92, 0.96)	**2.55E-07**
6.82E-02	1.00 (0.93, 1.06)	9.61E-01	1.01 (0.99, 1.03)	5.51E-01
**3.31E-02**	0.99 (0.93, 1.06)	8.76E-01	1.00 (0.98, 1.02)	8.63E-01
5.40E-01	1.02 (0.96, 1.09)	6.65E-01	1.04 (1.02, 1.06)	**7.86E-04**
9.32E-01	1.02 (0.95, 1.09)	7.97E-01	1.04 (1.02, 1.07)	**5.44E-05**
6.82E-02	0.93 (0.87, 1.00)	1.52E-01	0.97 (0.95, 0.99)	**6.94E-03**
2.23E-01	1.03 (0.96, 1.10)	6.40E-01	1.02 (1.00, 1.05)	**4.28E-02**

5.71E-02	0.92 (0.86, 0.99)	1.15E-01	0.96 (0.94, 0.98)	**7.01E-04**
1.67E-01	0.97 (0.91, 1.04)	6.48E-01	0.97 (0.95, 0.99)	**1.46E-02**
6.16E-02	0.93 (0.86, 1.00)	1.32E-01	0.96 (0.94, 0.98)	**1.07E-03**
8.50E-01	0.91 (0.84, 0.98)	9.61E-02	0.96 (0.94, 0.99)	**2.93E-03**
**7.12E-04**	0.95 (0.90, 1.02)	3.40E-01	0.95 (0.93, 0.97)	**1.79E-05**
**1.31E-02**	1.00 (0.93, 1.06)	9.78E-01	0.98 (0.96, 1.00)	7.51E-02
**2.84E-02**	0.97 (0.90, 1.03)	5.27E-01	0.99 (0.97, 1.01)	3.22E-01
**4.10E-02**	0.88 (0.82, 0.94)	**5.58E-03**	0.88 (0.86, 0.90)	**1.99E-28**
**4.26E-02**	0.90 (0.84, 0.97)	5.70E-02	0.90 (0.88, 0.92)	**9.02E-20**
**5.27E-03**	0.93 (0.87, 1.00)	1.29E-01	0.95 (0.93, 0.97)	**1.05E-06**
**8.57E-03**	0.90 (0.84, 0.97)	5.70E-02	0.91 (0.89, 0.93)	**1.71E-16**
**2.31E-03**	0.92 (0.86, 0.99)	1.15E-01	0.94 (0.92, 0.96)	**4.88E-07**
**9.09E-04**	0.91 (0.85, 0.97)	5.84E-02	0.93 (0.91, 0.95)	**4.29E-11**
2.91E-01	1.00 (0.93, 1.06)	9.78E-01	1.02 (1.00, 1.04)	**3.85E-02**
6.82E-02	1.00 (0.93, 1.06)	9.61E-01	1.01 (0.99, 1.03)	4.38E-01
6.26E-01	0.91 (0.85, 0.98)	9.61E-02	0.90 (0.88, 0.92)	**4.98E-19**
3.22E-01	0.93 (0.87, 0.99)	1.26E-01	0.93 (0.91, 0.95)	**6.74E-11**
6.82E-02	0.90 (0.84, 0.97)	5.45E-02	0.89 (0.87, 0.91)	**2.64E-21**
9.83E-01	0.94 (0.88, 1.01)	1.93E-01	0.94 (0.92, 0.96)	**7.87E-08**
4.89E-01	0.91 (0.85, 0.97)	5.40E-02	0.91 (0.89, 0.92)	**7.83E-20**
3.75E-01	1.07 (1.00, 1.14)	1.52E-01	1.10 (1.07, 1.12)	**8.71E-16**
8.62E-01	1.10 (1.03, 1.18)	5.21E-02	1.07 (1.05, 1.09)	**6.08E-11**
6.68E-02	1.07 (1.01, 1.13)	1.15E-01	1.06 (1.04, 1.08)	**6.59E-10**
5.50E-01	1.00 (0.94, 1.07)	9.90E-01	1.03 (1.01, 1.05)	**1.32E-02**
9.21E-02	1.00 (0.94, 1.07)	9.61E-01	1.05 (1.02, 1.07)	**3.59E-05**
5.40E-01	0.99 (0.93, 1.06)	9.52E-01	0.98 (0.96, 1.00)	**4.47E-02**
**1.97E-02**	0.96 (0.90, 1.02)	3.93E-01	0.94 (0.92, 0.96)	**1.73E-07**
6.72E-01	0.97 (0.90, 1.04)	5.45E-01	0.95 (0.93, 0.97)	**1.33E-05**
5.40E-01	0.98 (0.92, 1.05)	7.66E-01	0.99 (0.97, 1.02)	6.66E-01
**1.36E-02**	0.94 (0.88, 1.01)	1.89E-01	0.97 (0.95, 0.99)	**7.43E-03**
**1.33E-02**	0.93 (0.87, 1.00)	1.44E-01	0.97 (0.95, 0.99)	**2.27E-02**
7.00E-01	1.07 (1.01, 1.14)	1.25E-01	1.03 (1.01, 1.05)	**2.83E-03**
5.91E-01	1.04 (0.98, 1.11)	3.93E-01	1.02 (1.00, 1.04)	1.57E-01
**4.54E-05**	0.98 (0.90, 1.06)	7.65E-01	1.04 (1.03, 1.05)	**1.06E-08**
**1.10E-02**	1.03 (0.97, 1.10)	5.14E-01	1.05 (1.03, 1.06)	**4.41E-10**
**1.33E-02**	1.02 (0.95, 1.08)	7.97E-01	1.05 (1.04, 1.07)	**1.89E-09**
**2.88E-03**	1.05 (1.01, 1.09)	9.67E-02	1.02 (1.00, 1.04)	**2.20E-02**
**2.46E-02**	0.83 (0.78, 0.88)	**5.96E-06**	0.91 (0.89, 0.93)	**2.73E-18**
**3.11E-02**	1.16 (1.08, 1.24)	**6.41E-04**	1.14 (1.11, 1.16)	**1.73E-31**
1.10E-01	1.00 (0.93, 1.07)	9.90E-01	0.98 (0.96, 1.00)	1.19E-01
**2.84E-02**	0.94 (0.88, 1.01)	2.03E-01	0.98 (0.96, 1.00)	**4.27E-02**
**1.58E-04**	0.94 (0.88, 1.00)	1.66E-01	0.94 (0.92, 0.96)	**3.21E-08**
**4.56E-05**	0.93 (0.87, 0.99)	1.15E-01	0.93 (0.91, 0.94)	**2.55E-12**
**3.26E-05**	0.93 (0.87, 0.99)	1.25E-01	0.93 (0.91, 0.94)	**2.68E-12**
6.89E-01	0.92 (0.85, 0.99)	1.15E-01	0.95 (0.93, 0.98)	**2.41E-04**
5.46E-01	0.91 (0.85, 0.98)	7.83E-02	0.90 (0.88, 0.92)	**3.01E-19**
6.84E-02	1.10 (1.05, 1.17)	**9.18E-03**	1.07 (1.06, 1.09)	**1.10E-18**
**8.34E-03**	0.98 (0.92, 1.04)	7.10E-01	0.98 (0.96, 1.01)	1.84E-01

**6.63E-05**	0.93 (0.87, 0.99)	1.15E-01	0.92 (0.90, 0.94)	**1.90E-14**
6.00E-01	0.95 (0.88, 1.03)	3.80E-01	0.98 (0.96, 1.01)	1.71E-01
7.91E-01	0.99 (0.92, 1.06)	8.97E-01	1.03 (1.01, 1.05)	**5.10E-03**
**9.15E-05**	1.00 (0.94, 1.07)	9.90E-01	1.04 (1.03, 1.06)	**1.52E-06**
**3.57E-02**	0.88 (0.82, 0.95)	**1.10E-02**	0.93 (0.91, 0.95)	**1.99E-10**
**2.03E-02**	0.99 (0.92, 1.05)	8.44E-01	0.99 (0.97, 1.01)	5.24E-01
**5.96E-04**	0.95 (0.90, 1.02)	3.40E-01	0.95 (0.93, 0.97)	**6.34E-07**
1.17E-01	0.88 (0.82, 0.95)	**1.20E-02**	0.93 (0.91, 0.95)	**3.83E-09**
**2.00E-02**	0.93 (0.87, 1.00)	1.31E-01	0.96 (0.94, 0.98)	**6.63E-04**
**1.46E-02**	0.98 (0.92, 1.05)	7.97E-01	0.99 (0.97, 1.01)	5.51E-01
**3.98E-05**	0.93 (0.87, 0.99)	1.22E-01	0.93 (0.91, 0.95)	**1.09E-11**
6.84E-01	0.93 (0.86, 1.01)	1.88E-01	0.99 (0.96, 1.01)	3.07E-01
**1.83E-03**	0.94 (0.88, 1.00)	1.66E-01	0.95 (0.94, 0.98)	**4.37E-05**
**1.39E-02**	0.98 (0.92, 1.05)	7.87E-01	0.99 (0.97, 1.01)	5.00E-01
**4.54E-05**	0.93 (0.87, 1.00)	1.29E-01	0.93 (0.91, 0.95)	**2.94E-11**
6.80E-01	0.93 (0.86, 1.00)	1.64E-01	0.98 (0.95, 1.00)	7.56E-02
**1.12E-02**	0.97 (0.91, 1.04)	6.44E-01	0.98 (0.96, 1.00)	7.95E-02
6.74E-01	0.92 (0.86, 1.00)	1.34E-01	0.96 (0.94, 0.98)	**1.44E-03**
8.49E-01	1.10 (1.01, 1.19)	1.15E-01	1.04 (1.01, 1.06)	**3.90E-03**

Other mortality			
Never/former drinker		Current drinker	
Adjusted HR (95% CI)	P value	Adjusted HR (95% CI)	P value
0.87 (0.81, 0.93)	**6.63E-05**	0.94 (0.91, 0.96)	**6.16E-07**
0.87 (0.81, 0.93)	**6.63E-05**	0.94 (0.91, 0.96)	**5.00E-07**
0.86 (0.81, 0.92)	**4.46E-05**	0.94 (0.91, 0.96)	**2.17E-06**
0.85 (0.79, 0.91)	**6.00E-06**	0.93 (0.90, 0.95)	**2.40E-08**
0.84 (0.79, 0.90)	**3.10E-06**	0.92 (0.90, 0.95)	**4.07E-09**
0.86 (0.80, 0.92)	**1.86E-05**	0.93 (0.91, 0.96)	**2.74E-07**
0.87 (0.82, 0.94)	**1.90E-04**	0.94 (0.91, 0.96)	**1.21E-06**
0.82 (0.76, 0.87)	**2.35E-08**	0.88 (0.86, 0.90)	**5.67E-21**
0.82 (0.76, 0.87)	**1.97E-08**	0.88 (0.85, 0.90)	**1.62E-21**
0.82 (0.76, 0.87)	**2.28E-08**	0.88 (0.86, 0.90)	**2.42E-20**
0.79 (0.74, 0.85)	**1.38E-10**	0.86 (0.83, 0.88)	**6.71E-29**
0.78 (0.73, 0.83)	**1.09E-11**	0.84 (0.82, 0.87)	**1.65E-34**
0.81 (0.76, 0.87)	**7.04E-09**	0.88 (0.85, 0.90)	**5.32E-22**
0.83 (0.77, 0.89)	**2.59E-07**	0.89 (0.86, 0.91)	**1.18E-18**
0.80 (0.75, 0.86)	**1.07E-09**	0.86 (0.83, 0.88)	**7.93E-29**
0.79 (0.74, 0.85)	**1.85E-10**	0.84 (0.82, 0.87)	**4.94E-34**
0.80 (0.75, 0.86)	**9.70E-10**	0.86 (0.84, 0.88)	**2.77E-27**
0.78 (0.73, 0.84)	**2.11E-11**	0.84 (0.82, 0.87)	**5.08E-35**
0.78 (0.73, 0.84)	**1.80E-11**	0.84 (0.82, 0.86)	**8.05E-36**
0.79 (0.73, 0.84)	**7.04E-11**	0.85 (0.82, 0.87)	**1.66E-32**
0.80 (0.74, 0.86)	**1.09E-09**	0.84 (0.82, 0.87)	**2.30E-34**
0.78 (0.73, 0.84)	**9.07E-12**	0.83 (0.81, 0.85)	**1.22E-41**
0.77 (0.72, 0.83)	**1.58E-12**	0.82 (0.80, 0.85)	**3.84E-45**
0.79 (0.74, 0.84)	**2.31E-11**	0.84 (0.82, 0.86)	**9.11E-39**
0.82 (0.77, 0.88)	**1.20E-08**	0.86 (0.83, 0.88)	**1.14E-30**
0.85 (0.79, 0.90)	**1.61E-06**	0.88 (0.85, 0.90)	**3.03E-23**
0.80 (0.74, 0.85)	**1.42E-10**	0.84 (0.82, 0.86)	**4.60E-37**
0.78 (0.73, 0.84)	**3.41E-11**	0.83 (0.81, 0.86)	**3.60E-39**
0.81 (0.76, 0.87)	**4.08E-09**	0.87 (0.85, 0.90)	**7.44E-23**
0.80 (0.75, 0.86)	**8.77E-10**	0.87 (0.85, 0.89)	**6.42E-25**
0.80 (0.75, 0.85)	**2.87E-10**	0.86 (0.83, 0.88)	**1.36E-30**
0.81 (0.76, 0.87)	**3.10E-09**	0.87 (0.85, 0.89)	**7.84E-25**
0.82 (0.76, 0.87)	**9.63E-09**	0.88 (0.86, 0.90)	**1.01E-20**
0.81 (0.75, 0.86)	**8.67E-10**	0.85 (0.83, 0.88)	**7.63E-32**
0.84 (0.78, 0.89)	**6.11E-07**	0.90 (0.88, 0.92)	**1.43E-14**
0.92 (0.86, 0.98)	**1.69E-02**	0.98 (0.96, 1.01)	1.69E-01
0.92 (0.86, 0.98)	**2.27E-02**	0.99 (0.97, 1.02)	4.73E-01
0.94 (0.88, 1.01)	9.56E-02	1.01 (0.98, 1.03)	6.24E-01
0.92 (0.86, 0.98)	**2.22E-02**	0.98 (0.95, 1.00)	7.62E-02
0.92 (0.86, 0.98)	**1.89E-02**	0.97 (0.95, 1.00)	**4.33E-02**

0.93 (0.87, 0.99)	**4.20E-02**	0.99 (0.96, 1.01)	2.69E-01
0.93 (0.87, 0.99)	**4.37E-02**	1.01 (0.98, 1.03)	7.05E-01
0.87 (0.81, 0.93)	**4.31E-05**	0.91 (0.89, 0.93)	**3.11E-13**
0.88 (0.82, 0.94)	**2.41E-04**	0.92 (0.90, 0.95)	**2.23E-09**
0.89 (0.83, 0.95)	**6.46E-04**	0.94 (0.91, 0.96)	**8.84E-07**
0.88 (0.82, 0.94)	**1.63E-04**	0.91 (0.89, 0.93)	**3.11E-12**
0.87 (0.81, 0.93)	**5.55E-05**	0.91 (0.88, 0.93)	**1.93E-13**
0.90 (0.84, 0.96)	**2.98E-03**	0.93 (0.90, 0.95)	**1.03E-08**
0.96 (0.90, 1.03)	3.26E-01	1.04 (1.02, 1.07)	**1.22E-03**
0.82 (0.77, 0.88)	**1.63E-08**	0.86 (0.84, 0.89)	**2.17E-28**
0.81 (0.76, 0.87)	**1.77E-09**	0.85 (0.83, 0.88)	**5.60E-32**
0.81 (0.76, 0.87)	**2.37E-09**	0.85 (0.83, 0.88)	**5.13E-33**
0.81 (0.76, 0.86)	**7.74E-10**	0.85 (0.82, 0.87)	**2.82E-35**
0.80 (0.75, 0.85)	**1.10E-10**	0.84 (0.82, 0.86)	**5.60E-38**
0.84 (0.79, 0.89)	**3.00E-07**	0.87 (0.84, 0.89)	**7.99E-27**
0.96 (0.90, 1.02)	2.12E-01	1.03 (1.00, 1.05)	**3.47E-02**
0.80 (0.75, 0.85)	**1.77E-10**	0.83 (0.81, 0.86)	**3.78E-41**
0.77 (0.73, 0.83)	**4.76E-13**	0.82 (0.80, 0.84)	**3.07E-51**
0.77 (0.72, 0.83)	**4.15E-13**	0.81 (0.79, 0.84)	**5.98E-52**
0.77 (0.72, 0.82)	**2.07E-13**	0.81 (0.79, 0.83)	**4.67E-55**
0.77 (0.72, 0.82)	**1.09E-13**	0.81 (0.79, 0.83)	**4.37E-55**
0.80 (0.75, 0.85)	**4.55E-11**	0.82 (0.80, 0.85)	**1.22E-47**
0.91 (0.85, 0.97)	**9.46E-03**	0.98 (0.96, 1.01)	1.40E-01
0.82 (0.77, 0.87)	**4.72E-09**	0.86 (0.83, 0.88)	**1.04E-31**
0.80 (0.75, 0.86)	**1.59E-10**	0.83 (0.81, 0.86)	**7.87E-42**
0.84 (0.79, 0.89)	**1.56E-07**	0.86 (0.84, 0.88)	**1.21E-31**
0.79 (0.74, 0.84)	**1.80E-11**	0.82 (0.80, 0.84)	**4.46E-48**
0.78 (0.73, 0.84)	**4.07E-12**	0.82 (0.80, 0.85)	**1.73E-46**
0.83 (0.78, 0.89)	**4.99E-08**	0.84 (0.82, 0.86)	**2.96E-42**
0.88 (0.82, 0.94)	**2.57E-04**	0.95 (0.93, 0.97)	**7.39E-05**
1.24 (1.16, 1.31)	**3.89E-11**	1.20 (1.17, 1.23)	**1.73E-41**
1.27 (1.19, 1.34)	**1.66E-13**	1.22 (1.19, 1.25)	**3.30E-50**
1.28 (1.21, 1.36)	**6.35E-15**	1.23 (1.20, 1.26)	**7.93E-57**
1.25 (1.18, 1.33)	**5.83E-12**	1.19 (1.16, 1.22)	**3.08E-40**
1.24 (1.17, 1.32)	**2.43E-11**	1.19 (1.15, 1.22)	**3.14E-36**
1.26 (1.19, 1.33)	**1.30E-13**	1.21 (1.18, 1.24)	**2.74E-52**
0.99 (0.93, 1.06)	8.52E-01	1.06 (1.03, 1.08)	**1.08E-05**
1.24 (1.16, 1.32)	**7.66E-10**	1.18 (1.15, 1.21)	**5.83E-32**
1.25 (1.17, 1.33)	**1.75E-10**	1.19 (1.16, 1.22)	**5.54E-35**
1.25 (1.17, 1.33)	**2.57E-10**	1.19 (1.16, 1.22)	**6.68E-36**
1.25 (1.17, 1.33)	**1.49E-10**	1.18 (1.15, 1.21)	**1.61E-31**
1.24 (1.16, 1.33)	**2.25E-10**	1.17 (1.14, 1.20)	**1.42E-29**
1.25 (1.17, 1.33)	**5.45E-11**	1.20 (1.16, 1.23)	**7.48E-38**
1.04 (0.97, 1.11)	3.55E-01	1.11 (1.08, 1.14)	**9.25E-17**
0.99 (0.92, 1.07)	8.86E-01	1.03 (1.00, 1.06)	**4.87E-02**
0.98 (0.92, 1.06)	7.03E-01	1.03 (1.00, 1.06)	5.35E-02
0.97 (0.91, 1.04)	4.95E-01	1.03 (1.00, 1.06)	**4.95E-02**
1.02 (0.95, 1.09)	6.88E-01	1.03 (1.00, 1.06)	**4.78E-02**

1.02 (0.95, 1.09)	6.99E-01	1.02 (1.00, 1.05)	8.56E-02
1.02 (0.95, 1.10)	6.38E-01	1.04 (1.02, 1.07)	**2.69E-03**
0.89 (0.83, 0.96)	**1.65E-03**	1.00 (0.97, 1.02)	8.42E-01
0.75 (0.70, 0.80)	**6.24E-16**	0.82 (0.80, 0.84)	**1.23E-51**
0.77 (0.72, 0.83)	**6.47E-13**	0.86 (0.84, 0.88)	**9.85E-30**
0.79 (0.74, 0.85)	**1.10E-10**	0.89 (0.87, 0.91)	**5.34E-19**
0.77 (0.72, 0.82)	**5.09E-14**	0.83 (0.80, 0.85)	**3.68E-46**
0.76 (0.71, 0.81)	**5.77E-15**	0.81 (0.79, 0.83)	**1.98E-54**
0.82 (0.76, 0.87)	**1.20E-08**	0.89 (0.87, 0.92)	**1.24E-17**
0.84 (0.79, 0.90)	**2.52E-06**	0.93 (0.91, 0.96)	**4.03E-07**
1.00 (0.93, 1.08)	9.99E-01	1.02 (0.99, 1.05)	1.41E-01
1.05 (0.98, 1.12)	2.12E-01	1.04 (1.01, 1.07)	**6.08E-03**
1.03 (0.97, 1.10)	4.02E-01	1.03 (1.00, 1.06)	**2.92E-02**
1.06 (1.00, 1.13)	7.63E-02	1.04 (1.02, 1.07)	**1.65E-03**
0.96 (0.90, 1.03)	2.70E-01	0.96 (0.94, 0.99)	**3.72E-03**
1.11 (1.05, 1.19)	**1.57E-03**	1.07 (1.04, 1.10)	**1.51E-06**
1.10 (1.03, 1.18)	**4.64E-03**	1.05 (1.03, 1.08)	**2.80E-04**
1.13 (1.06, 1.20)	**3.28E-04**	1.12 (1.09, 1.15)	**4.50E-16**
0.91 (0.85, 0.97)	**3.92E-03**	0.94 (0.91, 0.96)	**3.87E-06**
0.91 (0.85, 0.97)	**4.97E-03**	0.96 (0.93, 0.99)	**3.45E-03**
1.11 (1.04, 1.18)	**2.29E-03**	1.09 (1.06, 1.11)	**3.00E-10**
1.13 (1.06, 1.20)	**2.76E-04**	1.10 (1.07, 1.13)	**7.79E-13**
1.02 (0.95, 1.09)	5.81E-01	1.03 (1.01, 1.06)	**2.30E-02**
0.97 (0.90, 1.03)	3.59E-01	0.96 (0.93, 0.98)	**1.89E-03**
1.04 (0.97, 1.10)	3.20E-01	1.02 (0.99, 1.04)	1.32E-01
1.08 (1.01, 1.15)	**2.55E-02**	1.03 (1.01, 1.06)	**2.11E-02**
1.09 (1.02, 1.16)	**1.74E-02**	1.03 (1.01, 1.06)	**1.48E-02**
1.06 (1.00, 1.13)	8.18E-02	1.02 (1.00, 1.05)	6.23E-02
0.93 (0.88, 0.99)	**4.59E-02**	0.97 (0.95, 1.00)	**3.05E-02**
0.99 (0.93, 1.06)	8.05E-01	0.94 (0.92, 0.97)	**9.51E-06**
1.00 (0.94, 1.06)	9.92E-01	0.98 (0.96, 1.00)	1.08E-01
1.01 (0.95, 1.07)	8.39E-01	1.01 (0.98, 1.03)	6.81E-01
0.99 (0.93, 1.06)	7.69E-01	0.95 (0.92, 0.97)	**3.34E-05**
1.00 (0.94, 1.07)	9.33E-01	1.03 (1.01, 1.06)	**1.35E-02**
1.15 (1.07, 1.22)	**8.41E-05**	1.14 (1.11, 1.17)	**5.98E-25**
0.99 (0.93, 1.06)	8.19E-01	0.96 (0.94, 0.99)	**3.57E-03**
0.99 (0.93, 1.05)	7.05E-01	0.96 (0.94, 0.99)	**3.68E-03**
1.03 (0.97, 1.09)	4.27E-01	0.97 (0.95, 1.00)	**2.11E-02**
0.98 (0.92, 1.05)	6.50E-01	1.01 (0.99, 1.04)	2.77E-01
1.08 (1.02, 1.15)	**1.47E-02**	1.12 (1.10, 1.15)	**7.64E-21**
0.93 (0.88, 0.99)	**2.04E-02**	0.89 (0.87, 0.91)	**1.10E-24**
0.89 (0.84, 0.95)	**2.36E-04**	0.87 (0.85, 0.89)	**1.41E-33**
1.05 (0.99, 1.12)	1.22E-01	0.98 (0.96, 1.01)	1.49E-01
1.06 (1.00, 1.12)	5.86E-02	1.10 (1.08, 1.13)	**6.27E-19**
1.04 (1.00, 1.09)	8.18E-02	1.03 (1.01, 1.05)	**1.81E-02**
0.93 (0.91, 0.96)	**2.96E-05**	0.91 (0.90, 0.92)	**5.75E-59**
0.92 (0.90, 0.94)	**4.08E-11**	0.91 (0.90, 0.92)	**8.17E-99**
1.04 (0.98, 1.11)	2.69E-01	0.98 (0.96, 1.00)	1.02E-01

1.09 (1.05, 1.13)	**5.58E-05**	1.12 (1.10, 1.13)	**2.27E-57**
1.05 (1.01, 1.10)	**3.18E-02**	1.05 (1.03, 1.08)	**1.06E-04**
0.94 (0.91, 0.96)	**3.90E-06**	0.90 (0.89, 0.91)	**1.55E-85**
0.90 (0.87, 0.92)	**2.31E-11**	0.85 (0.84, 0.87)	**1.13E-75**
1.05 (0.99, 1.12)	1.34E-01	0.99 (0.97, 1.02)	4.85E-01
1.07 (1.04, 1.11)	**8.54E-06**	1.13 (1.12, 1.15)	**2.08E-85**
1.13 (1.09, 1.16)	**7.87E-12**	1.18 (1.15, 1.21)	**2.84E-41**
0.92 (0.89, 0.94)	**7.86E-09**	0.86 (0.85, 0.87)	**2.68E-95**
0.88 (0.85, 0.91)	**5.83E-12**	0.86 (0.84, 0.88)	**1.41E-40**
1.02 (0.96, 1.08)	6.26E-01	0.94 (0.92, 0.96)	**2.39E-07**
1.02 (0.96, 1.08)	5.28E-01	1.08 (1.06, 1.10)	**1.26E-13**
1.31 (1.21, 1.42)	**1.43E-10**	1.21 (1.17, 1.24)	**3.28E-34**
0.82 (0.77, 0.88)	**1.09E-08**	0.84 (0.82, 0.86)	**4.13E-40**
0.93 (0.88, 0.98)	**1.46E-02**	0.91 (0.89, 0.93)	**2.55E-19**
0.82 (0.76, 0.89)	**1.82E-06**	0.88 (0.86, 0.91)	**3.43E-18**
0.87 (0.80, 0.93)	**2.23E-04**	0.95 (0.93, 0.98)	**1.18E-04**
0.93 (0.88, 0.98)	**1.43E-02**	0.96 (0.94, 0.98)	**1.49E-03**
1.12 (1.05, 1.20)	**5.26E-04**	1.04 (1.02, 1.07)	**5.90E-04**
1.11 (1.04, 1.18)	**1.82E-03**	1.03 (1.00, 1.05)	**4.01E-02**
1.13 (1.06, 1.21)	**2.98E-04**	1.11 (1.08, 1.14)	**1.24E-14**
0.88 (0.82, 0.94)	**3.47E-04**	0.96 (0.94, 0.99)	**4.98E-03**
0.95 (0.89, 1.02)	1.69E-01	1.02 (0.99, 1.04)	1.98E-01
1.07 (1.00, 1.14)	6.54E-02	1.00 (0.97, 1.02)	8.08E-01
1.06 (0.99, 1.13)	1.18E-01	0.99 (0.96, 1.01)	3.33E-01
1.08 (1.01, 1.16)	**4.20E-02**	1.04 (1.01, 1.07)	**3.49E-03**
0.93 (0.87, 0.99)	**4.37E-02**	0.99 (0.97, 1.02)	6.24E-01
1.17 (1.10, 1.25)	**3.44E-06**	1.21 (1.18, 1.24)	**1.84E-48**
0.93 (0.88, 0.99)	**4.43E-02**	0.89 (0.87, 0.91)	**8.70E-22**
0.89 (0.83, 0.94)	**3.10E-04**	0.86 (0.84, 0.89)	**6.93E-33**
1.18 (1.12, 1.25)	**1.13E-08**	1.12 (1.09, 1.14)	**7.04E-18**
0.96 (0.90, 1.03)	2.98E-01	1.01 (0.98, 1.03)	6.92E-01
0.78 (0.73, 0.83)	**1.39E-12**	0.82 (0.80, 0.84)	**1.54E-48**
1.01 (0.95, 1.07)	8.36E-01	1.01 (0.99, 1.04)	2.77E-01
1.34 (1.26, 1.42)	**2.28E-18**	1.26 (1.23, 1.29)	**4.40E-63**
0.84 (0.79, 0.90)	**6.60E-07**	0.88 (0.86, 0.91)	**1.42E-20**
0.81 (0.76, 0.86)	**1.89E-09**	0.87 (0.85, 0.89)	**2.17E-24**
0.79 (0.74, 0.85)	**4.08E-11**	0.83 (0.81, 0.85)	**2.40E-42**
0.83 (0.78, 0.88)	**5.84E-08**	0.88 (0.86, 0.90)	**1.38E-21**
0.84 (0.79, 0.90)	**1.01E-06**	0.88 (0.86, 0.91)	**4.97E-20**
0.84 (0.78, 0.89)	**3.05E-07**	0.88 (0.86, 0.90)	**3.29E-21**
**0.84 (0.78, 0.90)**	**8.94E-07**	**0.90 (0.88, 0.93)**	**4.23E-14**
**0.82 (0.77, 0.88)**	**5.84E-08**	**0.88 (0.86, 0.90)**	**1.51E-20**
0.93 (0.87, 1.00)	5.27E-02	1.00 (0.98, 1.03)	7.09E-01
0.91 (0.85, 0.97)	**6.70E-03**	1.00 (0.98, 1.03)	7.31E-01
0.88 (0.82, 0.94)	**6.42E-04**	0.95 (0.93, 0.98)	**5.98E-04**
0.85 (0.80, 0.91)	**7.21E-06**	0.91 (0.88, 0.93)	**1.96E-12**

0.89 (0.83, 0.96)	**1.99E-03**	0.95 (0.92, 0.98)	**2.48E-04**
0.94 (0.88, 1.01)	9.19E-02	0.98 (0.96, 1.01)	1.75E-01
0.88 (0.82, 0.94)	**5.79E-04**	0.95 (0.92, 0.97)	**1.23E-04**
1.00 (0.93, 1.07)	9.28E-01	1.03 (1.00, 1.06)	**4.84E-02**
0.82 (0.77, 0.87)	**8.05E-09**	0.86 (0.84, 0.88)	**4.04E-29**
0.85 (0.80, 0.91)	**3.15E-06**	0.88 (0.85, 0.90)	**1.48E-22**
0.84 (0.79, 0.90)	**9.01E-07**	0.92 (0.90, 0.94)	**1.11E-10**
0.89 (0.83, 0.95)	**6.65E-04**	0.85 (0.82, 0.87)	**2.34E-36**
0.85 (0.79, 0.91)	**3.32E-05**	0.86 (0.84, 0.89)	**7.05E-24**
0.82 (0.77, 0.88)	**7.05E-08**	0.86 (0.84, 0.88)	**7.11E-29**
0.79 (0.74, 0.85)	**3.87E-09**	0.83 (0.81, 0.86)	**1.73E-35**
0.81 (0.76, 0.87)	**8.66E-09**	0.86 (0.84, 0.88)	**2.11E-29**
0.79 (0.73, 0.84)	**8.30E-11**	0.83 (0.81, 0.86)	**7.94E-39**
0.88 (0.83, 0.94)	**3.53E-04**	0.96 (0.94, 0.99)	**5.29E-03**
0.87 (0.82, 0.94)	**1.70E-04**	0.96 (0.93, 0.98)	**1.05E-03**
0.95 (0.89, 1.02)	1.85E-01	0.91 (0.89, 0.93)	**8.77E-12**
0.97 (0.91, 1.03)	3.94E-01	0.91 (0.89, 0.93)	**2.20E-13**
0.85 (0.79, 0.91)	**2.13E-05**	0.84 (0.82, 0.87)	**3.89E-32**
1.04 (0.97, 1.11)	3.20E-01	0.96 (0.94, 0.98)	**1.84E-03**
0.98 (0.92, 1.04)	5.33E-01	0.91 (0.89, 0.93)	**1.02E-14**
0.99 (0.93, 1.05)	7.69E-01	1.06 (1.03, 1.08)	**3.34E-05**
1.06 (0.99, 1.13)	1.05E-01	1.12 (1.09, 1.14)	**1.17E-19**
1.17 (1.13, 1.22)	**2.07E-13**	1.09 (1.07, 1.11)	**1.67E-22**
1.00 (0.94, 1.07)	9.99E-01	1.07 (1.04, 1.09)	**5.25E-07**
1.10 (1.04, 1.17)	**3.19E-03**	1.08 (1.06, 1.10)	**2.70E-17**
1.02 (0.96, 1.08)	5.97E-01	0.97 (0.94, 0.99)	**1.19E-02**
0.97 (0.91, 1.03)	3.91E-01	0.95 (0.93, 0.97)	**5.67E-05**
0.89 (0.82, 0.95)	**2.13E-03**	0.87 (0.84, 0.89)	**4.52E-23**
0.87 (0.81, 0.93)	**2.00E-04**	0.93 (0.90, 0.95)	**7.98E-08**
0.80 (0.75, 0.86)	**2.55E-09**	0.85 (0.83, 0.88)	**6.62E-29**
0.78 (0.73, 0.84)	**2.43E-11**	0.85 (0.82, 0.87)	**2.65E-32**
1.08 (1.02, 1.15)	**1.09E-02**	1.04 (1.02, 1.06)	**8.86E-04**
1.01 (0.95, 1.08)	6.95E-01	1.03 (1.01, 1.06)	**1.87E-02**
1.06 (1.04, 1.09)	**3.10E-06**	1.09 (1.08, 1.10)	**1.26E-101**
1.05 (0.99, 1.11)	1.45E-01	1.07 (1.06, 1.08)	**1.08E-30**
1.05 (0.99, 1.11)	1.29E-01	1.10 (1.08, 1.11)	**3.95E-44**
1.08 (1.05, 1.10)	**5.02E-11**	1.05 (1.03, 1.06)	**6.32E-11**
0.72 (0.68, 0.77)	**2.34E-22**	0.81 (0.79, 0.83)	**8.46E-62**
1.02 (0.96, 1.09)	5.27E-01	1.10 (1.07, 1.13)	**1.10E-13**
1.07 (1.00, 1.14)	7.62E-02	1.02 (0.99, 1.05)	2.71E-01
0.80 (0.74, 0.85)	**8.27E-10**	0.86 (0.84, 0.89)	**5.50E-26**
0.81 (0.76, 0.86)	**8.67E-10**	0.85 (0.83, 0.88)	**1.62E-32**
0.81 (0.76, 0.87)	**1.39E-09**	0.84 (0.82, 0.87)	**4.06E-37**
0.79 (0.74, 0.84)	**7.05E-12**	0.83 (0.81, 0.85)	**1.39E-44**
1.09 (1.02, 1.17)	**2.02E-02**	1.06 (1.03, 1.09)	**2.33E-05**
1.00 (0.94, 1.07)	9.46E-01	0.93 (0.90, 0.95)	**4.19E-08**
1.15 (1.10, 1.20)	**5.81E-10**	1.11 (1.09, 1.12)	**6.14E-60**
0.80 (0.75, 0.86)	**6.08E-10**	0.86 (0.84, 0.89)	**9.53E-27**

0.83 (0.77, 0.88)	**1.96E-08**	0.85 (0.83, 0.87)	**5.76E-34**
1.11 (1.04, 1.19)	**4.70E-03**	1.10 (1.07, 1.13)	**8.00E-12**
1.05 (1.01, 1.09)	**2.57E-02**	1.06 (1.04, 1.07)	**9.61E-12**
1.06 (1.01, 1.13)	**3.40E-02**	1.09 (1.08, 1.10)	**5.94E-76**
0.86 (0.80, 0.93)	**8.44E-05**	0.92 (0.90, 0.95)	**1.16E-08**
0.82 (0.77, 0.88)	**2.59E-08**	0.88 (0.86, 0.91)	**9.81E-20**
0.81 (0.76, 0.87)	**2.13E-09**	0.85 (0.83, 0.87)	**2.26E-33**
0.89 (0.83, 0.96)	**2.29E-03**	0.94 (0.92, 0.97)	**5.07E-05**
0.86 (0.80, 0.92)	**3.43E-05**	0.93 (0.91, 0.96)	**2.06E-07**
0.81 (0.76, 0.87)	**2.91E-09**	0.88 (0.85, 0.90)	**1.34E-22**
0.80 (0.75, 0.86)	**1.85E-10**	0.84 (0.82, 0.86)	**3.26E-39**
1.06 (0.99, 1.14)	1.40E-01	1.08 (1.05, 1.11)	**6.91E-08**
0.82 (0.77, 0.88)	**1.68E-08**	0.88 (0.86, 0.91)	**1.04E-20**
0.80 (0.75, 0.86)	**1.61E-09**	0.87 (0.85, 0.89)	**1.73E-24**
0.80 (0.75, 0.85)	**1.07E-10**	0.84 (0.82, 0.86)	**9.11E-39**
1.07 (1.00, 1.15)	8.45E-02	1.08 (1.05, 1.11)	**2.23E-07**
0.82 (0.77, 0.87)	**1.18E-08**	0.88 (0.86, 0.90)	**1.76E-21**
1.10 (1.02, 1.18)	**1.38E-02**	1.07 (1.04, 1.10)	**1.21E-06**
0.99 (0.92, 1.06)	7.88E-01	1.00 (0.97, 1.03)	8.51E-01

Supplementary Table 13. Association of all metabolites with sleep duration

		CKB	
		Sleep duration	
**Metabolites**	**Group**	**Adjusted β (95% CI)**	**P value**
Concentration of chylomicrons	Lipoprotein subclasses	0.00 (-0.03, 0.04)	9.37E-01
Total lipids in chylomicrons ar	Lipoprotein subclasses	0.00 (-0.03, 0.04)	9.35E-01
Phospholipids in chylomicrons	Lipoprotein subclasses	0.00 (-0.03, 0.03)	9.95E-01
Cholesterol in chylomicrons ar	Lipoprotein subclasses	0.02 (-0.02, 0.05)	5.44E-01
Cholesteryl esters in chylomicr	Lipoprotein subclasses	0.03 (-0.01, 0.06)	2.10E-01
Free cholesterol in chylomicro	Lipoprotein subclasses	0.00 (-0.03, 0.03)	9.95E-01
Triglycerides in chylomicrons	Lipoprotein subclasses	0.00 (-0.03, 0.03)	9.68E-01
Concentration of very large VI	Lipoprotein subclasses	0.00 (-0.03, 0.03)	9.95E-01
Total lipids in very large VLD	Lipoprotein subclasses	0.00 (-0.03, 0.03)	9.95E-01
Phospholipids in very large VI	Lipoprotein subclasses	0.00 (-0.03, 0.04)	9.44E-01
Cholesterol in very large VLD	Lipoprotein subclasses	0.01 (-0.02, 0.04)	7.48E-01
Cholesteryl esters in very large	Lipoprotein subclasses	0.01 (-0.02, 0.05)	6.25E-01
Free cholesterol in very large V	Lipoprotein subclasses	0.01 (-0.03, 0.04)	8.82E-01
Triglycerides in very large VL	Lipoprotein subclasses	0.00 (-0.04, 0.03)	9.63E-01
Concentration of large VLDL p	Lipoprotein subclasses	0.00 (-0.03, 0.03)	9.95E-01
Total lipids in large VLDL	Lipoprotein subclasses	0.00 (-0.03, 0.03)	9.95E-01
Phospholipids in large VLDL	Lipoprotein subclasses	0.00 (-0.03, 0.03)	9.95E-01
Cholesterol in large VLDL	Lipoprotein subclasses	0.01 (-0.03, 0.04)	8.75E-01
Cholesteryl esters in large VLI	Lipoprotein subclasses	0.01 (-0.02, 0.05)	5.88E-01
Free cholesterol in large VLDI	Lipoprotein subclasses	0.00 (-0.03, 0.03)	9.95E-01
Triglycerides in large VLDL	Lipoprotein subclasses	0.00 (-0.03, 0.03)	9.68E-01
Concentration of medium VLD	Lipoprotein subclasses	0.01 (-0.03, 0.04)	8.79E-01
Total lipids in medium VLDL	Lipoprotein subclasses	0.01 (-0.02, 0.04)	8.44E-01
Phospholipids in medium VLD	Lipoprotein subclasses	0.01 (-0.02, 0.04)	8.14E-01
Cholesterol in medium VLDL	Lipoprotein subclasses	0.02 (-0.01, 0.05)	4.06E-01
Cholesteryl esters in medium V	Lipoprotein subclasses	0.03 (0.00, 0.06)	1.29E-01
Free cholesterol in medium VI	Lipoprotein subclasses	0.01 (-0.03, 0.04)	9.05E-01
Triglycerides in medium VLDI	Lipoprotein subclasses	0.00 (-0.03, 0.03)	9.94E-01
Concentration of small VLDL	Lipoprotein subclasses	0.01 (-0.02, 0.05)	5.95E-01
Total lipids in small VLDL	Lipoprotein subclasses	0.02 (-0.02, 0.05)	5.30E-01
Phospholipids in small VLDL	Lipoprotein subclasses	0.01 (-0.02, 0.04)	8.09E-01
Cholesterol in small VLDL	Lipoprotein subclasses	0.04 (-0.00, 0.07)	1.14E-01
Cholesteryl esters in small VLI	Lipoprotein subclasses	0.05 (-0.02, 0.08)	**4.79E-02**
Free cholesterol in small VLDI	Lipoprotein subclasses	0.01 (-0.02, 0.04)	7.23E-01
Triglycerides in small VLDL	Lipoprotein subclasses	0.00 (-0.03, 0.04)	9.28E-01
Concentration of very small VI	Lipoprotein subclasses	0.03 (-0.01, 0.06)	2.51E-01
Total lipids in very small VLD	Lipoprotein subclasses	0.03 (-0.01, 0.06)	2.07E-01
Phospholipids in very small VI	Lipoprotein subclasses	0.02 (-0.01, 0.06)	2.84E-01
Cholesterol in very small VLD	Lipoprotein subclasses	0.04 (-0.00, 0.07)	1.10E-01
Cholesteryl esters in very smal	Lipoprotein subclasses	0.04 (-0.01, 0.08)	6.71E-02
Free cholesterol in very small V	Lipoprotein subclasses	0.02 (-0.01, 0.05)	3.45E-01
Triglycerides in very small VL	Lipoprotein subclasses	0.00 (-0.03, 0.03)	9.95E-01

Concentration of IDL particles	Lipoprotein subclasses	0.03 (0.00, 0.07)	1.27E-01
Total lipids in IDL	Lipoprotein subclasses	0.03 (0.00, 0.07)	1.19E-01
Phospholipids in IDL	Lipoprotein subclasses	0.03 (0.00, 0.07)	1.19E-01
Cholesterol in IDL	Lipoprotein subclasses	0.04 (0.00, 0.07)	1.19E-01
Cholesteryl esters in IDL	Lipoprotein subclasses	0.04 (0.00, 0.07)	1.14E-01
Free cholesterol in IDL	Lipoprotein subclasses	0.03 (0.00, 0.06)	1.78E-01
Triglycerides in IDL	Lipoprotein subclasses	0.00 (-0.04, 0.03)	9.37E-01
Concentration of large LDL pa	Lipoprotein subclasses	0.03 (0.00, 0.07)	1.19E-01
Total lipids in large LDL	Lipoprotein subclasses	0.04 (0.00, 0.07)	1.19E-01
Phospholipids in large LDL	Lipoprotein subclasses	0.03 (0.00, 0.07)	1.19E-01
Cholesterol in large LDL	Lipoprotein subclasses	0.04 (0.00, 0.07)	1.11E-01
Cholesteryl esters in large LDI	Lipoprotein subclasses	0.04 (0.00, 0.07)	1.06E-01
Free cholesterol in large LDL	Lipoprotein subclasses	0.03 (0.00, 0.07)	1.27E-01
Triglycerides in large LDL	Lipoprotein subclasses	0.00 (-0.03, 0.03)	9.95E-01
Concentration of medium LDL	Lipoprotein subclasses	0.03 (0.00, 0.07)	1.19E-01
Total lipids in medium LDL	Lipoprotein subclasses	0.03 (0.00, 0.07)	1.19E-01
Phospholipids in medium LDL	Lipoprotein subclasses	0.03 (0.00, 0.07)	1.19E-01
Cholesterol in medium LDL	Lipoprotein subclasses	0.04 (0.00, 0.07)	1.19E-01
Cholesteryl esters in medium I	Lipoprotein subclasses	0.04 (0.00, 0.07)	1.19E-01
Free cholesterol in medium LI	Lipoprotein subclasses	0.03 (0.00, 0.06)	1.53E-01
Triglycerides in medium LDL	Lipoprotein subclasses	0.00 (-0.03, 0.04)	9.33E-01
Concentration of small LDL pa	Lipoprotein subclasses	0.03 (0.00, 0.06)	1.81E-01
Total lipids in small LDL	Lipoprotein subclasses	0.03 (0.00, 0.06)	1.81E-01
Phospholipids in small LDL	Lipoprotein subclasses	0.02 (-0.01, 0.05)	3.70E-01
Cholesterol in small LDL	Lipoprotein subclasses	0.03 (0.00, 0.06)	1.71E-01
Cholesteryl esters in small LDI	Lipoprotein subclasses	0.03 (0.00, 0.06)	1.81E-01
Free cholesterol in small LDL	Lipoprotein subclasses	0.02 (-0.01, 0.05)	3.54E-01
Triglycerides in small LDL	Lipoprotein subclasses	0.01 (-0.02, 0.05)	6.48E-01
Concentration of very large HI	Lipoprotein subclasses	-0.03 (-0.06, 0.00)	1.89E-01
Total lipids in very large HDL	Lipoprotein subclasses	-0.03 (-0.06, 0.00)	1.81E-01
Phospholipids in very large HI	Lipoprotein subclasses	-0.03 (-0.06, 0.00)	1.81E-01
Cholesterol in very large HDL	Lipoprotein subclasses	-0.03 (-0.06, 0.00)	1.81E-01
Cholesteryl esters in very large	Lipoprotein subclasses	-0.03 (-0.06, 0.01)	2.08E-01
Free cholesterol in very large H	Lipoprotein subclasses	-0.03 (-0.06, 0.00)	1.27E-01
Triglycerides in very large HD	Lipoprotein subclasses	0.01 (-0.02, 0.04)	7.64E-01
Concentration of large HDL pa	Lipoprotein subclasses	-0.04 (-0.07, -0.01)	9.45E-02
Total lipids in large HDL	Lipoprotein subclasses	-0.04 (-0.07, -0.01)	9.14E-02
Phospholipids in large HDL	Lipoprotein subclasses	-0.04 (-0.07, -0.01)	9.45E-02
Cholesterol in large HDL	Lipoprotein subclasses	-0.04 (-0.07, -0.01)	7.38E-02
Cholesteryl esters in large HDI	Lipoprotein subclasses	-0.04 (-0.08, -0.01)	6.91E-02
Free cholesterol in large HDL	Lipoprotein subclasses	-0.04 (-0.07, -0.01)	9.45E-02
Triglycerides in large HDL	Lipoprotein subclasses	0.00 (-0.03, 0.04)	9.63E-01
**Concentration of medium HI**	Lipoprotein subclasses	-0.05 (-0.08, -0.02)	**4.22E-02**
**Total lipids in medium HDL**	Lipoprotein subclasses	-0.05 (-0.09, -0.02)	**4.22E-02**
**Phospholipids in medium HI**	Lipoprotein subclasses	-0.05 (-0.08, -0.02)	**4.79E-02**
**Cholesterol in medium HDL**	Lipoprotein subclasses	-0.06 (-0.09, -0.02)	**3.25E-02**
**Cholesteryl esters in medium**	Lipoprotein subclasses	-0.06 (-0.09, -0.02)	**3.25E-02**
**Free cholesterol in medium H**	Lipoprotein subclasses	-0.05 (-0.09, -0.02)	**4.22E-02**

Triglycerides in medium HDL	Lipoprotein subclasses	0.01 (-0.02, 0.05)	6.41E-01
Concentration of small HDL pa	Lipoprotein subclasses	-0.03 (-0.07, 0.00)	1.27E-01
Total lipids in small HDL	Lipoprotein subclasses	-0.03 (-0.07, 0.00)	1.19E-01
Phospholipids in small HDL	Lipoprotein subclasses	-0.04 (-0.07, -0.01)	8.89E-02
Cholesterol in small HDL	Lipoprotein subclasses	-0.01 (-0.04, 0.02)	7.92E-01
Cholesteryl esters in small HD	Lipoprotein subclasses	0.00 (-0.03, 0.03)	9.95E-01
**Free cholesterol in small HDI**	Lipoprotein subclasses	-0.05 (-0.08, -0.02)	**4.22E-02**
Triglycerides in small HDL	Lipoprotein subclasses	0.01 (-0.03, 0.04)	8.79E-01
Phospholipids to total lipids ra	Relative lipoprotein lip	-0.01 (-0.04, 0.02)	7.92E-01
Cholesterol to total lipids ratio	Relative lipoprotein lip	0.04 (0.01, 0.08)	5.80E-02
Cholesteryl esters to total lipid	Relative lipoprotein lip	0.05 (0.01, 0.08)	5.42E-02
Free cholesterol to total lipids	Relative lipoprotein lip	0.01 (-0.02, 0.04)	7.48E-01
Triglycerides to total lipids rat	Relative lipoprotein lip	-0.04 (-0.07, 0.00)	1.19E-01
Phospholipids to total lipids ra	Relative lipoprotein lip	0.03 (0.00, 0.07)	1.27E-01
**Cholesterol to total lipids rat**	Relative lipoprotein lip	0.07 (0.04, 0.10)	**6.18E-03**
Cholesteryl esters to total lipid	Relative lipoprotein lip	0.05 (0.01, 0.08)	5.80E-02
**Free cholesterol to total lipid**	Relative lipoprotein lip	0.05 (0.01, 0.08)	5.47E-02
**Triglycerides to total lipids r**	Relative lipoprotein lip	-0.07 (-0.10, -0.04)	**6.18E-03**
Phospholipids to total lipids ra	Relative lipoprotein lip	0.01 (-0.02, 0.05)	5.88E-01
Cholesterol to total lipids ratio	Relative lipoprotein lip	0.04 (0.00, 0.07)	1.19E-01
Cholesteryl esters to total lipid	Relative lipoprotein lip	0.04 (0.01, 0.08)	8.89E-02
Free cholesterol to total lipids	Relative lipoprotein lip	0.01 (-0.03, 0.04)	8.74E-01
Triglycerides to total lipids rat	Relative lipoprotein lip	-0.03 (-0.07, 0.00)	1.32E-01
Phospholipids to total lipids ra	Relative lipoprotein lip	0.01 (-0.03, 0.04)	8.82E-01
**Cholesterol to total lipids rat**	Relative lipoprotein lip	0.05 (0.02, 0.09)	**4.22E-02**
**Cholesteryl esters to total lip**	Relative lipoprotein lip	0.05 (0.02, 0.09)	**4.22E-02**
Free cholesterol to total lipids	Relative lipoprotein lip	0.00 (-0.03, 0.03)	9.92E-01
Triglycerides to total lipids rat	Relative lipoprotein lip	-0.05 (-0.08, -0.02)	**4.79E-02**
**Phospholipids to total lipids r**	Relative lipoprotein lip	-0.06 (-0.09, -0.03)	**1.26E-02**
Cholesterol to total lipids ratio	Relative lipoprotein lip	0.04 (0.00, 0.07)	1.06E-01
Cholesteryl esters to total lipid	Relative lipoprotein lip	0.05 (0.01, 0.08)	5.56E-02
**Free cholesterol to total lipid**	Relative lipoprotein lip	-0.04 (-0.07, -0.01)	8.89E-02
Triglycerides to total lipids rat	Relative lipoprotein lip	-0.02 (-0.05, 0.01)	3.38E-01
Phospholipids to total lipids ra	Relative lipoprotein lip	0.00 (-0.03, 0.04)	9.22E-01
Cholesterol to total lipids ratio	Relative lipoprotein lip	0.04 (0.01, 0.07)	7.36E-02
Cholesteryl esters to total lipid	Relative lipoprotein lip	0.05 (0.01, 0.08)	**4.79E-02**
Free cholesterol to total lipids	Relative lipoprotein lip	0.01 (-0.02, 0.04)	8.44E-01
Triglycerides to total lipids rat	Relative lipoprotein lip	-0.03 (-0.07, 0.00)	1.19E-01
Phospholipids to total lipids ra	Relative lipoprotein lip	0.01 (-0.02, 0.04)	8.05E-01
Cholesterol to total lipids ratio	Relative lipoprotein lip	0.04 (0.01, 0.08)	6.41E-02
Cholesteryl esters to total lipid	Relative lipoprotein lip	0.03 (0.00, 0.07)	1.19E-01
Free cholesterol to total lipids	Relative lipoprotein lip	0.02 (-0.01, 0.05)	3.26E-01
Triglycerides to total lipids rat	Relative lipoprotein lip	-0.04 (-0.08, -0.01)	5.80E-02
Phospholipids to total lipids ra	Relative lipoprotein lip	-0.04 (-0.07, 0.00)	1.10E-01
Cholesterol to total lipids ratio	Relative lipoprotein lip	0.05 (0.01, 0.08)	5.42E-02
Cholesteryl esters to total lipid	Relative lipoprotein lip	0.05 (0.02, 0.08)	**4.79E-02**
Free cholesterol to total lipids	Relative lipoprotein lip	0.00 (-0.03, 0.04)	9.37E-01
Triglycerides to total lipids rat	Relative lipoprotein lip	-0.04 (-0.08, -0.01)	5.80E-02

Phospholipids to total lipids ra	Relative lipoprotein li	-0.03 (-0.06, 0.01)	2.37E-01
Cholesterol to total lipids ratio	Relative lipoprotein li	0.03 (0.00, 0.06)	1.30E-01
Cholesteryl esters to total lipid	Relative lipoprotein li	0.03 (0.00, 0.07)	1.19E-01
Free cholesterol to total lipids	Relative lipoprotein li	-0.03 (-0.07, 0.00)	1.19E-01
Triglycerides to total lipids rat	Relative lipoprotein li	-0.04 (-0.07, -0.01)	9.45E-02
Phospholipids to total lipids ra	Relative lipoprotein li	-0.03 (-0.06, 0.00)	1.32E-01
Cholesterol to total lipids ratio	Relative lipoprotein li	0.03 (0.00, 0.06)	1.77E-01
Cholesteryl esters to total lipid	Relative lipoprotein li	0.03 (0.00, 0.07)	1.27E-01
Free cholesterol to total lipids	Relative lipoprotein li	-0.04 (-0.07, -0.01)	9.02E-02
Triglycerides to total lipids rat	Relative lipoprotein li	-0.02 (-0.05, 0.01)	3.93E-01
Phospholipids to total lipids ra	Relative lipoprotein li	0.00 (-0.03, 0.03)	9.95E-01
Cholesterol to total lipids ratio	Relative lipoprotein li	-0.01 (-0.04, 0.03)	8.32E-01
Cholesteryl esters to total lipid	Relative lipoprotein li	0.00 (-0.03, 0.03)	9.95E-01
Free cholesterol to total lipids	Relative lipoprotein li	-0.04 (-0.07, 0.00)	1.13E-01
Triglycerides to total lipids rat	Relative lipoprotein li	0.02 (-0.01, 0.05)	3.52E-01
Phospholipids to total lipids ra	Relative lipoprotein li	0.03 (-0.01, 0.06)	2.50E-01
Cholesterol to total lipids ratio	Relative lipoprotein li	-0.04 (-0.08, -0.01)	6.45E-02
Cholesteryl esters to total lipid	Relative lipoprotein li	-0.05 (-0.08, -0.02)	**4.22E-02**
Free cholesterol to total lipids	Relative lipoprotein li	-0.02 (-0.06, 0.01)	2.99E-01
Triglycerides to total lipids rat	Relative lipoprotein li	0.04 (0.01, 0.07)	9.02E-02
Phospholipids to total lipids ra	Relative lipoprotein li	0.02 (-0.01, 0.06)	2.56E-01
Cholesterol to total lipids ratio	Relative lipoprotein li	-0.04 (-0.07, -0.01)	7.25E-02
Cholesteryl esters to total lipid	Relative lipoprotein li	-0.03 (-0.07, 0.00)	1.19E-01
Free cholesterol to total lipids	Relative lipoprotein li	-0.05 (-0.08, -0.01)	5.47E-02
Triglycerides to total lipids rat	Relative lipoprotein li	0.04 (0.01, 0.08)	6.71E-02
Phospholipids to total lipids ra	Relative lipoprotein li	-0.02 (-0.06, 0.01)	2.59E-01
Cholesterol to total lipids ratio	Relative lipoprotein li	0.02 (-0.02, 0.05)	5.54E-01
Cholesteryl esters to total lipid	Relative lipoprotein li	0.02 (-0.01, 0.05)	4.16E-01
Free cholesterol to total lipids	Relative lipoprotein li	-0.05 (-0.08, -0.01)	5.76E-02
Triglycerides to total lipids rat	Relative lipoprotein li	0.02 (-0.01, 0.05)	4.06E-01
Average diameter for VLDL p	Lipoprotein particle si	0.00 (-0.03, 0.03)	9.68E-01
Average diameter for LDL par	Lipoprotein particle si	0.03 (0.00, 0.06)	1.78E-01
Average diameter for HDL par	Lipoprotein particle si	-0.02 (-0.06, 0.01)	2.84E-01
Total cholesterol	Cholesterol	0.02 (-0.02, 0.05)	4.69E-01
VLDL cholesterol	Cholesterol	0.03 (0.00, 0.06)	1.77E-01
Remnant cholesterol (non-HDL	Cholesterol	0.04 (0.00, 0.07)	1.05E-01
LDL cholesterol	Cholesterol	0.03 (0.00, 0.07)	1.27E-01
**HDL cholesterol**	Cholesterol	-0.05 (-0.08, -0.02)	**4.27E-02**
**HDL2 cholesterol**	Cholesterol	-0.05 (-0.08, -0.02)	**4.22E-02**
HDL3 cholesterol	Cholesterol	-0.03 (-0.06, 0.00)	1.32E-01
Total esterified cholesterol	Cholesteryl esters	0.01 (-0.02, 0.05)	7.13E-01
Total free cholesterol	Free cholesterol	0.02 (-0.01, 0.05)	4.06E-01
Total triglycerides	Triglycerides	0.00 (-0.03, 0.03)	9.95E-01
Triglycerides in VLDL	Triglycerides	0.00 (-0.03, 0.03)	9.95E-01
Triglycerides in LDL	Triglycerides	-0.01 (-0.04, 0.03)	8.79E-01
Triglycerides in HDL	Triglycerides	0.01 (-0.02, 0.04)	7.92E-01
Phosphoglycerides	Other lipids	-0.01 (-0.04, 0.03)	9.06E-01
Ratio of triglycerides to phospl	Other lipids	0.01 (-0.02, 0.04)	7.14E-01

Phosphatidylcholines	Other lipids	-0.01 (-0.04, 0.02)	7.22E-01
Sphingomyelins	Other lipids	0.00 (-0.03, 0.03)	9.95E-01
Total cholines	Other lipids	-0.01 (-0.04, 0.02)	8.02E-01
Apolipoprotein A1	Apolipoproteins	-0.04 (-0.07, 0.00)	1.06E-01
Apolipoprotein B	Apolipoproteins	0.03 (0.00, 0.06)	1.81E-01
Ratio of apolipoprotein B to ap	Apolipoproteins	0.05 (0.01, 0.08)	5.56E-02
Total fatty acids	Fatty acids	0.00 (-0.03, 0.03)	9.95E-01
Degree of unsaturation	Fatty acids	0.02 (-0.01, 0.05)	4.06E-01
Docosahexaenoic acid	Fatty acids	0.02 (-0.01, 0.06)	2.58E-01
Linoleic acid	Fatty acids	0.00 (-0.03, 0.04)	9.35E-01
Omega-3 fatty acids	Fatty acids	0.02 (-0.01, 0.05)	3.37E-01
Omega-6 fatty acids	Fatty acids	0.00 (-0.03, 0.04)	9.37E-01
Polyunsaturated fatty acids	Fatty acids	0.01 (-0.03, 0.04)	8.44E-01
Monounsaturated fatty acids	Fatty acids	-0.01 (-0.04, 0.03)	8.79E-01
Saturated fatty acids	Fatty acids	0.00 (-0.03, 0.04)	9.60E-01
Ratio of docosahexaenoic acid	Fatty acids	0.03 (0.00, 0.06)	1.67E-01
Ratio of linoleic acid to total fa	Fatty acids	0.01 (-0.03, 0.04)	8.44E-01
Ratio of omega-3 fatty acids to	Fatty acids	0.04 (0.01, 0.07)	8.89E-02
Ratio of omega-6 fatty acids to	Fatty acids	0.00 (-0.03, 0.04)	9.37E-01
Ratio of polyunsaturated fatty a	Fatty acids	0.02 (-0.02, 0.05)	5.27E-01
Ratio of monounsaturated fatty	Fatty acids	-0.02 (-0.05, 0.01)	3.63E-01
Ratio of saturated fatty acids to	Fatty acids	0.00 (-0.03, 0.04)	9.68E-01
Glucose	Glycolysis related met	0.02 (-0.02, 0.05)	5.10E-01
Lactate	Glycolysis related met	-0.01 (-0.04, 0.03)	8.89E-01
Citrate	Glycolysis related met	-0.01 (-0.04, 0.03)	7.96E-01
Alanine	Amino acids	0.01 (-0.02, 0.04)	7.96E-01
Glutamine	Amino acids	0.00 (-0.04, 0.03)	9.28E-01
Histidine	Amino acids	0.03 (0.00, 0.07)	1.19E-01
Isoleucine	Amino acids	0.02 (-0.01, 0.05)	4.59E-01
Leucine	Amino acids	0.02 (-0.02, 0.05)	5.10E-01
Valine	Amino acids	0.01 (-0.02, 0.05)	5.82E-01
Phenylalanine	Amino acids	0.05 (0.02, 0.08)	**4.22E-02**
Tyrosine	Amino acids	0.03 (0.00, 0.07)	1.19E-01
Acetate	Ketone bodies	0.00 (-0.03, 0.04)	9.94E-01
Acetoacetate	Ketone bodies	-0.03 (-0.06, 0.00)	1.81E-01
**3-Hydroxybutyrate**	Ketone bodies	-0.04 (-0.08, -0.01)	6.82E-02
Creatinine	Fluid balance	-0.01 (-0.04, 0.03)	8.79E-01
Albumin	Fluid balance	-0.02 (-0.06, 0.01)	2.47E-01
Glycoprotein acetyls	Inflammation	0.02 (-0.02, 0.05)	5.04E-01
Acetone	Ketone bodies		
Cholesteryl esters in HDL	Cholesteryl esters		
Cholesteryl esters in LDL	Cholesteryl esters		
Cholesteryl esters in VLDL/ (C	Cholesteryl esters		
Clinical LDL cholesterol	Cholesterol		
Concentration of HDL particle	Lipoprotein particle concentrations		
Concentration of LDL particles	Lipoprotein particle concentrations		
Concentration of VLDL partic	Lipoprotein particle concentrations		
Free cholesterol in HDL	Free cholesterol		

Free cholesterol in LDL	Free cholesterol
Free cholesterol in VLDL	Free cholesterol
Glycine	Amino acids
Phospholipids in HDL	Phospholipids
Phospholipids in LDL	Phospholipids
Phospholipids in VLDL	Phospholipids
Pyruvate	Glycolysis related metabolites
Ratio of omega-6 fatty acids to	Fatty acids
Ratio of polyunsaturated fatty a	Fatty acids
Total cholesterol minus HDL-C	Cholesterol
Total concentration of branche	Amino acids
Total concentration of lipoprot	Lipoprotein particle concentrations
Total lipids in HDL	Total lipids
Total lipids in LDL	Total lipids
Total lipids in lipoprotein parti	Total lipids
Total lipids in VLDL	Total lipids
Total phospholipids in lipoprot	Phospholipids

Models were adjusted for age, sex, regions (CKB only), ethnicity (UKB only), levels of education, smoking

HR, hazard ratio; CI, confidence interval; LDL, Low Density Lipoprotein; HDL, High Density Lipoprotein

and all-cause and cause-specific mortality in the CKB and UKB after excluding

All causes mortality		Ischaemic heart diseases mortality	
**Adjusted HR (95% CI)**	**P value**	**Adjusted HR (95% CI)**	**P value**
0.96 (0.92, 1.02)	3.05E-01	1.00 (0.91, 1.10)	9.89E-01
0.97 (0.92, 1.02)	3.37E-01	1.00 (0.91, 1.10)	9.53E-01
0.98 (0.93, 1.03)	5.10E-01	1.02 (0.93, 1.12)	7.96E-01
1.00 (0.95, 1.05)	9.56E-01	1.04 (0.95, 1.14)	5.11E-01
1.01 (0.96, 1.06)	7.39E-01	1.06 (0.97, 1.16)	3.26E-01
0.99 (0.94, 1.04)	7.39E-01	1.01 (0.92, 1.11)	8.31E-01
0.96 (0.91, 1.01)	1.95E-01	0.99 (0.90, 1.09)	8.74E-01
0.99 (0.94, 1.04)	7.97E-01	1.01 (0.92, 1.10)	9.17E-01
0.99 (0.94, 1.04)	8.15E-01	1.01 (0.92, 1.11)	8.74E-01
0.99 (0.95, 1.05)	8.60E-01	1.02 (0.93, 1.12)	7.96E-01
1.01 (0.96, 1.06)	8.71E-01	1.03 (0.94, 1.13)	5.76E-01
1.01 (0.96, 1.06)	8.42E-01	1.04 (0.95, 1.14)	5.42E-01
1.00 (0.95, 1.05)	9.19E-01	1.03 (0.94, 1.13)	6.84E-01
0.99 (0.94, 1.04)	7.28E-01	1.00 (0.91, 1.10)	9.61E-01
0.98 (0.93, 1.03)	6.51E-01	1.02 (0.93, 1.12)	7.53E-01
0.98 (0.94, 1.03)	6.54E-01	1.02 (0.93, 1.12)	7.53E-01
0.98 (0.94, 1.03)	6.58E-01	1.02 (0.93, 1.12)	7.55E-01
0.98 (0.94, 1.03)	6.62E-01	1.02 (0.93, 1.12)	7.68E-01
0.98 (0.93, 1.03)	5.91E-01	1.01 (0.92, 1.11)	8.20E-01
0.99 (0.94, 1.04)	7.64E-01	1.02 (0.93, 1.12)	7.24E-01
0.98 (0.93, 1.03)	6.46E-01	1.02 (0.93, 1.12)	7.52E-01
0.98 (0.94, 1.03)	6.46E-01	1.04 (0.95, 1.14)	5.38E-01
0.98 (0.94, 1.03)	6.57E-01	1.04 (0.95, 1.14)	5.35E-01
0.98 (0.93, 1.03)	6.21E-01	1.04 (0.95, 1.14)	5.56E-01
0.99 (0.94, 1.04)	8.09E-01	1.05 (0.95, 1.15)	4.96E-01
0.99 (0.94, 1.04)	7.64E-01	1.04 (0.95, 1.15)	5.13E-01
0.99 (0.95, 1.04)	8.36E-01	1.05 (0.95, 1.15)	4.93E-01
0.98 (0.93, 1.03)	6.21E-01	1.04 (0.95, 1.14)	5.45E-01
0.99 (0.94, 1.04)	8.15E-01	1.07 (0.98, 1.18)	2.69E-01
1.00 (0.95, 1.05)	9.19E-01	1.09 (0.99, 1.19)	1.69E-01
0.98 (0.94, 1.03)	6.62E-01	1.07 (0.97, 1.17)	3.26E-01
1.04 (0.99, 1.09)	2.01E-01	1.20 (1.10, 1.31)	**3.00E-04**
1.05 (1.00, 1.10)	1.02E-01	1.22 (1.12, 1.34)	**5.11E-05**
1.02 (0.97, 1.07)	6.21E-01	1.13 (1.03, 1.24)	**2.26E-02**
0.98 (0.93, 1.02)	4.80E-01	1.02 (0.92, 1.12)	8.20E-01
1.04 (0.99, 1.09)	2.11E-01	1.16 (1.06, 1.27)	**3.15E-03**
1.05 (1.00, 1.10)	1.44E-01	1.18 (1.08, 1.28)	**1.14E-03**
1.01 (0.96, 1.06)	8.63E-01	1.08 (0.98, 1.18)	2.22E-01
1.08 (1.03, 1.13)	**5.05E-03**	1.26 (1.16, 1.37)	**6.46E-07**
1.08 (1.03, 1.13)	**2.82E-03**	1.27 (1.17, 1.38)	**2.78E-07**
1.06 (1.02, 1.11)	**2.59E-02**	1.22 (1.12, 1.33)	**2.54E-05**
1.00 (0.96, 1.05)	9.19E-01	1.04 (0.94, 1.14)	5.56E-01

1.01 (0.96, 1.05)	8.71E-01	1.06 (0.97, 1.16)	3.52E-01
1.00 (0.96, 1.05)	8.71E-01	1.06 (0.97, 1.17)	3.32E-01
0.99 (0.94, 1.04)	7.59E-01	1.04 (0.94, 1.14)	5.56E-01
1.00 (0.96, 1.05)	9.19E-01	1.07 (0.97, 1.17)	2.95E-01
1.00 (0.95, 1.05)	9.19E-01	1.06 (0.97, 1.17)	3.26E-01
1.02 (0.97, 1.07)	6.58E-01	1.08 (0.98, 1.18)	2.46E-01
1.04 (0.99, 1.09)	2.15E-01	1.04 (0.95, 1.14)	5.25E-01
0.99 (0.94, 1.04)	7.12E-01	1.05 (0.95, 1.15)	4.96E-01
0.98 (0.94, 1.03)	6.74E-01	1.04 (0.95, 1.15)	5.08E-01
0.97 (0.92, 1.02)	3.37E-01	1.02 (0.93, 1.12)	7.85E-01
0.98 (0.93, 1.03)	6.21E-01	1.05 (0.95, 1.15)	5.01E-01
0.98 (0.93, 1.03)	5.43E-01	1.05 (0.95, 1.15)	4.96E-01
0.99 (0.95, 1.04)	8.22E-01	1.04 (0.95, 1.15)	5.32E-01
1.07 (1.02, 1.12)	**2.26E-02**	1.08 (0.98, 1.18)	2.21E-01
0.96 (0.91, 1.01)	2.04E-01	1.02 (0.93, 1.13)	7.52E-01
0.96 (0.91, 1.01)	1.86E-01	1.02 (0.93, 1.13)	7.58E-01
0.92 (0.88, 0.97)	**5.68E-03**	0.94 (0.86, 1.04)	4.02E-01
0.96 (0.92, 1.01)	2.50E-01	1.04 (0.94, 1.14)	5.55E-01
0.97 (0.92, 1.01)	3.05E-01	1.05 (0.95, 1.15)	4.84E-01
0.95 (0.90, 1.00)	7.73E-02	0.99 (0.90, 1.09)	8.70E-01
1.04 (0.99, 1.10)	1.82E-01	1.04 (0.95, 1.15)	5.11E-01
0.96 (0.91, 1.01)	1.91E-01	1.04 (0.94, 1.14)	5.55E-01
0.96 (0.91, 1.01)	1.82E-01	1.04 (0.94, 1.14)	5.55E-01
0.92 (0.88, 0.97)	**4.44E-03**	0.95 (0.86, 1.05)	4.40E-01
0.97 (0.92, 1.02)	3.10E-01	1.06 (0.96, 1.17)	3.70E-01
0.97 (0.92, 1.02)	3.54E-01	1.07 (0.97, 1.18)	3.06E-01
0.95 (0.90, 1.00)	9.59E-02	1.01 (0.92, 1.12)	8.20E-01
1.02 (0.97, 1.07)	6.46E-01	1.07 (0.97, 1.17)	3.28E-01
1.18 (1.13, 1.23)	**1.09E-11**	1.25 (1.15, 1.36)	**1.88E-06**
1.18 (1.13, 1.23)	**1.27E-11**	1.25 (1.15, 1.36)	**1.62E-06**
1.17 (1.11, 1.22)	**3.34E-10**	1.17 (1.07, 1.28)	**1.59E-03**
1.18 (1.13, 1.23)	**1.95E-11**	1.32 (1.21, 1.44)	**2.27E-09**
1.18 (1.13, 1.23)	**1.44E-11**	1.36 (1.25, 1.48)	**3.46E-11**
1.17 (1.12, 1.22)	**2.06E-10**	1.23 (1.13, 1.34)	**1.55E-05**
1.15 (1.10, 1.20)	**1.50E-08**	1.22 (1.12, 1.33)	**2.24E-05**
1.14 (1.09, 1.19)	**2.04E-07**	1.11 (1.01, 1.21)	6.24E-02
1.14 (1.09, 1.19)	**2.04E-07**	1.11 (1.01, 1.21)	5.86E-02
1.13 (1.08, 1.19)	**1.11E-06**	1.11 (1.01, 1.21)	6.24E-02
1.14 (1.09, 1.19)	**1.85E-07**	1.11 (1.02, 1.21)	5.70E-02
1.14 (1.09, 1.19)	**2.61E-07**	1.10 (1.01, 1.21)	8.48E-02
1.15 (1.09, 1.20)	**5.92E-08**	1.14 (1.04, 1.24)	**1.52E-02**
1.14 (1.09, 1.20)	**3.47E-07**	1.07 (0.97, 1.17)	3.26E-01
0.94 (0.90, 0.99)	**3.98E-02**	0.84 (0.77, 0.93)	**2.51E-03**
0.94 (0.90, 0.99)	**4.99E-02**	0.85 (0.77, 0.93)	**3.22E-03**
0.97 (0.92, 1.02)	3.37E-01	0.88 (0.80, 0.97)	**3.03E-02**
0.94 (0.89, 0.98)	**2.37E-02**	0.85 (0.77, 0.94)	**3.15E-03**
0.93 (0.88, 0.97)	**8.63E-03**	0.84 (0.77, 0.93)	**1.59E-03**
0.97 (0.92, 1.02)	3.88E-01	0.89 (0.81, 0.98)	**4.05E-02**

0.92 (0.87, 0.97)	**4.31E-03**	0.82 (0.74, 0.91)	**9.09E-04**
0.86 (0.82, 0.90)	**3.38E-09**	0.78 (0.72, 0.84)	**3.56E-08**
0.86 (0.82, 0.90)	**4.04E-09**	0.78 (0.72, 0.85)	**7.45E-08**
0.90 (0.85, 0.94)	**7.39E-05**	0.81 (0.74, 0.89)	**9.67E-05**
0.90 (0.86, 0.95)	**1.35E-04**	0.86 (0.78, 0.94)	**3.15E-03**
0.92 (0.87, 0.96)	**1.52E-03**	0.88 (0.81, 0.97)	**2.37E-02**
0.92 (0.88, 0.96)	**2.73E-03**	0.84 (0.77, 0.93)	**1.47E-03**
0.94 (0.89, 0.98)	**2.55E-02**	0.87 (0.79, 0.96)	**1.94E-02**
0.97 (0.93, 1.02)	4.22E-01	1.06 (0.96, 1.18)	3.77E-01
1.17 (1.12, 1.21)	**1.46E-13**	1.19 (1.12, 1.27)	**7.73E-07**
1.16 (1.11, 1.20)	**1.08E-11**	1.19 (1.11, 1.27)	**2.94E-06**
1.10 (1.05, 1.15)	**3.41E-04**	1.11 (1.02, 1.21)	**4.64E-02**
0.87 (0.83, 0.91)	**3.47E-09**	0.82 (0.76, 0.88)	**1.62E-06**
0.99 (0.94, 1.04)	7.64E-01	1.06 (0.96, 1.17)	4.12E-01
1.07 (1.03, 1.11)	**1.28E-03**	1.11 (1.04, 1.18)	**3.22E-03**
1.07 (1.02, 1.11)	**5.68E-03**	1.10 (1.02, 1.19)	**3.17E-02**
1.04 (0.99, 1.09)	2.11E-01	1.08 (0.99, 1.18)	1.51E-01
0.94 (0.89, 0.99)	**3.38E-02**	0.87 (0.79, 0.95)	**6.33E-03**
0.97 (0.92, 1.02)	3.88E-01	0.95 (0.86, 1.04)	3.77E-01
0.99 (0.94, 1.04)	7.15E-01	0.94 (0.86, 1.03)	3.45E-01
0.99 (0.94, 1.04)	8.15E-01	0.91 (0.83, 1.00)	1.29E-01
0.99 (0.94, 1.04)	8.16E-01	1.01 (0.92, 1.12)	8.20E-01
1.02 (0.97, 1.07)	6.21E-01	1.07 (0.97, 1.17)	3.21E-01
0.95 (0.91, 1.00)	1.04E-01	0.83 (0.75, 0.91)	**8.42E-04**
1.04 (0.99, 1.09)	2.62E-01	0.95 (0.86, 1.05)	4.31E-01
1.02 (0.97, 1.07)	6.46E-01	0.92 (0.83, 1.02)	2.17E-01
1.05 (1.00, 1.11)	9.24E-02	1.08 (0.98, 1.20)	2.22E-01
0.97 (0.93, 1.02)	4.79E-01	1.08 (0.98, 1.20)	2.22E-01
0.95 (0.91, 1.00)	9.04E-02	0.83 (0.75, 0.92)	**1.52E-03**
1.09 (1.04, 1.14)	**2.08E-03**	1.22 (1.11, 1.34)	**1.58E-04**
1.07 (1.02, 1.13)	**1.14E-02**	1.20 (1.09, 1.32)	**8.44E-04**
1.16 (1.10, 1.21)	**1.34E-08**	1.29 (1.18, 1.40)	**2.45E-07**
0.93 (0.89, 0.98)	**9.46E-03**	0.85 (0.78, 0.94)	**3.22E-03**
0.92 (0.88, 0.96)	**1.51E-03**	0.84 (0.78, 0.91)	**1.62E-04**
1.11 (1.06, 1.17)	**7.85E-05**	1.41 (1.27, 1.57)	**2.27E-09**
1.11 (1.05, 1.16)	**2.17E-04**	1.35 (1.22, 1.49)	**3.65E-08**
1.08 (1.02, 1.13)	**1.24E-02**	1.28 (1.15, 1.43)	**6.99E-05**
0.96 (0.91, 1.01)	1.82E-01	0.85 (0.77, 0.95)	**8.13E-03**
0.92 (0.88, 0.97)	**2.63E-03**	0.86 (0.78, 0.94)	**2.51E-03**
0.99 (0.95, 1.04)	7.97E-01	1.07 (0.98, 1.18)	2.69E-01
0.96 (0.92, 1.00)	1.57E-01	1.03 (0.94, 1.13)	6.55E-01
1.03 (0.98, 1.09)	3.11E-01	1.08 (0.98, 1.19)	2.72E-01
1.04 (0.99, 1.08)	2.50E-01	0.99 (0.90, 1.08)	8.20E-01
0.98 (0.94, 1.04)	6.83E-01	0.95 (0.86, 1.05)	4.84E-01
0.96 (0.92, 1.01)	1.76E-01	0.99 (0.90, 1.08)	8.20E-01
0.94 (0.90, 0.99)	**3.42E-02**	0.97 (0.89, 1.07)	6.93E-01
1.05 (0.99, 1.10)	1.82E-01	1.03 (0.94, 1.14)	6.10E-01
1.08 (1.03, 1.12)	**4.12E-03**	1.06 (0.97, 1.15)	3.41E-01

0.99 (0.94, 1.04)	7.03E-01	0.90 (0.81, 1.00)	1.06E-01
0.98 (0.93, 1.03)	5.46E-01	1.06 (0.96, 1.16)	4.19E-01
0.97 (0.93, 1.02)	4.49E-01	1.05 (0.95, 1.16)	4.96E-01
1.03 (0.98, 1.09)	3.19E-01	0.98 (0.89, 1.08)	7.88E-01
1.09 (1.04, 1.14)	**1.08E-03**	1.04 (0.95, 1.14)	5.08E-01
0.99 (0.95, 1.04)	8.42E-01	0.89 (0.80, 0.98)	5.70E-02
0.98 (0.94, 1.03)	6.62E-01	1.07 (0.97, 1.19)	2.96E-01
0.98 (0.93, 1.03)	6.10E-01	1.07 (0.97, 1.18)	3.32E-01
1.03 (0.98, 1.09)	3.37E-01	0.97 (0.88, 1.07)	6.93E-01
1.06 (1.01, 1.11)	**4.98E-02**	1.03 (0.94, 1.13)	6.55E-01
1.00 (0.95, 1.05)	9.19E-01	0.83 (0.76, 0.90)	**1.62E-04**
1.02 (0.97, 1.07)	6.54E-01	1.31 (1.19, 1.45)	**5.16E-07**
1.01 (0.96, 1.06)	7.39E-01	1.32 (1.20, 1.44)	**1.37E-07**
1.03 (0.98, 1.08)	3.82E-01	1.10 (1.00, 1.21)	1.06E-01
0.97 (0.92, 1.01)	3.10E-01	0.93 (0.85, 1.03)	2.95E-01
0.88 (0.84, 0.93)	**5.35E-06**	0.90 (0.82, 1.00)	9.51E-02
1.11 (1.05, 1.16)	**3.41E-04**	1.13 (1.02, 1.25)	**4.16E-02**
1.07 (1.02, 1.12)	**2.08E-02**	1.02 (0.92, 1.12)	8.12E-01
1.14 (1.08, 1.20)	**4.72E-06**	1.29 (1.16, 1.44)	**2.10E-05**
1.01 (0.96, 1.06)	8.14E-01	0.94 (0.86, 1.04)	4.12E-01
1.15 (1.11, 1.19)	**1.95E-11**	1.19 (1.12, 1.26)	**5.16E-07**
0.93 (0.89, 0.98)	**9.36E-03**	0.92 (0.85, 1.00)	1.32E-01
0.92 (0.88, 0.96)	**1.43E-03**	0.92 (0.84, 1.00)	9.95E-02
1.01 (0.96, 1.06)	8.15E-01	0.97 (0.89, 1.06)	6.19E-01
0.97 (0.92, 1.02)	3.84E-01	0.93 (0.84, 1.02)	2.54E-01
1.01 (0.96, 1.06)	8.15E-01	0.99 (0.90, 1.09)	8.70E-01
1.00 (0.95, 1.04)	8.71E-01	1.02 (0.93, 1.12)	7.68E-01
0.99 (0.94, 1.04)	7.32E-01	1.01 (0.92, 1.11)	8.74E-01
1.08 (1.03, 1.13)	**8.79E-03**	1.09 (0.99, 1.19)	1.66E-01
0.99 (0.95, 1.04)	8.42E-01	0.96 (0.87, 1.06)	5.53E-01
0.95 (0.91, 1.00)	1.28E-01	0.98 (0.90, 1.08)	7.96E-01
1.10 (1.05, 1.15)	**8.67E-04**	0.99 (0.90, 1.09)	8.20E-01
1.17 (1.12, 1.23)	**3.23E-10**	1.23 (1.12, 1.34)	**4.24E-05**
1.03 (0.98, 1.08)	4.25E-01	1.12 (1.03, 1.23)	**3.23E-02**
1.02 (0.98, 1.07)	5.10E-01	1.15 (1.05, 1.26)	**6.73E-03**
1.02 (0.97, 1.07)	6.36E-01	1.14 (1.04, 1.25)	**1.52E-02**
0.98 (0.93, 1.03)	5.57E-01	1.06 (0.97, 1.17)	3.52E-01
1.09 (1.04, 1.14)	**1.59E-03**	1.08 (0.98, 1.18)	2.21E-01
1.08 (1.03, 1.13)	**6.66E-03**	1.05 (0.96, 1.15)	4.80E-01
1.16 (1.11, 1.22)	**1.38E-09**	1.33 (1.21, 1.45)	**5.58E-09**
0.93 (0.88, 0.98)	**1.24E-02**	0.89 (0.80, 0.98)	**4.67E-02**
0.93 (0.89, 0.98)	**2.65E-02**	0.93 (0.85, 1.03)	3.32E-01
0.99 (0.94, 1.04)	8.15E-01	1.04 (0.95, 1.14)	5.56E-01
0.98 (0.93, 1.03)	6.36E-01	1.04 (0.95, 1.14)	5.56E-01
1.06 (1.01, 1.11)	**3.47E-02**	1.08 (0.99, 1.18)	1.93E-01
1.02 (0.97, 1.07)	5.84E-01	0.95 (0.86, 1.05)	4.96E-01
1.01 (0.97, 1.07)	7.11E-01	1.02 (0.93, 1.12)	7.96E-01
0.99 (0.94, 1.04)	7.39E-01	1.04 (0.95, 1.14)	5.01E-01

0.91 (0.87, 0.96)	**1.52E-03**	0.83 (0.76, 0.92)	**1.27E-03**
0.87 (0.82, 0.91)	**5.09E-07**	0.81 (0.73, 0.89)	**1.65E-04**
0.98 (0.93, 1.03)	5.97E-01	0.94 (0.86, 1.04)	3.90E-01
1.09 (1.04, 1.15)	**7.16E-04**	1.14 (1.04, 1.24)	**1.73E-02**
0.99 (0.94, 1.04)	7.39E-01	1.08 (0.99, 1.19)	1.93E-01
0.95 (0.90, 0.99)	7.38E-02	1.01 (0.92, 1.11)	8.43E-01
0.96 (0.91, 1.00)	1.65E-01	0.96 (0.87, 1.06)	5.56E-01
0.79 (0.76, 0.83)	**6.70E-21**	0.69 (0.63, 0.74)	**5.09E-20**
1.08 (1.03, 1.13)	**4.19E-03**	1.40 (1.29, 1.51)	**5.60E-15**
0.92 (0.88, 0.97)	**9.36E-03**	0.91 (0.83, 1.01)	1.83E-01
1.00 (0.95, 1.05)	9.56E-01	1.19 (1.09, 1.30)	**4.98E-04**
0.91 (0.86, 0.96)	**1.80E-03**	0.87 (0.78, 0.96)	**2.26E-02**
0.92 (0.87, 0.97)	**7.98E-03**	0.92 (0.83, 1.02)	2.33E-01
0.97 (0.92, 1.02)	3.82E-01	0.99 (0.90, 1.09)	8.66E-01
0.98 (0.93, 1.03)	4.99E-01	0.98 (0.89, 1.08)	7.41E-01
1.15 (1.09, 1.20)	**1.78E-07**	1.65 (1.52, 1.81)	**5.34E-27**
0.90 (0.85, 0.94)	**4.26E-05**	0.83 (0.77, 0.90)	**7.37E-05**
1.05 (1.00, 1.10)	1.43E-01	1.38 (1.27, 1.51)	**3.26E-12**
0.89 (0.85, 0.94)	**2.62E-05**	0.77 (0.70, 0.85)	**3.44E-07**
0.90 (0.85, 0.94)	**1.35E-04**	0.85 (0.77, 0.93)	**2.37E-03**
1.03 (0.98, 1.08)	3.37E-01	1.10 (1.01, 1.21)	8.05E-02
1.14 (1.08, 1.19)	**2.37E-06**	1.14 (1.04, 1.25)	**1.81E-02**
1.13 (1.09, 1.17)	**4.68E-09**	1.17 (1.10, 1.25)	**1.36E-05**
0.95 (0.90, 1.00)	1.12E-01	1.12 (1.02, 1.22)	**3.64E-02**
0.99 (0.94, 1.04)	7.39E-01	0.83 (0.75, 0.92)	**1.37E-03**
0.93 (0.88, 0.97)	**8.79E-03**	1.05 (0.96, 1.15)	4.16E-01
0.90 (0.85, 0.94)	**9.37E-05**	0.94 (0.86, 1.03)	3.41E-01
0.98 (0.93, 1.03)	5.58E-01	1.20 (1.10, 1.31)	**2.66E-04**
0.94 (0.90, 0.99)	5.47E-02	1.01 (0.92, 1.11)	8.20E-01
0.96 (0.91, 1.01)	2.21E-01	1.13 (1.03, 1.24)	**2.38E-02**
0.83 (0.79, 0.88)	**2.46E-10**	0.84 (0.76, 0.93)	**3.15E-03**
1.11 (1.07, 1.15)	**1.85E-07**	1.20 (1.15, 1.25)	**6.43E-17**
1.02 (0.97, 1.07)	5.84E-01	1.15 (1.05, 1.26)	**6.33E-03**
1.06 (1.02, 1.10)	**4.31E-03**	1.11 (1.03, 1.18)	**1.09E-02**
1.04 (0.99, 1.09)	2.72E-01	0.86 (0.76, 0.97)	**4.05E-02**
1.04 (0.99, 1.09)	2.56E-01	0.93 (0.82, 1.06)	4.12E-01
1.04 (1.00, 1.07)	5.59E-02	1.05 (0.99, 1.10)	1.80E-01
0.87 (0.83, 0.91)	**1.78E-08**	0.74 (0.69, 0.79)	**1.13E-15**
1.14 (1.09, 1.20)	**3.44E-07**	1.37 (1.26, 1.49)	**1.77E-11**

; status, alcohol consumption, and self-rated health status. P values are correc
ı; VLDL, Very Low Density Lipoprotein; IDL, Intermediate Density Lipoprotein..

participants with mortality occured within two years of follow-up

Cerebrovascular diseases mortality		Cancer mortality	
**Adjusted HR (95% CI)**	**P value**	**Adjusted HR (95% CI)**	**P value**
0.97 (0.87, 1.07)	6.87E-01	0.94 (0.86, 1.03)	4.38E-01
0.97 (0.87, 1.07)	7.11E-01	0.95 (0.87, 1.03)	4.49E-01
0.98 (0.89, 1.08)	8.46E-01	0.95 (0.87, 1.04)	4.64E-01
0.99 (0.90, 1.10)	9.77E-01	0.98 (0.90, 1.06)	7.41E-01
1.00 (0.90, 1.10)	9.77E-01	0.99 (0.91, 1.07)	8.63E-01
0.99 (0.90, 1.09)	9.66E-01	0.97 (0.89, 1.06)	6.93E-01
0.96 (0.87, 1.06)	6.15E-01	0.94 (0.86, 1.02)	3.88E-01
0.99 (0.90, 1.10)	9.66E-01	1.00 (0.92, 1.08)	9.78E-01
0.99 (0.90, 1.10)	9.66E-01	1.00 (0.92, 1.09)	9.78E-01
0.99 (0.89, 1.09)	9.61E-01	1.00 (0.92, 1.08)	9.78E-01
1.00 (0.90, 1.10)	9.77E-01	1.00 (0.92, 1.09)	9.78E-01
1.00 (0.90, 1.11)	9.89E-01	1.00 (0.92, 1.09)	9.84E-01
0.99 (0.90, 1.10)	9.73E-01	1.01 (0.93, 1.09)	9.64E-01
0.99 (0.89, 1.09)	9.66E-01	1.00 (0.92, 1.08)	9.78E-01
1.00 (0.91, 1.11)	9.77E-01	0.96 (0.88, 1.04)	5.28E-01
1.00 (0.91, 1.11)	9.77E-01	0.96 (0.88, 1.04)	5.28E-01
1.00 (0.91, 1.11)	9.77E-01	0.96 (0.88, 1.04)	5.28E-01
0.99 (0.90, 1.10)	9.66E-01	0.97 (0.89, 1.05)	5.87E-01
0.97 (0.88, 1.08)	7.66E-01	0.97 (0.90, 1.05)	6.50E-01
1.01 (0.91, 1.11)	9.66E-01	0.96 (0.89, 1.05)	5.76E-01
1.01 (0.91, 1.11)	9.77E-01	0.96 (0.88, 1.04)	5.11E-01
1.00 (0.91, 1.11)	9.77E-01	0.95 (0.88, 1.03)	4.64E-01
1.00 (0.91, 1.11)	9.77E-01	0.95 (0.88, 1.03)	4.66E-01
1.00 (0.90, 1.10)	9.77E-01	0.95 (0.88, 1.03)	4.64E-01
0.99 (0.90, 1.10)	9.66E-01	0.97 (0.90, 1.05)	5.94E-01
0.97 (0.87, 1.07)	7.03E-01	0.98 (0.90, 1.05)	6.90E-01
1.02 (0.92, 1.12)	9.05E-01	0.96 (0.89, 1.04)	5.37E-01
1.01 (0.91, 1.11)	9.66E-01	0.95 (0.88, 1.03)	4.42E-01
1.00 (0.90, 1.11)	9.77E-01	0.95 (0.88, 1.03)	4.64E-01
1.01 (0.91, 1.12)	9.67E-01	0.95 (0.88, 1.03)	4.64E-01
0.99 (0.89, 1.10)	9.66E-01	0.95 (0.88, 1.02)	4.37E-01
1.05 (0.95, 1.17)	5.04E-01	0.96 (0.89, 1.03)	4.64E-01
1.05 (0.95, 1.17)	5.03E-01	0.96 (0.89, 1.03)	4.94E-01
1.04 (0.94, 1.16)	5.92E-01	0.96 (0.89, 1.03)	4.72E-01
0.99 (0.89, 1.09)	9.61E-01	0.96 (0.89, 1.04)	5.11E-01
1.05 (0.95, 1.16)	5.04E-01	0.95 (0.88, 1.03)	4.49E-01
1.06 (0.96, 1.17)	4.53E-01	0.95 (0.88, 1.03)	4.38E-01
0.97 (0.87, 1.07)	7.17E-01	0.97 (0.90, 1.04)	5.70E-01
1.11 (1.01, 1.23)	9.05E-02	0.94 (0.88, 1.02)	3.60E-01
1.13 (1.02, 1.24)	5.16E-02	0.94 (0.87, 1.01)	3.46E-01
1.08 (0.98, 1.19)	2.74E-01	0.95 (0.89, 1.03)	4.43E-01
1.03 (0.93, 1.14)	7.69E-01	0.98 (0.91, 1.05)	6.93E-01

0.97 (0.87, 1.07)	6.93E-01	0.97 (0.90, 1.05)	6.35E-01
0.96 (0.87, 1.07)	6.68E-01	0.97 (0.90, 1.05)	6.35E-01
0.94 (0.84, 1.04)	4.01E-01	0.97 (0.90, 1.04)	5.87E-01
0.96 (0.86, 1.06)	5.92E-01	0.97 (0.90, 1.05)	6.58E-01
0.95 (0.86, 1.06)	5.40E-01	0.97 (0.90, 1.05)	5.94E-01
0.97 (0.88, 1.08)	7.68E-01	0.98 (0.91, 1.06)	7.89E-01
1.06 (0.96, 1.17)	4.04E-01	1.00 (0.92, 1.08)	9.78E-01
0.94 (0.85, 1.05)	4.53E-01	0.96 (0.89, 1.04)	5.28E-01
0.94 (0.84, 1.04)	4.35E-01	0.96 (0.89, 1.04)	5.28E-01
0.91 (0.81, 1.01)	1.78E-01	0.96 (0.89, 1.04)	5.45E-01
0.93 (0.84, 1.04)	3.89E-01	0.96 (0.89, 1.03)	4.94E-01
0.93 (0.84, 1.03)	3.60E-01	0.95 (0.88, 1.03)	4.64E-01
0.94 (0.85, 1.05)	4.68E-01	0.97 (0.90, 1.05)	6.58E-01
1.09 (0.99, 1.20)	1.88E-01	1.01 (0.94, 1.09)	8.79E-01
0.92 (0.83, 1.02)	2.81E-01	0.94 (0.87, 1.01)	3.47E-01
0.92 (0.83, 1.02)	2.62E-01	0.94 (0.87, 1.01)	3.47E-01
0.87 (0.79, 0.97)	**3.50E-02**	0.94 (0.87, 1.02)	3.64E-01
0.92 (0.83, 1.03)	3.04E-01	0.94 (0.87, 1.01)	3.37E-01
0.93 (0.83, 1.03)	3.36E-01	0.93 (0.86, 1.01)	3.10E-01
0.91 (0.82, 1.01)	1.71E-01	0.94 (0.87, 1.02)	3.88E-01
1.07 (0.97, 1.18)	3.53E-01	1.00 (0.92, 1.08)	9.84E-01
0.93 (0.84, 1.03)	3.36E-01	0.93 (0.86, 1.01)	2.65E-01
0.93 (0.83, 1.03)	3.28E-01	0.93 (0.86, 1.01)	2.65E-01
0.89 (0.80, 0.98)	5.01E-02	0.94 (0.87, 1.02)	3.65E-01
0.94 (0.84, 1.04)	4.04E-01	0.93 (0.86, 1.00)	2.44E-01
0.94 (0.85, 1.04)	4.42E-01	0.93 (0.86, 1.00)	2.23E-01
0.92 (0.84, 1.02)	2.95E-01	0.93 (0.86, 1.01)	3.09E-01
1.04 (0.94, 1.16)	5.92E-01	0.97 (0.90, 1.05)	6.59E-01
1.24 (1.13, 1.36)	**1.26E-04**	1.10 (1.03, 1.18)	**4.59E-02**
1.24 (1.13, 1.36)	**1.26E-04**	1.10 (1.02, 1.18)	5.02E-02
1.19 (1.08, 1.31)	**1.96E-03**	1.13 (1.05, 1.21)	**6.33E-03**
1.26 (1.15, 1.39)	**3.22E-05**	1.06 (0.99, 1.13)	3.52E-01
1.28 (1.17, 1.41)	**1.06E-05**	1.04 (0.97, 1.11)	5.11E-01
1.21 (1.10, 1.33)	**4.64E-04**	1.10 (1.03, 1.18)	**3.45E-02**
1.22 (1.12, 1.34)	**1.26E-04**	1.04 (0.96, 1.12)	5.70E-01
1.17 (1.06, 1.29)	**6.71E-03**	1.13 (1.05, 1.21)	**1.07E-02**
1.17 (1.06, 1.29)	**6.81E-03**	1.13 (1.05, 1.21)	**1.07E-02**
1.16 (1.05, 1.28)	**1.19E-02**	1.12 (1.04, 1.21)	**1.70E-02**
1.17 (1.06, 1.29)	**7.59E-03**	1.13 (1.05, 1.21)	**8.99E-03**
1.17 (1.06, 1.28)	**8.42E-03**	1.13 (1.05, 1.22)	**8.48E-03**
1.18 (1.07, 1.29)	**5.14E-03**	1.12 (1.05, 1.21)	**1.36E-02**
1.21 (1.10, 1.33)	**7.94E-04**	1.09 (1.01, 1.18)	1.08E-01
0.91 (0.82, 1.01)	1.97E-01	1.06 (0.98, 1.14)	4.12E-01
0.91 (0.82, 1.01)	2.10E-01	1.06 (0.98, 1.14)	3.96E-01
0.96 (0.87, 1.07)	6.68E-01	1.05 (0.97, 1.14)	4.38E-01
0.89 (0.81, 0.99)	9.22E-02	1.06 (0.98, 1.15)	3.65E-01
0.88 (0.80, 0.98)	**4.86E-02**	1.06 (0.98, 1.15)	3.87E-01
0.95 (0.86, 1.05)	5.21E-01	1.07 (0.99, 1.15)	3.41E-01

0.87 (0.78, 0.97)	**4.96E-02**	1.01 (0.93, 1.09)	9.53E-01
0.82 (0.75, 0.91)	**4.64E-04**	0.99 (0.92, 1.07)	9.60E-01
0.82 (0.75, 0.90)	**4.02E-04**	1.00 (0.92, 1.08)	9.78E-01
0.89 (0.81, 0.99)	9.14E-02	1.00 (0.93, 1.08)	9.99E-01
0.84 (0.77, 0.93)	**3.45E-03**	1.00 (0.92, 1.08)	9.94E-01
0.86 (0.78, 0.94)	**8.31E-03**	0.99 (0.92, 1.08)	9.67E-01
0.92 (0.83, 1.02)	2.64E-01	1.03 (0.95, 1.11)	6.59E-01
0.94 (0.85, 1.04)	4.45E-01	0.97 (0.90, 1.05)	6.59E-01
1.04 (0.94, 1.16)	6.36E-01	0.88 (0.82, 0.95)	**8.21E-03**
1.17 (1.09, 1.26)	**1.34E-04**	1.12 (1.04, 1.21)	**1.58E-02**
1.17 (1.08, 1.26)	**4.02E-04**	1.11 (1.03, 1.20)	**3.97E-02**
1.11 (1.02, 1.21)	5.15E-02	1.08 (0.99, 1.17)	2.84E-01
0.84 (0.77, 0.91)	**2.79E-04**	0.95 (0.87, 1.03)	4.64E-01
0.95 (0.86, 1.04)	4.63E-01	0.95 (0.88, 1.03)	4.64E-01
1.08 (0.99, 1.18)	1.78E-01	1.01 (0.94, 1.08)	9.36E-01
1.10 (1.01, 1.20)	9.22E-02	1.01 (0.94, 1.07)	9.53E-01
1.00 (0.90, 1.10)	9.77E-01	1.01 (0.93, 1.09)	9.64E-01
0.97 (0.87, 1.07)	7.11E-01	1.02 (0.94, 1.10)	7.89E-01
1.00 (0.91, 1.11)	9.77E-01	0.94 (0.86, 1.02)	3.52E-01
0.95 (0.86, 1.04)	4.68E-01	1.01 (0.93, 1.10)	9.53E-01
0.86 (0.78, 0.96)	**2.47E-02**	1.08 (1.00, 1.17)	1.88E-01
1.06 (0.96, 1.18)	4.45E-01	0.93 (0.86, 1.01)	2.65E-01
1.04 (0.95, 1.15)	5.85E-01	1.01 (0.93, 1.10)	9.08E-01
0.85 (0.76, 0.95)	**1.25E-02**	1.08 (1.00, 1.16)	2.23E-01
0.95 (0.86, 1.06)	5.76E-01	1.11 (1.03, 1.20)	**4.59E-02**
0.90 (0.81, 1.01)	1.77E-01	1.11 (1.03, 1.19)	**4.27E-02**
1.16 (1.04, 1.30)	**2.90E-02**	0.98 (0.91, 1.06)	7.41E-01
1.07 (0.97, 1.19)	3.71E-01	0.89 (0.83, 0.97)	**3.84E-02**
0.89 (0.80, 0.98)	7.69E-02	1.06 (0.99, 1.14)	3.42E-01
1.09 (0.98, 1.20)	2.49E-01	1.01 (0.94, 1.09)	8.79E-01
1.06 (0.96, 1.17)	4.53E-01	1.01 (0.93, 1.08)	9.55E-01
1.23 (1.12, 1.35)	**2.60E-04**	1.04 (0.97, 1.13)	4.94E-01
0.95 (0.86, 1.05)	4.78E-01	0.97 (0.90, 1.05)	6.58E-01
0.83 (0.76, 0.90)	**1.26E-04**	1.06 (0.97, 1.15)	4.37E-01
1.21 (1.09, 1.35)	**3.45E-03**	0.94 (0.87, 1.00)	2.65E-01
1.21 (1.09, 1.34)	**3.10E-03**	0.93 (0.87, 1.00)	2.38E-01
1.09 (0.98, 1.21)	2.51E-01	0.97 (0.90, 1.04)	5.82E-01
0.98 (0.88, 1.08)	8.05E-01	1.03 (0.96, 1.11)	5.87E-01
0.88 (0.80, 0.97)	**2.73E-02**	1.00 (0.92, 1.08)	9.78E-01
0.95 (0.87, 1.04)	4.68E-01	0.97 (0.91, 1.04)	5.75E-01
0.94 (0.85, 1.03)	3.36E-01	0.95 (0.88, 1.02)	3.65E-01
1.00 (0.91, 1.10)	9.77E-01	1.01 (0.93, 1.09)	8.82E-01
1.09 (1.00, 1.20)	1.41E-01	1.03 (0.96, 1.11)	5.70E-01
0.97 (0.88, 1.07)	7.21E-01	1.03 (0.95, 1.12)	5.87E-01
0.94 (0.86, 1.03)	3.45E-01	0.96 (0.89, 1.03)	4.64E-01
0.93 (0.85, 1.01)	2.23E-01	0.94 (0.88, 1.01)	3.47E-01
1.03 (0.93, 1.15)	6.93E-01	1.04 (0.96, 1.13)	5.47E-01
1.12 (1.03, 1.22)	**2.48E-02**	1.05 (0.97, 1.13)	4.64E-01

0.99 (0.89, 1.09)	9.66E-01	1.04 (0.96, 1.12)	5.22E-01
0.96 (0.87, 1.05)	5.52E-01	0.95 (0.88, 1.02)	4.37E-01
0.95 (0.86, 1.04)	4.77E-01	0.95 (0.88, 1.02)	4.18E-01
1.06 (0.97, 1.17)	3.91E-01	1.05 (0.97, 1.14)	4.46E-01
1.14 (1.05, 1.25)	**1.11E-02**	1.07 (0.99, 1.15)	3.41E-01
0.99 (0.90, 1.10)	9.67E-01	1.07 (0.99, 1.15)	3.41E-01
0.97 (0.88, 1.07)	6.87E-01	0.94 (0.87, 1.02)	3.57E-01
0.96 (0.87, 1.06)	5.92E-01	0.94 (0.87, 1.01)	3.52E-01
1.07 (0.97, 1.18)	3.36E-01	1.06 (0.97, 1.14)	4.38E-01
1.11 (1.01, 1.21)	9.14E-02	1.04 (0.97, 1.13)	4.94E-01
0.89 (0.81, 0.99)	8.20E-02	1.16 (1.08, 1.26)	**2.12E-03**
1.16 (1.04, 1.29)	**2.79E-02**	0.86 (0.80, 0.93)	**1.32E-03**
1.16 (1.05, 1.29)	**1.86E-02**	0.83 (0.77, 0.90)	**5.83E-05**
1.04 (0.94, 1.16)	6.15E-01	1.02 (0.95, 1.10)	6.85E-01
0.99 (0.90, 1.10)	9.66E-01	0.96 (0.89, 1.04)	5.28E-01
0.87 (0.78, 0.97)	**4.24E-02**	0.90 (0.83, 0.98)	6.94E-02
1.11 (1.00, 1.24)	1.53E-01	1.09 (1.01, 1.17)	1.41E-01
1.05 (0.94, 1.17)	5.76E-01	1.11 (1.02, 1.20)	5.94E-02
1.19 (1.06, 1.33)	**1.44E-02**	1.05 (0.97, 1.13)	4.42E-01
1.03 (0.93, 1.14)	7.37E-01	0.99 (0.92, 1.07)	8.79E-01
1.22 (1.15, 1.30)	**1.80E-09**	0.96 (0.88, 1.05)	5.49E-01
0.87 (0.80, 0.95)	**8.38E-03**	1.03 (0.95, 1.12)	6.58E-01
0.86 (0.80, 0.93)	**1.65E-03**	1.02 (0.94, 1.11)	7.86E-01
1.00 (0.90, 1.10)	9.77E-01	1.05 (0.97, 1.13)	4.64E-01
0.96 (0.87, 1.07)	6.57E-01	0.99 (0.91, 1.06)	8.57E-01
1.06 (0.96, 1.17)	4.63E-01	1.01 (0.93, 1.09)	9.50E-01
0.95 (0.86, 1.05)	4.89E-01	1.00 (0.92, 1.08)	9.83E-01
0.94 (0.85, 1.04)	3.98E-01	0.99 (0.92, 1.07)	9.35E-01
1.16 (1.05, 1.28)	**1.69E-02**	1.06 (0.98, 1.14)	4.01E-01
1.02 (0.92, 1.13)	8.32E-01	0.98 (0.91, 1.06)	7.86E-01
1.00 (0.91, 1.11)	9.77E-01	0.92 (0.85, 0.99)	1.34E-01
1.05 (0.95, 1.16)	5.76E-01	1.15 (1.06, 1.25)	**6.33E-03**
1.24 (1.12, 1.37)	**1.94E-04**	1.09 (1.01, 1.17)	1.03E-01
1.00 (0.90, 1.11)	9.77E-01	0.98 (0.91, 1.06)	7.74E-01
1.04 (0.94, 1.15)	6.05E-01	0.95 (0.88, 1.02)	3.99E-01
1.01 (0.91, 1.12)	9.66E-01	0.95 (0.88, 1.02)	4.37E-01
0.94 (0.84, 1.04)	4.01E-01	0.94 (0.87, 1.02)	3.65E-01
1.10 (0.99, 1.21)	1.78E-01	1.10 (1.03, 1.19)	**4.59E-02**
1.07 (0.97, 1.18)	3.36E-01	1.11 (1.03, 1.19)	**3.60E-02**
1.27 (1.15, 1.40)	**3.22E-05**	1.04 (0.96, 1.11)	5.29E-01
0.85 (0.76, 0.95)	**1.78E-02**	0.98 (0.90, 1.06)	7.04E-01
0.85 (0.76, 0.95)	**1.96E-02**	0.96 (0.89, 1.04)	5.28E-01
1.02 (0.92, 1.13)	8.32E-01	0.96 (0.88, 1.04)	4.89E-01
1.01 (0.91, 1.12)	9.66E-01	0.95 (0.87, 1.03)	4.37E-01
1.10 (1.00, 1.21)	1.54E-01	1.00 (0.93, 1.08)	9.99E-01
1.04 (0.94, 1.16)	5.92E-01	1.03 (0.95, 1.11)	6.59E-01
0.97 (0.88, 1.08)	7.69E-01	1.04 (0.96, 1.12)	5.29E-01
1.02 (0.93, 1.13)	7.85E-01	0.95 (0.88, 1.03)	4.66E-01

0.83 (0.75, 0.93)	**3.89E-03**	1.00 (0.93, 1.09)	9.78E-01
0.79 (0.71, 0.88)	**1.92E-04**	0.93 (0.86, 1.01)	3.46E-01
0.92 (0.83, 1.03)	3.03E-01	1.02 (0.94, 1.10)	7.41E-01
1.12 (1.01, 1.23)	9.02E-02	1.08 (1.00, 1.16)	2.05E-01
0.97 (0.88, 1.08)	7.58E-01	0.94 (0.87, 1.02)	3.64E-01
0.93 (0.83, 1.03)	3.19E-01	0.91 (0.84, 0.99)	1.03E-01
0.91 (0.82, 1.02)	2.44E-01	0.99 (0.91, 1.07)	8.57E-01
0.73 (0.68, 0.80)	**2.90E-11**	0.97 (0.89, 1.05)	5.87E-01
1.23 (1.12, 1.35)	**1.94E-04**	0.88 (0.82, 0.95)	**8.63E-03**
0.86 (0.77, 0.95)	**1.96E-02**	0.98 (0.90, 1.06)	6.93E-01
1.08 (0.98, 1.20)	2.65E-01	0.88 (0.82, 0.96)	**2.28E-02**
0.83 (0.74, 0.93)	**6.31E-03**	0.99 (0.91, 1.07)	8.60E-01
0.87 (0.78, 0.97)	**4.20E-02**	0.96 (0.89, 1.05)	5.70E-01
0.97 (0.88, 1.08)	7.55E-01	0.97 (0.90, 1.05)	6.58E-01
0.91 (0.82, 1.01)	2.03E-01	1.02 (0.94, 1.10)	7.41E-01
1.42 (1.29, 1.57)	**6.61E-10**	0.84 (0.77, 0.90)	**9.86E-05**
0.85 (0.78, 0.92)	**8.51E-04**	1.00 (0.91, 1.09)	9.84E-01
1.25 (1.14, 1.38)	**9.48E-05**	0.80 (0.74, 0.87)	**2.48E-06**
0.81 (0.73, 0.89)	**2.22E-04**	1.03 (0.95, 1.12)	5.87E-01
0.86 (0.77, 0.95)	**1.19E-02**	0.96 (0.88, 1.05)	5.55E-01
1.16 (1.06, 1.28)	**1.09E-02**	0.93 (0.86, 1.00)	2.38E-01
1.04 (0.94, 1.15)	6.20E-01	1.18 (1.09, 1.28)	**8.62E-04**
1.23 (1.16, 1.31)	**8.84E-09**	0.95 (0.88, 1.03)	4.64E-01
1.05 (0.96, 1.16)	4.76E-01	0.77 (0.70, 0.84)	**1.38E-06**
0.79 (0.71, 0.89)	**4.02E-04**	1.23 (1.14, 1.34)	**1.41E-05**
0.99 (0.90, 1.09)	9.66E-01	0.80 (0.74, 0.87)	**9.11E-06**
0.99 (0.90, 1.10)	9.77E-01	0.82 (0.76, 0.89)	**5.02E-05**
1.10 (1.00, 1.22)	1.37E-01	0.76 (0.70, 0.83)	**1.02E-07**
0.97 (0.88, 1.08)	7.48E-01	0.89 (0.82, 0.97)	**4.59E-02**
1.03 (0.93, 1.14)	7.11E-01	0.85 (0.78, 0.92)	**1.83E-03**
0.80 (0.72, 0.89)	**5.80E-04**	0.84 (0.77, 0.91)	**9.48E-04**
1.15 (1.10, 1.21)	**1.30E-06**	0.84 (0.77, 0.92)	**1.48E-03**
1.09 (0.99, 1.20)	2.21E-01	0.86 (0.80, 0.94)	**5.11E-03**
1.14 (1.07, 1.20)	**1.77E-04**	0.97 (0.89, 1.05)	5.87E-01
1.06 (0.98, 1.16)	3.36E-01	1.15 (1.09, 1.22)	**3.08E-05**
1.05 (0.95, 1.16)	5.65E-01	1.08 (1.01, 1.15)	1.26E-01
1.06 (1.02, 1.11)	**2.20E-02**	0.70 (0.59, 0.85)	**2.64E-03**
0.82 (0.75, 0.90)	**1.58E-04**	1.09 (1.01, 1.18)	1.34E-01
1.26 (1.15, 1.39)	**2.18E-05**	0.97 (0.90, 1.06)	6.87E-01

UKB			
Other mortality		Sleep duration	
Adjusted HR (95% CI)	P value	Adjusted β (95% CI)	P value
1.00 (0.85, 1.16)	9.88E-01	0.02 (0.02, 0.02)	**4.13E-18**
1.00 (0.86, 1.16)	9.97E-01	0.02 (0.01, 0.02)	**1.60E-16**
1.00 (0.86, 1.17)	9.97E-01	0.02 (0.02, 0.02)	**9.59E-19**
1.04 (0.90, 1.20)	9.34E-01	0.02 (0.01, 0.02)	**6.63E-16**
1.05 (0.90, 1.21)	9.34E-01	0.02 (0.01, 0.02)	**9.34E-14**
1.02 (0.88, 1.19)	9.88E-01	0.02 (0.02, 0.02)	**3.05E-18**
0.99 (0.85, 1.15)	9.88E-01	0.02 (0.01, 0.02)	**8.58E-16**
0.99 (0.85, 1.16)	9.88E-01	0.02 (0.01, 0.02)	**3.27E-13**
1.00 (0.85, 1.16)	9.88E-01	0.02 (0.01, 0.02)	**8.28E-13**
1.00 (0.86, 1.17)	9.97E-01	0.02 (0.01, 0.02)	**4.96E-13**
1.01 (0.87, 1.17)	9.88E-01	0.01 (0.01, 0.02)	**9.29E-08**
1.01 (0.86, 1.17)	9.88E-01	0.01 (0.00, 0.01)	**4.58E-04**
1.01 (0.87, 1.18)	9.88E-01	0.02 (0.01, 0.02)	**1.84E-12**
0.99 (0.85, 1.15)	9.88E-01	0.02 (0.01, 0.02)	**3.50E-14**
0.99 (0.85, 1.15)	9.88E-01	0.02 (0.01, 0.02)	**2.30E-11**
0.99 (0.85, 1.15)	9.88E-01	0.01 (0.01, 0.02)	**9.72E-10**
1.00 (0.86, 1.16)	9.88E-01	0.02 (0.01, 0.02)	**6.60E-12**
0.99 (0.85, 1.15)	9.88E-01	0.01 (0.01, 0.02)	**4.19E-08**
0.98 (0.84, 1.14)	9.88E-01	0.01 (0.01, 0.01)	**2.51E-06**
1.00 (0.86, 1.17)	9.88E-01	0.01 (0.01, 0.02)	**1.13E-09**
0.99 (0.85, 1.15)	9.88E-01	0.01 (0.01, 0.02)	**3.09E-09**
0.96 (0.83, 1.12)	9.34E-01	0.00 (0.00, 0.01)	6.36E-01
0.97 (0.83, 1.12)	9.34E-01	0.00 (0.00, 0.01)	5.73E-02
0.97 (0.83, 1.12)	9.34E-01	0.00 (0.00, 0.00)	8.07E-01
0.98 (0.84, 1.14)	9.88E-01	-0.01 (-0.01, 0.00)	**1.34E-04**
0.98 (0.84, 1.14)	9.88E-01	-0.01 (-0.02, -0.01)	**2.68E-09**
0.98 (0.84, 1.14)	9.88E-01	0.00 (-0.01, 0.00)	3.96E-01
0.96 (0.82, 1.12)	9.34E-01	0.01 (0.01, 0.02)	**4.37E-08**
0.96 (0.83, 1.12)	9.34E-01	0.01 (0.01, 0.02)	**3.86E-10**
0.96 (0.83, 1.12)	9.34E-01	0.01 (0.01, 0.02)	**8.12E-08**
0.96 (0.83, 1.11)	9.34E-01	0.00 (0.00, 0.00)	1.16E-01
0.97 (0.84, 1.13)	9.57E-01	0.00 (0.00, 0.00)	4.07E-01
0.97 (0.84, 1.13)	9.57E-01	0.00 (0.00, 0.00)	**3.90E-02**
0.98 (0.84, 1.13)	9.83E-01	0.00 (-0.01, 0.00)	1.90E-01
0.96 (0.83, 1.12)	9.34E-01	0.02 (0.02, 0.02)	**8.45E-24**
1.03 (0.89, 1.19)	9.34E-01	0.01 (0.00, 0.01)	**4.17E-04**
1.04 (0.90, 1.19)	9.34E-01	0.01 (0.01, 0.01)	**1.04E-05**
1.02 (0.88, 1.18)	9.88E-01	0.01 (0.01, 0.02)	**1.71E-09**
1.05 (0.91, 1.21)	9.32E-01	0.00 (-0.01, 0.00)	7.49E-01
1.06 (0.92, 1.22)	8.49E-01	0.00 (-0.01, 0.00)	8.27E-02
1.03 (0.89, 1.19)	9.46E-01	0.01 (0.00, 0.01)	**3.02E-03**
1.01 (0.87, 1.16)	9.88E-01	0.03 (0.02, 0.03)	**4.32E-30**

1.04 (0.90, 1.20)	9.34E-01	-0.01 (-0.02, -0.01)	**1.14E-10**
1.03 (0.89, 1.19)	9.46E-01	-0.02 (-0.02, -0.01)	**1.82E-13**
1.01 (0.87, 1.17)	9.88E-01	-0.01 (-0.02, -0.01)	**1.01E-07**
1.01 (0.87, 1.16)	9.88E-01	-0.02 (-0.03, -0.02)	**1.92E-21**
1.00 (0.86, 1.15)	9.88E-01	-0.02 (-0.03, -0.02)	**2.61E-22**
1.03 (0.89, 1.19)	9.57E-01	-0.02 (-0.02, -0.02)	**3.86E-18**
1.13 (0.99, 1.29)	3.09E-01	0.02 (0.02, 0.03)	**5.41E-21**
0.99 (0.85, 1.15)	9.88E-01	-0.01 (-0.01, 0.00)	**2.97E-05**
0.98 (0.85, 1.14)	9.88E-01	-0.02 (-0.02, -0.01)	**8.65E-18**
0.96 (0.83, 1.12)	9.34E-01	-0.02 (-0.02, -0.02)	**1.04E-20**
0.97 (0.84, 1.13)	9.46E-01	-0.02 (-0.03, -0.02)	**5.53E-21**
0.96 (0.83, 1.12)	9.34E-01	-0.02 (-0.02, -0.01)	**4.69E-18**
0.99 (0.86, 1.15)	9.88E-01	-0.02 (-0.03, -0.02)	**3.94E-29**
1.16 (1.02, 1.32)	2.30E-01	0.02 (0.01, 0.02)	**4.00E-13**
0.94 (0.81, 1.09)	8.21E-01	-0.01 (-0.01, 0.00)	**4.58E-04**
0.93 (0.80, 1.09)	7.72E-01	-0.01 (-0.02, -0.01)	**1.44E-10**
0.91 (0.78, 1.06)	6.37E-01	-0.02 (-0.02, -0.01)	**3.24E-13**
0.93 (0.80, 1.08)	7.35E-01	-0.02 (-0.02, -0.01)	**1.40E-12**
0.93 (0.80, 1.08)	7.60E-01	-0.01 (-0.02, -0.01)	**2.74E-08**
0.91 (0.79, 1.06)	6.61E-01	-0.02 (-0.03, -0.02)	**7.44E-27**
1.13 (0.99, 1.30)	3.09E-01	0.02 (0.01, 0.02)	**4.59E-14**
0.91 (0.78, 1.06)	6.61E-01	0.00 (-0.01, 0.00)	**3.01E-02**
0.91 (0.78, 1.06)	6.38E-01	-0.01 (-0.02, -0.01)	**1.26E-07**
0.88 (0.76, 1.02)	3.63E-01	-0.01 (-0.02, -0.01)	**4.05E-08**
0.92 (0.79, 1.07)	6.63E-01	-0.01 (-0.02, -0.01)	**3.93E-11**
0.92 (0.79, 1.07)	6.84E-01	-0.01 (-0.02, -0.01)	**6.90E-07**
0.89 (0.77, 1.04)	5.00E-01	-0.02 (-0.03, -0.02)	**1.52E-24**
1.04 (0.90, 1.20)	9.34E-01	0.02 (0.02, 0.02)	**7.57E-19**
1.15 (1.01, 1.32)	2.41E-01	0.00 (-0.01, 0.00)	6.31E-01
1.15 (1.00, 1.31)	2.45E-01	0.00 (-0.01, 0.00)	6.79E-01
1.17 (1.02, 1.34)	2.30E-01	0.00 (0.00, 0.01)	7.43E-01
1.10 (0.96, 1.26)	5.69E-01	-0.01 (-0.01, 0.00)	**1.78E-02**
1.10 (0.95, 1.26)	6.34E-01	-0.01 (-0.01, 0.00)	**2.05E-03**
1.12 (0.97, 1.28)	4.48E-01	0.00 (0.00, 0.01)	7.68E-01
1.22 (1.07, 1.40)	7.85E-02	0.02 (0.02, 0.03)	**1.56E-28**
1.18 (1.02, 1.36)	2.18E-01	-0.01 (-0.02, -0.01)	**7.26E-06**
1.17 (1.02, 1.35)	2.30E-01	-0.01 (-0.02, -0.01)	**1.28E-05**
1.16 (1.00, 1.33)	2.41E-01	-0.01 (-0.01, -0.01)	**4.83E-05**
1.17 (1.02, 1.34)	2.30E-01	-0.01 (-0.02, -0.01)	**5.13E-08**
1.17 (1.02, 1.35)	2.30E-01	-0.01 (-0.02, -0.01)	**3.88E-09**
1.15 (1.00, 1.33)	2.41E-01	-0.01 (-0.01, 0.00)	**1.25E-04**
1.33 (1.16, 1.52)	**2.13E-03**	0.02 (0.02, 0.02)	**4.77E-20**
0.92 (0.79, 1.07)	6.67E-01	-0.02 (-0.03, -0.02)	**2.42E-19**
0.91 (0.79, 1.07)	6.63E-01	-0.02 (-0.02, -0.01)	**6.37E-16**
0.96 (0.83, 1.12)	9.34E-01	-0.02 (-0.02, -0.01)	**3.26E-11**
0.88 (0.76, 1.02)	3.92E-01	-0.03 (-0.03, -0.02)	**8.41E-28**
0.87 (0.75, 1.01)	2.88E-01	-0.03 (-0.03, -0.02)	**2.48E-30**
0.94 (0.81, 1.10)	8.78E-01	-0.02 (-0.03, -0.02)	**1.32E-17**

0.99 (0.85, 1.15)	9.88E-01	0.02 (0.01, 0.02)	**4.51E-18**
0.81 (0.71, 0.93)	6.40E-02	-0.03 (-0.03, -0.02)	**3.87E-39**
0.80 (0.70, 0.92)	5.70E-02	-0.02 (-0.02, -0.02)	**3.25E-19**
0.86 (0.74, 0.99)	2.41E-01	-0.02 (-0.02, -0.01)	**2.56E-14**
0.83 (0.72, 0.96)	1.66E-01	-0.03 (-0.04, -0.03)	**2.84E-45**
0.86 (0.75, 1.00)	2.41E-01	-0.03 (-0.04, -0.03)	**8.39E-54**
0.84 (0.73, 0.97)	2.07E-01	-0.02 (-0.02, -0.01)	**3.45E-16**
1.00 (0.86, 1.16)	9.97E-01	0.02 (0.02, 0.02)	**1.44E-19**
1.06 (0.90, 1.25)	8.81E-01	0.02 (0.01, 0.02)	**6.56E-13**
1.17 (1.04, 1.31)	1.28E-01	-0.01 (-0.02, -0.01)	**7.24E-07**
1.14 (1.01, 1.30)	2.41E-01	-0.01 (-0.02, -0.01)	**1.04E-07**
1.15 (1.00, 1.32)	2.41E-01	-0.01 (-0.01, 0.00)	**5.06E-03**
0.84 (0.74, 0.96)	1.32E-01	0.01 (0.00, 0.01)	**2.12E-02**
1.12 (0.94, 1.32)	6.35E-01	-0.02 (-0.02, -0.01)	**4.72E-13**
1.11 (1.00, 1.23)	2.81E-01	-0.02 (-0.02, -0.01)	**4.13E-16**
1.06 (0.93, 1.21)	7.60E-01	0.00 (-0.01, 0.00)	1.27E-01
1.14 (0.99, 1.30)	2.88E-01	0.01 (0.01, 0.02)	**5.25E-08**
0.85 (0.73, 0.98)	2.31E-01	0.02 (0.01, 0.02)	**1.16E-17**
1.13 (0.95, 1.34)	5.69E-01	0.00 (-0.01, 0.00)	5.30E-02
1.10 (0.93, 1.30)	6.67E-01	-0.01 (-0.01, 0.00)	**2.15E-04**
1.04 (0.89, 1.21)	9.34E-01	0.01 (0.00, 0.01)	**5.33E-03**
1.07 (0.91, 1.26)	7.78E-01	-0.01 (-0.01, 0.00)	**8.53E-04**
0.90 (0.76, 1.06)	6.35E-01	-0.02 (-0.03, -0.02)	**2.38E-20**
0.99 (0.85, 1.14)	9.88E-01	-0.03 (-0.03, -0.02)	**6.21E-32**
1.10 (0.95, 1.28)	6.35E-01	-0.03 (-0.03, -0.02)	**4.77E-34**
1.05 (0.91, 1.22)	9.13E-01	-0.02 (-0.03, -0.02)	**4.83E-24**
1.13 (0.96, 1.33)	5.02E-01	0.03 (0.02, 0.03)	**3.67E-30**
0.92 (0.79, 1.07)	6.67E-01	-0.04 (-0.04, -0.03)	**9.81E-58**
0.96 (0.83, 1.12)	9.34E-01	-0.03 (-0.03, -0.02)	**1.62E-34**
1.01 (0.88, 1.17)	9.88E-01	-0.02 (-0.02, -0.01)	**4.06E-16**
1.00 (0.87, 1.16)	9.88E-01	-0.04 (-0.04, -0.03)	**3.05E-57**
1.08 (0.94, 1.25)	6.84E-01	0.03 (0.03, 0.04)	**2.09E-42**
1.00 (0.86, 1.15)	9.88E-01	0.04 (0.03, 0.04)	**1.35E-56**
0.97 (0.84, 1.12)	9.34E-01	-0.04 (-0.04, -0.03)	**5.21E-55**
1.07 (0.92, 1.24)	7.60E-01	-0.04 (-0.04, -0.03)	**7.59E-57**
1.08 (0.93, 1.25)	6.84E-01	-0.02 (-0.03, -0.02)	**6.44E-23**
1.01 (0.88, 1.17)	9.88E-01	0.03 (0.03, 0.04)	**7.02E-44**
0.96 (0.83, 1.12)	9.34E-01	0.03 (0.03, 0.04)	**1.58E-49**
0.91 (0.79, 1.06)	6.37E-01	-0.04 (-0.05, -0.04)	**3.62E-84**
0.95 (0.83, 1.08)	8.49E-01	-0.04 (-0.05, -0.04)	**6.94E-82**
0.91 (0.79, 1.03)	5.20E-01	-0.02 (-0.03, -0.02)	**4.45E-22**
1.03 (0.89, 1.20)	9.46E-01	0.04 (0.04, 0.05)	**6.57E-71**
1.08 (0.95, 1.23)	6.61E-01	-0.01 (-0.01, 0.00)	**1.03E-02**
0.96 (0.82, 1.12)	9.34E-01	-0.03 (-0.04, -0.03)	**4.55E-50**
0.93 (0.82, 1.06)	6.84E-01	-0.01 (-0.02, -0.01)	**3.76E-10**
0.92 (0.81, 1.05)	6.35E-01	-0.03 (-0.04, -0.03)	**4.91E-51**
1.04 (0.89, 1.21)	9.34E-01	0.04 (0.03, 0.04)	**1.20E-61**
1.13 (1.00, 1.28)	2.41E-01	-0.01 (-0.01, -0.01)	**9.36E-07**

1.03 (0.90, 1.19)	9.34E-01	-0.03 (-0.03, -0.02)	**1.15E-36**
0.92 (0.80, 1.05)	6.35E-01	0.00 (0.00, 0.01)	**3.91E-02**
0.92 (0.81, 1.06)	6.68E-01	-0.03 (-0.04, -0.03)	**2.78E-42**
1.04 (0.90, 1.21)	9.34E-01	0.04 (0.03, 0.04)	**8.78E-60**
1.19 (1.05, 1.34)	1.28E-01	0.00 (0.00, 0.01)	2.25E-01
1.04 (0.90, 1.20)	9.34E-01	-0.03 (-0.04, -0.03)	**5.76E-48**
0.93 (0.81, 1.07)	7.35E-01	0.00 (-0.01, 0.00)	2.04E-01
0.94 (0.81, 1.08)	7.60E-01	-0.03 (-0.03, -0.03)	**7.81E-41**
1.04 (0.89, 1.20)	9.34E-01	0.03 (0.03, 0.04)	**1.38E-51**
1.12 (0.98, 1.28)	3.92E-01	0.01 (0.01, 0.02)	**1.23E-09**
1.14 (0.98, 1.32)	3.32E-01	-0.03 (-0.03, -0.02)	**6.92E-37**
0.84 (0.74, 0.97)	1.94E-01	-0.04 (-0.04, -0.03)	**1.11E-59**
0.84 (0.73, 0.98)	2.25E-01	0.00 (-0.01, 0.00)	1.94E-01
0.90 (0.80, 1.01)	3.00E-01	0.01 (0.01, 0.02)	**1.47E-09**
1.06 (0.93, 1.21)	7.60E-01	0.01 (0.01, 0.02)	**1.73E-09**
0.80 (0.68, 0.93)	9.14E-02	-0.02 (-0.03, -0.02)	**8.09E-20**
1.07 (0.92, 1.24)	7.60E-01	-0.02 (-0.03, -0.02)	**6.56E-26**
1.09 (0.93, 1.26)	6.84E-01	0.00 (0.00, 0.01)	7.78E-01
1.04 (0.90, 1.20)	9.34E-01	0.02 (0.02, 0.03)	**1.68E-21**
1.19 (1.05, 1.35)	1.07E-01	0.03 (0.03, 0.04)	**3.51E-47**
1.22 (1.11, 1.34)	**2.13E-03**	-0.03 (-0.04, -0.03)	**4.70E-50**
0.85 (0.76, 0.96)	1.28E-01	-0.03 (-0.04, -0.03)	**1.63E-49**
0.84 (0.75, 0.94)	5.70E-02	-0.02 (-0.02, -0.01)	**3.62E-15**
1.01 (0.87, 1.17)	9.88E-01	0.03 (0.03, 0.04)	**1.82E-44**
1.05 (0.91, 1.21)	9.34E-01	0.02 (0.01, 0.02)	**1.01E-12**
1.02 (0.88, 1.18)	9.88E-01	-0.03 (-0.04, -0.03)	**3.40E-50**
0.96 (0.83, 1.11)	9.34E-01	-0.03 (-0.04, -0.03)	**9.87E-53**
0.97 (0.83, 1.12)	9.34E-01	0.00 (0.00, 0.01)	1.71E-01
1.01 (0.87, 1.17)	9.88E-01	0.03 (0.03, 0.04)	**4.30E-52**
1.08 (0.94, 1.25)	6.84E-01	0.01 (0.01, 0.02)	**1.36E-08**
0.98 (0.85, 1.13)	9.88E-01	-0.01 (-0.02, -0.01)	**2.26E-08**
1.30 (1.13, 1.50)	**1.16E-02**	0.00 (0.00, 0.00)	9.97E-01
1.17 (1.01, 1.34)	2.34E-01	-0.02 (-0.02, -0.02)	**1.83E-19**
0.99 (0.86, 1.15)	9.88E-01	0.00 (0.00, 0.01)	**3.94E-02**
1.00 (0.86, 1.16)	9.97E-01	-0.02 (-0.02, -0.01)	**6.55E-18**
1.01 (0.87, 1.16)	9.88E-01	-0.01 (-0.01, 0.00)	**5.23E-04**
0.96 (0.83, 1.12)	9.34E-01	-0.02 (-0.03, -0.02)	**1.24E-23**
1.04 (0.90, 1.20)	9.34E-01	-0.01 (-0.02, -0.01)	**1.85E-10**
1.03 (0.89, 1.19)	9.46E-01		
1.08 (0.94, 1.25)	6.63E-01		
0.99 (0.85, 1.15)	9.88E-01	0.02 (0.01, 0.02)	**2.20E-16**
0.98 (0.84, 1.15)	9.88E-01	0.02 (0.01, 0.02)	**1.33E-14**
1.01 (0.87, 1.17)	9.88E-01	0.02 (0.01, 0.02)	**2.14E-14**
0.97 (0.84, 1.13)	9.57E-01	0.02 (0.02, 0.03)	**3.75E-22**
1.15 (1.01, 1.31)	2.41E-01	-0.01 (-0.01, 0.00)	**6.42E-05**
1.16 (1.01, 1.34)	2.41E-01	0.03 (0.02, 0.03)	**2.20E-30**
1.02 (0.88, 1.19)	9.83E-01	-0.01 (-0.02, -0.01)	**2.14E-06**
0.94 (0.81, 1.10)	8.56E-01	-0.02 (-0.02, -0.02)	**4.97E-17**

0.99 (0.85, 1.15)	9.88E-01	-0.01 (-0.02, -0.01)	**6.26E-09**
0.96 (0.82, 1.13)	9.34E-01	-0.02 (-0.03, -0.02)	**2.71E-19**
1.04 (0.90, 1.21)	9.34E-01	-0.01 (-0.01, 0.00)	**4.58E-04**
1.03 (0.89, 1.19)	9.46E-01	0.00 (0.00, 0.01)	1.51E-01
0.97 (0.84, 1.13)	9.46E-01	0.01 (0.00, 0.01)	**1.40E-02**
0.97 (0.84, 1.13)	9.46E-01	0.00 (-0.01, 0.00)	1.04E-01
0.95 (0.81, 1.11)	9.23E-01	0.01 (0.01, 0.02)	**4.76E-10**
0.81 (0.70, 0.94)	9.14E-02	-0.01 (-0.01, 0.00)	**1.85E-04**
0.88 (0.76, 1.02)	3.32E-01	0.02 (0.02, 0.03)	**1.52E-20**
0.93 (0.79, 1.09)	7.60E-01	-0.01 (-0.01, 0.00)	**3.67E-05**
0.84 (0.72, 0.99)	2.41E-01	0.00 (-0.01, 0.00)	3.24E-01
0.92 (0.78, 1.08)	6.84E-01	0.01 (0.01, 0.02)	**1.15E-07**
0.90 (0.76, 1.05)	6.13E-01	0.01 (0.00, 0.01)	**1.17E-02**
0.97 (0.84, 1.13)	9.46E-01	0.01 (0.01, 0.02)	**6.26E-08**
0.97 (0.84, 1.13)	9.60E-01	-0.02 (-0.03, -0.02)	**1.58E-27**
0.91 (0.79, 1.06)	6.37E-01	0.02 (0.02, 0.03)	**4.10E-23**
0.93 (0.80, 1.08)	7.60E-01	-0.03 (-0.03, -0.02)	**1.55E-30**
0.85 (0.74, 0.98)	2.30E-01	-0.02 (-0.02, -0.01)	**2.72E-12**
0.92 (0.79, 1.07)	6.84E-01	0.02 (0.02, 0.03)	**1.14E-20**
0.87 (0.75, 1.01)	3.09E-01	0.00 (0.00, 0.01)	4.07E-01
1.08 (0.94, 1.25)	6.84E-01	0.02 (0.01, 0.02)	**1.01E-12**
1.12 (0.96, 1.30)	5.16E-01	0.00 (0.00, 0.01)	1.73E-01
1.25 (1.14, 1.36)	**2.08E-04**	0.00 (-0.01, 0.00)	3.72E-01
0.90 (0.76, 1.05)	5.73E-01	0.01 (0.01, 0.01)	**1.20E-06**
1.04 (0.89, 1.21)	9.34E-01	-0.03 (-0.03, -0.02)	**2.97E-32**
0.97 (0.83, 1.12)	9.34E-01	0.00 (-0.01, 0.00)	6.84E-01
0.86 (0.74, 1.00)	2.41E-01	0.01 (0.00, 0.01)	**7.04E-03**
0.96 (0.82, 1.11)	9.34E-01	0.00 (0.00, 0.01)	**4.30E-02**
0.91 (0.78, 1.06)	6.35E-01	0.02 (0.01, 0.02)	**2.29E-12**
0.85 (0.72, 1.00)	2.41E-01	0.00 (0.00, 0.01)	**3.09E-02**
0.89 (0.76, 1.05)	5.52E-01	0.00 (0.00, 0.00)	8.57E-01
1.13 (1.03, 1.25)	1.41E-01	-0.01 (-0.01, 0.00)	**2.98E-03**
1.15 (1.00, 1.32)	2.81E-01	0.00 (0.00, 0.01)	1.93E-01
0.98 (0.84, 1.15)	9.88E-01	0.00 (0.00, 0.01)	**3.18E-02**
0.87 (0.72, 1.05)	5.52E-01	0.03 (0.02, 0.03)	**4.15E-32**
1.06 (0.93, 1.22)	7.86E-01	0.00 (-0.01, 0.00)	5.81E-02
1.08 (1.03, 1.12)	**3.70E-02**	0.02 (0.01, 0.02)	**5.73E-15**
0.82 (0.72, 0.94)	7.47E-02	-0.02 (-0.02, -0.01)	**1.69E-11**
0.99 (0.85, 1.16)	9.88E-01	0.01 (0.01, 0.01)	**2.18E-06**
		-0.01 (-0.02, -0.01)	**4.31E-10**
		-0.02 (-0.03, -0.02)	**1.25E-21**
		-0.02 (-0.02, -0.01)	**3.02E-14**
		-0.03 (-0.03, -0.02)	**1.39E-28**
		-0.02 (-0.02, -0.01)	**6.16E-17**
		-0.03 (-0.03, -0.02)	**2.48E-33**
		0.01 (0.00, 0.01)	**1.83E-04**
		-0.02 (-0.03, -0.02)	**1.51E-28**
		-0.02 (-0.02, -0.01)	**4.11E-12**

0.00 (0.00, 0.01)	**4.85E-02**
0.00 (-0.01, 0.00)	**2.46E-02**
-0.03 (-0.03, -0.02)	**1.59E-34**
0.01 (0.01, 0.01)	**6.88E-06**
-0.01 (-0.01, 0.00)	**1.26E-04**
-0.03 (-0.03, -0.02)	**3.38E-34**
-0.01 (-0.02, -0.01)	**5.69E-08**
0.01 (0.01, 0.02)	**1.99E-07**
-0.02 (-0.02, -0.01)	**6.00E-17**
-0.02 (-0.02, -0.01)	**1.10E-10**
-0.01 (-0.01, 0.00)	**2.83E-04**
0.01 (0.01, 0.02)	**2.07E-09**
-0.02 (-0.02, -0.01)	**1.07E-14**
-0.02 (-0.02, -0.01)	**2.66E-14**
0.00 (0.00, 0.01)	4.41E-01
-0.03 (-0.03, -0.02)	**1.12E-24**
0.01 (0.01, 0.01)	**9.34E-07**

All causes mortality		Ischaemic heart diseases mortality	
Adjusted HR (95% CI)	P value	Adjusted HR (95% CI)	P value
0.99 (0.97, 1.00)	1.47E-01	1.04 (1.00, 1.08)	8.42E-02
0.99 (0.97, 1.00)	1.16E-01	1.04 (0.99, 1.08)	1.11E-01
0.99 (0.98, 1.01)	2.26E-01	1.04 (1.00, 1.09)	7.89E-02
0.98 (0.97, 1.00)	**2.31E-02**	1.03 (0.98, 1.07)	2.98E-01
0.98 (0.96, 0.99)	**6.77E-03**	1.02 (0.97, 1.06)	4.50E-01
0.99 (0.97, 1.00)	8.49E-02	1.03 (0.99, 1.08)	1.59E-01
0.99 (0.98, 1.00)	1.56E-01	1.04 (1.00, 1.08)	9.01E-02
0.96 (0.94, 0.97)	**1.18E-08**	1.02 (0.98, 1.07)	4.27E-01
0.96 (0.94, 0.97)	**3.03E-09**	1.02 (0.97, 1.06)	5.46E-01
0.96 (0.94, 0.97)	**1.09E-08**	1.01 (0.97, 1.06)	5.84E-01
0.94 (0.92, 0.95)	**1.30E-16**	0.98 (0.94, 1.03)	4.36E-01
0.92 (0.91, 0.94)	**2.10E-23**	0.95 (0.91, 1.00)	6.86E-02
0.95 (0.94, 0.97)	**3.54E-10**	1.01 (0.96, 1.05)	8.21E-01
0.96 (0.95, 0.98)	**4.60E-07**	1.03 (0.98, 1.07)	2.90E-01
0.94 (0.93, 0.96)	**1.97E-14**	1.00 (0.96, 1.05)	9.27E-01
0.93 (0.92, 0.95)	**1.07E-18**	0.99 (0.95, 1.04)	7.09E-01
0.95 (0.93, 0.96)	**5.94E-13**	1.01 (0.96, 1.05)	7.76E-01
0.93 (0.92, 0.94)	**8.34E-21**	0.98 (0.93, 1.02)	3.45E-01
0.92 (0.91, 0.94)	**1.05E-23**	0.95 (0.91, 1.00)	6.97E-02
0.94 (0.92, 0.95)	**2.88E-17**	1.00 (0.95, 1.04)	8.62E-01
0.93 (0.92, 0.95)	**3.72E-19**	0.99 (0.95, 1.04)	7.15E-01
0.91 (0.89, 0.92)	**4.72E-38**	0.91 (0.87, 0.96)	**2.39E-04**
0.91 (0.89, 0.92)	**6.51E-38**	0.93 (0.88, 0.97)	**1.87E-03**
0.91 (0.89, 0.92)	**1.08E-36**	0.91 (0.87, 0.96)	**1.71E-04**
0.90 (0.89, 0.92)	**2.26E-39**	0.87 (0.83, 0.92)	**4.44E-08**
0.91 (0.90, 0.92)	**1.95E-36**	0.86 (0.82, 0.90)	**1.37E-09**
0.91 (0.89, 0.92)	**5.36E-38**	0.90 (0.86, 0.94)	**1.87E-05**
0.92 (0.91, 0.94)	**2.27E-24**	0.98 (0.93, 1.02)	3.78E-01
0.95 (0.93, 0.96)	**4.07E-13**	1.00 (0.95, 1.05)	9.56E-01
0.94 (0.93, 0.95)	**7.07E-16**	0.99 (0.94, 1.03)	5.93E-01
0.92 (0.91, 0.94)	**2.04E-26**	0.95 (0.90, 0.99)	**2.39E-02**
0.93 (0.91, 0.94)	**4.19E-22**	0.94 (0.90, 0.99)	**1.88E-02**
0.94 (0.92, 0.95)	**2.29E-16**	0.96 (0.92, 1.01)	1.24E-01
0.91 (0.90, 0.93)	**3.52E-33**	0.91 (0.87, 0.96)	**2.12E-04**
0.97 (0.96, 0.98)	**9.93E-05**	1.04 (1.00, 1.09)	7.77E-02
0.99 (0.98, 1.00)	1.77E-01	0.99 (0.94, 1.03)	6.21E-01
1.00 (0.98, 1.01)	5.35E-01	0.99 (0.95, 1.04)	8.21E-01
1.01 (1.00, 1.02)	2.05E-01	1.03 (0.98, 1.07)	3.32E-01
0.97 (0.96, 0.99)	**3.56E-04**	0.93 (0.88, 0.97)	**2.10E-03**
0.97 (0.95, 0.98)	**7.69E-06**	0.90 (0.86, 0.95)	**6.26E-05**
0.99 (0.98, 1.00)	1.86E-01	0.98 (0.94, 1.03)	4.41E-01
1.03 (1.01, 1.04)	**9.77E-05**	1.11 (1.06, 1.15)	**5.03E-06**

0.93 (0.91, 0.94)	**3.50E-23**	0.88 (0.84, 0.92)	**3.85E-07**
0.93 (0.91, 0.94)	**3.21E-23**	0.87 (0.83, 0.91)	**1.00E-08**
0.94 (0.92, 0.95)	**7.10E-17**	0.88 (0.84, 0.93)	**6.36E-07**
0.91 (0.90, 0.93)	**8.91E-31**	0.84 (0.80, 0.88)	**1.52E-11**
0.91 (0.90, 0.92)	**1.60E-33**	0.84 (0.80, 0.88)	**1.91E-12**
0.93 (0.91, 0.94)	**1.62E-22**	0.87 (0.83, 0.91)	**7.96E-09**
1.04 (1.03, 1.06)	**1.25E-08**	1.11 (1.06, 1.15)	**4.63E-06**
0.91 (0.89, 0.92)	**2.19E-38**	0.90 (0.86, 0.94)	**1.28E-05**
0.89 (0.88, 0.91)	**3.19E-50**	0.86 (0.82, 0.90)	**1.71E-09**
0.89 (0.88, 0.90)	**6.10E-52**	0.86 (0.82, 0.90)	**2.26E-10**
0.88 (0.87, 0.90)	**2.00E-56**	0.85 (0.81, 0.89)	**4.67E-11**
0.88 (0.87, 0.90)	**1.81E-57**	0.86 (0.82, 0.90)	**2.69E-10**
0.89 (0.88, 0.90)	**9.23E-51**	0.84 (0.80, 0.88)	**1.10E-12**
1.03 (1.02, 1.05)	**2.96E-05**	1.10 (1.06, 1.15)	**9.15E-06**
0.90 (0.89, 0.91)	**2.41E-44**	0.89 (0.85, 0.93)	**2.14E-06**
0.88 (0.87, 0.89)	**1.63E-60**	0.88 (0.84, 0.93)	**4.57E-07**
0.88 (0.87, 0.89)	**3.84E-63**	0.88 (0.84, 0.93)	**5.34E-07**
0.88 (0.86, 0.89)	**3.61E-66**	0.87 (0.83, 0.91)	**1.56E-08**
0.88 (0.87, 0.89)	**1.19E-61**	0.88 (0.84, 0.92)	**2.62E-07**
0.87 (0.86, 0.89)	**1.52E-69**	0.85 (0.81, 0.89)	**4.67E-11**
1.01 (0.99, 1.02)	3.14E-01	1.08 (1.04, 1.13)	**2.37E-04**
0.91 (0.90, 0.92)	**9.66E-36**	0.91 (0.87, 0.95)	**7.53E-05**
0.89 (0.88, 0.91)	**8.82E-51**	0.90 (0.86, 0.94)	**1.28E-05**
0.90 (0.89, 0.92)	**2.71E-41**	0.92 (0.88, 0.96)	**3.60E-04**
0.88 (0.87, 0.90)	**7.26E-60**	0.88 (0.84, 0.92)	**1.20E-07**
0.89 (0.88, 0.90)	**3.26E-54**	0.89 (0.85, 0.93)	**1.40E-06**
0.88 (0.87, 0.90)	**1.79E-62**	0.87 (0.83, 0.91)	**3.28E-09**
0.99 (0.98, 1.01)	3.22E-01	1.06 (1.02, 1.10)	**1.02E-02**
1.07 (1.05, 1.09)	**1.78E-15**	0.95 (0.90, 1.01)	1.07E-01
1.08 (1.06, 1.10)	**3.51E-21**	0.96 (0.91, 1.02)	2.43E-01
1.09 (1.07, 1.11)	**9.86E-27**	0.98 (0.92, 1.03)	4.41E-01
1.06 (1.05, 1.08)	**1.64E-13**	0.94 (0.89, 1.00)	5.53E-02
1.06 (1.04, 1.07)	**1.10E-10**	0.93 (0.88, 0.98)	**1.92E-02**
1.09 (1.07, 1.10)	**3.62E-25**	0.99 (0.94, 1.04)	7.18E-01
1.04 (1.03, 1.06)	**3.68E-08**	1.05 (1.01, 1.09)	**3.50E-02**
1.05 (1.03, 1.07)	**4.02E-08**	0.90 (0.85, 0.96)	**1.18E-03**
1.06 (1.04, 1.07)	**4.53E-10**	0.91 (0.86, 0.97)	**4.64E-03**
1.06 (1.04, 1.08)	**2.14E-11**	0.93 (0.87, 0.98)	**1.63E-02**
1.05 (1.03, 1.06)	**1.83E-07**	0.90 (0.85, 0.95)	**8.57E-04**
1.04 (1.02, 1.06)	**1.92E-06**	0.89 (0.84, 0.95)	**4.08E-04**
1.06 (1.04, 1.08)	**1.71E-11**	0.92 (0.87, 0.98)	**1.03E-02**
1.06 (1.05, 1.08)	**1.42E-16**	1.02 (0.98, 1.07)	4.14E-01
0.98 (0.96, 0.99)	**6.32E-03**	0.88 (0.84, 0.93)	**4.99E-06**
0.98 (0.97, 1.00)	**2.93E-02**	0.90 (0.86, 0.95)	**1.02E-04**
0.99 (0.97, 1.00)	1.39E-01	0.92 (0.87, 0.96)	**1.16E-03**
0.97 (0.96, 0.99)	**1.12E-03**	0.88 (0.83, 0.92)	**1.53E-06**
0.97 (0.96, 0.99)	**4.01E-04**	0.87 (0.83, 0.92)	**1.03E-06**
0.98 (0.97, 1.00)	**4.10E-02**	0.89 (0.84, 0.93)	**1.41E-05**

1.02 (1.00, 1.03)	**1.16E-02**	1.04 (0.99, 1.08)	1.39E-01
0.87 (0.86, 0.89)	**2.34E-67**	0.86 (0.82, 0.90)	**1.35E-09**
0.91 (0.89, 0.92)	**2.50E-36**	0.90 (0.86, 0.94)	**1.29E-05**
0.93 (0.91, 0.94)	**9.32E-24**	0.91 (0.87, 0.95)	**1.07E-04**
0.88 (0.86, 0.89)	**6.78E-65**	0.86 (0.82, 0.90)	**7.55E-10**
0.87 (0.86, 0.88)	**2.38E-74**	0.85 (0.81, 0.89)	**4.21E-11**
0.92 (0.90, 0.93)	**4.32E-28**	0.91 (0.87, 0.95)	**5.56E-05**
0.99 (0.98, 1.01)	4.58E-01	1.07 (1.02, 1.12)	**4.89E-03**
1.04 (1.02, 1.06)	**1.16E-05**	1.14 (1.08, 1.21)	**6.37E-06**
1.01 (1.00, 1.03)	9.90E-02	0.95 (0.90, 1.00)	9.55E-02
1.01 (0.99, 1.02)	3.93E-01	0.93 (0.88, 0.99)	**2.58E-02**
1.02 (1.01, 1.04)	**4.12E-03**	1.00 (0.95, 1.05)	8.69E-01
0.98 (0.96, 0.99)	**5.82E-03**	1.01 (0.96, 1.06)	7.64E-01
1.00 (0.99, 1.02)	5.78E-01	0.90 (0.85, 0.96)	**8.17E-04**
1.00 (0.98, 1.01)	5.96E-01	0.90 (0.84, 0.95)	**5.79E-04**
1.04 (1.02, 1.06)	**3.13E-06**	0.94 (0.89, 0.99)	**3.94E-02**
0.99 (0.98, 1.01)	3.68E-01	1.10 (1.05, 1.17)	**6.57E-04**
1.02 (1.00, 1.03)	6.60E-02	1.10 (1.03, 1.17)	**7.27E-03**
1.03 (1.02, 1.05)	**5.09E-05**	0.99 (0.94, 1.04)	6.83E-01
1.03 (1.01, 1.04)	**2.76E-04**	0.96 (0.91, 1.00)	9.58E-02
1.03 (1.02, 1.05)	**9.52E-05**	1.09 (1.03, 1.14)	**2.41E-03**
0.97 (0.95, 0.98)	**5.51E-05**	0.97 (0.92, 1.02)	3.32E-01
0.98 (0.97, 0.99)	**4.50E-03**	0.90 (0.86, 0.94)	**7.87E-07**
0.98 (0.96, 0.99)	**1.55E-03**	0.88 (0.84, 0.92)	**3.47E-08**
0.98 (0.96, 0.99)	**1.24E-03**	0.88 (0.84, 0.92)	**1.41E-08**
0.98 (0.96, 0.99)	**4.02E-03**	0.90 (0.86, 0.94)	**9.28E-07**
1.02 (1.01, 1.04)	**1.67E-03**	1.13 (1.09, 1.18)	**4.58E-08**
0.92 (0.91, 0.94)	**9.51E-25**	0.84 (0.80, 0.88)	**2.70E-13**
0.96 (0.94, 0.97)	**4.37E-09**	0.88 (0.84, 0.92)	**3.75E-08**
0.98 (0.97, 1.00)	**3.32E-02**	0.93 (0.89, 0.97)	**6.52E-04**
0.92 (0.91, 0.94)	**5.17E-24**	0.83 (0.79, 0.87)	**1.41E-14**
1.06 (1.04, 1.07)	**2.98E-13**	1.15 (1.10, 1.20)	**9.98E-10**
1.14 (1.12, 1.15)	**4.27E-64**	1.30 (1.25, 1.36)	**2.24E-30**
0.93 (0.92, 0.95)	**6.28E-21**	0.82 (0.79, 0.86)	**4.31E-20**
0.93 (0.92, 0.94)	**1.95E-22**	0.81 (0.78, 0.85)	**3.28E-22**
0.97 (0.95, 0.98)	**1.03E-06**	0.92 (0.89, 0.96)	**4.57E-05**
1.05 (1.04, 1.07)	**8.28E-12**	1.18 (1.13, 1.22)	**7.98E-15**
1.11 (1.09, 1.13)	**3.14E-46**	1.20 (1.15, 1.25)	**2.49E-15**
0.89 (0.88, 0.90)	**1.31E-66**	0.81 (0.78, 0.84)	**7.48E-31**
0.87 (0.86, 0.89)	**1.29E-85**	0.78 (0.75, 0.81)	**2.63E-38**
0.97 (0.96, 0.99)	**1.56E-04**	0.94 (0.90, 0.97)	**1.16E-03**
1.11 (1.09, 1.12)	**4.59E-54**	1.21 (1.17, 1.25)	**1.91E-27**
1.02 (1.00, 1.03)	**1.21E-02**	0.98 (0.94, 1.02)	3.83E-01
0.92 (0.92, 0.93)	**1.48E-100**	0.91 (0.90, 0.93)	**1.14E-24**
0.93 (0.92, 0.93)	**4.98E-111**	0.93 (0.91, 0.95)	**2.02E-12**
0.95 (0.93, 0.96)	**7.66E-15**	0.86 (0.83, 0.89)	**4.36E-16**
1.10 (1.09, 1.11)	**2.58E-98**	1.13 (1.11, 1.15)	**3.08E-31**
1.02 (1.00, 1.03)	**2.91E-02**	1.05 (1.01, 1.09)	**2.26E-02**

0.92 (0.91, 0.93)	**2.22E-106**	0.91 (0.89, 0.92)	**1.30E-28**
0.92 (0.90, 0.93)	**1.30E-43**	0.91 (0.88, 0.94)	**2.62E-07**
0.96 (0.94, 0.97)	**2.36E-09**	0.89 (0.86, 0.93)	**6.27E-08**
1.10 (1.09, 1.11)	**1.81E-120**	1.12 (1.10, 1.14)	**1.98E-31**
1.09 (1.08, 1.11)	**6.11E-37**	1.13 (1.09, 1.17)	**7.82E-10**
0.90 (0.89, 0.91)	**6.06E-118**	0.87 (0.86, 0.89)	**4.06E-36**
0.92 (0.90, 0.93)	**2.11E-35**	0.88 (0.85, 0.91)	**1.56E-11**
0.94 (0.93, 0.95)	**3.02E-19**	0.91 (0.88, 0.94)	**1.83E-06**
1.08 (1.06, 1.09)	**1.50E-32**	1.11 (1.08, 1.14)	**6.16E-13**
1.10 (1.08, 1.11)	**3.70E-28**	1.02 (0.98, 1.06)	4.36E-01
0.90 (0.89, 0.92)	**6.20E-40**	0.96 (0.92, 1.00)	7.61E-02
0.92 (0.91, 0.93)	**8.73E-37**	0.93 (0.90, 0.96)	**9.50E-05**
0.96 (0.95, 0.98)	**3.33E-07**	1.03 (0.99, 1.07)	1.50E-01
0.99 (0.97, 1.00)	5.52E-02	1.01 (0.98, 1.05)	4.41E-01
1.02 (1.00, 1.03)	**2.93E-02**	1.10 (1.06, 1.14)	**1.57E-06**
0.99 (0.97, 1.00)	**4.47E-02**	0.92 (0.89, 0.95)	**8.00E-06**
0.98 (0.96, 0.99)	**7.15E-04**	0.91 (0.88, 0.95)	**1.42E-06**
1.03 (1.02, 1.05)	**3.49E-05**	0.97 (0.94, 1.01)	1.58E-01
1.01 (1.00, 1.02)	1.86E-01	1.05 (1.02, 1.09)	**2.42E-03**
1.06 (1.05, 1.08)	**4.21E-16**	1.17 (1.12, 1.22)	**9.18E-12**
0.95 (0.94, 0.96)	**1.59E-11**	0.88 (0.84, 0.91)	**3.49E-09**
0.95 (0.94, 0.97)	**5.03E-11**	0.89 (0.86, 0.93)	**2.44E-07**
0.97 (0.95, 0.98)	**4.84E-05**	0.86 (0.82, 0.91)	**5.32E-09**
1.04 (1.03, 1.06)	**1.23E-07**	1.10 (1.06, 1.14)	**5.49E-08**
1.12 (1.11, 1.14)	**1.37E-50**	1.06 (1.01, 1.11)	**2.44E-02**
0.90 (0.89, 0.92)	**7.06E-44**	0.88 (0.85, 0.92)	**2.37E-08**
0.90 (0.88, 0.91)	**5.64E-52**	0.88 (0.85, 0.92)	**1.29E-08**
1.03 (1.02, 1.05)	**1.25E-05**	1.01 (0.96, 1.06)	7.97E-01
1.05 (1.04, 1.07)	**8.62E-11**	1.16 (1.11, 1.21)	**3.79E-10**
0.92 (0.91, 0.93)	**1.22E-26**	0.97 (0.93, 1.02)	3.04E-01
0.98 (0.97, 1.00)	**1.33E-02**	0.97 (0.93, 1.01)	1.71E-01
1.10 (1.08, 1.11)	**2.83E-27**	0.95 (0.90, 1.00)	8.46E-02
0.90 (0.89, 0.91)	**2.80E-41**	0.85 (0.81, 0.89)	**1.63E-11**
0.93 (0.92, 0.94)	**7.44E-21**	0.94 (0.89, 0.98)	**8.28E-03**
0.88 (0.87, 0.89)	**9.17E-61**	0.86 (0.82, 0.90)	**3.43E-10**
0.92 (0.90, 0.93)	**7.57E-30**	0.88 (0.84, 0.93)	**5.91E-07**
0.90 (0.89, 0.91)	**2.02E-42**	0.84 (0.80, 0.88)	**2.85E-12**
0.91 (0.89, 0.92)	**4.30E-37**	0.86 (0.82, 0.90)	**2.10E-09**
0.97 (0.96, 0.98)	**4.55E-05**	1.03 (0.99, 1.08)	2.08E-01
0.96 (0.94, 0.97)	**9.54E-09**	1.02 (0.98, 1.06)	4.45E-01
1.02 (1.01, 1.03)	**7.15E-03**	1.09 (1.05, 1.14)	**5.52E-05**
1.02 (1.01, 1.04)	**2.27E-03**	1.05 (1.00, 1.09)	5.53E-02
0.95 (0.93, 0.96)	**7.09E-11**	0.90 (0.86, 0.95)	**6.02E-05**
0.99 (0.97, 1.00)	1.70E-01	1.09 (1.05, 1.14)	**1.49E-04**
0.94 (0.93, 0.96)	**9.97E-14**	0.87 (0.83, 0.92)	**2.62E-07**
0.96 (0.94, 0.97)	**6.11E-08**	0.90 (0.86, 0.95)	**7.88E-05**

0.94 (0.93, 0.96)	**3.79E-14**	0.88 (0.84, 0.92)	**6.53E-07**
0.97 (0.96, 0.99)	**2.33E-03**	0.87 (0.83, 0.92)	**1.45E-06**
0.91 (0.90, 0.92)	**3.82E-36**	0.90 (0.86, 0.94)	**1.14E-05**
0.94 (0.92, 0.95)	**6.46E-17**	0.98 (0.93, 1.02)	3.70E-01
0.96 (0.94, 0.97)	**1.52E-09**	0.98 (0.94, 1.02)	4.09E-01
0.87 (0.86, 0.89)	**6.37E-69**	0.84 (0.81, 0.88)	**3.89E-12**
0.88 (0.87, 0.90)	**1.60E-49**	0.85 (0.80, 0.89)	**1.80E-09**
0.90 (0.89, 0.92)	**1.11E-40**	0.85 (0.81, 0.89)	**1.86E-10**
0.88 (0.87, 0.90)	**3.15E-51**	0.89 (0.85, 0.94)	**1.96E-05**
0.90 (0.89, 0.91)	**6.86E-41**	0.87 (0.83, 0.91)	**2.81E-08**
0.88 (0.87, 0.90)	**6.14E-55**	0.86 (0.82, 0.90)	**3.33E-09**
1.00 (0.98, 1.01)	8.40E-01	1.05 (1.01, 1.10)	**1.55E-02**
0.98 (0.97, 1.00)	**1.62E-02**	1.01 (0.96, 1.05)	7.97E-01
0.91 (0.90, 0.93)	**4.17E-31**	0.88 (0.84, 0.92)	**6.66E-07**
0.91 (0.90, 0.93)	**2.05E-33**	0.81 (0.78, 0.85)	**7.98E-20**
0.89 (0.87, 0.90)	**1.70E-49**	0.90 (0.86, 0.94)	**4.27E-05**
0.95 (0.93, 0.96)	**1.85E-13**	0.88 (0.84, 0.91)	**2.17E-09**
0.91 (0.89, 0.92)	**2.56E-39**	0.85 (0.82, 0.89)	**1.60E-13**
1.09 (1.07, 1.10)	**1.21E-27**	1.21 (1.16, 1.27)	**4.40E-17**
1.08 (1.06, 1.09)	**2.12E-25**	1.07 (1.03, 1.11)	**2.13E-03**
1.10 (1.08, 1.11)	**2.70E-67**	1.17 (1.15, 1.20)	**6.18E-42**
1.04 (1.03, 1.06)	**8.65E-08**	1.07 (1.03, 1.12)	**2.16E-03**
1.07 (1.05, 1.08)	**3.33E-23**	1.11 (1.07, 1.15)	**6.71E-08**
1.00 (0.99, 1.01)	9.42E-01	1.08 (1.03, 1.12)	**8.77E-04**
0.96 (0.95, 0.98)	**3.33E-07**	0.93 (0.89, 0.97)	**1.36E-03**
0.93 (0.92, 0.95)	**8.48E-18**	0.89 (0.85, 0.94)	**1.38E-05**
0.98 (0.97, 1.00)	**4.32E-02**	1.06 (1.02, 1.11)	**1.12E-02**
0.94 (0.93, 0.96)	**3.96E-13**	1.03 (0.99, 1.08)	2.09E-01
0.94 (0.93, 0.96)	**4.87E-13**	1.05 (1.01, 1.10)	**2.53E-02**
1.04 (1.02, 1.05)	**5.33E-09**	1.05 (1.02, 1.08)	**5.38E-04**
1.03 (1.02, 1.04)	**6.65E-05**	1.07 (1.02, 1.12)	**3.24E-03**
1.06 (1.06, 1.07)	**8.62E-76**	1.05 (1.03, 1.07)	**2.14E-06**
1.06 (1.05, 1.07)	**6.21E-36**	1.07 (1.05, 1.09)	**4.06E-10**
1.07 (1.06, 1.08)	**1.05E-34**	1.08 (1.04, 1.11)	**4.12E-06**
1.05 (1.04, 1.06)	**1.61E-35**	1.06 (1.05, 1.08)	**4.59E-15**
0.87 (0.86, 0.89)	**4.88E-69**	0.84 (0.81, 0.88)	**4.38E-13**
1.11 (1.10, 1.13)	**1.35E-44**	1.26 (1.21, 1.32)	**2.45E-25**
0.99 (0.97, 1.01)	2.68E-01	0.88 (0.83, 0.93)	**5.96E-05**
0.95 (0.94, 0.97)	**2.49E-10**	1.05 (1.01, 1.10)	**3.72E-02**
0.90 (0.88, 0.91)	**6.30E-46**	0.87 (0.83, 0.91)	**8.05E-09**
0.89 (0.87, 0.90)	**8.69E-57**	0.85 (0.81, 0.89)	**7.30E-11**
0.88 (0.87, 0.90)	**2.76E-60**	0.86 (0.83, 0.90)	**2.45E-09**
0.98 (0.96, 1.00)	**2.62E-02**	0.85 (0.80, 0.90)	**6.27E-08**
0.91 (0.90, 0.92)	**1.48E-32**	0.82 (0.78, 0.86)	**7.99E-16**
1.08 (1.07, 1.09)	**5.79E-57**	1.03 (0.99, 1.07)	1.51E-01
0.93 (0.92, 0.95)	**3.29E-19**	0.96 (0.92, 1.01)	1.04E-01
0.88 (0.87, 0.90)	**1.01E-57**	0.84 (0.81, 0.88)	**4.75E-12**
1.01 (0.99, 1.02)	3.77E-01	0.89 (0.84, 0.94)	**7.53E-05**

1.04 (1.03, 1.05)	**9.73E-12**	1.06 (1.03, 1.09)	**1.07E-05**
1.07 (1.06, 1.08)	**6.22E-51**	1.07 (1.04, 1.10)	**6.39E-07**
0.92 (0.90, 0.93)	**6.14E-27**	0.84 (0.80, 0.89)	**9.40E-11**
0.94 (0.93, 0.96)	**1.52E-13**	0.97 (0.93, 1.02)	3.12E-01
0.90 (0.89, 0.92)	**2.33E-41**	0.90 (0.86, 0.94)	**6.33E-06**
0.93 (0.91, 0.94)	**1.06E-19**	0.85 (0.81, 0.90)	**1.40E-09**
0.93 (0.92, 0.95)	**5.16E-18**	0.88 (0.84, 0.93)	**8.73E-07**
0.94 (0.93, 0.96)	**5.94E-14**	0.98 (0.94, 1.03)	4.84E-01
0.89 (0.87, 0.90)	**1.80E-55**	0.87 (0.83, 0.91)	**1.22E-08**
1.01 (0.99, 1.02)	5.29E-01	0.90 (0.86, 0.95)	**3.68E-04**
0.92 (0.90, 0.93)	**6.90E-30**	0.89 (0.85, 0.94)	**3.94E-06**
0.94 (0.93, 0.96)	**6.89E-14**	0.99 (0.95, 1.04)	6.93E-01
0.89 (0.87, 0.90)	**2.78E-54**	0.87 (0.83, 0.91)	**1.56E-08**
1.00 (0.98, 1.01)	8.39E-01	0.89 (0.84, 0.94)	**2.73E-05**
0.93 (0.92, 0.94)	**4.84E-21**	0.92 (0.88, 0.96)	**8.57E-04**
0.99 (0.97, 1.00)	1.22E-01	0.86 (0.81, 0.91)	**2.70E-07**
1.01 (0.99, 1.03)	2.74E-01	0.98 (0.93, 1.04)	5.95E-01

Cerebrovascular diseases mortality		Cancer mortality	
Adjusted HR (95% CI)	P value	Adjusted HR (95% CI)	P value
0.93 (0.86, 1.00)	7.87E-02	1.02 (1.00, 1.05)	**3.15E-02**
0.93 (0.86, 1.00)	6.82E-02	1.02 (1.00, 1.04)	**3.95E-02**
0.94 (0.87, 1.01)	1.12E-01	1.03 (1.00, 1.05)	**2.48E-02**
0.93 (0.87, 1.01)	1.05E-01	1.02 (1.00, 1.04)	5.50E-02
0.93 (0.87, 1.01)	1.07E-01	1.02 (1.00, 1.04)	7.80E-02
0.93 (0.87, 1.01)	1.08E-01	1.02 (1.00, 1.05)	**3.83E-02**
0.92 (0.86, 0.99)	5.57E-02	1.02 (1.00, 1.04)	**4.26E-02**
0.90 (0.83, 0.97)	**9.95E-03**	1.01 (0.99, 1.03)	5.57E-01
0.89 (0.83, 0.96)	**8.10E-03**	1.01 (0.99, 1.03)	6.34E-01
0.90 (0.83, 0.97)	**1.04E-02**	1.01 (0.99, 1.03)	5.36E-01
0.89 (0.82, 0.96)	**4.89E-03**	1.00 (0.97, 1.02)	7.45E-01
0.88 (0.82, 0.95)	**2.95E-03**	0.99 (0.97, 1.01)	2.67E-01
0.90 (0.83, 0.97)	**9.95E-03**	1.01 (0.98, 1.03)	6.77E-01
0.90 (0.83, 0.97)	**1.06E-02**	1.01 (0.99, 1.03)	4.66E-01
0.89 (0.83, 0.96)	**5.81E-03**	1.00 (0.98, 1.02)	8.63E-01
0.88 (0.82, 0.95)	**3.14E-03**	0.99 (0.97, 1.01)	5.04E-01
0.89 (0.83, 0.96)	**8.10E-03**	1.00 (0.98, 1.02)	9.60E-01
0.89 (0.83, 0.96)	**5.07E-03**	0.99 (0.97, 1.01)	4.35E-01
0.89 (0.83, 0.96)	**6.87E-03**	0.99 (0.97, 1.01)	2.62E-01
0.89 (0.82, 0.96)	**4.76E-03**	0.99 (0.97, 1.02)	6.67E-01
0.88 (0.81, 0.94)	**2.17E-03**	0.99 (0.97, 1.01)	3.65E-01
0.87 (0.81, 0.94)	**8.64E-04**	0.97 (0.95, 0.99)	**1.82E-03**
0.87 (0.81, 0.93)	**6.49E-04**	0.97 (0.95, 0.99)	**3.34E-03**
0.87 (0.81, 0.94)	**9.56E-04**	0.97 (0.95, 0.99)	**1.95E-03**
0.89 (0.83, 0.95)	**3.10E-03**	0.95 (0.93, 0.97)	**1.62E-05**
0.90 (0.84, 0.97)	**8.10E-03**	0.95 (0.93, 0.97)	**2.15E-06**
0.88 (0.82, 0.94)	**1.29E-03**	0.96 (0.94, 0.98)	**5.61E-04**
0.88 (0.82, 0.95)	**2.62E-03**	0.98 (0.96, 1.00)	1.36E-01
0.92 (0.86, 0.99)	**4.27E-02**	0.99 (0.97, 1.02)	6.77E-01
0.91 (0.85, 0.98)	**2.14E-02**	0.99 (0.97, 1.01)	4.37E-01
0.89 (0.83, 0.96)	**5.79E-03**	0.97 (0.95, 0.99)	**1.93E-02**
0.90 (0.84, 0.97)	**1.22E-02**	0.98 (0.96, 1.00)	5.03E-02
0.91 (0.85, 0.98)	**2.64E-02**	0.99 (0.97, 1.01)	2.57E-01
0.89 (0.83, 0.95)	**3.51E-03**	0.96 (0.94, 0.98)	**8.75E-04**
0.94 (0.88, 1.01)	1.58E-01	1.01 (0.99, 1.03)	3.17E-01
0.95 (0.88, 1.02)	2.00E-01	1.02 (0.99, 1.04)	1.92E-01
0.96 (0.89, 1.03)	2.87E-01	1.02 (1.00, 1.04)	9.25E-02
0.97 (0.91, 1.04)	5.02E-01	1.03 (1.01, 1.05)	**8.63E-03**
0.94 (0.87, 1.01)	1.12E-01	1.00 (0.98, 1.02)	9.23E-01
0.93 (0.87, 1.00)	7.87E-02	0.99 (0.97, 1.01)	5.09E-01
0.95 (0.89, 1.02)	2.69E-01	1.01 (0.99, 1.04)	2.17E-01
1.00 (0.93, 1.07)	9.63E-01	1.05 (1.02, 1.07)	**4.65E-05**

0.87 (0.81, 0.94)	**7.61E-04**	0.97 (0.95, 0.99)	**1.77E-03**
0.88 (0.82, 0.95)	**1.85E-03**	0.96 (0.94, 0.98)	**1.26E-04**
0.90 (0.84, 0.97)	**8.10E-03**	0.97 (0.95, 0.99)	**3.11E-03**
0.87 (0.81, 0.93)	**6.85E-04**	0.95 (0.93, 0.97)	**1.21E-06**
0.86 (0.80, 0.93)	**3.32E-04**	0.94 (0.92, 0.96)	**3.79E-07**
0.89 (0.83, 0.96)	**5.98E-03**	0.95 (0.93, 0.97)	**4.26E-05**
1.00 (0.93, 1.07)	9.94E-01	1.05 (1.03, 1.07)	**6.23E-06**
0.88 (0.82, 0.95)	**1.85E-03**	0.95 (0.93, 0.97)	**1.86E-06**
0.85 (0.79, 0.91)	**9.36E-05**	0.94 (0.92, 0.96)	**2.76E-09**
0.85 (0.79, 0.91)	**7.15E-05**	0.94 (0.92, 0.96)	**1.89E-09**
0.85 (0.79, 0.91)	**7.06E-05**	0.93 (0.91, 0.95)	**8.63E-11**
0.84 (0.79, 0.91)	**5.39E-05**	0.93 (0.91, 0.95)	**1.50E-10**
0.86 (0.80, 0.92)	**2.10E-04**	0.93 (0.91, 0.95)	**6.27E-11**
0.98 (0.91, 1.05)	6.30E-01	1.04 (1.02, 1.06)	**3.57E-04**
0.86 (0.80, 0.92)	**1.98E-04**	0.95 (0.93, 0.97)	**3.10E-05**
0.83 (0.78, 0.90)	**2.23E-05**	0.94 (0.92, 0.96)	**5.07E-09**
0.83 (0.77, 0.89)	**1.97E-05**	0.93 (0.91, 0.95)	**3.79E-10**
0.83 (0.77, 0.89)	**1.97E-05**	0.93 (0.91, 0.95)	**6.30E-10**
0.83 (0.77, 0.89)	**1.97E-05**	0.94 (0.92, 0.96)	**2.64E-08**
0.84 (0.78, 0.90)	**2.64E-05**	0.92 (0.90, 0.94)	**1.07E-13**
0.96 (0.89, 1.03)	3.00E-01	1.03 (1.01, 1.05)	**1.93E-02**
0.86 (0.80, 0.93)	**3.10E-04**	0.96 (0.94, 0.98)	**2.00E-04**
0.84 (0.79, 0.91)	**4.51E-05**	0.94 (0.92, 0.96)	**6.27E-08**
0.86 (0.80, 0.92)	**1.22E-04**	0.94 (0.92, 0.96)	**1.50E-07**
0.84 (0.78, 0.90)	**2.64E-05**	0.94 (0.92, 0.96)	**1.63E-09**
0.84 (0.78, 0.90)	**3.29E-05**	0.94 (0.92, 0.96)	**1.43E-07**
0.85 (0.79, 0.91)	**7.15E-05**	0.92 (0.90, 0.94)	**1.64E-13**
0.94 (0.88, 1.01)	1.63E-01	1.02 (1.00, 1.04)	6.09E-02
1.10 (1.02, 1.18)	**2.12E-02**	1.01 (0.99, 1.04)	3.39E-01
1.12 (1.04, 1.20)	**6.98E-03**	1.02 (0.99, 1.04)	1.68E-01
1.13 (1.05, 1.21)	**3.26E-03**	1.03 (1.00, 1.05)	**4.90E-02**
1.10 (1.02, 1.18)	**2.00E-02**	1.00 (0.98, 1.03)	7.47E-01
1.10 (1.02, 1.18)	**2.27E-02**	1.00 (0.97, 1.02)	8.63E-01
1.10 (1.03, 1.18)	**1.57E-02**	1.03 (1.01, 1.05)	**1.88E-02**
1.02 (0.95, 1.09)	6.31E-01	1.04 (1.02, 1.07)	**4.65E-05**
1.09 (1.01, 1.18)	**4.89E-02**	0.99 (0.97, 1.02)	5.99E-01
1.09 (1.01, 1.18)	**4.56E-02**	1.00 (0.97, 1.02)	8.34E-01
1.08 (1.00, 1.17)	6.44E-02	1.00 (0.98, 1.03)	9.33E-01
1.09 (1.01, 1.18)	**4.04E-02**	0.99 (0.96, 1.01)	3.65E-01
1.09 (1.01, 1.18)	**3.92E-02**	0.98 (0.96, 1.01)	2.47E-01
1.09 (1.01, 1.18)	**4.76E-02**	1.00 (0.98, 1.02)	9.91E-01
1.05 (0.98, 1.13)	2.24E-01	1.06 (1.04, 1.08)	**6.40E-07**
0.97 (0.89, 1.04)	4.54E-01	0.97 (0.95, 0.99)	**7.01E-03**
0.96 (0.89, 1.04)	4.15E-01	0.97 (0.95, 1.00)	**3.08E-02**
0.96 (0.89, 1.04)	3.65E-01	0.98 (0.96, 1.00)	1.51E-01
0.97 (0.90, 1.05)	5.08E-01	0.96 (0.94, 0.98)	**4.64E-04**
0.97 (0.90, 1.05)	5.08E-01	0.96 (0.93, 0.98)	**2.12E-04**
0.97 (0.90, 1.05)	5.08E-01	0.97 (0.95, 0.99)	**9.99E-03**

0.97 (0.91, 1.05)	5.49E-01	1.04 (1.02, 1.07)	**7.06E-05**
0.83 (0.77, 0.89)	**1.97E-05**	0.92 (0.90, 0.94)	**3.49E-13**
0.85 (0.79, 0.92)	**1.22E-04**	0.95 (0.93, 0.97)	**4.62E-06**
0.87 (0.81, 0.93)	**6.45E-04**	0.96 (0.94, 0.98)	**8.73E-04**
0.84 (0.78, 0.90)	**2.64E-05**	0.92 (0.90, 0.94)	**3.62E-14**
0.83 (0.77, 0.89)	**1.97E-05**	0.91 (0.89, 0.93)	**1.83E-16**
0.87 (0.81, 0.94)	**9.99E-04**	0.95 (0.93, 0.97)	**4.62E-06**
0.94 (0.88, 1.01)	1.62E-01	1.03 (1.01, 1.05)	**5.14E-03**
1.11 (1.03, 1.20)	**1.89E-02**	1.03 (1.01, 1.05)	**2.56E-02**
1.10 (1.03, 1.18)	**8.33E-03**	1.00 (0.98, 1.02)	9.05E-01
1.07 (1.00, 1.15)	6.61E-02	1.00 (0.98, 1.02)	9.68E-01
1.13 (1.06, 1.20)	**5.77E-04**	1.00 (0.98, 1.03)	8.08E-01
0.89 (0.83, 0.95)	**2.61E-03**	0.99 (0.97, 1.01)	4.64E-01
1.11 (1.04, 1.20)	**6.98E-03**	0.97 (0.95, 1.00)	**3.84E-02**
1.10 (1.02, 1.18)	**2.27E-02**	0.97 (0.94, 0.99)	**7.94E-03**
1.15 (1.08, 1.23)	**2.70E-04**	1.01 (0.99, 1.03)	5.48E-01
0.90 (0.84, 0.97)	**1.06E-02**	1.01 (0.99, 1.04)	2.77E-01
0.97 (0.90, 1.04)	4.42E-01	1.06 (1.04, 1.09)	**4.71E-06**
1.11 (1.03, 1.19)	**1.03E-02**	1.01 (0.99, 1.03)	3.30E-01
1.12 (1.04, 1.20)	**4.91E-03**	1.00 (0.98, 1.02)	8.57E-01
1.05 (0.97, 1.13)	3.00E-01	1.03 (1.01, 1.06)	**5.93E-03**
0.94 (0.87, 1.01)	1.55E-01	0.96 (0.94, 0.98)	**1.30E-03**
0.99 (0.93, 1.06)	9.18E-01	0.98 (0.96, 1.00)	1.06E-01
1.03 (0.95, 1.10)	5.70E-01	0.96 (0.94, 0.98)	**8.94E-04**
1.03 (0.96, 1.11)	5.01E-01	0.96 (0.94, 0.98)	**3.21E-04**
1.01 (0.94, 1.08)	8.40E-01	0.97 (0.95, 0.99)	**1.53E-02**
0.98 (0.92, 1.05)	6.95E-01	1.03 (1.01, 1.06)	**2.69E-03**
0.93 (0.86, 1.00)	7.40E-02	0.93 (0.91, 0.95)	**1.91E-10**
0.97 (0.91, 1.05)	5.51E-01	0.96 (0.94, 0.98)	**5.70E-04**
1.00 (0.94, 1.08)	9.35E-01	0.99 (0.97, 1.01)	3.14E-01
0.94 (0.87, 1.01)	1.16E-01	0.93 (0.91, 0.95)	**4.47E-10**
1.04 (0.97, 1.12)	3.45E-01	1.05 (1.03, 1.07)	**1.07E-05**
1.14 (1.06, 1.22)	**1.24E-03**	1.10 (1.08, 1.13)	**4.27E-18**
0.94 (0.87, 1.00)	9.06E-02	0.94 (0.92, 0.96)	**1.49E-07**
0.93 (0.87, 1.00)	6.82E-02	0.94 (0.92, 0.96)	**3.60E-08**
0.98 (0.92, 1.05)	6.75E-01	0.98 (0.96, 0.99)	**1.96E-02**
1.05 (0.98, 1.12)	2.45E-01	1.04 (1.02, 1.07)	**7.47E-05**
1.17 (1.09, 1.25)	**8.85E-05**	1.08 (1.06, 1.10)	**8.93E-13**
0.87 (0.82, 0.93)	**9.00E-05**	0.91 (0.90, 0.93)	**3.00E-18**
0.84 (0.79, 0.89)	**4.99E-06**	0.90 (0.89, 0.92)	**2.28E-21**
1.00 (0.94, 1.07)	9.35E-01	0.97 (0.95, 0.99)	**1.14E-02**
1.12 (1.05, 1.19)	**9.99E-04**	1.08 (1.06, 1.10)	**5.06E-15**
1.04 (0.98, 1.11)	3.00E-01	1.01 (0.99, 1.03)	2.76E-01
0.92 (0.89, 0.95)	**1.36E-05**	0.94 (0.92, 0.95)	**2.82E-23**
0.92 (0.89, 0.94)	**2.01E-06**	0.94 (0.93, 0.95)	**3.48E-19**
0.95 (0.89, 1.01)	1.65E-01	0.95 (0.93, 0.97)	**6.70E-07**
1.11 (1.07, 1.16)	**2.58E-05**	1.08 (1.07, 1.10)	**9.35E-22**
1.04 (0.97, 1.11)	3.15E-01	0.98 (0.96, 1.00)	7.49E-02

0.92 (0.89, 0.95)	**2.64E-05**	0.94 (0.93, 0.95)	**1.90E-16**
0.89 (0.84, 0.93)	**5.80E-05**	0.98 (0.96, 1.00)	5.97E-02
1.00 (0.93, 1.07)	9.96E-01	0.94 (0.92, 0.96)	**1.20E-07**
1.10 (1.06, 1.14)	**2.64E-05**	1.08 (1.06, 1.10)	**1.54E-23**
1.11 (1.04, 1.18)	**5.48E-03**	1.03 (1.01, 1.05)	**6.30E-03**
0.90 (0.86, 0.94)	**4.51E-05**	0.92 (0.91, 0.94)	**1.59E-18**
0.90 (0.85, 0.96)	**3.51E-03**	0.97 (0.95, 0.99)	**2.30E-02**
0.96 (0.90, 1.03)	3.65E-01	0.94 (0.92, 0.96)	**6.61E-10**
1.06 (1.00, 1.13)	9.50E-02	1.07 (1.05, 1.09)	**9.04E-12**
1.14 (1.05, 1.24)	**4.81E-03**	1.05 (1.02, 1.07)	**1.26E-04**
0.89 (0.83, 0.96)	**6.06E-03**	0.93 (0.91, 0.95)	**1.90E-09**
0.95 (0.89, 1.01)	1.17E-01	0.93 (0.91, 0.94)	**2.05E-15**
0.91 (0.84, 0.98)	**3.45E-02**	1.00 (0.98, 1.02)	8.71E-01
0.95 (0.88, 1.02)	2.04E-01	1.01 (0.99, 1.03)	4.03E-01
0.94 (0.88, 1.01)	1.17E-01	1.04 (1.02, 1.06)	**1.12E-03**
1.04 (0.97, 1.11)	3.82E-01	0.96 (0.94, 0.98)	**2.96E-04**
1.04 (0.97, 1.12)	3.45E-01	0.95 (0.94, 0.97)	**7.44E-06**
1.01 (0.95, 1.08)	8.12E-01	1.01 (0.99, 1.03)	3.37E-01
1.00 (0.93, 1.07)	9.51E-01	1.03 (1.01, 1.05)	**2.48E-03**
1.01 (0.94, 1.08)	9.20E-01	1.08 (1.06, 1.11)	**1.25E-12**
0.99 (0.92, 1.06)	8.57E-01	0.93 (0.91, 0.95)	**1.48E-11**
1.00 (0.93, 1.07)	9.63E-01	0.93 (0.91, 0.95)	**7.10E-11**
0.96 (0.89, 1.04)	4.30E-01	0.95 (0.93, 0.97)	**1.52E-05**
1.01 (0.94, 1.09)	8.32E-01	1.07 (1.05, 1.09)	**7.74E-10**
1.09 (1.01, 1.17)	**3.92E-02**	1.09 (1.07, 1.11)	**9.62E-14**
0.94 (0.88, 1.01)	1.15E-01	0.91 (0.89, 0.93)	**3.07E-19**
0.92 (0.86, 0.99)	**4.27E-02**	0.91 (0.89, 0.93)	**3.07E-19**
1.06 (0.99, 1.14)	1.35E-01	1.00 (0.98, 1.02)	8.63E-01
1.02 (0.95, 1.09)	7.30E-01	1.07 (1.05, 1.10)	**5.20E-10**
0.85 (0.79, 0.92)	**1.09E-04**	0.99 (0.96, 1.01)	2.75E-01
1.04 (0.97, 1.12)	3.52E-01	0.96 (0.94, 0.98)	**1.20E-03**
1.13 (1.05, 1.22)	**3.51E-03**	1.02 (1.00, 1.05)	8.37E-02
0.87 (0.81, 0.93)	**5.08E-04**	0.94 (0.92, 0.96)	**1.40E-08**
0.89 (0.83, 0.96)	**5.79E-03**	0.98 (0.96, 1.00)	1.65E-01
0.84 (0.78, 0.90)	**3.27E-05**	0.93 (0.91, 0.95)	**1.17E-10**
0.87 (0.81, 0.94)	**8.43E-04**	0.96 (0.94, 0.98)	**1.09E-03**
0.86 (0.80, 0.93)	**4.73E-04**	0.93 (0.91, 0.95)	**2.45E-09**
0.87 (0.81, 0.94)	**7.65E-04**	0.95 (0.93, 0.97)	**1.40E-06**
0.91 (0.85, 0.98)	**3.09E-02**	1.01 (0.99, 1.03)	2.91E-01
0.90 (0.84, 0.97)	**1.32E-02**	1.00 (0.98, 1.03)	6.90E-01
0.97 (0.90, 1.04)	4.51E-01	1.03 (1.01, 1.06)	**2.12E-03**
0.98 (0.92, 1.05)	7.07E-01	1.05 (1.02, 1.07)	**4.61E-05**
0.90 (0.84, 0.98)	**1.85E-02**	0.97 (0.95, 0.99)	**1.36E-02**
0.94 (0.87, 1.01)	1.55E-01	1.03 (1.01, 1.05)	**9.97E-03**
0.90 (0.84, 0.97)	**1.57E-02**	0.96 (0.94, 0.99)	**1.97E-03**
0.93 (0.86, 1.00)	8.31E-02	0.97 (0.95, 0.99)	**7.87E-03**

0.91 (0.84, 0.98)	**1.91E-02**	0.96 (0.94, 0.99)	**1.95E-03**
0.96 (0.89, 1.04)	4.26E-01	0.96 (0.94, 0.99)	**4.30E-03**
0.87 (0.81, 0.93)	**6.49E-04**	0.96 (0.94, 0.98)	**6.83E-05**
0.92 (0.85, 0.99)	**3.59E-02**	0.98 (0.96, 1.00)	9.43E-02
0.90 (0.83, 0.97)	**8.58E-03**	0.99 (0.97, 1.01)	4.46E-01
0.93 (0.87, 1.00)	9.27E-02	0.88 (0.86, 0.90)	**4.12E-27**
0.91 (0.85, 0.98)	**3.32E-02**	0.90 (0.88, 0.92)	**6.15E-17**
0.89 (0.83, 0.96)	**6.68E-03**	0.95 (0.93, 0.97)	**1.40E-06**
0.88 (0.81, 0.95)	**3.01E-03**	0.92 (0.90, 0.94)	**2.29E-13**
0.88 (0.81, 0.94)	**1.59E-03**	0.94 (0.92, 0.97)	**7.50E-07**
0.86 (0.80, 0.93)	**4.43E-04**	0.93 (0.91, 0.95)	**3.69E-10**
0.94 (0.88, 1.01)	1.65E-01	1.02 (1.00, 1.04)	**3.61E-02**
0.91 (0.84, 0.97)	**1.52E-02**	1.01 (0.99, 1.03)	2.95E-01
0.99 (0.92, 1.06)	8.40E-01	0.91 (0.89, 0.93)	**7.05E-17**
1.00 (0.93, 1.07)	9.41E-01	0.93 (0.91, 0.95)	**1.09E-11**
0.93 (0.86, 1.00)	6.85E-02	0.90 (0.88, 0.92)	**9.78E-18**
1.03 (0.96, 1.11)	5.02E-01	0.94 (0.92, 0.96)	**7.33E-09**
1.00 (0.93, 1.07)	9.63E-01	0.90 (0.89, 0.92)	**1.09E-19**
1.04 (0.96, 1.11)	4.35E-01	1.09 (1.07, 1.11)	**1.59E-14**
0.96 (0.90, 1.03)	3.53E-01	1.08 (1.06, 1.10)	**1.65E-12**
1.07 (1.02, 1.13)	**2.27E-02**	1.06 (1.04, 1.07)	**2.02E-09**
0.99 (0.92, 1.06)	8.41E-01	1.03 (1.01, 1.05)	**1.88E-02**
1.07 (1.01, 1.13)	**3.60E-02**	1.04 (1.02, 1.07)	**6.14E-05**
1.02 (0.96, 1.10)	5.70E-01	0.99 (0.97, 1.01)	3.27E-01
1.14 (1.06, 1.22)	**1.23E-03**	0.96 (0.94, 0.98)	**2.73E-04**
0.99 (0.93, 1.07)	9.32E-01	0.97 (0.95, 0.99)	**6.82E-03**
0.97 (0.90, 1.04)	4.42E-01	1.00 (0.98, 1.02)	8.20E-01
0.89 (0.83, 0.96)	**8.10E-03**	0.98 (0.96, 1.01)	2.06E-01
0.89 (0.82, 0.96)	**5.63E-03**	0.99 (0.97, 1.01)	2.56E-01
1.02 (0.95, 1.09)	7.45E-01	1.03 (1.02, 1.05)	**2.87E-04**
1.01 (0.94, 1.08)	8.32E-01	1.02 (1.00, 1.04)	1.31E-01
1.07 (1.03, 1.10)	**3.10E-04**	1.04 (1.03, 1.05)	**3.63E-09**
1.07 (1.03, 1.11)	**2.62E-03**	1.05 (1.03, 1.06)	**4.70E-09**
1.08 (1.03, 1.13)	**2.46E-03**	1.05 (1.03, 1.07)	**2.59E-07**
1.07 (1.04, 1.10)	**2.64E-05**	1.03 (1.01, 1.04)	**9.98E-03**
0.92 (0.86, 0.99)	**3.57E-02**	0.93 (0.91, 0.95)	**1.19E-11**
1.08 (1.00, 1.16)	6.66E-02	1.10 (1.08, 1.13)	**6.70E-19**
1.10 (1.02, 1.18)	**2.64E-02**	0.98 (0.95, 1.00)	7.41E-02
0.90 (0.84, 0.97)	**1.37E-02**	0.99 (0.97, 1.01)	3.85E-01
0.85 (0.79, 0.92)	**1.15E-04**	0.95 (0.93, 0.97)	**5.66E-07**
0.85 (0.79, 0.91)	**4.76E-05**	0.93 (0.91, 0.95)	**2.49E-10**
0.84 (0.78, 0.90)	**2.64E-05**	0.93 (0.91, 0.95)	**6.30E-10**
1.00 (0.93, 1.08)	9.63E-01	0.95 (0.93, 0.98)	**1.31E-04**
0.99 (0.92, 1.07)	8.86E-01	0.90 (0.88, 0.92)	**2.72E-18**
1.08 (1.02, 1.13)	**8.10E-03**	1.07 (1.05, 1.09)	**1.90E-16**
0.89 (0.83, 0.96)	**4.65E-03**	0.99 (0.97, 1.01)	3.27E-01
0.85 (0.79, 0.91)	**8.85E-05**	0.93 (0.91, 0.95)	**4.12E-12**
1.01 (0.93, 1.09)	9.02E-01	0.98 (0.95, 1.00)	7.99E-02

0.99 (0.92, 1.07)	9.10E-01	1.02 (1.00, 1.04)	**2.64E-02**
1.08 (1.04, 1.12)	**5.76E-04**	1.04 (1.02, 1.06)	**4.92E-06**
0.89 (0.82, 0.96)	**6.90E-03**	0.93 (0.91, 0.96)	**9.59E-09**
0.90 (0.84, 0.97)	**1.37E-02**	0.99 (0.97, 1.02)	6.55E-01
0.87 (0.81, 0.93)	**6.45E-04**	0.95 (0.93, 0.97)	**4.54E-06**
0.91 (0.84, 0.98)	**2.48E-02**	0.94 (0.92, 0.96)	**1.26E-07**
0.89 (0.82, 0.96)	**4.65E-03**	0.96 (0.94, 0.98)	**1.30E-03**
0.90 (0.83, 0.96)	**7.77E-03**	1.00 (0.98, 1.02)	7.72E-01
0.84 (0.78, 0.91)	**3.74E-05**	0.93 (0.92, 0.95)	**1.11E-09**
0.99 (0.92, 1.07)	9.02E-01	0.98 (0.96, 1.01)	2.35E-01
0.87 (0.81, 0.93)	**5.07E-04**	0.96 (0.94, 0.98)	**2.99E-04**
0.89 (0.83, 0.96)	**6.10E-03**	1.00 (0.98, 1.02)	8.40E-01
0.84 (0.79, 0.91)	**4.51E-05**	0.94 (0.92, 0.96)	**2.90E-09**
1.00 (0.92, 1.08)	9.51E-01	0.97 (0.95, 1.00)	**4.90E-02**
0.90 (0.84, 0.97)	**8.10E-03**	0.98 (0.96, 1.00)	9.34E-02
1.00 (0.93, 1.09)	9.51E-01	0.96 (0.93, 0.98)	**6.86E-04**
0.99 (0.91, 1.07)	8.40E-01	1.04 (1.01, 1.06)	**6.58E-03**

Other mortality	
Adjusted HR (95% CI)	P value
0.94 (0.91, 0.96)	**2.50E-07**
0.94 (0.91, 0.96)	**2.46E-07**
0.94 (0.91, 0.96)	**8.05E-07**
0.93 (0.90, 0.95)	**3.94E-09**
0.92 (0.90, 0.94)	**4.61E-10**
0.93 (0.91, 0.96)	**7.28E-08**
0.94 (0.92, 0.96)	**8.77E-07**
0.88 (0.86, 0.90)	**2.69E-21**
0.88 (0.86, 0.90)	**9.68E-22**
0.88 (0.86, 0.91)	**5.53E-21**
0.86 (0.84, 0.88)	**2.96E-30**
0.84 (0.82, 0.87)	**2.65E-36**
0.88 (0.85, 0.90)	**6.19E-23**
0.89 (0.87, 0.91)	**2.27E-18**
0.86 (0.84, 0.88)	**4.58E-29**
0.85 (0.83, 0.87)	**7.69E-34**
0.86 (0.84, 0.89)	**1.14E-27**
0.84 (0.82, 0.87)	**2.94E-36**
0.84 (0.82, 0.86)	**8.57E-38**
0.85 (0.83, 0.87)	**5.77E-33**
0.85 (0.83, 0.87)	**3.07E-33**
0.83 (0.81, 0.85)	**3.66E-44**
0.83 (0.80, 0.85)	**6.62E-47**
0.84 (0.82, 0.86)	**1.02E-41**
0.85 (0.83, 0.88)	**7.23E-34**
0.87 (0.85, 0.89)	**3.26E-26**
0.84 (0.82, 0.86)	**3.35E-40**
0.84 (0.82, 0.86)	**7.15E-39**
0.87 (0.85, 0.89)	**2.57E-25**
0.87 (0.84, 0.89)	**1.23E-27**
0.85 (0.83, 0.87)	**9.14E-34**
0.86 (0.84, 0.89)	**3.10E-28**
0.87 (0.85, 0.90)	**4.60E-24**
0.85 (0.83, 0.87)	**4.41E-35**
0.90 (0.88, 0.92)	**1.14E-15**
0.96 (0.94, 0.99)	**4.54E-03**
0.97 (0.95, 1.00)	**2.26E-02**
0.99 (0.96, 1.01)	2.88E-01
0.96 (0.93, 0.98)	**1.10E-03**
0.96 (0.93, 0.98)	**5.16E-04**
0.97 (0.94, 0.99)	**8.66E-03**
0.99 (0.97, 1.02)	5.17E-01

0.90 (0.88, 0.92)	**1.48E-16**
0.91 (0.89, 0.93)	**6.07E-13**
0.92 (0.90, 0.95)	**4.41E-10**
0.90 (0.88, 0.92)	**1.13E-15**
0.90 (0.87, 0.92)	**4.90E-17**
0.92 (0.89, 0.94)	**1.38E-11**
1.02 (1.00, 1.05)	8.72E-02
0.86 (0.84, 0.88)	**1.99E-32**
0.85 (0.83, 0.87)	**1.18E-35**
0.85 (0.83, 0.87)	**2.49E-36**
0.84 (0.82, 0.87)	**1.15E-38**
0.84 (0.82, 0.86)	**2.22E-41**
0.86 (0.84, 0.88)	**5.09E-30**
1.01 (0.98, 1.03)	5.17E-01
0.84 (0.81, 0.86)	**5.60E-43**
0.82 (0.80, 0.84)	**2.78E-53**
0.82 (0.80, 0.84)	**6.01E-54**
0.81 (0.79, 0.83)	**1.29E-56**
0.81 (0.79, 0.83)	**1.30E-56**
0.83 (0.81, 0.85)	**2.05E-49**
0.97 (0.94, 0.99)	**1.18E-02**
0.85 (0.83, 0.87)	**1.00E-34**
0.83 (0.81, 0.86)	**9.89E-45**
0.85 (0.83, 0.88)	**6.94E-35**
0.82 (0.80, 0.84)	**2.53E-50**
0.82 (0.80, 0.85)	**3.70E-49**
0.84 (0.82, 0.86)	**3.03E-43**
0.94 (0.92, 0.97)	**3.64E-06**
1.17 (1.14, 1.20)	**7.77E-32**
1.19 (1.16, 1.22)	**3.79E-40**
1.20 (1.17, 1.24)	**2.17E-46**
1.17 (1.14, 1.20)	**1.18E-31**
1.16 (1.13, 1.19)	**1.30E-28**
1.18 (1.15, 1.21)	**1.68E-40**
1.04 (1.01, 1.06)	**3.94E-03**
1.16 (1.13, 1.19)	**3.03E-26**
1.17 (1.14, 1.20)	**3.56E-29**
1.17 (1.14, 1.20)	**3.59E-30**
1.16 (1.13, 1.19)	**2.58E-26**
1.15 (1.12, 1.18)	**6.87E-25**
1.17 (1.14, 1.20)	**5.36E-31**
1.09 (1.06, 1.11)	**1.25E-11**
1.02 (0.99, 1.05)	1.46E-01
1.02 (0.99, 1.05)	1.55E-01
1.02 (0.99, 1.05)	1.49E-01
1.02 (1.00, 1.05)	1.12E-01
1.02 (0.99, 1.05)	1.55E-01
1.03 (1.01, 1.06)	**2.36E-02**

0.99 (0.96, 1.01)	3.78E-01
0.83 (0.80, 0.85)	**1.16E-48**
0.86 (0.84, 0.89)	**6.51E-29**
0.89 (0.87, 0.91)	**7.00E-19**
0.83 (0.81, 0.86)	**2.73E-43**
0.82 (0.80, 0.85)	**2.05E-49**
0.89 (0.87, 0.91)	**3.05E-19**
0.93 (0.91, 0.96)	**8.31E-08**
1.02 (1.00, 1.05)	1.14E-01
1.04 (1.01, 1.06)	**1.03E-02**
1.03 (1.00, 1.06)	5.17E-02
1.04 (1.02, 1.07)	**1.73E-03**
0.96 (0.94, 0.99)	**5.57E-03**
1.06 (1.03, 1.09)	**3.35E-05**
1.05 (1.02, 1.08)	**1.21E-03**
1.09 (1.06, 1.12)	**4.20E-11**
0.95 (0.93, 0.98)	**2.31E-04**
0.95 (0.93, 0.98)	**1.04E-03**
1.06 (1.04, 1.09)	**1.29E-06**
1.08 (1.05, 1.10)	**1.54E-08**
1.02 (0.99, 1.05)	1.73E-01
0.97 (0.95, 1.00)	5.99E-02
1.00 (0.98, 1.03)	8.41E-01
1.02 (1.00, 1.05)	1.23E-01
1.02 (1.00, 1.05)	7.57E-02
1.01 (0.99, 1.04)	4.00E-01
0.98 (0.96, 1.01)	2.02E-01
0.94 (0.92, 0.97)	**4.34E-06**
0.97 (0.95, 1.00)	**3.85E-02**
1.00 (0.97, 1.02)	8.41E-01
0.94 (0.92, 0.97)	**1.34E-05**
1.04 (1.01, 1.06)	**4.16E-03**
1.13 (1.11, 1.16)	**1.70E-23**
0.96 (0.94, 0.98)	**1.03E-03**
0.96 (0.94, 0.98)	**1.04E-03**
0.97 (0.95, 0.99)	**1.04E-02**
1.02 (1.00, 1.04)	1.19E-01
1.12 (1.09, 1.14)	**3.55E-19**
0.90 (0.88, 0.92)	**3.40E-23**
0.88 (0.86, 0.90)	**9.46E-32**
0.98 (0.96, 1.01)	1.96E-01
1.10 (1.07, 1.12)	**6.53E-18**
1.03 (1.01, 1.06)	**3.41E-03**
0.92 (0.91, 0.93)	**4.53E-51**
0.91 (0.91, 0.92)	**1.74E-83**
0.98 (0.96, 1.00)	8.37E-02
1.11 (1.09, 1.13)	**2.12E-48**
1.05 (1.03, 1.07)	**4.08E-05**

0.91 (0.90, 0.92)	**4.69E-69**
0.87 (0.86, 0.89)	**3.27E-65**
0.99 (0.97, 1.02)	5.31E-01
1.11 (1.10, 1.12)	**1.51E-71**
1.14 (1.12, 1.17)	**2.22E-42**
0.88 (0.87, 0.90)	**4.81E-78**
0.87 (0.86, 0.89)	**7.06E-40**
0.95 (0.93, 0.97)	**8.23E-06**
1.07 (1.05, 1.09)	**6.71E-12**
1.19 (1.16, 1.23)	**2.37E-32**
0.85 (0.83, 0.87)	**2.80E-36**
0.92 (0.90, 0.93)	**2.02E-17**
0.89 (0.87, 0.92)	**1.53E-16**
0.95 (0.93, 0.97)	**4.67E-05**
0.97 (0.95, 0.99)	**1.58E-02**
1.04 (1.01, 1.06)	**1.80E-03**
1.02 (1.00, 1.05)	**4.70E-02**
1.08 (1.06, 1.11)	**9.88E-11**
0.96 (0.94, 0.99)	**2.81E-03**
1.01 (0.99, 1.04)	2.56E-01
1.00 (0.98, 1.03)	9.25E-01
1.00 (0.97, 1.02)	7.28E-01
1.03 (1.00, 1.06)	5.38E-02
0.99 (0.96, 1.01)	3.44E-01
1.19 (1.16, 1.22)	**2.19E-42**
0.90 (0.88, 0.92)	**9.84E-18**
0.88 (0.86, 0.90)	**1.74E-25**
1.09 (1.06, 1.11)	**2.16E-11**
1.00 (0.97, 1.02)	9.44E-01
0.83 (0.81, 0.85)	**3.66E-44**
1.00 (0.98, 1.03)	8.44E-01
1.23 (1.20, 1.26)	**3.42E-53**
0.88 (0.85, 0.90)	**1.61E-24**
0.87 (0.84, 0.89)	**8.45E-28**
0.83 (0.81, 0.85)	**2.44E-45**
0.87 (0.85, 0.90)	**9.95E-26**
0.88 (0.85, 0.90)	**8.62E-24**
0.87 (0.85, 0.90)	**1.69E-25**
0.90 (0.88, 0.93)	**5.46E-15**
0.88 (0.86, 0.91)	**1.00E-20**
0.99 (0.97, 1.01)	3.85E-01
0.99 (0.97, 1.02)	6.32E-01
0.94 (0.92, 0.97)	**7.38E-06**
0.91 (0.89, 0.94)	**5.10E-12**
0.94 (0.91, 0.96)	**3.27E-06**
0.96 (0.94, 0.99)	**6.13E-03**

0.93 (0.91, 0.96)	**6.96E-07**
1.02 (0.99, 1.05)	1.99E-01
0.86 (0.84, 0.88)	**6.98E-33**
0.87 (0.85, 0.90)	**2.42E-24**
0.91 (0.89, 0.93)	**3.37E-13**
0.86 (0.84, 0.88)	**3.39E-32**
0.86 (0.84, 0.88)	**8.60E-26**
0.86 (0.84, 0.88)	**6.84E-31**
0.83 (0.81, 0.86)	**4.22E-37**
0.86 (0.83, 0.88)	**8.72E-32**
0.83 (0.81, 0.86)	**1.02E-41**
0.96 (0.93, 0.98)	**5.83E-04**
0.95 (0.92, 0.97)	**1.66E-05**
0.91 (0.89, 0.94)	**4.20E-11**
0.92 (0.90, 0.94)	**2.01E-11**
0.85 (0.83, 0.87)	**1.72E-30**
0.97 (0.95, 0.99)	**2.00E-02**
0.92 (0.90, 0.94)	**7.38E-12**
1.05 (1.03, 1.08)	**6.28E-05**
1.09 (1.07, 1.12)	**2.62E-14**
1.11 (1.09, 1.12)	**9.33E-35**
1.06 (1.03, 1.08)	**7.48E-06**
1.08 (1.06, 1.10)	**3.52E-16**
0.99 (0.96, 1.01)	3.29E-01
0.96 (0.93, 0.98)	**5.07E-04**
0.89 (0.86, 0.91)	**5.79E-18**
0.94 (0.91, 0.96)	**1.82E-06**
0.87 (0.85, 0.89)	**5.26E-24**
0.87 (0.84, 0.89)	**8.68E-27**
1.04 (1.02, 1.06)	**7.99E-04**
1.04 (1.01, 1.06)	**2.72E-03**
1.08 (1.08, 1.09)	**7.51E-80**
1.06 (1.05, 1.08)	**3.84E-19**
1.09 (1.07, 1.10)	**1.27E-26**
1.05 (1.04, 1.07)	**9.64E-23**
0.81 (0.79, 0.83)	**9.69E-60**
1.08 (1.06, 1.11)	**1.50E-10**
1.02 (0.99, 1.05)	1.56E-01
0.88 (0.85, 0.90)	**6.72E-22**
0.85 (0.83, 0.87)	**2.56E-36**
0.84 (0.82, 0.86)	**3.99E-40**
0.83 (0.81, 0.85)	**1.44E-47**
1.05 (1.02, 1.08)	**3.07E-04**
0.94 (0.91, 0.96)	**8.40E-07**
1.10 (1.09, 1.12)	**8.37E-51**
0.86 (0.84, 0.88)	**1.78E-29**
0.85 (0.83, 0.87)	**1.08E-36**
1.08 (1.05, 1.11)	**2.07E-08**

1.05 (1.04, 1.07)	**1.44E-10**
1.09 (1.08, 1.10)	**1.79E-55**
0.92 (0.89, 0.94)	**5.27E-10**
0.88 (0.86, 0.90)	**7.81E-23**
0.85 (0.83, 0.87)	**6.33E-37**
0.94 (0.92, 0.97)	**9.39E-06**
0.92 (0.90, 0.94)	**1.50E-10**
0.87 (0.85, 0.90)	**4.53E-25**
0.84 (0.82, 0.86)	**2.24E-42**
1.07 (1.04, 1.09)	**6.57E-06**
0.88 (0.85, 0.90)	**1.08E-24**
0.87 (0.85, 0.89)	**4.11E-26**
0.84 (0.82, 0.86)	**4.09E-42**
1.06 (1.03, 1.09)	**1.83E-05**
0.87 (0.85, 0.90)	**3.54E-25**
1.06 (1.03, 1.09)	**4.24E-05**
0.99 (0.96, 1.01)	3.23E-01

Supplementary Table 14. Association of all metabolites with sleep duration and al

Metabolites	Group	CKB	
		Sleep duration	
		Adjusted β (95% CI)	P value
Concentration of chylomicrons ar	Lipoprotein subclasses	0.01 (-0.02, 0.04)	7.77E-01
Total lipids in chylomicrons and	Lipoprotein subclasses	0.01 (-0.02, 0.04)	7.76E-01
Phospholipids in chylomicrons ar	Lipoprotein subclasses	0.01 (-0.03, 0.04)	8.53E-01
Cholesterol in chylomicrons and	Lipoprotein subclasses	0.02 (-0.01, 0.05)	4.48E-01
Cholesteryl esters in chylomicron	Lipoprotein subclasses	0.03 (0.00, 0.06)	1.86E-01
Free cholesterol in chylomicrons	Lipoprotein subclasses	0.00 (-0.03, 0.04)	8.71E-01
Triglycerides in chylomicrons and	Lipoprotein subclasses	0.01 (-0.03, 0.04)	8.19E-01
Concentration of very large VLD	Lipoprotein subclasses	0.00 (-0.03, 0.04)	8.53E-01
Total lipids in very large VLDL	Lipoprotein subclasses	0.01 (-0.03, 0.04)	8.47E-01
Phospholipids in very large VLDI	Lipoprotein subclasses	0.01 (-0.02, 0.04)	7.77E-01
Cholesterol in very large VLDL	Lipoprotein subclasses	0.01 (-0.02, 0.05)	5.86E-01
Cholesteryl esters in very large V	Lipoprotein subclasses	0.02 (-0.01, 0.05)	4.62E-01
Free cholesterol in very large VL	Lipoprotein subclasses	0.01 (-0.02, 0.04)	7.40E-01
Triglycerides in very large VLDL	Lipoprotein subclasses	0.00 (-0.03, 0.03)	9.16E-01
Concentration of large VLDL par	Lipoprotein subclasses	0.00 (-0.03, 0.04)	8.53E-01
Total lipids in large VLDL	Lipoprotein subclasses	0.01 (-0.03, 0.04)	8.53E-01
Phospholipids in large VLDL	Lipoprotein subclasses	0.01 (-0.03, 0.04)	8.47E-01
Cholesterol in large VLDL	Lipoprotein subclasses	0.01 (-0.02, 0.04)	6.57E-01
Cholesteryl esters in large VLDL	Lipoprotein subclasses	0.02 (-0.01, 0.05)	3.96E-01
Free cholesterol in large VLDL	Lipoprotein subclasses	0.00 (-0.03, 0.04)	8.62E-01
Triglycerides in large VLDL	Lipoprotein subclasses	0.00 (-0.03, 0.03)	9.01E-01
Concentration of medium VLDL	Lipoprotein subclasses	0.01 (-0.02, 0.04)	6.57E-01
Total lipids in medium VLDL	Lipoprotein subclasses	0.01 (-0.02, 0.04)	6.34E-01
Phospholipids in medium VLDL	Lipoprotein subclasses	0.01 (-0.02, 0.05)	5.96E-01
Cholesterol in medium VLDL	Lipoprotein subclasses	0.02 (-0.01, 0.06)	2.80E-01
Cholesteryl esters in medium VLI	Lipoprotein subclasses	0.04 (0.00, 0.07)	1.05E-01
Free cholesterol in medium VLDI	Lipoprotein subclasses	0.01 (-0.02, 0.04)	7.10E-01
Triglycerides in medium VLDL	Lipoprotein subclasses	0.01 (-0.02, 0.04)	8.09E-01
Concentration of small VLDL pa	Lipoprotein subclasses	0.02 (-0.01, 0.05)	3.98E-01
Total lipids in small VLDL	Lipoprotein subclasses	0.02 (-0.01, 0.05)	3.67E-01
Phospholipids in small VLDL	Lipoprotein subclasses	0.01 (-0.02, 0.05)	5.82E-01
Cholesterol in small VLDL	Lipoprotein subclasses	0.04 (0.01, 0.07)	8.81E-02
Cholesteryl esters in small VLDL	Lipoprotein subclasses	0.05 (0.02, 0.08)	**4.43E-02**
Free cholesterol in small VLDL	Lipoprotein subclasses	0.02 (-0.02, 0.05)	5.45E-01
Triglycerides in small VLDL	Lipoprotein subclasses	0.01 (-0.02, 0.04)	6.82E-01
Concentration of very small VLD	Lipoprotein subclasses	0.02 (-0.01, 0.06)	2.70E-01
Total lipids in very small VLDL	Lipoprotein subclasses	0.03 (-0.01, 0.06)	2.41E-01
Phospholipids in very small VLD	Lipoprotein subclasses	0.02 (-0.01, 0.06)	3.06E-01
Cholesterol in very small VLDL	Lipoprotein subclasses	0.03 (0.00, 0.06)	1.45E-01
Cholesteryl esters in very small V	Lipoprotein subclasses	0.04 (0.00, 0.07)	1.05E-01
Free cholesterol in very small VL	Lipoprotein subclasses	0.02 (-0.01, 0.05)	3.83E-01
Triglycerides in very small VLDI	Lipoprotein subclasses	0.00 (-0.03, 0.04)	8.69E-01

Concentration of IDL particles	Lipoprotein subclasses	0.03 (0.00, 0.06)	1.65E-01
Total lipids in IDL	Lipoprotein subclasses	0.03 (0.00, 0.06)	1.54E-01
Phospholipids in IDL	Lipoprotein subclasses	0.03 (0.00, 0.07)	1.46E-01
Cholesterol in IDL	Lipoprotein subclasses	0.03 (0.00, 0.06)	1.50E-01
Cholesteryl esters in IDL	Lipoprotein subclasses	0.03 (0.00, 0.07)	1.33E-01
Free cholesterol in IDL	Lipoprotein subclasses	0.03 (-0.01, 0.06)	2.60E-01
Triglycerides in IDL	Lipoprotein subclasses	0.00 (-0.04, 0.03)	8.60E-01
Concentration of large LDL parti	Lipoprotein subclasses	0.03 (0.00, 0.07)	1.39E-01
Total lipids in large LDL	Lipoprotein subclasses	0.03 (0.00, 0.07)	1.33E-01
Phospholipids in large LDL	Lipoprotein subclasses	0.03 (0.00, 0.07)	1.29E-01
Cholesterol in large LDL	Lipoprotein subclasses	0.04 (0.00, 0.07)	1.19E-01
Cholesteryl esters in large LDL	Lipoprotein subclasses	0.04 (0.00, 0.07)	1.05E-01
Free cholesterol in large LDL	Lipoprotein subclasses	0.03 (0.00, 0.06)	1.66E-01
Triglycerides in large LDL	Lipoprotein subclasses	0.00 (-0.04, 0.03)	8.81E-01
Concentration of medium LDL pa	Lipoprotein subclasses	0.03 (0.00, 0.07)	1.29E-01
Total lipids in medium LDL	Lipoprotein subclasses	0.03 (0.00, 0.07)	1.29E-01
Phospholipids in medium LDL	Lipoprotein subclasses	0.03 (0.00, 0.07)	1.24E-01
Cholesterol in medium LDL	Lipoprotein subclasses	0.03 (0.00, 0.07)	1.23E-01
Cholesteryl esters in medium LDL	Lipoprotein subclasses	0.04 (0.00, 0.07)	1.19E-01
Free cholesterol in medium LDL	Lipoprotein subclasses	0.03 (0.00, 0.06)	1.76E-01
Triglycerides in medium LDL	Lipoprotein subclasses	0.00 (-0.03, 0.03)	9.76E-01
Concentration of small LDL parti	Lipoprotein subclasses	0.03 (0.00, 0.06)	1.86E-01
Total lipids in small LDL	Lipoprotein subclasses	0.03 (0.00, 0.06)	1.90E-01
Phospholipids in small LDL	Lipoprotein subclasses	0.02 (-0.01, 0.05)	3.71E-01
Cholesterol in small LDL	Lipoprotein subclasses	0.03 (0.00, 0.06)	1.73E-01
Cholesteryl esters in small LDL	Lipoprotein subclasses	0.03 (0.00, 0.06)	1.86E-01
Free cholesterol in small LDL	Lipoprotein subclasses	0.02 (-0.01, 0.05)	3.83E-01
Triglycerides in small LDL	Lipoprotein subclasses	0.01 (-0.02, 0.04)	6.34E-01
Concentration of very large HDL	Lipoprotein subclasses	-0.04 (-0.07, -0.01)	8.91E-02
Total lipids in very large HDL	Lipoprotein subclasses	-0.04 (-0.07, -0.01)	8.63E-02
Phospholipids in very large HDL	Lipoprotein subclasses	-0.04 (-0.07, -0.01)	8.95E-02
Cholesterol in very large HDL	Lipoprotein subclasses	-0.04 (-0.07, -0.01)	8.63E-02
Cholesteryl esters in very large H	Lipoprotein subclasses	-0.04 (-0.07, 0.00)	1.03E-01
Free cholesterol in very large HD	Lipoprotein subclasses	-0.04 (-0.07, -0.01)	6.10E-02
Triglycerides in very large HDL	Lipoprotein subclasses	0.00 (-0.03, 0.04)	8.54E-01
Concentration of large HDL parti	Lipoprotein subclasses	-0.05 (-0.08, -0.01)	**4.43E-02**
Total lipids in large HDL	Lipoprotein subclasses	-0.05 (-0.08, -0.01)	**4.43E-02**
Phospholipids in large HDL	Lipoprotein subclasses	-0.05 (-0.08, -0.01)	**4.43E-02**
Cholesterol in large HDL	Lipoprotein subclasses	-0.05 (-0.08, -0.02)	**4.43E-02**
Cholesteryl esters in large HDL	Lipoprotein subclasses	-0.05 (-0.08, -0.02)	**4.43E-02**
Free cholesterol in large HDL	Lipoprotein subclasses	-0.05 (-0.08, -0.01)	**4.43E-02**
Triglycerides in large HDL	Lipoprotein subclasses	0.00 (-0.04, 0.03)	8.81E-01
**Concentration of medium HDL**	Lipoprotein subclasses	-0.05 (-0.08, -0.01)	**4.43E-02**
**Total lipids in medium HDL**	Lipoprotein subclasses	-0.05 (-0.08, -0.02)	**4.43E-02**
**Phospholipids in medium HDL**	Lipoprotein subclasses	-0.05 (-0.08, -0.01)	**4.43E-02**
**Cholesterol in medium HDL**	Lipoprotein subclasses	-0.05 (-0.08, -0.02)	**4.43E-02**
**Cholesteryl esters in medium H**	Lipoprotein subclasses	-0.05 (-0.08, -0.02)	**4.43E-02**
**Free cholesterol in medium HD**	Lipoprotein subclasses	-0.05 (-0.08, -0.02)	**4.43E-02**

Triglycerides in medium HDL	Lipoprotein subclasses	0.02 (-0.01, 0.05)	4.12E-01
Concentration of small HDL parti	Lipoprotein subclasses	-0.02 (-0.06, 0.01)	2.89E-01
Total lipids in small HDL	Lipoprotein subclasses	-0.02 (-0.06, 0.01)	2.63E-01
Phospholipids in small HDL	Lipoprotein subclasses	-0.03 (-0.06, 0.00)	1.34E-01
Cholesterol in small HDL	Lipoprotein subclasses	0.00 (-0.04, 0.03)	8.69E-01
Cholesteryl esters in small HDL	Lipoprotein subclasses	0.01 (-0.03, 0.04)	8.53E-01
**Free cholesterol in small HDL**	Lipoprotein subclasses	-0.04 (-0.08, -0.01)	5.51E-02
Triglycerides in small HDL	Lipoprotein subclasses	0.01 (-0.02, 0.04)	6.66E-01
Phospholipids to total lipids ratio	Relative lipoprotein li	-0.01 (-0.04, 0.02)	7.77E-01
Cholesterol to total lipids ratio in	Relative lipoprotein li	0.03 (0.00, 0.07)	1.33E-01
Cholesteryl esters to total lipids ra	Relative lipoprotein li	0.03 (0.00, 0.07)	1.20E-01
Free cholesterol to total lipids rati	Relative lipoprotein li	0.01 (-0.03, 0.04)	7.93E-01
Triglycerides to total lipids ratio i	Relative lipoprotein li	-0.03 (-0.06, 0.01)	2.41E-01
Phospholipids to total lipids ratio	Relative lipoprotein li	0.03 (0.00, 0.06)	1.54E-01
**Cholesterol to total lipids ratio**	Relative lipoprotein li	0.07 (0.03, 0.10)	**6.74E-03**
Cholesteryl esters to total lipids ra	Relative lipoprotein li	0.04 (0.01, 0.08)	6.39E-02
**Free cholesterol to total lipids r**	Relative lipoprotein li	0.05 (0.02, 0.08)	**4.43E-02**
**Triglycerides to total lipids rati**	Relative lipoprotein li	-0.07 (-0.10, -0.03)	**6.74E-03**
Phospholipids to total lipids ratio	Relative lipoprotein li	0.02 (-0.01, 0.05)	3.83E-01
Cholesterol to total lipids ratio in	Relative lipoprotein li	0.04 (0.01, 0.08)	6.07E-02
Cholesteryl esters to total lipids ra	Relative lipoprotein li	0.04 (0.01, 0.08)	6.43E-02
Free cholesterol to total lipids rati	Relative lipoprotein li	0.01 (-0.02, 0.05)	5.93E-01
Triglycerides to total lipids ratio i	Relative lipoprotein li	-0.04 (-0.07, -0.01)	8.02E-02
Phospholipids to total lipids ratio	Relative lipoprotein li	0.00 (-0.03, 0.04)	8.54E-01
**Cholesterol to total lipids ratio**	Relative lipoprotein li	0.05 (0.02, 0.08)	**4.43E-02**
**Cholesteryl esters to total lipids**	Relative lipoprotein li	0.05 (0.02, 0.08)	**4.43E-02**
Free cholesterol to total lipids rati	Relative lipoprotein li	0.00 (-0.03, 0.03)	9.47E-01
Triglycerides to total lipids ratio i	Relative lipoprotein li	-0.05 (-0.08, -0.01)	**4.83E-02**
**Phospholipids to total lipids rat**	Relative lipoprotein li	-0.06 (-0.10, -0.03)	**6.74E-03**
Cholesterol to total lipids ratio in	Relative lipoprotein li	0.03 (0.00, 0.06)	1.53E-01
Cholesteryl esters to total lipids ra	Relative lipoprotein li	0.04 (0.01, 0.07)	7.76E-02
**Free cholesterol to total lipids r**	Relative lipoprotein li	-0.05 (-0.08, -0.01)	**4.43E-02**
Triglycerides to total lipids ratio i	Relative lipoprotein li	-0.02 (-0.05, 0.02)	5.48E-01
Phospholipids to total lipids ratio	Relative lipoprotein li	0.01 (-0.03, 0.04)	8.47E-01
Cholesterol to total lipids ratio in	Relative lipoprotein li	0.03 (0.00, 0.06)	1.50E-01
Cholesteryl esters to total lipids ra	Relative lipoprotein li	0.04 (0.00, 0.07)	1.09E-01
Free cholesterol to total lipids rati	Relative lipoprotein li	0.01 (-0.03, 0.04)	8.53E-01
Triglycerides to total lipids ratio i	Relative lipoprotein li	-0.03 (-0.06, 0.00)	2.04E-01
Phospholipids to total lipids ratio	Relative lipoprotein li	0.01 (-0.02, 0.04)	6.82E-01
Cholesterol to total lipids ratio in	Relative lipoprotein li	0.04 (0.01, 0.07)	8.63E-02
Cholesteryl esters to total lipids ra	Relative lipoprotein li	0.03 (0.00, 0.07)	1.22E-01
Free cholesterol to total lipids rati	Relative lipoprotein li	0.02 (-0.02, 0.05)	5.19E-01
Triglycerides to total lipids ratio i	Relative lipoprotein li	-0.04 (-0.07, -0.01)	7.49E-02
Phospholipids to total lipids ratio	Relative lipoprotein li	-0.03 (-0.06, 0.00)	1.40E-01
Cholesterol to total lipids ratio in	Relative lipoprotein li	0.04 (0.01, 0.08)	5.51E-02
Cholesteryl esters to total lipids ra	Relative lipoprotein li	0.05 (0.02, 0.08)	**4.43E-02**
Free cholesterol to total lipids rati	Relative lipoprotein li	0.00 (-0.04, 0.03)	8.67E-01
Triglycerides to total lipids ratio i	Relative lipoprotein li	-0.04 (-0.08, -0.01)	5.75E-02

Phospholipids to total lipids ratio	Relative lipoprotein li	-0.03 (-0.06, 0.01)	2.04E-01
Cholesterol to total lipids ratio in	Relative lipoprotein li	0.03 (0.00, 0.07)	1.19E-01
Cholesteryl esters to total lipids ra	Relative lipoprotein li	0.04 (0.01, 0.07)	8.81E-02
Free cholesterol to total lipids rati	Relative lipoprotein li	-0.04 (-0.07, -0.01)	7.29E-02
Triglycerides to total lipids ratio i	Relative lipoprotein li	-0.04 (-0.08, -0.01)	6.07E-02
Phospholipids to total lipids ratio	Relative lipoprotein li	-0.03 (-0.07, 0.00)	1.29E-01
Cholesterol to total lipids ratio in	Relative lipoprotein li	0.03 (0.00, 0.06)	1.60E-01
Cholesteryl esters to total lipids ra	Relative lipoprotein li	0.03 (0.00, 0.07)	1.22E-01
Free cholesterol to total lipids rati	Relative lipoprotein li	-0.05 (-0.08, -0.01)	5.20E-02
Triglycerides to total lipids ratio i	Relative lipoprotein li	-0.02 (-0.05, 0.01)	3.96E-01
Phospholipids to total lipids ratio	Relative lipoprotein li	0.00 (-0.03, 0.03)	9.73E-01
Cholesterol to total lipids ratio in	Relative lipoprotein li	-0.01 (-0.04, 0.02)	6.80E-01
Cholesteryl esters to total lipids ra	Relative lipoprotein li	0.00 (-0.04, 0.03)	9.07E-01
Free cholesterol to total lipids rati	Relative lipoprotein li	-0.04 (-0.07, -0.01)	6.07E-02
Triglycerides to total lipids ratio i	Relative lipoprotein li	0.03 (-0.01, 0.06)	2.34E-01
Phospholipids to total lipids ratio	Relative lipoprotein li	0.03 (0.00, 0.06)	1.75E-01
Cholesterol to total lipids ratio in	Relative lipoprotein li	-0.05 (-0.08, -0.01)	**4.43E-02**
Cholesteryl esters to total lipids ra	Relative lipoprotein li	-0.05 (-0.09, -0.02)	**3.92E-02**
Free cholesterol to total lipids rati	Relative lipoprotein li	-0.03 (-0.06, 0.00)	1.94E-01
Triglycerides to total lipids ratio i	Relative lipoprotein li	0.04 (0.01, 0.07)	7.65E-02
Phospholipids to total lipids ratio	Relative lipoprotein li	0.01 (-0.02, 0.04)	6.82E-01
Cholesterol to total lipids ratio in	Relative lipoprotein li	-0.03 (-0.07, 0.00)	1.23E-01
Cholesteryl esters to total lipids ra	Relative lipoprotein li	-0.03 (-0.06, 0.01)	2.32E-01
Free cholesterol to total lipids rati	Relative lipoprotein li	-0.05 (-0.08, -0.01)	**4.57E-02**
Triglycerides to total lipids ratio i	Relative lipoprotein li	0.04 (0.01, 0.08)	5.51E-02
Phospholipids to total lipids ratio	Relative lipoprotein li	-0.02 (-0.06, 0.01)	2.60E-01
Cholesterol to total lipids ratio in	Relative lipoprotein li	0.02 (-0.02, 0.05)	5.50E-01
Cholesteryl esters to total lipids ra	Relative lipoprotein li	0.02 (-0.01, 0.05)	4.11E-01
Free cholesterol to total lipids rati	Relative lipoprotein li	-0.05 (-0.08, -0.01)	**4.57E-02**
Triglycerides to total lipids ratio i	Relative lipoprotein li	0.02 (-0.01, 0.05)	3.77E-01
Average diameter for VLDL parti	Lipoprotein particle si	0.01 (-0.02, 0.04)	7.70E-01
Average diameter for LDL particl	Lipoprotein particle si	0.02 (-0.01, 0.05)	3.68E-01
Average diameter for HDL partic	Lipoprotein particle si	-0.03 (-0.07, 0.00)	1.23E-01
Total cholesterol	Cholesterol	0.02 (-0.02, 0.05)	5.39E-01
VLDL cholesterol	Cholesterol	0.03 (0.00, 0.06)	1.45E-01
Remnant cholesterol (non-HDL, r	Cholesterol	0.04 (0.01, 0.07)	8.95E-02
LDL cholesterol	Cholesterol	0.03 (0.00, 0.07)	1.40E-01
**HDL cholesterol**	Cholesterol	-0.05 (-0.09, -0.02)	**3.92E-02**
**HDL2 cholesterol**	Cholesterol	-0.05 (-0.09, -0.02)	**3.92E-02**
HDL3 cholesterol	Cholesterol	-0.04 (-0.07, -0.01)	8.34E-02
Total esterified cholesterol	Cholesteryl esters	0.01 (-0.02, 0.05)	5.94E-01
Total free cholesterol	Free cholesterol	0.02 (-0.01, 0.05)	4.10E-01
Total triglycerides	Triglycerides	0.00 (-0.03, 0.04)	8.53E-01
Triglycerides in VLDL	Triglycerides	0.01 (-0.03, 0.04)	8.47E-01
Triglycerides in LDL	Triglycerides	-0.01 (-0.04, 0.02)	7.77E-01
Triglycerides in HDL	Triglycerides	0.01 (-0.02, 0.04)	7.02E-01
Phosphoglycerides	Other lipids	-0.01 (-0.04, 0.02)	7.40E-01
Ratio of triglycerides to phospho	Other lipids	0.02 (-0.01, 0.05)	4.62E-01

Phosphatidylcholines	Other lipids	-0.01 (-0.05, 0.02)	6.34E-01
Sphingomyelins	Other lipids	0.00 (-0.03, 0.03)	9.71E-01
Total cholines	Other lipids	-0.01 (-0.05, 0.02)	6.34E-01
Apolipoprotein A1	Apolipoproteins	-0.04 (-0.07, -0.01)	7.52E-02
Apolipoprotein B	Apolipoproteins	0.03 (0.00, 0.06)	1.53E-01
Ratio of apolipoprotein B to apol	Apolipoproteins	0.05 (0.02, 0.08)	**4.43E-02**
Total fatty acids	Fatty acids	0.00 (-0.03, 0.04)	8.54E-01
Degree of unsaturation	Fatty acids	0.02 (-0.01, 0.06)	3.05E-01
Docosahexaenoic acid	Fatty acids	0.02 (-0.01, 0.06)	2.99E-01
Linoleic acid	Fatty acids	0.01 (-0.02, 0.04)	7.29E-01
Omega-3 fatty acids	Fatty acids	0.02 (-0.01, 0.06)	2.80E-01
Omega-6 fatty acids	Fatty acids	0.01 (-0.02, 0.04)	7.40E-01
Polyunsaturated fatty acids	Fatty acids	0.01 (-0.02, 0.05)	6.34E-01
Monounsaturated fatty acids	Fatty acids	0.00 (-0.04, 0.03)	8.69E-01
Saturated fatty acids	Fatty acids	0.01 (-0.03, 0.04)	8.53E-01
Ratio of docosahexaenoic acid to	Fatty acids	0.02 (-0.01, 0.06)	2.60E-01
Ratio of linoleic acid to total fatty	Fatty acids	0.01 (-0.02, 0.04)	6.80E-01
Ratio of omega-3 fatty acids to to	Fatty acids	0.04 (0.01, 0.07)	8.32E-02
Ratio of omega-6 fatty acids to to	Fatty acids	0.01 (-0.02, 0.04)	7.77E-01
Ratio of polyunsaturated fatty aci	Fatty acids	0.02 (-0.01, 0.05)	3.83E-01
Ratio of monounsaturated fatty ac	Fatty acids	-0.02 (-0.05, 0.01)	3.49E-01
Ratio of saturated fatty acids to tc	Fatty acids	0.00 (-0.04, 0.03)	8.81E-01
Glucose	Glycolysis related met	0.01 (-0.03, 0.04)	8.47E-01
Lactate	Glycolysis related met	0.00 (-0.04, 0.03)	8.69E-01
Citrate	Glycolysis related met	-0.01 (-0.05, 0.02)	6.34E-01
Alanine	Amino acids	0.01 (-0.02, 0.04)	8.14E-01
Glutamine	Amino acids	0.00 (-0.03, 0.03)	9.95E-01
Histidine	Amino acids	0.03 (0.00, 0.06)	1.50E-01
Isoleucine	Amino acids	0.01 (-0.02, 0.04)	6.49E-01
Leucine	Amino acids	0.01 (-0.02, 0.04)	6.74E-01
Valine	Amino acids	0.01 (-0.03, 0.04)	8.41E-01
Phenylalanine	Amino acids	0.03 (0.00, 0.07)	1.19E-01
Tyrosine	Amino acids	0.02 (-0.02, 0.05)	5.15E-01
Acetate	Ketone bodies	0.00 (-0.03, 0.03)	9.92E-01
Acetoacetate	Ketone bodies	-0.03 (-0.06, 0.00)	1.61E-01
**3-Hydroxybutyrate**	Ketone bodies	-0.05 (-0.08, -0.01)	**4.43E-02**
Creatinine	Fluid balance	-0.01 (-0.04, 0.03)	8.51E-01
Albumin	Fluid balance	-0.01 (-0.05, 0.02)	5.82E-01
Glycoprotein acetyls	Inflammation	0.01 (-0.02, 0.05)	5.65E-01
Acetone	Ketone bodies		
Cholesteryl esters in HDL	Cholesteryl esters		
Cholesteryl esters in LDL	Cholesteryl esters		
Cholesteryl esters in VLDL/ (Chc	Cholesteryl esters		
Clinical LDL cholesterol	Cholesterol		
Concentration of HDL particles	Lipoprotein particle concentrations		
Concentration of LDL particles	Lipoprotein particle concentrations		
Concentration of VLDL particles	Lipoprotein particle concentrations		
Free cholesterol in HDL	Free cholesterol		

Free cholesterol in LDL	Free cholesterol
Free cholesterol in VLDL	Free cholesterol
Glycine	Amino acids
Phospholipids in HDL	Phospholipids
Phospholipids in LDL	Phospholipids
Phospholipids in VLDL	Phospholipids
Pyruvate	Glycolysis related metabolites
Ratio of omega-6 fatty acids to or	Fatty acids
Ratio of polyunsaturated fatty aci	Fatty acids
Total cholesterol minus HDL-C	Cholesterol
Total concentration of branched-c	Amino acids
Total concentration of lipoprotein	Lipoprotein particle concentrations
Total lipids in HDL	Total lipids
Total lipids in LDL	Total lipids
Total lipids in lipoprotein particle	Total lipids
Total lipids in VLDL	Total lipids
Total phospholipids in lipoprotein	Phospholipids

Models were adjusted for age, sex, regions (CKB only), ethnicity (UKB only), levels of education, smoking s

HR, hazard ratio; CI, confidence interval; LDL, Low Density Lipoprotein; HDL, High Density Lipoprotein;

l-cause and cause-specific mortality in the CKB and UKB after excluding particip

All causes mortality		Ischaemic heart diseases mortality	
**Adjusted HR (95% CI)**	**P value**	**Adjusted HR (95% CI)**	**P value**
0.94 (0.90, 0.99)	**3.13E-02**	0.99 (0.91, 1.07)	9.01E-01
0.94 (0.90, 0.99)	**3.99E-02**	0.99 (0.91, 1.07)	9.15E-01
0.95 (0.91, 1.00)	8.01E-02	1.00 (0.92, 1.09)	9.91E-01
0.98 (0.94, 1.03)	4.58E-01	1.02 (0.94, 1.11)	8.43E-01
0.99 (0.95, 1.04)	8.00E-01	1.03 (0.95, 1.12)	6.30E-01
0.96 (0.92, 1.01)	1.81E-01	1.00 (0.92, 1.08)	9.74E-01
0.93 (0.89, 0.98)	**1.37E-02**	0.98 (0.90, 1.07)	8.23E-01
0.96 (0.92, 1.01)	1.99E-01	0.99 (0.91, 1.08)	9.30E-01
0.97 (0.92, 1.01)	2.20E-01	0.99 (0.91, 1.08)	9.42E-01
0.97 (0.92, 1.02)	2.64E-01	1.00 (0.92, 1.09)	9.95E-01
0.98 (0.94, 1.03)	5.06E-01	1.01 (0.94, 1.10)	8.83E-01
0.98 (0.94, 1.03)	5.73E-01	1.02 (0.94, 1.10)	8.50E-01
0.98 (0.93, 1.02)	4.39E-01	1.01 (0.93, 1.09)	9.30E-01
0.96 (0.92, 1.01)	1.62E-01	0.98 (0.90, 1.07)	8.70E-01
0.96 (0.92, 1.01)	1.73E-01	1.01 (0.93, 1.10)	9.30E-01
0.96 (0.92, 1.01)	1.75E-01	1.01 (0.93, 1.10)	9.30E-01
0.96 (0.92, 1.01)	1.75E-01	1.01 (0.93, 1.09)	9.42E-01
0.96 (0.92, 1.01)	1.81E-01	1.00 (0.92, 1.09)	9.95E-01
0.96 (0.92, 1.00)	1.44E-01	0.99 (0.91, 1.08)	9.34E-01
0.97 (0.92, 1.01)	2.40E-01	1.01 (0.93, 1.10)	9.30E-01
0.96 (0.92, 1.01)	1.71E-01	1.01 (0.93, 1.10)	9.06E-01
0.96 (0.92, 1.01)	1.94E-01	1.03 (0.95, 1.12)	6.83E-01
0.97 (0.92, 1.01)	2.02E-01	1.03 (0.95, 1.12)	6.89E-01
0.96 (0.92, 1.01)	1.75E-01	1.02 (0.94, 1.11)	7.65E-01
0.97 (0.93, 1.02)	3.32E-01	1.02 (0.94, 1.11)	7.97E-01
0.97 (0.93, 1.02)	3.42E-01	1.01 (0.93, 1.10)	9.15E-01
0.97 (0.93, 1.02)	3.36E-01	1.03 (0.95, 1.12)	6.30E-01
0.96 (0.92, 1.01)	1.80E-01	1.03 (0.95, 1.12)	6.19E-01
0.98 (0.94, 1.02)	4.23E-01	1.05 (0.97, 1.14)	3.75E-01
0.98 (0.94, 1.03)	5.73E-01	1.07 (0.98, 1.16)	2.39E-01
0.97 (0.93, 1.01)	2.37E-01	1.04 (0.95, 1.12)	6.19E-01
1.04 (1.00, 1.09)	1.18E-01	1.17 (1.08, 1.27)	**2.98E-04**
1.06 (1.01, 1.11)	**2.08E-02**	1.20 (1.11, 1.30)	**2.60E-05**
1.01 (0.96, 1.05)	7.89E-01	1.10 (1.02, 1.19)	**4.35E-02**
0.96 (0.92, 1.00)	1.17E-01	1.00 (0.92, 1.09)	9.74E-01
1.05 (1.00, 1.09)	9.13E-02	1.13 (1.04, 1.22)	**8.53E-03**
1.05 (1.01, 1.10)	**3.96E-02**	1.14 (1.06, 1.23)	**2.30E-03**
1.01 (0.97, 1.06)	6.09E-01	1.04 (0.96, 1.13)	5.30E-01
1.10 (1.05, 1.14)	**6.10E-05**	1.24 (1.15, 1.33)	**2.10E-07**
1.10 (1.06, 1.15)	**2.00E-05**	1.25 (1.16, 1.35)	**3.43E-08**
1.08 (1.03, 1.13)	**1.31E-03**	1.19 (1.11, 1.29)	**2.76E-05**
0.99 (0.94, 1.03)	6.59E-01	1.01 (0.93, 1.09)	9.34E-01

1.01 (0.97, 1.06)	7.27E-01	1.02 (0.94, 1.11)	8.21E-01
1.01 (0.97, 1.06)	6.97E-01	1.03 (0.94, 1.11)	7.64E-01
0.99 (0.95, 1.04)	8.38E-01	1.00 (0.92, 1.09)	9.86E-01
1.01 (0.96, 1.05)	7.51E-01	1.04 (0.95, 1.12)	6.19E-01
1.00 (0.96, 1.05)	9.96E-01	1.03 (0.95, 1.12)	6.83E-01
1.03 (0.98, 1.07)	3.00E-01	1.04 (0.96, 1.13)	5.08E-01
1.03 (0.99, 1.08)	2.55E-01	1.00 (0.92, 1.08)	9.76E-01
0.99 (0.95, 1.04)	6.94E-01	1.01 (0.93, 1.10)	9.15E-01
0.99 (0.94, 1.03)	6.81E-01	1.01 (0.93, 1.10)	9.15E-01
0.97 (0.92, 1.01)	2.48E-01	0.99 (0.91, 1.07)	8.90E-01
0.99 (0.94, 1.03)	6.36E-01	1.02 (0.94, 1.11)	8.70E-01
0.98 (0.94, 1.03)	5.06E-01	1.02 (0.94, 1.11)	8.64E-01
1.00 (0.96, 1.05)	9.65E-01	1.01 (0.93, 1.10)	9.01E-01
1.06 (1.01, 1.10)	**2.81E-02**	1.02 (0.94, 1.11)	7.65E-01
0.96 (0.92, 1.01)	1.93E-01	1.00 (0.92, 1.09)	9.81E-01
0.96 (0.92, 1.01)	1.82E-01	1.00 (0.92, 1.09)	9.81E-01
0.92 (0.88, 0.96)	**8.80E-04**	0.92 (0.85, 1.00)	1.21E-01
0.97 (0.93, 1.02)	3.00E-01	1.02 (0.94, 1.11)	8.47E-01
0.98 (0.93, 1.02)	3.78E-01	1.03 (0.95, 1.12)	7.15E-01
0.95 (0.91, 1.00)	7.04E-02	0.97 (0.89, 1.06)	7.03E-01
1.04 (0.99, 1.08)	2.02E-01	0.99 (0.91, 1.08)	9.48E-01
0.96 (0.92, 1.01)	1.75E-01	1.02 (0.93, 1.11)	8.74E-01
0.96 (0.92, 1.01)	1.75E-01	1.02 (0.93, 1.11)	8.70E-01
0.91 (0.87, 0.96)	**3.58E-04**	0.93 (0.85, 1.01)	1.71E-01
0.98 (0.93, 1.02)	3.97E-01	1.04 (0.96, 1.13)	5.38E-01
0.98 (0.94, 1.03)	5.03E-01	1.05 (0.97, 1.14)	4.18E-01
0.95 (0.91, 1.00)	7.33E-02	1.00 (0.92, 1.09)	9.91E-01
1.00 (0.96, 1.05)	9.22E-01	1.03 (0.94, 1.11)	7.63E-01
1.18 (1.14, 1.23)	**3.90E-15**	1.22 (1.13, 1.31)	**1.92E-06**
1.18 (1.14, 1.23)	**4.79E-15**	1.22 (1.13, 1.31)	**1.44E-06**
1.17 (1.12, 1.22)	**8.78E-13**	1.13 (1.05, 1.22)	**4.74E-03**
1.19 (1.14, 1.23)	**4.79E-15**	1.30 (1.21, 1.40)	**5.09E-11**
1.19 (1.14, 1.24)	**1.83E-15**	1.34 (1.24, 1.44)	**2.96E-13**
1.17 (1.12, 1.22)	**2.79E-13**	1.20 (1.12, 1.30)	**6.56E-06**
1.14 (1.09, 1.19)	**1.13E-08**	1.18 (1.09, 1.27)	**7.95E-05**
1.13 (1.08, 1.18)	**1.85E-07**	1.07 (0.99, 1.16)	2.14E-01
1.13 (1.08, 1.18)	**1.71E-07**	1.07 (0.99, 1.16)	1.99E-01
1.12 (1.07, 1.17)	**1.48E-06**	1.07 (0.99, 1.16)	1.99E-01
1.13 (1.08, 1.18)	**7.85E-08**	1.07 (0.99, 1.16)	1.72E-01
1.13 (1.08, 1.18)	**1.83E-07**	1.06 (0.98, 1.15)	2.43E-01
1.14 (1.09, 1.19)	**5.72E-09**	1.10 (1.02, 1.19)	**3.69E-02**
1.12 (1.07, 1.17)	**1.68E-06**	1.00 (0.92, 1.09)	9.95E-01
0.89 (0.85, 0.94)	**7.48E-06**	0.82 (0.75, 0.89)	**1.73E-05**
0.90 (0.86, 0.94)	**1.39E-05**	0.82 (0.75, 0.89)	**2.90E-05**
0.92 (0.88, 0.97)	**1.54E-03**	0.85 (0.78, 0.93)	**8.12E-04**
0.90 (0.86, 0.94)	**8.19E-06**	0.83 (0.77, 0.90)	**6.47E-05**
0.89 (0.85, 0.93)	**1.21E-06**	0.83 (0.76, 0.90)	**2.90E-05**
0.93 (0.89, 0.97)	**4.47E-03**	0.86 (0.79, 0.94)	**1.83E-03**

0.88 (0.84, 0.93)	**1.20E-06**	0.79 (0.72, 0.86)	**1.26E-06**
0.82 (0.79, 0.86)	**1.29E-17**	0.77 (0.72, 0.83)	**8.50E-12**
0.83 (0.79, 0.86)	**2.66E-17**	0.77 (0.72, 0.83)	**2.97E-11**
0.85 (0.82, 0.89)	**5.93E-11**	0.81 (0.74, 0.88)	**2.62E-06**
0.89 (0.86, 0.94)	**3.90E-06**	0.85 (0.78, 0.92)	**2.06E-04**
0.92 (0.88, 0.96)	**5.05E-04**	0.88 (0.81, 0.95)	**4.42E-03**
0.87 (0.84, 0.91)	**2.86E-08**	0.83 (0.77, 0.90)	**4.92E-05**
0.91 (0.87, 0.95)	**2.07E-04**	0.85 (0.78, 0.92)	**6.15E-04**
0.95 (0.91, 0.99)	5.21E-02	1.01 (0.93, 1.10)	9.34E-01
1.16 (1.12, 1.20)	**1.04E-15**	1.14 (1.07, 1.21)	**3.67E-04**
1.16 (1.12, 1.20)	**1.04E-15**	1.14 (1.07, 1.21)	**1.52E-04**
1.07 (1.02, 1.12)	**1.02E-02**	1.03 (0.95, 1.13)	6.30E-01
0.88 (0.84, 0.91)	**3.07E-09**	0.88 (0.82, 0.95)	**1.94E-03**
0.96 (0.91, 1.00)	1.16E-01	1.02 (0.94, 1.11)	8.36E-01
1.10 (1.07, 1.14)	**2.88E-08**	1.11 (1.05, 1.18)	**8.79E-04**
1.12 (1.08, 1.16)	**1.73E-08**	1.12 (1.05, 1.20)	**3.32E-03**
1.03 (0.98, 1.08)	3.00E-01	1.06 (0.98, 1.14)	3.16E-01
0.93 (0.88, 0.97)	**3.38E-03**	0.88 (0.81, 0.96)	**9.33E-03**
0.96 (0.92, 1.00)	1.41E-01	0.92 (0.85, 1.00)	1.01E-01
0.98 (0.94, 1.03)	4.60E-01	0.91 (0.84, 0.98)	**3.35E-02**
1.01 (0.96, 1.06)	7.51E-01	0.89 (0.81, 0.96)	**1.57E-02**
0.97 (0.93, 1.01)	2.40E-01	0.98 (0.91, 1.07)	8.76E-01
1.03 (0.98, 1.08)	3.12E-01	1.11 (1.02, 1.20)	**2.94E-02**
0.95 (0.91, 0.99)	**4.64E-02**	0.80 (0.73, 0.88)	**6.56E-06**
1.05 (1.00, 1.10)	7.61E-02	0.91 (0.83, 0.99)	6.95E-02
1.04 (0.99, 1.09)	1.80E-01	0.89 (0.82, 0.97)	**2.54E-02**
1.03 (0.98, 1.08)	3.06E-01	1.06 (0.97, 1.15)	3.52E-01
0.96 (0.92, 1.01)	2.02E-01	1.13 (1.04, 1.23)	**1.43E-02**
0.93 (0.89, 0.97)	**5.51E-03**	0.80 (0.73, 0.87)	**4.89E-06**
1.12 (1.07, 1.17)	**1.35E-06**	1.22 (1.13, 1.33)	**9.48E-06**
1.11 (1.06, 1.16)	**1.71E-05**	1.21 (1.11, 1.31)	**4.22E-05**
1.16 (1.11, 1.21)	**1.35E-10**	1.23 (1.14, 1.33)	**7.58E-07**
0.91 (0.87, 0.95)	**4.66E-05**	0.86 (0.79, 0.93)	**1.19E-03**
0.93 (0.89, 0.96)	**7.89E-04**	0.83 (0.78, 0.89)	**1.92E-06**
1.16 (1.11, 1.21)	**2.94E-09**	1.43 (1.30, 1.57)	**6.59E-13**
1.14 (1.09, 1.19)	**3.74E-08**	1.37 (1.26, 1.50)	**1.08E-11**
1.11 (1.06, 1.16)	**6.81E-05**	1.26 (1.15, 1.38)	**1.01E-05**
0.93 (0.89, 0.97)	**4.35E-03**	0.86 (0.78, 0.93)	**1.94E-03**
0.93 (0.89, 0.97)	**2.35E-03**	0.87 (0.81, 0.95)	**2.57E-03**
1.00 (0.96, 1.04)	9.35E-01	1.08 (0.99, 1.17)	1.72E-01
0.96 (0.92, 1.00)	6.73E-02	1.03 (0.95, 1.12)	6.30E-01
1.06 (1.01, 1.11)	**3.52E-02**	1.07 (0.99, 1.17)	2.05E-01
1.02 (0.98, 1.07)	3.97E-01	0.98 (0.90, 1.06)	7.75E-01
0.97 (0.93, 1.02)	3.29E-01	0.96 (0.88, 1.05)	6.10E-01
0.97 (0.93, 1.01)	2.70E-01	0.99 (0.92, 1.08)	9.42E-01
0.95 (0.91, 0.99)	**3.40E-02**	0.98 (0.90, 1.06)	8.21E-01
1.07 (1.02, 1.12)	**1.27E-02**	1.04 (0.96, 1.13)	5.80E-01
1.06 (1.02, 1.11)	**6.64E-03**	1.04 (0.96, 1.12)	5.37E-01

0.97 (0.92, 1.01)	2.23E-01	0.91 (0.84, 1.00)	1.00E-01
1.00 (0.95, 1.04)	8.87E-01	1.06 (0.97, 1.15)	3.75E-01
0.99 (0.95, 1.03)	6.94E-01	1.04 (0.96, 1.14)	5.45E-01
1.03 (0.98, 1.08)	2.96E-01	1.00 (0.92, 1.09)	9.95E-01
1.07 (1.03, 1.12)	**2.41E-03**	1.02 (0.94, 1.11)	7.97E-01
0.97 (0.93, 1.02)	2.94E-01	0.90 (0.82, 0.98)	**4.07E-02**
1.01 (0.96, 1.05)	8.07E-01	1.07 (0.98, 1.17)	2.33E-01
1.00 (0.96, 1.05)	9.66E-01	1.06 (0.97, 1.16)	3.16E-01
1.02 (0.98, 1.07)	4.06E-01	0.99 (0.91, 1.08)	9.34E-01
1.04 (0.99, 1.08)	1.71E-01	1.02 (0.94, 1.10)	8.53E-01
0.99 (0.95, 1.03)	6.94E-01	0.81 (0.75, 0.87)	**7.58E-07**
1.03 (0.99, 1.08)	2.43E-01	1.36 (1.25, 1.48)	**3.68E-11**
1.03 (0.98, 1.08)	3.11E-01	1.36 (1.25, 1.47)	**4.88E-12**
1.04 (0.99, 1.08)	1.89E-01	1.12 (1.03, 1.22)	**2.33E-02**
0.95 (0.91, 1.00)	7.35E-02	0.93 (0.85, 1.01)	1.59E-01
0.88 (0.84, 0.92)	**1.94E-07**	0.93 (0.86, 1.01)	1.99E-01
1.12 (1.07, 1.17)	**9.28E-06**	1.12 (1.03, 1.22)	**2.58E-02**
1.07 (1.02, 1.12)	**6.79E-03**	1.01 (0.93, 1.10)	9.30E-01
1.16 (1.11, 1.22)	**5.72E-09**	1.28 (1.17, 1.41)	**1.44E-06**
1.00 (0.96, 1.04)	9.73E-01	0.91 (0.83, 0.99)	8.44E-02
1.17 (1.13, 1.21)	**5.99E-18**	1.18 (1.12, 1.25)	**7.36E-08**
0.92 (0.88, 0.96)	**4.39E-04**	0.94 (0.87, 1.02)	2.68E-01
0.92 (0.88, 0.95)	**8.19E-05**	0.94 (0.87, 1.02)	2.83E-01
0.98 (0.94, 1.03)	5.06E-01	0.96 (0.89, 1.04)	5.30E-01
0.96 (0.92, 1.01)	1.90E-01	0.90 (0.82, 0.98)	**3.18E-02**
0.98 (0.94, 1.02)	4.23E-01	1.00 (0.92, 1.08)	9.81E-01
1.02 (0.98, 1.07)	3.78E-01	1.02 (0.94, 1.11)	7.97E-01
1.02 (0.97, 1.06)	5.39E-01	1.01 (0.93, 1.10)	9.15E-01
1.04 (1.00, 1.09)	1.22E-01	1.09 (1.00, 1.18)	1.07E-01
0.98 (0.94, 1.03)	5.03E-01	0.93 (0.86, 1.02)	2.18E-01
0.93 (0.89, 0.97)	**4.09E-03**	0.98 (0.91, 1.07)	8.70E-01
1.10 (1.05, 1.15)	**1.76E-04**	0.96 (0.88, 1.05)	5.74E-01
1.18 (1.13, 1.23)	**2.59E-13**	1.19 (1.10, 1.29)	**4.41E-05**
1.03 (0.98, 1.07)	3.24E-01	1.08 (1.00, 1.17)	1.22E-01
1.02 (0.97, 1.06)	5.47E-01	1.12 (1.04, 1.21)	**1.23E-02**
1.02 (0.97, 1.06)	5.47E-01	1.10 (1.02, 1.19)	**4.07E-02**
0.99 (0.95, 1.03)	6.81E-01	1.04 (0.95, 1.13)	6.16E-01
1.07 (1.03, 1.12)	**4.88E-03**	1.04 (0.96, 1.13)	5.13E-01
1.06 (1.01, 1.10)	**2.57E-02**	1.01 (0.93, 1.10)	9.03E-01
1.16 (1.12, 1.21)	**2.00E-11**	1.30 (1.20, 1.40)	**2.61E-10**
0.91 (0.87, 0.96)	**5.05E-04**	0.83 (0.76, 0.91)	**2.06E-04**
0.93 (0.89, 0.98)	**9.11E-03**	0.90 (0.82, 0.99)	5.32E-02
0.97 (0.93, 1.02)	2.99E-01	1.02 (0.94, 1.11)	8.47E-01
0.96 (0.92, 1.01)	1.75E-01	1.03 (0.95, 1.11)	7.41E-01
1.05 (1.00, 1.10)	6.36E-02	1.03 (0.95, 1.12)	7.19E-01
0.99 (0.95, 1.04)	7.01E-01	0.90 (0.82, 0.98)	**4.35E-02**
0.99 (0.95, 1.04)	8.48E-01	0.99 (0.91, 1.07)	8.92E-01
0.97 (0.93, 1.02)	3.43E-01	1.05 (0.97, 1.14)	4.23E-01

0.89 (0.85, 0.93)	**3.65E-06**	0.80 (0.73, 0.87)	**1.44E-06**
0.86 (0.82, 0.90)	**2.94E-09**	0.79 (0.72, 0.86)	**7.58E-07**
0.97 (0.92, 1.01)	2.37E-01	0.91 (0.84, 0.99)	8.19E-02
1.07 (1.02, 1.12)	**5.49E-03**	1.09 (1.00, 1.18)	8.70E-02
0.98 (0.94, 1.03)	5.26E-01	1.05 (0.97, 1.14)	3.57E-01
0.95 (0.91, 1.00)	7.41E-02	1.01 (0.93, 1.09)	9.59E-01
0.93 (0.89, 0.98)	**1.18E-02**	0.93 (0.86, 1.02)	2.36E-01
0.80 (0.77, 0.84)	**3.79E-23**	0.72 (0.67, 0.77)	**8.88E-20**
1.11 (1.07, 1.16)	**4.83E-06**	1.44 (1.35, 1.55)	**1.05E-24**
0.91 (0.87, 0.96)	**4.39E-04**	0.88 (0.81, 0.96)	**1.34E-02**
1.01 (0.97, 1.06)	6.95E-01	1.22 (1.13, 1.32)	**2.75E-06**
0.89 (0.85, 0.94)	**1.78E-05**	0.83 (0.76, 0.91)	**2.65E-04**
0.91 (0.87, 0.95)	**3.57E-04**	0.89 (0.82, 0.98)	**3.30E-02**
0.95 (0.91, 1.00)	6.36E-02	0.97 (0.90, 1.06)	7.64E-01
0.95 (0.91, 1.00)	6.49E-02	0.94 (0.86, 1.02)	2.80E-01
1.21 (1.16, 1.26)	**7.13E-16**	1.76 (1.63, 1.90)	**2.75E-47**
0.91 (0.87, 0.95)	**1.06E-04**	0.84 (0.79, 0.91)	**3.08E-05**
1.08 (1.04, 1.13)	**9.88E-04**	1.47 (1.36, 1.58)	**1.12E-22**
0.90 (0.86, 0.94)	**1.78E-05**	0.77 (0.71, 0.84)	**3.79E-09**
0.92 (0.88, 0.96)	**7.96E-04**	0.87 (0.80, 0.94)	**2.30E-03**
1.02 (0.98, 1.07)	4.03E-01	1.13 (1.04, 1.22)	**1.03E-02**
1.11 (1.06, 1.17)	**1.58E-05**	1.07 (0.99, 1.17)	2.03E-01
1.12 (1.09, 1.16)	**2.08E-10**	1.15 (1.09, 1.22)	**1.02E-05**
0.97 (0.93, 1.02)	3.06E-01	1.13 (1.05, 1.22)	**4.43E-03**
0.99 (0.94, 1.04)	6.94E-01	0.81 (0.75, 0.89)	**2.76E-05**
0.92 (0.88, 0.97)	**1.56E-03**	1.03 (0.95, 1.12)	6.19E-01
0.90 (0.86, 0.95)	**4.74E-05**	0.97 (0.89, 1.05)	6.19E-01
0.99 (0.95, 1.04)	8.48E-01	1.22 (1.13, 1.32)	**2.71E-06**
0.93 (0.88, 0.97)	**3.00E-03**	1.00 (0.92, 1.08)	9.67E-01
0.95 (0.91, 1.00)	8.79E-02	1.13 (1.05, 1.23)	**6.32E-03**
0.82 (0.78, 0.86)	**4.74E-14**	0.84 (0.77, 0.92)	**7.42E-04**
1.12 (1.08, 1.15)	**1.23E-10**	1.19 (1.15, 1.24)	**1.33E-17**
1.03 (0.99, 1.08)	2.43E-01	1.11 (1.02, 1.20)	**3.28E-02**
1.06 (1.03, 1.10)	**1.73E-03**	1.09 (1.02, 1.16)	**3.28E-02**
1.02 (0.97, 1.07)	4.58E-01	0.86 (0.77, 0.95)	**1.43E-02**
1.05 (1.00, 1.09)	7.35E-02	0.92 (0.83, 1.03)	2.93E-01
1.04 (1.01, 1.07)	**2.12E-02**	1.04 (0.98, 1.09)	3.42E-01
0.85 (0.82, 0.88)	**2.80E-14**	0.74 (0.70, 0.79)	**1.73E-19**
1.15 (1.10, 1.21)	**1.23E-09**	1.38 (1.29, 1.49)	**9.17E-17**

tatus, alcohol consumption, self-rated health status, body mass index, and histo
VLDL, Very Low Density Lipoprotein; IDL, Intermediate Density Lipoprotein..

pants with prevalent cardiovascular diseaseas or cancer

Cerebrovascular diseases mortality		Cancer mortality	
**Adjusted HR (95% CI)**	**P value**	**Adjusted HR (95% CI)**	**P value**
0.91 (0.82, 0.99)	9.10E-02	0.92 (0.84, 1.00)	2.30E-01
0.91 (0.83, 1.00)	1.02E-01	0.92 (0.85, 1.01)	2.48E-01
0.92 (0.84, 1.01)	1.66E-01	0.92 (0.85, 1.01)	2.44E-01
0.94 (0.86, 1.03)	2.96E-01	0.97 (0.89, 1.05)	6.61E-01
0.94 (0.86, 1.03)	3.50E-01	0.98 (0.91, 1.07)	8.20E-01
0.93 (0.85, 1.02)	2.16E-01	0.95 (0.87, 1.03)	4.61E-01
0.90 (0.82, 0.99)	6.72E-02	0.91 (0.84, 1.00)	1.70E-01
0.93 (0.85, 1.02)	2.27E-01	0.97 (0.89, 1.05)	6.78E-01
0.93 (0.85, 1.02)	2.33E-01	0.97 (0.89, 1.05)	6.78E-01
0.93 (0.85, 1.02)	2.11E-01	0.97 (0.89, 1.05)	6.78E-01
0.94 (0.86, 1.03)	3.40E-01	0.98 (0.90, 1.07)	8.08E-01
0.95 (0.87, 1.04)	3.91E-01	0.98 (0.91, 1.07)	8.08E-01
0.93 (0.85, 1.02)	2.69E-01	0.98 (0.91, 1.07)	8.21E-01
0.93 (0.85, 1.02)	2.11E-01	0.97 (0.89, 1.05)	6.61E-01
0.94 (0.86, 1.03)	3.40E-01	0.93 (0.86, 1.01)	3.17E-01
0.94 (0.86, 1.03)	3.40E-01	0.94 (0.86, 1.02)	3.24E-01
0.94 (0.86, 1.03)	3.40E-01	0.94 (0.86, 1.02)	3.31E-01
0.94 (0.86, 1.03)	2.93E-01	0.95 (0.88, 1.03)	4.59E-01
0.93 (0.84, 1.01)	2.04E-01	0.96 (0.89, 1.04)	5.64E-01
0.95 (0.87, 1.04)	3.84E-01	0.94 (0.87, 1.02)	3.94E-01
0.95 (0.86, 1.03)	3.50E-01	0.93 (0.86, 1.01)	2.70E-01
0.95 (0.87, 1.04)	3.95E-01	0.93 (0.86, 1.01)	2.48E-01
0.95 (0.87, 1.04)	3.95E-01	0.93 (0.86, 1.01)	2.70E-01
0.95 (0.86, 1.04)	3.57E-01	0.93 (0.86, 1.01)	2.70E-01
0.95 (0.87, 1.04)	3.86E-01	0.96 (0.89, 1.04)	5.63E-01
0.94 (0.86, 1.03)	2.96E-01	0.98 (0.91, 1.06)	7.83E-01
0.96 (0.88, 1.05)	5.33E-01	0.94 (0.87, 1.02)	3.52E-01
0.95 (0.87, 1.04)	4.23E-01	0.92 (0.85, 1.00)	1.71E-01
0.97 (0.88, 1.06)	5.69E-01	0.95 (0.88, 1.02)	3.70E-01
0.98 (0.89, 1.07)	6.87E-01	0.95 (0.88, 1.02)	3.92E-01
0.96 (0.87, 1.05)	4.41E-01	0.94 (0.87, 1.01)	3.18E-01
1.06 (0.97, 1.15)	3.50E-01	0.97 (0.90, 1.04)	6.54E-01
1.08 (0.99, 1.18)	2.04E-01	0.98 (0.91, 1.05)	7.83E-01
1.01 (0.93, 1.11)	7.86E-01	0.95 (0.88, 1.03)	4.55E-01
0.94 (0.86, 1.03)	2.96E-01	0.94 (0.87, 1.02)	3.39E-01
1.06 (0.97, 1.15)	3.50E-01	0.98 (0.91, 1.06)	8.14E-01
1.07 (0.98, 1.17)	2.43E-01	0.99 (0.92, 1.06)	8.21E-01
1.00 (0.91, 1.09)	9.42E-01	1.01 (0.94, 1.09)	8.73E-01
1.14 (1.05, 1.24)	**7.01E-03**	0.98 (0.91, 1.06)	7.86E-01
1.15 (1.06, 1.25)	**4.07E-03**	0.98 (0.91, 1.05)	7.83E-01
1.12 (1.02, 1.22)	**3.27E-02**	0.99 (0.92, 1.06)	8.27E-01
0.98 (0.90, 1.08)	7.79E-01	0.98 (0.91, 1.05)	7.09E-01

0.98 (0.90, 1.08)	7.79E-01	1.02 (0.95, 1.10)	7.86E-01
0.98 (0.90, 1.08)	7.79E-01	1.02 (0.95, 1.10)	7.86E-01
0.96 (0.88, 1.06)	5.58E-01	1.01 (0.94, 1.09)	8.27E-01
0.98 (0.90, 1.07)	7.65E-01	1.02 (0.94, 1.09)	8.14E-01
0.97 (0.88, 1.06)	5.96E-01	1.01 (0.94, 1.09)	8.71E-01
1.02 (0.93, 1.11)	7.79E-01	1.03 (0.96, 1.11)	6.68E-01
1.04 (0.95, 1.13)	4.86E-01	1.02 (0.95, 1.10)	7.83E-01
0.96 (0.88, 1.06)	5.49E-01	1.00 (0.93, 1.08)	9.83E-01
0.96 (0.88, 1.06)	5.46E-01	1.00 (0.93, 1.08)	9.83E-01
0.93 (0.84, 1.02)	2.09E-01	1.00 (0.93, 1.08)	9.83E-01
0.96 (0.88, 1.05)	5.35E-01	0.99 (0.92, 1.07)	9.23E-01
0.96 (0.87, 1.05)	4.60E-01	0.99 (0.91, 1.07)	8.27E-01
0.98 (0.90, 1.07)	7.61E-01	1.02 (0.94, 1.10)	8.08E-01
1.07 (0.99, 1.17)	2.04E-01	1.04 (0.97, 1.12)	5.43E-01
0.95 (0.87, 1.04)	3.86E-01	0.97 (0.89, 1.04)	6.54E-01
0.95 (0.86, 1.04)	3.75E-01	0.97 (0.89, 1.04)	6.54E-01
0.89 (0.81, 0.97)	**2.07E-02**	0.96 (0.89, 1.04)	5.93E-01
0.96 (0.88, 1.05)	4.99E-01	0.96 (0.89, 1.04)	6.22E-01
0.96 (0.88, 1.06)	5.58E-01	0.96 (0.89, 1.04)	5.93E-01
0.94 (0.86, 1.02)	2.75E-01	0.97 (0.90, 1.05)	6.78E-01
1.06 (0.97, 1.16)	2.97E-01	1.03 (0.95, 1.11)	7.09E-01
0.95 (0.87, 1.04)	4.31E-01	0.95 (0.88, 1.03)	4.59E-01
0.95 (0.87, 1.05)	4.32E-01	0.95 (0.88, 1.03)	4.59E-01
0.89 (0.82, 0.97)	**2.32E-02**	0.95 (0.88, 1.03)	4.59E-01
0.97 (0.89, 1.07)	6.80E-01	0.95 (0.88, 1.03)	4.62E-01
0.98 (0.90, 1.07)	7.61E-01	0.95 (0.88, 1.03)	4.61E-01
0.95 (0.87, 1.04)	3.74E-01	0.95 (0.88, 1.03)	4.30E-01
1.02 (0.93, 1.11)	7.79E-01	0.98 (0.90, 1.05)	7.35E-01
1.24 (1.14, 1.34)	**2.30E-06**	1.13 (1.05, 1.20)	**3.64E-03**
1.24 (1.14, 1.34)	**2.43E-06**	1.12 (1.05, 1.20)	**4.52E-03**
1.20 (1.11, 1.31)	**6.83E-05**	1.17 (1.09, 1.25)	**9.25E-05**
1.26 (1.16, 1.37)	**6.36E-07**	1.07 (1.00, 1.15)	1.71E-01
1.28 (1.18, 1.39)	**9.82E-08**	1.05 (0.98, 1.13)	3.95E-01
1.21 (1.12, 1.32)	**3.34E-05**	1.13 (1.05, 1.20)	**3.23E-03**
1.18 (1.09, 1.28)	**2.87E-04**	1.05 (0.97, 1.13)	4.62E-01
1.16 (1.06, 1.26)	**2.40E-03**	1.14 (1.06, 1.23)	**1.66E-03**
1.16 (1.07, 1.26)	**2.34E-03**	1.14 (1.06, 1.23)	**1.66E-03**
1.15 (1.05, 1.25)	**5.26E-03**	1.13 (1.06, 1.22)	**3.64E-03**
1.16 (1.07, 1.26)	**2.17E-03**	1.14 (1.07, 1.23)	**1.37E-03**
1.15 (1.06, 1.25)	**2.97E-03**	1.15 (1.07, 1.23)	**1.32E-03**
1.18 (1.08, 1.28)	**6.21E-04**	1.14 (1.06, 1.22)	**1.72E-03**
1.17 (1.07, 1.27)	**1.45E-03**	1.13 (1.05, 1.21)	**6.47E-03**
0.85 (0.78, 0.93)	**1.90E-03**	1.02 (0.94, 1.10)	7.86E-01
0.85 (0.78, 0.93)	**2.40E-03**	1.02 (0.95, 1.10)	7.83E-01
0.90 (0.82, 0.98)	**4.82E-02**	1.02 (0.94, 1.10)	7.86E-01
0.85 (0.78, 0.93)	**1.49E-03**	1.02 (0.95, 1.10)	7.72E-01
0.84 (0.77, 0.91)	**4.45E-04**	1.02 (0.94, 1.10)	7.92E-01
0.90 (0.82, 0.98)	5.67E-02	1.04 (0.96, 1.12)	6.31E-01

0.82 (0.74, 0.90)	**1.95E-04**	1.00 (0.93, 1.08)	9.81E-01
0.79 (0.73, 0.85)	**1.64E-08**	0.95 (0.88, 1.03)	4.61E-01
0.79 (0.73, 0.85)	**2.09E-08**	0.96 (0.88, 1.03)	4.92E-01
0.82 (0.75, 0.89)	**6.54E-05**	0.94 (0.88, 1.02)	3.70E-01
0.86 (0.79, 0.94)	**2.19E-03**	1.01 (0.93, 1.09)	9.02E-01
0.89 (0.82, 0.97)	**1.67E-02**	1.01 (0.94, 1.10)	8.27E-01
0.85 (0.78, 0.93)	**1.49E-03**	0.97 (0.90, 1.04)	6.54E-01
0.89 (0.81, 0.97)	**2.89E-02**	0.96 (0.89, 1.04)	5.93E-01
1.02 (0.93, 1.12)	7.65E-01	0.86 (0.80, 0.92)	**9.83E-05**
1.17 (1.10, 1.25)	**2.43E-06**	1.17 (1.10, 1.25)	**2.73E-05**
1.17 (1.10, 1.24)	**2.83E-06**	1.17 (1.10, 1.24)	**3.12E-05**
1.09 (1.00, 1.18)	1.06E-01	1.07 (0.99, 1.16)	2.70E-01
0.84 (0.78, 0.90)	**2.11E-05**	0.91 (0.84, 0.99)	1.10E-01
0.91 (0.84, 0.99)	6.37E-02	0.93 (0.86, 1.01)	2.48E-01
1.13 (1.06, 1.21)	**9.29E-04**	1.06 (1.00, 1.13)	1.71E-01
1.19 (1.11, 1.28)	**9.82E-06**	1.06 (1.00, 1.13)	2.07E-01
0.97 (0.88, 1.05)	5.58E-01	1.03 (0.95, 1.12)	6.78E-01
0.95 (0.86, 1.04)	3.57E-01	0.97 (0.90, 1.05)	7.09E-01
0.98 (0.90, 1.07)	7.61E-01	0.95 (0.87, 1.03)	4.27E-01
0.95 (0.88, 1.04)	4.18E-01	1.06 (0.97, 1.15)	4.55E-01
0.93 (0.84, 1.02)	2.11E-01	1.16 (1.08, 1.24)	**7.84E-04**
1.01 (0.92, 1.10)	8.68E-01	0.92 (0.85, 0.99)	8.86E-02
1.04 (0.96, 1.14)	4.60E-01	0.97 (0.89, 1.06)	6.89E-01
0.88 (0.81, 0.97)	**2.57E-02**	1.10 (1.03, 1.18)	**3.26E-02**
1.00 (0.91, 1.10)	9.84E-01	1.19 (1.10, 1.29)	**8.36E-05**
0.97 (0.89, 1.07)	6.59E-01	1.18 (1.10, 1.27)	**5.98E-05**
1.09 (0.99, 1.19)	1.78E-01	0.96 (0.90, 1.04)	5.93E-01
1.02 (0.93, 1.12)	7.61E-01	0.84 (0.78, 0.90)	**6.96E-05**
0.89 (0.82, 0.98)	**3.79E-02**	1.05 (0.98, 1.13)	3.94E-01
1.18 (1.08, 1.29)	**1.72E-03**	1.05 (0.97, 1.13)	4.59E-01
1.15 (1.05, 1.26)	**7.88E-03**	1.04 (0.97, 1.12)	4.92E-01
1.26 (1.16, 1.36)	**9.29E-07**	1.05 (0.98, 1.14)	4.06E-01
0.88 (0.80, 0.96)	**1.18E-02**	0.94 (0.87, 1.01)	3.18E-01
0.86 (0.80, 0.93)	**5.67E-04**	1.10 (1.01, 1.19)	1.10E-01
1.28 (1.16, 1.41)	**5.69E-06**	0.97 (0.90, 1.04)	6.54E-01
1.24 (1.13, 1.36)	**3.11E-05**	0.96 (0.90, 1.03)	5.63E-01
1.17 (1.07, 1.29)	**3.67E-03**	1.00 (0.93, 1.07)	9.43E-01
0.91 (0.83, 1.00)	1.28E-01	0.99 (0.92, 1.06)	8.24E-01
0.91 (0.84, 0.99)	7.45E-02	0.99 (0.92, 1.07)	8.56E-01
0.99 (0.91, 1.07)	7.79E-01	0.99 (0.93, 1.07)	9.02E-01
0.93 (0.86, 1.01)	1.64E-01	0.95 (0.88, 1.02)	4.15E-01
1.07 (0.98, 1.17)	2.78E-01	1.05 (0.97, 1.13)	4.92E-01
1.05 (0.96, 1.13)	3.96E-01	1.01 (0.94, 1.09)	8.69E-01
0.93 (0.85, 1.02)	2.33E-01	0.99 (0.92, 1.08)	9.23E-01
0.98 (0.90, 1.07)	7.55E-01	0.98 (0.91, 1.06)	7.86E-01
0.96 (0.88, 1.03)	3.86E-01	0.96 (0.89, 1.03)	5.06E-01
1.09 (0.99, 1.19)	1.60E-01	1.07 (0.99, 1.16)	3.08E-01
1.08 (1.00, 1.17)	1.05E-01	1.03 (0.96, 1.11)	6.61E-01

0.93 (0.85, 1.02)	2.73E-01	1.01 (0.93, 1.09)	8.71E-01
1.01 (0.92, 1.10)	9.01E-01	0.97 (0.90, 1.05)	7.01E-01
1.00 (0.91, 1.09)	9.42E-01	0.97 (0.90, 1.05)	6.78E-01
1.03 (0.95, 1.13)	5.73E-01	1.03 (0.95, 1.12)	6.78E-01
1.10 (1.02, 1.19)	**3.79E-02**	1.05 (0.98, 1.14)	3.94E-01
0.93 (0.85, 1.02)	2.75E-01	1.03 (0.96, 1.11)	6.54E-01
1.03 (0.94, 1.12)	6.66E-01	0.97 (0.90, 1.05)	6.70E-01
1.02 (0.93, 1.11)	7.79E-01	0.97 (0.90, 1.05)	6.68E-01
1.03 (0.95, 1.13)	5.73E-01	1.03 (0.95, 1.11)	7.33E-01
1.05 (0.97, 1.14)	3.56E-01	1.02 (0.95, 1.10)	7.86E-01
0.92 (0.84, 1.00)	1.32E-01	1.20 (1.11, 1.30)	**5.98E-05**
1.14 (1.04, 1.25)	**1.86E-02**	0.84 (0.78, 0.90)	**2.89E-05**
1.15 (1.05, 1.26)	**1.04E-02**	0.80 (0.74, 0.87)	**4.17E-07**
1.03 (0.94, 1.13)	6.42E-01	1.02 (0.95, 1.10)	7.35E-01
0.94 (0.86, 1.03)	3.40E-01	0.95 (0.88, 1.02)	4.15E-01
0.87 (0.79, 0.95)	**8.69E-03**	0.88 (0.82, 0.96)	**1.12E-02**
1.15 (1.04, 1.26)	**1.86E-02**	1.09 (1.02, 1.18)	8.02E-02
1.06 (0.97, 1.16)	3.50E-01	1.11 (1.02, 1.19)	**4.56E-02**
1.25 (1.13, 1.38)	**1.77E-04**	1.07 (0.99, 1.15)	2.83E-01
1.00 (0.91, 1.09)	9.62E-01	1.01 (0.94, 1.09)	8.71E-01
1.23 (1.17, 1.30)	**8.24E-14**	1.01 (0.93, 1.10)	8.69E-01
0.87 (0.81, 0.94)	**1.58E-03**	0.99 (0.91, 1.07)	8.27E-01
0.87 (0.81, 0.93)	**3.60E-04**	0.98 (0.90, 1.05)	7.35E-01
0.97 (0.89, 1.06)	6.41E-01	1.03 (0.95, 1.11)	6.78E-01
0.94 (0.86, 1.03)	3.29E-01	1.01 (0.94, 1.09)	8.69E-01
0.98 (0.89, 1.07)	7.03E-01	0.96 (0.88, 1.03)	5.06E-01
1.02 (0.94, 1.12)	6.87E-01	1.04 (0.96, 1.13)	5.58E-01
1.01 (0.93, 1.11)	7.97E-01	1.04 (0.96, 1.12)	6.07E-01
1.08 (0.99, 1.18)	1.83E-01	1.00 (0.92, 1.08)	9.78E-01
0.99 (0.90, 1.08)	7.92E-01	0.99 (0.91, 1.06)	8.24E-01
0.94 (0.86, 1.03)	3.18E-01	0.88 (0.81, 0.95)	**5.16E-03**
1.05 (0.96, 1.15)	3.74E-01	1.20 (1.11, 1.29)	**6.96E-05**
1.26 (1.16, 1.37)	**2.07E-06**	1.12 (1.04, 1.20)	**1.17E-02**
1.01 (0.93, 1.11)	8.02E-01	1.02 (0.94, 1.09)	8.14E-01
1.02 (0.93, 1.11)	7.79E-01	0.95 (0.88, 1.02)	4.30E-01
1.00 (0.92, 1.10)	9.42E-01	0.97 (0.90, 1.05)	6.78E-01
0.97 (0.88, 1.06)	5.96E-01	0.98 (0.91, 1.06)	7.86E-01
1.08 (0.99, 1.18)	1.76E-01	1.11 (1.03, 1.19)	**2.56E-02**
1.06 (0.97, 1.15)	3.43E-01	1.11 (1.04, 1.20)	**1.66E-02**
1.26 (1.16, 1.38)	**1.14E-06**	1.04 (0.97, 1.11)	5.41E-01
0.85 (0.77, 0.94)	**3.68E-03**	1.02 (0.94, 1.10)	7.94E-01
0.86 (0.78, 0.95)	**7.86E-03**	1.01 (0.93, 1.09)	9.02E-01
0.96 (0.88, 1.05)	5.53E-01	0.94 (0.87, 1.01)	3.17E-01
0.95 (0.87, 1.04)	4.18E-01	0.92 (0.85, 1.00)	1.72E-01
1.07 (0.99, 1.17)	2.11E-01	1.02 (0.95, 1.10)	7.32E-01
0.98 (0.90, 1.07)	7.61E-01	1.03 (0.96, 1.11)	6.54E-01
0.94 (0.86, 1.03)	3.29E-01	1.06 (0.98, 1.14)	3.70E-01
0.97 (0.89, 1.06)	6.71E-01	0.93 (0.86, 1.00)	2.31E-01

0.82 (0.75, 0.89)	**7.00E-05**	1.03 (0.96, 1.12)	6.59E-01
0.79 (0.72, 0.86)	**3.32E-06**	0.97 (0.89, 1.05)	6.76E-01
0.91 (0.83, 0.99)	9.16E-02	1.06 (0.98, 1.15)	3.70E-01
1.08 (0.99, 1.17)	1.87E-01	1.08 (1.00, 1.16)	1.61E-01
0.96 (0.87, 1.05)	4.67E-01	0.96 (0.89, 1.03)	5.43E-01
0.93 (0.85, 1.01)	2.04E-01	0.93 (0.86, 1.00)	2.11E-01
0.87 (0.79, 0.96)	**1.36E-02**	1.00 (0.92, 1.08)	9.43E-01
0.75 (0.70, 0.81)	**8.24E-14**	0.99 (0.91, 1.07)	8.69E-01
1.22 (1.12, 1.32)	**2.90E-05**	0.88 (0.81, 0.94)	**3.53E-03**
0.85 (0.78, 0.93)	**2.33E-03**	1.00 (0.92, 1.08)	9.54E-01
1.04 (0.95, 1.14)	5.06E-01	0.88 (0.81, 0.95)	**1.04E-02**
0.81 (0.74, 0.90)	**1.85E-04**	1.01 (0.94, 1.10)	8.27E-01
0.84 (0.77, 0.93)	**2.34E-03**	0.99 (0.91, 1.07)	8.27E-01
0.92 (0.84, 1.01)	1.54E-01	0.97 (0.90, 1.05)	6.68E-01
0.87 (0.79, 0.95)	**8.93E-03**	1.03 (0.96, 1.11)	6.68E-01
1.46 (1.34, 1.59)	**3.98E-15**	0.83 (0.77, 0.89)	**1.69E-05**
0.90 (0.83, 0.97)	**1.67E-02**	1.02 (0.93, 1.11)	8.27E-01
1.23 (1.13, 1.34)	**1.23E-05**	0.79 (0.73, 0.85)	**1.47E-07**
0.86 (0.79, 0.93)	**2.17E-03**	1.07 (0.98, 1.16)	3.24E-01
0.91 (0.83, 0.99)	7.51E-02	0.99 (0.91, 1.07)	8.69E-01
1.10 (1.01, 1.20)	7.98E-02	0.90 (0.83, 0.97)	**2.60E-02**
1.03 (0.94, 1.13)	6.25E-01	1.19 (1.10, 1.29)	**1.70E-04**
1.21 (1.14, 1.28)	**9.09E-09**	0.97 (0.89, 1.05)	6.54E-01
1.05 (0.96, 1.14)	3.96E-01	0.77 (0.71, 0.84)	**4.17E-07**
0.83 (0.76, 0.92)	**1.23E-03**	1.28 (1.18, 1.38)	**8.51E-08**
0.98 (0.90, 1.07)	7.61E-01	0.79 (0.73, 0.85)	**3.07E-07**
1.02 (0.93, 1.11)	7.79E-01	0.79 (0.73, 0.85)	**2.76E-07**
1.14 (1.05, 1.24)	**7.88E-03**	0.74 (0.68, 0.80)	**1.72E-10**
0.92 (0.84, 1.01)	1.50E-01	0.88 (0.81, 0.95)	**9.07E-03**
0.97 (0.89, 1.06)	6.07E-01	0.82 (0.76, 0.89)	**6.96E-05**
0.77 (0.70, 0.85)	**2.07E-06**	0.82 (0.76, 0.89)	**6.96E-05**
1.16 (1.11, 1.21)	**1.86E-09**	0.85 (0.78, 0.92)	**1.37E-03**
1.11 (1.02, 1.21)	**3.71E-02**	0.86 (0.79, 0.93)	**1.76E-03**
1.13 (1.07, 1.19)	**2.66E-05**	0.97 (0.89, 1.05)	6.61E-01
1.01 (0.92, 1.10)	8.68E-01	1.14 (1.07, 1.20)	**6.96E-05**
1.02 (0.92, 1.12)	7.79E-01	1.10 (1.04, 1.16)	**6.37E-03**
1.07 (1.03, 1.10)	**2.48E-03**	0.71 (0.59, 0.85)	**1.38E-03**
0.78 (0.73, 0.84)	**3.05E-10**	1.10 (1.02, 1.19)	8.59E-02
1.21 (1.11, 1.31)	**5.67E-05**	0.98 (0.90, 1.06)	7.83E-01

UKB			
Other mortality		Sleep duration	
Adjusted HR (95% CI)	P value	Adjusted β (95% CI)	P value
1.02 (0.88, 1.17)	8.97E-01	0.02 (0.01, 0.02)	**3.80E-17**
1.02 (0.89, 1.18)	8.96E-01	0.02 (0.01, 0.02)	**9.55E-16**
1.03 (0.89, 1.18)	8.96E-01	0.02 (0.02, 0.02)	**9.97E-18**
1.06 (0.92, 1.21)	7.35E-01	0.02 (0.01, 0.02)	**5.90E-16**
1.06 (0.92, 1.21)	7.23E-01	0.02 (0.01, 0.02)	**2.84E-14**
1.05 (0.91, 1.21)	7.35E-01	0.02 (0.02, 0.02)	**1.14E-17**
1.01 (0.88, 1.17)	9.20E-01	0.02 (0.01, 0.02)	**8.43E-15**
1.02 (0.88, 1.18)	8.96E-01	0.02 (0.01, 0.02)	**6.78E-13**
1.02 (0.89, 1.18)	8.96E-01	0.02 (0.01, 0.02)	**1.38E-12**
1.04 (0.90, 1.20)	8.44E-01	0.02 (0.01, 0.02)	**3.06E-13**
1.03 (0.89, 1.18)	8.96E-01	0.01 (0.01, 0.02)	**3.84E-09**
1.02 (0.89, 1.18)	8.96E-01	0.01 (0.01, 0.01)	**1.16E-05**
1.04 (0.90, 1.20)	8.44E-01	0.02 (0.01, 0.02)	**4.61E-13**
1.02 (0.88, 1.17)	9.03E-01	0.02 (0.01, 0.02)	**4.19E-13**
1.01 (0.88, 1.17)	9.20E-01	0.02 (0.01, 0.02)	**1.71E-11**
1.01 (0.88, 1.17)	9.20E-01	0.01 (0.01, 0.02)	**5.93E-10**
1.02 (0.89, 1.17)	8.96E-01	0.02 (0.01, 0.02)	**6.17E-12**
1.01 (0.88, 1.16)	9.20E-01	0.01 (0.01, 0.02)	**2.88E-09**
0.99 (0.86, 1.14)	9.49E-01	0.01 (0.01, 0.02)	**5.54E-08**
1.03 (0.90, 1.18)	8.96E-01	0.01 (0.01, 0.02)	**3.51E-10**
1.01 (0.88, 1.17)	9.20E-01	0.01 (0.01, 0.02)	**5.52E-09**
0.98 (0.85, 1.13)	8.96E-01	0.00 (0.00, 0.01)	9.71E-02
0.98 (0.85, 1.13)	8.96E-01	0.01 (0.00, 0.01)	**4.76E-03**
0.98 (0.85, 1.13)	8.96E-01	0.00 (0.00, 0.01)	1.37E-01
0.98 (0.85, 1.13)	9.03E-01	0.00 (-0.01, 0.00)	**4.91E-02**
0.97 (0.84, 1.12)	8.96E-01	-0.01 (-0.01, 0.00)	**9.44E-05**
1.00 (0.87, 1.15)	9.74E-01	0.00 (0.00, 0.01)	5.68E-01
0.98 (0.85, 1.13)	8.96E-01	0.01 (0.01, 0.02)	**2.67E-08**
0.96 (0.83, 1.10)	8.17E-01	0.01 (0.01, 0.02)	**3.07E-11**
0.96 (0.83, 1.10)	7.99E-01	0.01 (0.01, 0.02)	**3.59E-09**
0.95 (0.83, 1.09)	7.35E-01	0.01 (0.00, 0.01)	**8.67E-03**
0.94 (0.81, 1.08)	6.56E-01	0.00 (0.00, 0.01)	**3.79E-02**
0.92 (0.80, 1.06)	5.50E-01	0.01 (0.00, 0.01)	**1.34E-03**
0.96 (0.84, 1.11)	8.44E-01	0.00 (0.00, 0.00)	9.38E-01
0.98 (0.85, 1.13)	8.96E-01	0.02 (0.02, 0.03)	**6.76E-22**
0.99 (0.86, 1.14)	9.33E-01	0.01 (0.01, 0.02)	**1.52E-06**
0.99 (0.86, 1.14)	9.20E-01	0.01 (0.01, 0.02)	**2.53E-08**
0.97 (0.84, 1.12)	8.96E-01	0.02 (0.01, 0.02)	**3.16E-12**
0.99 (0.86, 1.13)	9.20E-01	0.00 (0.00, 0.01)	1.39E-01
0.99 (0.87, 1.14)	9.58E-01	0.00 (0.00, 0.00)	8.28E-01
0.98 (0.86, 1.12)	8.96E-01	0.01 (0.01, 0.01)	**1.42E-05**
1.01 (0.88, 1.16)	9.20E-01	0.02 (0.02, 0.03)	**2.51E-27**

0.98 (0.85, 1.13)	8.97E-01	-0.01 (-0.01, -0.01)	**1.36E-05**
0.97 (0.85, 1.12)	8.96E-01	-0.01 (-0.02, -0.01)	**9.37E-08**
0.95 (0.82, 1.09)	7.35E-01	-0.01 (-0.01, 0.00)	**9.37E-04**
0.95 (0.82, 1.09)	7.35E-01	-0.02 (-0.02, -0.01)	**1.80E-13**
0.94 (0.82, 1.08)	7.06E-01	-0.02 (-0.02, -0.01)	**3.53E-14**
0.97 (0.84, 1.11)	8.52E-01	-0.02 (-0.02, -0.01)	**4.81E-11**
1.13 (0.99, 1.28)	2.19E-01	0.02 (0.02, 0.02)	**9.71E-20**
0.93 (0.81, 1.08)	6.51E-01	-0.01 (-0.01, 0.00)	**9.50E-03**
0.93 (0.80, 1.07)	5.97E-01	-0.02 (-0.02, -0.01)	**6.63E-12**
0.91 (0.78, 1.05)	4.45E-01	-0.02 (-0.02, -0.01)	**1.12E-13**
0.91 (0.79, 1.06)	4.80E-01	-0.02 (-0.02, -0.01)	**2.48E-14**
0.91 (0.79, 1.05)	4.49E-01	-0.02 (-0.02, -0.01)	**3.16E-12**
0.93 (0.81, 1.07)	6.30E-01	-0.02 (-0.03, -0.02)	**2.45E-20**
1.14 (1.01, 1.29)	1.87E-01	0.02 (0.01, 0.02)	**3.00E-12**
0.89 (0.77, 1.03)	3.34E-01	0.00 (-0.01, 0.00)	5.80E-02
0.88 (0.76, 1.02)	3.00E-01	-0.01 (-0.02, -0.01)	**7.89E-07**
0.87 (0.75, 1.00)	2.05E-01	-0.01 (-0.02, -0.01)	**5.52E-09**
0.88 (0.76, 1.02)	2.53E-01	-0.01 (-0.02, -0.01)	**3.83E-08**
0.88 (0.76, 1.02)	2.77E-01	-0.01 (-0.01, 0.00)	**4.59E-05**
0.87 (0.75, 1.00)	1.93E-01	-0.02 (-0.02, -0.02)	**3.82E-19**
1.11 (0.97, 1.27)	3.67E-01	0.02 (0.01, 0.02)	**9.66E-13**
0.87 (0.75, 1.00)	2.09E-01	0.00 (-0.01, 0.00)	5.16E-01
0.86 (0.75, 1.00)	2.01E-01	-0.01 (-0.01, 0.00)	**1.48E-04**
0.85 (0.74, 0.97)	1.46E-01	-0.01 (-0.01, 0.00)	**4.59E-05**
0.87 (0.75, 1.00)	2.09E-01	-0.01 (-0.02, -0.01)	**6.37E-07**
0.87 (0.76, 1.01)	2.23E-01	-0.01 (-0.01, 0.00)	**5.89E-04**
0.85 (0.74, 0.98)	1.46E-01	-0.02 (-0.02, -0.01)	**2.76E-17**
1.03 (0.89, 1.18)	8.96E-01	0.02 (0.01, 0.02)	**1.90E-17**
1.15 (1.01, 1.31)	1.87E-01	0.00 (0.00, 0.00)	9.89E-01
1.14 (1.00, 1.30)	1.89E-01	0.00 (0.00, 0.00)	9.95E-01
1.16 (1.02, 1.32)	1.46E-01	0.00 (0.00, 0.01)	5.20E-01
1.10 (0.96, 1.25)	4.17E-01	0.00 (-0.01, 0.00)	8.15E-02
1.09 (0.95, 1.24)	4.60E-01	-0.01 (-0.01, 0.00)	**1.40E-02**
1.12 (0.98, 1.27)	3.00E-01	0.00 (0.00, 0.01)	3.48E-01
1.22 (1.07, 1.38)	**4.03E-02**	0.02 (0.02, 0.03)	**2.56E-27**
1.17 (1.02, 1.33)	1.46E-01	-0.01 (-0.01, -0.01)	**6.82E-05**
1.16 (1.02, 1.33)	1.54E-01	-0.01 (-0.01, -0.01)	**8.29E-05**
1.14 (1.00, 1.30)	2.10E-01	-0.01 (-0.01, 0.00)	**2.03E-04**
1.16 (1.02, 1.33)	1.52E-01	-0.01 (-0.02, -0.01)	**8.76E-07**
1.17 (1.02, 1.33)	1.46E-01	-0.01 (-0.02, -0.01)	**8.60E-08**
1.15 (1.00, 1.31)	1.89E-01	-0.01 (-0.01, 0.00)	**8.77E-04**
1.30 (1.14, 1.47)	**4.28E-03**	0.02 (0.02, 0.02)	**6.79E-19**
0.91 (0.79, 1.05)	4.49E-01	-0.02 (-0.03, -0.02)	**1.90E-17**
0.91 (0.78, 1.05)	4.45E-01	-0.02 (-0.02, -0.01)	**1.29E-14**
0.95 (0.82, 1.10)	7.35E-01	-0.02 (-0.02, -0.01)	**1.84E-10**
0.88 (0.76, 1.01)	2.27E-01	-0.02 (-0.03, -0.02)	**2.51E-25**
0.86 (0.75, 1.00)	1.89E-01	-0.03 (-0.03, -0.02)	**9.05E-28**
0.93 (0.80, 1.07)	5.86E-01	-0.02 (-0.02, -0.01)	**1.29E-15**

0.98 (0.85, 1.13)	8.96E-01	0.02 (0.01, 0.02)	**9.28E-17**
0.81 (0.71, 0.92)	**2.03E-02**	-0.03 (-0.03, -0.02)	**1.89E-34**
0.80 (0.71, 0.91)	**1.53E-02**	-0.02 (-0.02, -0.01)	**3.01E-17**
0.87 (0.76, 1.00)	2.05E-01	-0.02 (-0.02, -0.01)	**5.57E-13**
0.81 (0.71, 0.92)	**3.35E-02**	-0.03 (-0.03, -0.03)	**2.79E-40**
0.84 (0.73, 0.96)	8.98E-02	-0.03 (-0.04, -0.03)	**9.78E-48**
0.85 (0.74, 0.97)	1.46E-01	-0.02 (-0.02, -0.01)	**3.69E-14**
1.01 (0.88, 1.16)	9.20E-01	0.02 (0.02, 0.02)	**2.41E-17**
1.08 (0.92, 1.27)	6.35E-01	0.02 (0.01, 0.02)	**2.65E-12**
1.13 (0.99, 1.28)	2.19E-01	-0.01 (-0.01, -0.01)	**6.76E-06**
1.09 (0.96, 1.25)	4.49E-01	-0.01 (-0.02, -0.01)	**2.50E-06**
1.14 (0.99, 1.30)	2.17E-01	-0.01 (-0.01, 0.00)	**5.18E-03**
0.87 (0.76, 1.00)	1.89E-01	0.00 (0.00, 0.01)	**4.91E-02**
1.06 (0.91, 1.25)	7.35E-01	-0.02 (-0.02, -0.01)	**6.48E-11**
1.08 (0.96, 1.22)	4.45E-01	-0.02 (-0.02, -0.01)	**1.84E-13**
1.03 (0.90, 1.18)	8.96E-01	0.00 (-0.01, 0.00)	1.95E-01
1.12 (0.98, 1.27)	3.00E-01	0.01 (0.01, 0.02)	**2.59E-06**
0.90 (0.77, 1.04)	3.99E-01	0.02 (0.01, 0.02)	**1.13E-16**
1.11 (0.94, 1.30)	4.71E-01	0.00 (-0.01, 0.00)	2.18E-01
1.04 (0.89, 1.21)	8.77E-01	-0.01 (-0.01, 0.00)	**4.29E-03**
0.98 (0.84, 1.14)	8.96E-01	0.01 (0.00, 0.01)	**2.72E-03**
1.06 (0.91, 1.23)	7.23E-01	-0.01 (-0.01, 0.00)	**1.93E-04**
0.95 (0.81, 1.11)	7.49E-01	-0.02 (-0.02, -0.01)	**3.53E-14**
0.96 (0.84, 1.11)	8.44E-01	-0.02 (-0.03, -0.02)	**3.37E-23**
1.07 (0.92, 1.23)	7.06E-01	-0.02 (-0.03, -0.02)	**4.22E-25**
1.03 (0.89, 1.19)	8.96E-01	-0.02 (-0.02, -0.01)	**9.98E-17**
1.10 (0.95, 1.28)	4.60E-01	0.02 (0.02, 0.03)	**1.02E-21**
0.95 (0.82, 1.10)	7.35E-01	-0.03 (-0.04, -0.03)	**3.75E-46**
1.00 (0.88, 1.15)	9.85E-01	-0.02 (-0.03, -0.02)	**1.64E-25**
0.96 (0.84, 1.10)	8.16E-01	-0.01 (-0.02, -0.01)	**8.02E-11**
0.95 (0.83, 1.09)	7.35E-01	-0.03 (-0.04, -0.03)	**2.01E-45**
1.05 (0.92, 1.21)	7.35E-01	0.03 (0.02, 0.03)	**3.01E-32**
1.04 (0.91, 1.19)	8.16E-01	0.03 (0.03, 0.04)	**1.08E-45**
0.95 (0.83, 1.08)	7.08E-01	-0.03 (-0.04, -0.03)	**3.20E-39**
1.01 (0.88, 1.15)	9.52E-01	-0.03 (-0.04, -0.03)	**1.08E-40**
1.02 (0.89, 1.17)	8.97E-01	-0.02 (-0.02, -0.01)	**1.17E-15**
0.98 (0.86, 1.11)	8.96E-01	0.03 (0.02, 0.03)	**4.23E-30**
1.02 (0.90, 1.17)	8.96E-01	0.03 (0.03, 0.03)	**1.15E-43**
0.92 (0.81, 1.06)	5.43E-01	-0.04 (-0.04, -0.03)	**2.85E-64**
0.89 (0.80, 0.99)	1.87E-01	-0.04 (-0.04, -0.03)	**1.80E-63**
0.86 (0.76, 0.97)	1.06E-01	-0.02 (-0.02, -0.01)	**8.92E-16**
0.98 (0.86, 1.11)	8.96E-01	0.04 (0.03, 0.04)	**1.14E-51**
1.15 (1.03, 1.29)	1.32E-01	0.00 (-0.01, 0.00)	9.99E-02
1.07 (0.94, 1.22)	6.04E-01	-0.03 (-0.04, -0.03)	**1.75E-41**
0.87 (0.78, 0.97)	9.83E-02	-0.01 (-0.02, -0.01)	**5.60E-09**
0.85 (0.76, 0.95)	**4.29E-02**	-0.03 (-0.03, -0.03)	**4.22E-39**
1.04 (0.90, 1.20)	8.44E-01	0.03 (0.03, 0.04)	**6.40E-48**
1.19 (1.07, 1.32)	**2.03E-02**	-0.01 (-0.02, -0.01)	**6.91E-08**

1.09 (0.96, 1.23)	4.60E-01	-0.02 (-0.03, -0.02)	**1.21E-26**
0.88 (0.78, 0.99)	1.87E-01	0.01 (0.00, 0.01)	**6.51E-03**
0.88 (0.78, 1.00)	1.89E-01	-0.03 (-0.03, -0.02)	**3.74E-35**
1.10 (0.96, 1.27)	3.99E-01	0.03 (0.03, 0.04)	**2.05E-47**
1.22 (1.09, 1.37)	**1.84E-02**	0.00 (0.00, 0.01)	4.78E-01
1.10 (0.96, 1.25)	4.17E-01	-0.03 (-0.03, -0.02)	**9.36E-37**
0.89 (0.78, 1.01)	2.36E-01	0.00 (-0.01, 0.00)	5.68E-01
0.89 (0.78, 1.01)	2.53E-01	-0.03 (-0.03, -0.02)	**1.20E-33**
1.08 (0.94, 1.25)	5.35E-01	0.03 (0.03, 0.03)	**4.42E-41**
1.15 (1.02, 1.31)	1.46E-01	0.02 (0.01, 0.02)	**9.96E-12**
1.12 (0.97, 1.29)	3.33E-01	-0.03 (-0.03, -0.02)	**1.37E-35**
0.85 (0.74, 0.97)	1.30E-01	-0.03 (-0.04, -0.03)	**1.03E-50**
0.84 (0.73, 0.97)	1.32E-01	-0.01 (-0.01, 0.00)	**1.75E-02**
0.91 (0.81, 1.02)	3.39E-01	0.01 (0.01, 0.02)	**4.04E-06**
1.10 (0.97, 1.24)	3.67E-01	0.01 (0.01, 0.01)	**2.13E-05**
0.76 (0.66, 0.89)	**1.53E-02**	-0.02 (-0.02, -0.01)	**2.93E-13**
1.11 (0.96, 1.28)	4.17E-01	-0.02 (-0.03, -0.02)	**5.17E-19**
1.15 (1.00, 1.33)	2.09E-01	0.01 (0.00, 0.01)	**1.75E-02**
1.03 (0.90, 1.18)	8.77E-01	0.02 (0.01, 0.02)	**6.05E-16**
1.19 (1.06, 1.34)	**4.29E-02**	0.03 (0.03, 0.04)	**1.75E-41**
1.23 (1.12, 1.34)	**2.10E-03**	-0.03 (-0.04, -0.03)	**1.18E-44**
0.85 (0.76, 0.95)	**4.98E-02**	-0.03 (-0.04, -0.03)	**3.38E-45**
0.84 (0.76, 0.94)	**3.05E-02**	-0.02 (-0.02, -0.01)	**1.05E-11**
0.95 (0.83, 1.09)	7.35E-01	0.03 (0.03, 0.03)	**3.60E-40**
1.06 (0.93, 1.22)	6.77E-01	0.02 (0.01, 0.02)	**3.22E-11**
1.06 (0.92, 1.22)	7.08E-01	-0.03 (-0.04, -0.03)	**1.89E-44**
0.92 (0.81, 1.06)	5.31E-01	-0.03 (-0.04, -0.03)	**5.40E-47**
0.93 (0.81, 1.06)	5.50E-01	0.00 (0.00, 0.01)	1.11E-01
1.03 (0.90, 1.19)	8.77E-01	0.03 (0.03, 0.04)	**3.75E-46**
1.11 (0.97, 1.27)	3.67E-01	0.01 (0.01, 0.02)	**2.23E-07**
0.99 (0.86, 1.13)	9.20E-01	-0.01 (-0.02, -0.01)	**6.24E-07**
1.31 (1.14, 1.49)	**4.71E-03**	0.00 (0.00, 0.01)	7.70E-01
1.16 (1.02, 1.33)	1.66E-01	-0.02 (-0.02, -0.01)	**6.62E-13**
0.94 (0.82, 1.08)	7.08E-01	0.01 (0.00, 0.01)	**8.49E-04**
0.98 (0.85, 1.13)	8.96E-01	-0.02 (-0.02, -0.01)	**5.31E-12**
0.96 (0.84, 1.11)	8.44E-01	0.00 (-0.01, 0.00)	1.05E-01
0.91 (0.79, 1.05)	4.49E-01	-0.02 (-0.02, -0.01)	**3.05E-16**
1.02 (0.89, 1.17)	8.96E-01	-0.01 (-0.01, -0.01)	**4.86E-06**
1.02 (0.89, 1.17)	8.98E-01		
1.05 (0.92, 1.20)	7.35E-01		
0.94 (0.81, 1.09)	7.08E-01	0.02 (0.01, 0.02)	**1.65E-15**
0.94 (0.81, 1.09)	7.23E-01	0.02 (0.01, 0.02)	**6.77E-14**
1.02 (0.89, 1.17)	8.96E-01	0.02 (0.01, 0.02)	**2.58E-13**
1.00 (0.87, 1.15)	9.67E-01	0.02 (0.02, 0.03)	**2.19E-20**
1.14 (1.00, 1.29)	2.01E-01	-0.01 (-0.01, 0.00)	**2.50E-03**
1.15 (1.01, 1.32)	1.87E-01	0.03 (0.02, 0.03)	**3.44E-26**
0.99 (0.86, 1.14)	9.20E-01	-0.01 (-0.01, 0.00)	**2.06E-04**
0.96 (0.84, 1.11)	8.44E-01	-0.02 (-0.02, -0.01)	**1.13E-11**

0.95 (0.82, 1.10)	7.35E-01	-0.01 (-0.02, -0.01)	**3.92E-06**
0.95 (0.82, 1.10)	7.35E-01	-0.02 (-0.02, -0.02)	**4.99E-17**
1.01 (0.87, 1.16)	9.49E-01	0.00 (-0.01, 0.00)	6.78E-02
1.01 (0.88, 1.16)	9.25E-01	0.01 (0.00, 0.01)	**8.83E-03**
0.94 (0.82, 1.09)	7.17E-01	0.01 (0.00, 0.01)	**2.30E-03**
0.96 (0.83, 1.10)	7.80E-01	0.00 (-0.01, 0.00)	4.35E-01
0.94 (0.81, 1.09)	7.06E-01	0.01 (0.01, 0.02)	**2.05E-10**
0.79 (0.70, 0.90)	**1.53E-02**	0.00 (-0.01, 0.00)	**2.84E-02**
0.88 (0.76, 1.01)	2.19E-01	0.02 (0.02, 0.03)	**2.83E-20**
0.92 (0.79, 1.07)	5.35E-01	-0.01 (-0.01, 0.00)	**8.90E-03**
0.85 (0.73, 0.99)	1.89E-01	0.00 (0.00, 0.00)	7.99E-01
0.91 (0.78, 1.05)	4.60E-01	0.01 (0.01, 0.02)	**1.34E-07**
0.89 (0.76, 1.04)	3.67E-01	0.01 (0.00, 0.01)	**3.05E-03**
0.96 (0.84, 1.11)	8.44E-01	0.01 (0.01, 0.02)	**1.12E-07**
0.96 (0.83, 1.11)	8.44E-01	-0.02 (-0.03, -0.02)	**3.20E-20**
0.93 (0.81, 1.06)	5.70E-01	0.02 (0.02, 0.03)	**7.65E-22**
0.90 (0.78, 1.03)	3.67E-01	-0.02 (-0.03, -0.02)	**2.42E-25**
0.86 (0.75, 0.99)	1.81E-01	-0.01 (-0.02, -0.01)	**1.59E-09**
0.90 (0.78, 1.03)	3.67E-01	0.02 (0.01, 0.02)	**4.81E-16**
0.85 (0.74, 0.98)	1.52E-01	0.00 (0.00, 0.01)	5.53E-01
1.09 (0.95, 1.25)	5.02E-01	0.02 (0.01, 0.02)	**1.28E-11**
1.16 (1.00, 1.33)	1.89E-01	0.00 (0.00, 0.01)	1.27E-01
1.24 (1.14, 1.36)	**1.72E-04**	0.00 (-0.01, 0.00)	1.22E-01
0.92 (0.79, 1.07)	5.50E-01	0.01 (0.00, 0.01)	**6.78E-05**
1.07 (0.92, 1.23)	7.06E-01	-0.03 (-0.03, -0.02)	**1.23E-32**
0.96 (0.83, 1.10)	8.09E-01	0.00 (0.00, 0.00)	8.06E-01
0.85 (0.73, 0.98)	1.46E-01	0.01 (0.00, 0.01)	**9.96E-03**
0.91 (0.79, 1.05)	4.49E-01	0.00 (0.00, 0.01)	9.16E-02
0.92 (0.80, 1.07)	5.72E-01	0.01 (0.01, 0.02)	**1.87E-10**
0.90 (0.78, 1.05)	4.45E-01	0.00 (0.00, 0.01)	6.94E-02
0.91 (0.79, 1.06)	5.09E-01	0.00 (0.00, 0.01)	7.01E-01
1.14 (1.04, 1.25)	5.72E-02	-0.01 (-0.01, 0.00)	**1.02E-02**
1.21 (1.06, 1.38)	5.73E-02	0.00 (0.00, 0.01)	1.19E-01
0.98 (0.85, 1.14)	9.05E-01	0.01 (0.00, 0.01)	**1.48E-02**
1.05 (0.91, 1.20)	7.44E-01	0.02 (0.02, 0.03)	**8.13E-18**
1.13 (1.03, 1.23)	9.83E-02	0.00 (-0.01, 0.00)	**3.79E-02**
1.08 (1.03, 1.12)	**1.38E-02**	0.02 (0.01, 0.02)	**5.69E-15**
0.83 (0.73, 0.94)	**4.29E-02**	-0.02 (-0.02, -0.01)	**4.56E-12**
1.00 (0.86, 1.16)	9.96E-01	0.01 (0.01, 0.01)	**1.93E-05**
		-0.01 (-0.01, -0.01)	**9.72E-06**
		-0.02 (-0.02, -0.01)	**2.48E-14**
		-0.01 (-0.02, -0.01)	**2.59E-09**
		-0.03 (-0.03, -0.02)	**4.20E-25**
		-0.02 (-0.02, -0.01)	**1.17E-13**
		-0.03 (-0.03, -0.02)	**3.00E-31**
		0.01 (0.01, 0.01)	**3.46E-06**
		-0.02 (-0.02, -0.02)	**4.59E-20**
		-0.02 (-0.02, -0.01)	**3.69E-10**

	0.00 (0.00, 0.01)	8.50E-02
	0.00 (-0.01, 0.00)	**4.64E-02**
	-0.03 (-0.03, -0.02)	**2.76E-29**
	0.01 (0.01, 0.02)	**1.02E-07**
	0.00 (-0.01, 0.00)	**2.67E-02**
	-0.03 (-0.03, -0.02)	**4.90E-30**
	-0.01 (-0.01, -0.01)	**3.95E-05**
	0.01 (0.01, 0.02)	**5.68E-09**
	-0.01 (-0.02, -0.01)	**3.25E-11**
	-0.01 (-0.02, -0.01)	**1.64E-09**
	-0.01 (-0.01, 0.00)	**2.16E-02**
	0.01 (0.01, 0.02)	**1.81E-10**
	-0.01 (-0.02, -0.01)	**1.16E-09**
	-0.02 (-0.02, -0.01)	**1.52E-12**
	0.01 (0.00, 0.01)	**2.22E-02**
	-0.02 (-0.03, -0.02)	**1.76E-21**
	0.01 (0.01, 0.02)	**4.69E-07**

All causes mortality		Ischaemic heart diseases mortality	
**Adjusted HR (95% CI)**	**P value**	**Adjusted HR (95% CI)**	**P value**
0.99 (0.97, 1.00)	1.53E-01	1.07 (1.02, 1.12)	**1.45E-02**
0.99 (0.97, 1.00)	1.13E-01	1.06 (1.01, 1.11)	**2.15E-02**
0.99 (0.98, 1.01)	2.32E-01	1.07 (1.02, 1.12)	**1.42E-02**
0.98 (0.97, 1.00)	5.78E-02	1.07 (1.02, 1.12)	**1.85E-02**
0.98 (0.97, 1.00)	**3.28E-02**	1.07 (1.02, 1.12)	**1.75E-02**
0.99 (0.97, 1.00)	1.14E-01	1.06 (1.02, 1.12)	**2.15E-02**
0.99 (0.97, 1.00)	1.17E-01	1.06 (1.01, 1.11)	**2.70E-02**
0.96 (0.94, 0.97)	**5.41E-08**	1.06 (1.01, 1.11)	**3.94E-02**
0.95 (0.94, 0.97)	**1.10E-08**	1.05 (1.00, 1.11)	6.39E-02
0.96 (0.94, 0.97)	**9.86E-08**	1.06 (1.01, 1.11)	**4.56E-02**
0.94 (0.93, 0.96)	**2.32E-13**	1.05 (0.99, 1.10)	1.28E-01
0.93 (0.92, 0.95)	**4.92E-18**	1.03 (0.98, 1.09)	2.81E-01
0.95 (0.94, 0.97)	**9.90E-09**	1.06 (1.00, 1.11)	6.06E-02
0.96 (0.94, 0.97)	**2.85E-07**	1.05 (1.00, 1.11)	6.22E-02
0.94 (0.93, 0.96)	**3.99E-13**	1.05 (1.00, 1.10)	1.06E-01
0.93 (0.92, 0.95)	**1.94E-17**	1.03 (0.98, 1.09)	2.50E-01
0.95 (0.93, 0.96)	**1.43E-11**	1.05 (1.00, 1.11)	6.87E-02
0.93 (0.92, 0.95)	**2.84E-17**	1.04 (0.99, 1.10)	1.97E-01
0.93 (0.92, 0.95)	**9.70E-19**	1.03 (0.98, 1.08)	3.66E-01
0.94 (0.92, 0.95)	**3.89E-15**	1.05 (1.00, 1.10)	1.04E-01
0.93 (0.91, 0.94)	**3.27E-19**	1.02 (0.97, 1.08)	4.34E-01
0.91 (0.90, 0.93)	**7.41E-30**	1.00 (0.95, 1.05)	9.86E-01
0.91 (0.90, 0.92)	**2.66E-31**	1.00 (0.95, 1.06)	8.94E-01
0.92 (0.90, 0.93)	**3.27E-28**	1.00 (0.95, 1.05)	9.63E-01
0.92 (0.90, 0.93)	**2.36E-28**	0.97 (0.92, 1.02)	3.08E-01
0.92 (0.91, 0.94)	**2.57E-25**	0.95 (0.91, 1.00)	1.12E-01
0.91 (0.90, 0.93)	**1.36E-28**	0.99 (0.94, 1.05)	8.50E-01
0.92 (0.91, 0.94)	**6.07E-23**	1.02 (0.97, 1.08)	4.20E-01
0.95 (0.94, 0.97)	**9.56E-10**	1.07 (1.02, 1.12)	**2.25E-02**
0.95 (0.93, 0.96)	**9.25E-12**	1.06 (1.01, 1.12)	**4.34E-02**
0.93 (0.92, 0.94)	**2.58E-19**	1.03 (0.98, 1.09)	2.80E-01
0.94 (0.93, 0.95)	**1.19E-14**	1.04 (0.98, 1.09)	2.31E-01
0.95 (0.94, 0.97)	**3.48E-10**	1.05 (1.00, 1.11)	7.43E-02
0.92 (0.91, 0.94)	**6.18E-24**	1.01 (0.96, 1.06)	8.53E-01
0.97 (0.96, 0.99)	**2.40E-04**	1.08 (1.03, 1.13)	**6.52E-03**
1.01 (0.99, 1.02)	3.91E-01	1.09 (1.04, 1.15)	**2.68E-03**
1.01 (1.00, 1.03)	1.32E-01	1.10 (1.04, 1.15)	**1.44E-03**
1.03 (1.01, 1.04)	**9.10E-04**	1.12 (1.06, 1.18)	**4.82E-05**
0.99 (0.98, 1.01)	2.63E-01	1.03 (0.98, 1.09)	2.63E-01
0.98 (0.97, 1.00)	**4.91E-02**	1.01 (0.96, 1.07)	6.47E-01
1.01 (0.99, 1.02)	3.62E-01	1.08 (1.03, 1.14)	**7.42E-03**
1.04 (1.02, 1.05)	**3.18E-06**	1.15 (1.10, 1.20)	**4.79E-08**

0.94 (0.93, 0.96)	**3.79E-14**	0.99 (0.94, 1.05)	8.66E-01
0.94 (0.93, 0.96)	**9.77E-14**	0.98 (0.93, 1.03)	5.17E-01
0.95 (0.94, 0.97)	**6.82E-09**	1.00 (0.95, 1.05)	9.06E-01
0.93 (0.91, 0.94)	**5.22E-20**	0.95 (0.90, 1.00)	1.09E-01
0.92 (0.91, 0.94)	**2.08E-22**	0.95 (0.90, 1.00)	7.18E-02
0.94 (0.93, 0.96)	**2.41E-13**	0.97 (0.92, 1.02)	3.08E-01
1.06 (1.04, 1.07)	**1.55E-12**	1.17 (1.11, 1.22)	**7.23E-10**
0.92 (0.90, 0.93)	**2.76E-27**	1.00 (0.95, 1.05)	9.39E-01
0.90 (0.89, 0.92)	**4.12E-37**	0.96 (0.92, 1.02)	2.31E-01
0.90 (0.89, 0.92)	**2.05E-38**	0.96 (0.91, 1.01)	1.45E-01
0.90 (0.88, 0.91)	**7.87E-43**	0.95 (0.90, 1.00)	8.05E-02
0.89 (0.88, 0.91)	**3.81E-44**	0.95 (0.91, 1.01)	1.18E-01
0.90 (0.89, 0.92)	**3.64E-37**	0.94 (0.89, 0.99)	**2.82E-02**
1.04 (1.03, 1.06)	**1.08E-08**	1.16 (1.11, 1.22)	**6.29E-10**
0.91 (0.89, 0.92)	**2.05E-33**	0.98 (0.93, 1.03)	5.62E-01
0.89 (0.88, 0.90)	**1.04E-47**	0.98 (0.93, 1.03)	5.00E-01
0.89 (0.87, 0.90)	**4.51E-50**	0.98 (0.93, 1.03)	5.62E-01
0.88 (0.87, 0.90)	**1.00E-52**	0.96 (0.92, 1.02)	2.31E-01
0.89 (0.87, 0.90)	**3.80E-49**	0.97 (0.93, 1.03)	3.83E-01
0.88 (0.87, 0.90)	**2.54E-55**	0.94 (0.90, 0.99)	**4.18E-02**
1.02 (1.00, 1.03)	**3.94E-02**	1.13 (1.08, 1.19)	**3.00E-07**
0.92 (0.91, 0.93)	**8.56E-26**	1.00 (0.95, 1.06)	9.07E-01
0.90 (0.89, 0.92)	**6.79E-39**	1.00 (0.95, 1.05)	8.95E-01
0.91 (0.90, 0.93)	**1.70E-30**	1.01 (0.96, 1.06)	7.60E-01
0.89 (0.88, 0.91)	**8.33E-47**	0.97 (0.93, 1.02)	3.82E-01
0.90 (0.88, 0.91)	**4.53E-42**	0.99 (0.94, 1.04)	6.38E-01
0.89 (0.88, 0.90)	**1.83E-49**	0.95 (0.90, 1.00)	7.01E-02
1.00 (0.98, 1.01)	6.83E-01	1.10 (1.05, 1.15)	**1.28E-04**
1.08 (1.06, 1.10)	**7.07E-20**	1.00 (0.94, 1.06)	9.99E-01
1.09 (1.07, 1.11)	**3.36E-25**	1.00 (0.94, 1.06)	9.66E-01
1.10 (1.08, 1.12)	**2.61E-30**	1.00 (0.94, 1.07)	9.81E-01
1.07 (1.06, 1.09)	**3.18E-17**	0.98 (0.92, 1.04)	6.13E-01
1.07 (1.05, 1.08)	**1.30E-13**	0.96 (0.90, 1.03)	3.40E-01
1.10 (1.08, 1.12)	**4.91E-32**	1.04 (0.98, 1.10)	2.95E-01
1.05 (1.04, 1.07)	**4.57E-11**	1.10 (1.05, 1.15)	**1.59E-04**
1.06 (1.04, 1.07)	**1.46E-09**	0.93 (0.87, 0.99)	**4.22E-02**
1.06 (1.04, 1.08)	**2.21E-11**	0.93 (0.87, 1.00)	7.18E-02
1.07 (1.05, 1.08)	**1.65E-12**	0.94 (0.88, 1.01)	1.29E-01
1.05 (1.03, 1.07)	**1.15E-08**	0.92 (0.86, 0.98)	**2.15E-02**
1.05 (1.03, 1.07)	**1.85E-07**	0.91 (0.85, 0.97)	**1.06E-02**
1.07 (1.05, 1.09)	**1.65E-13**	0.95 (0.89, 1.01)	1.85E-01
1.07 (1.06, 1.09)	**1.09E-19**	1.07 (1.02, 1.13)	**1.98E-02**
0.98 (0.96, 1.00)	**1.56E-02**	0.91 (0.86, 0.97)	**4.48E-03**
0.98 (0.97, 1.00)	5.12E-02	0.93 (0.88, 0.98)	**2.20E-02**
0.99 (0.97, 1.00)	1.87E-01	0.95 (0.89, 1.00)	8.05E-02
0.97 (0.96, 0.99)	**2.34E-03**	0.90 (0.84, 0.95)	**8.47E-04**
0.97 (0.95, 0.99)	**6.80E-04**	0.89 (0.84, 0.94)	**3.81E-04**
0.99 (0.97, 1.00)	1.26E-01	0.92 (0.87, 0.98)	**1.74E-02**

1.02 (1.01, 1.04)	**3.61E-03**	1.08 (1.03, 1.13)	**5.92E-03**
0.87 (0.86, 0.89)	**5.33E-64**	0.91 (0.86, 0.96)	**9.37E-04**
0.91 (0.89, 0.92)	**2.14E-34**	0.94 (0.90, 1.00)	6.01E-02
0.92 (0.91, 0.94)	**1.71E-22**	0.95 (0.91, 1.00)	1.13E-01
0.87 (0.86, 0.89)	**4.49E-62**	0.90 (0.85, 0.95)	**3.10E-04**
0.86 (0.85, 0.88)	**6.46E-73**	0.88 (0.84, 0.93)	**1.45E-05**
0.92 (0.91, 0.94)	**6.18E-24**	0.97 (0.92, 1.02)	2.80E-01
1.00 (0.98, 1.01)	6.27E-01	1.11 (1.05, 1.16)	**2.74E-04**
1.04 (1.02, 1.06)	**2.63E-05**	1.11 (1.04, 1.19)	**3.66E-03**
1.02 (1.00, 1.04)	**2.14E-02**	0.96 (0.90, 1.02)	2.75E-01
1.02 (1.00, 1.03)	8.05E-02	0.97 (0.91, 1.03)	3.68E-01
1.02 (1.01, 1.04)	**4.35E-03**	0.96 (0.90, 1.02)	2.19E-01
0.97 (0.96, 0.99)	**8.57E-04**	1.01 (0.95, 1.07)	8.50E-01
1.01 (1.00, 1.03)	1.29E-01	0.92 (0.86, 0.98)	**1.75E-02**
1.00 (0.99, 1.02)	7.29E-01	0.90 (0.84, 0.96)	**6.89E-03**
1.05 (1.03, 1.07)	**5.35E-09**	0.98 (0.92, 1.04)	5.88E-01
0.98 (0.96, 1.00)	**1.81E-02**	1.06 (0.99, 1.12)	1.20E-01
1.03 (1.01, 1.05)	**1.24E-03**	1.19 (1.10, 1.28)	**7.89E-05**
1.05 (1.03, 1.07)	**1.81E-09**	1.02 (0.97, 1.08)	5.03E-01
1.05 (1.03, 1.06)	**4.83E-08**	0.98 (0.93, 1.04)	6.14E-01
1.05 (1.03, 1.07)	**1.95E-08**	1.14 (1.07, 1.20)	**7.87E-05**
0.95 (0.93, 0.96)	**3.53E-10**	0.92 (0.87, 0.97)	**1.20E-02**
1.00 (0.98, 1.01)	8.17E-01	0.95 (0.91, 1.00)	8.33E-02
0.99 (0.97, 1.01)	2.07E-01	0.92 (0.87, 0.96)	**1.51E-03**
0.99 (0.97, 1.00)	1.55E-01	0.91 (0.86, 0.95)	**5.76E-04**
0.99 (0.98, 1.01)	4.45E-01	0.94 (0.89, 0.98)	**2.07E-02**
1.01 (0.99, 1.02)	2.90E-01	1.08 (1.03, 1.14)	**3.83E-03**
0.93 (0.92, 0.95)	**6.87E-18**	0.88 (0.83, 0.92)	**3.77E-06**
0.97 (0.96, 0.99)	**1.28E-04**	0.93 (0.88, 0.98)	**8.37E-03**
1.00 (0.98, 1.01)	9.66E-01	0.97 (0.93, 1.02)	3.72E-01
0.93 (0.92, 0.95)	**4.13E-17**	0.87 (0.83, 0.92)	**1.52E-06**
1.04 (1.03, 1.06)	**8.26E-08**	1.10 (1.04, 1.15)	**1.10E-03**
1.14 (1.12, 1.15)	**5.22E-56**	1.25 (1.19, 1.32)	**7.25E-17**
0.94 (0.93, 0.96)	**9.08E-15**	0.86 (0.82, 0.90)	**2.99E-09**
0.94 (0.93, 0.95)	**5.78E-16**	0.85 (0.81, 0.89)	**5.00E-10**
0.97 (0.96, 0.99)	**1.93E-04**	0.94 (0.90, 0.98)	**1.24E-02**
1.04 (1.03, 1.06)	**1.39E-07**	1.13 (1.08, 1.18)	**1.53E-06**
1.11 (1.09, 1.13)	**2.98E-41**	1.14 (1.08, 1.20)	**1.60E-06**
0.89 (0.88, 0.91)	**2.32E-57**	0.84 (0.81, 0.88)	**3.63E-15**
0.88 (0.86, 0.89)	**1.32E-75**	0.82 (0.78, 0.86)	**8.67E-18**
0.98 (0.96, 0.99)	**3.80E-03**	0.94 (0.90, 0.98)	**1.60E-02**
1.10 (1.09, 1.12)	**3.40E-46**	1.17 (1.13, 1.22)	**3.22E-14**
1.02 (1.00, 1.03)	**2.16E-02**	0.97 (0.93, 1.03)	3.85E-01
0.92 (0.91, 0.93)	**3.96E-108**	0.91 (0.90, 0.93)	**3.79E-17**
0.92 (0.91, 0.92)	**7.48E-131**	0.93 (0.91, 0.95)	**1.92E-09**
0.95 (0.94, 0.97)	**1.31E-10**	0.88 (0.84, 0.91)	**3.50E-09**
1.11 (1.10, 1.12)	**1.45E-99**	1.13 (1.10, 1.16)	**1.47E-20**
1.01 (1.00, 1.03)	1.16E-01	1.05 (1.00, 1.10)	6.37E-02

0.92 (0.91, 0.92)	**2.29E-108**	0.91 (0.89, 0.93)	**8.29E-20**
0.91 (0.90, 0.92)	**1.49E-40**	0.93 (0.88, 0.97)	**2.23E-03**
0.96 (0.94, 0.97)	**1.89E-08**	0.89 (0.85, 0.93)	**1.26E-06**
1.12 (1.11, 1.13)	**4.73E-114**	1.13 (1.10, 1.16)	**1.07E-19**
1.09 (1.08, 1.11)	**2.27E-32**	1.08 (1.03, 1.14)	**2.67E-03**
0.89 (0.88, 0.90)	**7.41E-110**	0.88 (0.86, 0.91)	**1.05E-18**
0.92 (0.90, 0.93)	**2.65E-29**	0.92 (0.88, 0.96)	**1.90E-03**
0.94 (0.92, 0.95)	**1.23E-18**	0.90 (0.86, 0.94)	**9.94E-06**
1.08 (1.06, 1.09)	**1.09E-29**	1.11 (1.07, 1.15)	**2.35E-09**
1.10 (1.09, 1.12)	**2.46E-29**	1.02 (0.97, 1.07)	5.62E-01
0.89 (0.88, 0.91)	**3.51E-41**	0.96 (0.92, 1.01)	1.95E-01
0.92 (0.91, 0.93)	**3.67E-37**	0.94 (0.90, 0.98)	**1.28E-02**
0.95 (0.94, 0.97)	**3.06E-08**	1.02 (0.97, 1.07)	4.82E-01
0.98 (0.97, 1.00)	**4.48E-02**	1.01 (0.97, 1.06)	5.62E-01
1.01 (0.99, 1.02)	4.32E-01	1.07 (1.02, 1.11)	**7.38E-03**
0.99 (0.98, 1.01)	2.85E-01	0.93 (0.89, 0.97)	**3.76E-03**
0.98 (0.97, 0.99)	**6.34E-03**	0.92 (0.88, 0.96)	**2.74E-04**
1.05 (1.03, 1.07)	**8.52E-10**	1.02 (0.98, 1.07)	3.66E-01
1.01 (0.99, 1.02)	2.86E-01	1.05 (1.01, 1.10)	**1.82E-02**
1.06 (1.05, 1.08)	**2.82E-14**	1.16 (1.10, 1.22)	**3.08E-08**
0.95 (0.93, 0.96)	**1.89E-11**	0.87 (0.82, 0.91)	**5.89E-08**
0.95 (0.93, 0.96)	**5.37E-12**	0.87 (0.83, 0.91)	**1.15E-07**
0.98 (0.96, 0.99)	**7.97E-03**	0.91 (0.86, 0.96)	**3.21E-03**
1.05 (1.03, 1.06)	**1.72E-08**	1.11 (1.07, 1.14)	**1.97E-09**
1.13 (1.11, 1.14)	**2.92E-48**	1.05 (1.00, 1.11)	1.11E-01
0.90 (0.89, 0.91)	**8.70E-44**	0.88 (0.84, 0.92)	**6.21E-07**
0.89 (0.87, 0.90)	**5.53E-56**	0.86 (0.82, 0.91)	**1.66E-08**
1.06 (1.04, 1.07)	**3.20E-11**	1.07 (1.01, 1.13)	**3.31E-02**
1.06 (1.04, 1.07)	**1.68E-11**	1.17 (1.12, 1.23)	**1.97E-09**
0.91 (0.90, 0.93)	**6.34E-28**	1.00 (0.95, 1.05)	9.52E-01
0.99 (0.97, 1.00)	1.18E-01	0.99 (0.95, 1.05)	8.73E-01
1.11 (1.09, 1.12)	**1.53E-30**	0.96 (0.90, 1.02)	2.29E-01
0.91 (0.90, 0.93)	**7.35E-30**	0.95 (0.90, 1.00)	7.05E-02
0.94 (0.93, 0.96)	**3.73E-14**	1.03 (0.98, 1.09)	3.08E-01
0.89 (0.88, 0.91)	**5.67E-47**	0.96 (0.91, 1.01)	1.24E-01
0.93 (0.92, 0.94)	**7.93E-20**	0.99 (0.94, 1.05)	8.73E-01
0.91 (0.90, 0.92)	**5.52E-31**	0.94 (0.89, 0.99)	**4.04E-02**
0.92 (0.90, 0.93)	**4.18E-26**	0.97 (0.92, 1.02)	2.50E-01
0.97 (0.95, 0.98)	**1.35E-04**	1.07 (1.02, 1.12)	**1.49E-02**
0.96 (0.94, 0.97)	**1.39E-08**	1.05 (1.00, 1.10)	6.69E-02
1.03 (1.02, 1.05)	**6.39E-05**	1.15 (1.10, 1.20)	**1.33E-08**
1.03 (1.01, 1.04)	**3.54E-04**	1.09 (1.04, 1.14)	**1.38E-03**
0.96 (0.94, 0.97)	**1.69E-07**	0.98 (0.93, 1.04)	6.26E-01
0.99 (0.97, 1.00)	7.57E-02	1.10 (1.04, 1.15)	**9.97E-04**
0.95 (0.93, 0.97)	**1.32E-09**	0.96 (0.90, 1.01)	1.58E-01
0.97 (0.96, 0.99)	**9.91E-04**	1.00 (0.95, 1.05)	9.71E-01

0.95 (0.93, 0.97)	**1.44E-09**	0.97 (0.92, 1.02)	2.87E-01
0.98 (0.96, 1.00)	**1.37E-02**	0.91 (0.86, 0.97)	**6.85E-03**
0.92 (0.91, 0.94)	**2.33E-25**	1.00 (0.95, 1.05)	9.81E-01
0.95 (0.93, 0.96)	**1.03E-10**	1.06 (1.00, 1.11)	5.82E-02
0.96 (0.95, 0.98)	**4.92E-07**	1.05 (1.00, 1.11)	6.53E-02
0.86 (0.85, 0.87)	**8.76E-74**	0.80 (0.76, 0.84)	**3.00E-15**
0.88 (0.86, 0.89)	**1.17E-48**	0.83 (0.78, 0.88)	**2.38E-09**
0.91 (0.89, 0.92)	**3.04E-32**	0.95 (0.90, 1.00)	6.37E-02
0.88 (0.86, 0.89)	**6.62E-52**	0.88 (0.83, 0.93)	**2.13E-05**
0.91 (0.89, 0.92)	**5.11E-33**	0.96 (0.91, 1.01)	2.02E-01
0.89 (0.87, 0.90)	**2.44E-47**	0.93 (0.89, 0.99)	**2.50E-02**
1.00 (0.99, 1.02)	7.06E-01	1.11 (1.06, 1.17)	**1.85E-05**
0.99 (0.98, 1.01)	2.06E-01	1.08 (1.03, 1.13)	**5.33E-03**
0.90 (0.89, 0.92)	**1.24E-34**	0.81 (0.76, 0.86)	**6.64E-12**
0.92 (0.90, 0.93)	**1.68E-28**	0.84 (0.80, 0.88)	**7.69E-11**
0.87 (0.86, 0.89)	**5.92E-55**	0.83 (0.78, 0.88)	**1.97E-09**
0.94 (0.93, 0.96)	**1.64E-13**	0.87 (0.83, 0.91)	**1.67E-08**
0.90 (0.89, 0.91)	**5.34E-42**	0.82 (0.78, 0.86)	**1.81E-15**
1.09 (1.07, 1.11)	**7.16E-26**	1.24 (1.18, 1.31)	**1.55E-16**
1.09 (1.08, 1.11)	**8.93E-32**	1.11 (1.06, 1.17)	**3.58E-05**
1.09 (1.07, 1.10)	**1.18E-47**	1.18 (1.15, 1.21)	**4.32E-37**
1.04 (1.03, 1.06)	**1.18E-07**	1.09 (1.04, 1.15)	**1.64E-03**
1.06 (1.04, 1.07)	**1.85E-15**	1.07 (1.02, 1.12)	**9.78E-03**
0.97 (0.96, 0.99)	**2.87E-04**	1.03 (0.98, 1.08)	3.21E-01
0.95 (0.93, 0.96)	**1.99E-11**	0.88 (0.84, 0.93)	**6.29E-06**
0.92 (0.90, 0.93)	**3.39E-23**	0.86 (0.81, 0.91)	**1.60E-06**
0.97 (0.95, 0.98)	**1.64E-04**	1.04 (0.99, 1.10)	1.33E-01
0.93 (0.91, 0.94)	**4.08E-20**	1.01 (0.96, 1.07)	6.44E-01
0.92 (0.91, 0.94)	**1.84E-21**	1.04 (0.99, 1.10)	1.71E-01
1.03 (1.02, 1.05)	**5.95E-06**	1.04 (1.01, 1.08)	**4.22E-02**
1.02 (1.00, 1.03)	**4.80E-02**	1.05 (1.00, 1.11)	7.26E-02
1.07 (1.06, 1.08)	**1.28E-83**	1.06 (1.04, 1.08)	**3.46E-07**
1.06 (1.05, 1.07)	**6.07E-38**	1.07 (1.05, 1.10)	**3.42E-09**
1.07 (1.06, 1.08)	**2.71E-38**	1.08 (1.04, 1.12)	**2.41E-05**
1.04 (1.03, 1.05)	**1.60E-13**	1.05 (1.03, 1.08)	**4.98E-06**
0.86 (0.85, 0.87)	**4.21E-81**	0.80 (0.76, 0.85)	**7.08E-16**
1.12 (1.10, 1.14)	**6.25E-48**	1.29 (1.23, 1.35)	**1.24E-22**
0.99 (0.97, 1.01)	1.87E-01	0.87 (0.81, 0.93)	**1.28E-04**
0.93 (0.92, 0.95)	**3.64E-17**	1.04 (0.98, 1.09)	2.41E-01
0.91 (0.89, 0.92)	**1.95E-33**	0.97 (0.93, 1.02)	3.74E-01
0.90 (0.88, 0.91)	**7.87E-43**	0.95 (0.90, 1.00)	9.52E-02
0.89 (0.88, 0.91)	**6.58E-47**	0.96 (0.91, 1.01)	2.02E-01
0.98 (0.97, 1.00)	8.25E-02	0.88 (0.82, 0.93)	**1.58E-04**
0.90 (0.89, 0.92)	**4.06E-33**	0.78 (0.74, 0.83)	**7.25E-17**
1.09 (1.08, 1.10)	**3.76E-68**	1.07 (1.03, 1.11)	**1.72E-03**
0.94 (0.93, 0.96)	**3.73E-14**	1.04 (0.99, 1.10)	1.69E-01
0.90 (0.88, 0.91)	**9.67E-44**	0.94 (0.89, 0.99)	**3.32E-02**
1.02 (1.00, 1.03)	7.11E-02	0.94 (0.88, 1.00)	8.82E-02

1.04 (1.02, 1.05)	**1.24E-08**	1.05 (1.02, 1.08)	**4.82E-03**
1.07 (1.06, 1.08)	**7.24E-51**	1.07 (1.04, 1.10)	**5.63E-05**
0.92 (0.90, 0.93)	**7.14E-23**	0.90 (0.85, 0.95)	**7.76E-04**
0.95 (0.94, 0.97)	**4.16E-09**	1.06 (1.01, 1.11)	5.14E-02
0.91 (0.90, 0.93)	**5.69E-30**	0.99 (0.95, 1.05)	8.66E-01
0.93 (0.91, 0.95)	**2.29E-17**	0.89 (0.85, 0.95)	**3.94E-04**
0.94 (0.93, 0.96)	**5.18E-12**	0.98 (0.93, 1.03)	5.17E-01
0.95 (0.94, 0.97)	**2.19E-10**	1.06 (1.00, 1.11)	6.06E-02
0.90 (0.88, 0.91)	**5.57E-42**	0.97 (0.92, 1.02)	3.34E-01
1.01 (0.99, 1.03)	2.65E-01	0.94 (0.89, 1.00)	7.40E-02
0.93 (0.91, 0.94)	**7.06E-22**	0.99 (0.94, 1.04)	7.98E-01
0.95 (0.93, 0.96)	**2.12E-11**	1.05 (1.00, 1.10)	8.07E-02
0.90 (0.88, 0.91)	**4.28E-41**	0.97 (0.92, 1.02)	3.40E-01
1.00 (0.99, 1.02)	7.11E-01	0.92 (0.87, 0.98)	**2.30E-02**
0.94 (0.93, 0.96)	**1.73E-13**	1.02 (0.97, 1.08)	4.72E-01
0.99 (0.97, 1.01)	3.31E-01	0.89 (0.83, 0.95)	**8.47E-04**
1.03 (1.01, 1.05)	**7.41E-03**	1.10 (1.02, 1.19)	**1.94E-02**

Cerebrovascular diseases mortality		Cancer mortality	
Adjusted HR (95% CI)	P value	Adjusted HR (95% CI)	P value
0.92 (0.85, 1.00)	8.10E-02	1.02 (1.00, 1.04)	5.08E-02
0.92 (0.85, 0.99)	6.92E-02	1.02 (1.00, 1.04)	6.62E-02
0.93 (0.86, 1.01)	1.10E-01	1.02 (1.00, 1.05)	**3.88E-02**
0.93 (0.86, 1.01)	1.37E-01	1.02 (1.00, 1.04)	6.99E-02
0.94 (0.86, 1.01)	1.62E-01	1.02 (1.00, 1.04)	8.72E-02
0.93 (0.86, 1.01)	1.15E-01	1.02 (1.00, 1.04)	5.64E-02
0.91 (0.84, 0.99)	5.23E-02	1.02 (1.00, 1.04)	7.66E-02
0.90 (0.83, 0.97)	**2.24E-02**	1.01 (0.98, 1.03)	6.82E-01
0.89 (0.82, 0.97)	**2.04E-02**	1.00 (0.98, 1.03)	7.78E-01
0.90 (0.83, 0.97)	**2.48E-02**	1.01 (0.98, 1.03)	6.58E-01
0.90 (0.83, 0.97)	**2.24E-02**	0.99 (0.97, 1.02)	6.58E-01
0.90 (0.83, 0.97)	**2.24E-02**	0.99 (0.96, 1.01)	2.45E-01
0.90 (0.83, 0.98)	**2.67E-02**	1.00 (0.98, 1.03)	7.90E-01
0.89 (0.82, 0.97)	**2.04E-02**	1.01 (0.98, 1.03)	6.49E-01
0.89 (0.82, 0.97)	**1.85E-02**	1.00 (0.97, 1.02)	6.91E-01
0.88 (0.81, 0.96)	**1.16E-02**	0.99 (0.97, 1.01)	3.44E-01
0.90 (0.83, 0.97)	**2.24E-02**	1.00 (0.98, 1.02)	9.06E-01
0.90 (0.83, 0.97)	**2.24E-02**	0.99 (0.97, 1.01)	3.42E-01
0.91 (0.84, 0.98)	**3.32E-02**	0.98 (0.96, 1.01)	1.99E-01
0.89 (0.82, 0.97)	**1.89E-02**	0.99 (0.97, 1.01)	5.40E-01
0.87 (0.80, 0.95)	**7.50E-03**	0.98 (0.96, 1.01)	2.01E-01
0.89 (0.82, 0.96)	**1.37E-02**	0.96 (0.94, 0.98)	**8.49E-04**
0.88 (0.81, 0.95)	**9.80E-03**	0.96 (0.94, 0.98)	**1.27E-03**
0.89 (0.82, 0.96)	**1.58E-02**	0.96 (0.94, 0.98)	**8.90E-04**
0.91 (0.85, 0.99)	**4.08E-02**	0.95 (0.93, 0.97)	**7.96E-06**
0.93 (0.86, 1.00)	9.49E-02	0.95 (0.93, 0.97)	**1.28E-06**
0.90 (0.83, 0.97)	**2.04E-02**	0.96 (0.94, 0.98)	**2.36E-04**
0.88 (0.81, 0.95)	**1.03E-02**	0.98 (0.96, 1.00)	6.94E-02
0.93 (0.86, 1.00)	9.12E-02	0.99 (0.97, 1.02)	6.30E-01
0.92 (0.85, 0.99)	6.39E-02	0.99 (0.97, 1.01)	3.93E-01
0.91 (0.84, 0.98)	**3.56E-02**	0.97 (0.95, 0.99)	**1.57E-02**
0.92 (0.86, 1.00)	7.47E-02	0.98 (0.96, 1.00)	5.88E-02
0.93 (0.86, 1.01)	1.21E-01	0.99 (0.97, 1.01)	3.09E-01
0.91 (0.84, 0.98)	**3.36E-02**	0.96 (0.94, 0.98)	**6.26E-04**
0.94 (0.86, 1.01)	1.52E-01	1.01 (0.99, 1.03)	4.03E-01
0.97 (0.90, 1.05)	6.02E-01	1.02 (1.00, 1.04)	1.61E-01
0.98 (0.91, 1.06)	7.10E-01	1.02 (1.00, 1.04)	8.14E-02
0.99 (0.92, 1.07)	8.78E-01	1.03 (1.01, 1.05)	**5.53E-03**
0.97 (0.90, 1.05)	5.61E-01	1.00 (0.98, 1.02)	8.81E-01
0.97 (0.90, 1.04)	4.96E-01	0.99 (0.97, 1.01)	4.39E-01
0.98 (0.91, 1.06)	7.31E-01	1.02 (0.99, 1.04)	1.83E-01
0.99 (0.92, 1.07)	9.17E-01	1.05 (1.03, 1.07)	**2.79E-05**

0.91 (0.84, 0.98)	**3.02E-02**	0.96 (0.94, 0.98)	**8.21E-04**
0.92 (0.85, 0.99)	6.20E-02	0.95 (0.93, 0.97)	**3.15E-05**
0.94 (0.87, 1.01)	1.39E-01	0.96 (0.94, 0.99)	**1.83E-03**
0.91 (0.84, 0.98)	**3.63E-02**	0.94 (0.92, 0.96)	**1.22E-07**
0.90 (0.84, 0.97)	**2.45E-02**	0.94 (0.92, 0.96)	**2.41E-08**
0.93 (0.87, 1.01)	1.21E-01	0.95 (0.93, 0.97)	**1.59E-05**
1.01 (0.94, 1.09)	8.65E-01	1.05 (1.03, 1.08)	**1.77E-06**
0.91 (0.84, 0.98)	**3.32E-02**	0.95 (0.93, 0.97)	**1.06E-06**
0.88 (0.82, 0.96)	**1.03E-02**	0.93 (0.91, 0.95)	**4.69E-10**
0.89 (0.82, 0.96)	**1.03E-02**	0.93 (0.91, 0.95)	**3.46E-10**
0.88 (0.81, 0.95)	**8.04E-03**	0.92 (0.90, 0.94)	**9.30E-12**
0.88 (0.81, 0.95)	**6.33E-03**	0.93 (0.91, 0.95)	**1.62E-11**
0.90 (0.83, 0.97)	**1.76E-02**	0.92 (0.90, 0.94)	**7.56E-12**
0.99 (0.92, 1.07)	8.87E-01	1.04 (1.02, 1.07)	**1.09E-04**
0.89 (0.82, 0.96)	**1.03E-02**	0.95 (0.93, 0.97)	**2.84E-05**
0.86 (0.80, 0.93)	**2.72E-03**	0.93 (0.91, 0.95)	**2.35E-09**
0.86 (0.80, 0.93)	**2.37E-03**	0.93 (0.91, 0.95)	**1.18E-10**
0.86 (0.80, 0.93)	**2.37E-03**	0.93 (0.91, 0.95)	**2.51E-10**
0.86 (0.79, 0.93)	**2.37E-03**	0.94 (0.92, 0.96)	**1.58E-08**
0.87 (0.81, 0.94)	**4.56E-03**	0.92 (0.90, 0.94)	**1.59E-14**
0.96 (0.89, 1.04)	4.35E-01	1.03 (1.01, 1.05)	**1.24E-02**
0.88 (0.82, 0.95)	**1.02E-02**	0.96 (0.94, 0.98)	**1.26E-04**
0.87 (0.81, 0.94)	**4.56E-03**	0.94 (0.92, 0.96)	**3.13E-08**
0.88 (0.82, 0.95)	**7.97E-03**	0.94 (0.92, 0.96)	**9.98E-08**
0.87 (0.80, 0.94)	**3.95E-03**	0.93 (0.91, 0.95)	**6.52E-10**
0.87 (0.80, 0.94)	**4.16E-03**	0.94 (0.92, 0.96)	**7.01E-08**
0.88 (0.82, 0.95)	**7.27E-03**	0.92 (0.90, 0.94)	**5.35E-14**
0.94 (0.87, 1.02)	1.82E-01	1.02 (1.00, 1.04)	6.97E-02
1.10 (1.02, 1.19)	**3.88E-02**	1.01 (0.99, 1.03)	4.95E-01
1.11 (1.03, 1.20)	**2.04E-02**	1.02 (0.99, 1.04)	2.44E-01
1.12 (1.04, 1.22)	**1.37E-02**	1.02 (1.00, 1.05)	8.13E-02
1.10 (1.02, 1.19)	**3.60E-02**	1.00 (0.98, 1.03)	8.77E-01
1.10 (1.01, 1.19)	**4.31E-02**	0.99 (0.97, 1.02)	6.91E-01
1.11 (1.03, 1.19)	**2.24E-02**	1.03 (1.01, 1.05)	**2.20E-02**
1.02 (0.94, 1.10)	7.41E-01	1.04 (1.02, 1.07)	**1.25E-04**
1.09 (1.00, 1.18)	7.56E-02	0.99 (0.96, 1.01)	3.39E-01
1.09 (1.00, 1.18)	6.99E-02	0.99 (0.97, 1.02)	5.38E-01
1.09 (1.00, 1.18)	8.54E-02	1.00 (0.97, 1.02)	7.48E-01
1.09 (1.01, 1.18)	6.92E-02	0.98 (0.96, 1.01)	1.99E-01
1.09 (1.00, 1.18)	6.99E-02	0.98 (0.96, 1.00)	1.19E-01
1.09 (1.01, 1.18)	6.74E-02	1.00 (0.97, 1.02)	7.21E-01
1.05 (0.98, 1.14)	2.54E-01	1.05 (1.03, 1.08)	**6.39E-06**
0.98 (0.90, 1.06)	7.39E-01	0.96 (0.94, 0.98)	**8.96E-04**
0.98 (0.90, 1.06)	6.95E-01	0.97 (0.94, 0.99)	**5.58E-03**
0.97 (0.90, 1.06)	6.39E-01	0.97 (0.95, 1.00)	**4.19E-02**
0.98 (0.91, 1.07)	7.79E-01	0.95 (0.93, 0.97)	**4.55E-05**
0.98 (0.90, 1.07)	7.71E-01	0.95 (0.93, 0.97)	**2.05E-05**
0.99 (0.91, 1.07)	8.16E-01	0.96 (0.94, 0.98)	**1.40E-03**

0.98 (0.91, 1.06)	7.07E-01	1.04 (1.02, 1.06)	**2.36E-04**
0.85 (0.79, 0.92)	**2.14E-03**	0.91 (0.89, 0.93)	**9.64E-15**
0.87 (0.81, 0.94)	**6.28E-03**	0.94 (0.92, 0.96)	**3.73E-07**
0.89 (0.82, 0.96)	**1.37E-02**	0.96 (0.94, 0.98)	**1.24E-04**
0.86 (0.79, 0.93)	**2.37E-03**	0.91 (0.89, 0.93)	**6.78E-16**
0.85 (0.79, 0.92)	**2.14E-03**	0.90 (0.89, 0.92)	**4.23E-18**
0.89 (0.82, 0.96)	**1.75E-02**	0.94 (0.92, 0.96)	**3.50E-07**
0.94 (0.87, 1.02)	2.13E-01	1.03 (1.01, 1.05)	**6.32E-03**
1.11 (1.02, 1.22)	**3.52E-02**	1.04 (1.01, 1.06)	**5.53E-03**
1.07 (1.00, 1.16)	1.04E-01	1.01 (0.99, 1.03)	5.40E-01
1.05 (0.97, 1.13)	3.43E-01	1.01 (0.98, 1.03)	6.58E-01
1.11 (1.03, 1.19)	**1.76E-02**	1.01 (0.99, 1.03)	4.16E-01
0.91 (0.85, 0.98)	**3.63E-02**	0.98 (0.96, 1.01)	1.69E-01
1.10 (1.01, 1.18)	**4.24E-02**	0.98 (0.96, 1.00)	1.50E-01
1.08 (1.00, 1.17)	9.39E-02	0.97 (0.95, 1.00)	5.08E-02
1.13 (1.05, 1.22)	**6.33E-03**	1.01 (0.99, 1.03)	3.99E-01
0.91 (0.85, 0.99)	**4.45E-02**	1.01 (0.98, 1.03)	5.62E-01
1.00 (0.92, 1.08)	9.39E-01	1.07 (1.04, 1.10)	**1.39E-06**
1.12 (1.04, 1.20)	**1.54E-02**	1.02 (1.00, 1.04)	9.23E-02
1.12 (1.04, 1.21)	**1.03E-02**	1.01 (0.99, 1.03)	4.44E-01
1.07 (0.99, 1.16)	1.63E-01	1.04 (1.02, 1.07)	**4.75E-04**
0.92 (0.84, 0.99)	6.63E-02	0.95 (0.93, 0.98)	**9.38E-05**
1.03 (0.96, 1.12)	4.88E-01	0.98 (0.96, 1.01)	1.83E-01
1.05 (0.97, 1.14)	2.74E-01	0.97 (0.94, 0.99)	**2.57E-03**
1.06 (0.98, 1.14)	2.49E-01	0.96 (0.94, 0.98)	**1.10E-03**
1.04 (0.97, 1.12)	3.84E-01	0.98 (0.95, 1.00)	**3.04E-02**
0.95 (0.88, 1.03)	3.07E-01	1.03 (1.01, 1.05)	**7.29E-03**
0.95 (0.88, 1.03)	3.38E-01	0.93 (0.91, 0.95)	**2.07E-10**
1.00 (0.93, 1.08)	9.83E-01	0.97 (0.94, 0.99)	**2.01E-03**
1.03 (0.95, 1.11)	5.61E-01	0.99 (0.97, 1.02)	6.76E-01
0.96 (0.89, 1.04)	4.66E-01	0.93 (0.91, 0.95)	**3.63E-10**
1.01 (0.94, 1.09)	8.16E-01	1.05 (1.03, 1.07)	**3.59E-05**
1.10 (1.02, 1.19)	**3.06E-02**	1.11 (1.09, 1.14)	**1.15E-20**
0.97 (0.90, 1.05)	5.61E-01	0.94 (0.92, 0.96)	**3.54E-08**
0.97 (0.90, 1.04)	4.90E-01	0.94 (0.92, 0.96)	**6.21E-09**
1.01 (0.94, 1.09)	8.60E-01	0.98 (0.96, 1.00)	**2.74E-02**
1.01 (0.94, 1.09)	8.29E-01	1.05 (1.03, 1.07)	**4.14E-05**
1.12 (1.04, 1.21)	**1.26E-02**	1.09 (1.07, 1.12)	**6.91E-16**
0.90 (0.84, 0.97)	**1.67E-02**	0.91 (0.89, 0.92)	**3.99E-21**
0.87 (0.81, 0.93)	**2.37E-03**	0.89 (0.88, 0.91)	**3.76E-26**
1.03 (0.95, 1.10)	6.16E-01	0.98 (0.96, 1.00)	**2.52E-02**
1.09 (1.01, 1.16)	**4.08E-02**	1.09 (1.07, 1.11)	**4.76E-17**
1.06 (0.99, 1.13)	1.30E-01	1.01 (0.99, 1.04)	1.99E-01
0.91 (0.88, 0.94)	**4.05E-05**	0.93 (0.92, 0.94)	**1.14E-29**
0.90 (0.88, 0.93)	**6.46E-08**	0.93 (0.92, 0.94)	**8.83E-26**
0.98 (0.91, 1.05)	6.36E-01	0.95 (0.93, 0.97)	**3.78E-07**
1.11 (1.06, 1.17)	**1.71E-03**	1.09 (1.07, 1.11)	**2.02E-26**
1.03 (0.96, 1.11)	5.61E-01	0.98 (0.95, 1.00)	**4.13E-02**

0.92 (0.88, 0.96)	**2.37E-03**	0.93 (0.92, 0.95)	**4.20E-19**
0.89 (0.83, 0.95)	**3.95E-03**	0.98 (0.96, 1.00)	6.08E-02
1.01 (0.94, 1.10)	8.25E-01	0.94 (0.92, 0.96)	**2.09E-08**
1.10 (1.05, 1.17)	**4.56E-03**	1.09 (1.08, 1.11)	**1.73E-26**
1.09 (1.01, 1.17)	**4.01E-02**	1.04 (1.01, 1.06)	**2.48E-03**
0.91 (0.86, 0.97)	**1.03E-02**	0.92 (0.90, 0.93)	**3.09E-20**
0.92 (0.85, 0.99)	**4.08E-02**	0.97 (0.95, 0.99)	**1.70E-02**
0.98 (0.91, 1.05)	6.72E-01	0.94 (0.92, 0.95)	**2.65E-10**
1.04 (0.97, 1.12)	3.95E-01	1.07 (1.05, 1.09)	**5.74E-12**
1.15 (1.04, 1.26)	**1.59E-02**	1.05 (1.02, 1.07)	**2.03E-04**
0.89 (0.82, 0.97)	**2.48E-02**	0.93 (0.91, 0.95)	**2.62E-09**
0.94 (0.88, 1.01)	1.63E-01	0.92 (0.90, 0.94)	**2.68E-16**
0.92 (0.84, 1.00)	8.94E-02	1.00 (0.98, 1.02)	9.20E-01
0.93 (0.86, 1.02)	1.70E-01	1.01 (0.99, 1.03)	3.09E-01
0.93 (0.87, 1.01)	1.17E-01	1.04 (1.02, 1.06)	**9.55E-04**
1.04 (0.97, 1.13)	3.71E-01	0.96 (0.94, 0.98)	**2.28E-04**
1.04 (0.97, 1.13)	3.92E-01	0.95 (0.93, 0.97)	**5.12E-06**
1.03 (0.96, 1.12)	4.97E-01	1.01 (0.99, 1.04)	2.48E-01
0.99 (0.92, 1.07)	9.00E-01	1.03 (1.01, 1.05)	**2.01E-03**
1.00 (0.93, 1.08)	9.84E-01	1.09 (1.07, 1.11)	**8.67E-14**
1.00 (0.92, 1.08)	9.30E-01	0.92 (0.90, 0.94)	**2.99E-12**
1.00 (0.92, 1.08)	9.70E-01	0.93 (0.91, 0.95)	**2.39E-11**
0.99 (0.91, 1.07)	8.10E-01	0.94 (0.92, 0.96)	**1.01E-06**
1.01 (0.93, 1.09)	9.00E-01	1.07 (1.05, 1.09)	**2.35E-10**
1.09 (1.01, 1.18)	6.20E-02	1.09 (1.07, 1.11)	**2.34E-13**
0.94 (0.88, 1.02)	1.99E-01	0.90 (0.89, 0.92)	**1.06E-19**
0.93 (0.86, 1.00)	9.02E-02	0.90 (0.89, 0.92)	**9.63E-20**
1.06 (0.99, 1.15)	1.62E-01	1.00 (0.98, 1.02)	8.92E-01
1.01 (0.93, 1.09)	9.00E-01	1.08 (1.05, 1.10)	**1.38E-10**
0.85 (0.79, 0.92)	**2.37E-03**	0.98 (0.96, 1.00)	9.51E-02
1.07 (0.99, 1.15)	1.58E-01	0.96 (0.94, 0.98)	**1.23E-03**
1.13 (1.04, 1.23)	**1.48E-02**	1.02 (1.00, 1.04)	1.47E-01
0.90 (0.83, 0.97)	**2.08E-02**	0.93 (0.91, 0.95)	**8.49E-10**
0.91 (0.85, 0.99)	**4.45E-02**	0.98 (0.96, 1.00)	1.33E-01
0.87 (0.81, 0.94)	**4.84E-03**	0.93 (0.91, 0.95)	**1.88E-11**
0.90 (0.84, 0.98)	**2.62E-02**	0.96 (0.94, 0.98)	**3.81E-04**
0.90 (0.83, 0.97)	**2.04E-02**	0.93 (0.91, 0.95)	**1.28E-10**
0.90 (0.84, 0.97)	**2.39E-02**	0.94 (0.92, 0.96)	**1.68E-07**
0.91 (0.84, 0.99)	**4.54E-02**	1.01 (0.99, 1.03)	3.99E-01
0.90 (0.83, 0.97)	**2.24E-02**	1.00 (0.98, 1.02)	8.81E-01
0.98 (0.90, 1.06)	6.72E-01	1.04 (1.02, 1.06)	**9.81E-04**
0.99 (0.91, 1.06)	7.90E-01	1.04 (1.02, 1.07)	**1.39E-04**
0.92 (0.85, 1.00)	9.51E-02	0.96 (0.94, 0.99)	**2.28E-03**
0.93 (0.86, 1.01)	1.39E-01	1.03 (1.01, 1.05)	**1.24E-02**
0.92 (0.85, 1.00)	9.28E-02	0.96 (0.93, 0.98)	**1.84E-04**
0.96 (0.89, 1.04)	4.17E-01	0.97 (0.94, 0.99)	**5.09E-03**

0.93 (0.85, 1.01)	1.10E-01	0.96 (0.93, 0.98)	**2.35E-04**
0.98 (0.90, 1.06)	7.31E-01	0.96 (0.93, 0.98)	**4.93E-04**
0.90 (0.83, 0.97)	**2.04E-02**	0.95 (0.93, 0.97)	**3.99E-05**
0.94 (0.87, 1.01)	1.60E-01	0.98 (0.96, 1.01)	1.99E-01
0.91 (0.84, 0.98)	**3.76E-02**	0.99 (0.97, 1.01)	3.03E-01
0.92 (0.85, 0.99)	6.39E-02	0.88 (0.86, 0.90)	**1.74E-26**
0.89 (0.82, 0.97)	**2.24E-02**	0.90 (0.88, 0.92)	**5.20E-17**
0.91 (0.85, 0.99)	**4.66E-02**	0.94 (0.92, 0.96)	**2.58E-07**
0.86 (0.79, 0.94)	**4.74E-03**	0.91 (0.89, 0.94)	**9.92E-14**
0.90 (0.83, 0.97)	**2.08E-02**	0.94 (0.92, 0.96)	**1.34E-07**
0.87 (0.81, 0.95)	**7.23E-03**	0.93 (0.90, 0.95)	**4.51E-11**
0.95 (0.88, 1.02)	2.53E-01	1.02 (1.00, 1.04)	5.10E-02
0.92 (0.85, 0.99)	6.63E-02	1.01 (0.99, 1.03)	4.38E-01
0.95 (0.88, 1.03)	3.30E-01	0.91 (0.89, 0.93)	**5.13E-16**
1.01 (0.93, 1.09)	8.87E-01	0.93 (0.91, 0.95)	**1.39E-11**
0.90 (0.83, 0.97)	**2.24E-02**	0.90 (0.88, 0.92)	**3.55E-17**
1.03 (0.95, 1.12)	5.61E-01	0.94 (0.92, 0.96)	**1.98E-08**
0.98 (0.91, 1.06)	7.87E-01	0.90 (0.88, 0.92)	**2.66E-19**
1.03 (0.95, 1.12)	5.61E-01	1.09 (1.07, 1.12)	**1.36E-14**
0.99 (0.92, 1.07)	8.59E-01	1.08 (1.06, 1.10)	**1.77E-11**
1.09 (1.03, 1.15)	**1.59E-02**	1.05 (1.03, 1.07)	**4.52E-08**
1.02 (0.94, 1.10)	7.41E-01	1.03 (1.01, 1.05)	**2.02E-02**
1.08 (1.02, 1.14)	**3.05E-02**	1.04 (1.01, 1.06)	**1.83E-03**
0.99 (0.92, 1.07)	8.65E-01	0.98 (0.95, 1.00)	**3.95E-02**
1.10 (1.02, 1.19)	**3.36E-02**	0.95 (0.93, 0.97)	**1.42E-06**
0.97 (0.90, 1.06)	6.26E-01	0.95 (0.93, 0.97)	**2.75E-05**
0.97 (0.89, 1.04)	4.88E-01	1.00 (0.98, 1.02)	9.22E-01
0.89 (0.82, 0.96)	**1.71E-02**	0.98 (0.95, 1.00)	**4.01E-02**
0.89 (0.82, 0.96)	**1.59E-02**	0.98 (0.96, 1.00)	6.09E-02
1.03 (0.96, 1.11)	5.49E-01	1.03 (1.01, 1.05)	**2.47E-03**
0.99 (0.92, 1.07)	8.65E-01	1.01 (0.99, 1.04)	2.45E-01
1.08 (1.05, 1.11)	**3.28E-05**	1.04 (1.03, 1.05)	**5.24E-08**
1.07 (1.03, 1.11)	**9.40E-03**	1.05 (1.03, 1.06)	**3.95E-09**
1.08 (1.03, 1.14)	**9.09E-03**	1.05 (1.03, 1.07)	**3.03E-08**
1.06 (1.02, 1.10)	**2.04E-02**	1.02 (1.00, 1.04)	7.23E-02
0.90 (0.83, 0.97)	**2.45E-02**	0.91 (0.89, 0.93)	**6.01E-18**
1.07 (0.99, 1.16)	1.15E-01	1.13 (1.11, 1.15)	**1.49E-26**
1.07 (0.99, 1.16)	1.58E-01	0.97 (0.95, 0.99)	**2.23E-02**
0.90 (0.83, 0.97)	**2.65E-02**	0.98 (0.96, 1.00)	1.18E-01
0.89 (0.82, 0.96)	**1.03E-02**	0.94 (0.92, 0.96)	**1.22E-07**
0.88 (0.82, 0.95)	**7.97E-03**	0.93 (0.91, 0.95)	**4.51E-11**
0.87 (0.80, 0.94)	**4.22E-03**	0.93 (0.91, 0.95)	**1.27E-10**
1.01 (0.93, 1.10)	8.29E-01	0.95 (0.92, 0.97)	**1.40E-05**
0.98 (0.90, 1.06)	7.31E-01	0.90 (0.88, 0.92)	**6.11E-18**
1.09 (1.05, 1.14)	**2.37E-03**	1.07 (1.05, 1.09)	**4.11E-16**
0.90 (0.83, 0.98)	**2.67E-02**	0.99 (0.97, 1.01)	2.51E-01
0.89 (0.82, 0.96)	**1.03E-02**	0.92 (0.90, 0.94)	**5.38E-13**
1.02 (0.94, 1.11)	7.02E-01	0.97 (0.95, 0.99)	**2.30E-02**

1.00 (0.92, 1.08)	9.65E-01	1.02 (1.00, 1.04)	**2.20E-02**
1.08 (1.05, 1.12)	**2.08E-04**	1.04 (1.02, 1.06)	**8.66E-06**
0.91 (0.84, 0.99)	6.09E-02	0.93 (0.90, 0.95)	**1.83E-10**
0.92 (0.85, 0.99)	6.20E-02	0.99 (0.97, 1.01)	5.80E-01
0.90 (0.83, 0.97)	**1.94E-02**	0.95 (0.93, 0.97)	**2.84E-06**
0.93 (0.85, 1.01)	1.20E-01	0.93 (0.91, 0.95)	**2.84E-09**
0.91 (0.84, 0.99)	5.63E-02	0.96 (0.93, 0.98)	**1.60E-04**
0.91 (0.84, 0.98)	**3.32E-02**	0.99 (0.97, 1.02)	6.58E-01
0.88 (0.81, 0.95)	**6.28E-03**	0.93 (0.91, 0.95)	**2.65E-10**
1.01 (0.93, 1.10)	9.00E-01	0.98 (0.95, 1.00)	7.40E-02
0.89 (0.82, 0.96)	**1.46E-02**	0.95 (0.93, 0.97)	**3.92E-05**
0.90 (0.83, 0.97)	**2.24E-02**	0.99 (0.97, 1.02)	6.73E-01
0.88 (0.81, 0.95)	**6.30E-03**	0.93 (0.91, 0.95)	**6.97E-10**
1.01 (0.93, 1.10)	8.65E-01	0.97 (0.94, 0.99)	**1.09E-02**
0.92 (0.86, 1.00)	7.32E-02	0.98 (0.96, 1.00)	8.14E-02
1.02 (0.93, 1.10)	8.03E-01	0.95 (0.93, 0.97)	**8.70E-05**
1.00 (0.92, 1.09)	9.67E-01	1.03 (1.01, 1.06)	**1.24E-02**

Other mortality	
Adjusted HR (95% CI)	P value
0.93 (0.90, 0.95)	**4.91E-08**
0.93 (0.90, 0.95)	**4.16E-08**
0.93 (0.91, 0.96)	**1.53E-07**
0.92 (0.89, 0.94)	**1.05E-09**
0.91 (0.89, 0.94)	**1.45E-10**
0.93 (0.90, 0.95)	**1.70E-08**
0.93 (0.91, 0.96)	**1.40E-07**
0.87 (0.85, 0.90)	**2.39E-22**
0.87 (0.85, 0.89)	**5.28E-23**
0.87 (0.85, 0.90)	**9.02E-22**
0.85 (0.83, 0.87)	**2.36E-30**
0.84 (0.81, 0.86)	**7.63E-36**
0.87 (0.85, 0.89)	**2.09E-23**
0.88 (0.85, 0.90)	**4.55E-20**
0.85 (0.83, 0.87)	**3.46E-30**
0.84 (0.81, 0.86)	**1.86E-35**
0.85 (0.83, 0.88)	**1.13E-28**
0.84 (0.81, 0.86)	**3.90E-36**
0.84 (0.81, 0.86)	**1.24E-36**
0.84 (0.82, 0.86)	**8.19E-34**
0.84 (0.81, 0.86)	**7.62E-36**
0.83 (0.81, 0.85)	**2.73E-42**
0.82 (0.80, 0.84)	**3.21E-46**
0.83 (0.81, 0.86)	**2.00E-39**
0.86 (0.83, 0.88)	**2.92E-30**
0.88 (0.86, 0.90)	**1.67E-22**
0.84 (0.82, 0.86)	**3.23E-37**
0.83 (0.80, 0.85)	**7.01E-41**
0.87 (0.85, 0.89)	**1.15E-23**
0.86 (0.84, 0.89)	**6.50E-26**
0.85 (0.83, 0.88)	**3.61E-31**
0.87 (0.84, 0.89)	**5.58E-25**
0.88 (0.86, 0.90)	**6.03E-21**
0.85 (0.83, 0.87)	**7.49E-32**
0.89 (0.87, 0.92)	**6.44E-16**
0.98 (0.95, 1.00)	1.13E-01
0.99 (0.96, 1.01)	2.98E-01
1.00 (0.98, 1.03)	8.92E-01
0.97 (0.95, 1.00)	5.07E-02
0.97 (0.95, 1.00)	**2.80E-02**
0.98 (0.96, 1.01)	1.87E-01
1.00 (0.97, 1.02)	9.48E-01

0.91 (0.88, 0.93)	**1.39E-13**
0.92 (0.90, 0.95)	**1.03E-09**
0.93 (0.91, 0.96)	**4.04E-07**
0.91 (0.89, 0.93)	**2.13E-12**
0.90 (0.88, 0.93)	**9.05E-14**
0.93 (0.90, 0.95)	**1.65E-08**
1.04 (1.01, 1.06)	**6.92E-03**
0.86 (0.84, 0.88)	**2.24E-28**
0.85 (0.83, 0.88)	**4.92E-32**
0.85 (0.83, 0.87)	**5.96E-33**
0.85 (0.82, 0.87)	**3.78E-35**
0.84 (0.82, 0.86)	**5.58E-38**
0.87 (0.84, 0.89)	**2.69E-26**
1.02 (1.00, 1.05)	9.99E-02
0.83 (0.81, 0.85)	**6.09E-41**
0.81 (0.79, 0.84)	**1.97E-51**
0.81 (0.79, 0.83)	**6.32E-52**
0.81 (0.79, 0.83)	**3.85E-55**
0.81 (0.78, 0.83)	**1.52E-55**
0.82 (0.80, 0.85)	**1.70E-46**
0.98 (0.95, 1.00)	6.15E-02
0.85 (0.83, 0.88)	**1.67E-31**
0.83 (0.81, 0.85)	**6.93E-42**
0.86 (0.84, 0.88)	**2.68E-31**
0.82 (0.80, 0.84)	**7.07E-48**
0.82 (0.80, 0.84)	**4.36E-47**
0.84 (0.82, 0.86)	**4.16E-40**
0.94 (0.92, 0.97)	**9.93E-06**
1.20 (1.17, 1.23)	**3.45E-41**
1.22 (1.19, 1.25)	**1.69E-49**
1.23 (1.20, 1.27)	**9.04E-56**
1.20 (1.17, 1.23)	**4.24E-40**
1.19 (1.16, 1.22)	**3.90E-36**
1.22 (1.19, 1.25)	**4.88E-52**
1.05 (1.03, 1.08)	**6.50E-05**
1.18 (1.15, 1.22)	**1.04E-31**
1.19 (1.16, 1.22)	**2.22E-34**
1.19 (1.16, 1.23)	**6.20E-35**
1.18 (1.15, 1.21)	**2.04E-31**
1.18 (1.14, 1.21)	**1.23E-29**
1.20 (1.16, 1.23)	**3.32E-37**
1.11 (1.08, 1.13)	**5.32E-15**
1.02 (1.00, 1.05)	9.99E-02
1.02 (0.99, 1.05)	1.30E-01
1.02 (0.99, 1.05)	1.45E-01
1.03 (1.00, 1.06)	8.29E-02
1.02 (0.99, 1.05)	1.34E-01
1.04 (1.01, 1.07)	**7.29E-03**

0.99 (0.96, 1.01)	4.25E-01
0.81 (0.79, 0.83)	**1.66E-53**
0.85 (0.83, 0.87)	**3.14E-32**
0.88 (0.85, 0.90)	**2.01E-21**
0.82 (0.80, 0.84)	**1.25E-47**
0.81 (0.79, 0.83)	**1.67E-55**
0.88 (0.86, 0.91)	**4.49E-19**
0.93 (0.90, 0.95)	**3.74E-08**
1.02 (0.99, 1.05)	1.64E-01
1.04 (1.02, 1.07)	**1.96E-03**
1.04 (1.01, 1.07)	**1.07E-02**
1.05 (1.02, 1.08)	**6.18E-04**
0.96 (0.93, 0.98)	**1.26E-03**
1.07 (1.04, 1.10)	**1.07E-06**
1.06 (1.03, 1.09)	**1.40E-04**
1.11 (1.08, 1.14)	**5.30E-15**
0.93 (0.91, 0.96)	**1.49E-06**
0.97 (0.94, 0.99)	**2.01E-02**
1.09 (1.06, 1.12)	**4.27E-11**
1.10 (1.07, 1.13)	**1.76E-13**
1.04 (1.01, 1.07)	**8.53E-03**
0.95 (0.92, 0.97)	**2.22E-04**
1.03 (1.00, 1.05)	5.29E-02
1.04 (1.01, 1.06)	**5.66E-03**
1.04 (1.01, 1.07)	**3.66E-03**
1.03 (1.01, 1.06)	**2.23E-02**
0.97 (0.94, 0.99)	**8.54E-03**
0.95 (0.93, 0.98)	**1.59E-04**
0.99 (0.96, 1.01)	2.78E-01
1.01 (0.99, 1.04)	4.16E-01
0.95 (0.93, 0.98)	**4.17E-04**
1.03 (1.00, 1.05)	5.59E-02
1.14 (1.11, 1.17)	**3.01E-22**
0.96 (0.94, 0.99)	**5.72E-03**
0.96 (0.94, 0.99)	**5.51E-03**
0.98 (0.95, 1.00)	**4.25E-02**
1.01 (0.99, 1.04)	2.97E-01
1.12 (1.09, 1.15)	**4.69E-19**
0.89 (0.87, 0.91)	**4.25E-23**
0.87 (0.85, 0.89)	**1.36E-32**
0.99 (0.97, 1.01)	3.86E-01
1.10 (1.08, 1.13)	**5.68E-18**
1.03 (1.00, 1.05)	**2.69E-02**
0.91 (0.90, 0.92)	**5.84E-59**
0.91 (0.90, 0.91)	**1.77E-101**
0.98 (0.96, 1.01)	2.28E-01
1.12 (1.11, 1.14)	**2.92E-55**
1.05 (1.03, 1.08)	**6.01E-05**

0.91 (0.90, 0.92)	**3.44E-79**
0.86 (0.85, 0.87)	**7.97E-76**
1.00 (0.97, 1.02)	9.00E-01
1.13 (1.12, 1.15)	**2.23E-76**
1.15 (1.13, 1.18)	**6.44E-47**
0.88 (0.86, 0.89)	**9.29E-89**
0.86 (0.84, 0.88)	**1.35E-43**
0.95 (0.93, 0.97)	**1.14E-05**
1.08 (1.05, 1.10)	**2.90E-12**
1.23 (1.19, 1.27)	**1.25E-36**
0.83 (0.81, 0.85)	**2.64E-41**
0.91 (0.89, 0.92)	**3.84E-20**
0.87 (0.85, 0.90)	**2.24E-19**
0.94 (0.92, 0.97)	**1.31E-05**
0.96 (0.94, 0.98)	**4.49E-04**
1.05 (1.02, 1.08)	**1.43E-04**
1.03 (1.01, 1.06)	**1.33E-02**
1.11 (1.08, 1.14)	**8.83E-15**
0.96 (0.93, 0.98)	**1.83E-03**
1.01 (0.98, 1.04)	4.67E-01
1.00 (0.98, 1.03)	9.26E-01
0.99 (0.97, 1.02)	5.41E-01
1.05 (1.02, 1.08)	**1.92E-03**
0.99 (0.96, 1.02)	5.08E-01
1.20 (1.17, 1.23)	**1.66E-42**
0.89 (0.87, 0.91)	**5.14E-19**
0.87 (0.85, 0.89)	**1.07E-30**
1.13 (1.10, 1.16)	**2.25E-21**
1.01 (0.98, 1.03)	7.30E-01
0.82 (0.80, 0.84)	**3.04E-48**
1.01 (0.99, 1.04)	3.96E-01
1.27 (1.23, 1.30)	**1.38E-63**
0.88 (0.86, 0.90)	**3.23E-21**
0.87 (0.84, 0.89)	**3.07E-25**
0.83 (0.81, 0.85)	**2.73E-42**
0.88 (0.86, 0.90)	**2.89E-22**
0.88 (0.86, 0.90)	**1.15E-20**
0.88 (0.86, 0.90)	**7.74E-22**
0.90 (0.87, 0.92)	**1.73E-15**
0.87 (0.85, 0.90)	**4.23E-22**
1.00 (0.97, 1.02)	9.48E-01
1.00 (0.97, 1.02)	8.60E-01
0.95 (0.92, 0.97)	**7.48E-05**
0.90 (0.88, 0.93)	**2.72E-13**
0.94 (0.92, 0.97)	**5.64E-05**
0.98 (0.95, 1.00)	9.99E-02

0.94 (0.92, 0.97)	**1.72E-05**
1.03 (1.00, 1.05)	9.21E-02
0.86 (0.84, 0.88)	**2.31E-29**
0.88 (0.85, 0.90)	**1.35E-21**
0.91 (0.88, 0.93)	**7.07E-13**
0.84 (0.82, 0.86)	**2.52E-36**
0.86 (0.83, 0.88)	**5.24E-25**
0.85 (0.83, 0.88)	**6.43E-31**
0.82 (0.80, 0.85)	**1.11E-37**
0.85 (0.83, 0.87)	**3.61E-32**
0.82 (0.80, 0.85)	**2.73E-42**
0.95 (0.93, 0.98)	**4.27E-04**
0.95 (0.93, 0.97)	**1.07E-04**
0.91 (0.88, 0.93)	**2.06E-11**
0.91 (0.89, 0.94)	**7.74E-12**
0.84 (0.81, 0.86)	**2.09E-32**
0.96 (0.94, 0.99)	**6.24E-03**
0.91 (0.89, 0.93)	**8.75E-14**
1.05 (1.02, 1.08)	**2.64E-04**
1.12 (1.10, 1.15)	**3.56E-20**
1.09 (1.07, 1.11)	**3.75E-21**
1.06 (1.03, 1.08)	**5.05E-05**
1.08 (1.06, 1.10)	**1.79E-13**
0.95 (0.92, 0.97)	**8.19E-05**
0.95 (0.93, 0.98)	**2.40E-04**
0.88 (0.86, 0.91)	**4.74E-18**
0.91 (0.88, 0.93)	**1.74E-11**
0.84 (0.81, 0.86)	**4.25E-33**
0.83 (0.80, 0.85)	**4.24E-40**
1.03 (1.01, 1.05)	**1.46E-02**
1.01 (0.99, 1.04)	3.49E-01
1.09 (1.08, 1.10)	**9.29E-89**
1.07 (1.05, 1.08)	**6.06E-23**
1.09 (1.07, 1.11)	**2.56E-31**
1.04 (1.03, 1.06)	**1.83E-09**
0.81 (0.79, 0.83)	**1.70E-59**
1.08 (1.05, 1.11)	**2.13E-08**
1.03 (1.00, 1.06)	5.63E-02
0.84 (0.82, 0.86)	**2.34E-32**
0.85 (0.83, 0.87)	**6.71E-33**
0.84 (0.82, 0.87)	**9.83E-37**
0.83 (0.80, 0.85)	**8.30E-45**
1.06 (1.03, 1.10)	**3.11E-05**
0.93 (0.90, 0.96)	**3.46E-07**
1.11 (1.10, 1.12)	**5.21E-57**
0.86 (0.84, 0.88)	**7.54E-28**
0.85 (0.83, 0.87)	**4.00E-33**
1.10 (1.07, 1.13)	**3.18E-11**

1.05 (1.03, 1.07)	**2.87E-07**
1.09 (1.08, 1.10)	**1.34E-57**
0.92 (0.89, 0.94)	**1.56E-09**
0.88 (0.86, 0.90)	**1.26E-20**
0.85 (0.83, 0.87)	**2.60E-33**
0.94 (0.91, 0.97)	**1.32E-05**
0.92 (0.90, 0.95)	**1.25E-08**
0.87 (0.85, 0.89)	**6.34E-24**
0.84 (0.82, 0.86)	**3.93E-39**
1.08 (1.05, 1.11)	**5.34E-07**
0.88 (0.85, 0.90)	**2.73E-22**
0.86 (0.84, 0.89)	**5.34E-26**
0.84 (0.82, 0.86)	**7.28E-39**
1.08 (1.04, 1.11)	**9.60E-07**
0.88 (0.85, 0.90)	**3.76E-22**
1.07 (1.04, 1.10)	**1.96E-06**
1.00 (0.97, 1.03)	8.61E-01

Supplementary Table 15. Association of all metabolites with sleep duration

		CKB	
		Sleep duration	
**Metabolites**	**Group**	**Adjusted β (95% CI)**	**P value**
Concentration of chylomicror	Lipoprotein subclasses	-0.01 (-0.04, 0.03)	8.67E-01
Total lipids in chylomicrons a	Lipoprotein subclasses	-0.01 (-0.04, 0.03)	8.70E-01
Phospholipids in chylomicror	Lipoprotein subclasses	-0.01 (-0.04, 0.02)	7.36E-01
Cholesterol in chylomicrons a	Lipoprotein subclasses	0.01 (-0.03, 0.04)	8.94E-01
Cholesteryl esters in chylomi	Lipoprotein subclasses	0.02 (-0.02, 0.05)	4.96E-01
Free cholesterol in chylomicr	Lipoprotein subclasses	-0.01 (-0.04, 0.02)	7.31E-01
Triglycerides in chylomicrons	Lipoprotein subclasses	-0.01 (-0.04, 0.03)	8.14E-01
Concentration of very large V	Lipoprotein subclasses	-0.01 (-0.04, 0.02)	7.31E-01
Total lipids in very large VLI	Lipoprotein subclasses	-0.01 (-0.04, 0.02)	7.51E-01
Phospholipids in very large V	Lipoprotein subclasses	-0.01 (-0.04, 0.03)	8.48E-01
Cholesterol in very large VLI	Lipoprotein subclasses	0.00 (-0.03, 0.04)	9.69E-01
Cholesteryl esters in very larg	Lipoprotein subclasses	0.01 (-0.03, 0.04)	8.94E-01
Free cholesterol in very large	Lipoprotein subclasses	0.00 (-0.04, 0.03)	9.04E-01
Triglycerides in very large VI	Lipoprotein subclasses	-0.01 (-0.05, 0.02)	6.67E-01
Concentration of large VLDL	Lipoprotein subclasses	-0.01 (-0.05, 0.02)	7.20E-01
Total lipids in large VLDL	Lipoprotein subclasses	-0.01 (-0.04, 0.02)	7.31E-01
Phospholipids in large VLDL	Lipoprotein subclasses	-0.01 (-0.04, 0.02)	7.36E-01
Cholesterol in large VLDL	Lipoprotein subclasses	0.00 (-0.04, 0.03)	9.41E-01
Cholesteryl esters in large VI	Lipoprotein subclasses	0.01 (-0.03, 0.04)	8.68E-01
Free cholesterol in large VLD	Lipoprotein subclasses	-0.01 (-0.05, 0.02)	7.19E-01
Triglycerides in large VLDL	Lipoprotein subclasses	-0.01 (-0.05, 0.02)	6.67E-01
Concentration of medium VL	Lipoprotein subclasses	0.00 (-0.04, 0.03)	9.03E-01
Total lipids in medium VLDI	Lipoprotein subclasses	0.00 (-0.04, 0.03)	9.31E-01
Phospholipids in medium VL	Lipoprotein subclasses	0.00 (-0.04, 0.03)	9.41E-01
Cholesterol in medium VLDI	Lipoprotein subclasses	0.01 (-0.02, 0.04)	7.36E-01
Cholesteryl esters in medium	Lipoprotein subclasses	0.03 (-0.01, 0.06)	3.16E-01
Free cholesterol in medium V	Lipoprotein subclasses	-0.01 (-0.04, 0.03)	8.68E-01
Triglycerides in medium VLI	Lipoprotein subclasses	-0.01 (-0.04, 0.02)	7.41E-01
Concentration of small VLDI	Lipoprotein subclasses	0.00 (-0.03, 0.04)	9.39E-01
Total lipids in small VLDL	Lipoprotein subclasses	0.00 (-0.03, 0.04)	9.01E-01
Phospholipids in small VLDI	Lipoprotein subclasses	0.00 (-0.04, 0.03)	9.39E-01
Cholesterol in small VLDL	Lipoprotein subclasses	0.03 (-0.01, 0.06)	2.82E-01
Cholesteryl esters in small VI	Lipoprotein subclasses	0.04 (-0.01, 0.07)	1.12E-01
Free cholesterol in small VLI	Lipoprotein subclasses	0.00 (-0.03, 0.03)	9.70E-01
Triglycerides in small VLDL	Lipoprotein subclasses	-0.01 (-0.04, 0.03)	8.57E-01
Concentration of very small V	Lipoprotein subclasses	0.01 (-0.02, 0.05)	6.51E-01
Total lipids in very small VLI	Lipoprotein subclasses	0.02 (-0.02, 0.05)	5.43E-01
Phospholipids in very small V	Lipoprotein subclasses	0.02 (-0.02, 0.05)	5.81E-01
Cholesterol in very small VLI	Lipoprotein subclasses	0.03 (-0.00, 0.06)	2.55E-01
Cholesteryl esters in very sma	Lipoprotein subclasses	0.03 (-0.00, 0.06)	1.73E-01
Free cholesterol in very small	Lipoprotein subclasses	0.01 (-0.02, 0.05)	6.15E-01
Triglycerides in very small V	Lipoprotein subclasses	-0.01 (-0.05, 0.02)	6.67E-01

Concentration of IDL particle	Lipoprotein subclasses	0.02 (-0.01, 0.06)	3.17E-01
Total lipids in IDL	Lipoprotein subclasses	0.03 (-0.01, 0.06)	2.82E-01
Phospholipids in IDL	Lipoprotein subclasses	0.03 (-0.00, 0.06)	2.55E-01
Cholesterol in IDL	Lipoprotein subclasses	0.03 (-0.01, 0.06)	2.63E-01
Cholesteryl esters in IDL	Lipoprotein subclasses	0.03 (-0.01, 0.06)	2.55E-01
Free cholesterol in IDL	Lipoprotein subclasses	0.02 (-0.01, 0.06)	2.90E-01
Triglycerides in IDL	Lipoprotein subclasses	-0.02 (-0.05, 0.02)	5.85E-01
Concentration of large LDL p	Lipoprotein subclasses	0.03 (-0.01, 0.06)	2.82E-01
Total lipids in large LDL	Lipoprotein subclasses	0.03 (-0.01, 0.06)	2.79E-01
Phospholipids in large LDL	Lipoprotein subclasses	0.03 (-0.01, 0.06)	2.79E-01
Cholesterol in large LDL	Lipoprotein subclasses	0.03 (-0.00, 0.06)	2.36E-01
Cholesteryl esters in large LD	Lipoprotein subclasses	0.03 (-0.00, 0.06)	2.34E-01
Free cholesterol in large LDL	Lipoprotein subclasses	0.03 (-0.01, 0.06)	2.55E-01
Triglycerides in large LDL	Lipoprotein subclasses	-0.01 (-0.05, 0.02)	6.94E-01
Concentration of medium LD	Lipoprotein subclasses	0.03 (-0.01, 0.06)	2.86E-01
Total lipids in medium LDL	Lipoprotein subclasses	0.03 (-0.01, 0.06)	2.82E-01
Phospholipids in medium LD	Lipoprotein subclasses	0.02 (-0.01, 0.06)	3.30E-01
Cholesterol in medium LDL	Lipoprotein subclasses	0.03 (-0.01, 0.06)	2.55E-01
Cholesteryl esters in medium	Lipoprotein subclasses	0.03 (-0.00, 0.06)	2.53E-01
Free cholesterol in medium L	Lipoprotein subclasses	0.02 (-0.01, 0.06)	3.51E-01
Triglycerides in medium LDL	Lipoprotein subclasses	-0.01 (-0.04, 0.02)	7.68E-01
Concentration of small LDL p	Lipoprotein subclasses	0.02 (-0.01, 0.05)	4.32E-01
Total lipids in small LDL	Lipoprotein subclasses	0.02 (-0.01, 0.05)	4.27E-01
Phospholipids in small LDL	Lipoprotein subclasses	0.01 (-0.02, 0.04)	7.31E-01
Cholesterol in small LDL	Lipoprotein subclasses	0.02 (-0.01, 0.06)	3.30E-01
Cholesteryl esters in small LD	Lipoprotein subclasses	0.02 (-0.01, 0.06)	3.51E-01
Free cholesterol in small LDL	Lipoprotein subclasses	0.01 (-0.02, 0.05)	6.56E-01
Triglycerides in small LDL	Lipoprotein subclasses	0.00 (-0.04, 0.03)	9.54E-01
Concentration of very large H	Lipoprotein subclasses	-0.03 (-0.06, 0.01)	2.82E-01
Total lipids in very large HDL	Lipoprotein subclasses	-0.03 (-0.06, 0.01)	2.79E-01
Phospholipids in very large H	Lipoprotein subclasses	-0.03 (-0.06, 0.01)	3.16E-01
Cholesterol in very large HDL	Lipoprotein subclasses	-0.03 (-0.06, 0.00)	2.53E-01
Cholesteryl esters in very larg	Lipoprotein subclasses	-0.03 (-0.06, 0.01)	2.64E-01
Free cholesterol in very large	Lipoprotein subclasses	-0.03 (-0.06, 0.00)	2.12E-01
Triglycerides in very large HI	Lipoprotein subclasses	0.00 (-0.03, 0.03)	9.46E-01
Concentration of large HDL p	Lipoprotein subclasses	-0.04 (-0.07, 0.00)	1.48E-01
Total lipids in large HDL	Lipoprotein subclasses	-0.04 (-0.07, 0.00)	1.48E-01
Phospholipids in large HDL	Lipoprotein subclasses	-0.04 (-0.07, 0.00)	1.48E-01
Cholesterol in large HDL	Lipoprotein subclasses	-0.04 (-0.07, 0.00)	1.36E-01
Cholesteryl esters in large HD	Lipoprotein subclasses	-0.04 (-0.07, -0.01)	1.22E-01
Free cholesterol in large HDL	Lipoprotein subclasses	-0.04 (-0.07, 0.00)	1.73E-01
Triglycerides in large HDL	Lipoprotein subclasses	0.00 (-0.03, 0.04)	9.41E-01
**Concentration of medium H**	Lipoprotein subclasses	-0.05 (-0.08, -0.02)	**4.39E-02**
**Total lipids in medium HDL**	Lipoprotein subclasses	-0.05 (-0.08, -0.02)	**4.39E-02**
**Phospholipids in medium H**	Lipoprotein subclasses	-0.05 (-0.08, -0.02)	**4.52E-02**
**Cholesterol in medium HDL**	Lipoprotein subclasses	-0.05 (-0.09, -0.02)	**4.30E-02**
**Cholesteryl esters in mediu**	Lipoprotein subclasses	-0.05 (-0.09, -0.02)	**4.30E-02**
**Free cholesterol in medium**	Lipoprotein subclasses	-0.05 (-0.08, -0.02)	**4.39E-02**

Triglycerides in medium HDI	Lipoprotein subclasses	0.00 (-0.03, 0.04)	9.01E-01
Concentration of small HDL]	Lipoprotein subclasses	-0.04 (-0.07, -0.01)	1.12E-01
Total lipids in small HDL	Lipoprotein subclasses	-0.04 (-0.07, -0.01)	1.12E-01
Phospholipids in small HDL	Lipoprotein subclasses	-0.05 (-0.08, -0.01)	6.32E-02
Cholesterol in small HDL	Lipoprotein subclasses	-0.01 (-0.04, 0.02)	7.36E-01
Cholesteryl esters in small HI	Lipoprotein subclasses	0.00 (-0.03, 0.03)	9.65E-01
**Free cholesterol in small HI**	Lipoprotein subclasses	-0.05 (-0.09, -0.02)	**4.30E-02**
Triglycerides in small HDL	Lipoprotein subclasses	0.00 (-0.04, 0.03)	9.07E-01
Phospholipids to total lipids r	Relative lipoprotein li]	-0.02 (-0.05, 0.01)	4.32E-01
Cholesterol to total lipids rati	Relative lipoprotein li]	0.03 (-0.00, 0.06)	1.74E-01
Cholesteryl esters to total lipi	Relative lipoprotein li]	0.04 (-0.01, 0.07)	1.12E-01
Free cholesterol to total lipids	Relative lipoprotein li]	0.00 (-0.03, 0.03)	9.70E-01
Triglycerides to total lipids ra	Relative lipoprotein li]	-0.02 (-0.05, 0.01)	3.94E-01
Phospholipids to total lipids r	Relative lipoprotein li]	0.02 (-0.01, 0.06)	3.65E-01
**Cholesterol to total lipids ra**	Relative lipoprotein li]	0.07 (-0.04, 0.10)	**4.80E-03**
Cholesteryl esters to total lipi	Relative lipoprotein li]	0.05 (-0.02, 0.09)	**4.39E-02**
**Free cholesterol to total lipi**	Relative lipoprotein li]	0.05 (-0.01, 0.08)	6.32E-02
**Triglycerides to total lipids**	Relative lipoprotein li]	-0.07 (-0.10, -0.03)	**1.08E-02**
Phospholipids to total lipids r	Relative lipoprotein li]	0.01 (-0.03, 0.04)	8.94E-01
Cholesterol to total lipids rati	Relative lipoprotein li]	0.04 (-0.01, 0.07)	1.12E-01
Cholesteryl esters to total lipi	Relative lipoprotein li]	0.05 (-0.02, 0.08)	**4.88E-02**
Free cholesterol to total lipids	Relative lipoprotein li]	0.00 (-0.03, 0.04)	9.46E-01
Triglycerides to total lipids ra	Relative lipoprotein li]	-0.03 (-0.07, 0.00)	1.82E-01
Phospholipids to total lipids r	Relative lipoprotein li]	0.00 (-0.03, 0.04)	8.94E-01
**Cholesterol to total lipids ra**	Relative lipoprotein li]	0.05 (-0.02, 0.08)	**4.39E-02**
**Cholesteryl esters to total li**	Relative lipoprotein li]	0.06 (-0.02, 0.09)	**4.30E-02**
Free cholesterol to total lipids	Relative lipoprotein li]	-0.01 (-0.05, 0.02)	6.94E-01
Triglycerides to total lipids ra	Relative lipoprotein li]	-0.05 (-0.08, -0.01)	5.41E-02
**Phospholipids to total lipids**	Relative lipoprotein li]	-0.06 (-0.09, -0.03)	**3.39E-02**
Cholesterol to total lipids rati	Relative lipoprotein li]	0.04 (-0.01, 0.07)	1.12E-01
Cholesteryl esters to total lipi	Relative lipoprotein li]	0.05 (-0.02, 0.08)	5.41E-02
**Free cholesterol to total lipi**	Relative lipoprotein li]	-0.04 (-0.08, -0.01)	7.01E-02
Triglycerides to total lipids ra	Relative lipoprotein li]	-0.03 (-0.06, 0.01)	2.82E-01
Phospholipids to total lipids r	Relative lipoprotein li]	0.01 (-0.02, 0.04)	7.36E-01
Cholesterol to total lipids rati	Relative lipoprotein li]	0.04 (-0.01, 0.08)	7.41E-02
Cholesteryl esters to total lipi	Relative lipoprotein li]	0.05 (-0.01, 0.08)	5.41E-02
Free cholesterol to total lipids	Relative lipoprotein li]	0.01 (-0.02, 0.05)	6.67E-01
Triglycerides to total lipids ra	Relative lipoprotein li]	-0.04 (-0.07, -0.01)	1.12E-01
Phospholipids to total lipids r	Relative lipoprotein li]	0.02 (-0.01, 0.05)	3.94E-01
Cholesterol to total lipids rati	Relative lipoprotein li]	0.04 (-0.01, 0.07)	9.11E-02
Cholesteryl esters to total lipi	Relative lipoprotein li]	0.03 (-0.00, 0.06)	2.53E-01
Free cholesterol to total lipids	Relative lipoprotein li]	0.03 (-0.00, 0.06)	2.53E-01
Triglycerides to total lipids ra	Relative lipoprotein li]	-0.05 (-0.08, -0.01)	5.41E-02
Phospholipids to total lipids r	Relative lipoprotein li]	-0.03 (-0.06, 0.00)	2.36E-01
Cholesterol to total lipids rati	Relative lipoprotein li]	0.04 (-0.01, 0.08)	7.01E-02
Cholesteryl esters to total lipi	Relative lipoprotein li]	0.04 (-0.01, 0.07)	9.11E-02
Free cholesterol to total lipids	Relative lipoprotein li]	0.01 (-0.02, 0.05)	6.40E-01
Triglycerides to total lipids ra	Relative lipoprotein li]	-0.05 (-0.08, -0.01)	6.00E-02

Phospholipids to total lipids r	Relative lipoprotein li	-0.03 (-0.06, 0.00)	2.55E-01
Cholesterol to total lipids rati	Relative lipoprotein li	0.04 (-0.00, 0.07)	1.41E-01
Cholesteryl esters to total lipi	Relative lipoprotein li	0.04 (-0.00, 0.07)	1.39E-01
Free cholesterol to total lipids	Relative lipoprotein li	-0.03 (-0.07, 0.00)	1.82E-01
Triglycerides to total lipids ra	Relative lipoprotein li	-0.04 (-0.08, -0.01)	7.01E-02
Phospholipids to total lipids r	Relative lipoprotein li	-0.03 (-0.06, 0.00)	1.73E-01
Cholesterol to total lipids rati	Relative lipoprotein li	0.03 (-0.00, 0.06)	1.73E-01
Cholesteryl esters to total lipi	Relative lipoprotein li	0.03 (-0.00, 0.07)	1.48E-01
Free cholesterol to total lipids	Relative lipoprotein li	-0.04 (-0.07, 0.00)	1.41E-01
Triglycerides to total lipids ra	Relative lipoprotein li	-0.03 (-0.06, 0.00)	2.55E-01
Phospholipids to total lipids r	Relative lipoprotein li	0.01 (-0.02, 0.05)	7.14E-01
Cholesterol to total lipids rati	Relative lipoprotein li	-0.02 (-0.05, 0.02)	4.93E-01
Cholesteryl esters to total lipi	Relative lipoprotein li	-0.01 (-0.05, 0.02)	6.98E-01
Free cholesterol to total lipids	Relative lipoprotein li	-0.03 (-0.07, 0.00)	1.48E-01
Triglycerides to total lipids ra	Relative lipoprotein li	0.01 (-0.02, 0.05)	6.67E-01
Phospholipids to total lipids r	Relative lipoprotein li	0.01 (-0.02, 0.05)	6.67E-01
Cholesterol to total lipids rati	Relative lipoprotein li	-0.03 (-0.07, 0.00)	2.20E-01
Cholesteryl esters to total lipi	Relative lipoprotein li	-0.04 (-0.07, -0.01)	1.12E-01
Free cholesterol to total lipids	Relative lipoprotein li	-0.01 (-0.05, 0.02)	7.19E-01
Triglycerides to total lipids ra	Relative lipoprotein li	0.03 (-0.00, 0.07)	1.73E-01
Phospholipids to total lipids r	Relative lipoprotein li	0.01 (-0.02, 0.05)	6.16E-01
Cholesterol to total lipids rati	Relative lipoprotein li	-0.03 (-0.06, 0.00)	2.27E-01
Cholesteryl esters to total lipi	Relative lipoprotein li	-0.02 (-0.06, 0.01)	3.58E-01
Free cholesterol to total lipids	Relative lipoprotein li	-0.04 (-0.08, -0.01)	8.35E-02
Triglycerides to total lipids ra	Relative lipoprotein li	0.04 (-0.00, 0.07)	1.48E-01
Phospholipids to total lipids r	Relative lipoprotein li	-0.03 (-0.06, 0.01)	2.82E-01
Cholesterol to total lipids rati	Relative lipoprotein li	0.02 (-0.01, 0.05)	4.45E-01
Cholesteryl esters to total lipi	Relative lipoprotein li	0.02 (-0.01, 0.05)	3.51E-01
Free cholesterol to total lipids	Relative lipoprotein li	-0.04 (-0.08, -0.01)	7.01E-02
Triglycerides to total lipids ra	Relative lipoprotein li	0.01 (-0.02, 0.04)	7.20E-01
Average diameter for VLDL	Lipoprotein particle si	0.00 (-0.04, 0.03)	9.01E-01
Average diameter for LDL pa	Lipoprotein particle si	0.03 (-0.00, 0.07)	1.84E-01
Average diameter for HDL pa	Lipoprotein particle si	-0.02 (-0.05, 0.01)	4.45E-01
Total cholesterol	Cholesterol	0.01 (-0.02, 0.04)	7.31E-01
VLDL cholesterol	Cholesterol	0.02 (-0.02, 0.05)	4.84E-01
Remnant cholesterol (non-HI	Cholesterol	0.03 (-0.01, 0.06)	2.79E-01
LDL cholesterol	Cholesterol	0.03 (-0.01, 0.06)	2.82E-01
**HDL cholesterol**	Cholesterol	-0.05 (-0.08, -0.01)	5.41E-02
**HDL2 cholesterol**	Cholesterol	-0.05 (-0.08, -0.01)	5.41E-02
HDL3 cholesterol	Cholesterol	-0.03 (-0.07, 0.00)	1.48E-01
Total esterified cholesterol	Cholesteryl esters	0.01 (-0.03, 0.04)	8.23E-01
Total free cholesterol	Free cholesterol	0.01 (-0.02, 0.05)	6.89E-01
Total triglycerides	Triglycerides	-0.01 (-0.05, 0.02)	6.99E-01
Triglycerides in VLDL	Triglycerides	-0.01 (-0.05, 0.02)	7.19E-01
Triglycerides in LDL	Triglycerides	-0.02 (-0.05, 0.01)	4.68E-01
Triglycerides in HDL	Triglycerides	0.00 (-0.03, 0.03)	9.70E-01
Phosphoglycerides	Other lipids	-0.02 (-0.05, 0.01)	4.94E-01
Ratio of triglycerides to phos	Other lipids	0.00 (-0.03, 0.04)	9.41E-01

Phosphatidylcholines	Other lipids	-0.02 (-0.05, 0.01)	3.99E-01
Sphingomyelins	Other lipids	0.00 (-0.04, 0.03)	9.19E-01
Total cholines	Other lipids	-0.02 (-0.05, 0.01)	4.50E-01
Apolipoprotein A1	Apolipoproteins	-0.04 (-0.07, -0.01)	1.12E-01
Apolipoprotein B	Apolipoproteins	0.02 (-0.02, 0.05)	5.28E-01
Ratio of apolipoprotein B to a	Apolipoproteins	0.04 (-0.00, 0.07)	1.39E-01
Total fatty acids	Fatty acids	-0.01 (-0.05, 0.02)	7.10E-01
Degree of unsaturation	Fatty acids	0.02 (-0.01, 0.06)	3.51E-01
Docosahexaenoic acid	Fatty acids	0.02 (-0.02, 0.05)	5.18E-01
Linoleic acid	Fatty acids	0.00 (-0.04, 0.03)	9.02E-01
Omega-3 fatty acids	Fatty acids	0.01 (-0.02, 0.05)	6.67E-01
Omega-6 fatty acids	Fatty acids	-0.01 (-0.04, 0.03)	8.94E-01
Polyunsaturated fatty acids	Fatty acids	0.00 (-0.04, 0.03)	9.46E-01
Monounsaturated fatty acids	Fatty acids	-0.02 (-0.06, 0.01)	4.03E-01
Saturated fatty acids	Fatty acids	-0.01 (-0.04, 0.02)	7.36E-01
Ratio of docosahexaenoic aci	Fatty acids	0.03 (-0.00, 0.06)	2.27E-01
Ratio of linoleic acid to total	Fatty acids	0.01 (-0.02, 0.05)	6.89E-01
Ratio of omega-3 fatty acids t	Fatty acids	0.04 (-0.00, 0.07)	1.26E-01
Ratio of omega-6 fatty acids t	Fatty acids	0.01 (-0.02, 0.05)	6.75E-01
Ratio of polyunsaturated fatty	Fatty acids	0.03 (-0.01, 0.06)	3.07E-01
Ratio of monounsaturated fatt	Fatty acids	-0.03 (-0.07, 0.00)	1.82E-01
Ratio of saturated fatty acids	Fatty acids	0.00 (-0.03, 0.04)	9.31E-01
Glucose	Glycolysis related met	-0.01 (-0.05, 0.03)	8.10E-01
Lactate	Glycolysis related met	-0.01 (-0.04, 0.02)	8.14E-01
Citrate	Glycolysis related met	-0.01 (-0.05, 0.02)	6.72E-01
Alanine	Amino acids	0.00 (-0.03, 0.03)	9.43E-01
Glutamine	Amino acids	0.00 (-0.03, 0.03)	9.46E-01
Histidine	Amino acids	0.03 (-0.00, 0.06)	1.73E-01
Isoleucine	Amino acids	0.00 (-0.03, 0.04)	9.19E-01
Leucine	Amino acids	0.00 (-0.03, 0.04)	9.01E-01
Valine	Amino acids	0.00 (-0.03, 0.03)	9.97E-01
Phenylalanine	Amino acids	0.04 (-0.01, 0.07)	1.12E-01
Tyrosine	Amino acids	0.02 (-0.01, 0.05)	4.82E-01
Acetate	Ketone bodies	0.00 (-0.03, 0.03)	9.46E-01
Acetoacetate	Ketone bodies	-0.04 (-0.07, -0.01)	1.01E-01
**3-Hydroxybutyrate**	Ketone bodies	-0.05 (-0.08, -0.02)	**4.39E-02**
Creatinine	Fluid balance	-0.01 (-0.04, 0.02)	7.78E-01
Albumin	Fluid balance	-0.02 (-0.06, 0.01)	2.82E-01
Glycoprotein acetyls	Inflammation	0.00 (-0.03, 0.03)	9.78E-01
Acetone	Ketone bodies		
Cholesteryl esters in HDL	Cholesteryl esters		
Cholesteryl esters in LDL	Cholesteryl esters		
Cholesteryl esters in VLDL/ (	Cholesteryl esters		
Clinical LDL cholesterol	Cholesterol		
Concentration of HDL particl	Lipoprotein particle concentrations		
Concentration of LDL particl	Lipoprotein particle concentrations		
Concentration of VLDL parti	Lipoprotein particle concentrations		
Free cholesterol in HDL	Free cholesterol		

Free cholesterol in LDL	Free cholesterol
Free cholesterol in VLDL	Free cholesterol
Glycine	Amino acids
Phospholipids in HDL	Phospholipids
Phospholipids in LDL	Phospholipids
Phospholipids in VLDL	Phospholipids
Pyruvate	Glycolysis related metabolites
Ratio of omega-6 fatty acids t	Fatty acids
Ratio of polyunsaturated fatty	Fatty acids
Total cholesterol minus HDL	Cholesterol
Total concentration of branch	Amino acids
Total concentration of lipopro	Lipoprotein particle concentrations
Total lipids in HDL	Total lipids
Total lipids in LDL	Total lipids
Total lipids in lipoprotein par	Total lipids
Total lipids in VLDL	Total lipids
Total phospholipids in lipopro	Phospholipids

Models were adjusted for age, sex, regions (CKB only), ethnicity (UKB only), levels of education, smok

HR, hazard ratio; CI, confidence interval; LDL, Low Density Lipoprotein; HDL, High Density Lipopro

and all-cause and cause-specific mortality in the CKB and UKB after after adj

All causes mortality		Ischaemic heart diseases mortality	
**Adjusted HR (95% CI)**	**P value**	**Adjusted HR (95% CI)**	**P value**
0.92 (0.88, 0.97)	**3.44E-03**	0.97 (0.88, 1.05)	6.56E-01
0.92 (0.88, 0.97)	**4.80E-03**	0.97 (0.89, 1.06)	6.85E-01
0.93 (0.88, 0.98)	**9.56E-03**	0.98 (0.90, 1.07)	8.00E-01
0.96 (0.92, 1.01)	1.67E-01	1.00 (0.92, 1.09)	9.72E-01
0.98 (0.93, 1.03)	4.42E-01	1.03 (0.94, 1.12)	7.46E-01
0.94 (0.90, 0.99)	**3.55E-02**	0.97 (0.89, 1.06)	7.45E-01
0.91 (0.87, 0.96)	**1.22E-03**	0.96 (0.88, 1.05)	5.44E-01
0.94 (0.90, 0.99)	**3.55E-02**	0.97 (0.89, 1.06)	6.85E-01
0.94 (0.90, 0.99)	**4.25E-02**	0.97 (0.89, 1.06)	7.11E-01
0.95 (0.90, 1.00)	5.50E-02	0.98 (0.90, 1.07)	7.98E-01
0.96 (0.92, 1.01)	1.93E-01	1.00 (0.92, 1.09)	9.88E-01
0.97 (0.92, 1.02)	2.67E-01	1.01 (0.92, 1.10)	9.63E-01
0.96 (0.91, 1.01)	1.35E-01	0.99 (0.91, 1.08)	9.29E-01
0.94 (0.89, 0.99)	**2.37E-02**	0.96 (0.88, 1.05)	5.65E-01
0.94 (0.89, 0.98)	**2.10E-02**	0.98 (0.90, 1.07)	8.00E-01
0.94 (0.89, 0.98)	**2.12E-02**	0.98 (0.90, 1.07)	8.00E-01
0.94 (0.89, 0.98)	**2.13E-02**	0.98 (0.89, 1.07)	7.90E-01
0.94 (0.90, 0.99)	**2.79E-02**	0.98 (0.89, 1.07)	7.56E-01
0.94 (0.89, 0.99)	**2.34E-02**	0.97 (0.89, 1.06)	7.01E-01
0.94 (0.90, 0.99)	**3.55E-02**	0.98 (0.90, 1.07)	8.00E-01
0.94 (0.89, 0.98)	**1.96E-02**	0.98 (0.90, 1.07)	8.16E-01
0.94 (0.90, 0.99)	**2.48E-02**	1.00 (0.91, 1.09)	9.88E-01
0.94 (0.90, 0.99)	**2.77E-02**	1.00 (0.91, 1.09)	9.88E-01
0.94 (0.89, 0.98)	**2.12E-02**	0.99 (0.91, 1.09)	9.54E-01
0.95 (0.91, 1.00)	7.49E-02	1.00 (0.91, 1.09)	9.72E-01
0.96 (0.91, 1.00)	1.04E-01	0.99 (0.91, 1.08)	9.42E-01
0.95 (0.91, 1.00)	6.22E-02	1.00 (0.92, 1.09)	9.81E-01
0.94 (0.89, 0.98)	**2.12E-02**	1.00 (0.92, 1.09)	9.85E-01
0.95 (0.91, 1.00)	8.22E-02	1.02 (0.94, 1.12)	7.83E-01
0.96 (0.92, 1.01)	1.44E-01	1.04 (0.95, 1.13)	6.08E-01
0.94 (0.90, 0.99)	**2.98E-02**	1.01 (0.92, 1.10)	9.48E-01
1.02 (0.98, 1.07)	4.07E-01	1.16 (1.07, 1.26)	**1.58E-03**
1.04 (1.00, 1.09)	9.82E-02	1.19 (1.10, 1.29)	**1.04E-04**
0.98 (0.94, 1.03)	5.58E-01	1.08 (0.99, 1.17)	1.65E-01
0.93 (0.89, 0.98)	**1.08E-02**	0.97 (0.88, 1.06)	6.85E-01
1.03 (0.98, 1.07)	3.20E-01	1.11 (1.03, 1.21)	**2.26E-02**
1.04 (0.99, 1.08)	1.70E-01	1.13 (1.05, 1.22)	**6.10E-03**
1.00 (0.96, 1.05)	8.87E-01	1.04 (0.96, 1.13)	5.45E-01
1.08 (1.03, 1.12)	**1.31E-03**	1.23 (1.14, 1.32)	**5.37E-07**
1.08 (1.04, 1.13)	**6.25E-04**	1.24 (1.15, 1.34)	**1.11E-07**
1.06 (1.02, 1.11)	**1.13E-02**	1.18 (1.10, 1.28)	**5.02E-05**
0.97 (0.92, 1.01)	2.26E-01	0.98 (0.89, 1.07)	7.73E-01

1.00 (0.96, 1.05)	8.88E-01	1.03 (0.95, 1.11)	7.11E-01
1.01 (0.96, 1.05)	8.48E-01	1.03 (0.95, 1.12)	6.45E-01
1.00 (0.95, 1.04)	8.63E-01	1.02 (0.94, 1.10)	8.39E-01
1.01 (0.96, 1.05)	8.41E-01	1.04 (0.96, 1.13)	4.78E-01
1.00 (0.95, 1.04)	8.85E-01	1.04 (0.96, 1.13)	5.70E-01
1.03 (0.99, 1.07)	2.73E-01	1.06 (0.98, 1.15)	3.02E-01
1.02 (0.97, 1.06)	5.33E-01	0.98 (0.90, 1.07)	7.93E-01
0.99 (0.94, 1.03)	6.35E-01	1.02 (0.94, 1.11)	8.00E-01
0.99 (0.94, 1.03)	6.35E-01	1.02 (0.94, 1.11)	7.98E-01
0.97 (0.93, 1.01)	2.26E-01	1.00 (0.92, 1.08)	9.72E-01
0.99 (0.94, 1.03)	6.34E-01	1.03 (0.95, 1.12)	7.19E-01
0.98 (0.94, 1.03)	4.93E-01	1.03 (0.95, 1.11)	7.25E-01
1.00 (0.96, 1.05)	8.85E-01	1.03 (0.95, 1.12)	7.01E-01
1.04 (1.00, 1.09)	8.87E-02	1.01 (0.93, 1.10)	8.70E-01
0.96 (0.92, 1.01)	1.61E-01	1.00 (0.92, 1.09)	9.78E-01
0.96 (0.92, 1.01)	1.57E-01	1.00 (0.92, 1.09)	9.72E-01
0.91 (0.87, 0.96)	**5.02E-04**	0.92 (0.85, 1.00)	1.23E-01
0.97 (0.93, 1.02)	3.12E-01	1.03 (0.94, 1.12)	7.25E-01
0.98 (0.94, 1.02)	3.95E-01	1.04 (0.95, 1.12)	6.11E-01
0.95 (0.91, 1.00)	6.71E-02	0.98 (0.90, 1.07)	8.00E-01
1.02 (0.98, 1.07)	4.14E-01	0.98 (0.90, 1.07)	8.11E-01
0.96 (0.92, 1.01)	1.27E-01	1.02 (0.94, 1.11)	8.00E-01
0.96 (0.92, 1.01)	1.35E-01	1.02 (0.94, 1.11)	7.90E-01
0.91 (0.87, 0.95)	**1.46E-04**	0.93 (0.86, 1.01)	1.65E-01
0.98 (0.94, 1.02)	4.13E-01	1.05 (0.97, 1.14)	4.34E-01
0.98 (0.94, 1.03)	5.28E-01	1.06 (0.97, 1.15)	3.26E-01
0.95 (0.91, 1.00)	6.51E-02	1.01 (0.93, 1.10)	9.32E-01
0.98 (0.94, 1.03)	5.70E-01	1.00 (0.92, 1.09)	9.76E-01
1.19 (1.14, 1.24)	**3.72E-15**	1.25 (1.16, 1.35)	**1.32E-07**
1.19 (1.14, 1.24)	**5.17E-15**	1.26 (1.16, 1.36)	**1.02E-07**
1.19 (1.14, 1.24)	**7.70E-14**	1.17 (1.08, 1.27)	**5.54E-04**
1.18 (1.13, 1.23)	**4.57E-14**	1.33 (1.23, 1.44)	**4.81E-12**
1.18 (1.14, 1.23)	**2.83E-14**	1.37 (1.27, 1.48)	**2.93E-14**
1.18 (1.13, 1.23)	**4.69E-13**	1.24 (1.14, 1.34)	**7.87E-07**
1.12 (1.07, 1.16)	**6.94E-07**	1.16 (1.08, 1.25)	**4.16E-04**
1.14 (1.09, 1.19)	**5.56E-08**	1.09 (1.01, 1.19)	7.70E-02
1.14 (1.09, 1.19)	**4.40E-08**	1.10 (1.01, 1.19)	6.93E-02
1.13 (1.08, 1.18)	**9.06E-07**	1.09 (1.01, 1.19)	7.70E-02
1.15 (1.10, 1.20)	**1.05E-08**	1.10 (1.01, 1.20)	5.21E-02
1.14 (1.09, 1.19)	**2.86E-08**	1.09 (1.00, 1.19)	8.40E-02
1.16 (1.11, 1.21)	**3.75E-10**	1.14 (1.05, 1.24)	**5.95E-03**
1.12 (1.07, 1.17)	**2.46E-06**	1.01 (0.93, 1.10)	9.32E-01
0.89 (0.85, 0.93)	**8.76E-07**	0.81 (0.75, 0.89)	**1.20E-05**
0.89 (0.85, 0.93)	**1.87E-06**	0.82 (0.75, 0.89)	**2.33E-05**
0.91 (0.87, 0.95)	**2.60E-04**	0.85 (0.78, 0.92)	**5.29E-04**
0.89 (0.85, 0.93)	**2.02E-06**	0.83 (0.77, 0.91)	**7.31E-05**
0.88 (0.85, 0.92)	**3.21E-07**	0.83 (0.76, 0.90)	**3.37E-05**
0.92 (0.88, 0.96)	**1.17E-03**	0.86 (0.79, 0.94)	**1.84E-03**

0.86 (0.82, 0.90)	**1.24E-08**	0.74 (0.67, 0.82)	**1.57E-08**
0.81 (0.78, 0.85)	**1.16E-20**	0.76 (0.71, 0.81)	**2.93E-14**
0.82 (0.78, 0.85)	**5.94E-20**	0.76 (0.71, 0.82)	**3.30E-13**
0.84 (0.80, 0.88)	**4.69E-13**	0.79 (0.73, 0.86)	**1.11E-07**
0.90 (0.86, 0.94)	**5.98E-06**	0.86 (0.79, 0.93)	**5.01E-04**
0.92 (0.89, 0.96)	**9.53E-04**	0.89 (0.82, 0.96)	**1.11E-02**
0.86 (0.83, 0.90)	**1.27E-09**	0.82 (0.76, 0.89)	**8.13E-06**
0.89 (0.85, 0.94)	**1.32E-05**	0.80 (0.73, 0.88)	**2.67E-05**
0.93 (0.89, 0.97)	**4.88E-03**	0.98 (0.90, 1.07)	8.14E-01
1.16 (1.12, 1.21)	**2.29E-16**	1.15 (1.08, 1.22)	**1.35E-04**
1.17 (1.13, 1.21)	**2.57E-17**	1.16 (1.09, 1.24)	**2.09E-05**
1.06 (1.01, 1.11)	**2.85E-02**	1.02 (0.93, 1.11)	8.31E-01
0.88 (0.84, 0.92)	**1.22E-08**	0.88 (0.81, 0.95)	**2.34E-03**
0.94 (0.89, 0.98)	**1.32E-02**	0.99 (0.91, 1.09)	9.52E-01
1.10 (1.07, 1.14)	**1.39E-08**	1.13 (1.06, 1.19)	**1.33E-04**
1.12 (1.08, 1.16)	**3.88E-10**	1.15 (1.07, 1.22)	**1.31E-04**
1.02 (0.97, 1.07)	5.28E-01	1.05 (0.96, 1.14)	4.78E-01
0.93 (0.89, 0.98)	**6.65E-03**	0.88 (0.81, 0.96)	**9.17E-03**
0.94 (0.90, 0.99)	**2.82E-02**	0.89 (0.82, 0.97)	**2.22E-02**
0.99 (0.94, 1.03)	6.34E-01	0.91 (0.84, 0.98)	**4.78E-02**
1.03 (0.99, 1.08)	2.09E-01	0.92 (0.84, 1.00)	8.94E-02
0.95 (0.91, 1.00)	5.31E-02	0.96 (0.88, 1.04)	5.42E-01
1.03 (0.98, 1.07)	3.48E-01	1.11 (1.03, 1.20)	**2.43E-02**
0.96 (0.92, 1.00)	8.47E-02	0.82 (0.75, 0.89)	**4.71E-05**
1.05 (1.00, 1.10)	5.95E-02	0.92 (0.85, 1.01)	1.27E-01
1.05 (1.00, 1.09)	8.22E-02	0.91 (0.84, 1.00)	8.43E-02
1.01 (0.96, 1.06)	7.06E-01	1.03 (0.94, 1.12)	7.25E-01
0.96 (0.92, 1.01)	1.57E-01	1.11 (1.02, 1.21)	**4.02E-02**
0.94 (0.90, 0.99)	**2.37E-02**	0.81 (0.74, 0.89)	**3.06E-05**
1.12 (1.07, 1.17)	**2.51E-06**	1.25 (1.15, 1.36)	**2.39E-06**
1.11 (1.06, 1.16)	**2.57E-05**	1.23 (1.13, 1.34)	**1.20E-05**
1.15 (1.10, 1.20)	**3.02E-09**	1.25 (1.15, 1.35)	**4.86E-07**
0.90 (0.86, 0.95)	**3.81E-05**	0.84 (0.77, 0.91)	**2.02E-04**
0.94 (0.90, 0.98)	**4.88E-03**	0.85 (0.79, 0.91)	**2.76E-05**
1.15 (1.10, 1.21)	**1.11E-08**	1.49 (1.35, 1.64)	**1.49E-14**
1.13 (1.09, 1.19)	**1.94E-07**	1.40 (1.28, 1.53)	**1.01E-12**
1.11 (1.06, 1.16)	**8.56E-05**	1.31 (1.18, 1.44)	**8.77E-07**
0.93 (0.89, 0.97)	**3.62E-03**	0.83 (0.75, 0.91)	**2.02E-04**
0.95 (0.91, 1.00)	5.31E-02	0.90 (0.83, 0.97)	**2.22E-02**
1.00 (0.96, 1.05)	8.89E-01	1.09 (1.01, 1.19)	7.65E-02
0.95 (0.91, 0.99)	**2.34E-02**	1.03 (0.95, 1.12)	6.72E-01
1.07 (1.03, 1.13)	**7.34E-03**	1.11 (1.01, 1.22)	5.47E-02
1.01 (0.97, 1.06)	6.38E-01	0.95 (0.88, 1.03)	4.14E-01
0.97 (0.93, 1.02)	3.39E-01	0.95 (0.88, 1.04)	4.78E-01
0.98 (0.94, 1.02)	4.17E-01	1.01 (0.93, 1.10)	8.70E-01
0.95 (0.91, 0.99)	**3.55E-02**	0.99 (0.91, 1.07)	8.80E-01
1.10 (1.05, 1.16)	**4.23E-04**	1.09 (0.99, 1.19)	1.43E-01
1.05 (1.01, 1.09)	**2.99E-02**	1.02 (0.94, 1.10)	8.22E-01

0.96 (0.92, 1.01)	1.58E-01	0.90 (0.83, 0.99)	5.78E-02
1.00 (0.96, 1.05)	9.19E-01	1.07 (0.98, 1.17)	2.15E-01
0.99 (0.95, 1.04)	8.27E-01	1.05 (0.97, 1.14)	4.14E-01
1.03 (0.99, 1.08)	2.22E-01	1.00 (0.92, 1.09)	9.72E-01
1.06 (1.02, 1.11)	**1.08E-02**	1.00 (0.92, 1.09)	9.72E-01
0.97 (0.93, 1.01)	2.15E-01	0.89 (0.82, 0.97)	**2.64E-02**
1.01 (0.97, 1.06)	5.74E-01	1.09 (1.00, 1.19)	1.06E-01
1.01 (0.96, 1.05)	7.97E-01	1.07 (0.99, 1.17)	1.94E-01
1.03 (0.98, 1.08)	3.12E-01	1.00 (0.92, 1.09)	9.72E-01
1.02 (0.98, 1.07)	4.04E-01	0.99 (0.91, 1.07)	8.80E-01
1.00 (0.95, 1.05)	9.65E-01	0.80 (0.74, 0.87)	**1.26E-06**
1.02 (0.98, 1.07)	4.14E-01	1.37 (1.25, 1.50)	**8.21E-11**
1.02 (0.97, 1.07)	5.10E-01	1.38 (1.26, 1.50)	**7.07E-12**
1.03 (0.99, 1.07)	2.64E-01	1.13 (1.04, 1.23)	**1.59E-02**
0.95 (0.90, 0.99)	**3.47E-02**	0.90 (0.82, 0.99)	5.57E-02
0.86 (0.82, 0.90)	**4.12E-09**	0.90 (0.83, 0.99)	5.50E-02
1.15 (1.09, 1.21)	**2.66E-07**	1.17 (1.07, 1.29)	**3.09E-03**
1.09 (1.04, 1.14)	**1.14E-03**	1.03 (0.95, 1.12)	7.01E-01
1.20 (1.14, 1.26)	**5.81E-11**	1.40 (1.26, 1.55)	**7.49E-09**
1.00 (0.95, 1.04)	9.31E-01	0.91 (0.83, 0.99)	6.52E-02
1.17 (1.13, 1.21)	**2.57E-17**	1.18 (1.12, 1.25)	**1.11E-07**
0.93 (0.89, 0.97)	**1.18E-03**	0.95 (0.88, 1.03)	4.44E-01
0.92 (0.89, 0.96)	**4.65E-04**	0.96 (0.88, 1.04)	4.76E-01
0.98 (0.94, 1.02)	3.91E-01	0.97 (0.89, 1.05)	6.28E-01
0.95 (0.91, 1.00)	8.47E-02	0.86 (0.79, 0.95)	**6.10E-03**
0.97 (0.93, 1.02)	2.77E-01	0.98 (0.90, 1.07)	8.00E-01
1.03 (0.99, 1.08)	2.28E-01	1.04 (0.96, 1.13)	5.39E-01
1.02 (0.98, 1.07)	3.64E-01	1.03 (0.95, 1.12)	7.01E-01
1.04 (0.99, 1.08)	1.74E-01	1.08 (0.99, 1.17)	1.53E-01
0.97 (0.93, 1.02)	3.58E-01	0.91 (0.83, 0.99)	7.70E-02
0.91 (0.87, 0.96)	**5.02E-04**	0.95 (0.87, 1.04)	4.78E-01
1.10 (1.06, 1.15)	**6.23E-05**	0.97 (0.89, 1.06)	7.01E-01
1.21 (1.15, 1.26)	**9.57E-15**	1.25 (1.15, 1.36)	**1.57E-06**
1.02 (0.97, 1.06)	5.58E-01	1.08 (1.00, 1.17)	9.92E-02
1.00 (0.95, 1.04)	8.78E-01	1.11 (1.02, 1.20)	**4.51E-02**
1.00 (0.96, 1.05)	9.99E-01	1.09 (1.01, 1.19)	7.24E-02
0.99 (0.94, 1.03)	6.26E-01	1.04 (0.96, 1.13)	4.78E-01
1.07 (1.02, 1.12)	**7.47E-03**	1.06 (0.98, 1.15)	3.12E-01
1.06 (1.01, 1.10)	**2.85E-02**	1.03 (0.95, 1.12)	7.11E-01
1.15 (1.10, 1.20)	**1.39E-09**	1.31 (1.21, 1.41)	**2.32E-10**
0.92 (0.88, 0.96)	**8.93E-04**	0.85 (0.78, 0.93)	**1.14E-03**
0.93 (0.89, 0.97)	**5.01E-03**	0.91 (0.83, 0.99)	7.70E-02
0.95 (0.90, 0.99)	5.29E-02	0.99 (0.90, 1.08)	8.59E-01
0.94 (0.89, 0.98)	**2.12E-02**	0.99 (0.91, 1.08)	9.52E-01
1.03 (0.99, 1.08)	1.91E-01	1.01 (0.93, 1.10)	8.70E-01
0.98 (0.93, 1.02)	4.17E-01	0.88 (0.80, 0.96)	**1.68E-02**
0.98 (0.93, 1.02)	3.74E-01	0.97 (0.90, 1.06)	7.40E-01
0.95 (0.91, 1.00)	8.47E-02	1.01 (0.93, 1.11)	8.59E-01

0.89 (0.85, 0.93)	**1.56E-06**	0.80 (0.73, 0.87)	**2.71E-06**
0.86 (0.82, 0.90)	**5.79E-09**	0.80 (0.74, 0.88)	**4.86E-06**
0.95 (0.91, 1.00)	7.06E-02	0.91 (0.83, 0.99)	6.37E-02
1.06 (1.01, 1.10)	**2.85E-02**	1.09 (1.01, 1.18)	7.63E-02
0.97 (0.92, 1.01)	1.92E-01	1.04 (0.96, 1.13)	5.65E-01
0.94 (0.90, 0.98)	**2.10E-02**	0.99 (0.90, 1.08)	8.70E-01
0.91 (0.87, 0.96)	**5.68E-04**	0.90 (0.82, 0.99)	6.73E-02
0.81 (0.77, 0.84)	**8.24E-22**	0.73 (0.68, 0.78)	**5.69E-19**
1.08 (1.03, 1.13)	**1.16E-03**	1.42 (1.32, 1.52)	**4.57E-21**
0.89 (0.85, 0.94)	**1.51E-05**	0.86 (0.78, 0.94)	**3.01E-03**
0.98 (0.94, 1.03)	4.54E-01	1.19 (1.10, 1.28)	**1.15E-04**
0.88 (0.83, 0.92)	**6.71E-07**	0.81 (0.74, 0.89)	**4.84E-05**
0.89 (0.84, 0.93)	**8.80E-06**	0.87 (0.79, 0.95)	**8.04E-03**
0.92 (0.88, 0.97)	**3.44E-03**	0.94 (0.86, 1.03)	3.26E-01
0.93 (0.89, 0.98)	**1.08E-02**	0.92 (0.84, 1.00)	1.07E-01
1.19 (1.14, 1.24)	**1.84E-13**	1.75 (1.62, 1.89)	**1.03E-45**
0.91 (0.87, 0.95)	**6.14E-05**	0.85 (0.79, 0.91)	**3.69E-05**
1.06 (1.01, 1.10)	**2.98E-02**	1.43 (1.33, 1.54)	**2.58E-19**
0.91 (0.87, 0.95)	**5.65E-05**	0.78 (0.72, 0.85)	**2.52E-08**
0.92 (0.88, 0.96)	**6.35E-04**	0.87 (0.80, 0.95)	**3.74E-03**
1.01 (0.96, 1.05)	8.48E-01	1.10 (1.02, 1.20)	5.10E-02
1.14 (1.09, 1.19)	**6.25E-08**	1.10 (1.01, 1.20)	5.17E-02
1.03 (0.98, 1.07)	3.67E-01	1.04 (0.96, 1.13)	5.46E-01
0.95 (0.91, 1.00)	7.39E-02	1.12 (1.03, 1.20)	**1.28E-02**
0.99 (0.94, 1.04)	7.22E-01	0.80 (0.74, 0.88)	**7.96E-06**
0.90 (0.86, 0.94)	**2.04E-05**	1.00 (0.93, 1.09)	9.72E-01
0.93 (0.89, 0.97)	**2.74E-03**	0.99 (0.91, 1.08)	9.32E-01
0.98 (0.94, 1.03)	5.70E-01	1.20 (1.11, 1.29)	**1.20E-05**
0.90 (0.86, 0.95)	**2.19E-04**	0.96 (0.88, 1.05)	5.70E-01
0.93 (0.89, 0.98)	**7.41E-03**	1.10 (1.02, 1.20)	**4.96E-02**
0.80 (0.76, 0.84)	**2.57E-17**	0.80 (0.73, 0.88)	**2.76E-05**
1.11 (1.07, 1.14)	**1.74E-09**	1.18 (1.14, 1.23)	**9.38E-16**
1.05 (1.00, 1.10)	6.63E-02	1.11 (1.03, 1.21)	**2.53E-02**
1.07 (1.03, 1.11)	**6.13E-04**	1.09 (1.02, 1.16)	**2.71E-02**
0.99 (0.95, 1.04)	8.40E-01	0.80 (0.72, 0.90)	**4.16E-04**
1.02 (0.97, 1.06)	4.98E-01	0.88 (0.78, 0.99)	6.93E-02
1.03 (1.01, 1.06)	**2.21E-02**	1.04 (0.98, 1.09)	3.16E-01
0.84 (0.81, 0.87)	**2.12E-17**	0.74 (0.69, 0.78)	**4.31E-22**
1.12 (1.07, 1.17)	**6.63E-06**	1.35 (1.25, 1.47)	**9.93E-13**

‹ing status, alcohol consumption, and self-rated health status. P values are cc

tein; VLDL, Very Low Density Lipoprotein; IDL, Intermediate Density Lipoprotein..

justed additional covariates

Cerebrovascular diseases mortality		Cancer mortality	
**Adjusted HR (95% CI)**	**P value**	**Adjusted HR (95% CI)**	**P value**
0.84 (0.77, 0.93)	**2.76E-03**	0.92 (0.85, 1.01)	2.96E-01
0.85 (0.77, 0.94)	**3.17E-03**	0.93 (0.85, 1.01)	3.20E-01
0.85 (0.77, 0.94)	**5.18E-03**	0.93 (0.85, 1.01)	3.14E-01
0.88 (0.80, 0.97)	**1.97E-02**	0.97 (0.89, 1.05)	6.62E-01
0.89 (0.81, 0.98)	**3.78E-02**	0.98 (0.91, 1.07)	8.17E-01
0.87 (0.79, 0.96)	**9.69E-03**	0.96 (0.88, 1.04)	5.99E-01
0.84 (0.76, 0.93)	**1.79E-03**	0.92 (0.84, 1.00)	2.29E-01
0.87 (0.79, 0.96)	**9.69E-03**	0.97 (0.89, 1.06)	7.52E-01
0.87 (0.79, 0.96)	**1.01E-02**	0.97 (0.89, 1.06)	7.52E-01
0.86 (0.78, 0.95)	**8.52E-03**	0.97 (0.89, 1.06)	7.52E-01
0.89 (0.81, 0.98)	**2.80E-02**	0.98 (0.91, 1.07)	8.47E-01
0.90 (0.82, 0.99)	5.13E-02	0.98 (0.91, 1.07)	8.47E-01
0.87 (0.79, 0.96)	**1.37E-02**	0.99 (0.91, 1.07)	8.64E-01
0.86 (0.78, 0.95)	**8.86E-03**	0.97 (0.89, 1.06)	7.36E-01
0.87 (0.78, 0.95)	**9.69E-03**	0.94 (0.87, 1.03)	4.92E-01
0.87 (0.78, 0.95)	**9.69E-03**	0.94 (0.87, 1.03)	4.99E-01
0.86 (0.78, 0.95)	**9.63E-03**	0.95 (0.87, 1.03)	5.12E-01
0.86 (0.78, 0.95)	**9.63E-03**	0.96 (0.88, 1.04)	6.13E-01
0.86 (0.78, 0.95)	**6.27E-03**	0.97 (0.89, 1.05)	6.62E-01
0.87 (0.79, 0.96)	**1.39E-02**	0.95 (0.87, 1.04)	5.64E-01
0.87 (0.79, 0.96)	**1.01E-02**	0.94 (0.86, 1.02)	4.65E-01
0.87 (0.78, 0.96)	**9.69E-03**	0.94 (0.87, 1.02)	4.65E-01
0.87 (0.78, 0.96)	**9.69E-03**	0.94 (0.87, 1.02)	4.83E-01
0.86 (0.78, 0.95)	**7.94E-03**	0.94 (0.87, 1.02)	4.83E-01
0.87 (0.79, 0.96)	**1.12E-02**	0.97 (0.90, 1.05)	6.62E-01
0.86 (0.78, 0.95)	**9.69E-03**	0.99 (0.91, 1.07)	8.47E-01
0.88 (0.80, 0.97)	**1.85E-02**	0.95 (0.88, 1.03)	5.64E-01
0.87 (0.79, 0.96)	**1.11E-02**	0.93 (0.86, 1.01)	3.40E-01
0.87 (0.79, 0.97)	**1.65E-02**	0.96 (0.88, 1.03)	5.72E-01
0.88 (0.80, 0.98)	**2.71E-02**	0.96 (0.88, 1.03)	5.73E-01
0.86 (0.78, 0.95)	**9.69E-03**	0.95 (0.88, 1.03)	5.12E-01
0.98 (0.89, 1.08)	7.30E-01	0.97 (0.90, 1.04)	6.50E-01
1.02 (0.93, 1.11)	7.90E-01	0.98 (0.91, 1.05)	7.02E-01
0.92 (0.84, 1.02)	1.68E-01	0.96 (0.89, 1.04)	5.94E-01
0.85 (0.77, 0.94)	**4.47E-03**	0.96 (0.88, 1.04)	5.87E-01
0.99 (0.91, 1.09)	9.16E-01	0.98 (0.91, 1.05)	7.52E-01
1.01 (0.92, 1.11)	8.21E-01	0.98 (0.91, 1.05)	7.31E-01
0.96 (0.87, 1.05)	4.19E-01	1.00 (0.93, 1.07)	9.77E-01
1.10 (1.01, 1.20)	**4.84E-02**	0.96 (0.90, 1.03)	5.87E-01
1.11 (1.02, 1.21)	**2.84E-02**	0.96 (0.90, 1.03)	5.53E-01
1.07 (0.98, 1.17)	1.62E-01	0.97 (0.91, 1.04)	6.62E-01
0.90 (0.82, 1.00)	6.94E-02	0.99 (0.92, 1.07)	9.50E-01

0.96 (0.87, 1.05)	4.10E-01	1.00 (0.93, 1.07)	9.88E-01
0.96 (0.88, 1.05)	4.54E-01	1.00 (0.93, 1.07)	9.88E-01
0.95 (0.87, 1.04)	3.64E-01	1.00 (0.93, 1.07)	9.88E-01
0.96 (0.88, 1.05)	4.59E-01	1.00 (0.93, 1.07)	9.88E-01
0.94 (0.86, 1.03)	2.61E-01	0.99 (0.93, 1.07)	9.27E-01
1.01 (0.93, 1.10)	8.39E-01	1.01 (0.94, 1.08)	8.64E-01
0.99 (0.90, 1.09)	8.76E-01	1.03 (0.96, 1.11)	6.62E-01
0.94 (0.86, 1.03)	2.29E-01	0.99 (0.92, 1.06)	8.66E-01
0.94 (0.86, 1.03)	2.47E-01	0.99 (0.92, 1.06)	8.64E-01
0.90 (0.82, 0.99)	5.15E-02	0.99 (0.92, 1.06)	8.64E-01
0.94 (0.86, 1.03)	2.69E-01	0.98 (0.92, 1.06)	8.08E-01
0.93 (0.85, 1.02)	1.89E-01	0.98 (0.91, 1.05)	7.52E-01
0.98 (0.89, 1.07)	6.53E-01	1.00 (0.93, 1.07)	9.98E-01
1.03 (0.95, 1.13)	5.23E-01	1.04 (0.97, 1.12)	5.72E-01
0.92 (0.84, 1.01)	1.04E-01	0.97 (0.90, 1.04)	6.32E-01
0.92 (0.84, 1.01)	1.04E-01	0.96 (0.90, 1.04)	6.32E-01
0.85 (0.77, 0.92)	**7.20E-04**	0.96 (0.89, 1.04)	6.25E-01
0.94 (0.86, 1.02)	2.18E-01	0.96 (0.89, 1.03)	5.94E-01
0.94 (0.86, 1.03)	2.63E-01	0.96 (0.89, 1.03)	5.87E-01
0.91 (0.84, 1.00)	7.81E-02	0.97 (0.90, 1.04)	6.32E-01
1.02 (0.93, 1.11)	7.58E-01	1.03 (0.96, 1.11)	6.62E-01
0.92 (0.84, 1.01)	1.04E-01	0.95 (0.88, 1.02)	4.99E-01
0.92 (0.84, 1.01)	1.17E-01	0.95 (0.88, 1.02)	4.92E-01
0.85 (0.78, 0.92)	**5.83E-04**	0.95 (0.88, 1.03)	5.12E-01
0.95 (0.87, 1.04)	3.61E-01	0.95 (0.88, 1.02)	4.92E-01
0.96 (0.88, 1.05)	4.38E-01	0.95 (0.88, 1.02)	4.92E-01
0.92 (0.85, 1.01)	1.14E-01	0.95 (0.88, 1.02)	4.46E-01
0.94 (0.86, 1.04)	2.88E-01	0.99 (0.91, 1.07)	8.64E-01
1.28 (1.18, 1.39)	**1.19E-07**	1.10 (1.02, 1.17)	5.08E-02
1.28 (1.17, 1.39)	**1.28E-07**	1.09 (1.02, 1.17)	6.07E-02
1.26 (1.16, 1.37)	**1.13E-06**	1.14 (1.07, 1.23)	**1.73E-03**
1.28 (1.18, 1.39)	**1.35E-07**	1.04 (0.97, 1.11)	5.94E-01
1.29 (1.19, 1.41)	**5.48E-08**	1.02 (0.95, 1.09)	8.17E-01
1.24 (1.14, 1.35)	**4.42E-06**	1.10 (1.02, 1.17)	**4.78E-02**
1.13 (1.05, 1.22)	**5.42E-03**	1.04 (0.96, 1.12)	6.13E-01
1.19 (1.09, 1.30)	**4.58E-04**	1.12 (1.05, 1.21)	**1.19E-02**
1.19 (1.09, 1.30)	**3.75E-04**	1.12 (1.05, 1.21)	**1.19E-02**
1.17 (1.07, 1.27)	**1.84E-03**	1.11 (1.03, 1.20)	**2.45E-02**
1.21 (1.10, 1.32)	**1.44E-04**	1.13 (1.05, 1.21)	**9.75E-03**
1.20 (1.10, 1.31)	**2.93E-04**	1.13 (1.05, 1.22)	**8.88E-03**
1.23 (1.13, 1.35)	**1.54E-05**	1.12 (1.04, 1.21)	**1.33E-02**
1.16 (1.07, 1.27)	**1.26E-03**	1.12 (1.04, 1.20)	**1.55E-02**
0.81 (0.74, 0.88)	**2.25E-05**	1.02 (0.95, 1.10)	7.71E-01
0.81 (0.74, 0.89)	**4.28E-05**	1.02 (0.95, 1.10)	7.65E-01
0.85 (0.78, 0.94)	**2.24E-03**	1.02 (0.95, 1.10)	7.96E-01
0.82 (0.75, 0.89)	**5.14E-05**	1.02 (0.95, 1.10)	7.90E-01
0.81 (0.74, 0.88)	**1.26E-05**	1.02 (0.94, 1.09)	8.17E-01
0.87 (0.79, 0.95)	**4.86E-03**	1.03 (0.96, 1.11)	6.62E-01

0.74 (0.67, 0.82)	**8.51E-08**	1.03 (0.95, 1.11)	7.36E-01
0.76 (0.71, 0.81)	**1.80E-14**	0.97 (0.90, 1.05)	6.80E-01
0.76 (0.71, 0.81)	**5.78E-14**	0.97 (0.90, 1.05)	6.99E-01
0.77 (0.71, 0.84)	**4.39E-08**	0.96 (0.89, 1.04)	6.25E-01
0.85 (0.79, 0.93)	**5.78E-04**	1.01 (0.93, 1.08)	9.51E-01
0.89 (0.82, 0.96)	**9.69E-03**	1.01 (0.93, 1.09)	9.20E-01
0.81 (0.74, 0.88)	**3.42E-06**	0.98 (0.91, 1.06)	8.17E-01
0.82 (0.75, 0.91)	**5.22E-04**	0.99 (0.91, 1.07)	8.64E-01
0.95 (0.87, 1.04)	3.74E-01	0.86 (0.80, 0.92)	**2.11E-04**
1.19 (1.12, 1.27)	**1.06E-07**	1.15 (1.08, 1.23)	**3.32E-04**
1.22 (1.14, 1.29)	**1.84E-08**	1.15 (1.07, 1.22)	**5.28E-04**
1.06 (0.97, 1.16)	2.92E-01	1.07 (0.99, 1.16)	2.94E-01
0.84 (0.78, 0.91)	**3.28E-05**	0.93 (0.86, 1.00)	2.62E-01
0.85 (0.78, 0.93)	**8.87E-04**	0.93 (0.86, 1.01)	2.62E-01
1.16 (1.09, 1.23)	**3.04E-05**	1.05 (0.99, 1.11)	3.60E-01
1.22 (1.15, 1.30)	**2.24E-09**	1.05 (0.99, 1.11)	3.68E-01
0.93 (0.85, 1.02)	1.83E-01	1.02 (0.94, 1.11)	7.65E-01
0.95 (0.87, 1.04)	3.72E-01	0.99 (0.92, 1.06)	8.47E-01
0.92 (0.84, 1.00)	9.54E-02	0.95 (0.88, 1.04)	5.77E-01
0.96 (0.88, 1.05)	4.62E-01	1.05 (0.97, 1.14)	5.64E-01
1.00 (0.91, 1.10)	9.56E-01	1.15 (1.07, 1.23)	**1.73E-03**
0.95 (0.87, 1.04)	3.74E-01	0.91 (0.84, 0.98)	7.99E-02
1.05 (0.97, 1.15)	3.06E-01	0.97 (0.89, 1.06)	7.20E-01
0.90 (0.82, 0.98)	**4.02E-02**	1.11 (1.03, 1.19)	**2.92E-02**
1.01 (0.92, 1.11)	8.76E-01	1.16 (1.07, 1.25)	**1.23E-03**
1.00 (0.92, 1.10)	9.26E-01	1.16 (1.08, 1.24)	**6.95E-04**
1.01 (0.92, 1.11)	8.21E-01	0.97 (0.90, 1.05)	6.62E-01
1.01 (0.92, 1.11)	8.39E-01	0.86 (0.80, 0.93)	**7.12E-04**
0.94 (0.85, 1.03)	2.19E-01	1.05 (0.98, 1.13)	4.65E-01
1.19 (1.09, 1.31)	**7.20E-04**	1.02 (0.95, 1.10)	7.65E-01
1.16 (1.06, 1.28)	**3.39E-03**	1.02 (0.95, 1.09)	7.96E-01
1.25 (1.15, 1.36)	**1.45E-06**	1.03 (0.96, 1.11)	6.62E-01
0.86 (0.78, 0.94)	**2.77E-03**	0.97 (0.90, 1.04)	6.32E-01
0.88 (0.82, 0.95)	**3.17E-03**	1.09 (1.00, 1.18)	1.68E-01
1.34 (1.21, 1.48)	**2.16E-07**	0.93 (0.87, 1.00)	1.59E-01
1.28 (1.17, 1.41)	**2.21E-06**	0.93 (0.87, 0.99)	1.37E-01
1.20 (1.09, 1.33)	**9.66E-04**	0.97 (0.90, 1.04)	6.32E-01
0.88 (0.80, 0.97)	**1.97E-02**	1.02 (0.95, 1.10)	7.22E-01
0.98 (0.90, 1.07)	6.77E-01	1.00 (0.92, 1.07)	9.51E-01
0.99 (0.91, 1.07)	8.01E-01	0.97 (0.91, 1.04)	6.50E-01
0.89 (0.82, 0.97)	**1.53E-02**	0.94 (0.88, 1.00)	2.62E-01
1.12 (1.02, 1.24)	**3.29E-02**	1.03 (0.95, 1.11)	6.99E-01
1.02 (0.94, 1.11)	7.06E-01	1.03 (0.96, 1.11)	6.32E-01
0.94 (0.86, 1.04)	2.95E-01	1.00 (0.93, 1.09)	9.51E-01
0.98 (0.91, 1.07)	7.57E-01	0.97 (0.90, 1.04)	6.62E-01
0.93 (0.86, 1.01)	1.39E-01	0.95 (0.89, 1.02)	4.99E-01
1.21 (1.09, 1.34)	**9.66E-04**	1.06 (0.97, 1.15)	5.12E-01
1.07 (0.99, 1.15)	1.62E-01	1.04 (0.97, 1.12)	5.53E-01

0.93 (0.85, 1.02)	2.01E-01	1.02 (0.95, 1.10)	7.96E-01
1.01 (0.93, 1.10)	8.21E-01	0.97 (0.90, 1.04)	6.25E-01
0.99 (0.91, 1.08)	8.50E-01	0.97 (0.90, 1.04)	6.32E-01
1.07 (0.98, 1.16)	1.80E-01	1.02 (0.95, 1.11)	7.52E-01
1.09 (1.01, 1.18)	5.13E-02	1.06 (0.99, 1.14)	3.18E-01
0.94 (0.86, 1.03)	2.19E-01	1.04 (0.97, 1.12)	5.94E-01
1.04 (0.95, 1.14)	4.54E-01	0.96 (0.89, 1.03)	5.87E-01
1.02 (0.93, 1.11)	7.28E-01	0.96 (0.90, 1.04)	6.18E-01
1.07 (0.98, 1.17)	1.67E-01	1.02 (0.94, 1.10)	8.19E-01
1.02 (0.93, 1.11)	7.58E-01	1.04 (0.96, 1.11)	6.32E-01
0.95 (0.87, 1.05)	3.78E-01	1.21 (1.12, 1.31)	**1.54E-05**
1.10 (1.00, 1.21)	7.68E-02	0.83 (0.77, 0.89)	**3.01E-06**
1.11 (1.01, 1.22)	5.48E-02	0.80 (0.74, 0.86)	**8.97E-08**
1.03 (0.94, 1.12)	6.35E-01	0.99 (0.93, 1.06)	9.44E-01
0.90 (0.82, 0.99)	5.67E-02	0.97 (0.90, 1.05)	6.62E-01
0.81 (0.74, 0.89)	**8.87E-05**	0.89 (0.83, 0.97)	**3.26E-02**
1.26 (1.13, 1.40)	**1.53E-04**	1.07 (0.99, 1.16)	2.62E-01
1.12 (1.02, 1.24)	**3.15E-02**	1.09 (1.01, 1.18)	1.26E-01
1.40 (1.24, 1.57)	**2.16E-07**	1.04 (0.96, 1.12)	6.32E-01
0.98 (0.89, 1.07)	7.12E-01	1.03 (0.96, 1.11)	6.62E-01
1.26 (1.19, 1.32)	**1.48E-15**	1.00 (0.92, 1.09)	9.88E-01
0.88 (0.81, 0.95)	**3.02E-03**	0.98 (0.91, 1.06)	7.57E-01
0.87 (0.81, 0.94)	**1.26E-03**	0.97 (0.90, 1.05)	6.62E-01
0.96 (0.88, 1.05)	4.47E-01	1.02 (0.94, 1.10)	8.24E-01
0.89 (0.81, 0.99)	5.13E-02	1.03 (0.96, 1.11)	6.62E-01
0.96 (0.88, 1.05)	4.19E-01	0.97 (0.90, 1.05)	6.62E-01
1.05 (0.96, 1.14)	3.59E-01	1.02 (0.95, 1.10)	7.52E-01
1.04 (0.95, 1.13)	4.73E-01	1.02 (0.95, 1.10)	7.82E-01
1.07 (0.98, 1.16)	2.18E-01	1.00 (0.93, 1.08)	9.73E-01
0.95 (0.87, 1.05)	3.78E-01	1.01 (0.93, 1.09)	9.17E-01
0.89 (0.81, 0.97)	**2.19E-02**	0.88 (0.81, 0.96)	**1.66E-02**
1.10 (1.01, 1.20)	5.13E-02	1.18 (1.10, 1.28)	**2.01E-04**
1.34 (1.23, 1.47)	**6.38E-09**	1.09 (1.02, 1.18)	8.37E-02
0.97 (0.89, 1.06)	5.74E-01	1.00 (0.93, 1.07)	9.51E-01
0.93 (0.85, 1.03)	2.19E-01	0.95 (0.88, 1.03)	5.12E-01
0.94 (0.85, 1.03)	2.39E-01	0.97 (0.90, 1.04)	6.32E-01
0.95 (0.87, 1.04)	3.03E-01	0.97 (0.90, 1.04)	6.50E-01
1.08 (0.99, 1.18)	1.40E-01	1.08 (1.01, 1.16)	1.37E-01
1.06 (0.97, 1.16)	2.82E-01	1.09 (1.01, 1.17)	8.89E-02
1.24 (1.13, 1.35)	**1.00E-05**	1.01 (0.94, 1.08)	8.70E-01
0.83 (0.76, 0.92)	**6.65E-04**	1.01 (0.94, 1.08)	9.27E-01
0.83 (0.75, 0.91)	**5.83E-04**	1.00 (0.92, 1.07)	9.51E-01
0.88 (0.80, 0.97)	**2.15E-02**	0.95 (0.88, 1.03)	5.53E-01
0.87 (0.79, 0.96)	**1.11E-02**	0.93 (0.86, 1.02)	3.68E-01
1.02 (0.93, 1.12)	7.10E-01	1.03 (0.96, 1.11)	6.62E-01
0.93 (0.85, 1.02)	1.86E-01	1.05 (0.97, 1.13)	5.23E-01
0.88 (0.80, 0.96)	**9.69E-03**	1.05 (0.97, 1.13)	5.50E-01
0.90 (0.82, 0.99)	5.48E-02	0.94 (0.87, 1.02)	4.83E-01

0.78 (0.71, 0.85)	**8.04E-07**	1.03 (0.96, 1.11)	6.62E-01
0.77 (0.70, 0.84)	**1.28E-07**	0.97 (0.90, 1.05)	6.62E-01
0.86 (0.79, 0.94)	**3.18E-03**	1.05 (0.97, 1.13)	5.53E-01
1.03 (0.95, 1.13)	5.17E-01	1.05 (0.98, 1.13)	4.72E-01
0.88 (0.80, 0.97)	**1.97E-02**	0.96 (0.89, 1.03)	5.87E-01
0.87 (0.78, 0.96)	**1.01E-02**	0.94 (0.87, 1.01)	3.23E-01
0.78 (0.71, 0.86)	**9.53E-06**	1.01 (0.93, 1.09)	9.45E-01
0.76 (0.71, 0.81)	**1.01E-13**	1.01 (0.93, 1.09)	9.02E-01
1.12 (1.03, 1.22)	**1.90E-02**	0.88 (0.82, 0.95)	**4.24E-03**
0.79 (0.72, 0.87)	**5.57E-06**	1.00 (0.92, 1.08)	9.88E-01
0.94 (0.85, 1.03)	2.61E-01	0.89 (0.82, 0.96)	**1.97E-02**
0.75 (0.68, 0.82)	**1.04E-07**	1.02 (0.94, 1.10)	8.07E-01
0.76 (0.69, 0.85)	**1.37E-06**	0.99 (0.92, 1.07)	9.27E-01
0.82 (0.74, 0.91)	**5.78E-04**	0.98 (0.91, 1.06)	7.96E-01
0.78 (0.71, 0.87)	**1.24E-05**	1.04 (0.97, 1.13)	5.87E-01
1.43 (1.31, 1.56)	**2.18E-13**	0.83 (0.77, 0.89)	**5.47E-06**
0.91 (0.84, 0.98)	**2.51E-02**	1.00 (0.92, 1.09)	9.97E-01
1.16 (1.07, 1.27)	**2.25E-03**	0.80 (0.74, 0.86)	**1.18E-07**
0.88 (0.81, 0.96)	**9.69E-03**	1.06 (0.98, 1.14)	4.87E-01
0.92 (0.84, 1.00)	8.87E-02	0.98 (0.90, 1.06)	7.82E-01
1.05 (0.96, 1.14)	3.78E-01	0.90 (0.83, 0.98)	5.08E-02
1.09 (0.99, 1.19)	1.04E-01	1.18 (1.09, 1.27)	**3.32E-04**
1.18 (1.08, 1.28)	**6.27E-04**	0.84 (0.76, 0.92)	**2.36E-03**
1.03 (0.95, 1.12)	5.35E-01	0.77 (0.70, 0.84)	**9.59E-08**
0.84 (0.76, 0.93)	**1.72E-03**	1.26 (1.17, 1.36)	**8.97E-08**
0.94 (0.86, 1.02)	2.01E-01	0.79 (0.73, 0.85)	**1.34E-07**
1.05 (0.96, 1.14)	3.74E-01	0.80 (0.74, 0.87)	**9.92E-07**
1.11 (1.02, 1.21)	**2.19E-02**	0.74 (0.69, 0.81)	**6.77E-11**
0.88 (0.79, 0.97)	**1.64E-02**	0.88 (0.81, 0.95)	**1.26E-02**
0.92 (0.84, 1.02)	1.58E-01	0.81 (0.74, 0.88)	**2.74E-05**
0.73 (0.66, 0.81)	**5.11E-08**	0.81 (0.75, 0.89)	**4.33E-05**
1.13 (1.08, 1.18)	**2.77E-07**	0.85 (0.78, 0.92)	**1.04E-03**
1.14 (1.04, 1.24)	**8.86E-03**	0.88 (0.81, 0.95)	**1.03E-02**
1.16 (1.10, 1.22)	**1.04E-07**	0.94 (0.87, 1.02)	4.83E-01
0.95 (0.87, 1.05)	3.95E-01	1.13 (1.07, 1.19)	**6.13E-05**
0.98 (0.89, 1.08)	7.58E-01	1.08 (1.02, 1.15)	**2.92E-02**
1.05 (1.01, 1.09)	**2.43E-02**	0.79 (0.67, 0.94)	**3.32E-02**
0.75 (0.70, 0.80)	**8.47E-16**	1.12 (1.04, 1.20)	**2.45E-02**
1.09 (1.00, 1.19)	1.00E-01	0.99 (0.91, 1.07)	8.95E-01

UKB			
Other mortality		Sleep duration	
Adjusted HR (95% CI)	P value	Adjusted β (95% CI)	P value
1.04 (0.89, 1.20)	8.57E-01	0.03 (0.02, 0.03)	**5.95E-27**
1.04 (0.90, 1.20)	8.54E-01	0.02 (0.02, 0.03)	**6.56E-25**
1.04 (0.90, 1.21)	8.17E-01	0.03 (0.02, 0.03)	**8.91E-28**
1.08 (0.93, 1.24)	6.05E-01	0.02 (0.02, 0.03)	**2.15E-25**
1.08 (0.94, 1.25)	5.90E-01	0.02 (0.02, 0.03)	**4.05E-23**
1.07 (0.92, 1.23)	6.61E-01	0.03 (0.02, 0.03)	**1.68E-27**
1.03 (0.89, 1.19)	8.92E-01	0.02 (0.02, 0.03)	**2.32E-23**
1.03 (0.89, 1.20)	8.57E-01	0.02 (0.02, 0.03)	**3.37E-22**
1.04 (0.89, 1.20)	8.57E-01	0.02 (0.02, 0.03)	**1.24E-21**
1.05 (0.91, 1.22)	7.59E-01	0.02 (0.02, 0.03)	**1.43E-22**
1.04 (0.90, 1.21)	8.17E-01	0.02 (0.01, 0.02)	**2.05E-16**
1.04 (0.89, 1.20)	8.57E-01	0.01 (0.01, 0.02)	**5.71E-11**
1.05 (0.91, 1.22)	7.59E-01	0.02 (0.02, 0.03)	**4.25E-22**
1.03 (0.89, 1.20)	8.80E-01	0.02 (0.02, 0.03)	**3.48E-22**
1.03 (0.89, 1.20)	8.57E-01	0.02 (0.02, 0.03)	**9.17E-20**
1.04 (0.89, 1.20)	8.57E-01	0.02 (0.02, 0.02)	**2.22E-17**
1.04 (0.90, 1.21)	8.18E-01	0.02 (0.02, 0.03)	**6.18E-21**
1.03 (0.89, 1.20)	8.57E-01	0.02 (0.01, 0.02)	**2.20E-16**
1.02 (0.87, 1.18)	9.53E-01	0.02 (0.01, 0.02)	**7.09E-14**
1.05 (0.91, 1.22)	7.60E-01	0.02 (0.02, 0.02)	**3.02E-18**
1.03 (0.89, 1.20)	8.57E-01	0.02 (0.01, 0.02)	**1.78E-15**
1.00 (0.86, 1.17)	9.80E-01	0.01 (0.00, 0.01)	**1.81E-03**
1.00 (0.86, 1.17)	9.80E-01	0.01 (0.01, 0.01)	**8.86E-06**
1.00 (0.86, 1.17)	9.80E-01	0.01 (0.00, 0.01)	**4.00E-03**
1.01 (0.87, 1.17)	9.74E-01	0.00 (-0.01, 0.00)	1.72E-01
1.00 (0.86, 1.15)	9.80E-01	-0.01 (-0.01, 0.00)	**1.79E-04**
1.02 (0.88, 1.19)	9.17E-01	0.00 (0.00, 0.01)	8.96E-02
1.00 (0.86, 1.17)	9.80E-01	0.02 (0.01, 0.02)	**3.42E-14**
0.98 (0.84, 1.14)	9.17E-01	0.02 (0.01, 0.02)	**6.19E-18**
0.97 (0.84, 1.13)	8.96E-01	0.02 (0.01, 0.02)	**5.32E-15**
0.96 (0.83, 1.12)	8.57E-01	0.01 (0.00, 0.01)	**3.07E-05**
0.94 (0.82, 1.09)	7.20E-01	0.01 (0.00, 0.01)	**3.84E-04**
0.93 (0.81, 1.07)	6.05E-01	0.01 (0.01, 0.02)	**1.15E-06**
0.98 (0.84, 1.13)	9.12E-01	0.00 (0.00, 0.01)	2.33E-01
1.00 (0.86, 1.17)	9.80E-01	0.03 (0.02, 0.03)	**3.46E-32**
1.00 (0.87, 1.15)	9.80E-01	0.01 (0.01, 0.02)	**4.93E-09**
0.99 (0.87, 1.14)	9.76E-01	0.01 (0.01, 0.02)	**3.53E-11**
0.98 (0.86, 1.13)	9.17E-01	0.02 (0.01, 0.02)	**1.59E-16**
0.98 (0.86, 1.12)	9.24E-01	0.00 (0.00, 0.01)	6.87E-02
0.99 (0.87, 1.13)	9.55E-01	0.00 (0.00, 0.01)	7.57E-01
0.98 (0.86, 1.12)	9.04E-01	0.01 (0.01, 0.02)	**1.41E-07**
1.03 (0.90, 1.19)	8.57E-01	0.03 (0.02, 0.03)	**2.92E-38**

0.99 (0.87, 1.14)	9.74E-01	-0.01 (-0.01, 0.00)	**6.44E-05**
0.98 (0.86, 1.13)	9.33E-01	-0.01 (-0.02, -0.01)	**2.74E-08**
0.96 (0.84, 1.10)	8.17E-01	-0.01 (-0.01, 0.00)	**5.31E-04**
0.96 (0.84, 1.10)	8.02E-01	-0.02 (-0.02, -0.01)	**3.22E-15**
0.95 (0.83, 1.09)	7.59E-01	-0.02 (-0.02, -0.01)	**5.30E-16**
0.97 (0.85, 1.11)	8.61E-01	-0.02 (-0.02, -0.01)	**1.98E-12**
1.14 (1.00, 1.29)	2.07E-01	0.02 (0.02, 0.03)	**1.06E-27**
0.95 (0.83, 1.09)	7.59E-01	0.00 (-0.01, 0.00)	6.51E-02
0.94 (0.82, 1.09)	7.20E-01	-0.02 (-0.02, -0.01)	**1.51E-11**
0.92 (0.80, 1.06)	5.80E-01	-0.02 (-0.02, -0.01)	**2.77E-13**
0.93 (0.81, 1.07)	6.05E-01	-0.02 (-0.02, -0.01)	**1.46E-14**
0.93 (0.81, 1.07)	5.89E-01	-0.02 (-0.02, -0.01)	**5.72E-12**
0.94 (0.82, 1.08)	7.09E-01	-0.02 (-0.03, -0.02)	**3.34E-22**
1.15 (1.01, 1.30)	1.95E-01	0.02 (0.02, 0.02)	**1.64E-18**
0.91 (0.79, 1.05)	4.54E-01	0.00 (-0.01, 0.00)	3.14E-01
0.90 (0.78, 1.04)	4.07E-01	-0.01 (-0.01, 0.00)	**5.00E-05**
0.89 (0.77, 1.02)	3.15E-01	-0.01 (-0.02, -0.01)	**6.71E-07**
0.90 (0.78, 1.03)	3.65E-01	-0.01 (-0.01, -0.01)	**2.23E-06**
0.90 (0.78, 1.04)	3.76E-01	-0.01 (-0.01, 0.00)	**1.91E-03**
0.88 (0.77, 1.01)	3.00E-01	-0.02 (-0.02, -0.02)	**1.02E-18**
1.11 (0.98, 1.27)	3.15E-01	0.02 (0.02, 0.03)	**5.91E-20**
0.89 (0.77, 1.02)	3.11E-01	0.00 (0.00, 0.00)	7.54E-01
0.88 (0.77, 1.02)	3.00E-01	-0.01 (-0.01, 0.00)	**3.98E-03**
0.86 (0.75, 0.99)	2.06E-01	-0.01 (-0.01, 0.00)	**9.74E-04**
0.88 (0.77, 1.02)	3.00E-01	-0.01 (-0.01, -0.01)	**2.59E-05**
0.89 (0.77, 1.02)	3.15E-01	-0.01 (-0.01, 0.00)	**1.41E-02**
0.87 (0.75, 0.99)	2.06E-01	-0.02 (-0.02, -0.01)	**4.74E-17**
1.04 (0.91, 1.20)	8.07E-01	0.02 (0.02, 0.03)	**1.61E-26**
1.12 (0.98, 1.28)	3.11E-01	-0.01 (-0.01, 0.00)	**1.44E-02**
1.11 (0.97, 1.27)	3.38E-01	-0.01 (-0.01, 0.00)	**1.04E-02**
1.14 (0.99, 1.31)	2.47E-01	0.00 (-0.01, 0.00)	6.38E-02
1.06 (0.93, 1.21)	6.43E-01	-0.01 (-0.02, -0.01)	**5.53E-06**
1.06 (0.93, 1.20)	7.20E-01	-0.01 (-0.02, -0.01)	**5.14E-08**
1.08 (0.95, 1.24)	5.20E-01	0.00 (-0.01, 0.00)	2.87E-01
1.21 (1.07, 1.37)	**3.81E-02**	0.03 (0.02, 0.03)	**3.65E-30**
1.15 (1.00, 1.33)	2.06E-01	-0.02 (-0.02, -0.01)	**2.77E-13**
1.15 (1.00, 1.32)	2.21E-01	-0.02 (-0.02, -0.01)	**6.71E-13**
1.12 (0.98, 1.29)	3.15E-01	-0.02 (-0.02, -0.01)	**9.65E-12**
1.15 (1.00, 1.32)	2.21E-01	-0.02 (-0.03, -0.02)	**4.70E-17**
1.16 (1.01, 1.33)	2.06E-01	-0.02 (-0.03, -0.02)	**4.51E-19**
1.13 (0.98, 1.31)	3.00E-01	-0.02 (-0.02, -0.01)	**1.07E-10**
1.29 (1.14, 1.47)	**6.81E-03**	0.02 (0.01, 0.02)	**7.28E-18**
0.90 (0.78, 1.04)	3.83E-01	-0.03 (-0.03, -0.02)	**7.69E-27**
0.90 (0.78, 1.03)	3.68E-01	-0.02 (-0.03, -0.02)	**5.80E-22**
0.94 (0.81, 1.08)	6.76E-01	-0.02 (-0.02, -0.01)	**1.89E-15**
0.86 (0.75, 0.99)	2.06E-01	-0.03 (-0.04, -0.03)	**1.74E-38**
0.85 (0.74, 0.98)	1.88E-01	-0.03 (-0.04, -0.03)	**8.08E-42**
0.91 (0.79, 1.05)	4.76E-01	-0.03 (-0.03, -0.02)	**6.56E-25**

1.01 (0.87, 1.17)	9.76E-01	0.02 (0.02, 0.03)	**3.48E-22**
0.81 (0.71, 0.93)	**3.50E-02**	-0.03 (-0.03, -0.02)	**7.15E-35**
0.80 (0.71, 0.92)	**2.44E-02**	-0.02 (-0.02, -0.01)	**5.02E-17**
0.87 (0.76, 1.00)	2.45E-01	-0.02 (-0.02, -0.01)	**4.67E-13**
0.82 (0.72, 0.94)	**4.55E-02**	-0.03 (-0.03, -0.03)	**4.28E-42**
0.85 (0.74, 0.97)	1.46E-01	-0.03 (-0.04, -0.03)	**3.54E-50**
0.85 (0.74, 0.97)	1.78E-01	-0.02 (-0.02, -0.01)	**3.99E-15**
1.05 (0.91, 1.22)	7.59E-01	0.03 (0.02, 0.03)	**1.62E-28**
1.09 (0.93, 1.28)	5.93E-01	0.02 (0.01, 0.02)	**1.33E-14**
1.14 (1.01, 1.29)	2.03E-01	-0.01 (-0.02, -0.01)	**2.06E-07**
1.11 (0.97, 1.26)	3.68E-01	-0.01 (-0.02, -0.01)	**2.15E-07**
1.16 (1.02, 1.31)	1.88E-01	-0.01 (-0.01, 0.00)	**1.73E-04**
0.86 (0.75, 0.98)	1.79E-01	0.01 (0.00, 0.01)	**1.78E-02**
1.09 (0.93, 1.28)	5.89E-01	-0.02 (-0.02, -0.01)	**8.13E-16**
1.08 (0.96, 1.21)	4.91E-01	-0.02 (-0.03, -0.02)	**2.71E-19**
1.02 (0.89, 1.17)	9.24E-01	0.00 (-0.01, 0.00)	5.79E-02
1.12 (0.99, 1.27)	2.56E-01	0.01 (0.01, 0.02)	**4.08E-08**
0.89 (0.78, 1.03)	3.52E-01	0.03 (0.02, 0.03)	**5.07E-29**
1.15 (0.98, 1.36)	3.04E-01	0.00 (-0.01, 0.00)	2.49E-01
1.06 (0.91, 1.24)	7.20E-01	-0.01 (-0.01, 0.00)	**3.75E-04**
0.98 (0.85, 1.13)	9.24E-01	0.01 (0.01, 0.01)	**2.31E-06**
1.11 (0.95, 1.30)	4.91E-01	-0.01 (-0.02, -0.01)	**1.65E-07**
0.92 (0.79, 1.07)	5.89E-01	-0.02 (-0.02, -0.01)	**2.05E-15**
0.95 (0.82, 1.09)	7.27E-01	-0.03 (-0.03, -0.02)	**2.20E-32**
1.07 (0.93, 1.23)	6.61E-01	-0.03 (-0.04, -0.03)	**7.44E-36**
1.02 (0.89, 1.18)	9.16E-01	-0.02 (-0.03, -0.02)	**2.06E-21**
1.14 (0.98, 1.33)	3.00E-01	0.03 (0.02, 0.03)	**4.85E-29**
0.95 (0.82, 1.09)	7.59E-01	-0.04 (-0.04, -0.03)	**1.99E-56**
0.97 (0.85, 1.12)	8.92E-01	-0.03 (-0.03, -0.02)	**1.02E-28**
0.94 (0.82, 1.08)	6.76E-01	-0.01 (-0.02, -0.01)	**1.65E-10**
0.94 (0.82, 1.07)	6.11E-01	-0.04 (-0.04, -0.03)	**6.51E-57**
1.02 (0.89, 1.17)	9.17E-01	0.03 (0.03, 0.03)	**2.37E-37**
1.07 (0.93, 1.23)	6.17E-01	0.04 (0.03, 0.04)	**4.06E-61**
0.95 (0.83, 1.09)	7.59E-01	-0.04 (-0.04, -0.03)	**1.01E-54**
0.98 (0.85, 1.12)	9.12E-01	-0.04 (-0.04, -0.04)	**5.88E-58**
0.99 (0.87, 1.14)	9.74E-01	-0.02 (-0.02, -0.01)	**1.17E-17**
0.95 (0.84, 1.09)	7.59E-01	0.03 (0.03, 0.04)	**1.33E-41**
1.04 (0.91, 1.19)	8.02E-01	0.03 (0.03, 0.04)	**8.18E-46**
0.92 (0.80, 1.06)	5.19E-01	-0.05 (-0.05, -0.04)	**6.65E-82**
0.90 (0.80, 1.00)	2.34E-01	-0.04 (-0.05, -0.04)	**4.42E-79**
0.88 (0.78, 0.99)	1.95E-01	-0.02 (-0.02, -0.02)	**3.73E-20**
0.96 (0.84, 1.10)	8.17E-01	0.04 (0.04, 0.05)	**3.03E-70**
1.14 (1.02, 1.28)	1.78E-01	0.00 (-0.01, 0.00)	1.22E-01
1.05 (0.92, 1.20)	7.40E-01	-0.04 (-0.04, -0.03)	**2.26E-51**
0.88 (0.79, 0.98)	1.87E-01	-0.01 (-0.02, -0.01)	**4.58E-09**
0.87 (0.78, 0.97)	1.50E-01	-0.04 (-0.04, -0.03)	**3.48E-56**
1.01 (0.87, 1.17)	9.74E-01	0.04 (0.03, 0.04)	**3.37E-60**
1.17 (1.06, 1.31)	**3.81E-02**	-0.01 (-0.02, -0.01)	**3.58E-09**

1.08 (0.95, 1.23)	5.24E-01	-0.03 (-0.03, -0.02)	**9.73E-30**
0.89 (0.79, 1.00)	2.31E-01	0.01 (0.01, 0.01)	**1.05E-05**
0.89 (0.79, 1.01)	2.88E-01	-0.04 (-0.04, -0.03)	**2.51E-52**
1.08 (0.94, 1.24)	5.80E-01	0.04 (0.03, 0.04)	**1.17E-54**
1.20 (1.07, 1.34)	**3.50E-02**	0.00 (-0.01, 0.00)	6.23E-01
1.09 (0.95, 1.24)	4.95E-01	-0.03 (-0.03, -0.02)	**2.53E-38**
0.90 (0.79, 1.02)	3.01E-01	0.00 (0.00, 0.01)	4.31E-01
0.90 (0.79, 1.02)	3.28E-01	-0.03 (-0.04, -0.03)	**2.64E-45**
1.06 (0.92, 1.22)	7.26E-01	0.04 (0.03, 0.04)	**8.47E-53**
1.16 (1.02, 1.31)	1.78E-01	0.01 (0.01, 0.02)	**2.01E-07**
1.10 (0.95, 1.27)	4.60E-01	-0.03 (-0.03, -0.02)	**4.51E-32**
0.86 (0.75, 0.98)	1.82E-01	-0.04 (-0.04, -0.03)	**2.64E-59**
0.86 (0.74, 0.99)	1.95E-01	0.00 (-0.01, 0.00)	8.97E-01
0.90 (0.80, 1.01)	2.63E-01	0.02 (0.01, 0.02)	**2.32E-11**
1.14 (1.00, 1.28)	2.06E-01	0.02 (0.01, 0.02)	**3.96E-11**
0.76 (0.66, 0.89)	**2.29E-02**	-0.03 (-0.03, -0.02)	**2.15E-25**
1.09 (0.94, 1.27)	5.80E-01	-0.03 (-0.03, -0.02)	**7.90E-34**
1.14 (0.98, 1.32)	3.00E-01	0.00 (0.00, 0.01)	4.13E-01
1.00 (0.87, 1.16)	9.95E-01	0.03 (0.02, 0.03)	**3.64E-27**
1.22 (1.09, 1.38)	**2.44E-02**	0.04 (0.04, 0.05)	**5.16E-66**
1.22 (1.11, 1.34)	**5.64E-03**	-0.04 (-0.05, -0.04)	**3.92E-68**
0.83 (0.75, 0.93)	**2.44E-02**	-0.04 (-0.04, -0.04)	**1.56E-65**
0.83 (0.75, 0.92)	**2.29E-02**	-0.02 (-0.03, -0.02)	**1.09E-21**
0.92 (0.80, 1.06)	5.24E-01	0.04 (0.03, 0.04)	**9.94E-59**
1.11 (0.97, 1.27)	3.69E-01	0.01 (0.01, 0.02)	**4.38E-09**
1.05 (0.92, 1.20)	7.59E-01	-0.03 (-0.04, -0.03)	**8.55E-54**
0.92 (0.81, 1.06)	5.47E-01	-0.03 (-0.04, -0.03)	**1.32E-53**
0.93 (0.81, 1.06)	5.89E-01	0.00 (0.00, 0.01)	6.24E-01
1.01 (0.88, 1.16)	9.65E-01	0.04 (0.04, 0.05)	**3.73E-68**
1.15 (1.00, 1.32)	2.06E-01	0.02 (0.01, 0.02)	**2.43E-15**
1.02 (0.88, 1.18)	9.17E-01	-0.01 (-0.02, -0.01)	**1.35E-08**
1.28 (1.12, 1.46)	**1.65E-02**	-0.01 (-0.01, 0.00)	**1.02E-02**
1.13 (0.98, 1.31)	3.00E-01	-0.02 (-0.02, -0.01)	**1.77E-14**
0.95 (0.83, 1.09)	7.40E-01	0.01 (0.01, 0.02)	**9.22E-07**
1.00 (0.86, 1.15)	9.80E-01	-0.01 (-0.02, -0.01)	**3.14E-11**
0.98 (0.85, 1.13)	9.17E-01	0.00 (-0.01, 0.00)	3.44E-01
0.93 (0.81, 1.06)	5.80E-01	-0.02 (-0.02, -0.02)	**1.06E-18**
0.99 (0.86, 1.14)	9.72E-01	-0.01 (-0.01, -0.01)	**4.08E-06**
0.99 (0.86, 1.14)	9.53E-01		
1.03 (0.90, 1.17)	8.92E-01		
0.96 (0.83, 1.11)	8.18E-01	0.02 (0.02, 0.03)	**1.33E-24**
0.96 (0.83, 1.11)	8.07E-01	0.02 (0.02, 0.03)	**1.24E-22**
1.05 (0.91, 1.22)	7.59E-01	0.02 (0.02, 0.03)	**2.39E-20**
1.02 (0.88, 1.19)	9.17E-01	0.02 (0.02, 0.03)	**1.88E-26**
1.14 (1.01, 1.30)	2.06E-01	-0.01 (-0.01, 0.00)	**1.79E-04**
1.18 (1.03, 1.35)	1.59E-01	0.03 (0.03, 0.04)	**6.87E-44**
0.99 (0.86, 1.14)	9.65E-01	-0.01 (-0.02, -0.01)	**2.12E-06**
0.99 (0.85, 1.15)	9.72E-01	-0.02 (-0.02, -0.01)	**9.00E-15**

0.97 (0.84, 1.11)	8.57E-01	-0.01 (-0.02, -0.01)	**2.82E-08**
0.96 (0.83, 1.11)	8.07E-01	-0.03 (-0.03, -0.02)	**1.30E-26**
1.01 (0.87, 1.16)	9.80E-01	0.00 (-0.01, 0.00)	3.39E-01
0.99 (0.86, 1.13)	9.55E-01	0.01 (0.01, 0.01)	**5.53E-06**
0.96 (0.83, 1.11)	8.45E-01	0.01 (0.00, 0.01)	**3.68E-05**
0.99 (0.85, 1.15)	9.65E-01	-0.01 (-0.01, 0.00)	**2.95E-03**
0.95 (0.82, 1.11)	8.02E-01	0.01 (0.01, 0.02)	**7.46E-08**
0.80 (0.70, 0.91)	**2.44E-02**	-0.01 (-0.01, 0.00)	**1.40E-02**
0.87 (0.75, 0.99)	2.06E-01	0.02 (0.02, 0.02)	**4.99E-20**
0.92 (0.80, 1.07)	5.95E-01	-0.01 (-0.01, 0.00)	**5.94E-03**
0.85 (0.73, 0.99)	2.06E-01	0.00 (0.00, 0.00)	8.95E-01
0.92 (0.79, 1.07)	5.80E-01	0.02 (0.01, 0.02)	**3.09E-13**
0.90 (0.77, 1.05)	4.38E-01	0.01 (0.00, 0.01)	**3.62E-05**
0.98 (0.84, 1.14)	9.24E-01	0.01 (0.00, 0.01)	**3.75E-04**
0.99 (0.85, 1.14)	9.53E-01	-0.03 (-0.03, -0.02)	**2.66E-33**
0.91 (0.79, 1.04)	3.89E-01	0.02 (0.02, 0.02)	**1.16E-18**
0.89 (0.78, 1.02)	3.00E-01	-0.03 (-0.04, -0.03)	**1.44E-40**
0.85 (0.74, 0.98)	1.78E-01	-0.02 (-0.03, -0.02)	**6.82E-20**
0.88 (0.77, 1.02)	3.00E-01	0.03 (0.02, 0.03)	**1.12E-32**
0.83 (0.72, 0.96)	1.27E-01	0.00 (0.00, 0.01)	1.37E-01
1.11 (0.96, 1.27)	3.96E-01	0.01 (0.00, 0.01)	**5.59E-04**
1.17 (1.02, 1.34)	1.92E-01	0.00 (0.00, 0.01)	3.87E-01
1.20 (1.07, 1.36)	**3.71E-02**	0.00 (-0.01, 0.00)	5.42E-02
0.90 (0.77, 1.04)	3.76E-01	0.01 (0.00, 0.01)	**9.68E-05**
1.08 (0.93, 1.24)	6.05E-01	-0.03 (-0.03, -0.02)	**1.59E-33**
0.95 (0.83, 1.09)	7.59E-01	0.00 (0.00, 0.00)	9.06E-01
0.89 (0.77, 1.02)	3.00E-01	0.01 (0.00, 0.01)	**5.72E-04**
0.93 (0.81, 1.07)	6.14E-01	0.01 (0.00, 0.01)	**6.98E-03**
0.93 (0.80, 1.08)	6.37E-01	0.02 (0.01, 0.02)	**2.08E-16**
0.89 (0.76, 1.04)	3.76E-01	0.01 (0.00, 0.01)	**3.10E-03**
0.91 (0.78, 1.06)	5.11E-01	0.00 (0.00, 0.01)	1.01E-01
1.14 (1.05, 1.25)	**3.81E-02**	-0.01 (-0.01, 0.00)	**1.47E-03**
1.23 (1.08, 1.41)	**3.50E-02**	0.00 (0.00, 0.01)	1.32E-01
0.97 (0.84, 1.13)	8.92E-01	0.00 (0.00, 0.01)	6.38E-02
1.02 (0.89, 1.18)	9.16E-01	0.03 (0.03, 0.04)	**1.13E-37**
1.09 (0.98, 1.20)	3.27E-01	-0.01 (-0.01, 0.00)	**3.63E-04**
1.08 (1.04, 1.12)	**9.88E-03**	0.02 (0.02, 0.03)	**1.24E-25**
0.84 (0.74, 0.95)	5.68E-02	-0.02 (-0.02, -0.01)	**2.81E-14**
0.99 (0.85, 1.16)	9.76E-01	0.01 (0.01, 0.02)	**1.35E-08**
		-0.01 (-0.01, 0.00)	**7.85E-05**
		-0.02 (-0.02, -0.01)	**6.97E-14**
		-0.01 (-0.02, -0.01)	**3.34E-08**
		-0.03 (-0.04, -0.03)	**6.80E-43**
		-0.03 (-0.03, -0.02)	**1.45E-28**
		-0.03 (-0.03, -0.02)	**4.74E-31**
		0.01 (0.01, 0.02)	**7.27E-11**
		-0.02 (-0.03, -0.02)	**4.65E-21**
		-0.02 (-0.03, -0.02)	**2.71E-19**

0.00 (0.00, 0.01)	**4.60E-02**
-0.01 (-0.01, 0.00)	**8.56E-03**
-0.03 (-0.03, -0.03)	**1.13E-37**
0.02 (0.01, 0.02)	**1.24E-12**
0.00 (-0.01, 0.00)	1.68E-01
-0.03 (-0.04, -0.03)	**5.98E-40**
-0.01 (-0.02, -0.01)	**5.09E-06**
0.02 (0.01, 0.02)	**2.87E-15**
-0.01 (-0.02, -0.01)	**6.17E-10**
-0.02 (-0.03, -0.02)	**1.13E-16**
0.00 (-0.01, 0.00)	5.06E-02
0.02 (0.01, 0.02)	**1.44E-17**
-0.01 (-0.02, -0.01)	**1.54E-08**
-0.02 (-0.03, -0.02)	**4.73E-22**
0.01 (0.00, 0.01)	**3.18E-04**
-0.03 (-0.04, -0.03)	**1.65E-37**
0.01 (0.01, 0.02)	**1.08E-11**

All causes mortality		Ischaemic heart diseases mortality	
Adjusted HR (95% CI)	P value	Adjusted HR (95% CI)	P value
0.97 (0.95, 0.98)	**1.75E-05**	0.98 (0.94, 1.02)	3.68E-01
0.97 (0.95, 0.98)	**9.44E-06**	0.98 (0.94, 1.02)	3.12E-01
0.97 (0.96, 0.98)	**4.83E-05**	0.98 (0.94, 1.02)	3.88E-01
0.97 (0.95, 0.98)	**6.57E-06**	0.98 (0.94, 1.02)	3.79E-01
0.97 (0.95, 0.98)	**4.06E-06**	0.98 (0.94, 1.02)	4.22E-01
0.97 (0.95, 0.98)	**1.59E-05**	0.98 (0.94, 1.02)	3.40E-01
0.97 (0.95, 0.98)	**9.53E-06**	0.97 (0.94, 1.01)	2.92E-01
0.94 (0.92, 0.95)	**1.45E-17**	0.97 (0.92, 1.01)	1.74E-01
0.94 (0.92, 0.95)	**1.47E-18**	0.96 (0.92, 1.00)	1.18E-01
0.94 (0.93, 0.95)	**1.20E-15**	0.97 (0.93, 1.01)	2.21E-01
0.93 (0.92, 0.95)	**1.07E-20**	0.97 (0.92, 1.01)	2.12E-01
0.93 (0.91, 0.94)	**3.19E-24**	0.97 (0.92, 1.01)	2.11E-01
0.94 (0.93, 0.95)	**3.14E-16**	0.97 (0.93, 1.01)	2.34E-01
0.94 (0.92, 0.95)	**6.72E-18**	0.96 (0.92, 1.00)	8.34E-02
0.93 (0.91, 0.94)	**4.55E-24**	0.96 (0.92, 1.00)	1.09E-01
0.92 (0.90, 0.93)	**2.84E-30**	0.95 (0.91, 0.99)	**3.97E-02**
0.93 (0.91, 0.94)	**5.26E-22**	0.96 (0.92, 1.01)	1.57E-01
0.92 (0.91, 0.94)	**1.31E-25**	0.96 (0.92, 1.01)	1.74E-01
0.93 (0.91, 0.94)	**2.08E-23**	0.97 (0.93, 1.02)	2.66E-01
0.92 (0.91, 0.93)	**1.76E-26**	0.96 (0.92, 1.00)	1.24E-01
0.91 (0.90, 0.92)	**1.25E-34**	0.94 (0.90, 0.98)	**8.58E-03**
0.92 (0.90, 0.93)	**3.02E-31**	0.96 (0.92, 1.01)	1.43E-01
0.91 (0.90, 0.92)	**8.01E-37**	0.95 (0.91, 0.99)	5.72E-02
0.92 (0.91, 0.93)	**2.95E-28**	0.97 (0.92, 1.01)	2.01E-01
0.93 (0.92, 0.95)	**1.31E-20**	0.98 (0.94, 1.02)	4.55E-01
0.94 (0.93, 0.96)	**4.66E-15**	0.99 (0.94, 1.03)	6.52E-01
0.92 (0.91, 0.94)	**6.60E-26**	0.97 (0.93, 1.02)	2.87E-01
0.91 (0.89, 0.92)	**3.05E-37**	0.94 (0.90, 0.98)	**1.75E-02**
0.95 (0.93, 0.96)	**2.23E-13**	1.00 (0.96, 1.05)	9.28E-01
0.94 (0.93, 0.96)	**2.14E-15**	1.00 (0.95, 1.04)	9.18E-01
0.93 (0.92, 0.95)	**1.07E-19**	0.99 (0.95, 1.04)	8.32E-01
0.95 (0.93, 0.96)	**2.79E-13**	1.01 (0.97, 1.06)	7.28E-01
0.96 (0.94, 0.97)	**1.75E-09**	1.02 (0.98, 1.07)	4.40E-01
0.93 (0.92, 0.95)	**6.12E-21**	0.99 (0.95, 1.04)	7.22E-01
0.95 (0.94, 0.97)	**1.70E-10**	0.99 (0.95, 1.03)	6.79E-01
1.02 (1.01, 1.04)	**4.24E-03**	1.06 (1.02, 1.11)	**1.60E-02**
1.03 (1.01, 1.04)	**3.70E-04**	1.07 (1.02, 1.11)	**1.19E-02**
1.04 (1.02, 1.05)	**1.11E-07**	1.08 (1.04, 1.13)	**8.78E-04**
1.02 (1.00, 1.03)	**3.46E-02**	1.05 (1.00, 1.09)	1.01E-01
1.01 (1.00, 1.03)	1.31E-01	1.03 (0.99, 1.08)	2.18E-01
1.03 (1.01, 1.04)	**6.34E-04**	1.07 (1.02, 1.12)	**1.22E-02**
1.02 (1.01, 1.04)	**1.23E-03**	1.06 (1.02, 1.10)	**1.36E-02**

0.96 (0.95, 0.97)	**1.15E-07**	1.00 (0.95, 1.04)	9.18E-01
0.96 (0.95, 0.98)	**1.10E-06**	0.99 (0.95, 1.04)	7.28E-01
0.98 (0.96, 0.99)	**3.72E-03**	1.01 (0.97, 1.06)	7.22E-01
0.95 (0.94, 0.96)	**5.38E-11**	0.97 (0.93, 1.02)	2.93E-01
0.95 (0.93, 0.96)	**3.18E-13**	0.96 (0.92, 1.01)	1.68E-01
0.97 (0.95, 0.98)	**1.19E-05**	1.00 (0.95, 1.04)	9.18E-01
1.05 (1.03, 1.06)	**1.10E-10**	1.08 (1.04, 1.13)	**4.51E-04**
0.93 (0.92, 0.95)	**5.66E-21**	1.00 (0.95, 1.04)	9.18E-01
0.91 (0.90, 0.93)	**3.48E-32**	0.96 (0.92, 1.00)	1.23E-01
0.91 (0.90, 0.93)	**8.19E-33**	0.96 (0.91, 1.00)	9.77E-02
0.91 (0.89, 0.92)	**3.05E-37**	0.95 (0.91, 0.99)	5.75E-02
0.90 (0.89, 0.92)	**6.09E-40**	0.95 (0.91, 0.99)	5.21E-02
0.92 (0.91, 0.93)	**1.49E-28**	0.95 (0.91, 1.00)	9.05E-02
1.03 (1.02, 1.05)	**2.79E-06**	1.08 (1.04, 1.12)	**9.39E-04**
0.91 (0.90, 0.93)	**5.31E-33**	0.96 (0.92, 1.00)	1.23E-01
0.89 (0.88, 0.91)	**4.94E-51**	0.95 (0.91, 0.99)	**4.71E-02**
0.89 (0.88, 0.90)	**2.21E-53**	0.95 (0.91, 1.00)	6.39E-02
0.89 (0.88, 0.90)	**2.11E-55**	0.94 (0.90, 0.98)	**1.93E-02**
0.89 (0.88, 0.90)	**6.32E-54**	0.94 (0.90, 0.98)	**1.93E-02**
0.89 (0.88, 0.91)	**3.25E-52**	0.94 (0.90, 0.99)	**3.12E-02**
1.00 (0.99, 1.02)	8.49E-01	1.04 (1.00, 1.09)	6.37E-02
0.93 (0.92, 0.94)	**1.91E-23**	0.98 (0.94, 1.02)	4.74E-01
0.91 (0.90, 0.92)	**1.17E-37**	0.97 (0.93, 1.01)	2.77E-01
0.93 (0.91, 0.94)	**4.12E-26**	1.00 (0.96, 1.04)	9.18E-01
0.90 (0.89, 0.91)	**1.14E-45**	0.96 (0.92, 1.00)	9.47E-02
0.90 (0.89, 0.92)	**3.92E-43**	0.96 (0.92, 1.00)	8.93E-02
0.90 (0.89, 0.92)	**8.35E-43**	0.96 (0.92, 1.01)	1.68E-01
0.98 (0.97, 0.99)	**7.71E-03**	1.01 (0.97, 1.05)	6.11E-01
1.13 (1.12, 1.15)	**2.76E-54**	1.11 (1.05, 1.17)	**7.48E-04**
1.15 (1.13, 1.17)	**6.27E-65**	1.12 (1.06, 1.18)	**3.03E-04**
1.16 (1.14, 1.18)	**1.26E-72**	1.12 (1.06, 1.18)	**2.56E-04**
1.13 (1.11, 1.15)	**6.50E-52**	1.11 (1.05, 1.17)	**8.78E-04**
1.12 (1.11, 1.14)	**5.44E-44**	1.09 (1.03, 1.16)	**5.55E-03**
1.16 (1.14, 1.17)	**7.99E-79**	1.15 (1.09, 1.21)	**5.63E-07**
1.05 (1.03, 1.06)	**9.54E-12**	1.05 (1.01, 1.09)	5.50E-02
1.10 (1.08, 1.12)	**3.13E-28**	1.02 (0.97, 1.08)	5.29E-01
1.10 (1.08, 1.12)	**2.55E-30**	1.02 (0.97, 1.08)	5.18E-01
1.10 (1.08, 1.12)	**1.06E-29**	1.02 (0.96, 1.08)	6.23E-01
1.10 (1.08, 1.12)	**1.18E-27**	1.03 (0.97, 1.09)	4.78E-01
1.09 (1.08, 1.11)	**4.33E-25**	1.02 (0.96, 1.08)	6.03E-01
1.11 (1.10, 1.13)	**1.09E-36**	1.05 (0.99, 1.11)	1.83E-01
1.07 (1.06, 1.09)	**9.19E-22**	1.03 (0.98, 1.07)	3.12E-01
0.98 (0.97, 1.00)	**1.72E-02**	0.91 (0.86, 0.95)	**5.27E-04**
0.98 (0.97, 1.00)	**2.04E-02**	0.91 (0.87, 0.96)	**8.78E-04**
0.98 (0.97, 1.00)	**3.86E-02**	0.92 (0.87, 0.96)	**1.12E-03**
0.98 (0.96, 0.99)	**6.76E-03**	0.91 (0.86, 0.95)	**6.57E-04**
0.97 (0.96, 0.99)	**1.66E-03**	0.90 (0.86, 0.95)	**3.94E-04**
0.99 (0.98, 1.01)	3.92E-01	0.92 (0.88, 0.97)	**6.75E-03**

1.00 (0.99, 1.02)	5.89E-01	0.99 (0.94, 1.03)	5.85E-01
0.86 (0.85, 0.87)	**2.28E-90**	0.86 (0.83, 0.90)	**1.38E-09**
0.89 (0.88, 0.90)	**2.83E-56**	0.88 (0.84, 0.92)	**6.20E-08**
0.91 (0.89, 0.92)	**6.92E-40**	0.88 (0.84, 0.92)	**2.12E-07**
0.86 (0.85, 0.88)	**1.45E-85**	0.87 (0.83, 0.90)	**2.51E-09**
0.85 (0.84, 0.87)	**7.16E-100**	0.85 (0.82, 0.89)	**8.58E-11**
0.91 (0.90, 0.93)	**1.55E-33**	0.92 (0.88, 0.96)	**3.97E-04**
0.97 (0.95, 0.98)	**3.51E-05**	0.99 (0.95, 1.04)	7.82E-01
1.03 (1.02, 1.05)	**1.64E-04**	1.11 (1.05, 1.17)	**9.03E-04**
1.03 (1.02, 1.05)	**7.56E-05**	1.02 (0.97, 1.07)	5.32E-01
1.02 (1.01, 1.04)	**2.33E-03**	1.01 (0.96, 1.06)	8.32E-01
1.04 (1.02, 1.05)	**2.08E-06**	1.04 (0.99, 1.09)	1.85E-01
0.97 (0.95, 0.98)	**3.61E-06**	0.96 (0.92, 1.01)	1.51E-01
1.05 (1.04, 1.07)	**2.81E-10**	1.05 (0.99, 1.11)	1.30E-01
1.04 (1.02, 1.06)	**5.93E-06**	1.04 (0.98, 1.09)	2.77E-01
1.09 (1.08, 1.11)	**2.60E-30**	1.09 (1.03, 1.14)	**6.75E-03**
0.94 (0.93, 0.96)	**1.19E-13**	0.95 (0.90, 1.00)	6.93E-02
1.01 (1.00, 1.03)	1.49E-01	1.06 (1.00, 1.13)	1.09E-01
1.08 (1.07, 1.10)	**1.86E-27**	1.11 (1.07, 1.16)	**1.39E-05**
1.09 (1.07, 1.11)	**5.24E-30**	1.10 (1.05, 1.15)	**1.82E-04**
1.05 (1.04, 1.07)	**2.50E-11**	1.13 (1.08, 1.19)	**3.22E-06**
0.93 (0.92, 0.95)	**1.83E-18**	0.89 (0.85, 0.94)	**2.96E-05**
1.04 (1.02, 1.05)	**7.31E-07**	1.03 (0.99, 1.08)	1.82E-01
1.03 (1.02, 1.05)	**1.15E-05**	1.02 (0.98, 1.07)	3.65E-01
1.03 (1.02, 1.05)	**2.70E-05**	1.02 (0.98, 1.07)	4.58E-01
1.04 (1.02, 1.05)	**2.04E-06**	1.03 (0.99, 1.08)	1.94E-01
0.97 (0.95, 0.98)	**4.58E-06**	0.97 (0.93, 1.02)	3.11E-01
0.96 (0.95, 0.98)	**1.15E-06**	0.96 (0.92, 1.01)	1.68E-01
1.00 (0.99, 1.02)	7.79E-01	1.01 (0.97, 1.06)	6.95E-01
1.03 (1.01, 1.04)	**1.33E-04**	1.05 (1.00, 1.09)	7.15E-02
0.96 (0.95, 0.98)	**5.66E-06**	0.96 (0.92, 1.00)	1.32E-01
1.01 (0.99, 1.02)	2.10E-01	1.00 (0.96, 1.05)	9.20E-01
1.12 (1.11, 1.14)	**2.23E-55**	1.19 (1.14, 1.24)	**2.14E-13**
0.98 (0.96, 0.99)	**2.80E-03**	0.94 (0.91, 0.99)	**2.23E-02**
0.97 (0.96, 0.99)	**6.29E-04**	0.94 (0.90, 0.98)	**7.64E-03**
1.00 (0.99, 1.02)	7.32E-01	1.01 (0.97, 1.04)	8.00E-01
1.00 (0.98, 1.01)	9.16E-01	1.03 (0.99, 1.07)	2.93E-01
1.11 (1.09, 1.12)	**6.93E-48**	1.14 (1.10, 1.19)	**2.58E-09**
0.91 (0.90, 0.92)	**1.54E-41**	0.89 (0.86, 0.93)	**2.31E-08**
0.89 (0.88, 0.90)	**1.46E-64**	0.86 (0.83, 0.89)	**2.14E-13**
1.00 (0.99, 1.02)	5.81E-01	1.01 (0.98, 1.05)	5.50E-01
1.08 (1.06, 1.09)	**5.02E-27**	1.09 (1.05, 1.13)	**1.09E-05**
1.02 (1.01, 1.04)	**6.06E-04**	1.00 (0.96, 1.04)	9.31E-01
0.93 (0.92, 0.94)	**1.57E-77**	0.94 (0.92, 0.96)	**8.52E-08**
0.93 (0.92, 0.93)	**1.21E-122**	0.94 (0.92, 0.96)	**8.99E-09**
0.98 (0.97, 1.00)	**2.87E-02**	0.96 (0.92, 1.00)	5.99E-02
1.09 (1.08, 1.10)	**1.01E-71**	1.08 (1.05, 1.11)	**1.47E-08**
1.01 (1.00, 1.03)	**4.90E-02**	1.05 (1.01, 1.09)	**2.30E-02**

0.93 (0.92, 0.93)	**3.22E-85**	0.93 (0.91, 0.95)	**6.92E-11**
0.91 (0.90, 0.92)	**9.32E-57**	0.90 (0.88, 0.94)	**3.71E-08**
0.98 (0.97, 1.00)	**2.96E-02**	0.98 (0.94, 1.02)	3.16E-01
1.09 (1.08, 1.10)	**3.60E-96**	1.09 (1.06, 1.12)	**1.89E-09**
1.11 (1.09, 1.12)	**1.80E-51**	1.13 (1.09, 1.17)	**2.58E-09**
0.90 (0.89, 0.91)	**3.99E-100**	0.90 (0.88, 0.93)	**1.80E-12**
0.91 (0.90, 0.92)	**5.92E-43**	0.89 (0.86, 0.92)	**5.82E-09**
0.96 (0.95, 0.97)	**8.62E-10**	0.98 (0.94, 1.01)	3.01E-01
1.05 (1.04, 1.07)	**2.25E-15**	1.04 (1.00, 1.08)	5.75E-02
1.14 (1.12, 1.15)	**2.50E-54**	1.08 (1.04, 1.12)	**9.01E-04**
0.89 (0.88, 0.91)	**1.54E-53**	0.95 (0.92, 0.99)	**3.90E-02**
0.93 (0.92, 0.94)	**2.25E-29**	0.98 (0.95, 1.01)	2.56E-01
0.92 (0.91, 0.94)	**8.41E-23**	0.96 (0.92, 1.00)	9.36E-02
0.96 (0.94, 0.97)	**4.00E-10**	0.95 (0.91, 0.98)	**1.47E-02**
0.96 (0.95, 0.98)	**9.52E-08**	0.96 (0.93, 1.00)	8.25E-02
1.03 (1.02, 1.05)	**1.48E-05**	1.02 (0.99, 1.06)	3.12E-01
1.02 (1.00, 1.03)	**3.52E-02**	1.01 (0.97, 1.05)	7.10E-01
1.09 (1.07, 1.10)	**2.13E-29**	1.07 (1.03, 1.11)	**2.24E-03**
0.98 (0.97, 1.00)	**2.43E-02**	1.00 (0.96, 1.03)	9.20E-01
1.04 (1.02, 1.06)	**3.13E-07**	1.06 (1.01, 1.11)	**2.32E-02**
0.97 (0.95, 0.98)	**1.08E-05**	0.94 (0.90, 0.99)	**2.57E-02**
0.96 (0.95, 0.98)	**6.39E-07**	0.95 (0.91, 0.99)	**4.19E-02**
1.00 (0.99, 1.02)	6.54E-01	0.95 (0.91, 1.00)	8.24E-02
1.03 (1.01, 1.04)	**3.57E-04**	1.05 (1.01, 1.10)	**4.07E-02**
1.12 (1.10, 1.13)	**3.87E-51**	1.00 (0.96, 1.05)	8.97E-01
0.91 (0.90, 0.93)	**1.84E-37**	0.95 (0.91, 0.99)	**4.80E-02**
0.90 (0.88, 0.91)	**3.06E-55**	0.93 (0.89, 0.97)	**1.55E-03**
1.09 (1.07, 1.10)	**1.63E-29**	1.12 (1.07, 1.17)	**2.76E-06**
1.04 (1.02, 1.05)	**3.64E-06**	1.08 (1.03, 1.13)	**3.22E-03**
0.88 (0.87, 0.90)	**1.22E-58**	0.89 (0.85, 0.93)	**4.67E-06**
1.01 (1.00, 1.03)	6.56E-02	1.06 (1.01, 1.10)	**2.57E-02**
1.16 (1.14, 1.18)	**2.92E-69**	1.06 (1.00, 1.12)	7.51E-02
0.93 (0.91, 0.94)	**2.35E-22**	0.95 (0.91, 0.99)	5.75E-02
0.95 (0.93, 0.96)	**1.42E-13**	0.99 (0.95, 1.03)	7.22E-01
0.90 (0.89, 0.91)	**1.23E-43**	0.95 (0.91, 0.99)	**4.40E-02**
0.94 (0.93, 0.96)	**1.66E-14**	0.98 (0.94, 1.02)	4.61E-01
0.93 (0.91, 0.94)	**2.34E-23**	0.94 (0.90, 0.99)	**3.96E-02**
0.93 (0.92, 0.95)	**2.95E-19**	0.96 (0.92, 1.01)	1.51E-01

0.95 (0.94, 0.96)	**1.83E-11**	0.98 (0.94, 1.02)	3.12E-01
0.94 (0.92, 0.95)	**1.47E-18**	0.96 (0.92, 1.00)	1.09E-01
1.02 (1.00, 1.03)	**1.01E-02**	1.06 (1.02, 1.10)	**1.19E-02**
1.01 (1.00, 1.03)	1.37E-01	1.00 (0.96, 1.04)	9.82E-01
0.96 (0.95, 0.98)	**2.81E-07**	0.94 (0.90, 0.99)	**2.75E-02**
0.96 (0.94, 0.97)	**6.31E-08**	1.00 (0.96, 1.05)	9.78E-01
0.96 (0.94, 0.97)	**1.35E-08**	0.93 (0.88, 0.97)	**4.40E-03**
0.99 (0.98, 1.01)	4.62E-01	1.01 (0.97, 1.06)	6.79E-01

0.96 (0.94, 0.97)	**9.80E-08**	0.94 (0.90, 0.99)	**2.65E-02**
0.98 (0.97, 1.00)	**4.29E-02**	0.91 (0.87, 0.96)	**1.55E-03**
0.93 (0.92, 0.95)	**1.36E-20**	0.99 (0.95, 1.03)	6.79E-01
0.96 (0.94, 0.97)	**1.55E-08**	1.05 (1.00, 1.09)	6.90E-02
0.96 (0.94, 0.97)	**4.14E-10**	0.98 (0.94, 1.02)	3.12E-01
0.88 (0.86, 0.89)	**9.96E-65**	0.89 (0.85, 0.93)	**1.29E-06**
0.88 (0.87, 0.90)	**3.94E-52**	0.85 (0.81, 0.90)	**8.78E-09**
0.92 (0.91, 0.93)	**1.93E-27**	0.93 (0.89, 0.97)	**4.53E-03**
0.87 (0.86, 0.89)	**1.84E-63**	0.86 (0.82, 0.91)	**3.98E-08**
0.92 (0.90, 0.93)	**4.45E-30**	0.93 (0.89, 0.98)	**1.01E-02**
0.90 (0.88, 0.91)	**5.97E-47**	0.91 (0.87, 0.95)	**1.89E-04**
0.99 (0.97, 1.00)	1.04E-01	1.01 (0.98, 1.06)	5.49E-01
0.98 (0.97, 0.99)	**5.66E-03**	0.99 (0.95, 1.03)	7.20E-01
0.91 (0.89, 0.92)	**4.21E-34**	0.88 (0.84, 0.93)	**2.05E-06**
0.94 (0.93, 0.96)	**8.57E-15**	0.92 (0.88, 0.96)	**9.39E-04**
0.87 (0.86, 0.89)	**7.36E-65**	0.86 (0.82, 0.90)	**2.31E-08**
0.97 (0.96, 0.98)	**5.77E-05**	0.96 (0.92, 1.00)	1.25E-01
0.92 (0.91, 0.94)	**2.95E-28**	0.92 (0.88, 0.96)	**2.56E-04**
1.06 (1.05, 1.08)	**2.04E-15**	1.11 (1.06, 1.16)	**1.23E-05**
1.07 (1.06, 1.09)	**8.29E-24**	1.03 (0.99, 1.07)	1.95E-01
1.06 (1.04, 1.07)	**6.06E-19**	1.08 (1.05, 1.11)	**1.31E-06**
1.03 (1.01, 1.04)	**3.79E-04**	1.02 (0.98, 1.07)	3.34E-01
1.06 (1.05, 1.08)	**4.36E-19**	1.09 (1.05, 1.13)	**4.07E-05**
0.96 (0.95, 0.98)	**1.28E-07**	0.99 (0.95, 1.03)	6.45E-01
0.97 (0.95, 0.98)	**5.08E-06**	0.99 (0.95, 1.04)	8.07E-01
0.92 (0.91, 0.93)	**2.61E-26**	0.89 (0.85, 0.94)	**1.36E-05**
0.96 (0.94, 0.97)	**1.03E-08**	1.00 (0.95, 1.04)	8.84E-01
0.91 (0.89, 0.92)	**9.85E-36**	0.96 (0.92, 1.00)	9.67E-02
0.90 (0.88, 0.91)	**5.06E-44**	0.94 (0.90, 0.99)	**3.01E-02**
1.03 (1.02, 1.05)	**8.23E-08**	1.03 (1.00, 1.07)	9.05E-02
1.02 (1.01, 1.04)	**2.90E-03**	1.03 (0.99, 1.08)	1.82E-01
1.07 (1.06, 1.07)	**2.20E-93**	1.06 (1.04, 1.08)	**5.10E-07**
1.06 (1.05, 1.07)	**4.53E-43**	1.07 (1.05, 1.09)	**6.53E-10**
1.07 (1.06, 1.08)	**1.42E-46**	1.08 (1.05, 1.11)	**9.92E-08**
1.05 (1.04, 1.06)	**3.40E-34**	1.06 (1.05, 1.08)	**1.39E-13**
0.85 (0.84, 0.87)	**8.45E-103**	0.84 (0.81, 0.88)	**3.37E-13**
1.12 (1.10, 1.13)	**8.07E-50**	1.18 (1.13, 1.23)	**3.45E-13**
1.01 (0.99, 1.03)	1.94E-01	0.96 (0.90, 1.01)	2.12E-01
0.91 (0.89, 0.92)	**7.44E-34**	0.96 (0.92, 1.00)	9.88E-02
0.92 (0.91, 0.93)	**1.89E-28**	0.96 (0.92, 1.01)	1.52E-01
0.91 (0.90, 0.92)	**1.09E-36**	0.95 (0.91, 1.00)	8.25E-02
0.90 (0.89, 0.91)	**2.70E-45**	0.95 (0.91, 0.99)	**3.97E-02**
1.00 (0.99, 1.02)	6.69E-01	0.93 (0.88, 0.98)	**2.00E-02**
0.93 (0.91, 0.94)	**9.58E-22**	0.89 (0.85, 0.94)	**1.39E-05**
1.09 (1.08, 1.10)	**9.77E-95**	1.08 (1.05, 1.11)	**3.83E-06**
0.94 (0.93, 0.95)	**6.05E-17**	0.98 (0.94, 1.03)	4.78E-01
0.91 (0.90, 0.92)	**2.46E-36**	0.95 (0.91, 1.00)	7.15E-02
1.04 (1.02, 1.06)	**4.57E-06**	0.97 (0.92, 1.03)	4.05E-01

1.04 (1.03, 1.05)	**2.95E-10**	1.04 (1.01, 1.08)	**1.36E-02**
1.07 (1.06, 1.08)	**2.07E-63**	1.08 (1.05, 1.10)	**9.38E-09**
0.92 (0.91, 0.94)	**3.94E-24**	0.89 (0.85, 0.93)	**1.15E-05**
0.95 (0.94, 0.97)	**3.22E-10**	1.00 (0.96, 1.05)	9.78E-01
0.93 (0.91, 0.94)	**2.69E-25**	0.98 (0.94, 1.03)	5.79E-01
0.93 (0.92, 0.95)	**2.19E-19**	0.89 (0.85, 0.93)	**6.03E-06**
0.95 (0.94, 0.97)	**1.44E-09**	0.95 (0.91, 0.99)	5.72E-02
0.95 (0.93, 0.96)	**4.30E-14**	0.99 (0.94, 1.03)	5.94E-01
0.91 (0.89, 0.92)	**1.30E-38**	0.96 (0.92, 1.00)	1.24E-01
1.02 (1.00, 1.03)	**3.26E-02**	0.94 (0.89, 0.99)	**3.58E-02**
0.93 (0.92, 0.95)	**7.33E-21**	0.95 (0.91, 0.99)	5.72E-02
0.94 (0.92, 0.95)	**5.21E-18**	0.97 (0.93, 1.01)	2.56E-01
0.91 (0.89, 0.92)	**2.15E-38**	0.96 (0.92, 1.00)	1.00E-01
1.02 (1.00, 1.03)	5.88E-02	0.94 (0.89, 0.99)	**3.99E-02**
0.95 (0.94, 0.97)	**1.02E-10**	1.00 (0.95, 1.04)	9.20E-01
1.01 (1.00, 1.03)	1.74E-01	0.94 (0.89, 0.99)	**4.40E-02**
1.03 (1.02, 1.05)	**1.98E-04**	1.04 (0.98, 1.10)	2.77E-01

Cerebrovascular diseases mortality		Cancer mortality	
Adjusted HR (95% CI)	P value	Adjusted HR (95% CI)	P value
0.94 (0.88, 1.01)	1.75E-01	1.01 (0.99, 1.03)	2.21E-01
0.94 (0.88, 1.01)	1.61E-01	1.01 (0.99, 1.03)	2.74E-01
0.95 (0.88, 1.02)	2.32E-01	1.02 (0.99, 1.04)	1.81E-01
0.95 (0.89, 1.02)	2.60E-01	1.01 (0.99, 1.03)	2.52E-01
0.95 (0.89, 1.03)	2.74E-01	1.01 (0.99, 1.03)	2.94E-01
0.95 (0.89, 1.02)	2.47E-01	1.01 (0.99, 1.04)	2.13E-01
0.94 (0.87, 1.01)	1.28E-01	1.01 (0.99, 1.03)	3.08E-01
0.92 (0.85, 0.99)	**3.86E-02**	0.99 (0.97, 1.02)	6.45E-01
0.91 (0.85, 0.98)	**3.48E-02**	0.99 (0.97, 1.01)	5.48E-01
0.92 (0.86, 0.99)	**4.80E-02**	1.00 (0.98, 1.02)	8.20E-01
0.92 (0.85, 0.98)	**3.73E-02**	0.99 (0.97, 1.01)	3.46E-01
0.91 (0.85, 0.98)	**3.08E-02**	0.98 (0.96, 1.00)	1.32E-01
0.92 (0.86, 0.99)	5.24E-02	1.00 (0.98, 1.02)	7.54E-01
0.91 (0.85, 0.98)	**3.45E-02**	0.99 (0.97, 1.01)	5.62E-01
0.91 (0.85, 0.98)	**2.80E-02**	0.99 (0.97, 1.01)	2.29E-01
0.90 (0.84, 0.97)	**1.65E-02**	0.98 (0.96, 1.00)	6.46E-02
0.92 (0.85, 0.98)	**3.86E-02**	0.99 (0.97, 1.01)	3.52E-01
0.92 (0.85, 0.98)	**3.73E-02**	0.98 (0.96, 1.00)	1.63E-01
0.92 (0.86, 0.99)	5.67E-02	0.98 (0.96, 1.00)	1.77E-01
0.91 (0.84, 0.98)	**2.66E-02**	0.98 (0.96, 1.00)	1.61E-01
0.89 (0.83, 0.96)	**8.98E-03**	0.97 (0.95, 0.99)	**1.48E-02**
0.90 (0.84, 0.97)	**1.27E-02**	0.97 (0.95, 0.99)	**1.24E-03**
0.90 (0.84, 0.96)	**8.89E-03**	0.96 (0.94, 0.98)	**6.40E-04**
0.90 (0.84, 0.97)	**1.50E-02**	0.97 (0.95, 0.99)	**2.08E-03**
0.92 (0.86, 0.98)	**3.73E-02**	0.96 (0.94, 0.98)	**3.59E-04**
0.93 (0.87, 1.00)	7.47E-02	0.96 (0.94, 0.98)	**2.26E-04**
0.91 (0.85, 0.97)	**1.99E-02**	0.97 (0.95, 0.99)	**1.46E-03**
0.90 (0.84, 0.97)	**1.25E-02**	0.97 (0.95, 0.99)	**4.83E-03**
0.94 (0.88, 1.01)	1.66E-01	0.99 (0.97, 1.01)	5.39E-01
0.94 (0.87, 1.00)	1.04E-01	0.99 (0.97, 1.01)	3.46E-01
0.92 (0.86, 0.99)	**4.63E-02**	0.98 (0.96, 1.00)	**3.10E-02**
0.93 (0.87, 1.00)	9.68E-02	0.98 (0.96, 1.00)	1.51E-01
0.94 (0.88, 1.01)	1.71E-01	0.99 (0.97, 1.01)	5.18E-01
0.92 (0.86, 0.98)	**3.73E-02**	0.97 (0.95, 0.99)	**3.30E-03**
0.95 (0.89, 1.02)	2.74E-01	1.00 (0.98, 1.02)	7.87E-01
0.98 (0.92, 1.05)	6.80E-01	1.03 (1.01, 1.05)	**4.34E-03**
0.99 (0.92, 1.06)	8.22E-01	1.04 (1.02, 1.06)	**1.08E-03**
1.00 (0.94, 1.08)	9.10E-01	1.05 (1.03, 1.07)	**1.36E-05**
0.97 (0.91, 1.05)	5.63E-01	1.02 (1.00, 1.04)	1.05E-01
0.97 (0.90, 1.04)	4.59E-01	1.01 (0.99, 1.03)	3.08E-01
0.99 (0.92, 1.06)	8.32E-01	1.03 (1.01, 1.05)	**2.41E-03**
1.01 (0.94, 1.08)	8.22E-01	1.05 (1.03, 1.07)	**1.64E-05**

0.90 (0.84, 0.97)	**1.50E-02**	0.97 (0.95, 0.99)	**2.03E-02**
0.91 (0.84, 0.97)	**1.99E-02**	0.97 (0.95, 0.99)	**3.26E-03**
0.93 (0.86, 0.99)	6.48E-02	0.98 (0.96, 1.00)	1.05E-01
0.89 (0.83, 0.96)	**8.62E-03**	0.95 (0.93, 0.97)	**3.31E-05**
0.89 (0.82, 0.95)	**4.90E-03**	0.95 (0.93, 0.97)	**6.22E-06**
0.92 (0.85, 0.99)	**4.13E-02**	0.97 (0.95, 0.99)	**2.69E-03**
1.01 (0.95, 1.09)	7.45E-01	1.06 (1.04, 1.08)	**1.51E-07**
0.91 (0.85, 0.97)	**1.94E-02**	0.96 (0.94, 0.98)	**3.73E-05**
0.87 (0.81, 0.94)	**1.06E-03**	0.94 (0.92, 0.96)	**4.56E-09**
0.87 (0.81, 0.94)	**1.06E-03**	0.94 (0.92, 0.96)	**3.25E-09**
0.87 (0.81, 0.93)	**7.00E-04**	0.93 (0.91, 0.95)	**9.21E-11**
0.86 (0.80, 0.93)	**5.99E-04**	0.93 (0.91, 0.95)	**9.55E-11**
0.88 (0.81, 0.94)	**1.73E-03**	0.93 (0.91, 0.95)	**2.47E-10**
0.99 (0.92, 1.06)	8.22E-01	1.04 (1.02, 1.06)	**4.93E-05**
0.89 (0.83, 0.95)	**4.26E-03**	0.96 (0.94, 0.98)	**2.71E-05**
0.86 (0.80, 0.92)	**2.77E-04**	0.93 (0.92, 0.95)	**3.24E-10**
0.85 (0.80, 0.92)	**2.44E-04**	0.93 (0.91, 0.95)	**1.20E-11**
0.85 (0.80, 0.92)	**2.43E-04**	0.93 (0.91, 0.95)	**3.47E-11**
0.86 (0.80, 0.92)	**2.61E-04**	0.94 (0.92, 0.96)	**1.24E-09**
0.86 (0.80, 0.92)	**2.81E-04**	0.92 (0.90, 0.94)	**9.87E-15**
0.97 (0.90, 1.04)	4.08E-01	1.02 (1.00, 1.04)	**2.94E-02**
0.89 (0.83, 0.96)	**5.50E-03**	0.96 (0.94, 0.98)	**4.06E-04**
0.87 (0.81, 0.93)	**8.11E-04**	0.94 (0.92, 0.96)	**4.01E-08**
0.88 (0.82, 0.94)	**2.04E-03**	0.95 (0.93, 0.97)	**8.45E-07**
0.86 (0.81, 0.93)	**4.91E-04**	0.94 (0.92, 0.95)	**5.11E-10**
0.87 (0.81, 0.93)	**5.99E-04**	0.94 (0.92, 0.96)	**3.10E-08**
0.87 (0.81, 0.93)	**8.31E-04**	0.92 (0.91, 0.94)	**1.98E-13**
0.96 (0.89, 1.02)	2.74E-01	1.01 (1.00, 1.04)	1.81E-01
1.10 (1.02, 1.19)	**2.80E-02**	1.04 (1.02, 1.07)	**4.06E-04**
1.12 (1.04, 1.21)	**9.25E-03**	1.05 (1.03, 1.08)	**3.72E-05**
1.13 (1.05, 1.22)	**4.90E-03**	1.06 (1.04, 1.08)	**2.36E-06**
1.10 (1.02, 1.19)	**2.57E-02**	1.04 (1.01, 1.06)	**3.27E-03**
1.10 (1.02, 1.19)	**3.24E-02**	1.03 (1.00, 1.05)	**3.18E-02**
1.11 (1.03, 1.19)	**1.25E-02**	1.07 (1.04, 1.09)	**2.60E-08**
1.03 (0.96, 1.10)	5.41E-01	1.05 (1.03, 1.07)	**2.27E-06**
1.08 (1.00, 1.17)	9.68E-02	1.01 (0.99, 1.04)	3.28E-01
1.08 (1.00, 1.17)	9.56E-02	1.02 (0.99, 1.04)	2.18E-01
1.07 (0.99, 1.16)	1.32E-01	1.02 (1.00, 1.04)	1.55E-01
1.08 (1.00, 1.17)	8.29E-02	1.01 (0.98, 1.03)	5.23E-01
1.08 (1.00, 1.17)	8.46E-02	1.00 (0.98, 1.03)	7.54E-01
1.08 (1.00, 1.17)	8.12E-02	1.02 (1.00, 1.05)	7.80E-02
1.05 (0.98, 1.12)	2.64E-01	1.06 (1.04, 1.08)	**2.51E-08**
0.95 (0.88, 1.03)	2.75E-01	0.96 (0.94, 0.98)	**1.17E-03**
0.95 (0.88, 1.02)	2.64E-01	0.97 (0.95, 0.99)	**4.77E-03**
0.95 (0.88, 1.02)	2.56E-01	0.97 (0.95, 1.00)	**3.18E-02**
0.95 (0.88, 1.03)	2.85E-01	0.95 (0.93, 0.97)	**5.69E-05**
0.95 (0.88, 1.03)	2.75E-01	0.95 (0.93, 0.97)	**1.70E-05**
0.96 (0.89, 1.03)	3.41E-01	0.97 (0.94, 0.99)	**4.77E-03**

0.98 (0.92, 1.05)	6.78E-01	1.04 (1.02, 1.06)	**8.32E-04**
0.83 (0.78, 0.90)	**2.39E-05**	0.90 (0.88, 0.92)	**1.33E-21**
0.86 (0.80, 0.92)	**2.77E-04**	0.93 (0.91, 0.95)	**2.08E-11**
0.87 (0.81, 0.94)	**9.54E-04**	0.94 (0.92, 0.96)	**1.02E-07**
0.84 (0.78, 0.90)	**4.09E-05**	0.90 (0.88, 0.92)	**1.14E-22**
0.84 (0.78, 0.90)	**2.39E-05**	0.89 (0.87, 0.91)	**4.43E-26**
0.88 (0.82, 0.94)	**1.48E-03**	0.94 (0.92, 0.96)	**3.02E-09**
0.95 (0.88, 1.02)	2.51E-01	1.02 (1.00, 1.04)	8.28E-02
1.10 (1.02, 1.19)	**3.08E-02**	1.04 (1.01, 1.06)	**2.89E-03**
1.09 (1.02, 1.16)	**2.61E-02**	1.01 (0.99, 1.03)	3.79E-01
1.06 (0.99, 1.14)	1.27E-01	1.01 (0.99, 1.03)	5.48E-01
1.11 (1.05, 1.18)	**2.56E-03**	1.01 (0.99, 1.04)	2.28E-01
0.90 (0.85, 0.97)	**8.62E-03**	0.98 (0.96, 1.00)	1.05E-01
1.11 (1.03, 1.19)	**1.25E-02**	1.00 (0.98, 1.02)	9.57E-01
1.09 (1.01, 1.17)	**4.57E-02**	0.99 (0.97, 1.01)	3.79E-01
1.16 (1.09, 1.24)	**2.19E-04**	1.04 (1.02, 1.06)	**1.27E-03**
0.90 (0.83, 0.96)	**8.89E-03**	0.99 (0.96, 1.01)	2.28E-01
0.99 (0.92, 1.07)	8.83E-01	1.07 (1.04, 1.09)	**5.71E-07**
1.12 (1.04, 1.19)	**5.89E-03**	1.04 (1.02, 1.07)	**8.73E-05**
1.13 (1.06, 1.21)	**2.54E-03**	1.04 (1.02, 1.06)	**9.67E-04**
1.06 (0.98, 1.14)	1.85E-01	1.05 (1.03, 1.07)	**1.44E-05**
0.92 (0.86, 0.99)	5.24E-02	0.94 (0.92, 0.96)	**2.51E-08**
1.02 (0.95, 1.09)	6.78E-01	1.01 (0.99, 1.03)	4.04E-01
1.04 (0.97, 1.12)	3.26E-01	0.99 (0.97, 1.01)	3.08E-01
1.05 (0.97, 1.13)	2.86E-01	0.98 (0.96, 1.01)	1.77E-01
1.03 (0.96, 1.11)	4.89E-01	1.00 (0.98, 1.02)	9.99E-01
0.96 (0.90, 1.04)	3.87E-01	1.01 (0.99, 1.03)	5.29E-01
0.94 (0.87, 1.01)	1.50E-01	0.94 (0.92, 0.96)	**9.92E-08**
0.99 (0.93, 1.07)	8.76E-01	0.98 (0.96, 1.00)	9.31E-02
1.03 (0.96, 1.10)	5.41E-01	1.01 (0.99, 1.03)	3.53E-01
0.95 (0.88, 1.02)	2.28E-01	0.94 (0.92, 0.96)	**1.91E-07**
1.02 (0.95, 1.10)	6.08E-01	1.03 (1.01, 1.05)	**4.55E-03**
1.14 (1.06, 1.22)	**1.48E-03**	1.12 (1.09, 1.14)	**1.25E-23**
0.95 (0.89, 1.02)	2.74E-01	0.96 (0.94, 0.98)	**6.58E-05**
0.95 (0.88, 1.02)	2.05E-01	0.95 (0.93, 0.97)	**1.17E-05**
1.01 (0.94, 1.08)	8.22E-01	0.99 (0.97, 1.01)	5.48E-01
1.02 (0.95, 1.10)	6.21E-01	1.03 (1.00, 1.05)	**2.87E-02**
1.15 (1.08, 1.23)	**3.74E-04**	1.10 (1.08, 1.12)	**5.73E-20**
0.88 (0.82, 0.93)	**4.96E-04**	0.91 (0.89, 0.93)	**5.16E-20**
0.84 (0.79, 0.90)	**2.08E-05**	0.90 (0.88, 0.91)	**1.37E-26**
1.01 (0.95, 1.08)	7.80E-01	0.99 (0.97, 1.01)	3.08E-01
1.11 (1.04, 1.18)	**4.51E-03**	1.08 (1.06, 1.10)	**4.95E-14**
1.06 (1.00, 1.12)	8.58E-02	1.01 (1.00, 1.03)	1.66E-01
0.92 (0.89, 0.94)	**4.29E-06**	0.94 (0.92, 0.95)	**1.03E-25**
0.92 (0.89, 0.94)	**3.73E-07**	0.94 (0.93, 0.95)	**1.13E-25**
0.95 (0.89, 1.01)	1.89E-01	0.96 (0.94, 0.98)	**8.34E-05**
1.11 (1.06, 1.16)	**4.09E-05**	1.08 (1.07, 1.10)	**4.81E-24**
1.02 (0.96, 1.10)	5.58E-01	0.98 (0.96, 1.00)	**3.18E-02**

0.92 (0.89, 0.96)	**2.46E-04**	0.94 (0.93, 0.95)	**9.07E-18**
0.90 (0.85, 0.95)	**1.26E-03**	0.97 (0.95, 0.99)	**4.36E-03**
0.99 (0.93, 1.07)	8.66E-01	0.95 (0.93, 0.97)	**2.64E-06**
1.10 (1.05, 1.14)	**1.68E-04**	1.08 (1.07, 1.10)	**7.50E-27**
1.10 (1.03, 1.17)	**9.90E-03**	1.05 (1.03, 1.07)	**8.76E-06**
0.90 (0.86, 0.95)	**2.78E-04**	0.92 (0.91, 0.94)	**6.92E-23**
0.91 (0.86, 0.97)	**1.18E-02**	0.96 (0.94, 0.98)	**4.14E-04**
0.96 (0.90, 1.03)	3.55E-01	0.94 (0.93, 0.96)	**6.76E-09**
1.05 (0.99, 1.12)	1.61E-01	1.06 (1.04, 1.08)	**5.18E-10**
1.14 (1.05, 1.24)	**4.83E-03**	1.07 (1.05, 1.10)	**1.11E-09**
0.90 (0.84, 0.96)	**8.89E-03**	0.92 (0.90, 0.94)	**1.56E-13**
0.95 (0.90, 1.01)	1.63E-01	0.93 (0.91, 0.94)	**1.80E-16**
0.91 (0.84, 0.98)	**3.15E-02**	0.98 (0.96, 1.00)	7.15E-02
0.94 (0.88, 1.01)	1.61E-01	0.99 (0.97, 1.01)	4.00E-01
0.93 (0.87, 0.99)	5.31E-02	1.01 (0.99, 1.03)	2.74E-01
1.05 (0.98, 1.13)	2.56E-01	0.98 (0.96, 1.00)	8.73E-02
1.05 (0.98, 1.12)	2.75E-01	0.97 (0.95, 0.99)	**3.00E-03**
1.04 (0.97, 1.11)	3.28E-01	1.04 (1.02, 1.06)	**3.16E-04**
0.99 (0.93, 1.06)	8.48E-01	1.02 (1.00, 1.04)	7.15E-02
1.03 (0.95, 1.10)	5.63E-01	1.08 (1.06, 1.11)	**5.64E-13**
0.97 (0.91, 1.05)	5.60E-01	0.93 (0.91, 0.95)	**1.08E-11**
0.98 (0.91, 1.05)	6.52E-01	0.93 (0.91, 0.95)	**1.73E-11**
0.96 (0.89, 1.04)	4.03E-01	0.96 (0.94, 0.98)	**5.70E-04**
1.02 (0.95, 1.10)	5.85E-01	1.07 (1.04, 1.09)	**7.46E-10**
1.09 (1.01, 1.17)	**4.18E-02**	1.09 (1.07, 1.12)	**5.24E-17**
0.93 (0.87, 1.00)	7.35E-02	0.90 (0.89, 0.92)	**1.75E-22**
0.92 (0.86, 0.98)	**3.24E-02**	0.90 (0.88, 0.92)	**1.50E-24**
1.06 (0.99, 1.14)	1.67E-01	1.02 (1.00, 1.05)	**4.15E-02**
1.03 (0.96, 1.11)	5.10E-01	1.07 (1.05, 1.10)	**1.96E-10**
0.86 (0.80, 0.92)	**4.86E-04**	0.96 (0.94, 0.98)	**5.22E-04**
1.05 (0.97, 1.12)	2.90E-01	0.98 (0.96, 1.00)	1.19E-01
1.13 (1.05, 1.23)	**5.89E-03**	1.06 (1.03, 1.08)	**1.54E-05**
0.88 (0.82, 0.95)	**4.40E-03**	0.94 (0.92, 0.96)	**7.40E-08**
0.93 (0.86, 0.99)	6.29E-02	0.99 (0.97, 1.01)	2.82E-01
0.86 (0.80, 0.92)	**4.35E-04**	0.93 (0.91, 0.95)	**4.93E-11**
0.91 (0.84, 0.97)	**1.62E-02**	0.97 (0.95, 0.99)	**6.60E-03**
0.88 (0.82, 0.95)	**3.36E-03**	0.94 (0.92, 0.96)	**9.15E-09**
0.90 (0.83, 0.96)	**8.46E-03**	0.95 (0.93, 0.97)	**1.22E-05**
0.93 (0.87, 1.00)	8.27E-02	1.00 (0.98, 1.02)	8.87E-01
0.92 (0.85, 0.99)	**4.16E-02**	0.99 (0.97, 1.01)	4.64E-01
0.98 (0.91, 1.05)	6.28E-01	1.04 (1.01, 1.06)	**1.10E-03**
0.99 (0.92, 1.06)	8.05E-01	1.04 (1.02, 1.06)	**2.98E-04**
0.92 (0.85, 0.99)	**3.86E-02**	0.97 (0.95, 0.99)	**8.15E-03**
0.95 (0.88, 1.03)	2.75E-01	1.02 (1.00, 1.04)	1.54E-01
0.91 (0.85, 0.98)	**3.60E-02**	0.96 (0.94, 0.98)	**1.08E-03**
0.94 (0.88, 1.02)	1.90E-01	0.98 (0.96, 1.00)	1.38E-01

0.92 (0.85, 0.99)	**4.11E-02**	0.97 (0.94, 0.99)	**2.41E-03**
0.95 (0.88, 1.03)	2.70E-01	0.96 (0.94, 0.98)	**1.31E-03**
0.90 (0.84, 0.97)	**1.07E-02**	0.96 (0.94, 0.98)	**4.54E-04**
0.95 (0.89, 1.02)	2.32E-01	0.99 (0.97, 1.01)	3.25E-01
0.92 (0.85, 0.98)	**3.55E-02**	0.99 (0.97, 1.01)	2.70E-01
0.92 (0.86, 0.99)	**4.32E-02**	0.88 (0.86, 0.90)	**7.38E-29**
0.91 (0.84, 0.98)	**2.61E-02**	0.90 (0.88, 0.92)	**2.57E-20**
0.91 (0.85, 0.98)	**2.33E-02**	0.95 (0.93, 0.97)	**9.77E-06**
0.88 (0.82, 0.95)	**4.74E-03**	0.91 (0.89, 0.93)	**8.84E-18**
0.89 (0.83, 0.96)	**7.31E-03**	0.95 (0.93, 0.97)	**2.40E-06**
0.88 (0.82, 0.94)	**2.04E-03**	0.93 (0.91, 0.95)	**1.36E-10**
0.96 (0.89, 1.03)	3.11E-01	1.02 (1.00, 1.04)	9.61E-02
0.93 (0.86, 0.99)	5.66E-02	1.01 (0.99, 1.03)	4.70E-01
0.97 (0.90, 1.04)	4.89E-01	0.90 (0.88, 0.92)	**6.65E-20**
0.99 (0.92, 1.07)	8.43E-01	0.94 (0.92, 0.96)	**4.57E-09**
0.92 (0.85, 0.99)	**4.11E-02**	0.89 (0.87, 0.91)	**4.25E-23**
1.02 (0.95, 1.09)	6.78E-01	0.95 (0.93, 0.97)	**1.08E-06**
0.98 (0.91, 1.05)	6.80E-01	0.91 (0.89, 0.92)	**1.25E-19**
1.05 (0.97, 1.13)	3.06E-01	1.09 (1.07, 1.11)	**2.88E-14**
0.98 (0.92, 1.05)	6.54E-01	1.07 (1.05, 1.09)	**1.56E-11**
1.04 (0.98, 1.11)	2.41E-01	1.05 (1.03, 1.07)	**9.07E-06**
0.99 (0.92, 1.06)	8.22E-01	1.02 (1.00, 1.04)	5.71E-02
1.05 (0.99, 1.12)	1.40E-01	1.04 (1.02, 1.06)	**4.74E-04**
1.00 (0.93, 1.07)	9.99E-01	0.97 (0.95, 0.99)	**2.41E-03**
1.12 (1.04, 1.20)	**5.35E-03**	0.95 (0.93, 0.97)	**1.25E-06**
0.99 (0.93, 1.06)	8.73E-01	0.95 (0.93, 0.97)	**1.06E-05**
0.97 (0.90, 1.04)	4.46E-01	0.99 (0.97, 1.01)	2.18E-01
0.89 (0.82, 0.96)	**7.20E-03**	0.96 (0.94, 0.98)	**7.59E-05**
0.88 (0.82, 0.95)	**5.35E-03**	0.96 (0.94, 0.98)	**7.12E-05**
1.03 (0.96, 1.09)	5.51E-01	1.03 (1.01, 1.05)	**1.57E-03**
1.01 (0.94, 1.08)	8.20E-01	1.01 (0.99, 1.04)	1.88E-01
1.07 (1.04, 1.10)	**9.28E-05**	1.04 (1.03, 1.05)	**9.87E-09**
1.07 (1.03, 1.10)	**1.97E-03**	1.05 (1.03, 1.06)	**1.00E-09**
1.08 (1.03, 1.13)	**4.83E-03**	1.05 (1.03, 1.07)	**5.11E-09**
1.07 (1.04, 1.10)	**4.74E-05**	1.03 (1.01, 1.04)	**2.59E-03**
0.89 (0.83, 0.96)	**5.55E-03**	0.90 (0.88, 0.92)	**1.30E-23**
1.09 (1.01, 1.17)	**4.13E-02**	1.14 (1.12, 1.17)	**1.24E-34**
1.08 (1.01, 1.17)	6.24E-02	0.99 (0.97, 1.01)	3.13E-01
0.90 (0.83, 0.97)	**1.37E-02**	0.96 (0.94, 0.98)	**5.06E-04**
0.88 (0.82, 0.95)	**2.36E-03**	0.95 (0.93, 0.97)	**1.75E-06**
0.87 (0.81, 0.93)	**7.99E-04**	0.93 (0.91, 0.95)	**4.13E-10**
0.86 (0.80, 0.92)	**3.86E-04**	0.93 (0.91, 0.95)	**1.82E-10**
0.99 (0.91, 1.07)	7.71E-01	0.95 (0.93, 0.98)	**2.86E-04**
0.97 (0.90, 1.05)	5.85E-01	0.90 (0.88, 0.92)	**7.46E-19**
1.08 (1.03, 1.13)	**5.35E-03**	1.08 (1.06, 1.09)	**1.88E-23**
0.92 (0.86, 0.99)	**4.21E-02**	0.99 (0.97, 1.01)	2.81E-01
0.87 (0.81, 0.93)	**8.43E-04**	0.93 (0.91, 0.95)	**6.82E-12**
1.00 (0.92, 1.08)	9.68E-01	0.99 (0.96, 1.01)	3.08E-01

1.00 (0.93, 1.07)	9.64E-01	1.02 (1.00, 1.04)	**2.29E-02**
1.08 (1.05, 1.12)	**9.82E-05**	1.04 (1.02, 1.06)	**5.72E-06**
0.89 (0.83, 0.96)	**7.20E-03**	0.93 (0.91, 0.95)	**5.31E-11**
0.93 (0.87, 1.00)	9.68E-02	1.00 (0.98, 1.02)	7.41E-01
0.90 (0.84, 0.96)	**8.98E-03**	0.96 (0.94, 0.98)	**3.10E-05**
0.90 (0.84, 0.97)	**1.72E-02**	0.93 (0.91, 0.95)	**4.14E-10**
0.91 (0.84, 0.97)	**1.95E-02**	0.97 (0.95, 0.99)	**2.11E-03**
0.92 (0.86, 0.99)	5.66E-02	0.99 (0.97, 1.01)	5.84E-01
0.87 (0.81, 0.93)	**5.99E-04**	0.94 (0.92, 0.95)	**7.46E-10**
0.98 (0.91, 1.06)	6.91E-01	0.99 (0.96, 1.01)	2.57E-01
0.89 (0.83, 0.95)	**4.90E-03**	0.96 (0.94, 0.98)	**2.01E-04**
0.92 (0.85, 0.99)	**3.86E-02**	0.99 (0.97, 1.01)	4.00E-01
0.87 (0.81, 0.93)	**5.99E-04**	0.94 (0.92, 0.96)	**1.80E-09**
0.98 (0.91, 1.06)	7.37E-01	0.98 (0.96, 1.00)	7.76E-02
0.93 (0.87, 1.00)	9.47E-02	0.99 (0.97, 1.01)	2.96E-01
0.99 (0.91, 1.07)	8.18E-01	0.96 (0.94, 0.98)	**2.03E-03**
1.03 (0.95, 1.11)	6.06E-01	1.05 (1.03, 1.08)	**1.21E-04**

Other mortality	
Adjusted HR (95% CI)	P value
0.91 (0.89, 0.93)	**2.77E-13**
0.91 (0.89, 0.93)	**3.02E-13**
0.91 (0.89, 0.94)	**9.18E-13**
0.91 (0.88, 0.93)	**2.41E-14**
0.90 (0.88, 0.93)	**1.21E-14**
0.91 (0.89, 0.93)	**1.30E-13**
0.91 (0.89, 0.94)	**1.08E-12**
0.86 (0.83, 0.88)	**2.51E-31**
0.85 (0.83, 0.88)	**6.74E-32**
0.86 (0.84, 0.88)	**3.85E-29**
0.85 (0.83, 0.87)	**1.58E-35**
0.84 (0.82, 0.86)	**8.00E-39**
0.86 (0.84, 0.88)	**3.74E-30**
0.86 (0.84, 0.88)	**4.69E-30**
0.84 (0.82, 0.86)	**2.16E-39**
0.83 (0.81, 0.85)	**1.32E-45**
0.84 (0.82, 0.86)	**6.29E-38**
0.83 (0.81, 0.86)	**1.32E-42**
0.84 (0.82, 0.86)	**1.17E-39**
0.83 (0.81, 0.85)	**2.95E-43**
0.82 (0.80, 0.84)	**1.42E-47**
0.84 (0.82, 0.86)	**2.37E-40**
0.83 (0.81, 0.85)	**1.45E-47**
0.85 (0.83, 0.87)	**1.67E-36**
0.88 (0.86, 0.90)	**3.77E-22**
0.91 (0.89, 0.93)	**1.21E-13**
0.86 (0.84, 0.88)	**2.12E-32**
0.82 (0.80, 0.84)	**3.43E-51**
0.87 (0.85, 0.89)	**8.20E-27**
0.87 (0.84, 0.89)	**1.92E-28**
0.86 (0.84, 0.89)	**1.68E-29**
0.88 (0.86, 0.91)	**4.98E-22**
0.89 (0.87, 0.91)	**6.32E-19**
0.87 (0.85, 0.89)	**4.64E-27**
0.88 (0.86, 0.90)	**1.15E-23**
1.00 (0.98, 1.03)	8.47E-01
1.01 (0.98, 1.03)	4.95E-01
1.02 (1.00, 1.05)	8.48E-02
1.01 (0.99, 1.04)	3.95E-01
1.01 (0.99, 1.04)	3.74E-01
1.01 (0.99, 1.03)	4.67E-01
0.99 (0.96, 1.01)	2.30E-01

0.94 (0.92, 0.96)	**1.86E-06**
0.96 (0.93, 0.98)	**1.05E-03**
0.97 (0.95, 1.00)	**2.83E-02**
0.95 (0.92, 0.97)	**4.34E-05**
0.94 (0.92, 0.97)	**5.07E-06**
0.97 (0.94, 0.99)	**8.01E-03**
1.03 (1.01, 1.05)	**1.76E-02**
0.89 (0.86, 0.91)	**2.52E-21**
0.88 (0.86, 0.90)	**2.20E-24**
0.88 (0.86, 0.90)	**1.49E-24**
0.87 (0.85, 0.89)	**5.47E-27**
0.86 (0.84, 0.88)	**1.35E-30**
0.90 (0.87, 0.92)	**3.96E-17**
1.01 (0.99, 1.04)	2.23E-01
0.85 (0.83, 0.87)	**6.49E-36**
0.83 (0.81, 0.85)	**1.27E-47**
0.83 (0.81, 0.85)	**5.24E-48**
0.83 (0.81, 0.85)	**1.29E-50**
0.82 (0.80, 0.84)	**1.23E-52**
0.85 (0.83, 0.87)	**5.25E-39**
0.96 (0.94, 0.99)	**2.94E-03**
0.87 (0.85, 0.90)	**4.24E-26**
0.85 (0.83, 0.87)	**3.16E-36**
0.88 (0.86, 0.90)	**1.08E-24**
0.84 (0.82, 0.86)	**1.38E-41**
0.84 (0.82, 0.86)	**3.12E-42**
0.86 (0.84, 0.89)	**1.21E-31**
0.93 (0.91, 0.95)	**5.62E-09**
1.26 (1.23, 1.30)	**9.38E-76**
1.29 (1.26, 1.32)	**1.85E-88**
1.30 (1.27, 1.33)	**1.38E-95**
1.27 (1.24, 1.30)	**8.39E-77**
1.26 (1.23, 1.30)	**3.86E-70**
1.28 (1.25, 1.31)	**4.35E-94**
1.05 (1.03, 1.07)	**3.82E-05**
1.24 (1.21, 1.28)	**6.34E-56**
1.25 (1.21, 1.28)	**2.30E-58**
1.24 (1.21, 1.28)	**2.36E-56**
1.24 (1.21, 1.28)	**6.96E-57**
1.24 (1.21, 1.27)	**3.33E-54**
1.26 (1.23, 1.29)	**4.41E-65**
1.10 (1.08, 1.13)	**5.05E-16**
1.03 (1.01, 1.06)	**1.87E-02**
1.03 (1.00, 1.05)	5.65E-02
1.02 (1.00, 1.05)	1.18E-01
1.04 (1.01, 1.06)	**7.50E-03**
1.03 (1.01, 1.06)	**1.81E-02**
1.05 (1.03, 1.08)	**1.07E-04**

0.97 (0.95, 1.00)	**2.04E-02**
0.81 (0.79, 0.83)	**7.71E-62**
0.84 (0.82, 0.87)	**1.77E-40**
0.87 (0.85, 0.89)	**6.15E-28**
0.82 (0.80, 0.84)	**2.34E-54**
0.81 (0.79, 0.83)	**7.05E-64**
0.89 (0.87, 0.91)	**6.83E-21**
0.90 (0.88, 0.92)	**6.45E-16**
1.01 (0.98, 1.03)	7.19E-01
1.06 (1.03, 1.08)	**1.82E-05**
1.05 (1.02, 1.08)	**2.08E-04**
1.06 (1.03, 1.08)	**1.52E-05**
0.95 (0.93, 0.97)	**8.47E-05**
1.12 (1.09, 1.15)	**7.72E-18**
1.10 (1.07, 1.13)	**8.96E-13**
1.16 (1.13, 1.19)	**1.33E-32**
0.89 (0.87, 0.92)	**1.05E-17**
0.94 (0.92, 0.97)	**1.83E-05**
1.13 (1.10, 1.16)	**1.36E-22**
1.15 (1.13, 1.18)	**1.44E-30**
1.04 (1.02, 1.07)	**2.33E-03**
0.94 (0.91, 0.96)	**8.34E-07**
1.08 (1.05, 1.11)	**5.75E-10**
1.10 (1.08, 1.13)	**5.79E-14**
1.11 (1.08, 1.14)	**1.52E-14**
1.09 (1.06, 1.12)	**1.47E-11**
0.91 (0.89, 0.93)	**2.10E-13**
0.99 (0.97, 1.02)	7.09E-01
1.03 (1.01, 1.06)	**1.63E-02**
1.05 (1.03, 1.08)	**7.03E-05**
1.00 (0.97, 1.03)	9.90E-01
0.98 (0.96, 1.00)	1.08E-01
1.11 (1.09, 1.14)	**2.61E-17**
1.02 (1.00, 1.05)	8.62E-02
1.02 (1.00, 1.05)	8.21E-02
1.01 (0.99, 1.04)	2.64E-01
0.95 (0.93, 0.98)	**1.87E-04**
1.10 (1.08, 1.13)	**3.10E-16**
0.93 (0.91, 0.95)	**3.13E-11**
0.90 (0.88, 0.92)	**8.33E-20**
1.02 (1.00, 1.04)	8.36E-02
1.06 (1.03, 1.08)	**9.51E-07**
1.04 (1.01, 1.06)	**8.47E-04**
0.93 (0.92, 0.94)	**1.54E-39**
0.91 (0.91, 0.92)	**6.29E-93**
1.03 (1.01, 1.06)	**5.76E-03**
1.10 (1.08, 1.11)	**1.85E-35**
1.05 (1.03, 1.07)	**2.85E-05**

0.92 (0.91, 0.93)	**4.53E-60**
0.87 (0.85, 0.88)	**8.57E-85**
1.03 (1.01, 1.06)	**1.20E-02**
1.10 (1.09, 1.12)	**2.04E-60**
1.16 (1.14, 1.18)	**3.72E-56**
0.89 (0.88, 0.90)	**9.58E-70**
0.87 (0.85, 0.88)	**1.39E-47**
0.97 (0.95, 0.99)	**1.82E-02**
1.04 (1.02, 1.07)	**5.42E-05**
1.25 (1.22, 1.29)	**1.80E-51**
0.84 (0.82, 0.86)	**7.13E-47**
0.93 (0.91, 0.95)	**6.38E-14**
0.84 (0.82, 0.87)	**3.40E-33**
0.91 (0.89, 0.94)	**3.65E-12**
0.91 (0.89, 0.93)	**1.89E-17**
1.11 (1.08, 1.14)	**4.30E-16**
1.08 (1.06, 1.11)	**2.66E-10**
1.17 (1.14, 1.20)	**3.77E-33**
0.93 (0.91, 0.95)	**5.70E-09**
0.98 (0.95, 1.00)	1.02E-01
1.03 (1.00, 1.06)	**2.29E-02**
1.01 (0.99, 1.04)	2.94E-01
1.09 (1.06, 1.12)	**3.88E-10**
0.97 (0.94, 0.99)	**1.17E-02**
1.19 (1.16, 1.22)	**3.01E-44**
0.91 (0.89, 0.93)	**8.63E-15**
0.88 (0.86, 0.90)	**2.79E-28**
1.16 (1.14, 1.19)	**9.46E-38**
0.98 (0.95, 1.00)	8.36E-02
0.79 (0.77, 0.81)	**7.72E-75**
1.04 (1.02, 1.07)	**1.49E-03**
1.34 (1.30, 1.37)	**6.19E-103**
0.91 (0.89, 0.93)	**3.12E-13**
0.88 (0.86, 0.90)	**1.79E-22**
0.85 (0.83, 0.88)	**7.77E-35**
0.90 (0.88, 0.93)	**4.06E-15**
0.91 (0.89, 0.93)	**1.10E-12**
0.91 (0.88, 0.93)	**3.65E-14**
0.88 (0.86, 0.90)	**1.36E-22**
0.86 (0.84, 0.88)	**3.20E-31**
0.99 (0.97, 1.01)	4.26E-01
0.98 (0.96, 1.00)	1.19E-01
0.96 (0.94, 0.98)	**2.04E-03**
0.87 (0.85, 0.89)	**7.97E-25**
0.96 (0.94, 0.99)	**3.84E-03**
1.01 (0.99, 1.04)	3.73E-01

0.96 (0.94, 0.99)	**3.08E-03**
1.04 (1.01, 1.07)	**6.40E-03**
0.88 (0.86, 0.91)	**2.07E-22**
0.89 (0.87, 0.92)	**7.15E-18**
0.91 (0.89, 0.94)	**1.94E-13**
0.86 (0.84, 0.88)	**4.36E-31**
0.87 (0.84, 0.89)	**3.49E-25**
0.88 (0.86, 0.90)	**1.47E-23**
0.83 (0.81, 0.85)	**7.78E-43**
0.87 (0.85, 0.90)	**7.27E-26**
0.85 (0.82, 0.87)	**2.48E-37**
0.95 (0.92, 0.97)	**5.56E-06**
0.95 (0.93, 0.97)	**1.83E-05**
0.92 (0.89, 0.94)	**2.74E-11**
0.95 (0.93, 0.98)	**1.08E-04**
0.84 (0.82, 0.86)	**5.38E-38**
1.00 (0.97, 1.02)	8.35E-01
0.93 (0.91, 0.96)	**4.53E-08**
1.02 (0.99, 1.04)	1.80E-01
1.10 (1.08, 1.12)	**2.01E-16**
1.06 (1.04, 1.08)	**1.99E-08**
1.04 (1.02, 1.06)	**1.59E-03**
1.08 (1.06, 1.10)	**3.80E-15**
0.94 (0.92, 0.97)	**1.82E-06**
0.97 (0.95, 0.99)	**1.01E-02**
0.88 (0.85, 0.90)	**9.11E-23**
0.91 (0.88, 0.93)	**3.58E-14**
0.83 (0.80, 0.85)	**1.48E-43**
0.80 (0.78, 0.82)	**3.57E-57**
1.04 (1.02, 1.06)	**1.59E-04**
1.03 (1.00, 1.05)	**2.03E-02**
1.09 (1.08, 1.10)	**2.47E-106**
1.06 (1.05, 1.08)	**1.44E-25**
1.09 (1.08, 1.11)	**1.85E-38**
1.05 (1.04, 1.06)	**9.65E-21**
0.79 (0.78, 0.81)	**1.41E-79**
1.07 (1.04, 1.10)	**1.14E-07**
1.05 (1.02, 1.08)	**1.25E-03**
0.82 (0.80, 0.85)	**2.24E-44**
0.88 (0.85, 0.90)	**4.40E-25**
0.87 (0.85, 0.89)	**6.95E-28**
0.85 (0.83, 0.87)	**2.45E-38**
1.10 (1.07, 1.13)	**7.64E-11**
0.96 (0.94, 0.99)	**3.47E-03**
1.12 (1.10, 1.13)	**3.19E-80**
0.87 (0.85, 0.89)	**2.04E-28**
0.88 (0.86, 0.90)	**4.99E-24**
1.14 (1.11, 1.17)	**1.34E-20**

1.05 (1.03, 1.07)	**3.25E-10**
1.09 (1.08, 1.10)	**9.29E-72**
0.93 (0.91, 0.96)	**1.35E-07**
0.89 (0.87, 0.91)	**3.19E-20**
0.87 (0.85, 0.89)	**1.49E-26**
0.95 (0.93, 0.97)	**1.18E-04**
0.95 (0.92, 0.97)	**3.42E-05**
0.87 (0.85, 0.89)	**4.82E-26**
0.86 (0.84, 0.88)	**9.90E-32**
1.09 (1.06, 1.12)	**2.11E-10**
0.90 (0.88, 0.92)	**7.72E-18**
0.86 (0.84, 0.88)	**7.90E-31**
0.86 (0.84, 0.88)	**5.36E-32**
1.10 (1.07, 1.13)	**3.04E-11**
0.90 (0.87, 0.92)	**1.95E-17**
1.11 (1.08, 1.14)	**5.83E-13**
1.01 (0.98, 1.04)	4.90E-01
